# Programmable regiodivergent light-driven cyclization of acyclic 1,5-dienes unlocks rigid bicyclic architectures

**DOI:** 10.1038/s41557-026-02238-y

**Published:** 2026-08-27

**Authors:** Ze-Xin Zhang, KaiChen Shu, Mihai V. Popescu, Yiheng Guo, Jasper L. Tyler, Robert S. Paton, Varinder K. Aggarwal

**Affiliations:** 1 School of Chemistry, University of Bristol, Cantocks Close, Bristol, UK.; 2 Department of Chemistry, Colorado State University, Fort Collins, CO, USA.; 3 These authors contributed equally: Ze-Xin Zhang, KaiChen Shu, Mihai V. Popescu.

## Abstract

Controlling regioselectivity in radical cyclizations remains a major challenge in synthetic chemistry, hindering the efficient assembly of strained, rigid bicyclic architectures from simple acyclic precursors. Here we show that a visible-light-mediated intramolecular [2 + 2] photocycloaddition of aza-1,5-dienes can successfully overcome the classical ‘rule-of-five’ selectivity governing radical cyclizations. By tuning the electronic properties of an easily removable amide N-substituent, we reprogram the initial energy-transfer-driven cyclization event, diverging from the usually kinetically favoured 5-*exo*-trig to the 6-*endo*-trig pathway. As a result, closely related substrates can be selectively directed to generate either bridged bicyclo[2.1.1] or vastly underexplored fused bicyclo[2.2.0] architectures, both of which offer rich downstream derivatization potential. Furthermore, an extensive computational study of this transformation revealed how the intricate interplay of electronic effects controls reaction regioselectivity. Overall, this work establishes a programmable, light-driven cyclization strategy that enables precise and selective construction of distinct bicyclic frameworks within a unified reaction manifold.

Since the discovery of benzene over 200 years ago, the pharmaceutical landscape has been dominated by aromatic rings^1^, but there is growing awareness of the success of more structurally complex, three-dimensional scaffolds that better mimic the intricacies of biological targets^2-4^. This has created an urgent need for novel architectures, particularly rigid, saturated and metabolically stable ring systems, that can evade enzymatic degradation. Rigid, three-dimensional scaffolds with well-defined exit vectors can not only improve binding specificity by reducing entropic penalties but also enhance solubility, reduce off-target effects and improve pharmacokinetic properties^2-4^. A growing body of evidence demonstrates that rigid fragments, such as bicyclo[2.1.1]hexane (BCH), function as effective bioisosteres of substituted benzene rings, often conferring substantial improvements in physicochemical and biological profiles^2-6^. For example, the reported BCH analogues of axitinib^7^ and conivaptan^8^ exhibit enhanced solubility, increased metabolic stability and preserved or improved potency, underscoring the privileged nature of this rigid, saturated scaffold (Fig. 1a). Despite these advantages, the synthetic inaccessibility of this and other related frameworks has hindered their adoption in drug discovery. Current strategies to access rigid bicyclic motifs typically rely on strain-release functionalization of preassembled frameworks such as bicyclo[1.1.0]butane, [1.1.1]propellane or [3.1.1]propellane^4,5,9^. While effective, these approaches intrinsically restrict the substitution patterns that can be accessed, thereby limiting the potential for late-stage diversification. A far more powerful paradigm would be to start from a single, modular acyclic precursor and, through subtle changes, selectively generate distinct rigid frameworks.

The photochemistry of acyclic 1,5-dienes provides a suitable platform to study this type of divergent synthesis^4,7,9-14^; a process that has been previously leveraged in the creation of carbocyclic and, more recently, azacyclic scaffolds bearing the [2.1.1] framework^15-17^. In these cases, bridged [2.1.1] products are exclusively formed because, according to the established ‘rule-of-five’^18-20^, 5-*exo*-trig cyclization dominates over the alternative 6-*endo*-trig (or 4-*exo*-trig) cyclization, which would lead to the alternative fused [2.2.0] products (Fig. 1b). This effect arises from the markedly unfavourable orbital alignment of the 6-*endo*-trig pathway, rendering it >50-fold slower than the competing 5-*exo*-trig closure^21^. In addition, even if the 6-*endo* cyclization were to occur, the subsequent 1,4-biradical recombination would still have to overcome substantial transannular strain and bridgehead compression, making the subsequent C─C bond-forming step towards the [2.2.0] framework equally unfavourable^22,23^. This ring-closure preference is also observed in related 1,6-diene systems, where 5-*exo*-trig cyclizations are kinetically favoured over 6-*exo*-trig cyclizations, typically affording fused [3.2.0] frameworks^24^. Despite this, we recently demonstrated that, for hex-5-enal oximes, this intrinsic 5-*exo*-trig bias can be overridden, leading instead to bridged [3.1.1] bicyclic architectures^25^. However, overriding 5-*exo*-trig cyclization in favour of the much less favourable 6-*endo*-trig cyclization is highly uncommon, underscoring the considerable mechanistic challenge associated with breaking the rule-of-five selectivity for 1,5-dienes.

As a result, access to [2.2.0] scaffolds from simple 1,5-dienes has been considered effectively unattainable, despite it representing a highly strained and rigid framework of potential pharmacological value^26-29^. The only reported synthetic strategy to access aza-[2.2.0] ring systems relies on ultraviolet-light-promoted 4*π* electrocyclization, a transformation that is severely limited with respect to the attainable substitution patterns^26,30,31^. Accordingly, the development of a strategy that enables pathway-controlled, selective construction of either the rule-breaking [2.2.0] or the rule-compliant [2.1.1] bicyclic framework from acyclic 1,5-dienes would be of substantial value in synthetic methodology; however, such a capability has not yet been realized.

Here, we report that, through precise electronic tuning of the nitrogen atom in a class of amide-tethered 1,5-diene precursors, we can successfully divert the reactivity from the classically favoured 5-*exo*-trig towards the rarely observed 6-*endo*-trig pathway^32^, breaking the rule-of-five paradigm (Fig. 1c). By using an essentially common intermediate, either bridged [2.1.1] or fused [2.2.0] skeletons can be accessed selectively, providing synthetic chemists with a unified methodology for generating structurally diverse building blocks. The rigid, three-dimensional β-lactam-like^33^ architecture and hydrogen-bonding capacity of these products underscore their potential for biological applications^34^. Furthermore, the rich downstream chemistry enabled by these scaffolds further highlights their synthetic utility in the construction of structurally complex molecules.

## Results and discussion

### Reaction design and optimization

**Fig. 2 ∣:**
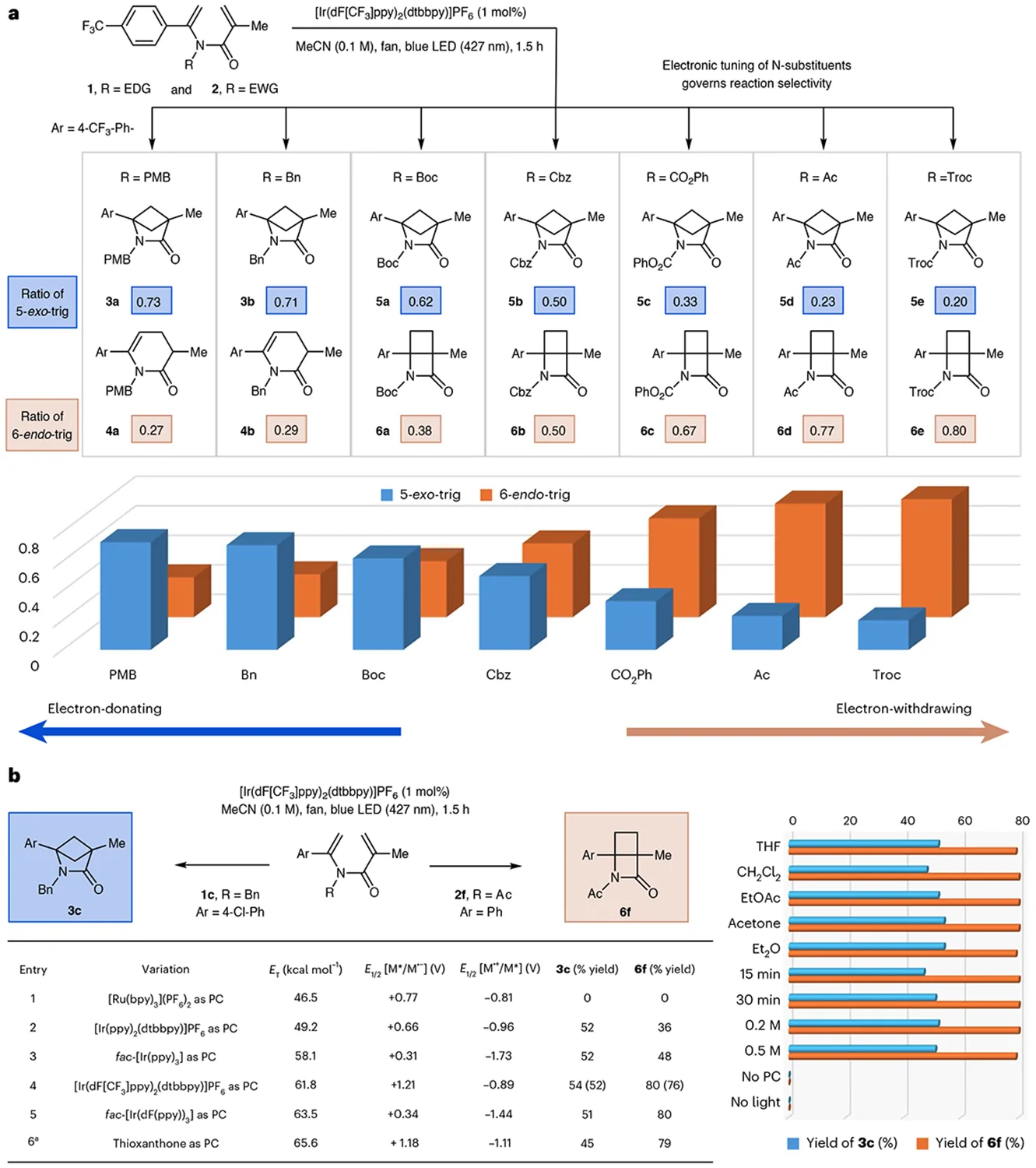
Electronic control of cyclization selectivity and reaction optimization study. **a**, Electronic tuning of nitrogen substituent governs 5-*exo*/6-*endo* cyclization selectivity. Ratios were determined by quantitative ^1^H nuclear magnetic resonance (NMR) spectroscopy of the crude reaction mixtures. For **3a/4a** and **3b/4b**, ratios were determined after 0.5 h owing to the instability of **4. b**, Optimization of reaction parameters for selective cyclization. The yields were determined by quantitative ^1^H NMR spectroscopy of the crude reaction mixture using CH_2_Br_2_ as the internal standard. The yields in parentheses are of isolated products. ^a^Under 390-nm light-emitting diode (LED) irradiation. *E*_1/2_, half-wave potential; ET, triplet energy; ppy, 2-phenylpyridine; dtbbpy, 4,4′-di-*tert*-butyl-2,2′-bipyridine; PMB, *p*-methoxybenzyl; Bn, benzyl; Boc, *tert*-butyloxycarbonyl; Cbz, benzyloxycarbonyl; Ac, acetyl; Troc, 2,2,2-trichloroethoxycarbonyl; THF, tetrahydrofuran; PC, photocatalyst.

Our reaction design was guided by the hypothesis that an amide-tethered 1,5-diene could bias the initial radical addition towards the β-position of the α,β-unsaturated amide through polarity matching, thereby opening access to the typically disfavoured 6-*endo* pathway. Therefore, we subjected N-alkylated substrate **1a** (R = *p*-methoxybenzyl) to [Ir(dF[CF_3_]ppy)_2_(dtbbpy)]PF_6_ (1 mol%) under 427-nm irradiation in MeCN. Under these conditions, we observed that the formation of [2.1.1] product **3a**, arising from the 5-*exo*-trig pathway, dominated the reactivity (Fig. 2a). Despite this, we were encouraged by the detection of an unstable 3,4-dihydro-2-pyridone byproduct **4a** (0.73:0.27 ratio), which presumably forms via an initial 6-*endo* cyclization event. However, the inability of the 6-*endo* radical addition intermediate to undergo the desired recombination to the targeted [2.2.0] product demonstrates the challenges associated with synthesizing these frameworks.

To probe the influence of the nitrogen-appended substituent on cyclization outcome, we synthesized a series of amide-tethered 1,5-dienes with varying electronic properties (**1a–b** and **2a–e**). Remarkably, we observed that, as the electron-withdrawing capacity of the substituent increased, the selectivity of the reaction completely switched from 5-*exo*-trig to favouring the 6-*endo*-trig pathway (Fig. 2a, PMB → Bn → Boc → Cbz → CO_2_Ph → Ac → Troc). More importantly, upon acylation of the nitrogen tether, radical recombination to generate the fused 4,4-ring system became feasible, allowing us to obtain the rarely observed [2.2.0] scaffold as the major product (see below for mechanistic discussion). This highly unexpected trend in selectivity clearly demonstrates that fine-tuning the electronic properties of the nitrogen substituent enables precise control over the reaction pathway, allowing the selective construction of distinct bicyclic architectures.

With this in mind, we then further probed the reaction conditions in an effort to obtain a deeper understanding of this unique system, as well as to identify a single protocol that could deliver both [2.1.1] and [2.2.0] products upon judicious choice of the substrate. We first examined the influence of the photocatalyst. The yields of **3c** and **6f** correlated closely with the triplet energy of the sensitizer but showed no dependence on redox potentials, supporting an energy transfer (EnT), rather than a photoredox mechanism^35,36^ (Fig. 2b, left). In addition, both *N*–Bn and *N*–Ac systems display minimal dependence on solvent polarity, consistent with a neutral EnT process rather than a mechanism involving charge separation^37^ (Fig. 2b, right). This assertion is further corroborated by ultraviolet–visible absorption spectra and cyclic voltammetry studies, which together rule out direct substrate excitation and redox pathways accessible to the excited photocatalyst, and by triplet-quenching experiments showing markedly diminished product yields in the presence of 2,5-dimethyl-2,4-hexadiene (Supplementary Information section 3). As would be predicted, performing the reaction in the absence of the photocatalyst and light revealed that these elements were necessary to promote reactivity. Exploration of reaction time revealed that the transformation reaches completion within 15–30 min, and that yield was largely independent of concentration. Collectively, these observations highlight the intrinsic robustness of the reaction, which operates efficiently over a broad range of solvents, times and concentrations without loss of performance. Nevertheless, to maintain consistency across substrates with differing solubilities, we adopted a 0.1 M concentration and 1.5 h irradiation time for all subsequent studies.

### Substrate scope

Building on the discovery of programmable cyclization regiodivergence, we explored the substrate scope with respect to both pathways. To target the [2.1.1] scaffold, a series of *N*–Bn 1,5-dienes was used under our standard conditions (Table 1). A variety of *C*-2 aryl-substituted precursors, encompassing electron-rich, electron-neutral and electron-deficient arenes (**3b**–**3i**), were readily transformed to the desired bridged products. The presence of a conjugated system at the *C*-2 position is essential for promoting the desired cycloaddition, as it provides a chromophore capable of facilitating EnT to the substrate. Interestingly, the yield of the [2.1.1] scaffold correlated with the electronic nature of the aryl substituent: substrates bearing electron-deficient aryl groups (**3b**–**3e**) generally afforded the [2.1.1] product in higher yields compared with those with electron-rich aryl substituents (**3h** and **3i**). Given that the intrinsically unstable six-membered-ring species (**4**) arising from the 6-*endo* pathway (for *N*–alkyl substrates; Fig. 2a) could typically be observed as a minor component of the reaction, we hypothesize that this decrease in [2.1.1] product yield probably reflects a shift in pathway selectivity driven by changes in *C*-2 electronics. Specifically, whereas electron-deficient aryl substituents favour the 5-*exo*-trig cyclization, electron-rich aryl groups promote the 6-*endo*-trig closure (see below for further discussion). With respect to the nitrogen substituent, *p*-methoxybenzyl (**3a**) and *p*-methoxyphenyl (**3j**) groups were well tolerated. Importantly, debenzylation of **3c** could be achieved under mild conditions to access the corresponding N–H substrate **3c′**.

With respect to *C*-5 substitution, a series of 5-CF_3_-BCH analogues could be prepared in comparable yields to their 5-Me counterparts (**3k**–**3p**). In addition, the trend associated with aryl electronic effects remained consistent: electron-deficient aryl groups exhibited a higher propensity for the formation of the [2.1.1] product via a 5-*exo*-trig pathway. Furthermore, 5-H-BCH (**3q**) and 5-Ph-BCH (**3r**) products could be obtained in moderate yields. When inverting the position of the amide tether, we observed that these substrates (4-aza-1,5-dienes, **7**) uniformly furnished the corresponding [2.1.1] scaffolds (**8a**–**8h**) in good-to-excellent yields. Here, no evidence of 6-*endo*-trig cyclization was detected, probably a consequence of the 6-*endo* pathway now involving a polarity-mismatched radical addition. Importantly, we demonstrated that the selective cleavage of either the amide N-substituent (**8a′**) or the internal amide bond (**8a′′**) could be achieved under acidic and basic conditions, respectively.

We next investigated the scope of [2.2.0] synthesis by using the analogous N–electron-withdrawing 1,5-dienes **2** (Table 2). As expected based on our previous experiments, examination of the *C*-2 aryl substituent (**6d** and **6f**–**6p**) revealed a strong correlation between product distribution and aryl electronics: electron-rich aryl groups promoted the 6-*endo*-trig pathway with excellent levels of selectivity (**6g**–**6k**), while electron-deficient aryl groups (**6d**, **6o** and **6p**) partially eroded this preference. The lower selectivity observed with electron-deficient aryl groups (**6d**, **6o** and **6p**) encouraged us to explore alternative conditions to see if we could override this substrate-dependent regioselectivity. Specifically, we discovered that replacing MeCN with hexafluoroisopropanol (HFIP) as the reaction solvent noticeably increased the ratio of 6-*endo* to 5-*exo* products, presumably a result of partial polarization of the enone via hydrogen bonding, providing a more polarity-matched 6-*endo* pathway. Heteroaryl substituents of varying electronic character (**6q**–**6t**) followed the same trend and consistently afforded the [2.2.0] scaffold in good-to-excellent yields. When the aryl substituent at *C*-2 was exchanged for an ester group, the strongly electron-withdrawing effect completely switched the outcome, exclusively furnishing the [2.1.1] scaffold (**5u**).

As we had initially discovered that altering the substituent at the nitrogen tether had a pronounced impact on regioselectivity, we further explored this electronic effect by using a range of electron-withdrawing groups. Generally, the stronger the electron-withdrawing nature of the substituent, the more the system favoured cyclization to the challenging [2.2.0] scaffold (**6v**–**6ab**). When a sterically hindered pivaloyl group was used, the reaction delivered an unexpected six-membered-ring species **9** in high yield. This outcome can be plausibly attributed to the perpendicular orientation of the pivaloyl carbonyl relative to the six-membered ring, which predisposes the intermediate formed from the initial 6-*endo*-trig cyclization towards intramolecular acyl transfer. When an enantiomerically enriched carbonyl substituent was used, the reaction delivered a pair of diastereomeric [2.2.0] products (**6ad** and **6ad′**), which were successfully separated by flash chromatography, providing the potential to access enantioenriched [2.2.0] scaffolds.

Using substrates that are unsubstituted at the *C*-5 position revealed that the presence of the alkyl group is not a requirement for reactivity (**6ae**–**6ag**). However, replacing Me with H partially lowers the reaction selectivity, presumably due to the decrease in steric hindrance around the *C*-5 position in the 5-*exo*-trig transition state. By contrast, bulkier alkyl groups at *C*-5 (**6ah** and **6ai**) were well tolerated, delivering excellent yields and high levels of selectivity. However, the introduction of an aryl group at *C*-5 (**6aj**) lowers reaction selectivity, as both alkene moieties can be photoexcited for this substrate. Substitution at *C*-1 could also be achieved, providing **6ak** and **6al** as a single diastereomer. Here, the relative increase in the steric hindrance associated with the 6-*endo*-trig transition state decreases the reaction selectivity when compared with the *C*-1 unsubstituted analogue **6g**. However, we observed a reversal of selectivity with 6-Me-substituted substrate **6am**, where steric congestion at this position disfavours the radical attack required for the 6-*endo* pathway. To highlight the applicability of this methodology to accessing challenging scaffolds relevant to medicinal chemistry, we applied our 1,5-diene cyclization strategy to the synthesis of **6an**, a [2.2.0] analogue of the δ-lactam-containing cognitive enhancer HT-0712^38^.

### Product derivatization

**Fig. 3 ∣:**
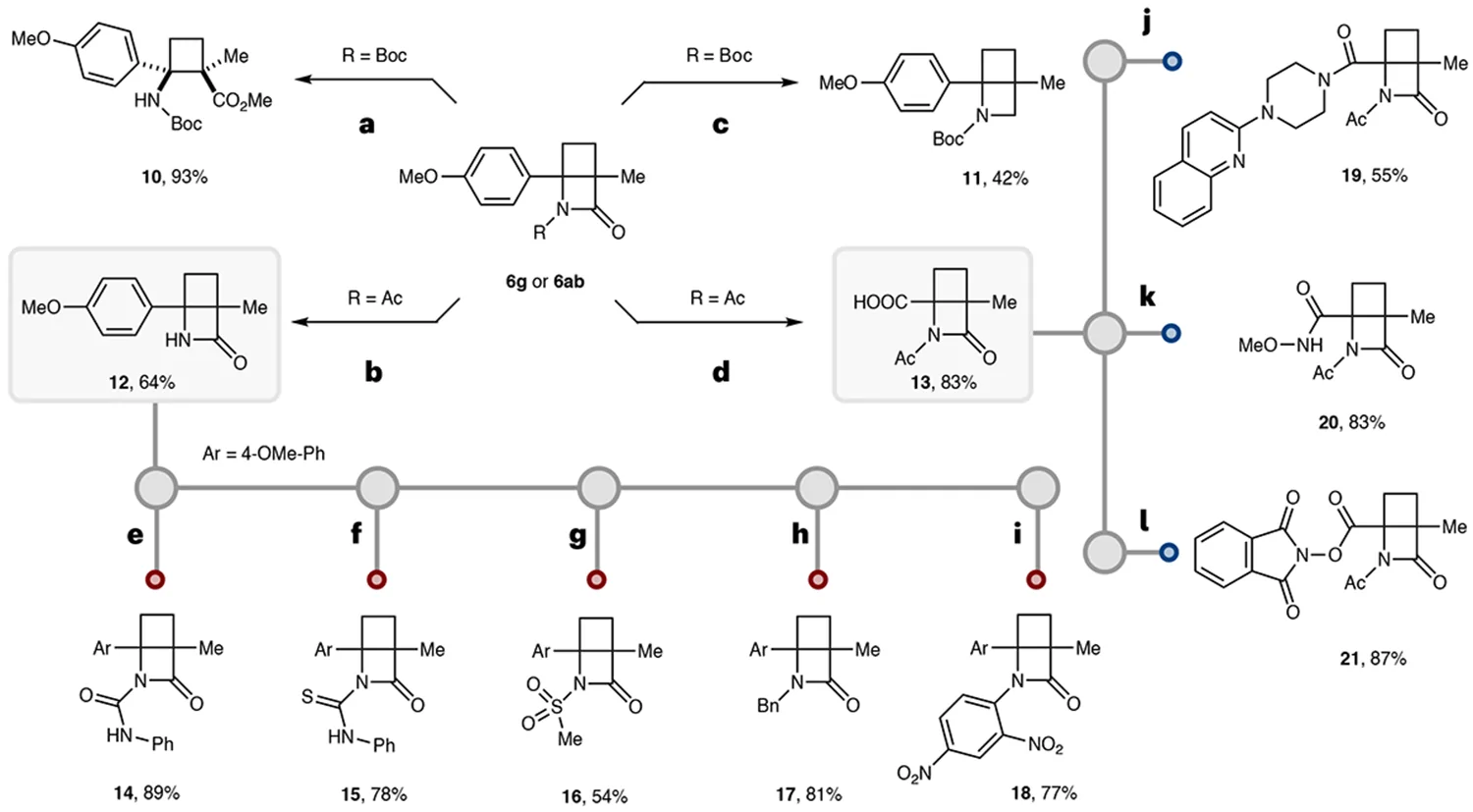
Product diversification study. **a**, Amide bond cleavage, NaOMe, MeOH. **b**, Amide deprotection, NaOMe, THF. **c**, Amide reduction, RhH(CO) (PPh_3_)_3_, PhSiH_3_. **d**, Oxidative cleavage to carboxylic acid, RuCl_3_/NaIO_4_. **e–i**, N-functionalization of β-lactam **12**: *n*-BuLi, phenyl isocyanate (**e**); *n*-BuLi, phenyl isothiocyanate (**f**); *n*-BuLi, methanesulfonyl chloride (**g**); NaH, BnBr (**h**); NaH, 1-fluoro-2,4-dinitrobenzene (**i**). **j–l**, Derivatization of carboxylic acid **13**: oxalyl chloride, then Et_3_N, 2-(piperazin-1-yl)quinoline (**j**); oxalyl chloride, then NH_2_OMe·HCl, NaHCO_3_ (**k**); *N*–hydroxyphthalimide (NHPI), *N,N*′-diisopropylcarbodiimide (DIC), 4-dimethylaminopyridine (DMAP) (**l**).

Having established that precise electronic tuning of this class of 1,5-dienes can enable the synthesis of challenging to access [2.2.0] scaffolds, we next sought to extend the structural diversity of these frameworks through downstream derivatization (Fig. 3). Deprotection of the nitrogen substituent was first targeted to access the corresponding secondary amide. Treatment of **6g** with NaOMe promoted selective cleavage of the *N*–Ac amide bond, presumably due to lower steric hindrance relative to the strained β-lactam, affording **12** in 64% yield. This intermediate proved versatile, undergoing high-yielding transformations to give urea (**14**), thiourea (**15**), sulfonamide (**16**), N-alkylated (**17**) and N-arylated derivatives (**18**), thereby expanding the scope of functionalities accessible. Treatment of *N*–Boc [2.2.0] species **6ab** under basic conditions resulted in selective cleavage of the internal amide bond to afford β-amino acid derivative **10**. Further diversification was achieved through RuCl_3_-mediated oxidation of the aryl ring, delivering the corresponding carboxylic acid (**13**) in excellent yield. This species was then converted to amide **19,** *O*-methylhydroxamic acid **20** and redox-active ester derivative **21**. Finally, the amide carbonyl within the [2.2.0] core was reduced by Rh-catalysed hydrogenation^39^, furnishing fully saturated 2-*N*-[2.2.0] scaffold **11**.

### Computational studies

**Fig. 4 ∣:**
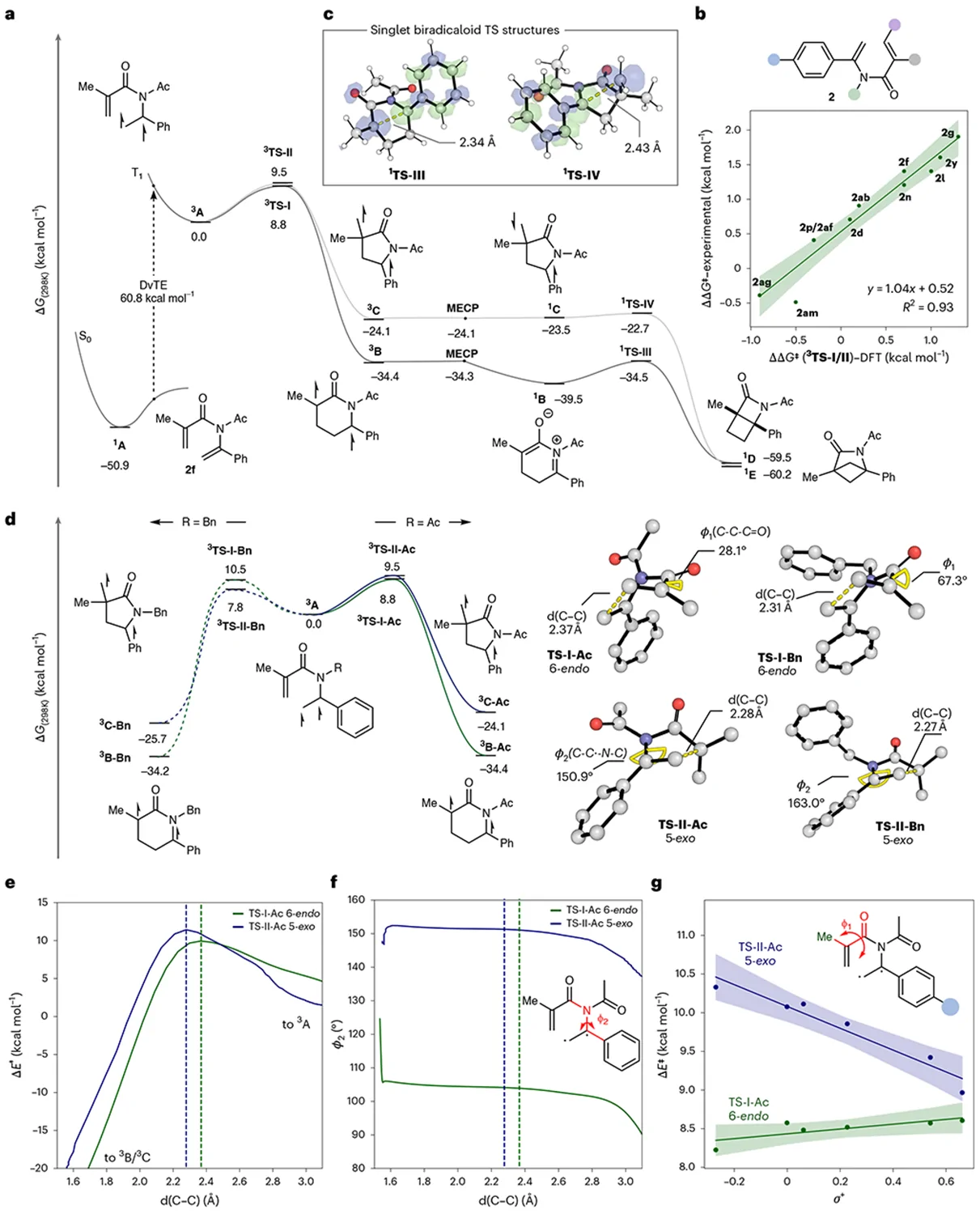
Computational investigation of the reaction mechanism and origin of selectivity. **a**, Gibbs energy surface for the triplet cyclization of **2f (^1^A)** computed at the Pr2SCAN50-D4/def2-TZVPPD,(SMD=MeCN)//M06-2X-D3/6-31+G(d,p),(SMD=MeCN) level of theory. **b**, Computed values of ΔΔ*G*^‡^ (**^3^TS-I/^3^TS-II**) for a series of substrates are highly correlated with experimentally derived values. **c**, Spin density plots of key biradicaloid transition structures illustrate the radical nature of **^1^TS-III** and **^1^TS-IV. d**, Selectivity-determining steps showing reversal of selectivity for N–Bn and N–Ac systems, together with the key TSs. **e**, IRCs of **^3^TS-I/II-Ac** with the forming C–C bond lengths. **f**, The 5-*exo* pathway is characterized by much greater planarity (*ϕ*_2_ = C–C–N–C dihedral angle) in the TS and along the reaction coordinate than the 6-*endo* pathway. **g**, Hammett correlation of computed activation barriers: greater structural planarity in the 5-*exo* TS makes this pathway (blue) more sensitive to electronic effects. For **b** and **g**, the shaded regions represent the 95% confidence intervals of the fitted regression lines. For **e** and **f**, the vertical dotted lines denote the geometric parameters at the corresponding transition-state geometries. MECP, minimum energy crossing point; S_0_, singlet ground state; T_1_, first triplet state; SMD, solvation model based on density; *σ*^+^, Hammett substituent constant.

To unravel the intricate effects that give rise to the observed programmable cyclization regioselectivity, we developed a computational model using density functional theory (DFT) at the Pr2SCAN50-D4/def2-TZVPPD,(SMD=MeCN)//M06-2X-D3/6-31+G(d,p),(SMD=MeCN) level of theory, based on recent benchmarking efforts^40^ (Fig. 4a). We first verified the feasibility of triplet sensitization by computing the triplet energy of model substrate **2f** (**^1^A**). The dynamically vertically accessible triplet energy (DvTE)^41^ was calculated to be 60.8 kcal mol^−1^, while the adiabatic triplet energy was found to be 50.9 kcal mol^−1^ (**^1^A** to **^3^A**), consistent with our initial screen of photocatalysts (Fig. 2b). Analysis of low-energy triplet geometries showed that the spin density is localized on the styrene motif, identifying it as the primary chromophore, consistent with Stern–Volmer quenching studies (Supplementary Information section 3.1). Upon excitation to **^3^A**, the system can undergo cyclization either via a 6-*endo*-trig (**^3^TS-I**) or 5-*exo*-trig (**^3^TS-II**) transition structure (TS), both proceeding in a highly exergonic fashion. The computed selectivity favours 6-*endo* cyclization (**^3^TS-I**), with a ΔΔ*G*^‡^ of 0.7 kcal mol^−1^, in line with the observed experimental product distribution of 10:1 (ΔΔ*G*^‡^ = 1.4 kcal mol^−1^). In addition, computed selectivity values across a range of 1,5-diene substrates (Fig. 4b) were successful in reproducing the general trend in 6-*endo* selectivities (*R*^2^ = 0.93). This was found to be the case for substrates displaying different substituents at the nitrogen atom as well as the *C*-2, *C*-5 and *C*-6 positions, indicating that these calculations accurately capture the electronic and steric factors that together dictate the level of selectivity observed for each substrate.

In the case of 6-*endo* cyclization, we discovered that rather than forming the [2.2.0] scaffold directly via radical recombination, intersystem crossing generates zwitterionic intermediate **^1^B**. This closed-shell species, calculated to be energetically favoured over the corresponding open-shell form (Supplementary Fig. 11), can then deliver [2.2.0] fused product **^1^D** (**6f**) via a 4*π* Staudinger-like electrocyclization. Despite the fact that **^1^B** favours a closed-shell form, the TS for electrocyclization is diradicaloid in nature (**^1^TS-III**; Fig. 4c), consistent with recent studies^42^. This is a consequence of the cyclization having to occur in a disrotatory fashion, as conrotatory closure (as might be expected on the basis of the Woodward–Hoffmann rules) would require the formation of a *trans*-fused [2.2.0] ring system^43^. The inability of the analogous *N*–alkyl-substituted substrate to access the [2.2.0] scaffold can be understood by the relative increase in stability of zwitterionic intermediate **^1^B**, leading to a greater 4*π* electrocyclization energy barrier (Supplementary Fig. 12). Consequently, this pathway becomes less favourable than competing side reactions such as the H-shift pathway and thus generates the observed 3,4-dihydro-2-pyridone byproduct (**4**; Fig. 2a). By contrast, the reaction profile arising from 5-*exo*-trig cyclization (**^3^TS-II**) leads to the formation of triplet intermediate **^3^C**, which, upon intersystem crossing to open-shell diradical **^1^C**, can recombine via **^1^TS-IV** (Δ*G*^‡^ = 0.8 kcal mol^−1^) to give [2.1.1] bicyclic product **^1^E**.

With a general understanding of the reaction mechanism in hand, we next examined the origins of regioselectivity. Using a model *N*–Bn 1,5-diene system as a reference, we calculated that the analogous *N*–Ac substrate displays a 1.7 kcal mol^−1^ lower 6-*endo*-trig (**^3^TS-I**) barrier, in full agreement with our experimental results (Fig. 4d, left), as a result of several factors. In addition to the *N*–Ac system representing a better Giese acceptor owing to its more electron-deficient nature, this difference can be rationalized by the increased TS planarity in **^3^TS-I-Ac** (*φ*_1_ = 28.1°) that facilitates conjugation between the developing α-carbonyl radical and the carbonyl *π*-bond (Fig. 4d, right). This arises due to the cross-conjugation in the imide system enabling partial deconjugation of the endocyclic N─C(=O) bond, increasing conformational flexibility relative to the more rigid amide bond in **^3^TS-I-Bn** (*φ*_1_ = 67.3°).

The favourability of the *N*–Bn system to undergo 5-*exo*-trig cyclization (ΔΔ*G*^‡^ = 1.7 kcal mol^−1^ compared with *N*–Ac) can be explained through consideration of the spectator benzylic radical in the cyclization TS. As well as representing the more planar system (163.0° versus 150.9°), the greater availability of the nitrogen lone pair in **^3^TS-II-Bn** increases benzylic radical stability via captodative effects^44,45^, stabilizing the TS to a greater extent than for the corresponding *N*–Ac system. Indeed, analysis of the *φ*_2_ dihedral change along the intrinsic reaction coordinate (IRC; Fig. 4e,f) for the 5-*exo* TS indicates that planarity is maximized (see Supplementary Fig. 15 for further TS analysis). Moreover, this captodative stabilization can explain the strong correlation between the electronics of the styrene fragment and reaction regioselectivity (Table 2). Analysing the IRC of the 6-*endo*-trig pathway shows that the nitrogen atom remains deconjugated from the benzylic radical throughout, suggesting that changing the electronics of the aryl substituents primarily affects the 5-*exo*-trig pathway. This was confirmed through computational Hammett analysis, clearly demonstrating that electron-withdrawing substituents lower the 5-*exo*-trig (**^3^TS-II**) barrier with a strong correlation to Hammett *σ*^+^ values, while the 6-*endo*-trig TS was minimally impacted (Fig. 4g).

## Conclusion

In this study, we have established a programmable regiodivergent photochemical strategy that fundamentally challenges the long-standing rule-of-five paradigm in radical cyclization. Through electronic modulation of an amide-tethered aza-1,5-diene, we demonstrate that the inherent kinetic preference for 5-*exo*-trig cyclization can be overridden, enabling selective access to either bridged [2.1.1] or fused [2.2.0] heterobicyclic frameworks from closely related precursors. Computational investigations reveal that varying the electronics of the substrate substantially affects the relative stability of the developing and spectator radicals in the transition states, giving rise to the observed tuneable regioselectivity. The robustness of the EnT process, broad substrate scope and extensive downstream derivatization collectively establish these heterobicycles as highly valuable building blocks. The ability to exert such precise pathway control over a strained intramolecular [2 + 2] cycloaddition engenders considerable synthetic potential and addresses multiple intrinsic energetic and geometric constraints associated with 6-*endo*-trig cyclization and subsequent ring closure. We anticipate that this concept will inspire the development of further rule-breaking cyclization strategies and accelerate the discovery of structurally novel, *sp*^3^-rich scaffolds for drug discovery and beyond.

## Methods

### General procedure for intramolecular [2 + 2] cyclization

An oven-dried round-bottom flask containing the 1,5-diene substrate (1.0 equiv.) and [Ir(dF(CF_3_)ppy)_2_(dtbbpy)]PF_6_ (1.0 mol%) was sealed and subjected to three evacuation–backfill cycles with N_2_. Anhydrous MeCN or HFIP (0.1 M) presparged with N2 was added, and the flask was positioned ~1 cm from a 40 W KSPR160L-427 nm Kessil light source (100% intensity). The mixture was stirred under continuous irradiation at ambient temperature (fan-assisted cooling) under N_2_ for 1.5 h. Concentration in vacuo and purification using a Biotage automated flash chromatography system afforded the desired product.

## Supplementary Material

supporting information

**Supplementary information** The online version contains supplementary material available at https://doi.org/10.1038/s41557-026-02238-y.

## Figures and Tables

**Fig. 1 ∣:**
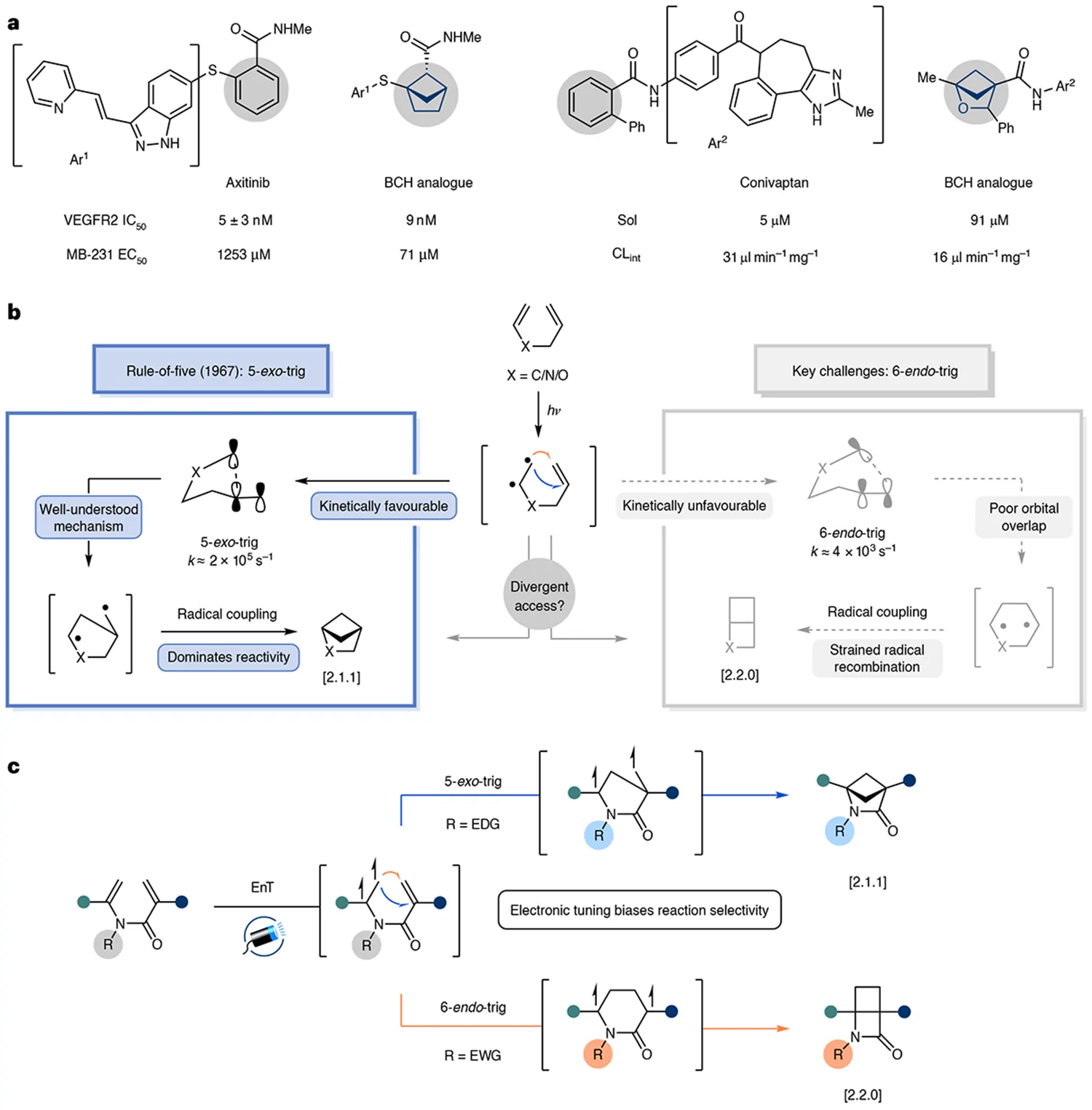
The rule-of-five in radical cyclization reactions and programmable access to rigid bicyclic scaffolds. **a**, BCHs as saturated bioisosteres of aromatic rings in pharmaceuticals. **b,** The rule-of-five: kinetic preference for 5-*exo*-trig cyclization and the challenges associated with the alternative 6-*endo*-trig pathway. **c**, This work: programmable control of cyclization pathways enables divergent access to [2.1.1] and [2.2.0] scaffolds. Sol, kinetic solubility in phosphate-buffered saline, pH 7.4 (μM); CL_int_, intrinsic clearance in human liver microsomes (μl min^−1^ mg^−1^); *k*, rate constant; EDG, electron-donating group; EWG, electron-withdrawing group.

**Table 1 ∣:** Substrate scope of bicyclo[2.1.1]hexane synthesis^a^

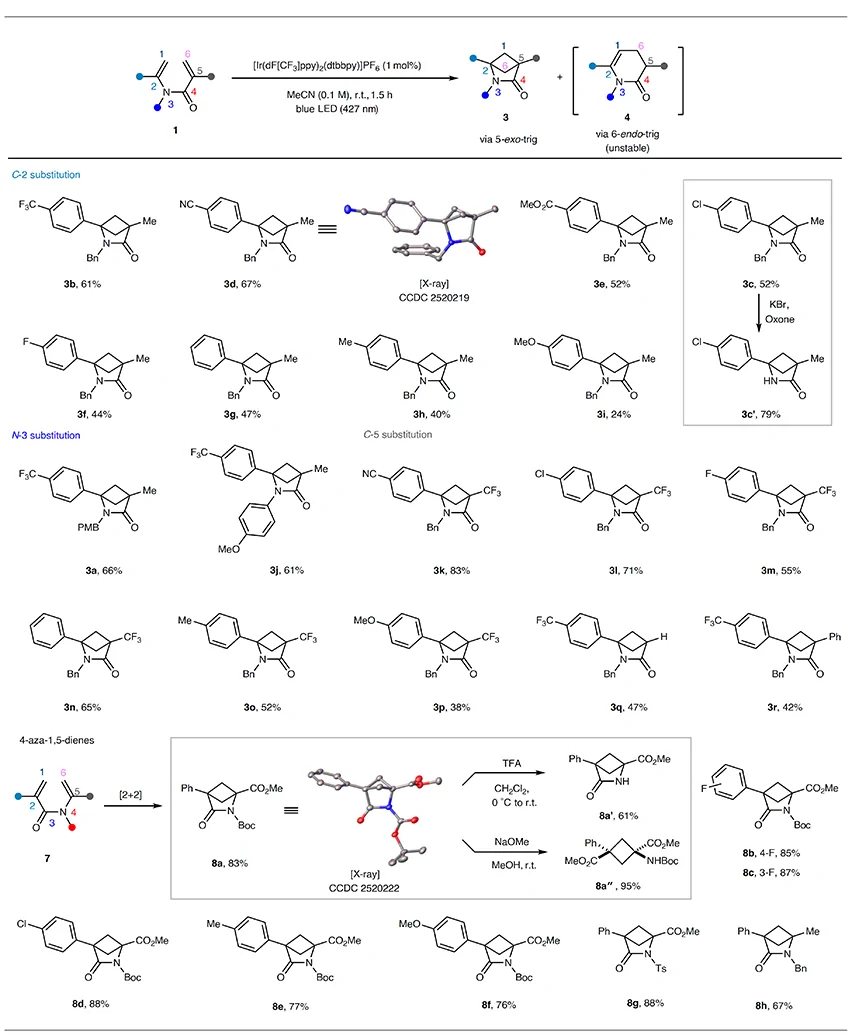

a Conditions: Reactions were performed using 1 mol% [Ir(dF[CF_3_]ppy)_2_(dtbbpy)]PF_6_ in MeCN (0.1 M) under blue LED (427 nm) irradiation for 1.5 h. Yields are of isolated products. Ts, *p*-toluenesulfonyl; Oxone, potassium peroxymonosulfate; TFA, trifluoroacetic acid; r.t., room temperature.

**Table 2 ∣:** Substrate scope of bicyclo[2.2.0]hexane synthesis^a^

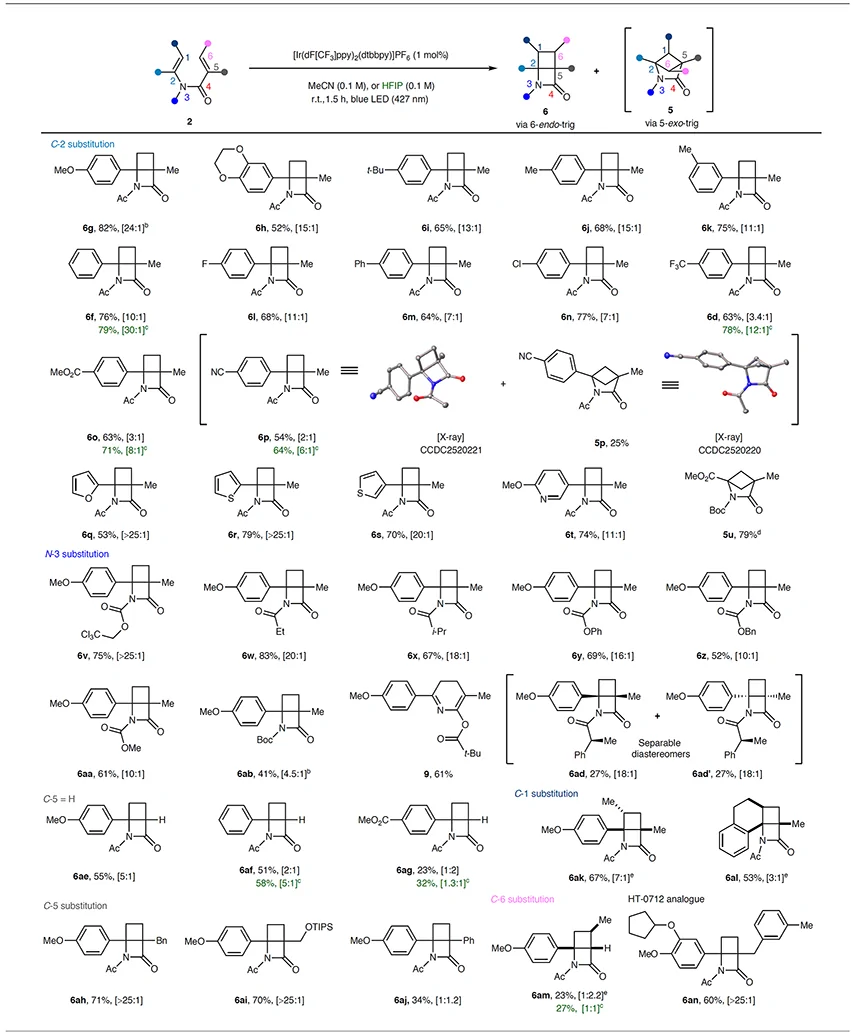

a Reactions were performed using 1 mol% [Ir(dF[CF_3_]ppy)_2_(dtbbpy)]PF_6_ in MeCN (0.1 M) under blue LED (427 nm) irradiation for 1.5 h. Yields are for the specific isolated isomers pictured, while the ratios in square brackets represent the molar ratio of the [2.2.0] to [2.1.1] frameworks, as determined by quantitative ^1^H NMR spectroscopy of the crude reaction mixture.

b Reaction conducted on gram scale with 0.5 mol% [Ir(dF[CF_3_]ppy)_2_(dtbbpy)]PF_6_.

c Reactions performed with HFIP (0.1 M).

d Benzophenone (25 mol%) was used as the photocatalyst under 370-nm LED irradiation at 0.016 M.

e Formed as a single diastereomer. *i*-Pr, *iso*-propyl; *t*-Bu, *tert*-butyl; TIPS, triisopropylsilyl.

## Data Availability

Crystallographic data for the structures reported in this Article have been deposited at the Cambridge Crystallographic Data Centre, under deposition numbers CCDC 2520219 (**3d**), 2520220 (**5p**), 2520221 (**6p**) and 2520222 (**8a**). Copies of the data can be obtained free of charge at https://www.ccdc.cam.ac.uk/structures. Associated data are available in the Article or its Supplementary Information, with coordinates for computationally derived structures available as a separate file.
