# Supplementary material for: Programmable regiodivergent light-driven cyclization of acyclic 1,5-dienes unlocks rigid bicyclic architectures
Source: Nat Chem. Author manuscript; Available in PMC 2026 Sep 20. (PMC13590158; doi:10.1038/s41557-026-02238-y)
Supplement: Supplementary file 1 [file NIHMS2207858-supplement-supporting_information.pdf]

nature chemistry

Supplementary information

https://doi.org/10.1038/s41557-026-02238-y

Programmable regiodivergent light-driven cyclization of acyclic 1,5-dienes unlocks rigid bicyclic architectures

In the format provided by the authors and unedited

Table of Contents

1. Safety Statement, Materials and General Methods 3
1.1. Safety Statement on Handling of β-Lactam-Containing Compounds 3
1.2. Glassware, Solvents and Reagents 3
1.3. Chromatography and Instrumentation 3
1.4. Naming of Compounds 4
2. Experimental Data 5
2.1. Reaction Optimisation Studies 5
2.2 General Procedure 7
2.2.1. General Procedure A: Synthesis of Cyclisation Precursors (1) 7
2.2.2. General Procedure B: Synthesis of Cyclisation Precursors (7) 7
2.2.3. General Procedure C: Synthesis of Cyclisation Precursors (2) 8
2.2.4. General Procedure D: Synthesis of Cyclisation Products 11
2.3 Synthesis of Cyclisation Precursors 13
2.3.1 Synthesis and Characterisation of Precursors (1) 13
2.3.2 Synthesis and Characterisation of Precursors (7) 24
2.3.3 Synthesis and Characterisation of Precursors (2) 29
2.4 Synthesis of Cyclisation Products 55
2.4.1 Synthesis and Characterisation of Products (3 and 4) 55
2.4.2 Synthesis and Characterisation of Products (8) 67
2.4.3 Synthesis and Characterisation of Products (5, 6 and 9) 72
2.5 Product Derivatisation Studies 102
2.6 Unsuccessful Substrates 114
3. Mechanistic studies 115
3.1 Stern-Volmer Quenching Studies 115
3.2 UV-Vis Spectroscopy 116
3.3. Cyclic Voltammetry 117
3.4 Control Experiments with Triplet Quenchers 118
4. Crystallographic Data (X-Ray) 119
4.1 Structure Report for 3d 119
4.2 Structure Report for 5p 123
4.3 Structure Report for 6p 127

4.4 Structure Report for 8a 139
5. NMR Spectra 145
5.1 NMR Spectra of Cyclisation Precursors (1) 145
5.2 NMR Spectra of Cyclisation Precursors (7) 175
5.3 NMR Spectra of Cyclisation Precursors (2) 185
5.4 NMR Spectra of Products (3 and 4) 237
5.5 NMR Spectra of Products (8) 272
5.6 NMR Spectra of Products (5, 6 and 9) 282
5.7 NMR Spectra of Product Derivatives 347
6. Computational Details 363
6.1. General Information 363
6.2. Reaction Mechanism: Thermochemical Summary 365
6.3. Dynamic vertical triplet energy analysis 369
6.4. Thermochemistry 370
7. References 374

1. Safety Statement, Materials and General Methods

1.1. Safety Statement on Handling of β-Lactam-Containing Compounds

Compounds bearing a β-lactam ring, including synthetic intermediates and derivatives, are known sensitising agents that can induce hypersensitivity reactions in susceptible individuals. Reactions range from mild skin rashes to life-threatening anaphylaxis, and prior sensitisation may occur without the individual's awareness.[1,2] Laboratory personnel should be aware of their β-lactam allergy status before undertaking synthetic work with these compounds. Appropriate personal protective equipment (gloves, lab coat, eye protection) should be worn at all times, and work should be conducted in a well-ventilated fume hood to minimise inhalation and dermal exposure.[3] In pharmaceutical manufacturing environments in the United States, β-lactam-containing compounds are subject to zero-threshold cross-contamination policies and must be handled in dedicated, physically separated facilities with independent air-handling systems, in accordance with FDA CGMP guidance (FDA-2011-D-0104).

1.2. Glassware, Solvents and Reagents

All reactions were conducted under an inert atmosphere of nitrogen using standard Schlenk manifold techniques unless mentioned otherwise. All glassware was oven-dried at >100 °C and allowed to cool to room temperature under a positive pressure of nitrogen. Reactions were monitored by TLC until deemed complete using aluminium-backed silica plates. Plates were visualised under ultraviolet light (254 nm) and/or by staining with $KMnO_4$ solution. Cooling of reaction mixtures to 0 °C was achieved using an ice-water bath. Cooling of reaction mixtures to −78 °C was achieved using a dry ice-acetone bath. "Room temperature" refers to an ambient temperature of 23 ± 2 °C.

All anhydrous solvents (MeOH, CH_3CN, acetone, DMF, DMSO, 1,4-dioxane, Et_2O, THF, CH_2Cl_2, 1,2-Dichloroethane, etc.) were commercially supplied (Thermo Scientific Chemicals). All reagents were purchased from commercial sources [Sigma Aldrich (Merck), Thermo Scientific Chemicals, Fluorochem Ltd, TCI, etc.] and used as received unless otherwise stated. Irradiation of reaction mixtures was achieved using a 40 W KSPR160L-427 nm (or 370 and 390 nm) Kessil light (100% intensity). Brine refers to a saturated aqueous solution of NaCl.

1.3. Chromatography and Instrumentation

Flash column chromatography (FCC) was carried out using the Biotage IsoleraTM flash purification system equipped with a high-efficiency silica column (Biotage® Sfär Silica HC D High Capacity Duo, 20 μm).

NMR spectra were recorded at various field strengths, as indicated, using Bruker 400 MHz and Bruker 500 MHz for ^{1}H, ^{13}C, ^{19}F acquisitions. All NMR spectra were recorded at ~25 °C in $CDCl_3$ unless otherwise stated. All reported ^{1}H and ^{13}C chemical shifts (δ_H, δ_C) are referenced to the residual

signal of deuterated solvents ($CDCl_3$: δ_H = 7.26 ppm, δ_C = 77.16 ppm; $(CD_3)_2SO$: δ_H = 2.50 ppm, δ_C = 39.52 ppm; $(CD_3)_2CO$: δ_H = 2.05 ppm, δ_C = 206.26 ppm; CD_3CN: δ_H = 1.94 ppm, δ_C = 118.26 ppm; CD_2Cl_2: δ_H = 5.32 ppm, δ_C = 53.84 ppm). Chemical shifts (δ) are reported in parts per million (ppm) to the nearest 0.01 ppm for ^{1}H, ^{19}F NMR and 0.1 ppm for ^{13}C NMR. Coupling constants (*J*) are reported in Hertz (Hz). Multiplicities are reported as follows: s (singlet), d (doublet), t (triplet), q (quartet), pent. (quintet), hept. (septet), m (multiplet), br. (broad signal), app. (apparent). The ^{1}H NMR spectra are reported as follows: chemical shift (multiplicity, coupling constants, number of protons).

High resolution mass spectra (HRMS) were recorded on a Bruker Daltonics MicrOTOF II by Electrospray Ionisation (ESI); a Thermo Scientific QExactive by Electron Ionisation (EI); a Thermo Scientific Orbitrap Elite by ESI or Atmospheric Pressure Chemical Ionisation (APCI); or a Bruker UltrafleXtreme by Matrix-assisted Laser Desorption/Ionisation (MALDI).

IR spectra were recorded neat as a thin film on a Perkin Elmer Spectrum One FT-IR. Selected absorption maxima (ν_{max}) are reported in wavenumbers (cm^{-1}).

1.4. Naming of Compounds

Compound names are those generated by ChemDraw Professional 20.0 software (PerkinElmer), following the IUPAC nomenclature.

2. Experimental Data

2.1. Reaction Optimisation Studies

Supplementary Table S1. Optimisation of the intramolecular cycloaddition of precursor **1c**

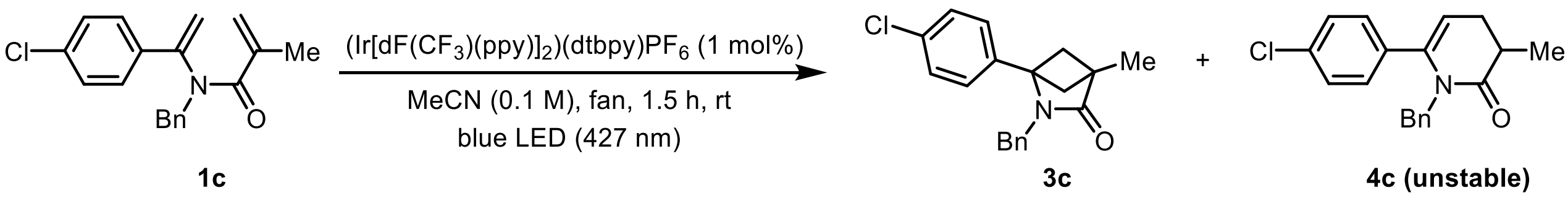

**Entry**	variation	**E$_T$** (kcal/mol)	**E$_{1/2}$ [M*/M$^{•-}$]** (V)	**E$_{1/2}$ [M$^{•+}$/M*]** (V)	**3c** (% yield)	**4c** (% yield)
**1**	$[Ru(bpy)_3](PF_6)_2$ as PC	46.5	+0.77	-0.81	0	0
**2**	$[Ir(ppy)_2(dtbbpy)]PF_6$ as PC	49.2	+0.66	-0.96	52	35
**3**	*fac*-$[Ir(ppy)_3]$ as PC	58.1	+0.31	-1.73	52	35
**4**	$(Ir[dF(CF_3)(ppy)]_2)(dtbpy)PF_6$ as PC	61.8	+1.21	-0.89	54 (52)	16 (14)
**5**	*fac*-$[Ir(dF(ppy))_3]$ as PC	63.5	+0.34	-1.44	51	34
**6**	thioxanthone as PC[a]	65.6	+ 1.18	-1.11	45	0
**7**	THF as solvent				52	33
**8**	CH_2Cl_2 as solvent[b]				48	45
**9**	EtOAc as solvent				52	31
**10**	Acetone as solvent				54	30
**11**	Et_2O as solvent				54	33
**12**	HFIP as solvent				0	72 (72)
**13**	15 min (reaction time)				47	35
**14**	30 min (reaction time)				51	20
**15**	0.2 M (concentration)				52	26
**16**	0.5 M (concentration)				51	27
**17**	without PC				0	0
**18**	without light				0	0

Yields determined by quantitative NMR spectroscopy of the crude reaction mixture using CH_2Br_2 as the internal standard. Isolated yields in the parentheses. a. 390 nm blue LED was used. b. gram scal, $Ir[dF(CF_3)(ppy)]_2)(dtbpy)PF_6$ (0.5 mol%), 1.5 h.

Supplementary Table S2. Optimisation of the intramolecular cycloaddition of precursor **2f**

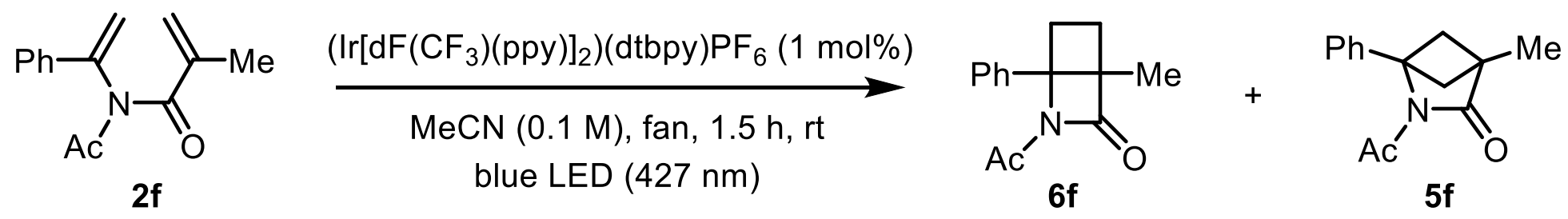

**Entry**	variation	**E$_T$** (kcal/mol)	**E$_{1/2}$ [M*/M$^{•-}$]** (V)	**E$_{1/2}$ [M$^{•+}$/M*]** (V)	**6f** (% yield)	**5f** (% yield)
**1**	$[Ru(bpy)_3](PF_6)_2$ as PC	46.5	+0.77	-0.81	0	0
**2**	$[Ir(ppy)_2(dtbbpy)]PF_6$ as PC	49.2	+0.66	-0.96	36	3
**3**	*fac*-$[Ir(ppy)_3]$ as PC	58.1	+0.31	-1.73	48	5
**4**	$(Ir[dF(CF_3)(ppy)]_2)(dtbpy)PF_6$ as PC	61.8	+1.21	-0.89	80 (76)	8 (5)
**5**	*fac*-$[Ir(dF(ppy))_3]$ as PC	63.5	+0.34	-1.44	80	8
**6**	thioxanthone as PC[a]	65.6	+ 1.18	-1.11	79	8
**7**	THF as solvent				79	10
**8**	CH_2Cl_2 as solvent				80	9
**9**	EtOAc as solvent				80	9
**10**	Acetone as solvent				80	9
**11**	Et_2O as solvent				79	10
**12**	HFIP as solvent				85 (79)	3
**13**	15 min (reaction time)				80	8
**14**	30 min (reaction time)				80	8
**15**	0.2 M (concentration)				80	8
**16**	0.5 M (concentration)				79	9
**17**	without PC				0	0
**18**	without light				0	0

Yields determined by quantitative NMR spectroscopy of the crude reaction mixture using CH_2Br_2 as the internal standard. Isolated yields in the parentheses. a. 390 nm blue LED was used.

For substrates bearing *N*-substituents with varying electronic properties (**Figure 2A in the main text**), the product distributions resulting from the competing 5-*exo*-trig and 6-*endo*-trig cyclisations under the optimal conditions, together with the corresponding product characterisation data, are provided in **Section 2.4 (Synthesis of Cyclisation Products)** of the Supporting Information.

2.2 General Procedure

2.2.1. General Procedure A: Synthesis of Cyclisation Precursors (1)

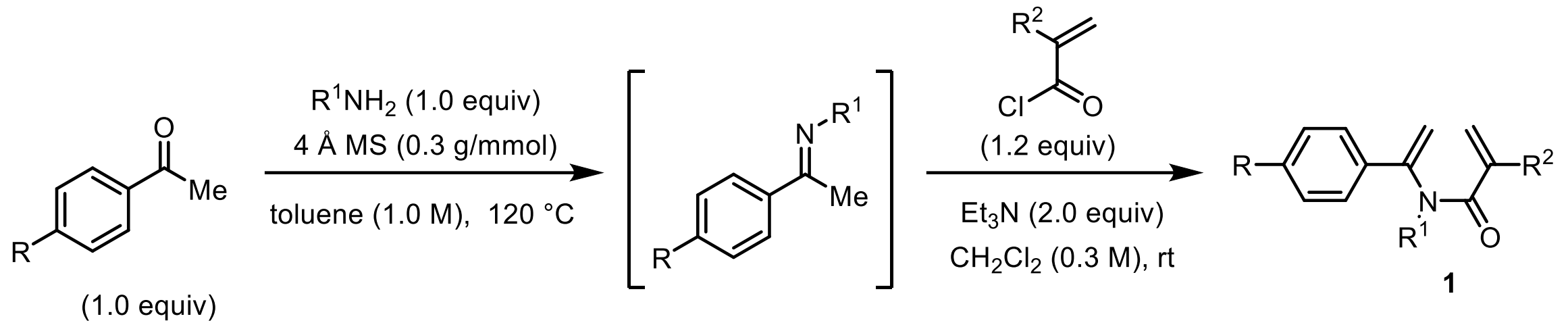

An oven-dried round-bottom flask equipped with a magnetic stir bar and a 15 cm condenser was charged with 4 Å molecular sieves (powder, 0.3 g/mmol) under an argon atmosphere in a glovebox. The flask was sealed with a rubber septum, removed from the glovebox, and kept under a nitrogen atmosphere using a balloon. Anhydrous toluene (1.0 M), the ketone (1.0 equiv), and amine (1.0 equiv) were added sequentially via syringe. The reaction mixture was heated to 120 °C under nitrogen for 5 h, then cooled to room temperature. The resulting mixture was rapidly filtered through anhydrous $MgSO_4$ and washed with anhydrous CH_2Cl_2 (approximately 0.3 M with respect to ketone).[A] The filtrate was collected in an oven-dried round-bottom flask and the crude imine was used directly in the next step without further purification.

The flask containing the crude imine (1.0 equiv) was sealed and kept under a nitrogen atmosphere using a balloon. Triethylamine (2.0 equiv) was added, followed by the acyl chloride (1.2 equiv). The reaction mixture was stirred at room temperature until completion of the reaction (TLC). The reaction was quenched with saturated aqueous $NaHCO_3$ and extracted with CH_2Cl_2 three times. The combined organic extracts were dried over anhydrous $MgSO_4$, filtered, and concentrated *in vacuo*. The crude product was purified using a Biotage automated flash chromatography system to afford the desired products.

Notes: (**A**) Due to the inherent instability of the imine, which is prone to hydrolysis, the crude imine was immediately and rapidly filtered (over $MgSO_4$) upon cooling to room temperature, and the residue was washed with anhydrous CH_2Cl_2. The receiving round-bottom flask was also oven-dried to minimise exposure to moisture and achieve the highest possible yield.

2.2.2. General Procedure B: Synthesis of Cyclisation Precursors (7)

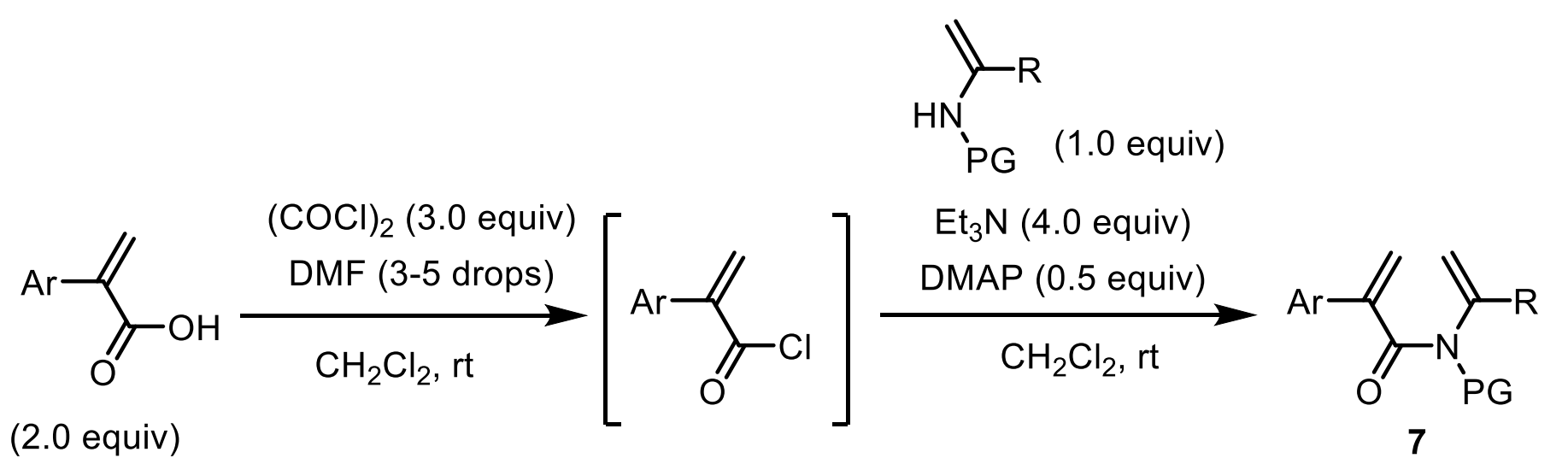

An oven-dried round-bottom flask containing carboxylic acid (2.0 equiv) was sealed and subjected to three N_2 evacuation/refill cycles before pre-sparged anhydrous CH_2Cl_2 (0.3 M with respect to carboxylic acid) was added. The solution was cooled to 0 °C before oxalyl chloride (3.0 equiv) was added, followed by DMF (3-5 drops). The reaction mixture was allowed to warm to room temperature and stirred for 1 h. The reaction was then concentrated under reduced pressure to afford the crude acyl chloride which was used directly for the next step without further purification.

The crude acyl chloride (2.0 equiv) was re-dissolved in anhydrous CH_2Cl_2 (0.3 M with respect to enamide) at room temperature. Et_3N (4.0 equiv) was then added, followed by the addition of the enamide (1.0 equiv) in anhydrous CH_2Cl_2 (1.0 M with respect to enamide) and DMAP (0.5 equiv). The reaction mixture was stirred overnight until completion of the reaction (TLC). The reaction was quenched with brine and extracted with CH_2Cl_2 three times. The combined organic extracts were dried over anhydrous $MgSO_4$ and concentrated *in vacuo*. The crude product was purified using a Biotage automated flash chromatography system to afford the desired product.

2.2.3. General Procedure C: Synthesis of Cyclisation Precursors (2)

2.2.3.1 General Procedure C1: Synthesis of Cyclisation Precursors (2)

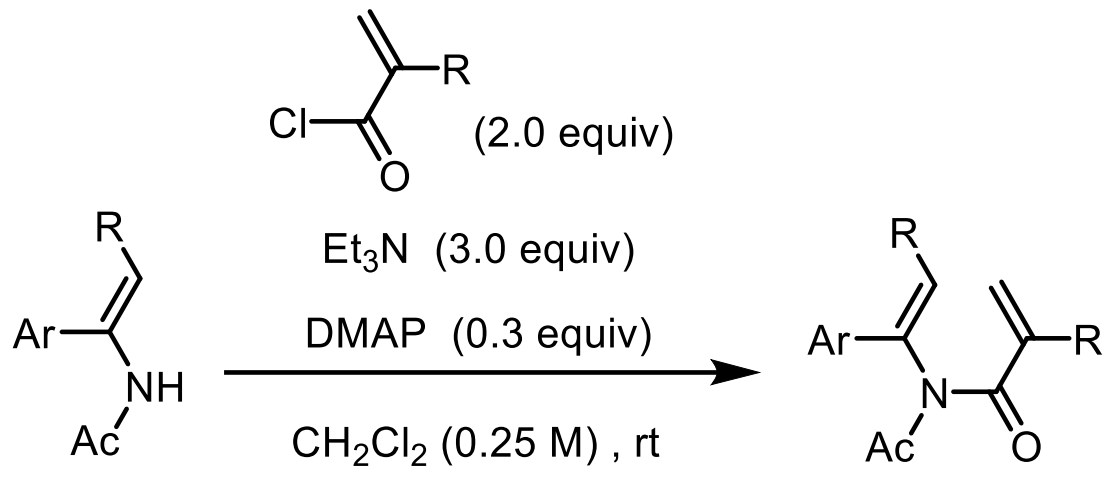

S2
(1.0 equiv)
2

In an oven-dried round-bottom flask, the *N*-acetyl enamide (1.0 equiv) was dissolved in anhydrous CH_2Cl_2 (0.25 M) at room temperature. Et_3N (3.0 equiv) was then added, followed by the addition of acyl chloride (2.0 equiv) and DMAP (0.3 equiv). The reaction mixture was stirred overnight. The reaction was quenched with brine and extracted with CH_2Cl_2 three times. The combined organic extracts were dried over anhydrous $MgSO_4$ and concentrated *in vacuo*. The crude product was purified using a Biotage automated flash chromatography system to afford the needed product **2**.

2.2.3.2 General Procedure C2: Synthesis of Cyclisation Precursors (2)

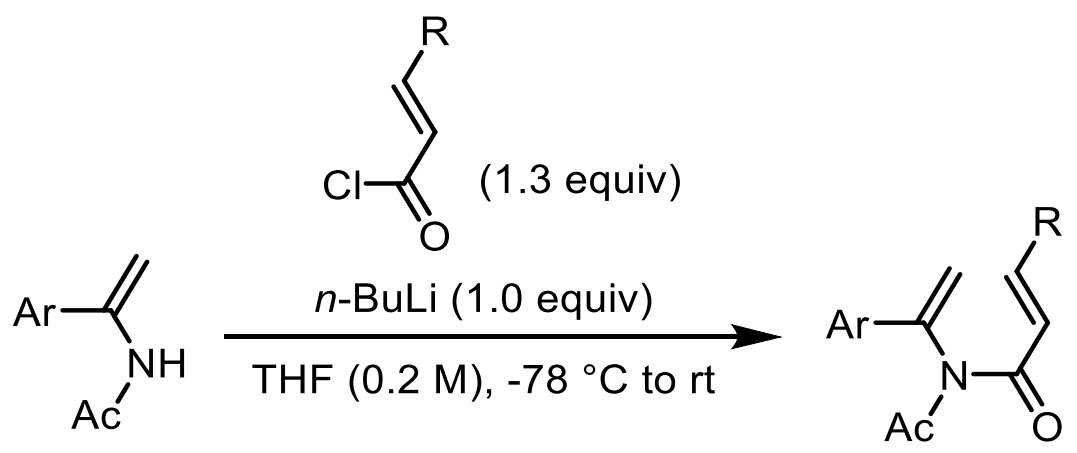

S2
(1.0 equiv)
2

An oven-dried round-bottom flask charged with *N*-acetyl enamide (1.0 equiv) was sealed and subjected to three cycles of evacuation and nitrogen backfilling. Anhydrous THF (0.2 M) was then added, and the reaction mixture was cooled to –78 °C. *n*-BuLi (1.0 equiv) was added dropwise at –78 °C, and the mixture was stirred at this temperature for 10 minutes. Acyl chloride (1.3 equiv) was subsequently added at –78 °C, and the reaction was allowed to warm to room temperature over 1 h. The reaction was then quenched with brine and extracted with ethyl acetate three times. The combined organic layers were dried over anhydrous $MgSO_4$, filtered, and concentrated under reduced pressure. The crude product was purified using a Biotage automated flash chromatography system to afford the desired compound **2**.

2.2.3.3 General Procedure C3: Synthesis of Cyclisation Precursors (2)

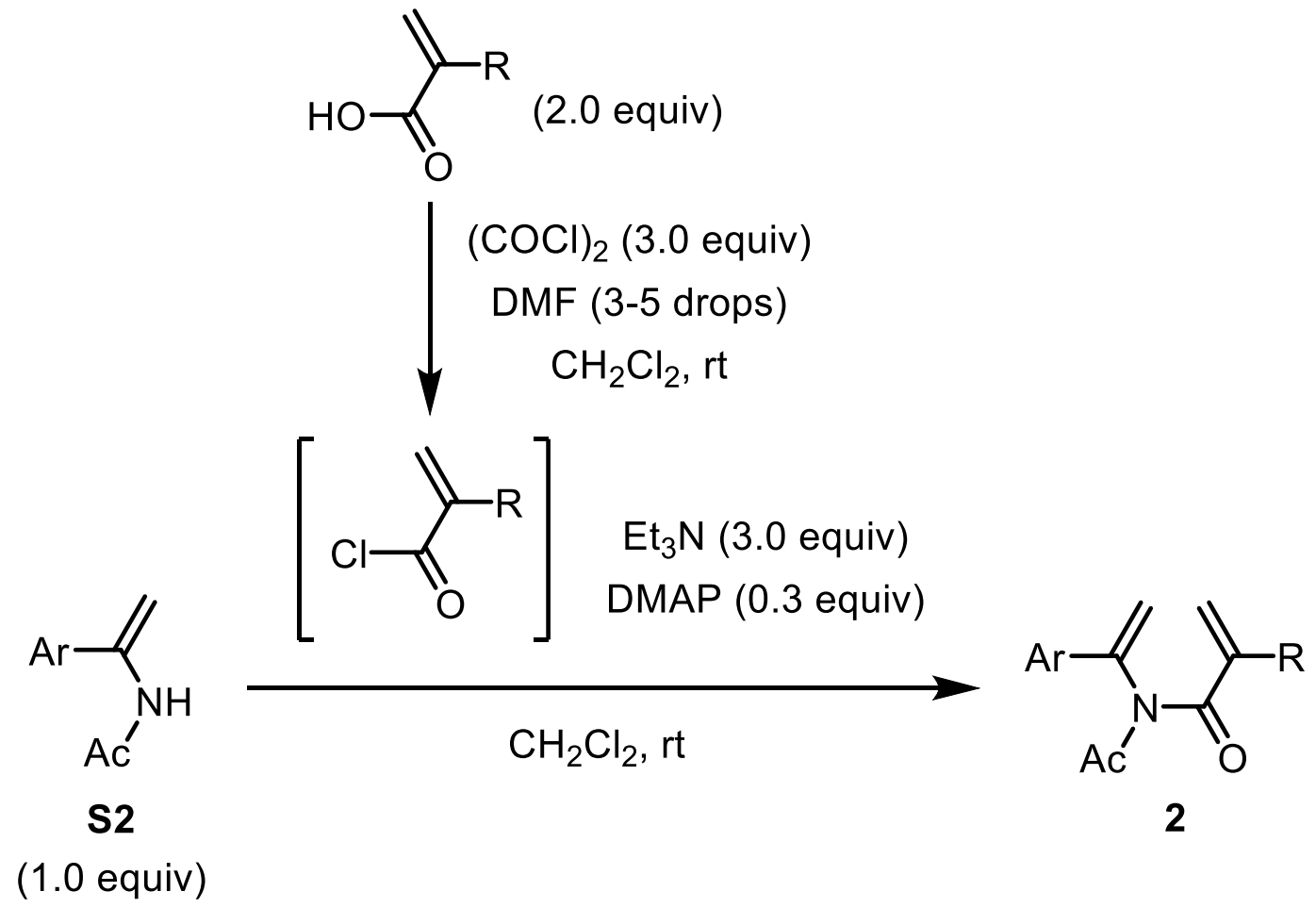

An oven-dried round-bottom flask containing carboxylic acid (2.0 equiv) was sealed and subjected to three N_2 evacuation/refill cycles before pre-sparged anhydrous CH_2Cl_2 (0.3 M with respect to carboxylic acid) was added. Oxalyl chloride (3.0 equiv) was then added, followed by the addition of DMF (3-5 drops). The reaction mixture was allowed to stir at room temperature for 1 h. The reaction was then concentrated under reduced pressure to afford the crude acyl chloride which was used directly for the next step without further purification.

The crude acyl chloride (2.0 equiv) was redissolved in anhydrous CH_2Cl_2 (0.25 M with respect to *N*-acetyl enamide) at room temperature. Et_3N (3.0 equiv) was then added, followed by the addition of *N*-acetyl enamide (1.0 equiv) and DMAP (0.3 equiv). The reaction mixture was stirred overnight until completion of the reaction (TLC). The reaction was quenched with brine and extracted with CH_2Cl_2 three times. The combined organic extracts were dried over anhydrous $MgSO_4$ and concentrated *in vacuo*. The crude product was purified using a Biotage automated flash chromatography system to afford the desired product **2**.

2.2.3.4 General Procedure C4: Synthesis of Cyclisation Precursors (2)

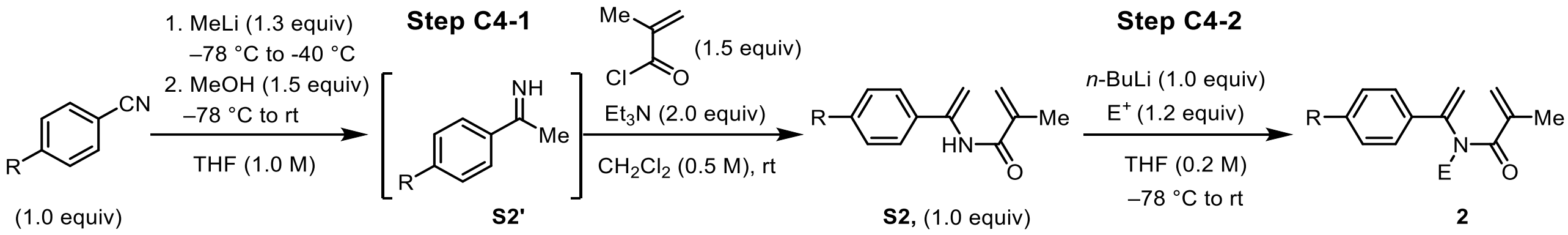

Step C4-1: To an oven-dried round-bottom flask charged with benzonitrile (1.0 equiv) and a magnetic stir bar was added anhydrous THF (1.0 M) under a nitrogen atmosphere. The solution was cooled to –78 °C, and MeLi solution (1.3 equiv) was added dropwise. After stirring at –78 °C for 5 min, the reaction was warmed to –40 °C and stirred for 2 h. Then the mixture was cooled again to –78 °C, followed by the dropwise addition of anhydrous MeOH (1.5 equiv). The reaction was allowed to warm to room temperature over the course of 1 h. The reaction mixture was then immediately filtered through anhydrous $MgSO_4$ under reduced pressure and washed with anhydrous CH_2Cl_2 (approximately 0.5 M with respect to benzonitrile) under a nitrogen atmosphere to prevent decomposition of the imine intermediate.[A] The filtrate was collected in an oven-dried round-bottom flask, sealed with a rubber septum, and placed under a nitrogen atmosphere. Et_3N (2.0 equiv) and methacryloyl chloride (1.5 equiv) were then added to the filtrate under a nitrogen atmosphere. The reaction mixture was stirred at room temperature for 12 h.[B] Upon completion, the reaction mixture was then quenched with brine and extracted with ethyl acetate three times. The combined organic layers were dried over anhydrous $MgSO_4$, filtered, and concentrated under reduced pressure. The crude product was purified using a Biotage automated flash chromatography system to afford *N*-H 1,5-diene **S2**.

Notes: (**A**) The *N*-H imine intermediate **S2’** is unstable at room temperature; hence, the $MgSO_4$ filtration of the crude *N*-H imine intermediate **S2’** should be carried out rapidly under reduced pressure and under a nitrogen atmosphere. The filtrate containing the crude *N*-H imine **S2’** was immediately subjected to acylation without further purification.

(**B**) Although acylation reactions typically do not require long reaction times, we found that upon addition of methacryloyl chloride, the reaction mixture rapidly formed a mixture of enamine **S2** and its imine isomer, as evidenced by crude ^{1}H NMR analysis. Importantly, stirring the reaction at room temperature for 12 h led to substantial conversion of the imine into the enamine, thereby affording **S2** in improved yield.

Step C4-2: An oven-dried round-bottom flask charged with *N*-H 1,5-diene **S2** (1.0 equiv) was sealed and subjected to three cycles of evacuation and nitrogen backfilling. Anhydrous THF (0.2 M) was then added, and the reaction mixture was cooled to –78 °C. *n*-BuLi solution (1.0 equiv) was added dropwise at –78 °C, and the mixture was stirred at this temperature for 10 minutes. The electrophile

(1.2 equiv) was subsequently added at –78 °C, and the reaction was allowed to warm to room temperature over 1 hour. The reaction was then quenched with brine and extracted with ethyl acetate three times. The combined organic layers were dried over anhydrous $MgSO_4$, filtered, and concentrated under reduced pressure. The crude product was purified using a Biotage automated flash chromatography system to afford the desired compound **2**.

2.2.4. General Procedure D: Synthesis of Cyclisation Products

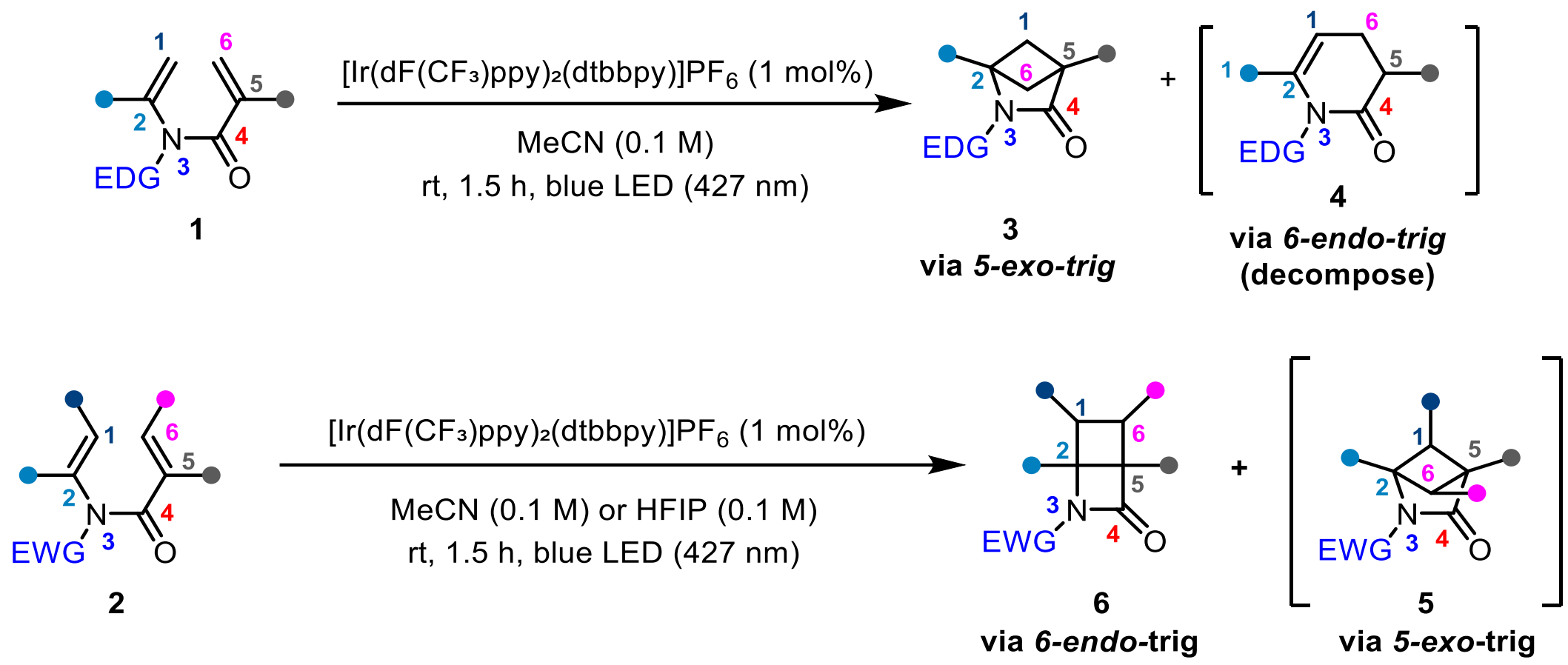

An oven-dried round-bottom flask containing the substrate (1.0 equiv) and $[Ir(dF(CF_3)ppy)_2(dtbbpy)]PF_6$ (1.0 mol%) was sealed and subjected to three evacuation–backfill cycles with N_2. Anhydrous solvent[A] (0.1 M) pre-sparged with N_2 was added, and the flask was positioned ~1 cm from a 40 W KSPR160L-427 nm Kessil light source (100% intensity).[B] The mixture was stirred under continuous irradiation at ambient temperature[C] under N_2 for 1.5 h.[D,E] Concentration *in vacuo* and purification using a Biotage automated flash chromatography system afforded the desired product.[F,G]

Notes: (**A**) All substrates were evaluated in MeCN as the solvent. For substrates exhibiting poor selectivity toward the 6-*endo*-trig pathway, experiments using HFIP as the solvent were additionally performed and are specifically indicated in the corresponding characterisation data.

(**B**) When thioxanthone (0.1 equiv) is used as a photosensitizer, a 40W KSPR160L-390 nm Kessil light (100% intensity) was used. When benzophenone (0.25 equiv) is used as a photosensitizer, a 40W KSPR160L-370 nm Kessil light (100% intensity) was used.

(**C**) The actual temperature of the reaction mixture was higher than room temperature. Fan-assisted cooling was used to maintain the temperature at around 35-40 °C. The solution temperature was monitored with a thermometer probe placed 1 cm below the solvent surface.

(**D**) Most substrates react completely in 1.5 h; substrates requiring extended reaction times are specifically indicated in the subsequent detailed data descriptions.

(**E**) The reaction was typically monitored by TLC. The product is usually slightly more polar than the starting material; however, in many cases, the products and starting precursor exhibit very similar R_f values. A relatively straightforward way to distinguish them is by their UV response under 254 nm light: product spots appear significantly weaker due to the loss of the styrenyl chromophore. Additionally, the product generally shows a slower colour development when treated with the $KMnO_4$ stain, as the reactive alkene moiety has been consumed. Of course, the most reliable method of identification is by sampling the crude reaction mixture for ^{1}H NMR analysis.

(**F**) When 1,5-diene **1** was subjected to the [2+2] cyclisation, the corresponding [2.1.1] bicyclic compound **3** was the only product that could be isolated. The six-membered-ring product (**4**) arising from the 6-*endo*-trig pathway could occasionally be detected only in trace amounts in the crude ^{1}H NMR spectrum. Time-course ^{1}H NMR monitoring at different reaction intervals (15, 30, 60, and 90 min) revealed that this species gradually decomposes over time. Furthermore, when an isolated sample of compound **4** was resubjected to the standard photochemical conditions, substantial decomposition was observed within 1 h. Owing to the intrinsic instability of this compound, except for a few selected substrates that could be isolated and fully characterised, only the yield and characterisation data of the [2.1.1] scaffolds (**3**) are reported.

(**G**) When using 1,5-diene **2** for the [2+2] cyclisation, both the [2.2.0] product (**6**) and the [2.1.1] product (**5**) could be observed in the crude ^{1}H NMR spectrum (shown below) and isolated. All products in this study were ultimately purified using a Biotage automated flash chromatography system equipped with a high-efficiency silica column (Biotage® Sfär Silica HC D High Capacity Duo, 20 µm). When the molar ratio of [2.2.0] product **6** to [2.1.1] product **5** in the crude mixture was less than 5:1, the [2.1.1] isomer **5** was also isolated and fully characterised.

2.3 Synthesis of Cyclisation Precursors

2.3.1 Synthesis and Characterisation of Precursors (1)

***N*-(4-methoxybenzyl)-*N*-(1-(4-(trifluoromethyl)phenyl)vinyl)methacrylamide (1a)**

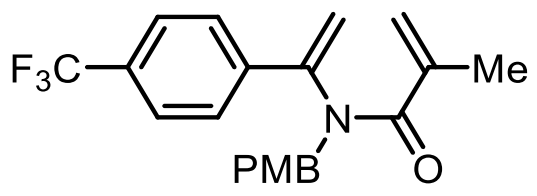

Prepared according to **General Procedure A** using 4-(trifluoromethyl)acetophenone (1.50 g, 8.00 mmol, 1.00 equiv), toluene (8.0 mL), 4-methoxybenzylamine (1.04 mL, 8.00 mmol, 1.00 equiv) and 4 Å molecular sieves (2.40 g). Then using CH_2Cl_2 (approximately 30.0 mL), Et_3N (2.23 mL, 16.0 mmol, 2.00 equiv) and methacryloyl chloride (0.94 mL, 9.60 mmol, 1.20 equiv). The crude reaction mixture was purified using a Biotage automated flash chromatography system with a gradient elution of ethyl acetate/pentane (0% to 35%), affording **1a** as a colourless oil (2.33 g, 6.21 mmol, 78%).

^{1}H NMR (500 MHz, $CDCl_3$) δ 7.60 (d, J = 8.2 Hz, 2H), 7.41 (d, J = 8.1 Hz, 2H), 7.20 (d, J = 8.6 Hz, 2H), 6.82 (d, J = 8.6 Hz, 2H), 5.46 (d, J = 0.9 Hz, 1H), 5.26 (s, 1H), 5.05 (s, 1H), 4.98 (d, J = 0.9 Hz, 1H), 4.73 (s, 2H), 3.78 (s, 3H), 1.73 (s, 3H).

^{13}C NMR (126 MHz, $CDCl_3$) δ 172.4, 159.2, 146.5, 141.2, 140.6, 130.8 (q, J = 32.6 Hz), 130.5, 129.4, 126.8, 125.9 (q, J = 3.8 Hz), 124.0 (q, J = 272.1 Hz), 118.3, 114.0, 113.9, 55.3, 50.5, 20.1.

^{19}F NMR (471 MHz, $CDCl_3$) δ –62.68.

IR (ATR): $\tilde{\nu}$ (cm^{-1}) = 1654, 1612, 1511, 1384, 1246, 1165, 1113, 1063.

HRMS (ESI^+) calcd. for $C_{21}H_{21}F_3NO_2^+$ $[M+H]^+$: 376.1519, found: 376.1519.

***N*-benzyl-*N*-(1-(4-(trifluoromethyl)phenyl)vinyl)methacrylamide (1b)**

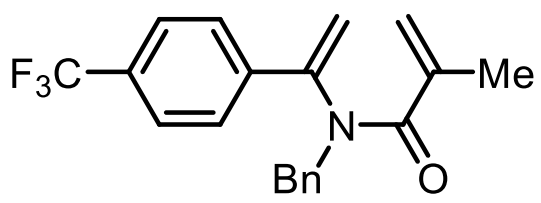

Prepared according to **General Procedure A** using 4-(trifluoromethyl) acetophenone (1.50 g, 8.00 mmol, 1.00 equiv), toluene (8.0 mL), benzylamine (0.87 mL, 8.0 mmol, 1.0 equiv) and 4 Å molecular sieves (2.40 g). Then using CH_2Cl_2 (approximately 30.0 mL), Et_3N (2.23 mL, 16.0 mmol, 2.00 equiv) and methacryloyl chloride (0.94 mL, 9.6 mmol, 1.2 equiv). The crude reaction mixture was purified using a Biotage automated flash chromatography system with a gradient elution of ethyl acetate/pentane (0% to 30%), affording **1b** as a colourless oil (2.13 g, 6.16 mmol, 77%).

^{1}H NMR (500 MHz, $CDCl_3$) δ 7.60 (d, J = 8.2 Hz, 2H), 7.42 (d, J = 8.2 Hz, 2H), 7.32 – 7.23 (m, 5H), 5.46 (s, 1H), 5.30 (s, 1H), 5.07 (s, 1H), 5.02 (s, 1H), 4.81 (s, 2H), 1.75 (s, 3H).

^{13}C NMR (126 MHz, $CDCl_3$) δ 172.3, 146.5, 141.0, 140.6, 137.2, 130.7 (q, *J* = 32.6 Hz), 128.9, 128.5, 127.6, 126.7, 125.8 (q, *J* = 3.8 Hz), 124.0 (q, *J* = 272.1 Hz), 118.3, 113.7, 51.1, 20.0.

^{19}F NMR (471 MHz, $CDCl_3$) δ -62.65.

IR (ATR): $\tilde{\nu}$ (cm^{-1}) = 1656, 1615, 1410, 1385, 1323, 1166, 1115, 1064, 1014.

HRMS (ESI^+) calcd. for $C_{20}H_{19}F_3NO^+$ $[M+H]^+$: 346.1413, found: 346.1400.

***N*-benzyl-*N*-(1-(4-chlorophenyl)vinyl)methacrylamide (1c)**

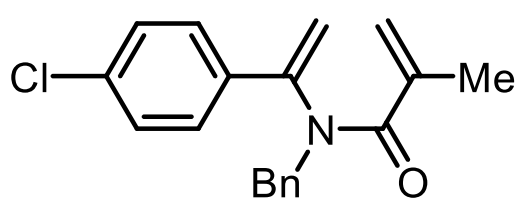

Prepared according to **General Procedure A** using 4-chloroacetophenone (1.24 g, 8.00 mmol, 1.00 equiv), toluene (8.0 mL), benzylamine (0.87 mL, 8.0 mmol, 1.0 equiv) and 4 Å molecular sieves (2.40 g). Then using CH_2Cl_2 (approximately 30.0 mL), Et_3N (2.23 mL, 16.0 mmol, 2.00 equiv) and methacryloyl chloride (0.94 mL, 9.6 mmol, 1.2 equiv). The crude reaction mixture was purified using a Biotage automated flash chromatography system with a gradient elution of ethyl acetate/pentane (0% to 30%), affording **1c** as a colourless oil (1.99 g, 6.40 mmol, 80%).

^{1}H NMR (500 MHz, $CDCl_3$) δ 7.32 – 7.21 (m, 9H), 5.34 (d, *J* = 0.9 Hz, 1H), 5.28 (s, 1H), 5.04 (p, *J* = 1.6 Hz, 1H), 4.89 (d, *J* = 0.9 Hz, 1H), 4.77 (s, 2H), 1.77 – 1.75 (m, 3H).

^{13}C NMR (126 MHz, $CDCl_3$) δ 172.2, 146.4, 141.0, 137.2, 135.3, 134.6, 128.9, 128.8, 128.4, 127.7, 127.4, 117.9, 112.3, 50.8, 20.0.

IR (ATR): $\tilde{\nu}$ (cm^{-1}) = 1652, 1615, 1489, 1382, 1326, 1209, 1196, 1092, 1011.

HRMS (ESI^+) calcd. for $C_{19}H_{19}ClNO^+$ $[M+H]^+$: 312.1150, found: 312.1139.

***N*-benzyl-*N*-(1-(4-cyanophenyl)vinyl)methacrylamide (1d)**

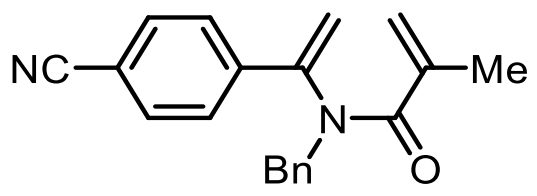

Prepared according to **General Procedure A** using 4-acetylbenzonitrile (1.16 g, 8.00 mmol, 1.00 equiv), toluene (8.0 mL), benzylamine (0.87 mL, 8.0 mmol, 1.0 equiv) and 4 Å molecular sieves (2.40 g). Then using CH_2Cl_2 (approximately 30.0 mL), Et_3N (2.23 mL, 16.0 mmol, 2.00 equiv) and methacryloyl chloride (0.94 mL, 9.6 mmol, 1.2 equiv). The crude reaction mixture was purified using a Biotage automated flash chromatography system with a gradient elution of ethyl acetate/CH_2Cl_2 (0% to 10%), affording **1d** as a colourless oil (1.30 g, 4.30 mmol, 54%).

^{1}H NMR (500 MHz, $CDCl_3$) δ 7.62 (d, *J* = 8.5 Hz, 2H), 7.37 (d, *J* = 8.5 Hz, 2H), 7.31 – 7.25 (m, 5H), 5.47 (d, *J* = 1.1 Hz, 1H), 5.26 (s, 1H), 5.07 (s, 2H), 4.81 (s, 2H), 1.71 (t, *J* = 1.3 Hz, 3H).

^{13}C NMR (126 MHz, $CDCl_3$) δ 172.3, 146.5, 141.7, 141.0, 137.1, 132.6, 128.9, 128.7, 127.8, 127.0, 118.7, 118.5, 114.5, 112.4, 51.4, 20.0.

IR (ATR): $\tilde{\nu}$ (cm^{-1}) = 2228, 1655, 1614, 1495, 1453, 1352, 1261, 1197, 1030.

HRMS (ESI$^+$) calcd. for $C_{20}H_{19}N_2O^+$ [M+H]$^+$: 303.1492, found: 303.1480.

Methyl 4-(1-(*N*-benzylmethacrylamido)vinyl)benzoate (1e)

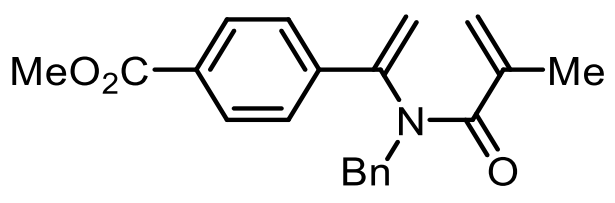

Prepared according to **General Procedure A** using methyl 4-acetylbenzoate (1.42 g, 8.00 mmol, 1.00 equiv), toluene (8.0 mL), benzylamine (0.87 mL, 8.0 mmol, 1.0 equiv) and 4 Å molecular sieves (2.40 g). Then using CH_2Cl_2 (approximately 30.0 mL), Et_3N (2.23 mL, 16.0 mmol, 2.00 equiv) and methacryloyl chloride (0.94 mL, 9.6 mmol, 1.2 equiv). The crude reaction mixture was purified using a Biotage automated flash chromatography system with a gradient elution of Et_2O/pentane (0% to 50%), affording **1e** as a colourless oil (588 mg, 1.76 mmol, 22%).

^{1}H NMR (500 MHz, $CDCl_3$) δ 8.01 (d, *J* = 8.5 Hz, 2H), 7.37 (d, *J* = 8.5 Hz, 2H), 7.31 – 7.25 (m, 5H), 5.46 (d, *J* = 0.9 Hz, 1H), 5.28 (s, 1H), 5.05 (app. p, *J* = 1.6 Hz, 1H), 5.00 (d, *J* = 0.9 Hz, 1H), 4.81 (s, 2H), 3.92 (s, 3H), 1.73 (t, *J* = 1.3 Hz, 3H).

^{13}C NMR (126 MHz, $CDCl_3$) δ 172.5, 166.6, 147.0, 141.5, 141.1, 137.3, 130.4, 130.1, 129.0, 128.6, 127.6, 126.4, 118.4, 113.7, 52.4, 51.2, 20.1.

IR (ATR): $\tilde{\nu}$ (cm^{-1}) = 1721, 1675, 1656, 1609, 1521, 1435, 1277, 1196, 1108.

HRMS (ESI$^+$) calcd. for $C_{21}H_{22}NO_3^+$ [M+H]$^+$: 336.1594, found: 336.1580.

N-benzyl-*N*-(1-(4-fluorophenyl)vinyl)methacrylamide (1f)

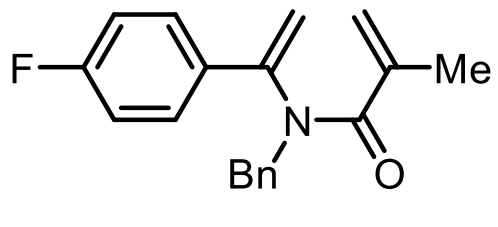

Prepared according to **General Procedure A** using 4-fluoroacetophenone (1.10 g, 8.00 mmol, 1.00 equiv), toluene (8.0 mL), benzylamine (0.87 mL, 8.0 mmol, 1.0 equiv) and 4 Å molecular sieves (2.40 g). Then using CH_2Cl_2 (approximately 30.0 mL), Et_3N (2.23 mL, 16.0 mmol, 2.00 equiv) and methacryloyl chloride (0.94 mL, 9.6 mmol, 1.2 equiv). The crude reaction mixture was purified using

a Biotage automated flash chromatography system with a gradient elution of ethyl acetate/pentane (0% to 30%), affording **1f** as a colourless oil (1.91 g, 6.47 mmol, 81%).

^{1}H NMR (500 MHz, $CDCl_3$) δ 7.31 – 7.23 (m, 7H), 7.06 – 7.00 (m, 2H), 5.30 (s, 1H), 5.29 (s, 1H), 5.05 (p, J = 1.5 Hz, 1H), 4.86 (s, 1H), 4.78 (s, 2H), 1.77 (t, J = 1.4 Hz, 3H).

^{13}C NMR (126 MHz, $CDCl_3$) δ 172.5, 163.0 (d, J = 249.2 Hz), 146.6, 141.2, 137.4, 133.0 (d, J = 3.5 Hz), 128.9, 128.5, 128.3 (d, J = 8.3 Hz), 127.5, 117.9, 115.8 (d, J = 21.8 Hz), 111.7, 50.9, 20.1.

^{19}F NMR (471 MHz, $CDCl_3$) δ -112.38 (tt, J = 8.8, 5.3 Hz).

IR (ATR): $\tilde{\nu}$ (cm^{-1}) = 1652, 1615, 1507, 1384, 1326, 1224, 1196, 1158, 1079.

HRMS (ESI$^+$) calcd. for $C_{19}H_{19}FNO^+$ [M+H]$^+$: 296.1445, found: 296.1433.

***N*-benzyl-*N*-(1-phenylvinyl)methacrylamide (1g)**

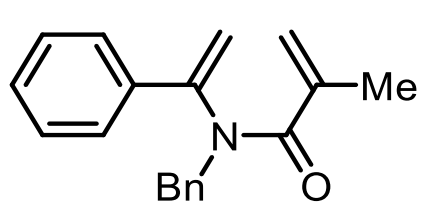

Prepared according to **General Procedure A** using acetophenone (960 mg, 8.00 mmol, 1.00 equiv), toluene (8.0 mL), benzylamine (0.87 mL, 8.0 mmol, 1.0 equiv) and 4 Å molecular sieves (2.40 g). Then using CH_2Cl_2 (approximately 30.0 mL), Et_3N (2.23 mL, 16.0 mmol, 2.00 equiv) and methacryloyl chloride (0.94 mL, 9.6 mmol, 1.2 equiv). The crude reaction mixture was purified using a Biotage automated flash chromatography system with a gradient elution of ethyl acetate/pentane (0% to 30%), affording **1g** as a colourless oil (1.64 g, 5.92 mmol, 74%).

^{1}H NMR (500 MHz, $CDCl_3$) δ 7.37-7.33 (m, 5H), 7.31 – 7.24 (m, 5H), 5.37 (s, 1H), 5.31 (s, 1H), 5.05 (p, J = 1.6 Hz, 1H), 4.87 (s, 1H), 4.79 (s, 2H), 1.78 (t, J = 1.4 Hz, 3H).

^{13}C NMR (126 MHz, $CDCl_3$) δ 172.62, 147.45, 141.19, 137.57, 136.73, 128.96, 128.91, 128.82, 128.43, 127.44, 126.54, 117.83, 112.04, 50.82, 20.20.

IR (ATR): $\tilde{\nu}$ (cm^{-1}) = 1652, 1614, 1494, 1384, 1326, 1197, 1078.

HRMS (ESI$^+$) calcd. for $C_{19}H_{20}NO^+$ [M+H]$^+$: 278.1539, found: 278.1529.

***N*-benzyl-*N*-(1-(*p*-tolyl)vinyl)methacrylamide (1h)**

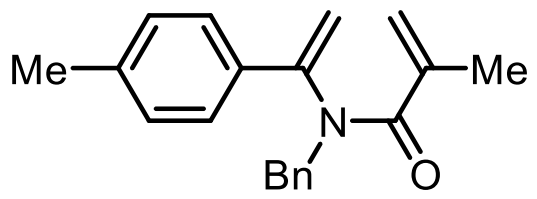

Prepared according to **General Procedure A** using 4-methylacetophenone (1.07 g, 8.00 mmol, 1.00 equiv), toluene (8.0 mL), benzylamine (0.87 mL, 8.0 mmol, 1.0 equiv) and 4 Å molecular sieves (2.40

g). Then using CH_2Cl_2 (approximately 30.0 mL), Et_3N (2.23 mL, 16.0 mmol, 2.00 equiv) and methacryloyl chloride (0.94 mL, 9.6 mmol, 1.2 equiv). The crude reaction mixture was purified using a Biotage automated flash chromatography system with a gradient elution of ethyl acetate/pentane (0% to 30%), affording **1h** as a colourless oil (1.21 g, 4.16 mmol, 52%).

^{1}H NMR (500 MHz, $CDCl_3$) δ 7.31 – 7.24 (m, 7H), 7.18 (d, *J* = 7.8 Hz, 2H), 5.34 (s, 1H), 5.31 (p, *J* = 1.1 Hz, 1H), 5.04 (p, *J* = 1.5 Hz, 1H), 4.80 (s, 1H), 4.78 (s, 2H), 2.38 (s, 3H), 1.81 (t, *J* = 1.3 Hz, 3H).

^{13}C NMR (126 MHz, $CDCl_3$) δ 172.6, 147.2, 141.2, 139.0, 137.6, 133.7, 129.5, 129.0, 128.4, 127.4, 126.4, 117.6, 111.4, 50.6, 21.2, 20.3.

IR (ATR): $\tilde{\nu}$ (cm^{-1}) = 1651, 1614, 1511, 1452, 1385, 1326, 1209, 1198, 1079.

HRMS (ESI^+) calcd. for $C_{20}H_{22}NO^+$ $[M+H]^+$: 292.1696, found: 292.1684.

***N*-benzyl-*N*-(1-(4-methoxyphenyl)vinyl)methacrylamide (1i)**

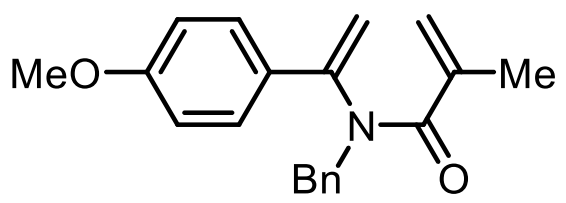

Prepared according to **General Procedure A** using methyl 4-methoxyacetophenone (750 mg, 5.00 mmol, 1.00 equiv), toluene (5.0 mL), benzylamine (0.55 mL, 5.0 mmol, 1.0 equiv) and 4 Å molecular sieves (1.50 g). Then using CH_2Cl_2 (approximately 17.0 mL), Et_3N (1.39 mL, 10.0 mmol, 2.00 equiv) and methacryloyl chloride (0.59 mL, 6.0 mmol, 1.2 equiv). The crude reaction mixture was purified using a Biotage automated flash chromatography system with a gradient elution of Et_2O/pentane (0% to 35%), affording **1i** as a colourless oil (810 mg, 2.64 mmol, 53%).

^{1}H NMR (500 MHz, $CDCl_3$) δ 7.31 – 7.23 (m, 7H), 6.89 (d, *J* = 8.8 Hz, 2H), 5.30 (s, 1H), 5.27 (s, 1H), 5.03 (p, *J* = 1.5 Hz, 1H), 4.77 (s, 2H), 4.75 (s, 1H), 3.82 (s, 3H), 1.81 (s, 3H).

^{13}C NMR (126 MHz, $CDCl_3$) δ 172.6, 160.2, 146.9, 141.2, 137.6, 129.03, 128.97, 128.4, 127.9, 127.4, 117.4, 114.2, 110.5, 55.4, 50.6, 20.3.

IR (ATR): $\tilde{\nu}$ (cm^{-1}) = 1651, 1606, 1510, 1385, 1250, 1175, 1029.

HRMS (ESI^+) calcd. for $C_{20}H_{22}NO_2^+$ $[M+H]^+$: 308.1645, found: 308.1640.

_N_-(4-methoxyphenyl)-_N_-(1-(4-(trifluoromethyl)phenyl)vinyl)methacrylamide (1j)

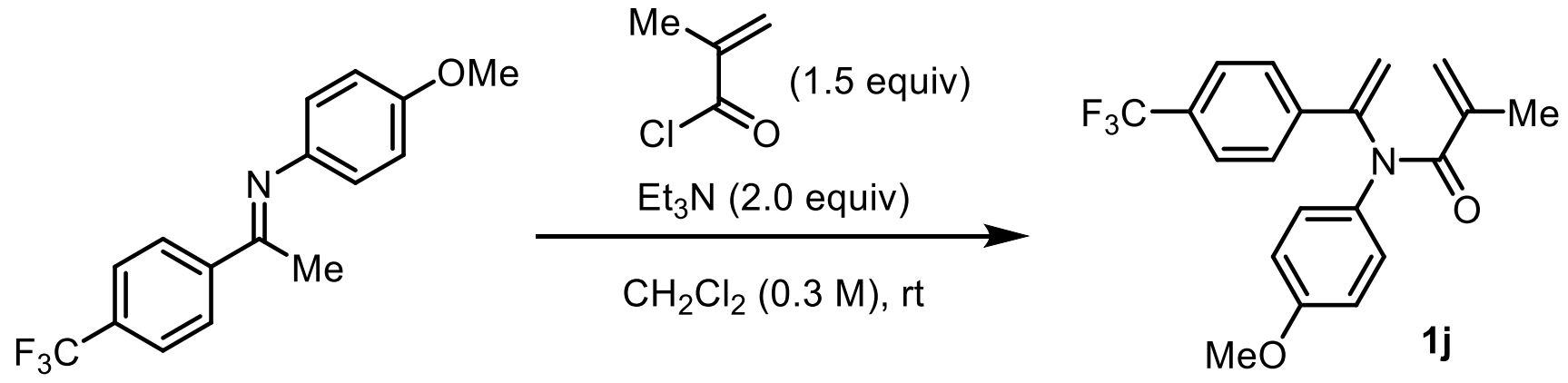

In an oven-dried round-bottom flask, _N_-(4-methoxyphenyl)-1-(4-(trifluoromethyl)phenyl)ethan-1-imine[4] (1.04 g, 3.60 mmol, 1.00 equiv) was dissolved in anhydrous CH_2Cl_2 (12.0 mL) at room temperature. Et_3N (0.99 mL, 7.1 mmol, 2.0 equiv) was then added, followed by the addition of methacryloyl chloride (0.52 mL, 5.3 mmol, 1.5 equiv). The reaction mixture was stirred overnight. The reaction was then quenched with brine (100 mL) and extracted with CH_2Cl_2 (3 × 50 mL). The combined organic extracts were dried over anhydrous $MgSO_4$ and concentrated _in vacuo_. The crude product was purified using a Biotage automated flash chromatography system with a gradient elution of ethyl acetate/pentane (0% to 50%), affording **1j** as a pale-yellow oil (1.25 g, 3.46 mmol, 98%).

^{1}H NMR (500 MHz, $CDCl_3$) δ 7.62 (d, _J_ = 9.1 Hz, 2H), 7.59 (d, _J_ = 8.6 Hz, 2H), 7.15 (d, _J_ = 9.0 Hz, 2H), 6.85 (d, _J_ = 8.9 Hz, 2H), 5.59 (d, _J_ = 1.0 Hz, 1H), 5.41 (s, 1H), 5.21 (app. p, _J_ = 1.4 Hz, 1H), 5.13 (s, 1H), 3.79 (s, 3H), 1.82 (t, _J_ = 1.3 Hz, 3H).

^{13}C NMR (126 MHz, $CDCl_3$) δ 171.8, 158.1, 148.2, 141.0, 140.9, 135.2, 130.1 (q, _J_ = 32.6 Hz), 127.4, 126.2, 125.5 (q, _J_ = 3.8 Hz), 124.0 (q, _J_ = 272.1 Hz), 120.5, 114.4, 113.6, 55.2, 19.6.

^{19}F NMR (471 MHz, $CDCl_3$) δ –62.65.

IR (ATR): $\tilde{\nu}$ (cm^{-1}) = 1660, 1617, 1508, 1321, 1299, 1243, 1165, 1112, 1065.

HRMS (ESI^+) calcd. for $C_{20}H_{19}F_3NO_2^+$ $[M+H]^+$: 362.1362, found: 362.1349.

_N_-benzyl-_N_-(1-(4-cyanophenyl)vinyl)-2-(trifluoromethyl)acrylamide (1k)

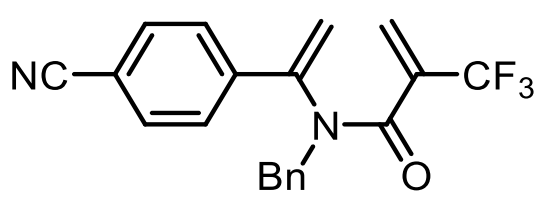

Prepared according to **General Procedure A** using 4-acetylbenzonitrile (870 mg, 6.00 mmol, 1.00 equiv), toluene (6.0 mL), benzylamine (0.65 mL, 6.0 mmol, 1.0 equiv) and 4 Å molecular sieves (1.80 g). Then using CH_2Cl_2 (approximately 20.0 mL), Et_3N (1.67 mL, 12.0 mmol, 2.00 equiv) and 2-(trifluoromethyl)-2-propenoyl chloride[5] (1.14 g, 7.20 mmol, 1.20 equiv).[A] The crude reaction mixture was purified using a Biotage automated flash chromatography system with a gradient elution of acetone/pentane (0% to 35%) and recrystallisation, affording **1k** as a white solid (1.60 g, 4.49 mmol, 75%).

Notes: (**A**) 2-(Trifluoromethyl)-2-propenoyl chloride[5] was purified by distillation before use. It was stored in a glovebox freezer at –30 °C, where it remained stable for at least three months.

M.P.: 115-117 °C

^{1}H NMR (500 MHz, $CDCl_3$) δ 7.67 (d, *J* = 8.5 Hz, 2H), 7.43 (d, *J* = 8.6 Hz, 2H), 7.32 – 7.27 (m, 3H), 7.25 – 7.22 (m, 2H), 5.88 (s, 1H), 5.73 (s, 1H), 5.66 (d, *J* = 1.4 Hz, 1H), 5.08 (s, 1H), 4.80 (s, 2H).

^{13}C NMR (126 MHz, $CDCl_3$) δ 164.3, 144.8, 140.1, 136.0, 134.9 (q, *J* = 31.9 Hz), 133.0, 129.1, 128.8, 128.2, 126.7, 123.8 (q, *J* = 5.3 Hz), 121.5 (q, *J* = 274.1 Hz), 118.3, 117.6, 113.0, 51.1.

^{19}F NMR (471 MHz, $CDCl_3$) δ –64.09.

IR (ATR): $\tilde{\nu}$ (cm^{-1}) = 2231, 1664, 1625, 1396, 1345, 1275, 1263, 1172, 1122.

HRMS (ESI$^+$) calcd. for $C_{20}H_{16}F_3N_2O^+$ [M+H]$^+$: 357.1209, found: 357.1219.

***N*-benzyl-*N*-(1-(4-chlorophenyl)vinyl)-2-(trifluoromethyl)acrylamide (1l)**

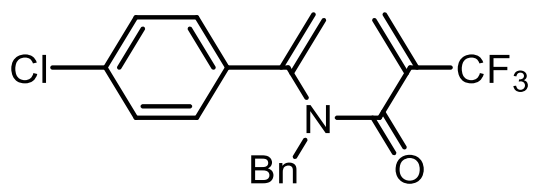

Prepared according to **General Procedure A** using 4-chloroacetophenone (0.78 mL, 6.0 mmol, 1.0 equiv), toluene (6.0 mL), benzylamine (0.65 mL, 6.0 mmol, 1.0 equiv) and 4 Å molecular sieves (1.80 g). Then using CH_2Cl_2 (approximately 20.0 mL), Et_3N (1.67 mL, 12.0 mmol, 2.00 equiv) and 2-(trifluoromethyl)-2-propenoyl chloride[5] (1.14 g, 7.20 mmol, 1.20 equiv).**[A]** The crude reaction mixture was purified using a Biotage automated flash chromatography system with a gradient elution of acetone/pentane (0% to 20%), affording **1l** as a white solid (1.71 g, 4.68 mmol, 78%).

Notes: (**A**) 2-(Trifluoromethyl)-2-propenoyl chloride[5] was purified by distillation before use. It was stored in a glovebox freezer at –30 °C, where it remained stable for at least three months.

M.P.: 44-46 °C

^{1}H NMR (500 MHz, $CDCl_3$) δ 7.37 (d, *J* = 8.5 Hz, 2H), 7.33 – 7.27 (m, 5H), 7.27 – 7.23 (m, 2H), 5.86 (s, 1H), 5.74 (s, 1H), 5.53 (d, *J* = 1.2 Hz, 1H), 4.89 (s, 1H), 4.78 (s, 2H).

^{13}C NMR (126 MHz, $CDCl_3$) δ 164.5, 145.0, 136.3, 135.5, 134.8 (q, *J* = 31.8 Hz), 134.0, 129.4, 129.1, 128.7, 128.0, 127.5, 123.1 (q, *J* = 5.3 Hz), 121.6 (q, *J* = 274.1 Hz), 115.2, 50.6.

^{19}F NMR (471 MHz, $CDCl_3$) δ –64.12.

IR (ATR): $\tilde{\nu}$ (cm^{-1}) = 1663, 1626, 1396, 1342, 1276, 1261, 1171, 1136.

HRMS (ESI$^+$) calcd. for $C_{19}H_{16}ClF_3NO^+$ [M+H]$^+$: 366.0867, found: 366.0860.

_N_-benzyl-_N_-(1-(4-fluorophenyl)vinyl)-2-(trifluoromethyl)acrylamide (1m)

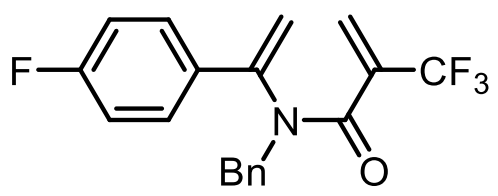

Prepared according to **General Procedure A** using 4-fluoroacetophenone (0.73 mL, 6.0 mmol, 1.0 equiv), toluene (6.0 mL), benzylamine (0.65 mL, 6.0 mmol, 1.0 equiv) and 4 Å molecular sieves (1.80 g). Then using CH_2Cl_2 (approximately 20.0 mL), Et_3N (1.67 mL, 12.0 mmol, 2.00 equiv) and 2-(trifluoromethyl)-2-propenoyl chloride[5] (1.14 g, 7.20 mmol, 1.20 equiv).[**A**] The crude reaction mixture was purified using a Biotage automated flash chromatography system with a gradient elution of CH_2Cl_2/pentane (0% to 100%), affording **1m** as a white solid (1.47 g, 4.21 mmol, 70%).

Notes: (**A**) 2-(Trifluoromethyl)-2-propenoyl chloride[5] was purified by distillation before use. It was stored in a glovebox freezer at –30 °C, where it remained stable for at least three months.

M.P.: 88-90 °C

^{1}H NMR (500 MHz, $CDCl_3$) δ 7.37 – 7.33 (m, 2H), 7.31 – 7.24 (m, 5H), 7.11 – 7.06 (m, 2H), 5.86 (s, 1H), 5.76 (s, 1H), 5.47 (d, J = 1.2 Hz, 1H), 4.87 (s, 1H), 4.79 (s, 2H).

^{13}C NMR (126 MHz, $CDCl_3$) δ 164.5, 163.4 (d, J = 249.9 Hz), 145.1, 136.4, 134.9 (q, J = 31.8 Hz), 131.7 (d, J = 3.5 Hz), 129.1, 128.6, 128.1 (d, J = 8.3 Hz), 127.9, 123.0 (q, J = 5.3 Hz), 121.6 (q, J = 273.9 Hz), 116.2 (d, J = 21.9 Hz), 114.4, 50.7.

^{19}F NMR (471 MHz, $CDCl_3$) δ –64.13, –111.55.

IR (ATR): $\tilde{\nu}$ (cm^{-1}) = 1661, 1628, 1598, 1507, 1404, 1275, 1228, 1125.

HRMS (ESI^+) calcd. for $C_{19}H_{16}F_4NO^+$ $[M+H]^+$: 350.1163, found: 350.1155.

_N_-benzyl-_N_-(1-phenylvinyl)-2-(trifluoromethyl)acrylamide (1n)

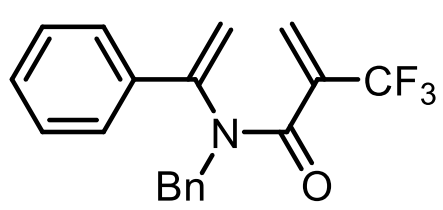

Prepared according to **General Procedure A** using acetophenone (240 mg, 2.00 mmol, 1.00 equiv), toluene (2.0 mL), benzylamine (0.22 mL, 2.0 mmol, 1.0 equiv) and 4 Å molecular sieves (0.60 g). Then using CH_2Cl_2 (approximately 10.0 mL), Et_3N (0.56 mL, 4.0 mmol, 2.00 equiv) and 2-(trifluoromethyl)-2-propenoyl chloride[5] (379 g, 2.40 mmol, 1.20 equiv).[**A**] The crude reaction mixture was purified using a Biotage automated flash chromatography system with a gradient elution of ethyl acetate/pentane (0% to 30%), affording **1n** as a colorless oil (166 mg, 0.50 mmol, 25%).

Notes: (**A**) 2-(Trifluoromethyl)-2-propenoyl chloride[5] was purified by distillation before use. It was stored in a glovebox freezer at –30 °C, where it remained stable for at least three months.

^{1}H NMR (500 MHz, $CDCl_3$) δ 7.46 – 7.42 (m, 5H), 7.34 – 7.28 (m, 5H), 5.87 (s, 1H), 5.81 (s, 1H), 5.57 (d, *J* = 1.0 Hz, 1H), 4.90 (s, 1H), 4.83 (s, 2H).

^{13}C NMR (126 MHz, $CDCl_3$) δ 164.5, 145.9, 136.5, 135.3, 134.8 (q, *J* = 31.7 Hz), 129.4, 129.2, 129.1, 128.6, 127.8, 126.1, 122.8 (q, *J* = 5.5 Hz), 121.7 (d, *J* = 274.1 Hz), 114.7, 50.5.

^{19}F NMR (471 MHz, $CDCl_3$) δ -64.16.

IR (ATR): $\tilde{\nu}$ (cm^{-1}) = 1664, 1633, 1397, 1343, 1276, 1172, 1138, 1122.

HRMS (ESI$^+$) calcd. for $C_{19}H_{17}F_3NO^+$ [M+H]$^+$: 332.1257, found: 332.1245.

***N*-benzyl-*N*-(1-(p-tolyl)vinyl)-2-(trifluoromethyl)acrylamide (1o)**

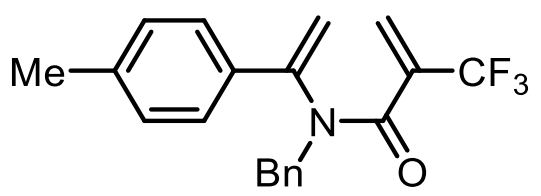

Prepared according to **General Procedure A** using 4-methylacetophenone (0.80 mL, 6.0 mmol, 1.0 equiv), toluene (6.0 mL), benzylamine (0.65 mL, 6.0 mmol, 1.0 equiv) and 4 Å molecular sieves (1.80 g). Then using CH_2Cl_2 (approximately 20.0 mL), Et_3N (1.67 mL, 12.0 mmol, 2.00 equiv) and 2-(trifluoromethyl)-2-propenoyl chloride[5] (1.14 g, 7.20 mmol, 1.20 equiv).[**A**] The crude reaction mixture was purified using a Biotage automated flash chromatography system with a gradient elution of CH_2Cl_2/pentane (0% to 100%), affording **1o** as a white solid (610 mg, 1.77 mmol, 29%).

Notes: (**A**) 2-(Trifluoromethyl)-2-propenoyl chloride[5] was purified by distillation before use. It was stored in a glovebox freezer at –30 °C, where it remained stable for at least three months.

M.P.: 80-82 °C

^{1}H NMR (500 MHz, $CDCl_3$) δ 7.33 – 7.19 (m, 9H), 5.82 (s, 1H), 5.76 (s, 1H), 5.49 (s, 1H), 4.78 (s, 2H), 4.77 (s, 1H), 2.39 (s, 3H).

^{13}C NMR (126 MHz, $CDCl_3$) δ 164.6, 145.7, 139.6, 136.6, 134.8 (q, *J* = 31.8 Hz), 132.4, 129.9, 129.2, 128.6, 127.8, 126.1, 122.6 (q, *J* = 5.3 Hz), 121.7 (q, *J* = 274.1 Hz), 114.0, 50.4, 21.3.

^{19}F NMR (471 MHz, $CDCl_3$) δ –64.20.

IR (ATR): $\tilde{\nu}$ (cm^{-1}) = 1663, 1627, 1395, 1342, 1275, 1170, 1136, 1119.

HRMS (ESI$^+$) calcd. for $C_{20}H_{19}F_3NO^+$ [M+H]$^+$: 346.1413, found: 346.1404.

N-benzyl-N-(1-(4-methoxyphenyl)vinyl)-2-(trifluoromethyl)acrylamide (1p)

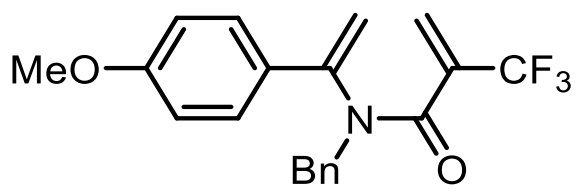

Prepared according to **General Procedure A** using 4-methoxyacetophenone (1.05 g, 7.00 mmol, 1.00 equiv), toluene (7.0 mL), benzylamine (0.76 mL, 7.0 mmol, 1.0 equiv) and 4 Å molecular sieves (2.1 g). Then using CH_2Cl_2 (approximately 25.0 mL), Et_3N (1.95 mL, 14.0 mmol, 2.00 equiv) and 2-(trifluoromethyl)-2-propenoyl chloride[5] (1.33 g, 8.40 mmol, 1.20 equiv).[A] The crude reaction mixture was purified using a Biotage automated flash chromatography system with a gradient elution of ethyl acetate/CH_2Cl_2 (0% to 10%), affording **1p** as a colourless oil (1.38 g, 3.82 mmol, 55%).

Notes: (**A**) 2-(Trifluoromethyl)-2-propenoyl chloride[5] was purified by distillation before use. It was stored in a glovebox freezer at –30 °C, where it remained stable for at least three months.

^{1}H NMR (500 MHz, $CDCl_3$) δ 7.33 (d, J = 8.8 Hz, 2H), 7.31 – 7.24 (m, 5H), 6.93 (d, J = 8.9 Hz, 2H), 5.83 (s, 1H), 5.77 (s, 1H), 5.41 (s, 1H), 4.78 (s, 2H), 4.74 (s, 1H), 3.85 (s, 3H).

^{13}C NMR (126 MHz, $CDCl_3$) δ 164.6, 160.6, 145.4, 136.6, 134.8 (q, J = 31.7 Hz), 129.2, 128.6, 127.79, 127.77, 127.6, 122.5 (q, J = 5.3 Hz), 121.7 (q, J = 273.9 Hz), 114.5, 112.9, 55.5, 50.4.

^{19}F NMR (471 MHz, $CDCl_3$) δ –64.16.

IR (ATR): $\tilde{\nu}$ (cm^{-1}) = 1663, 1627, 1606, 1395, 1277, 1173, 1136, 1120.

HRMS (ESI$^+$) calcd. for $C_{20}H_{19}F_3NO_2^+$ [M+H]$^+$: 362.1362, found: 362.1344.

N-benzyl-N-(1-(4-(trifluoromethyl)phenyl)vinyl)acrylamide (1q)

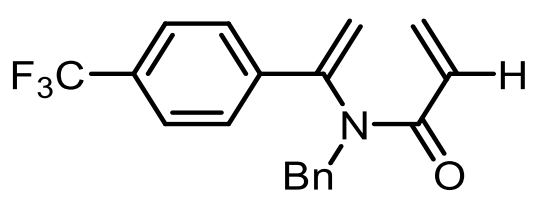

Prepared according to **General Procedure A** using 4-(trifluoromethyl)acetophenone (1.13 g, 6.00 mmol, 1.00 equiv), toluene (6.0 mL), benzylamine (0.65 mL, 6.0 mmol, 1.0 equiv) and 4 Å molecular sieves (1.80 g). Then using CH_2Cl_2 (approximately 20.0 mL), Et_3N (1.67 mL, 12.0 mmol, 2.00 equiv) and acryloyl chloride (0.59 mL, 7.2 mmol, 1.2 equiv). The crude reaction mixture was purified using a Biotage automated flash chromatography system with a gradient elution of ethyl acetate/pentane (0% to 30%), affording **1q** as a colourless oil (1.55 g, 4.68 mmol, 78%).

^{1}H NMR (500 MHz, $CDCl_3$) δ 7.64 (d, J = 8.2 Hz, 2H), 7.50 (d, J = 8.2 Hz, 2H), 7.29 – 7.21 (m, 5H), 6.53 – 6.44 (m, 2H), 5.70 (s, 1H), 5.64 (dd, J = 7.6, 4.8 Hz, 1H), 5.00 (s, 1H), 4.70 (s, 2H).

^{13}C NMR (126 MHz, $CDCl_3$) δ 166.1, 144.5, 139.4, 137.0, 131.2 (q, J = 32.7 Hz), 129.1, 128.8, 128.4, 128.1, 127.6, 126.5, 126.0 (q, J = 3.8 Hz), 123.9 (q, J = 272.1 Hz), 117.5, 49.9.

^{19}F NMR (471 MHz, $CDCl_3$) δ –62.69.

IR (ATR): $\tilde{\nu}$ (cm^{-1}) = 1657, 1616, 1407, 1321, 1275, 1267, 1223, 1166, 1115.

HRMS (ESI^+) calcd. for $C_{19}H_{17}F_3NO^+$ $[M+H]^+$: 332.1257, found: 332.1249.

***N*-benzyl-2-phenyl-*N*-(1-(4-(trifluoromethyl)phenyl)vinyl)acrylamide (1r)**

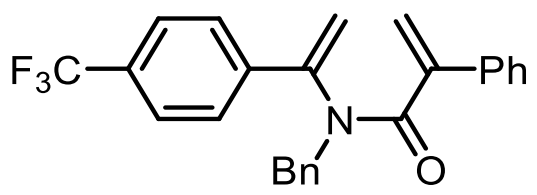

Prepared according to **General Procedure A** using 4-(trifluoromethyl)acetophenone (1.50 g, 8.00 mmol, 1.00 equiv), toluene (8.0 mL), benzylamine (0.87 mL, 8.0 mmol, 1.0 equiv) and 4 Å molecular sieves (2.40 g). Then using CH_2Cl_2 (approximately 30.0 mL), Et_3N (2.23 mL, 16.0 mmol, 2.00 equiv) and crude 2-phenylacryloyl chloride[A] (12.0 mmol, 1.50 equiv) in CH_2Cl_2 (9.0 mL). The crude reaction mixture was purified by two successive flash chromatography separations using a Biotage automated flash chromatography system with a gradient elution of acetone/pentane (0% to 20%) and Et_2O/pentane (0% to 50%), affording **1r** as a colourless oil (1.81 g, 4.45 mmol, 56%).

Notes: (**A**) Crude 2-phenylacryloyl chloride was prepared according to **General Procedure B** (acyl chloride synthesis step) using 2-phenylacrylic acid (1.78 g, 12.0 mmol, 1.50 equiv), CH_2Cl_2 (40.0 mL), oxalyl chloride (1.22 mL, 14.4 mmol, 1.80 equiv) and DMF (5 drops).

^{1}H NMR (500 MHz, $CDCl_3$) δ 7.49 (d, *J* = 8.1 Hz, 2H), 7.33 – 7.21 (m, 12H), 5.46 (s, 1H), 5.39 (s, 1H), 5.37 (s, 1H), 4.84 (s, 2H), 4.79 (s, 1H).

^{13}C NMR (126 MHz, $CDCl_3$) δ 170.7, 145.5, 145.1, 139.7, 137.1, 136.8, 130.7 (q, *J* = 32.4 Hz), 129.1, 128.6, 128.5, 128.3, 127.8, 126.7, 126.2, 125.6 (q, *J* = 3.8 Hz), 124.0 (q, *J* = 272.3 Hz), 116.1, 116.0, 50.4.

^{19}F NMR (471 MHz, $CDCl_3$) δ –62.68.

IR (ATR): $\tilde{\nu}$ (cm^{-1}) = 1651, 1617, 1388, 1324, 1276, 1261, 1167, 1123, 1064.

HRMS (ESI^+) calcd. for $C_{25}H_{21}F_3NO^+$ $[M+H]^+$: 408.1570, found: 408.1563.

2.3.2 Synthesis and Characterisation of Precursors (7)

Methyl 2-(*N*-(*tert*-butoxycarbonyl)-2-phenylacrylamido)acrylate (7a)

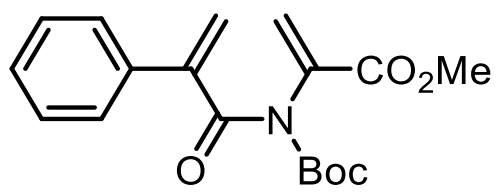

Prepared according to **General Procedure B** using 2-phenylacrylic acid (5.92 g, 40.0 mmol, 2.00 equiv), CH_2Cl_2 (133.0 mL), oxalyl chloride (5.15 mL, 60.0 mmol, 3.00 equiv) and DMF (5 drops). Then using CH_2Cl_2 (67.0 mL), Et_3N (11.2 mL, 80.0 mmol, 4.00 equiv), methyl 2-((*tert*-butoxycarbonyl)amino)acrylate[6] (4.02 g, 20.0 mmol, 1.00 equiv) in CH_2Cl_2 (20.0 mL), and DMAP (1.22 g, 10.0 mmol, 0.50 equiv). The crude reaction mixture was purified by two successive flash chromatography separations using a Biotage automated flash chromatography system with a gradient elution of ethyl acetate/pentane (0% to 30%) and ethyl acetate/CH_2Cl_2 (0% to 10%), affording **7a** (5.10 g, 15.41 mmol, 77%) as a colourless oil.

^{1}H NMR (500 MHz, $CDCl_3$) δ 7.52 – 7.48 (m, 2H), 7.37 – 7.32 (m, 2H), 7.31 – 7.28 (m, 1H), 6.49 (d, J = 0.9 Hz, 1H), 5.79 (s, 1H), 5.78 (d, J = 0.9 Hz, 1H), 5.74 (s, 1H), 3.80 (s, 3H), 1.24 (s, 9H).

^{13}C NMR (126 MHz, $CDCl_3$) δ 171.5, 163.6, 151.2, 146.2, 135.9, 135.5, 128.5, 128.4, 126.7, 126.1, 117.8, 84.3, 52.6, 27.5.

IR (ATR): $\tilde{\nu}$ (cm^{-1}) = 1732, 1689, 1439, 1368, 1275, 1212, 1113, 1091.

HRMS (ESI$^+$) calcd. for $C_{18}H_{21}NO_5Na^+$ [M+Na]$^+$: 354.1312, found: 354.1296.

Methyl 2-(*N*-(*tert*-butoxycarbonyl)-2-(4-fluorophenyl)acrylamido)acrylate (7b)

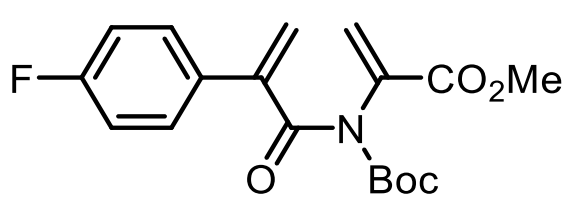

Prepared according to **General Procedure B** using 2-(4-fluorophenyl)acrylic acid[7] (664 mg, 4.00 mmol, 2.00 equiv), CH_2Cl_2 (13.3 mL), oxalyl chloride (0.52 mL, 6.0 mmol, 3.0 equiv) and DMF (3 drops). Then using CH_2Cl_2 (6.70 mL), Et_3N (1.12 mL, 8.00 mmol, 4.00 equiv), methyl 2-((*tert*-butoxycarbonyl)amino)acrylate[6] (402 mg, 2.00 mmol, 1.00 equiv) in CH_2Cl_2 (2.0 mL), and DMAP (122 mg, 1.00 mmol, 0.50 equiv). The crude reaction mixture was purified by two successive flash chromatography separations using a Biotage automated flash chromatography system with a gradient elution of ethyl acetate/pentane (0% to 20%) and Et_2O/CH_2Cl_2 (5% to 10%), affording **7b** (402 mg, 1.15 mmol, 58%) as a colourless oil.

^{1}H NMR (500 MHz, $CDCl_3$) δ 7.50 – 7.45 (m, 2H), 7.03 – 6.98 (m, 2H), 6.46 (d, J = 0.9 Hz, 1H), 5.74 (d, J = 0.9 Hz, 1H), 5.70 (s, 1H), 5.69 (s, 1H), 3.77 (s, 3H), 1.24 (s, 9H).

^{13}C NMR (126 MHz, $CDCl_3$) δ 171.2, 163.4, 162.7 (d, J = 248.0 Hz), 151.1, 145.1, 135.3, 132.1 (d, J = 3.5 Hz), 128.6 (d, J = 8.2 Hz), 126.1, 117.4, 115.3 (d, J = 21.7 Hz), 84.3, 52.6, 27.4.

^{19}F NMR (471 MHz, $CDCl_3$) δ –113.41.

IR (ATR): $\tilde{\nu}$ (cm^{-1}) = 1736, 1692, 1511, 1370, 1275, 1225, 1155, 1114.

HRMS (ESI$^+$) calcd. for $C_{18}H_{20}FNO_5Na^+$ [M+Na]$^+$: 372.1218, found: 372.1201.

Methyl 2-(*N*-(*tert*-butoxycarbonyl)-2-(3-fluorophenyl)acrylamido)acrylate (7c)

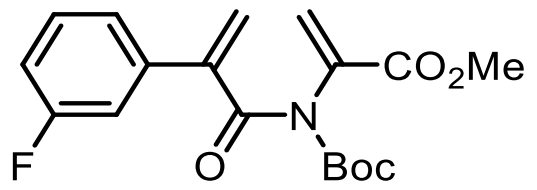

Prepared according to **General Procedure B** using 2-(3-fluorophenyl)acrylic acid[7] (664 mg, 4.00 mmol, 2.00 equiv), CH_2Cl_2 (13.3 mL), oxalyl chloride (0.52 mL, 6.0 mmol, 3.0 equiv) and DMF (3 drops). Then using CH_2Cl_2 (6.70 mL), Et_3N (1.12 mL, 8.00 mmol, 4.00 equiv), methyl 2-((*tert*-butoxycarbonyl)amino)acrylate[6] (402 mg, 2.00 mmol, 1.00 equiv) in CH_2Cl_2 (2.0 mL), and DMAP (122 mg, 1.00 mmol, 0.50 equiv). The crude reaction mixture was purified by two successive flash chromatography separations using a Biotage automated flash chromatography system with a gradient elution of ethyl acetate/pentane (0% to 20%) and Et_2O/CH_2Cl_2 (5% to 10%), affording **7c** (351 mg, 1.01 mmol, 50%) as a colourless oil.

^{1}H NMR (500 MHz, $CDCl_3$) δ 7.33 – 7.27 (m, 2H), 7.25 – 7.21 (m, 1H), 7.01 – 6.96 (m, 1H), 6.50 (d, J = 0.9 Hz, 1H), 5.79 (s, 1H), 5.771 (s, 1H), 5.768 (s, 1H), 3.81 (s, 3H), 1.25 (s, 9H).

^{13}C NMR (126 MHz, $CDCl_3$) δ 170.9, 163.5, 162.9 (d, J = 245.5 Hz), 151.2, 145.2 (d, J = 2.5 Hz), 138.1 (d, J = 7.8 Hz), 135.3, 130.1 (d, J = 8.3 Hz), 126.3, 122.5 (d, J = 3.0 Hz), 118.6, 115.3 (d, J = 21.0 Hz), 113.7 (d, J = 22.6 Hz), 84.5, 52.7, 27.5.

^{19}F NMR (471 MHz, $CDCl_3$) δ –113.05.

IR (ATR): $\tilde{\nu}$ (cm^{-1}) = 1737, 1693, 1581, 1440, 1370, 1284, 1259, 1154, 1109.

HRMS (ESI$^+$) calcd. for $C_{18}H_{20}FNO_5Na^+$ [M+Na]$^+$: 372.1218, found: 372.1203.

Methyl 2-(*N*-(*tert*-butoxycarbonyl)-2-(4-chlorophenyl)acrylamido)acrylate (7d)

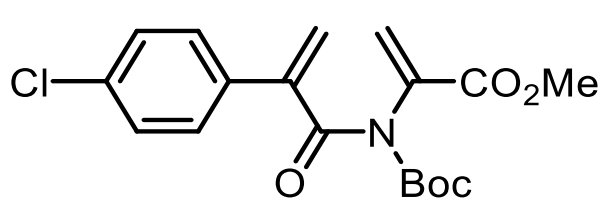

Prepared according to **General Procedure B** using 2-(4-chlorophenyl)acrylic acid[8] (728 mg, 4.00 mmol, 2.00 equiv), CH_2Cl_2 (13.3 mL), oxalyl chloride (0.52 mL, 6.0 mmol, 3.0 equiv) and DMF (3

drops). Then using CH_2Cl_2 (6.70 mL), Et_3N (1.12 mL, 8.00 mmol, 4.00 equiv), methyl 2-((*tert*-butoxycarbonyl)amino)acrylate[6] (402 mg, 2.00 mmol, 1.00 equiv) in CH_2Cl_2 (2.0 mL) and DMAP (122 mg, 1.00 mmol, 0.50 equiv). The crude reaction mixture was purified by two successive flash chromatography separations using a Biotage automated flash chromatography system with a gradient elution of ethyl acetate/pentane (0% to 20%) and Et_2O/CH_2Cl_2 (5% to 10%), affording **7d** (410 mg, 1.12 mmol, 56%) as a colourless oil.

^{1}H NMR (500 MHz, $CDCl_3$) δ 7.44 (d, J = 8.7 Hz, 2H), 7.31 (d, J = 8.6 Hz, 2H), 6.48 (d, J = 0.9 Hz, 1H), 5.76 (d, J = 1.0 Hz, 1H), 5.75 (s, 1H), 5.73 (s, 1H), 3.79 (s, 3H), 1.25 (s, 9H).

^{13}C NMR (126 MHz, $CDCl_3$) δ 171.1, 163.5, 151.2, 145.2, 135.3, 134.4, 134.3, 128.7, 128.1, 126.2, 118.0, 84.4, 52.7, 27.5.

IR (ATR): $\tilde{\nu}$ (cm^{-1}) = 1735, 1691, 1581, 1493, 1369, 1285, 1257, 1154, 1111.

HRMS (ESI$^+$) calcd. for $C_{18}H_{20}ClNO_5Na^+$ $[M+Na]^+$: 388.0922, found: 388.0905.

Methyl 2-(*N*-(*tert*-butoxycarbonyl)-2-(*p*-tolyl)acrylamido)acrylate (7e)

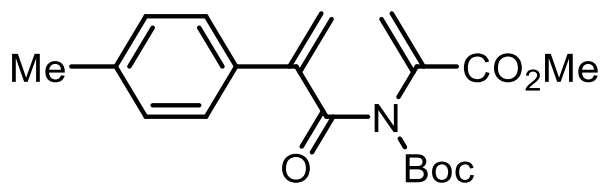

Prepared according to **General Procedure B** using 2-(*p*-tolyl)acrylic acid (648 mg, 4.00 mmol, 2.00 equiv)[7], CH_2Cl_2 (13.3 mL), oxalyl chloride (0.52 mL, 6.0 mmol, 3.0 equiv) and DMF (3 drops). Then using CH_2Cl_2 (6.70 mL), Et_3N (1.12 mL, 8.00 mmol, 4.00 equiv), methyl 2-((*tert*-butoxycarbonyl)amino)acrylate[6] (402 mg, 2.00 mmol, 1.00 equiv) in CH_2Cl_2 (2.0 mL) and DMAP (122 mg, 1.00 mmol, 0.50 equiv). The crude reaction mixture was purified using a Biotage automated flash chromatography system with a gradient elution of ethyl acetate/pentane (0% to 20%), affording **7e** (450 mg, 1.30 mmol, 65%) as a colourless oil.

^{1}H NMR (500 MHz, $CDCl_3$) δ 7.39 (d, J = 8.2 Hz, 2H), 7.15 (d, J = 8.0 Hz, 2H), 6.48 (s, 1H), 5.77 (s, 1H), 5.75 (d, J = 1.0 Hz, 1H), 5.68 (d, J = 1.1 Hz, 1H), 3.80 (s, 3H), 2.33 (s, 3H), 1.26 (s, 9H).

^{13}C NMR (126 MHz, $CDCl_3$) δ 171.7, 163.6, 151.3, 146.1, 138.3, 135.5, 133.0, 129.2, 126.6, 126.1, 116.6, 84.3, 52.6, 27.5, 21.3.

IR (ATR): $\tilde{\nu}$ (cm^{-1}) = 1734, 1691, 1643, 1515, 1439, 1369, 1286, 1211, 1155, 1084, 975.

HRMS (ESI$^+$) calcd. for $C_{19}H_{23}NO_5Na^+$ $[M+Na]^+$: 368.1468, found: 368.1451.

Methyl 2-(*N*-(*tert*-butoxycarbonyl)-2-(4-methoxyphenyl)acrylamido)acrylate (7f)

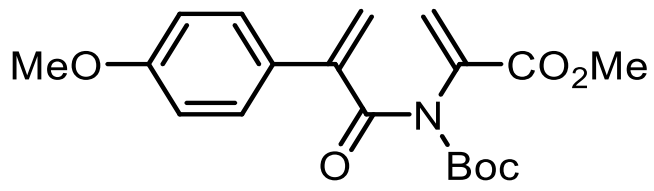

Prepared according to **General Procedure B** using 2-(4-methoxyphenyl)acrylic acid[7] (712 mg, 4.00 mmol, 2.00 equiv), CH_2Cl_2 (13.3 mL), oxalyl chloride (0.52 mL, 6.0 mmol, 3.0 equiv) and DMF (3 drops). Then using CH_2Cl_2 (6.70 mL), Et_3N (1.12 mL, 8.00 mmol, 4.00 equiv), methyl 2-((*tert*-butoxycarbonyl)amino)acrylate[6] (402 mg, 2.00 mmol, 1.00 equiv) in CH_2Cl_2 (2.0 mL) and DMAP (122 mg, 1.00 mmol, 0.50 equiv). The crude reaction mixture was purified using a Biotage automated flash chromatography system with a gradient elution of ethyl acetate/pentane (0% to 30%), affording **7f** (430 mg, 1.19 mmol, 60%) as a colourless oil.

^{1}H NMR (500 MHz, $CDCl_3$) δ 7.44 (d, *J* = 8.9 Hz, 2H), 6.87 (d, *J* = 8.8 Hz, 2H), 6.48 (s, 1H), 5.77 (s, 1H), 5.69 (s, 1H), 5.62 (s, 1H), 3.81 (s, 3H), 3.79 (s, 3H), 1.27 (s, 9H).

^{13}C NMR (126 MHz, $CDCl_3$) δ 171.8, 163.6, 159.9, 151.4, 145.7, 135.5, 128.5, 128.1, 126.1, 115.6, 113.9, 84.3, 55.4, 52.7, 27.6.

IR (ATR): $\tilde{\nu}$ (cm^{-1}) = 1733, 1690, 1608, 1514, 1440, 1369, 1279, 1216, 1154, 1113.

HRMS (ESI$^+$) calcd. for $C_{19}H_{23}NO_6Na^+$ [M+Na]$^+$: 384.1418, found: 384.1406.

Methyl 2-(2-phenyl-*N*-tosylacrylamido)acrylate (7g)

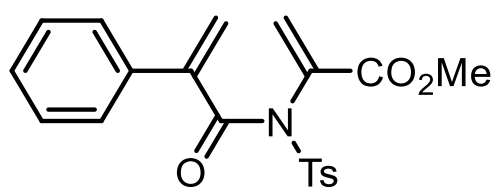

Prepared according to **General Procedure B** using 2-phenylacrylic acid (4.74 g, 32.0 mmol, 2.00 equiv), CH_2Cl_2 (100.0 mL), oxalyl chloride (4.06 mL, 48.0 mmol, 3.00 equiv) and DMF (6 drops). Then using CH_2Cl_2 (54.0 mL), Et_3N (8.92 mL, 64.0 mmol, 4.00 equiv), methyl 2-((4-methylphenyl)sulfonamido)acrylate[9] (4.10 g, 16.0 mmol, 1.00 equiv) in CH_2Cl_2 (16.0 mL) and DMAP (976 mg, 8.00 mmol, 0.50 equiv). The crude reaction mixture was purified using a Biotage automated flash chromatography system with a gradient elution of ethyl acetate/pentane (0% to 30%), affording **7g** (1.55 g, 4.03 mmol, 25%) as a colourless oil.

^{1}H NMR (500 MHz, $CDCl_3$) δ 8.02 (d, *J* = 8.4 Hz, 2H), 7.35 – 7.31 (m, 2H), 7.30 – 7.25 (m, 3H), 7.23 – 7.19 (m, 2H), 6.40 (d, *J* = 0.9 Hz, 1H), 5.67 (d, *J* = 0.9 Hz, 1H), 5.61 (s, 1H), 5.45 (s, 1H), 3.63 (s, 3H), 2.43 (s, 3H).

^{13}C NMR (126 MHz, $CDCl_3$) δ 168.4, 163.3, 145.3, 144.1, 135.2, 134.7, 133.6, 132.0, 129.7, 129.2, 128.9, 128.7, 126.3, 119.0, 52.7, 21.7.

IR (ATR): $\tilde{\nu}$ (cm^{-1}) = 1733, 1699, 1631, 1438, 1362, 1321, 1206, 1166, 1084, 1048.

HRMS (ESI$^+$) calcd. for $C_{20}H_{19}NO_5SNa^+$ [M+Na]$^+$: 408.0876, found: 408.0857.

_N_-benzyl-2-phenyl-_N_-(prop-1-en-2-yl)acrylamide (7h)

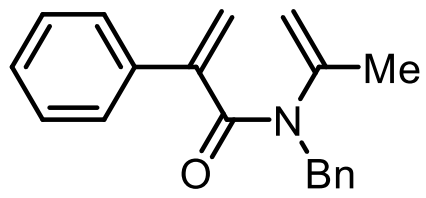

Prepared according to **General Procedure A** using acetone (1.18 mL, 16.00 mmol, 2.00 equiv), toluene (8.0 mL), benzylamine (0.87 mL, 8.0 mmol, 1.0 equiv) and 4 Å molecular sieves (2.40 g). Then using CH_2Cl_2 (approximately 30.0 mL), Et_3N (2.23 mL, 16.0 mmol, 2.00 equiv) and 2-phenylacryloyl chloride**[A]** (1.50 equiv) in CH_2Cl_2 (9.0 mL). The crude reaction mixture was purified using a Biotage automated flash chromatography system with a gradient elution of ethyl acetate/pentane (0% to 30%), affording **7h** as a colourless oil (1.84 g, 6.64 mmol, 83%).

Notes: (**A**) Crude 2-phenylacryloyl chloride was prepared according to **General Procedure B** (acyl chloride synthesis step) using 2-phenylacrylic acid (1.78 g, 12.0 mmol, 1.50 equiv), CH_2Cl_2 (40.0 mL), oxalyl chloride (1.22 mL, 14.4 mmol, 1.80 equiv) and DMF (5 drops). The crude 2-phenylacryloyl chloride was rapidly purified using a Biotage automated flash chromatography system with a gradient elution of ethyl acetate/pentane (0% to 30%). Owing to the instability of the acyl chloride on silica gel, the chromatography was completed within 10 min of loading the sample onto the column. The acyl chloride was concentrated under reduced pressure and used immediately without further storage.

^{1}H NMR (500 MHz, CD_2Cl_2) δ 7.46 – 7.42 (m, 2H), 7.37 – 7.26 (m, 8H), 5.64 (s, 1H), 5.48 (s, 1H), 4.75 (s, 2H), 4.73 (s, 1H), 4.53 (s, 1H), 1.63 (s, 3H).

^{13}C NMR (126 MHz, CD_2Cl_2) δ 170.1, 146.5, 144.0, 138.2, 137.4, 128.9, 128.8, 128.7, 128.5, 127.6, 126.7, 115.7, 115.4, 49.5, 20.6.

IR (ATR): $\tilde{\nu}$ (cm^{-1}) = 1638, 1495, 1387, 1252, 1212, 1076, 1029.

HRMS (ESI$^+$) calcd. for $C_{19}H_{20}NO^+$ [M+H]$^+$: 278.1539, found: 278.1529.

2.3.3 Synthesis and Characterisation of Precursors (2)

***N*-(1-(4-(trifluoromethyl)phenyl)vinyl)methacrylamide (S2a)**

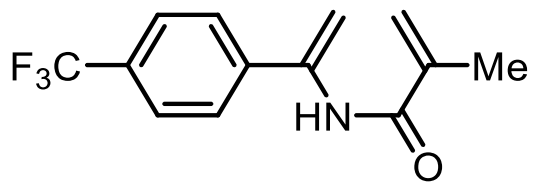

Prepared according to **General Procedure C4 (step C4-1)** using 4-(trifluoromethyl)benzonitrile (3.42 g, 20.0 mmol, 1.00 equiv), THF (20.0 mL), MeLi (8.40 mL, 3.1 M in diethoxymethane, 26.0 mmol, 1.30 equiv), MeOH (1.22 mL, 30.0 mmol, 1.50 equiv), CH_2Cl_2 (ca. 30–40 mL), Et_3N (5.58 mL, 40.0 mmol, 2.00 equiv) and methacryloyl chloride (2.93 mL, 30.0 mmol, 1.50 equiv). Purification using a Biotage automated flash chromatography system (ethyl acetate/pentane, 0% to 25%) afforded **S2a** (2.70 g, 10.6 mmol, 53%) as a white solid.

M.P.: 66-68 °C

^{1}H NMR (500 MHz, $CDCl_3$) δ 7.60 (d, *J* = 8.2 Hz, 2H), 7.51 (d, *J* = 8.2 Hz, 2H), 7.41 (s, 1H), 5.81 (s, 1H), 5.74 (s, 1H), 5.42 (q, *J* = 1.7 Hz, 1H), 5.18 (s, 1H), 1.99 (s, 3H).

^{13}C NMR (126 MHz, $CDCl_3$) δ 167.3, 141.8, 140.4, 139.8, 130.6 (q, *J* = 32.5 Hz), 126.4, 125.7 (q, *J* = 3.8 Hz), 124.0 (q, *J* = 272.1 Hz), 120.3, 105.5, 18.7.

^{19}F NMR (471 MHz, $CDCl_3$) δ –62.68.

IR (ATR): $\tilde{\nu}$ (cm^{-1}) = 1664, 1619, 1500, 1320, 1163, 1114, 1064, 1015.

HRMS (ESI^+) calcd. for $C_{13}H_{13}F_3NO^+$ $[M+H]^+$: 256.0944, found: 256.0941.

***Tert*-butyl methacryloyl(1-(4-(trifluoromethyl)phenyl)vinyl)carbamate (2a)**

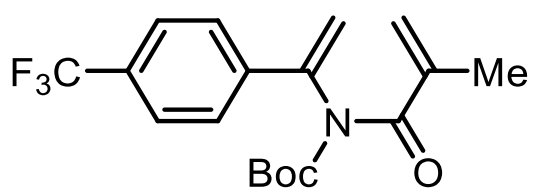

Prepared according to **General Procedure C4 (step C4-2)** using **S2a** (510 mg, 2.00 mmol, 1.00 equiv) and THF (10.0 mL), then *n*-BuLi (0.80 mL, 2.5 M in hexane, 2.0 mmol, 1.0 equiv), and di-tert-butyl dicarbonate (523 mg, 2.40 mmol, 1.20 equiv). Purification using a Biotage automated flash chromatography system (ethyl acetate/pentane, 0% to 30%) afforded **2a** (590 mg, 1.66 mmol, 83 %) as a white solid.

M.P.: 75-77 °C

^{1}H NMR (500 MHz, $CDCl_3$) δ 7.60 (d, *J* = 8.4 Hz, 2H), 7.55 (d, *J* = 8.5 Hz, 2H), 5.74 (d, *J* = 1.2 Hz, 1H), 5.58 (s, 1H), 5.41 (q, *J* = 1.8 Hz, 1H), 5.29 (d, *J* = 1.3 Hz, 1H), 2.07 (s, 3H), 1.31 (s, 9H).

^{13}C NMR (126 MHz, $CDCl_3$) δ 173.8, 152.6, 143.5, 142.5, 140.9, 130.7 (q, J = 32.5 Hz), 126.2, 125.7 (q, J = 3.8 Hz), 124.1 (q, J = 272.1 Hz), 119.4, 115.5, 83.9, 27.7, 19.0.

^{19}F NMR (471 MHz, $CDCl_3$) δ –62.68.

IR (ATR): $\tilde{\nu}$ (cm^{-1}) = 1737, 1690, 1636, 1370, 1323, 1276, 1155, 1115, 1064.

HRMS (ESI^+) calcd. for $C_{18}H_{20}F_3NO_3Na^+$ $[M+Na]^+$: 378.1287, found: 378.1285.

Benzyl methacryloyl(1-(4-(trifluoromethyl)phenyl)vinyl)carbamate (2b)

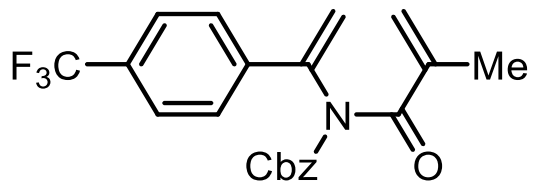

Prepared according to **General Procedure C4 (step C4-2)** using **S2a** (510 mg, 2.00 mmol, 1.00 equiv) and THF (10.0 mL), then *n*-BuLi (0.80 mL, 2.5 M in hexane, 2.0 mmol, 1.0 equiv) and benzyl chloroformate (0.34 mL, 2.4 mmol, 1.2 equiv). Purification using a Biotage automated flash chromatography system (ethyl acetate/pentane, 0% to 30%) afforded **2b** (660 mg, 1.70 mmol, 85 %) as a white solid.

M.P.: 58-60 °C

^{1}H NMR (500 MHz, $CDCl_3$) δ 7.55 (d, J = 8.2 Hz, 2H), 7.49 (d, J = 8.1 Hz, 2H), 7.31 – 7.23 (m, 3H), 7.09 – 7.05 (m, 2H), 5.79 (d, J = 1.4 Hz, 1H), 5.55 (s, 1H), 5.39 (q, J = 1.6 Hz, 1H), 5.31 (d, J = 1.4 Hz, 1H), 5.10 (s, 2H), 2.04 (dd, J = 1.6, 1.0 Hz, 3H).

^{13}C NMR (126 MHz, $CDCl_3$) δ 173.5, 153.9, 143.0, 142.0, 140.3 (q, J = 1.6 Hz), 134.5, 130.7 (q, J = 32.5 Hz), 128.7, 128.6, 128.5, 126.0, 125.8 (q, J = 3.8 Hz), 124.1 (q, J = 272.1 Hz), 120.0, 116.1, 69.1, 18.9.

^{19}F NMR (471 MHz, $CDCl_3$) δ –62.64.

IR (ATR): $\tilde{\nu}$ (cm^{-1}) = 1739, 1694, 1636, 1456, 1408, 1380, 1322, 1261, 1166, 1115.

HRMS (ESI^+) calcd. for $C_{21}H_{19}F_3NO_3^+$ $[M+H]^+$: 390.1312, found: 390.1315.

Phenyl methacryloyl(1-(4-(trifluoromethyl)phenyl)vinyl)carbamate (2c)

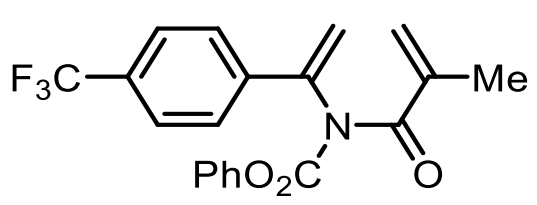

Prepared according to **General Procedure C4 (step C4-2)** using **S2a** (510 mg, 2.00 mmol, 1.00 equiv) and THF (10.0 mL), then *n*-BuLi (0.80 mL, 2.5 M in hexane, 2.0 mmol, 1.0 equiv) and phenyl chloroformate (0.30 mL, 2.4 mmol, 1.2 equiv). Purification using a Biotage automated flash

chromatography system (ethyl acetate/pentane, 0% to 30%) afforded **2c** (610 mg, 1.63 mmol, 81 %) as a white solid.

M.P.: 88-90 °C

^{1}H NMR (500 MHz, $CDCl_3$) δ 7.67 (s, 4H), 7.35 – 7.30 (m, 2H), 7.24 – 7.19 (m, 1H), 6.93 – 6.89 (m, 2H), 5.90 (d, J = 1.5 Hz, 1H), 5.74 (d, J = 1.2 Hz, 1H), 5.54 (q, J = 1.6 Hz, 1H), 5.49 (d, J = 1.5 Hz, 1H), 2.13 (t, J = 1.3 Hz, 3H).

^{13}C NMR (126 MHz, $CDCl_3$) δ 173.3, 152.6, 150.3, 142.9, 142.0, 140.2, 131.0 (q, J = 32.7 Hz), 129.6, 126.5, 126.2, 125.9 (q, J = 3.8 Hz), 124.0 (q, J = 272.1 Hz), 120.9, 120.4, 116.5, 18.9.

^{19}F NMR (471 MHz, $CDCl_3$) δ –62.66.

IR (ATR): $\tilde{\nu}$ (cm^{-1}) = 1750, 1701, 1637, 1618, 1490, 1409, 1325, 1264, 1158, 1115, 1062.

HRMS (ESI^+) calcd. for $C_{20}H_{17}F_3NO_3^+$ $[M+H]^+$: 376.1155, found: 376.1152.

***N*-acetyl-*N*-(1-(4-(trifluoromethyl)phenyl)vinyl)methacrylamide (2d)**

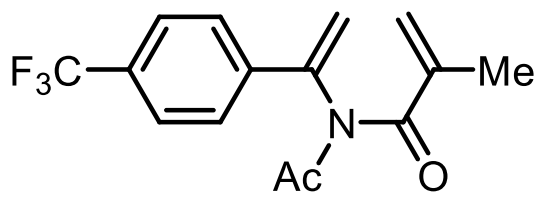

Prepared according to **General Procedure C1** using *N*-(1-(4-(trifluoromethyl)phenyl)vinyl) acetamide[10] (458 mg, 2.00 mmol, 1.00 equiv) and CH_2Cl_2 (8.0 mL), then Et_3N (0.84 mL, 6.0 mmol, 3.0 equiv), methacryloyl chloride (0.39 mL, 4.0 mmol, 2.0 equiv), and DMAP (73 mg, 0.60 mmol, 0.30 equiv). Purification using a Biotage automated flash chromatography system (ethyl acetate/pentane, 0% to 30%) afforded **2d** (499 mg, 1.68 mmol, 84 %) as a white solid.

M.P.: 60-62 °C

^{1}H NMR (500 MHz, $CDCl_3$) δ 7.63 (d, J = 8.1 Hz, 2H), 7.54 (d, J = 8.1 Hz, 2H), 5.92 (d, J = 1.4 Hz, 1H), 5.52 (s, 1H), 5.37 (q, J = 1.6 Hz, 1H), 5.33 (d, J = 1.3 Hz, 1H), 2.35 (s, 3H), 1.94 (t, J = 1.3 Hz, 3H).

^{13}C NMR (126 MHz, $CDCl_3$) δ 174.1, 173.4, 143.8, 141.2, 139.6 (q, J = 1.4 Hz), 131.1 (q, J = 32.7 Hz), 126.1 (q, J = 3.8 Hz), 126.0, 124.0 (q, J = 272.3 Hz), 121.0, 116.9, 25.4, 19.2.

^{19}F NMR (471 MHz, $CDCl_3$) δ -62.79.

IR (ATR): $\tilde{\nu}$ (cm^{-1}) = 1698, 1633, 1410, 1369, 1324, 1256, 1166, 1119, 1067, 1014.

HRMS (ESI^+) calcd. for $C_{15}H_{15}F_3NO_2^+$ $[M+H]^+$: 298.1049, found: 298.1041.

2,2,2-Trichloroethyl methacryloyl(1-(4-(trifluoromethyl)phenyl)vinyl)carbamate (2e)

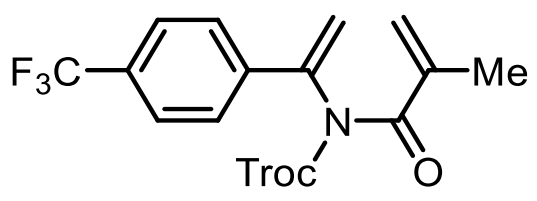

Prepared according to **General Procedure C4 (step C4-2)** using **S2a** (510 mg, 2.00 mmol, 1.00 equiv) and THF (10.0 mL), then *n*-BuLi (0.80 mL, 2.5 M in hexane, 2.0 mmol, 1.0 equiv) and 2,2,2-trichloroethyl chloroformate (0.33 mL, 2.4 mmol, 1.2 equiv). Purification using a Biotage automated flash chromatography system (ethyl acetate/pentane, 0% to 25%) afforded **2e** (740 mg, 1.72 mmol, 86 %) as a colourless oil.

^{1}H NMR (500 MHz, $CDCl_3$) δ 7.62 (d, *J* = 9.0 Hz, 2H), 7.58 (d, *J* = 8.4 Hz, 2H), 5.87 (d, *J* = 1.5 Hz, 1H), 5.64 (s, 1H), 5.51 (q, *J* = 1.6 Hz, 1H), 5.41 (d, *J* = 1.5 Hz, 1H), 4.72 (s, 2H), 2.09 (s, 3H).

^{13}C NMR (126 MHz, $CDCl_3$) δ 172.9, 152.3, 142.6, 141.5, 139.9, 131.0 (q, *J* = 32.7 Hz), 126.3, 125.8 (q, *J* = 3.7 Hz), 124.0 (q, *J* = 272.1 Hz), 121.0, 116.9, 94.1, 75.8, 18.9.

^{19}F NMR (471 MHz, $CDCl_3$) δ –62.72.

IR (ATR): $\tilde{\nu}$ (cm^{-1}) = 1749, 1700, 1637, 1619, 1452, 1376, 1260, 1166, 1116, 1064.

HRMS (ESI^+) calcd. for $C_{16}H_{14}Cl_3F_3NO_3^+$ $[M+H]^+$: 429.9986, found: 429.9986.

***N*-acetyl-*N*-(1-phenylvinyl) methacrylamide (2f)**

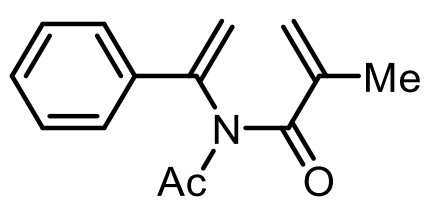

Prepared according to **General Procedure C1** using *N*-(1-phenylvinyl)acetamide[10-12] (483 mg, 3.00 mmol, 1.00 equiv) and CH_2Cl_2 (12.0 mL), then Et_3N (1.25 mL, 9.00 mmol, 3.00 equiv), methacryloyl chloride (0.59 mL, 6.0 mmol, 2.0 equiv), and DMAP (110 mg, 0.90 mmol, 0.30 equiv). Purification using a Biotage automated flash chromatography system (ethyl acetate/pentane, 0% to 40%) afforded **2f** (460 mg, 2.01 mmol, 67 %) as a white solid.

M.P.: 65-67 °C

^{1}H NMR (500 MHz, $CDCl_3$) δ 7.45 – 7.30 (m, 5H), 5.83 (s, 1H), 5.51 (s, 1H), 5.33 (s, 1H), 5.21 (s, 1H), 2.32 (s, 3H), 1.92 (s, 3H).

^{13}C NMR (126 MHz, $CDCl_3$) δ 174.2, 173.5, 144.7, 141.4, 136.0, 129.1, 128.9, 125.6, 120.2, 114.8, 25.3, 19.2.

IR (ATR): $\tilde{\nu}$ (cm^{-1}) = 1689, 1632, 1495, 1446, 1365, 1316, 1246, 1170, 1027.

HRMS (ESI^+) calcd. for $C_{14}H_{15}NO_2Na^+$ $[M+Na]^+$: 252.0995, found: 252.0987.

_N_-acetyl-_N_-(1-(4-methoxyphenyl)vinyl)methacrylamide (2g)

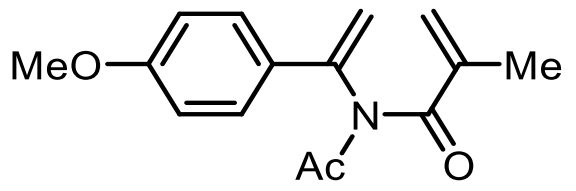

Prepared according to **General Procedure C1** using *N*-(1-(4-methoxyphenyl)vinyl)acetamide[10-12] (383 mg, 2.00 mmol, 1.00 equiv) and CH_2Cl_2 (8.0 mL), then Et_3N (0.84 mL, 6.0 mmol, 3.0 equiv), methacryloyl chloride (0.39 mL, 4.0 mmol, 2.0 equiv), and DMAP (73 mg, 0.60 mmol, 0.30 equiv). Purification using a Biotage automated flash chromatography system (ethyl acetate/pentane, 0% to 50%) afforded **2g** (410 mg, 1.58 mmol, 79 %) as a colourless oil.

^{1}H NMR (500 MHz, $CDCl_3$) δ 7.33 (d, J = 8.8 Hz, 2H), 6.83 (d, J = 8.8 Hz, 2H), 5.67 (s, 1H), 5.47 (s, 1H), 5.28 (s, 1H), 5.06 (s, 1H), 3.74 (s, 3H), 2.27 (s, 3H), 1.90 (s, 3H).

^{13}C NMR (126 MHz, $CDCl_3$) δ 174.1, 173.4, 160.1, 144.1, 141.2, 128.4, 126.9, 119.8, 114.1, 112.6, 55.2, 25.0, 19.0.

IR (ATR): $\tilde{\nu}$ (cm^{-1}) = 1691, 1606, 1511, 1366, 1302, 1248, 1176, 1028, 892.

HRMS (ESI$^+$) calcd. for $C_{15}H_{17}NO_3Na^+$ [M+Na]$^+$: 282.1101, found: 282.1094.

_N_-acetyl-_N_-(1-(2,3-dihydrobenzo[_b_][1,4]dioxin-6-yl)vinyl)methacrylamide (2h)

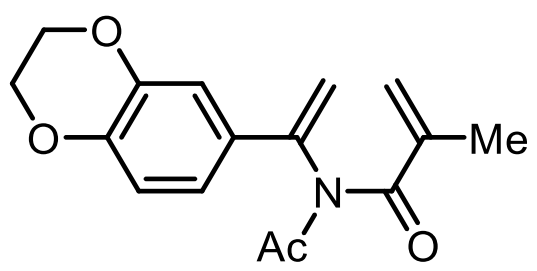

Prepared according to **General Procedure C1** using *N*-(1-(2,3-dihydrobenzo[*b*][1,4]dioxin-6-yl)vinyl)acetamide[10] (548 mg, 2.50 mmol, 1.00 equiv) and CH_2Cl_2 (10.0 mL), then Et_3N (1.05 mL, 7.50 mmol, 3.00 equiv), methacryloyl chloride (0.49 mL, 5.0 mmol, 2.0 equiv), and DMAP (92 mg, 0.75 mmol, 0.30 equiv). Purification using a Biotage automated flash chromatography system (ethyl acetate/pentane, 0% to 50%) afforded **2h** (550 mg, 1.92 mmol, 77 %) as a colourless oil.

^{1}H NMR (500 MHz, $CDCl_3$) δ 6.94 – 6.90 (m, 2H), 6.81 (d, J = 8.3 Hz, 1H), 5.69 (s, 1H), 5.49 (s, 1H), 5.31 (d, J = 1.7 Hz, 1H), 5.08 (d, J = 1.1 Hz, 1H), 4.27 – 4.19 (m, 4H), 2.30 (s, 3H), 1.94 (s, 3H).

^{13}C NMR (126 MHz, $CDCl_3$) δ 174.1, 173.5, 144.5, 144.0, 143.7, 141.4, 129.5, 120.0, 118.9, 117.7, 114.6, 113.3, 64.5, 64.3, 25.2, 19.2.

IR (ATR): $\tilde{\nu}$ (cm^{-1}) = 1691, 1629, 1580, 1506, 1457, 1366, 1276, 1199, 1065.

HRMS (ESI$^+$) calcd. for $C_{16}H_{17}NO_4Na^+$ [M+Na]$^+$: 310.1050, found: 310.1039.

_N_-acetyl-_N_-(1-(4-(_tert_-butyl)phenyl)vinyl)methacrylamide (2i)

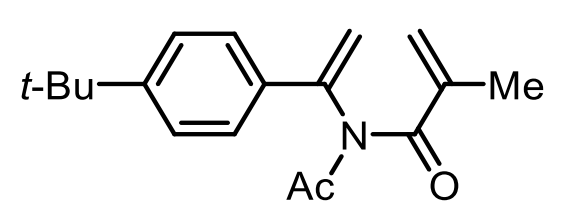

Prepared according to **General Procedure C1** using *N*-(1-(4-(*tert*-butyl)phenyl)vinyl)acetamide[10] (651 mg, 3.00 mmol, 1.00 equiv) and CH_2Cl_2 (12.0 mL), then Et_3N (1.25 mL, 9.00 mmol, 3.00 equiv), methacryloyl chloride (0.59 mL, 6.0 mmol, 2.0 equiv), and DMAP (110 mg, 0.90 mmol, 0.30 equiv). Purification using a Biotage automated flash chromatography system (ethyl acetate/pentane, 0% to 40%) afforded **2i** (630 mg, 2.21 mmol, 74 %) as a colourless oil.

^{1}H NMR (500 MHz, $CDCl_3$) δ 7.37 (s, 4H), 5.81 (d, *J* = 1.0 Hz, 1H), 5.52 (s, 1H), 5.33 (d, *J* = 0.9 Hz, 1H), 5.16 (d, *J* = 1.0 Hz, 1H), 2.33 (s, 3H), 1.95 (s, 3H), 1.31 (s, 9H).

^{13}C NMR (126 MHz, $CDCl_3$) δ 174.3, 173.6, 152.4, 144.6, 141.5, 133.0, 125.9, 125.4, 120.0, 114.0, 34.8, 31.3, 25.3, 19.3.

IR (ATR): $\tilde{\nu}$ (cm^{-1}) = 1713, 1695, 1632, 1366, 1299, 1274, 1258, 1176, 840.

HRMS (ESI$^+$) calcd. for $C_{18}H_{23}NO_2Na^+$ [M+Na]$^+$: 308.1621, found: 308.1606.

_N_-acetyl-_N_-(1-(_p_-tolyl)vinyl)methacrylamide (2j)

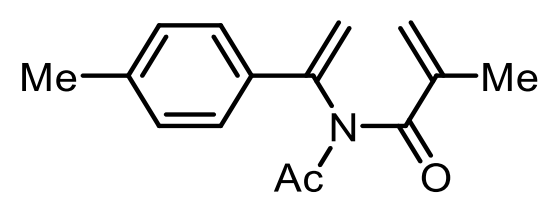

Prepared according to **General Procedure C1** using *N*-(1-(*p*-tolyl)vinyl)acetamide[10-12] (525 mg, 3.00 mmol, 1.00 equiv) and CH_2Cl_2 (12.0 mL), then Et_3N (1.25 mL, 9.00 mmol, 3.00 equiv), methacryloyl chloride (0.59 mL, 6.0 mmol, 2.0 equiv), and DMAP (110 mg, 0.90 mmol, 0.30 equiv). Purification using a Biotage automated flash chromatography system (ethyl acetate/pentane, 0% to 40%) afforded **2j** (500 mg, 2.06 mmol, 69 %) as a white solid.

M.P.: 37-39 °C

^{1}H NMR (500 MHz, $CDCl_3$) δ 7.32 (d, *J* = 8.2 Hz, 2H), 7.15 (d, *J* = 8.0 Hz, 2H), 5.78 (s, 1H), 5.51 (s, 1H), 5.31 (s, 1H), 5.15 (s, 1H), 2.33 (s, 3H), 2.31 (s, 3H), 1.93 (s, 3H).

^{13}C NMR (126 MHz, $CDCl_3$) δ 174.2, 173.5, 144.6, 141.4, 139.1, 133.1, 129.6, 125.5, 120.0, 113.8, 25.2, 21.2, 19.1.

IR (ATR): $\tilde{\nu}$ (cm^{-1}) = 1691, 1629, 1512, 1452, 1366, 1315, 1295, 1250, 1187, 1109, 1017, 893.

HRMS (ESI$^+$) calcd. for $C_{15}H_{17}NO_2Na^+$ [M+Na]$^+$: 266.1152, found: 266.1143.

***N*-Acetyl-*N*-(1-(*m*-tolyl)vinyl)methacrylamide (2k)**

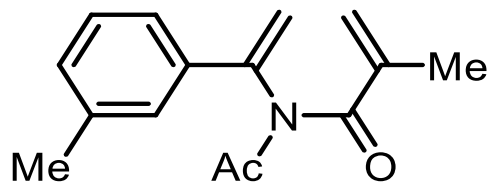

Prepared according to **General Procedure C1** using *N*-(1-(*m*-tolyl)vinyl)acetamide[12,13] (525 mg, 3.00 mmol, 1.00 equiv) and CH_2Cl_2 (12.0 mL), then Et_3N (1.25 mL, 9.00 mmol, 3.00 equiv), methacryloyl chloride (0.59 mL, 6.0 mmol, 2.0 equiv), and DMAP (110 mg, 0.90 mmol, 0.30 equiv). Purification using a Biotage automated flash chromatography system (ethyl acetate/pentane, 0% to 35%) afforded **2k** (480 mg, 1.98 mmol, 66 %) as a white solid.

M.P.: 39-41 °C

^{1}H NMR (500 MHz, $CDCl_3$) δ 7.26 – 7.20 (m, 3H), 7.15 – 7.12 (m, 1H), 5.81 (d, *J* = 1.0 Hz, 1H), 5.51 (s, 1H), 5.32 (d, *J* = 1.7 Hz, 1H), 5.19 (d, *J* = 1.1 Hz, 1H), 2.35 (s, 3H), 2.32 (s, 3H), 1.94 (s, 3H).

^{13}C NMR (126 MHz, $CDCl_3$) δ 174.2, 173.5, 144.8, 141.4, 138.5, 135.9, 129.9, 128.8, 126.3, 122.8, 120.1, 114.5, 25.2, 21.5, 19.1.

IR (ATR): $\tilde{\nu}$ (cm^{-1}) = 1691, 1629, 1512, 1453, 1366, 1275, 1260, 1162, 907.

HRMS (ESI$^+$) calcd. for $C_{15}H_{17}NO_2Na^+$ [M+Na]$^+$: 266.1152, found: 266.1144.

***N*-acetyl-*N*-(1-(4-fluorophenyl)vinyl)methacrylamide (2l)**

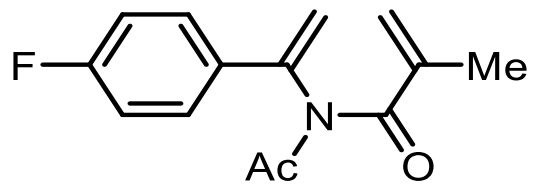

Prepared according to **General Procedure C1** using *N*-(1-(4-fluorophenyl)vinyl)acetamide[10,11] (537 mg, 3.00 mmol, 1.00 equiv) and CH_2Cl_2 (12.0 mL), then Et_3N (1.25 mL, 9.00 mmol, 3.00 equiv), methacryloyl chloride (0.59 mL, 6.0 mmol, 2.0 equiv), and DMAP (110 mg, 0.90 mmol, 0.30 equiv). Purification using a Biotage automated flash chromatography system (ethyl acetate/pentane, 0% to 40%) afforded **2l** (530 mg, 2.15 mmol, 72 %) as a white solid.

M.P.: 55-57 °C

^{1}H NMR (500 MHz, $CDCl_3$) δ 7.41-7.36 (m, 2H), 7.06 – 6.99 (m, 2H), 5.74 (s, 1H), 5.48 (s, 1H), 5.33 (d, *J* = 2.1 Hz, 1H), 5.18 (s, 1H), 2.31 (s, 3H), 1.91 (s, 3H).

^{13}C NMR (126 MHz, $CDCl_3$) δ 174.1, 173.4, 163.1 (d, *J* = 249.4 Hz), 143.8, 141.3, 132.3 (d, *J* = 3.4 Hz), 127.6 (d, *J* = 8.3 Hz), 120.5, 115.9 (d, *J* = 21.9 Hz), 114.6, 25.2, 19.1.

^{19}F NMR (471 MHz, $CDCl_3$) δ –112.17.

IR (ATR): $\tilde{\nu}$ (cm^{-1}) = 1691, 1632, 1602, 1508, 1453, 1366, 1306, 1231, 1160, 1110, 1012, 895.

HRMS (ESI^+) calcd. for $C_{14}H_{14}FNO_2Na^+$ $[M+Na]^+$: 270.0901, found: 270.0898.

N-(1-([1,1'-biphenyl]-4-yl)vinyl)-*N*-acetylmethacrylamide (2m)

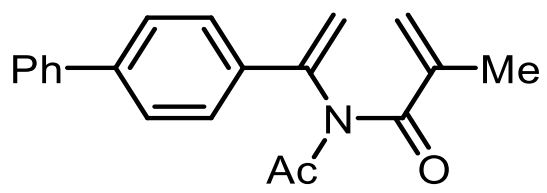

Prepared according to **General Procedure C1** using *N*-(1-([1,1'-biphenyl]-4-yl)vinyl)acetamide[10] (474 mg, 2.00 mmol, 1.00 equiv) and CH_2Cl_2 (8.0 mL), then Et_3N (0.84 mL, 6.00 mmol, 3.00 equiv), methacryloyl chloride (0.39 mL, 4.0 mmol, 2.0 equiv), and DMAP (73 mg, 0.60 mmol, 0.30 equiv). Purification using a Biotage automated flash chromatography system (ethyl acetate/pentane, 0% to 50%) afforded **2m** (440 mg, 1.44 mmol, 72 %) as a white solid.

M.P.: 140-142 °C

^{1}H NMR (500 MHz, $CDCl_3$) δ 7.63 – 7.58 (m, 4H), 7.54 – 7.50 (m, 2H), 7.48 – 7.43 (m, 2H), 7.39 – 7.34 (m, 1H), 5.90 (d, *J* = 1.2 Hz, 1H), 5.56 (s, 1H), 5.37 (q, *J* = 1.6 Hz, 1H), 5.25 (d, *J* = 1.2 Hz, 1H), 2.38 (s, 3H), 1.98 (s, 3H).

^{13}C NMR (126 MHz, $CDCl_3$) δ 174.2, 173.5, 144.4, 141.9, 141.4, 140.2, 134.8, 128.9, 127.8, 127.6, 127.1, 126.1, 120.3, 114.7, 25.3, 19.2.

IR (ATR): $\tilde{\nu}$ (cm^{-1}) = 1712, 1685, 1637, 1607, 1487, 1366, 1311, 1265, 1166, 846.

HRMS (ESI^+) calcd. for $C_{20}H_{19}NO_2Na^+$ $[M+Na]^+$: 328.1308, found: 328.1298.

N-acetyl-*N*-(1-(4-chlorophenyl)vinyl)methacrylamide (2n)

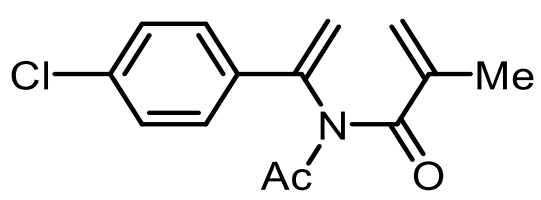

Prepared according to **General Procedure C1** using *N*-(1-(4-chlorophenyl)vinyl)acetamide[10-12] (390 mg, 2.00 mmol, 1.00 equiv) and CH_2Cl_2 (8.0 mL), then Et_3N (0.84 mL, 6.0 mmol, 3.0 equiv), methacryloyl chloride (0.39 mL, 4.0 mmol, 2.0 equiv), and DMAP (73 mg, 0.60 mmol, 0.30 equiv). Purification using a Biotage automated flash chromatography system (ethyl acetate/pentane, 0% to 50%) afforded **2n** (424 mg, 1.61 mmol, 81 %) as a colourless oil.

^{1}H NMR (500 MHz, $CDCl_3$) δ 7.33 (d, *J* = 8.7 Hz, 2H), 7.27 (d, *J* = 8.8 Hz, 2H), 5.76 (d, *J* = 1.4 Hz, 1H), 5.46 (s, 1H), 5.30 (q, *J* = 1.6 Hz, 1H), 5.18 (d, *J* = 1.4 Hz, 1H), 2.28 (s, 3H), 1.88 (s, 3H).

^{13}C NMR (126 MHz, $CDCl_3$) δ 173.8, 173.1, 143.6, 141.0, 134.7, 134.5, 128.9, 126.8, 120.4, 115.1, 25.1, 18.9.

IR (ATR): $\tilde{\nu}$ (cm^{-1}) = 1693, 1632, 1491, 1367, 1259, 1172, 1090, 1011, 834.

HRMS (ESI^+) calcd. for $C_{14}H_{15}ClNO_2^+$ $[M+H]^+$: 264.0786, found: 264.0783.

Methyl 4-(1-(N-acetylmethacrylamido)vinyl)benzoate (2o)

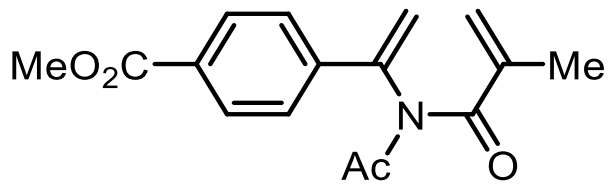

Prepared according to **General Procedure C1** using methyl 4-(1-acetamidovinyl)benzoate[12] (657 mg, 3.00 mmol, 1.00 equiv) and CH_2Cl_2 (12.0 mL), then Et_3N (1.25 mL, 9.00 mmol, 3.00 equiv), methacryloyl chloride (0.59 mL, 6.0 mmol, 2.0 equiv), and DMAP (110 mg, 0.90 mmol, 0.30 equiv). Purification using a Biotage automated flash chromatography system (ethyl acetate/pentane, 0% to 40%) afforded **2o** (680 mg, 2.37 mmol, 79 %) as a colourless oil.

^{1}H NMR (500 MHz, $CDCl_3$) δ 7.99 (d, J = 8.6 Hz, 2H), 7.46 (d, J = 8.6 Hz, 2H), 5.90 (d, J = 1.4 Hz, 1H), 5.47 (s, 1H), 5.31 (q, J = 1.6 Hz, 1H), 5.29 (d, J = 1.4 Hz, 1H), 3.87 (s, 3H), 2.31 (s, 3H), 1.88 (s, 3H).

^{13}C NMR (126 MHz, $CDCl_3$) δ 173.9, 173.3, 166.4, 143.9, 141.1, 140.3, 130.5, 130.2, 125.6, 120.7, 116.7, 52.2, 25.3, 19.1.

IR (ATR): $\tilde{\nu}$ (cm^{-1}) = 1713, 1703, 1631, 1609, 1436, 1368, 1276, 1261, 1187,1107.

HRMS (ESI^+) calcd. for $C_{16}H_{18}NO_4^+$ $[M+H]^+$: 288.1230, found: 288.1221.

N-acetyl-*N*-(1-(4-cyanophenyl)vinyl)methacrylamide (2p)

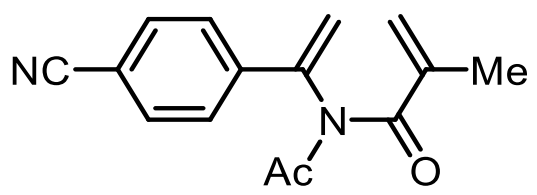

Prepared according to **General Procedure C1** using *N*-(1-(4-cyanophenyl)vinyl)acetamide[12] (558 mg, 3.00 mmol, 1.00 equiv) and CH_2Cl_2 (12.0 mL), then Et_3N (1.25 mL, 9.00 mmol, 3.00 equiv), methacryloyl chloride (0.59 mL, 6.0 mmol, 2.0 equiv), and DMAP (110 mg, 0.90 mmol, 0.30 equiv). Purification using a Biotage automated flash chromatography system (ethyl acetate/pentane, 0% to 50%) afforded **2p** (630 mg, 2.48 mmol, 83 %) as a colourless oil.

^{1}H NMR (500 MHz, $CDCl_3$) δ 7.61 (d, J = 8.5 Hz, 2H), 7.49 (d, J = 8.6 Hz, 2H), 5.90 (d, J = 1.6 Hz, 1H), 5.47 (d, J = 1.7 Hz, 1H), 5.34-5.32 (m, 2H), 2.30 (s, 3H), 1.88 (s, 3H).

^{13}C NMR (126 MHz, $CDCl_3$) δ 173.7, 173.0, 143.2, 140.9, 140.3, 132.6, 126.1, 121.1, 118.3, 117.6, 112.4, 25.3, 19.0.

IR (ATR): $\tilde{\nu}$ (cm^{-1}) = 2228, 1692, 1631, 1506, 1367, 1315, 1299, 1239, 1188, 1108.

HRMS (ESI$^+$) calcd. for $C_{15}H_{14}N_2O_2Na^+$ [M+Na]$^+$: 277.0947, found: 277.0953.

N-acetyl-*N*-(1-(furan-2-yl)vinyl)methacrylamide (2q)

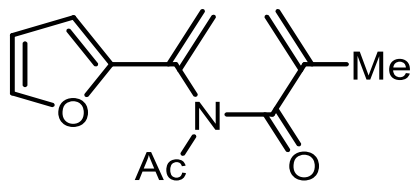

Prepared according to **General Procedure C1** using *N*-(1-(furan-2-yl)vinyl)acetamide[10,12] (350 mg, 2.32 mmol, 1.00 equiv) and CH_2Cl_2 (9.3 mL) then Et_3N (0.97 mL, 7.0 mmol, 3.0 equiv), methacryloyl chloride (0.45 mL, 4.6 mmol, 2.0 equiv), and DMAP (85 mg, 0.70 mmol, 0.30 equiv). Purification using a Biotage automated flash chromatography system (ethyl acetate/pentane, 0% to 40%) afforded **2q** (270 mg, 1.23 mmol, 53 %) as a pale-yellow oil.

^{1}H NMR (500 MHz, $CDCl_3$) δ 7.32 (d, *J* = 1.8 Hz, 1H), 6.33 (dd, *J* = 3.5, 1.9 Hz, 1H), 6.29 (d, *J* = 3.5 Hz, 1H), 5.77 (s, 1H), 5.46 (q, *J* = 0.9 Hz, 1H), 5.27 (s, 1H), 5.06 (s, 1H), 2.29 (s, 3H), 1.90 (s, 3H).

^{13}C NMR (126 MHz, $CDCl_3$) δ 173.4, 172.8, 150.3, 143.1, 140.8, 135.2, 120.0, 112.7, 111.6, 108.2, 24.7, 19.0.

IR (ATR): $\tilde{\nu}$ (cm^{-1}) = 1698, 1636, 1454, 1368, 1319, 1275, 1260, 1193, 1014.

HRMS (ESI$^+$) calcd. for $C_{12}H_{13}NO_3Na^+$ [M+Na]$^+$: 242.0788, found: 242.0784.

N-acetyl-*N*-(1-(thiophen-2-yl)vinyl)methacrylamide (2r)

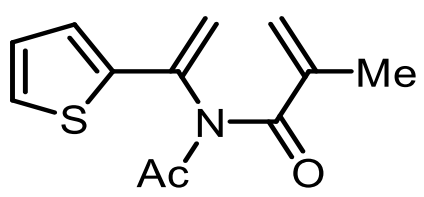

Prepared according to **General Procedure C1** using *N*-(1-(thiophen-2-yl)vinyl)acetamide[12,13] (334 mg, 2.00 mmol, 1.00 equiv) and CH_2Cl_2 (8.0 mL), then Et_3N (0.84 mL, 6.0 mmol, 3.0 equiv), methacryloyl chloride (0.39 mL, 4.0 mmol, 2.0 equiv), and DMAP (73 mg, 0.60 mmol, 0.30 equiv). Purification using a Biotage automated flash chromatography system (ethyl acetate/pentane, 0% to 40%) afforded **2r** (343 mg, 1.46 mmol, 73 %) as a pale-yellow oil.

^{1}H NMR (500 MHz, $CDCl_3$) δ 7.23 (dd, *J* = 5.0, 1.2 Hz, 1H), 7.04 (dd, *J* = 3.7, 1.2 Hz, 1H), 6.96 (dd, *J* = 5.1, 3.6 Hz, 1H), 5.74 (d, *J* = 1.4 Hz, 1H), 5.52 (s, 1H), 5.32 (q, *J* = 1.7 Hz, 1H), 5.09 (d, *J* = 1.4 Hz, 1H), 2.35 (s, 3H), 1.96 (s, 3H).

^{13}C NMR (126 MHz, $CDCl_3$) δ 173.6, 173.1, 141.1, 140.7, 139.0, 127.8, 126.3, 125.6, 120.1, 114.0, 25.0, 19.2.

IR (ATR): $\tilde{\nu}$ (cm^{-1}) = 1691, 1623, 1434, 1365, 1276, 1259, 1164, 1037.

HRMS (ESI^+) calcd. for $C_{12}H_{13}NO_2SNa^+$ $[M+Na]^+$: 258.0559, found: 258.0553.

N-acetyl-*N*-(1-(thiophen-3-yl)vinyl)methacrylamide (2s)

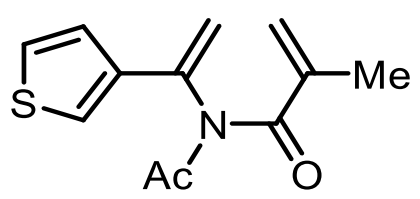

Prepared according to **General Procedure C1** using *N*-(1-(thiophen-3-yl)vinyl)acetamide[14] (501 mg, 3.00 mmol, 1.00 equiv) and CH_2Cl_2 (12.0 mL), then Et_3N (1.25 mL, 9.00 mmol, 3.00 equiv), methacryloyl chloride (0.59 mL, 6.0 mmol, 2.0 equiv), and DMAP (110 mg, 0.90 mmol, 0.30 equiv). Purification using a Biotage automated flash chromatography system (ethyl acetate/pentane, 0% to 35%) afforded **2s** (479 mg, 2.04 mmol, 68 %) as a pale-yellow solid.

M.P.: 47-49 °C

^{1}H NMR (500 MHz, $CDCl_3$) δ 7.28 (dd, J = 5.1, 3.0 Hz, 1H), 7.20 (dd, J = 3.0, 1.4 Hz, 1H), 7.16 (dd, J = 5.1, 1.4 Hz, 1H), 5.73 (d, J = 1.2 Hz, 1H), 5.47 (s, 1H), 5.30 (q, J = 1.6 Hz, 1H), 5.13 (d, J = 1.1 Hz, 1H), 2.31 (s, 3H), 1.93 (dd, J = 1.7, 1.1 Hz, 3H).

^{13}C NMR (126 MHz, $CDCl_3$) δ 173.9, 173.3, 141.1, 140.2, 138.6, 127.0, 125.3, 122.6, 120.0, 114.2, 25.0, 19.2.

IR (ATR): $\tilde{\nu}$ (cm^{-1}) = 1688, 1630, 1423, 1366, 1276, 1260, 1205, 1185, 1161.

HRMS (ESI^+) calcd. for $C_{12}H_{13}NO_2SNa^+$ $[M+Na]^+$: 258.0559, found: 258.0554.

N-(1-(6-methoxypyridin-3-yl)vinyl)acetamide (S2t)

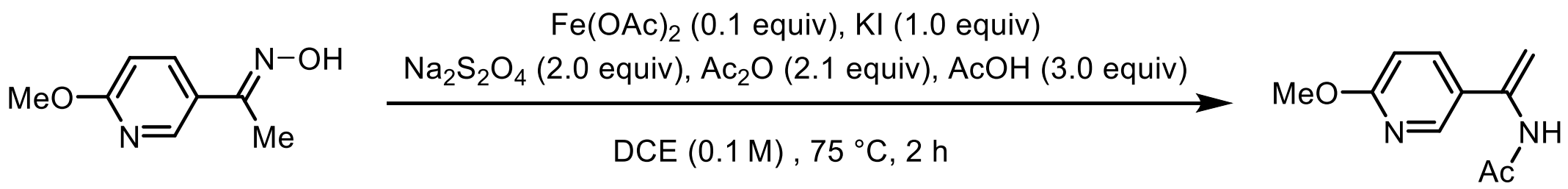

To an oven-dried round-bottom flask equipped with a magnetic stir bar and a 15 cm condenser, (*E*)-1-(6-methoxypyridin-3-yl)ethan-1-one oxime[15] (1.66 g, 10.0 mmol, 1.00 equiv), KI (1.66 g, 10.0 mmol, 1.00 equiv), $Na_2S_2O_4$ (3.48 g, 20.0 mmol, 2.00 equiv), $Fe(OAc)_2$ (174 mg, 1.00 mmol, 0.10 equiv) were added under an argon atmosphere in a glovebox. The flask was sealed with a rubber septum, removed from the glovebox, and kept under a nitrogen atmosphere using a balloon. 1,2-Dichloroethane (100 mL), acetic acid (1.72 mL, 30.0 mmol, 3.00 equiv), and acetic anhydride (1.99 mL, 21.0 mmol, 2.10 equiv) were added to the mixture via syringe. The reaction mixture was heated to 75 °C for 2 h, then cooled to room temperature. The resulting mixture was quenched with saturated $NaHCO_3$ solution and extracted with CH_2Cl_2 three times. The combined organic layers were dried over anhydrous $MgSO_4$, filtered, concentrated under reduced pressure, and purified using a Biotage

automated flash chromatography system (ethyl acetate/pentane, 0% to 60%) and recrystallisation[A] (pentane/Et_2O) to afford the desired product **S2t** (1.10 g, 5.73 mmol, 57 %) as a white solid.

Notes: (**A**) If the crude product after column chromatography is not sufficiently pure, further purification can be performed either by a second round of chromatography or by recrystallisation. Since the product is typically a solid, it often precipitates during rotary evaporation. In such cases, recrystallisation from a petroleum ether/Et_2O solvent system can be used for purification.

M.P.: 74-76 °C

^{1}H NMR (500 MHz, $CDCl_3$) δ 8.19 (d, *J* = 2.5 Hz, 1H), 7.57 (dd, *J* = 8.7, 2.6 Hz, 1H), 7.09 (br. s, 1H), 6.70 (d, *J* = 8.5 Hz, 1H), 5.66 (s, 1H), 5.00 (s, 1H), 3.92 (s, 3H), 2.07 (s, 3H).

^{13}C NMR (126 MHz, $CDCl_3$) δ 169.3, 164.4, 144.7, 138.0, 136.7, 127.4, 110.7, 103.3, 53.7, 24.4.

IR (ATR): $\tilde{\nu}$ (cm^{-1}) = 1667, 1633, 1603, 1527, 1489, 1368, 1281, 1254, 1131, 1022.

HRMS (ESI^+) calcd. for $C_{10}H_{13}N_2O_2^+$ $[M+H]^+$: 193.0972, found: 193.0964.

***N*-acetyl-*N*-(1-(6-methoxypyridin-3-yl)vinyl)methacrylamide (2t)**

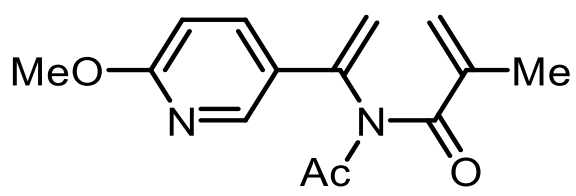

Prepared according to **General Procedure C1** using *N*-(1-(6-methoxypyridin-3-yl)vinyl)acetamide **S2t** (576 mg, 3.00 mmol, 1.00 equiv) and CH_2Cl_2 (12.0 mL), then Et_3N (1.25 mL, 9.00 mmol, 3.00 equiv), methacryloyl chloride (0.59 mL, 6.0 mmol, 2.0 equiv), and DMAP (110 mg, 0.90 mmol, 0.30 equiv). Purification using a Biotage automated flash chromatography system (ethyl acetate/pentane, 0% to 40%) afforded **2t** (537 mg, 2.07 mmol, 69 %) as a colourless oil.

^{1}H NMR (500 MHz, $CDCl_3$) δ 8.17 (dd, *J* = 2.7, 0.8 Hz, 1H), 7.58 (dd, *J* = 8.7, 2.6 Hz, 1H), 6.66 (dd, *J* = 8.7, 0.8 Hz, 1H), 5.67 (d, *J* = 1.3 Hz, 1H), 5.43 (s, 1H), 5.27 (q, *J* = 1.7 Hz, 1H), 5.09 (d, *J* = 1.3 Hz, 1H), 3.86 (s, 3H), 2.27 (s, 3H), 1.88 (s, 3H).

^{13}C NMR (126 MHz, $CDCl_3$) δ 173.8, 173.1, 164.3, 144.3, 142.0, 141.1, 135.9, 125.1, 120.3, 113.9, 111.1, 53.6, 25.1, 19.1.

IR (ATR): $\tilde{\nu}$ (cm^{-1}) = 1692, 1631, 1600, 1494, 1454, 1367, 1286, 1258, 1173, 1019.

HRMS (ESI^+) calcd. for $C_{14}H_{17}N_2O_3^+$ $[M+H]^+$: 261.1234, found: 261.1223.

Methyl 2-(*N*-(tert-butoxycarbonyl)methacrylamido)acrylate (2u)

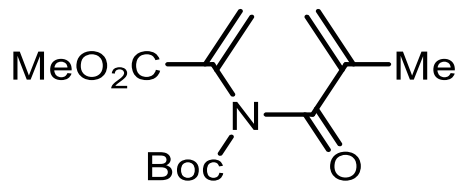

Prepared according to **General Procedure C2** using methyl 2-((*tert*-butoxycarbonyl)amino)acrylate[6] (402 mg, 2.00 mmol, 1.00 equiv) and THF (10.0 mL), then *n*-BuLi (0.80 mL, 2.5 M in hexane, 2.0 mmol, 1.0 equiv) and methacryloyl chloride (0.25 mL, 2.6 mmol, 1.3 equiv). Purification using a Biotage automated flash chromatography system (ethyl acetate/pentane, 0% to 20%) afforded **2u** (461 mg, 1.71 mmol, 86 %) as a colourless oil.

^{1}H NMR (500 MHz, $CDCl_3$) δ 6.42 (d, *J* = 0.8 Hz, 1H), 5.69 (d, *J* = 0.8 Hz, 1H), 5.54 (p, *J* = 1.1 Hz, 1H), 5.35 – 5.33 (m, 1H), 3.78 (s, 3H), 2.02 (s, 3H), 1.44 (s, 9H).

^{13}C NMR (126 MHz, $CDCl_3$) δ 173.3, 163.7, 152.0, 142.1, 135.7, 125.6, 119.2, 84.1, 52.7, 27.8, 19.0.

IR (ATR): $\tilde{\nu}$ (cm^{-1}) = 1735, 1694, 1439, 1370, 1281, 1208, 1155, 1100.

HRMS (ESI$^+$) calcd. for $C_{13}H_{19}NO_5Na^+$ [M+Na]$^+$: 292.1155, found: 292.1146.

***N*-(1-(4-methoxyphenyl)vinyl)methacrylamide (S2v)**

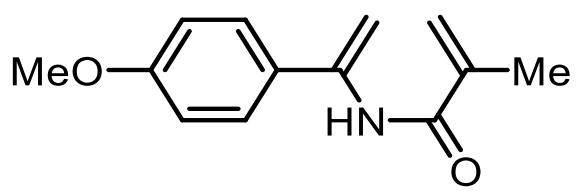

Prepared according to **General Procedure C4 (Step C4-1)** using 4-methoxybenzonitrile (2.66 g, 20.0 mmol, 1.00 equiv), THF (20.0 mL), MeLi (16.3 mL, 1.60 M in Et_2O, 26.0 mmol, 1.30 equiv), MeOH (1.22 mL, 30.0 mmol, 1.50 equiv), CH_2Cl_2 (ca. 30–40 mL), Et_3N (5.58 mL, 40.0 mmol, 2.00 equiv) and methacryloyl chloride (2.93 mL, 30.0 mmol, 1.50 equiv). Purification using a Biotage automated flash chromatography system (ethyl acetate/pentane, 0% to 30%) afforded **S2v** (1.09 g, 5.02 mmol, 25%) as a white solid.

M.P.: 41-43 °C

^{1}H NMR (500 MHz, $CDCl_3$) δ 7.36 (d, *J* = 8.8 Hz, 2H), 7.13 (br. s, 1H), 6.90 (d, *J* = 8.7 Hz, 2H), 5.86 (s, 1H), 5.74 (s, 1H), 5.44 – 5.41 (m, 1H), 5.05 (d, *J* = 1.0 Hz, 1H), 3.82 (s, 3H), 2.03 (dd, *J* = 1.5, 0.9 Hz, 3H).

^{13}C NMR (126 MHz, $CDCl_3$) δ 167.2, 160.1, 141.0, 140.1, 131.2, 127.3, 119.8, 114.3, 101.6, 55.5, 18.9.

IR (ATR): $\tilde{\nu}$ (cm^{-1}) = 1667, 1625, 1608, 1507, 1248, 1177, 1031.

HRMS (ESI$^+$) calcd. for $C_{13}H_{16}NO_2^+$ [M+H]$^+$: 218.1176, found: 218.1187.

2,2,2-Trichloroethyl methacryloyl(1-(4-methoxyphenyl)vinyl)carbamate (2v)

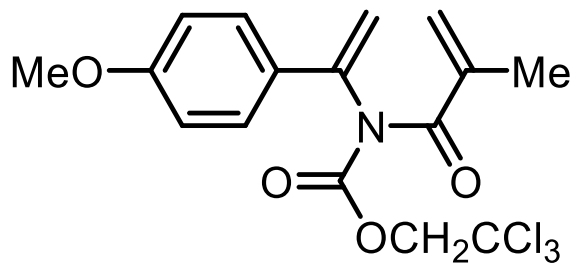

Prepared according to **General Procedure C4 (Step C4-2)** using **S2v** (326 mg, 1.50 mmol, 1.00 equiv) and THF (7.5 mL), then *n*-BuLi (0.60 mL, 2.5 M in hexane, 1.5 mmol, 1.0 equiv) and 2,2,2-trichloroethyl chloroformate (0.25 mL, 1.8 mmol, 1.2 equiv). Purification using a Biotage automated flash chromatography system (ethyl acetate/pentane, 0% to 25%) afforded **2v** (475 mg, 1.21 mmol, 81 %) as a colourless oil.

^{1}H NMR (500 MHz, $CDCl_3$) δ 7.40 (d, J = 8.8 Hz, 2H), 6.86 (d, J = 8.9 Hz, 2H), 5.67 (s, 1H), 5.62 (s, 1H), 5.46 (s, 1H), 5.18 (s, 1H), 4.72 (s, 2H), 3.79 (s, 3H), 2.06 (s, 3H).

^{13}C NMR (126 MHz, $CDCl_3$) δ 173.0, 160.3, 152.7, 143.3, 141.6, 128.8, 127.3, 120.5, 114.1, 112.9, 94.2, 75.7, 55.4, 19.0.

IR (ATR): $\tilde{\nu}$ (cm^{-1}) = 1746, 1698, 1634, 1608, 1511, 1452, 1248, 1178, 1130, 1087.

HRMS (ESI$^+$) calcd. for $C_{16}H_{16}Cl_3NO_4Na^+$ [M+Na]$^+$: 414.0037, found: 414.0026.

***N*-(1-(4-methoxyphenyl)vinyl)-*N*-propionylmethacrylamide (2w)**

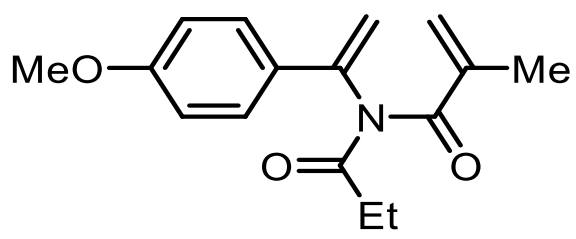

Prepared according to **General Procedure C4 (Step C4-2)** using **S2v** (326 mg, 1.50 mmol, 1.00 equiv) and THF (7.5 mL), then *n*-BuLi (0.60 mL, 2.5 M in hexane, 1.5 mmol, 1.0 equiv) and propionic anhydride (0.23 mL, 1.8 mmol, 1.2 equiv). Purification using a Biotage automated flash chromatography system (ethyl acetate/pentane, 0% to 30%) afforded **2w** (332 mg, 1.22 mmol, 81 %) as a colourless oil.

^{1}H NMR (500 MHz, $CDCl_3$) δ 7.36 (d, J = 8.8 Hz, 2H), 6.87 (d, J = 8.9 Hz, 2H), 5.71 (d, J = 1.2 Hz, 1H), 5.48 (s, 1H), 5.31 (dq, J = 2.2, 1.4 Hz, 1H), 5.10 (d, J = 1.1 Hz, 1H), 3.80 (s, 3H), 2.59 (q, J = 7.4 Hz, 2H), 1.97 (dd, J = 1.6, 1.0 Hz, 3H), 1.11 (t, J = 7.4 Hz, 3H).

^{13}C NMR (126 MHz, $CDCl_3$) δ 177.6, 174.5, 160.3, 144.2, 141.9, 128.8, 127.1, 119.4, 114.3, 112.9, 55.4, 30.6, 19.2, 9.4.

IR (ATR): $\tilde{\nu}$ (cm^{-1}) = 1690, 1627, 1607, 1511, 1454, 1252, 1172, 1029.

HRMS (ESI$^+$) calcd. for $C_{16}H_{19}NO_3Na^+$ [M+Na]$^+$: 296.1257, found: 296.1266.

***N*-isobutyryl-*N*-(1-(4-methoxyphenyl)vinyl)methacrylamide (2x)**

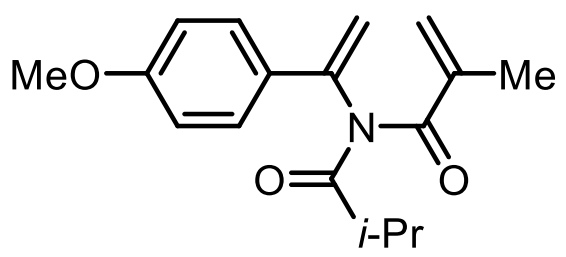

Prepared according to **General Procedure C4 (Step C4-2)** using **S2v** (326 mg, 1.50 mmol, 1.00 equiv) and THF (7.5 mL), then *n*-BuLi (0.60 mL, 2.5 M in hexane, 1.5 mmol, 1.0 equiv) and isobutyric anhydride (0.30 mL, 1.8 mmol, 1.2 equiv). Purification using a Biotage automated flash chromatography system (ethyl acetate/pentane, 0% to 25%) afforded **2x** (370 mg, 1.29 mmol, 86 %) as a colourless oil.

^{1}H NMR (500 MHz, $CDCl_3$) δ 7.38 (d, *J* = 8.9 Hz, 2H), 6.88 (d, *J* = 8.9 Hz, 2H), 5.69 (d, *J* = 1.0 Hz, 1H), 5.54 (s, 1H), 5.34 (qd, *J* = 1.6, 0.7 Hz, 1H), 5.09 (d, *J* = 1.0 Hz, 1H), 3.80 (s, 3H), 2.95 (hept, *J* = 6.7 Hz, 1H), 1.99 (dd, *J* = 1.6, 1.0 Hz, 3H), 1.12 (d, *J* = 6.8 Hz, 6H).

^{13}C NMR (126 MHz, $CDCl_3$) δ 181.6, 175.0, 160.4, 144.4, 142.1, 129.1, 127.2, 119.4, 114.3, 112.5, 55.5, 35.2, 19.7, 19.1.

IR (ATR): $\tilde{\nu}$ (cm^{-1}) = 1691, 1627, 1607, 1511, 1458, 1383, 1301, 1251, 1168.

HRMS (ESI^+) calcd. for $C_{17}H_{21}NO_3Na^+$ $[M+Na]^+$: 310.1414, found: 310.1412.

Phenyl methacryloyl(1-(4-methoxyphenyl)vinyl)carbamate (2y)

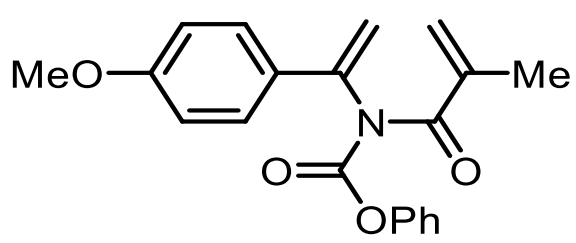

Prepared according to **General Procedure C4 (Step C4-2)** using **S2v** (326 mg, 1.50 mmol, 1.00 equiv) and THF (7.5 mL), then *n*-BuLi (0.60 mL, 2.5 M in hexane, 1.5 mmol, 1.0 equiv) and phenyl chloroformate (0.22 mL, 1.8 mmol, 1.2 equiv). Purification using a Biotage automated flash chromatography system (ethyl acetate/pentane, 0% to 25%) afforded **2y** (410 mg, 1.22 mmol, 81 %) as a white solid.

M.P.: 105-107 °C

^{1}H NMR (500 MHz, $CDCl_3$) δ 7.48 (d, *J* = 8.9 Hz, 2H), 7.34 – 7.28 (m, 2H), 7.22 – 7.17 (m, 1H), 6.92 (d, *J* = 8.9 Hz, 2H), 6.91 – 6.88 (m, 2H), 5.72-5.69 (m, 2H), 5.48 (q, *J* = 1.6 Hz, 1H), 5.25 (d, *J* = 1.3 Hz, 1H), 3.83 (s, 3H), 2.10 (t, *J* = 1.3 Hz, 3H).

^{13}C NMR (126 MHz, $CDCl_3$) δ 173.5, 160.3, 153.0, 150.5, 143.7, 142.1, 129.5, 129.2, 127.2, 126.3, 121.1, 119.9, 114.3, 112.4, 55.5, 19.1.

IR (ATR): $\tilde{\nu}$ (cm^{-1}) = 1749, 1695, 1633, 1607, 1511, 1493, 1250, 1157, 1028.

HRMS (ESI$^+$) calcd. for $C_{20}H_{19}NO_4Na^+$ $[M+Na]^+$: 360.1206, found: 360.1221.

Benzyl methacryloyl(1-(4-methoxyphenyl)vinyl) carbamate (2z)

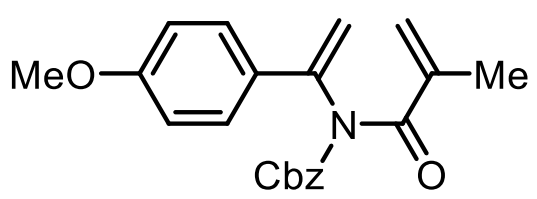

Prepared according to **General Procedure C4 (Step C4-2)** using **S2v** (326 mg, 1.50 mmol, 1.00 equiv) and THF (7.5 mL), then *n*-BuLi (0.60 mL, 2.5 M in hexane, 1.5 mmol, 1.0 equiv) and benzyl chloroformate (0.26 mL, 1.8 mmol, 1.2 equiv). Purification using a Biotage automated flash chromatography system (ethyl acetate/pentane, 0% to 25%) afforded **2z** (430 mg, 1.23 mmol, 82 %) as a colourless oil.

^{1}H NMR (500 MHz, $CDCl_3$) δ 7.35 (d, J = 8.8 Hz, 2H), 7.30 – 7.24 (m, 3H), 7.13 – 7.10 (m, 2H), 6.84 (d, J = 8.8 Hz, 2H), 5.61 (d, J = 1.1 Hz, 1H), 5.53 (s, 1H), 5.34 (q, J = 1.6 Hz, 1H), 5.11 (s, 2H), 5.10 (d, J = 1.1 Hz, 1H), 3.81 (s, 3H), 2.01 (s, 3H).

^{13}C NMR (126 MHz, $CDCl_3$) δ 173.5, 160.2, 154.3, 143.6, 142.1, 134.8, 129.2, 128.50, 128.46, 128.3, 127.1, 119.4, 114.1, 112.1, 68.8, 55.4, 19.1.

IR (ATR): $\tilde{\nu}$ (cm^{-1}) = 1736, 1690, 1633, 1607, 1511, 1455, 1380, 1248, 1178, 1124, 1076.

HRMS (ESI$^+$) calcd. for $C_{21}H_{21}NO_4Na^+$ $[M+Na]^+$: 374.1363, found: 374.1381.

Methyl methacryloyl(1-(4-methoxyphenyl)vinyl)carbamate (2aa)

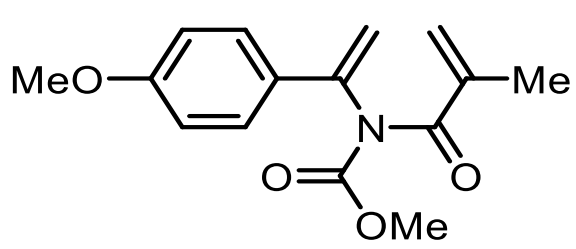

Prepared according to **General Procedure C4 (Step C4-2)** using **S2v** (326 mg, 1.50 mmol, 1.00 equiv) and THF (7.5 mL), then *n*-BuLi (0.60 mL, 2.5 M in hexane, 1.5 mmol, 1.0 equiv) and methyl chloroformate (0.14 mL, 1.8 mmol, 1.2 equiv). Purification using a Biotage automated flash chromatography system (ethyl acetate/pentane, 0% to 30%) afforded **2aa** (350 mg, 1.27 mmol, 85 %) as a colourless oil.

^{1}H NMR (500 MHz, $CDCl_3$) δ 7.37 (d, J = 8.8 Hz, 2H), 6.86 (d, J = 8.9 Hz, 2H), 5.63 (d, J = 1.2 Hz, 1H), 5.51 (s, 1H), 5.36 (qd, J = 1.6, 0.6 Hz, 1H), 5.11 (d, J = 1.1 Hz, 1H), 3.79 (s, 3H), 3.69 (s, 3H), 2.03 (dd, J = 1.7, 1.0 Hz, 3H).

^{13}C NMR (126 MHz, $CDCl_3$) δ 173.3, 160.2, 155.0, 143.5, 142.1, 129.0, 127.0, 119.0, 114.1, 112.2, 55.4, 53.9, 19.1.

IR (ATR): $\tilde{\nu}$ (cm^{-1}) = 1742, 1690, 1633, 1607, 1511, 1438, 1248, 1178, 1129, 1081.

HRMS (ESI^+) calcd. for $C_{15}H_{17}NO_4Na^+$ $[M+Na]^+$: 298.1050, found: 298.1063.

Tert-butyl methacryloyl(1-(4-methoxyphenyl)vinyl)carbamate (2ab)

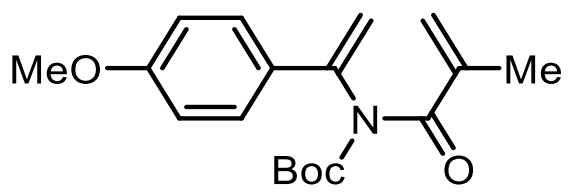

Prepared according to **General Procedure C4 (Step C4-2)** using **S2v** (326 mg, 1.50 mmol, 1.00 equiv) and THF (7.5 mL), then *n*-BuLi (0.60 mL, 2.5 M in hexane, 1.5 mmol, 1.0 equiv) and di-*tert*-butyl dicarbonate (392 mg, 1.80 mmol, 1.20 equiv). Purification using a Biotage automated flash chromatography system (ethyl acetate/pentane, 0% to 30%) afforded **2ab** (396 mg, 1.25 mmol, 83 %) as a colourless oil.

^{1}H NMR (500 MHz, $CDCl_3$) δ 7.37 (d, J = 8.9 Hz, 2H), 6.86 (d, J = 8.9 Hz, 2H), 5.56 (t, J = 1.0 Hz, 1H), 5.54 (d, J = 0.9 Hz, 1H), 5.39 – 5.34 (m, 1H), 5.07 (d, J = 0.9 Hz, 1H), 3.80 (s, 3H), 2.06 (t, J = 1.3 Hz, 3H), 1.31 (s, 9H).

^{13}C NMR (126 MHz, $CDCl_3$) δ 173.9, 160.1, 153.0, 144.3, 142.7, 129.9, 127.2, 118.9, 114.0, 111.6, 83.4, 55.4, 27.7, 19.2.

IR (ATR): $\tilde{\nu}$ (cm^{-1}) = 1735, 1691, 1634, 1608, 1512, 1457, 1369, 1276, 1260, 1157, 1031.

HRMS (ESI^+) calcd. for $C_{18}H_{23}NO_4Na^+$ $[M+Na]^+$: 340.1519, found: 340.1507.

N-(1-(4-methoxyphenyl)vinyl)-*N*-pivaloylmethacrylamide (2ac)

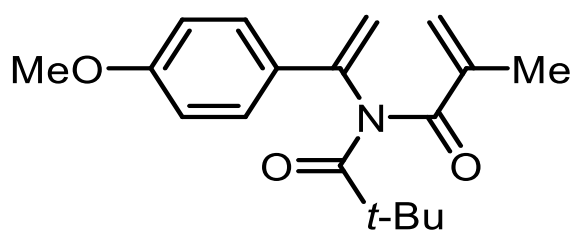

Prepared according to **General Procedure C4 (Step C4-2)** using **S2v** (326 mg, 1.50 mmol, 1.00 equiv) and THF (7.5 mL), then *n*-BuLi (0.60 mL, 2.5 M in hexane, 1.5 mmol, 1.0 equiv) and trimethylacetic anhydride (0.36 mL, 1.8 mmol, 1.2 equiv). Purification using a Biotage automated flash chromatography system (ethyl acetate/pentane, 0% to 25%) afforded **2ac** (381 mg, 1.27 mmol, 84 %) as a colourless oil.

^{1}H NMR (500 MHz, $CDCl_3$) δ 7.40 (d, J = 8.8 Hz, 2H), 6.87 (d, J = 8.9 Hz, 2H), 5.71 (s, 1H), 5.52 (d, J = 1.1 Hz, 1H), 5.37 (s, 1H), 4.90 (d, J = 1.1 Hz, 1H), 3.80 (s, 3H), 1.88 (dd, J = 1.6, 0.9 Hz, 3H), 1.31 (s, 9H).

^{13}C NMR (126 MHz, $CDCl_3$) δ 187.4, 175.2, 160.2, 145.0, 141.4, 129.0, 127.5, 120.8, 114.2, 110.7, 55.4, 43.5, 29.0, 19.1.

IR (ATR): $\tilde{\nu}$ (cm^{-1}) = 1689, 1608, 1511, 1459, 1276, 1258, 1177, 1144, 1031.

HRMS (ESI$^+$) calcd. for $C_{18}H_{24}NO_3^+$ [M+H]$^+$: 302.1751, found: 302.1750.

(*S*)-*N*-(1-(4-methoxyphenyl)vinyl)-*N*-(2-phenylpropanoyl)methacrylamide (2ad)

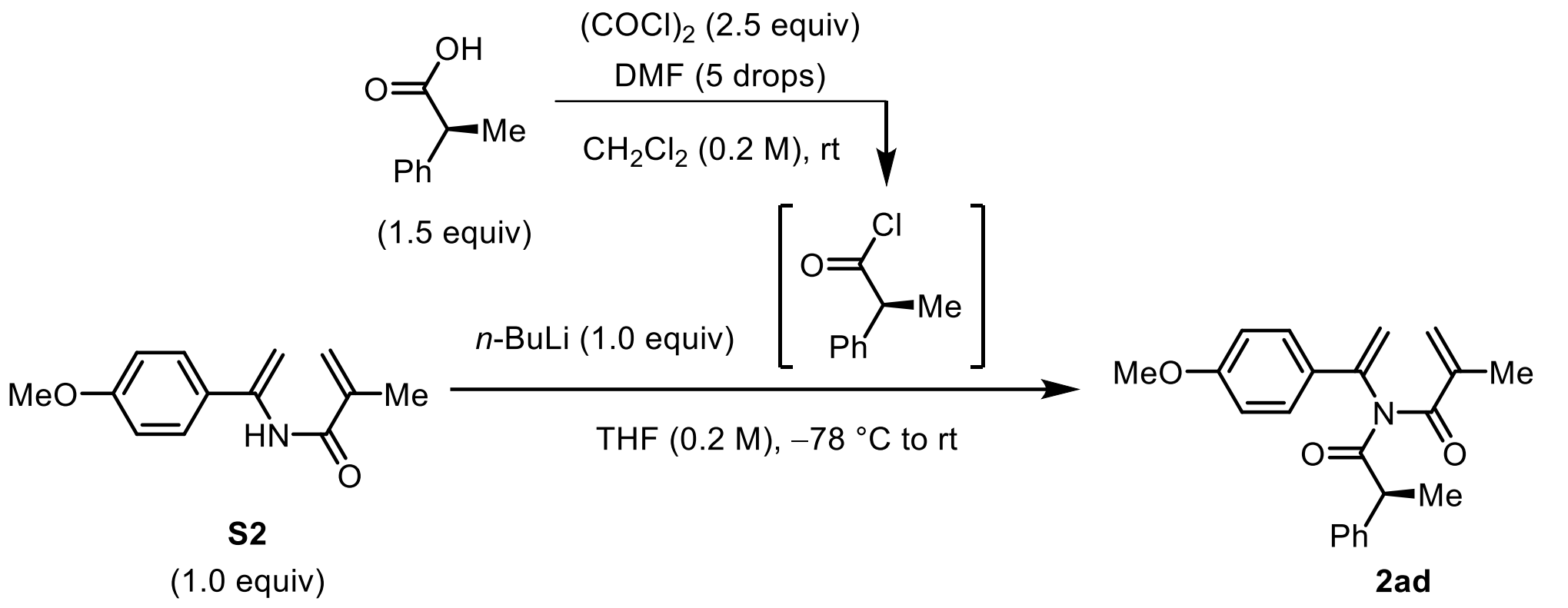

An oven-dried 100 mL round-bottom flask containing (*S*)-(+)-2-phenylpropionic acid (266 mg, 1.77 mmol, 1.50 equiv) was sealed and subjected to three N_2 evacuation/refill cycles before pre-sparged anhydrous CH_2Cl_2 (9.0 mL) was added. Oxalyl chloride (0.25 mL, 3.0 mmol, 2.5 equiv) was then added at 0 °C, followed by the addition of DMF (5 drops). The reaction mixture was allowed to warm up to room temperature and stirred for 1 h. The reaction was then concentrated under reduced pressure to afford the crude acyl chloride and used directly for the next step without further purification.

An oven-dried 50 mL round-bottom flask charged with **S2v** (255 mg, 1.18 mmol, 1.00 equiv) was sealed and subjected to three cycles of evacuation and nitrogen backfilling. Anhydrous THF (6.0 mL) was then added, and the reaction mixture was cooled to –78 °C. *n*-BuLi (2.5 M in hexane, 0.47 mL, 1.2 mmol, 1.0 equiv) was added dropwise at –78 °C, and the mixture was stirred at this temperature for 10 minutes. Crude acyl chloride (dissolved in 2.0 mL anhydrous THF) was subsequently added at –78 °C, and the reaction was allowed to warm to room temperature over 1 hour. The reaction was then quenched with brine (50 mL) and extracted with ethyl acetate (3 × 40 mL). The combined organic layers were dried over anhydrous $MgSO_4$, filtered, and concentrated under reduced pressure. The crude product was purified using a Biotage automated flash chromatography system (ethyl acetate/pentane, 0% to 25%) to afford **2ad** (165 mg, 0.47 mmol, 40 %) as a colourless oil.

^{1}H NMR (500 MHz, $CDCl_3$) δ 7.33 – 7.23 (m, 7H), 6.81 (d, J = 8.8 Hz, 2H), 5.63 (d, J = 1.1 Hz, 1H), 5.18 (d, J = 1.7 Hz, 1H), 5.15 (s, 1H), 4.89 (d, J = 1.1 Hz, 1H), 4.24 (q, J = 6.9 Hz, 1H), 3.79 (s, 3H), 1.88 (s, 3H), 1.43 (d, J = 7.0 Hz, 3H).

^{13}C NMR (126 MHz, $CDCl_3$) δ 178.1, 174.9, 160.3, 144.2, 141.4, 140.3, 128.8, 128.7, 128.1, 127.5, 127.2, 119.7, 114.2, 112.6, 55.4, 46.4, 19.1, 18.9.

IR (ATR): $\tilde{\nu}$ (cm^{-1}) = 1696, 1626, 1607, 1512, 1454, 1304, 1275, 1172, 1030.

HRMS (ESI$^+$) calcd. for $C_{22}H_{23}NO_3Na^+$ [M+Na]$^+$: 372.1570, found: 372.1568.

***N*-acetyl-*N*-(1-(4-methoxyphenyl)vinyl)acrylamide (2ae)**

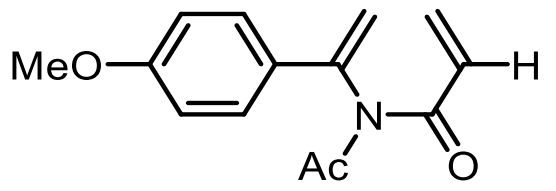

Prepared according to **General Procedure C1** using *N*-(1-(4-methoxyphenyl)vinyl)acetamide[10-12] (573 mg, 3.00 mmol, 1.00 equiv) and CH_2Cl_2 (12.0 mL), then Et_3N (1.25 mL, 9.00 mmol, 3.00 equiv) and acryloyl chloride (0.49 mL, 6.0 mmol, 2.0 equiv).[A] Purification using a Biotage automated flash chromatography system (ethyl acetate/pentane, 0% to 40%) afforded **2ae** (285 mg, 1.16 mmol, 39 %) as a pale-yellow oil.

Notes: (**A**) When acryloyl chloride is used as the electrophile, the addition of DMAP is not required to promote the reaction. Acryloyl chloride is a highly reactive reagent and is usually stored in a glovebox freezer at −30 °C. The reaction was complete within 1 h.

^{1}H NMR (500 MHz, $CDCl_3$) δ 7.33 (d, J = 8.9 Hz, 2H), 6.85 (d, J = 8.9 Hz, 2H), 6.62 (dd, J = 16.8, 10.2 Hz, 1H), 6.44 (dd, J = 16.8, 1.7 Hz, 1H), 5.83 (d, J = 1.1 Hz, 1H), 5.70 (dd, J = 10.2, 1.7 Hz, 1H), 5.14 (d, J = 1.2 Hz, 1H), 3.75 (s, 3H), 2.46 (s, 3H).

^{13}C NMR (126 MHz, $CDCl_3$) δ 173.0, 167.5, 160.3, 143.3, 130.5, 130.1, 127.9, 126.4, 114.3, 114.1, 55.2, 26.2.

IR (ATR): $\tilde{\nu}$ (cm^{-1}) = 1693, 1606, 1511, 1461, 1400, 1367, 1303, 1256, 1218, 1121, 1028.

HRMS (ESI$^+$) calcd. for $C_{14}H_{15}NO_3Na^+$ [M+Na]$^+$: 268.0944, found: 268.0934.

***N*-acetyl-*N*-(1-phenylvinyl)acrylamide (2af)**

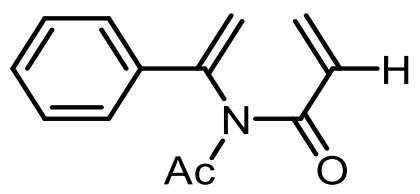

Prepared according to **General Procedure C1** using *N*-(1-phenylvinyl)acetamide[10-12] (483 mg, 3.00 mmol, 1.00 equiv) and CH_2Cl_2 (12.0 mL), then Et_3N (1.25 mL, 9.00 mmol, 3.00 equiv) and acryloyl chloride (0.49 mL, 6.0 mmol, 2.0 equiv).[**A**] Purification using a Biotage automated flash chromatography system (ethyl acetate/pentane, 0% to 30%) afforded **2af** (230 mg, 1.07 mmol, 36 %) as a colourless oil.

Notes: (**A**) When acryloyl chloride is used as the electrophile, the addition of DMAP is not required to promote the reaction. Acryloyl chloride is a highly reactive reagent and is usually stored in a glovebox freezer at −30 °C. The reaction was complete within 1 h.

^{1}H NMR (500 MHz, $CDCl_3$) δ 7.44 – 7.41 (m, 2H), 7.39 – 7.32 (m, 3H), 6.65 (dd, *J* = 16.7, 10.2 Hz, 1H), 6.49 (dd, *J* = 16.8, 1.7 Hz, 1H), 5.99 (d, *J* = 1.1 Hz, 1H), 5.74 (dd, *J* = 10.2, 1.7 Hz, 1H), 5.29 (d, *J* = 1.1 Hz, 1H), 2.50 (s, 3H).

^{13}C NMR (126 MHz, $CDCl_3$) δ 173.2, 167.7, 143.9, 135.5, 130.9, 130.2, 129.3, 129.1, 125.1, 116.3, 26.4.

IR (ATR): $\tilde{\nu}$ (cm^{-1}) = 1697, 1634, 1518, 1401, 1367, 1306, 1221, 1191, 1050.

HRMS (ESI^+) calcd. for $C_{13}H_{13}NO_2Na^+$ $[M+Na]^+$: 238.0838, found: 238.0837.

Methyl 4-(1-(*N*-acetylacrylamido)vinyl)benzoate (2ag)

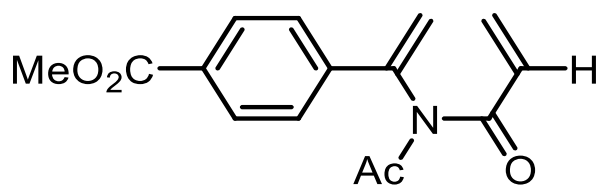

Prepared according to **General Procedure C1** using methyl 4-(1-acetamidovinyl)benzoate[12] (420 mg, 1.92 mmol, 1.00 equiv), CH_2Cl_2 (7.7 mL), Et_3N (0.80 mL, 5.8 mmol, 3.0 equiv) and acryloyl chloride (0.31 mL, 3.8 mmol, 2.0 equiv).**[A]** The reaction mixture was stirred at room temperature for 1 h**[B]**, after which a second portion of Et_3N (0.80 mL, 5.8 mmol, 3.0 equiv) and acryloyl chloride (0.31 mL, 3.8 mmol, 2.0 equiv) were added. The mixture was stirred at room temperature for an additional 1 h. Purification using a Biotage automated flash chromatography system (ethyl acetate/pentane, 0% to 30%) afforded **2ag** (210 mg, 0.77 mmol, 40 %) as a colourless oil.

Notes: (**A**) When acryloyl chloride is used as the electrophile, the addition of DMAP is not required to promote the reaction. Acryloyl chloride is a highly reactive reagent and is usually stored in a glovebox freezer at −30 °C.

(**B**) After 1 h, TLC analysis indicated the presence of unreacted starting material.

^{1}H NMR (500 MHz, $CDCl_3$) δ 8.03 (d, *J* = 8.5 Hz, 2H), 7.48 (d, *J* = 8.5 Hz, 2H), 6.61 (dd, *J* = 16.7, 10.1 Hz, 1H), 6.50 (dd, *J* = 16.8, 1.8 Hz, 1H), 6.08 (d, *J* = 1.3 Hz, 1H), 5.77 (dd, *J* = 10.1, 1.7 Hz, 1H), 5.40 (d, *J* = 1.2 Hz, 1H), 3.91 (s, 3H), 2.50 (s, 3H).

^{13}C NMR (126 MHz, $CDCl_3$) δ 173.1, 167.5, 166.5, 143.3, 139.9, 131.4, 130.8, 130.4, 129.9, 125.2, 118.4, 52.4, 26.4.

IR (ATR): $\tilde{\nu}$ (cm^{-1}) = 1700, 1632, 1609, 1435, 1401, 1368, 1275, 1217, 1185, 1107.

HRMS (ESI^+) calcd. for $C_{15}H_{15}NO_4Na^+$ $[M+Na]^+$: 296.0893, found: 296.0895.

N-acetyl-2-benzyl-N-(1-(4-methoxyphenyl)vinyl)acrylamide (2ah)

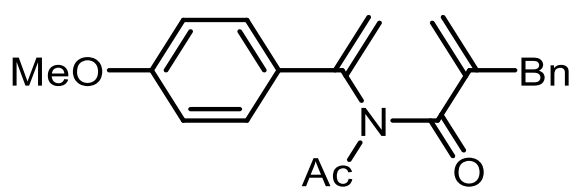

Prepared according to **General Procedure C3** using 2-benzylacrylic acid (972 mg, 6.00 mmol, 2.00 equiv), CH_2Cl_2 (20.0 mL), oxalyl chloride (0.76 mL, 9.0 mmol, 3.0 equiv) and DMF (5 drops). Then using CH_2Cl_2 (12.0 mL), Et_3N (1.25 mL, 9.00 mmol, 3.00 equiv), *N*-(1-(4-methoxyphenyl)vinyl)acetamide[10-12] (573 mg, 3.00 mmol, 1.00 equiv), and DMAP (110 mg, 0.90 mmol, 0.30 equiv). Purification by two successive flash chromatography separations using a Biotage automated flash chromatography system (ethyl acetate/pentane, 0% to 35% and ethyl acetate/CH_2Cl_2, 0% to 10%) afforded **2ah** (719 mg, 2.15 mmol, 72 %) as a pale-yellow oil.

^{1}H NMR (500 MHz, $CDCl_3$) δ 7.32 – 7.27 (m, 4H), 7.25 – 7.21 (m, 1H), 7.18 – 7.15 (m, 2H), 6.85 (d, *J* = 8.9 Hz, 2H), 5.63 – 5.60 (m, 2H), 5.17 (t, *J* = 1.7 Hz, 1H), 4.77 (d, *J* = 1.1 Hz, 1H), 3.80 (s, 3H), 3.62 (s, 2H), 2.29 (s, 3H).

^{13}C NMR (126 MHz, $CDCl_3$) δ 173.7, 173.6, 160.3, 145.3, 144.1, 137.6, 129.5, 128.6, 127.1, 126.7, 120.8, 114.3, 113.1, 55.4, 39.0, 25.3.

IR (ATR): $\tilde{\nu}$ (cm^{-1}) = 1688, 1627, 1605, 1511, 1366, 1252, 1178, 1029.

HRMS (ESI^+) calcd. for $C_{21}H_{21}NO_3Na^+$ $[M+Na]^+$: 358.1414, found: 358.1409.

N-acetyl-N-(1-(4-methoxyphenyl)vinyl)-2-(((triisopropylsilyl)oxy)methyl)acrylamide (2ai)

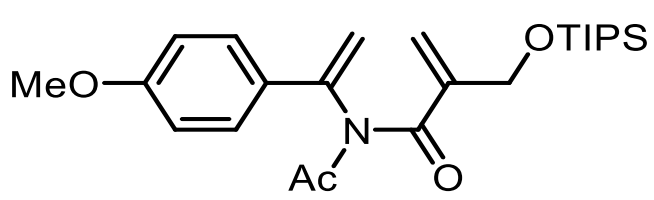

Prepared according to **General Procedure C3** using 2-(((triisopropylsilyl)oxy)methyl)acrylic acid[16] (3.10 g, 12.0 mmol, 2.00 equiv), CH_2Cl_2 (40.0 mL), oxalyl chloride (1.52 mL, 18.0 mmol, 3.00 equiv) and DMF (8 drops). Then using CH_2Cl_2 (24.0 mL), Et_3N (2.51 mL, 18.0 mmol, 3.00 equiv), *N*-(1-(4-methoxyphenyl)vinyl)acetamide[10-12] (1.15 g, 6.00 mmol, 1.00 equiv), and DMAP (220 mg, 1.80 mmol, 0.30 equiv). Purification by two successive flash chromatography separations using a Biotage automated flash chromatography system (ethyl acetate/pentane, 0% to 35% and ethyl acetate/CH_2Cl_2, 0% to 10%) afforded **2ai** (1.73 g, 4.01 mmol, 67 %) as a pale-yellow oil.

^{1}H NMR (500 MHz, $CDCl_3$) δ 7.36 (d, *J* = 8.9 Hz, 2H), 6.86 (d, *J* = 8.9 Hz, 2H), 5.70 (d, *J* = 1.1 Hz, 1H), 5.67 (td, *J* = 2.1, 0.8 Hz, 1H), 5.65 (td, *J* = 1.8 Hz, 0.9, 1H), 5.11 (d, *J* = 1.0 Hz, 1H), 4.40 (t, *J* = 1.9 Hz, 2H), 3.80 (s, 3H), 2.36 (s, 3H), 1.13 – 1.02 (m, 21H).

^{13}C NMR (126 MHz, $CDCl_3$) δ 173.7, 172.7, 160.3, 145.0, 144.3, 128.7, 127.1, 118.6, 114.3, 113.0, 62.8, 55.5, 25.6, 18.1, 12.0.

IR (ATR): $\tilde{\nu}$ (cm^{-1}) = 2942, 2865, 1691, 1629, 1607, 1512, 1461, 1366, 1303, 1250, 1119.

HRMS (ESI$^+$) calcd. for $C_{24}H_{38}NO_4Si^+$ [M+H]$^+$: 432.2565, found: 432.2581.

N-acetyl-*N*-(1-(4-methoxyphenyl)vinyl)-2-phenylacrylamide (2aj)

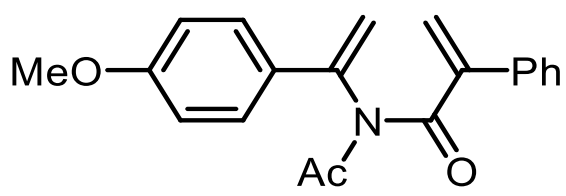

Prepared according to **General Procedure C3** using 2-phenylacrylic acid (888 mg, 6.00 mmol, 2.00 equiv), CH_2Cl_2 (20.0 mL), oxalyl chloride (0.76 mL, 9.0 mmol, 3.0 equiv), and DMF (5 drops). Then using CH_2Cl_2 (12.0 mL), Et_3N (1.25 mL, 9.00 mmol, 3.00 equiv), *N*-(1-(4-methoxyphenyl)vinyl)acetamide[10-12] (573 mg, 3.00 mmol, 1.00 equiv), and DMAP (110 mg, 0.90 mmol, 0.30 equiv). Purification by two successive flash chromatography separations using a Biotage automated flash chromatography system (ethyl acetate/pentane, 0% to 30% and ethyl acetate/CH_2Cl_2, 0% to 8%) afforded **2aj** (800 mg, 2.49 mmol, 83 %) as a colourless oil.

^{1}H NMR (500 MHz, $CDCl_3$) δ 7.38 – 7.30 (m, 5H), 7.29 (d, *J* = 8.9 Hz, 2H), 6.85 (d, *J* = 8.9 Hz, 2H), 5.70 (d, *J* = 1.2 Hz, 1H), 5.57 (s, 1H), 5.49 (s, 1H), 5.01 (d, *J* = 1.2 Hz, 1H), 3.80 (s, 3H), 2.36 (s, 3H).

^{13}C NMR (126 MHz, $CDCl_3$) δ 173.0, 172.5, 160.3, 146.2, 143.5, 136.4, 128.6, 128.50, 128.49, 126.9, 126.7, 118.2, 114.2, 55.4, 25.7.

IR (ATR): $\tilde{\nu}$ (cm^{-1}) = 1692, 1630, 1606, 1511, 1366, 1256, 1179, 1029.

HRMS (ESI$^+$) calcd. for $C_{20}H_{19}NO_3Na^+$ [M+Na]$^+$: 344.1257, found: 344.1254.

(*E*)-*N*-acetyl-*N*-(1-(4-methoxyphenyl)prop-1-en-1-yl)methacrylamide (2ak)

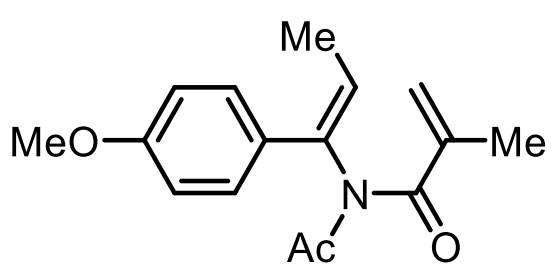

Prepared according to **General Procedure C1** using *N*-(1-phenylprop-1-en-1-yl)acetamide[10] (*Z*/*E* mixture, 615 mg, 3.00 mmol, 1.00 equiv) and CH_2Cl_2 (12.0 mL), then Et_3N (1.25 mL, 9.00 mmol, 3.00 equiv), methacryloyl chloride (0.59 mL, 6.0 mmol, 2.0 equiv), and DMAP (110 mg, 0.90 mmol, 0.30 equiv). Purification using a Biotage automated flash chromatography system (ethyl acetate/pentane, 0% to 30%) afforded **2ak** (680 mg, 2.49 mmol, 83 %) as a colourless oil.

^{1}H NMR (500 MHz, $CDCl_3$) δ 7.22 (d, *J* = 8.9 Hz, 2H), 6.82 (d, *J* = 8.9 Hz, 2H), 6.12 (q, *J* = 7.0 Hz, 1H), 5.35 (s, 1H), 5.24 (q, *J* = 1.6 Hz, 1H), 3.75 (s, 3H), 2.28 (s, 3H), 1.84 (dd, *J* = 1.7, 1.0 Hz, 3H), 1.72 (d, *J* = 7.0 Hz, 3H).

^{13}C NMR (126 MHz, $CDCl_3$) δ 173.9, 173.5, 159.5, 141.5, 137.4, 130.1, 126.6, 123.1, 118.9, 114.2, 55.3, 25.1, 19.1, 13.7.

IR (ATR): $\tilde{\nu}$ (cm^{-1}) = 1709, 1691, 1608, 1511, 1443, 1365, 1275, 1259, 1176, 1031.

HRMS (ESI$^+$) calcd. for $C_{16}H_{19}NO_3Na^+$ [M+Na]$^+$: 296.1257, found: 296.1246.

***N*-acetyl-*N*-(3,4-dihydronaphthalen-1-yl)methacrylamide (2al)**

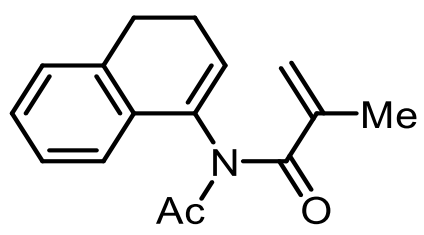

Prepared according to **General Procedure C1** using *N*-(3,4-dihydronaphthalen-1-yl)acetamide[11,12] (561 mg, 3.00 mmol, 1.00 equiv) and CH_2Cl_2 (12.0 mL), then Et_3N (1.25 mL, 9.00 mmol, 3.00 equiv), methacryloyl chloride (0.59 mL, 6.0 mmol, 2.0 equiv), and DMAP (110 mg, 0.90 mmol, 0.30 equiv). Purification using a Biotage automated flash chromatography system (ethyl acetate/pentane, 0% to 35%) afforded **2al** (630 mg, 2.47 mmol, 82 %) as a colourless oil.

^{1}H NMR (500 MHz, $CDCl_3$) δ 7.21 – 7.14 (m, 3H), 7.04 – 6.99 (m, 1H), 5.92 (t, *J* = 4.7 Hz, 1H), 5.47 – 5.43 (m, 1H), 5.26 – 5.24 (m, 1H), 2.92 – 2.80 (m, 2H), 2.50 – 2.40 (m, 2H), 2.34 (s, 3H), 1.96 (dd, *J* = 1.7, 1.0 Hz, 3H).

^{13}C NMR (126 MHz, $CDCl_3$) δ 174.0, 173.4, 141.4, 136.4, 136.3, 131.6, 128.7, 128.1, 128.0, 126.8, 121.9, 119.2, 27.1, 25.0, 23.0, 19.4.

IR (ATR): $\tilde{\nu}$ (cm^{-1}) = 1688, 1635, 1451, 1427, 1365, 1316, 1275, 1261, 1195.

HRMS (ESI$^+$) calcd. for $C_{16}H_{17}NO_2Na^+$ [M+Na]$^+$: 278.1152, found: 278.1153.

(*E*)-*N*-acetyl-*N*-(1-(4-methoxyphenyl)vinyl)but-2-enamide (2am)

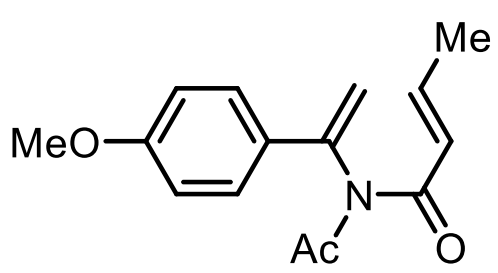

Prepared according to **General Procedure C2** using *N*-(1-(4-methoxyphenyl)vinyl)acetamide[10-12] (252 mg, 1.32 mmol, 1.00 equiv) and THF (6.6 mL), then *n*-BuLi (0.53 mL, 2.5 M in hexane, 1.3 mmol, 1.0 equiv) and crotonoyl chloride (0.17 mL, 1.7 mmol, 1.3 equiv). Purification using a Biotage automated flash chromatography system (ethyl acetate/pentane, 0% to 30%) afforded **2am** (140 mg, 0.54 mmol, 41 %) as a colourless oil.

^{1}H NMR (500 MHz, $CDCl_3$) δ 7.35 (d, J = 8.9 Hz, 2H), 7.07 (dq, J = 15.1, 7.0 Hz, 1H), 6.87 (d, J = 8.9 Hz, 2H), 6.34 (dq, J = 15.1, 1.7 Hz, 1H), 5.83 (d, J = 1.0 Hz, 1H), 5.14 (d, J = 1.0 Hz, 1H), 3.78 (s, 3H), 2.46 (s, 3H), 1.84 (dd, J = 7.0, 1.7 Hz, 3H).

^{13}C NMR (126 MHz, $CDCl_3$) δ 173.2, 167.8, 160.3, 145.6, 143.6, 128.3, 126.5, 124.5, 114.3, 114.0, 55.4, 26.3, 18.3.

IR (ATR): $\tilde{\nu}$ (cm^{-1}) = 1693, 1636, 1606, 1513, 1462, 1367, 1275, 1260, 1214, 1181.

HRMS (ESI^+) calcd. for $C_{15}H_{17}NO_3Na^+$ $[M+Na]^+$: 282.1101, found: 282.1089.

2-(3-Methylbenzyl)acrylic acid (S2an')

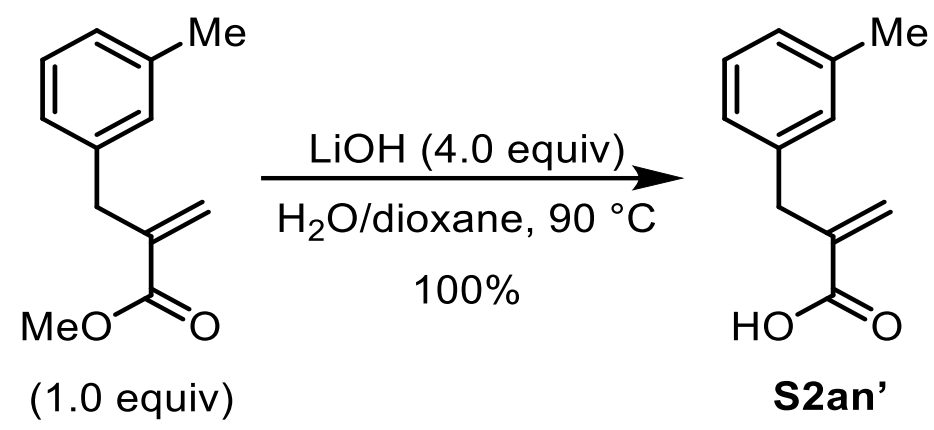

In a 250 mL round-bottom flask containing a solution of methyl 2-(3-methylbenzyl)acrylate[17] (11.3 g, 59.2 mmol, 1.00 equiv) in 1,4-dioxane (42.0 mL) and H_2O (120 mL) was added $LiOH·H_2O$ (9.95 g, 237 mmol, 4.00 equiv) at room temperature. The mixture was then stirred at 90 °C for 2 h. After that, the reaction was cooled to room temperature and extracted with Et_2O (50 mL). The aqueous phase was acidified with 2 N HCl until the solution reached pH = 1 and extracted with ethyl acetate (3 × 100 mL). The combined organic layers were dried over anhydrous $MgSO_4$, filtered, and concentrated under reduced pressure, affording **S2an'** (10.4 g, 59.1 mmol, 100%) as a colourless oil.

^{1}H NMR (500 MHz, $CDCl_3$) δ 11.96 (br. s, 1H), 7.25 (t, J = 7.4 Hz, 1H), 7.12-7.04 (m, 3H), 6.45 (d, J = 1.4 Hz, 1H), 5.64 (q, J = 1.5 Hz, 1H), 3.66 (s, 2H), 2.39 (s, 3H).

^{13}C NMR (126 MHz, $CDCl_3$) δ 172.9, 139.7, 138.5, 138.2, 129.9, 128.8, 128.5, 127.3, 126.2, 37.5, 21.5.

IR (ATR): $\tilde{\nu}$ (cm^{-1}) = 1691, 1627, 1608, 1433, 1294, 1219, 1148.

HRMS (Nanospray) calcd. for $C_{11}H_{11}O_2$ $[M-H]^-$: 175.0765, found: 175.0753.

N-(1-(3-(cyclopentyloxy)-4-methoxyphenyl)vinyl)acetamide (S2an)

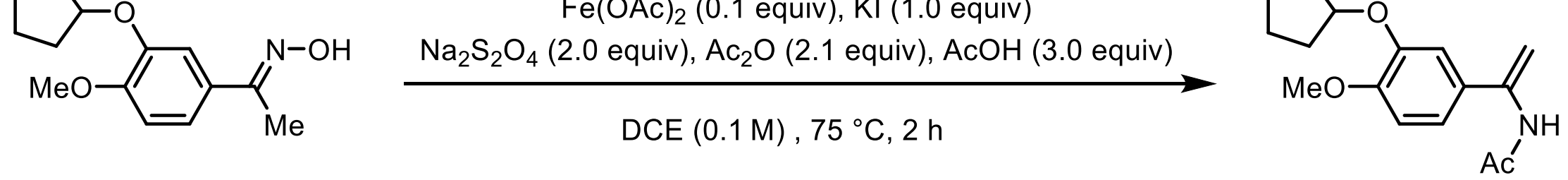

To an oven-dried round-bottom flask equipped with a magnetic stir bar and a 15 cm condenser, (*E*)-1-(3-(cyclopentyloxy)-4-methoxyphenyl)ethan-1-one oxime[18] (12.5 g, 50.0 mmol, 1.00 equiv), KI (8.30 g, 50.0 mmol, 1.00 equiv), $Na_2S_2O_4$ (17.4 g, 100 mmol, 2.00 equiv), $Fe(OAc)_2$ (870 mg, 5.00 mmol, 0.10 equiv) were added under an argon atmosphere in a glovebox. The flask was sealed with a rubber septum, removed from the glovebox, and kept under a nitrogen atmosphere using a balloon. 1,2-dichloroethane (500 mL), acetic acid (8.65 mL, 150 mmol, 3.00 equiv), and acetic anhydride (9.92 mL, 105 mmol, 2.10 equiv) were added to the mixture via syringe. The reaction mixture was heated to 75 °C for 2 h, then cooled to room temperature. The resulting mixture was quenched with saturated $NaHCO_3$ solution and extracted with CH_2Cl_2 three times. The combined organic layers were dried over anhydrous $MgSO_4$, filtered, concentrated under reduced pressure, and purified using a Biotage automated flash chromatography system with a gradient elution of ethyl acetate/pentane (0% to 60%) to afford the **S2an** (6.45 g, 23.5 mmol, 47 %) as a pale-yellow oil.

^{1}H NMR (500 MHz, $CDCl_3$) δ 7.26 (s, 1H), 6.91-6.86 (m, 2H), 6.78-6.73 (m, 1H), 5.70 (s, 1H), 4.94 (s, 1H), 4.74 (hept, *J* = 6.3, 3.2 Hz, 1H), 3.79 (s, 3H), 2.04 (s, 3H), 1.92 – 1.76 (m, 6H), 1.60 – 1.55 (m, 2H).

^{13}C NMR (126 MHz, $CDCl_3$) δ 169.3, 150.5, 147.5, 140.5, 131.3, 118.4, 113.4, 111.6, 101.3, 80.6, 56.0, 32.8, 24.4, 24.0.

IR (ATR): $\tilde{\nu}$ (cm^{-1}) = 1670, 1509, 1258, 1172, 1142.

HRMS (ESI^+) calcd. for $C_{16}H_{22}NO_3^+$ $[M+H]^+$: 276.1594, found: 276.1587.

N-acetyl-*N*-(1-(3-(cyclopentyloxy)-4-methoxyphenyl)vinyl)-2-(3-methylbenzyl) acrylamide (2an)

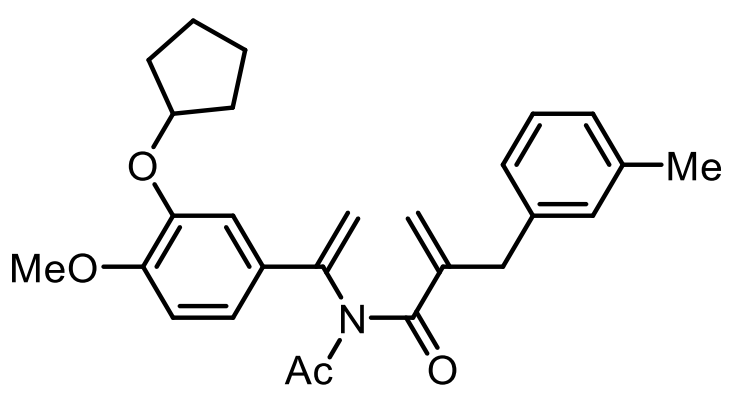

Prepared according to **General Procedure C3** using 2-(3-methylbenzyl)acrylic acid **S2an'** (2.07 g, 11.8 mmol, 2.00 equiv), CH_2Cl_2 (40.0 mL), oxalyl chloride (1.50 mL, 17.7 mmol, 3.00 equiv), and DMF (8 drops). Then using CH_2Cl_2 (23.6 mL), Et_3N (2.47 mL, 17.7 mmol, 3.00 equiv), *N*-(1-(3-(cyclopentyloxy)-4-methoxyphenyl)vinyl)acetamide **S2an** (1.62 g, 5.90 mmol, 1.00 equiv) in CH_2Cl_2 (6.0 mL), and DMAP (216 mg, 1.77 mmol, 0.30 equiv). Purification by two successive flash chromatography separations using a Biotage automated flash chromatography system with a gradient

elution of ethyl acetate/pentane (0% to 35%) and ethyl acetate/CH_2Cl_2 (0% to 10%) afforded **2an** (1.76 g, 4.06 mmol, 69 %) as a colourless oil.

^{1}H NMR (500 MHz, $CDCl_3$) δ 7.17 (t, *J* = 7.5 Hz, 1H), 7.06-7.02 (m, 1H), 6.98-6.93 (m, 2H), 6.92 – 6.89 (m, 2H), 6.82-6.78 (m, 1H), 5.63 (s, 1H), 5.60 (d, *J* = 1.1 Hz, 1H), 5.18 (t, *J* = 1.7 Hz, 1H), 4.80 (d, *J* = 1.2 Hz, 1H), 4.73 (hept, *J* =3.0 Hz, 1H), 3.83 (s, 3H), 3.57 (s, 2H), 2.32 (s, 3H), 2.30 (s, 3H), 1.92 – 1.80 (m, 6H), 1.64 – 1.57 (m, 2H).

^{13}C NMR (126 MHz, $CDCl_3$) δ 173.70, 173.66, 150.9, 147.8, 145.2, 144.2, 138.1, 137.5, 130.2, 128.9, 128.4, 127.3, 126.5, 120.9, 118.5, 113.0, 112.4, 111.8, 80.6, 56.0, 38.7, 32.8, 25.2, 24.1, 21.4.

IR (ATR): $\tilde{\nu}$ (cm^{-1}) = 2958, 1689, 1628, 1602, 1512, 1421, 1365, 1256, 1222, 1140.

HRMS (ESI^+) calcd. for $C_{27}H_{32}NO_4^+$ $[M+H]^+$: 434.2326, found: 434.2314.

2.4 Synthesis of Cyclisation Products

2.4.1 Synthesis and Characterisation of Products (3 and 4)

2-(4-Methoxybenzyl)-4-methyl-1-(4-(trifluoromethyl)phenyl)-2-azabicyclo[2.1.1]hexan-3-one (3a)

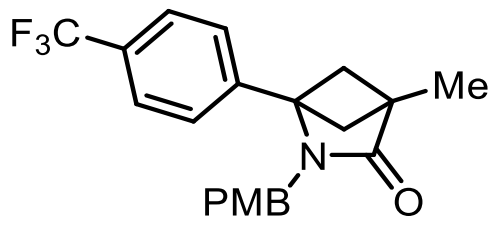

Prepared according to **General Procedure D** using **1a** (780 mg, 2.08 mmol, 1.00 equiv), CH_3CN (20.8 mL), and [Ir(dF(CF_3)ppy)$_2$(dtbbpy)](PF_6) (23.3 mg, 20.8 µmol, 1.0 mol%). The mixture was purified using a Biotage automated flash chromatography system with a gradient elution of ethyl acetate/pentane (0% to 30%), affording **3a** (513 mg, 1.37 mmol, 66%) as a white solid and **4a** (174 mg, 0.46 mmol, 22%) as a colourless oil.[A]

Notes: (**A**) The reaction time was limited to 0.5 h. Compound **4a** is not stable under the optimised conditions and gradually decomposes as the reaction progresses. After 30 minutes, the ^{1}H NMR spectrum showed a 2.7:1 molar ratio of **3a**/**4a**, and the starting material **1a** was completely consumed (as confirmed by TLC).

M.P.: 109-111 °C

^{1}H NMR (500 MHz, $CDCl_3$) δ 7.48 (d, J = 7.9 Hz, 2H), 7.09 (d, J = 7.9 Hz, 2H), 6.60 (d, J = 8.6 Hz, 2H), 6.57 (d, J = 8.6 Hz, 2H), 4.11 (s, 2H), 3.66 (s, 3H), 2.65 (dd, J = 4.0, 1.9 Hz, 2H), 2.37-2.31 (m, 2H), 1.31 (s, 3H).

^{13}C NMR (126 MHz, $CDCl_3$) δ 182.2, 158.6, 139.3, 130.4 (q, J = 32.4 Hz), 130.1, 129.3, 128.0, 125.1 (q, J = 3.8 Hz), 123.9 (q, J = 272.3 Hz), 113.4, 66.4, 57.9, 55.0, 48.2, 43.8, 13.3.

^{19}F NMR (471 MHz, $CDCl_3$) δ –62.62.

IR (ATR): $\tilde{\nu}$ (cm^{-1}) = 1715, 1612, 1512, 1324, 1266, 1241, 1167, 1125, 1067.

HRMS (ESI$^+$) calcd. for $C_{21}H_{21}F_3NO_2^+$ [M+H]$^+$: 376.1519, found: 376.1520.

1-(4-Methoxybenzyl)-3-methyl-6-(4-(trifluoromethyl)phenyl)-3,4-dihydropyridin-2(1*H*)-one (4a)

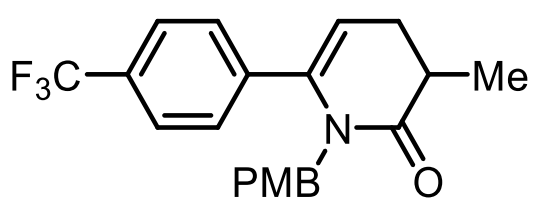

^{1}H NMR (500 MHz, $CDCl_3$) δ 7.55 (d, J = 8.0 Hz, 2H), 7.23 (d, J = 7.9 Hz, 2H), 6.77 (d, J = 8.7 Hz, 2H), 6.69 (d, J = 8.8 Hz, 2H), 5.37 (dd, J = 6.1, 3.9 Hz, 1H), 4.97 (d, J = 15.1 Hz, 1H), 4.34 (d, J =

15.3 Hz, 1H), 3.73 (s, 3H), 2.72 – 2.61 (m, 1H), 2.40 (dt, J = 16.4, 6.0 Hz, 1H), 2.13 (ddd, J = 16.2, 11.7, 4.0 Hz, 1H), 1.29 (d, J = 7.0 Hz, 3H).

^{13}C NMR (126 MHz, $CDCl_3$) δ 174.5, 158.7, 141.3, 139.8, 130.2 (q, J = 32.6 Hz), 129.9, 128.7, 128.1, 125.3 (q, J = 3.8 Hz), 124.1 (q, J = 272.1 Hz), 113.7, 111.7, 55.2, 45.7, 35.7, 27.9, 15.5.

^{19}F NMR (471 MHz, $CDCl_3$) δ –62.58.

IR (ATR): $\tilde{\nu}$ (cm^{-1}) = 1671, 1614, 1512, 1457, 1384, 1323, 1246, 1164, 1066.

HRMS (ESI^+) calcd. for $C_{21}H_{21}F_3NO_2^+$ $[M+H]^+$: 376.1519, found: 376.1521.

2-Benzyl-4-methyl-1-(4-(trifluoromethyl)phenyl)-2-azabicyclo[2.1.1]hexan-3-one (3b)

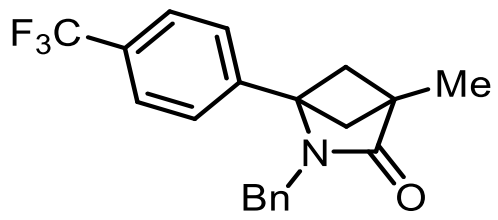

Prepared according to **General Procedure D** using **1b** (390 mg, 1.13 mmol, 1.00 equiv), CH_3CN (11.3 mL), and $[Ir(dF(CF_3)ppy)_2(dtbbpy)](PF_6)$ (12.7 mg, 11.3 μmol, 1.0 mol%). The mixture was purified using a Biotage automated flash chromatography system with a gradient elution of ethyl acetate/pentane (0% to 35%), affording **3b** (239 mg, 0.69 mmol, 61%) as a white solid and **4b** (81 mg, 0.23 mmol, 21%) as a colourless oil.[A]

Notes: (**A**) The reaction time was limited to 0.5 h. Compound **4b** is not stable under the optimised conditions and gradually decomposes as the reaction progresses. After 30 minutes, the ^{1}H NMR spectrum showed a 2.5:1 molar ratio of **3b**/**4b**, and the starting material **1b** was completely consumed (as confirmed by TLC).

M.P.: 60-62 °C

^{1}H NMR (500 MHz, $CDCl_3$) δ 7.48 (d, J = 8.1 Hz, 2H), 7.13 – 7.05 (m, 5H), 6.74 (d, J = 6.4 Hz, 2H), 4.20 (s, 2H), 2.73 (dd, J = 4.0, 2.0 Hz, 2H), 2.40 (dd, J = 3.8, 2.1 Hz, 2H), 1.37 (s, 3H).

^{13}C NMR (126 MHz, $CDCl_3$) δ 182.3, 139.1, 138.2, 130.6 (q, J = 32.4 Hz), 128.14, 128.07, 128.05, 127.1, 125.2 (q, J = 3.7 Hz), 124.0 (q, J = 272.1 Hz), 66.6, 58.1, 48.4, 44.6, 13.5.

^{19}F NMR (471 MHz, $CDCl_3$) δ –62.65.

IR (ATR): $\tilde{\nu}$ (cm^{-1}) = 1722, 1623, 1496, 1411, 1378, 1323, 1276, 1164, 1121.

HRMS (ESI^+) calcd. for $C_{20}H_{19}F_3NO^+$ $[M+H]^+$: 346.1413, found: 346.1401.

1-Benzyl-3-methyl-6-(4-(trifluoromethyl)phenyl)-3,4-dihydropyridin-2(1*H*)-one (4b)

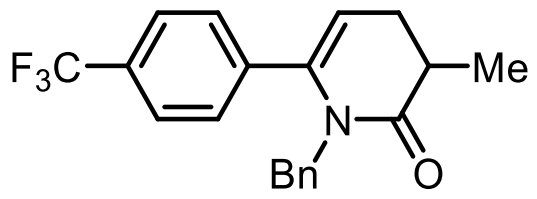

^{1}H NMR (500 MHz, $CDCl_3$) δ 7.54 (d, *J* = 8.1 Hz, 2H), 7.22 (d, *J* = 8.0 Hz, 2H), 7.19 – 7.15 (m, 3H), 6.88 – 6.84 (m, 2H), 5.39 (dd, *J* = 6.0, 3.9 Hz, 1H), 5.03 (d, *J* = 15.3 Hz, 1H), 4.42 (d, *J* = 15.4 Hz, 1H), 2.74-2.66 (m, 1H), 2.44 (dt, *J* = 16.5, 6.1 Hz, 1H), 2.18 (ddd, *J* = 16.2, 11.8, 4.0 Hz, 1H), 1.31 (d, *J* = 7.0 Hz, 3H).

^{13}C NMR (126 MHz, $CDCl_3$) δ 174.5, 141.4, 139.7 (q, *J* = 1.6 Hz), 137.8, 130.3 (q, *J* = 32.6 Hz), 128.4, 128.2, 127.3, 127.1, 125.3 (q, *J* = 3.7 Hz), 124.1 (d, *J* = 272.1 Hz), 111.6, 46.4, 35.8, 27.9, 15.6.

^{19}F NMR (471 MHz, $CDCl_3$) δ –62.61.

IR (ATR): $\tilde{\nu}$ (cm^{-1}) = 1670, 1486, 1455, 1380, 1220, 1087, 1015.

HRMS (ESI^+) calcd. for $C_{20}H_{19}F_3NO^+$ $[M+H]^+$: 346.1413, found: 346.1398.

2-Benzyl-1-(4-chlorophenyl)-4-methyl-2-azabicyclo[2.1.1]hexan-3-one (3c)

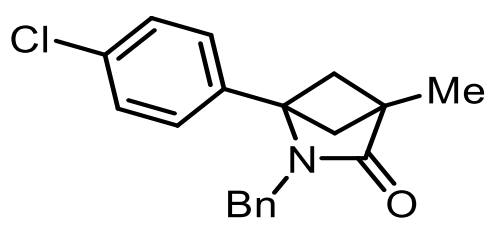

Prepared according to **General Procedure D** using **1c** (385 mg, 1.24 mmol, 1.00 equiv), CH_3CN (12.4 mL), and $[Ir(dF(CF_3)ppy)_2(dtbbpy)](PF_6)$ (13.9 mg, 12.4 μmol, 1.0 mol%). The mixture was purified using a Biotage automated flash chromatography system with a gradient elution of ethyl acetate/pentane (0% to 40%), affording **3c** (201 mg, 0.64 mmol, 52%) as a white solid and **4c** (54 mg, 0.17 mmol, 14%) as a colourless oil.[A,B]

Notes: (**A**) Due to the instability of **4c** under photoirradiation, a low yield (14%) was obtained. Notably, a higher yield of **4c** was observed when the reaction time was shortened to 15 min (see **Supplementary Table S1**).

(**B**) When HFIP was used as the solvent, **4c** was isolated in 72% yield while **3c** was detected in trace amounts in the crude NMR spectrum.

M.P.: 105-107 °C

^{1}H NMR (500 MHz, $CDCl_3$) δ 7.23 (d, *J* = 8.4 Hz, 2H), 7.15 – 7.10 (m, 3H), 6.93 (d, *J* = 8.4 Hz, 2H), 6.83 – 6.79 (m, 2H), 4.20 (s, 2H), 2.69 (dd, *J* = 4.0, 1.8 Hz, 2H), 2.37 (dd, *J* = 4.0, 1.9 Hz, 2H), 1.36 (s, 3H).

^{13}**C NMR** (126 MHz, $CDCl_3$) δ 182.5, 138.5, 134.5, 133.8, 129.1, 128.6, 128.2, 127.1, 66.7, 58.4, 48.4, 44.4, 13.6.

IR (ATR): $\tilde{\nu}$ (cm^{-1}) = 1722, 1601, 1495, 1400, 1377, 1276, 1260, 1090, 1016.

HRMS (ESI^+) calcd. for $C_{19}H_{19}ClNO^+$ $[M+H]^+$: 312.1150, found: 312.1138.

1-Benzyl-6-(4-chlorophenyl)-3-methyl-3,4-dihydropyridin-2(1*H*)-one (4c)

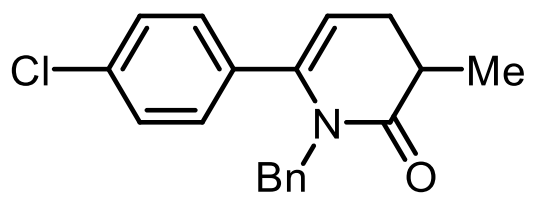

^{1}**H NMR** (500 MHz, $CDCl_3$) δ 7.25 (d, J = 8.5 Hz, 2H), 7.20 – 7.14 (m, 3H), 7.03 (d, J = 8.5 Hz, 2H), 6.91 – 6.87 (m, 2H), 5.31 (dd, J = 6.0, 3.9 Hz, 1H), 5.01 (d, J = 15.3 Hz, 1H), 4.42 (d, J = 15.4 Hz, 1H), 2.73 – 2.62 (m, 1H), 2.40 (dt, J = 16.4, 6.0 Hz, 1H), 2.14 (ddd, J = 16.2, 11.7, 3.9 Hz, 1H), 1.30 (d, J = 6.9 Hz, 3H).

^{13}**C NMR** (126 MHz, $CDCl_3$) δ 174.4, 141.3, 137.9, 134.6, 134.1, 129.2, 128.5, 128.3, 127.2, 127.0, 110.5, 46.3, 35.7, 27.8, 15.5.

IR (ATR): $\tilde{\nu}$ (cm^{-1}) = 1672, 1488, 1454, 1381, 1222, 1088, 1015.

HRMS (ESI^+) calcd. for $C_{19}H_{19}ClNO^+$ $[M+H]^+$: 312.1150, found: 312.1149.

4-(2-Benzyl-4-methyl-3-oxo-2-azabicyclo[2.1.1]hexan-1-yl)benzonitrile (3d)

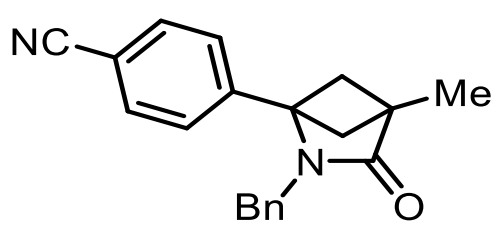

Prepared according to **General Procedure D** using **1d** (410 mg, 1.36 mmol, 1.00 equiv), CH_3CN (13.6 mL), and $[Ir(dF(CF_3)ppy)_2(dtbbpy)](PF_6)$ (15.2 mg, 13.5 μmol, 1.0 mol%). The crude reaction mixture was purified using a Biotage automated flash chromatography system with a gradient elution of ethyl acetate/pentane (0% to 50%), affording **3d** (275 mg, 0.91 mmol, 67%) as a white solid.

M.P.: 124-126 °C

^{1}**H NMR** (500 MHz, $CDCl_3$) δ 7.52 (d, J = 7.9 Hz, 2H), 7.13 – 7.07 (m, 5H), 6.74 (d, J = 6.7 Hz, 2H), 4.20 (s, 2H), 2.72 (dd, J = 4.1, 1.8 Hz, 2H), 2.40 (dd, J = 4.3, 1.5 Hz, 2H), 1.37 (s, 3H).

^{13}**C NMR** (126 MHz, $CDCl_3$) δ 182.3, 140.3, 138.0, 132.1, 128.5, 128.3, 128.1, 127.3, 118.5, 112.4, 66.6, 58.1, 48.4, 44.7, 13.5.

IR (ATR): $\tilde{\nu}$ (cm^{-1}) = 2228, 1718, 1612, 1454, 1395, 1357, 1276, 1132, 1017.

HRMS (ESI^+) calcd. for $C_{20}H_{19}N_2O^+$ $[M+H]^+$: 303.1492, found: 303.1486.

Methyl 4-(2-benzyl-4-methyl-3-oxo-2-azabicyclo[2.1.1]hexan-1-yl)benzoate (3e)

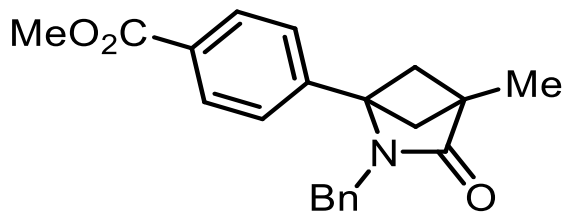

Prepared according to **General Procedure D** using **1e** (288 mg, 0.86 mmol, 1.00 equiv), CH_3CN (8.6 mL), and $[Ir(dF(CF_3)ppy)_2(dtbbpy)](PF_6)$ (9.6 mg, 8.6 μmol, 1.0 mol%). The crude reaction mixture was purified using a Biotage automated flash chromatography system with a gradient elution of ethyl acetate/pentane (0% to 40%), affording **3e** (151 mg, 0.45 mmol, 52%) as a colourless oil.

^{1}H NMR (500 MHz, $CDCl_3$) δ 7.90 (d, J = 8.4 Hz, 2H), 7.10 – 7.04 (m, 5H), 6.76 – 6.72 (m, 2H), 4.18 (s, 2H), 3.89 (s, 3H), 2.69 (dd, J = 4.0, 1.9 Hz, 2H), 2.38 (dd, J = 3.9, 2.0 Hz, 2H), 1.34 (s, 3H).

^{13}C NMR (126 MHz, $CDCl_3$) δ 182.4, 166.6, 140.0, 138.2, 130.1, 129.5, 128.12, 128.09, 127.7, 127.0, 66.8, 58.2, 52.2, 48.2, 44.4, 13.5.

IR (ATR): $\tilde{\nu}$ (cm^{-1}) = 1717, 1615, 1495, 1435, 1377, 1275, 1178, 1110, 1020.

HRMS (ESI^+) calcd. for $C_{21}H_{22}NO_3^+$ $[M+H]^+$: 336.1594, found: 336.1587.

2-Benzyl-1-(4-fluorophenyl)-4-methyl-2-azabicyclo[2.1.1]hexan-3-one (3f)

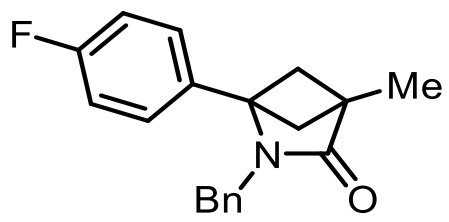

Prepared according to **General Procedure D** using **1f** (225 mg, 0.76 mmol, 1.00 equiv), CH_3CN (7.6 mL), and $[Ir(dF(CF_3)ppy)_2(dtbbpy)](PF_6)$ (8.6 mg, 7.6 μmol, 1 mol%). The crude reaction mixture was purified using a Biotage automated flash chromatography system with a gradient elution of ethyl acetate/pentane (0% to 40%), affording **3f** (98 mg, 0.33 mmol, 44%) as a white solid.

M.P.: 84-86 °C

^{1}H NMR (500 MHz, $CDCl_3$) δ 7.13 – 7.08 (m, 3H), 6.98 – 6.90 (m, 4H), 6.82 – 6.78 (m, 2H), 4.19 (s, 2H), 2.70 (dd, J = 4.0, 1.8 Hz, 2H), 2.37 (dd, J = 3.8, 2.0 Hz, 2H), 1.35 (s, 3H).

^{13}C NMR (126 MHz, $CDCl_3$) δ 182.4, 162.7 (d, J = 248.0 Hz), 138.5, 131.1 (d, J = 3.2 Hz), 129.5 (d, J = 8.3 Hz), 128.103, 128.097, 127.0, 115.3 (d, J = 21.5 Hz), 66.6, 58.4, 48.2, 44.3, 13.6.

^{19}F NMR (377 MHz, $CDCl_3$) δ –112.98.

IR (ATR): $\tilde{\nu}$ (cm^{-1}) = 1723, 1611, 1516, 1377, 1276, 1260, 1224, 1158, 1015.

HRMS (ESI$^+$) calcd. for $C_{19}H_{19}FNO^+$ $[M+H]^+$: 296.1445, found: 296.1431.

2-Benzyl-4-methyl-1-phenyl-2-azabicyclo[2.1.1]hexan-3-one (3g)

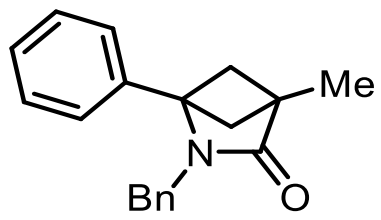

Prepared according to **General Procedure D** using **1g** (290 mg, 1.05 mmol, 1.00 equiv), CH_3CN (10.5 mL), and $[Ir(dF(CF_3)ppy)_2(dtbbpy)](PF_6)$ (11.7 mg, 10.4 μmol, 1.0 mol%). The crude reaction mixture was purified using a Biotage automated flash chromatography system with a gradient elution of ethyl acetate/pentane (0% to 40%), affording **3g** (136 mg, 0.49 mmol, 47%) as a colourless oil.

^{1}H NMR (500 MHz, $CDCl_3$) δ 7.33 – 7.24 (m, 3H), 7.13 – 7.08 (m, 3H), 7.03 – 7.00 (m, 2H), 6.82 – 6.78 (m, 2H), 4.20 (s, 2H), 2.71 (dd, J = 4.0, 1.9 Hz, 2H), 2.39 (dd, J = 3.9, 2.0 Hz, 2H), 1.36 (s, 3H).

^{13}C NMR (126 MHz, $CDCl_3$) δ 182.6, 138.6, 135.1, 128.4, 128.3, 128.2, 128.0, 127.6, 126.8, 67.2, 58.3, 48.2, 44.2, 13.6.

IR (ATR): $\tilde{\nu}$ (cm^{-1}) = 1717, 1495, 1448, 1376, 1357, 1276, 1260, 1130.

HRMS (ESI$^+$) calcd. for $C_{19}H_{20}NO^+$ $[M+H]^+$: 278.1539, found: 278.1534.

2-Benzyl-4-methyl-1-(*p*-tolyl)-2-azabicyclo[2.1.1]hexan-3-one (3h)

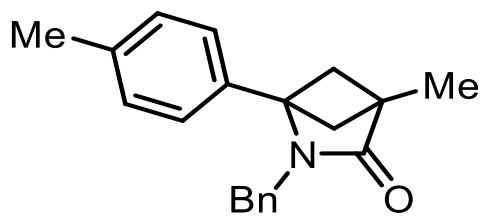

Prepared according to **General Procedure D** using **1h** (285 mg, 0.98 mmol, 1.00 equiv), CH_3CN (9.8 mL), and $[Ir(dF(CF_3)ppy)_2(dtbbpy)](PF_6)$ (11.0 mg, 9.80 μmol, 1.0 mol%). The crude reaction mixture was purified using a Biotage automated flash chromatography system with a gradient elution of ethyl acetate/pentane (0% to 35%), affording **3h** (113 mg, 0.39 mmol, 40%) as a white solid.

M.P.: 71-73 °C

^{1}H NMR (500 MHz, $CDCl_3$) δ 7.14 – 7.10 (m, 3H), 7.08 (d, J = 7.9 Hz, 2H), 6.92 (d, J = 8.0 Hz, 2H), 6.85 – 6.82 (m, 2H), 4.20 (s, 2H), 2.70 (dd, J = 4.0, 1.9 Hz, 2H), 2.37 (dd, J = 4.0, 1.9 Hz, 2H), 2.35 (s, 3H), 1.36 (s, 3H).

^{13}C NMR (126 MHz, $CDCl_3$) δ 182.7, 138.8, 138.3, 132.1, 129.0, 128.2, 128.0, 127.6, 126.8, 67.2, 58.4, 48.2, 44.2, 21.3, 13.6.

IR (ATR): $\tilde{\nu}$ (cm^{-1}) = 1722, 1475, 1454, 1394, 1376, 1276, 1261.

HRMS (ESI$^+$) calcd. for $C_{20}H_{22}NO^+$ $[M+H]^+$: 292.1696, found: 292.1690.

2-Benzyl-1-(4-methoxyphenyl)-4-methyl-2-azabicyclo[2.1.1]hexan-3-one (3i)

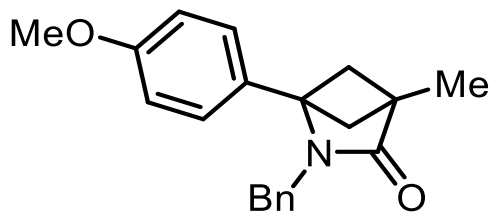

Prepared according to **General Procedure D** using **1i** (307 mg, 1.00 mmol, 1.00 equiv), CH_3CN (10.0 mL), and [Ir(dF(CF_3)ppy)$_2$(dtbbpy)](PF_6) (11.2 mg, 10.0 μmol, 1.0 mol%). The crude reaction mixture was purified using a Biotage automated flash chromatography system with a gradient elution of ethyl acetate/pentane (0% to 30%), affording **3i** (74 mg, 0.24 mmol, 24%) as a white solid.

M.P.: 70-72 °C

^{1}H NMR (500 MHz, $CDCl_3$) δ 7.14 – 7.10 (m, 3H), 6.92 (d, J = 8.7 Hz, 2H), 6.85 – 6.82 (m, 2H), 6.78 (d, J = 8.7 Hz, 2H), 4.19 (s, 2H), 3.80 (s, 3H), 2.69 (dd, J = 4.0, 1.9 Hz, 2H), 2.36 (dd, J = 3.7, 1.9 Hz, 2H), 1.35 (s, 3H).

^{13}C NMR (126 MHz, $CDCl_3$) δ 182.6, 159.7, 138.8, 129.0, 128.2, 128.1, 127.3, 126.9, 113.7, 67.0, 58.5, 55.4, 48.2, 44.1, 13.6.

IR (ATR): $\tilde{\nu}$ (cm^{-1}) = 1716, 1615, 1519, 1376, 1247, 1175, 1032.

HRMS (ESI$^+$) calcd. for $C_{20}H_{22}NO_2^+$ $[M+H]^+$: 308.1645, found: 308.1642.

2-(4-Methoxyphenyl)-4-methyl-1-(4-(trifluoromethyl)phenyl)-2-azabicyclo[2.1.1]hexan-3-one (3j)

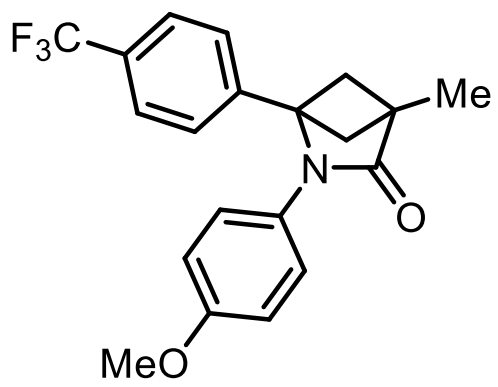

Prepared according to **General Procedure D** using **1j** (600 mg, 1.66 mmol, 1.00 equiv), CH_3CN (16.6 mL), and [Ir(dF(CF_3)ppy)$_2$(dtbbpy)](PF_6) (18.6 mg, 16.6 μmol, 1.0 mol%). The crude reaction mixture was purified using a Biotage automated flash chromatography system with a gradient elution of ethyl acetate/pentane (0% to 50%), affording **3j** (363 mg, 1.01 mmol, 61%) as a pale-yellow solid.

M.P.: 80-82 °C

^{1}H NMR (500 MHz, $CDCl_3$) δ 7.49 (d, *J* = 8.2 Hz, 2H), 7.23 (d, *J* = 7.9 Hz, 2H), 6.73 (d, *J* = 9.0 Hz, 2H), 6.63 (d, *J* = 9.1 Hz, 2H), 3.63 (s, 3H), 2.95 (dd, *J* = 4.2, 2.0 Hz, 2H), 2.59 (dd, *J* = 4.2, 2.0 Hz, 2H), 1.40 (s, 3H).

^{13}C NMR (126 MHz, $CDCl_3$) δ 181.3, 157.3, 139.6, 130.2 (q, *J* = 32.5 Hz), 129.5, 127.8, 127.1, 125.3 (q, *J* = 3.7 Hz), 123.9 (q, *J* = 272.3 Hz), 113.9, 69.1, 57.4, 55.1, 48.6, 13.4.

^{19}F NMR (471 MHz, $CDCl_3$) δ –62.57.

IR (ATR): $\tilde{\nu}$ (cm^{-1}) = 1748, 1725, 1509, 1324, 1290, 1165, 1123, 1067.

HRMS (ESI^+) calcd. for $C_{20}H_{19}F_3NO_2^+$ $[M+H]^+$: 362.1362, found: 362.1351.

4-(2-Benzyl-3-oxo-4-(trifluoromethyl)-2-azabicyclo[2.1.1]hexan-1-yl)benzonitrile (3k)

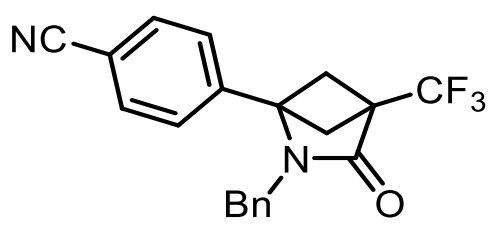

Prepared according to **General Procedure D** using **1k** (356 mg, 1.00 mmol, 1.00 equiv), CH_3CN (10.0 mL), and $[Ir(dF(CF_3)ppy)_2(dtbbpy)](PF_6)$ (11.2 mg, 10.0 µmol, 1.0 mol%). The crude reaction mixture was purified using a Biotage automated flash chromatography system with a gradient elution of ethyl acetate/pentane (0% to 50%), affording **3k** (295 mg, 0.83 mmol, 83%) as a white solid.

M.P.: 134-136 °C

^{1}H NMR (500 MHz, $CDCl_3$) δ 7.56 (d, *J* = 7.9 Hz, 2H), 7.23-7.01 (m, 5H), 6.72 (d, *J* = 7.5 Hz, 2H), 4.23 (s, 2H), 2.97 (s, 2H), 2.79 (s, 2H).

^{13}C NMR (126 MHz, $CDCl_3$) δ 173.2, 137.9, 136.8, 132.3, 128.4, 128.3, 127.9, 127.6, 122.6 (q, *J* = 274.8 Hz), 118.0, 113.1, 65.8, 53.7, 51.5 (q, *J* = 34.7 Hz), 44.4.

^{19}F NMR (471 MHz, $CDCl_3$) δ –71.21.

IR (ATR): $\tilde{\nu}$ (cm^{-1}) = 2231, 1737, 1482, 1405, 1265, 1218, 1182, 1161.

HRMS (ESI^+) calcd. for $C_{20}H_{16}F_3N_2O^+$ $[M+H]^+$: 357.1209, found: 357.1210.

2-Benzyl-1-(4-chlorophenyl)-4-(trifluoromethyl)-2-azabicyclo[2.1.1]hexan-3-one (3l)

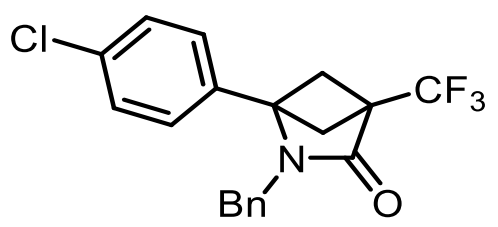

Prepared according to **General Procedure D** using **1l** (425 mg, 1.16 mmol, 1.00 equiv), CH_3CN (11.6 mL), and $[Ir(dF(CF_3)ppy)_2(dtbbpy)](PF_6)$ (13.1 mg, 11.7 µmol, 1.0 mol%). The crude reaction

mixture was purified using a Biotage automated flash chromatography system with a gradient elution of ethyl acetate/pentane (0% to 35%), affording **3l** (301 mg, 0.82 mmol, 71%) as a white solid.

M.P.: 67-69 °C

^{1}H NMR (500 MHz, $CDCl_3$) δ 7.28 (d, J = 8.5 Hz, 2H), 7.19 – 7.12 (m, 3H), 6.94 (d, J = 8.4 Hz, 2H), 6.81 – 6.77 (m, 2H), 4.24 (s, 2H), 2.93 (dd, J = 4.1, 1.9 Hz, 2H), 2.76 (dd, J = 4.1, 2.0 Hz, 2H).

^{13}C NMR (126 MHz, $CDCl_3$) δ 173.5 (q, J = 2.6 Hz), 137.3, 135.5, 131.5, 129.0, 128.9, 128.4, 128.2, 127.5, 122.8 (q, J = 274.9 Hz), 66.0, 53.9 (q, J = 2.6 Hz), 51.6 (q, J = 34.7 Hz), 44.3.

^{19}F NMR (471 MHz, $CDCl_3$) δ –71.29.

IR (ATR): $\tilde{\nu}$ (cm^{-1}) = 1734, 1480, 1403, 1320, 1217, 1182, 1153, 1119, 1091.

HRMS (ESI^+) calcd. for $C_{19}H_{16}ClF_3NO^+$ $[M+H]^+$: 366.0867, found: 366.0862.

2-Benzyl-1-(4-fluorophenyl)-4-(trifluoromethyl)-2-azabicyclo[2.1.1]hexan-3-one (3m)

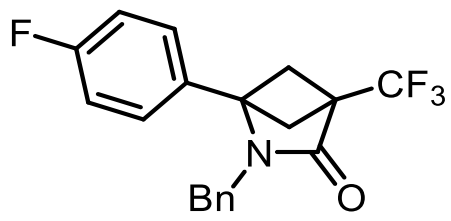

Prepared according to **General Procedure D** using **1m** (349 mg, 1.00 mmol, 1.00 equiv), CH_3CN (10.0 mL), and $[Ir(dF(CF_3)ppy)_2(dtbbpy)](PF_6)$ (11.2 mg, 10.0 μmol, 1.0 mol%). The crude reaction mixture was purified using a Biotage automated flash chromatography system with a gradient elution of ethyl acetate/pentane (0% to 30%), affording **3m** (192 mg, 0.55 mmol, 55%) as a colourless oil.

^{1}H NMR (500 MHz, $CDCl_3$) δ 7.18 – 7.11 (m, 3H), 7.02 – 6.96 (m, 4H), 6.80 – 6.77 (m, 2H), 4.23 (s, 2H), 2.94 (dd, J = 4.1, 1.9 Hz, 2H), 2.76 (dd, J = 3.8, 2.1 Hz, 2H).

^{13}C NMR (126 MHz, $CDCl_3$) δ 173.5 (q, J = 2.6 Hz), 163.2 (d, J = 249.2 Hz), 137.4, 129.5 (d, J = 8.4 Hz), 128.9 (d, J = 3.2 Hz), 128.4, 128.1, 127.4, 122.8 (q, J = 274.8 Hz), 115.7 (d, J = 21.8 Hz), 66.0, 54.0 (q, J = 2.3 Hz), 51.5 (q, J = 34.5 Hz), 44.2.

^{19}F NMR (471 MHz, $CDCl_3$) δ –71.29, -111.37.

IR (ATR): $\tilde{\nu}$ (cm^{-1}) = 1733, 1604, 1518, 1481, 1403, 1276, 1217, 1181, 1118.

HRMS (ESI^+) calcd. for $C_{19}H_{16}F_4NO^+$ $[M+H]^+$: 350.1163, found: 350.1159.

2-Benzyl-1-phenyl-4-(trifluoromethyl)-2-azabicyclo[2.1.1]hexan-3-one (3n)

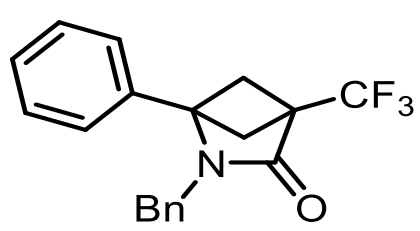

Prepared according to **General Procedure D** using **1n** (185 mg, 0.56 mmol, 1.00 equiv), CH_3CN (5.6 mL), and $[Ir(dF(CF_3)ppy)_2(dtbbpy)](PF_6)$ (6.3 mg, 5.6 µmol, 1.0 mol%). The crude reaction mixture was purified using a Biotage automated flash chromatography system with a gradient elution of ethyl acetate/pentane (0% to 35%), affording **3n** (120 mg, 0.36 mmol, 65%) as a white solid.

M.P.: 55-57 °C

^{1}H NMR (500 MHz, $CDCl_3$) δ 7.41 – 7.37 (m, 1H), 7.37 – 7.29 (m, 2H), 7.18 – 7.11 (m, 3H), 7.04 – 7.00 (m, 2H), 6.80 – 6.76 (m, 2H), 4.25 (s, 2H), 2.95 (dd, J = 4.1, 1.9 Hz, 2H), 2.78 (dd, J = 3.7, 2.2 Hz, 2H).

^{13}C NMR (126 MHz, $CDCl_3$) δ 173.7 (q, J = 2.6 Hz), 137.5, 132.9, 129.3, 128.7, 128.3, 127.5, 127.3, 122.9 (q, J = 274.7 Hz), 66.6, 54.0 (q, J = 2.3 Hz), 51.6 (q, J = 34.5 Hz), 44.1.

^{19}F NMR (471 MHz, $CDCl_3$) δ –71.26.

IR (ATR): $\tilde{\nu}$ (cm^{-1}) = 1735, 1481, 1450, 1404, 1321, 1276, 1261, 1217, 1181.

HRMS (ESI$^+$) calcd. for $C_{19}H_{17}F_3NO^+$ [M+H]$^+$: 332.1257, found: 332.1249.

2-Benzyl-1-(*p*-tolyl)-4-(trifluoromethyl)-2-azabicyclo[2.1.1]hexan-3-one (3o)

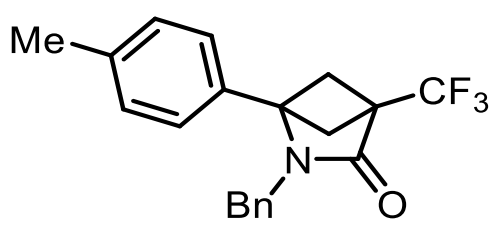

Prepared according to **General Procedure D** using **1o** (345 mg, 1.00 mmol, 1.00 equiv), CH_3CN (10.0 mL), and $[Ir(dF(CF_3)ppy)_2(dtbbpy)](PF_6)$ (11.2 mg, 10.0 µmol, 1.0 mol%). The crude reaction mixture was purified using a Biotage automated flash chromatography system with a gradient elution of ethyl acetate/pentane (0% to 35%), affording **3o** (178 mg, 0.52 mmol, 52%) as a white solid.

M.P.: 66-68 °C

^{1}H NMR (500 MHz, $CDCl_3$) δ 7.19 – 7.12 (m, 5H), 6.92 (d, J = 8.2 Hz, 2H), 6.84 – 6.80 (m, 2H), 4.24 (s, 2H), 2.93 (dd, J = 4.1, 1.9 Hz, 2H), 2.75 (dd, J = 4.0, 2.1 Hz, 2H), 2.39 (s, 3H).

^{13}C NMR (126 MHz, $CDCl_3$) δ 173.8 (q, J = 2.3 Hz), 139.3, 137.6, 129.9, 129.3, 128.3, 128.2, 127.4, 127.3, 122.9 (q, J = 274.8 Hz), 66.5, 54.0 (q, J = 2.3 Hz), 51.6 (q, J = 34.3 Hz), 44.1, 21.3.

^{19}F NMR (471 MHz, $CDCl_3$) δ –71.26.

IR (ATR): $\tilde{\nu}$ (cm^{-1}) = 1733, 1403, 1275, 1261, 1218, 1180, 1150, 1109.

HRMS (ESI$^+$) calcd. for $C_{20}H_{19}F_3NO^+$ [M+H]$^+$: 346.1413, found: 346.1403.

2-Benzyl-1-(4-methoxyphenyl)-4-(trifluoromethyl)-2-azabicyclo[2.1.1]hexan-3-one (3p)

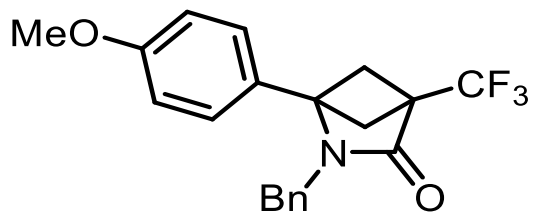

Prepared according to **General Procedure D** using **1p** (375 mg, 1.04 mmol, 1.00 equiv), CH_3CN (10.4 mL), and [Ir(dF(CF_3)ppy)$_2$(dtbbpy)](PF_6) (11.7 mg, 10.4 μmol, 1.0 mol%). The crude reaction mixture was purified using a Biotage automated flash chromatography system with a gradient elution of Et_2O/pentane (0% to 50%), affording **3p** (143 mg, 0.40 mmol, 38%) as a colourless oil.

^{1}H NMR (500 MHz, $CDCl_3$) δ 7.18 – 7.12 (m, 3H), 6.92 (d, J = 8.7 Hz, 2H), 6.86 – 6.80 (m, 4H), 4.23 (s, 2H), 3.83 (s, 3H), 2.92 (dd, J = 4.1, 1.9 Hz, 2H), 2.74 (dd, J = 3.9, 2.0 Hz, 2H).

^{13}C NMR (126 MHz, $CDCl_3$) δ 173.7 (q, J = 2.6 Hz), 160.3, 137.7, 128.9, 128.29, 128.28, 127.3, 125.0, 122.9 (q, J = 274.9 Hz), 114.1, 66.4, 55.5, 54.1 (q, J = 2.2 Hz), 51.6 (q, J = 34.3 Hz), 44.0.

^{19}F NMR (471 MHz, $CDCl_3$) δ –71.29.

IR (ATR): $\tilde{\nu}$ (cm^{-1}) = 1732, 1616, 1521, 1481, 1403, 1275, 1217, 1179, 1152.

HRMS (ESI$^+$) calcd. for $C_{20}H_{19}F_3NO_2^+$ [M+H]$^+$: 362.1362, found: 362.1353.

2-Benzyl-1-(4-(trifluoromethyl)phenyl)-2-azabicyclo[2.1.1]hexan-3-one (3q)

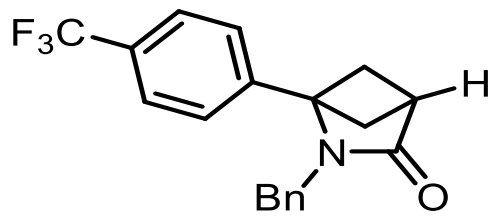

Prepared according to **General Procedure D** using **1q** (331 mg, 1.00 mmol, 1.00 equiv), CH_3CN (11.2 mL), and [Ir(dF(CF_3)ppy)$_2$(dtbbpy)](PF_6) (11.2 mg, 10.0 μmol, 1.0 mol%). The crude reaction mixture was purified using a Biotage automated flash chromatography system with a gradient elution of ethyl acetate/pentane (0% to 50%), affording **3q** (156 mg, 0.47 mmol, 47%) as a pale-yellow solid.

M.P.: 52-54 °C

^{1}H NMR (500 MHz, $CDCl_3$) δ 7.50 (d, J = 8.0 Hz, 2H), 7.14 – 7.07 (m, 5H), 6.77 – 6.74 (m, 2H), 4.21 (s, 2H), 2.93 (t, J = 2.7 Hz, 1H), 2.80 – 2.75 (m, 2H), 2.62 – 2.59 (m, 2H).

^{13}C NMR (126 MHz, $CDCl_3$) δ 181.9, 139.1 (q, J = 1.6 Hz), 138.0, 130.8 (q, J = 32.6 Hz), 128.2, 128.1, 128.0, 127.2, 125.4 (q, J = 3.8 Hz), 124.0 (q, J = 272.1 Hz), 70.6, 54.3, 44.3, 43.2.

^{19}F NMR (471 MHz, $CDCl_3$) δ –62.70.

IR (ATR): $\tilde{\nu}$ (cm^{-1}) = 1721, 1621, 1496, 1411, 1382, 1322, 1164, 1119, 1105.

HRMS (ESI$^+$) calcd. for $C_{19}H_{17}F_3NO^+$ [M+H]$^+$: 332.1257, found: 332.1250.

2-Benzyl-4-phenyl-1-(4-(trifluoromethyl)phenyl)-2-azabicyclo[2.1.1]hexan-3-one (3r)

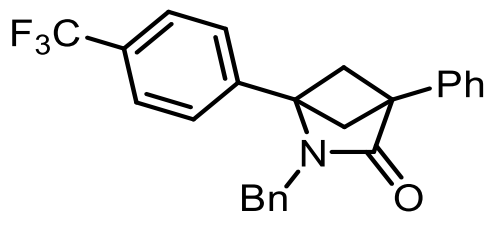

Prepared according to **General Procedure D** using **1r** (430 mg, 1.06 mmol, 1.00 equiv), CH_3CN (10.6 mL), and [Ir(dF(CF_3)ppy)$_2$(dtbbpy)](PF_6) (11.9 mg, 10.6 μmol, 1.0 mol%). The crude reaction mixture was purified using a Biotage automated flash chromatography system with a gradient elution of ethyl acetate/pentane (0% to 35%), affording **3r** (180 mg, 0.44 mmol, 42%) as a white solid.

M.P.: 89-91 °C

^{1}H NMR (500 MHz, $CDCl_3$) δ 7.58 (d, *J* = 8.0 Hz, 2H), 7.46 – 7.40 (m, 4H), 7.37 – 7.33 (m, 1H), 7.23 (d, *J* = 8.0 Hz, 2H), 7.20 – 7.14 (m, 3H), 6.87 – 6.83 (m, 2H), 4.34 (s, 2H), 3.18 (dd, *J* = 4.0, 1.9 Hz, 2H), 2.82 (dd, *J* = 3.9, 1.9 Hz, 2H).

^{13}C NMR (126 MHz, $CDCl_3$) δ 179.9, 138.8, 138.0, 135.8, 130.8 (q, *J* = 32.7 Hz), 128.5, 128.24, 128.22, 128.1, 127.6, 127.22, 127.17, 125.4 (q, *J* = 3.7 Hz), 124.0 (q, *J* = 272.1 Hz), 65.8, 57.6, 55.0, 44.8.

^{19}F NMR (471 MHz, $CDCl_3$) δ –62.57.

IR (ATR): $\tilde{\nu}$ (cm^{-1}) = 1722, 1411, 1321, 1165, 1120, 1067.

HRMS (ESI$^+$) calcd. for $C_{25}H_{21}F_3NO^+$ [M+H]$^+$: 408.1570, found: 408.1562.

2.4.2 Synthesis and Characterisation of Products (8)

2-(*tert*-Butyl) 1-methyl 3-oxo-4-phenyl-2-azabicyclo[2.1.1]hexane-1,2-dicarboxylate (8a)

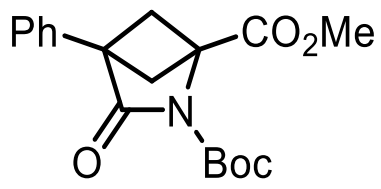

Prepared according to **General Procedure D** using **7a** (460 mg, 1.39 mmol, 1.00 equiv), CH_3CN (14.0 mL), and [Ir(dF(CF_3)ppy)$_2$(dtbbpy)](PF_6) (15.6 mg, 13.9 µmol, 1.0 mol%). The crude reaction mixture was purified using a Biotage automated flash chromatography system with a gradient elution of ethyl acetate/pentane (0% to 30%), affording **8a** (382 mg, 1.15 mmol, 83%) as a white solid.

M.P.: 124-126 °C

^{1}H NMR (500 MHz, $CDCl_3$) δ 7.39 – 7.35 (m, 2H), 7.34 – 7.30 (m, 1H), 7.25 – 7.22 (m, 2H), 3.83 (s, 3H), 2.91 (dd, J = 4.8, 2.0 Hz, 2H), 2.81 (dd, J = 4.8, 2.0 Hz, 2H), 1.51 (s, 9H).

^{13}C NMR (126 MHz, $CDCl_3$) δ 174.2, 166.7, 149.6, 133.7, 128.7, 128.2, 127.0, 83.9, 62.9, 56.1, 53.0, 52.6, 28.1.

IR (ATR): $\tilde{\nu}$ (cm^{-1}) = 1801, 1751, 1718, 1439, 1370, 1309, 1259, 1218, 1117.

HRMS (ESI$^+$) calcd. for $C_{18}H_{21}NO_5Na^+$ [M+Na]$^+$: 354.1312, found: 354.1296.

2-(*tert*-Butyl) 1-methyl 4-(4-fluorophenyl)-3-oxo-2-azabicyclo[2.1.1]hexane-1,2-dicarboxylate (8b)

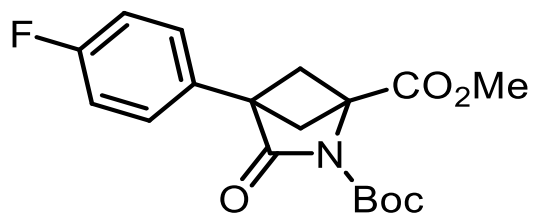

Prepared according to **General Procedure D** using **7b** (386 mg, 1.10 mmol, 1.00 equiv), CH_3CN (11.0 mL), and [Ir(dF(CF_3)ppy)$_2$(dtbbpy)](PF_6) (12.4 mg, 11.1 µmol, 1.0 mol%). The crude reaction mixture was purified using a Biotage automated flash chromatography system with a gradient elution of ethyl acetate/pentane (0% to 25%), affording **8b** (330 mg, 0.95 mmol, 85%) as a white solid.

M.P.: 99-101 °C

^{1}H NMR (500 MHz, $CDCl_3$) δ 7.19 – 7.16 (m, 2H), 7.02 – 6.97 (m, 2H), 3.78 (s, 3H), 2.84 (dd, J = 4.8, 2.0 Hz, 2H), 2.76 (dd, J = 4.8, 2.0 Hz, 2H), 1.46 (s, 9H).

^{13}C NMR (126 MHz, $CDCl_3$) δ 174.0, 166.4, 162.4 (d, J = 247.0 Hz), 149.3, 129.5 (d, J = 3.1 Hz), 128.8 (d, J = 8.3 Hz), 115.5 (d, J = 21.5 Hz), 83.8, 62.7, 55.3, 52.9, 52.4, 27.9.

^{19}F NMR (471 MHz, $CDCl_3$) δ –113.63.

IR (ATR): $\tilde{\nu}$ (cm^{-1}) = 1798, 1749, 1716, 1610, 1517, 1439, 1369, 1218, 1116.

HRMS (ESI^+) calcd. for $C_{18}H_{20}FNO_5Na^+$ $[M+Na]^+$: 372.1218, found: 372.1200.

2-(*tert*-Butyl) 1-methyl 4-(3-fluorophenyl)-3-oxo-2-azabicyclo[2.1.1]hexane-1,2-dicarboxylate (8c)

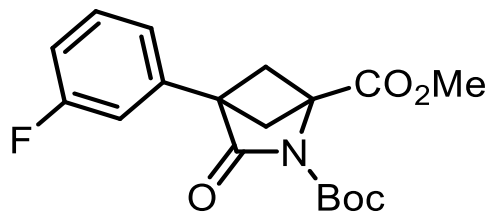

Prepared according to **General Procedure D** using **7c** (174 mg, 0.50 mmol, 1.00 equiv), CH_3CN (5.0 mL), and $[Ir(dF(CF_3)ppy)_2(dtbbpy)](PF_6)$ (5.6 mg, 5.0 µmol, 1.0 mol%). The crude reaction mixture was purified using a Biotage automated flash chromatography system with a gradient elution of ethyl acetate/pentane (0% to 25%), affording **8c** (151 mg, 0.43 mmol, 87%) as a white solid.

M.P.: 128-130 °C

^{1}H NMR (500 MHz, $CDCl_3$) δ 7.32 (td, J = 8.0, 5.8 Hz, 1H), 7.02 – 6.97 (m, 2H), 6.95 (ddd, J = 9.6, 2.6, 1.7 Hz, 1H), 3.82 (s, 3H), 2.89 (dd, J = 4.8, 2.0 Hz, 2H), 2.79 (dd, J = 4.8, 2.0 Hz, 2H), 1.49 (s, 9H).

^{13}C NMR (126 MHz, $CDCl_3$) δ 173.7, 166.5, 162.8 (d, J = 246.7 Hz), 149.4, 136.1 (d, J = 7.7 Hz), 130.3 (d, J = 8.3 Hz), 122.8 (d, J = 3.0 Hz), 115.2 (d, J = 21.0 Hz), 114.3 (d, J = 22.3 Hz), 84.0, 62.8, 55.6 (d, J = 2.1 Hz), 53.0, 52.6, 28.0.

^{19}F NMR (471 MHz, $CDCl_3$) δ –112.36.

IR (ATR): $\tilde{\nu}$ (cm^{-1}) = 1801, 1787, 1749, 1716, 1589, 1442, 1369, 1300, 1156.

HRMS (ESI^+) calcd. for $C_{18}H_{20}FNO_5Na^+$ $[M+Na]^+$: 372.1218, found: 372.1201.

2-(*tert*-Butyl) 1-methyl 4-(4-chlorophenyl)-3-oxo-2-azabicyclo[2.1.1]hexane-1,2-dicarboxylate (8d)

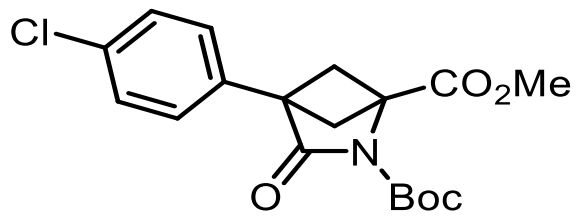

Prepared according to **General Procedure D** using **7d** (295 mg, 0.81 mmol, 1.00 equiv), CH_3CN (8.1 mL), and $[Ir(dF(CF_3)ppy)_2(dtbbpy)](PF_6)$ (9.1 mg, 8.1 µmol, 1.0 mol%). The crude reaction mixture was purified using a Biotage automated flash chromatography system with a gradient elution of ethyl acetate/pentane (0% to 25%), affording **8d** (256 mg, 0.70 mmol, 88%) as a white solid.

M.P.: 109-111 °C

^{1}H NMR (500 MHz, $CDCl_3$) δ 7.31 (d, J = 8.5 Hz, 2H), 7.15 (d, J = 8.5 Hz, 2H), 3.80 (s, 3H), 2.86 (dd, J = 4.8, 2.0 Hz, 2H), 2.77 (dd, J = 4.8, 2.0 Hz, 2H), 1.48 (s, 9H).

^{13}C NMR (126 MHz, $CDCl_3$) δ 173.8, 166.4, 149.3, 134.1, 132.2, 128.8, 128.5, 83.9, 62.8, 55.4, 52.9, 52.5, 27.9.

IR (ATR): $\tilde{\nu}$ (cm^{-1}) = 1798, 1748, 1716, 1498, 1369, 1301, 1218, 1149.

HRMS (ESI^+) calcd. for $C_{18}H_{20}ClNO_5Na^+$ $[M+Na]^+$: 388.0922, found: 388.0903.

2-(*tert*-Butyl) 1-methyl 3-oxo-4-(*p*-tolyl)-2-azabicyclo[2.1.1]hexane-1,2-dicarboxylate (8e)

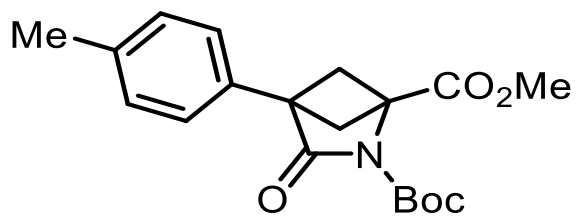

Prepared according to **General Procedure D** using **7e** (365 mg, 1.06 mmol, 1.00 equiv), CH_3CN (10.6 mL), and $[Ir(dF(CF_3)ppy)_2(dtbbpy)](PF_6)$ (11.9 mg, 10.6 μmol, 1.0 mol%). The crude reaction mixture was purified using a Biotage automated flash chromatography system with a gradient elution of ethyl acetate/pentane (0% to 25%), affording **8e** (281 mg, 0.81 mmol, 77%) as a white solid.

M.P.: 140-142 °C

^{1}H NMR (500 MHz, $CDCl_3$) δ 7.17 (d, J = 7.9 Hz, 2H), 7.12 (d, J = 8.3 Hz, 2H), 3.82 (s, 3H), 2.87 (dd, J = 4.7, 2.0 Hz, 2H), 2.77 (dd, J = 4.8, 2.0 Hz, 2H), 2.33 (s, 3H), 1.51 (s, 9H).

^{13}C NMR (126 MHz, $CDCl_3$) δ 174.2, 166.6, 149.4, 137.8, 130.6, 129.2, 126.8, 83.6, 62.7, 55.7, 52.8, 52.4, 27.9, 21.1.

IR (ATR): $\tilde{\nu}$ (cm^{-1}) = 1785, 1751, 1718, 1444, 1369, 1309, 1210, 1151, 1113.

HRMS (ESI^+) calcd. for $C_{19}H_{23}NO_5Na^+$ $[M+Na]^+$: 368.1468, found: 368.1453.

2-(*tert*-Butyl) 1-methyl 4-(4-methoxyphenyl)-3-oxo-2-azabicyclo[2.1.1]hexane-1,2-dicarboxylate (8f)

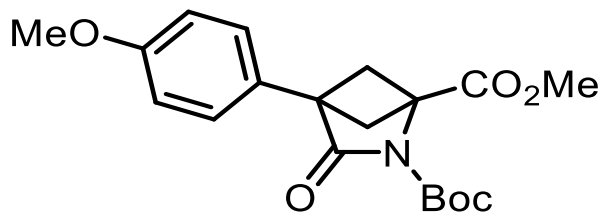

Prepared according to **General Procedure D** using **7f** (330 mg, 0.91 mmol, 1.00 equiv), CH_3CN (9.1 mL), and $[Ir(dF(CF_3)ppy)_2(dtbbpy)](PF_6)$ (10.2 mg, 9.1 μmol, 1 mol%). The crude reaction mixture was purified using a Biotage automated flash chromatography system with a gradient elution of ethyl acetate/pentane (0% to 30%), affording **8f** (252 mg, 0.70 mmol, 76%) as a white solid.

M.P.: 125-127 °C

^{1}H NMR (500 MHz, $CDCl_3$) δ 7.14 (d, *J* = 8.7 Hz, 2H), 6.87 (d, *J* = 8.8 Hz, 2H), 3.80 (s, 3H), 3.75 (s, 3H), 2.84 (dd, *J* = 4.7, 2.0 Hz, 2H), 2.75 (dd, *J* = 4.8, 1.9 Hz, 2H), 1.48 (s, 9H).

^{13}C NMR (126 MHz, $CDCl_3$) δ 174.4, 166.7, 159.3, 149.4, 128.1, 125.7, 114.0, 83.6, 62.7, 55.5, 55.2, 52.9, 52.4, 27.9.

IR (ATR): $\tilde{\nu}$ (cm^{-1}) = 1799, 1786, 1748, 1715, 1616, 1519, 1439, 1369, 1248, 1152.

HRMS (ESI^+) calcd. for $C_{19}H_{23}NO_6Na^+$ $[M+Na]^+$: 384.1418, found: 384.1405.

Methyl 3-oxo-4-phenyl-2-tosyl-2-azabicyclo[2.1.1]hexane-1-carboxylate (8g)

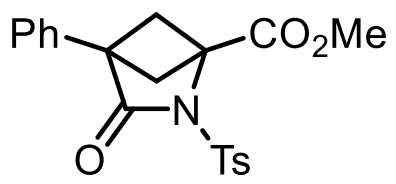

Prepared according to **General Procedure D** using **7g** (1.19 g, 3.09 mmol, 1.00 equiv), CH_3CN (31.0 mL), and [Ir(dF(CF_3)ppy)$_2$(dtbbpy)](PF_6) (34.8 mg, 31.0 μmol, 1 mol%). The crude reaction mixture was purified using a Biotage automated flash chromatography system with a gradient elution of ethyl acetate/pentane (0% to 40%), affording **8g** (1.05 g, 2.73 mmol, 88%) as a white solid.

M.P.: 138-140 °C

^{1}H NMR (500 MHz, $CDCl_3$) δ 7.92 (d, *J* = 8.3 Hz, 2H), 7.39 – 7.28 (m, 5H), 7.13 (d, *J* = 8.3 Hz, 2H), 3.92 (s, 3H), 2.81-2.73 (m, 4H), 2.44 (s, 3H).

^{13}C NMR (126 MHz, $CDCl_3$) δ 174.4, 166.0, 145.3, 136.2, 132.9, 129.9, 128.7, 128.4, 128.0, 126.9, 66.6, 55.9, 53.1, 52.9, 21.8.

IR (ATR): $\tilde{\nu}$ (cm^{-1}) = 1782, 1747, 1597, 1439, 1363, 1225, 1201, 1140, 1089.

HRMS (ESI^+) calcd. for $C_{20}H_{19}NO_5SNa^+$ $[M+Na]^+$: 408.0876, found: 408.0860.

2-Benzyl-1-methyl-4-phenyl-2-azabicyclo[2.1.1]hexan-3-one (8h)

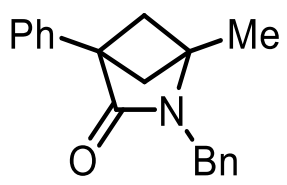

Prepared according to **General Procedure D** using **7h** (1.26 g, 4.55 mmol, 1.00 equiv), CH_3CN (45.5 mL), and [Ir(dF(CF_3)ppy)$_2$(dtbbpy)](PF_6) (51.1 mg, 0.046 mmol, 1 mol%). The crude reaction mixture was purified using a Biotage automated flash chromatography system with a gradient elution of ethyl acetate/pentane (0% to 35%), affording **8h** (845 mg, 3.05 mmol, 67%) as a colourless oil.

^{1}H NMR (500 MHz, $CDCl_3$) δ 7.42 – 7.26 (m, 10H), 4.58 (s, 2H), 2.76 (dd, *J* = 4.0, 1.9 Hz, 2H), 2.51 (dd, *J* = 3.9, 2.0 Hz, 2H), 1.37 (s, 3H).

^{13}C NMR (126 MHz, $CDCl_3$) δ 181.0, 139.4, 136.6, 128.6, 128.4, 127.7, 127.23, 127.18, 62.1, 59.3, 55.4, 43.8, 16.5.

IR (ATR): $\tilde{\nu}$ (cm^{-1}) = 1713, 1604, 1503, 1454, 1399, 1334, 1276, 1164, 1075.

HRMS (ESI^+) calcd. for $C_{19}H_{20}NO^+$ $[M+H]^+$: 278.1539, found: 278.1533.

2.4.3 Synthesis and Characterisation of Products (5, 6 and 9)

***tert*-Butyl 4-methyl-3-oxo-1-(4-(trifluoromethyl)phenyl)-2-azabicyclo[2.2.0]hexane-2-carboxylate (6a)**

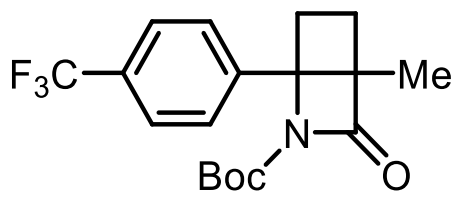

Prepared according to **General Procedure D** using **2a** (490 mg, 1.38 mmol, 1.00 equiv), CH_3CN (13.8 mL), and [Ir(dF(CF_3)ppy)$_2$(dtbbpy)](PF_6) (15.5 mg, 13.8 μmol, 1.0 mol%). The crude reaction mixture was purified using a Biotage automated flash chromatography system with a gradient elution of ethyl acetate/pentane (0% to 30%), affording **6a** (132 mg, 0.37 mmol, 27%) as a white solid and **5a** (222 mg, 0.63 mmol, 45 %) as a white solid.[A]

Notes: (**A**) The crude ^{1}H NMR spectrum indicated a 1:1.6 molar ratio of **6a**/**5a**.

M.P.: 94-96 °C

^{1}H NMR (500 MHz, $CDCl_3$) δ 7.65 (d, J = 8.2 Hz, 2H), 7.41 (d, J = 8.2 Hz, 2H), 3.11 – 2.96 (m, 1H), 2.57 – 2.48 (m, 2H), 2.22 – 2.12 (m, 1H), 1.41 (s, 9H), 1.06 (s, 3H).

^{13}C NMR (126 MHz, $CDCl_3$) δ 170.6, 147.7, 139.7, 130.1 (q, J = 32.7 Hz), 126.7, 125.6 (q, J = 3.7 Hz), 124.1 (q, J = 272.1 Hz), 83.7, 65.9, 61.9, 28.0, 26.3, 25.7, 13.1.

^{19}F NMR (471 MHz, $CDCl_3$) δ –62.63.

IR (ATR): $\tilde{\nu}$ (cm^{-1}) = 1800, 1720, 1620, 1371, 1320, 1278, 1151, 1119, 1067.

HRMS (ESI$^+$) calcd. for $C_{18}H_{20}F_3NO_3Na^+$ [M+Na]$^+$: 378.1287, found: 378.1288.

***tert*-Butyl 4-methyl-3-oxo-1-(4-(trifluoromethyl)phenyl)-2-azabicyclo[2.1.1]hexane-2-carboxylate (5a)**

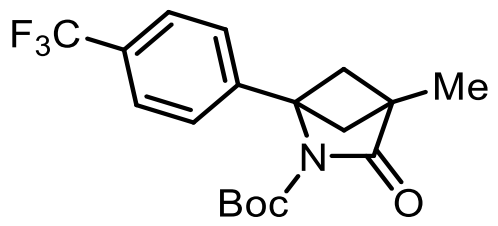

M.P.: 121-123 °C

^{1}H NMR (500 MHz, $CDCl_3$) δ 7.61 (d, J = 8.1 Hz, 2H), 7.37 (d, J = 8.1 Hz, 2H), 2.73 (dd, J = 4.7, 2.0 Hz, 2H), 2.41 (dd, J = 4.7, 2.0 Hz, 2H), 1.35 (s, 3H), 1.13 (s, 9H).

^{13}C NMR (126 MHz, $CDCl_3$) δ 178.6, 149.5, 141.4, 130.2 (q, J = 32.4 Hz), 127.0, 125.4 (q, J = 3.8 Hz), 124.1 (q, J = 272.1 Hz), 82.5, 67.7, 54.8, 49.9, 27.6, 13.2.

^{19}F NMR (471 MHz, $CDCl_3$) δ –62.62.

IR (ATR): $\tilde{\nu}$ (cm^{-1}) = 1790, 1720, 1623, 1368, 1324, 1266, 1159, 1125, 1068.

HRMS (ESI$^+$) calcd. for $C_{18}H_{20}F_3NO_3Na^+$ [M+Na]$^+$: 378.1287, found: 378.1289.

Benzyl 4-methyl-3-oxo-1-(4-(trifluoromethyl)phenyl)-2-azabicyclo[2.2.0]hexane-2-carboxylate (6b)

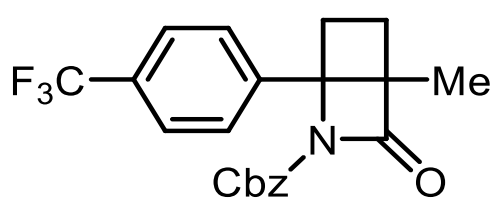

Prepared according to **General Procedure D** using **2b** (530 mg, 1.36 mmol, 1.00 equiv), CH_3CN (13.6 mL), and [Ir(dF(CF_3)ppy)$_2$(dtbbpy)](PF_6) (15.3 mg, 13.6 µmol, 1.0 mol%). The crude reaction mixture was purified using a Biotage automated flash chromatography system with a gradient elution of Et_2O/CH_2Cl_2 (2% to 5%), affording **6b** (176 mg, 0.45 mmol, 33%) as a white solid and **5b** (178 mg, 0.45 mmol, 33 %) as a white solid.[**A**]

Notes: (**A**) The crude ^{1}H NMR spectrum indicated a 1:1 molar ratio of **6b**/**5b**.

M.P.: 142-144 °C

^{1}H NMR (500 MHz, $CDCl_3$) δ 7.64 (d, *J* = 8.2 Hz, 2H), 7.41 (d, *J* = 8.1 Hz, 2H), 7.32 – 7.29 (m, 3H), 7.25 – 7.21 (m, 2H), 5.26 (d, *J* = 12.4 Hz, 1H), 5.19 (d, *J* = 12.4 Hz, 1H), 3.10 – 3.00 (m, 1H), 2.61 – 2.50 (m, 2H), 2.25 – 2.15 (m, 1H), 1.10 (s, 3H).

^{13}C NMR (126 MHz, $CDCl_3$) δ 170.1, 148.8, 139.2, 134.9, 130.2 (q, *J* = 32.6 Hz), 128.6, 128.5, 128.1, 126.8, 125.7 (q, *J* = 3.8 Hz), 124.0 (q, *J* = 272.1 Hz), 68.1, 66.0, 62.4, 26.3, 25.6, 13.0.

^{19}F NMR (471 MHz, $CDCl_3$) δ –62.58.

IR (ATR): $\tilde{\nu}$ (cm^{-1}) = 1812, 1715, 1620, 1393, 1320, 1205, 1169, 1116, 1067.

HRMS (ESI$^+$) calcd. for $C_{21}H_{19}F_3NO_3^+$ [M+H]$^+$: 390.1312, found: 390.1317.

Benzyl 4-methyl-3-oxo-1-(4-(trifluoromethyl)phenyl)-2-azabicyclo[2.1.1]hexane-2-carboxylate (5b)

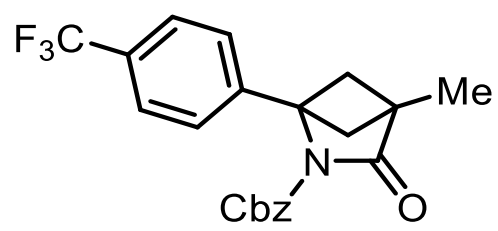

M.P.: 124-126 °C

^{1}H NMR (500 MHz, $CDCl_3$) δ 7.56 (d, *J* = 8.1 Hz, 2H), 7.36 (d, *J* = 7.6 Hz, 2H), 7.29 – 7.26 (m, 3H), 7.15 – 7.11 (m, 2H), 5.01 (s, 2H), 2.77 (dd, *J* = 4.8, 2.0 Hz, 2H), 2.45 (dd, *J* = 4.7, 2.0 Hz, 2H), 1.39 (s, 3H).

^{13}C NMR (126 MHz, $CDCl_3$) δ 177.8, 151.0, 140.4, 134.9, 130.2 (q, *J* = 32.4 Hz), 128.4, 128.2, 127.9, 127.1, 125.2 (q, *J* = 3.8 Hz), 124.0 (q, *J* = 272.2 Hz), 67.9, 67.7, 54.4, 49.9, 13.1.

^{19}F NMR (471 MHz, $CDCl_3$) δ –62.44.

IR (ATR): $\tilde{\nu}$ (cm^{-1}) = 1797, 1720, 1623, 1412, 1382, 1324, 1276, 1265, 1169, 1123.

HRMS (ESI$^+$) calcd. for $C_{21}H_{19}F_3NO_3^+$ [M+H]$^+$: 390.1312, found: 390.1313.

Phenyl 4-methyl-3-oxo-1-(4-(trifluoromethyl)phenyl)-2-azabicyclo[2.2.0]hexane-2-carboxylate (6c)

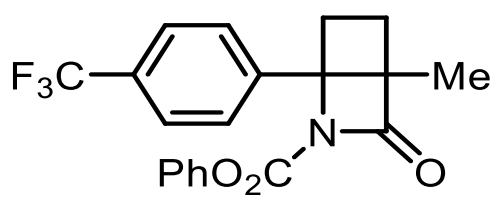

Prepared according to **General Procedure D** using **2c** (490 mg, 1.31 mmol, 1.00 equiv), CH_3CN (13.1 mL), and [Ir(dF(CF_3)ppy)$_2$(dtbbpy)](PF_6) (14.7 mg, 13.1 µmol, 1.0 mol%). The crude reaction mixture was purified using a Biotage automated flash chromatography system with a gradient elution of Et_2O/CH_2Cl_2 (2% to 8%), affording **6c** (220 mg, 0.59 mmol, 45%) as a white solid and **5c** (90 mg, 0.24 mmol, 18 %) as a white solid.[**A**]

Notes: (**A**) The crude ^{1}H NMR spectrum indicated a 2:1 molar ratio of **6c**/**5c**.

M.P.: 134-136 °C

^{1}H NMR (500 MHz, $CDCl_3$) δ 7.70 (d, J = 8.2 Hz, 2H), 7.52 (d, J = 8.1 Hz, 2H), 7.37 – 7.33 (m, 2H), 7.24 – 7.20 (m, 1H), 7.16 – 7.13 (m, 2H), 3.12 (ddd, J = 13.6, 11.5, 6.3 Hz, 1H), 2.72 (ddd, J = 13.8, 8.7, 5.6 Hz, 1H), 2.63 (ddd, J = 13.0, 8.7, 6.2 Hz, 1H), 2.27 (ddd, J = 13.1, 11.5, 5.6 Hz, 1H), 1.15 (s, 3H).

^{13}C NMR (126 MHz, $CDCl_3$) δ 170.0, 149.9, 147.1, 139.0, 130.3 (q, J = 32.6 Hz), 129.5, 126.8, 126.4, 125.8 (q, J = 3.8 Hz), 124.0 (q, J = 272.1 Hz), 121.2, 66.3, 62.6, 26.4, 25.8, 13.1.

^{19}F NMR (471 MHz, $CDCl_3$) δ –62.58.

IR (ATR): $\tilde{\nu}$ (cm^{-1}) = 1810, 1736, 1620, 1494, 1411, 1321, 1275, 1190, 1164, 1067.

HRMS (ESI$^+$) calcd. for $C_{20}H_{16}F_3NO_3Na^+$ [M+Na]$^+$: 398.0974, found: 398.0970.

Phenyl 4-methyl-3-oxo-1-(4-(trifluoromethyl)phenyl)-2-azabicyclo[2.1.1]hexane-2-carboxylate (5c)

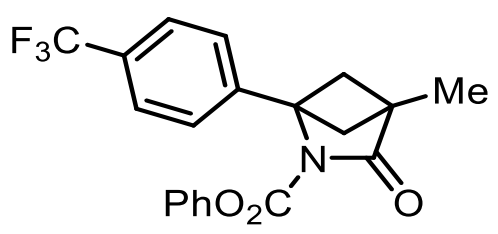

M.P.: 154-156 °C

^{1}H NMR (500 MHz, $CDCl_3$) δ 7.63 (d, *J* = 8.1 Hz, 2H), 7.46 (d, *J* = 7.8 Hz, 2H), 7.29 – 7.25 (m, 2H), 7.17 – 7.13 (m, 1H), 6.99 – 6.95 (m, 2H), 2.88 (dd, *J* = 4.8, 2.1 Hz, 2H), 2.52 (dd, *J* = 4.8, 2.0 Hz, 2H), 1.44 (s, 3H).

^{13}C NMR (126 MHz, $CDCl_3$) δ 177.3, 150.2, 149.6, 140.0, 130.5 (q, *J* = 32.5 Hz), 129.4, 127.3, 126.0, 125.4 (q, *J* = 3.8 Hz), 124.0 (d, *J* = 272.1 Hz), 121.3, 68.2, 54.3, 50.1, 13.2.

^{19}F NMR (471 MHz, $CDCl_3$) δ –62.61.

IR (ATR): $\tilde{\nu}$ (cm^{-1}) = 1793, 1733, 1623, 1494, 1413, 1324, 1275, 1199, 1166, 1067.

HRMS (ESI^+) calcd. for $C_{20}H_{16}F_3NO_3Na^+$ $[M+Na]^+$: 398.0974, found: 398.0971.

2-acetyl-4-methyl-1-(4-(trifluoromethyl)phenyl)-2-azabicyclo[2.2.0]hexan-3-one (6d)

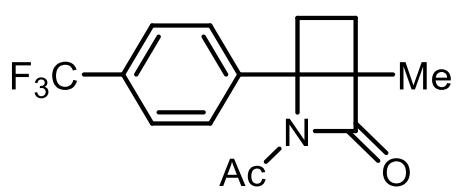

Prepared according to **General Procedure D** using **2d** (280 mg, 0.94 mmol, 1.00 equiv), CH_3CN (9.4 mL), and $[Ir(dF(CF_3)ppy)_2(dtbbpy)](PF_6)$ (10.5 mg, 9.4 μmol, 1.0 mol%). The crude reaction mixture was purified using a Biotage automated flash chromatography system with a gradient elution of Et_2O/pentane (0% to 60%), affording **6d** (175 mg, 0.59 mmol, 63%) as a white solid and **5d** (49 mg, 0.16 mmol, 18%) as a white solid.[A,B]

Notes: (**A**) The crude ^{1}H NMR spectrum indicated a 3.4:1 molar ratio of **6d**/**5d**.

(**B**) When HFIP was used as the solvent, **6d** was isolated in 78% yield. The crude ^{1}H NMR spectrum indicated a 12:1 molar ratio of **6d**:**5d**.

M.P.: 70-72 °C

^{1}H NMR (500 MHz, $CDCl_3$) δ 7.63 (d, *J* = 8.2 Hz, 2H), 7.37 (d, *J* = 8.1 Hz, 2H), 3.07 – 2.96 (m, 1H), 2.60 – 2.47 (m, 5H), 2.29 – 2.20 (m, 1H), 1.09 (s, 3H).

^{13}C NMR (126 MHz, $CDCl_3$) δ 170.9, 168.1, 139.1, 130.1 (q, *J* = 32.6 Hz), 126.6, 125.7 (q, *J* = 3.8 Hz), 124.0 (q, *J* = 272.1 Hz), 65.6, 61.5, 26.44, 26.37, 24.4, 13.0.

^{19}F NMR (471 MHz, $CDCl_3$) δ –62.65.

IR (ATR): $\tilde{\nu}$ (cm^{-1}) = 1777, 1703, 1619, 1411, 1374, 1320, 1275, 1267, 1163, 1066.

HRMS (ESI^+) calcd. for $C_{15}H_{14}F_3NO_2Na^+$ $[M+Na]^+$: 320.0869, found: 320.0865.

2-Acetyl-4-methyl-1-(4-(trifluoromethyl)phenyl)-2-azabicyclo[2.1.1]hexan-3-one (5d)

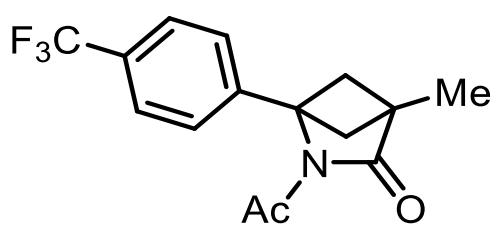

M.P.: 60-62 °C

^{1}H NMR (500 MHz, $CDCl_3$) δ 7.61 (d, J = 8.0 Hz, 2H), 7.37 (d, J = 7.8 Hz, 2H), 2.74 (dd, J = 4.6, 2.0 Hz, 2H), 2.44 (dd, J = 4.7, 2.1 Hz, 2H), 2.40 (s, 3H), 1.39 (s, 3H).

^{13}C NMR (126 MHz, $CDCl_3$) δ 179.3, 169.6, 141.0, 130.2 (q, J = 32.5 Hz), 126.9, 125.3 (q, J = 3.8 Hz), 124.2 (q, J = 272.1 Hz), 67.9, 54.6, 49.9, 25.8, 13.3.

^{19}F NMR (471 MHz, $CDCl_3$) δ –62.58.

IR (ATR): $\tilde{\nu}$ (cm^{-1}) = 1763, 1696, 1372, 1320, 1287, 1161, 1119, 1067.

HRMS (ESI^+) calcd. for $C_{15}H_{14}F_3NO_2Na^+$ $[M+Na]^+$: 320.0869, found: 320.0863.

2,2,2-Trichloroethyl 4-methyl-3-oxo-1-(4-(trifluoromethyl)phenyl)-2-azabicyclo[2.2.0]hexane-2-carboxylate (6e)

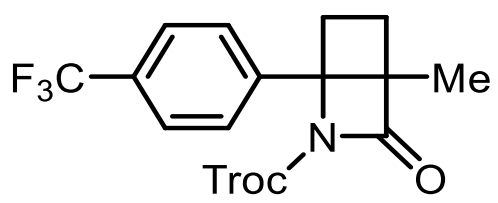

Prepared according to **General Procedure D** using **2e** (580 mg, 1.35 mmol, 1.00 equiv), CH_3CN (13.5 mL), and [Ir(dF(CF_3)ppy)$_2$(dtbbpy)](PF_6) (15.2 mg, 13.5 µmol, 1.0 mol%). The crude reaction mixture was purified using a Biotage automated flash chromatography system with a gradient elution of ethyl acetate/CH_2Cl_2 (2% to 5%), affording **6e** (375 mg, 0.87 mmol, 65 %) as a white solid.[**A**]

Notes: (**A**) The crude ^{1}H NMR spectrum indicated a 4:1 molar ratio of **6e**/**5e**.

M.P.: 117-119 °C

^{1}H NMR (500 MHz, $CDCl_3$) δ 7.65 (d, J = 8.2 Hz, 2H), 7.45 (d, J = 8.1 Hz, 2H), 4.77 (d, J = 11.9 Hz, 1H), 4.72 (d, J = 12.0 Hz, 1H), 3.12 (ddd, J = 13.5, 11.4, 6.3 Hz, 1H), 2.64 (ddd, J = 13.7, 8.7, 5.5 Hz, 1H), 2.56 (ddd, J = 13.1, 8.7, 6.2 Hz, 1H), 2.22 (ddd, J = 13.2, 11.5, 5.5 Hz, 1H), 1.13 (s, 3H).

^{13}C NMR (126 MHz, $CDCl_3$) δ 170.1, 147.0, 138.7, 130.4 (q, J = 32.6 Hz), 126.9, 125.8 (q, J = 3.8 Hz), 123.9 (q, J = 272.4 Hz), 94.2, 74.8, 66.6, 63.1, 26.1, 25.5, 13.0.

^{19}F NMR (471 MHz, $CDCl_3$) δ –62.66.

IR (ATR): $\tilde{\nu}$ (cm^{-1}) = 1814, 1733, 1620, 1379, 1316, 1276, 1166, 1118, 1067, 1015.

HRMS (ESI^+) calcd. for $C_{16}H_{14}Cl_3F_3NO_3^+$ $[M+H]^+$: 429.9986, found: 429.9986.

2-acetyl-4-methyl-1-phenyl-2-azabicyclo[2.2.0]hexan-3-one (6f)

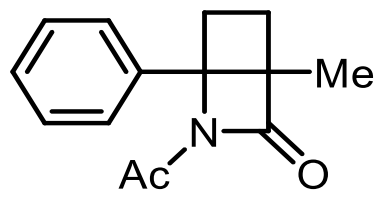

Prepared according to **General Procedure D** using 2f (540 mg, 2.36 mmol, 1.00 equiv), CH_3CN (23.6 mL), and $[Ir(dF(CF_3)ppy)_2(dtbbpy)](PF_6)$ (26.5 mg, 23.6 μmol, 1.0 mol%). The crude reaction mixture was purified using a Biotage automated flash chromatography system with a gradient elution of Et_2O/CH_2Cl_2 (2% to 6%), affording **6f** (411 mg, 1.79 mmol, 76%) as a colourless oil and **5f** (26 mg, 0.11 mmol, 5 %) as a white solid.**[A,B]**

Notes: (**A**) The crude ^1H NMR spectrum indicated a 10:1 molar ratio of **6f**/**5f**.

(**B**) When HFIP was used as the solvent, **6f** was isolated in 79% yield. The crude ^1H NMR spectrum indicated a 30:1 molar ratio of **6f**:**5f**.

^{1}H NMR (500 MHz, $CDCl_3$) δ 7.41 – 7.34 (m, 2H), 7.31 – 7.27 (m, 1H), 7.27 – 7.24 (m, 2H), 3.08 – 2.99 (m, 1H), 2.58 – 2.46 (m, 5H), 2.24 – 2.17 (m, 1H), 1.09 (s, 3H).

^{13}C NMR (126 MHz, $CDCl_3$) δ 171.6, 168.1, 134.9, 128.7, 127.9, 126.1, 66.2, 61.1, 26.5, 26.4, 24.5, 13.0.

IR (ATR): $\tilde{\nu}$ (cm^{-1}) = 1771, 1701, 1497, 1371, 1309, 1270, 1193, 1075, 970.

HRMS (ESI^+) calcd. for $C_{14}H_{15}NO_2Na^+$ $[M+Na]^+$: 252.0995, found: 252.0986.

2-Acetyl-4-methyl-1-phenyl-2-azabicyclo[2.1.1]hexan-3-one (5f)

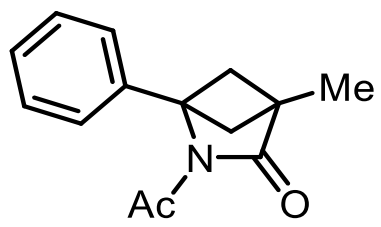

M.P.: 100-102 °C

^{1}H NMR (500 MHz, $CDCl_3$) δ 7.39 – 7.31 (m, 3H), 7.29 – 7.26 (m, 2H), 2.73 (dd, J = 4.7, 2.1 Hz, 2H), 2.42 (dd, J = 4.6, 2.1 Hz, 2H), 2.38 (s, 3H), 1.38 (s, 3H).

^{13}C NMR (126 MHz, $CDCl_3$) δ 179.9, 169.4, 137.1, 128.3, 128.1, 126.5, 68.7, 54.6, 49.7, 26.0, 13.3.

IR (ATR): $\tilde{\nu}$ (cm^{-1}) = 1766, 1681, 1449, 1374, 1359, 1297, 1163.

HRMS (ESI^+) calcd. for $C_{14}H_{16}NO_2^+$ $[M+H]^+$: 230.1176, found: 230.1169.

2-Acetyl-1-(4-methoxyphenyl)-4-methyl-2-azabicyclo[2.2.0]hexan-3-one (6g)

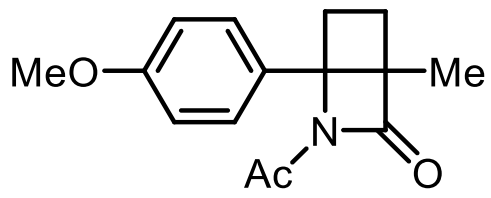

Gram scale: Prepared according to **General Procedure D** using **2g** (3.31 g, 12.8 mmol, 1.00 equiv), CH_3CN (128 mL), and [Ir(dF(CF_3)ppy)$_2$(dtbbpy)](PF_6) (71.7 mg, 63.9 µmol, 0.50 mol%). The crude reaction mixture was purified using a Biotage automated flash chromatography system with a gradient elution of Et_2O/pentane (0% to 60%), affording **6g** (2.71 g, 10.5 mmol, 82%) as a colourless oil.[A]

Notes: (**A**) The crude ^{1}H NMR spectrum indicated a 24:1 molar ratio of **6g**/**5g**.

^{1}H NMR (500 MHz, $CDCl_3$) δ 7.17 (d, J = 8.8 Hz, 2H), 6.89 (d, J = 8.8 Hz, 2H), 3.76 (s, 3H), 2.97 (ddd, J = 12.9, 11.4, 6.0 Hz, 1H), 2.56 – 2.42 (m, 5H), 2.20 – 2.11 (m, 1H), 1.08 (s, 3H).

^{13}C NMR (126 MHz, $CDCl_3$) δ 171.7, 168.0, 159.2, 127.3, 127.0, 114.0, 65.9, 60.8, 55.2, 26.4, 26.3, 24.4, 12.9.

IR (ATR): $\tilde{\nu}$ (cm^{-1}) = 1771, 1700, 1614, 1515, 1445, 1371, 1309, 1272, 1179, 1104, 1031.

HRMS (ESI$^+$) calcd. for $C_{15}H_{17}NO_3Na^+$ $[M+Na]^+$: 282.1101, found: 282.1093.

2-Acetyl-1-(2,3-dihydrobenzo[*b*][1,4]dioxin-6-yl)-4-methyl-2-azabicyclo[2.2.0]hexan-3-one (6h)

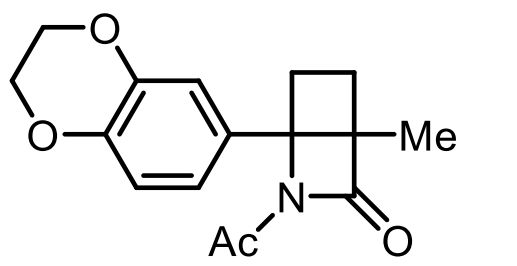

Prepared according to **General Procedure D** using **2h** (430 mg, 1.50 mmol, 1.00 equiv), CH_3CN (15.0 mL), and [Ir(dF(CF_3)ppy)$_2$(dtbbpy)](PF_6) (16.8 mg, 15.0 µmol, 1.0 mol%). The crude reaction mixture was purified using a Biotage automated flash chromatography system with a gradient elution of (Et_2O/ethyl acetate 3:1)/pentane (10% to 50%), affording **6h** (225 mg, 0.78 mmol, 52%) as a colourless oil.[A]

Notes: (**A**) The crude ^{1}H NMR spectrum indicated a 15:1 molar ratio of **6h**/**5h**.

^{1}H NMR (500 MHz, $CDCl_3$) δ 6.83 (d, J = 8.4 Hz, 1H), 6.73 (d, J = 2.3 Hz, 1H), 6.70 (dd, J = 8.4, 2.2 Hz, 1H), 4.20 (s, 4H), 2.97 – 2.89 (m, 1H), 2.51 – 2.39 (m, 5H), 2.17 – 2.10 (m, 1H), 1.07 (s, 3H).

^{13}C NMR (126 MHz, $CDCl_3$) δ 171.6, 168.0, 143.6, 143.3, 128.1, 119.1, 117.4, 115.2, 65.7, 64.31, 64.30, 60.9, 26.23, 26.21, 24.4, 12.9.

IR (ATR): $\tilde{\nu}$ (cm^{-1}) = 1770, 1701, 1588, 1510, 1425, 1372, 1310, 1276, 1261, 1194, 1124.

HRMS (ESI$^+$) calcd. for $C_{16}H_{17}NO_4Na^+$ $[M+Na]^+$: 310.1050, found: 310.1039.

2-acetyl-1-(4-(*tert*-butyl)phenyl)-4-methyl-2-azabicyclo[2.2.0]hexan-3-one (6i)

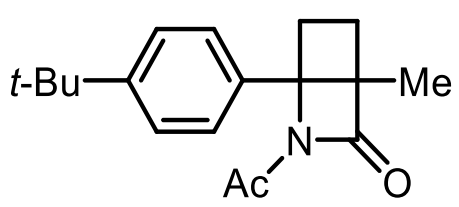

Prepared according to **General Procedure D** using **2i** (540 mg, 1.90 mmol, 1.00 equiv), CH_3CN (19.0 mL), and [Ir(dF(CF_3)ppy)$_2$(dtbbpy)](PF_6) (21.3 mg, 19.0 µmol, 1.0 mol%). The crude reaction mixture was purified using a Biotage automated flash chromatography system with a gradient elution of Et_2O/pentane (10% to 50%), affording **6i** (350 mg, 1.23 mmol, 65%) as a colourless oil.[A]

Notes: (**A**) The crude ^{1}H NMR spectrum indicated a 13:1 molar ratio of **6i**/**5i**.

^{1}H NMR (500 MHz, $CDCl_3$) δ 7.38 (d, J = 8.5 Hz, 2H), 7.18 (d, J = 8.5 Hz, 2H), 3.01 (ddd, J = 12.6, 11.4, 5.8 Hz, 1H), 2.58 – 2.44 (m, 5H), 2.24 – 2.15 (m, 1H), 1.30 (s, 9H), 1.10 (s, 3H).

^{13}C NMR (126 MHz, $CDCl_3$) δ 171.7, 168.1, 150.7, 131.8, 125.8, 125.6, 66.1, 61.0, 34.6, 31.3, 26.48, 26.46, 24.5, 13.1.

IR (ATR): $\tilde{\nu}$ (cm^{-1}) = 1774, 1703, 1371, 1311, 1277, 1191, 970.

HRMS (ESI$^+$) calcd. for $C_{18}H_{23}NO_2Na^+$ [M+Na]$^+$: 308.1621, found: 308.1629.

2-Acetyl-4-methyl-1-(*p*-tolyl)-2-azabicyclo[2.2.0]hexan-3-one (6j)

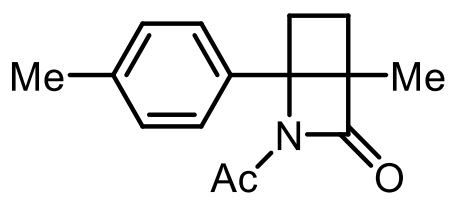

Prepared according to **General Procedure D** using **2j** (302 mg, 1.24 mmol, 1.00 equiv), CH_3CN (12.4 mL), and [Ir(dF(CF_3)ppy)$_2$(dtbbpy)](PF_6) (13.9 mg, 12.4 µmol, 1.0 mol%). The crude reaction mixture was purified using a Biotage automated flash chromatography system with a gradient elution of Et_2O/pentane (0% to 60%), affording **6j** (206 mg, 0.85 mmol, 68%) as a colourless oil.[A]

Notes: (**A**) The crude ^{1}H NMR spectrum indicated a 15:1 molar ratio of **6j**:**5j**.

^{1}H NMR (500 MHz, $CDCl_3$) δ 7.19 (d, J = 8.0 Hz, 2H), 7.14 (d, J = 8.2 Hz, 2H), 3.06 – 2.98 (m, 1H), 2.58 – 2.44 (m, 5H), 2.34 (s, 3H), 2.24 – 2.13 (m, 1H), 1.10 (s, 3H).

^{13}C NMR (126 MHz, $CDCl_3$) δ 171.8, 168.1, 137.8, 132.0, 129.5, 126.1, 66.2, 61.0, 26.5, 26.4, 24.6, 21.2, 13.1.

IR (ATR): $\tilde{\nu}$ (cm^{-1}) = 1772, 1702, 1518, 1447, 1371, 1310, 1270, 1186, 1105, 1037.

HRMS (ESI$^+$) calcd. for $C_{15}H_{17}NO_2Na^+$ [M+Na]$^+$: 266.1152, found: 266.1140.

2-Acetyl-4-methyl-1-(*m*-tolyl)-2-azabicyclo[2.2.0]hexan-3-one (6k)

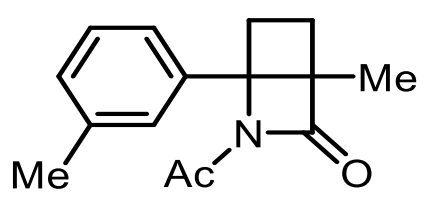

Prepared according to **General Procedure D** using **2k** (365 mg, 1.50 mmol, 1.00 equiv), CH_3CN (15.0 mL), and [Ir(dF(CF_3)ppy)$_2$(dtbbpy)](PF_6) (16.9 mg, 15.1 µmol, 1.0 mol%). The crude reaction

mixture was purified using a Biotage automated flash chromatography system with a gradient elution of Et_2O/pentane (0% to 60%), affording **6k** (272 mg, 1.12 mmol, 75%) as a colourless oil.[A]

Notes: (**A**) The crude ^{1}H NMR spectrum indicated a 11:1 molar ratio of **6k**/**5k**.

^{1}H NMR (500 MHz, $CDCl_3$) δ 7.29 – 7.25 (m, 1H), 7.11 (dp, *J* = 7.5, 0.9 Hz, 1H), 7.07 – 7.03 (m, 2H), 3.07 – 2.98 (m, 1H), 2.57 – 2.46 (m, 5H), 2.36 (s, 3H), 2.25 – 2.17 (m, 1H), 1.09 (s, 3H).

^{13}C NMR (126 MHz, $CDCl_3$) δ 171.7, 168.1, 138.3, 134.9, 128.8, 128.6, 126.8, 123.2, 66.2, 61.0, 26.5, 26.3, 24.5, 21.7, 13.1.

IR (ATR): $\tilde{\nu}$ (cm^{-1}) = 1772, 1702, 1609, 1448, 1371, 1310, 1275, 1276, 1193, 1038.

HRMS (ESI$^+$) calcd. for $C_{15}H_{17}NO_2Na^+$ [M+Na]$^+$: 266.1152, found: 266.1142.

2-Acetyl-1-(4-fluorophenyl)-4-methyl-2-azabicyclo[2.2.0]hexan-3-one (6l)

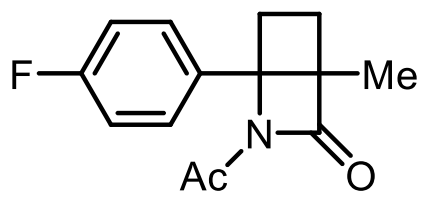

Prepared according to **General Procedure D** using **2l** (305 mg, 1.23 mmol, 1.00 equiv), CH_3CN (12.3 mL), and [Ir(dF(CF_3)ppy)$_2$(dtbbpy)](PF_6) (13.9 mg, 12.4 µmol, 1.0 mol%). The crude reaction mixture was purified using a Biotage automated flash chromatography system with a gradient elution of (Et_2O/ethyl acetate 3:1)/pentane (10% to 40%), affording **6l** (208 mg, 0.84 mmol, 68%) as a colourless oil.[A]

Notes: (**A**) The crude ^{1}H NMR spectrum indicated a 11:1 molar ratio of **6l**/**5l**.

^{1}H NMR (500 MHz, $CDCl_3$) δ 7.27 – 7.22 (m, 2H), 7.11 – 7.05 (m, 2H), 3.00 (ddd, *J* = 13.0, 11.5, 6.0 Hz, 1H), 2.60 – 2.45 (m, 5H), 2.26 – 2.18 (m, 1H), 1.11 (s, 3H).

^{13}C NMR (126 MHz, $CDCl_3$) δ 171.3, 168.1, 162.3 (d, *J* = 247.2 Hz), 130.9 (d, *J* = 3.2 Hz), 127.9 (d, *J* = 8.3 Hz), 115.6 (d, *J* = 21.8 Hz), 65.6, 61.0, 26.5, 26.4, 24.4, 12.9.

^{19}F NMR (471 MHz, $CDCl_3$) δ –114.23.

IR (ATR): $\tilde{\nu}$ (cm^{-1}) = 1773, 1701, 1603, 1513, 1448, 1372, 1311, 1269, 1230, 1161, 1097.

HRMS (ESI$^+$) calcd. for $C_{14}H_{14}FNO_2Na^+$ [M+Na]$^+$: 270.0901, found: 270.0893.

1-([1,1'-biphenyl]-4-yl)-2-acetyl-4-methyl-2-azabicyclo[2.2.0]hexan-3-one (6m)

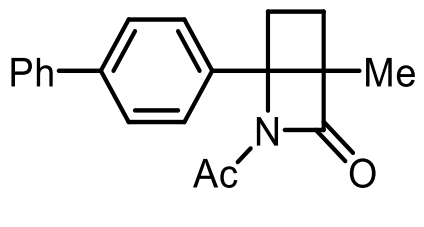

Prepared according to **General Procedure D** using **2m** (305 mg, 1.00 mmol, 1.00 equiv), CH_3CN (10.0 mL), and [Ir(dF(CF_3)ppy)$_2$(dtbbpy)](PF_6) (11.2 mg, 10.0 μmol, 1.0 mol%). The crude reaction mixture was purified using a Biotage automated flash chromatography system with a gradient elution of (Et_2O/ethyl acetate 3:1)/pentane (10% to 40%), affording **6m** (194 mg, 0.64 mmol, 64%) as a white solid.[A]

Notes: (**A**) The crude ^{1}H NMR spectrum indicated a 7:1 molar ratio of **6m**/**5m**.

M.P.: 77-79 °C

^{1}H NMR (500 MHz, $CDCl_3$) δ 7.62 (d, J = 8.4 Hz, 2H), 7.61 – 7.58 (m, 2H), 7.45 (t, J = 7.6 Hz, 2H), 7.38 – 7.33 (m, 3H), 3.08 (ddd, J = 12.8, 11.5, 5.9 Hz, 1H), 2.63 – 2.50 (m, 5H), 2.28 – 2.21 (m, 1H), 1.16 (s, 3H).

^{13}C NMR (126 MHz, $CDCl_3$) δ 171.6, 168.2, 140.8, 140.5, 133.9, 128.9, 127.6, 127.4, 127.2, 126.6, 66.1, 61.2, 26.49, 26.47, 24.5, 13.1.

IR (ATR): $\tilde{\nu}$ (cm^{-1}) = 1773, 1702, 1487, 1371, 1311, 1276, 1191, 1104, 970.

HRMS (ESI$^+$) calcd. for $C_{20}H_{19}NO_2Na^+$ [M+Na]$^+$: 328.1308, found: 328.1299.

2-acetyl-1-(4-chlorophenyl)-4-methyl-2-azabicyclo[2.2.0]hexan-3-one (6n)

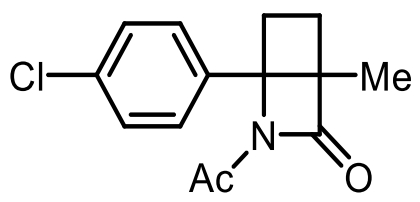

Prepared according to **General Procedure D** using **2n** (380 mg, 1.44 mmol, 1.00 equiv), CH_3CN (14.4 mL), and [Ir(dF(CF_3)ppy)$_2$(dtbbpy)](PF_6) (16.2 mg, 14.4 μmol, 1.0 mol%). The crude reaction mixture was purified using a Biotage automated flash chromatography system with a gradient elution of Et_2O/pentane (10% to 60%), affording **6n** (294 mg, 1.12 mmol, 77%) as a colourless oil.[A]

Notes: (**A**) The crude ^{1}H NMR spectrum indicated a 7:1 molar ratio of **6n**/**5n**.

^{1}H NMR (500 MHz, $CDCl_3$) δ 7.32 (d, J = 8.6 Hz, 2H), 7.17 (d, J = 8.6 Hz, 2H), 2.99 – 2.91 (m, 1H), 2.54 – 2.42 (m, 5H), 2.21 – 2.14 (m, 1H), 1.06 (s, 3H).

^{13}C NMR (126 MHz, $CDCl_3$) δ 171.1, 167.9, 133.8, 133.6, 128.8, 127.5, 65.5, 61.1, 26.29, 26.27, 24.3, 12.9.

IR (ATR): $\tilde{\nu}$ (cm^{-1}) = 1773, 1701, 1495, 1371, 1310, 1277, 1264, 1194, 1094.

HRMS (ESI$^+$) calcd. for $C_{14}H_{14}ClNO_2Na^+$ [M+Na]$^+$: 286.0605, found: 286.0601.

Methyl 4-(2-acetyl-4-methyl-3-oxo-2-azabicyclo[2.2.0]hexan-1-yl)benzoate (6o)

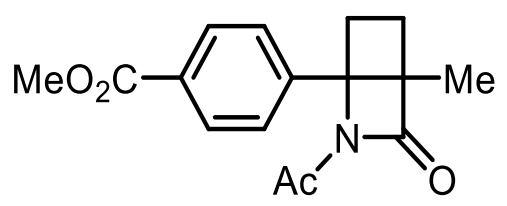

Prepared according to **General Procedure D** using **2o** (550 mg, 1.92 mmol, 1.00 equiv), CH_3CN (19.2 mL), and [Ir(dF(CF_3)ppy)$_2$(dtbbpy)](PF_6) (21.5 mg, 19.2 µmol, 1.0 mol%). The crude reaction mixture was purified using a Biotage automated flash chromatography system with a gradient elution of CH_2Cl_2/ethyl acetate (0% to 10%), affording **6o** (345 mg, 1.20 mmol, 63%) as a colourless oil and **5o** (95 mg, 0.33 mmol, 17 %) as a white solid.[A,B]

Notes: (**A**) The crude ^{1}H NMR spectrum indicated a 3:1 molar ratio of **6o**/**5o**.

(**B**) When HFIP was used as the solvent, **6o** was isolated in 71% yield. The crude ^{1}H NMR spectrum indicated a 8:1 molar ratio of **6o**/**5o**.

^{1}H NMR (500 MHz, $CDCl_3$) δ 7.99 (d, J = 8.5 Hz, 2H), 7.28 (d, J = 8.5 Hz, 2H), 3.84 (s, 3H), 3.04 – 2.93 (m, 1H), 2.54 – 2.42 (m, 5H), 2.24 – 2.15 (m, 1H), 1.02 (s, 3H).

^{13}C NMR (126 MHz, $CDCl_3$) δ 170.8, 167.8, 166.4, 139.9, 129.8, 129.5, 126.1, 65.6, 61.4, 52.1, 26.3, 26.2, 24.3, 12.8.

IR (ATR): $\tilde{\nu}$ (cm^{-1}) = 1775, 1702, 1612, 1435, 1372, 1311, 1275, 1192, 1110.

HRMS (ESI$^+$) calcd. for $C_{16}H_{17}NO_4Na^+$ [M+Na]$^+$: 310.1050, found: 310.1056.

Methyl 4-(2-acetyl-4-methyl-3-oxo-2-azabicyclo[2.1.1]hexan-1-yl)benzoate (5o)

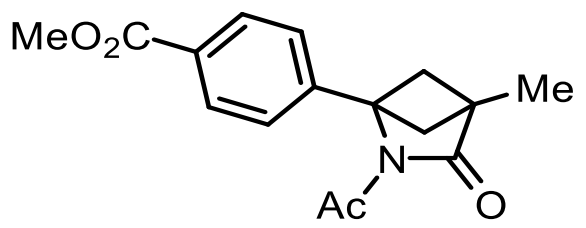

M.P.: 141-143 °C

^{1}H NMR (500 MHz, $CDCl_3$) δ 8.01 (d, J = 8.5 Hz, 2H), 7.31 (d, J = 8.4 Hz, 2H), 3.89 (s, 3H), 2.71 (dd, J = 4.6, 2.1 Hz, 2H), 2.43 (dd, J = 4.6, 2.1 Hz, 2H), 2.37 (s, 3H), 1.36 (s, 3H).

^{13}C NMR (126 MHz, $CDCl_3$) δ 179.3, 169.2, 166.7, 141.9, 129.6, 129.5, 126.4, 67.9, 54.4, 52.1, 49.7, 25.7, 13.1.

IR (ATR): $\tilde{\nu}$ (cm^{-1}) = 1758, 1721, 1692, 1368, 1275, 1262, 1180, 1099.

HRMS (ESI$^+$) calcd. for $C_{16}H_{17}NO_4Na^+$ [M+Na]$^+$: 310.1050, found: 310.1040.

4-(2-Acetyl-4-methyl-3-oxo-2-azabicyclo[2.2.0]hexan-1-yl)benzonitrile (6p)

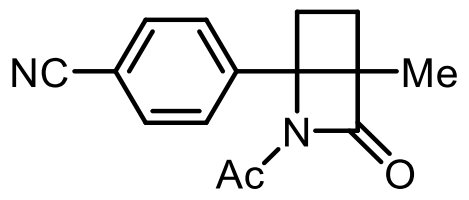

Prepared according to **General Procedure D** using **2p** (508 mg, 2.00 mmol, 1.00 equiv), CH_3CN (20.0 mL), and [Ir(dF(CF_3)ppy)$_2$(dtbbpy)](PF_6) (22.4 mg, 20.0 µmol, 1.0 mol%). The crude reaction mixture was purified using a Biotage automated flash chromatography system with a gradient elution of Et_2O/CH_2Cl_2 (0% to 10%), affording **6p** (272 mg, 1.07 mmol, 54%) as a white solid and **5p** (125 mg, 0.49 mmol, 25 %) as a white solid.[A,B]

Notes: (**A**) The crude ^{1}H NMR spectrum indicated a 2:1 molar ratio of **6p**/**5p**.

(**B**) When HFIP was used as the solvent, **6p** was isolated in 64% yield. The crude ^{1}H NMR spectrum indicated a 6:1 molar ratio of **6p**:**5p**.

M.P.: 79-81 °C

^{1}H NMR (500 MHz, $CDCl_3$) δ 7.64 (d, J = 8.5 Hz, 2H), 7.34 (d, J = 8.7 Hz, 2H), 3.02 – 2.92 (m, 1H), 2.55 – 2.47 (m, 2H), 2.45 (s, 3H), 2.27 – 2.19 (m, 1H), 1.05 (s, 3H).

^{13}C NMR (126 MHz, $CDCl_3$) δ 170.4, 167.9, 140.3, 132.4, 126.9, 118.4, 111.6, 65.3, 61.6, 26.3, 26.2, 24.3, 12.9.

IR (ATR): $\tilde{\nu}$ (cm^{-1}) = 2228, 1775, 1701, 1609, 1372, 1310, 1275, 1266, 1190, 970.

HRMS (ESI$^+$) calcd. for $C_{15}H_{14}N_2O_2Na^+$ [M+Na]$^+$: 277.0947, found: 277.0937.

4-(2-Acetyl-4-methyl-3-oxo-2-azabicyclo[2.1.1]hexan-1-yl)benzonitrile (5p)

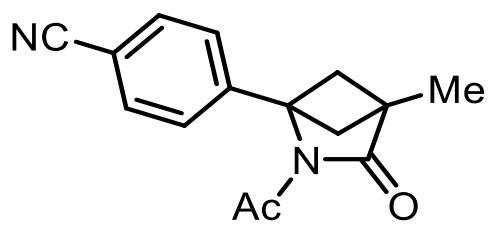

M.P.: 141-143 °C

^{1}H NMR (500 MHz, $CDCl_3$) δ 7.62 (d, J = 8.4 Hz, 2H), 7.35 (d, J = 8.4 Hz, 2H), 2.71 (dd, J = 4.7, 2.1 Hz, 2H), 2.44 (dd, J = 4.6, 2.1 Hz, 2H), 2.37 (s, 3H), 1.37 (s, 3H).

^{13}C NMR (126 MHz, $CDCl_3$) δ 178.9, 169.4, 142.2, 132.0, 127.2, 118.7, 111.7, 67.6, 54.3, 49.8, 25.6, 13.1.

IR (ATR): $\tilde{\nu}$ (cm^{-1}) = 2227, 1762, 1693, 1613, 1371, 1357, 1276, 1261, 1175.

HRMS (ESI$^+$) calcd. for $C_{15}H_{15}N_2O_2^+$ [M+H]$^+$: 255.1128, found: 255.1120.

2-Acetyl-1-(furan-2-yl)-4-methyl-2-azabicyclo[2.2.0]hexan-3-one (6q)

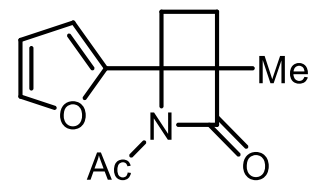

Prepared according to **General Procedure D** using **2q** (257 mg, 1.17 mmol, 1.00 equiv), CH_3CN (11.7 mL), and [Ir(dF(CF_3)ppy)$_2$(dtbbpy)](PF_6) (13.2 mg, 11.8 µmol, 1.0 mol%). The crude reaction mixture was purified using a Biotage automated flash chromatography system with a gradient elution of Et_2O/pentane (0% to 60%), affording **6q** (136 mg, 0.62 mmol, 53%) as a colourless oil.[A]

Notes: (**A**) The crude ^{1}H NMR spectrum indicated a >25:1 molar ratio of **6q**/**5q**.

^{1}H NMR (500 MHz, $CDCl_3$) δ 7.35 (dd, *J* = 1.9, 0.9 Hz, 1H), 6.33 (dd, *J* = 3.3, 1.8 Hz, 1H), 6.28 (dd, *J* = 3.3, 0.9 Hz, 1H), 2.90 (ddd, *J* = 13.2, 11.4, 6.5 Hz, 1H), 2.48 (ddd, *J* = 13.5, 8.6, 5.4 Hz, 1H), 2.43 – 2.36 (m, 4H), 2.15 (ddd, *J* = 13.0, 11.4, 5.4 Hz, 1H), 1.17 (s, 3H).

^{13}C NMR (126 MHz, $CDCl_3$) δ 170.7, 167.8, 148.9, 143.1, 110.7, 108.9, 61.6, 60.8, 25.80, 25.76, 24.2, 13.1.

IR (ATR): $\tilde{\nu}$ (cm^{-1}) = 1775, 1704, 1449, 1372, 1275, 1268, 1190, 970.

HRMS (ESI$^+$) calcd. for $C_{12}H_{13}NO_3Na^+$ [M+Na]$^+$: 242.0788, found: 242.0785.

2-Acetyl-4-methyl-1-(thiophen-2-yl)-2-azabicyclo[2.2.0]hexan-3-one (6r)

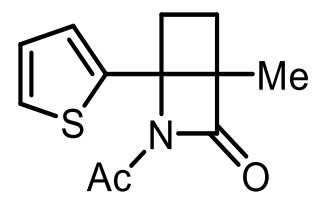

Prepared according to **General Procedure D** using **2r** (310 mg, 1.32 mmol, 1.00 equiv), CH_3CN (13.2 mL), and [Ir(dF(CF_3)ppy)$_2$(dtbbpy)](PF_6) (14.8 mg, 13.2 µmol, 1 mol%). The crude reaction mixture was purified using a Biotage automated flash chromatography system with a gradient elution of Et_2O/pentane (0% to 60%), affording **6r** (244 mg, 1.04 mmol, 79%) as a pale-yellow oil.[A]

Notes: (**A**) The crude ^{1}H NMR spectrum indicated a >25:1 molar ratio of **6r**/**5r**.

^{1}H NMR (500 MHz, $CDCl_3$) δ 7.25 (dd, *J* = 5.1, 1.3 Hz, 1H), 6.98 (dd, *J* = 5.0, 3.5 Hz, 1H), 6.94 (dd, *J* = 3.6, 1.3 Hz, 1H), 2.91 (ddd, *J* = 13.6, 11.4, 6.6 Hz, 1H), 2.63 (ddd, *J* = 13.9, 8.6, 5.6 Hz, 1H), 2.46 – 2.38 (m, 4H), 2.17 (ddd, *J* = 13.1, 11.4, 5.6 Hz, 1H), 1.13 (s, 3H).

^{13}C NMR (126 MHz, $CDCl_3$) δ 170.8, 167.7, 139.0, 127.5, 125.7, 125.5, 63.4, 62.0, 28.3, 25.9, 24.3, 12.7.

IR (ATR): $\tilde{\nu}$ (cm^{-1}) = 1773, 1703, 1442, 1370, 1293, 1266, 1188, 962.

HRMS (ESI$^+$) calcd. for $C_{12}H_{13}NO_2SNa^+$ [M+Na]$^+$: 258.0559, found: 258.0553.

2-Acetyl-4-methyl-1-(thiophen-3-yl)-2-azabicyclo[2.2.0]hexan-3-one (6s)

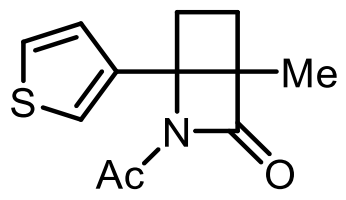

Prepared according to **General Procedure D** using **2s** (300 mg, 1.28 mmol, 1.00 equiv), CH_3CN (12.8 mL), and [Ir(dF(CF_3)ppy)$_2$(dtbbpy)](PF_6) (14.3 mg, 12.7 μmol, 1.0 mol%). The crude reaction mixture was purified using a Biotage automated flash chromatography system with a gradient elution of Et_2O/CH_2Cl_2 (0% to 10%), affording **6s** (210 mg, 0.89 mmol, 70%) as a colourless oil.[A]

Notes: (**A**) The crude ^{1}H NMR spectrum indicated a 20:1 molar ratio of **6s**/**5s**.

^{1}H NMR (500 MHz, $CDCl_3$) δ 7.32 (dd, J = 5.0, 3.0 Hz, 1H), 7.15 (dd, J = 3.0, 1.4 Hz, 1H), 6.94 (dd, J = 5.0, 1.4 Hz, 1H), 2.89 (ddd, J = 13.6, 11.4, 6.6 Hz, 1H), 2.59 (ddd, J = 13.7, 8.7, 5.7 Hz, 1H), 2.48 – 2.42 (m, 4H), 2.19 (ddd, J = 13.0, 10.7, 5.6 Hz, 1H), 1.12 (s, 3H).

^{13}C NMR (126 MHz, $CDCl_3$) δ 171.4, 168.1, 137.0, 126.7, 125.5, 122.6, 63.7, 61.2, 27.8, 26.2, 24.4, 13.1.

IR (ATR): $\tilde{\nu}$ (cm^{-1}) = 1770, 1699, 1371, 1295, 1275, 1266, 1200, 1036.

HRMS (ESI$^+$) calcd. for $C_{12}H_{13}NO_2SNa^+$ [M+Na]$^+$: 258.0559, found: 258.0551.

2-Acetyl-1-(6-methoxypyridin-3-yl)-4-methyl-2-azabicyclo[2.2.0]hexan-3-one (6t)

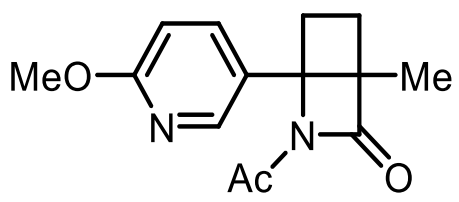

Prepared according to **General Procedure D** using **2t** (370 mg, 1.42 mmol, 1.00 equiv), CH_3CN (14.2 mL), and [Ir(dF(CF_3)ppy)$_2$(dtbbpy)](PF_6) (16.0 mg, 14.3 μmol, 1.0 mol%). The crude reaction mixture was purified using a Biotage automated flash chromatography system with a gradient elution of ethyl acetate/pentane (0% to 40%), affording **6t** (275 mg, 1.06 mmol, 74%) as a colourless oil.[A]

Notes: (**A**) The crude ^{1}H NMR spectrum indicated a 11:1 molar ratio of **6t**/**5t**.

^{1}H NMR (500 MHz, $CDCl_3$) δ 8.01 (d, J = 2.6 Hz, 1H), 7.37 (dd, J = 8.7, 2.7 Hz, 1H), 6.65 (d, J = 8.6 Hz, 1H), 3.82 (s, 3H), 2.87 (ddd, J = 13.0, 11.4, 6.0 Hz, 1H), 2.50 – 2.34 (m, 5H), 2.11 (td, J = 11.8, 5.4 Hz, 1H), 1.03 (s, 3H).

^{13}C NMR (126 MHz, $CDCl_3$) δ 170.9, 167.8, 163.6, 144.9, 136.4, 123.4, 110.7, 64.1, 61.0, 53.4, 26.19, 26.15, 24.2, 12.8.

IR (ATR): $\tilde{\nu}$ (cm^{-1}) = 1774, 1701, 1607, 1568, 1497, 1446, 1375, 1311, 1277, 1192, 1023.

HRMS (ESI$^+$) calcd. for $C_{14}H_{17}N_2O_3^+$ [M+H]$^+$: 261.1234, found: 261.1224.

2-(*tert*-Butyl) 1-methyl 4-methyl-3-oxo-2-azabicyclo[2.1.1]hexane-1,2-dicarboxylate (5u)

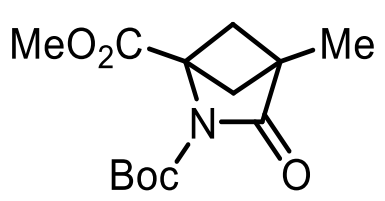

Prepared according to **General Procedure D** using **2u** (1.05 g, 3.90 mmol, 1.00 equiv), CH_3CN (240 mL, 0.016 M) and benzophenone (177 mg, 0.97 mmol, 0.25 equiv).[A] The crude reaction mixture was purified using a Biotage automated flash chromatography system with a gradient elution of ethyl acetate/pentane (0% to 30%), affording **5u** (830 mg, 3.09 mmol, 79%) as a white solid.

Notes: (**A**) 40 W Kessil KSPR160L lamp (370 nm, 100% intensity) was used.

M.P.: 77-79 °C

^{1}H NMR (500 MHz, $CDCl_3$) δ 3.76 (s, 3H), 2.46 (dd, J = 4.9, 1.8 Hz, 2H), 2.41 (dd, J = 4.9, 1.8 Hz, 2H), 1.45 (s, 9H), 1.26 (s, 3H).

^{13}C NMR (126 MHz, $CDCl_3$) δ 176.7, 166.9, 149.5, 83.5, 63.8, 53.4, 52.4, 49.7, 28.0, 13.0.

IR (ATR): $\tilde{\nu}$ (cm^{-1}) = 1801, 1749, 1715, 1439, 1306, 1210, 1157, 1088, 1036.

HRMS (ESI$^+$) calcd. for $C_{13}H_{19}NO_5Na^+$ [M+Na]$^+$: 292.1155, found: 292.1145.

2,2,2-Trichloroethyl 1-(4-methoxyphenyl)-4-methyl-3-oxo-2-azabicyclo[2.2.0]hexane-2-carboxylate (6v)

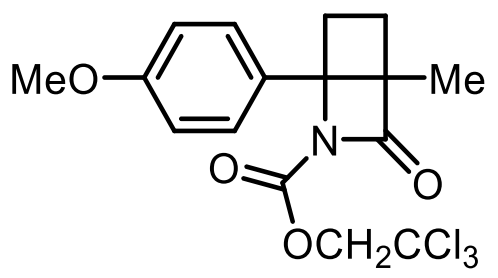

Prepared according to **General Procedure D** using **2v** (630 mg, 1.61 mmol, 1.00 equiv), CH_3CN (16.1 mL), and $[Ir(dF(CF_3)ppy)_2(dtbbpy)](PF_6)$ (18.0 mg, 16.0 μmol, 1.0 mol%). The crude reaction mixture was purified using a Biotage automated flash chromatography system with a gradient elution of ethyl acetate/pentane (0% to 25%), affording **6v** (470 mg, 1.20 mmol, 75%) as a colourless oil.[A]

Notes: (**A**) The crude ^{1}H NMR spectrum indicated a >25:1 molar ratio of **6v**/**5v**.

^{1}H NMR (500 MHz, $CDCl_3$) δ 7.21 (d, J = 8.8 Hz, 2H), 6.88 (d, J = 8.8 Hz, 2H), 4.77 (d, J = 12.0 Hz, 1H), 4.66 (d, J = 11.9 Hz, 1H), 3.76 (s, 3H), 3.05 (ddd, J = 13.5, 11.4, 6.4 Hz, 1H), 2.58 (ddd, J = 13.7, 8.7, 5.5 Hz, 1H), 2.48 (ddd, J = 13.0, 8.7, 6.4 Hz, 1H), 2.13 (ddd, J = 13.0, 11.4, 5.5 Hz, 1H), 1.10 (s, 3H).

^{13}C NMR (126 MHz, $CDCl_3$) δ 170.9, 159.4, 147.0, 127.6, 126.4, 114.0, 94.3, 74.5, 66.9, 62.2, 55.2, 26.1, 25.4, 13.0.

IR (ATR): $\tilde{\nu}$ (cm^{-1}) = 1810, 1732, 1614, 1516, 1445, 1379, 1315, 1278, 1252, 1178, 1093.

HRMS (ESI$^+$) calcd. for $C_{16}H_{16}Cl_3NO_4Na^+$ [M+Na]$^+$: 414.0037, found: 414.0030.

1-(4-Methoxyphenyl)-4-methyl-2-propionyl-2-azabicyclo[2.2.0]hexan-3-one (6w)

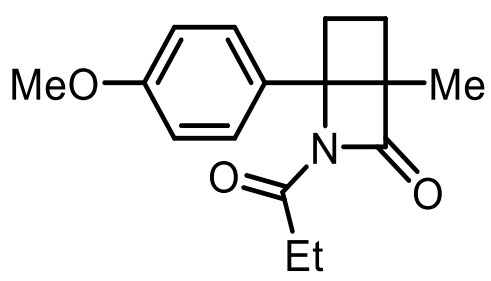

Prepared according to **General Procedure D** using **2w** (280 mg, 1.03 mmol, 1.00 equiv), CH_3CN (10.3 mL), and [Ir(dF(CF_3)ppy)$_2$(dtbbpy)](PF_6) (11.5 mg, 0.010 mmol, 1 mol%). The crude reaction mixture was purified using a Biotage automated flash chromatography system with a gradient elution of ethyl acetate/pentane (0% to 25%), affording **6w** (232 mg, 0.85 mmol, 83%) as a colourless oil.[A]

Notes: (**A**) The crude ^{1}H NMR spectrum indicated a 20:1 molar ratio of **6w**/**5w**.

^{1}H NMR (500 MHz, $CDCl_3$) δ 7.16 (d, J = 8.9 Hz, 2H), 6.89 (d, J = 8.8 Hz, 2H), 3.76 (s, 3H), 2.97 (ddd, J = 12.9, 11.4, 6.0 Hz, 1H), 2.88 – 2.72 (m, 2H), 2.50 (ddd, J = 12.8, 8.7, 5.3 Hz, 1H), 2.44 (ddd, J = 12.8, 8.7, 6.0 Hz, 1H), 2.15 (ddd, J = 12.8, 11.4, 5.3 Hz, 1H), 1.17 (t, J = 7.5 Hz, 3H), 1.08 (s, 3H).

^{13}C NMR (126 MHz, $CDCl_3$) δ 172.0, 171.5, 159.2, 127.3, 127.1, 114.0, 65.5, 60.6, 55.2, 30.5, 26.4, 26.3, 12.9, 8.0.

IR (ATR): $\tilde{\nu}$ (cm^{-1}) = 1772, 1699, 1614, 1515, 1461, 1371, 1249, 1178, 1031.

HRMS (ESI$^+$) calcd. for $C_{16}H_{19}NO_3Na^+$ [M+Na]$^+$: 296.1257, found: 296.1271.

2-Isobutyryl-1-(4-methoxyphenyl)-4-methyl-2-azabicyclo[2.2.0]hexan-3-one (6x)

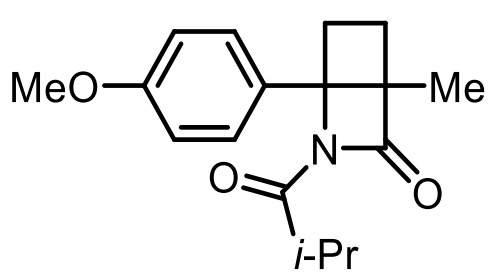

Prepared according to **General Procedure D** using **2x** (275 mg, 0.96 mmol, 1.00 equiv), CH_3CN (9.6 mL), and [Ir(dF(CF_3)ppy)$_2$(dtbbpy)](PF_6) (10.8 mg, 9.6 μmol, 1.0 mol%). The crude reaction mixture was purified using a Biotage automated flash chromatography system with a gradient elution of ethyl acetate/pentane (0% to 30%), affording **6x** (183 mg, 0.64 mmol, 67%) as a colourless oil.[A]

Notes: (**A**) The crude ^{1}H NMR spectrum indicated an 18:1 molar ratio of **6x**/**5x**.

^{1}H NMR (500 MHz, $CDCl_3$) δ 7.15 (d, J = 8.8 Hz, 2H), 6.89 (d, J = 8.8 Hz, 2H), 3.77 (s, 3H), 3.34 (hept, J = 6.9 Hz, 1H), 3.04 – 2.91 (m, 1H), 2.52 – 2.41 (m, 2H), 2.21 – 2.12 (m, 1H), 1.25 (d, J = 7.0 Hz, 3H), 1.17 (d, J = 6.8 Hz, 3H), 1.09 (s, 3H).

^{13}C NMR (126 MHz, $CDCl_3$) δ 175.3, 171.2, 159.2, 127.3, 127.2, 114.1, 65.4, 60.4, 55.3, 35.1, 26.5, 26.3, 19.1, 17.4, 13.0.

IR (ATR): $\tilde{\nu}$ (cm^{-1}) = 1773, 1698, 1614, 1516, 1462, 1386, 1248, 1179, 1032.

HRMS (ESI$^+$) calcd. for $C_{17}H_{21}NO_3Na^+$ [M+Na]$^+$: 310.1414, found: 310.1427.

Phenyl 1-(4-methoxyphenyl)-4-methyl-3-oxo-2-azabicyclo[2.2.0]hexane-2-carboxylate (6y)

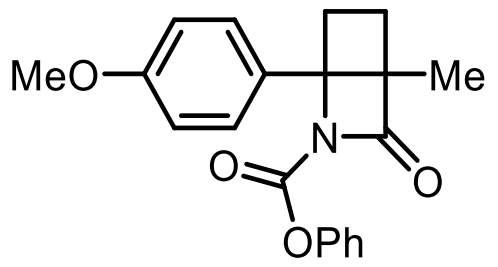

Prepared according to **General Procedure D** using **2y** (337 mg, 1.00 mmol, 1.00 equiv), CH_3CN (10.0 mL), and [Ir(dF(CF_3)ppy)$_2$(dtbbpy)](PF_6) (11.2 mg, 10.0 µmol, 1.0 mol%). The crude reaction mixture was purified using a Biotage automated flash chromatography system with a gradient elution of ethyl acetate/pentane (0% to 30%), affording **6y** (232 mg, 0.69 mmol, 69%) as a white solid.[A]

Notes: (**A**) The crude ^{1}H NMR spectrum indicated a 16:1 molar ratio of **6y**/**5y**.

M.P.: 143-145 °C

^{1}H NMR (500 MHz, $CDCl_3$) δ 7.34 – 7.28 (m, 4H), 7.18 (t, *J* = 7.4 Hz, 1H), 7.11 (d, *J* = 8.1 Hz, 2H), 6.95 (d, *J* = 8.9 Hz, 2H), 3.78 (s, 3H), 3.07 (ddd, *J* = 13.6, 11.4, 6.3 Hz, 1H), 2.66 (ddd, *J* = 13.8, 8.7, 5.6 Hz, 1H), 2.56 (ddd, *J* = 13.0, 8.7, 6.3 Hz, 1H), 2.18 (ddd, *J* = 13.0, 11.4, 5.6 Hz, 1H), 1.15 (s, 3H).

^{13}C NMR (126 MHz, $CDCl_3$) δ 170.8, 159.3, 149.9, 147.0, 129.3, 127.5, 126.7, 126.1, 121.2, 114.1, 66.7, 61.8, 55.2, 26.4, 25.5, 13.0.

IR (ATR): $\tilde{\nu}$ (cm^{-1}) = 1806, 1737, 1614, 1516, 1320, 1276, 1253, 1180, 1024.

HRMS (ESI$^+$) calcd. for $C_{20}H_{19}NO_4Na^+$ [M+Na]$^+$: 360.1206, found: 360.1222.

Benzyl 1-(4-methoxyphenyl)-4-methyl-3-oxo-2-azabicyclo[2.2.0]hexane-2-carboxylate (6z)

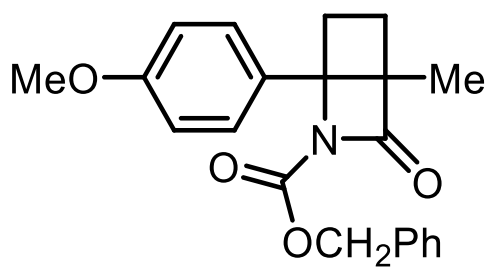

Prepared according to **General Procedure D** using **2z** (365 mg, 1.04 mmol, 1.00 equiv), CH_3CN (10.4 mL), and [Ir(dF(CF_3)ppy)$_2$(dtbbpy)](PF_6) (11.7 mg, 10.4 µmol, 1.0 mol%). The crude reaction mixture was purified using a Biotage automated flash chromatography system with a gradient elution of ethyl acetate/pentane (0% to 25%), affording **6z** (191 mg, 0.54 mmol, 52%) as a white solid.[A]

Notes: (**A**) The crude ^{1}H NMR spectrum indicated a 10:1 molar ratio of **6z**/**5z**.

M.P.: 137-139 °C

^{1}**H NMR** (500 MHz, $CDCl_3$) δ 7.31 – 7.27 (m, 3H), 7.24 – 7.19 (m, 4H), 6.90 (d, *J* = 8.9 Hz, 2H), 5.23 (d, *J* = 12.5 Hz, 1H), 5.18 (d, *J* = 12.5 Hz, 1H), 3.80 (s, 3H), 3.06 – 2.96 (m, 1H), 2.55 – 2.43 (m, 2H), 2.15 – 2.08 (m, 1H), 1.10 (s, 3H).

^{13}**C NMR** (126 MHz, $CDCl_3$) δ 170.9, 159.3, 148.8, 135.1, 128.4, 128.2, 127.8, 127.5, 126.9, 114.0, 67.6, 66.4, 61.6, 55.2, 26.3, 25.5, 13.0.

IR (ATR): $\tilde{\nu}$ (cm^{-1}) = 1803, 1713, 1613, 1516, 1455, 1387, 1314, 1283, 1252, 1207, 1180.

HRMS (ESI^+) calcd. for $C_{21}H_{21}NO_4Na^+$ $[M+Na]^+$: 374.1363, found: 374.1382.

Methyl 1-(4-methoxyphenyl)-4-methyl-3-oxo-2-azabicyclo[2.2.0]hexane-2-carboxylate (6aa)

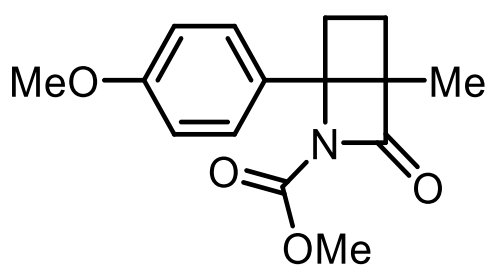

Prepared according to **General Procedure D** using **2aa** (285 mg, 1.04 mmol, 1.00 equiv), CH_3CN (10.4 mL), and $[Ir(dF(CF_3)ppy)_2(dtbbpy)](PF_6)$ (11.7 mg, 10.4 µmol, 1.0 mol%). The crude reaction mixture was purified using a Biotage automated flash chromatography system with a gradient elution of ethyl acetate/pentane (0% to 25%), affording **6aa** (173 mg, 0.63 mmol, 61%) as a colourless oil.[A]

Notes: (**A**) The crude ^{1}H NMR spectrum indicated a 10:1 molar ratio of **6aa**/**5aa**.

^{1}**H NMR** (500 MHz, $CDCl_3$) δ 7.20 (d, *J* = 8.8 Hz, 2H), 6.90 (d, *J* = 8.8 Hz, 2H), 3.78 (s, 3H), 3.77 (s, 3H), 2.99 (ddd, *J* = 13.1, 11.4, 6.0 Hz, 1H), 2.51 (ddd, *J* = 13.3, 8.7, 5.4 Hz, 1H), 2.45 (ddd, *J* = 12.9, 8.7, 6.1 Hz, 1H), 2.12 (ddd, *J* = 13.0, 11.5, 5.4 Hz, 1H), 1.07 (s, 3H).

^{13}**C NMR** (126 MHz, $CDCl_3$) δ 170.9, 159.3, 149.6, 127.5, 126.9, 114.1, 66.2, 61.4, 55.2, 53.3, 26.3, 25.7, 13.0.

IR (ATR): $\tilde{\nu}$ (cm^{-1}) = 1790, 1729, 1614, 1516, 1438, 1320, 1278, 1252, 1178, 1030.

HRMS (ESI^+) calcd. for $C_{15}H_{17}NO_4Na^+$ $[M+Na]^+$: 298.1050, found: 298.1064.

***tert*-Butyl 1-(4-methoxyphenyl)-4-methyl-3-oxo-2-azabicyclo[2.2.0]hexane-2-carboxylate (6ab)**

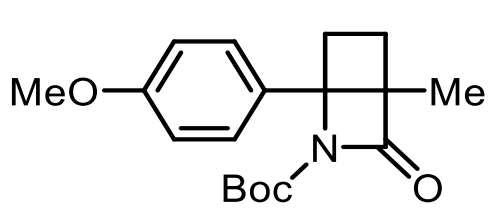

Prepared according to **General Procedure D** using **2ab** (2.63 g, 8.30 mmol, 1.00 equiv), CH_3CN (83.0 mL), and $[Ir(dF(CF_3)ppy)_2(dtbbpy)](PF_6)$ (46.6 mg, 41.5 µmol, 0.50 mol%). The crude reaction mixture was purified using a Biotage automated flash chromatography system with a gradient elution

of ethyl acetate/pentane (0% to 35%), affording **6ab** (1.09 g, 3.44 mmol, 41%) as a colourless oil and **5ab** (240 mg, 0.76 mmol, 9%) as a white solid.[A]

Notes: (**A**) The crude ^{1}H NMR spectrum indicated a 4.5:1 molar ratio of **6ab**/**5ab**.

^{1}H NMR (500 MHz, $CDCl_3$) δ 7.17 (d, J = 8.9 Hz, 2H), 6.88 (d, J = 8.8 Hz, 2H), 3.77 (s, 3H), 3.01 – 2.91 (m, 1H), 2.49 – 2.39 (m, 2H), 2.11 – 2.03 (m, 1H), 1.36 (s, 9H), 1.04 (s, 3H).

^{13}C NMR (126 MHz, $CDCl_3$) δ 171.4, 159.1, 147.7, 127.5, 127.4, 113.8, 82.9, 66.2, 61.1, 55.2, 27.9, 26.3, 25.5, 13.0.

IR (ATR): $\tilde{\nu}$ (cm^{-1}) = 1796, 1718, 1614, 1516, 1457, 1370, 1321, 1251, 1152, 1035.

HRMS (ESI^{+}) calcd. for $C_{18}H_{23}NO_4Na^{+}$ [M+Na]$^{+}$: 340.1519, found: 340.1507.

***tert*-Butyl 1-(4-methoxyphenyl)-4-methyl-3-oxo-2-azabicyclo[2.1.1]hexane-2-carboxylate (5ab)**

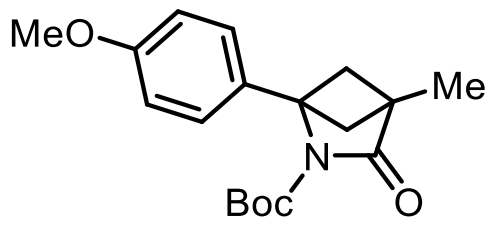

M.P.: 96-98 °C

^{1}H NMR (500 MHz, $CDCl_3$) δ 7.14 (d, J = 8.8 Hz, 2H), 6.86 (d, J = 8.8 Hz, 2H), 3.78 (s, 3H), 2.68 (dd, J = 4.6, 2.1 Hz, 2H), 2.34 (dd, J = 4.6, 2.1 Hz, 2H), 1.31 (s, 3H), 1.14 (s, 9H).

^{13}C NMR (126 MHz, $CDCl_3$) δ 179.1, 159.4, 149.7, 129.5, 127.9, 113.6, 81.7, 68.4, 55.3, 54.9, 49.6, 27.7, 13.2.

IR (ATR): $\tilde{\nu}$ (cm^{-1}) = 1786, 1722, 1702, 1615, 1519, 1455, 1360, 1291, 1246, 1157, 1032.

HRMS (ESI^{+}) calcd. for $C_{18}H_{23}NO_4Na^{+}$ [M+Na]$^{+}$: 340.1519, found: 340.1508.

6-(4-Methoxyphenyl)-3-methyl-4,5-dihydropyridin-2-yl pivalate (9)

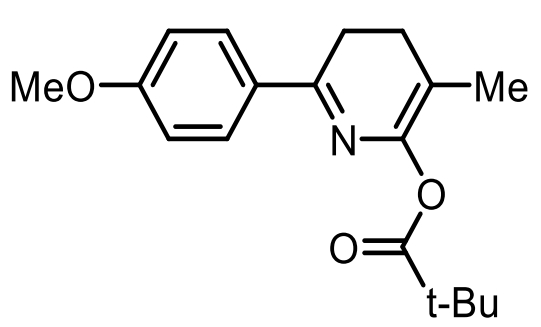

Prepared according to **General Procedure D** using **2ac** (315 mg, 1.05 mmol, 1.00 equiv), CH_3CN (10.5 mL), and [Ir(dF(CF_3)ppy)$_2$(dtbbpy)](PF_6) (11.7 mg, 10.4 µmol, 1.0 mol%). The crude reaction mixture was purified using a Biotage automated flash chromatography system with a gradient elution of ethyl acetate/pentane (0% to 25%), affording **9** (193 mg, 0.64 mmol, 61%) as a colourless oil.

^{1}H NMR (500 MHz, CD_2Cl_2) δ 7.87 (d, *J* = 8.9 Hz, 2H), 6.93 (d, *J* = 8.9 Hz, 2H), 3.84 (s, 3H), 2.77 (t, *J* = 9.8 Hz, 2H), 2.36-2.28 (m, 2H), 1.73 (s, 3H), 1.36 (s, 9H).

^{13}C NMR (126 MHz, CD_2Cl_2) δ 176.8, 162.7, 162.0, 145.9, 130.9, 128.8, 114.0, 108.3, 55.7, 39.2, 27.4, 26.1, 24.3, 15.6.

IR (ATR): $\tilde{\nu}$ (cm^{-1}) = 1745, 1607, 1512, 1461, 1276, 1259, 1116, 1029.

HRMS (ESI^+) calcd. for $C_{18}H_{24}NO_3^+$ $[M+H]^+$: 302.1751, found: 302.1743.

(1*S*,4*S*)-1-(4-methoxyphenyl)-4-methyl-2-((*S*)-2-phenylpropanoyl)-2-azabicyclo[2.2.0] hexan-3-one & (1*R*,4*R*)-1-(4-methoxyphenyl)-4-methyl-2-((*S*)-2-phenylpropanoyl)-2-azabicyclo[2.2.0]hexan-3-one (6ad and 6ad')

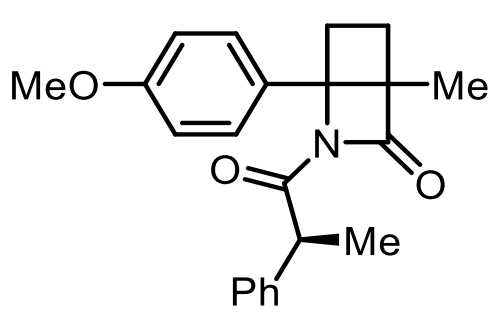

Diastereoisomer 1

Prepared according to **General Procedure D** using **2ad** (151 mg, 0.43 mmol, 1.00 equiv), CH_3CN (4.3 mL), and $[Ir(dF(CF_3)ppy)_2(dtbbpy)](PF_6)$ (4.8 mg, 4.3 μmol, 1 mol%). The crude reaction mixture was purified using a Biotage automated flash chromatography system with a gradient elution of ethyl acetate/pentane (0% to 25%) and CH_2Cl_2/pentane (0% to 100%), affording **6ad** (41 mg, 0.12 mmol, 27%) as a colourless oil and **6ad'** (40.3 mg, 0.12 mmol, 27%) as a colourless oil.[A]

Notes: (**A**) The crude ^{1}H NMR spectrum indicated an 18:1 molar ratio of (**6ad+6ad'**)/(**5ad+5ad'**). The specific identity of the diastereomers could not be confirmed so no assignment has been made.

^{1}H NMR (500 MHz, $CDCl_3$) δ 7.42 – 7.39 (m, 2H), 7.35 – 7.31 (m, 2H), 7.29 – 7.25 (m, 1H), 7.20 (d, *J* = 8.8 Hz, 2H), 6.92 (d, *J* = 8.7 Hz, 2H), 4.64 (q, *J* = 7.0 Hz, 1H), 3.82 (s, 3H), 2.87 (dt, *J* = 13.5, 8.7 Hz, 1H), 2.20 (ddd, *J* = 13.7, 7.7, 6.4 Hz, 1H), 2.01-1.96 (m, 2H), 1.52 (d, *J* = 7.1 Hz, 3H), 1.07 (s, 3H).

^{13}C NMR (126 MHz, $CDCl_3$) δ 172.4, 171.5, 159.3, 139.7, 128.7, 128.2, 127.5, 127.4, 114.2, 66.0, 60.6, 55.4, 45.9, 26.4, 26.1, 18.9, 12.9.

IR (ATR): $\tilde{\nu}$ (cm^{-1}) = 1777, 1700, 1517, 1454, 1358, 1275, 1260, 1179.

HRMS (ESI^+) calcd. for $C_{22}H_{23}NO_3Na^+$ $[M+Na]^+$: 372.1570, found: 372.1568.

Specific rotation $[\alpha]_D^{20}$ = +77 (c = 1.0, $CHCl_3$)

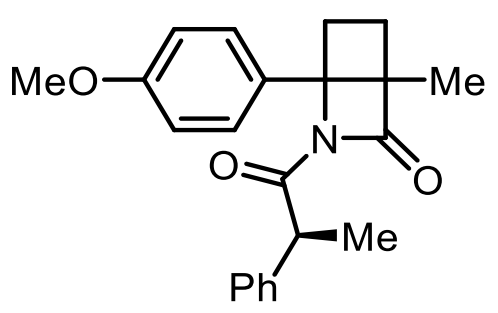

Diastereoisomer 2

^{1}H NMR (500 MHz, $CDCl_3$) δ 7.39 – 7.27 (m, 5H), 6.80 (d, *J* = 8.9 Hz, 2H), 6.74 (d, *J* = 8.8 Hz, 2H), 4.60 (q, *J* = 7.3 Hz, 1H), 3.76 (s, 3H), 3.02 (ddd, *J* = 13.0, 11.4, 6.1 Hz, 1H), 2.56 (dt, *J* = 12.8, 6.9 Hz, 1H), 2.49 (ddd, *J* = 12.7, 8.6, 6.1 Hz, 1H), 2.14 (ddd, *J* = 12.7, 11.4, 5.4 Hz, 1H), 1.48 (d, *J* = 7.0 Hz, 3H), 0.97 (s, 3H).

^{13}C NMR (126 MHz, $CDCl_3$) δ 172.0, 171.1, 159.2, 140.4, 128.8, 128.2, 127.4, 127.2, 126.9, 114.0, 65.6, 60.7, 55.3, 46.6, 26.5, 26.0, 17.9, 13.0.

IR (ATR): $\tilde{\nu}$ (cm^{-1}) = 1775, 1701, 1516, 1453, 1356, 1275, 1260, 1179.

HRMS (ESI^+) calcd. for $C_{22}H_{23}NO_3Na^+$ $[M+Na]^+$: 372.1570, found: 372.1566.

Specific rotation $[\alpha]_D^{20}$ = +22 (c = 1.0, $CHCl_3$)

2-Acetyl-1-(4-methoxyphenyl)-2-azabicyclo[2.2.0]hexan-3-one (6ae)

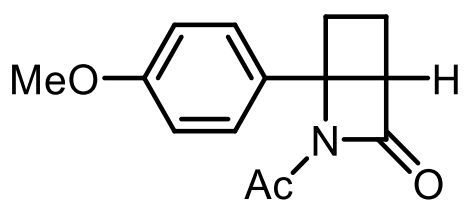

Prepared according to **General Procedure D** using **2ae** (220 mg, 0.90 mmol, 1.00 equiv), CH_3CN (9.0 mL), and [Ir(dF(CF_3)ppy)$_2$(dtbbpy)](PF_6) (10.0 mg, 9.0 µmol, 1.0 mol%). The crude reaction mixture was purified using a Biotage automated flash chromatography system with a gradient elution of ethyl acetate/pentane (0% to 35%), affording **6ae** (120 mg, 0.49 mmol, 55%) as a colourless oil and **5ae** (17 mg, 0.07 mmol, 8%) as a white solid.[A]

Notes: (**A**) The crude ^{1}H NMR spectrum indicated a 5:1 molar ratio of **6ae**/**5ae**.

^{1}H NMR (500 MHz, $CDCl_3$) δ 7.28 (d, *J* = 8.8 Hz, 2H), 6.87 (d, *J* = 8.8 Hz, 2H), 3.76 (s, 3H), 3.61 (dd, *J* = 9.4, 2.9 Hz, 1H), 2.90 (ddd, *J* = 13.3, 11.4, 6.6 Hz, 1H), 2.68 (ddd, *J* = 13.3, 8.6, 5.8 Hz, 1H), 2.63 – 2.53 (m, 1H), 2.39 (s, 3H), 2.26 (dddd, *J* = 13.1, 8.6, 6.6, 2.9 Hz, 1H).

^{13}C NMR (126 MHz, $CDCl_3$) δ 168.7, 167.9, 159.3, 129.0, 127.4, 113.8, 63.1, 55.2, 54.3, 28.9, 24.3, 18.6.

IR (ATR): $\tilde{\nu}$ (cm^{-1}) = 1771, 1699, 1613, 1516, 1463, 1371, 1288, 1250, 1211, 1178, 1032.

HRMS (ESI^+) calcd. for $C_{14}H_{15}NO_3Na^+$ $[M+Na]^+$: 268.0944, found: 268.0937.

2-Acetyl-1-(4-methoxyphenyl)-2-azabicyclo[2.1.1]hexan-3-one (5ae)

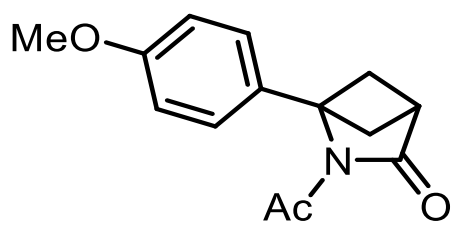

M.P.: 86-88 °C

^{1}**H NMR** (500 MHz, $CDCl_3$) δ 7.19 (d, J = 8.7 Hz, 2H), 6.89 (d, J = 8.7 Hz, 2H), 3.81 (s, 3H), 3.02 (t, J = 2.7 Hz, 1H), 2.76-2.71 (m, 2H), 2.61-2.55 (m, 2H), 2.38 (s, 3H).

^{13}**C NMR** (126 MHz, $CDCl_3$) δ 178.8, 169.6, 159.5, 129.3, 127.7, 113.7, 72.7, 55.3, 50.6, 44.4, 26.3.

IR (ATR): $\tilde{\nu}$ (cm^{-1}) = 1759, 1693, 1614, 1519, 1369, 1348, 1289, 1246, 1177, 1126, 1036.

HRMS (ESI^+) calcd. for $C_{14}H_{15}NO_3Na^+$ $[M+Na]^+$: 268.0944, found: 268.0943.

2-Acetyl-1-phenyl-2-azabicyclo[2.2.0]hexan-3-one (6af)

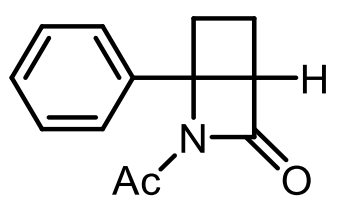

Prepared according to **General Procedure D** using **2af** (150 mg, 0.70 mmol, 1.00 equiv), CH_3CN (7.0 mL), and $[Ir(dF(CF_3)ppy)_2(dtbbpy)](PF_6)$ (7.8 mg, 7.0 µmol, 1 mol%). The crude reaction mixture was purified using a Biotage automated flash chromatography system with a gradient elution of ethyl acetate/pentane (0% to 35%), affording **6af** (77 mg, 0.36 mmol, 51%) as a colourless oil and **5af** (38 mg, 0.18 mmol, 25%) as a white solid.[A,B]

Notes: (**A**) The crude ^{1}H NMR spectrum indicated a 2:1 molar ratio of **6af**/**5af**.

(**B**) When HFIP was used as the solvent, **6af** was isolated in 58% yield. The crude ^{1}H NMR spectrum indicated a 5:1 molar ratio of **6af**:**5af**.

^{1}**H NMR** (500 MHz, $CDCl_3$) δ 7.374 (s, 2H), 7.366 (s, 2H), 7.34 – 7.26 (m, 1H), 3.66 (dd, J = 9.5, 2.9 Hz, 1H), 2.97 (ddd, J = 13.3, 11.4, 6.6 Hz, 1H), 2.72 (ddd, J = 13.4, 8.6, 5.8 Hz, 1H), 2.63 (dddd, J = 13.1, 11.4, 9.5, 5.8 Hz, 1H), 2.43 (s, 3H), 2.31 (dddd, J = 13.1, 8.6, 6.6, 2.9 Hz, 1H).

^{13}**C NMR** (126 MHz, $CDCl_3$) δ 168.6, 168.0, 137.0, 128.6, 128.2, 126.2, 63.4, 54.6, 28.8, 24.4, 18.8.

IR (ATR): $\tilde{\nu}$ (cm^{-1}) = 1773, 1700, 1371, 1285, 1210, 1183, 997.

HRMS (ESI^+) calcd. for $C_{13}H_{13}NO_2Na^+$ $[M+Na]^+$: 238.0838, found: 238.0839.

2-Acetyl-1-phenyl-2-azabicyclo[2.1.1]hexan-3-one (5af)

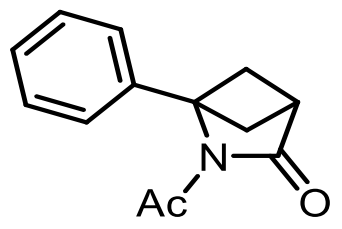

M.P.: 94-96 °C

^{1}H NMR (500 MHz, $CDCl_3$) δ 7.39 – 7.32 (m, 3H), 7.29 – 7.27 (m, 2H), 3.04 (t, J = 2.7 Hz, 1H), 2.78-2.74 (m, 2H), 2.63-2.60 (m, 2H), 2.39 (s, 3H).

^{13}C NMR (126 MHz, $CDCl_3$) δ 178.7, 169.5, 137.1, 128.3, 128.2, 126.3, 72.7, 50.6, 44.4, 26.1.

IR (ATR): $\tilde{\nu}$ (cm^{-1}) = 1760, 1678, 1450, 1375, 1305, 1198, 1127, 1088, 985.

HRMS (ESI^+) calcd. for $C_{13}H_{13}NO_2Na^+$ $[M+Na]^+$: 238.0838, found: 238.0837.

Methyl 4-(2-acetyl-3-oxo-2-azabicyclo[2.2.0]hexan-1-yl)benzoate (6ag)

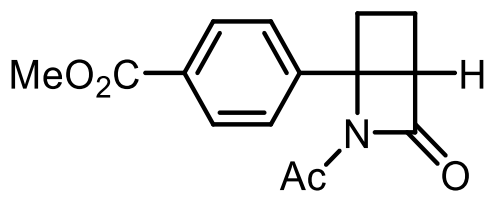

Prepared according to **General Procedure D** using **2ag** (185 mg, 0.68 mmol, 1.00 equiv), CH_3CN (6.8 mL), and $[Ir(dF(CF_3)ppy)_2(dtbbpy)](PF_6)$ (7.6 mg, 6.8 µmol, 1.0 mol%). The crude reaction mixture was purified using a Biotage automated flash chromatography system with a gradient elution of ethyl acetate/pentane (0% to 40%), affording **6ag** as a colourless oil (43 mg, 0.16 mmol, 23%) and **5ag** (92 mg, 0.34 mmol, 50%) as a white solid.[A,B]

Notes: (**A**) The crude ^{1}H NMR spectrum indicated a 1:2 molar ratio of **6ag**/**5ag**.

(**B**) When HFIP was used as the solvent, **6ag** was isolated in 32% yield. The crude ^{1}H NMR spectrum indicated a 1.3:1 molar ratio of **6ag**/**5ag**.

^{1}H NMR (500 MHz, $CDCl_3$) δ 8.02 (d, J = 8.4 Hz, 2H), 7.42 (d, J = 8.5 Hz, 2H), 3.90 (s, 3H), 3.68 (dd, J = 9.5, 2.9 Hz, 1H), 3.02 – 2.94 (m, 1H), 2.75 – 2.62 (m, 2H), 2.43 (s, 3H), 2.37 – 2.29 (m, 1H).

^{13}C NMR (126 MHz, $CDCl_3$) δ 168.2, 168.0, 166.6, 141.9, 129.94, 129.91, 126.3, 63.0, 54.9, 52.3, 28.8, 24.4, 18.9.

IR (ATR): $\tilde{\nu}$ (cm^{-1}) = 1778, 1702, 1613, 1435, 1409, 1372, 1275, 1185, 1108, 1019.

HRMS (ESI^+) calcd. for $C_{15}H_{15}NO_4Na^+$ $[M+Na]^+$: 296.0893, found: 296.0891.

Methyl 4-(2-acetyl-3-oxo-2-azabicyclo[2.1.1]hexan-1-yl)benzoate (5ag)

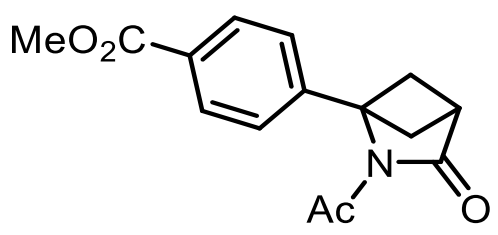

M.P.: 107-109 °C

^{1}H NMR (500 MHz, $CDCl_3$) δ 8.01 (d, *J* = 8.4 Hz, 2H), 7.31 (d, *J* = 8.4 Hz, 2H), 3.89 (s, 3H), 3.03 (t, *J* = 2.8 Hz, 1H), 2.76-2.72 (m, 2H), 2.64-2.58 (m, 2H), 2.36 (s, 3H).

^{13}C NMR (126 MHz, $CDCl_3$) δ 178.1, 169.4, 166.6, 141.9, 129.7, 129.5, 126.2, 71.9, 52.2, 50.4, 44.3, 25.8.

IR (ATR): $\tilde{\nu}$ (cm^{-1}) = 1768, 1696, 1613, 1436, 1371, 1347, 1276, 1195, 1113.

HRMS (ESI^+) calcd. for $C_{15}H_{15}NO_4Na^+$ $[M+Na]^+$: 296.0893, found: 296.0893.

2-Acetyl-4-benzyl-1-(4-methoxyphenyl)-2-azabicyclo[2.2.0]hexan-3-one (6ah)

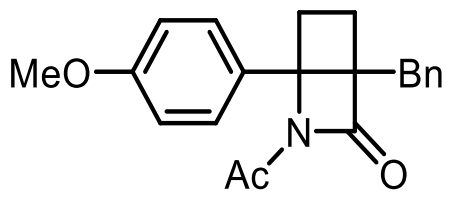

Prepared according to **General Procedure D** using **2ah** (595 mg, 1.78 mmol, 1.00 equiv), CH_3CN (17.8 mL), and $[Ir(dF(CF_3)ppy)_2(dtbbpy)](PF_6)$ (19.9 mg, 17.7 µmol, 1.0 mol%). The crude reaction mixture was purified using a Biotage automated flash chromatography system with a gradient elution of ethyl acetate/pentane (0% to 30%), affording **6ah** (422 mg, 1.26 mmol, 71%) as a colourless oil.[A]

Notes: (**A**) The crude ^{1}H NMR spectrum indicated a >25:1 molar ratio of **6ah**/**5ah**.

^{1}H NMR (500 MHz, $CDCl_3$) δ 7.24 – 7.19 (m, 5H), 7.00 – 6.95 (m, 4H), 3.86 (s, 3H), 2.97 (ddd, *J* = 13.6, 11.3, 6.6 Hz, 1H), 2.93 (d, *J* = 14.8 Hz, 1H), 2.77 (d, *J* = 15.0 Hz, 1H), 2.55 – 2.48 (m, 4H), 2.36 – 2.23 (m, 2H).

^{13}C NMR (126 MHz, $CDCl_3$) δ 171.1, 168.0, 159.4, 135.4, 129.7, 128.3, 127.6, 126.8, 126.5, 114.2, 66.1, 64.4, 55.4, 33.8, 26.1, 24.5, 23.6.

IR (ATR): $\tilde{\nu}$ (cm^{-1}) = 1773, 1702, 1614, 1515, 1454, 1371, 1276, 1253, 1179, 1029.

HRMS (ESI^+) calcd. for $C_{21}H_{21}NO_3Na^+$ $[M+Na]^+$: 358.1414, found: 358.1410.

2-acetyl-1-(4-methoxyphenyl)-4-(((triisopropylsilyl)oxy)methyl)-2-azabicyclo[2.2.0] hexan-3-one (6ai)

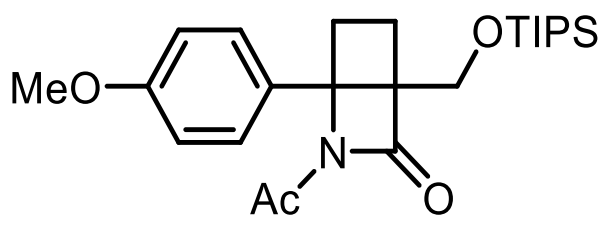

Prepared according to **General Procedure D** using **2ai** (530 mg, 1.23 mmol, 1.00 equiv), CH_3CN (12.3 mL), and [Ir(dF(CF_3)ppy)$_2$(dtbbpy)](PF_6) (13.8 mg, 12.3 µmol, 1.0 mol%). The crude reaction mixture was purified by two successive flash chromatography separations using a Biotage automated flash chromatography system with a gradient elution of Et_2O/pentane (0% to 50%) and ethyl acetate/CH_2Cl_2 (0% to 10%), affording **6ai** (372 mg, 0.86 mmol, 70%) as a colourless oil.[A]

Notes: (**A**) The crude ^{1}H NMR spectrum indicated a >25:1 molar ratio of **6ai**/**5ai**.

^{1}H NMR (500 MHz, $CDCl_3$) δ 7.27 (d, J = 8.8 Hz, 2H), 6.86 (d, J = 8.8 Hz, 2H), 3.84 (d, J = 11.6 Hz, 1H), 3.78 (d, J = 11.6 Hz, 1H), 3.76 (s, 3H), 3.01 – 2.92 (m, 1H), 2.58 – 2.44 (m, 5H), 2.28 – 2.21 (m, 1H), 0.98 – 0.86 (m, 21H).

^{13}C NMR (126 MHz, $CDCl_3$) δ 169.6, 168.0, 159.3, 127.8, 127.1, 113.9, 66.9, 66.5, 58.5, 55.4, 27.1, 24.6, 20.1, 17.84, 17.79, 11.7.

IR (ATR): $\tilde{\nu}$ (cm^{-1}) = 2942, 2865, 1777, 1706, 1615, 1516, 1462, 1371, 1307, 1276, 1179.

HRMS (ESI$^+$) calcd. for $C_{24}H_{38}NO_4Si^+$ [M+H]$^+$: 432.2565, found: 432.2586.

2-Acetyl-1-(4-methoxyphenyl)-4-phenyl-2-azabicyclo[2.2.0]hexan-3-one (6aj)

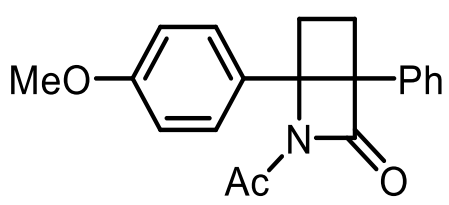

Prepared according to **General Procedure D** using **2aj** (500 mg, 1.56 mmol, 1.00 equiv), CH_3CN (15.6 mL), and [Ir(dF(CF_3)ppy)$_2$(dtbbpy)](PF_6) (17.5 mg, 15.6 µmol, 1.0 mol%). The crude reaction mixture was purified by two successive flash chromatography separations using a Biotage automated flash chromatography system with a gradient elution of ethyl acetate/pentane (0% to 40%) and ethyl acetate/CH_2Cl_2 (0% to 10%), affording **6aj** (168 mg, 0.52 mmol, 34%) as a pale-yellow oil and **5aj** (211 mg, 0.66 mmol, 42%) as a pale-yellow solid.[A]

Notes: (**A**) The crude ^{1}H NMR spectrum indicated a 1:1.2 molar ratio of **6aj**/**5aj**.

^{1}H NMR (500 MHz, $CDCl_3$) δ 7.25 – 7.16 (m, 5H), 7.06 (d, J = 8.8 Hz, 2H), 6.77 (d, J = 8.8 Hz, 2H), 3.72 (s, 3H), 3.18 (ddd, J = 13.1, 10.0, 7.7 Hz, 1H), 2.82 – 2.77 (m, 2H), 2.72 (ddd, J = 13.1, 8.3, 5.6 Hz, 1H), 2.53 (s, 3H).

^{13}C NMR (126 MHz, $CDCl_3$) δ 169.8, 168.2, 159.2, 133.6, 128.6, 127.9, 127.8, 126.9, 126.7, 113.8, 68.6, 67.7, 55.2, 27.6, 27.1, 24.7.

IR (ATR): $\tilde{\nu}$ (cm^{-1}) = 1774, 1707, 1612, 1516, 1448, 1372, 1276, 1259, 1179.

HRMS (ESI^+) calcd. for $C_{20}H_{20}NO_3^+$ $[M+H]^+$: 322.1438, found: 322.1434.

2-acetyl-1-(4-methoxyphenyl)-4-phenyl-2-azabicyclo[2.1.1]hexan-3-one (5aj)

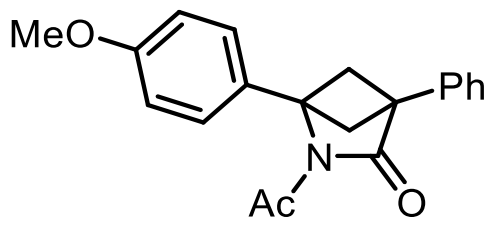

M.P.: 134-136 °C

^{1}H NMR (500 MHz, $CDCl_3$) δ 7.45 (t, J = 7.4 Hz, 2H), 7.40 – 7.36 (m, 1H), 7.34 – 7.29 (m, 4H), 6.95 (d, J = 8.7 Hz, 2H), 3.84 (s, 3H), 3.17 (dd, J = 4.2, 2.5 Hz, 2H), 2.79 (dd, J = 4.2, 2.3 Hz, 2H), 2.45 (s, 3H).

^{13}C NMR (126 MHz, $CDCl_3$) δ 177.6, 169.4, 159.5, 134.5, 128.8, 128.6, 128.0, 127.8, 127.0, 113.7, 67.6, 56.2, 55.3, 54.0, 26.2.

IR (ATR): $\tilde{\nu}$ (cm^{-1}) = 1766, 1707, 1612, 1516, 1448, 1372, 1276, 1259, 1179.

HRMS (ESI^+) calcd. for $C_{20}H_{19}NO_3Na^+$ $[M+Na]^+$: 344.1257, found: 344.1254.

(±)-(1*S*,4*S*,6*R*)-2-acetyl-1-(4-methoxyphenyl)-4,6-dimethyl-2-azabicyclo[2.2.0]hexan-3-one (6ak)

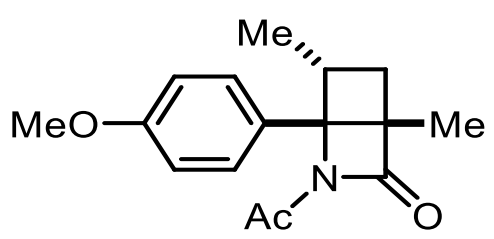

Prepared according to **General Procedure D** using **2ak** (510 mg, 1.87 mmol, 1.00 equiv), CH_3CN (18.7 mL), and [Ir(dF(CF_3)ppy)$_2$(dtbbpy)](PF_6) (21.0 mg, 18.7 μmol, 1.0 mol%). The crude reaction mixture was purified using a Biotage automated flash chromatography system with a gradient elution of pentane/ethyl acetate (0% to 35%), affording **6ak** (340 mg, 1.25 mmol, 67%) as a colourless oil.[A,B]

Notes: (**A**) The crude ^{1}H NMR spectrum indicated a 7:1 molar ratio of **6ak**/**5ak**.

(**B**) The compounds **6ak** were obtained as a single diastereomer (dr > 20:1 by crude ^{1}H NMR). The relative configuration was assigned by 1D NOE analysis.

^{1}H NMR (500 MHz, $CDCl_3$) δ 7.18 (d, J = 8.8 Hz, 2H), 6.90 (d, J = 8.8 Hz, 2H), 3.77 (s, 3H), 3.47 (dp, J = 10.5, 7.1 Hz, 1H), 2.46 (s, 3H), 2.21 (dd, J = 12.9, 10.6 Hz, 1H), 1.99 (dd, J = 12.9, 7.2 Hz, 1H), 1.17 (d, J = 7.0 Hz, 3H), 1.06 (s, 3H).

^{13}C NMR (126 MHz, $CDCl_3$) δ 172.2, 168.3, 159.2, 127.3, 127.0, 114.1, 68.9, 57.5, 55.2, 33.1, 32.7, 24.5, 16.8, 12.4.

IR (ATR): $\tilde{\nu}$ (cm^{-1}) = 1771, 1704, 1612, 1515, 1445, 1371, 1275, 1179, 1033.

HRMS (ESI^+) calcd. for $C_{16}H_{19}NO_3Na^+$ $[M+Na]^+$: 296.1257, found: 296.1246.

(±)-(2a*S*,3a*S*,9b*S*)-1-acetyl-2a-methyl-3,3a,4,5-tetrahydro-1*H*-naphtho[1',2':1,4] cyclobuta[1,2-b]azet-2(2a*H*)-one (6al)

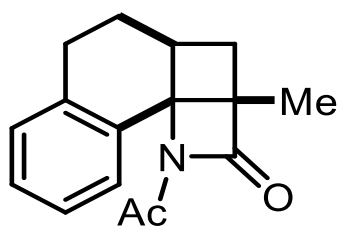

Prepared according to **General Procedure D** using **2al** (465 mg, 1.82 mmol, 1.00 equiv), CH_3CN (18.2 mL), and [Ir(dF(CF_3)ppy)$_2$(dtbbpy)](PF_6) (20.0 mg, 17.8 μmol, 1.0 mol%). The crude reaction mixture was purified using a Biotage automated flash chromatography system with a gradient elution of Et_2O/pentane (0% to 40%), affording **6al** (246 mg, 0.96 mmol, 53%) as a colourless oil and **5al** (78 mg, 0.31 mmol, 17%) as a white solid.[A, B]

Notes: (**A**) The crude ^{1}H NMR spectrum indicated a 3:1 molar ratio of **6al**/**5al**.

(**B**) The compounds **6al** and **5al** were obtained as a single diastereomer (dr > 20:1 by crude ^{1}H NMR). The relative configuration of **6al** was assigned by 1D NOE analysis. The relative configuration of **5al** at the bridge position was not determined.

^{1}H NMR (500 MHz, $CDCl_3$) δ 7.22 – 7.17 (m, 3H), 7.09 – 7.05 (m, 1H), 2.93 – 2.87 (m, 1H), 2.85 (ddd, *J* = 15.6, 6.8, 3.7 Hz, 1H), 2.74 – 2.66 (m, 1H), 2.64 (dd, *J* = 13.2, 8.1 Hz, 1H), 2.47 (s, 3H), 2.02 (dddd, *J* = 13.5, 10.2, 6.7, 3.8 Hz, 1H), 1.84 (dd, *J* = 13.3, 5.5 Hz, 1H), 1.79 (dddd, *J* = 13.4, 6.8, 5.4, 3.9 Hz, 1H), 1.05 (s, 3H).

^{13}C NMR (126 MHz, $CDCl_3$) δ 171.7, 167.8, 139.3, 130.3, 128.5, 127.7, 126.7, 124.9, 64.5, 60.2, 36.1, 31.9, 28.0, 27.4, 24.4, 13.2.

IR (ATR): $\tilde{\nu}$ (cm^{-1}) = 1774, 1701, 1457, 1371, 1308, 1276, 1199, 962.

HRMS (ESI^+) calcd. for $C_{16}H_{17}NO_2Na^+$ $[M+Na]^+$: 278.1152, found: 278.1151.

(±)-(3*R*, 9b*S*)-1-acetyl-3-methyl-3,3a,4,5-tetrahydro-3,9b-methanobenzo[g]indol-2(1*H*)-one (5al)

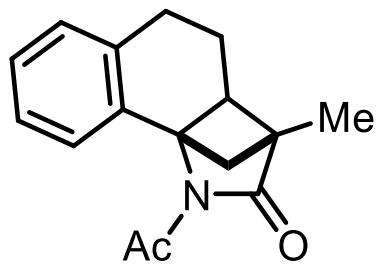

M.P.: 114-116 °C

^{1}H NMR (500 MHz, $CDCl_3$) δ 7.19 – 7.15 (m, 3H), 7.14 – 7.10 (m, 1H), 2.95 – 2.87 (m, 1H), 2.82 (dt, J = 16.2, 3.7 Hz, 1H), 2.77 – 2.71 (m, 1H), 2.68 (d, J = 8.2 Hz, 1H), 2.49 (s, 3H), 2.44 (dd, J = 8.2, 7.0 Hz, 1H), 2.15 – 2.09 (m, 2H), 1.26 (s, 3H).

^{13}C NMR (126 MHz, $CDCl_3$) δ 181.3, 169.7, 136.2, 132.3, 129.2, 127.6, 127.5, 126.0, 66.4, 61.1, 54.0, 52.1, 28.3, 26.0, 22.2, 10.8.

IR (ATR): $\tilde{\nu}$ (cm^{-1}) = 1758, 1696, 1447, 1361, 1276, 1261, 1182, 1146.

HRMS (ESI^+) calcd. for $C_{16}H_{17}NO_2Na^+$ $[M+Na]^+$: 278.1152, found: 278.1155.

(±)-(1*R*,4*S*,5*R*)-2-acetyl-1-(4-methoxyphenyl)-5-methyl-2-azabicyclo[2.2.0]hexan-3-one (6am)

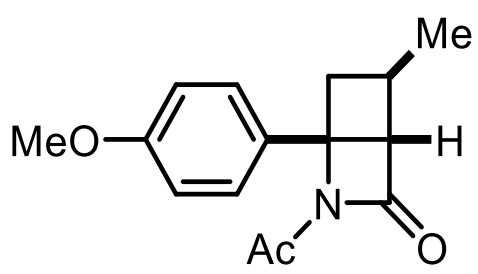

Prepared according to **General Procedure D** using **2am** (135 mg, 0.52 mmol, 1.00 equiv), CH_3CN (5.2 mL), and $[Ir(dF(CF_3)ppy)_2(dtbbpy)](PF_6)$ (5.8 mg, 5.2 μmol, 1.0 mol%). The crude reaction mixture was purified using a Biotage automated flash chromatography system with a gradient elution of pentane/ethyl acetate (0% to 40%), affording **6am** (31 mg, 0.12 mmol, 23%) as a white solid and **5am** (60 mg, 0.23 mmol, 44%) as a white solid.[A, B, C]

Notes: (**A**) The crude ^{1}H NMR spectrum indicated a 1:2.2 molar ratio of **6am**/**5am**.

(**B**) When HFIP was used as the solvent, **6am** was isolated in 27% yield. The crude ^{1}H NMR spectrum indicated a 1:1 molar ratio of **6am**:**5am**.

(**C**) The compounds **6am** and **5am** were obtained as a single diastereomer (dr > 20:1 by crude ^{1}H NMR). The relative configuration was assigned by 1D NOE analysis.

M.P.: 55-57 °C

^{1}H NMR (500 MHz, $CDCl_3$) δ 7.30 (d, J = 8.8 Hz, 2H), 6.89 (d, J = 8.8 Hz, 2H), 3.80 (s, 3H), 3.27 (d, J = 2.4 Hz, 1H), 2.93 (dd, J = 13.4, 7.7 Hz, 1H), 2.75-2.66 (m, 1H), 2.44-2.38 (m, 4H), 1.33 (d, J = 7.0 Hz, 3H).

^{13}C NMR (126 MHz, $CDCl_3$) δ 168.2, 168.2, 159.5, 129.6, 127.6, 114.0, 61.3, 60.9, 55.4, 37.6, 28.5, 24.5, 22.2.

IR (ATR): $\tilde{\nu}$ (cm^{-1}) = 1774, 1700, 1613, 1516, 1457, 1371, 1277, 1259, 1179, 1027.

HRMS (ESI^+) calcd. for $C_{15}H_{17}NO_3Na^+$ $[M+Na]^+$: 282.1101, found: 282.1090.

1D NOE analysis of **6am:**

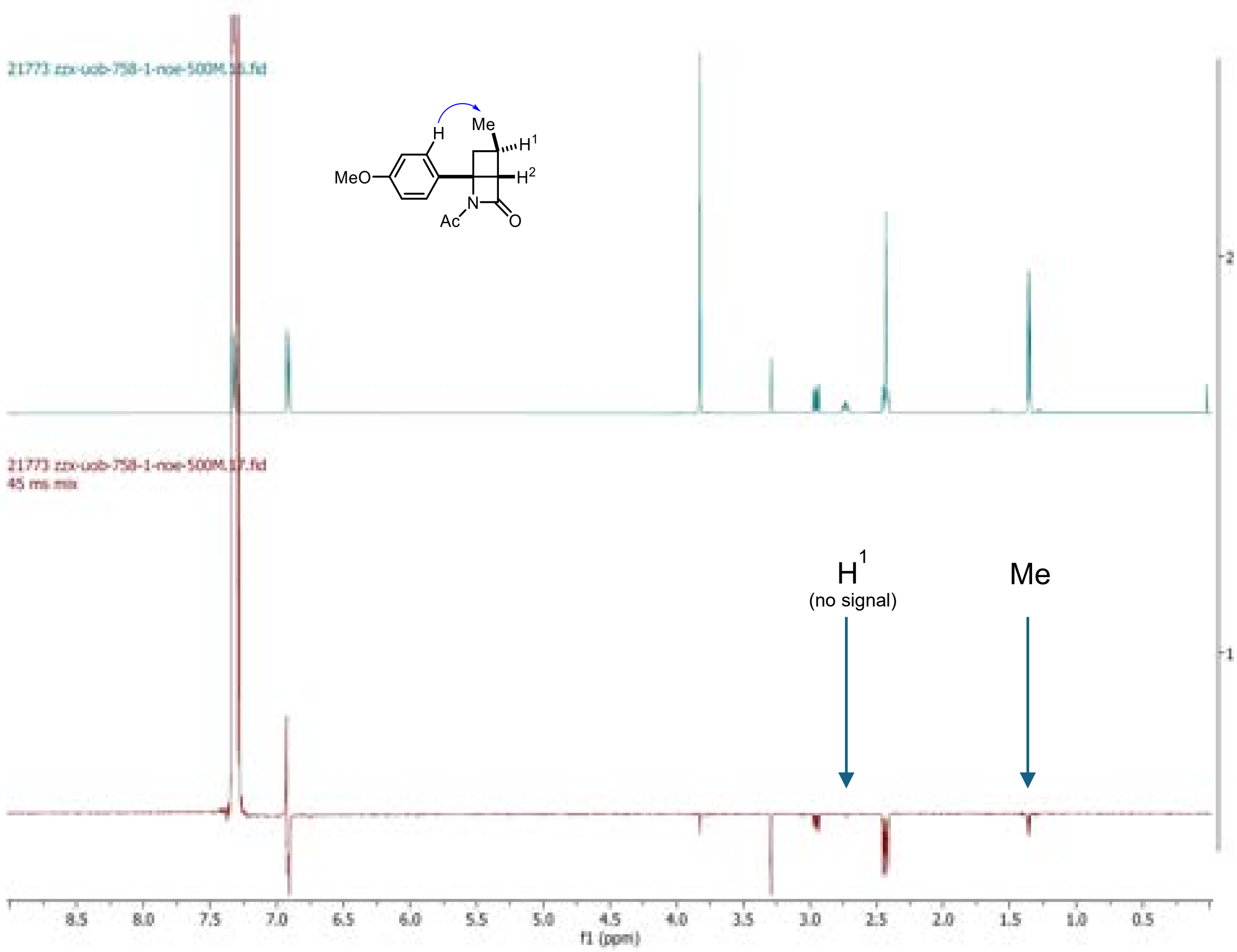

(±)-(1*S*,4*R*,5*R*)-2-acetyl-1-(4-methoxyphenyl)-5-methyl-2-azabicyclo[2.1.1]hexan-3-one (5am)

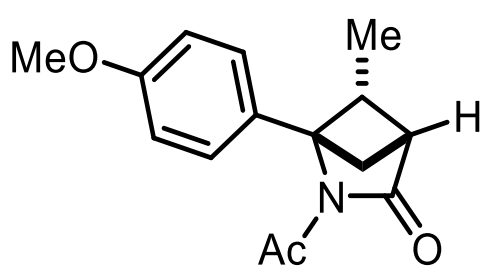

M.P.: 106-108 °C

^{1}H NMR (500 MHz, $CDCl_3$) δ 7.17 (d, *J* = 8.7 Hz, 2H), 6.88 (d, *J* = 8.7 Hz, 2H), 3.80 (s, 3H), 2.94 (qd, *J* = 6.4, 2.8 Hz, 1H), 2.89 (t, *J* = 2.7 Hz, 1H), 2.64 (d, *J* = 7.4 Hz, 1H), 2.43 (s, 3H), 2.39 (dd, *J* = 7.3, 2.6 Hz, 1H), 1.25 (d, *J* = 6.4 Hz, 3H).

^{13}C NMR (126 MHz, $CDCl_3$) δ 178.4, 170.3, 159.3, 129.4, 127.5, 113.6, 74.5, 56.6, 55.3, 49.3, 48.3, 26.0, 11.9.

IR (ATR): $\tilde{\nu}$ (cm^{-1}) = 1760, 1694, 1614, 1519, 1462, 1364, 1276, 1177, 1030.

HRMS (ESI^{+}) calcd. for $C_{15}H_{17}NO_3Na^{+}$ $[M+Na]^{+}$: 282.1101, found: 282.1091.

2-Acetyl-1-(3-(cyclopentyloxy)-4-methoxyphenyl)-4-(3-methylbenzyl)-2-azabicyclo[2.2.0] hexan-3-one (6an)

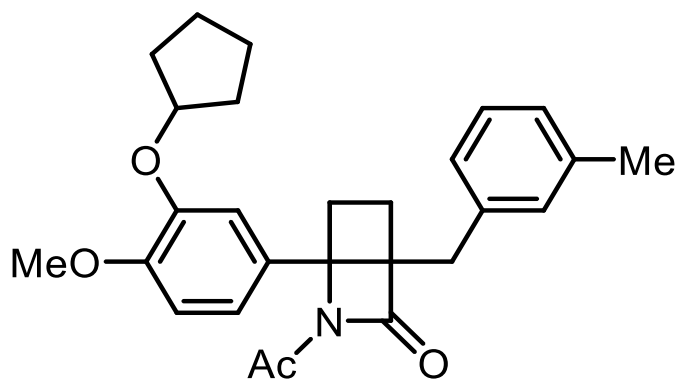

Prepared according to **General Procedure D** using **2an** (1.69 g, 3.90 mmol, 1.00 equiv), CH_3CN (39.0 mL), and [Ir(dF(CF_3)ppy)$_2$(dtbbpy)](PF_6) (43.8 mg, 39.0 µmol, 1.0 mol%). The crude reaction mixture was purified by two successive flash chromatography separations using a Biotage automated flash chromatography system with a gradient elution of Et_2O/pentane (0% to 60%) and pentane/ethyl acetate (0% to 30%), affording **6an** (1.02 g, 2.36 mmol, 60%) as a white solid.[A]

Notes: (**A**) The crude ^{1}H NMR spectrum indicated a >25:1 molar ratio of **6an**/**5an**.

M.P.: 89-91 °C

^{1}H NMR (400 MHz, $CDCl_3$) δ 7.07 (t, J = 7.5 Hz, 1H), 6.98 – 6.94 (m, 1H), 6.89 (d, J = 8.4 Hz, 1H), 6.81 (dd, J = 8.4, 2.2 Hz, 1H), 6.77 (d, J = 7.5 Hz, 1H), 6.68 (d, J = 2.1 Hz, 1H), 6.58 (s, 1H), 4.72-4.66 (m, 1H), 3.86 (s, 3H), 2.90 (ddd, J = 13.5, 11.3, 6.7 Hz, 1H), 2.85 (d, J = 14.9 Hz, 1H), 2.76 (d, J = 14.8 Hz, 1H), 2.50 – 2.42 (m, 4H), 2.34 – 2.22 (m, 2H), 2.20 (s, 3H), 1.91 – 1.81 (m, 6H), 1.66 – 1.55 (m, 2H).

^{13}C NMR (126 MHz, $CDCl_3$) δ 171.2, 167.8, 149.9, 147.7, 137.7, 135.3, 130.6, 128.0, 127.21, 127.15, 126.7, 119.0, 113.2, 111.9, 80.5, 66.3, 64.6, 56.1, 33.6, 32.9, 32.7, 26.0, 24.4, 24.1, 23.5, 21.3.

IR (ATR): $\tilde{\nu}$ (cm^{-1}) = 2955, 1773, 1705, 1516, 1419, 1371, 1276, 1254.

HRMS (ESI$^+$) calcd. for $C_{27}H_{32}NO_4^+$ [M+H]$^+$: 434.2326, found: 434.2311.

2.5 Product Derivatisation Studies

1-(4-Chlorophenyl)-4-methyl-2-azabicyclo[2.1.1]hexan-3-one (3c')

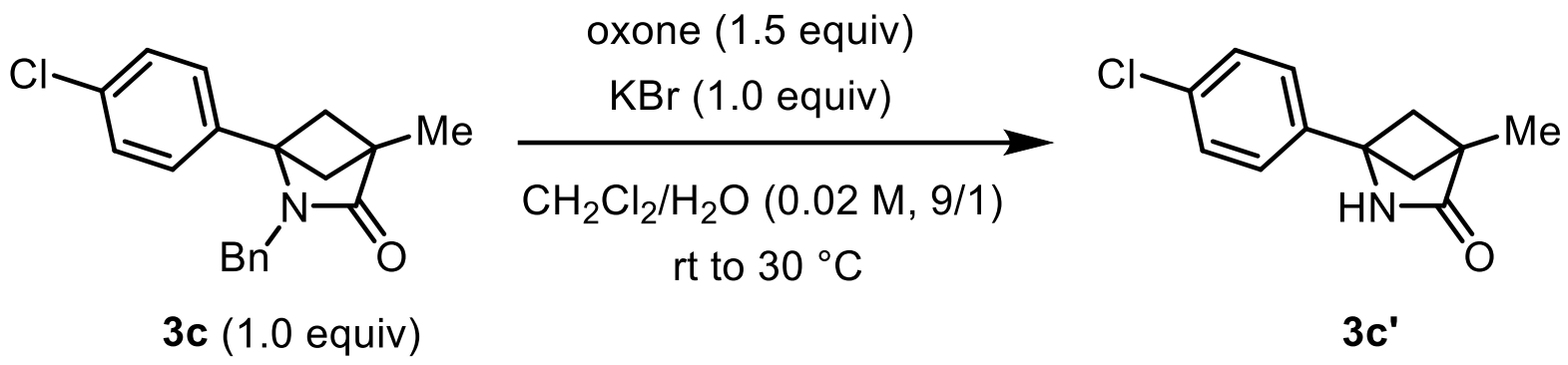

An oven-dried 50 mL round-bottom flask charged with **3c** (62 mg, 0.20 mmol, 1.0 equiv), KBr (33.8 mg, 0.20 mmol, 1.0 equiv) and oxone (92 mg, 0.30 mmol, 1.5 equiv) were sealed and subjected to three cycles of evacuation and nitrogen backfilling. CH_2Cl_2 (9.0 mL) and H_2O (1.0 mL) were added at room temperature, and the mixture was stirred at 30 ℃ for 7 h. The reaction was quenched with sat. aq. $NaHCO_3$ solution (50 mL) and extracted with CH_2Cl_2 (3×30 mL). The combined organic layers were dried over anhydrous $MgSO_4$, filtered, and concentrated under reduced pressure. The crude reaction mixture was purified using a Biotage automated flash chromatography system with a gradient elution of ethyl acetate/pentane (0% to 60%), affording **3c'** (35 mg, 0.16 mmol, 79%) as a white solid.

M.P.: 149-151 ℃

^{1}H NMR (500 MHz, $CDCl_3$) δ 7.35 (d, J = 8.5 Hz, 2H), 7.24 (d, J = 8.5 Hz, 2H), 5.69 (s, 1H), 2.69 (dd, J = 3.9, 2.0 Hz, 2H), 2.49 (d, J = 4.9 Hz, 2H), 1.33 (s, 3H).

^{13}C NMR (126 MHz, $CDCl_3$) δ 185.8, 135.4, 134.4, 129.1, 127.5, 63.4, 59.4, 50.6, 13.3.

IR (ATR): $\tilde{\nu}$ (cm^{-1}) = 3186, 1720, 1600, 1496, 1400, 1378, 1245, 1129, 1088.

HRMS (ESI$^+$) calcd. for $C_{12}H_{13}ClNO^+$ $[M+H]^+$: 222.0680, found: 222.0675.

Methyl 3-oxo-4-phenyl-2-azabicyclo[2.1.1]hexane-1-carboxylate (8a')

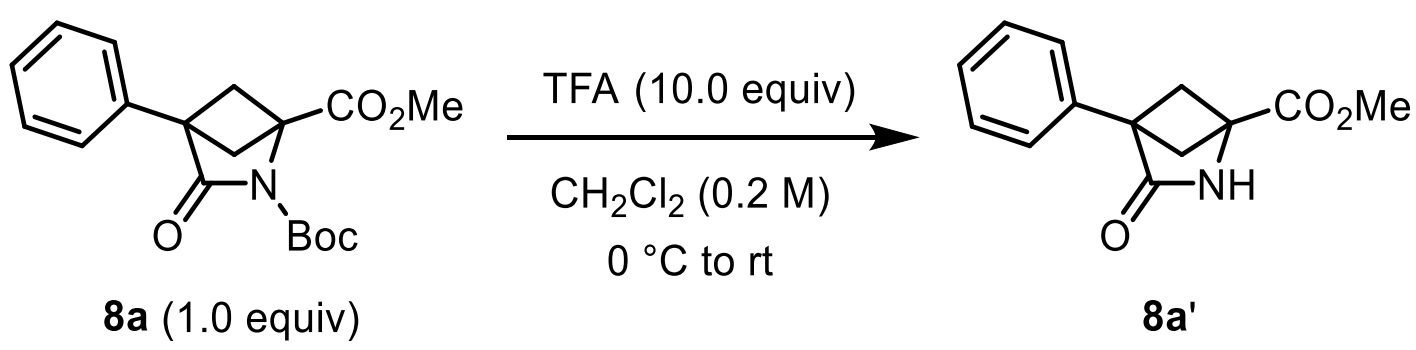

To an oven-dried 100 mL round-bottom flask containing a solution of **8a** (1.32 g, 4.00 mmol, 1.00 equiv) in CH_2Cl_2 (20.0 mL, 0.2 M), was added TFA (3.00 mL, 40.0 mmol, 10.0 equiv) at 0 °C. The mixture was then stirred at room temperature for 1 h before being concentrated *in vacuo* and diluted with ethyl acetate (50 mL). Then, the mixture was basified to pH 10-11 using 1 M aq. NaOH solution and the aqueous phase were extracted with ethyl acetate (2×50 mL). The combined organic layers were dried over anhydrous $MgSO_4$, filtered, and concentrated under reduced pressure. The crude

reaction mixture was purified using a Biotage automated flash chromatography system with a gradient elution of ethyl acetate/pentane (0% to 100%), affording **8a'** (561 mg, 2.43 mmol, 61%) as a white solid.

M.P.: 116-118 °C

^{1}H NMR (500 MHz, $CDCl_3$) δ 7.40 – 7.35 (m, 2H), 7.33 – 7.27 (m, 3H), 6.38 (s, 1H), 3.82 (s, 3H), 3.05 – 2.97 (m, 4H).

^{13}C NMR (126 MHz, $CDCl_3$) δ 180.8, 167.6, 134.9, 128.5, 127.8, 126.9, 60.3, 58.7, 58.0, 52.7.

IR (ATR): $\tilde{\nu}$ (cm^{-1}) = 1731, 1438, 1293, 1266, 1209, 1154, 1140, 1101.

HRMS (ESI^+) calcd. for $C_{13}H_{14}NO_3^+$ $[M+H]^+$: 232.0968, found: 232.0957.

Dimethyl 1-((tert-butoxycarbonyl)amino)-3-phenylcyclobutane-1,3-dicarboxylate (8a")

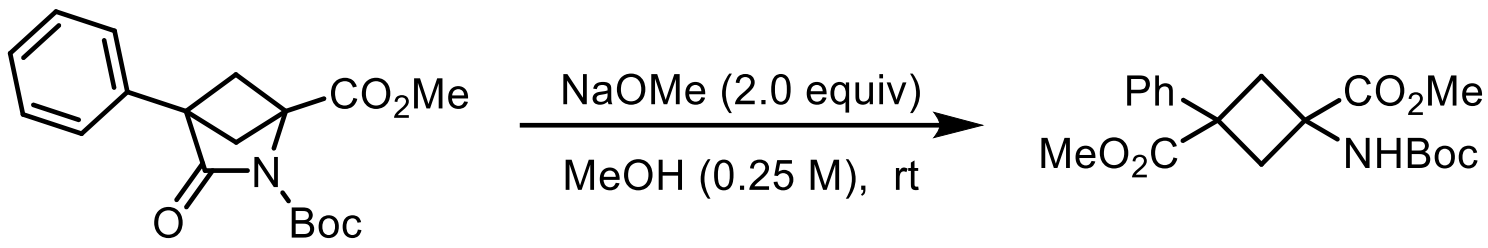

8a (1.0 equiv) **8a"**

An oven-dried 25 mL round-bottom flask charged with **8a** (180 mg, 0.54 mmol, 1.00 equiv) was sealed and subjected to three cycles of evacuation and nitrogen backfilling. Sodium methoxide solution (0.5 M in MeOH, 2.17 mL, 1.08 mmol, 2.00 equiv) was added dropwise at room temperature, and the mixture was stirred at this temperature for 1 h. Then the reaction was quenched with brine (50 mL) and extracted with CH_2Cl_2 (3 × 30 mL). The combined organic layers were dried over anhydrous $MgSO_4$, filtered, and concentrated under reduced pressure. The crude reaction mixture was purified using a Biotage automated flash chromatography system with a gradient elution of ethyl acetate/pentane (0% to 35%), affording **8a"** (186 mg, 0.51 mmol, 95%) as a white solid.

M.P.: 153-155 °C

^{1}H NMR (500 MHz, $CDCl_3$) δ 7.33 – 7.29 (m, 2H), 7.27 – 7.20 (m, 3H), 5.30 (s, 1H), 3.65 (s, 3H), 3.62 (s, 3H), 3.11 (s, 4H), 1.43 (s, 9H).

^{13}C NMR (126 MHz, $CDCl_3$) δ 175.8, 173.0, 155.1, 142.2, 128.5, 127.1, 126.0, 80.2, 55.0, 52.7, 52.5, 47.7, 41.2, 28.3.

IR (ATR): $\tilde{\nu}$ (cm^{-1}) = 1721, 1495, 1448, 1367, 1275, 1260, 1215, 1158, 1096.

HRMS (ESI^+) calcd. for $C_{19}H_{25}NO_6Na^+$ $[M+Na]^+$: 386.1574, found: 386.1582.

(±)-Methyl (1*R*,2*R*)-2-((tert-butoxycarbonyl)amino)-2-(4-methoxyphenyl)-1methylcyclobutane -1-carboxylate (10)

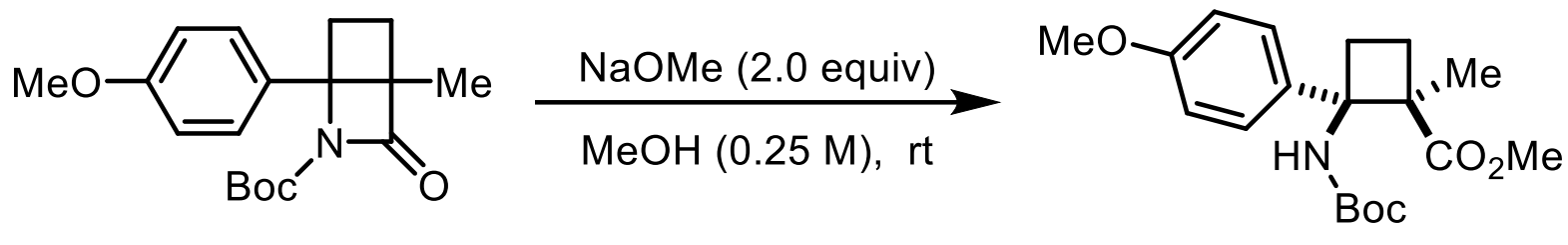

6ab (1.0 equiv) **10**

An oven-dried 50 mL round-bottom flask charged with **6ab** (160 mg, 0.50 mmol, 1.00 equiv) was sealed and subjected to three cycles of evacuation and nitrogen backfilling. Sodium methoxide solution (0.5 M in MeOH, 2.0 mL, 1.0 mmol, 2.0 equiv) was added dropwise at room temperature, and the mixture was stirred at this temperature for 2 h. Then, the reaction was quenched with brine (50 mL) and extracted with CH_2Cl_2 (3 × 30 mL). The combined organic layers were dried over anhydrous $MgSO_4$, filtered, and concentrated under reduced pressure. The crude reaction mixture was purified using a Biotage automated flash chromatography system with a gradient elution of ethyl acetate/pentane (0% to 35%), affording **10** (162 mg, 0.46 mmol, 93%) as a colourless oil.

^{1}H NMR (500 MHz, $CDCl_3$) δ 7.31 (d, *J* = 8.8 Hz, 2H), 6.82 (d, *J* = 8.8 Hz, 2H), 6.04 (br. s, 1H), 3.76 (s, 3H), 3.75 (s, 3H), 2.72 (dt, *J* = 11.6, 8.7 Hz, 1H), 2.63 (dt, *J* = 10.9, 8.9 Hz, 1H), 2.50 (br. s, 1H), 1.69 (ddd, *J* = 11.0, 8.5, 3.4 Hz, 1H), 1.25 (s, 9H), 0.94 (s, 3H).

^{13}C NMR (126 MHz, $CDCl_3$) δ 176.3, 158.3, 154.7, 134.3, 128.1, 113.2, 79.3, 64.0, 55.2, 52.1, 50.5, 28.3, 27.2, 24.8, 22.3.

IR (ATR): $\tilde{\nu}$ (cm^{-1}) = 1714, 1612, 1513, 1462, 1366, 1275, 1260, 1153, 1120, 1062.

HRMS (ESI$^+$) calcd. for $C_{19}H_{27}NO_5Na^+$ [M+Na]$^+$: 372.1781, found: 372.1784.

***tert*-Butyl 1-(4-methoxyphenyl)-4-methyl-2-azabicyclo[2.2.0]hexane-2-carboxylate (11)**

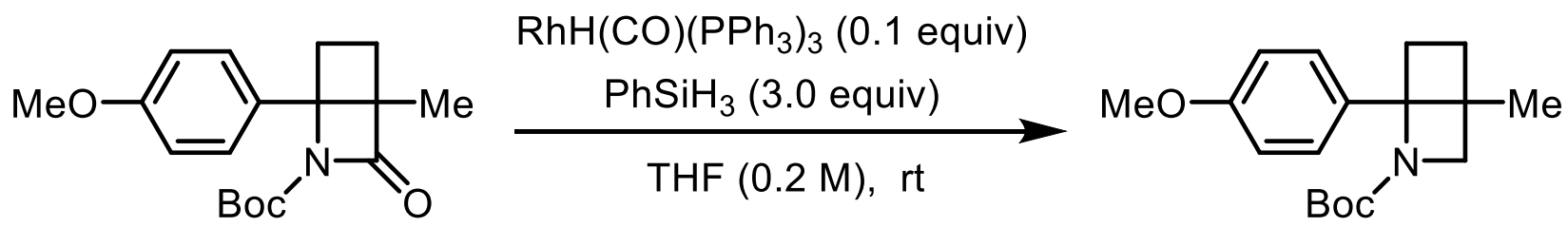

6ab (1.0 equiv) **11**

To an oven-dried 25 mL round-bottom flask equipped with a magnetic stir bar was added **6ab** (160 mg, 0.50 mmol, 1.0 equiv) and $RhH(CO)(PPh_3)_3$ (46.0 mg, 50.0 μmol, 0.10 equiv) under an argon atmosphere in a glovebox. Anhydrous THF (2.5 mL, 0.2 M) and phenylsilane ($PhSiH_3$, 0.18 mL, 1.5 mmol, 3.0 equiv) were added and the vessel was removed from the glovebox. The reaction was stirred at room temperature overnight under a nitrogen atmosphere. After completion, the crude reaction mixture was concentrated under reduced pressure and purified by two successive flash chromatography separations using a Biotage automated flash chromatography system with a gradient

elution of ethyl acetate/pentane (0% to 30%) and ethyl acetate/CH_2Cl_2 (0% to 10%). The desired product **11** (64 mg, 0.21 mmol, 42%) was obtained as a colourless oil.[A]

Notes: (**A**) Two sets of peaks were observed in the ^{1}H and ^{13}C NMR spectra due to carbamate rotamers.

^{1}H NMR (500 MHz, $CDCl_3$) δ 7.25 – 7.20 (m, 2H, *overlapping*), 6.91 – 6.86 (m, 2H, *overlapping*), 4.13 (d, *J* = 8.5 Hz, 1H, *major*), 4.10 (d, *J* = 8.5 Hz, 1H, *minor*), 3.94-3.87 (m, 1H, *overlapping*), 3.81 (s, 3H, *major*), 3.79 (s, 3H, *minor*), 2.86-2.76 (m, 1H, *overlapping*), 2.72 – 2.64 (m, 1H, *minor*), 2.54 (ddd, *J* = 13.2, 9.0, 6.0 Hz, 1H, *major*), 2.41-2.28 (m, 1H, *overlapping*), 2.12-1.97 (m, 1H, *overlapping*), 1.47 (s, 9H, *minor*), 1.26 (s, 9H, *major*), 0.93 (s, 3H, *major*), 0.91 (s, 3H, *minor*).

^{13}C NMR (126 MHz, $CDCl_3$) δ 158.62 (*minor*), 158.58 (*major*), 155.9 (*major*), 155.1 (*minor*), 131.5 (*major*), 131.2 (*minor*), 127.6 (*major*), 127.4 (*minor*), 113.8 (*minor*), 113.4 (*major*), 79.5 (*minor*), 79.3 (*major*), 75.2 (*major*), 74.7 (*minor*), 62.7 (*minor*), 61.1 (*major*), 55.3 (*overlapping*), 43.0 (*major*), 42.8 (*minor*), 30.8 (*minor*), 30.6 (*major*), 28.9 (*overlapping*), 28.7 (*minor*), 28.5 (*major*), 19.3 (*overlapping*).

IR (ATR): $\tilde{\nu}$ (cm^{-1}) = 1693, 1613, 1513, 1455, 1384, 1364, 1275, 1260, 1176, 1147, 1035.

HRMS (ESI$^+$) calcd. for $C_{18}H_{25}NO_3Na^+$ [M+Na]$^+$: 326.1727, found: 326.1732.

1-(4-Methoxyphenyl)-4-methyl-2-azabicyclo[2.2.0]hexan-3-one (12)

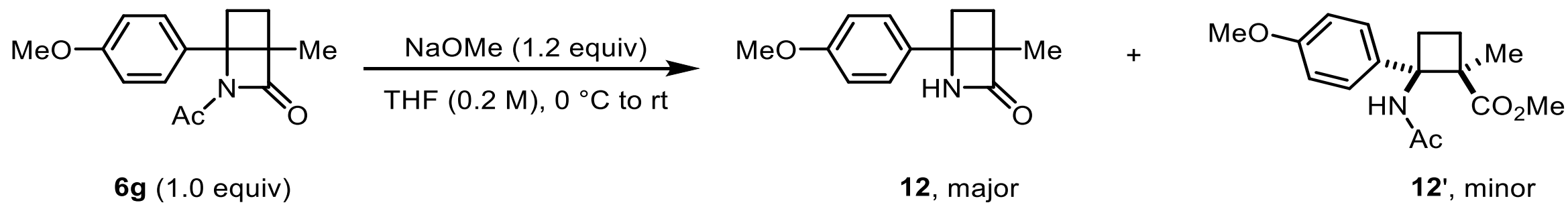

An oven-dried 50 mL round-bottom flask charged with **6g** (430 mg, 1.66 mmol, 1.00 equiv) was sealed and subjected to three cycles of evacuation and nitrogen backfilling. Anhydrous THF (8.30 mL, 0.2 M) was then added, and the reaction mixture was cooled to 0 °C. Sodium methoxide solution (0.5 M in MeOH, 4.0 mL, 2.0 mmol, 1.2 equiv) was added dropwise at 0°C, and the mixture was stirred at this temperature for 45 minutes. Then, the reaction was allowed to warm to room temperature and quenched with brine (60 mL) and extracted with CH_2Cl_2 (3 × 40 mL). The combined organic layers were dried over anhydrous $MgSO_4$, filtered, and concentrated under reduced pressure. The crude reaction mixture was purified using a Biotage automated flash chromatography system with a gradient elution of ethyl acetate/pentane (0% to 60%), affording **12** (231 mg, 1.06 mmol, 64%) as a white solid and **12'** (106 mg, 0.36 mmol, 22%) as a colourless oil.

M.P.: 126-128 °C

^{1}H NMR (500 MHz, $CDCl_3$) δ 7.27 (d, *J* = 8.9 Hz, 2H), 6.92 (d, *J* = 8.8 Hz, 2H), 6.26 (s, 1H), 3.82 (s, 3H), 2.98 – 2.87 (m, 1H), 2.38 – 2.35 (m, 1H), 2.34 – 2.30 (m, 1H), 2.05 – 1.99 (m, 1H), 1.04 (s, 3H).

^{13}C NMR (126 MHz, $CDCl_3$) δ 174.6, 159.2, 129.5, 127.7, 114.1, 63.4, 62.6, 55.4, 29.6, 24.2, 13.6.

IR (ATR): $\tilde{\nu}$ (cm^{-1}) = 1741, 1612, 1515, 1463, 1276, 1260, 1179, 1032.

HRMS (ESI^+) calcd. for $C_{13}H_{16}NO_2^+$ $[M+H]^+$: 218.1176, found: 218.1179.

(±)-Methyl (1*R*,2*R*)-2-acetamido-2-(4-methoxyphenyl)-1-methylcyclobutane-1-carboxylate (12')

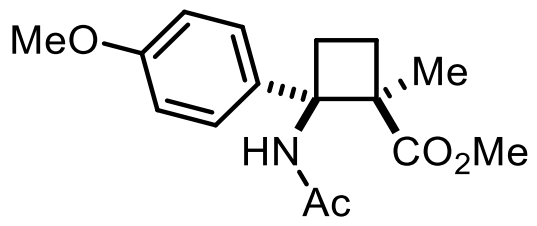

^{1}H NMR (500 MHz, $CDCl_3$) δ 7.34 – 7.30 (m, 3H), 6.83 (d, *J* = 8.8 Hz, 2H), 3.79 (s, 3H), 3.77 (s, 3H), 2.83 – 2.74 (m, 1H), 2.67 – 2.55 (m, 2H), 1.88 (s, 3H), 1.78 – 1.71 (m, 1H), 1.00 (s, 3H).

^{13}C NMR (126 MHz, $CDCl_3$) δ 177.0, 169.5, 158.4, 134.0, 128.2, 113.4, 64.4, 55.3, 52.3, 50.2, 28.0, 24.9, 24.4, 22.3.

IR (ATR): $\tilde{\nu}$ (cm^{-1}) = 1716, 1682, 1611, 1513, 1460, 1369, 1275, 1260, 1181, 1148, 1035.

HRMS (ESI^+) calcd. for $C_{16}H_{21}NO_4Na^+$ $[M+Na]^+$: 314.1363, found: 314.1367.

2-Acetyl-4-methyl-3-oxo-2-azabicyclo[2.2.0]hexane-1-carboxylic acid (13)

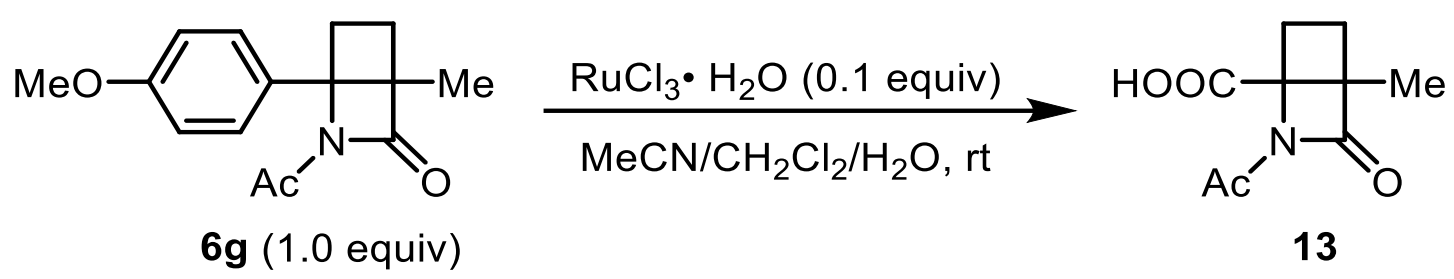

In an oven-dried 250 mL round-bottom flask, **6g** (990 mg, 3.82 mmol, 1.00 equiv) was dissolved in anhydrous MeCN (20 mL), CH_2Cl_2 (20 mL) and H_2O (30 mL) at room temperature. Ruthenium(III) chloride hydrate (86 mg, 0.38 mmol, 0.10 equiv) was then added, followed by $NaIO_4$ (16.4 g, 76.4 mmol, 20.0 equiv). The reaction mixture was stirred at room temperature overnight until completion of the reaction (TLC). The reaction was quenched with 1 M aqueous HCl solution (80 mL) and extracted with ethyl acetate three times (3 × 50 mL). The combined organic layers were dried over anhydrous $MgSO_4$, filtered, and concentrated under reduced pressure. The crude reaction mixture was purified by two successive flash chromatography separations using a Biotage automated flash chromatography system with a gradient elution of ethyl acetate/pentane (0% to 100%) and ethyl acetate/CH_2Cl_2 (0% to 50%), affording **13** (625 mg, 3.17 mmol, 83%) as a white solid.

M.P.: 138-140 °C

^{1}H NMR (500 MHz, $CDCl_3$) δ 11.42 (s, 1H), 3.06 – 2.94 (m, 1H), 2.49-2.37 (m, 4H), 2.29 – 2.16 (m, 2H), 1.38 (s, 3H).

^{13}C NMR (126 MHz, $CDCl_3$) δ 172.3, 168.8, 167.6, 61.8, 61.7, 26.2, 23.8, 23.3, 13.2.

IR (ATR): $\tilde{\nu}$ (cm^{-1}) = 1783, 1708, 1376, 1318, 1276, 1261, 1194, 1106.

HRMS (Nanospray) calcd. for $C_9H_{10}NO_4$ [M-H]$^-$: 196.0615, found: 196.0614.

1-(4-Methoxyphenyl)-4-methyl-3-oxo-N-phenyl-2-azabicyclo[2.2.0]hexane-2-carboxamide (14)

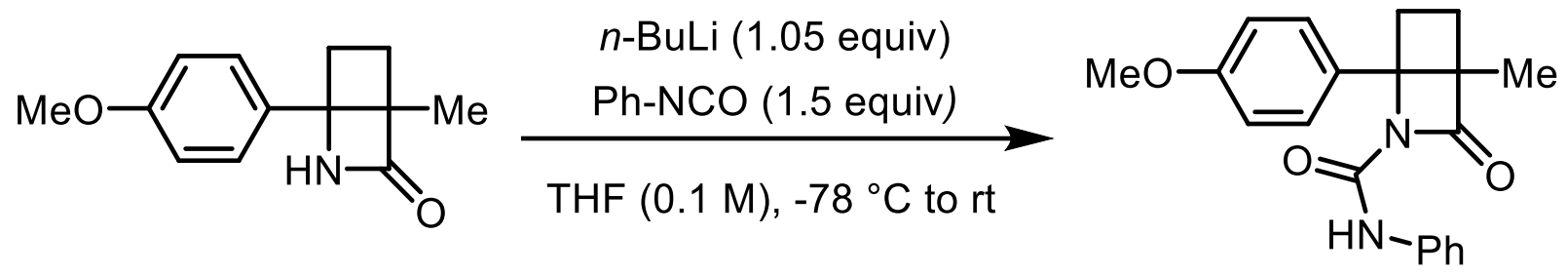

12 (1.0 equiv) **14**

An oven-dried 25 mL round-bottom flask charged with **12** (44 mg, 0.20 mmol, 1.0 equiv) was sealed and subjected to three cycles of evacuation and nitrogen backfilling. Anhydrous THF (2.0 mL, 0.1 M) was then added, and the reaction mixture was cooled to –78 °C. *n*-BuLi (2.5 M in hexane, 84 µL, 0.21 mmol, 1.05 equiv) was added dropwise at –78 °C, and the mixture was stirred at this temperature for 5 minutes. Phenyl isocyanate (32 µL, 0.30 mmol, 1.5 equiv) was subsequently added at –78 °C, and the reaction was allowed to warm to room temperature over 1 hour. The reaction was then quenched with brine (50 mL) and extracted with ethyl acetate (3 × 30 mL). The combined organic layers were dried over anhydrous $MgSO_4$, filtered, and concentrated under reduced pressure. The crude reaction mixture was purified using a Biotage automated flash chromatography system with a gradient elution of ethyl acetate/pentane (0% to 30%), affording **14** (60 mg, 0.18 mmol, 89%) as a white solid.

M.P.: 124-126 °C

^{1}H NMR (500 MHz, $CDCl_3$) δ 8.80 (s, 1H), 7.57 – 7.50 (m, 2H), 7.34 – 7.27 (m, 4H), 7.10 (tt, J = 7.2, 1.2 Hz, 1H), 6.94 (d, J = 8.9 Hz, 2H), 3.81 (s, 3H), 3.05 (ddd, J = 13.7, 11.3, 6.3 Hz, 1H), 2.68 (ddd, J = 13.8, 8.7, 5.9 Hz, 1H), 2.51 (ddd, J = 13.0, 8.7, 6.3 Hz, 1H), 2.22 (ddd, J = 13.1, 11.3, 5.8 Hz, 1H), 1.15 (s, 3H).

^{13}C NMR (126 MHz, $CDCl_3$) δ 173.9, 159.4, 147.8, 137.4, 129.2, 127.6, 127.1, 124.2, 119.6, 114.2, 67.2, 61.6, 55.4, 26.8, 26.2, 13.1.

IR (ATR): $\tilde{\nu}$ (cm^{-1}) = 1752, 1710, 1602, 1546, 1446, 1252, 1180.

HRMS (ESI$^+$) calcd. for $C_{20}H_{21}N_2O_3^+$ [M+H]$^+$: 337.1547, found: 337.1546.

1-(4-Methoxyphenyl)-4-methyl-3-oxo-N-phenyl-2-azabicyclo[2.2.0]hexane-2-carbothioamide (15)

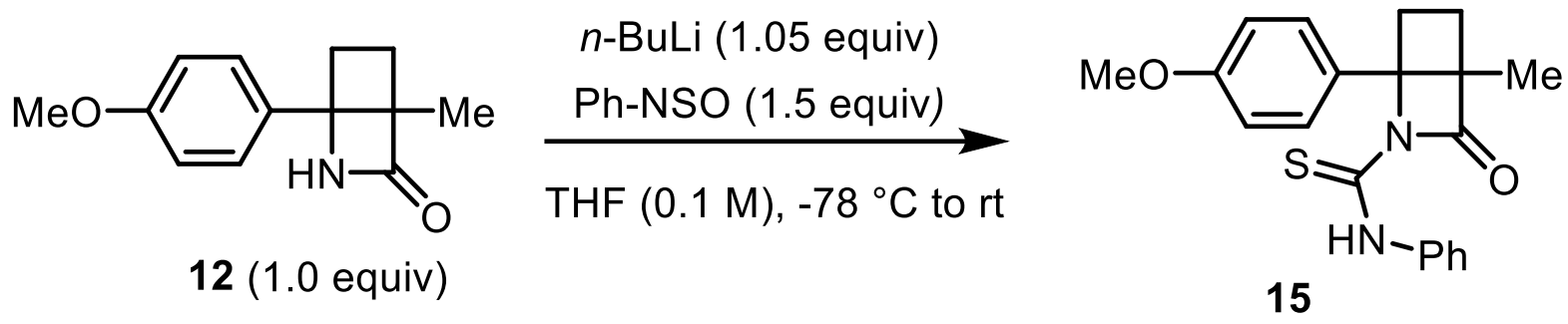

An oven-dried 25 mL round-bottom flask charged with **12** (44 mg, 0.20 mmol, 1.0 equiv) was sealed and subjected to three cycles of evacuation and nitrogen backfilling. Anhydrous THF (2.0 mL, 0.1 M) was then added, and the reaction mixture was cooled to –78 °C. *n*-BuLi (2.5 M in hexane, 84 µL, 0.21 mmol, 1.05 equiv) was added dropwise at –78 °C, and the mixture was stirred at this temperature for 5 minutes. Phenyl isothiocyanate (36 µL, 0.30 mmol, 1.5 equiv) was subsequently added at –78 °C, and the reaction was allowed to warm to room temperature over 1 hour. The reaction was then quenched with brine (50 mL) and extracted with ethyl acetate (3×30 mL). The combined organic layers were dried over anhydrous $MgSO_4$, filtered, and concentrated under reduced pressure. The crude reaction mixture was purified using a Biotage automated flash chromatography system with a gradient elution of ethyl acetate/pentane (0% to 35%), affording **15** (55 mg, 0.16 mmol, 78%) as a white solid.

M.P.: 70-72 ℃

^{1}H NMR (500 MHz, $CDCl_3$) δ 10.58 (s, 1H), 7.75 – 7.72 (m, 2H), 7.41 – 7.37 (m, 2H), 7.29 (d, *J* = 8.9 Hz, 2H), 7.25 – 7.21 (m, 1H), 6.94 (d, *J* = 8.8 Hz, 2H), 3.82 (s, 3H), 3.09 (ddd, *J* = 14.0, 11.4, 6.4 Hz, 1H), 2.92 (ddd, *J* = 14.0, 8.8, 6.0 Hz, 1H), 2.58 (ddd, *J* = 13.0, 8.8, 6.4 Hz, 1H), 2.27 (ddd, *J* = 13.1, 11.3, 6.0 Hz, 1H), 1.12 (s, 3H).

^{13}C NMR (126 MHz, $CDCl_3$) δ 174.9, 172.5, 159.2, 137.6, 128.9, 127.8, 126.7, 126.4, 123.5, 114.0, 69.6, 60.6, 55.3, 27.3, 25.6, 12.9.

IR (ATR): $\tilde{\nu}$ (cm^{-1}) = 1741, 1601, 1515, 1374, 1312, 1250, 1227, 1178, 1031.

HRMS (ESI$^+$) calcd. for $C_{20}H_{21}N_2O_2S^+$ $[M+H]^+$: 353.1318, found: 353.1317.

1-(4-Methoxyphenyl)-4-methyl-2-(methylsulfonyl)-2-azabicyclo[2.2.0]hexan-3-one (16)

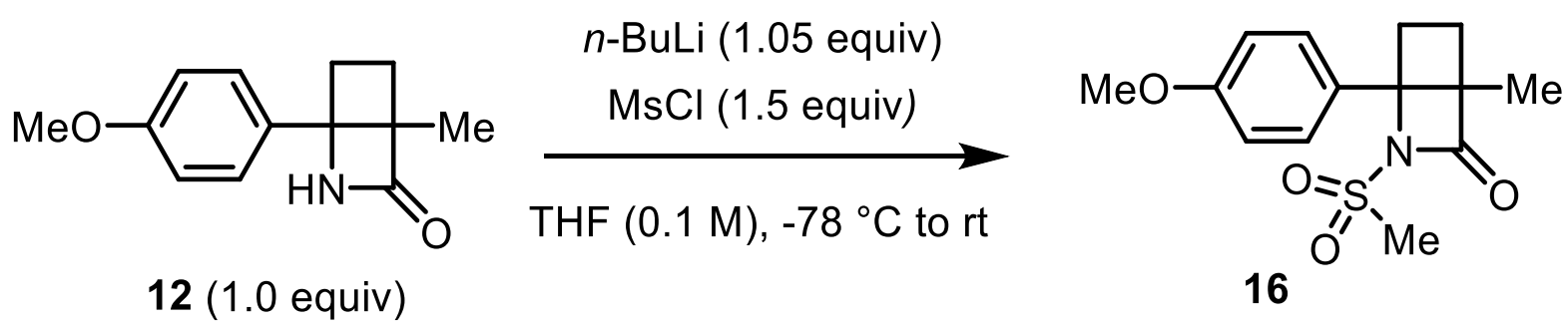

An oven-dried 25 mL round-bottom flask charged with **12** (44 mg, 0.20 mmol, 1.0 equiv) was sealed and subjected to three cycles of evacuation and nitrogen backfilling. Anhydrous THF (2.0 mL, 0.1 M) was then added, and the reaction mixture was cooled to –78 °C. *n*-BuLi (2.5 M in hexane, 84 µL, 0.21

mmol, 1.05 equiv) was added dropwise at –78 °C, and the mixture was stirred at this temperature for 5 minutes. Methanesulfonyl chloride (23 μL, 0.30 mmol, 1.5 equiv) was subsequently added at –78 °C, and the reaction was allowed to warm to room temperature over 1 hour. The reaction was then quenched with brine (50 mL) and extracted with ethyl acetate (3 × 30 mL). The combined organic layers were dried over anhydrous $MgSO_4$, filtered, and concentrated under reduced pressure. The crude reaction mixture was purified using a Biotage automated flash chromatography system with a gradient elution of ethyl acetate/pentane (0% to 50%), affording **16** (32 mg, 0.11 mmol, 54%) as a colourless oil.

^{1}H NMR (500 MHz, $CDCl_3$) δ 7.29 (d, *J* = 8.8 Hz, 2H), 6.95 (d, *J* = 8.8 Hz, 2H), 3.82 (s, 3H), 3.08 (ddd, *J* = 13.9, 11.4, 6.8 Hz, 1H), 3.02 (s, 3H), 2.87 (ddd, *J* = 14.0, 8.7, 5.4 Hz, 1H), 2.58 (ddd, *J* = 13.1, 8.7, 6.8 Hz, 1H), 2.16 (ddd, *J* = 13.1, 11.4, 5.4 Hz, 1H), 1.20 (s, 3H).

^{13}C NMR (126 MHz, $CDCl_3$) δ 170.8, 159.9, 128.2, 126.3, 114.4, 70.9, 62.9, 55.4, 43.3, 26.6, 25.5, 13.3.

IR (ATR): $\tilde{\nu}$ (cm^{-1}) = 1780, 1676, 1601, 1513, 1349, 1245, 1151, 1028.

HRMS (ESI^+) calcd. for $C_{14}H_{17}NO_4SNa^+$ $[M+Na]^+$: 318.0770, found: 318.0770.

2-Benzyl-1-(4-methoxyphenyl)-4-methyl-2-azabicyclo[2.2.0]hexan-3-one (17)

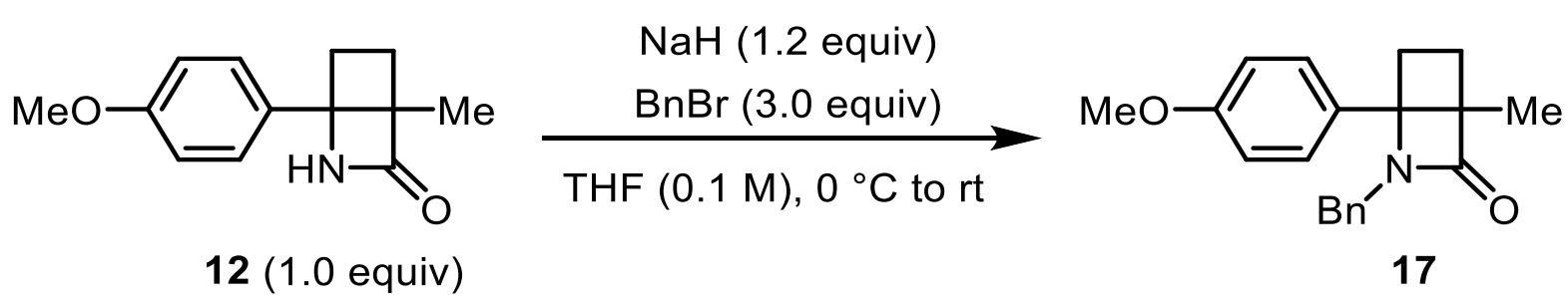

An oven-dried 25 mL round-bottom flask charged with **12** (44 mg, 0.20 mmol, 1.0 equiv) was sealed and subjected to three cycles of evacuation and nitrogen backfilling. Anhydrous THF (2.0 mL, 0.1 M) was then added, and the reaction mixture was cooled to 0 °C. NaH (60% dispersion in mineral oil, 9.6 mg, 0.24 mmol, 1.2 equiv) was added at 0 °C, and the mixture was stirred at this temperature for 30 minutes. Benzyl bromide (71 μL, 0.60 mmol, 3.0 equiv) was subsequently added at 0 °C, and the reaction was allowed to warm to room temperature over 1 hour. The reaction was then quenched with brine (50 mL) and extracted with ethyl acetate three times (3 × 30 mL). The combined organic layers were dried over anhydrous $MgSO_4$, filtered, and concentrated under reduced pressure. The crude reaction mixture was purified using a Biotage automated flash chromatography system with a gradient elution of ethyl acetate/pentane (0% to 40%), affording **17** (50 mg, 0.16 mmol, 81%) as a colourless oil.

^{1}H NMR (500 MHz, $CDCl_3$) δ 7.27 – 7.18 (m, 5H), 7.10 (d, *J* = 8.8 Hz, 2H), 6.87 (d, *J* = 8.8 Hz, 2H), 4.59 (d, *J* = 14.8 Hz, 1H), 4.02 (d, *J* = 14.8 Hz, 1H), 3.80 (s, 3H), 2.67 (ddd, *J* = 13.0, 11.0, 6.2 Hz,

1H), 2.26 (ddd, J = 12.5, 8.6, 6.2 Hz, 1H), 1.88 (ddd, J = 12.6, 11.0, 5.5 Hz, 1H), 1.78 (ddd, J = 13.0, 8.6, 5.6 Hz, 1H), 1.06 (s, 3H).

^{13}C NMR (126 MHz, $CDCl_3$) δ 173.5, 159.2, 136.6, 129.0, 128.7, 128.2, 127.7, 127.6, 114.1, 67.3, 62.5, 55.4, 44.2, 25.4, 24.8, 13.3.

IR (ATR): $\tilde{\nu}$ (cm^{-1}) = 1733, 1611, 1513, 1454, 1297, 1250, 1177, 1030.

HRMS (ESI$^+$) calcd. for $C_{20}H_{22}NO_2^+$ [M+H]$^+$: 308.1645, found: 308.1642.

2-(2,4-Dinitrophenyl)-1-(4-methoxyphenyl)-4-methyl-2-azabicyclo[2.2.0]hexan-3-one (18)

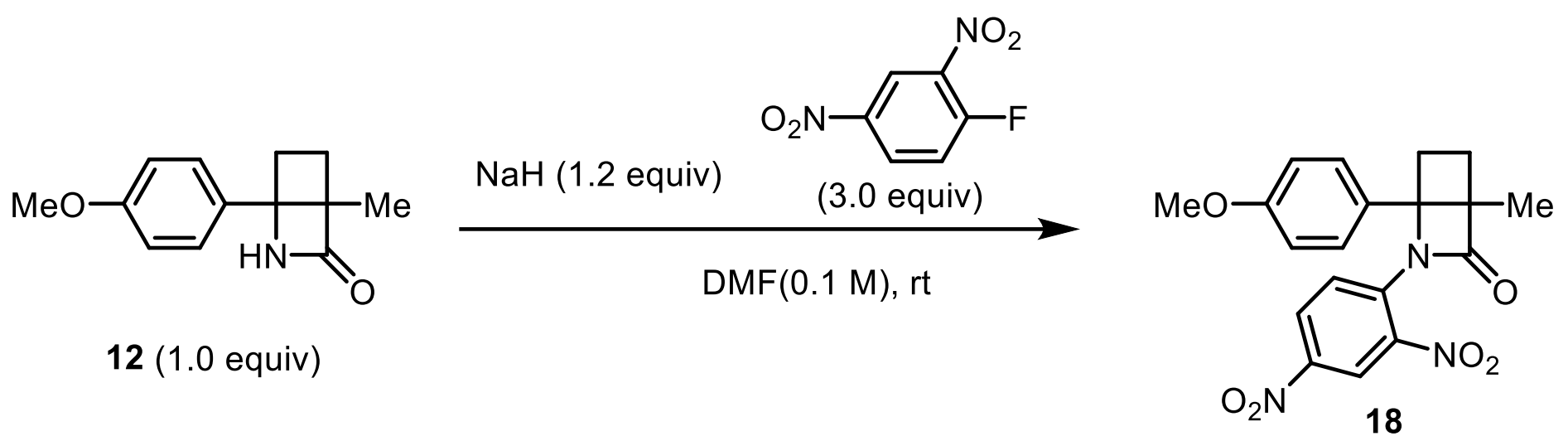

An oven-dried 25 mL round-bottom flask charged with **12** (65 mg, 0.30 mmol, 1.0 equiv) was sealed and subjected to three cycles of evacuation and nitrogen backfilling. Anhydrous DMF (3.0 mL, 0.1 M) was then added at room temperature, followed by the addition of NaH (60% dispersion in mineral oil, 14.4 mg, 0.360 mmol, 1.2 equiv). The mixture was stirred at room temperature for 30 minutes. 1-Fluoro-2,4-dinitrobenzene (113 µL, 0.90 mmol, 3.0 equiv) was then added and the reaction was stirred at room temperature for 1 hour. The reaction was then quenched with brine (50 mL) and extracted with ethyl acetate (3 × 30 mL). The combined organic layers were dried over anhydrous $MgSO_4$, filtered, and concentrated under reduced pressure. The crude reaction mixture was purified by two successive flash chromatography separations using a Biotage automated flash chromatography system with a gradient elution of ethyl acetate/pentane (0% to 50%) and ethyl acetate/CH_2Cl_2 (0% to 10%), affording **18** (88 mg, 0.23 mmol, 77%) as a pale-yellow solid.

M.P.: 140-142 °C

^{1}H NMR (500 MHz, $CDCl_3$) δ 8.67 (d, J = 2.5 Hz, 1H), 8.25 (dd, J = 9.0, 2.5 Hz, 1H), 7.36 (d, J = 8.9 Hz, 2H), 7.04 (d, J = 9.0 Hz, 1H), 6.95 (d, J = 8.8 Hz, 2H), 3.80 (s, 3H), 3.26 (ddd, J = 13.7, 11.4, 5.8 Hz, 1H), 2.66 (ddd, J = 13.6, 8.9, 6.4 Hz, 1H), 2.51 (ddd, J = 13.0, 8.9, 5.7 Hz, 1H), 2.20 (ddd, J = 13.1, 11.3, 6.3 Hz, 1H), 1.15 (s, 3H).

^{13}C NMR (126 MHz, $CDCl_3$) δ 170.0, 160.0, 142.7, 141.9, 133.7, 128.2, 127.9, 125.1, 121.9, 120.1, 114.8, 68.8, 63.0, 55.4, 25.74, 25.66, 13.3.

IR (ATR): $\tilde{\nu}$ (cm^{-1}) = 1762, 1600, 1515, 1334, 1253, 1180, 1137, 1030.

HRMS (ESI^+) calcd. for $C_{19}H_{18}N_3O_6^+$ $[M+H]^+$: 384.1190, found: 384.1190.

2-Acetyl-4-methyl-1-(4-(quinolin-2-yl)piperazine-1-carbonyl)-2-azabicyclo[2.2.0]hexan-3-one (19)

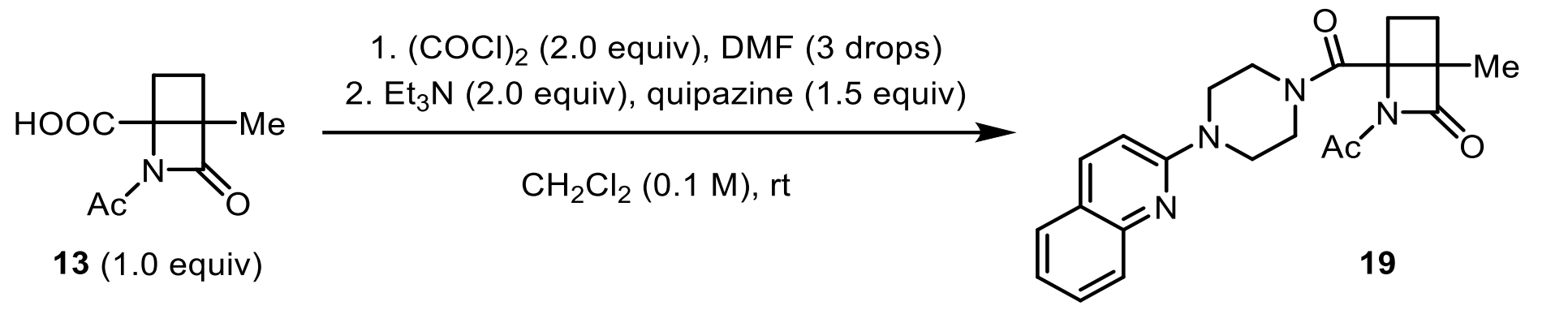

An oven-dried 25 mL round-bottom flask containing **13** (78 mg, 0.40 mmol, 1.0 equiv) was sealed and subjected to three N_2 evacuation/refill cycles before pre-sparged anhydrous CH_2Cl_2 (4.0 mL, 0.1 M) was added. Oxalyl chloride (68 µL, 0.80 mmol, 2.0 equiv) was then added at room temperature, followed by the addition of DMF (3 drops). The reaction mixture was stirred at room temperature for 1 h. The reaction was then concentrated under reduced pressure to afford the crude acyl chloride, which was used directly for the next step without further purification.

The crude acyl chloride was re-dissolved in anhydrous CH_2Cl_2 (4.0 mL, 0.1 M) at room temperature. Et_3N (112 µL, 0.80 mmol, 2.0 equiv) was then added, followed by the addition of quipazine (150 mg, 0.60 mmol, 1.5 equiv). The reaction mixture was stirred at room temperature for 1 h until complete (TLC). The reaction was then quenched with brine (50 mL) and extracted with CH_2Cl_2 (3 × 30 mL). The combined organic extracts were dried over anhydrous $MgSO_4$ and concentrated *in vacuo*. The crude reaction mixture was purified using a Biotage automated flash chromatography system with a gradient elution of ethyl acetate/CH_2Cl_2 (0% to 50%), affording **19** (87 mg, 0.22 mmol, 55%) as a white solid.

M.P.: 189-191 °C

^{1}H NMR (500 MHz, $CDCl_3$) δ 7.91 (d, J = 9.0 Hz, 1H), 7.73 (s, 1H), 7.60 (dd, J = 8.1, 1.5 Hz, 1H), 7.54 (ddd, J = 8.5, 7.0, 1.5 Hz, 1H), 7.24 (ddd, J = 8.0, 7.2, 1.4 Hz, 1H), 6.95 (d, J = 9.1 Hz, 1H), 4.14 – 3.91 (m, 4H), 3.72 – 3.35 (m, 4H), 2.78 (ddd, J = 13.0, 11.4, 7.1 Hz, 1H), 2.51 (ddd, J = 13.0, 8.3, 4.8 Hz, 1H), 2.42 (s, 3H), 2.36 (ddd, J = 12.9, 8.3, 7.0 Hz, 1H), 2.07 (ddd, J = 12.9, 11.4, 4.8 Hz, 1H), 1.48 (s, 3H).

^{13}C NMR (126 MHz, $CDCl_3$) δ 170.3, 168.1, 166.3, 156.9, 147.6, 137.9, 129.8, 127.3, 126.7, 123.2, 122.9, 109.6, 62.1, 45.5, 44.9, 42.6, 27.9, 25.9, 24.1, 14.0.

IR (ATR): $\tilde{\nu}$ (cm^{-1}) = 1781, 1701, 1635, 1603, 1506, 1428, 1376, 1302, 1276, 1230.

HRMS (ESI^+) calcd. for $C_{22}H_{25}N_4O_3^+$ $[M+H]^+$: 393.1921, found: 393.1911.

2-Acetyl-*N*-methoxy-4-methyl-3-oxo-2-azabicyclo[2.2.0]hexane-1-carboxamide (20)

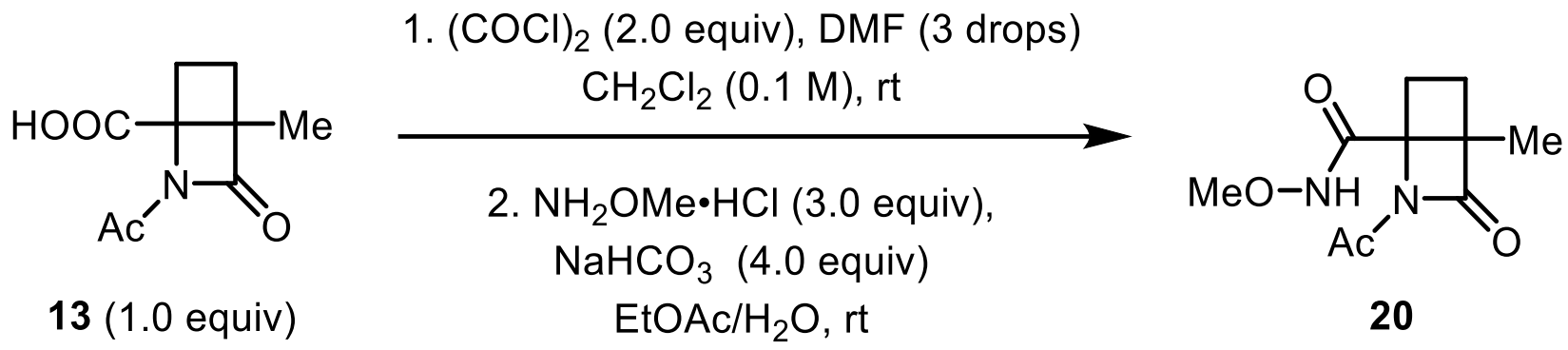

An oven-dried 25 mL round-bottom flask containing **13** (60 mg, 0.30 mmol, 1.0 equiv) was sealed and subjected to three N_2 evacuation/refill cycles before pre-sparged anhydrous CH_2Cl_2 (3.0 mL, 0.1 M) was added. Oxalyl chloride (51 µL, 0.60 mmol, 2.0 equiv) was then added at room temperature, followed by the addition of DMF (3 drops). The reaction mixture was stirred at room temperature for 1 h. The reaction was then concentrated under reduced pressure to afford the crude acyl chloride which was used directly for the next step without further purification.

In another oven-dried 25 mL round-bottom flask, methoxyamine hydrochloride (76 mg, 0.90 mmol, 3.0 equiv) was added to a biphasic mixture of $NaHCO_3$ (101 mg, 1.20 mmol, 4.00 equiv) in EtOAc/H_2O (2:1, 3.0 mL). The mixture was stirred at room temperature for 5 minutes and then transferred to a round-bottom flask containing the crude acyl chloride. The reaction mixture was then stirred at room temperature for 1 h until complete (TLC). The reaction was then quenched with brine (50 mL) and extracted with CH_2Cl_2 (3 × 30 mL). The combined organic extracts were dried over anhydrous $MgSO_4$ and concentrated *in vacuo*. The crude reaction mixture was purified using a Biotage automated flash chromatography system with a gradient elution of ethyl acetate/pentane (0% to 65%), affording **20** (56 mg, 0.25 mmol, 83%) as a white solid.

M.P.: 100-102 °C

^{1}H NMR (500 MHz, $CDCl_3$) δ 9.51 (s, 1H), 3.72 (s, 3H), 2.91 (ddd, *J* = 13.4, 11.3, 6.7 Hz, 1H), 2.45 (s, 3H), 2.37 (ddd, *J* = 12.9, 8.6, 6.7 Hz, 1H), 2.26 (ddd, *J* = 13.0, 11.3, 5.6 Hz, 1H), 2.18 (ddd, *J* = 13.4, 8.6, 5.7 Hz, 1H), 1.39 (s, 3H).

^{13}C NMR (126 MHz, $CDCl_3$) δ 170.2, 169.9, 165.2, 64.5, 62.6, 61.3, 26.3, 25.2, 24.1, 13.3.

IR (ATR): $\tilde{\nu}$ (cm^{-1}) = 1785, 1709, 1659, 1504, 1374, 1312, 1276, 1195, 1065.

HRMS (ESI^+) calcd. for $C_{10}H_{15}N_2O_4^+$ $[M+H]^+$: 227.1026, found: 227.1026.

1,3-dioxoisoindolin-2-yl 2-acetyl-4-methyl-3-oxo-2-azabicyclo[2.2.0]hexane-1-carboxylate (21)

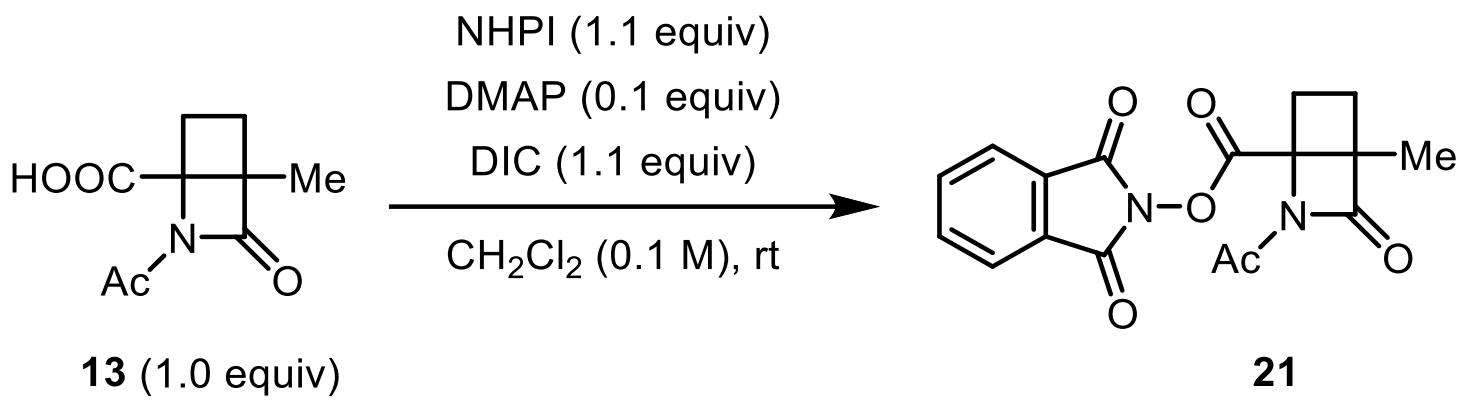

In an oven-dried 25 mL round-bottom flask carboxylic acid **13** (60 mg, 0.30 mmol, 1.0 equiv), *N*-hydroxyphthalimide (NHPI, 54 mg, 0.33 mmol, 1.1 equiv) and DMAP (3.6 mg, 0.03 mmol, 0.10 equiv) were dissolved in anhydrous CH_2Cl_2 (3.0 mL, 0.1 M) at room temperature. *N*,*N'*-Diisopropylcarbodiimide (DIC, 51 µL, 0.33 mmol, 1.1 equiv) was then added and the reaction was stirred at room temperature for 1 h. The crude product was directly purified using a Biotage automated flash chromatography system with a gradient elution of ethyl acetate/pentane (0% to 50%), affording **21** (89 mg, 0.26 mmol, 87%) as a colourless oil.

^{1}H NMR (500 MHz, $CDCl_3$) δ 7.86 (dd, *J* = 5.5, 3.1 Hz, 2H), 7.79 (dd, *J* = 5.5, 3.1 Hz, 2H), 3.15 – 3.07 (m, 1H), 2.52 – 2.46 (m, 4H), 2.40 – 2.31 (m, 2H), 1.56 (s, 3H).

^{13}C NMR (126 MHz, $CDCl_3$) δ 167.9, 166.7, 163.8, 161.3, 135.0, 128.8, 124.2, 63.2, 60.1, 26.3, 23.84, 23.80, 13.5.

IR (ATR): $\tilde{\nu}$ (cm^{-1}) = 1785, 1742, 1712, 1372, 1316, 1275, 1260, 1185, 1074.

HRMS (ESI$^+$) calcd. for $C_{17}H_{14}N_2O_6Na^+$ [M+Na]$^+$: 365.0744, found: 365.0738.

2.6 Unsuccessful Substrates

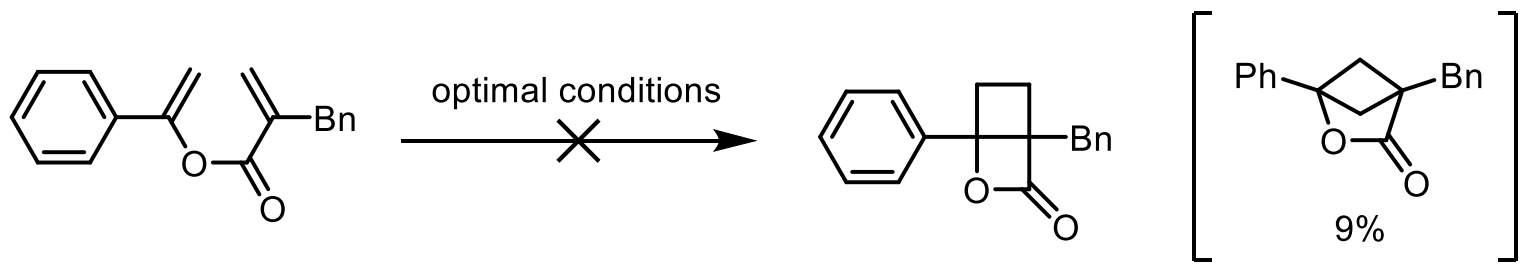

Crude ^{1}H NMR analysis after 1.5 h showed significant amounts of returned starting material; after 24 h, the spectrum appeared complex, from which the [2.1.1] bicycle could be isolated in 9% yield following column chromatography. No [2.2.0] product was detected.

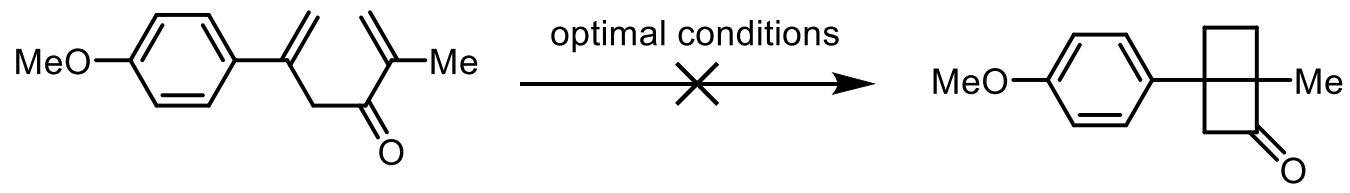

No product formation was detected, and the crude ^{1}H NMR spectrum after 1.5 h exhibited significant complexity.

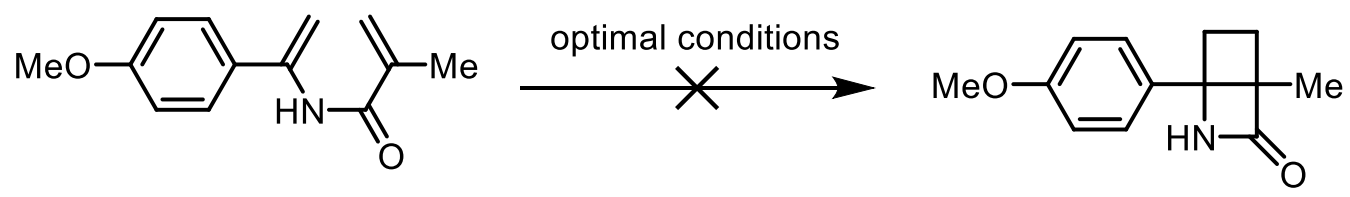

Crude ^{1}H NMR analysis after 1.5 h showed a significant amount of unreacted starting material, whereas after 24 h, the spectrum appeared complex and the desired product could not be identified.

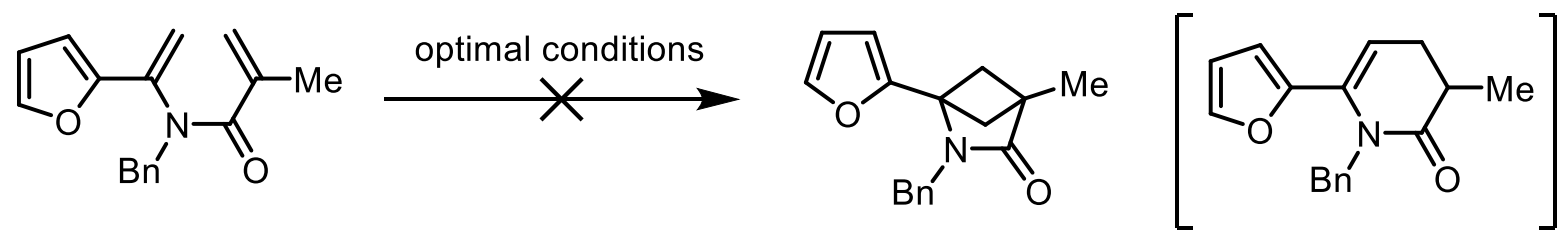

products of type **3**
not observed

products of type **4**
unstable

No product formation was detected, and the crude ^{1}H NMR spectrum after 1.5 h exhibited significant complexity. This may be due to the electron-rich nature of the furan moiety favouring the 6-*endo*-trig pathway, leading predominantly to the formation of the corresponding six-membered-ring product **4**. However, due to the photoinstability of **4** under the reaction conditions, no identifiable species were observed in the reaction mixture.

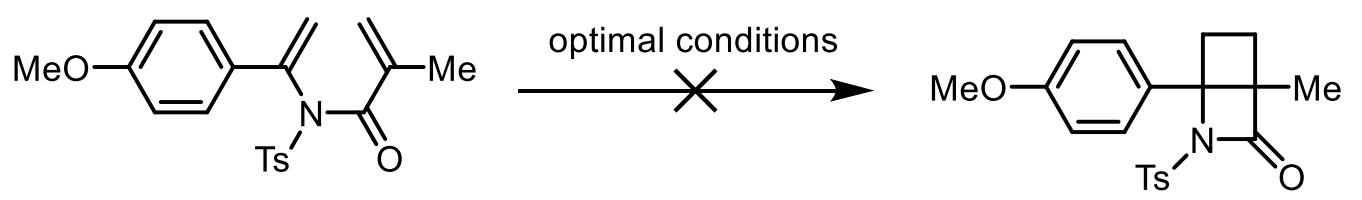

No product formation was detected, and the crude ^{1}H NMR spectrum after 1.5 h exhibited significant complexity.

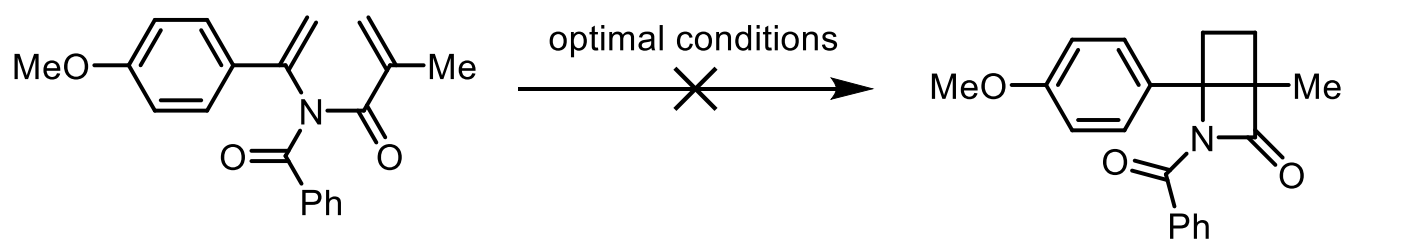

<20% ^{1}H NMR yield

The crude ^{1}H NMR spectrum after 1.5 h showed that the desired product was present in less than 20% yield.

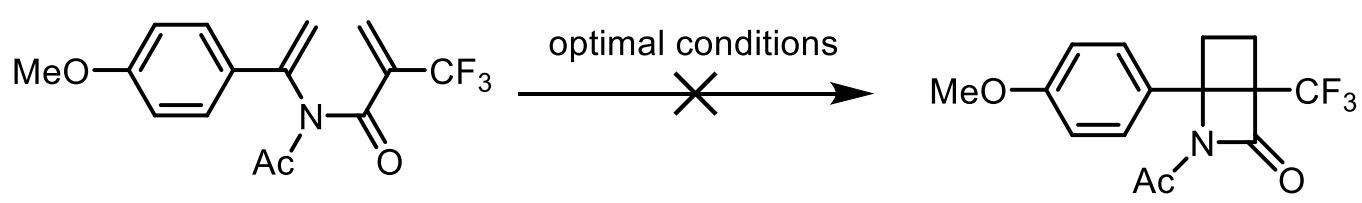

<10% ^{1}H NMR yield

The crude ^{1}H NMR spectrum after 1.5 h showed that the desired product was present in less than 10% yield.

Supplementary Figure S1. Unsuccessful substrates

3. Mechanistic studies

3.1 Stern-Volmer Quenching Studies

All samples were prepared using stock solutions of (Ir[dF(CF_3)(ppy)]$_2$)(dtbbpy)PF_6 (0.10 mM), **2f** (0.01 M), **2f'** (0.01 M) or **2f''** (0.01 M) in dry MeCN. To a volumetric flask was added (Ir[dF(CF_3)(ppy)]$_2$)(dtbbpy)PF_6 (200 µL, 0.10 mM) and the respective amount of quencher. The volume was then adjusted to 3 mL with dry MeCN. The solution was transferred to a 1-cm quartz cuvette and degassed by sparging with nitrogen gas for 15 min. Emission spectra were recorded using a FluoroMax spectrofluorometer with an excitation wavelength of 420 nm. The emission intensities for the Stern-Volmer analysis were observed at 472 nm. The ratio of I_0/I was plotted as a function of the quencher concentration (I_0: emission intensity of (Ir[dF(CF_3)(ppy)]$_2$)(dtbbpy)PF_6 without quencher; I: emission intensity of (Ir[dF(CF_3)(ppy)]$_2$)(dtbbpy)PF_6 in the presence of quencher). The Stern-Volmer analysis shows that (Ir[dF(CF_3)(ppy)]$_2$)(dtbbpy)PF_6 is only efficiently quenched by the styrene moiety in **2f** or **2f'**, while the enone moiety (**2f''**) does not quench the photocatalyst.

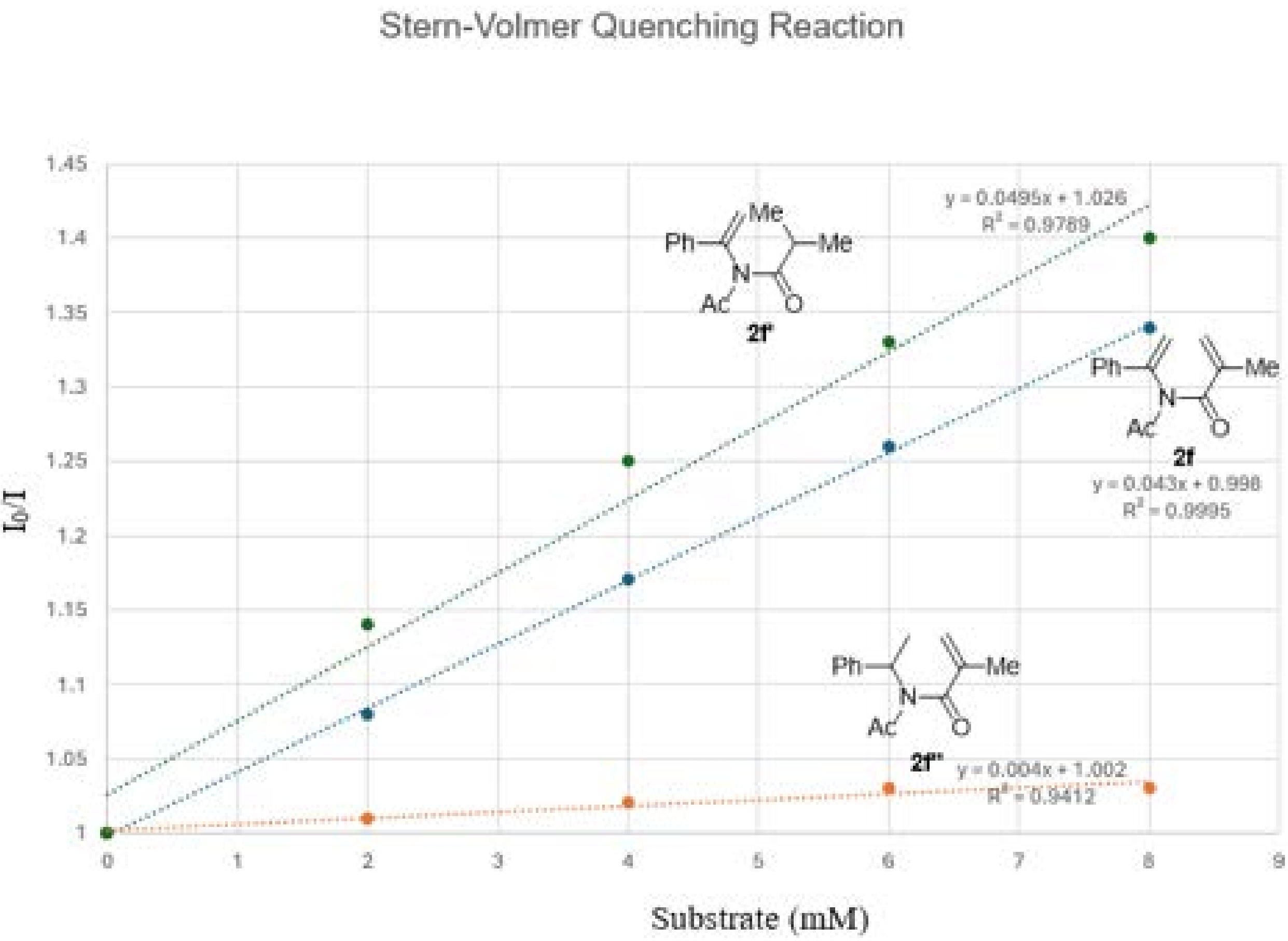

Supplementary Figure S2. Stern-Volmer quenching study

3.2 UV-Vis Spectroscopy

In MeCN: UV-Vis-NIR spectroscopy measurements were carried out using a Shimadzu UV-2600 spectrometer fitted with an ISR-2600 integrating sphere attachment. Measurements were recorded in 10 mm path-length quartz cuvettes. The concentrations of the components are as follows: **1c** (0.01 M), **2f** (0.01 M).

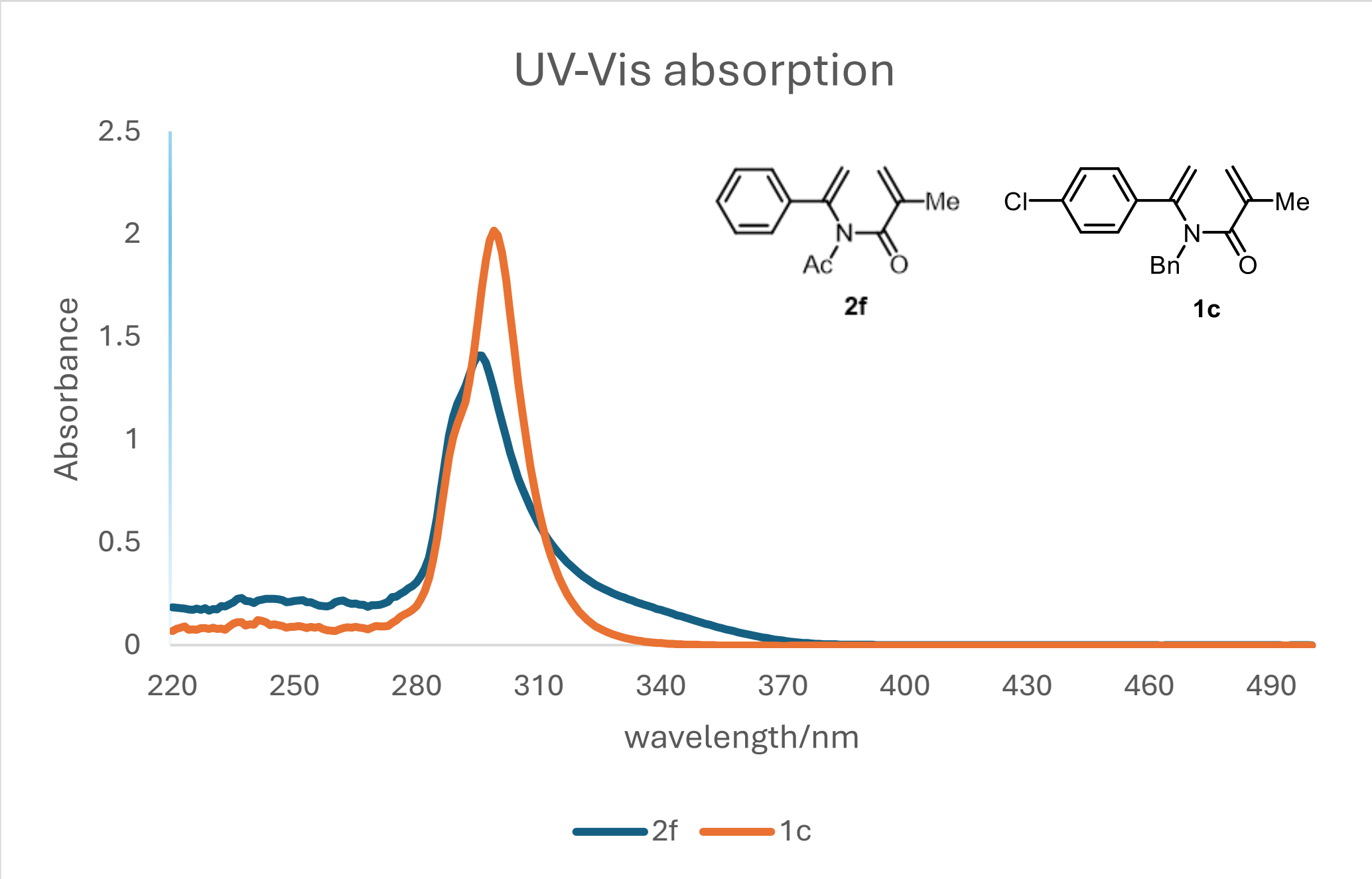

Supplementary Figure S3. UV-Vis absorption in MeCN

Comment: The UV-Vis absorption spectra of representative substrates **1c** and **2f** recorded in MeCN confirm that neither compound exhibits any appreciable absorption at 427 nm (the operational irradiation wavelength), thereby ruling out the possibility of direct photoexcitation of the substrate and supporting the proposed triplet energy-transfer (EnT) mechanism mediated by the iridium photocatalyst.

3.3. Cyclic Voltammetry

Cyclic voltammetry was performed at room temperature using a MultiPalmSens 4 potentiostat. CV analysis with a glassy carbon working electrode, a platinum counter electrode, an Ag/AgNO$_3$ (0.1 M) reference electrode, and a scan rate of 100 mV/s. The experimental setup was calibrated using ferrocene (Fc+/Fc) prior to each experiment. Samples were prepared with 0.05 mmol of substrate in 10 mL *n*-Bu$_4$NPF$_6$ electrolyte (0.05 M in MeCN) and degassed by sparging with nitrogen gas for 10 min prior to use. The potential ($E_{1/2}$) was determined and referenced to the SCE as described by Addison.[19] The background was recorded using the electrolyte solution (0.05 M n-Bu$_4$NPF$_6$ in MeCN) in the absence of substrate.

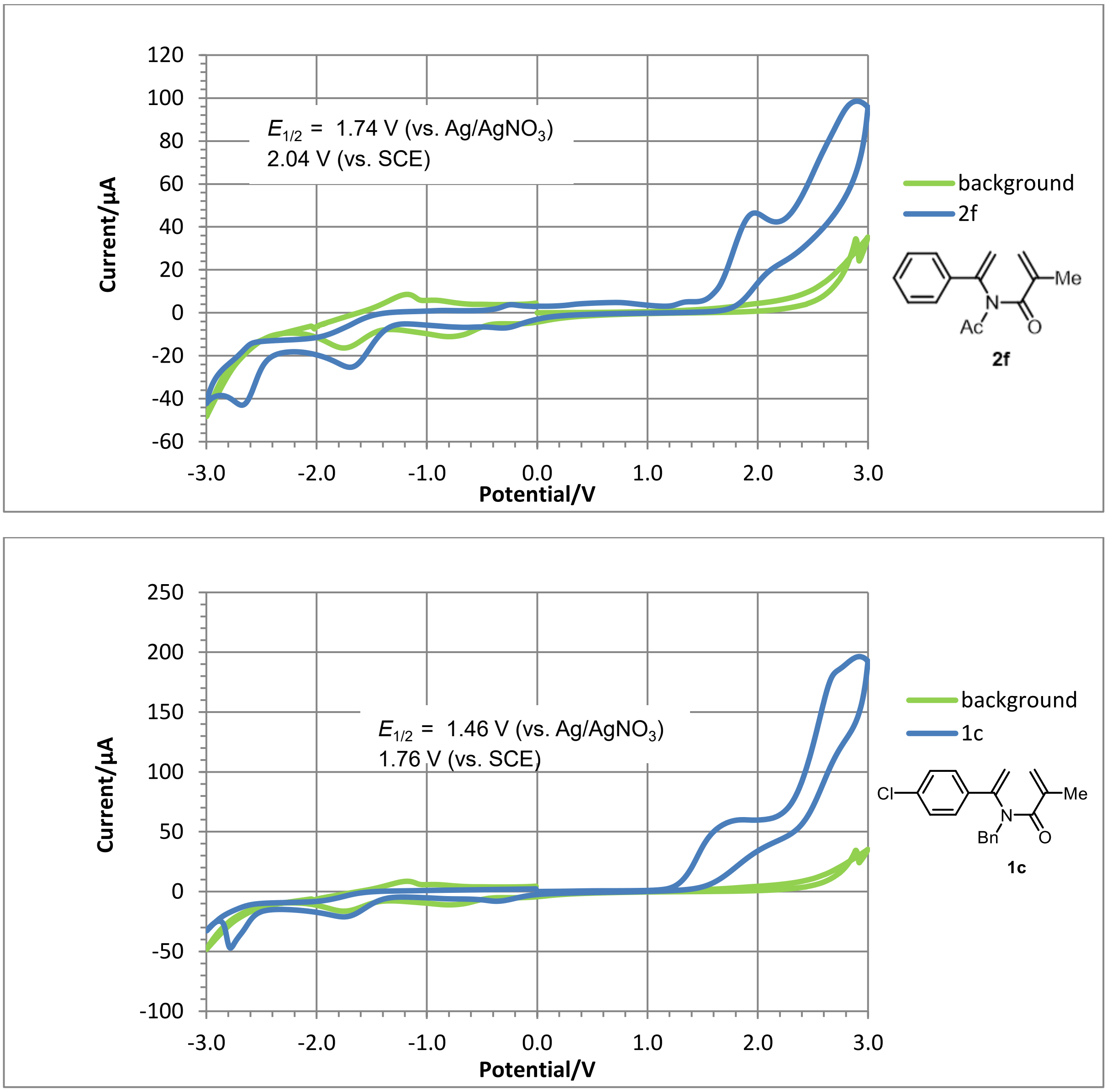

Supplementary Figure S4. Cyclic voltammogram of compounds **1c** and **2f**

Comment: The cyclic voltammogram shows an irreversible oxidation process with $E_{1/2}$ = +2.04 V (vs. SCE) of compound **2f** and $E_{1/2}$ = +1.76 V (vs. SCE) of compound **1c**. [Ir(dF(CF$_3$)ppy)$_2$(dtbbpy)]PF$_6$

($E_{1/2}^{III*/II}$ = +1.21 V vs. SCE)[20] does not possess an excited state oxidation potential sufficient to oxidise **1c** and **2f**; thus, a photoredox process is unlikely.

3.4 Control Experiments with Triplet Quenchers

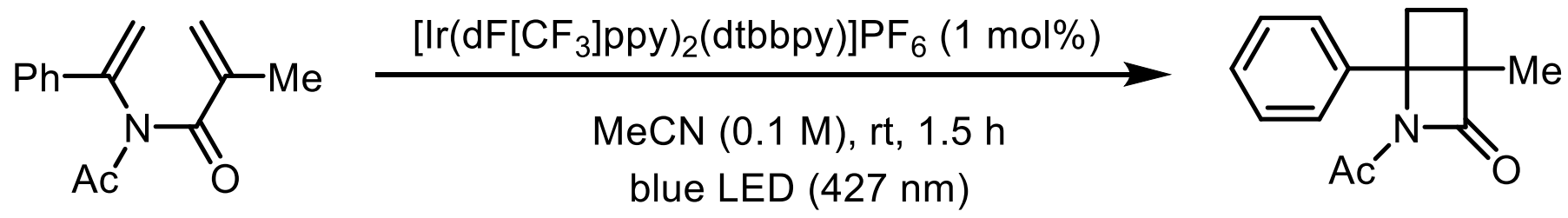

2f **6f**

quencher	yield (%)
none	76
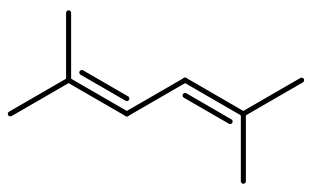 (3 equiv.)	21[a]

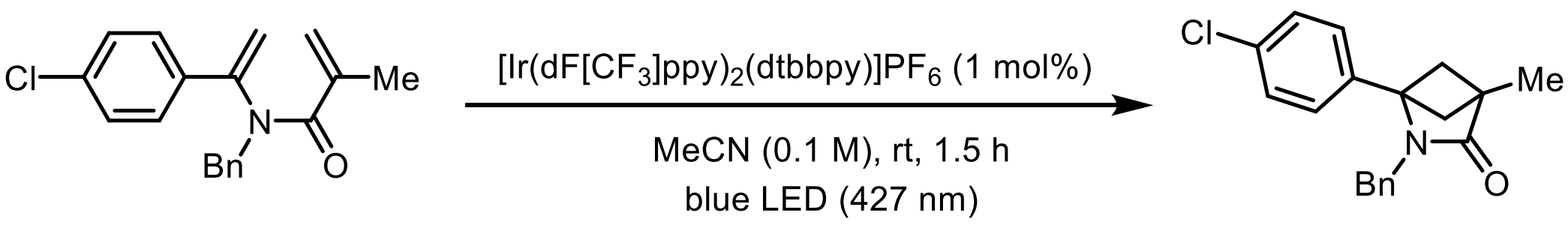

1c **3c**

quencher	yield (%)
none	52
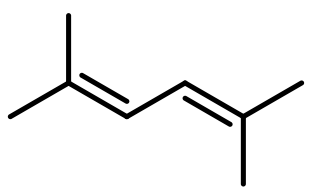 (3 equiv.)	16[a]

[a] The yields were determined by quantitative ^{1}H NMR spectroscopy of the crude reaction mixture using CH_2Br_2 as the internal standard.

4. Crystallographic Data (X-Ray)

4.1 Structure Report for 3d

The crystallographic data for **3d** have been deposited at the Cambridge Crystallographic Data Centre under deposition number CCDC 2520219. Copies of the data can be obtained free of charge via www.ccdc.cam.ac.uk/structures.

Crystallographic data are presented in the Tables below. A single crystal of **3d** was coated in perfluoropolyether oil and mounted on a glass fibre. X-ray measurements were made using a Bruker D8 Venture dual source kappa CPAD diffractometer with Cu K_a radiation (λ= 1.54184 Å).[21] Intensities were integrated from several series of exposures, each exposure covering 0.5° in ω or ϕ. Absorption corrections were applied, based on multiple and symmetry-equivalent measurements.[22] The structure was solved by direct methods and refined by least squares on weighted F^2 values for all reflections (see **Table S3**).[23]

All non-hydrogen atoms were assigned anisotropic displacement parameters and refined without positional constraints. All hydrogen atoms were constrained to ideal geometries and were assigned isotropic displacement parameters equal to 1.2 times that of their parent atom.

Refinement proceeded smoothly to give the residuals shown in **Table S3**. Complex neutral-atom scattering factors were used.[24]

The absolute configuration of the molecule has been established with a Flack/Parsons parameter value of 0.02(9).[25]

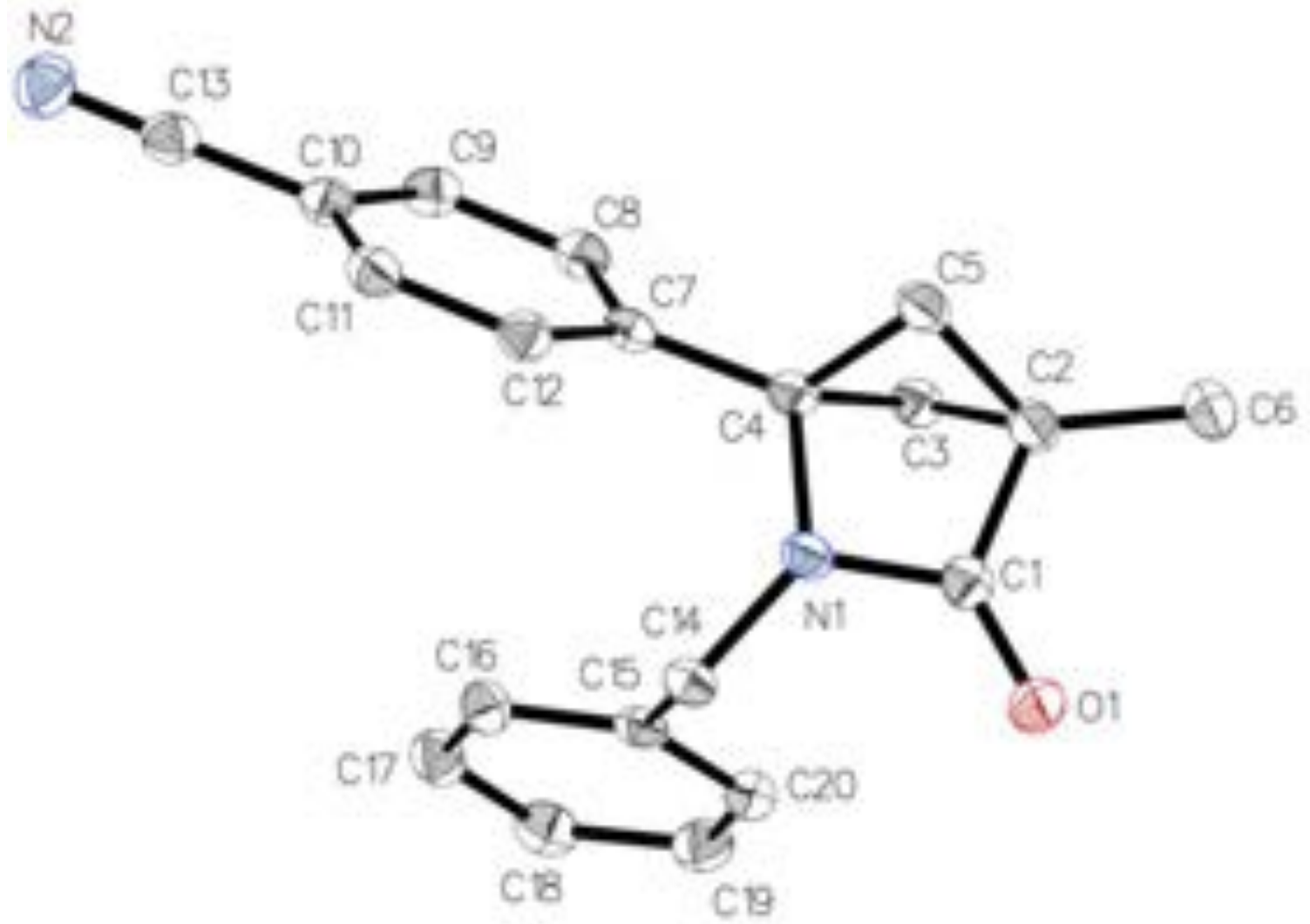

Supplementary Figure S5: Molecular structure of **3d** with thermal ellipsoids shown at the 50% probability level; hydrogen atoms have been omitted for clarity

Supplementary Table S3. Crystal data and structure refinement for **3d**.

Identification code	cu_ZZX652Cu2_0m	
Empirical formula	$C_{20} H_{18} N_2 O$	
Formula weight	302.36	
Temperature	108(2) K	
Wavelength	1.54184 Å	
Crystal system	Monoclinic	
Space group	C2	
Unit cell dimensions	a = 17.4943(12) Å	α = 90°
	b = 7.3922(5) Å	β = 103.709(2)°
	c = 12.5448(8) Å	γ = 90°
Volume	1576.09(18) Å^3	
Z	4	
Density (calculated)	1.274 Mg/m^3	
Absorption coefficient	0.624 mm^{-1}	
F(000)	640	
Crystal size	0.320 x 0.240 x 0.160 mm	
θ range for data collection	3.627 to 68.742°	
Index ranges	-21<=h<=20, -8<=k<=8, -15<=l<=15	
Reflections collected	11966	
Independent reflections	2821 [R_{int} = 0.0313]	
Completeness to θ = 67.684°	99.0 %	
Absorption correction	Numerical	
Max. and min. transmission	1.0000 and 0.7772	
Refinement method	Full-matrix least-squares on F^2	
Data / restraints / parameters	2821 / 1 / 210	
Goodness-of-fit on F^2	S = 1.078	
R indices [for 2779 reflections with I>2σ(I)]		R_1 = 0.0317, wR_2 = 0.0765
R indices (for all 2821 data)	R_1 = 0.0329, wR_2 = 0.0771	
Weighting scheme	$w^{-1} = \sigma^2(F_o{}^2) + (aP)^2 + (bP)$,	
	where $P = [\max(F_o{}^2, 0) + 2F_c{}^2]/3$	
	a = 0.0487, b = 0.3852	
Absolute structure (Flack) parameter	0.02(9)	
Extinction coefficient	0.0032(5)	
Largest diff. peak and hole	0.274 and -0.206 eÅ^{-3}	

Supplementary Table S4. Bond lengths [Å] and angles [°] for **3d**.

C(1)-O(1)	1.224(2)
C(1)-N(1)	1.369(2)
C(1)-C(2)	1.527(3)
C(2)-C(6)	1.501(3)
C(2)-C(5)	1.562(3)
C(2)-C(3)	1.571(3)
C(3)-C(4)	1.562(2)
C(4)-C(7)	1.487(2)
C(4)-N(1)	1.501(2)
C(4)-C(5)	1.549(3)
C(7)-C(8)	1.391(3)
C(7)-C(12)	1.400(2)
C(8)-C(9)	1.382(3)
C(9)-C(10)	1.399(3)
C(10)-C(11)	1.394(3)
C(10)-C(13)	1.435(2)
C(11)-C(12)	1.382(3)
C(13)-N(2)	1.152(3)
C(14)-N(1)	1.456(2)
C(14)-C(15)	1.518(2)
C(15)-C(16)	1.391(3)
C(15)-C(20)	1.397(2)
C(16)-C(17)	1.391(3)
C(17)-C(18)	1.390(3)
C(18)-C(19)	1.390(3)
C(19)-C(20)	1.382(3)
O(1)-C(1)-N(1)	127.64(17)
O(1)-C(1)-C(2)	129.23(17)
N(1)-C(1)-C(2)	103.08(15)
C(6)-C(2)-C(1)	117.65(16)
C(6)-C(2)-C(5)	124.64(17)
C(1)-C(2)-C(5)	99.99(14)
C(6)-C(2)-C(3)	123.83(16)
C(1)-C(2)-C(3)	97.93(14)
C(5)-C(2)-C(3)	85.60(13)
C(4)-C(3)-C(2)	81.62(13)
C(7)-C(4)-N(1)	115.98(14)

C(7)-C(4)-C(5)	121.93(15)
N(1)-C(4)-C(5)	99.25(14)
C(7)-C(4)-C(3)	125.50(14)
N(1)-C(4)-C(3)	101.60(13)
C(5)-C(4)-C(3)	86.32(13)
C(4)-C(5)-C(2)	82.31(13)
C(8)-C(7)-C(12)	118.98(17)
C(8)-C(7)-C(4)	120.09(15)
C(12)-C(7)-C(4)	120.85(15)
C(9)-C(8)-C(7)	120.78(16)
C(8)-C(9)-C(10)	119.65(16)
C(11)-C(10)-C(9)	120.25(16)
C(11)-C(10)-C(13)	120.46(16)
C(9)-C(10)-C(13)	119.29(16)
C(12)-C(11)-C(10)	119.39(16)
C(11)-C(12)-C(7)	120.92(17)
N(2)-C(13)-C(10)	178.6(2)
N(1)-C(14)-C(15)	114.84(14)
C(16)-C(15)-C(20)	118.68(16)
C(16)-C(15)-C(14)	120.44(15)
C(20)-C(15)-C(14)	120.84(16)
C(17)-C(16)-C(15)	120.80(17)
C(18)-C(17)-C(16)	119.85(17)
C(19)-C(18)-C(17)	119.72(17)
C(20)-C(19)-C(18)	120.18(16)
C(19)-C(20)-C(15)	120.73(17)
C(1)-N(1)-C(14)	123.15(15)
C(1)-N(1)-C(4)	102.64(14)
C(14)-N(1)-C(4)	126.14(14)

4.2 Structure Report for 5p

The crystallographic data for **5p** have been deposited at the Cambridge Crystallographic Data Centre under deposition number CCDC 2520220. Copies of the data can be obtained free of charge via www.ccdc.cam.ac.uk/structures.

Crystallographic data are presented in the Tables below. A single crystal of **5p** was coated in perfluoropolyether oil and mounted on a glass fibre. X-ray measurements were made using a Bruker D8 Venture dual source kappa CPAD diffractometer with Mo K_α radiation (λ = 0.71073 Å).[21] Intensities were integrated from several series of exposures, each exposure covering 0.5° in ω or ϕ. Absorption corrections were applied, based on multiple and symmetry-equivalent measurements.[22] The structure was solved by direct methods and refined by least squares on weighted F^2 values for all reflections (see **Table S5**).[23]

All non-hydrogen atoms were assigned anisotropic displacement parameters and refined without positional constraints. The positions of the methyl hydrogen atoms were assigned by a rotating group refinement with fixed, idealised C-H distances. All other hydrogen atoms were constrained to ideal geometries. All hydrogen atoms were assigned isotropic displacement parameters equal to 1.5 times (methyl hydrogen atoms) or 1.2 times (all other hydrogen atoms) that of their parent atom.

Refinement proceeded smoothly to give the residuals shown in **Table S5**. Complex neutral-atom scattering factors were used.[24]

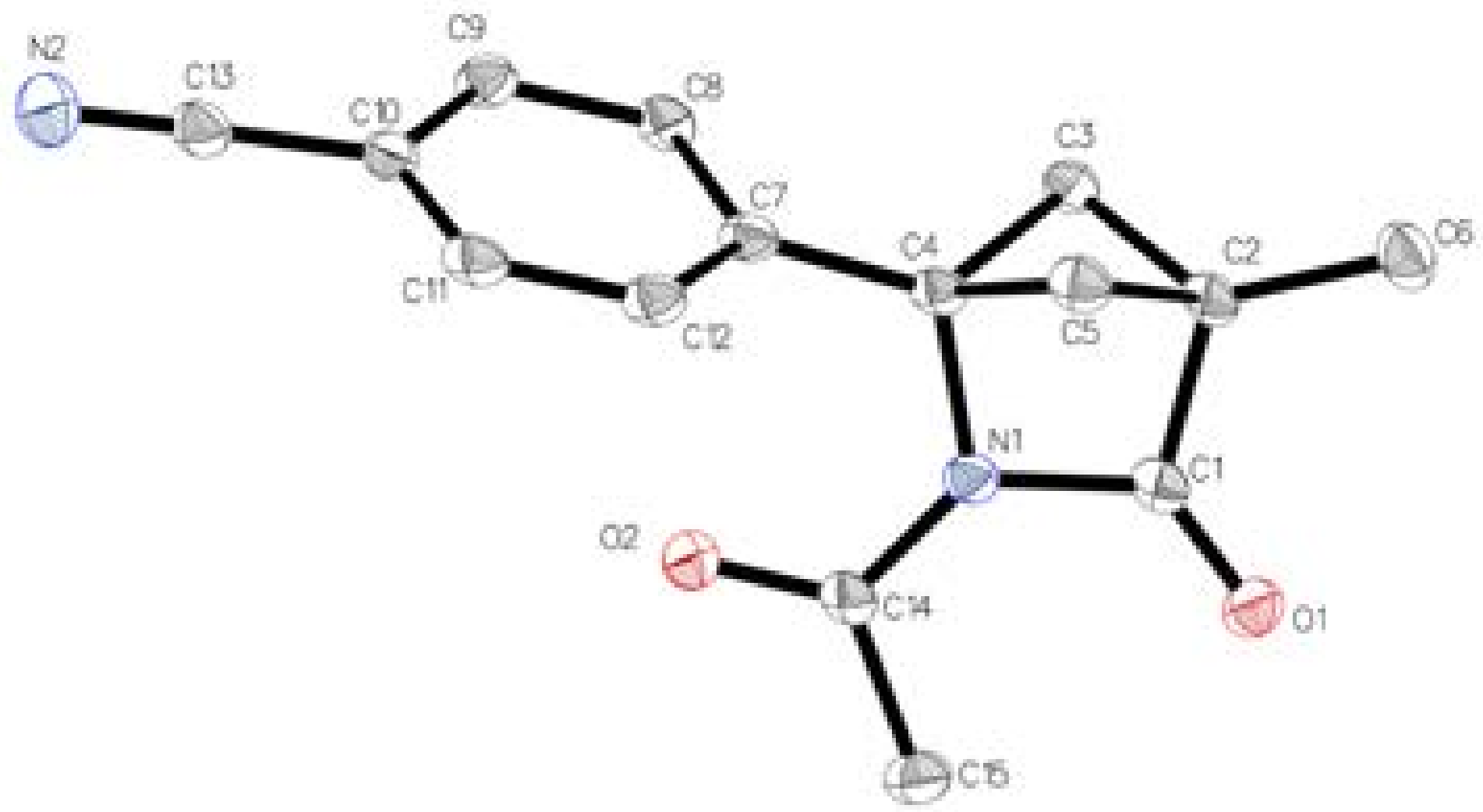

Supplementary Figure S6: Molecular structure of **5p** with thermal ellipsoids shown at the 50% probability level; hydrogen atoms have been omitted for clarity

Supplementary Table S5. Crystal data and structure refinement for **5p**.

Identification code	zzx752_2	
Empirical formula	$C_{15} H_{14} N_2 O_2$	
Formula weight	254.28	
Temperature	100(2) K	
Wavelength	0.71073 Å	
Crystal system	Monoclinic	
Space group	$P2_1/n$	
Unit cell dimensions	a = 7.5188(6) Å	α = 90°
	b = 12.0570(10) Å	β = 95.497(3)°
	c = 14.2757(12) Å	γ = 90°
Volume	1288.20(18) $Å^3$	
Z	4	
Density (calculated)	1.311 Mg/m^3	
Absorption coefficient	0.089 mm^{-1}	
F(000)	536	
Crystal size	0.590 x 0.330 x 0.230 mm	
θ range for data collection	2.215 to 28.343°	
Index ranges	$-10<=h<=10$, $-15<=k<=16$, $-18<=l<=19$	
Reflections collected	13926	
Independent reflections	3165 [R_{int} = 0.0961]	
Completeness to θ = 25.242°	98.9 %	
Absorption correction	Numerical	
Max. and min. transmission	1.0000 and 0.5363	
Refinement method	Full-matrix least-squares on F^2	
Data / restraints / parameters	3165 / 0 / 174	
Goodness-of-fit on F^2	S = 1.076	
R indices [for 2224 reflections with $I>2\sigma(I)$]		R_1 = 0.0887, wR_2 = 0.2660
R indices (for all 3165 data)	R_1 = 0.1174, wR_2 = 0.2908	
Weighting scheme	$w^{-1} = \sigma^2(F_o^2) + (aP)^2$,	
	where $P = [\max(F_o^2, 0) + 2F_c^2]/3$	
	a = 0.1986	
Largest diff. peak and hole	0.530 and -0.528 $eÅ^{-3}$	

Supplementary Table S6. Bond lengths [Å] and angles [°] for **5p**

C(1)-O(1)	1.206(3)
C(1)-N(1)	1.414(3)
C(1)-C(2)	1.521(3)
C(2)-C(6)	1.500(4)
C(2)-C(3)	1.562(4)
C(2)-C(5)	1.563(3)
C(3)-C(4)	1.548(3)
C(4)-C(7)	1.490(3)
C(4)-N(1)	1.511(3)
C(4)-C(5)	1.547(4)
C(7)-C(8)	1.389(4)
C(7)-C(12)	1.401(3)
C(8)-C(9)	1.388(4)
C(9)-C(10)	1.393(4)
C(10)-C(11)	1.399(4)
C(10)-C(13)	1.437(4)
C(11)-C(12)	1.379(4)
C(13)-N(2)	1.152(4)
C(14)-O(2)	1.215(3)
C(14)-N(1)	1.389(3)
C(14)-C(15)	1.501(3)
O(1)-C(1)-N(1)	128.2(2)
O(1)-C(1)-C(2)	129.9(2)
N(1)-C(1)-C(2)	101.87(19)
C(6)-C(2)-C(1)	116.9(2)
C(6)-C(2)-C(3)	123.7(2)
C(1)-C(2)-C(3)	99.7(2)
C(6)-C(2)-C(5)	124.8(2)
C(1)-C(2)-C(5)	98.54(19)
C(3)-C(2)-C(5)	86.34(19)
C(4)-C(3)-C(2)	82.33(18)
C(7)-C(4)-N(1)	116.94(19)
C(7)-C(4)-C(5)	123.21(19)
N(1)-C(4)-C(5)	99.23(19)
C(7)-C(4)-C(3)	123.8(2)
N(1)-C(4)-C(3)	99.97(17)
C(5)-C(4)-C(3)	87.39(19)

C(4)-C(5)-C(2)	82.32(18)
C(8)-C(7)-C(12)	119.4(2)
C(8)-C(7)-C(4)	120.3(2)
C(12)-C(7)-C(4)	120.2(2)
C(9)-C(8)-C(7)	120.8(2)
C(8)-C(9)-C(10)	119.0(2)
C(9)-C(10)-C(11)	120.6(2)
C(9)-C(10)-C(13)	119.5(3)
C(11)-C(10)-C(13)	119.8(2)
C(12)-C(11)-C(10)	119.6(2)
C(11)-C(12)-C(7)	120.2(3)
N(2)-C(13)-C(10)	178.8(2)
O(2)-C(14)-N(1)	121.3(2)
O(2)-C(14)-C(15)	121.8(2)
N(1)-C(14)-C(15)	116.9(2)
C(14)-N(1)-C(1)	128.6(2)
C(14)-N(1)-C(4)	128.0(2)
C(1)-N(1)-C(4)	102.23(18)

4.3 Structure Report for 6p

The crystallographic data for **6p** have been deposited at the Cambridge Crystallographic Data Centre under deposition number CCDC 2520221. Copies of the data can be obtained free of charge via www.ccdc.cam.ac.uk/structures.

Crystallographic data are presented in the Tables below. A single crystal of **6p** was coated in perfluoropolyether oil and mounted on a glass fibre. X-ray measurements were made using a Bruker D8 Venture dual source kappa CPAD diffractometer with Mo K_α radiation (λ = 0.71073 Å).[21] Intensities were integrated from several series of exposures, each exposure covering 0.5° in ω or ϕ. Absorption corrections were applied, based on multiple and symmetry-equivalent measurements.[22] The structure was solved by direct methods and refined by least squares on weighted F^2 values for all reflections (see **Table S7**).[23]

All non-hydrogen atoms were assigned anisotropic displacement parameters and refined without positional constraints. The positions of the methyl hydrogen atoms were assigned by a rotating group refinement with fixed, idealised C-H distances. All other hydrogen atoms were constrained to ideal geometries. All hydrogen atoms and were assigned isotropic displacement parameters equal to 1.5 times (methyl hydrogen atoms) or 1.2 times (all other hydrogen atoms) that of their parent atom.

Refinement proceeded smoothly to give the residuals shown in **Table S7**. Complex neutral-atom scattering factors were used.[24]

The unit cell contains two distinct molecules (shown in **Figure S7**) with identical stereochemistry. An overlay of the two molecules (shown in **Figure S8**) shows that they mainly differ in the conformation of the cyanophenyl ring with the N1-C5-C7-C8 torsion angle differing from its N101-C105-C107-C108 counterpart by 58.59(16)° with an additional slight difference in the positions of the acyl substituent of the N1-C5 ring, presumably accounted for by packing forces within the structure.

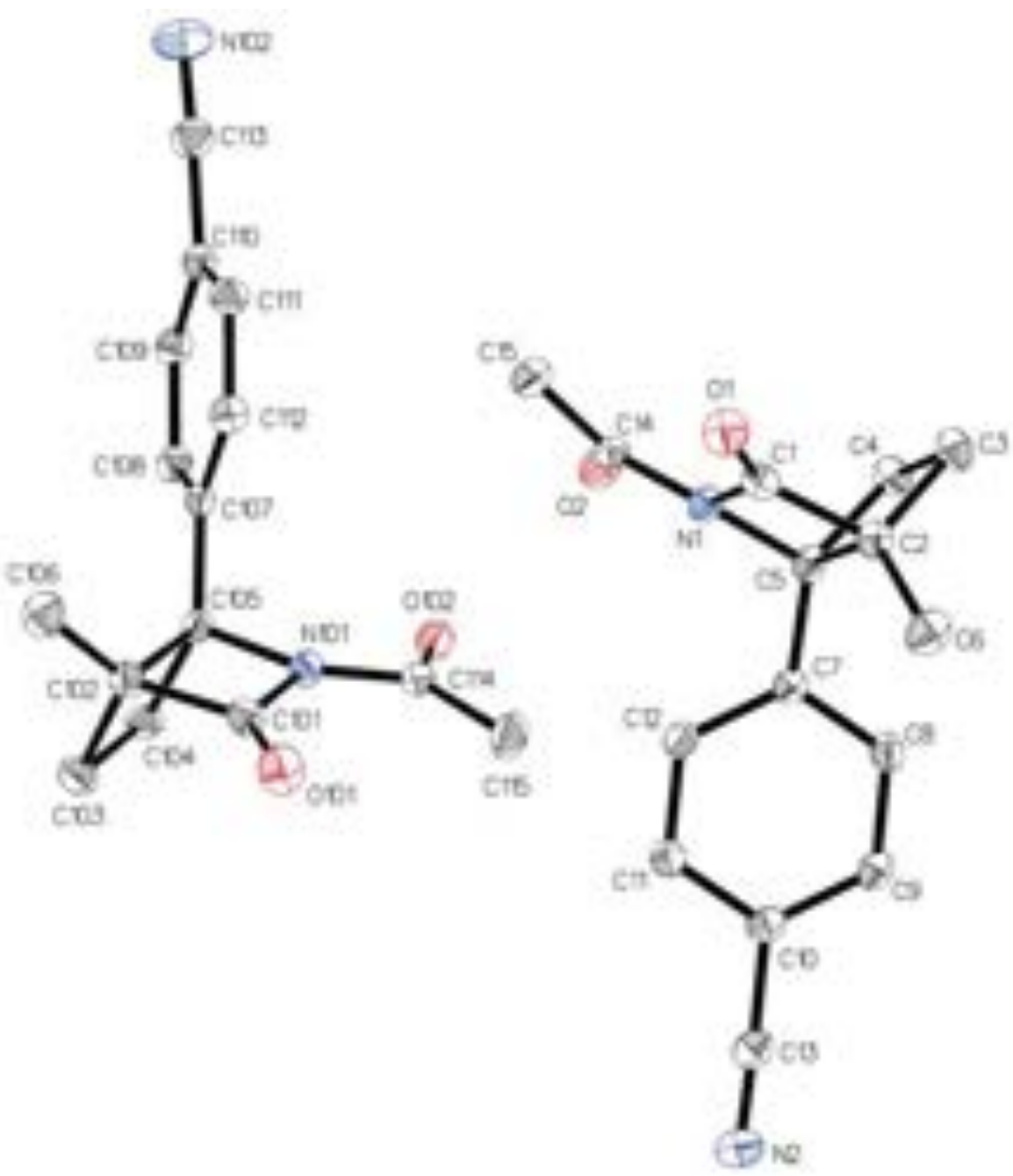

Supplementary Figure S7: Molecular structures of the two independent molecules found in the unit cell for **6p** with thermal ellipsoids shown at the 50% probability level; hydrogen atoms have been omitted for clarity.

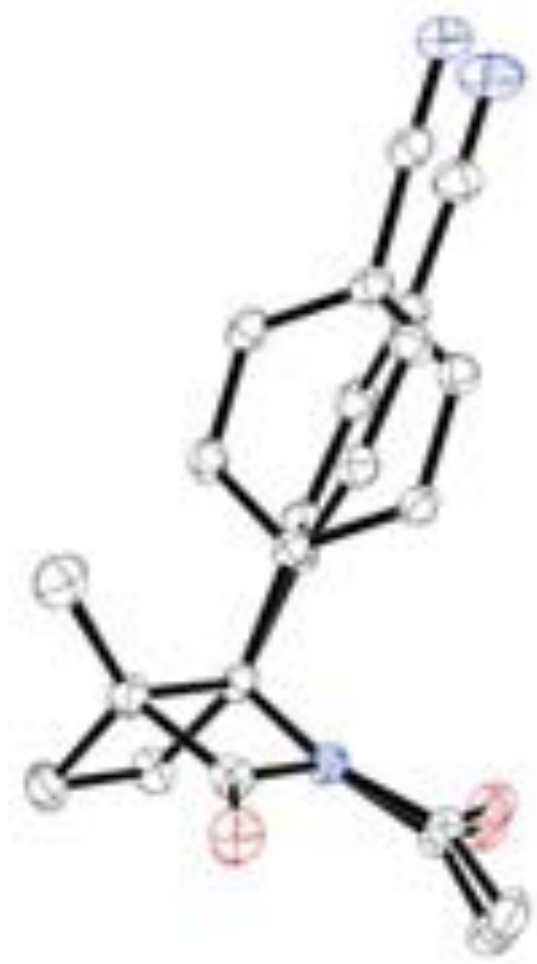

Supplementary Figure S8: Overlay of the two independent molecules found in the unit cell for **6p** with thermal ellipsoids shown at the 50% probability level; hydrogen atoms have been omitted for clarity.

The molecular structure of the first molecule within the unit cell is shown in **Figure S9**. Within the four-membered C2-C5 ring, the C2-C3 bond is slightly [0.015(3) Å] longer than those seen for C3-C4 and C4-C5, whilst the C2-C5 bond that links the bridgehead atoms is appreciably [0.047(2) Å] longer than the C4-C5 bond.

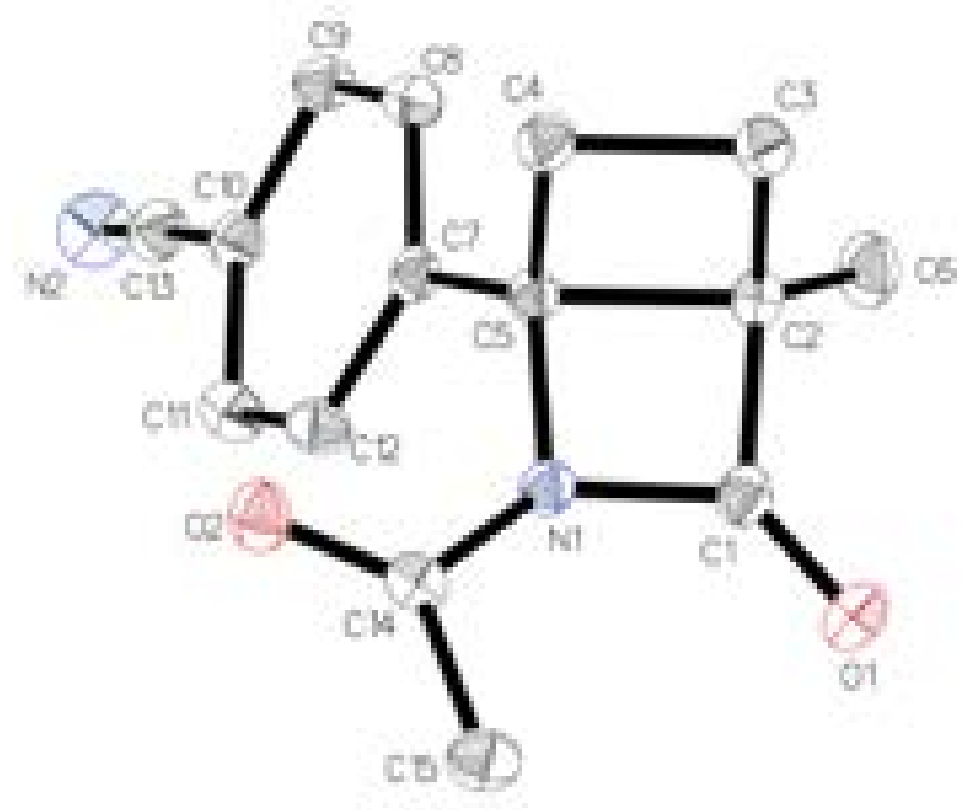

Supplementary Figure S9: Molecular structure of one of the two independent molecules found in the unit cell for 6p with thermal ellipsoids shown at the 50% probability level; hydrogen atoms have been omitted for clarity.

Supplementary Table S7. Crystal data and structure refinement for **6p**

Identification code	mo_ZZX752_1_0m	
Empirical formula	C_{15} H_{14} N_2 O_2	
Formula weight	254.28	
Temperature	100(2) K	
Wavelength	0.71073 Å	
Crystal system	Triclinic	
Space group	P-1	
Unit cell dimensions	a = 9.696(3) Å	α= 84.369(7)°.
	b = 12.448(4) Å	β= 68.207(7)°.
	c = 12.746(4) Å	γ = 68.928(7)°.
Volume	1331.8(7) $Å^3$	
Z	4	
Density (calculated)	1.268 Mg/m^3	
Absorption coefficient	0.086 mm^{-1}	
F(000)	536	
Crystal size	0.960 x 0.520 x 0.190 mm^3	
Theta range for data collection	1.755 to 28.271°.	
Index ranges	-12<=h<=12, -16<=k<=16, -16<=l<=16	
Reflections collected	28961	
Independent reflections	6407 [R_{int} = 0.0495]	
Completeness to theta = 25.242°	99.2 %	
Absorption correction	Numerical	
Max. and min. transmission	1.0000 and 0.8452	
Refinement method	Full-matrix least-squares on F^2	
Data / restraints / parameters	6407 / 0 / 347	
Goodness-of-fit on F^2	1.079	
Final R indices [I>2σ(I)]	R_1 = 0.0472, wR_2 = 0.1115	
R indices (all 6407 data)	R_1 = 0.0557, wR_2 = 0.1181	
Weighting scheme	$w^{-1} = \sigma^2(F_o^2) + (aP)^2 + (bP)$,	
	where $P = [\max(F_o^2, 0) + 2F_c^2]/3$	
	a = 0.0577, b = 0.5294	
Largest diff. peak and hole	0.407 and -0.346 e.$Å^{-3}$	

Supplementary Table S8. Bond lengths [Å] and angles [°] for **6p**

C(1)-O(1)	1.2056(16)
C(1)-N(1)	1.4112(17)
C(1)-C(2)	1.5247(18)
C(2)-C(6)	1.5106(18)
C(2)-C(3)	1.5676(18)
C(2)-C(5)	1.5990(18)
C(3)-C(4)	1.5528(19)
C(3)-H(3A)	0.9900
C(3)-H(3B)	0.9900
C(4)-C(5)	1.5523(17)
C(4)-H(4A)	0.9900
C(4)-H(4B)	0.9900
C(5)-N(1)	1.4867(15)
C(5)-C(7)	1.4952(16)
C(6)-H(6A)	0.9800
C(6)-H(6B)	0.9800
C(6)-H(6C)	0.9800
C(7)-C(8)	1.3992(17)
C(7)-C(12)	1.4003(17)
C(8)-C(9)	1.3884(17)
C(8)-H(8)	0.9500
C(9)-C(10)	1.3997(18)
C(9)-H(9)	0.9500
C(10)-C(11)	1.3973(17)
C(10)-C(13)	1.4461(17)
C(11)-C(12)	1.3866(17)
C(11)-H(11)	0.9500
C(12)-H(12)	0.9500
C(13)-N(2)	1.1507(17)
C(14)-O(2)	1.2160(16)
C(14)-N(1)	1.3971(16)
C(14)-C(15)	1.5036(18)
C(15)-H(15A)	0.9800
C(15)-H(15B)	0.9800
C(15)-H(15C)	0.9800
C(101)-O(101)	1.2073(16)
C(101)-N(101)	1.4080(16)
C(101)-C(102)	1.5280(18)

C(102)-C(106)	1.5084(18)
C(102)-C(103)	1.5661(18)
C(102)-C(105)	1.5987(17)
C(103)-C(104)	1.5537(18)
C(103)-H(10C)	0.9900
C(103)-H(10D)	0.9900
C(104)-C(105)	1.5485(17)
C(104)-H(10A)	0.9900
C(104)-H(10B)	0.9900
C(105)-N(101)	1.4895(16)
C(105)-C(107)	1.5036(16)
C(106)-H(10E)	0.9800
C(106)-H(10F)	0.9800
C(106)-H(10G)	0.9800
C(107)-C(108)	1.3959(18)
C(107)-C(112)	1.4036(17)
C(108)-C(109)	1.3960(17)
C(108)-H(108)	0.9500
C(109)-C(110)	1.4020(19)
C(109)-H(109)	0.9500
C(110)-C(111)	1.398(2)
C(110)-C(113)	1.4507(17)
C(111)-C(112)	1.3895(17)
C(111)-H(111)	0.9500
C(112)-H(112)	0.9500
C(113)-N(102)	1.1488(18)
C(114)-O(102)	1.2178(16)
C(114)-N(101)	1.3927(16)
C(114)-C(115)	1.5048(18)
C(115)-H(11A)	0.9800
C(115)-H(11B)	0.9800
C(115)-H(11C)	0.9800
O(1)-C(1)-N(1)	131.31(12)
O(1)-C(1)-C(2)	136.16(12)
N(1)-C(1)-C(2)	92.51(9)
C(6)-C(2)-C(1)	118.59(11)
C(6)-C(2)-C(3)	120.37(11)
C(1)-C(2)-C(3)	110.72(10)
C(6)-C(2)-C(5)	124.92(11)
C(1)-C(2)-C(5)	86.00(9)

C(3)-C(2)-C(5)	88.40(9)
C(4)-C(3)-C(2)	91.05(9)
C(4)-C(3)-H(3A)	113.5
C(2)-C(3)-H(3A)	113.5
C(4)-C(3)-H(3B)	113.5
C(2)-C(3)-H(3B)	113.5
H(3A)-C(3)-H(3B)	110.8
C(5)-C(4)-C(3)	90.63(9)
C(5)-C(4)-H(4A)	113.5
C(3)-C(4)-H(4A)	113.5
C(5)-C(4)-H(4B)	113.5
C(3)-C(4)-H(4B)	113.5
H(4A)-C(4)-H(4B)	110.8
N(1)-C(5)-C(7)	116.63(10)
N(1)-C(5)-C(4)	111.59(9)
C(7)-C(5)-C(4)	122.85(10)
N(1)-C(5)-C(2)	86.84(9)
C(7)-C(5)-C(2)	120.76(10)
C(4)-C(5)-C(2)	89.90(9)
C(2)-C(6)-H(6A)	109.5
C(2)-C(6)-H(6B)	109.5
H(6A)-C(6)-H(6B)	109.5
C(2)-C(6)-H(6C)	109.5
H(6A)-C(6)-H(6C)	109.5
H(6B)-C(6)-H(6C)	109.5
C(8)-C(7)-C(12)	119.00(11)
C(8)-C(7)-C(5)	119.40(11)
C(12)-C(7)-C(5)	121.39(10)
C(9)-C(8)-C(7)	120.81(11)
C(9)-C(8)-H(8)	119.6
C(7)-C(8)-H(8)	119.6
C(8)-C(9)-C(10)	119.42(11)
C(8)-C(9)-H(9)	120.3
C(10)-C(9)-H(9)	120.3
C(11)-C(10)-C(9)	120.28(11)
C(11)-C(10)-C(13)	118.91(11)
C(9)-C(10)-C(13)	120.78(11)
C(12)-C(11)-C(10)	119.69(11)
C(12)-C(11)-H(11)	120.2
C(10)-C(11)-H(11)	120.2

C(11)-C(12)-C(7)	120.65(11)
C(11)-C(12)-H(12)	119.7
C(7)-C(12)-H(12)	119.7
N(2)-C(13)-C(10)	177.77(13)
O(2)-C(14)-N(1)	118.99(11)
O(2)-C(14)-C(15)	124.88(12)
N(1)-C(14)-C(15)	116.11(11)
C(14)-C(15)-H(15A)	109.5
C(14)-C(15)-H(15B)	109.5
H(15A)-C(15)-H(15B)	109.5
C(14)-C(15)-H(15C)	109.5
H(15A)-C(15)-H(15C)	109.5
H(15B)-C(15)-H(15C)	109.5
C(14)-N(1)-C(1)	132.98(10)
C(14)-N(1)-C(5)	128.16(10)
C(1)-N(1)-C(5)	94.64(9)
O(101)-C(101)-N(101)	131.57(12)
O(101)-C(101)-C(102)	136.30(12)
N(101)-C(101)-C(102)	92.11(10)
C(106)-C(102)-C(101)	118.59(12)
C(106)-C(102)-C(103)	119.67(11)
C(101)-C(102)-C(103)	109.89(10)
C(106)-C(102)-C(105)	126.92(10)
C(101)-C(102)-C(105)	86.32(9)
C(103)-C(102)-C(105)	88.07(9)
C(104)-C(103)-C(102)	91.30(9)
C(104)-C(103)-H(10C)	113.4
C(102)-C(103)-H(10C)	113.4
C(104)-C(103)-H(10D)	113.4
C(102)-C(103)-H(10D)	113.4
H(10C)-C(103)-H(10D)	110.7
C(105)-C(104)-C(103)	90.33(10)
C(105)-C(104)-H(10A)	113.6
C(103)-C(104)-H(10A)	113.6
C(105)-C(104)-H(10B)	113.6
C(103)-C(104)-H(10B)	113.6
H(10A)-C(104)-H(10B)	110.9
N(101)-C(105)-C(107)	114.59(10)
N(101)-C(105)-C(104)	111.74(9)
C(107)-C(105)-C(104)	121.79(11)

N(101)-C(105)-C(102)	86.41(9)
C(107)-C(105)-C(102)	125.29(10)
C(104)-C(105)-C(102)	90.27(9)
C(102)-C(106)-H(10E)	109.5
C(102)-C(106)-H(10F)	109.5
H(10E)-C(106)-H(10F)	109.5
C(102)-C(106)-H(10G)	109.5
H(10E)-C(106)-H(10G)	109.5
H(10F)-C(106)-H(10G)	109.5
C(108)-C(107)-C(112)	119.25(11)
C(108)-C(107)-C(105)	121.14(11)
C(112)-C(107)-C(105)	119.61(11)
C(107)-C(108)-C(109)	120.72(12)
C(107)-C(108)-H(108)	119.6
C(109)-C(108)-H(108)	119.6
C(108)-C(109)-C(110)	119.32(12)
C(108)-C(109)-H(109)	120.3
C(110)-C(109)-H(109)	120.3
C(111)-C(110)-C(109)	120.38(11)
C(111)-C(110)-C(113)	119.34(12)
C(109)-C(110)-C(113)	120.24(12)
C(112)-C(111)-C(110)	119.69(12)
C(112)-C(111)-H(111)	120.2
C(110)-C(111)-H(111)	120.2
C(111)-C(112)-C(107)	120.59(12)
C(111)-C(112)-H(112)	119.7
C(107)-C(112)-H(112)	119.7
N(102)-C(113)-C(110)	177.20(14)
O(102)-C(114)-N(101)	118.50(11)
O(102)-C(114)-C(115)	124.94(12)
N(101)-C(114)-C(115)	116.55(12)
C(114)-C(115)-H(11A)	109.5
C(114)-C(115)-H(11B)	109.5
H(11A)-C(115)-H(11B)	109.5
C(114)-C(115)-H(11C)	109.5
H(11A)-C(115)-H(11C)	109.5
H(11B)-C(115)-H(11C)	109.5
C(114)-N(101)-C(101)	135.89(11)
C(114)-N(101)-C(105)	126.90(10)
C(101)-N(101)-C(105)	95.15(9)

Supplementary Table S9. Torsion angles [°] for **6p**.

O(1)-C(1)-C(2)-C(6)	53.2(2)
N(1)-C(1)-C(2)-C(6)	-128.40(12)
O(1)-C(1)-C(2)-C(3)	-92.10(17)
N(1)-C(1)-C(2)-C(3)	86.35(11)
O(1)-C(1)-C(2)-C(5)	-178.87(15)
N(1)-C(1)-C(2)-C(5)	-0.42(8)
C(6)-C(2)-C(3)-C(4)	129.21(12)
C(1)-C(2)-C(3)-C(4)	-86.25(11)
C(5)-C(2)-C(3)-C(4)	-1.13(9)
C(2)-C(3)-C(4)-C(5)	1.16(9)
C(3)-C(4)-C(5)-N(1)	85.42(11)
C(3)-C(4)-C(5)-C(7)	-128.56(12)
C(3)-C(4)-C(5)-C(2)	-1.14(9)
C(6)-C(2)-C(5)-N(1)	122.83(12)
C(1)-C(2)-C(5)-N(1)	0.40(8)
C(3)-C(2)-C(5)-N(1)	-110.50(9)
C(6)-C(2)-C(5)-C(7)	3.52(18)
C(1)-C(2)-C(5)-C(7)	-118.91(11)
C(3)-C(2)-C(5)-C(7)	130.19(11)
C(6)-C(2)-C(5)-C(4)	-125.54(13)
C(1)-C(2)-C(5)-C(4)	112.03(9)
C(3)-C(2)-C(5)-C(4)	1.13(9)
N(1)-C(5)-C(7)-C(8)	-172.97(11)
C(4)-C(5)-C(7)-C(8)	42.57(17)
C(2)-C(5)-C(7)-C(8)	-69.87(15)
N(1)-C(5)-C(7)-C(12)	1.66(17)
C(4)-C(5)-C(7)-C(12)	-142.80(12)
C(2)-C(5)-C(7)-C(12)	104.76(14)
C(12)-C(7)-C(8)-C(9)	-3.07(19)
C(5)-C(7)-C(8)-C(9)	171.69(12)
C(7)-C(8)-C(9)-C(10)	-0.45(19)
C(8)-C(9)-C(10)-C(11)	3.10(19)
C(8)-C(9)-C(10)-C(13)	-174.59(12)
C(9)-C(10)-C(11)-C(12)	-2.18(19)
C(13)-C(10)-C(11)-C(12)	175.55(12)
C(10)-C(11)-C(12)-C(7)	-1.4(2)
C(8)-C(7)-C(12)-C(11)	4.01(19)
C(5)-C(7)-C(12)-C(11)	-170.65(12)

O(2)-C(14)-N(1)-C(1)	160.89(12)
C(15)-C(14)-N(1)-C(1)	-17.57(18)
O(2)-C(14)-N(1)-C(5)	10.09(18)
C(15)-C(14)-N(1)-C(5)	-168.36(11)
O(1)-C(1)-N(1)-C(14)	21.7(2)
C(2)-C(1)-N(1)-C(14)	-156.91(12)
O(1)-C(1)-N(1)-C(5)	179.02(13)
C(2)-C(1)-N(1)-C(5)	0.45(9)
C(7)-C(5)-N(1)-C(14)	-78.37(15)
C(4)-C(5)-N(1)-C(14)	69.94(15)
C(2)-C(5)-N(1)-C(14)	158.58(11)
C(7)-C(5)-N(1)-C(1)	122.61(11)
C(4)-C(5)-N(1)-C(1)	-89.07(11)
C(2)-C(5)-N(1)-C(1)	-0.43(9)
O(101)-C(101)-C(102)-C(106)	51.3(2)
N(101)-C(101)-C(102)-C(106)	-130.18(11)
O(101)-C(101)-C(102)-C(103)	-91.51(18)
N(101)-C(101)-C(102)-C(103)	87.01(11)
O(101)-C(101)-C(102)-C(105)	-178.12(16)
N(101)-C(101)-C(102)-C(105)	0.40(9)
C(106)-C(102)-C(103)-C(104)	130.97(12)
C(101)-C(102)-C(103)-C(104)	-86.69(11)
C(105)-C(102)-C(103)-C(104)	-1.30(9)
C(102)-C(103)-C(104)-C(105)	1.34(9)
C(103)-C(104)-C(105)-N(101)	84.93(11)
C(103)-C(104)-C(105)-C(107)	-134.36(11)
C(103)-C(104)-C(105)-C(102)	-1.31(9)
C(106)-C(102)-C(105)-N(101)	123.09(13)
C(101)-C(102)-C(105)-N(101)	-0.38(8)
C(103)-C(102)-C(105)-N(101)	-110.46(9)
C(106)-C(102)-C(105)-C(107)	5.3(2)
C(101)-C(102)-C(105)-C(107)	-118.18(12)
C(103)-C(102)-C(105)-C(107)	131.74(12)
C(106)-C(102)-C(105)-C(104)	-125.15(13)
C(101)-C(102)-C(105)-C(104)	111.38(9)
C(103)-C(102)-C(105)-C(104)	1.30(9)
N(101)-C(105)-C(107)-C(108)	128.44(12)
C(104)-C(105)-C(107)-C(108)	-11.26(17)
C(102)-C(105)-C(107)-C(108)	-127.71(13)
N(101)-C(105)-C(107)-C(112)	-51.71(14)

C(104)-C(105)-C(107)-C(112)	168.59(11)
C(102)-C(105)-C(107)-C(112)	52.15(16)
C(112)-C(107)-C(108)-C(109)	-1.66(17)
C(105)-C(107)-C(108)-C(109)	178.20(11)
C(107)-C(108)-C(109)-C(110)	-0.23(18)
C(108)-C(109)-C(110)-C(111)	1.96(18)
C(108)-C(109)-C(110)-C(113)	-176.03(11)
C(109)-C(110)-C(111)-C(112)	-1.76(18)
C(113)-C(110)-C(111)-C(112)	176.25(11)
C(110)-C(111)-C(112)-C(107)	-0.17(18)
C(108)-C(107)-C(112)-C(111)	1.87(18)
C(105)-C(107)-C(112)-C(111)	-177.99(11)
O(102)-C(114)-N(101)-C(101)	166.89(13)
C(115)-C(114)-N(101)-C(101)	-14.4(2)
O(102)-C(114)-N(101)-C(105)	7.50(18)
C(115)-C(114)-N(101)-C(105)	-173.77(11)
O(101)-C(101)-N(101)-C(114)	14.6(2)
C(102)-C(101)-N(101)-C(114)	-164.02(14)
O(101)-C(101)-N(101)-C(105)	178.20(14)
C(102)-C(101)-N(101)-C(105)	-0.43(9)
C(107)-C(105)-N(101)-C(114)	-66.39(15)
C(104)-C(105)-N(101)-C(114)	77.32(14)
C(102)-C(105)-N(101)-C(114)	166.17(11)
C(107)-C(105)-N(101)-C(101)	127.85(11)
C(104)-C(105)-N(101)-C(101)	-88.44(11)
C(102)-C(105)-N(101)-C(101)	0.41(9)

4.4 Structure Report for 8a

The crystallographic data for **8a** have been deposited at the Cambridge Crystallographic Data Centre under deposition number CCDC 2520222. Copies of the data can be obtained free of charge via www.ccdc.cam.ac.uk/structures.

Crystallographic data are presented in the Tables below. A single crystal of **8a** was coated in perfluoropolyether oil and mounted on a glass fibre. X-ray measurements were made using a Bruker D8 Venture dual source kappa CPAD diffractometer with Mo K_{α} radiation (λ = 0.71073 Å).[21] Intensities were integrated from several series of exposures, each exposure covering 0.5° in ω or ϕ. Absorption corrections were applied, based on multiple and symmetry-equivalent measurements.[22] The structure was solved by direct methods and refined by least squares on weighted F^2 values for all reflections (see **Table S10**).[23]

All non-hydrogen atoms were assigned anisotropic displacement parameters and refined without positional constraints. The positions of the methyl hydrogen atoms were assigned by a rotating group refinement with fixed, idealised C-H distances. All other hydrogen atoms were constrained to ideal geometries. All hydrogen atoms were assigned isotropic displacement parameters equal to 1.5 times (methyl) or 1.2 times (all other hydrogen atoms) that of their parent atom.

Refinement proceeded smoothly to give the residuals shown in **Table S10**. Complex neutral-atom scattering factors were used.[24]

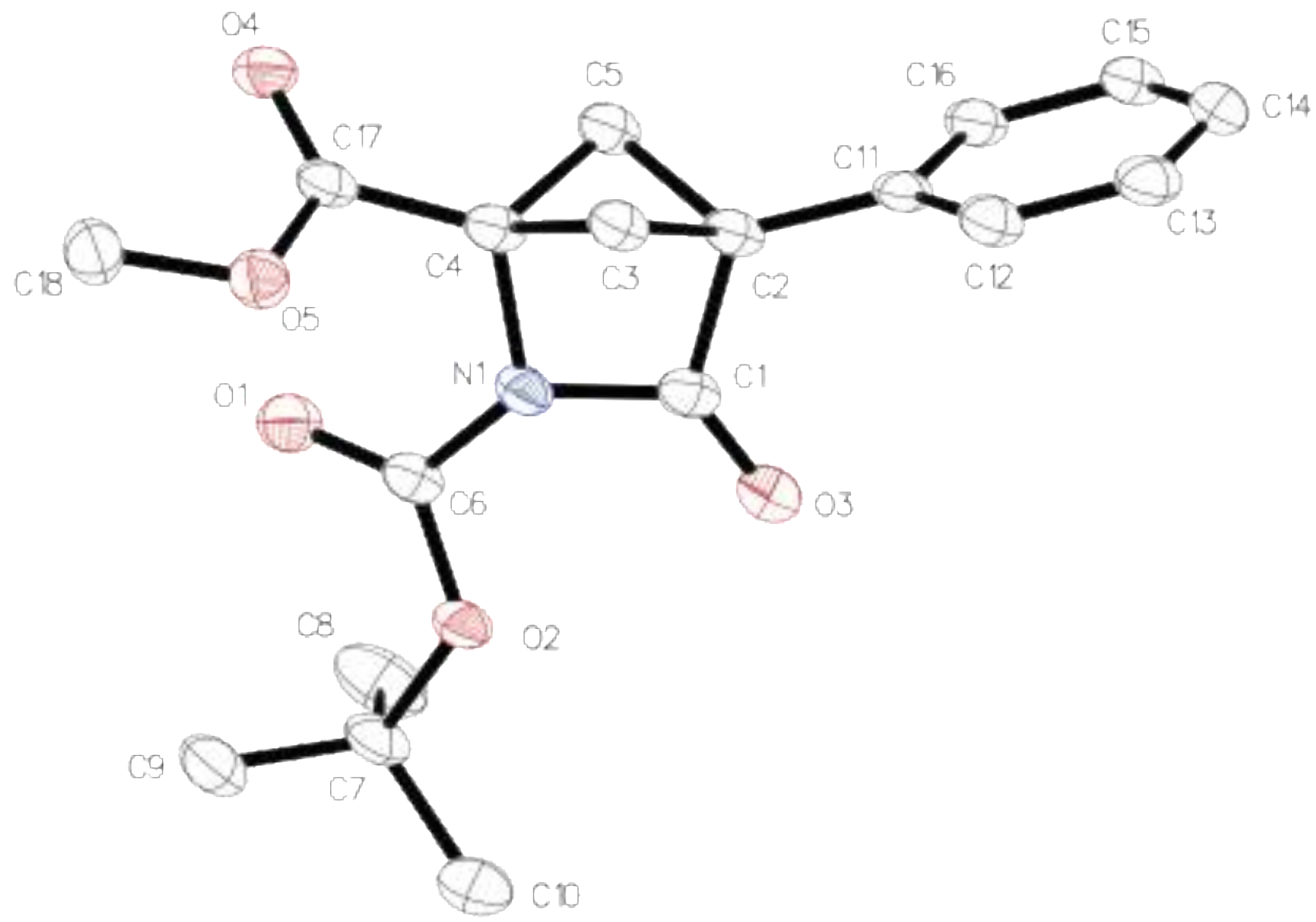

Supplementary Figure S10: Molecular structure of **8a** with thermal ellipsoids shown at the 50% probability level; hydrogen atoms have been omitted for clarity

Supplementary Table S10. Crystal data and structure refinement for **8a**.

Identification code	zzx606	
Empirical formula	$C_{18}H_{21}NO_5$	
Formula weight	331.36	
Temperature	100(2) K	
Wavelength	0.71073 Å	
Crystal system	Monoclinic	
Space group	$P2_1/n$	
Unit cell dimensions	a = 5.986(8) Å	α = 90°
	b = 30.35(4) Å	β = 98.61(3)°
	c = 9.570(14) Å	γ = 90°
Volume	1719(4) Å^3	
Z	4	
Density (calculated)	1.280 Mg/m^3	
Absorption coefficient	0.094 mm^{-1}	
F(000)	704	
Crystal size	0.200 x 0.100 x 0.020 mm	
θ range for data collection	2.255 to 24.998°	
Index ranges	$-7<=h<=7$, $-36<=k<=36$, $-11<=l<=11$	
Reflections collected	31906	
Independent reflections	3026 [R_{int} = 0.0839]	
Completeness to θ = 24.998°	100.0 %	
Absorption correction	Semi-empirical from equivalents	
Max. and min. transmission	0.9590 and 0.8116	
Refinement method	Full-matrix least-squares on F^2	
Data / restraints / parameters	3026 / 0 / 221	
Goodness-of-fit on F^2	S = 1.076	
R indices [for 2236 reflections with $I>2\sigma(I)$]		R_1 = 0.0488, wR_2 = 0.1262
R indices (for all 3026 data)	R_1 = 0.0725, wR_2 = 0.1408	
Weighting scheme	$w^{-1} = \sigma^2(F_o^2) + (aP)^2 + (bP)$,	
	where $P = [\max(F_o^2, 0) + 2F_c^2]/3$	
	a = 0.0759, b = 0.3963	
Largest diff. peak and hole	0.211 and -0.418 eÅ^{-3}	

Supplementary Table S11. Bond lengths [Å] and angles [°] for **8a**.

C(1)-C(2)	1.545(3)
C(1)-N(1)	1.427(3)
C(1)-O(3)	1.215(3)
C(2)-C(3)	1.576(3)
C(2)-C(5)	1.589(3)
C(2)-C(11)	1.500(3)
C(3)-H(3A)	0.9900
C(3)-H(3B)	0.9900
C(3)-C(4)	1.559(3)
C(4)-C(5)	1.549(3)
C(4)-C(17)	1.513(4)
C(4)-N(1)	1.509(3)
C(5)-H(5A)	0.9900
C(5)-H(5B)	0.9900
C(6)-N(1)	1.403(3)
C(6)-O(1)	1.218(3)
C(6)-O(2)	1.333(3)
C(7)-C(8)	1.525(3)
C(7)-C(9)	1.521(3)
C(7)-C(10)	1.531(4)
C(7)-O(2)	1.506(3)
C(8)-H(8A)	0.9800
C(8)-H(8B)	0.9800
C(8)-H(8C)	0.9800
C(9)-H(9A)	0.9800
C(9)-H(9B)	0.9800
C(9)-H(9C)	0.9800
C(10)-H(10A)	0.9800
C(10)-H(10B)	0.9800
C(10)-H(10C)	0.9800
C(11)-C(12)	1.401(3)
C(11)-C(16)	1.404(3)
C(12)-H(12)	0.9500
C(12)-C(13)	1.395(3)
C(13)-H(13)	0.9500
C(13)-C(14)	1.399(3)
C(14)-H(14)	0.9500
C(14)-C(15)	1.396(4)

C(15)-H(15)	0.9500
C(15)-C(16)	1.394(3)
C(16)-H(16)	0.9500
C(17)-O(4)	1.210(3)
C(17)-O(5)	1.348(3)
C(18)-H(18A)	0.9800
C(18)-H(18B)	0.9800
C(18)-H(18C)	0.9800
C(18)-O(5)	1.463(3)

N(1)-C(1)-C(2)	101.4(2)
O(3)-C(1)-C(2)	130.44(19)
O(3)-C(1)-N(1)	128.10(19)
C(1)-C(2)-C(3)	99.68(18)
C(1)-C(2)-C(5)	98.88(18)
C(3)-C(2)-C(5)	85.60(17)
C(11)-C(2)-C(1)	118.2(2)
C(11)-C(2)-C(3)	124.41(18)
C(11)-C(2)-C(5)	122.91(18)
C(2)-C(3)-H(3A)	115.0
C(2)-C(3)-H(3B)	115.0
H(3A)-C(3)-H(3B)	112.0
C(4)-C(3)-C(2)	81.95(15)
C(4)-C(3)-H(3A)	115.0
C(4)-C(3)-H(3B)	115.0
C(5)-C(4)-C(3)	87.60(15)
C(17)-C(4)-C(3)	122.84(18)
C(17)-C(4)-C(5)	122.99(18)
N(1)-C(4)-C(3)	98.95(16)
N(1)-C(4)-C(5)	102.59(19)
N(1)-C(4)-C(17)	116.13(18)
C(2)-C(5)-H(5A)	115.0
C(2)-C(5)-H(5B)	115.0
C(4)-C(5)-C(2)	81.85(16)
C(4)-C(5)-H(5A)	115.0
C(4)-C(5)-H(5B)	115.0
H(5A)-C(5)-H(5B)	112.0
O(1)-C(6)-N(1)	122.2(2)
O(1)-C(6)-O(2)	128.07(19)
O(2)-C(6)-N(1)	109.67(18)

C(8)-C(7)-C(10)	112.1(2)
C(9)-C(7)-C(8)	113.0(2)
C(9)-C(7)-C(10)	110.3(2)
O(2)-C(7)-C(8)	108.47(19)
O(2)-C(7)-C(9)	110.52(19)
O(2)-C(7)-C(10)	101.81(18)
C(7)-C(8)-H(8A)	109.5
C(7)-C(8)-H(8B)	109.5
C(7)-C(8)-H(8C)	109.5
H(8A)-C(8)-H(8B)	109.5
H(8A)-C(8)-H(8C)	109.5
H(8B)-C(8)-H(8C)	109.5
C(7)-C(9)-H(9A)	109.5
C(7)-C(9)-H(9B)	109.5
C(7)-C(9)-H(9C)	109.5
H(9A)-C(9)-H(9B)	109.5
H(9A)-C(9)-H(9C)	109.5
H(9B)-C(9)-H(9C)	109.5
C(7)-C(10)-H(10A)	109.5
C(7)-C(10)-H(10B)	109.5
C(7)-C(10)-H(10C)	109.5
H(10A)-C(10)-H(10B)	109.5
H(10A)-C(10)-H(10C)	109.5
H(10B)-C(10)-H(10C)	109.5
C(12)-C(11)-C(2)	121.45(19)
C(12)-C(11)-C(16)	118.86(18)
C(16)-C(11)-C(2)	119.68(19)
C(11)-C(12)-H(12)	119.8
C(13)-C(12)-C(11)	120.4(2)
C(13)-C(12)-H(12)	119.8
C(12)-C(13)-H(13)	119.9
C(12)-C(13)-C(14)	120.3(2)
C(14)-C(13)-H(13)	119.9
C(13)-C(14)-H(14)	120.2
C(15)-C(14)-C(13)	119.61(19)
C(15)-C(14)-H(14)	120.2
C(14)-C(15)-H(15)	119.9
C(16)-C(15)-C(14)	120.1(2)
C(16)-C(15)-H(15)	119.9
C(11)-C(16)-H(16)	119.7

C(15)-C(16)-C(11)	120.7(2)
C(15)-C(16)-H(16)	119.7
O(4)-C(17)-C(4)	123.85(19)
O(4)-C(17)-O(5)	125.4(2)
O(5)-C(17)-C(4)	110.70(17)
H(18A)-C(18)-H(18B)	109.5
H(18A)-C(18)-H(18C)	109.5
H(18B)-C(18)-H(18C)	109.5
O(5)-C(18)-H(18A)	109.5
O(5)-C(18)-H(18B)	109.5
O(5)-C(18)-H(18C)	109.5
C(1)-N(1)-C(4)	102.27(18)
C(6)-N(1)-C(1)	128.19(19)
C(6)-N(1)-C(4)	124.02(17)
C(6)-O(2)-C(7)	121.02(16)
C(17)-O(5)-C(18)	115.34(16)

5. NMR Spectra

5.1 NMR Spectra of Cyclisation Precursors (1)

^{1}H NMR (500 MHz, $CDCl_3$) of **1a**

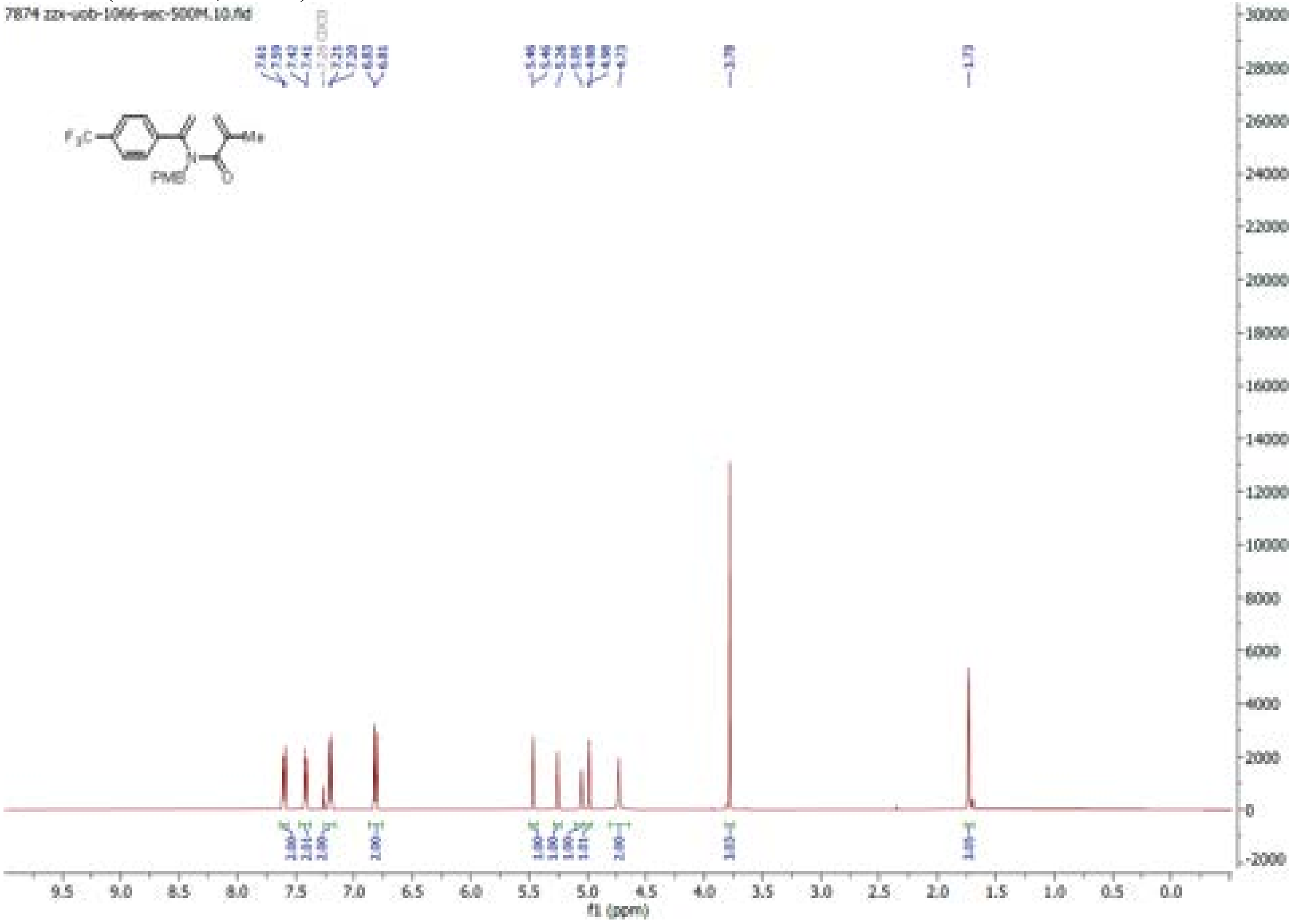

^{13}C NMR (126 MHz, $CDCl_3$) of **1a**

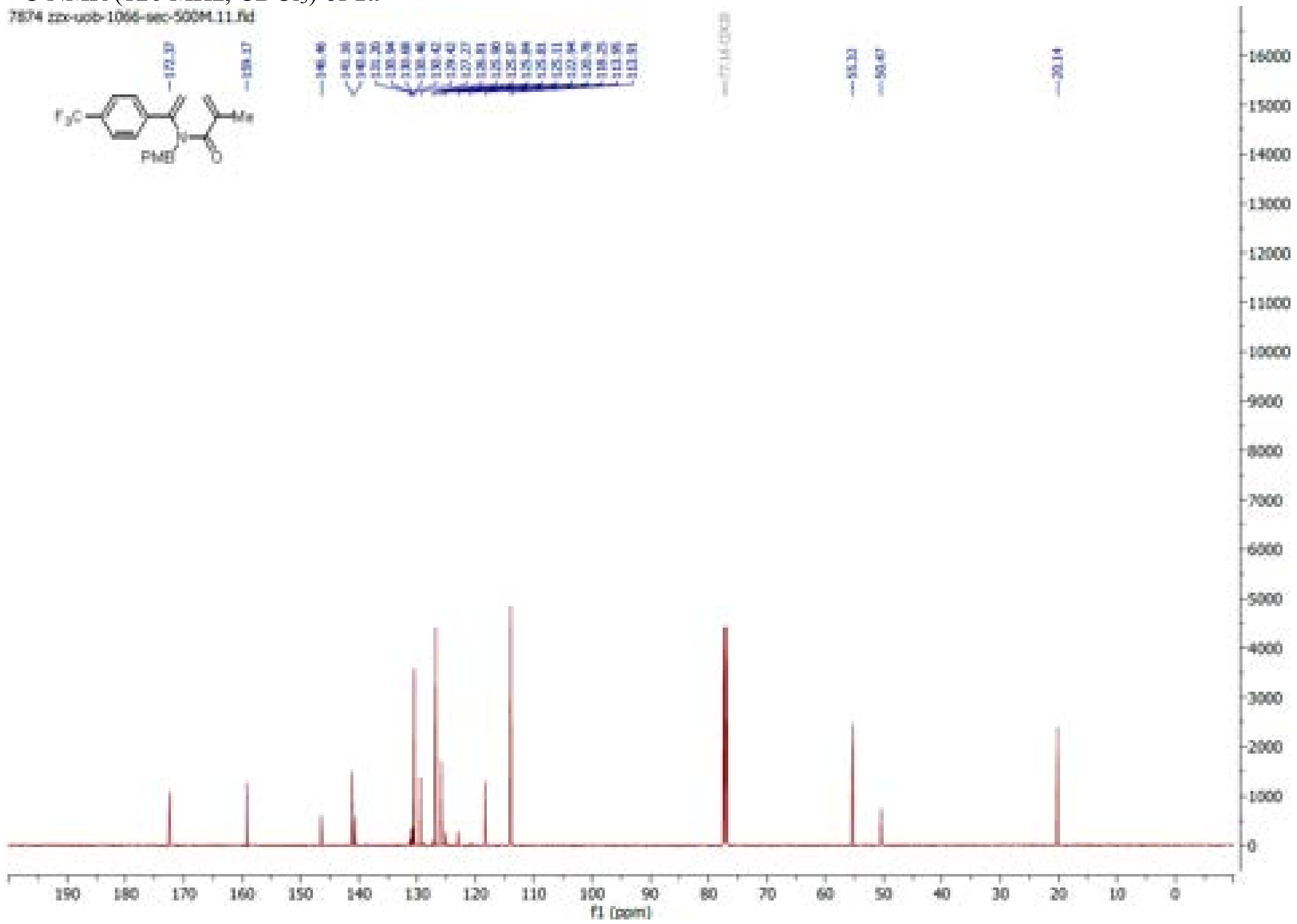

^{19}F NMR (471 MHz, $CDCl_3$) of **1a**

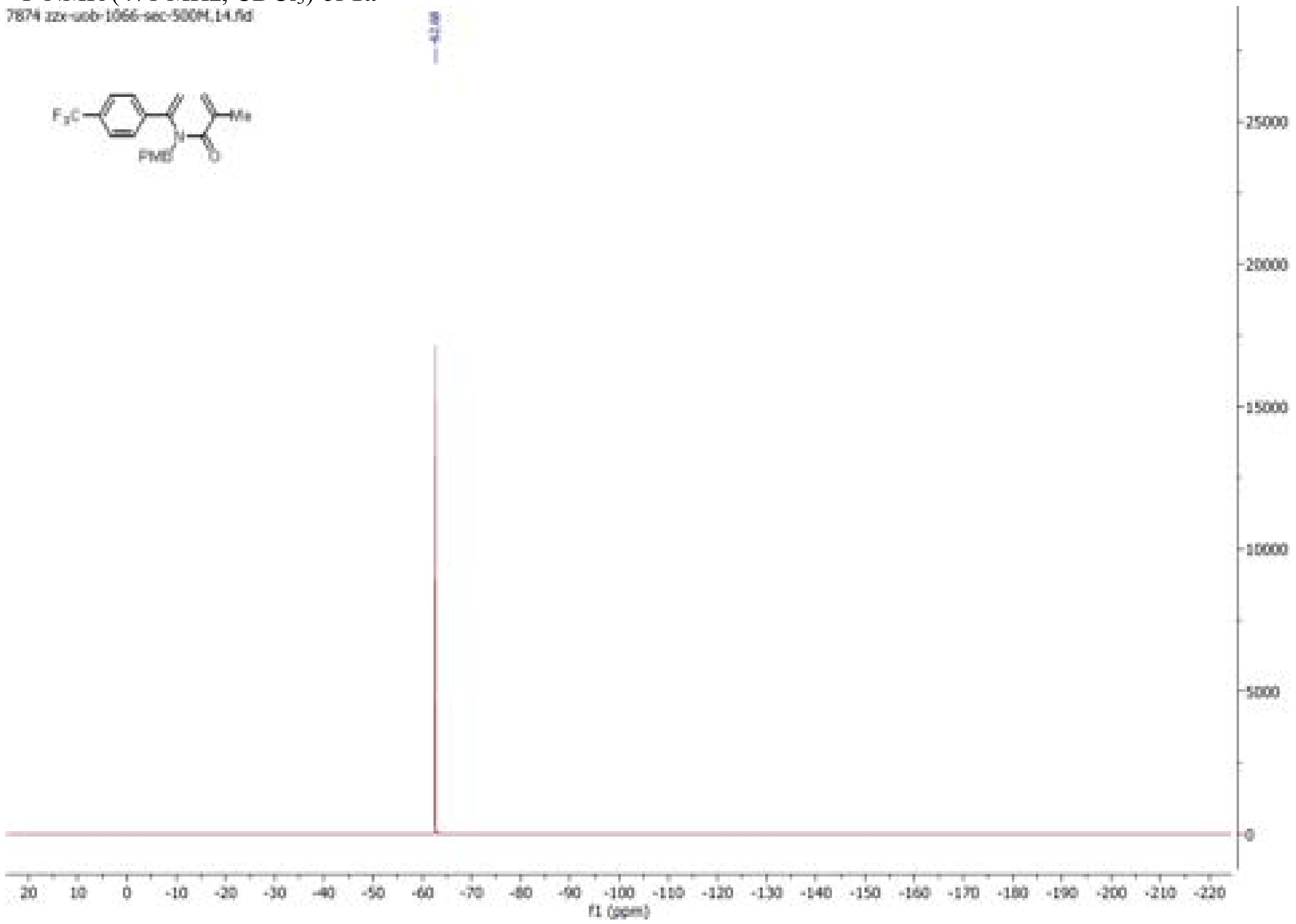

^{1}H NMR (500 MHz, $CDCl_3$) of **1b**

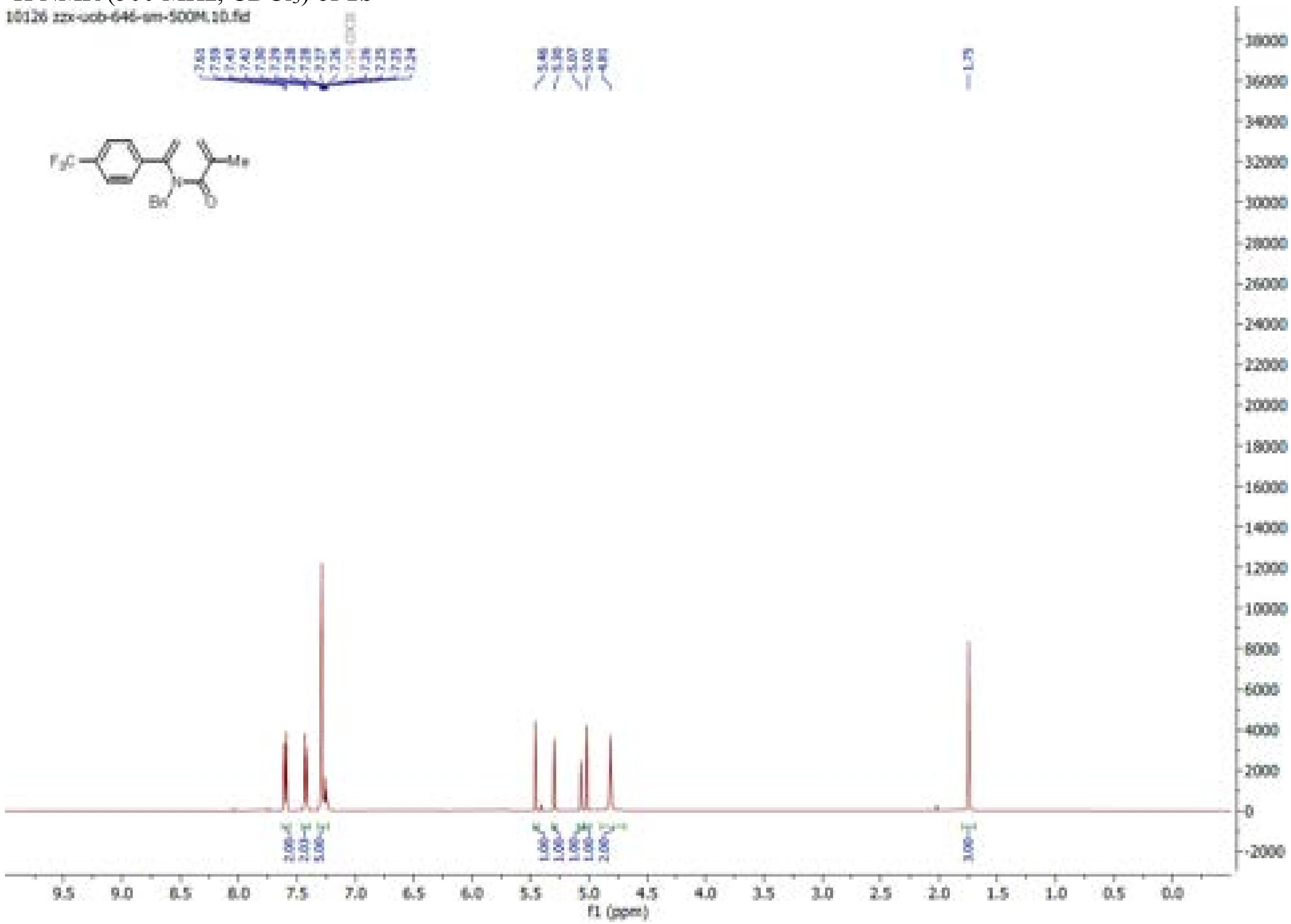

^{13}C NMR (126 MHz, $CDCl_3$) of **1b**

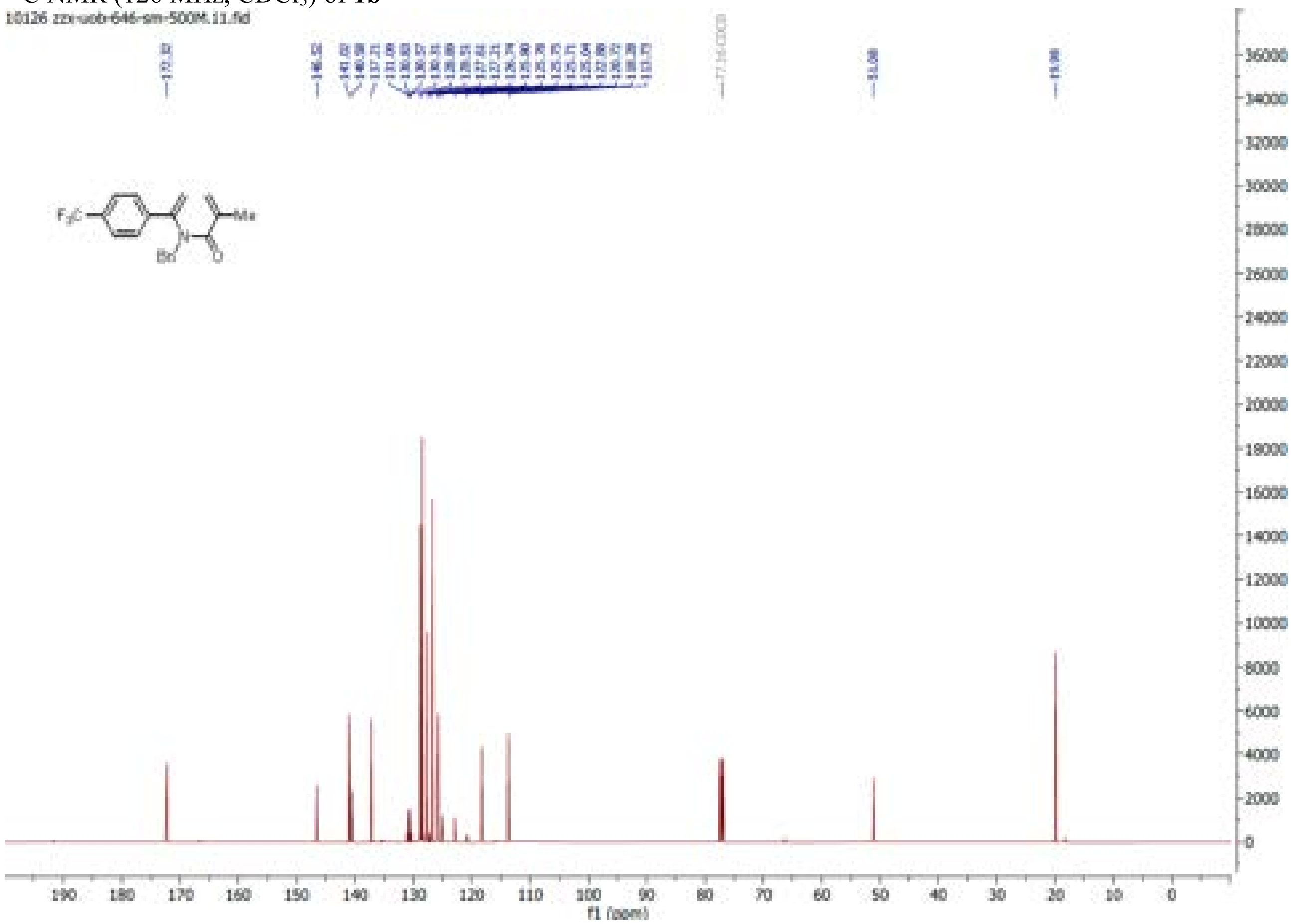

^{19}F NMR (471 MHz, $CDCl_3$) of **1b**

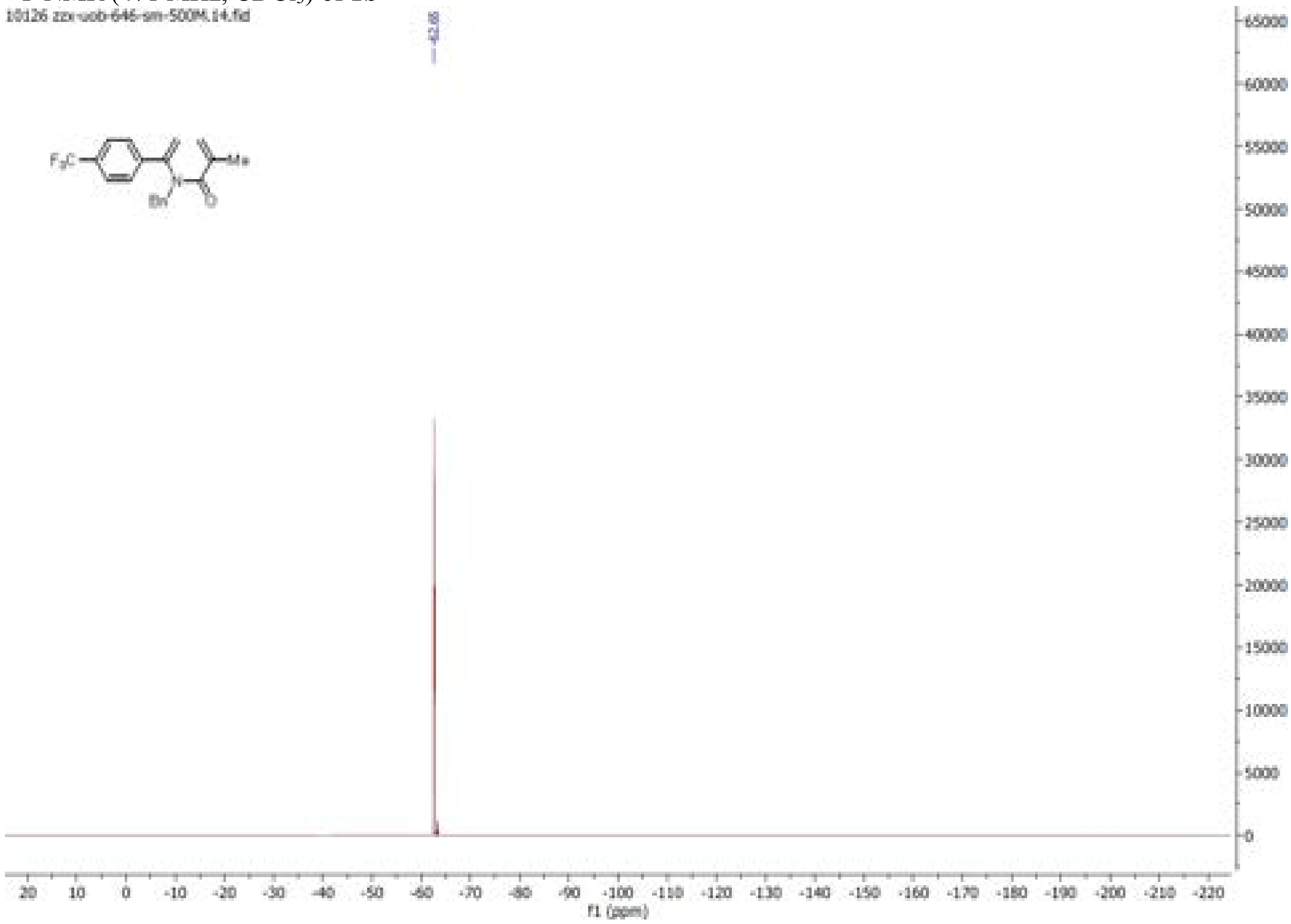

^{1}H NMR (500 MHz, $CDCl_3$) of **1c**

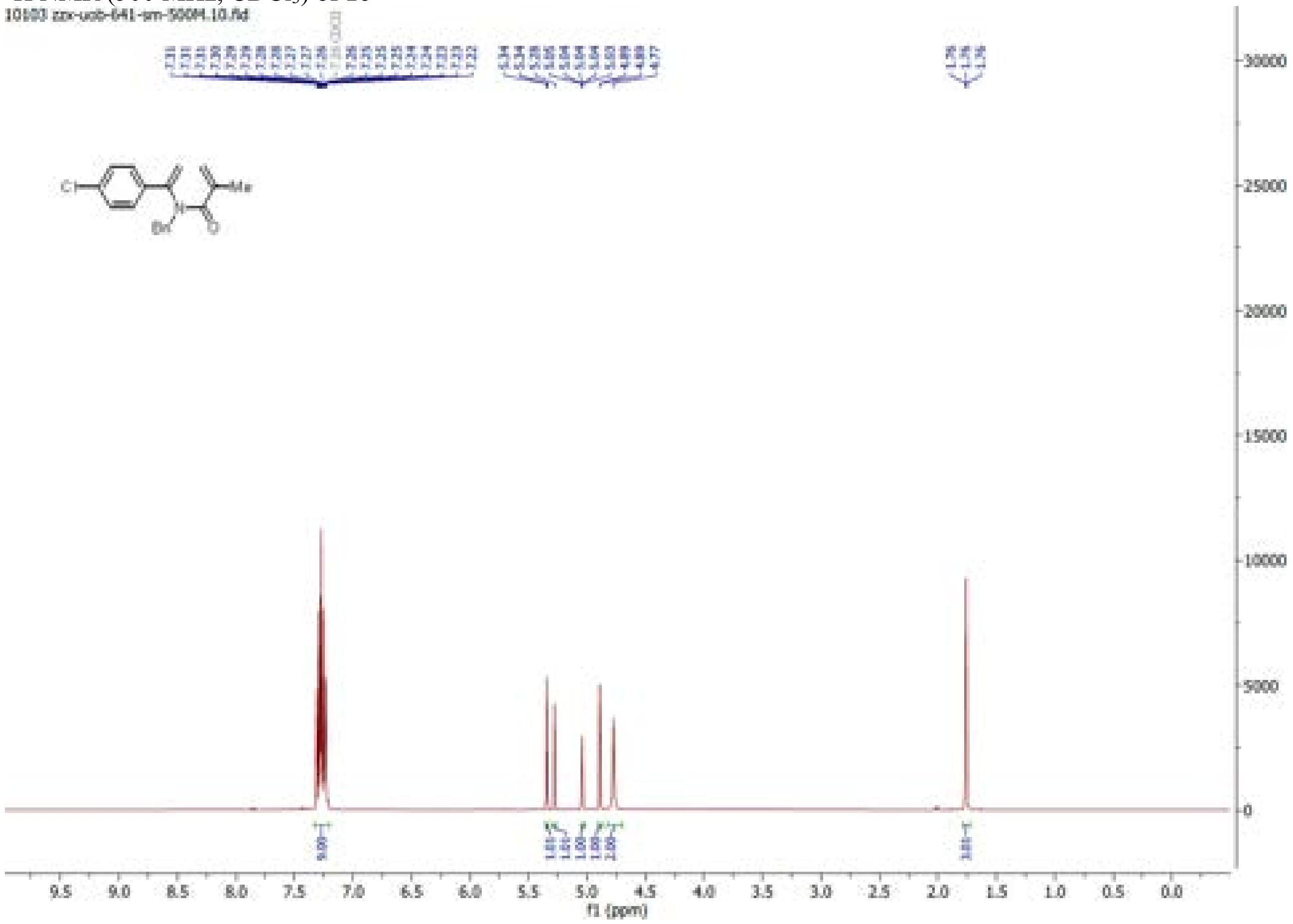

^{13}C NMR (126 MHz, $CDCl_3$) of **1c**

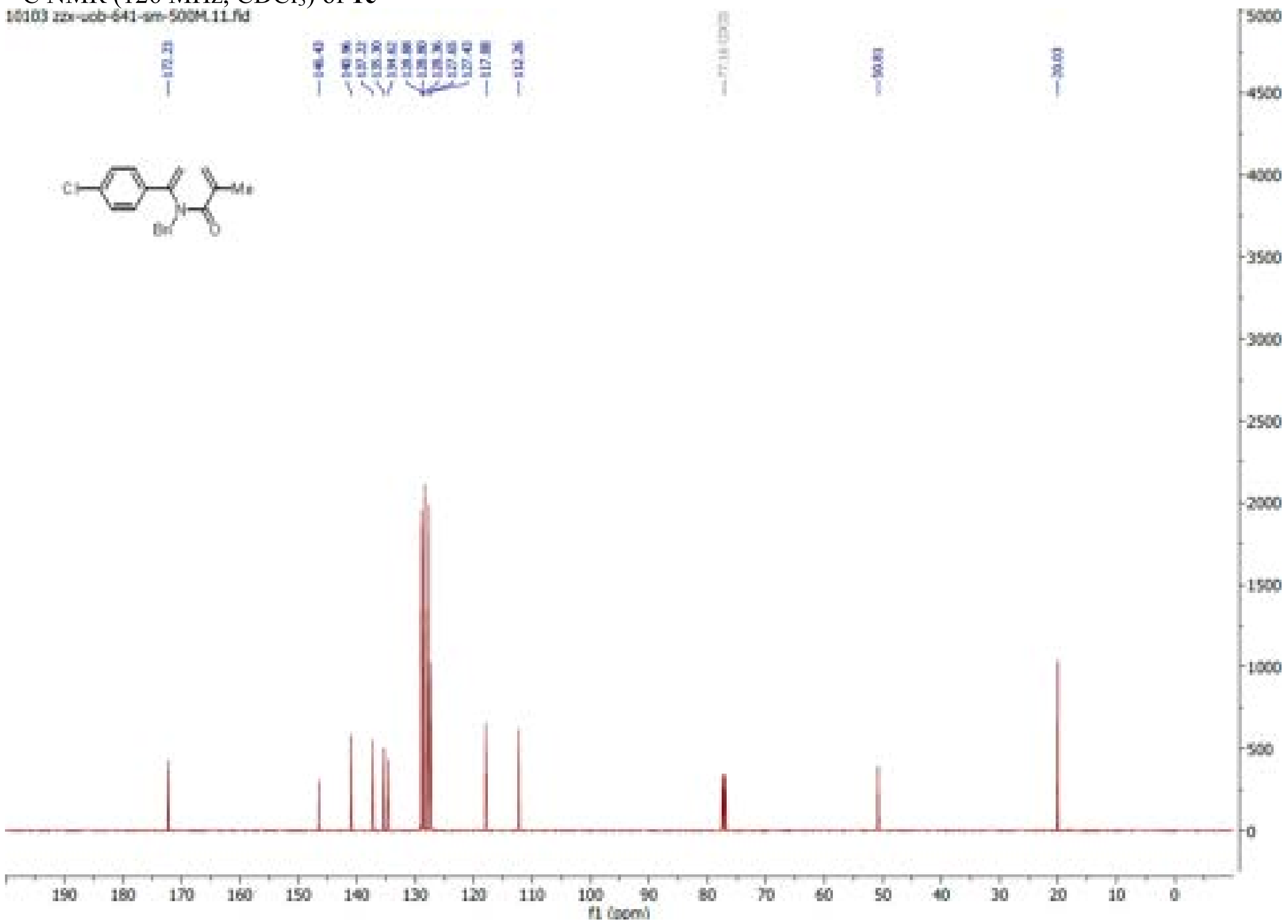

^{1}H NMR (500 MHz, $CDCl_3$) of **1d**

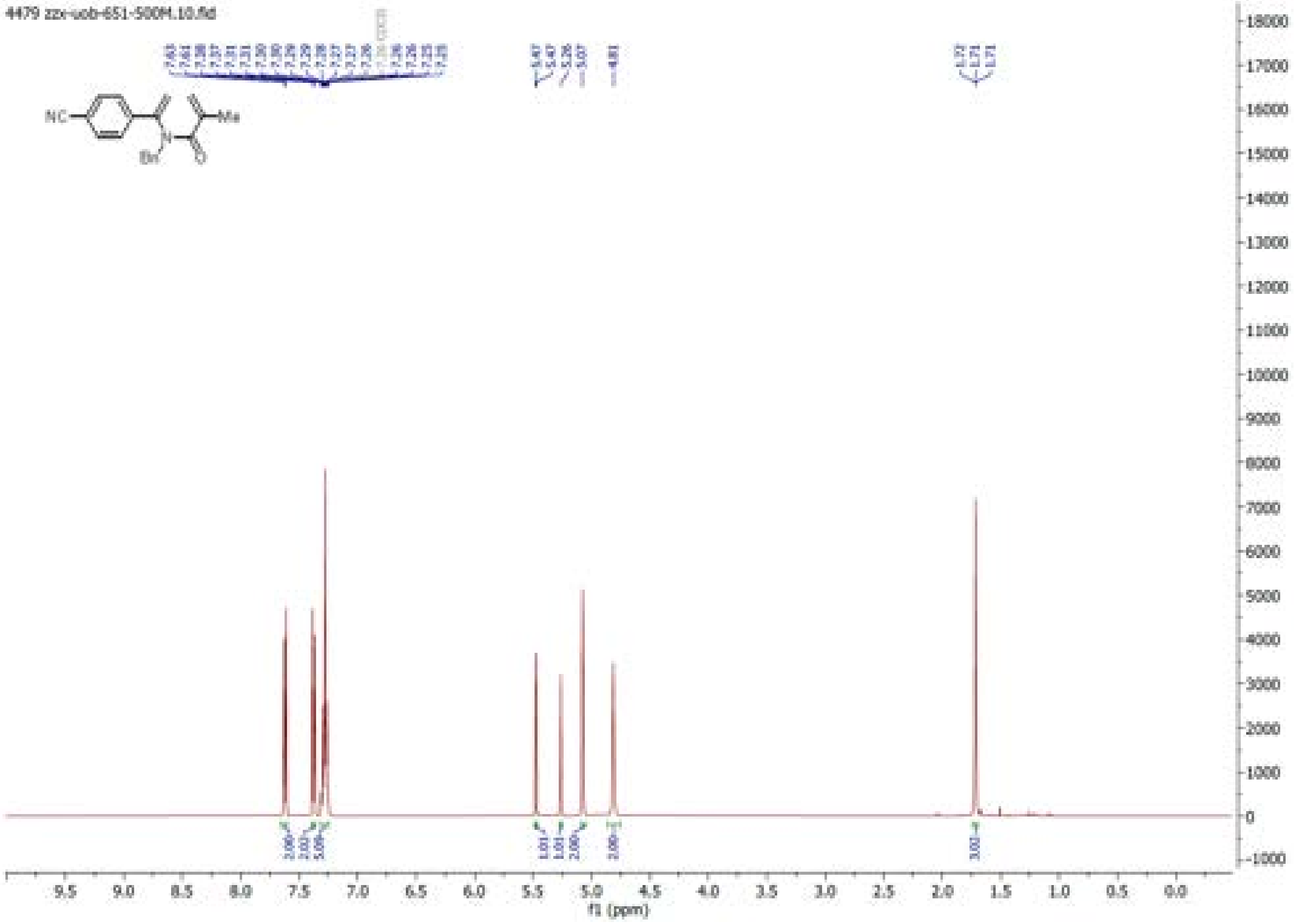

^{13}C NMR (126 MHz, $CDCl_3$) of **1d**

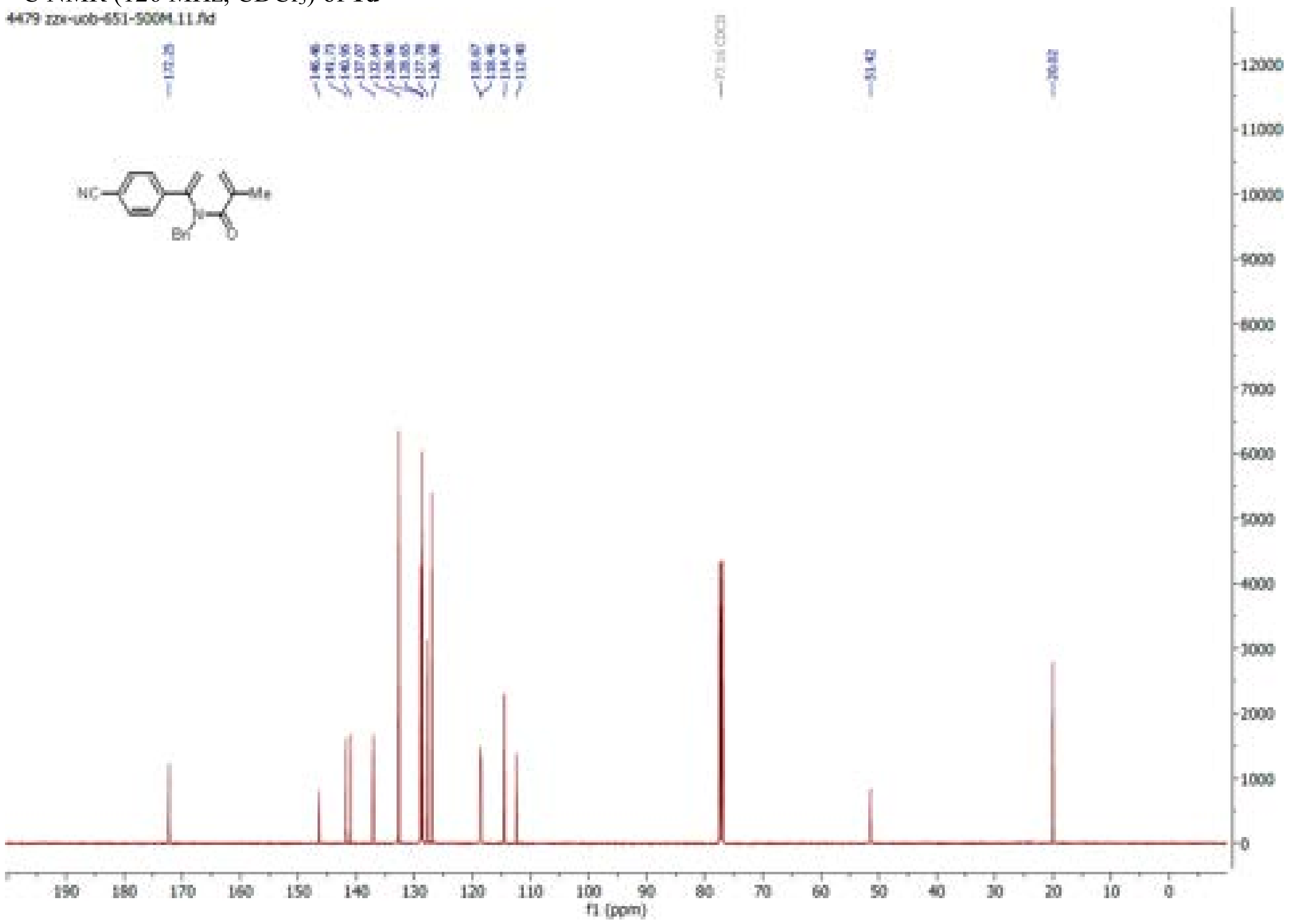

^{1}H NMR (500 MHz, $CDCl_3$) of **1e**

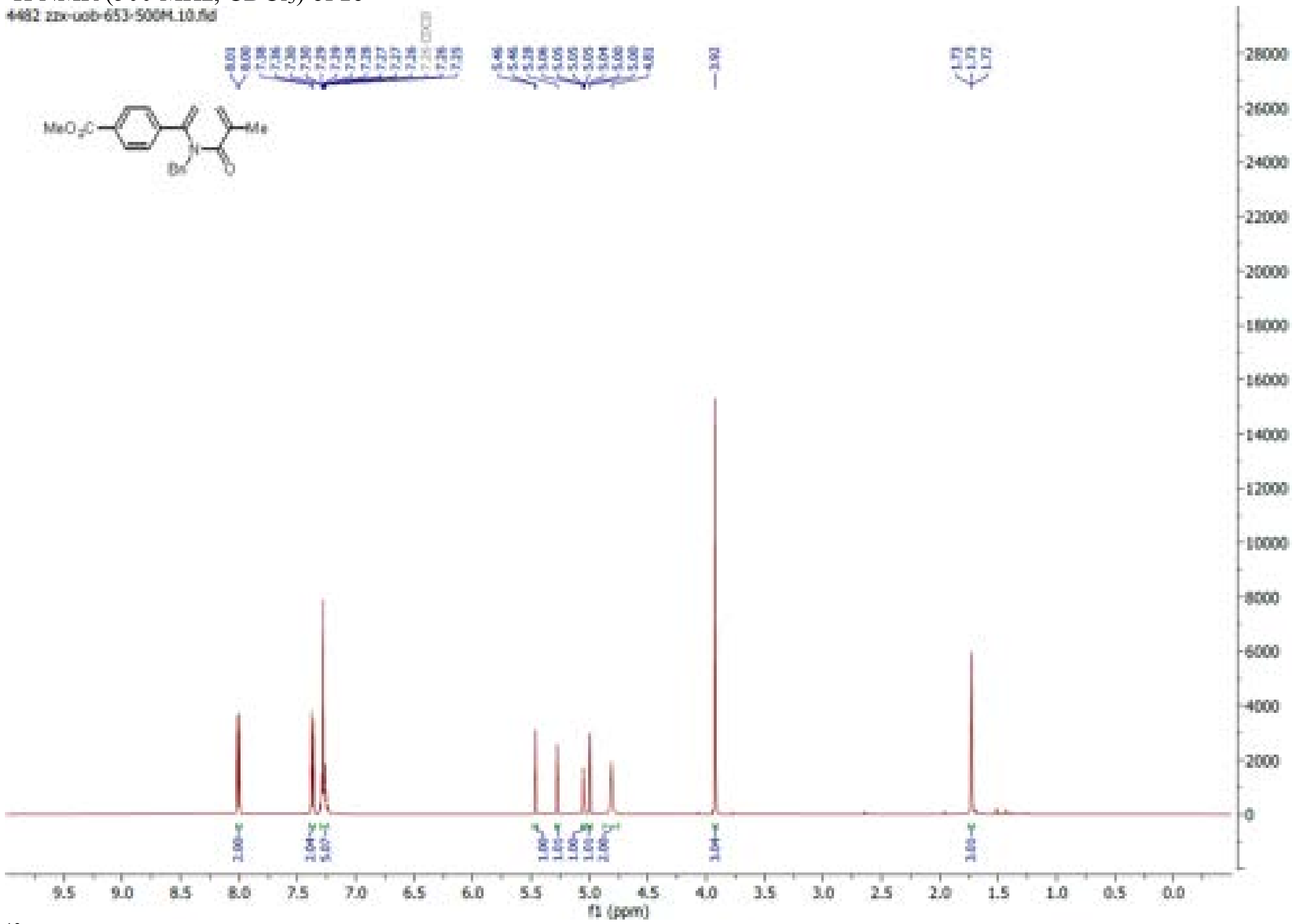

^{13}C NMR (126 MHz, $CDCl_3$) of **1e**

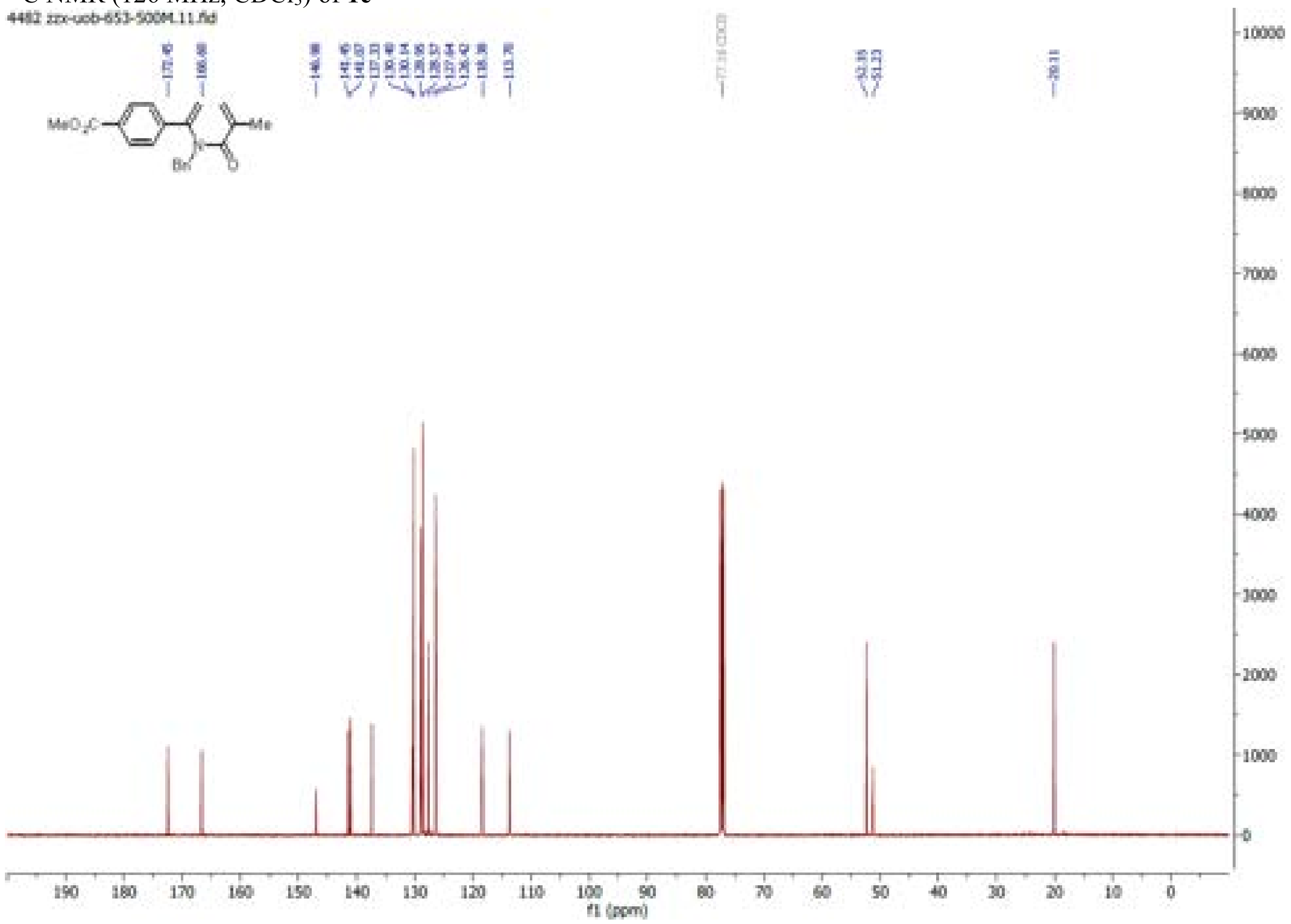

^{1}H NMR (500 MHz, $CDCl_3$) of **1f**

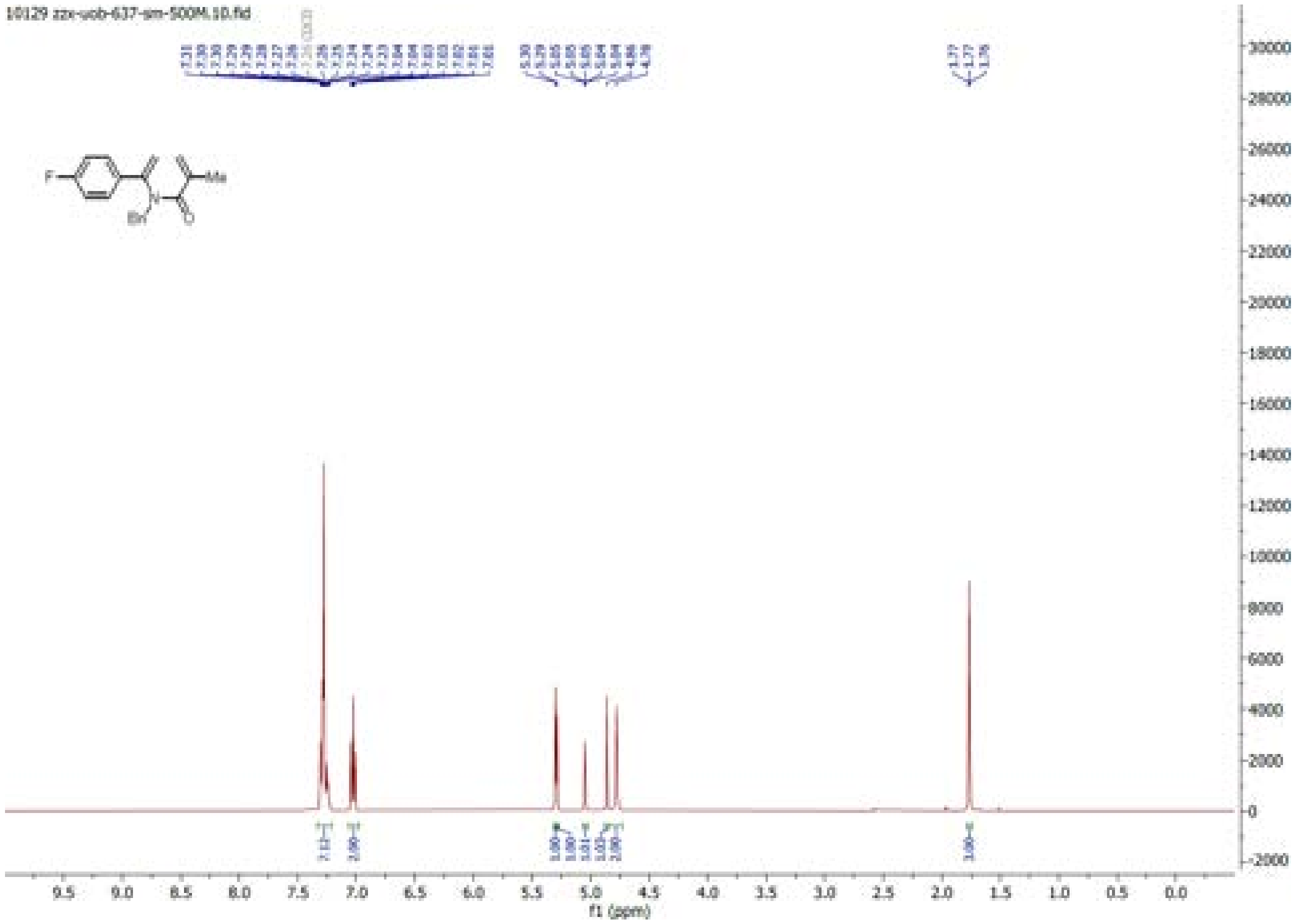

^{13}C NMR (126 MHz, $CDCl_3$) of **1f**

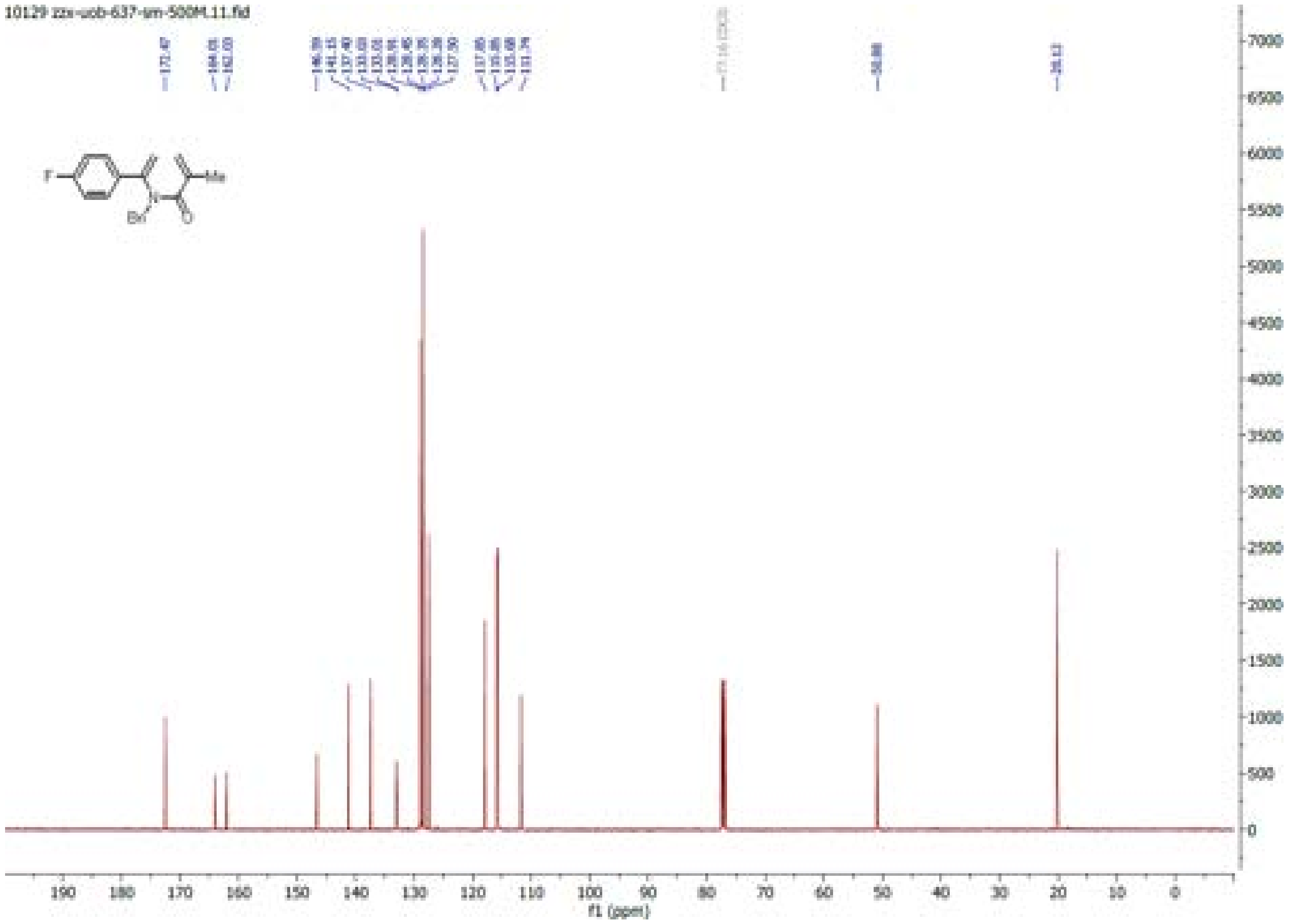

^{19}F NMR (471 MHz, $CDCl_3$) of **1f**

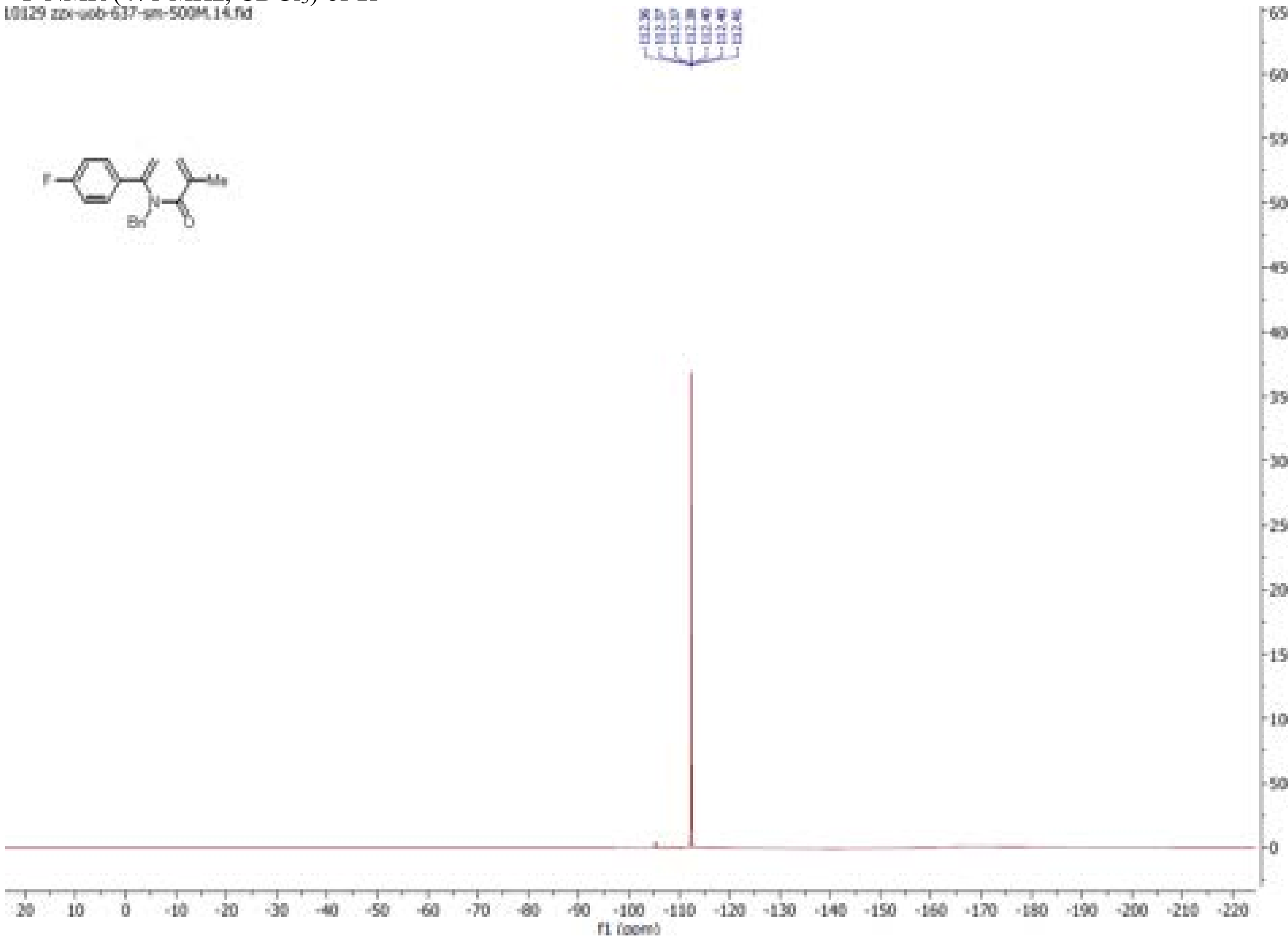

^{1}H NMR (500 MHz, $CDCl_3$) of **1g**

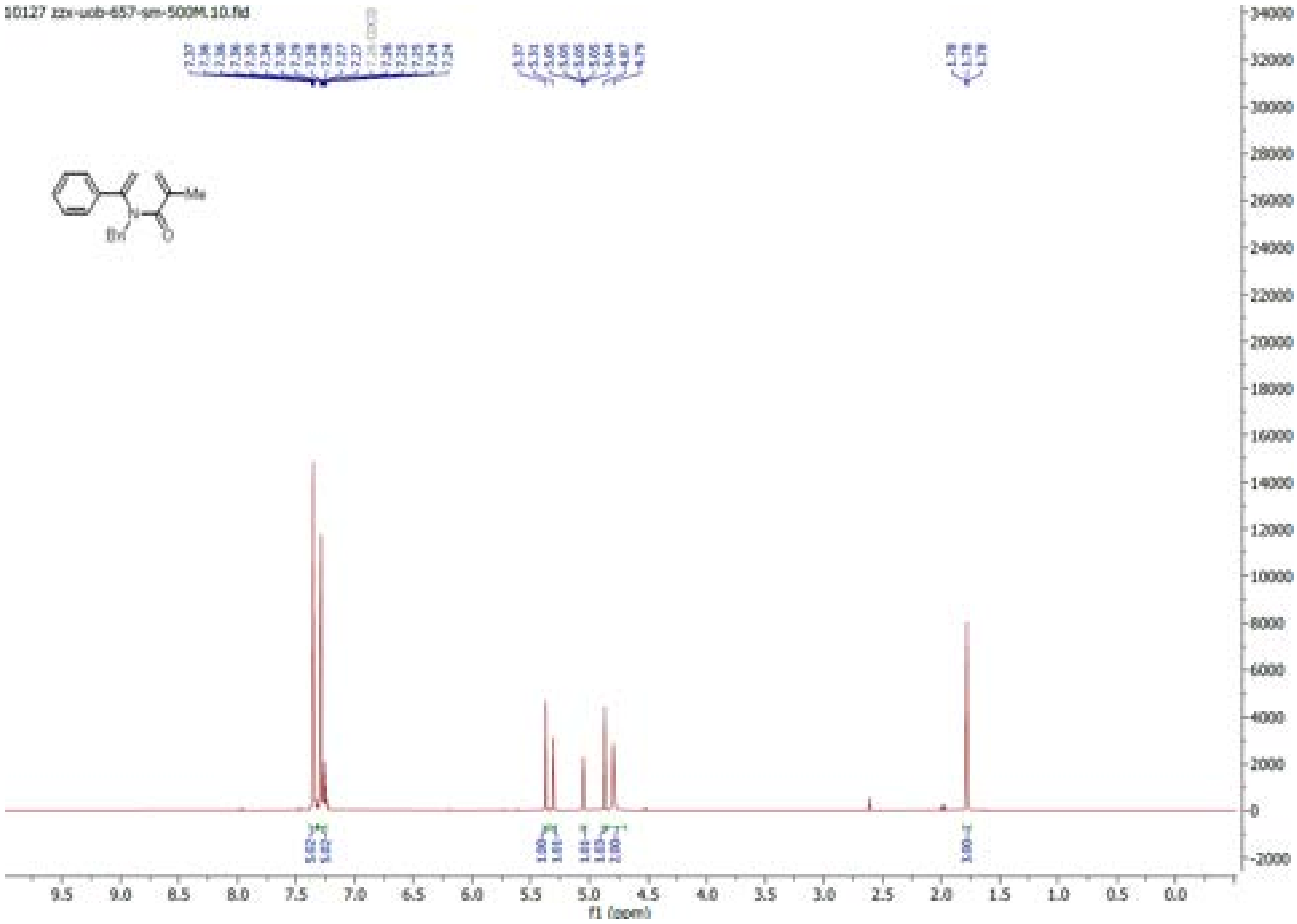

^{13}C NMR (126 MHz, $CDCl_3$) of **1g**

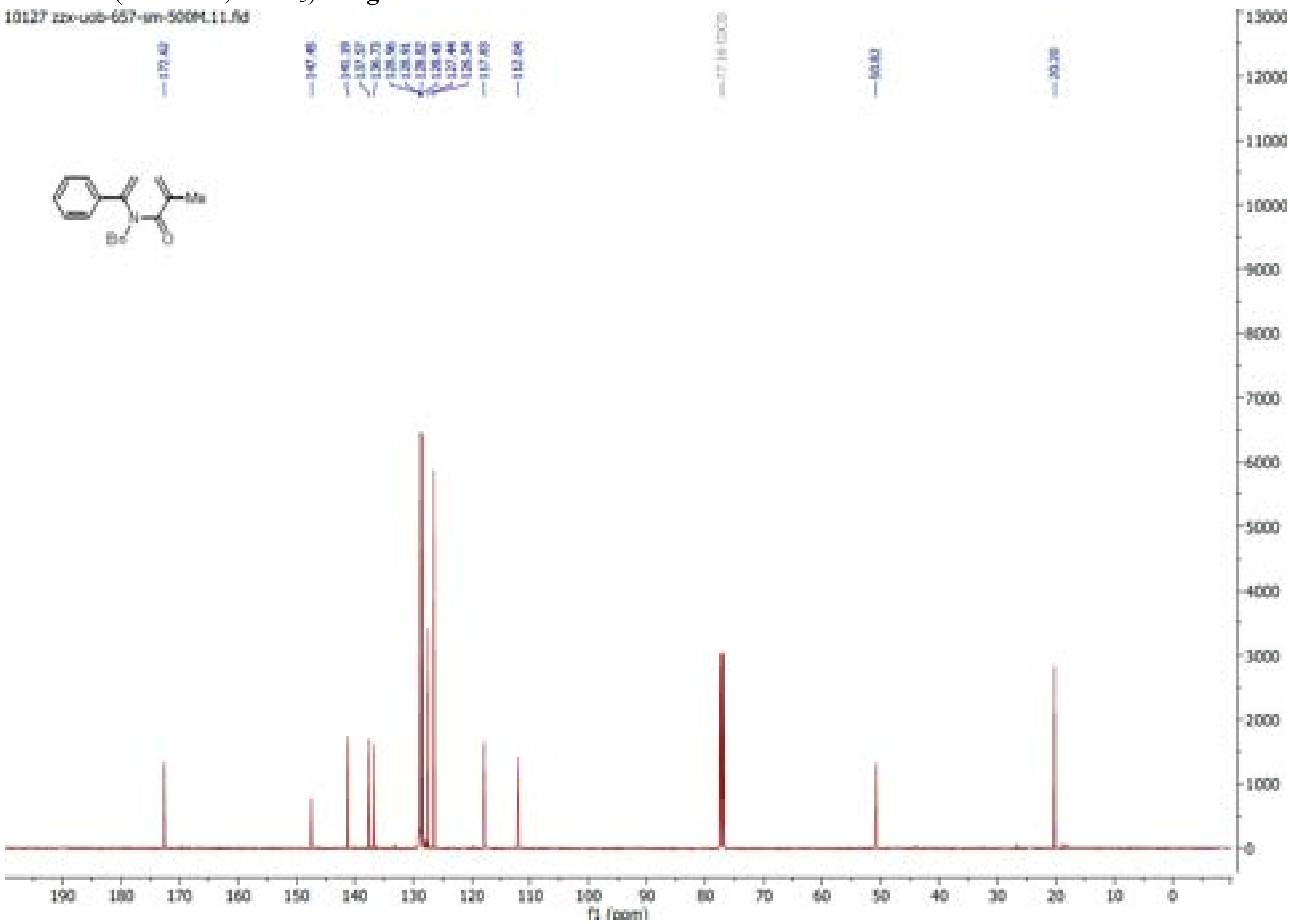

^{1}H NMR (500 MHz, $CDCl_3$) of **1h**

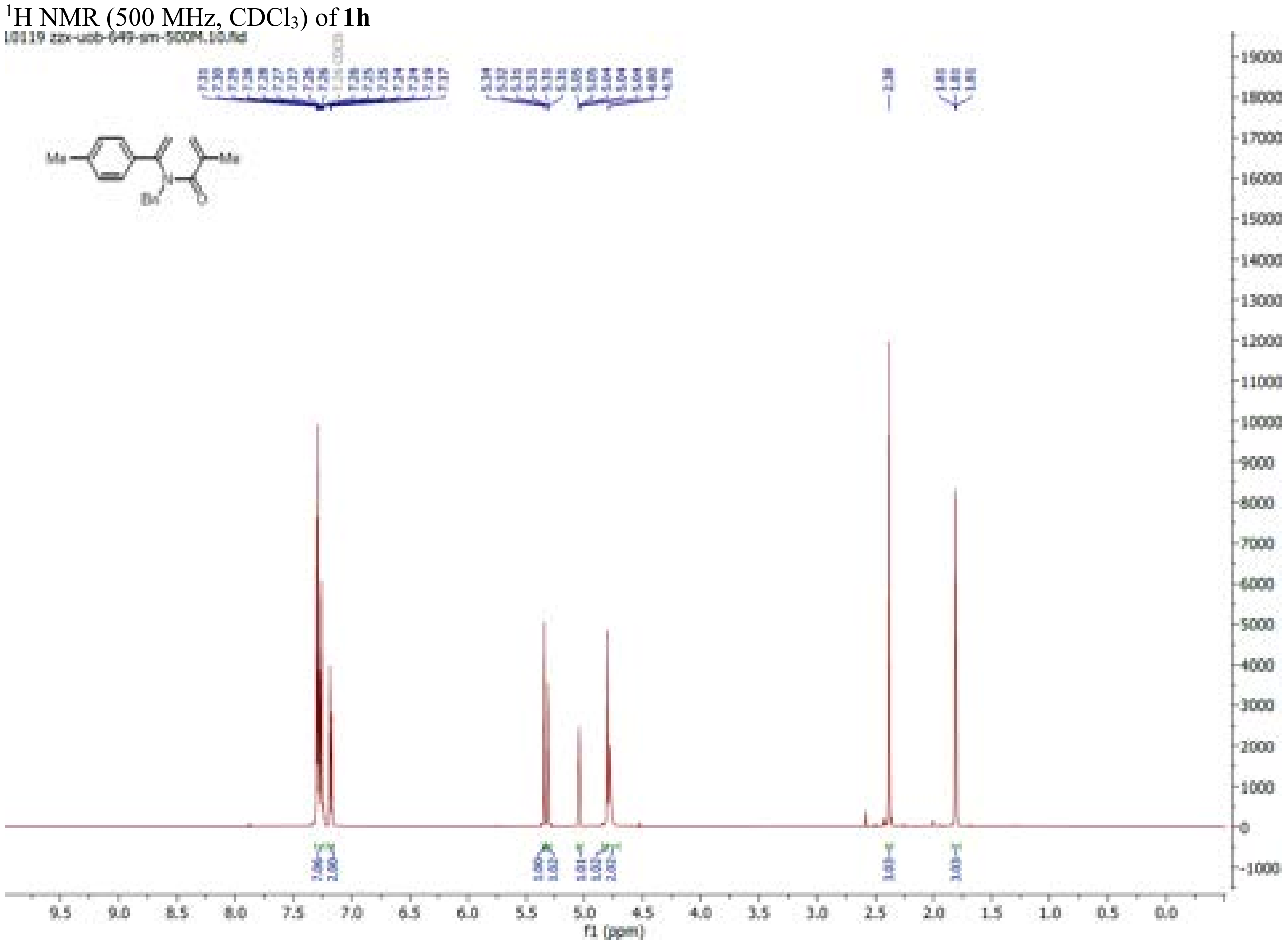

^{13}C NMR (126 MHz, $CDCl_3$) of **1h**

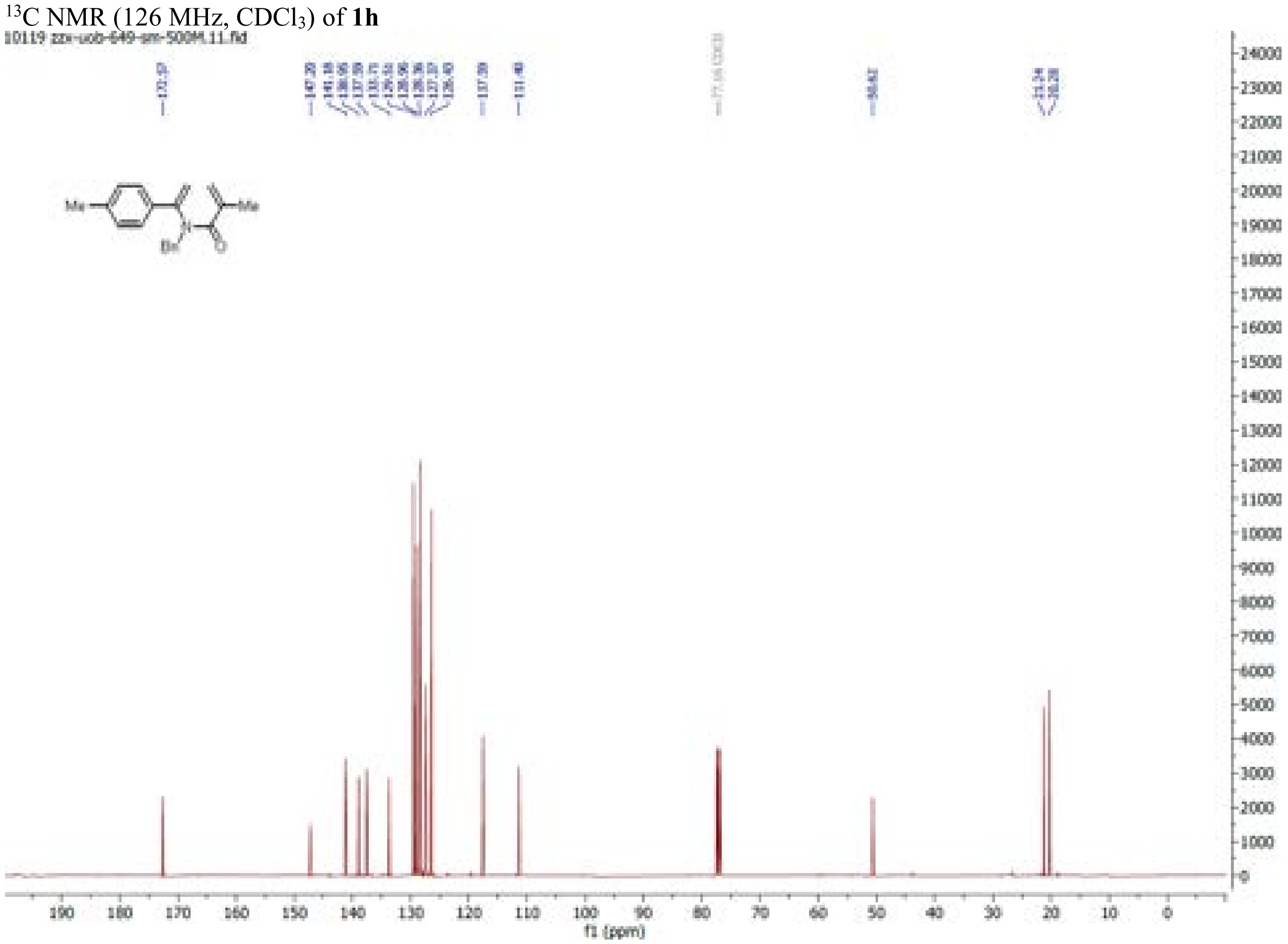

^1H NMR (500 MHz, $CDCl_3$) of **1i**

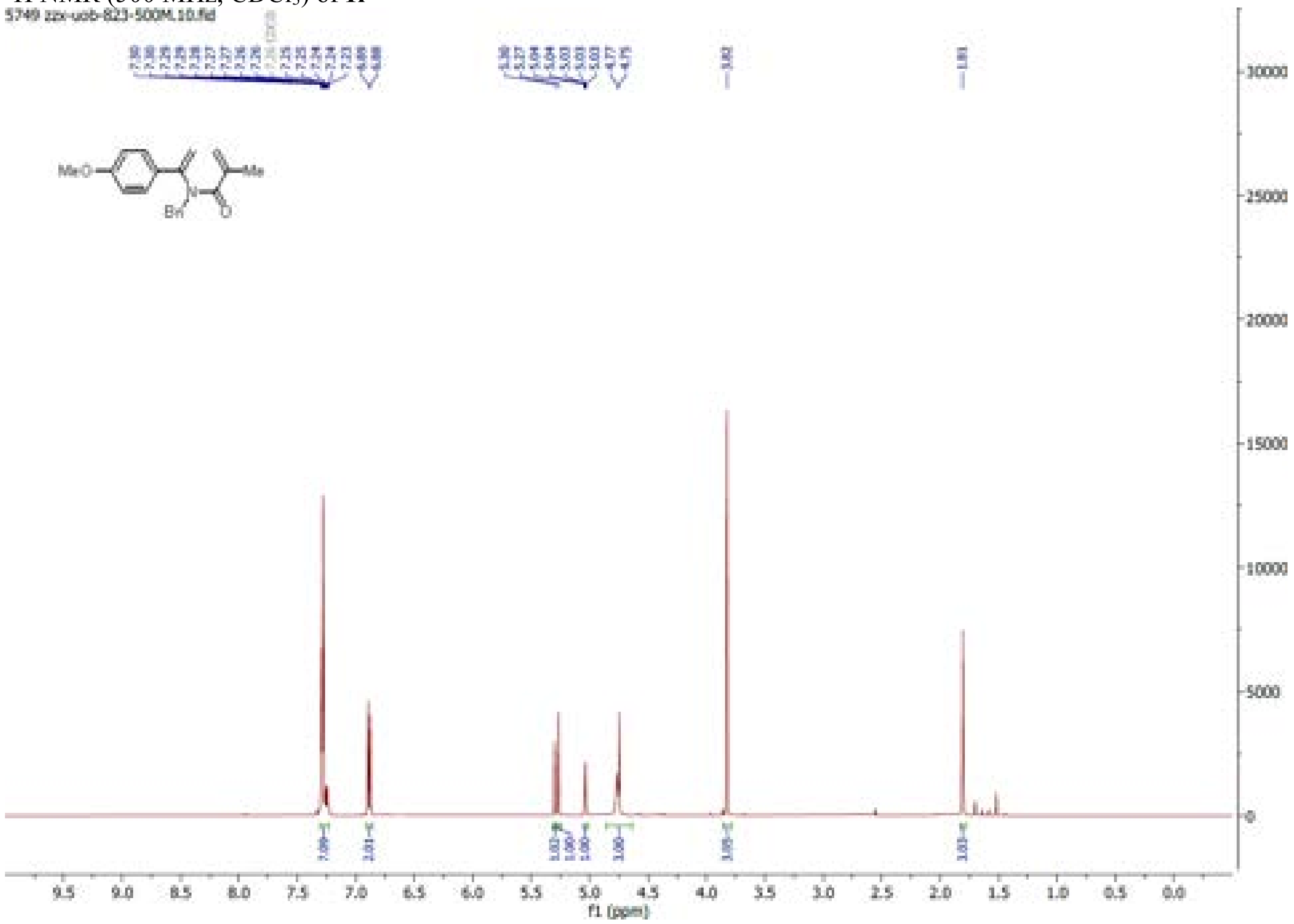

^{13}C NMR (126 MHz, $CDCl_3$) of **1i**

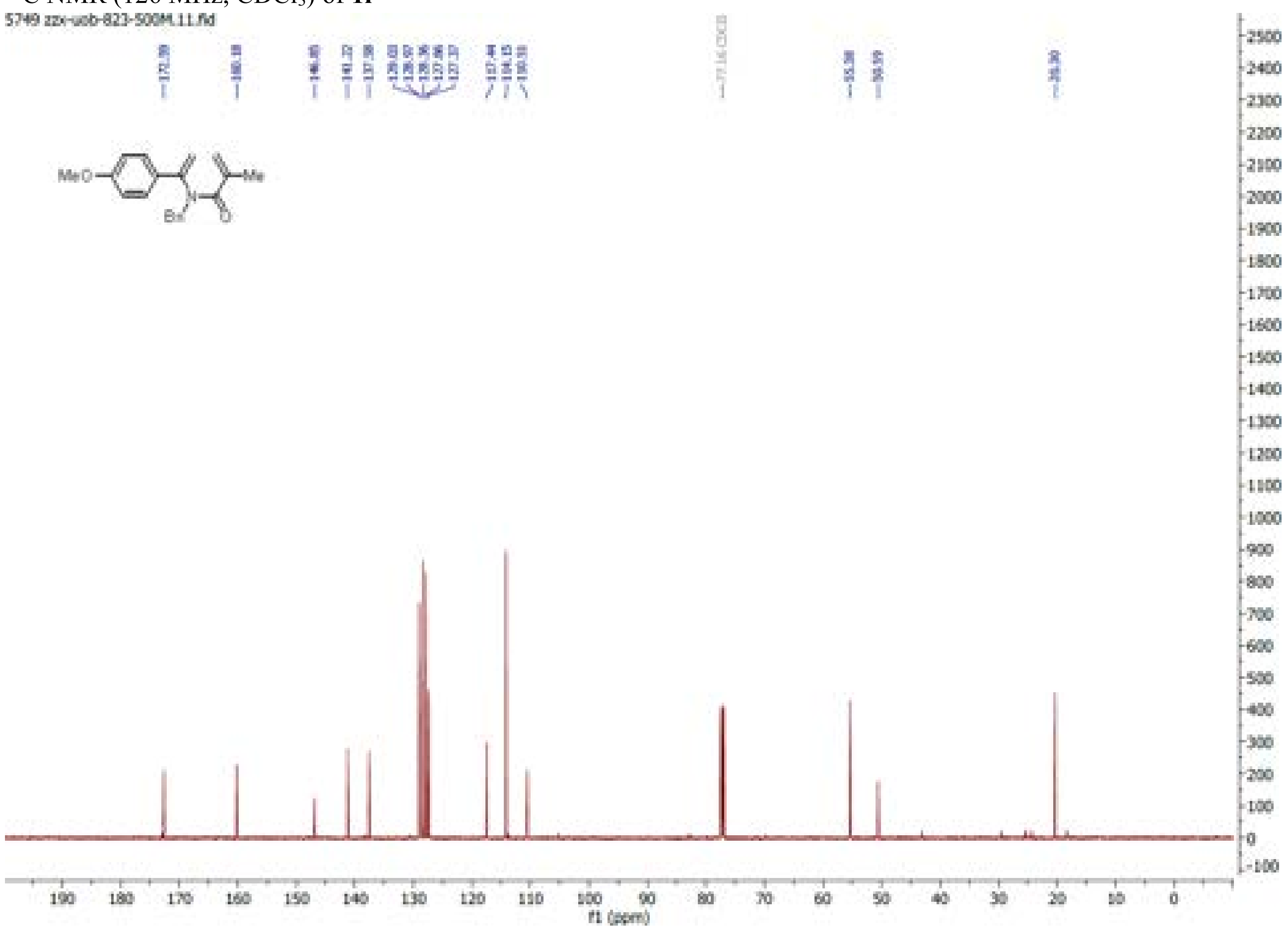

^{1}H NMR (500 MHz, $CDCl_3$) of **1j**

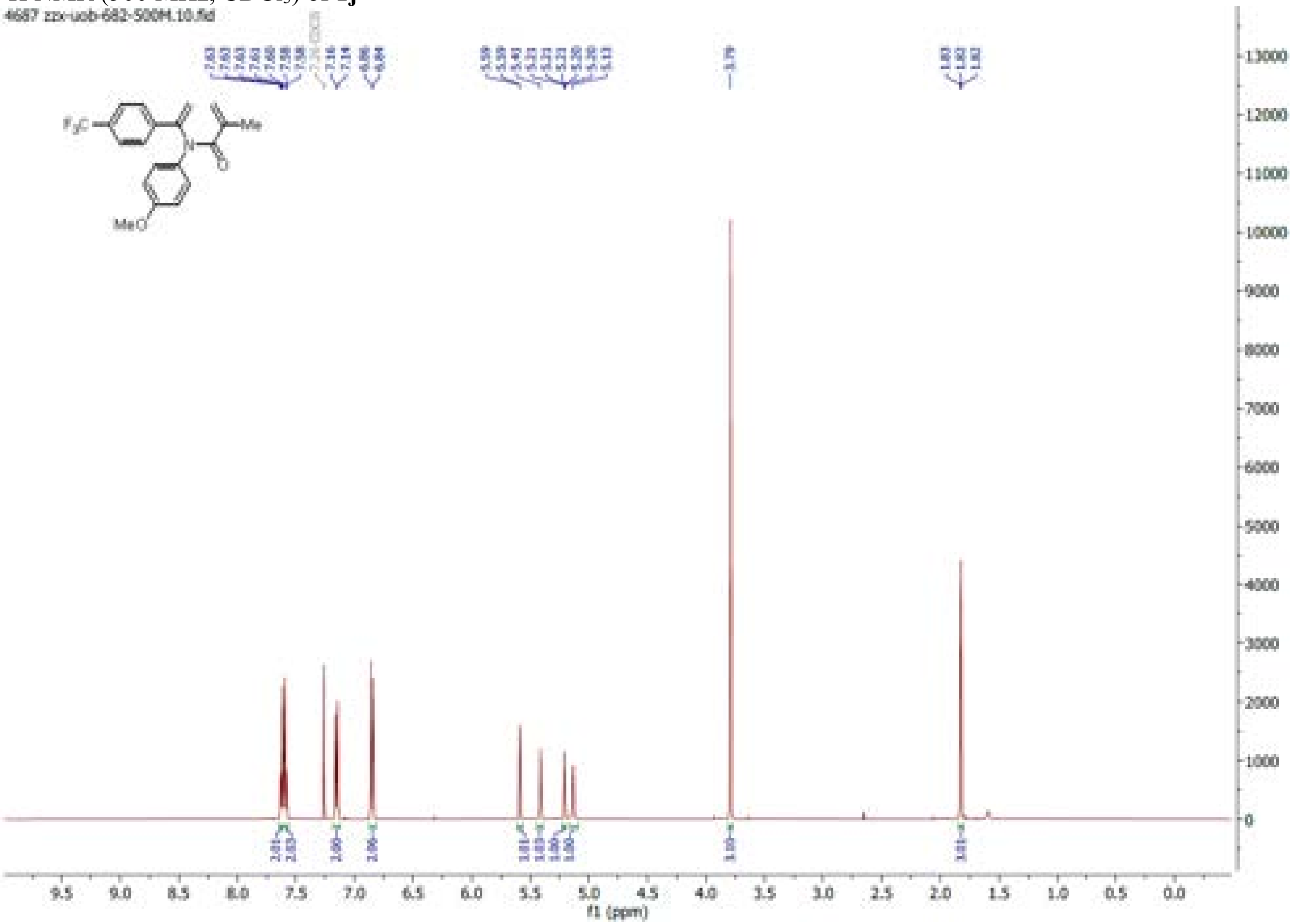

^{13}C NMR (126 MHz, $CDCl_3$) of **1j**

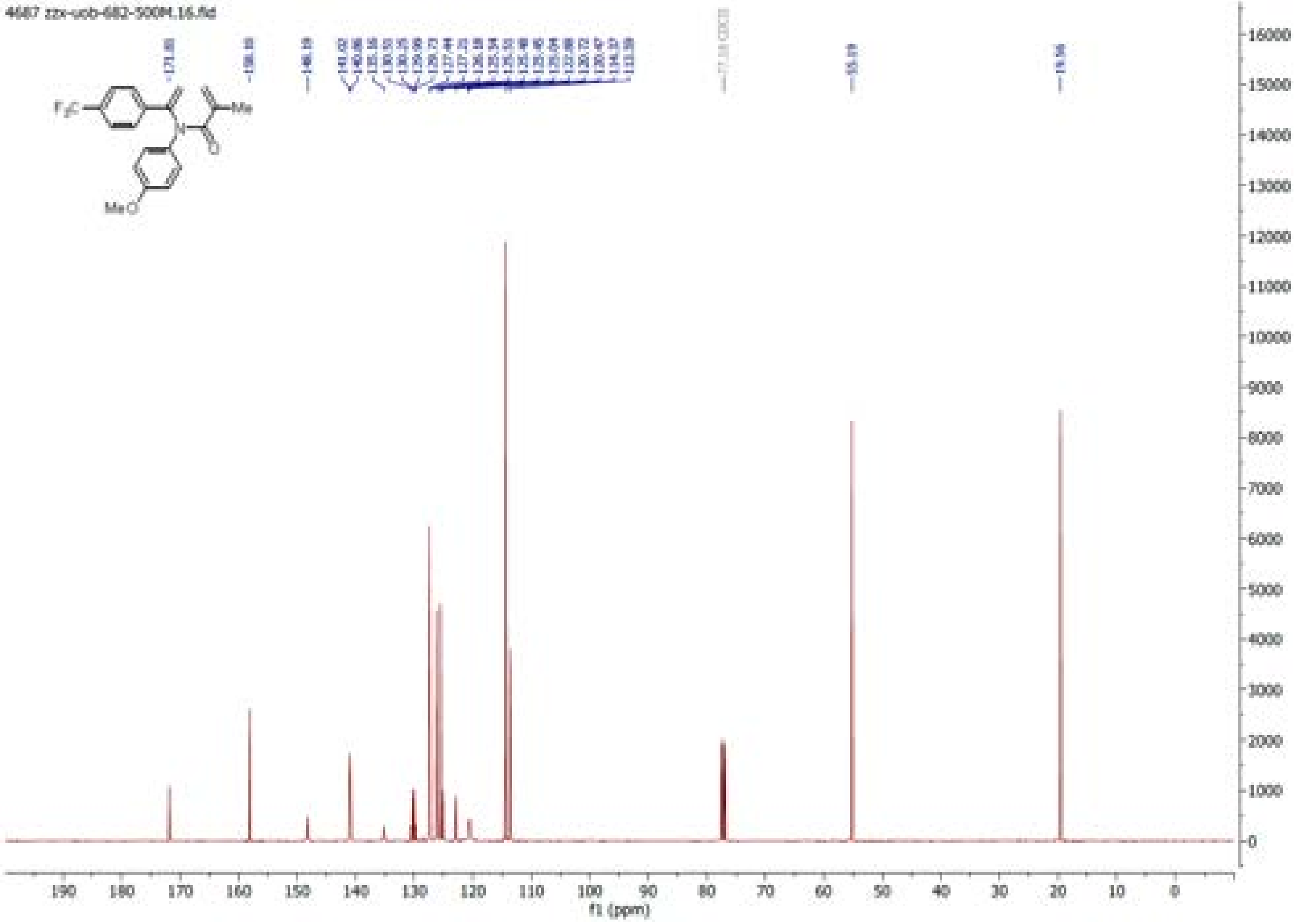

^{19}F NMR (471 MHz, $CDCl_3$) of **1j**

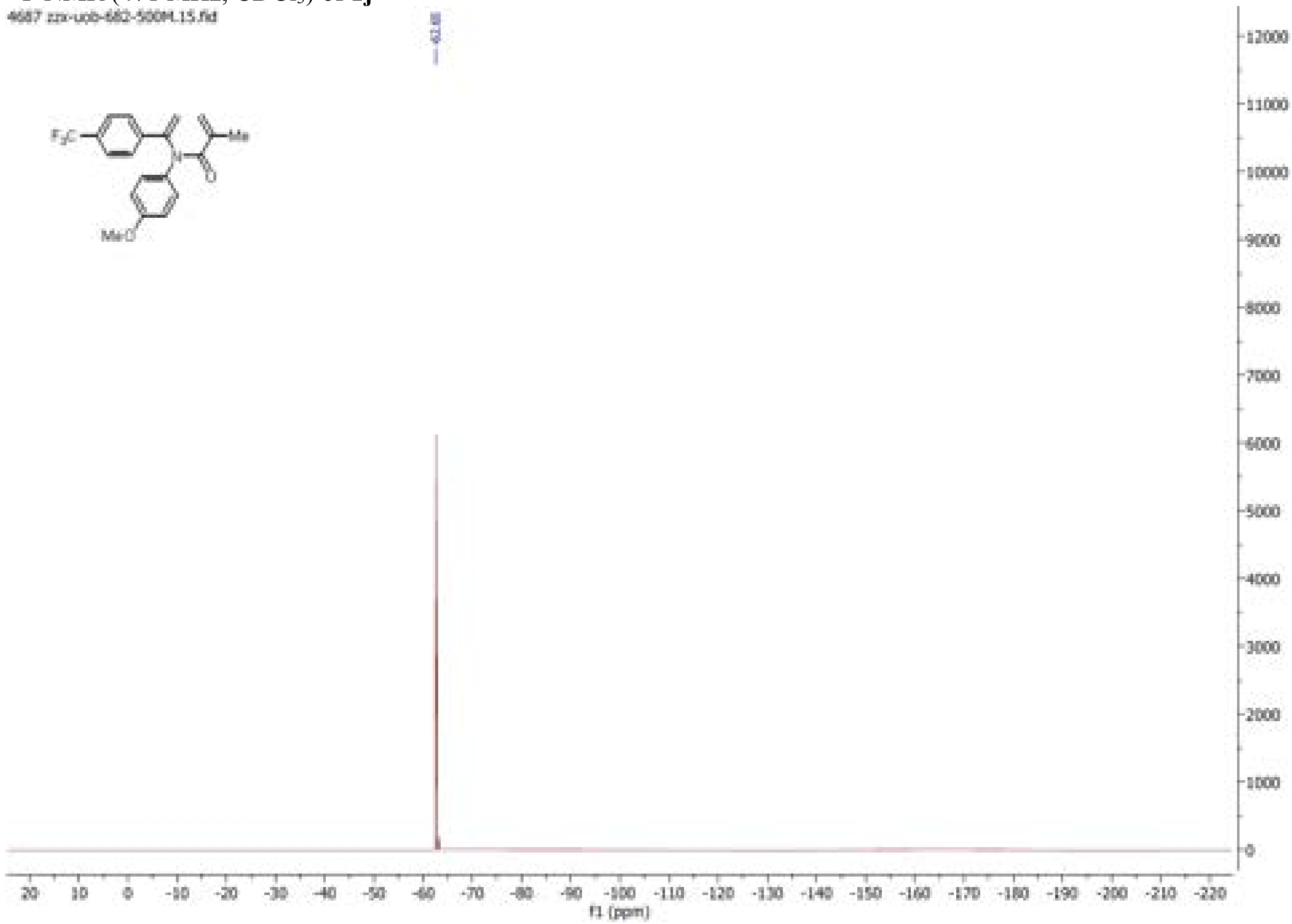

^{1}H NMR (500 MHz, $CDCl_3$) of **1k**

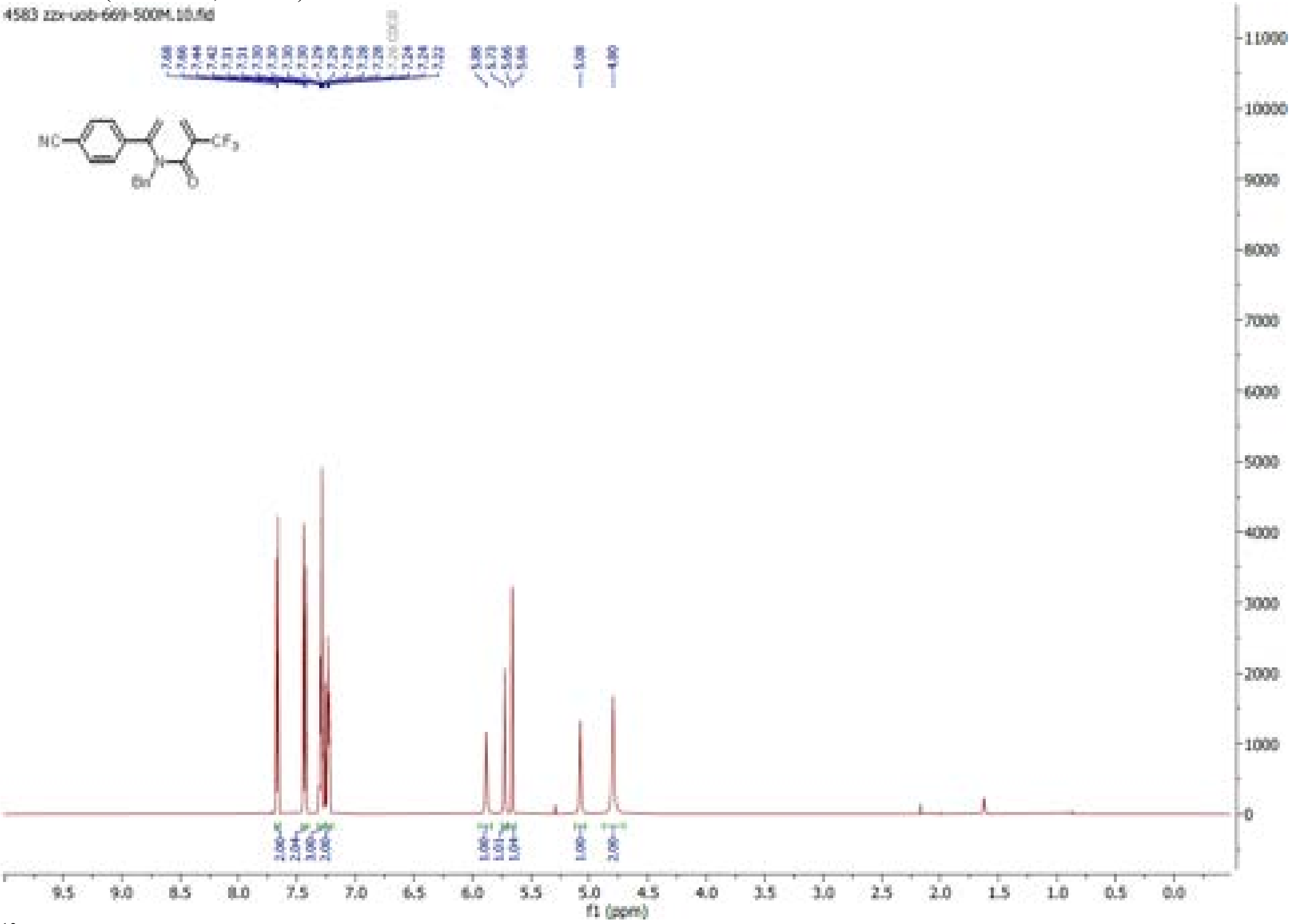

^{13}C NMR (126 MHz, $CDCl_3$) of **1k**

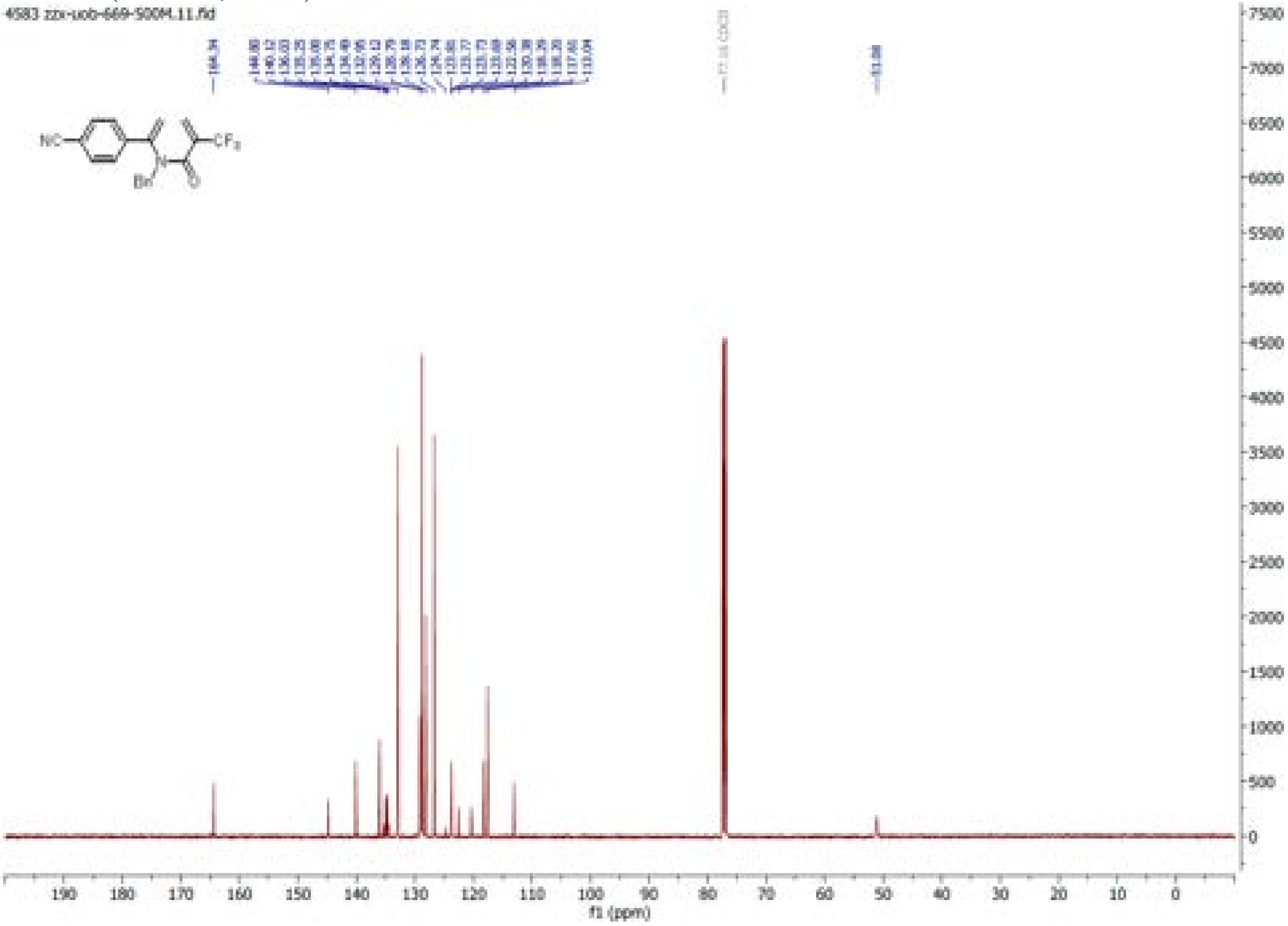

^{19}F NMR (471 MHz, $CDCl_3$) of **1k**

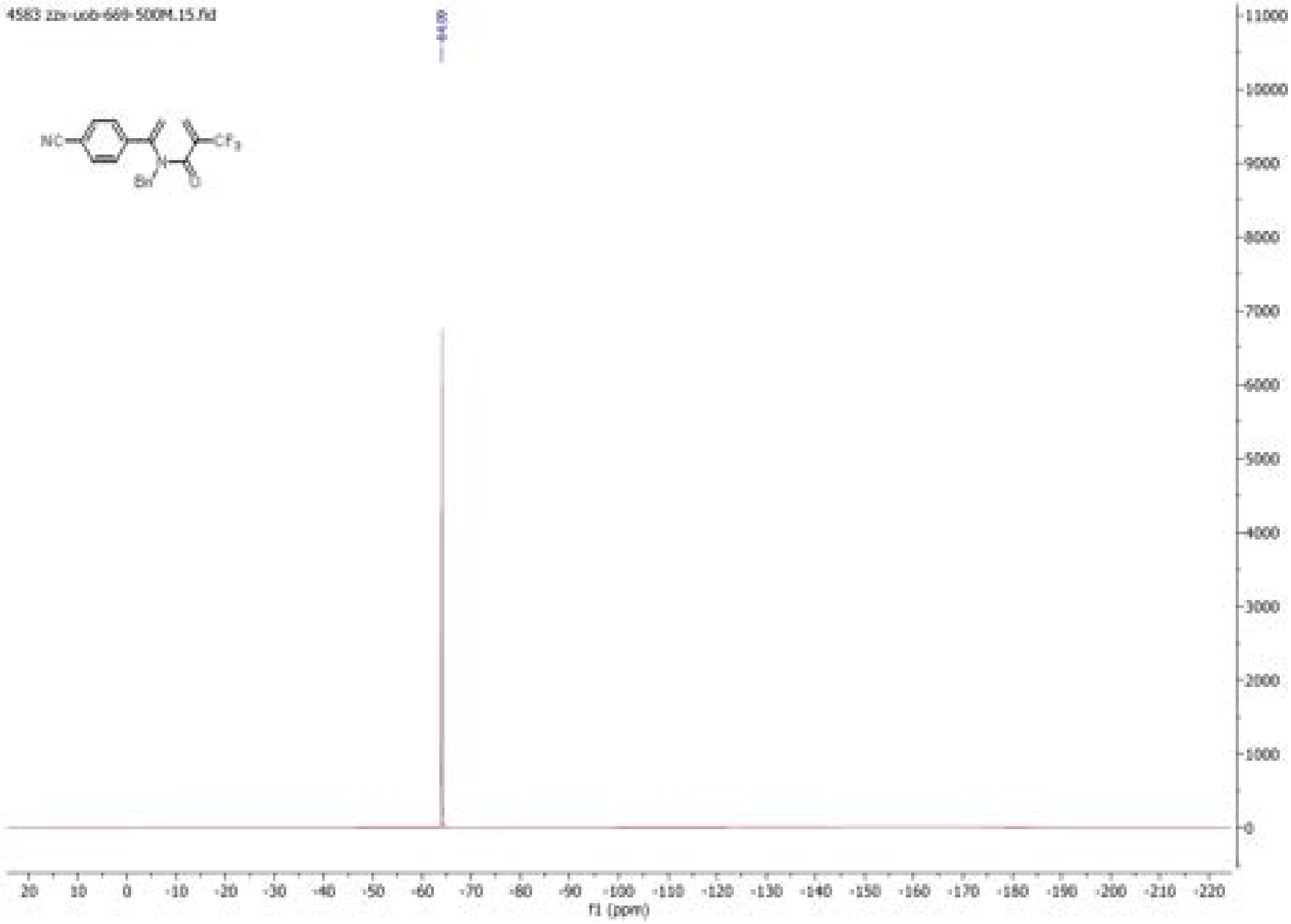

^{1}H NMR (500 MHz, $CDCl_3$) of **1l**

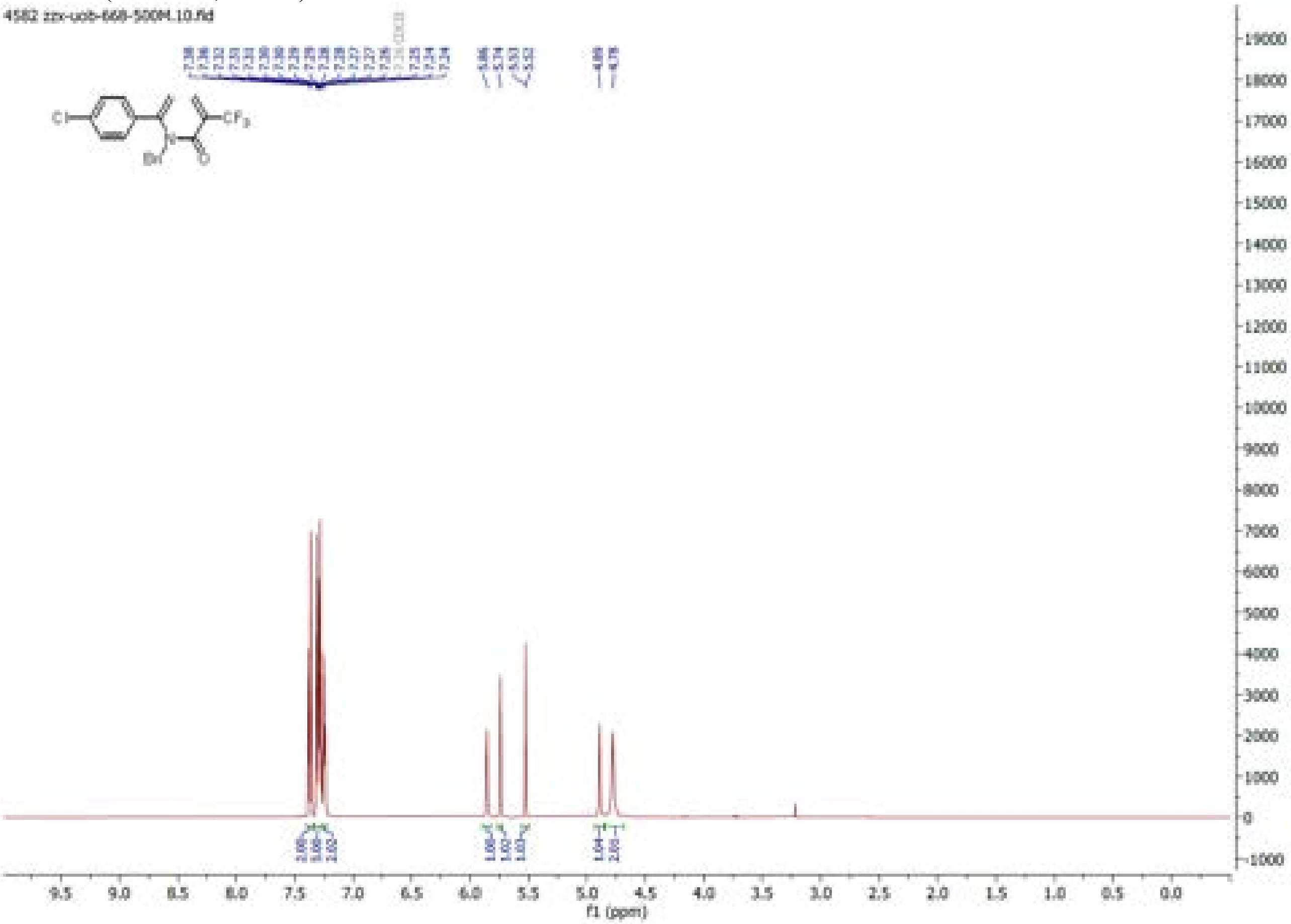

^{13}C NMR (126 MHz, $CDCl_3$) of **1l**

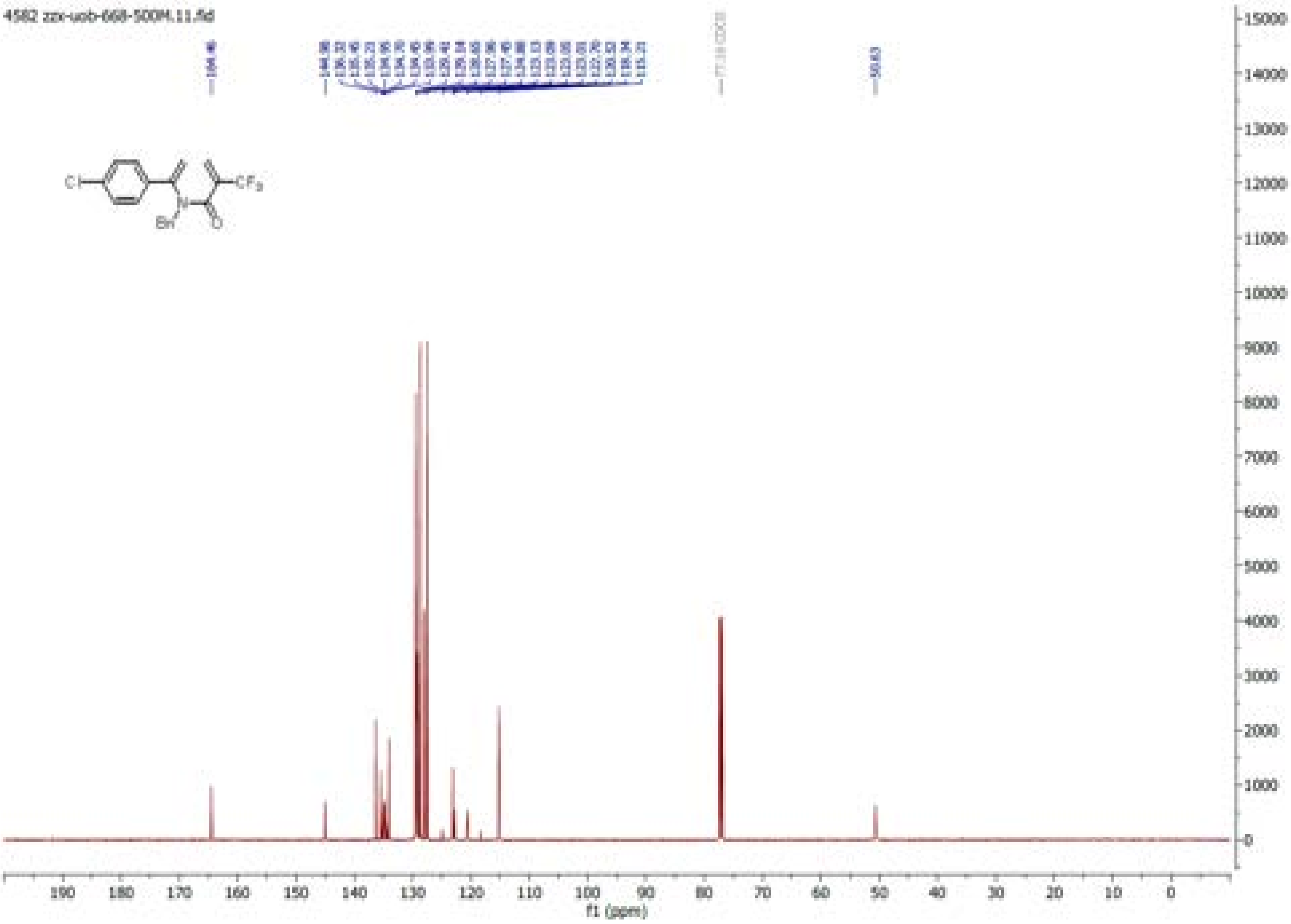

^{19}F NMR (471 MHz, $CDCl_3$) of **1l**

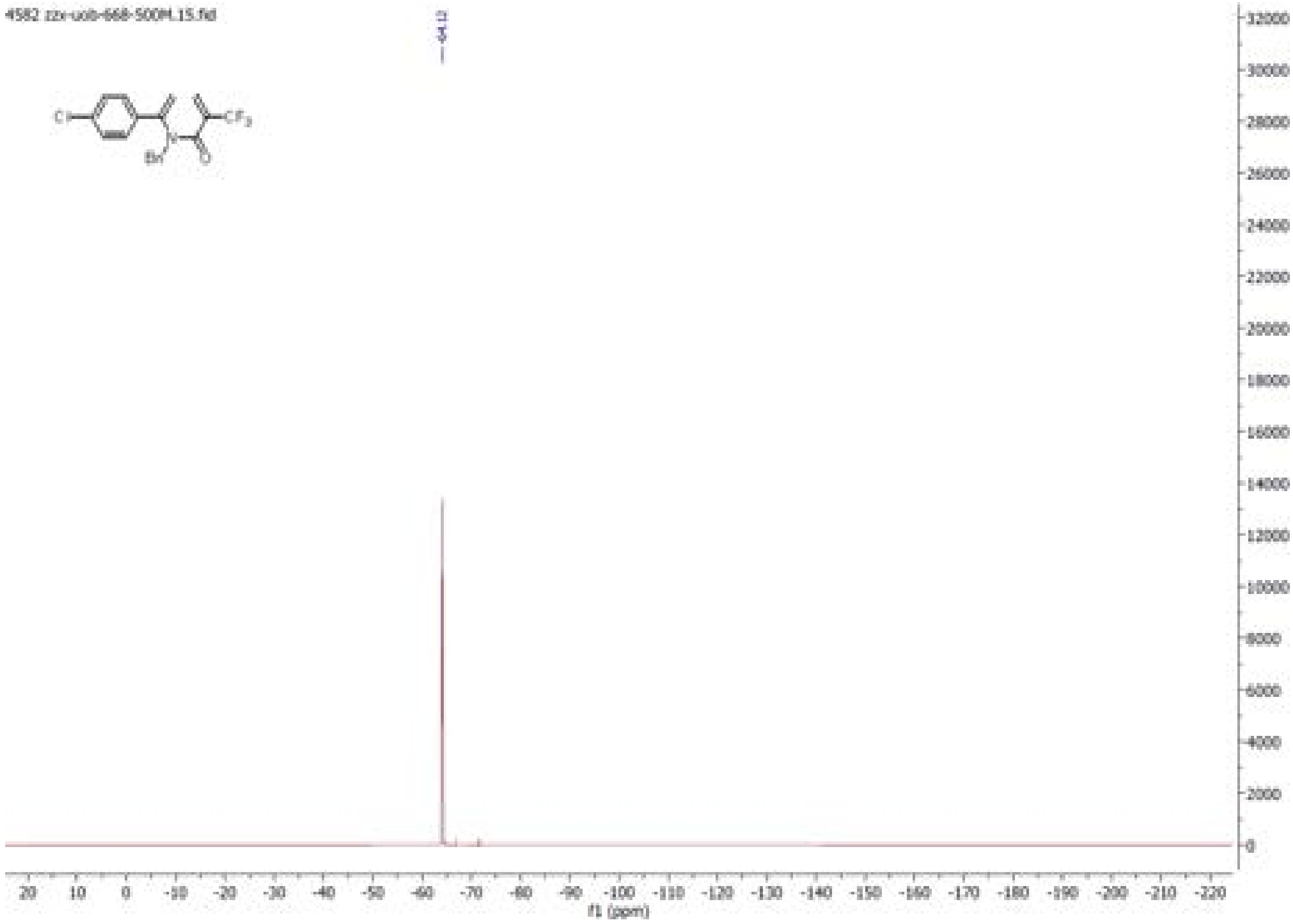

^{1}H NMR (500 MHz, $CDCl_3$) of **1m**

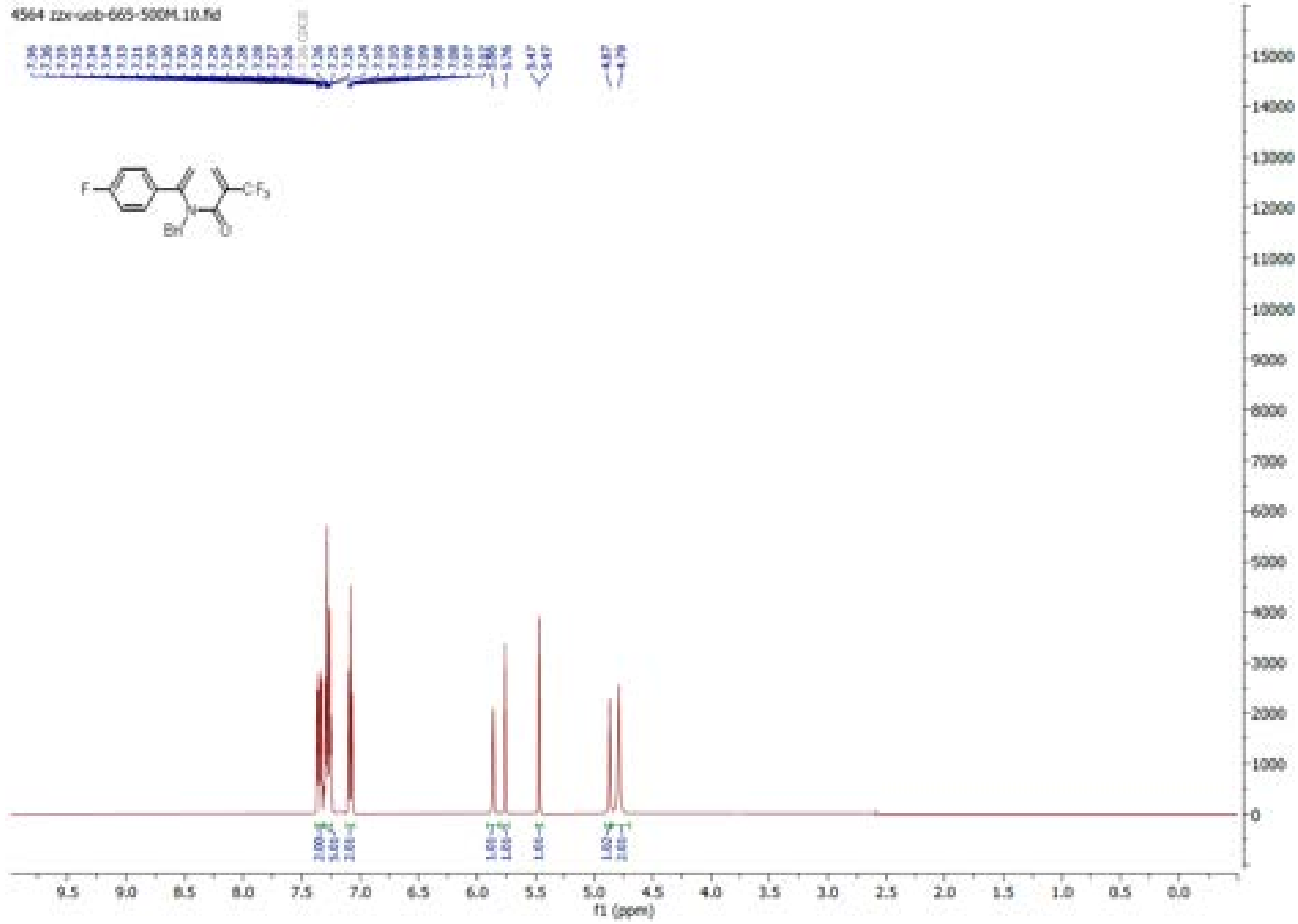

^{13}C NMR (126 MHz, $CDCl_3$) of **1m**

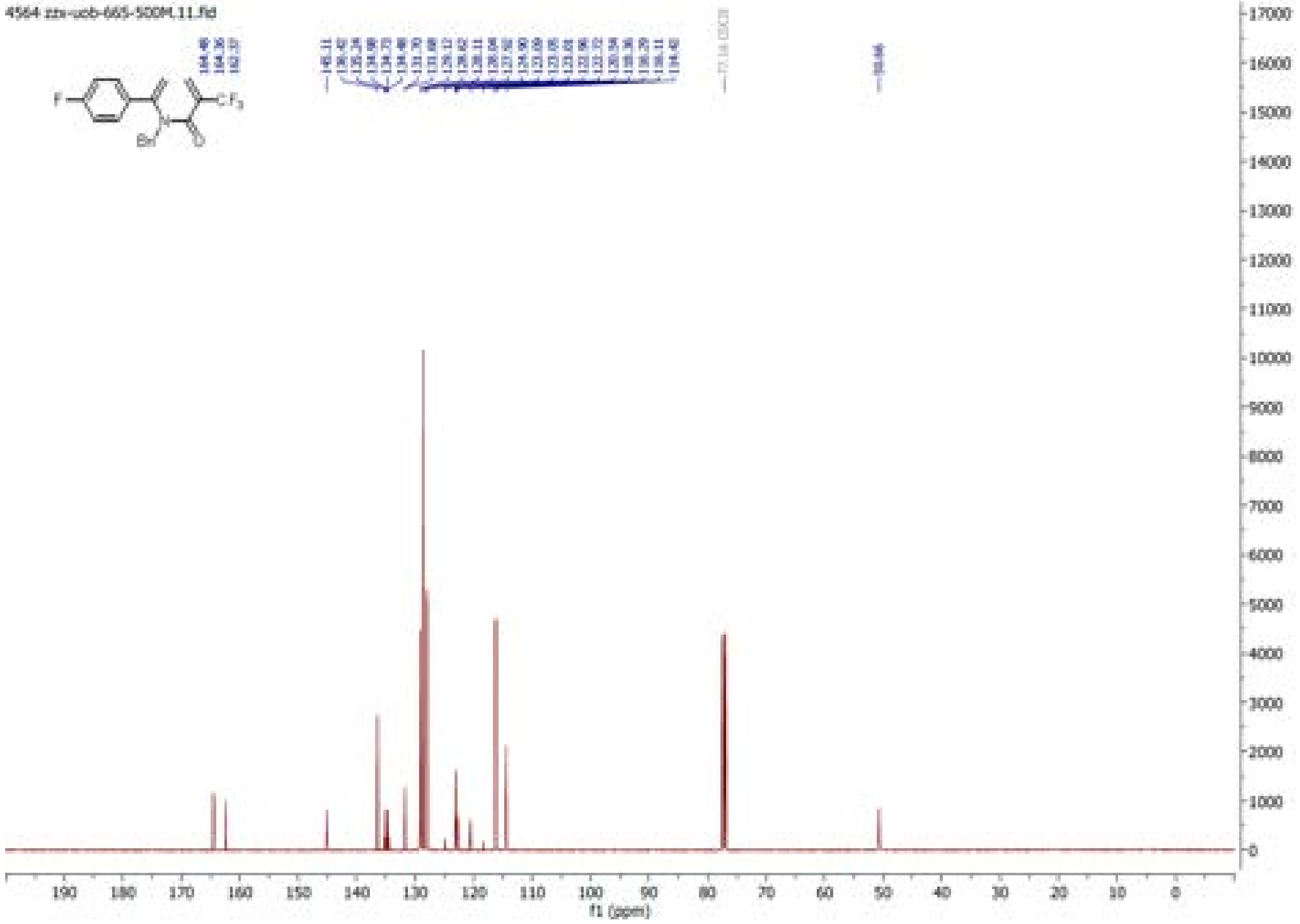

^{19}F NMR (471 MHz, $CDCl_3$) of **1m**

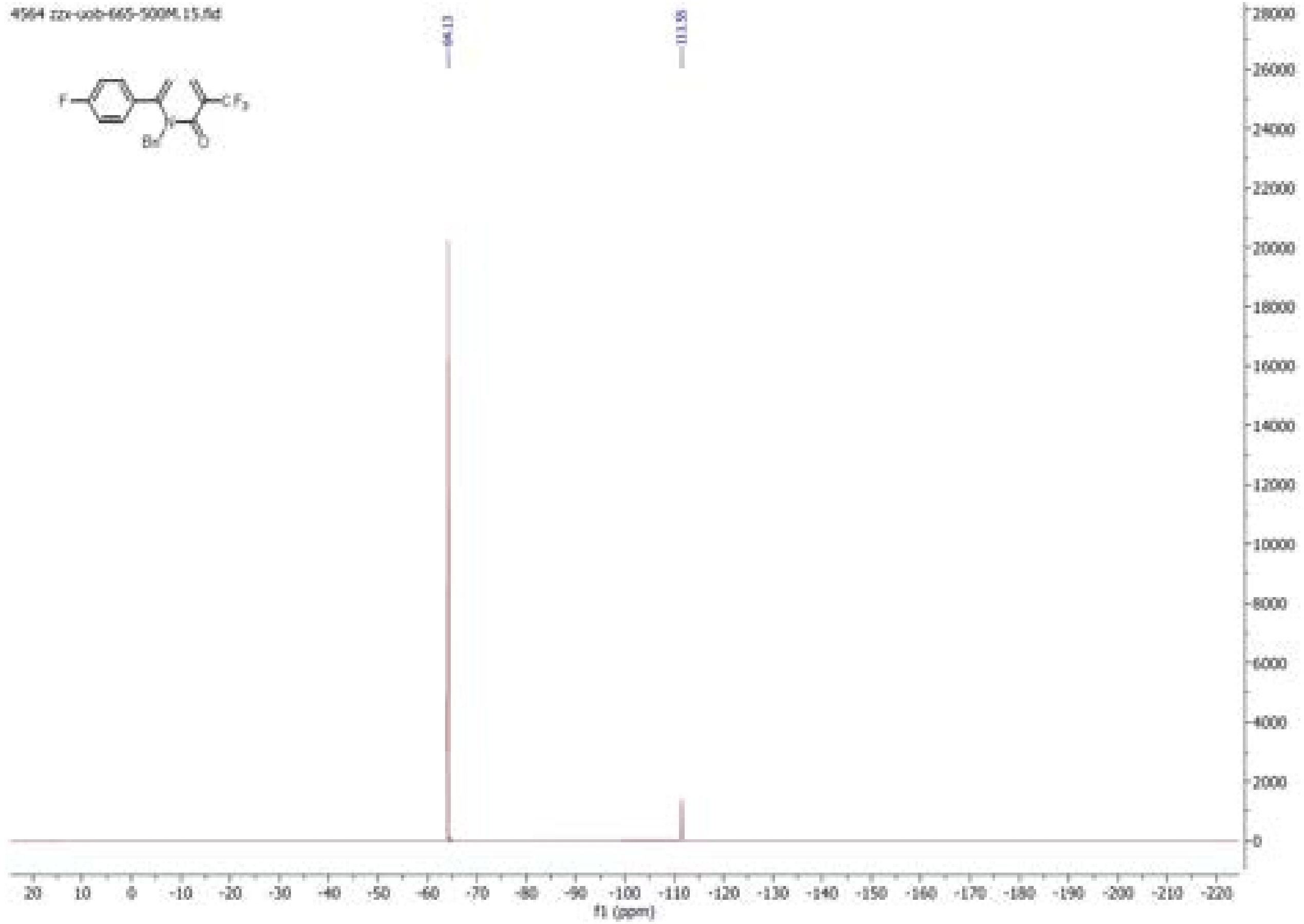

^{1}H NMR (500 MHz, $CDCl_3$) of **1n**

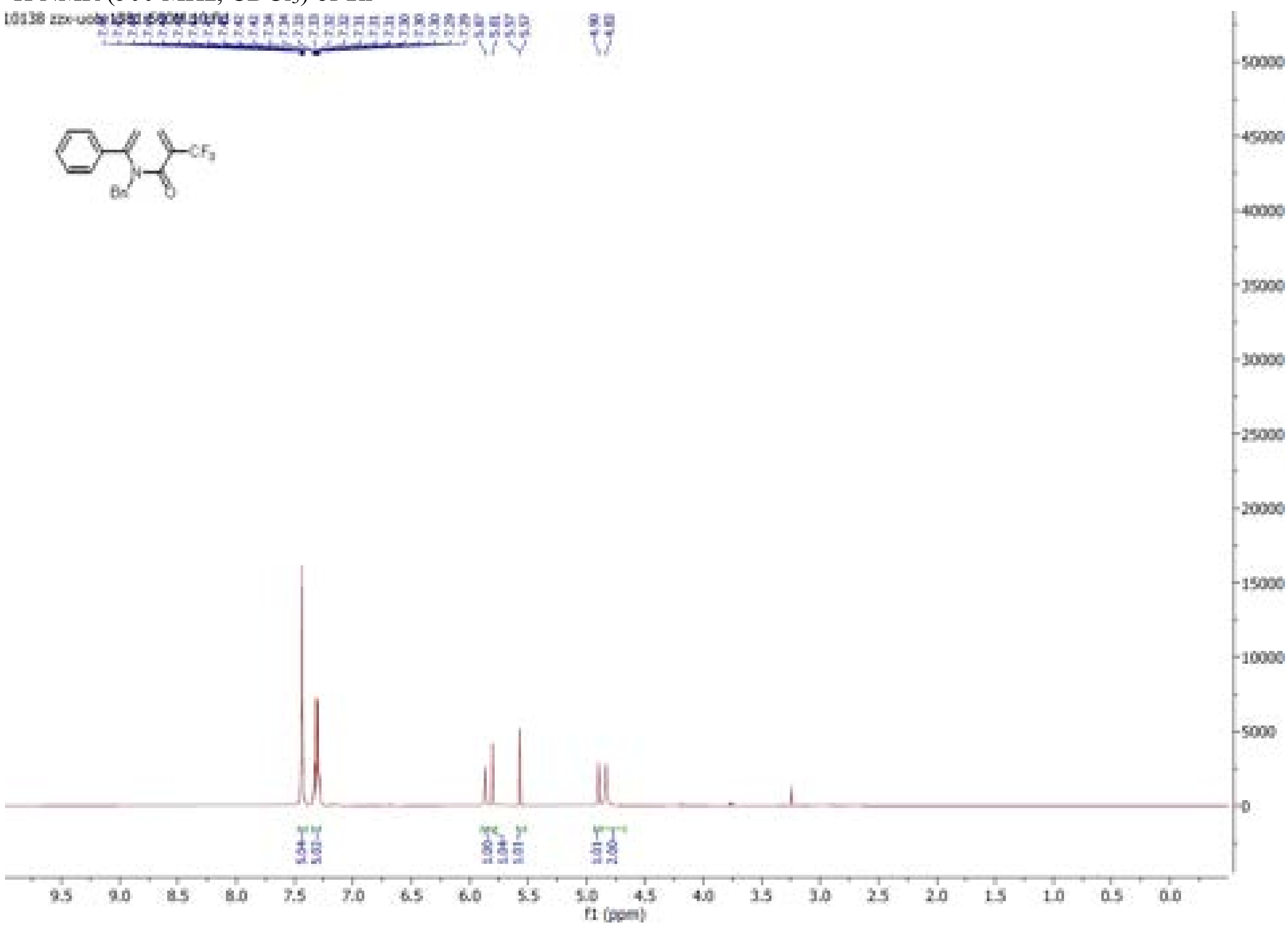

^{13}C NMR (126 MHz, $CDCl_3$) of **1n**

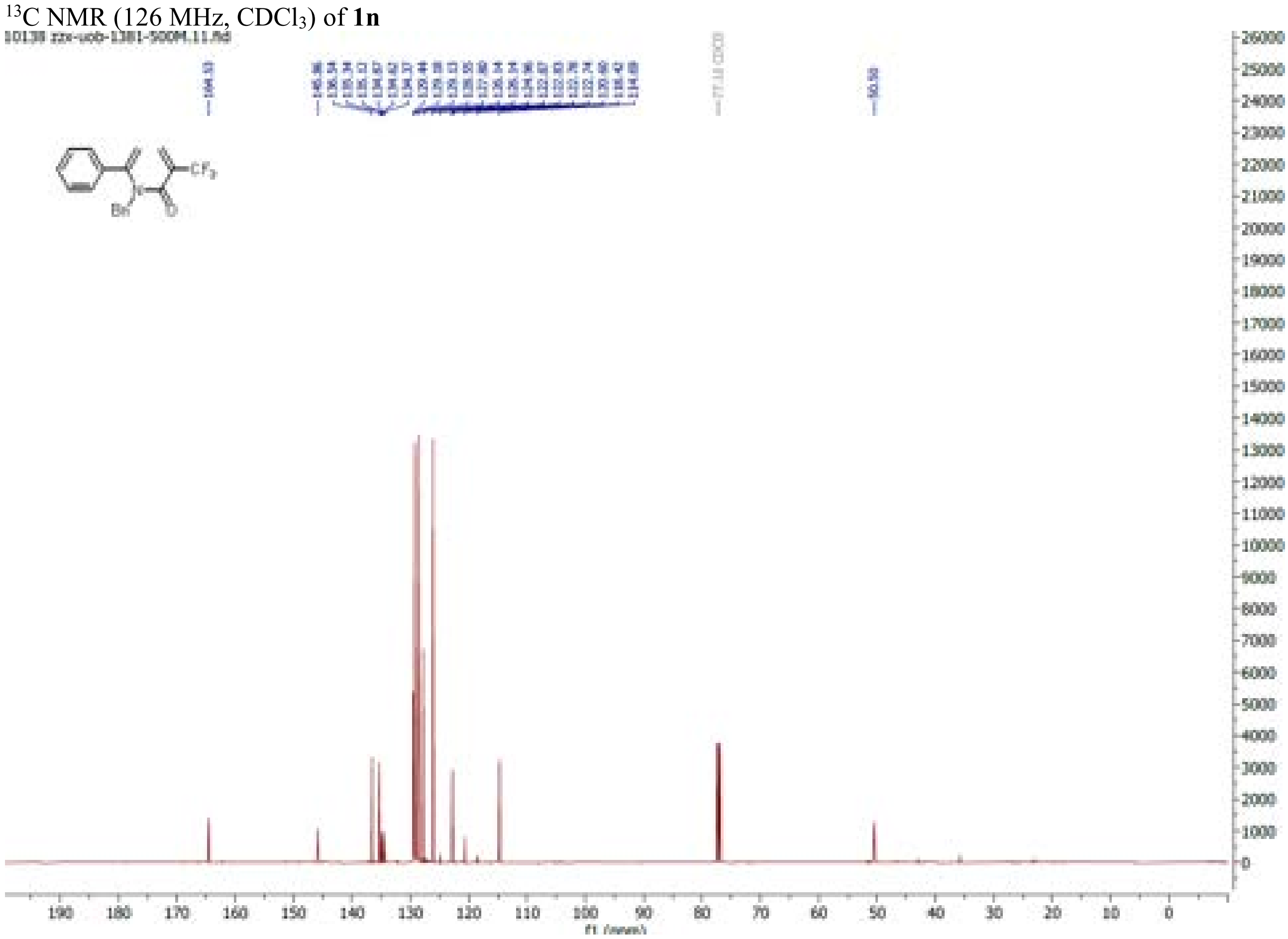

^{19}F NMR (471 MHz, $CDCl_3$) of **1n**

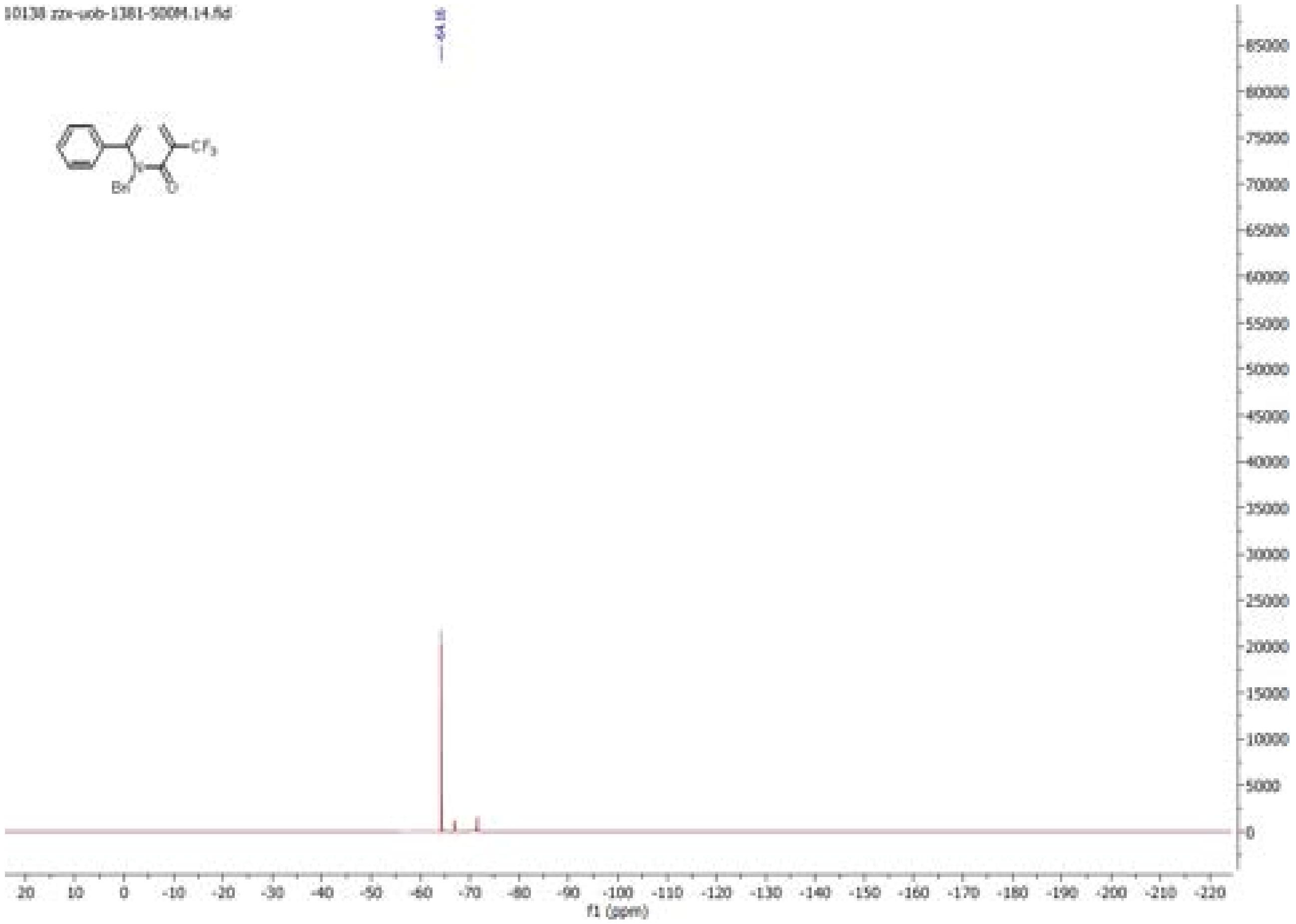

^{1}H NMR (500 MHz, $CDCl_3$) of **1o**

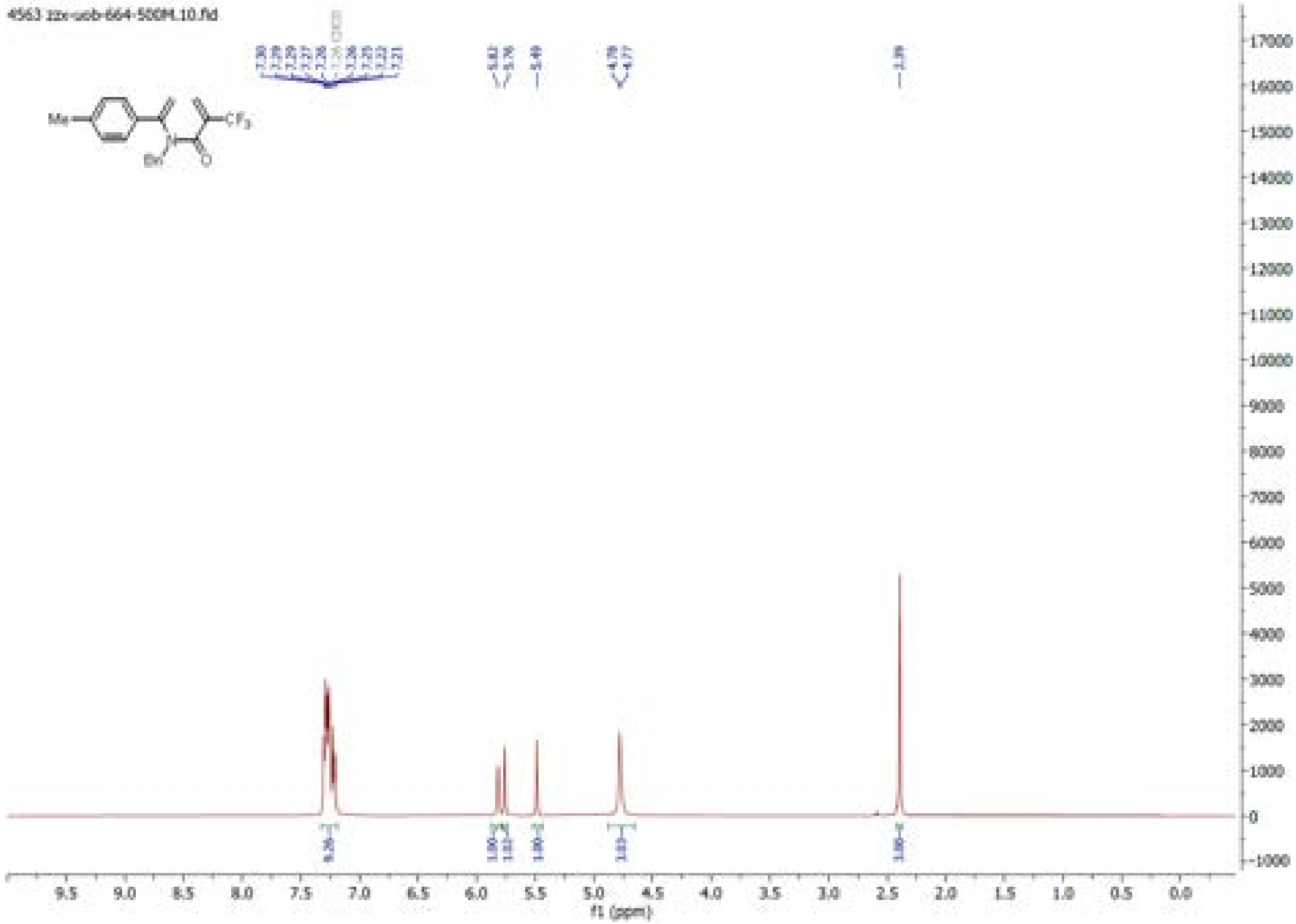

^{13}C NMR (126 MHz, $CDCl_3$) of **1o**

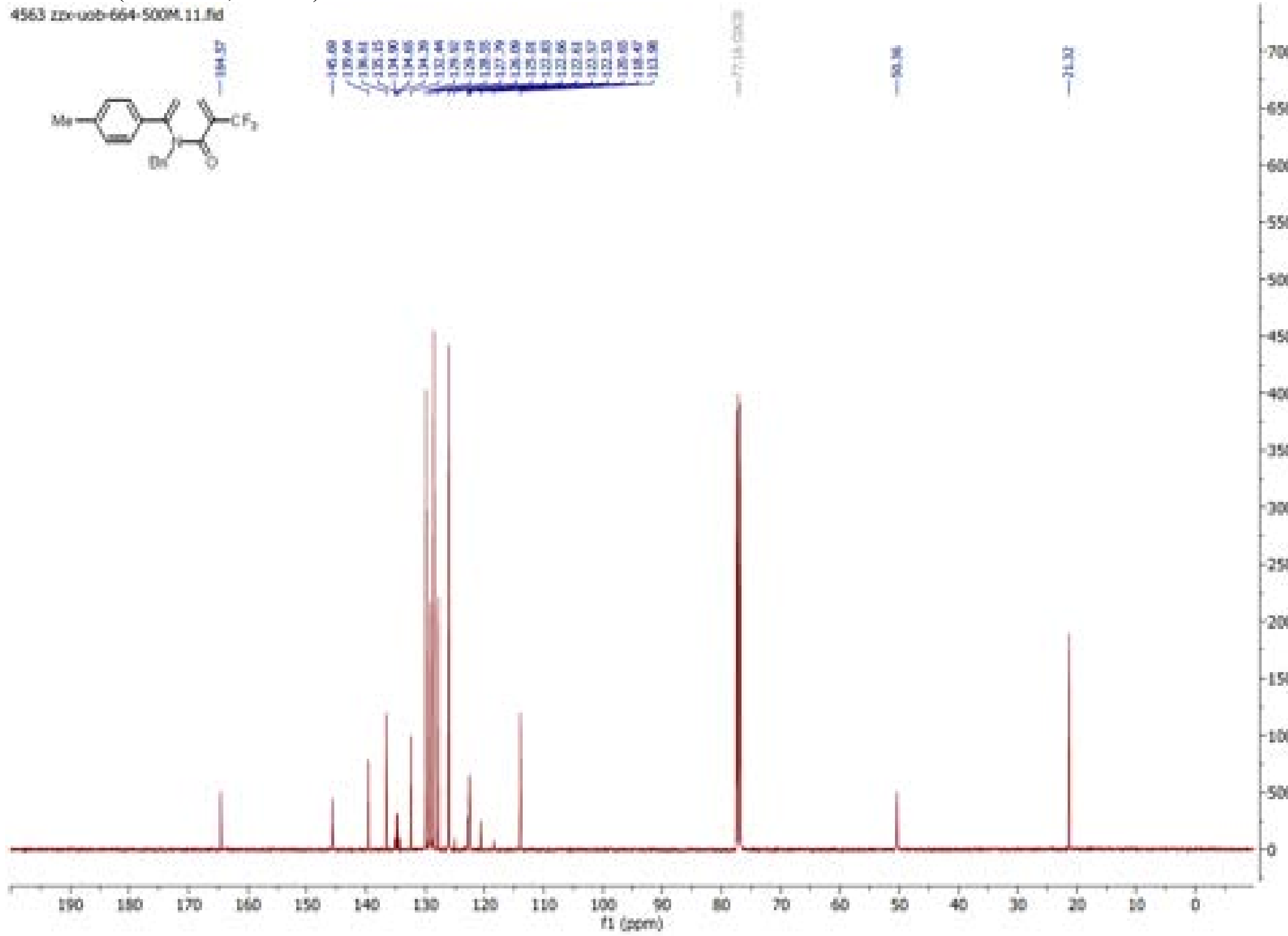

^{19}F NMR (471 MHz, $CDCl_3$) of **1o**

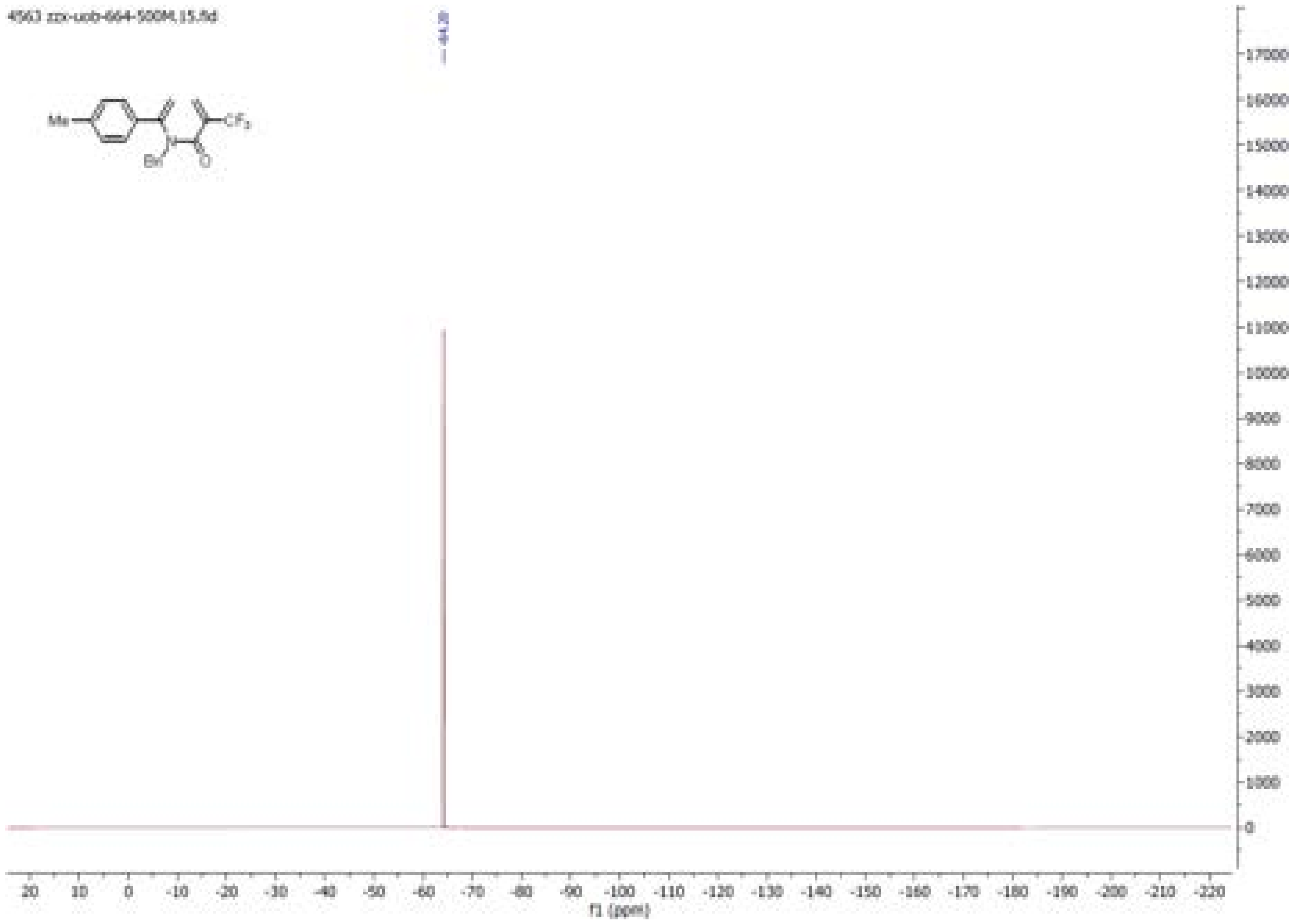

^{1}H NMR (500 MHz, $CDCl_3$) of **1p**

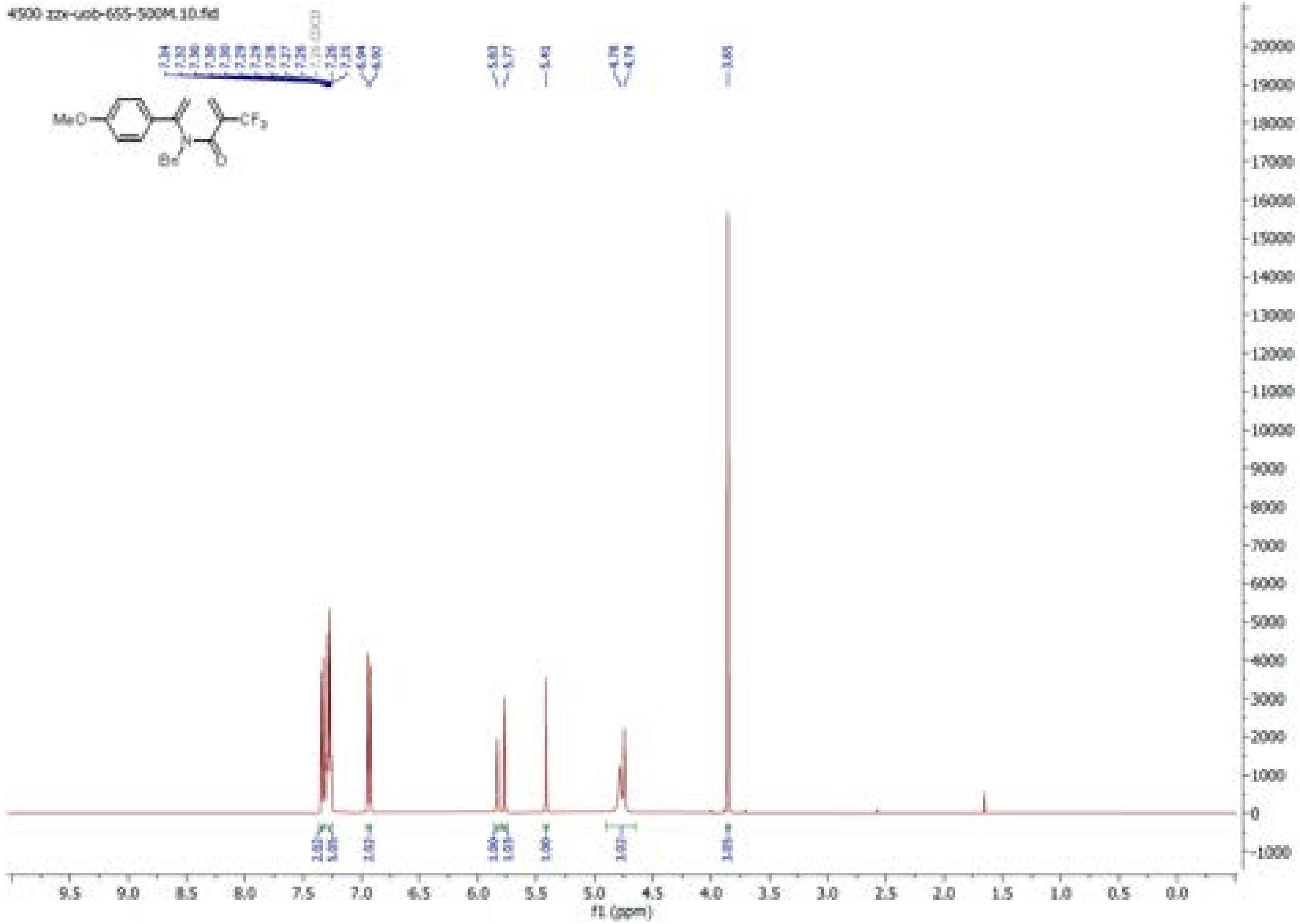

^{13}C NMR (126 MHz, $CDCl_3$) of **1p**

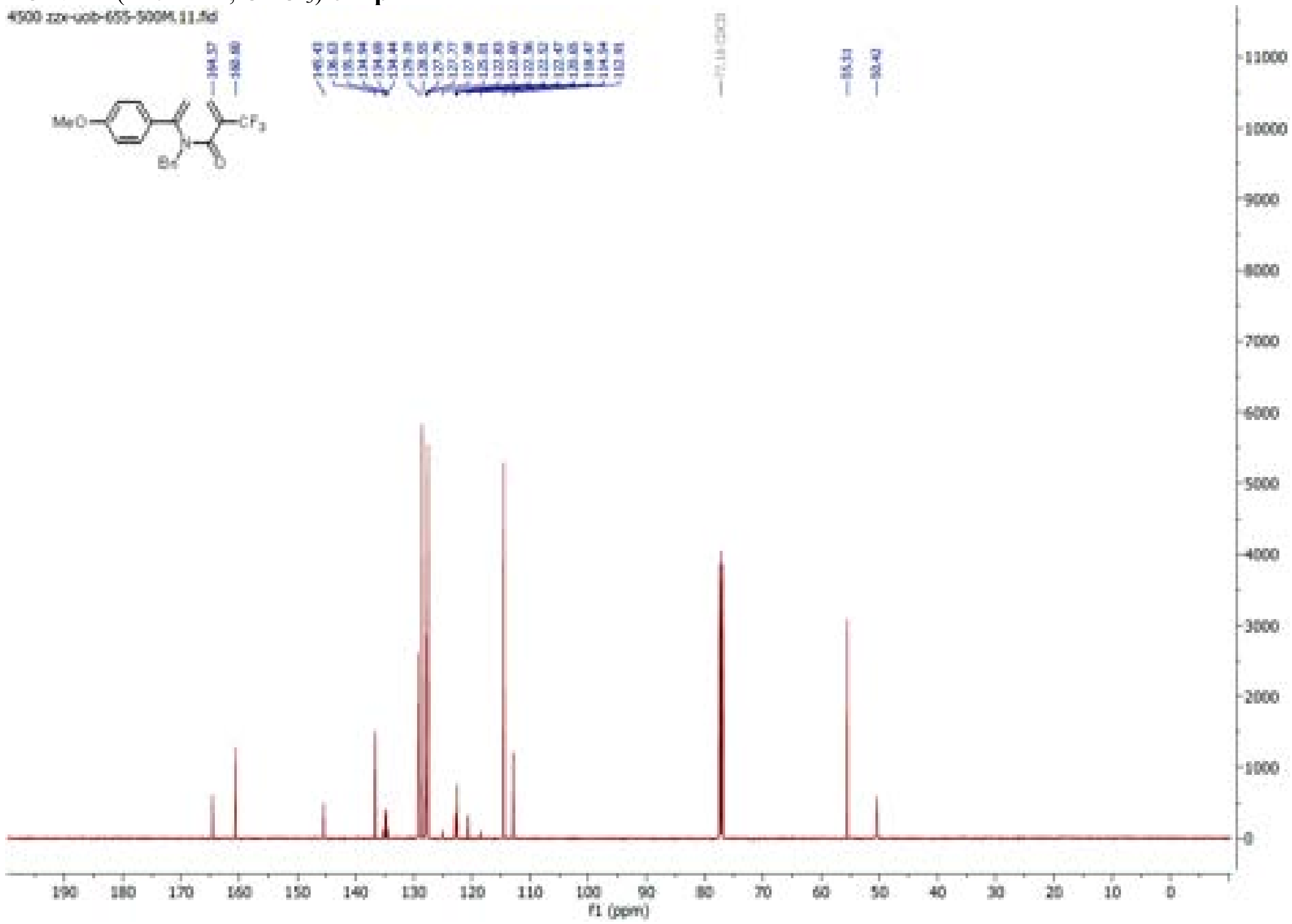

^{19}F NMR (471 MHz, $CDCl_3$) of **1p**

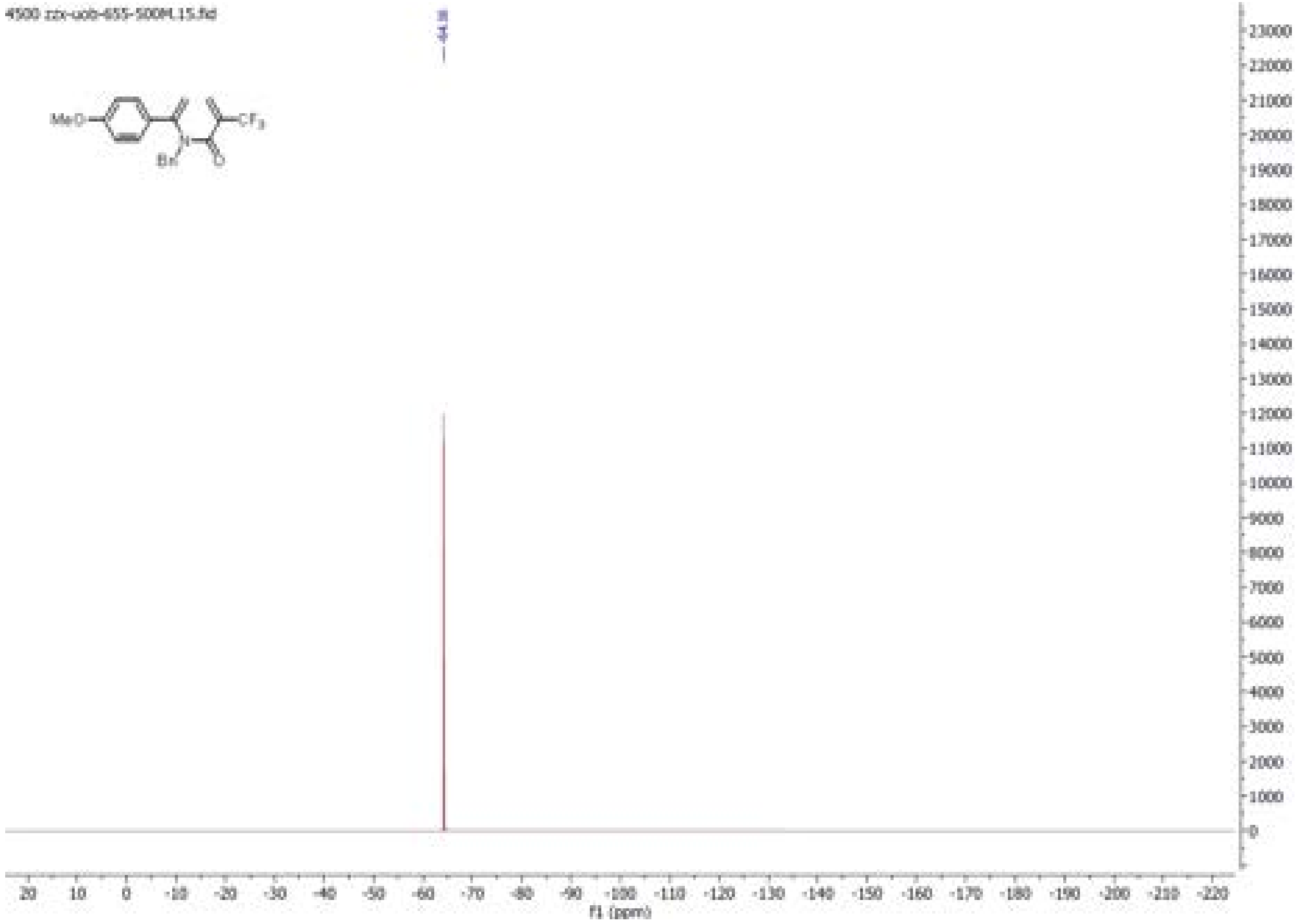

^{1}H NMR (500 MHz, $CDCl_3$) of **1q**

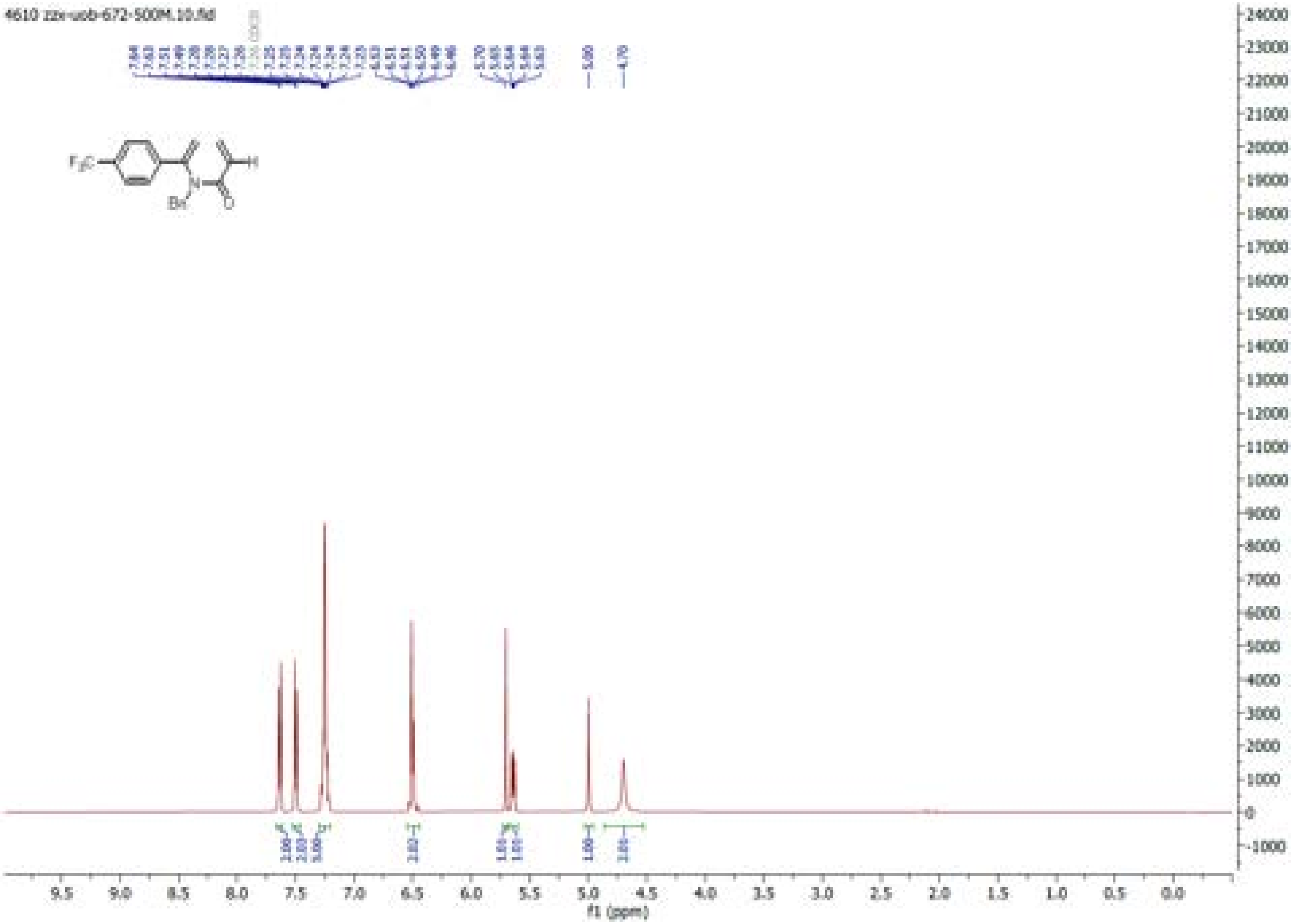

^{13}C NMR (126 MHz, $CDCl_3$) of **1q**

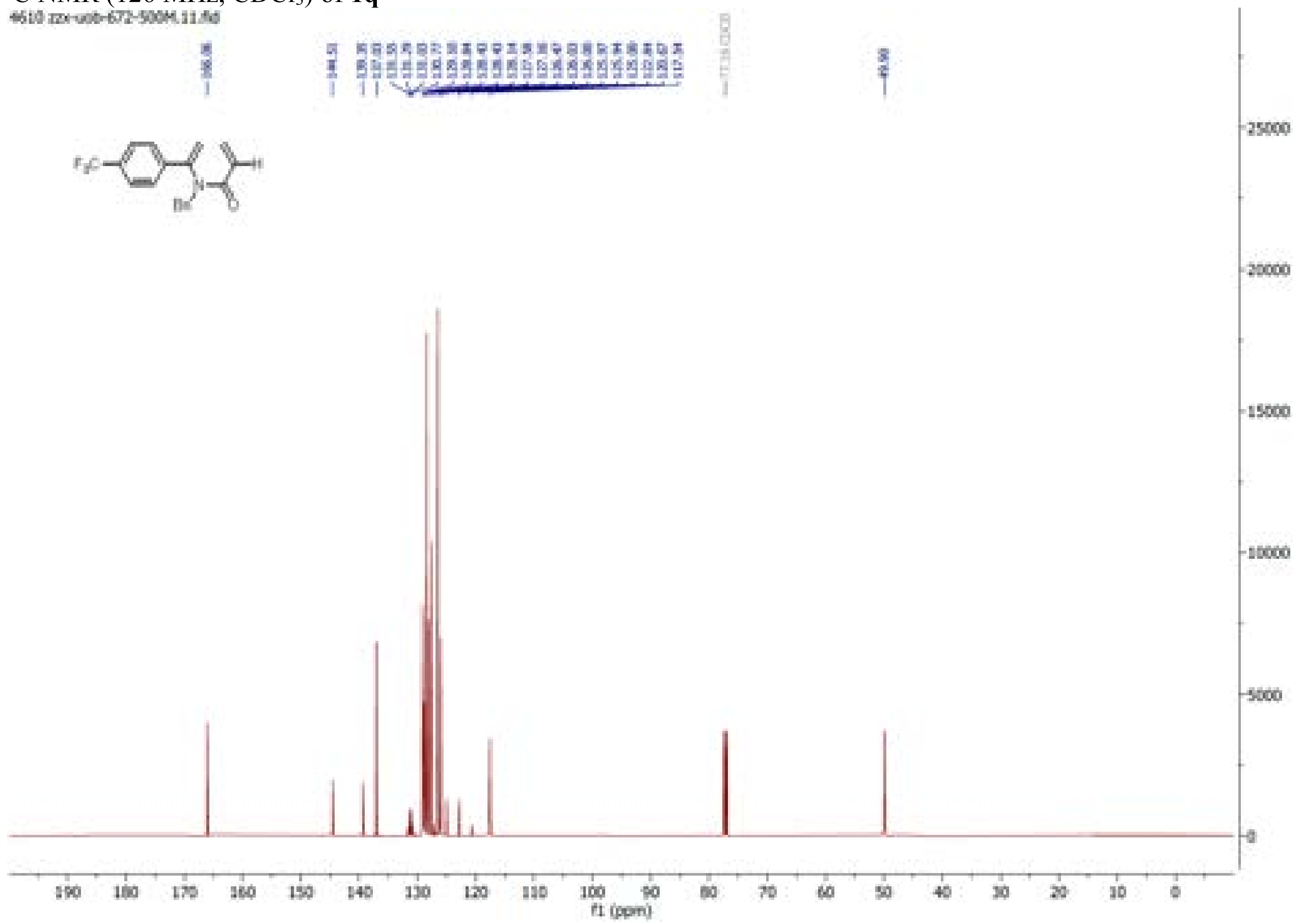

^{19}F NMR (471 MHz, $CDCl_3$) of **1q**

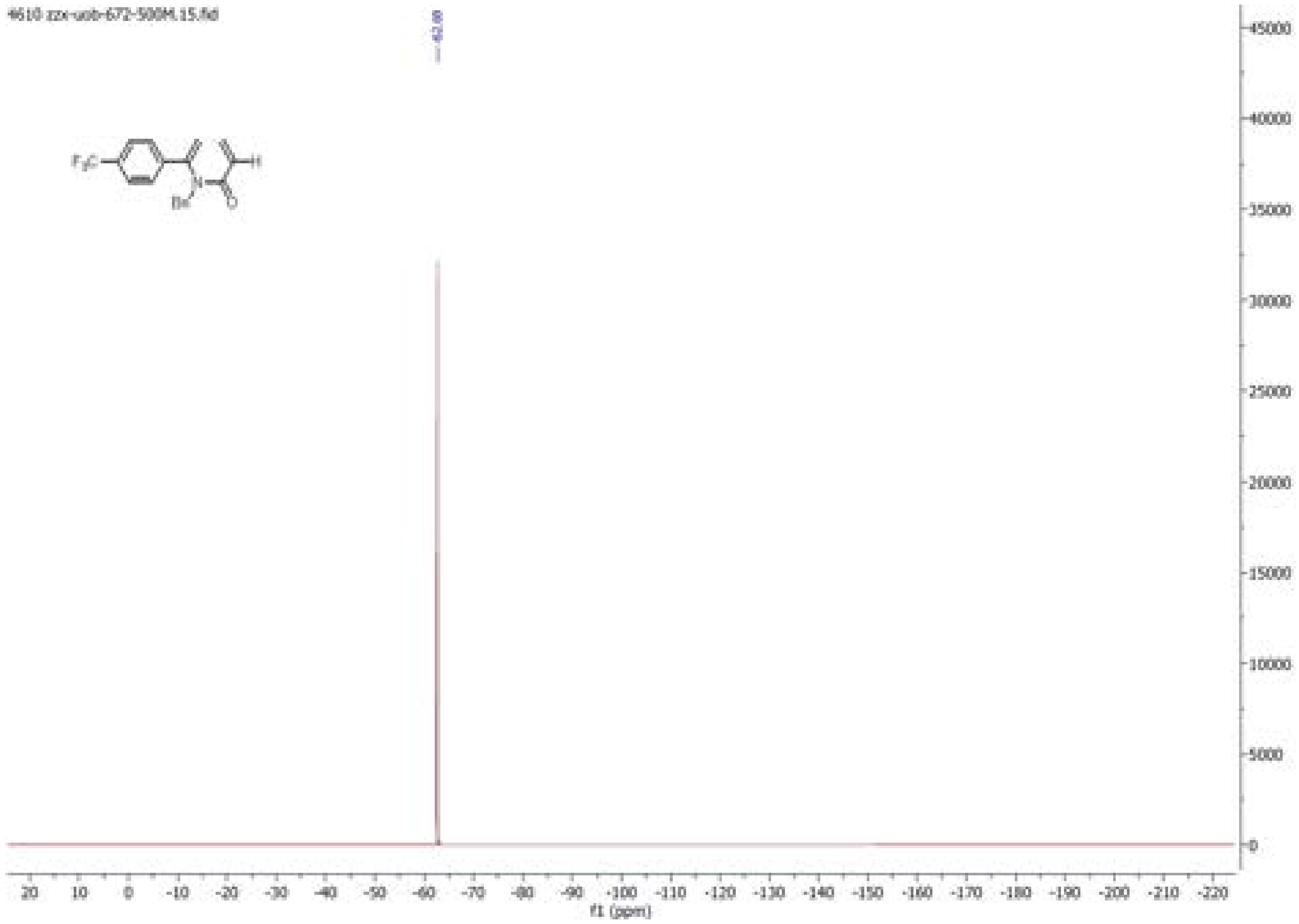

^{1}H NMR (500 MHz, $CDCl_3$) of **1r**

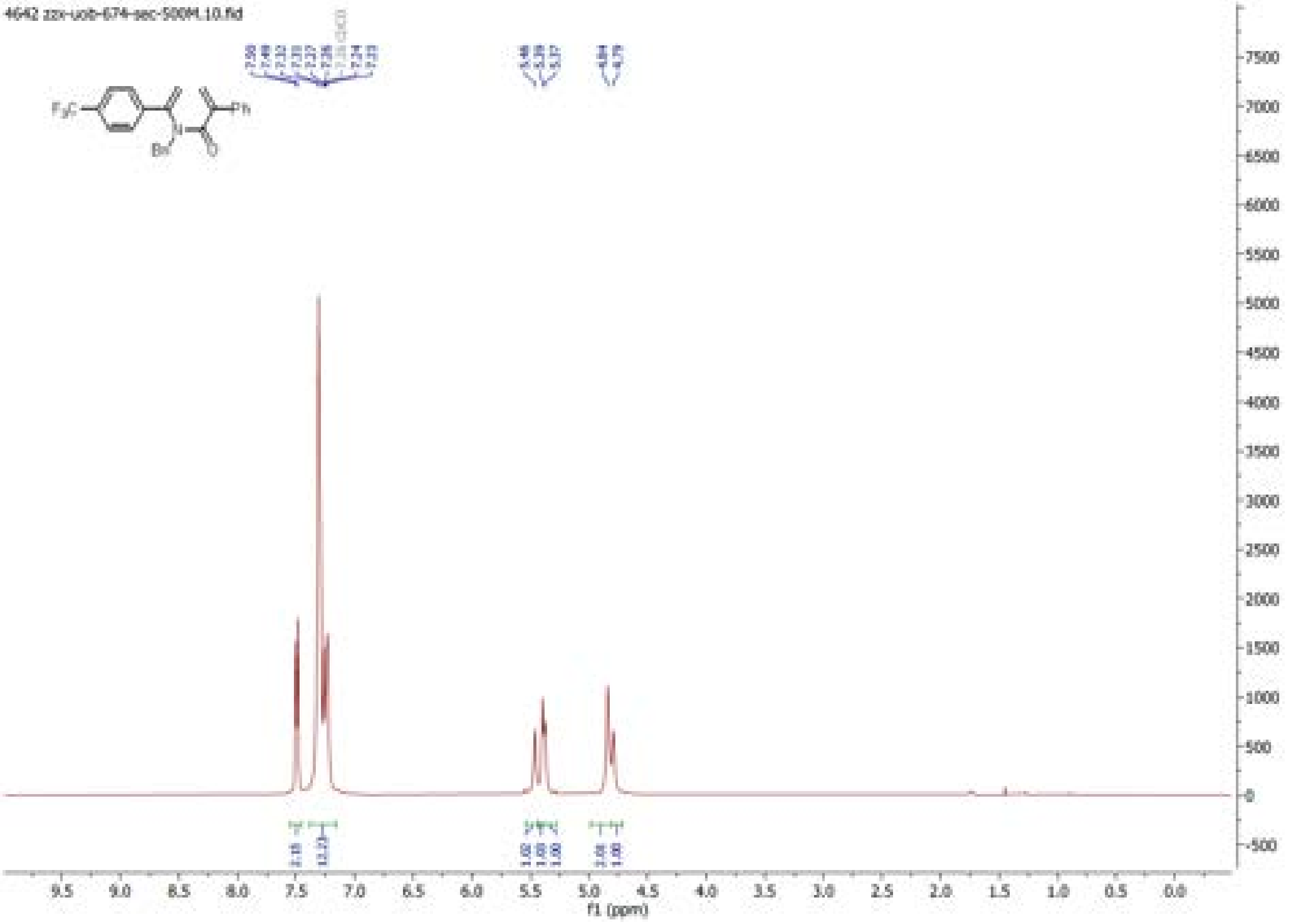

^{13}C NMR (126 MHz, $CDCl_3$) of **1r**

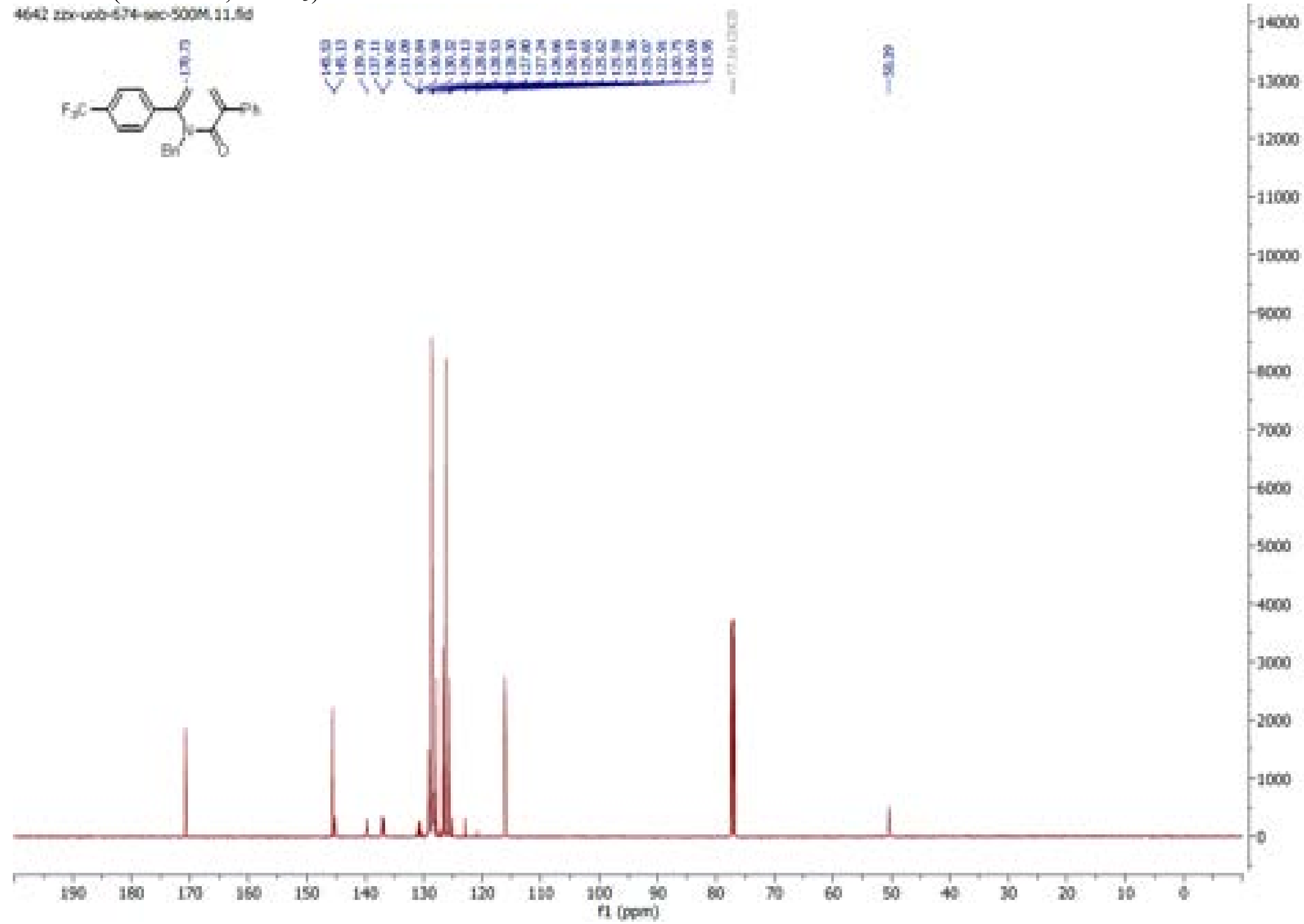

^{19}F NMR (471 MHz, $CDCl_3$) of **1r**

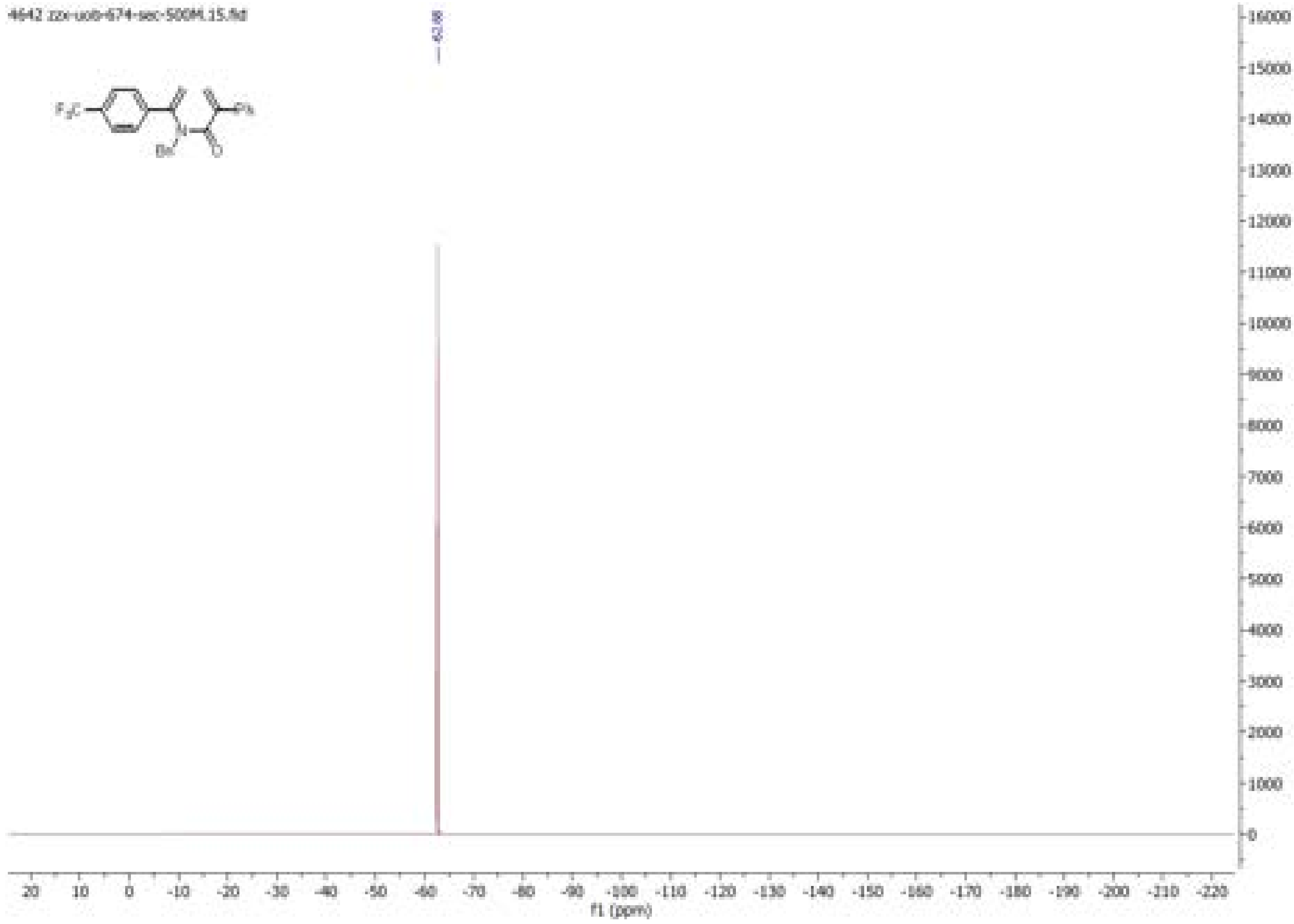

5.2 NMR Spectra of Cyclisation Precursors (7)

^{1}H NMR (500 MHz, $CDCl_3$) of **7a**

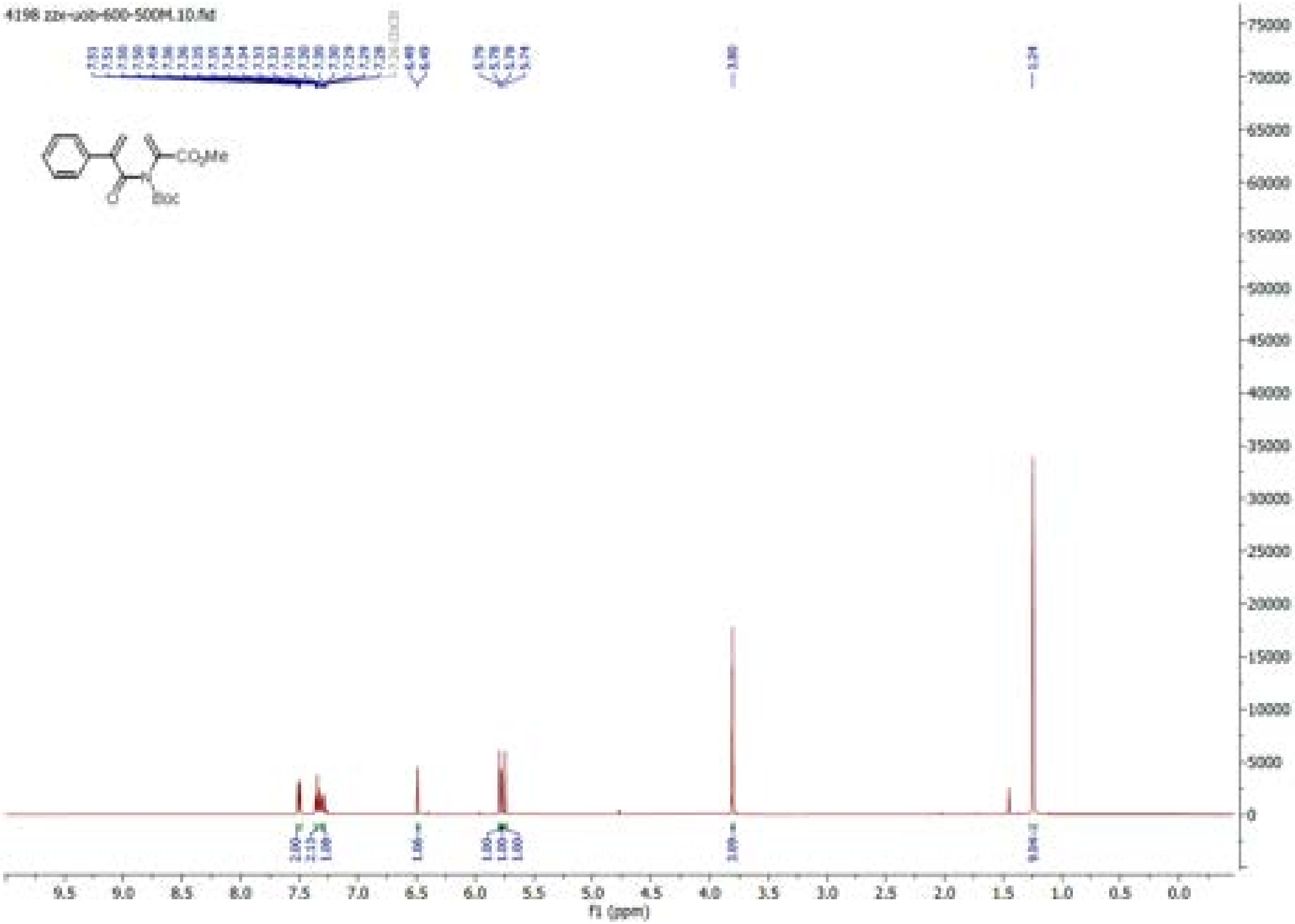

^{13}C NMR (126 MHz, $CDCl_3$) of **7a**

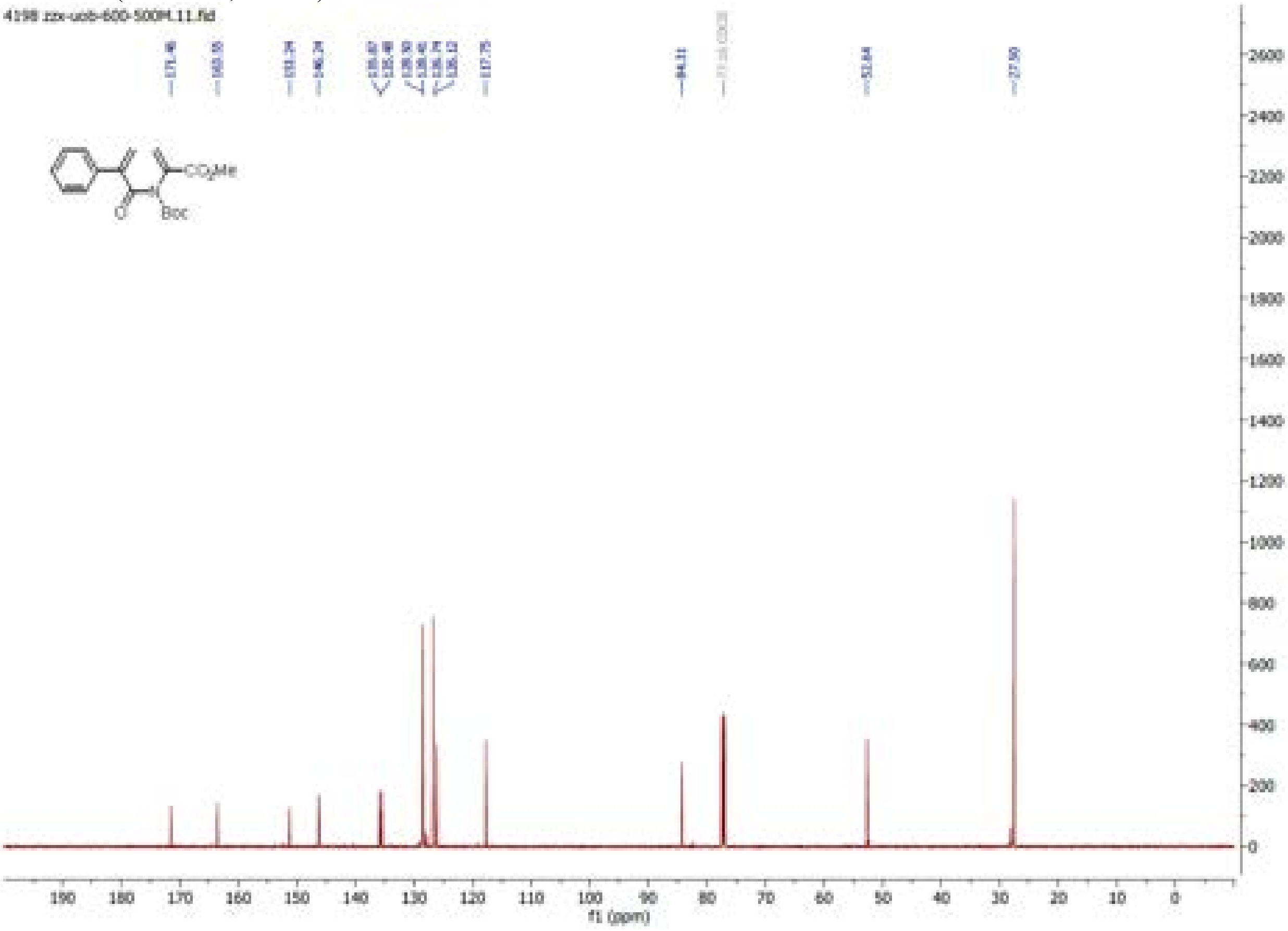

^{1}H NMR (500 MHz, $CDCl_3$) of **7b**

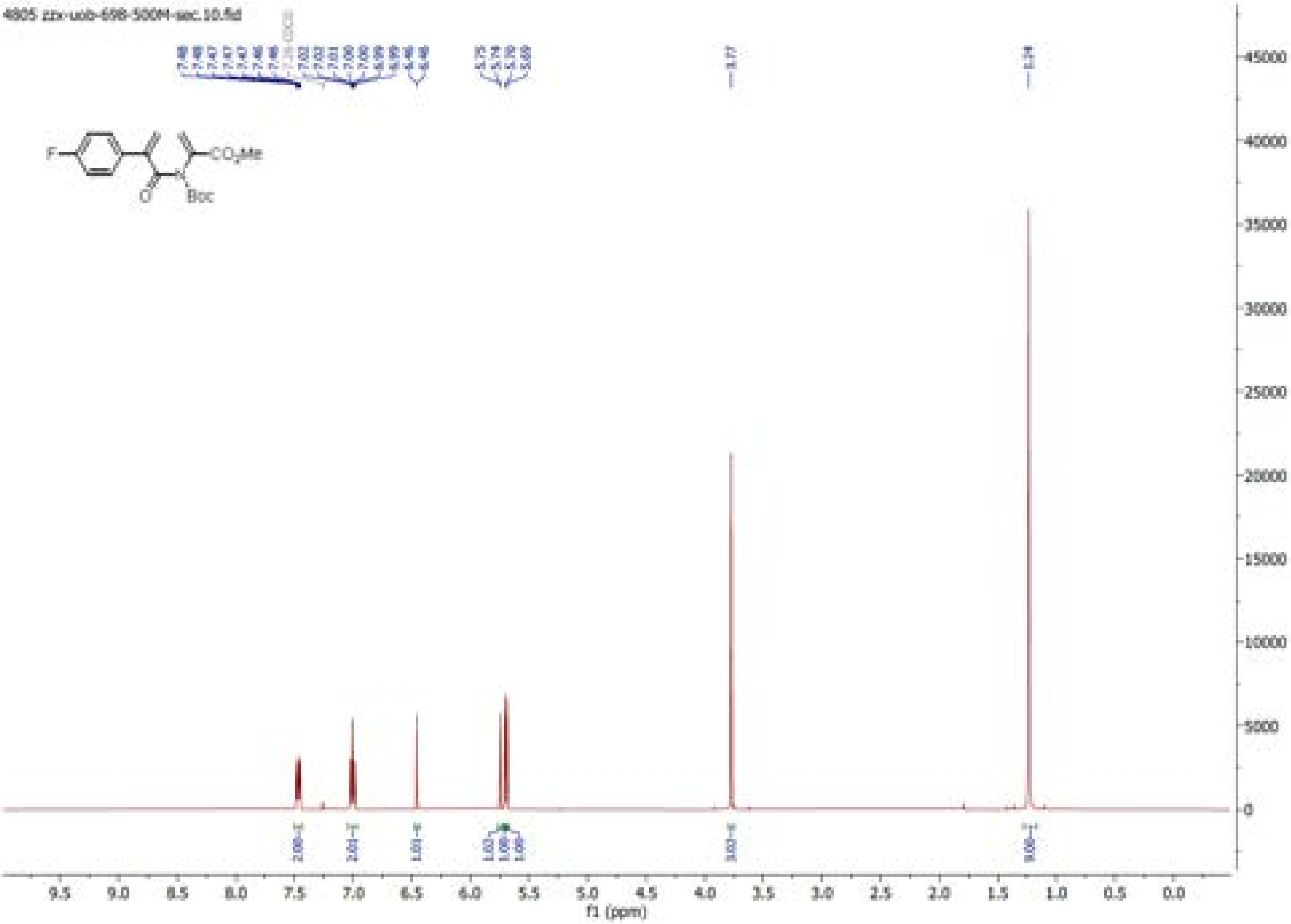

^{13}C NMR (126 MHz, $CDCl_3$) of **7b**

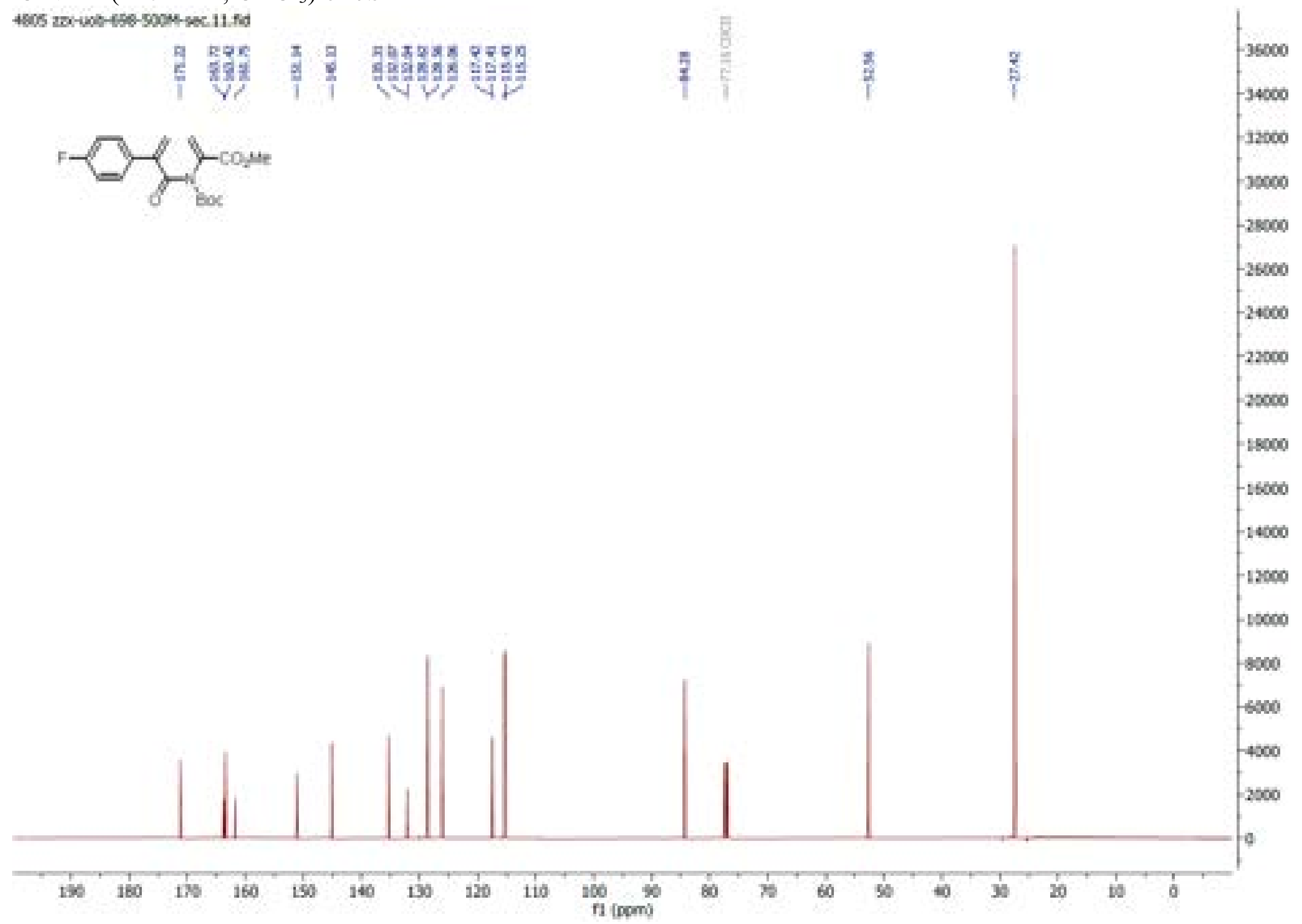

^{19}F NMR (471 MHz, $CDCl_3$) of **7b**

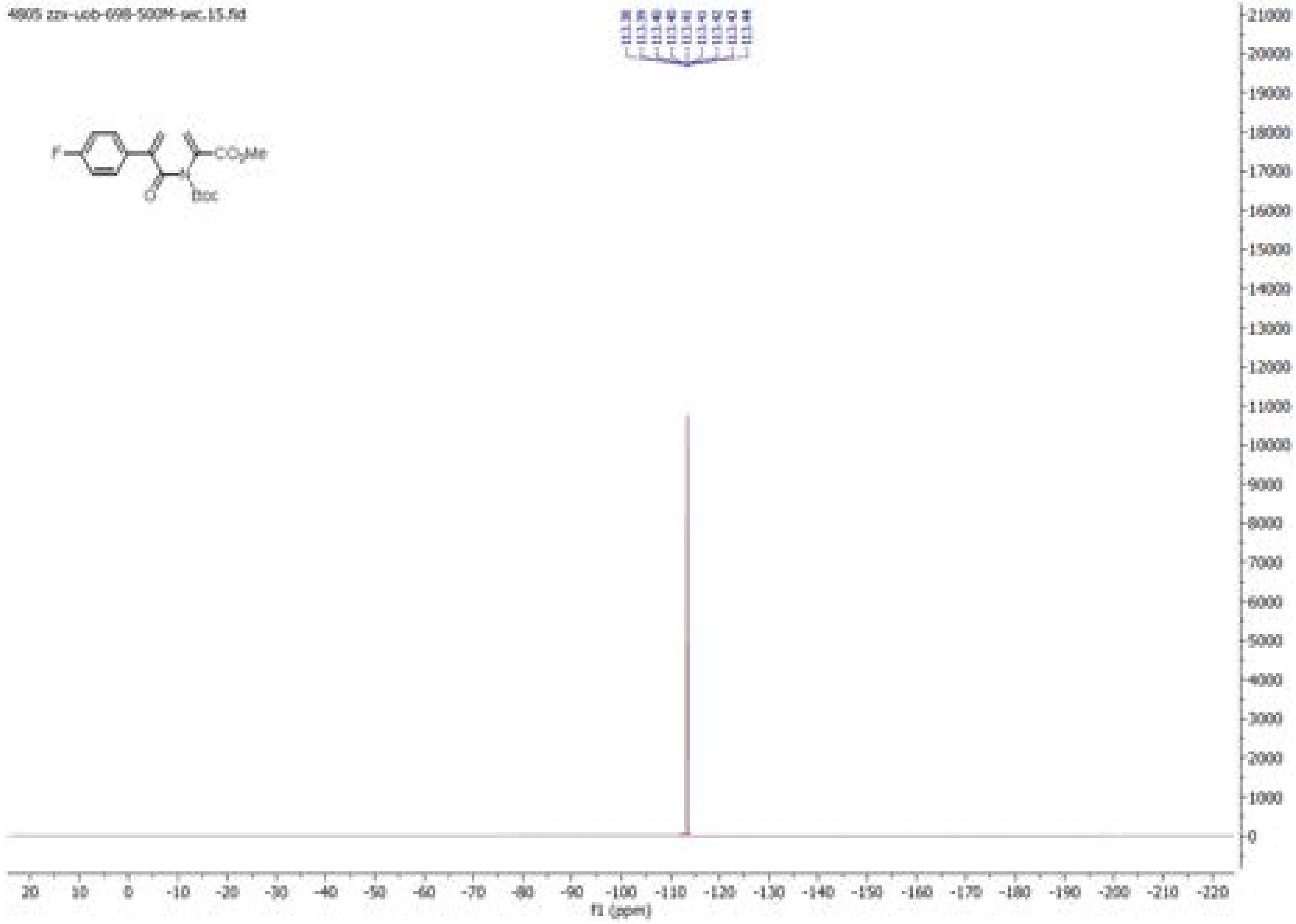

^{1}H NMR (500 MHz, $CDCl_3$) of **7c**

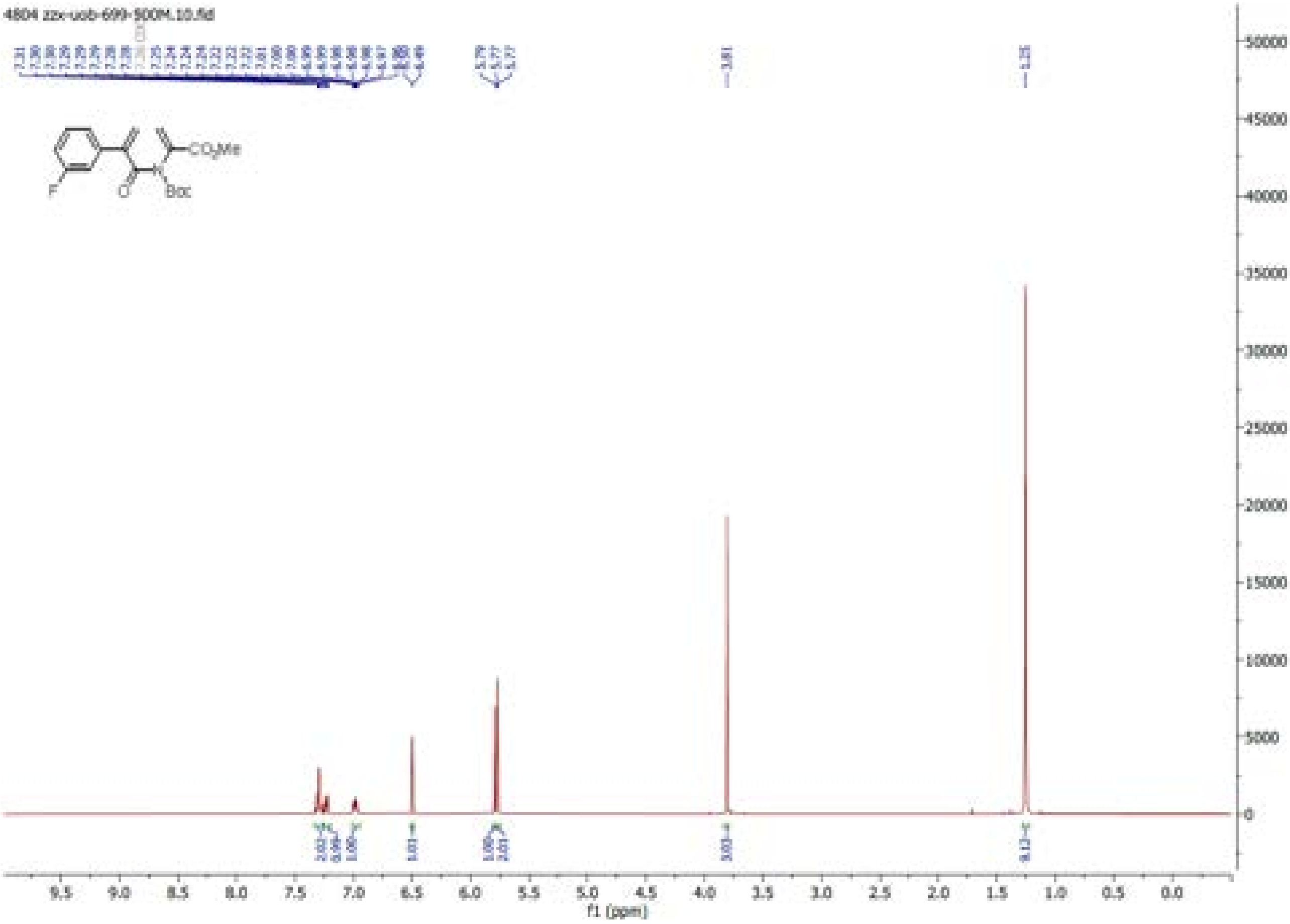

^{13}C NMR (126 MHz, $CDCl_3$) of **7c**

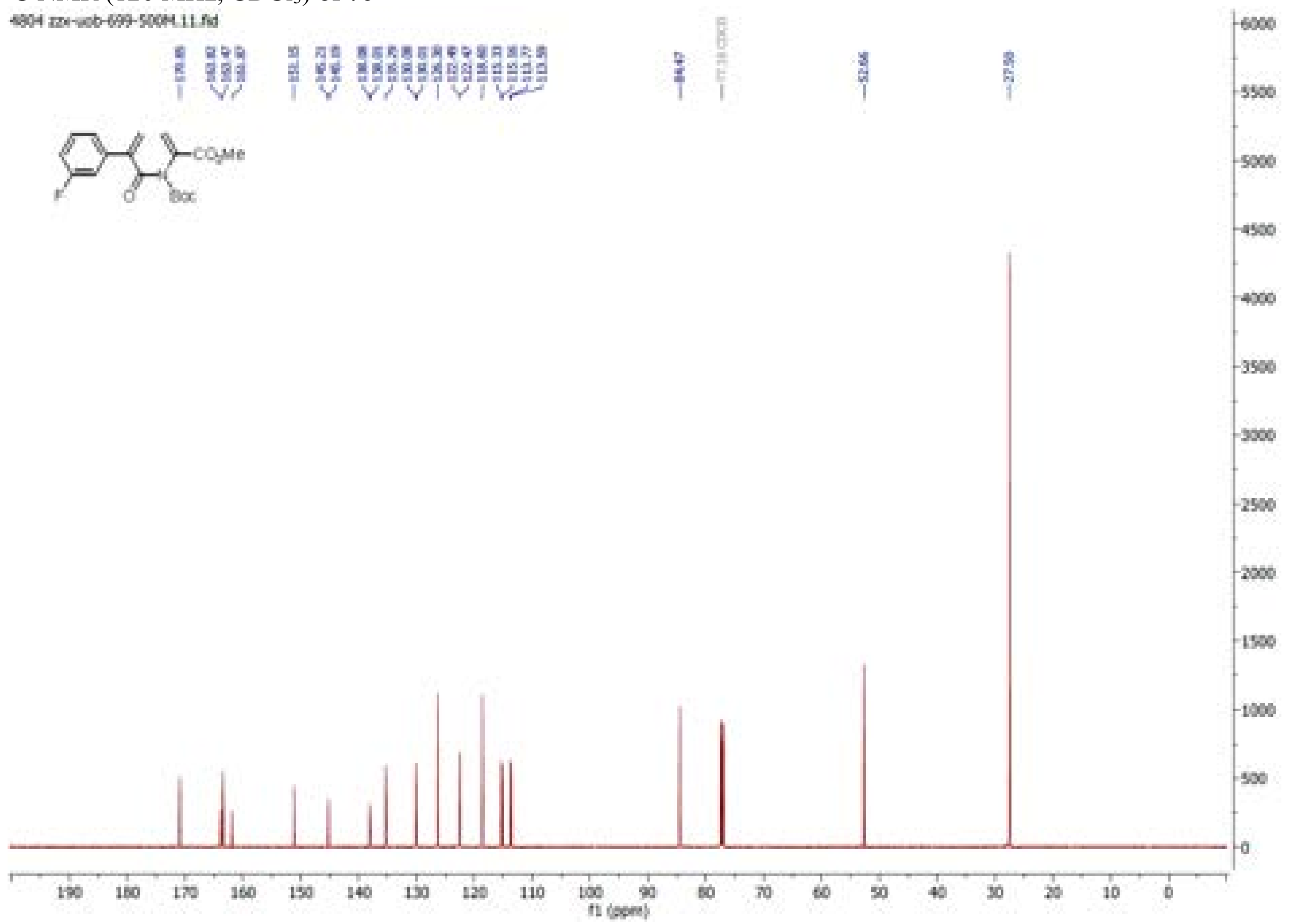

^{19}F NMR (471 MHz, $CDCl_3$) of **7c**

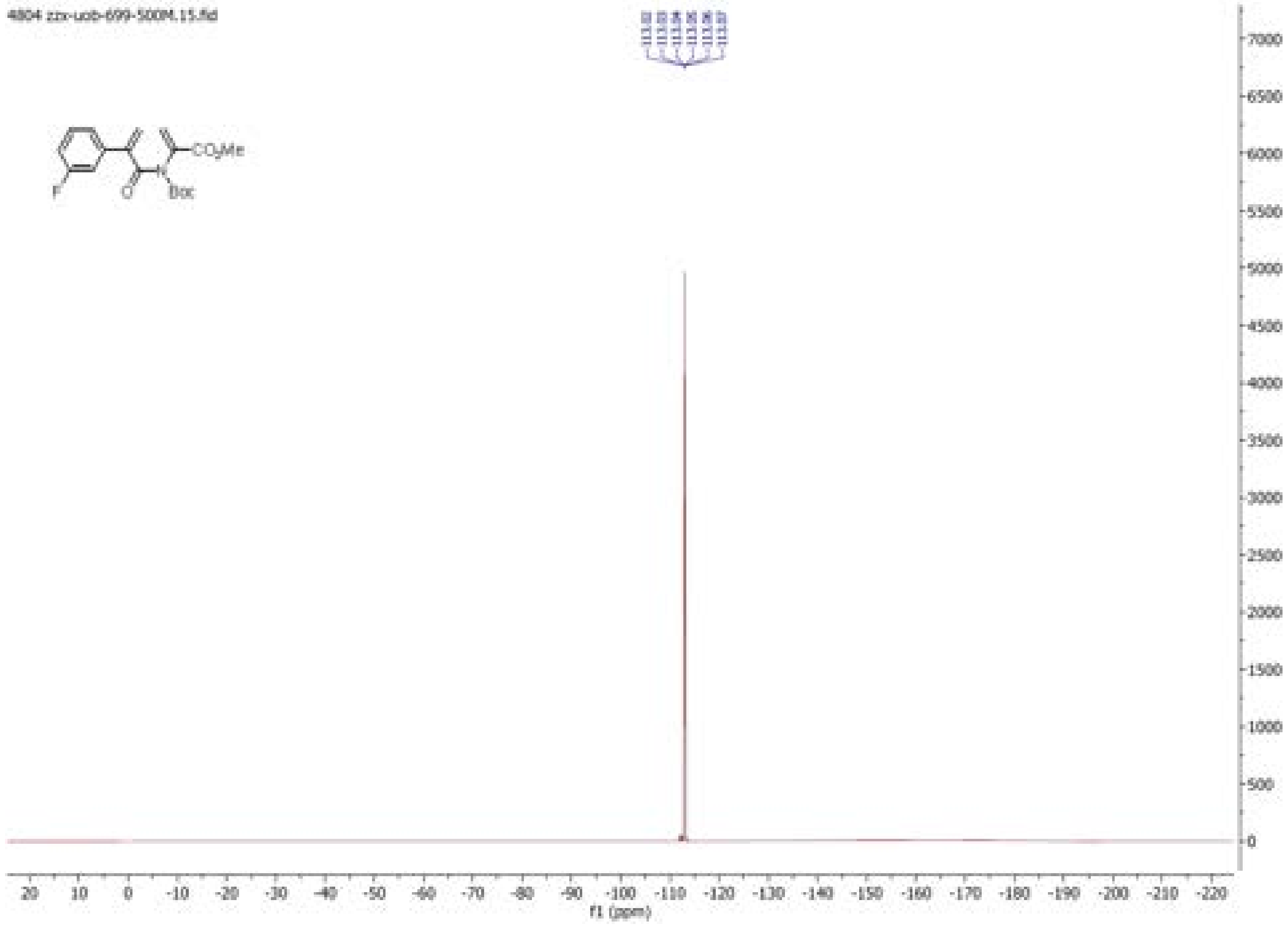

^{1}H NMR (500 MHz, $CDCl_3$) of **7d**

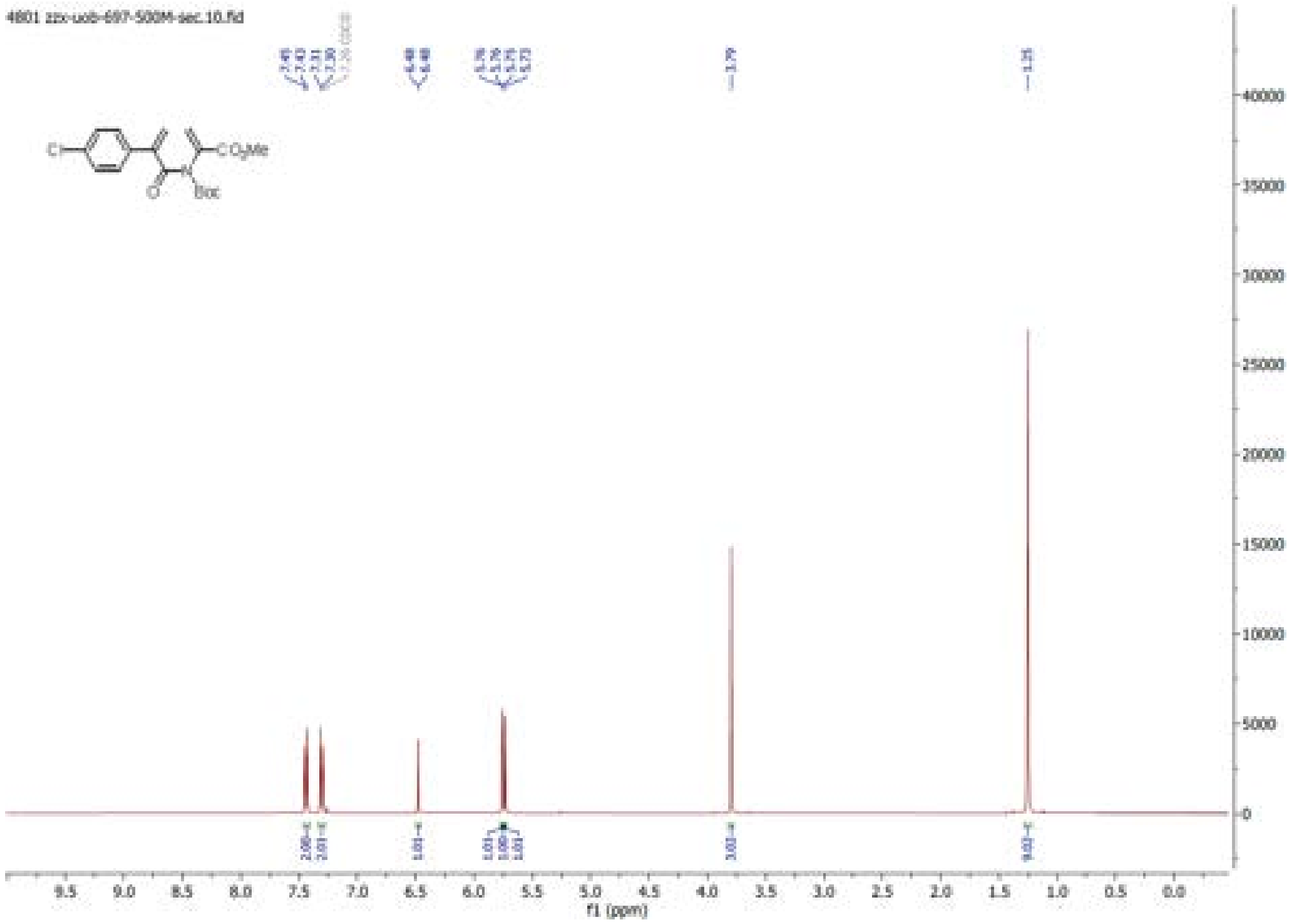

^{13}C NMR (126 MHz, $CDCl_3$) of **7d**

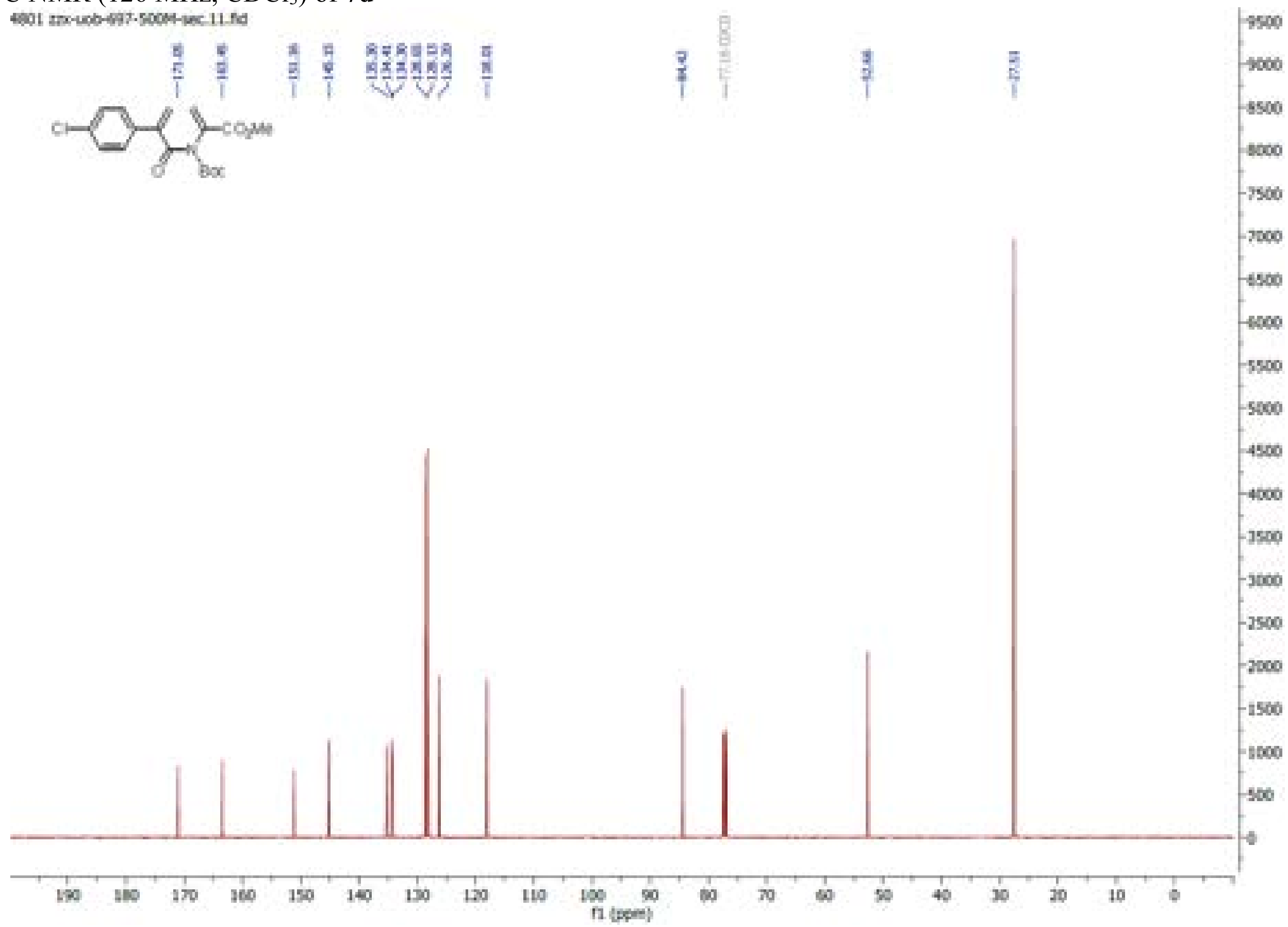

^{1}H NMR (500 MHz, $CDCl_3$) of **7e**

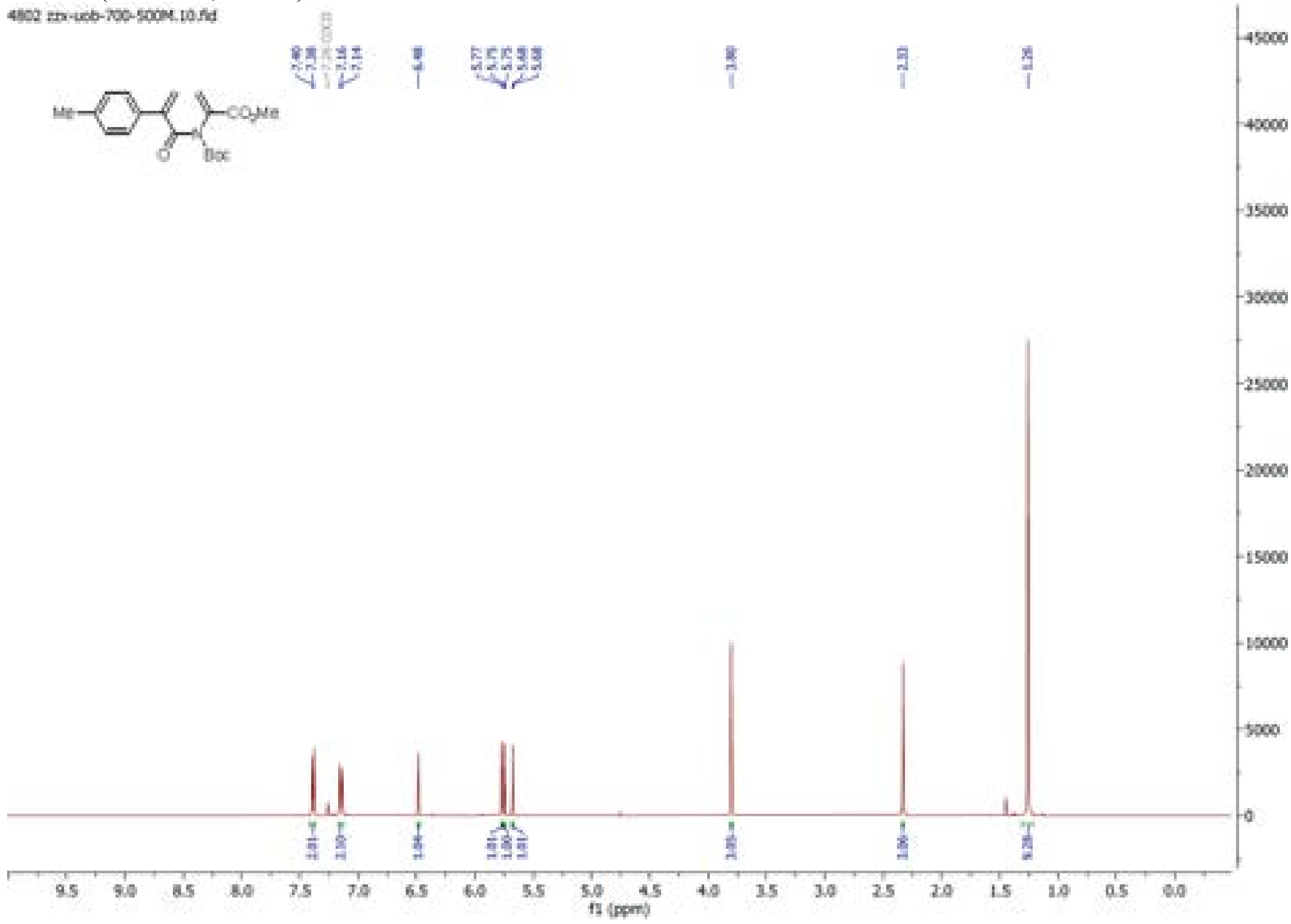

^{13}C NMR (126 MHz, $CDCl_3$) of **7e**

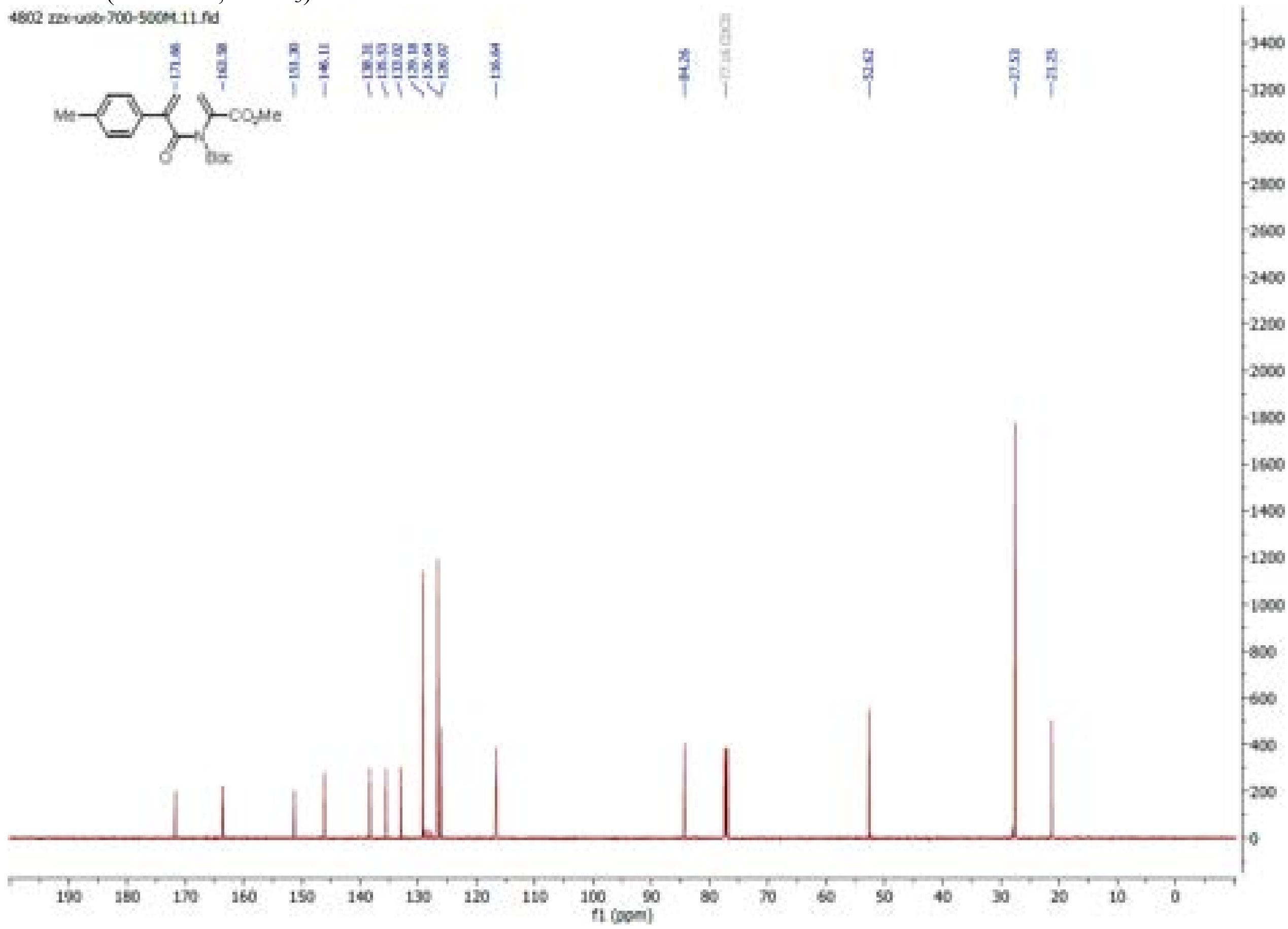

^{1}H NMR (500 MHz, $CDCl_3$) of **7f**

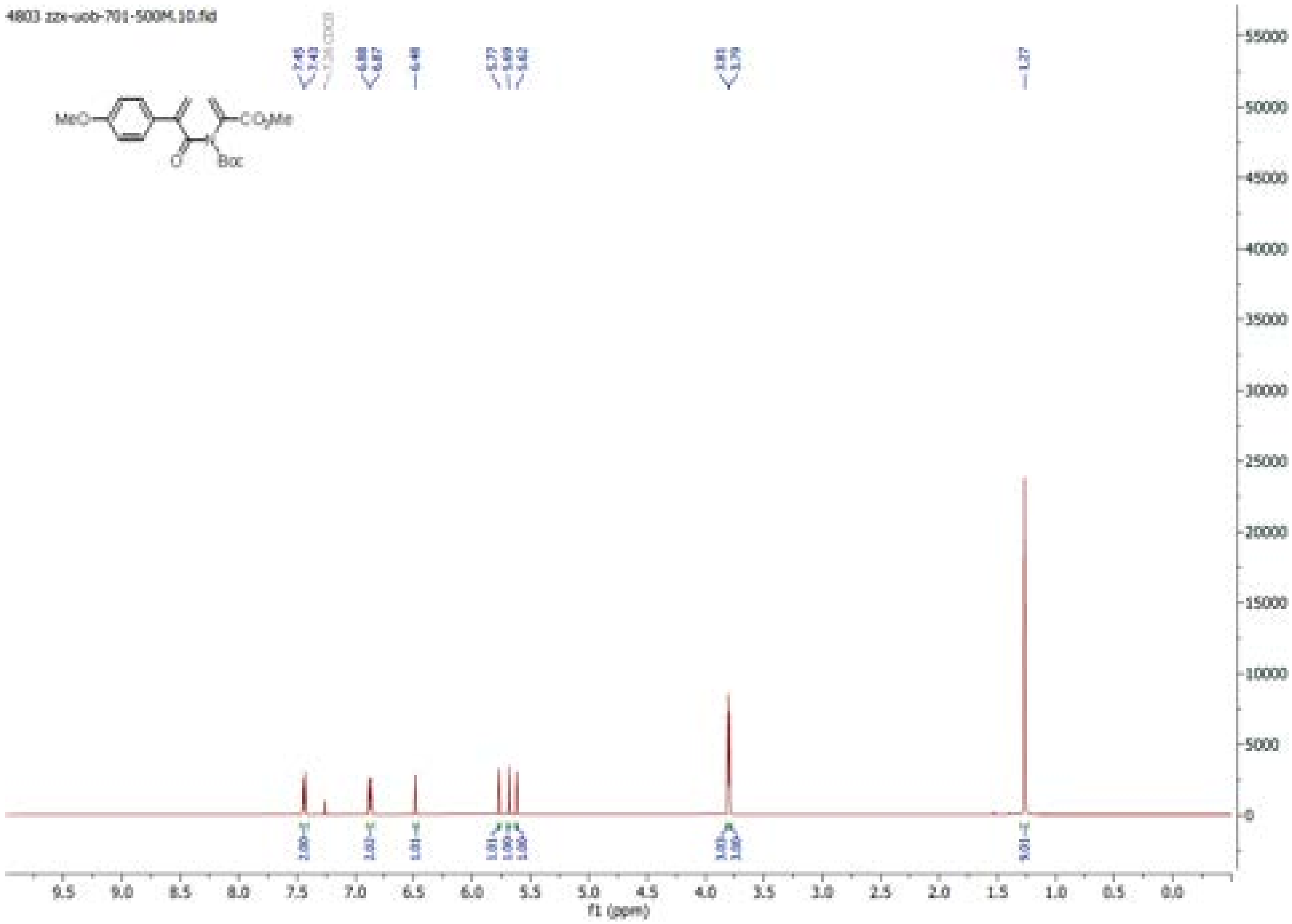

^{13}C NMR (126 MHz, $CDCl_3$) of **7f**

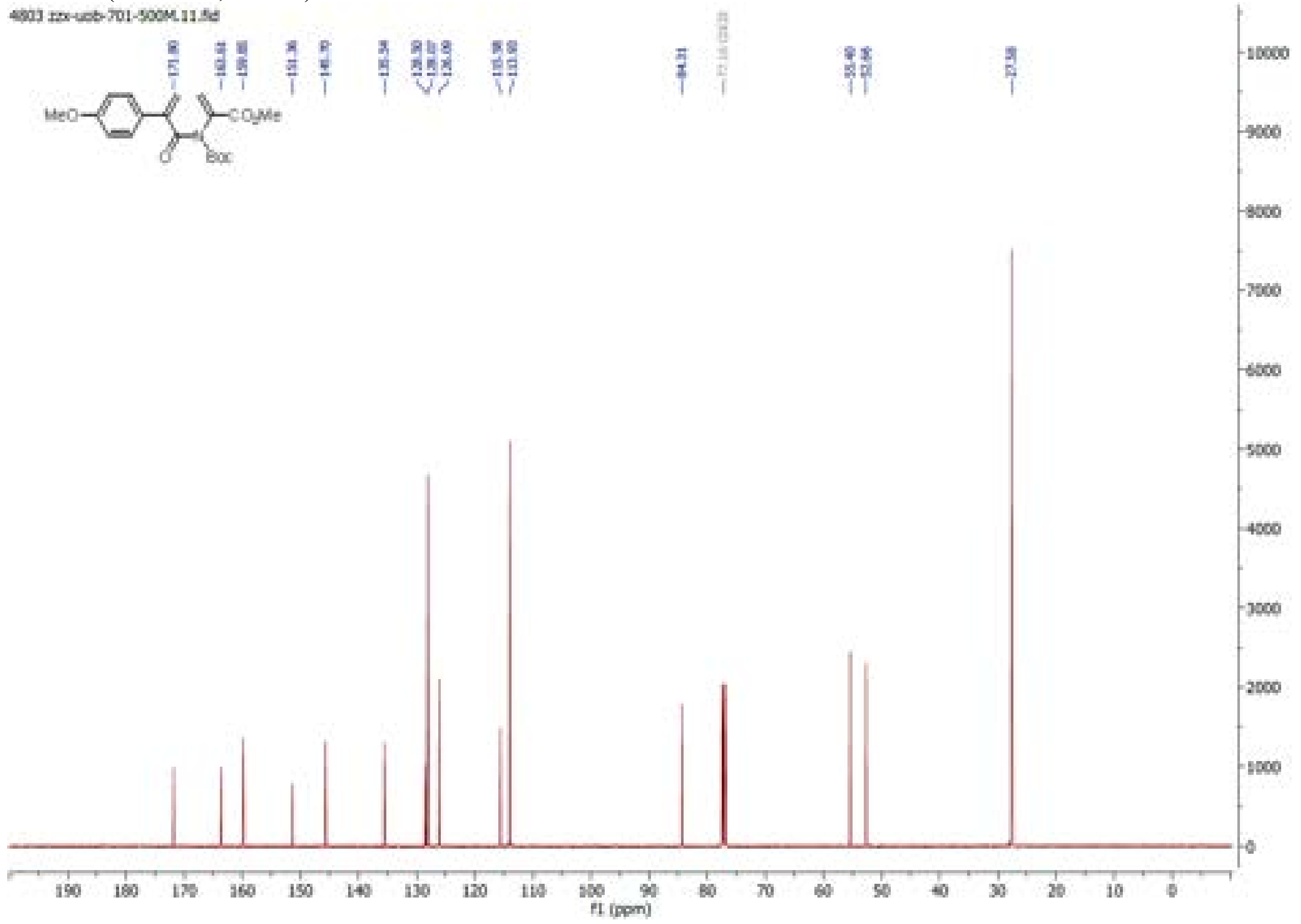

^{1}H NMR (500 MHz, $CDCl_3$) of **7g**

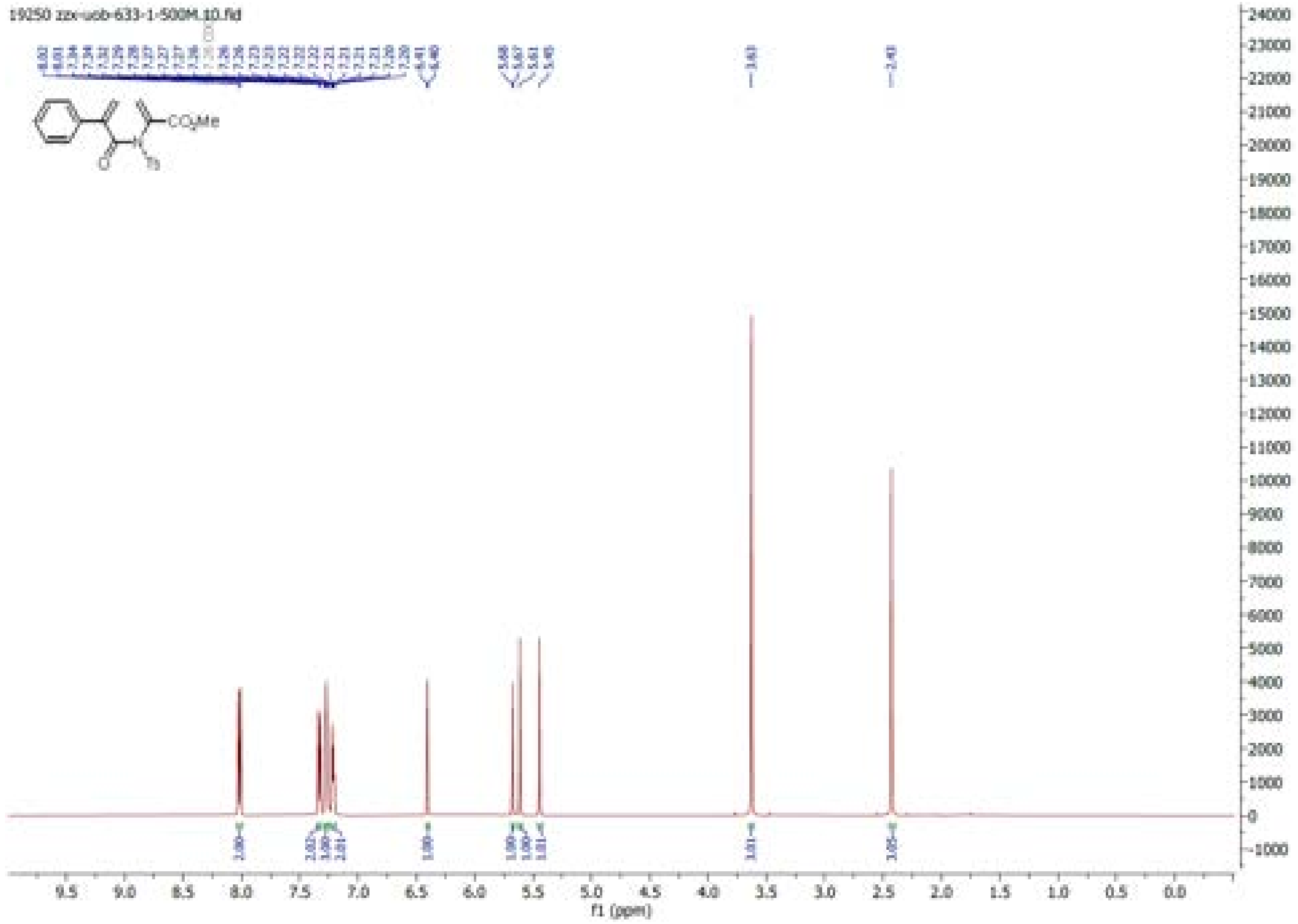

^{13}C NMR (126 MHz, $CDCl_3$) of **7g**

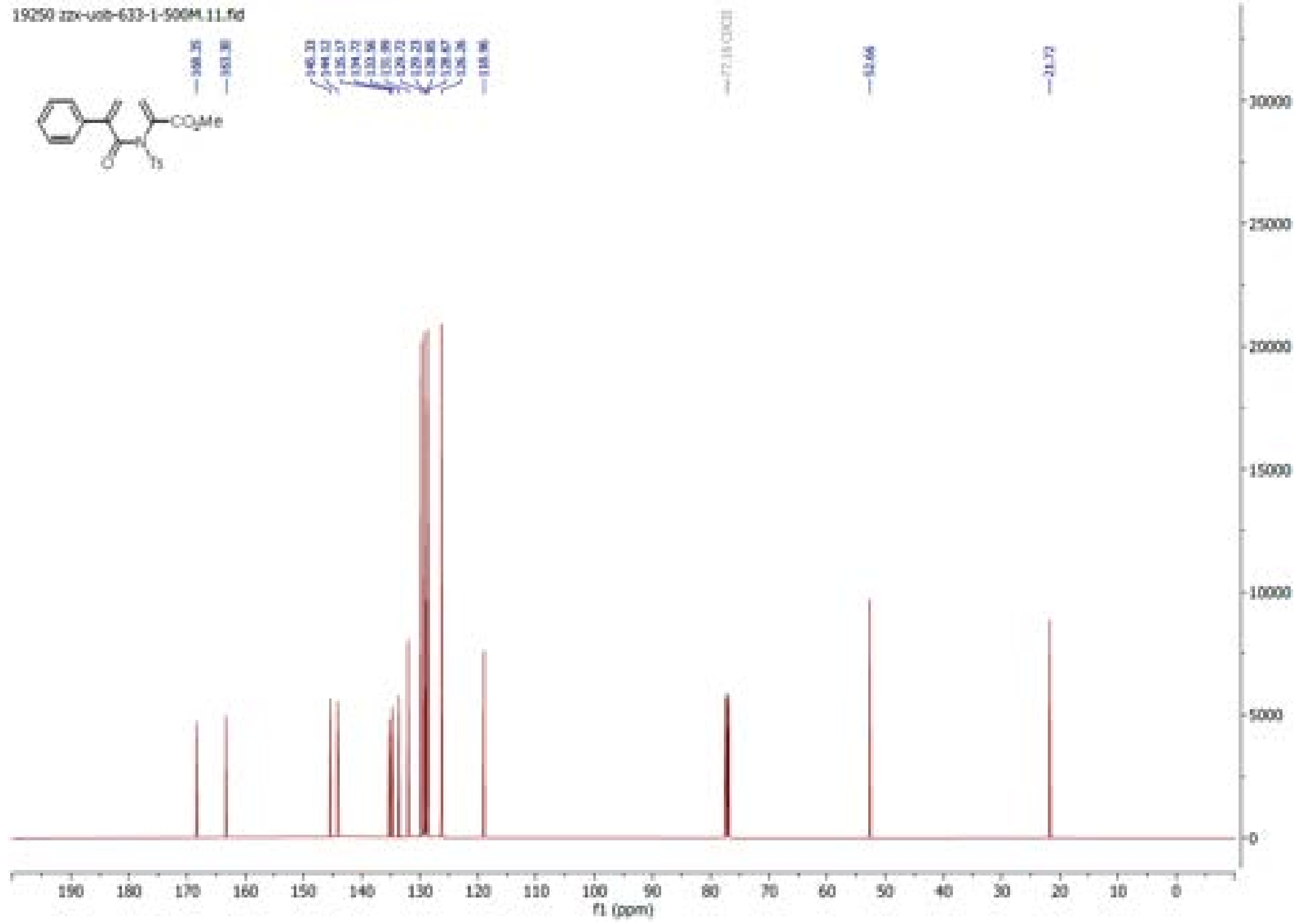

^{1}H NMR (500 MHz, CD_2Cl_2) of **7h**

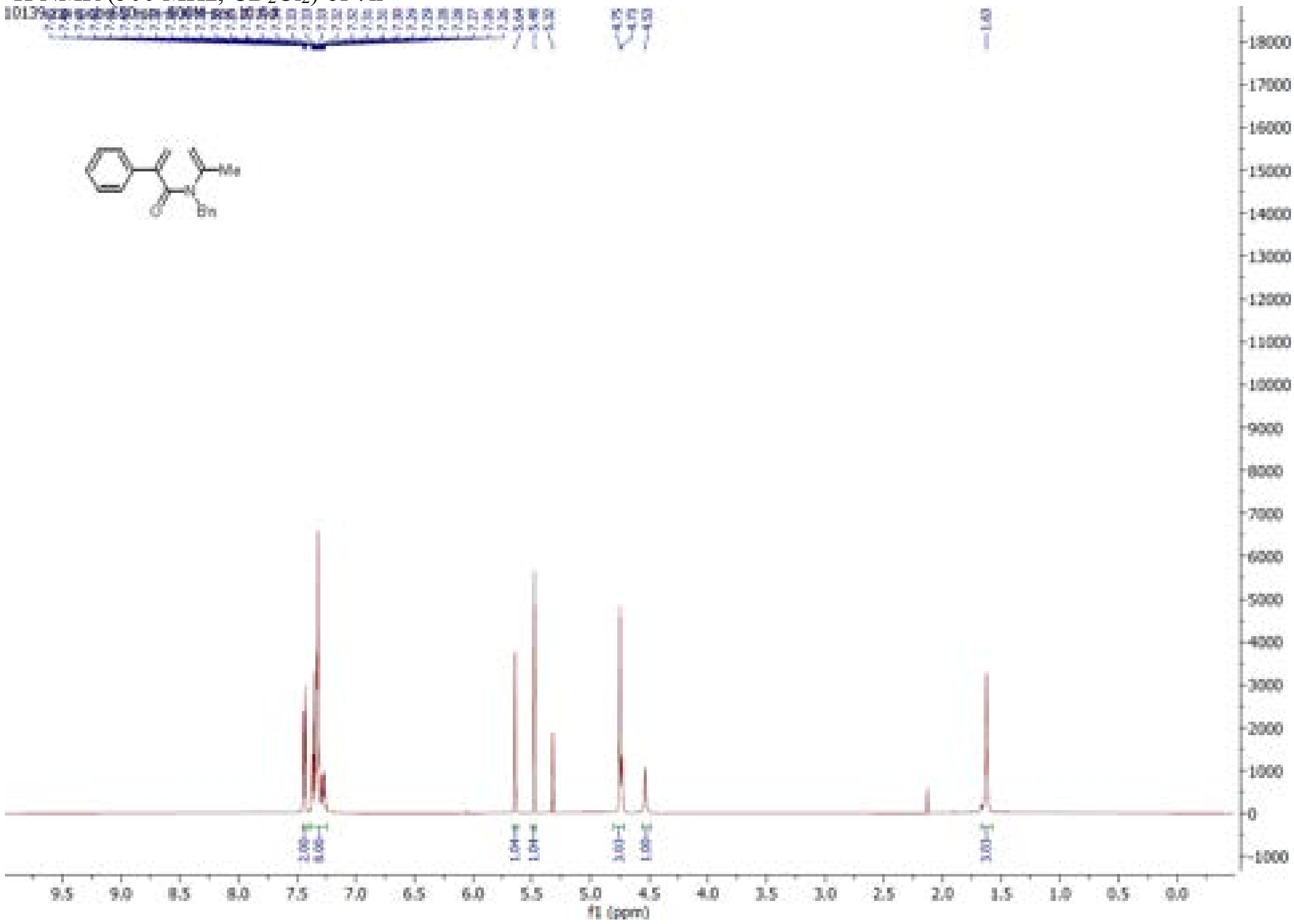

^{13}C NMR (126 MHz, CD_2Cl_2) of **7h**

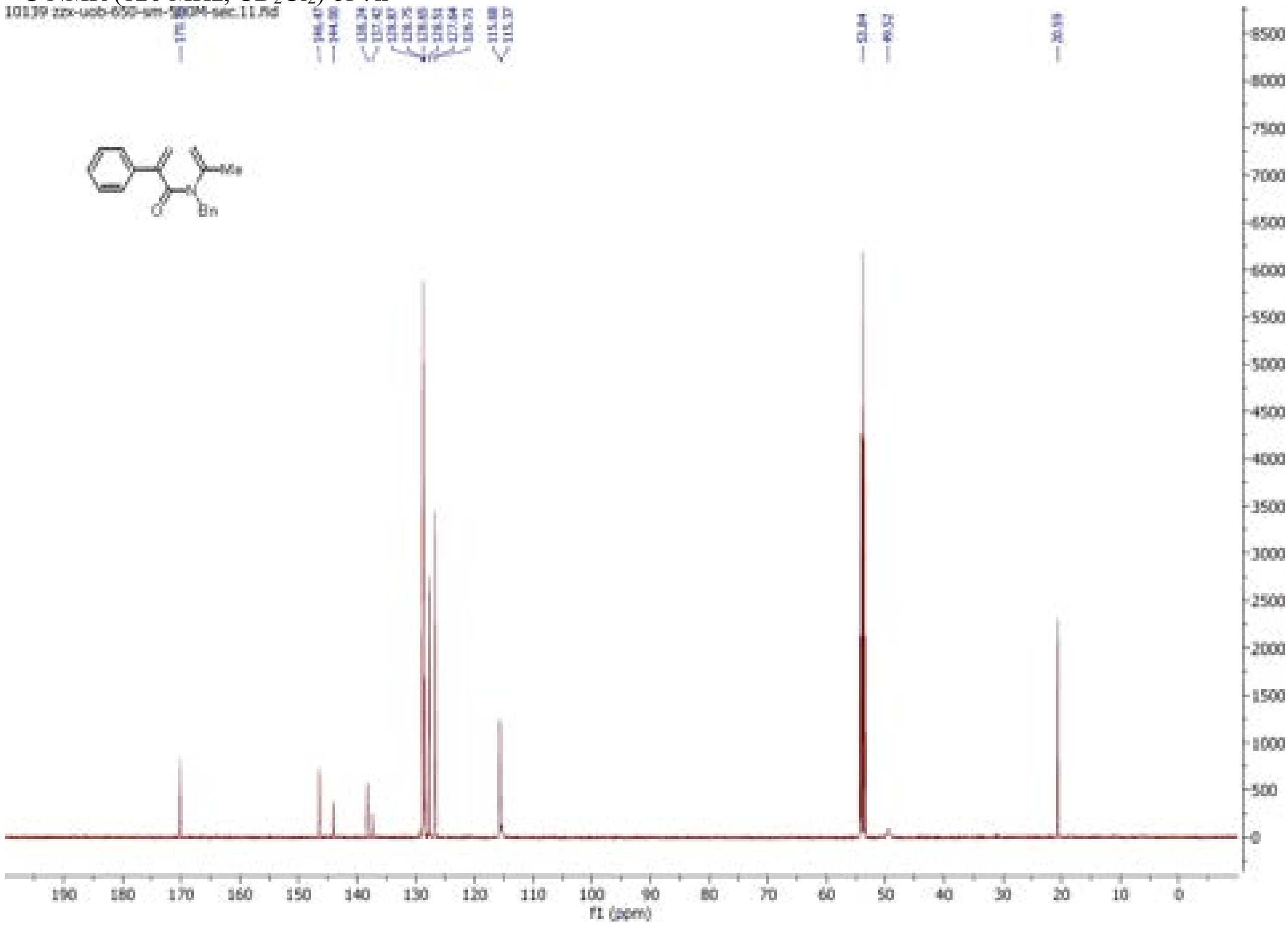

5.3 NMR Spectra of Cyclisation Precursors (2)

^{1}H NMR (500 MHz, $CDCl_3$) of **S2a**

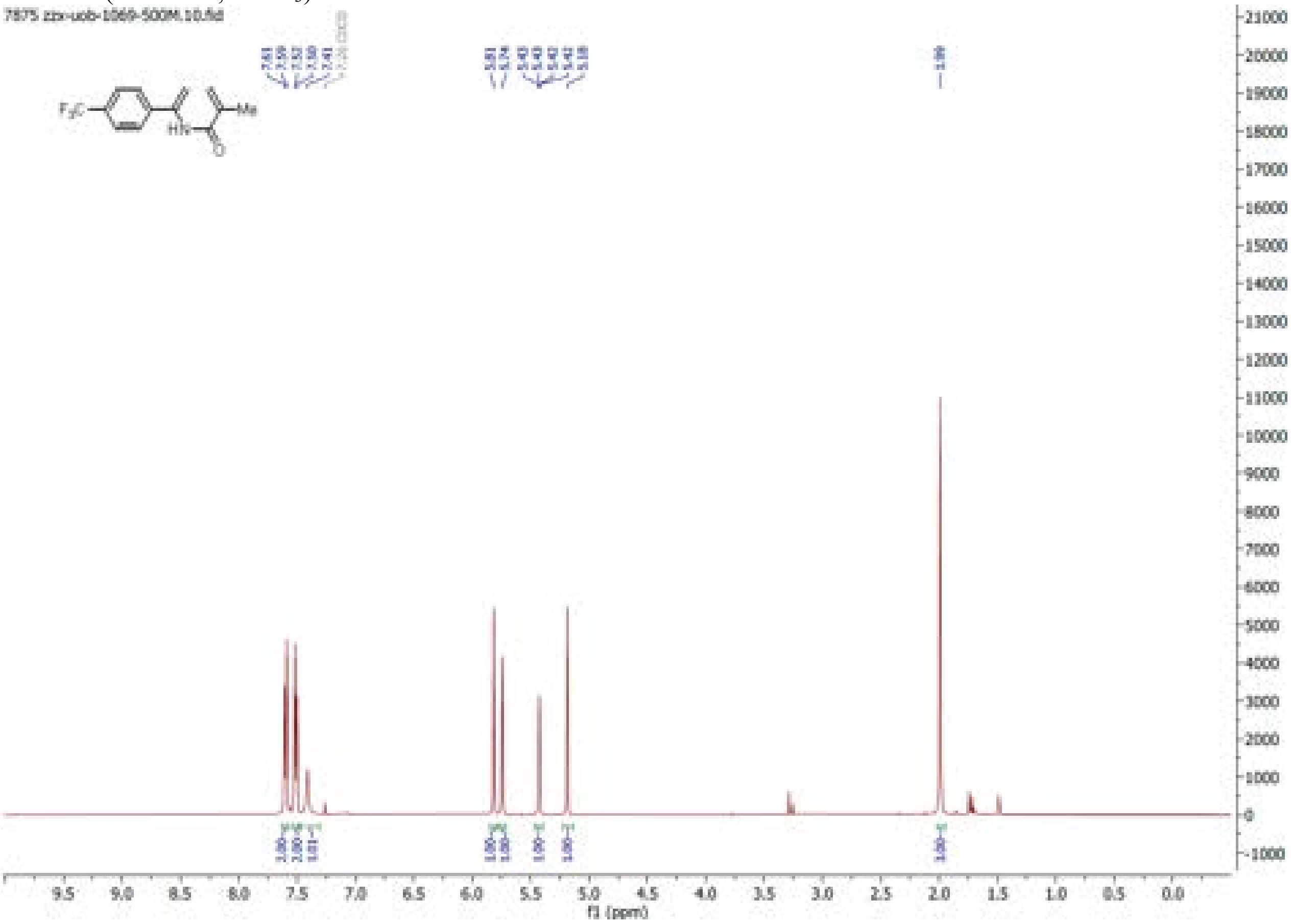

^{13}C NMR (126 MHz, $CDCl_3$) of **S2a**

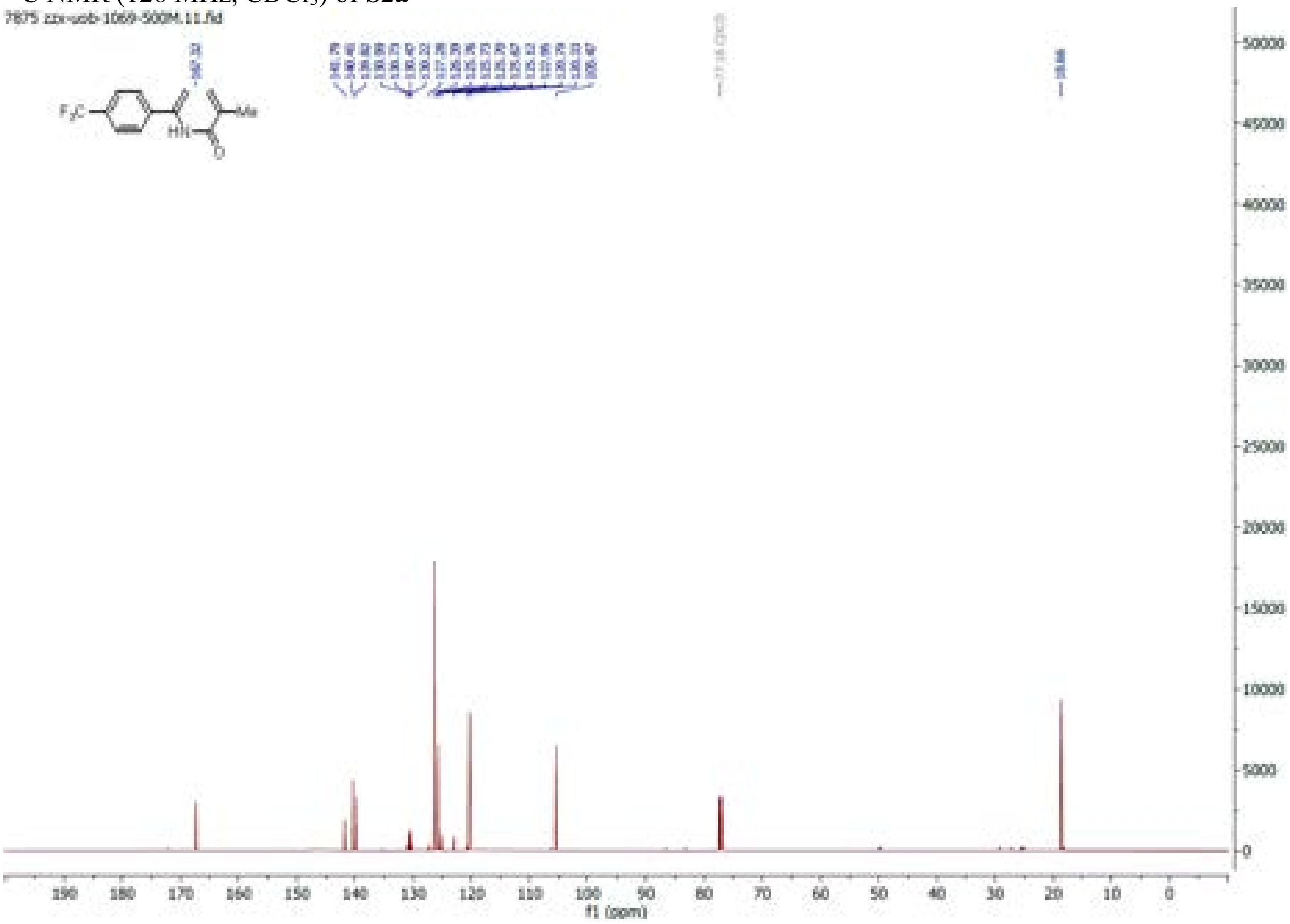

^{19}F NMR (471 MHz, $CDCl_3$) of **S2a**

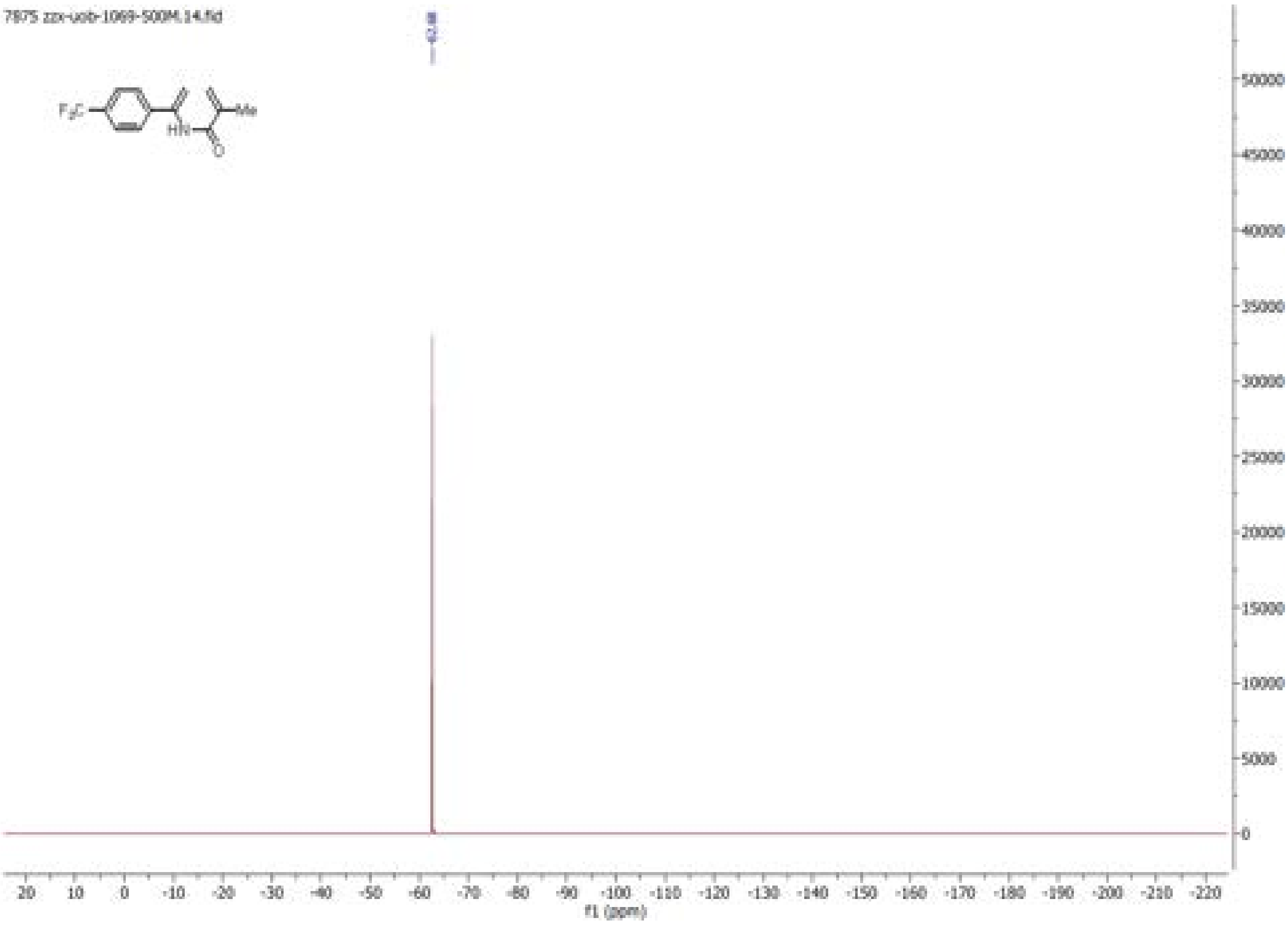

^{1}H NMR (500 MHz, $CDCl_3$) of **2a**

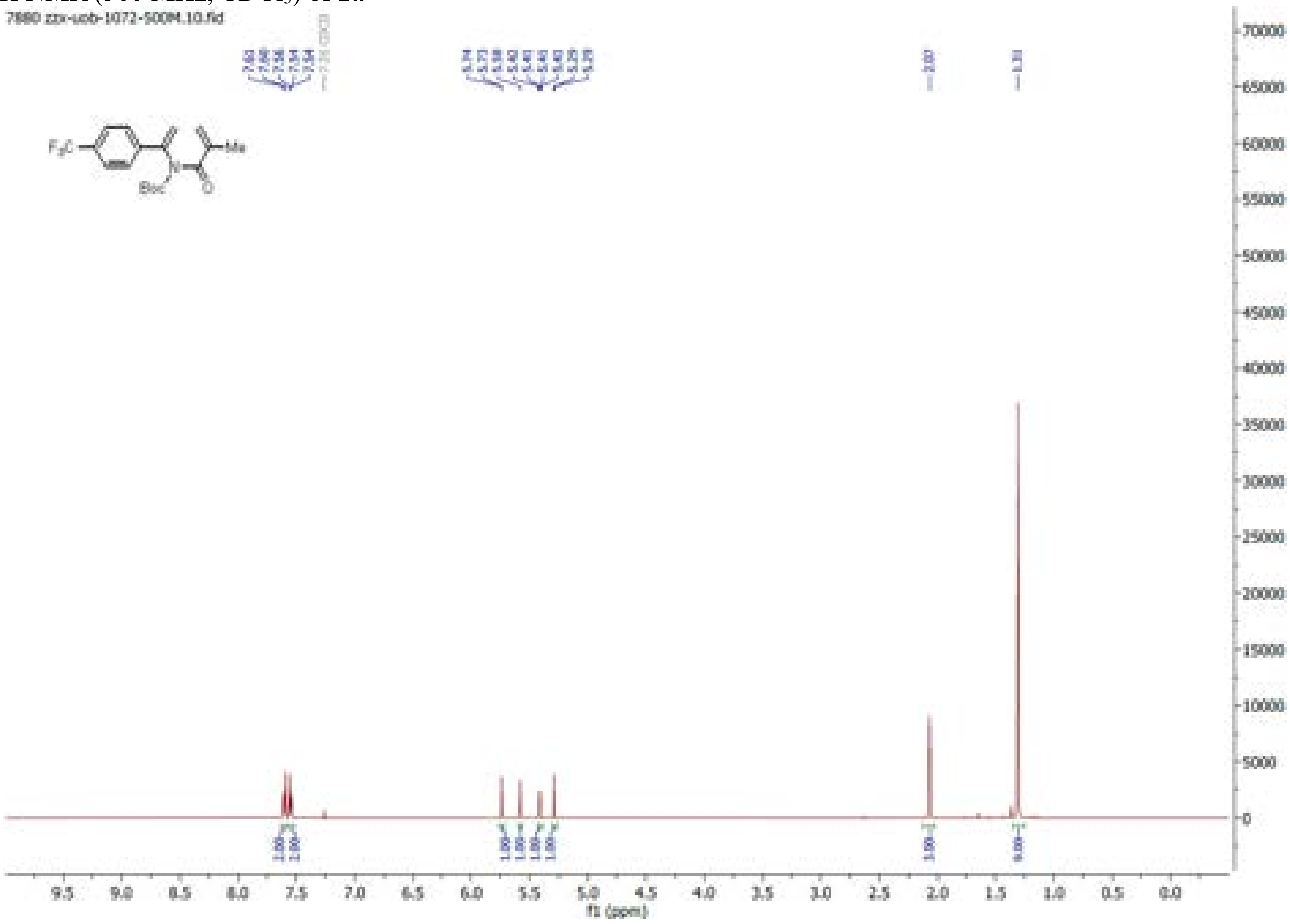

^{13}C NMR (126 MHz, $CDCl_3$) of **2a**

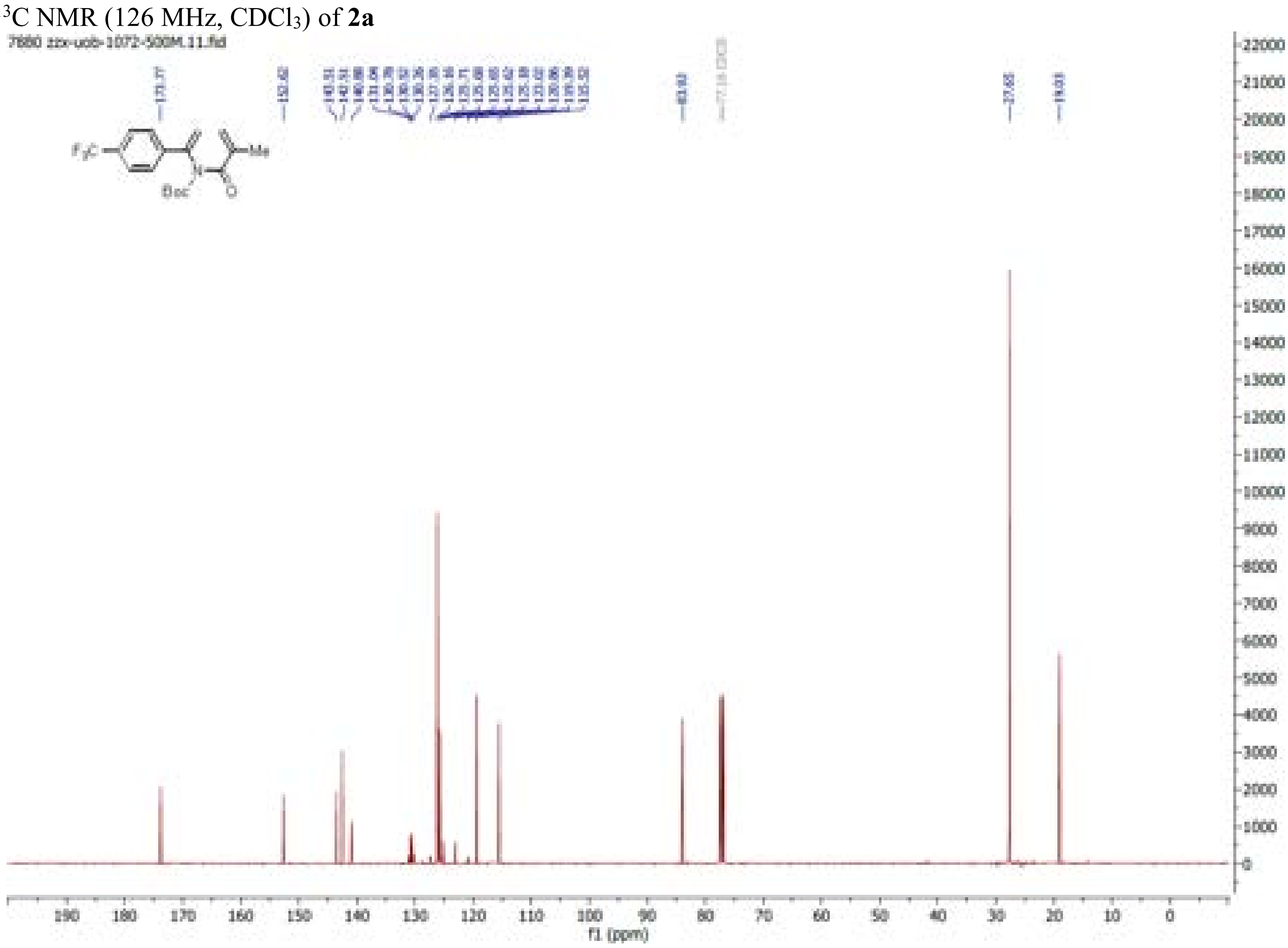

^{19}F NMR (471 MHz, $CDCl_3$) of **2a**

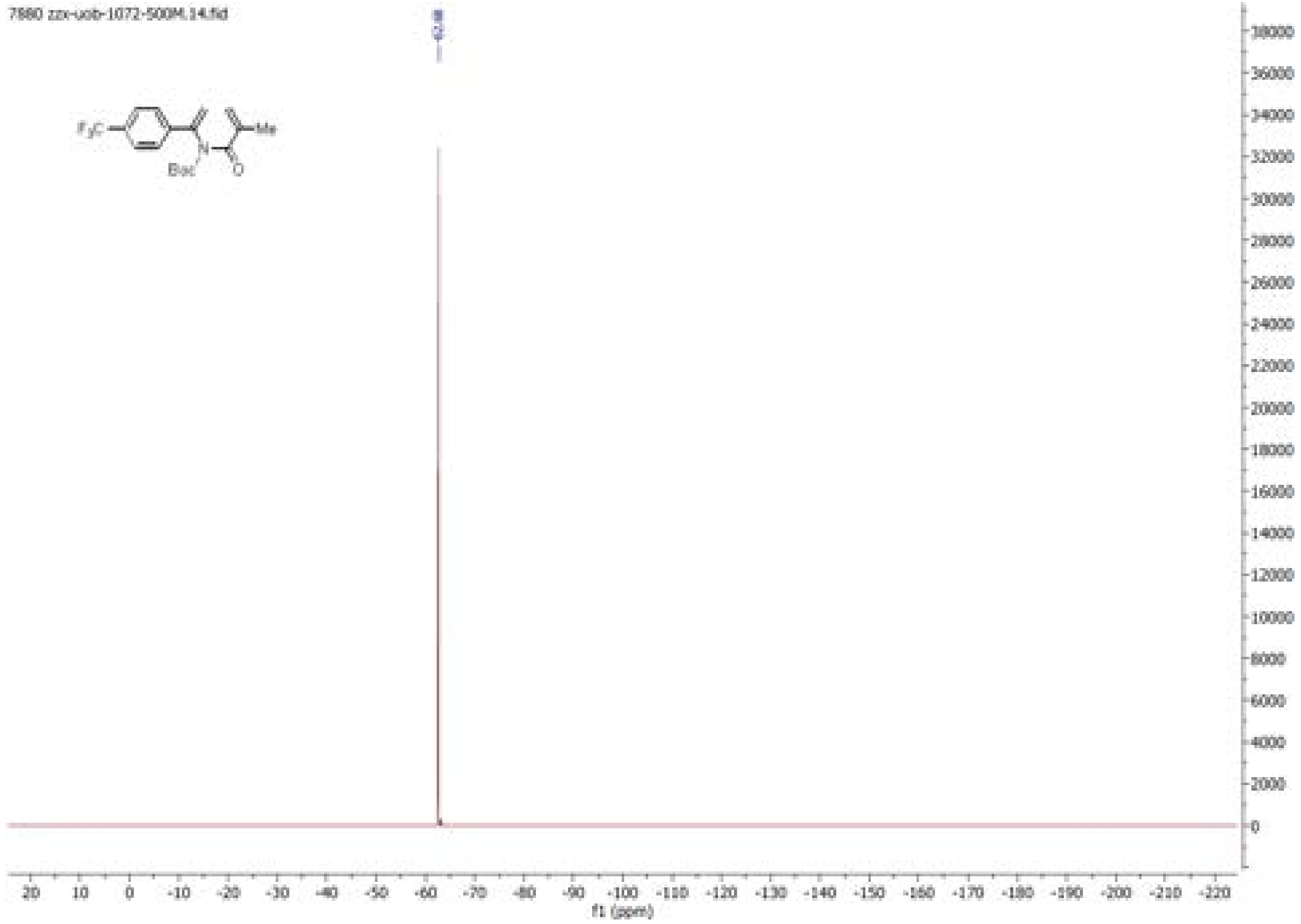

^{1}H NMR (500 MHz, $CDCl_3$) of **2b**

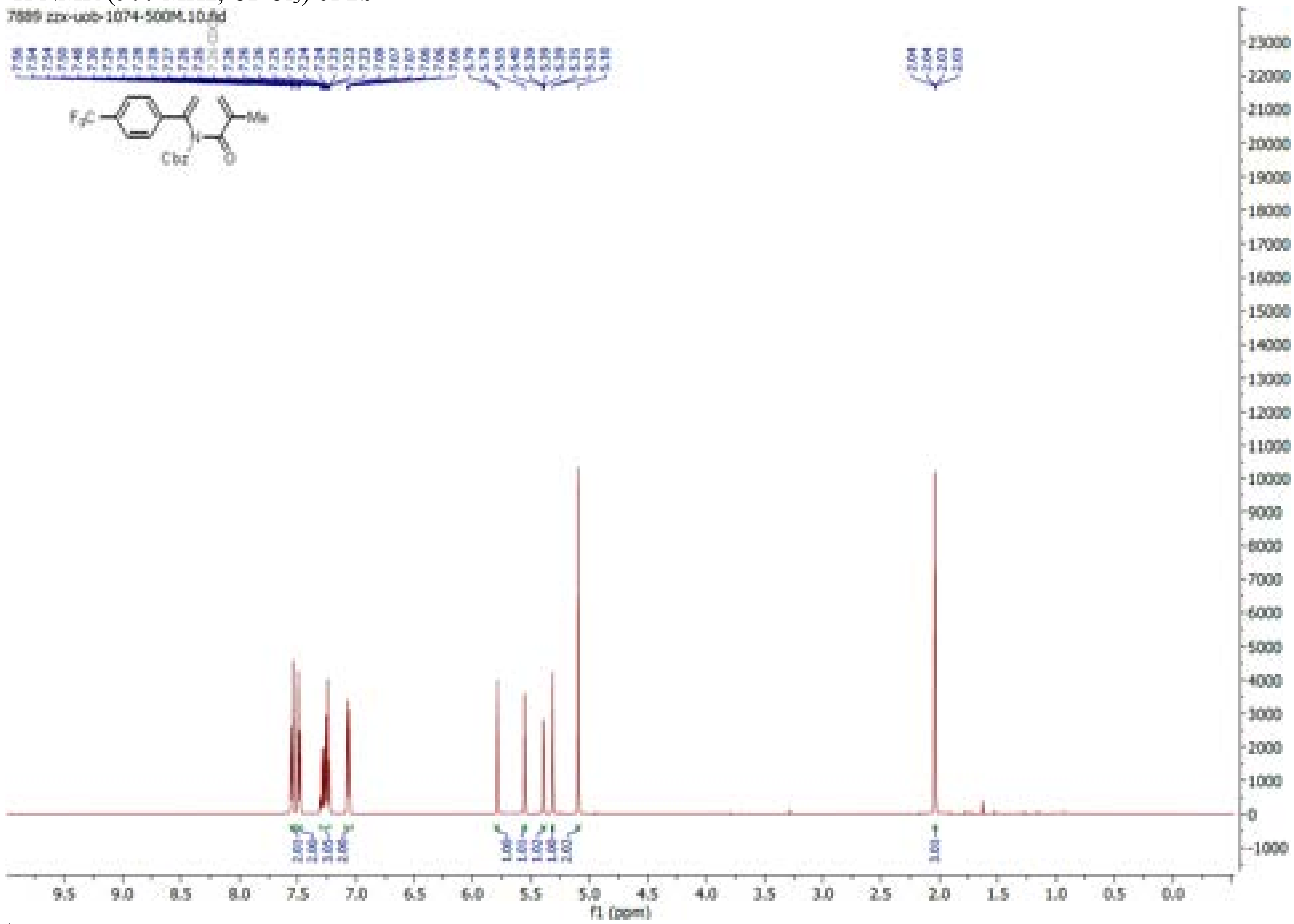

1

^{13}C NMR (126 MHz, $CDCl_3$) of **2b**

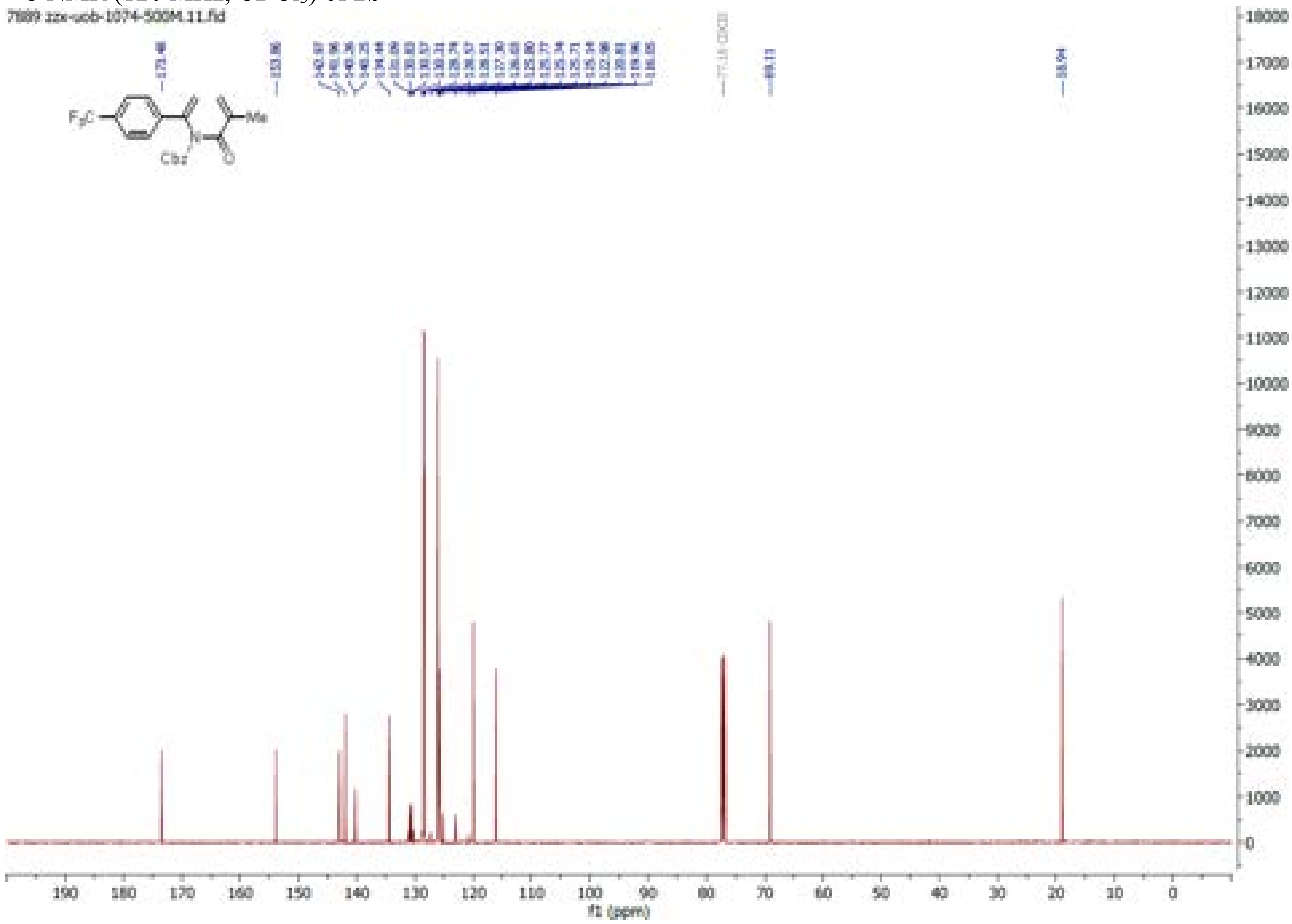

^{19}F NMR (471 MHz, $CDCl_3$) of **2b**

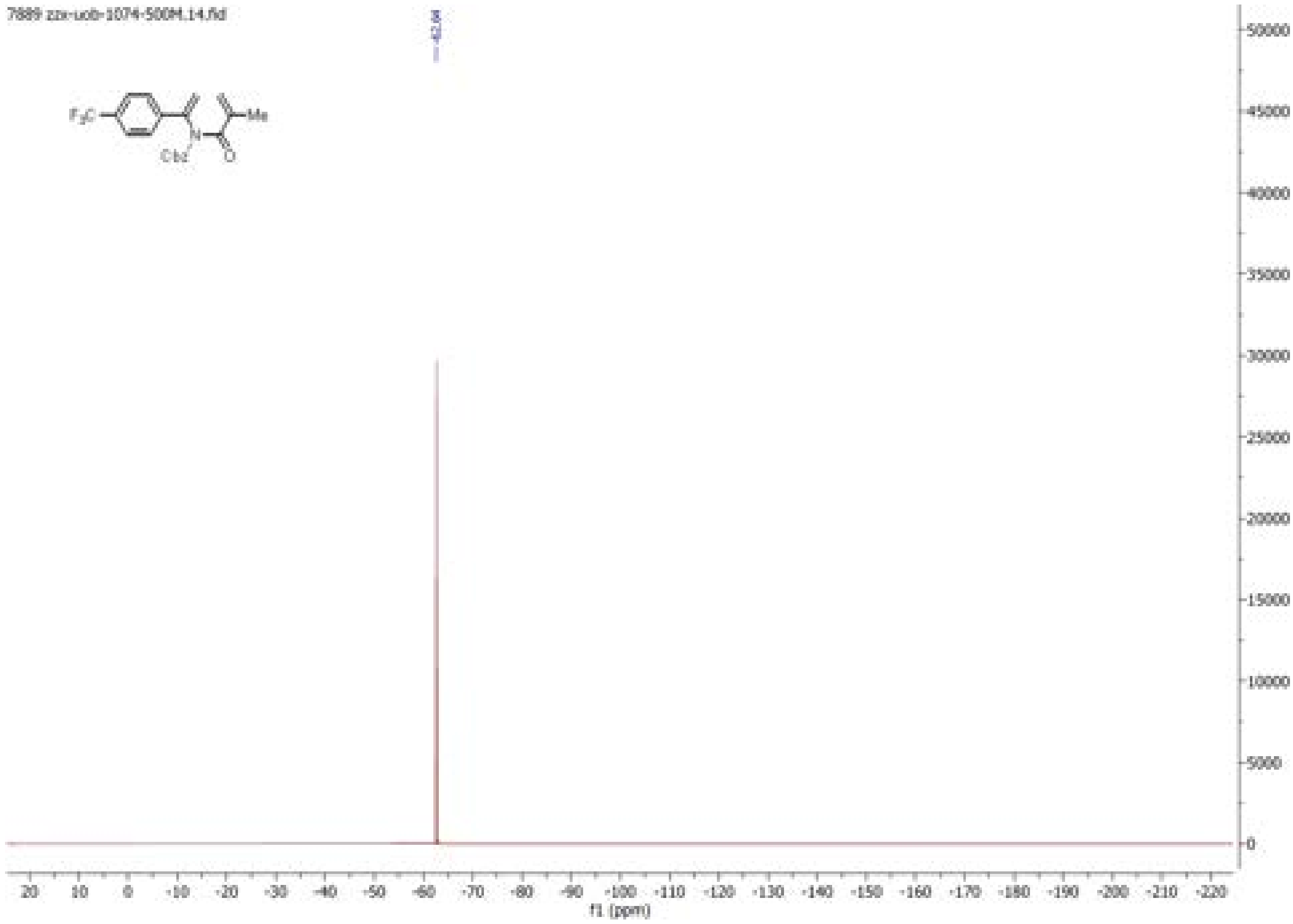

^{1}H NMR (500 MHz, $CDCl_3$) of **2c**

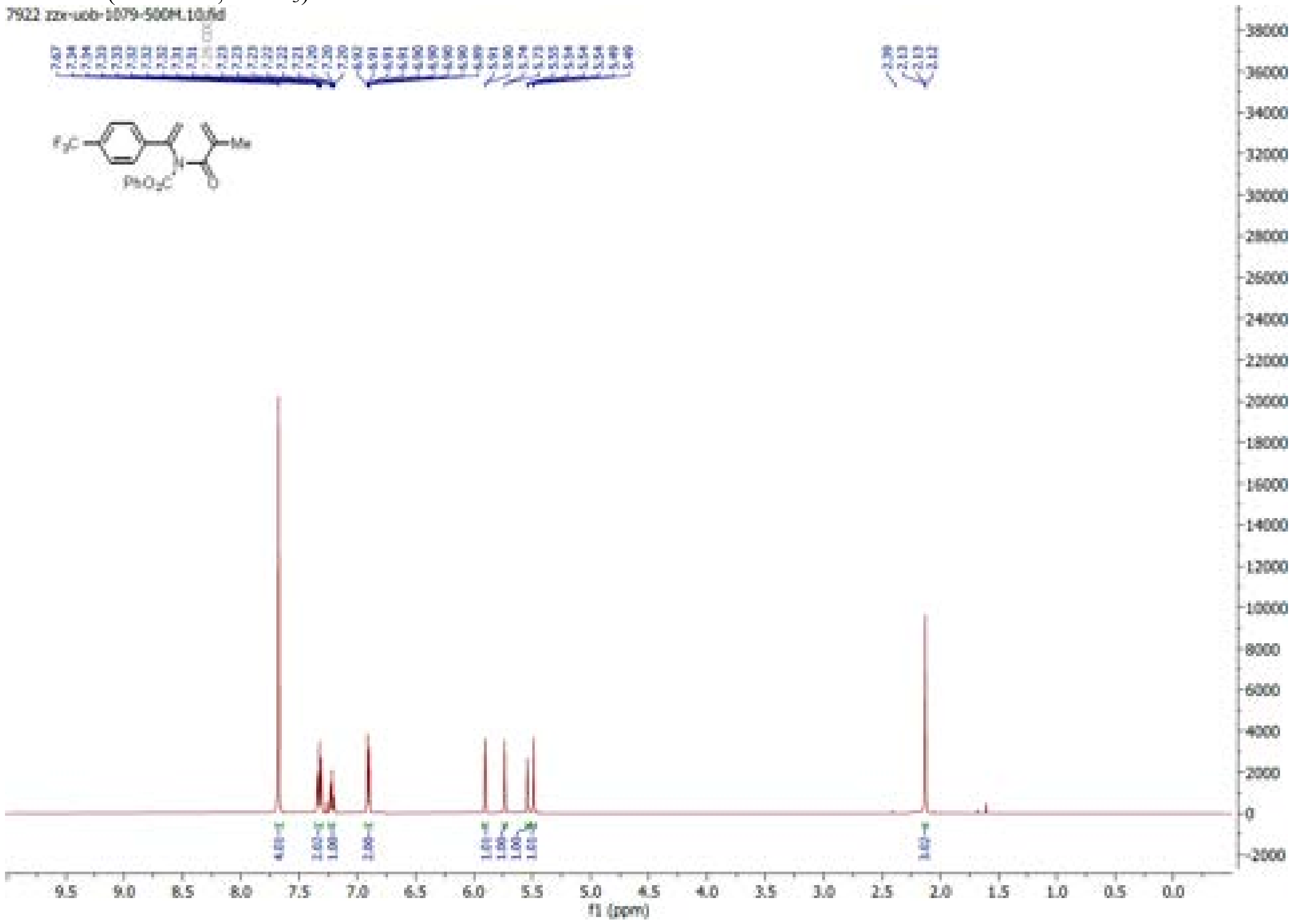

^{13}C NMR (126 MHz, $CDCl_3$) of **2c**

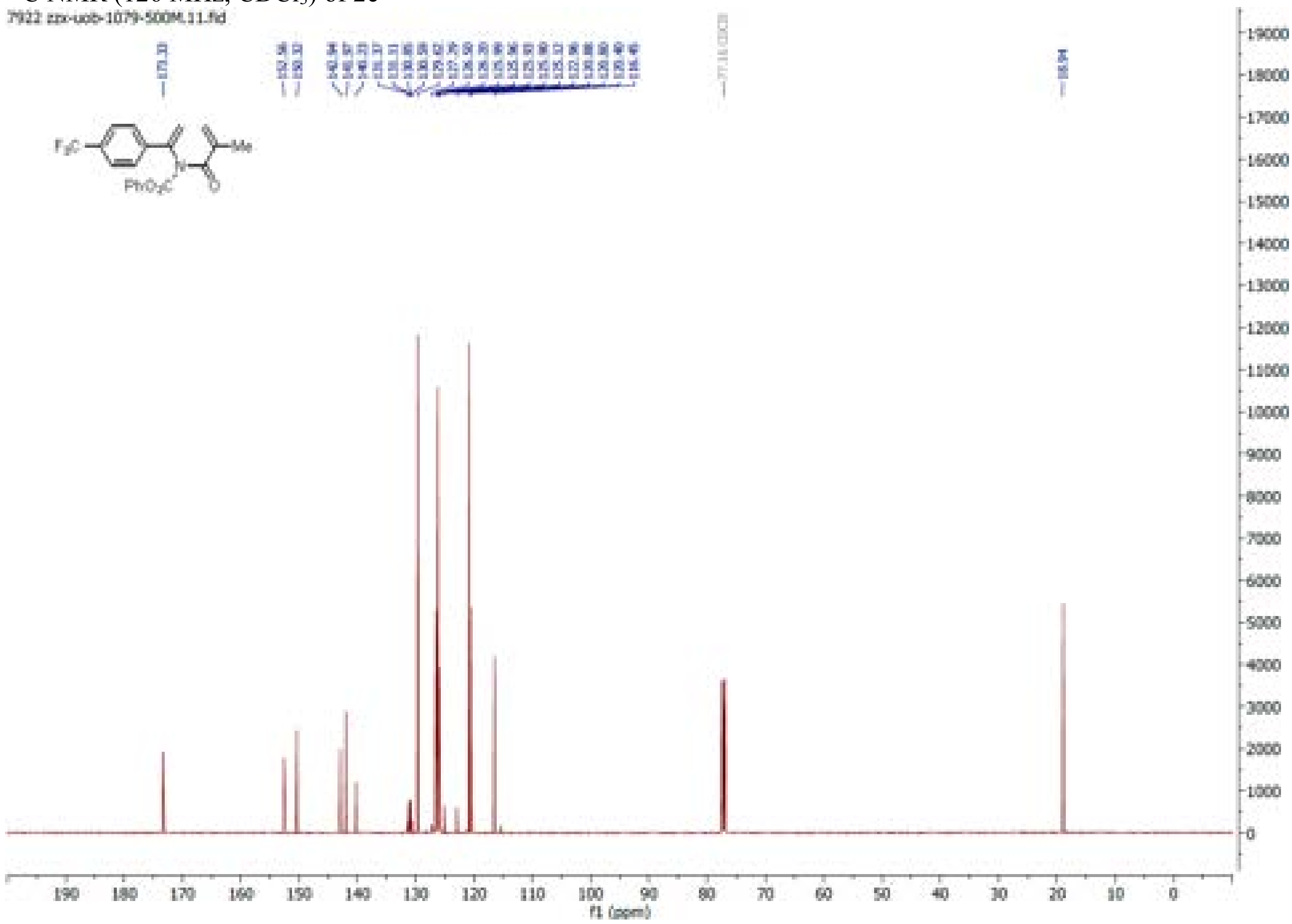

^{19}F NMR (471 MHz, $CDCl_3$) of **2c**

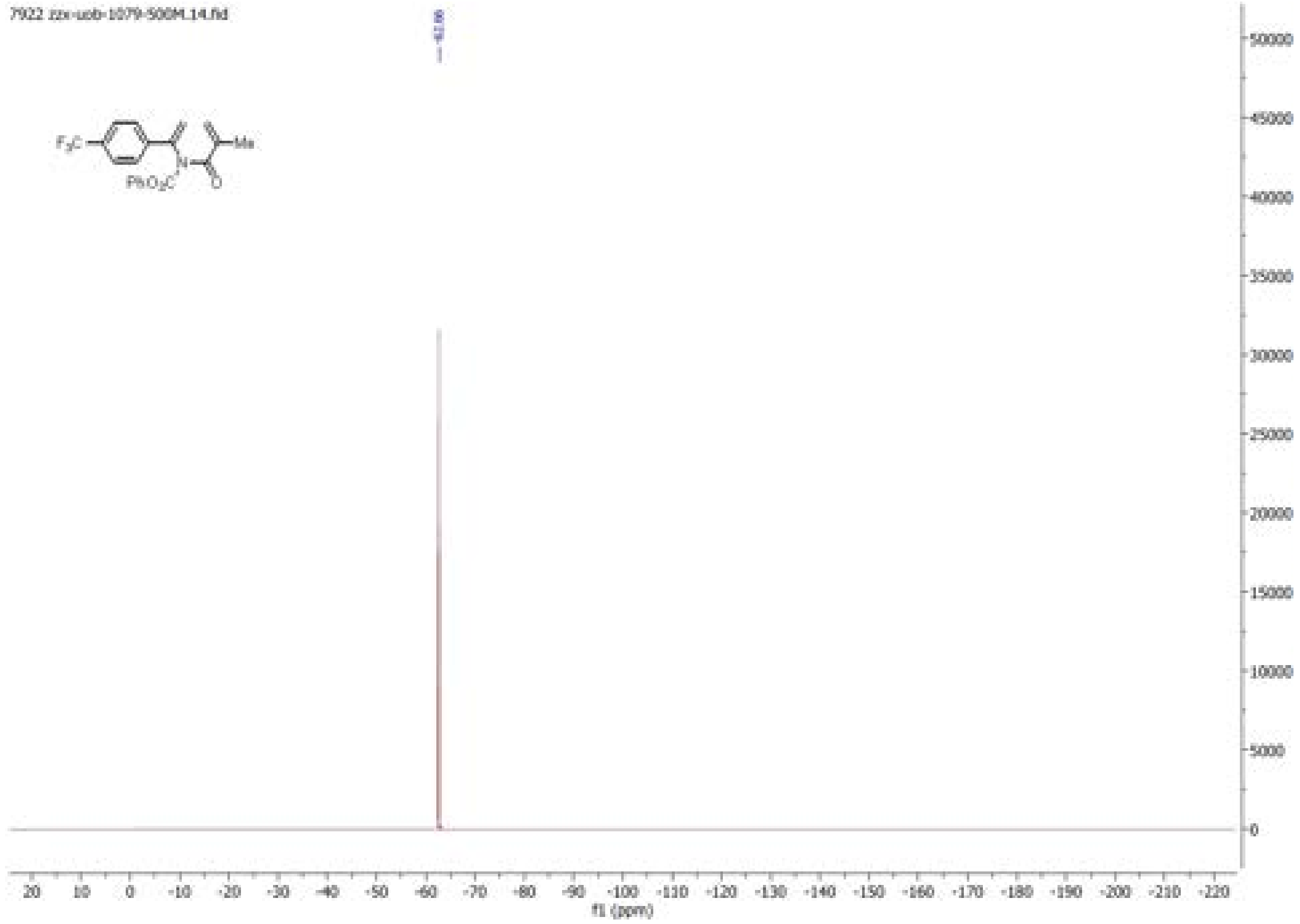

^{1}H NMR (500 MHz, $CDCl_3$) of **2d**

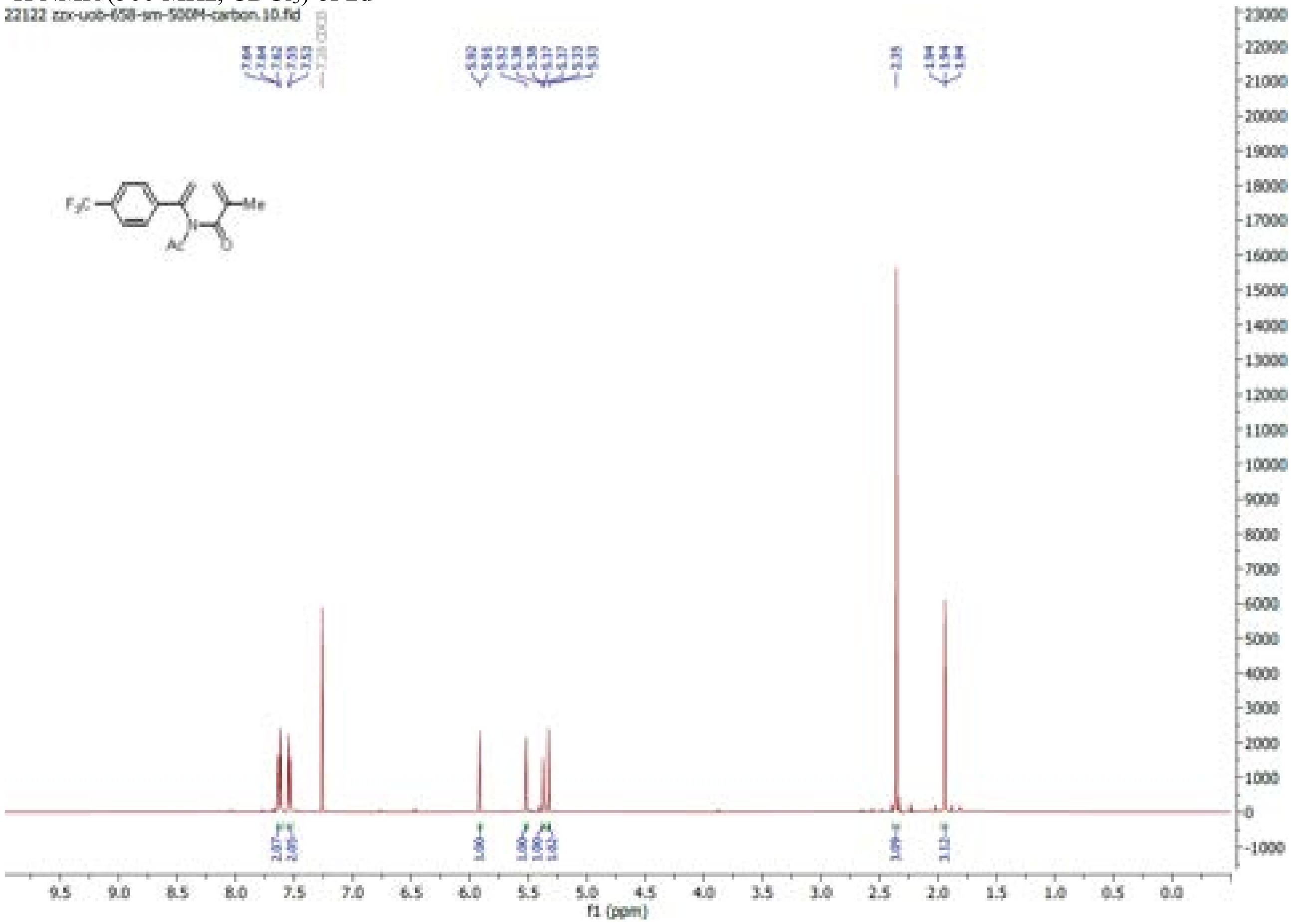

^{13}C NMR (126 MHz, $CDCl_3$) of **2d**

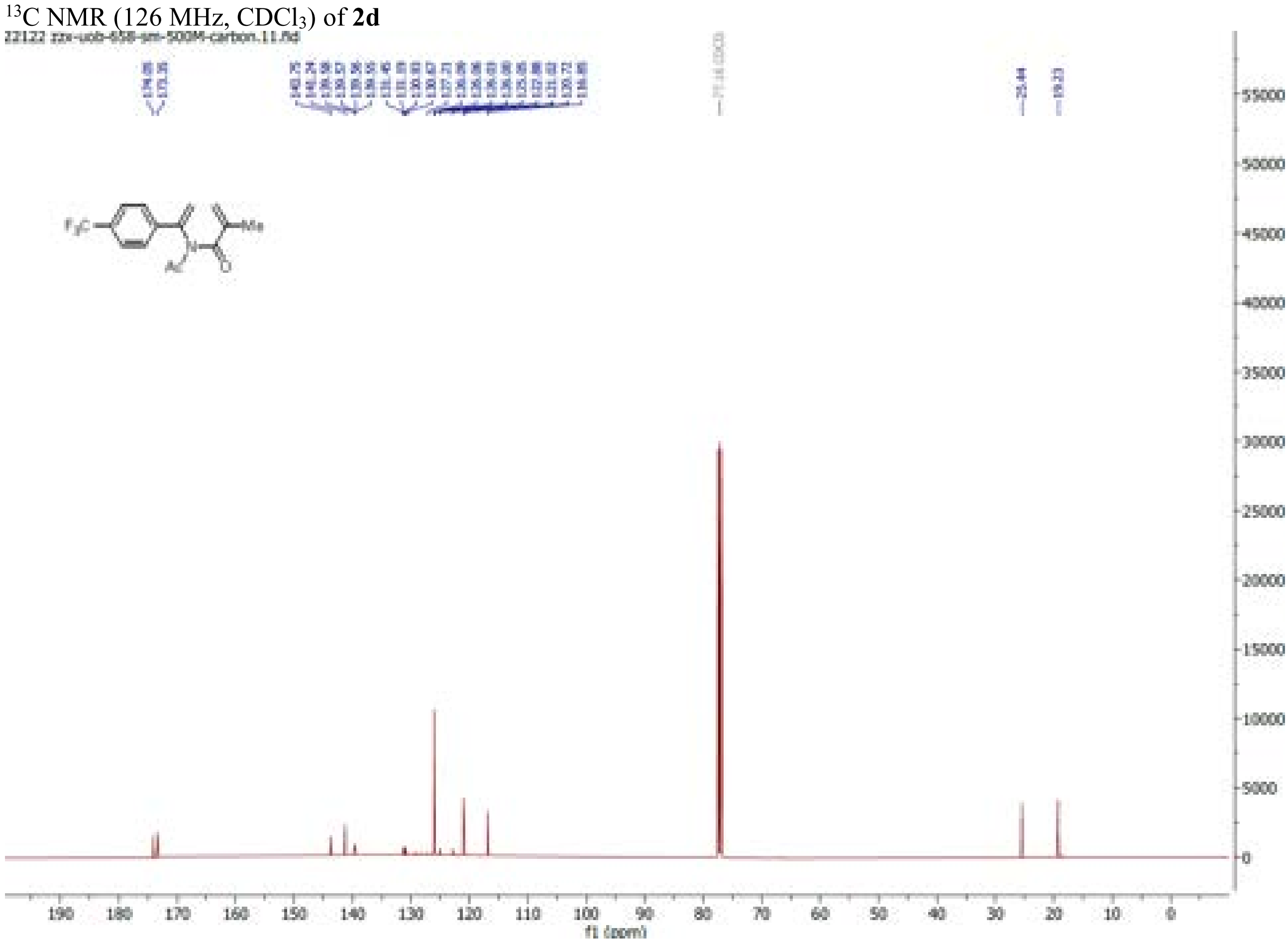

^{19}F NMR (471 MHz, $CDCl_3$) of **2d**

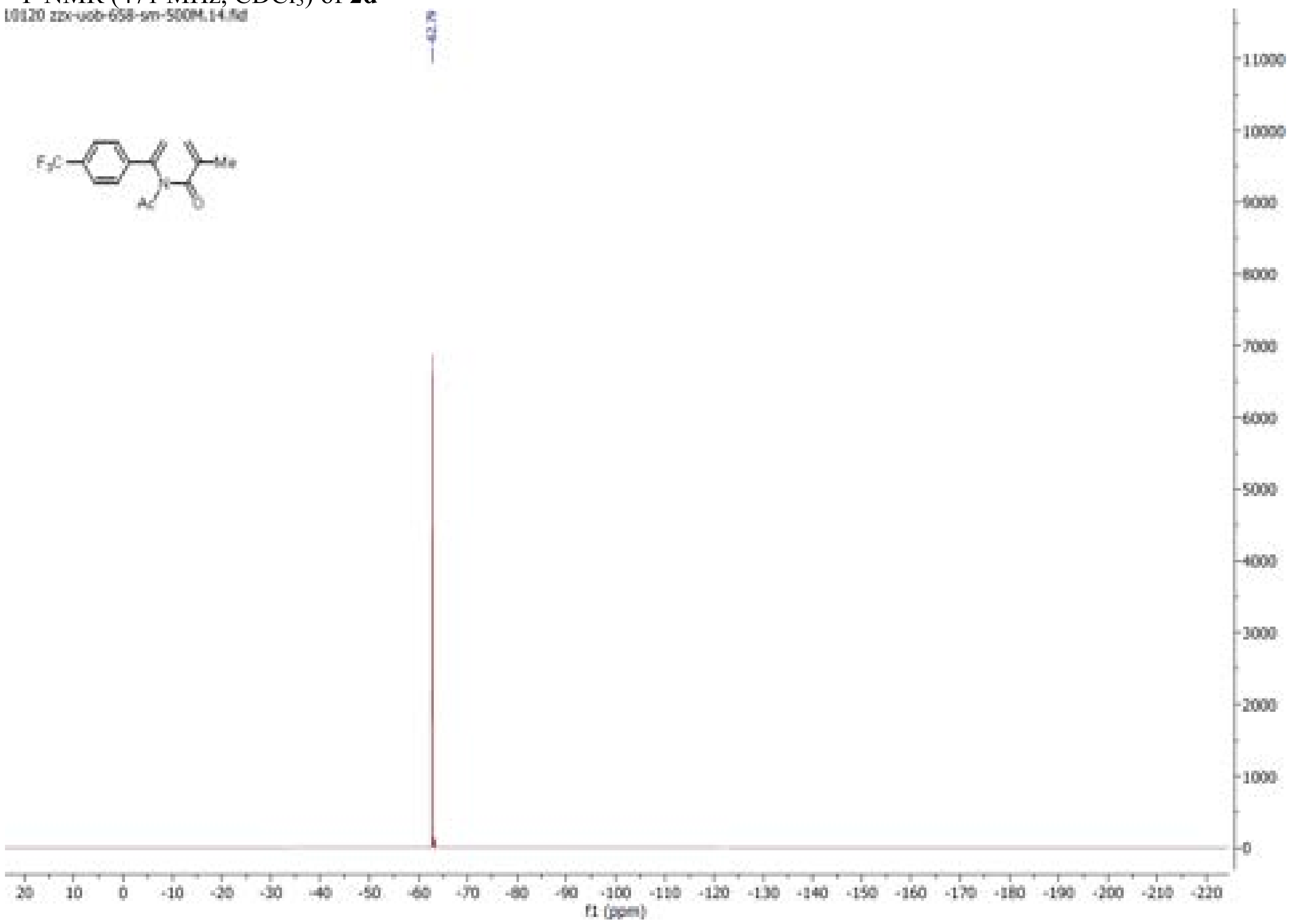

^{1}H NMR (500 MHz, $CDCl_3$) of **2e**

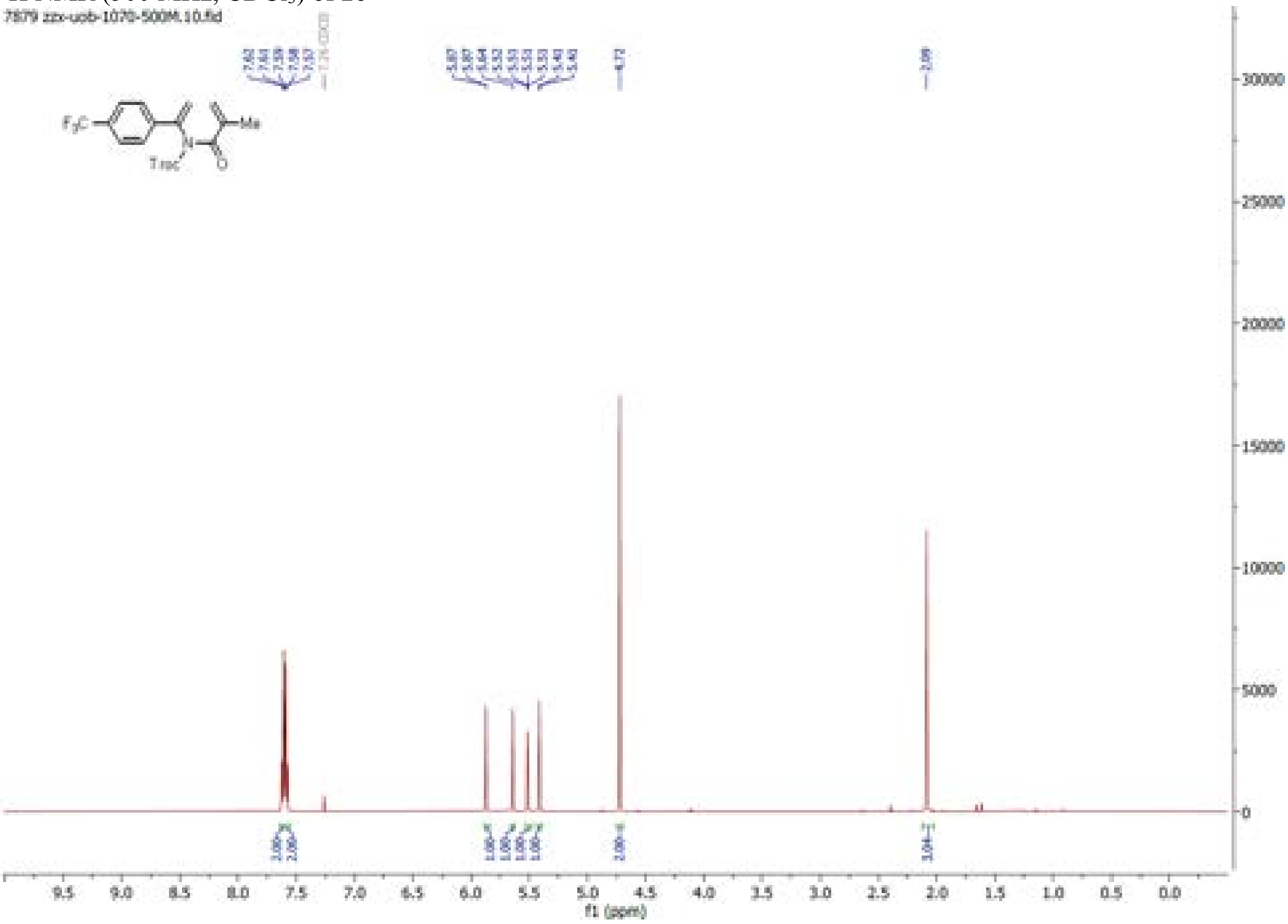

^{13}C NMR (126 MHz, $CDCl_3$) of **2e**

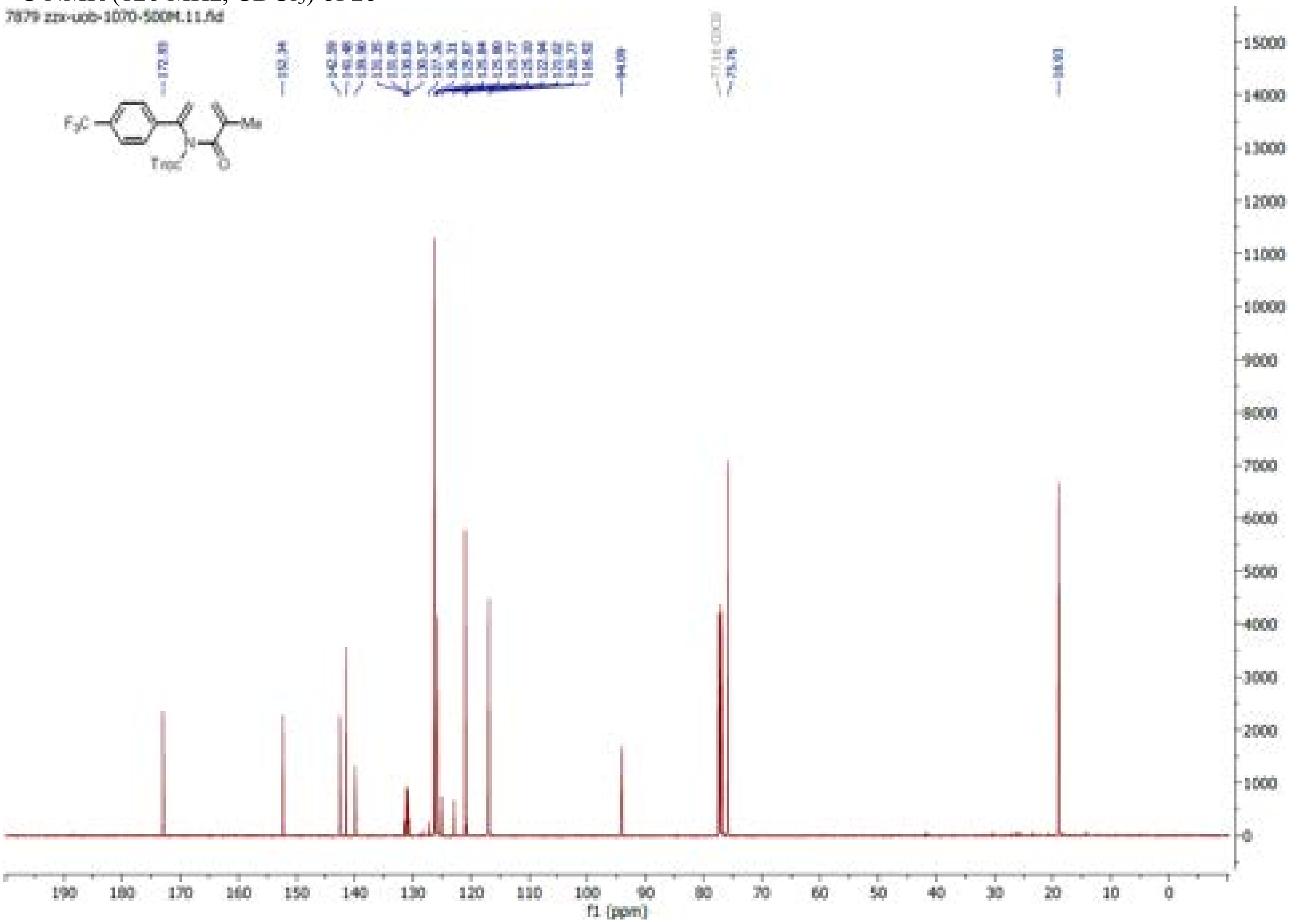

^{19}F NMR (471 MHz, $CDCl_3$) of **2e**

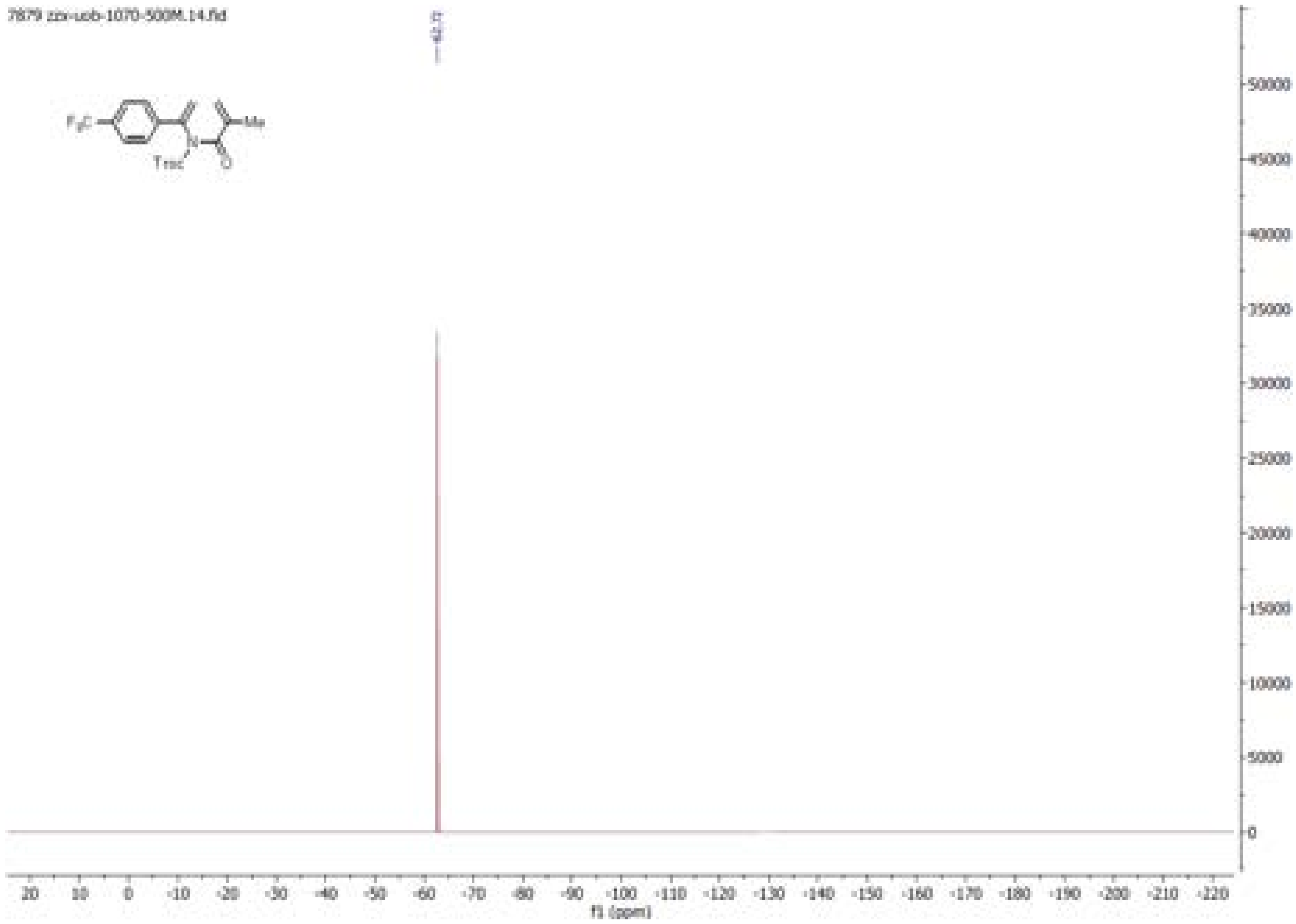

^{1}H NMR (500 MHz, $CDCl_3$) of **2f**

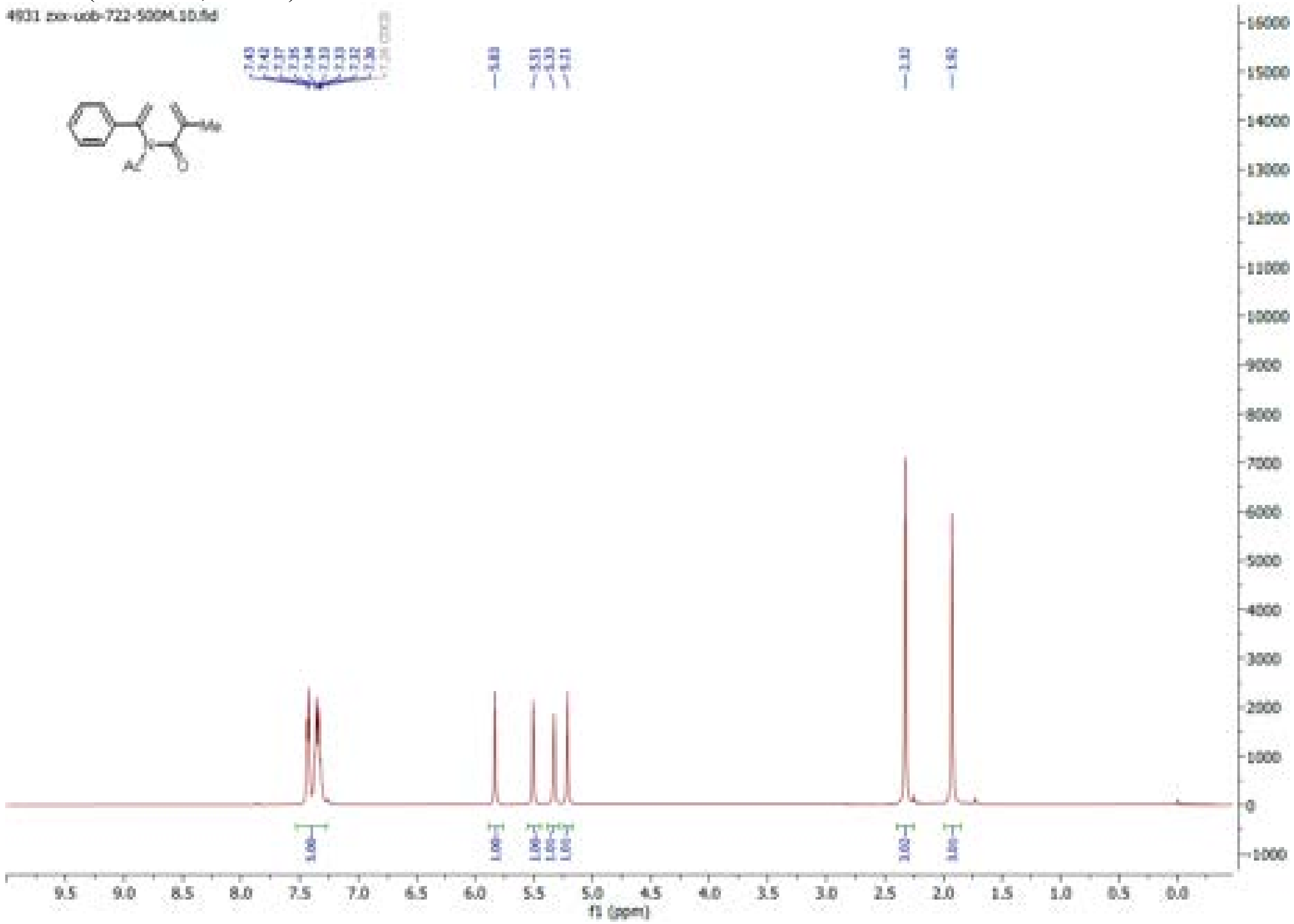

^{13}C NMR (126 MHz, $CDCl_3$) of **2f**

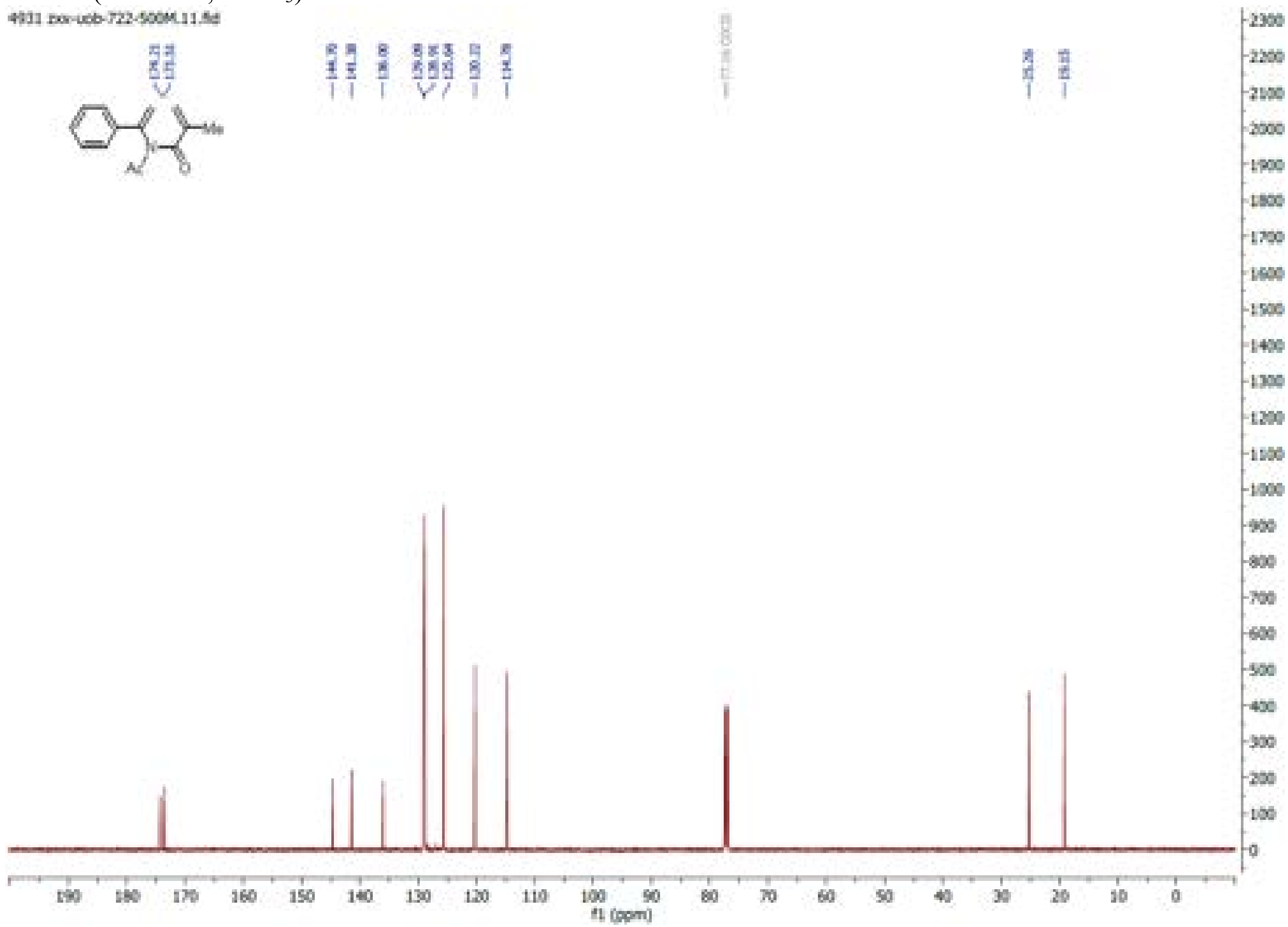

^{1}H NMR (500 MHz, $CDCl_3$) of **2g**

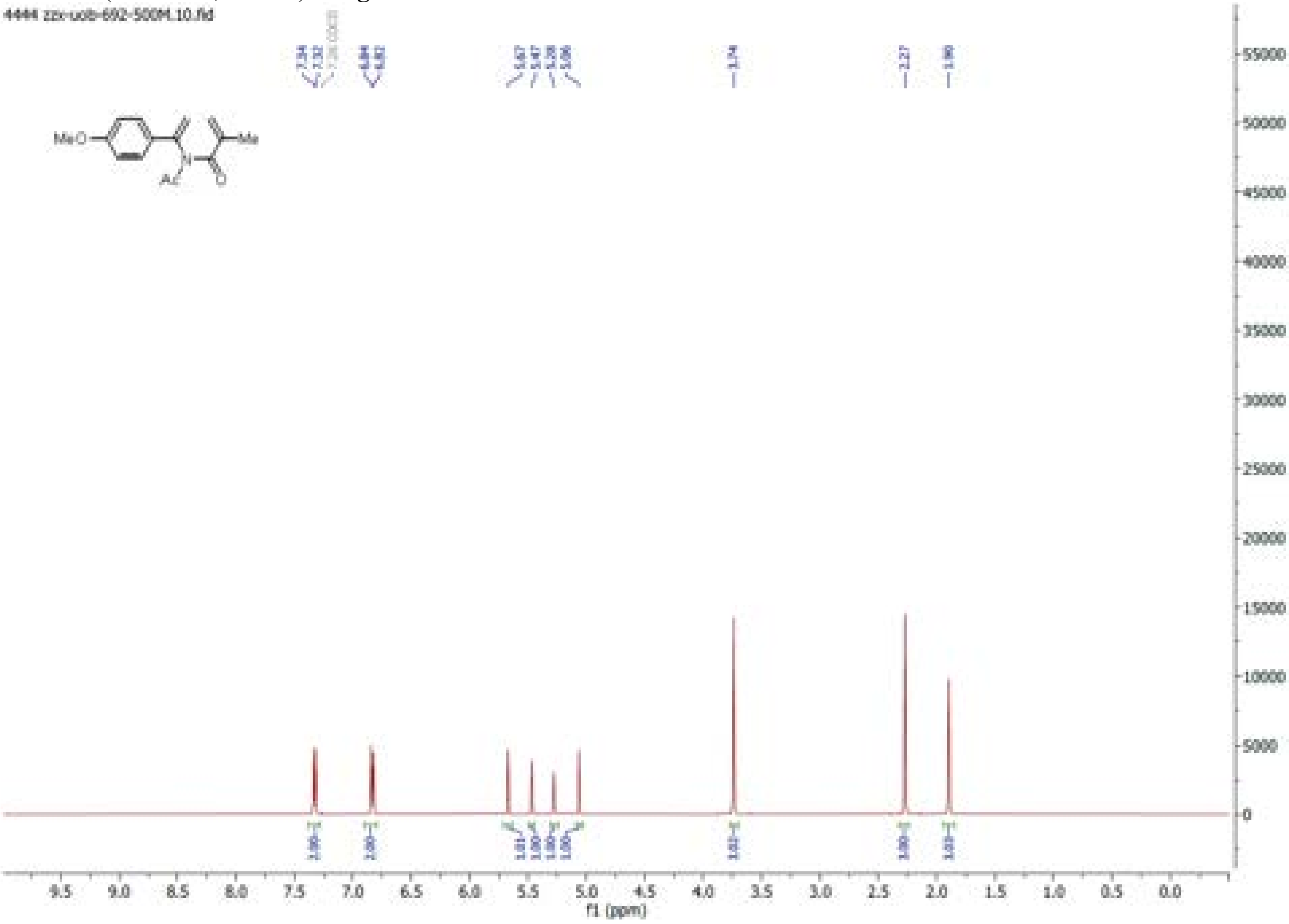

^{13}C NMR (126 MHz, $CDCl_3$) of **2g**

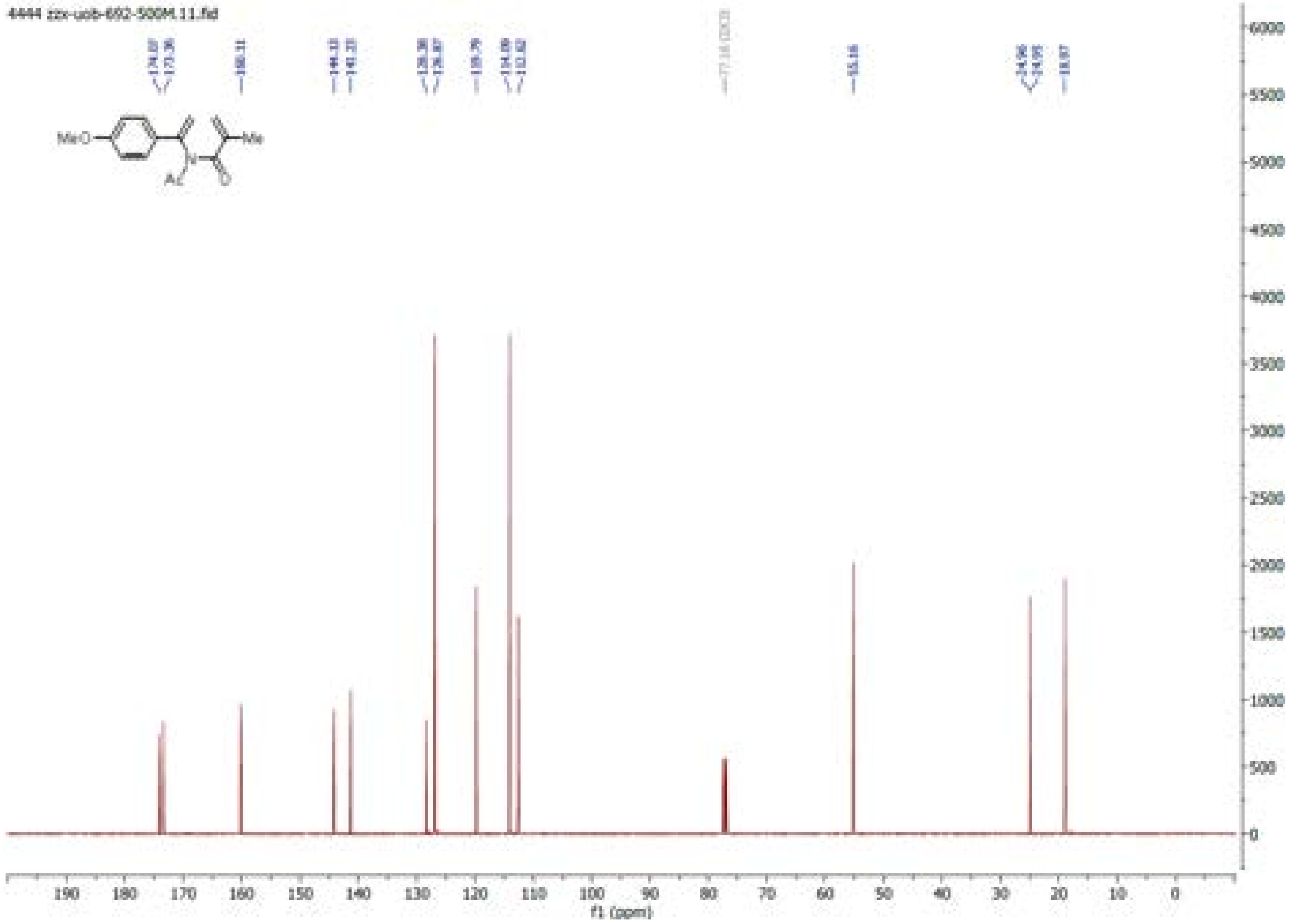

^{1}H NMR (500 MHz, $CDCl_3$) of **2h**

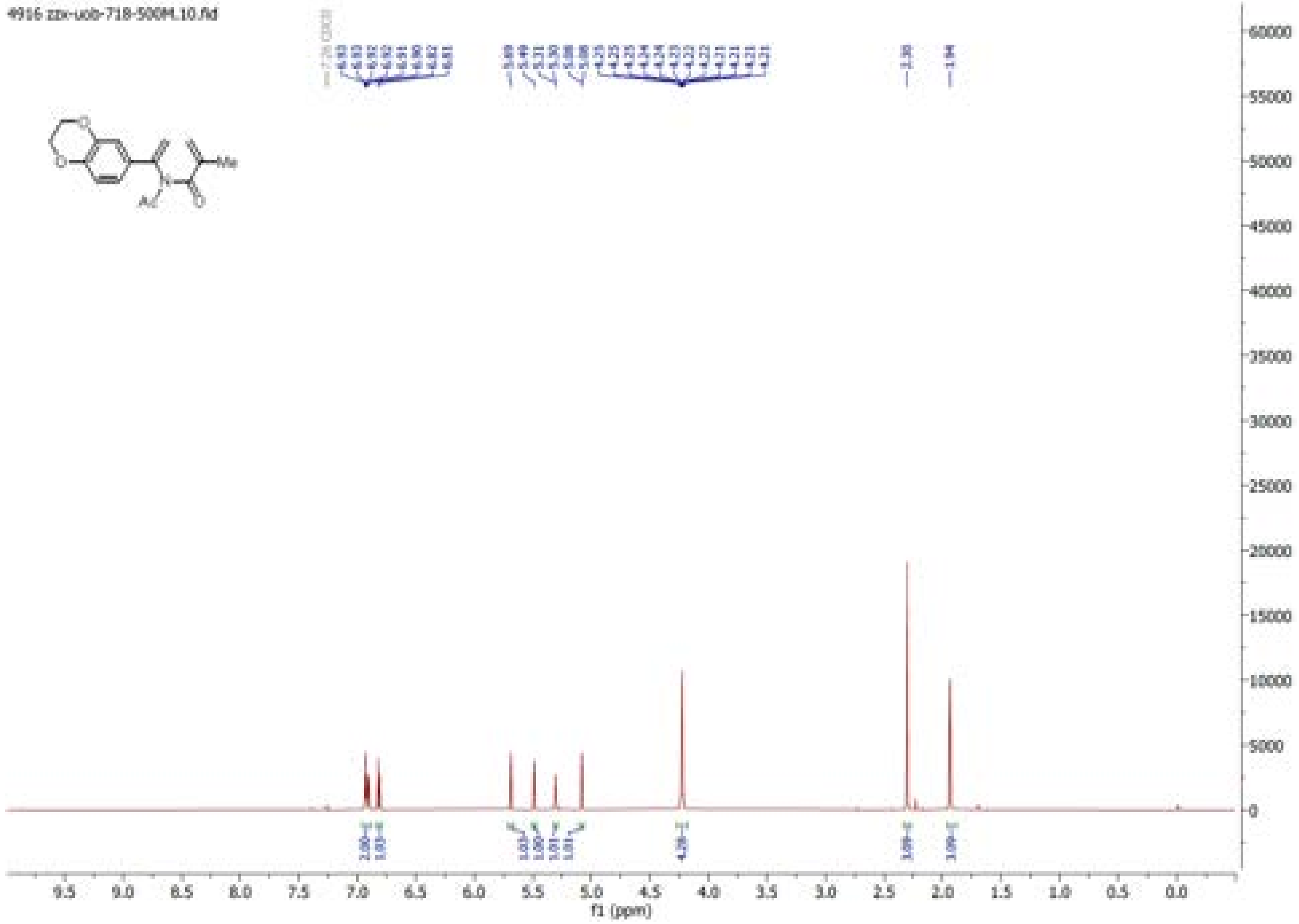

^{13}C NMR (126 MHz, $CDCl_3$) of **2h**

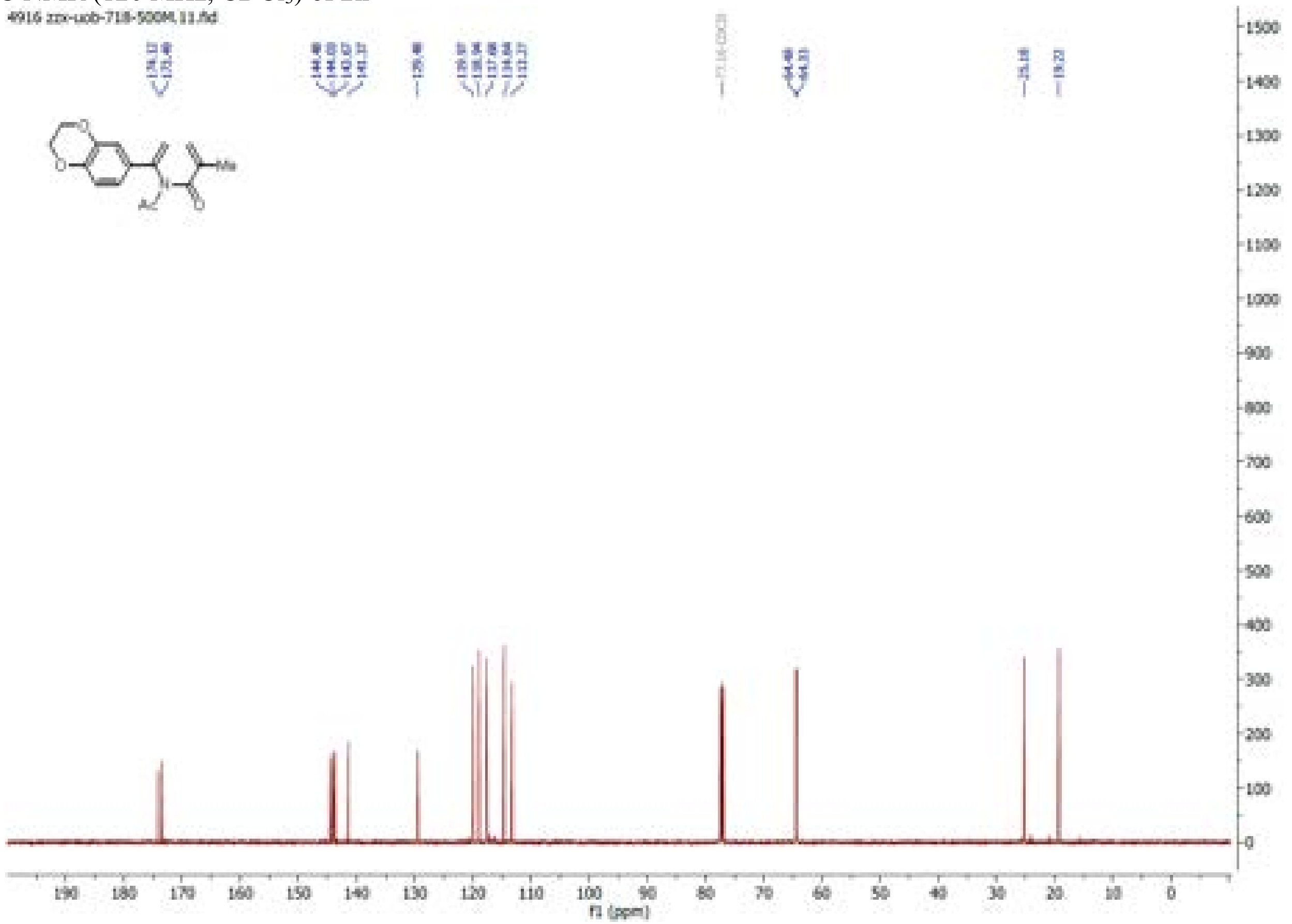

^{1}H NMR (500 MHz, $CDCl_3$) of **2i**

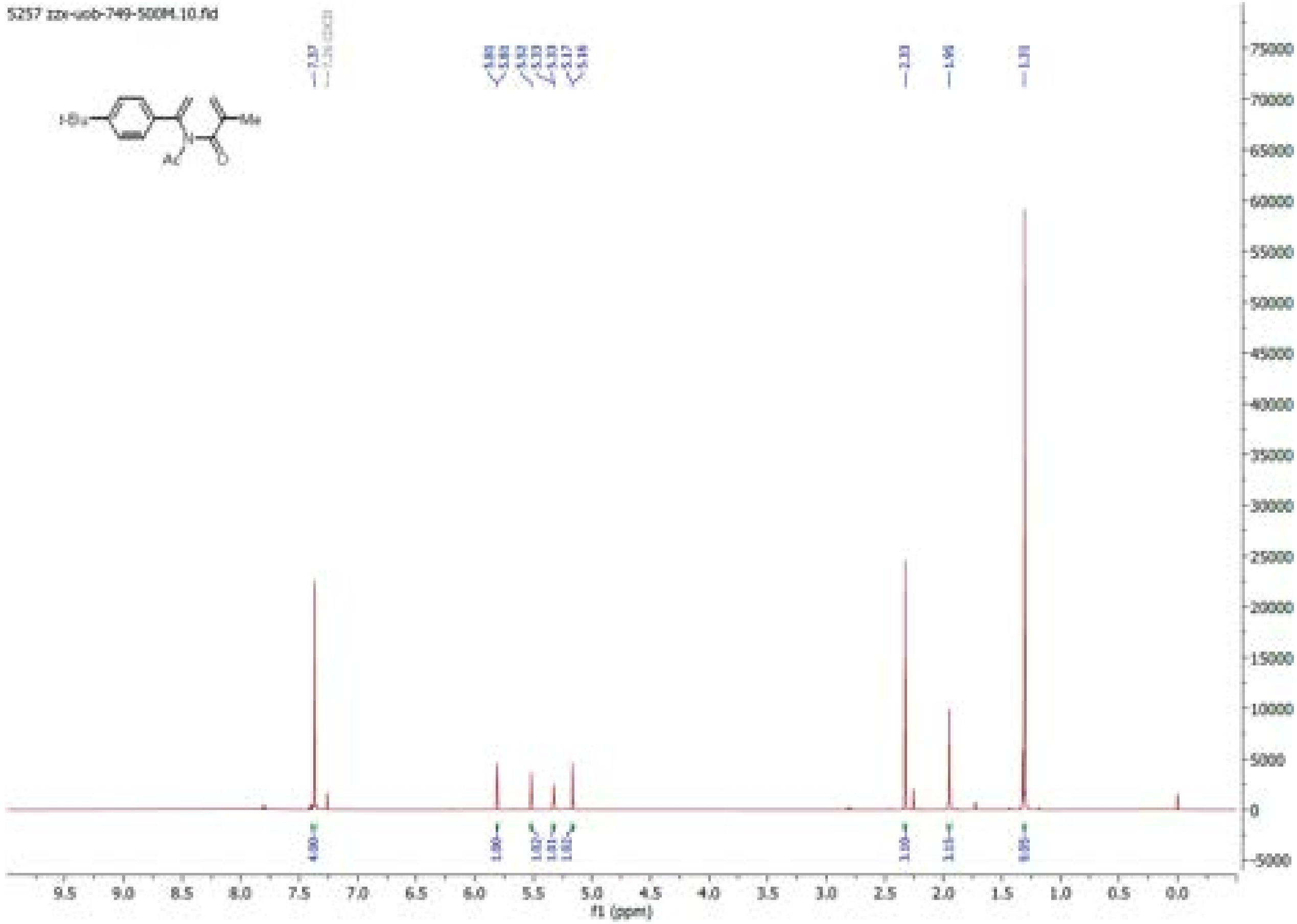

^{13}C NMR (126 MHz, $CDCl_3$) of **2i**

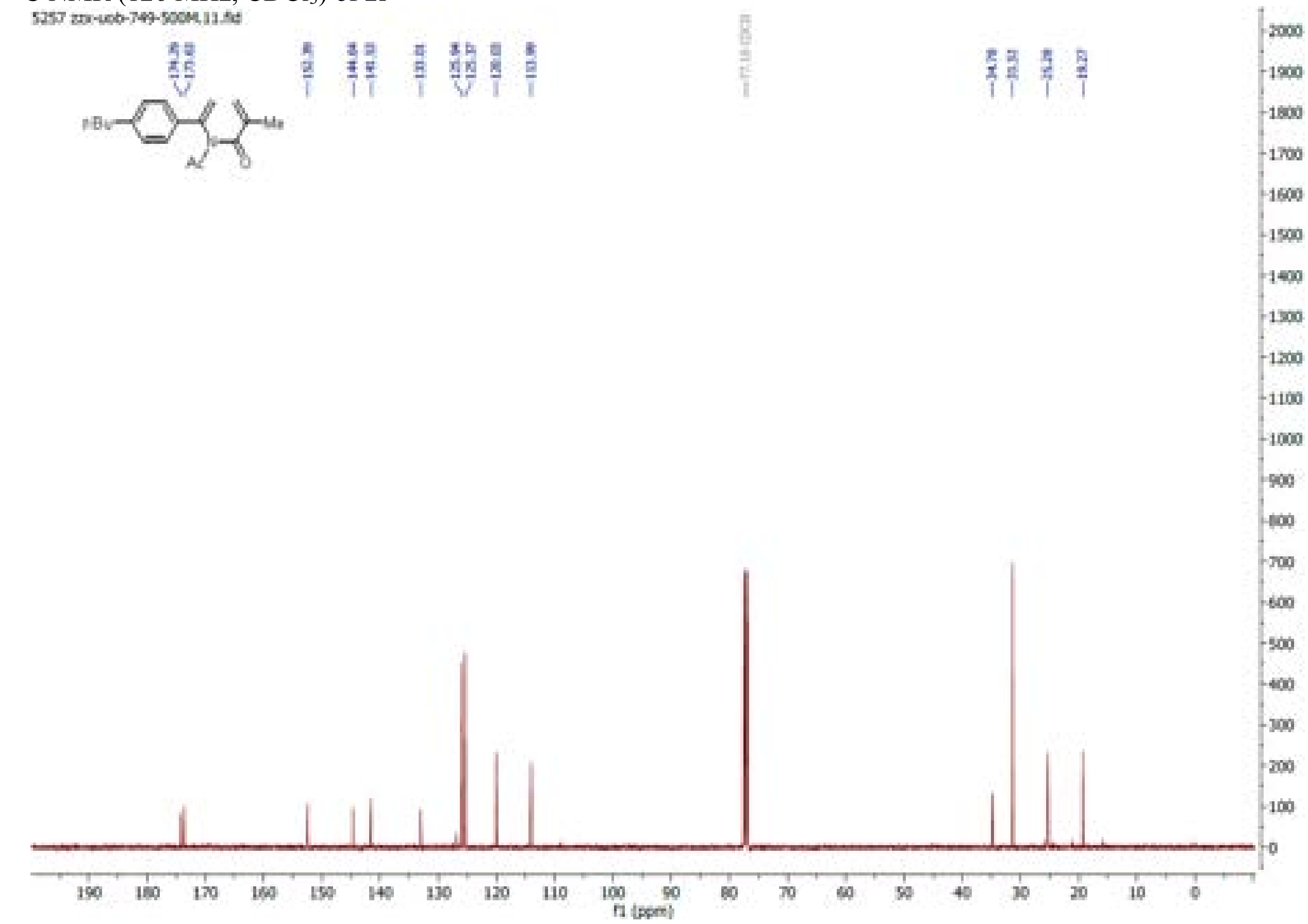

^{1}H NMR (500 MHz, $CDCl_3$) of **2j**

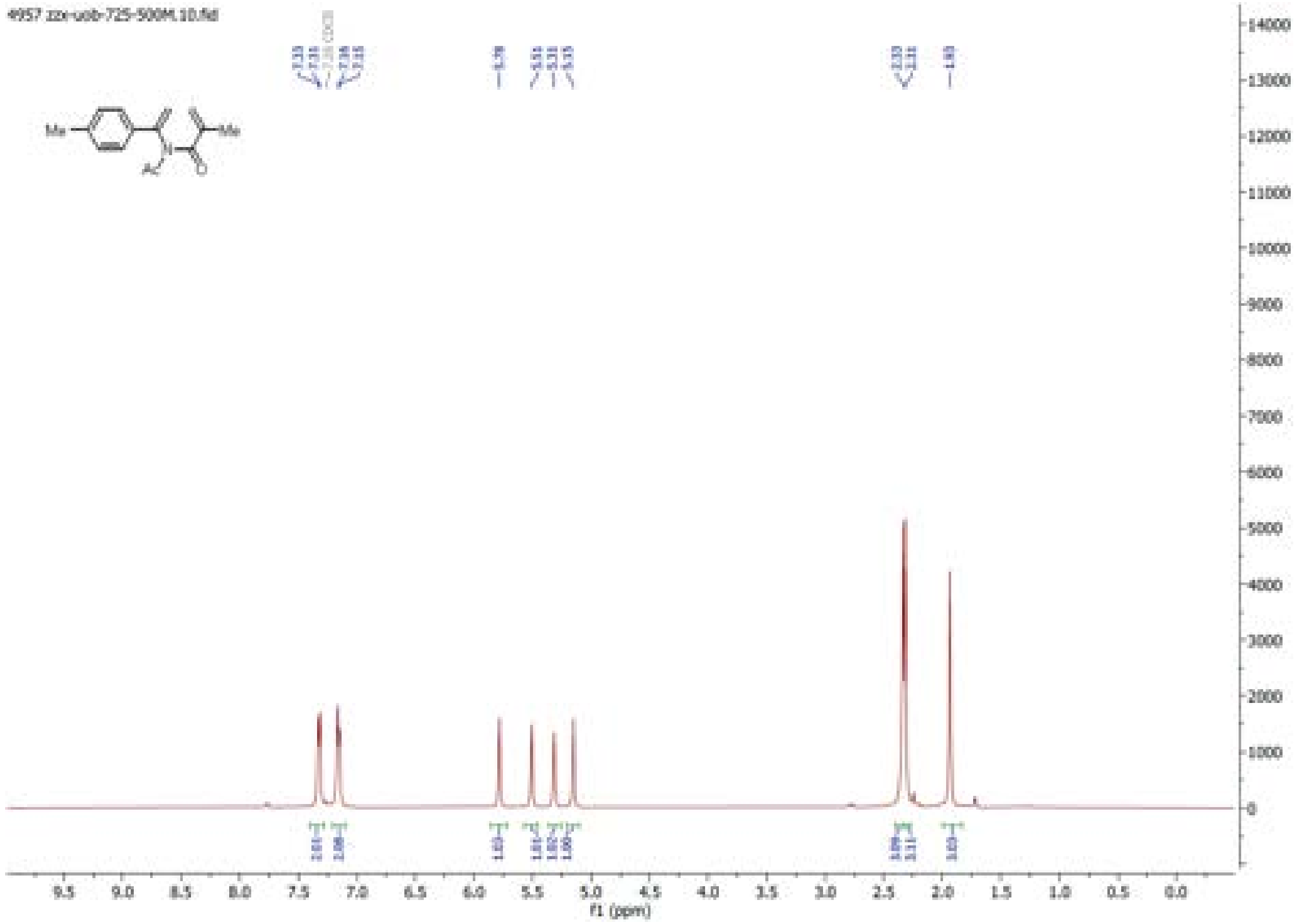

^{13}C NMR (126 MHz, $CDCl_3$) of **2j**

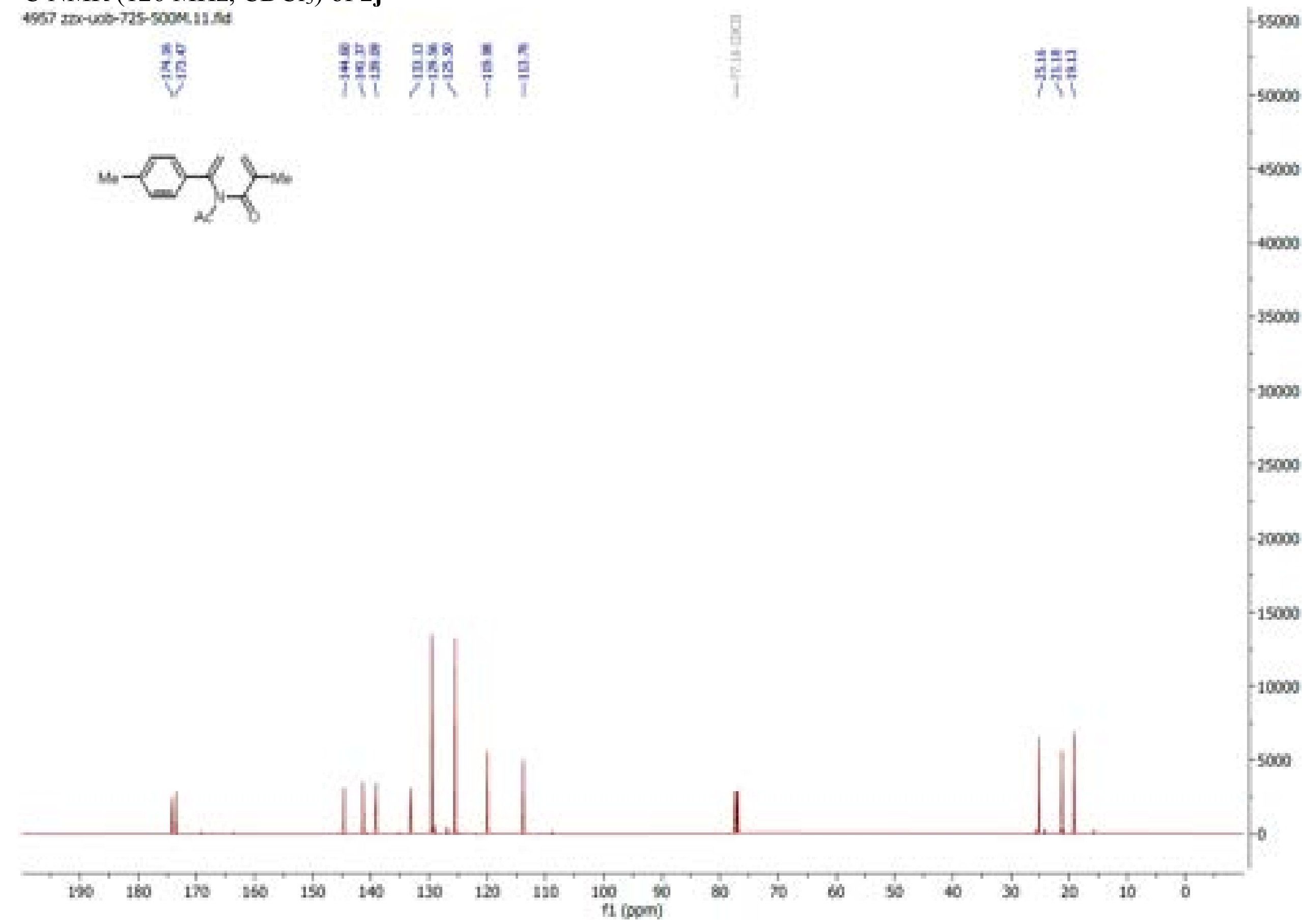

^{1}H NMR (500 MHz, $CDCl_3$) of **2k**

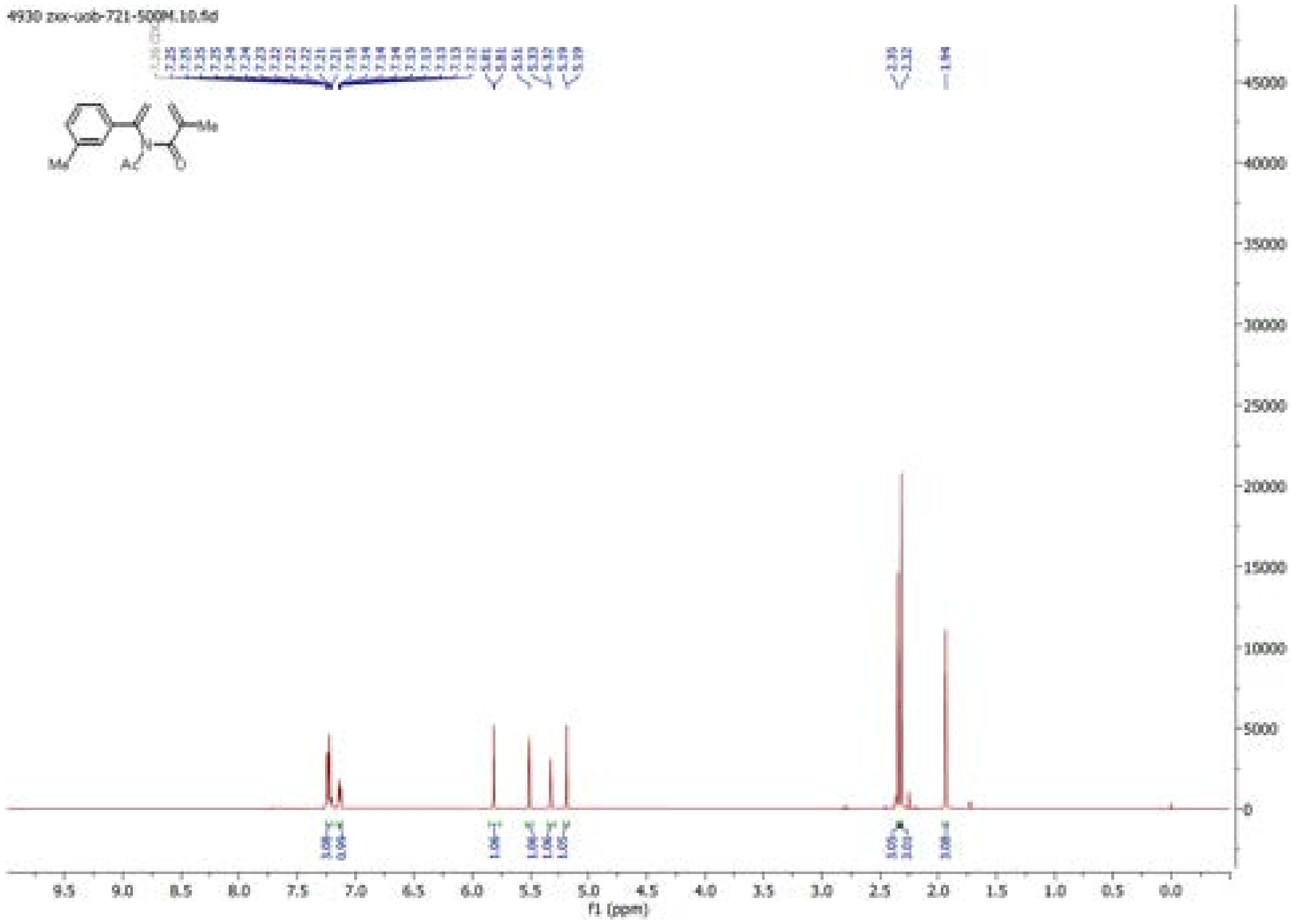

^{13}C NMR (126 MHz, $CDCl_3$) of **2k**

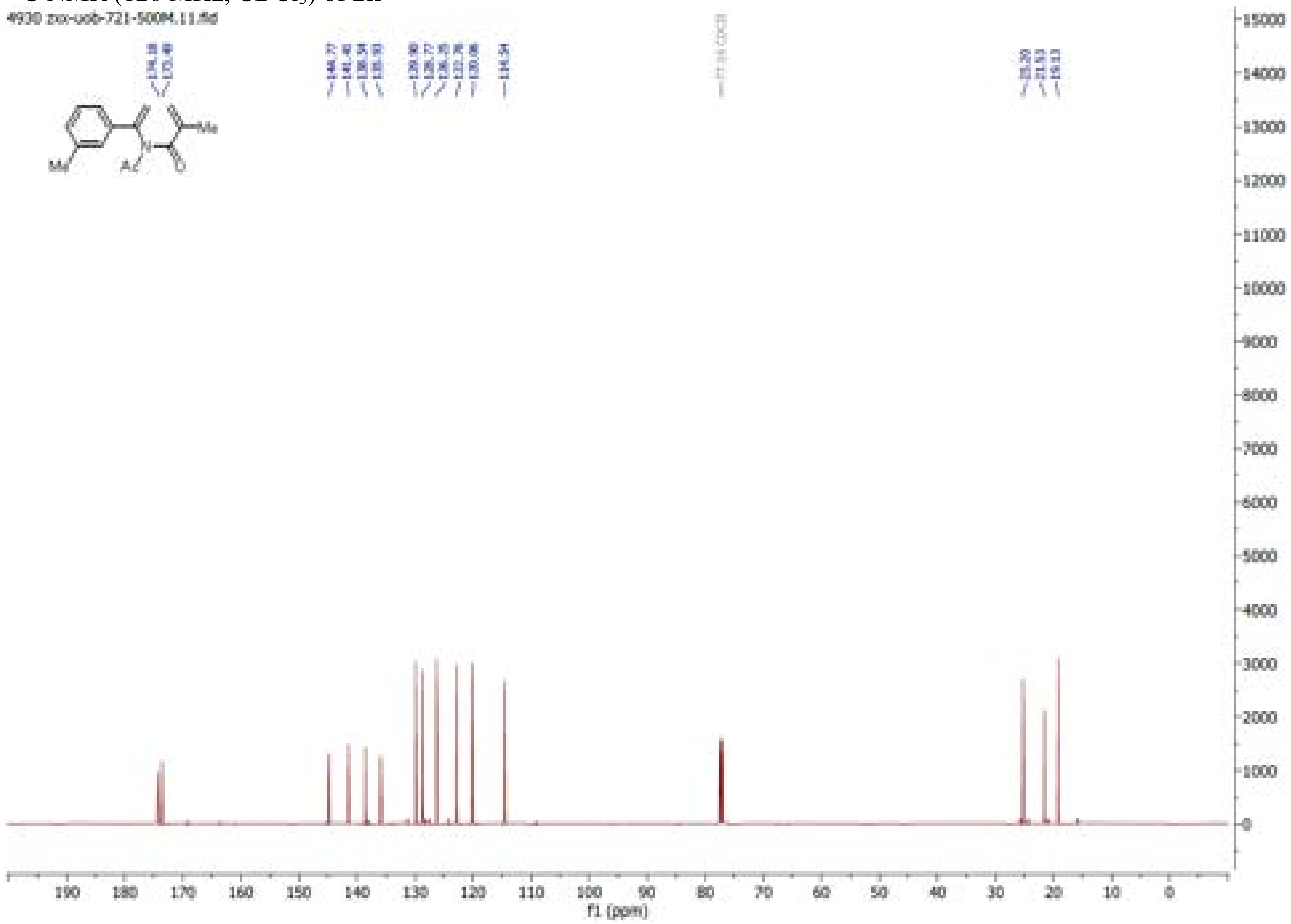

^{1}H NMR (500 MHz, $CDCl_3$) of **2l**

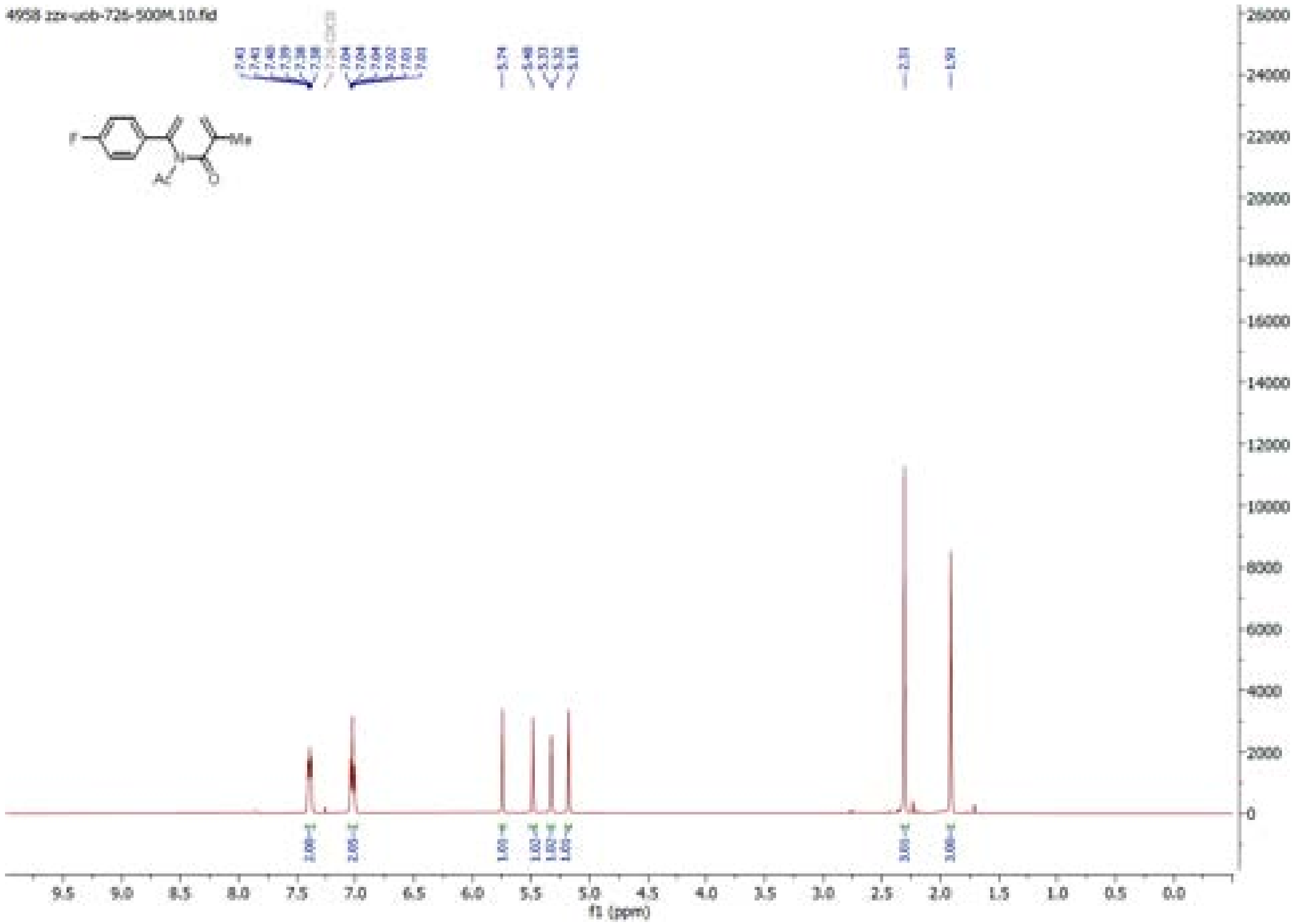

^{13}C NMR (126 MHz, $CDCl_3$) of **2l**

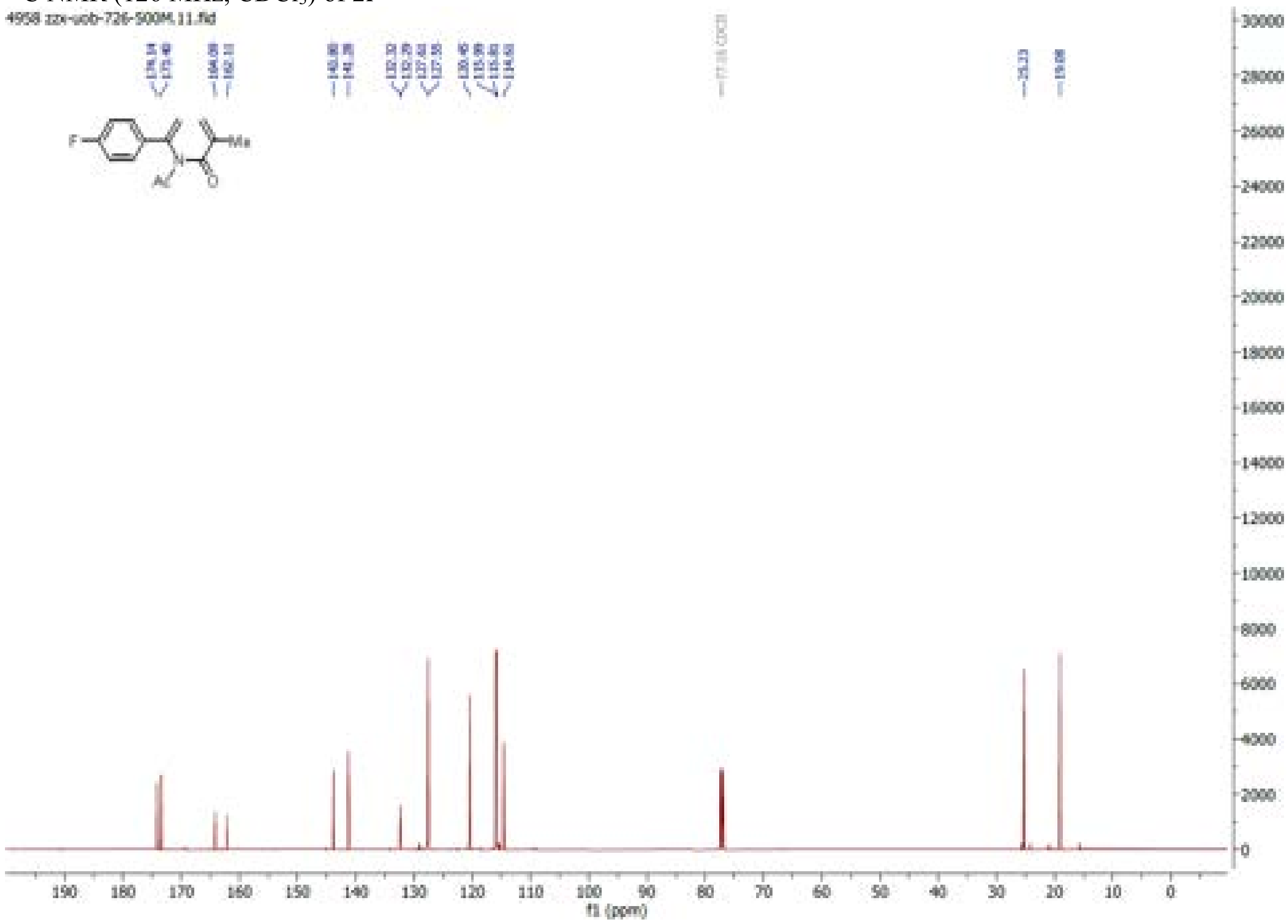

^{19}F NMR (471 MHz, $CDCl_3$) of **2l**

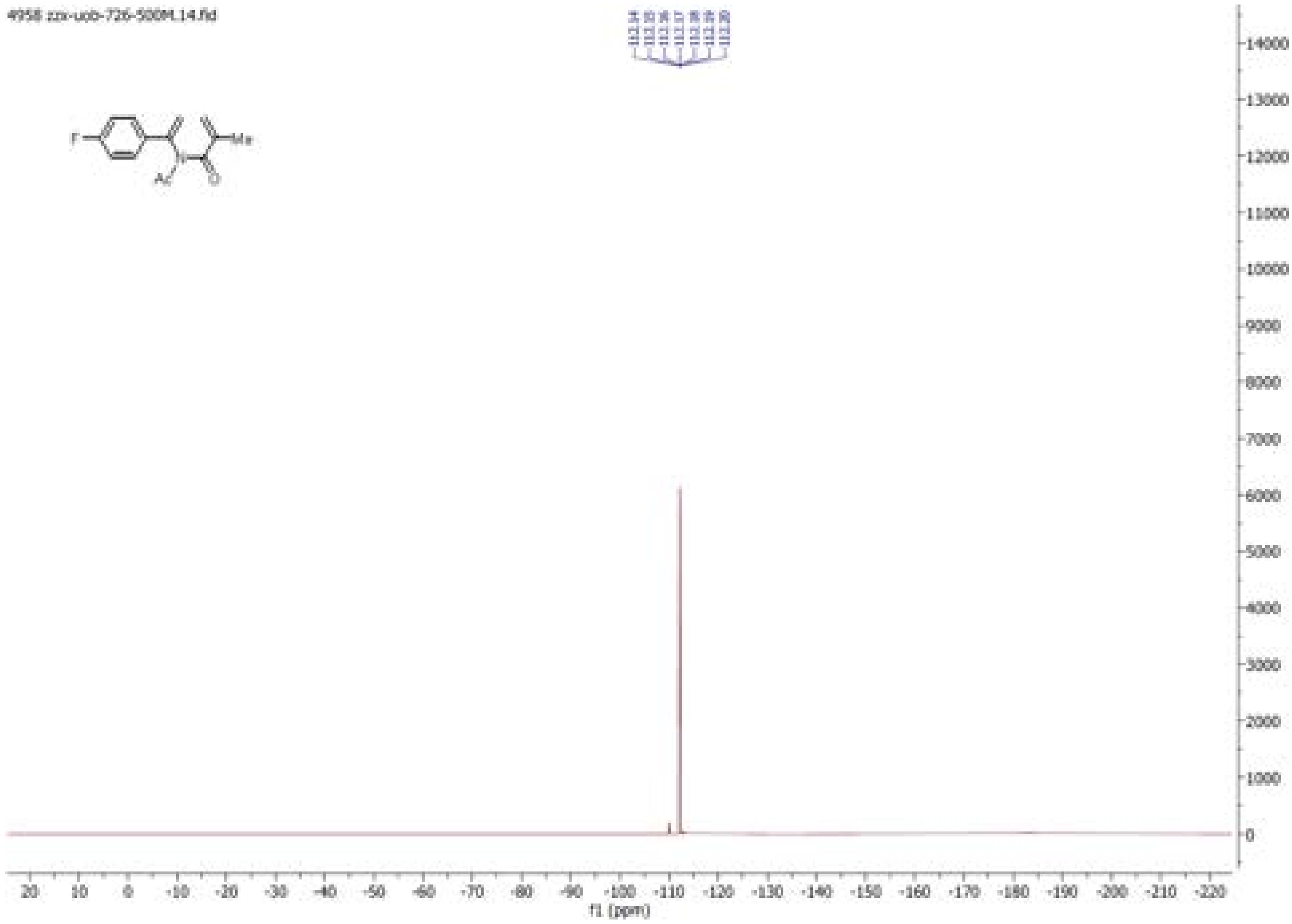

^{1}H NMR (500 MHz, $CDCl_3$) of **2m**

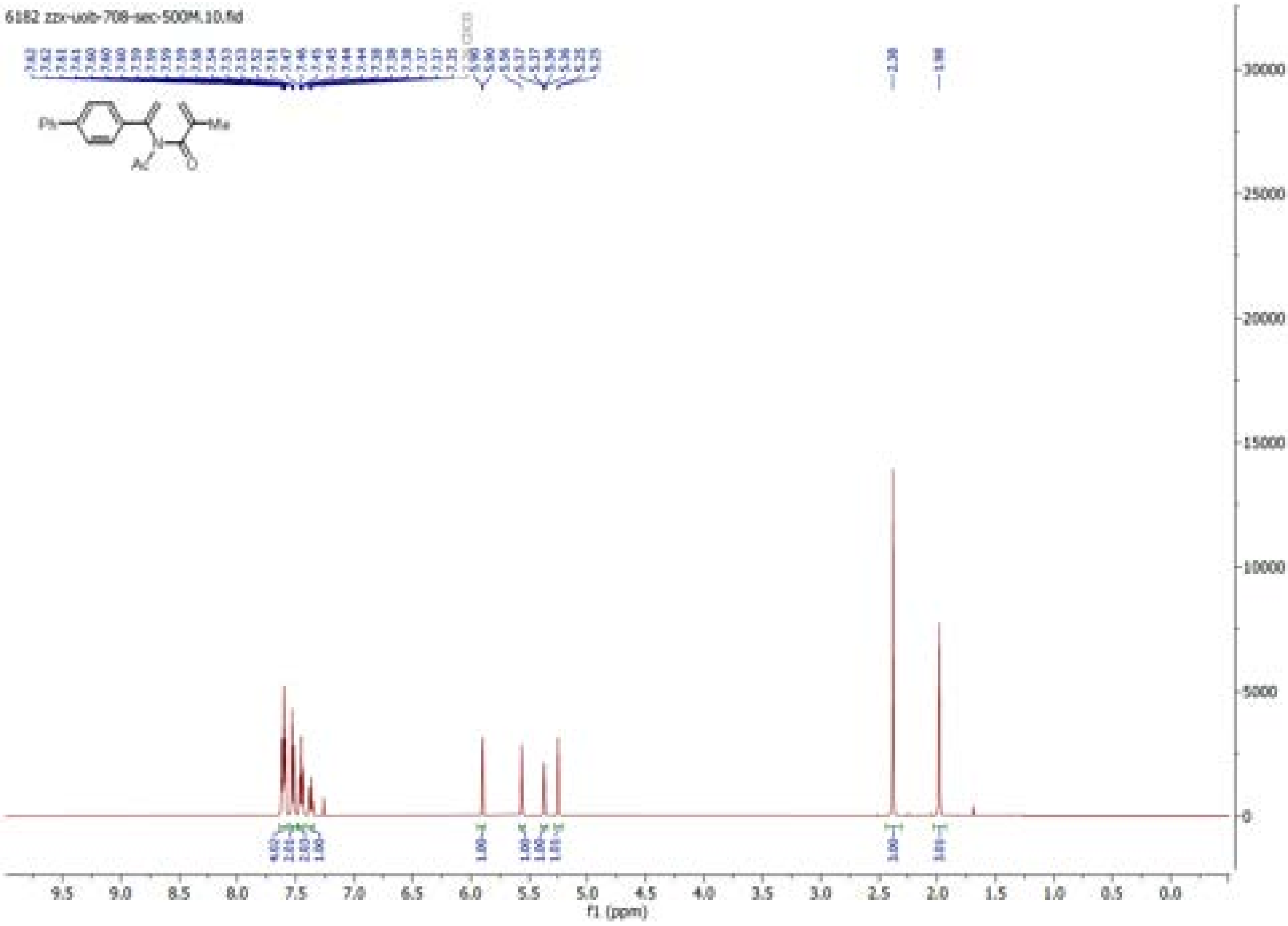

^{13}C NMR (126 MHz, $CDCl_3$) of **2m**

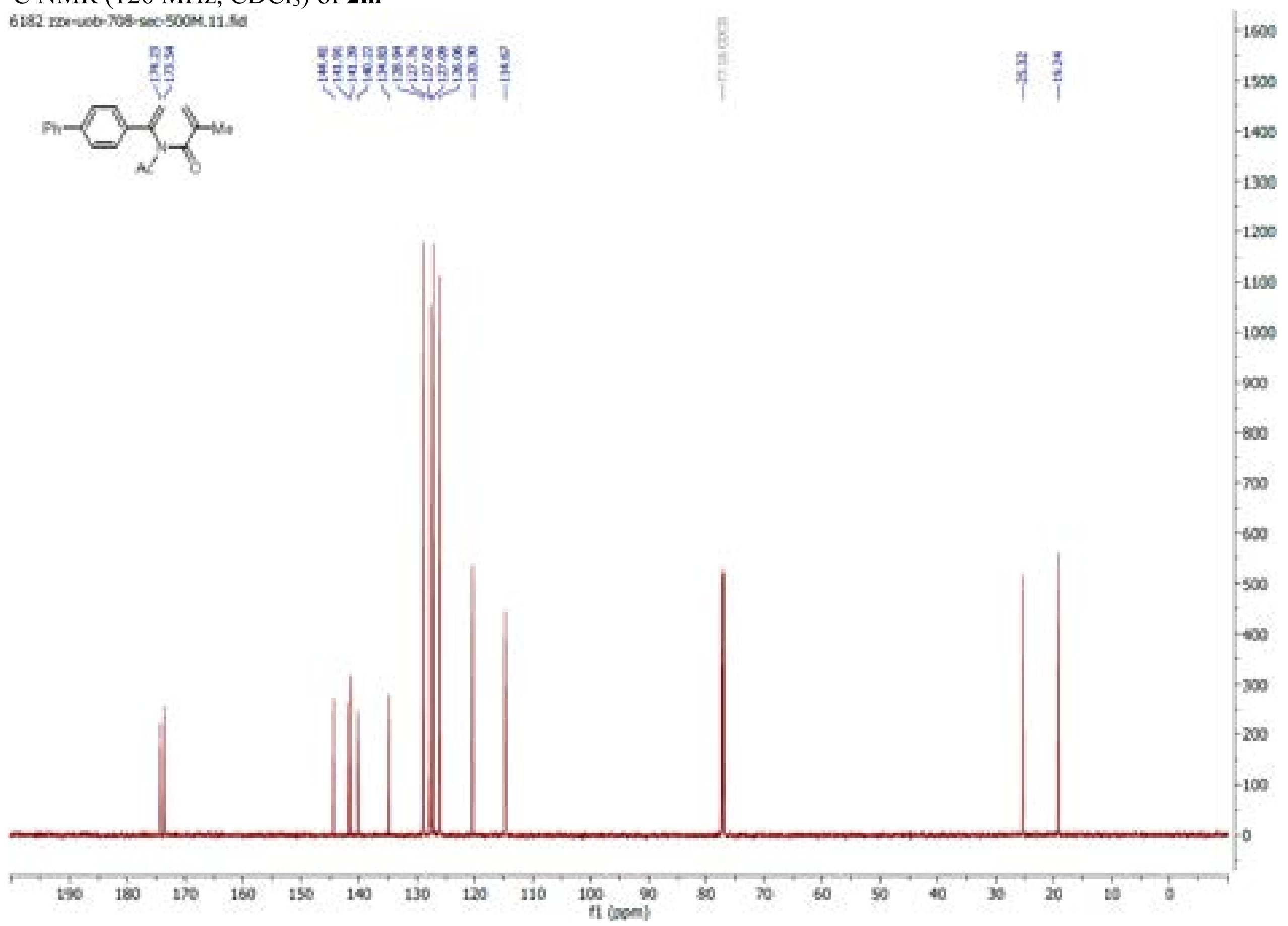

^{1}H NMR (500 MHz, $CDCl_3$) of **2n**

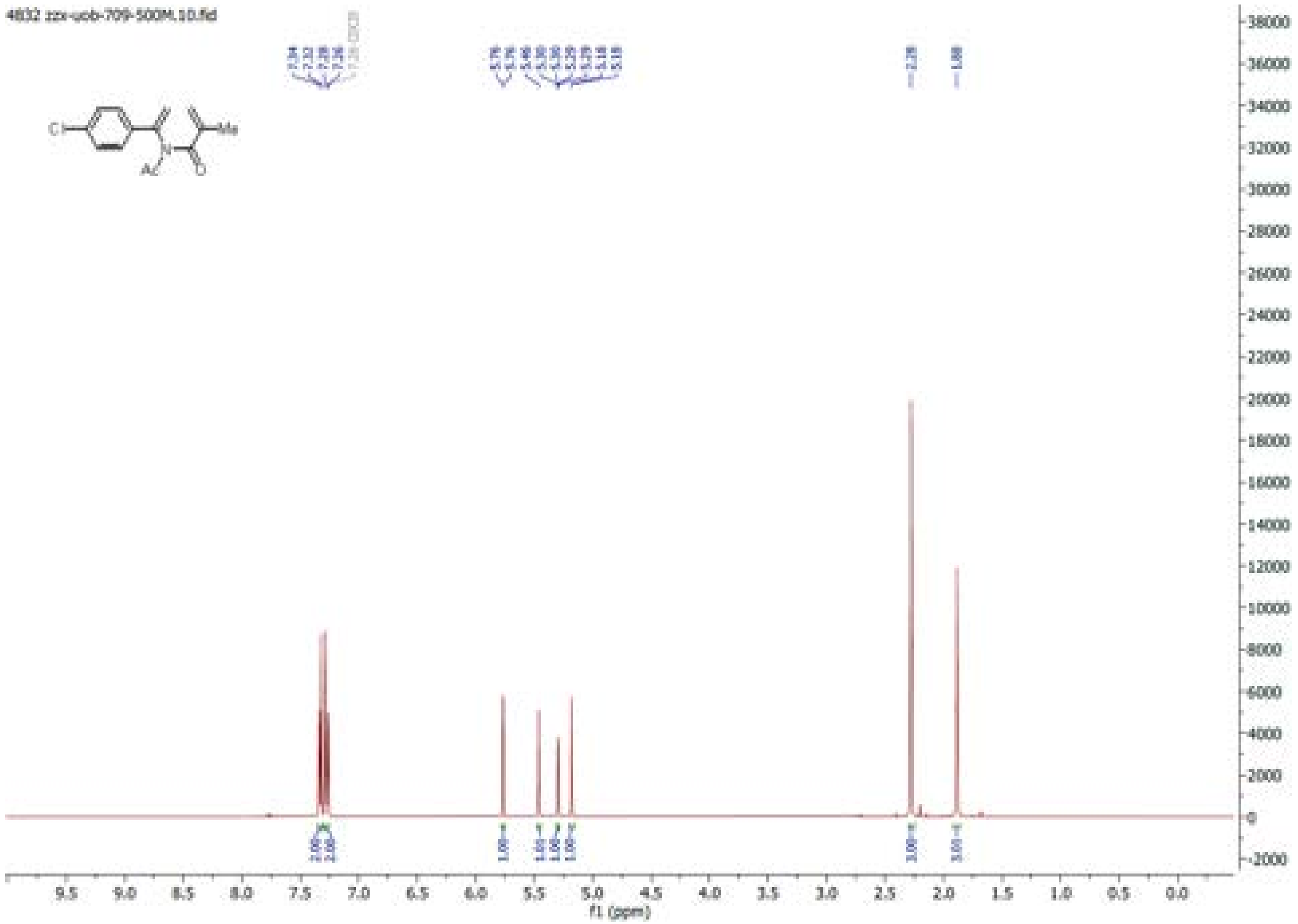

^{13}C NMR (126 MHz, $CDCl_3$) of **2n**

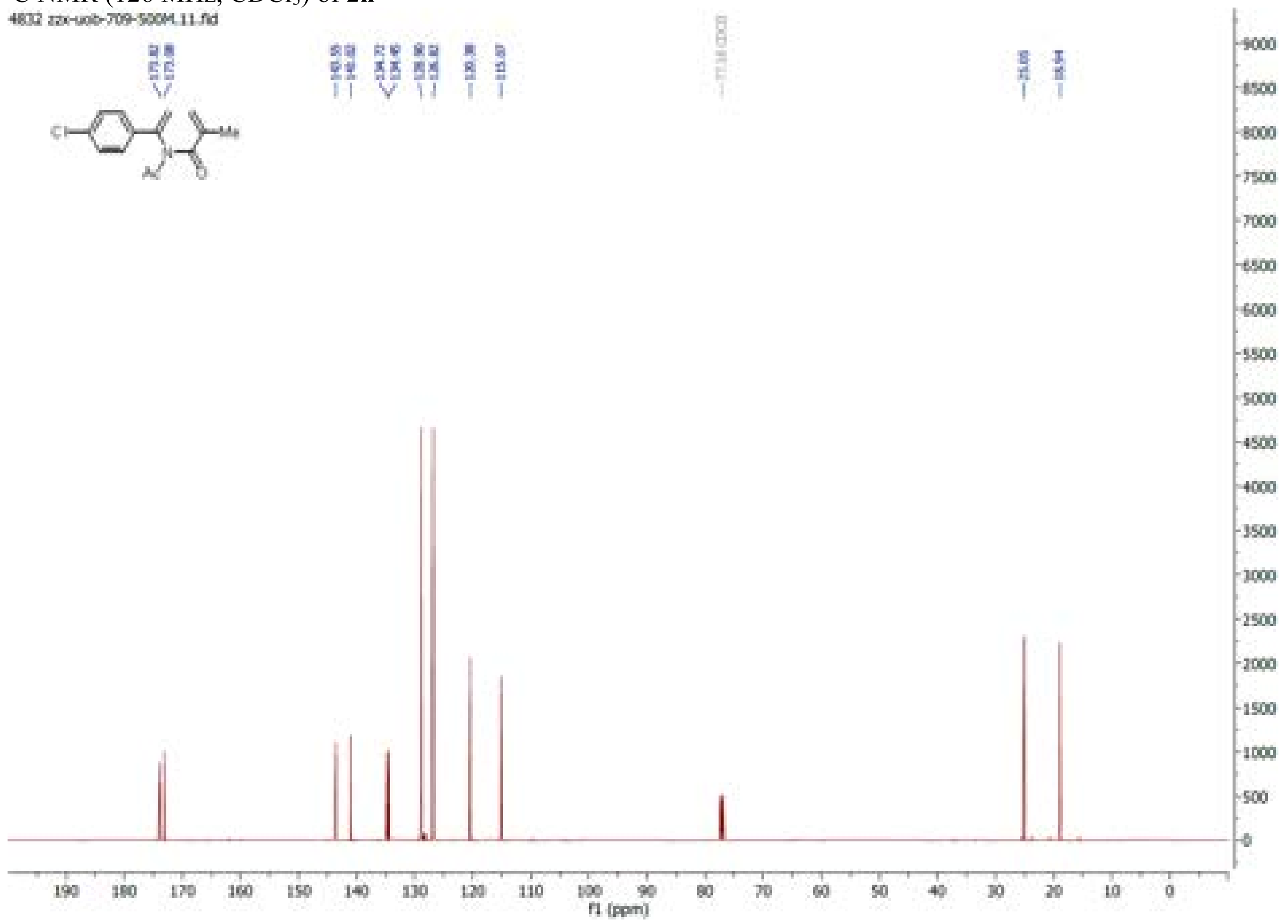

^{1}H NMR (500 MHz, $CDCl_3$) of **2o**

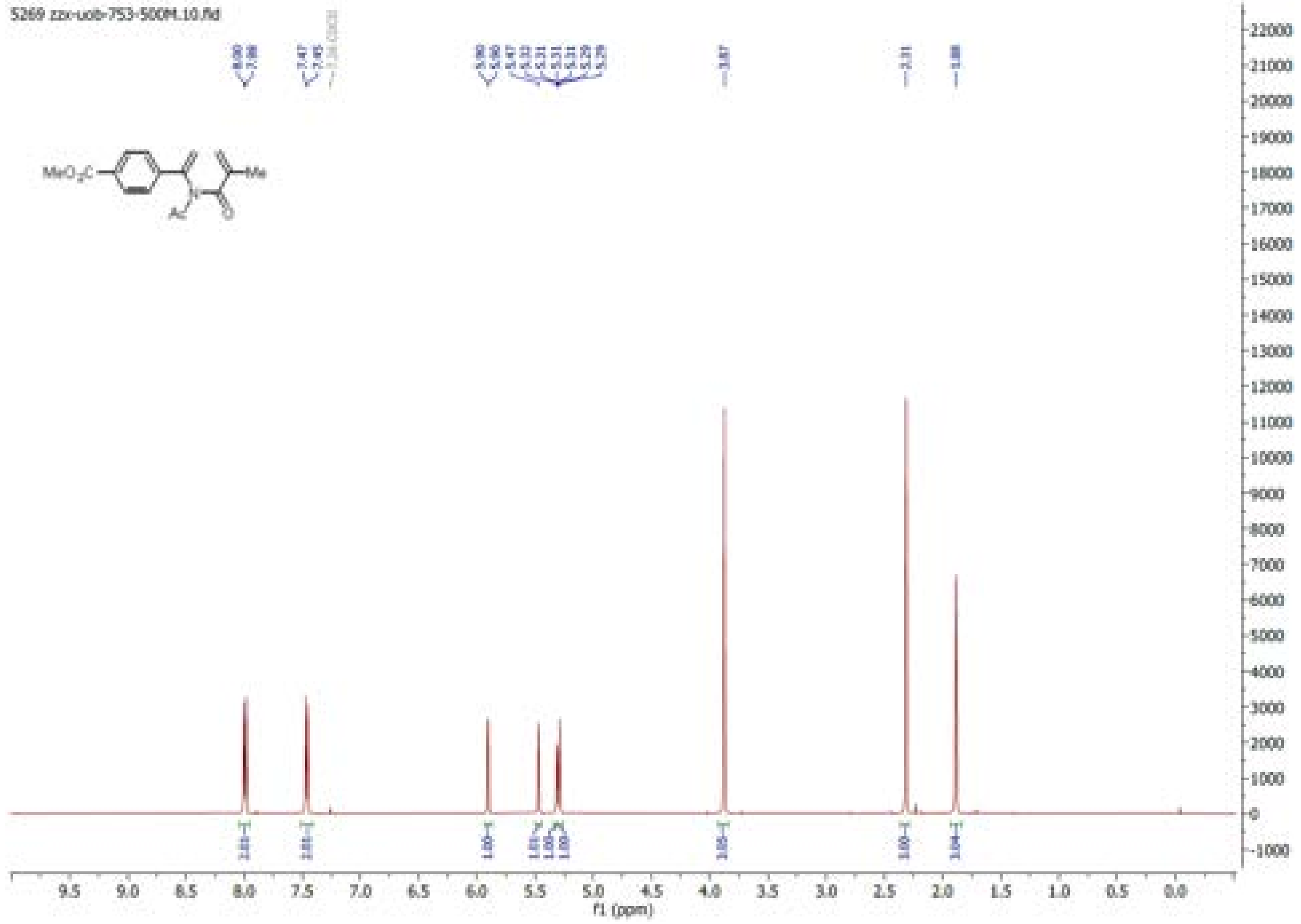

^{13}C NMR (126 MHz, $CDCl_3$) of **2o**

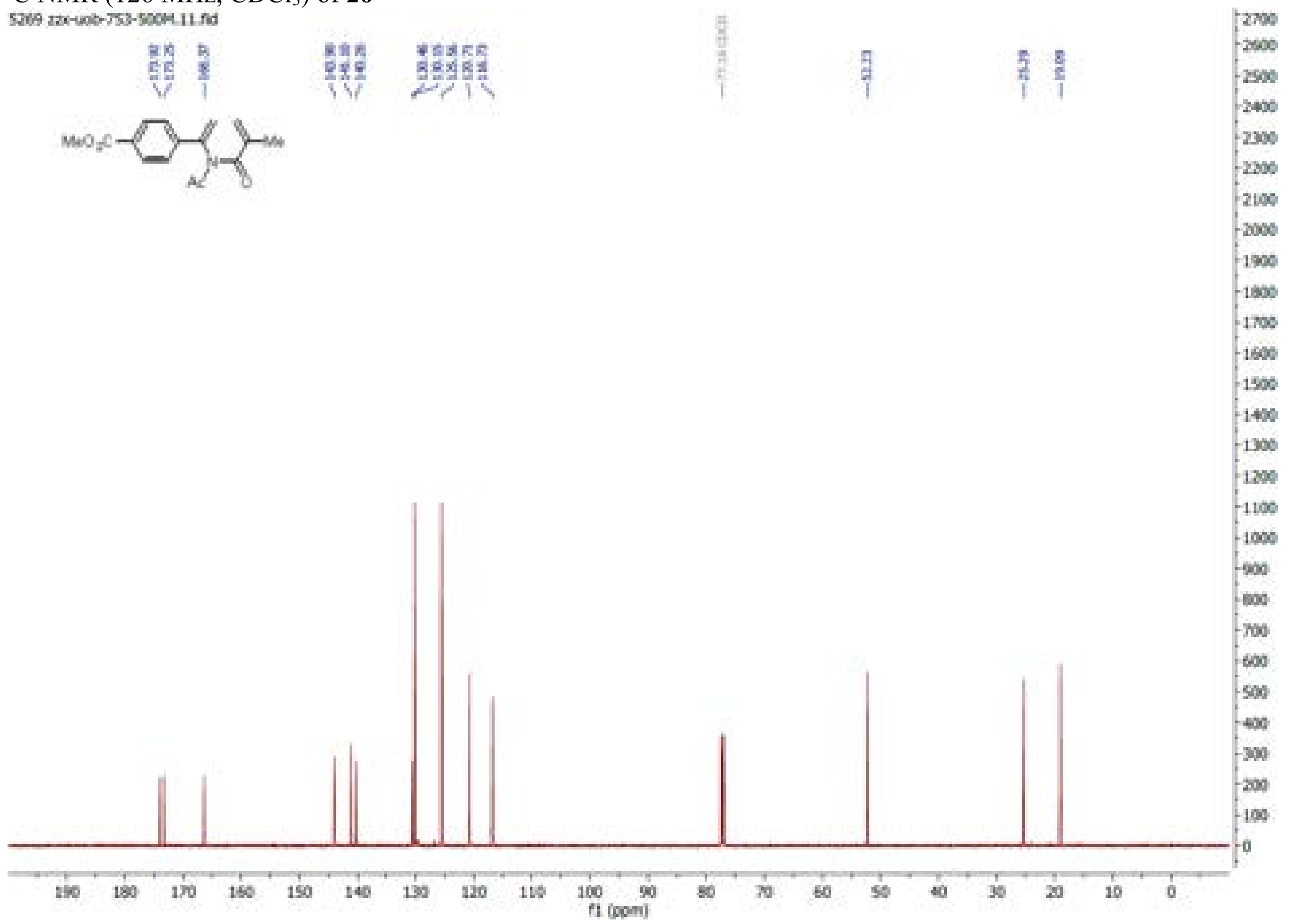

^{1}H NMR (500 MHz, $CDCl_3$) of **2p**

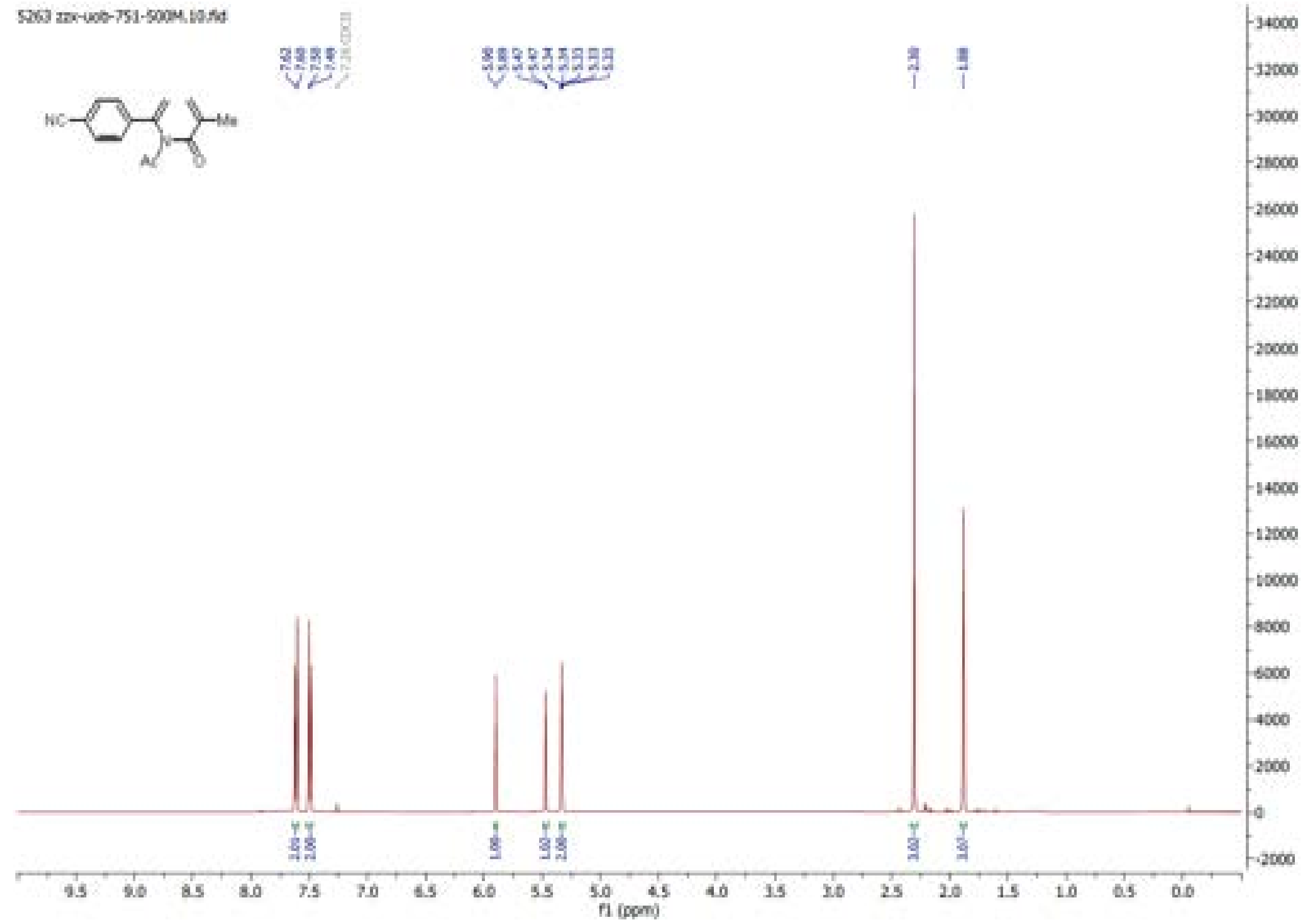

^{13}C NMR (126 MHz, $CDCl_3$) of **2p**

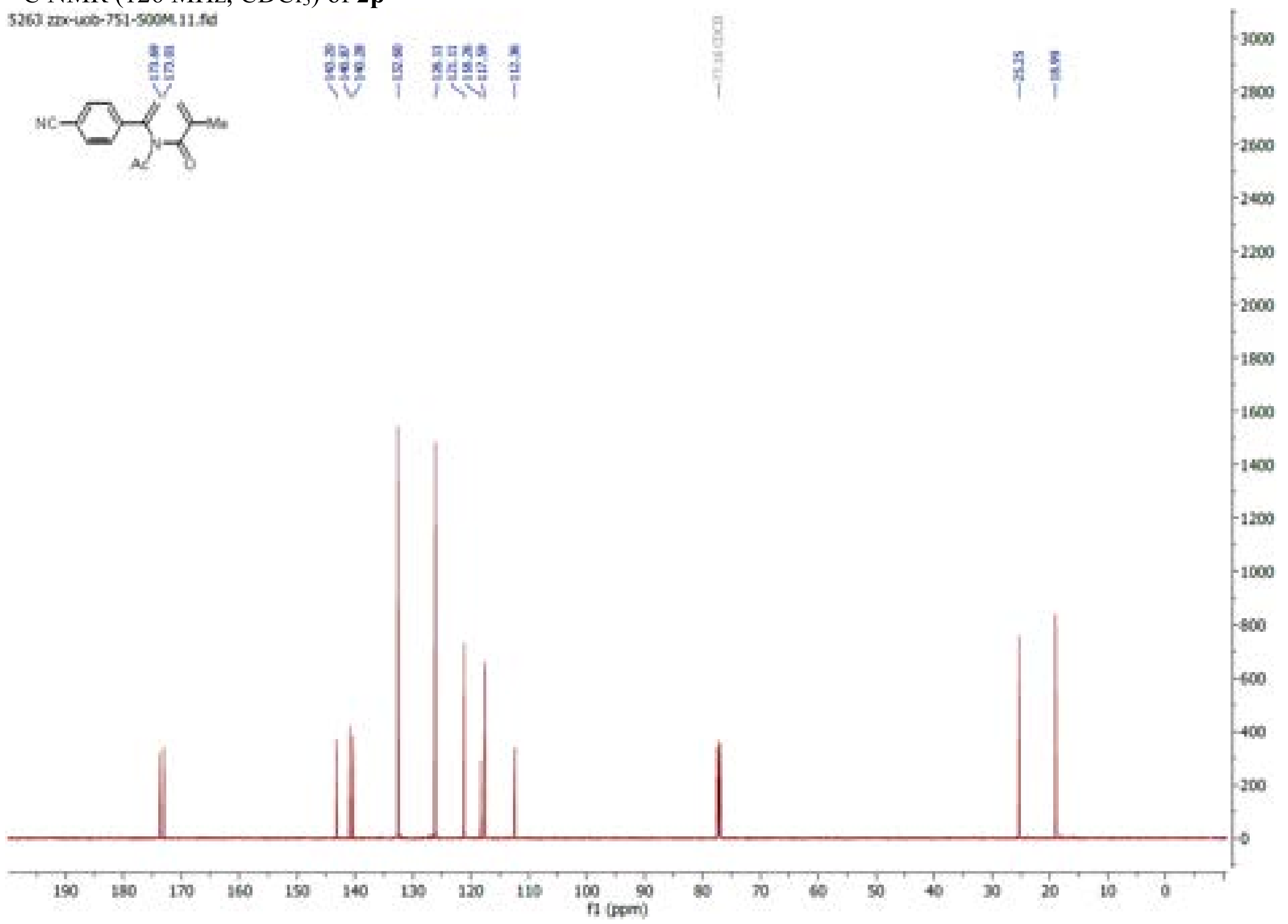

^{1}H NMR (500 MHz, $CDCl_3$) of **2q**

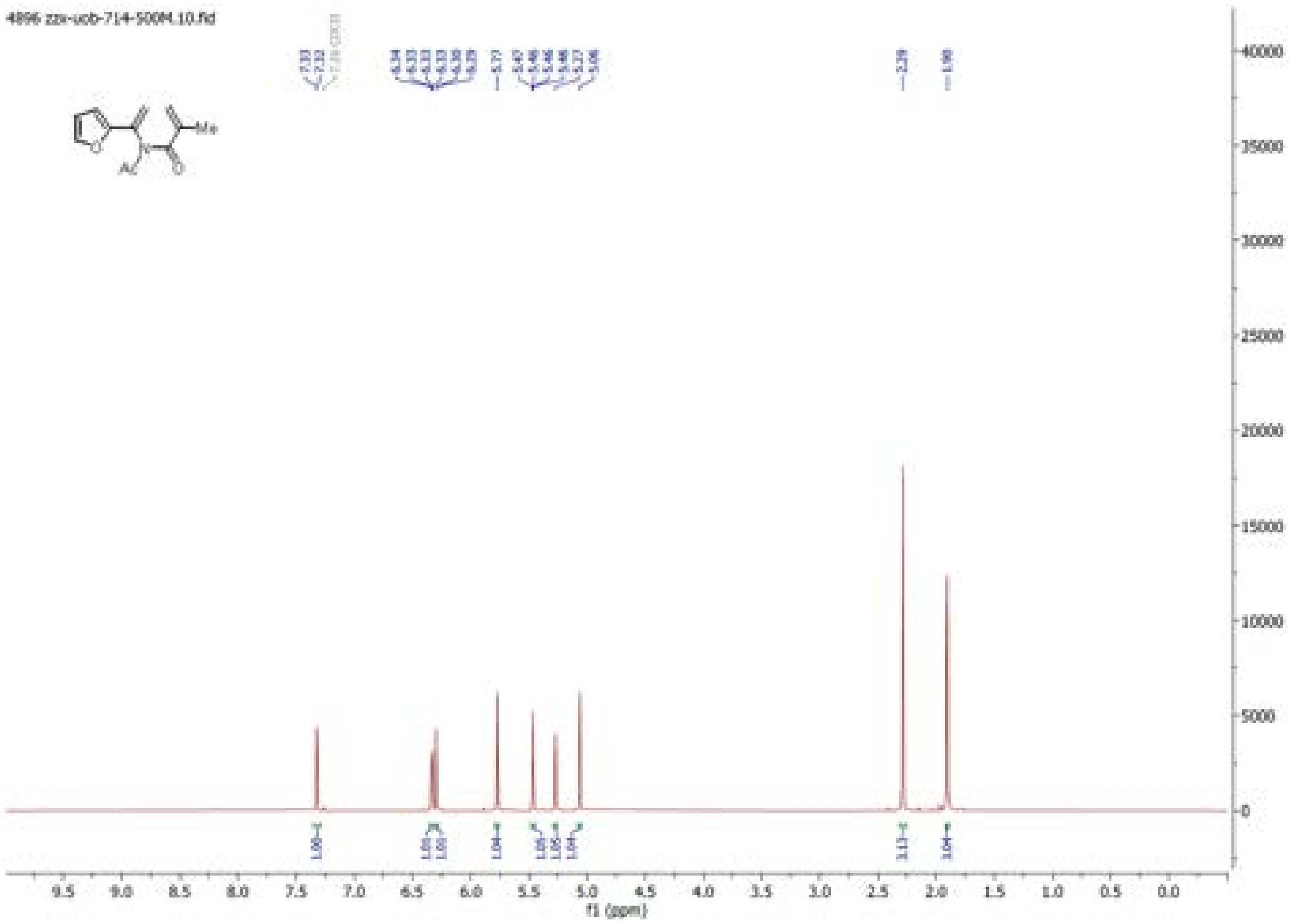

^{13}C NMR (126 MHz, $CDCl_3$) of **2q**

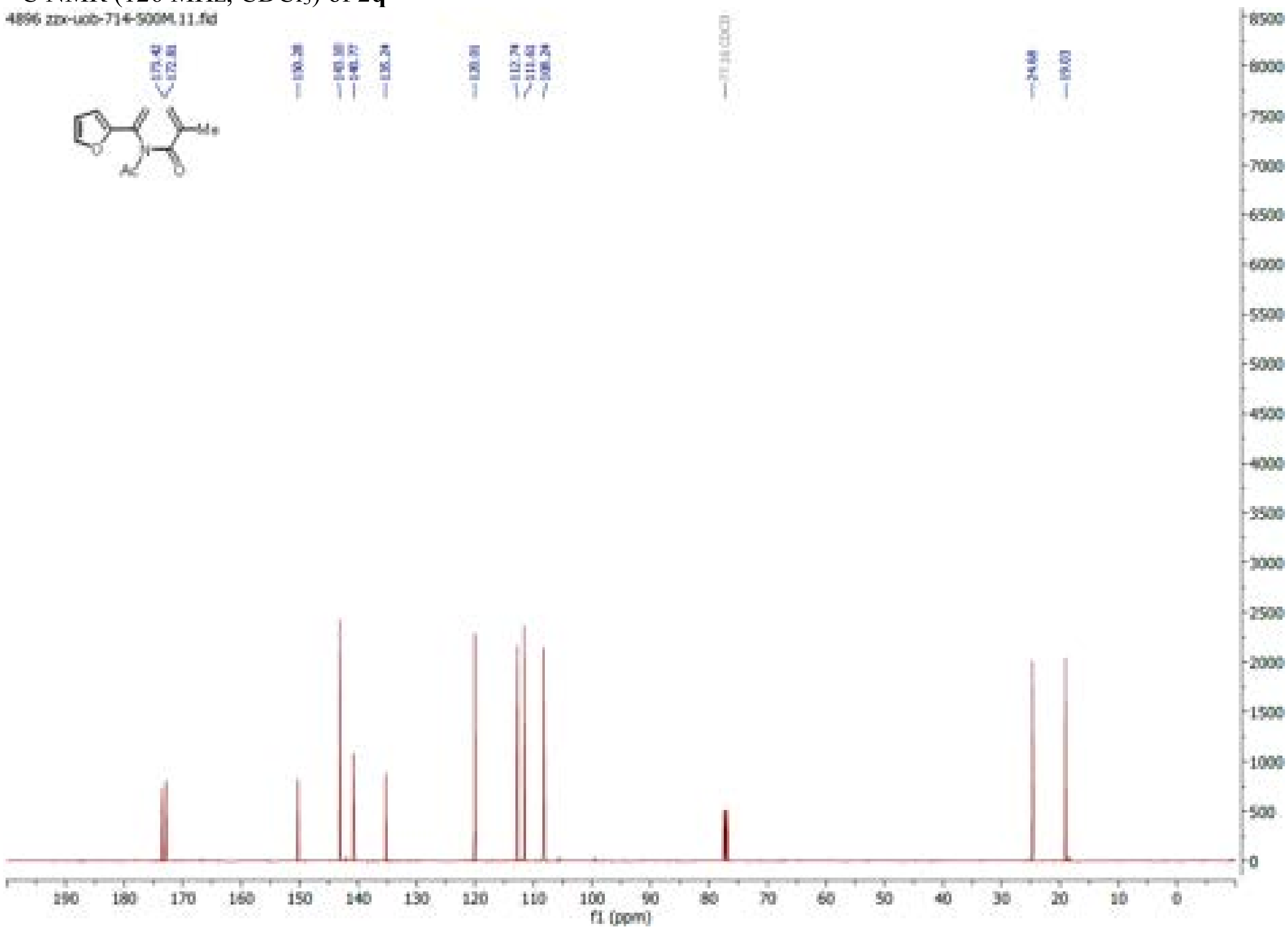

^1H NMR (500 MHz, $CDCl_3$) of **2r**

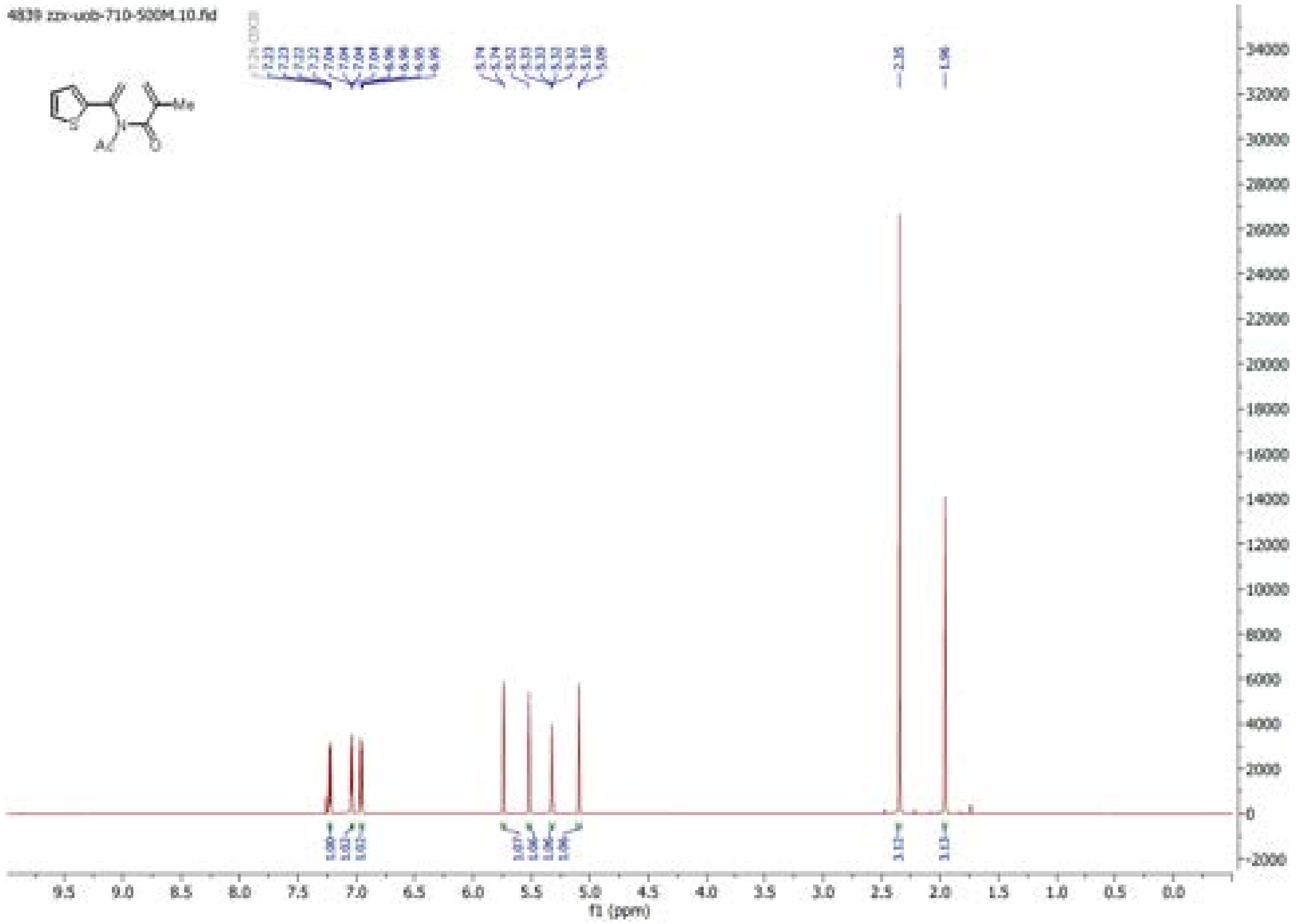

^{13}C NMR (126 MHz, $CDCl_3$) of **2r**

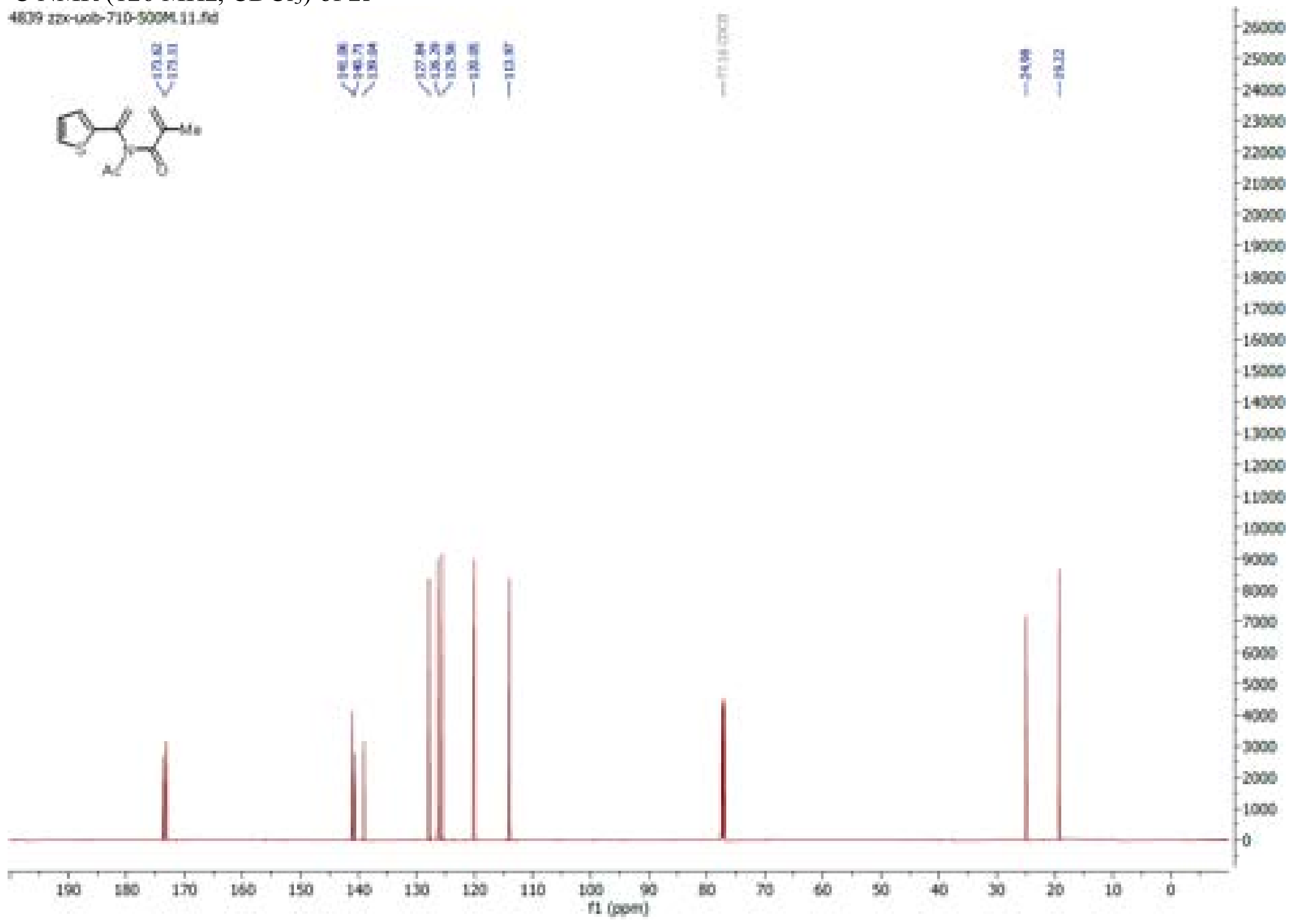

^{1}H NMR (500 MHz, $CDCl_3$) of **2s**

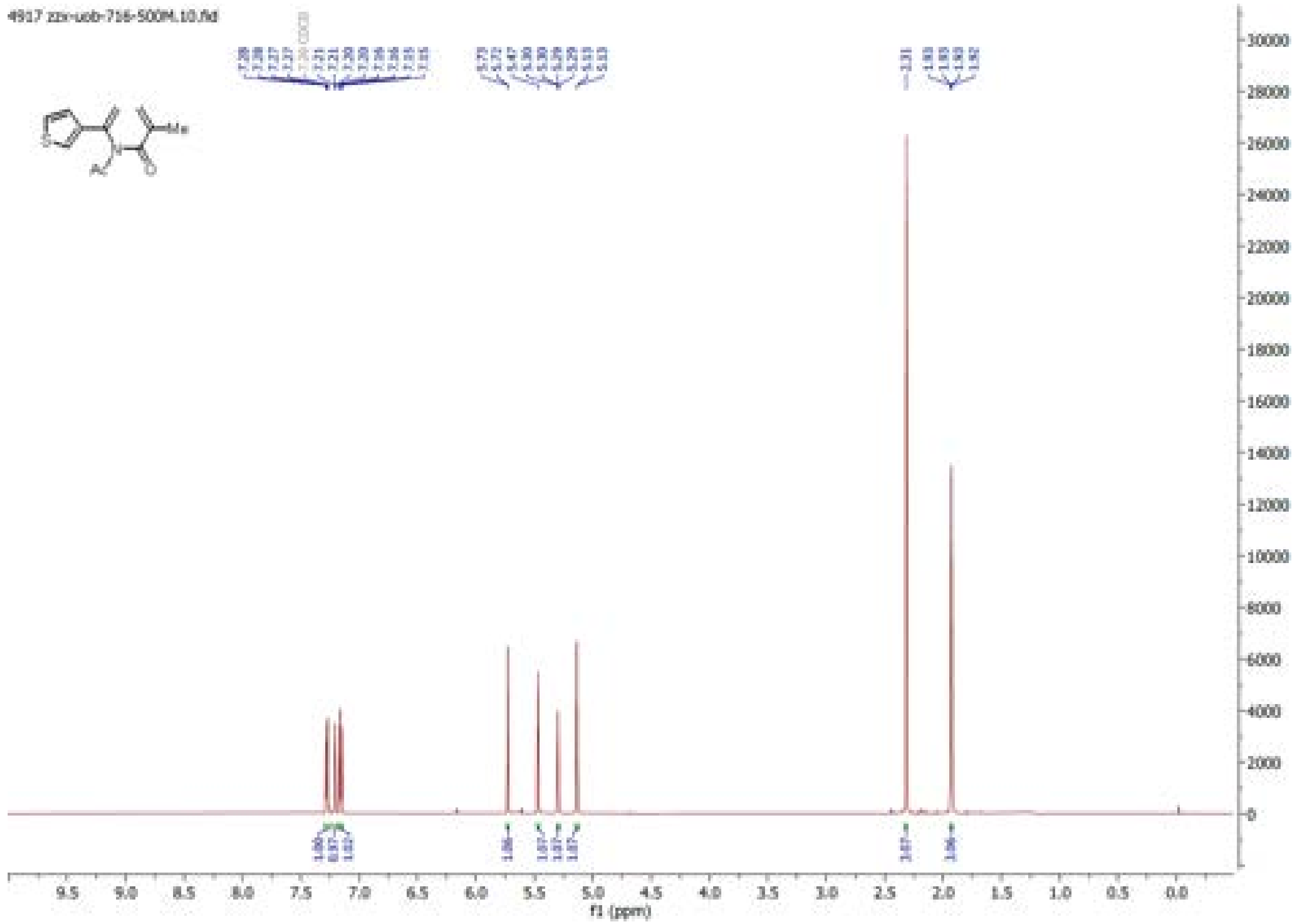

^{13}C NMR (126 MHz, $CDCl_3$) of **2s**

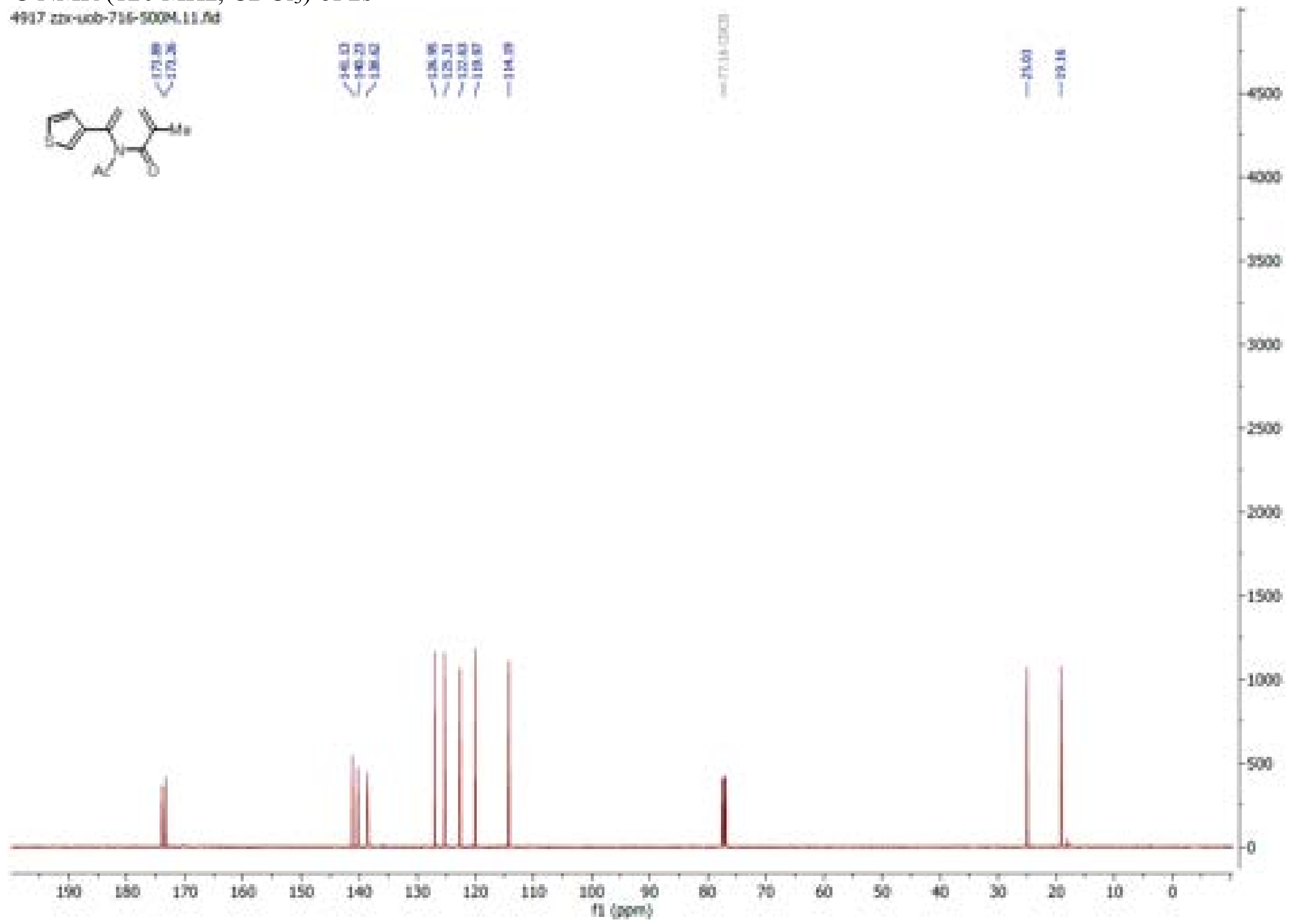

^{1}H NMR (500 MHz, $CDCl_3$) of **S2t**

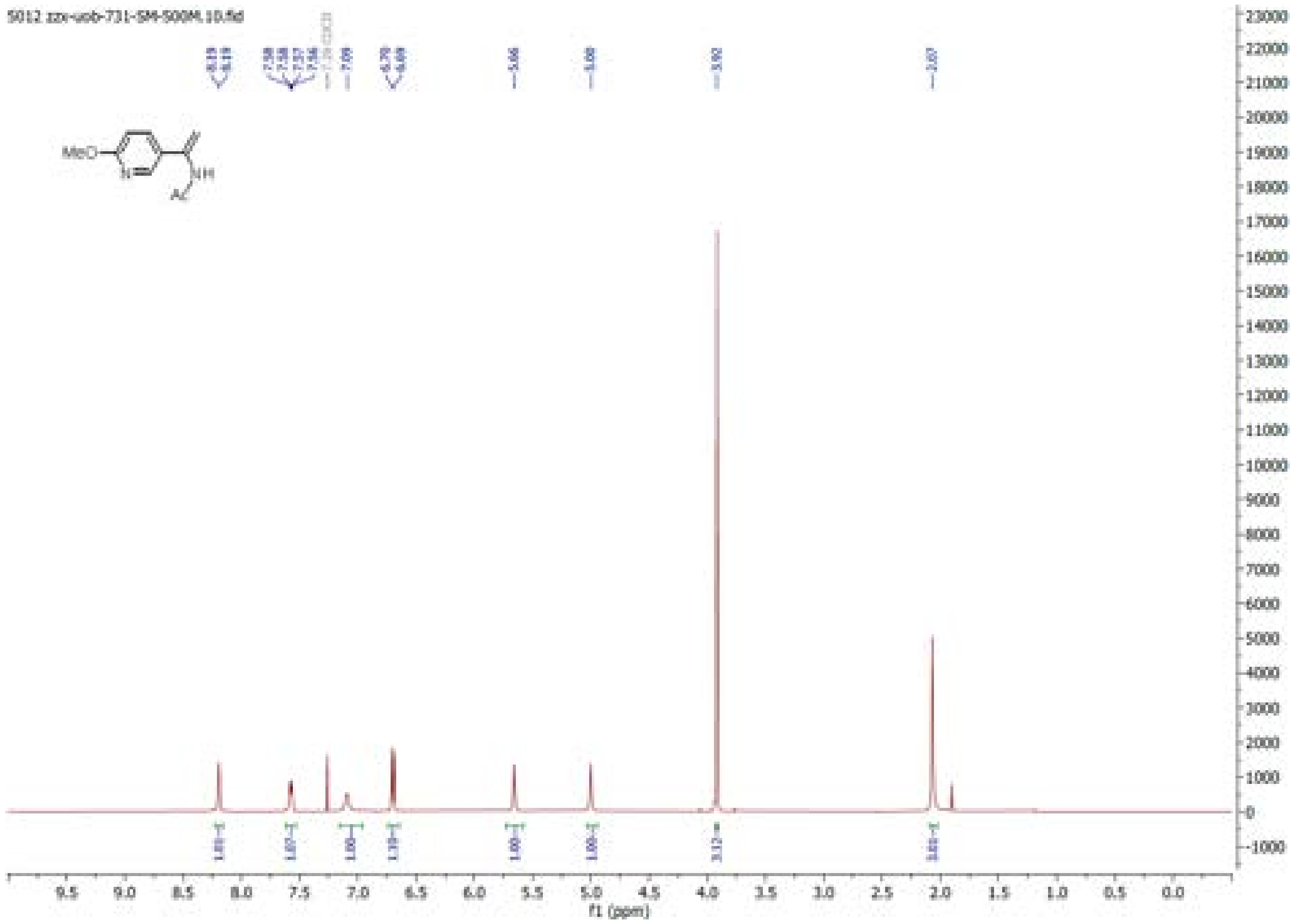

^{13}C NMR (126 MHz, $CDCl_3$) of **S2t**

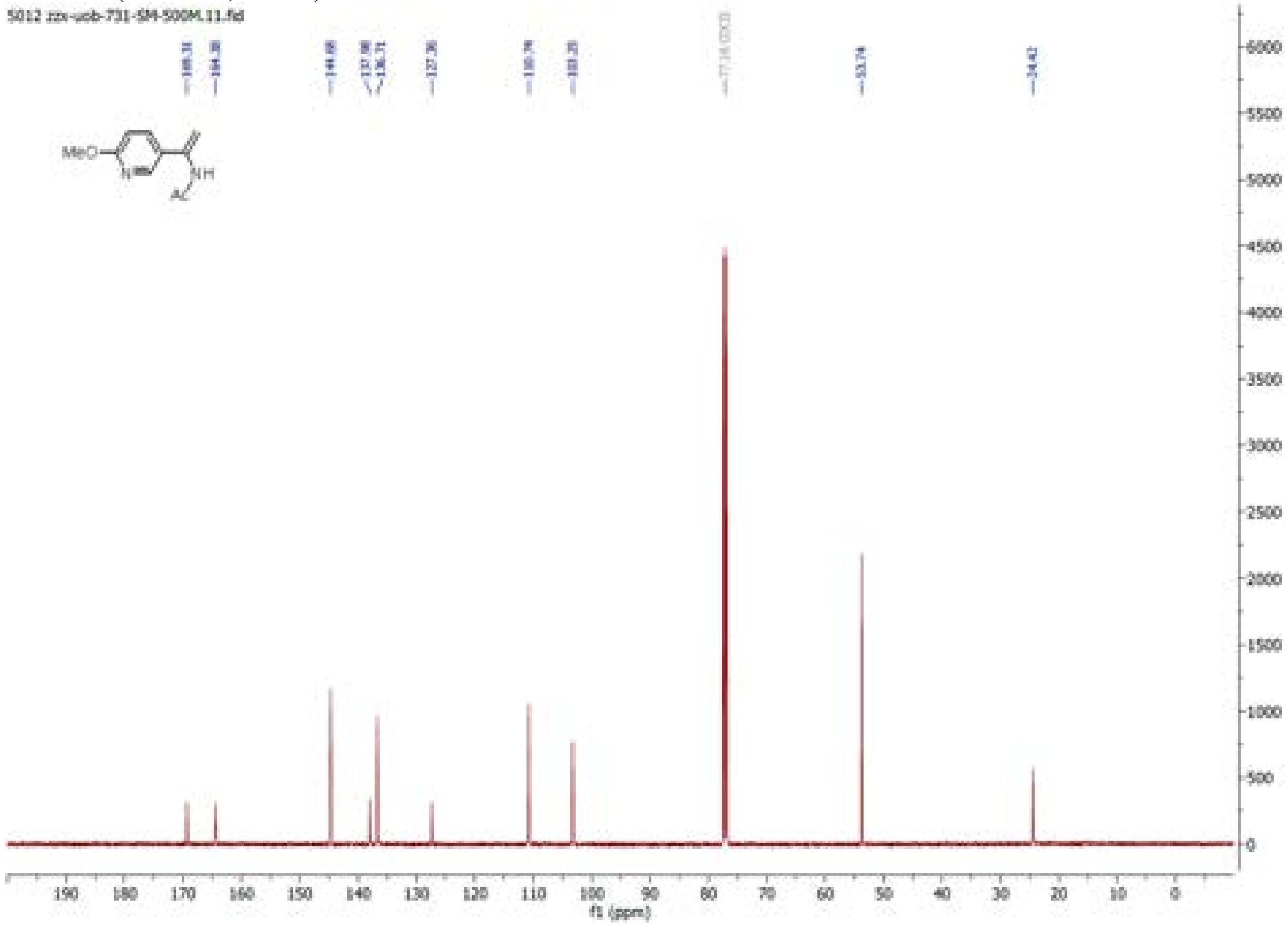

^{1}H NMR (500 MHz, $CDCl_3$) of **2t**

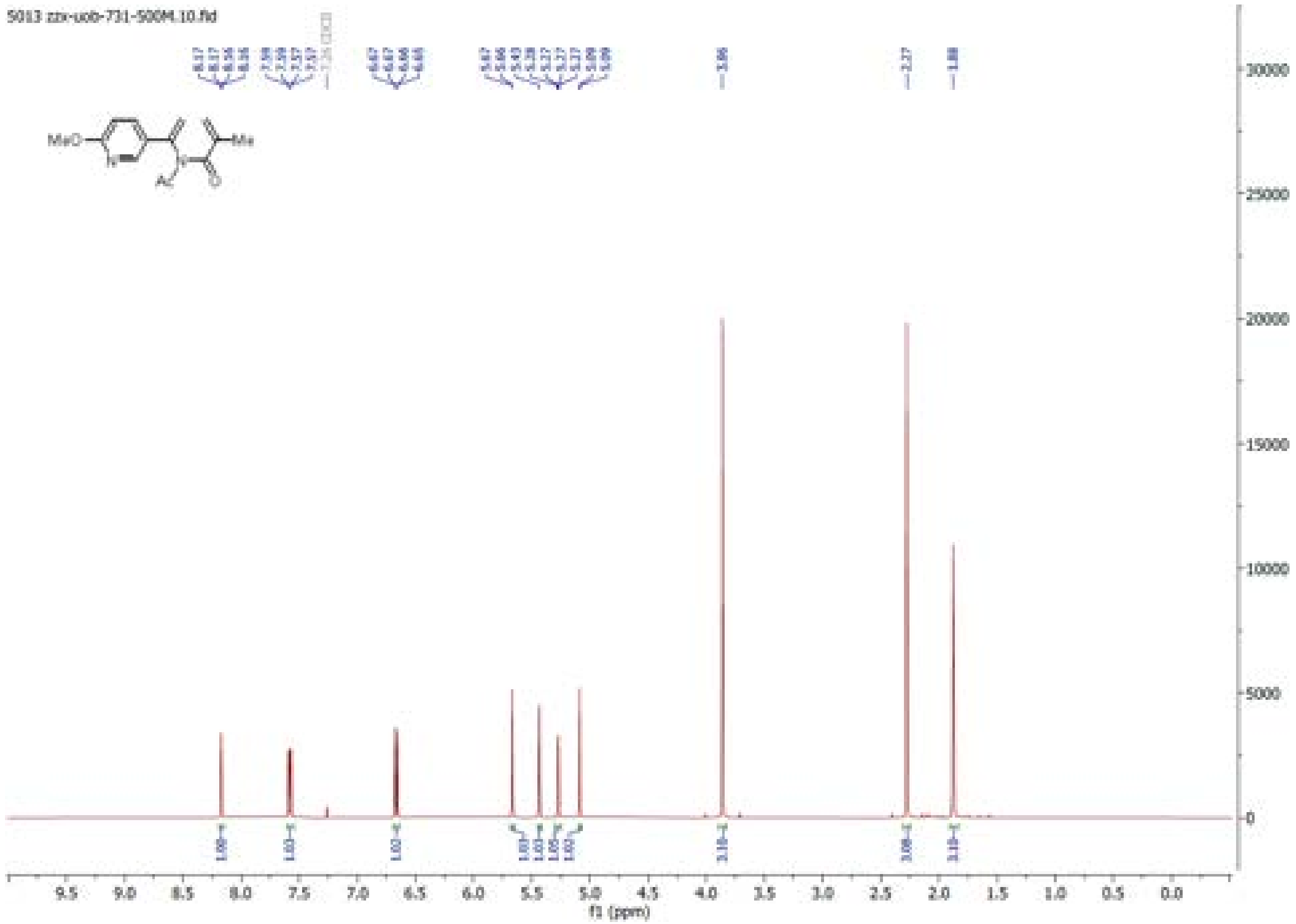

^{13}C NMR (126 MHz, $CDCl_3$) of **2t**

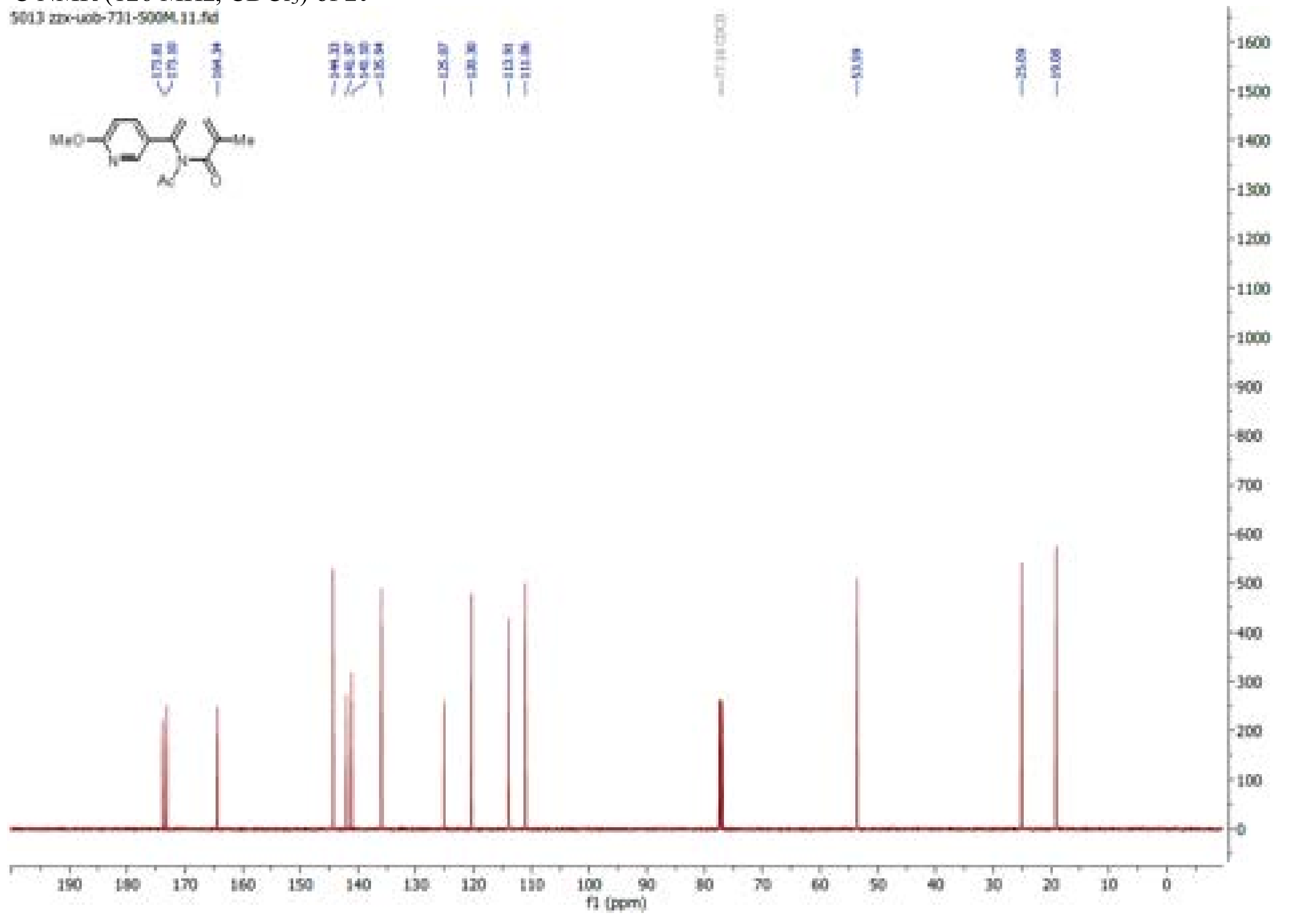

^{1}H NMR (500 MHz, $CDCl_3$) of **2u**

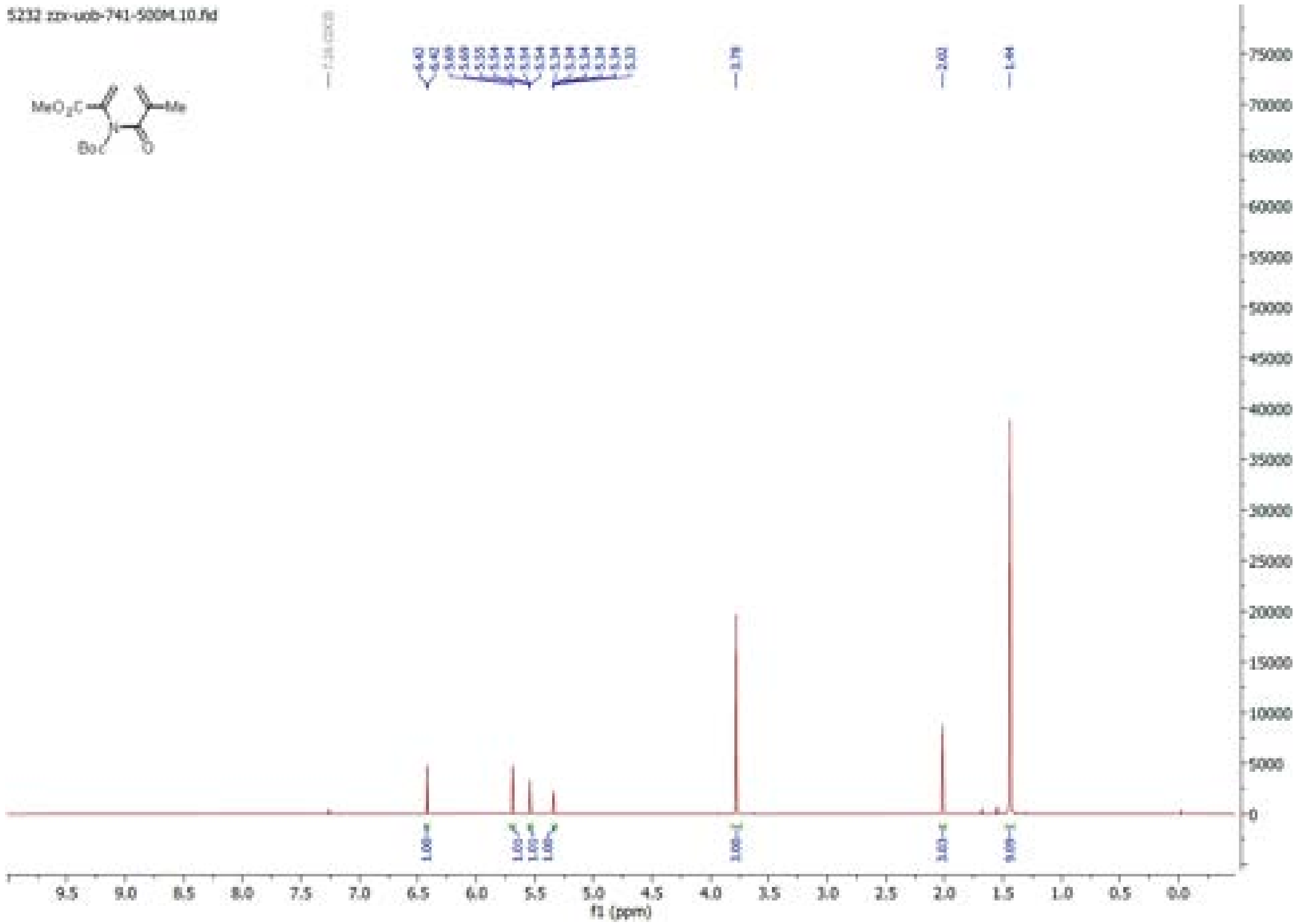

^{13}C NMR (126 MHz, $CDCl_3$) of **2u**

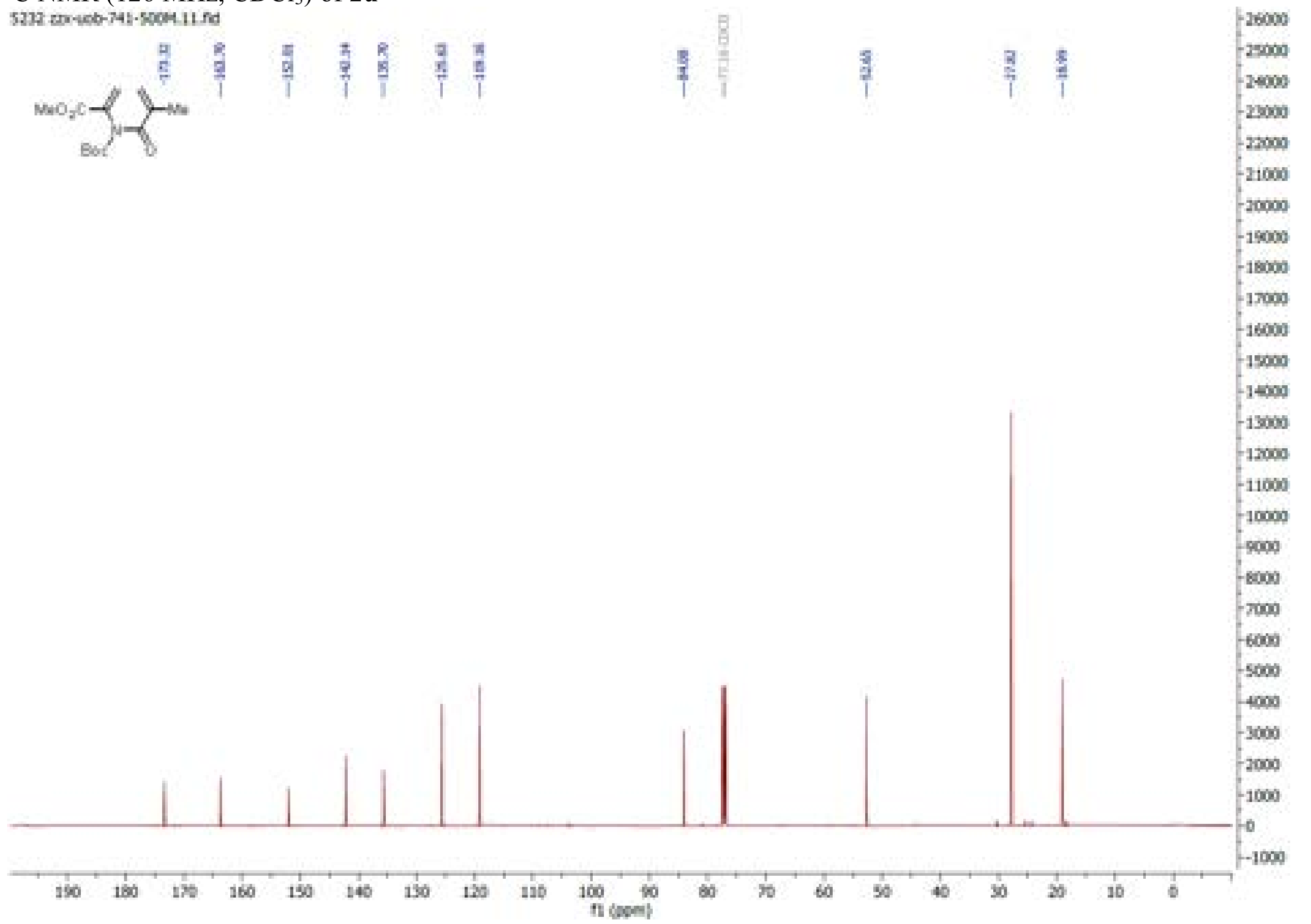

^{1}H NMR (500 MHz, $CDCl_3$) of S**2v**

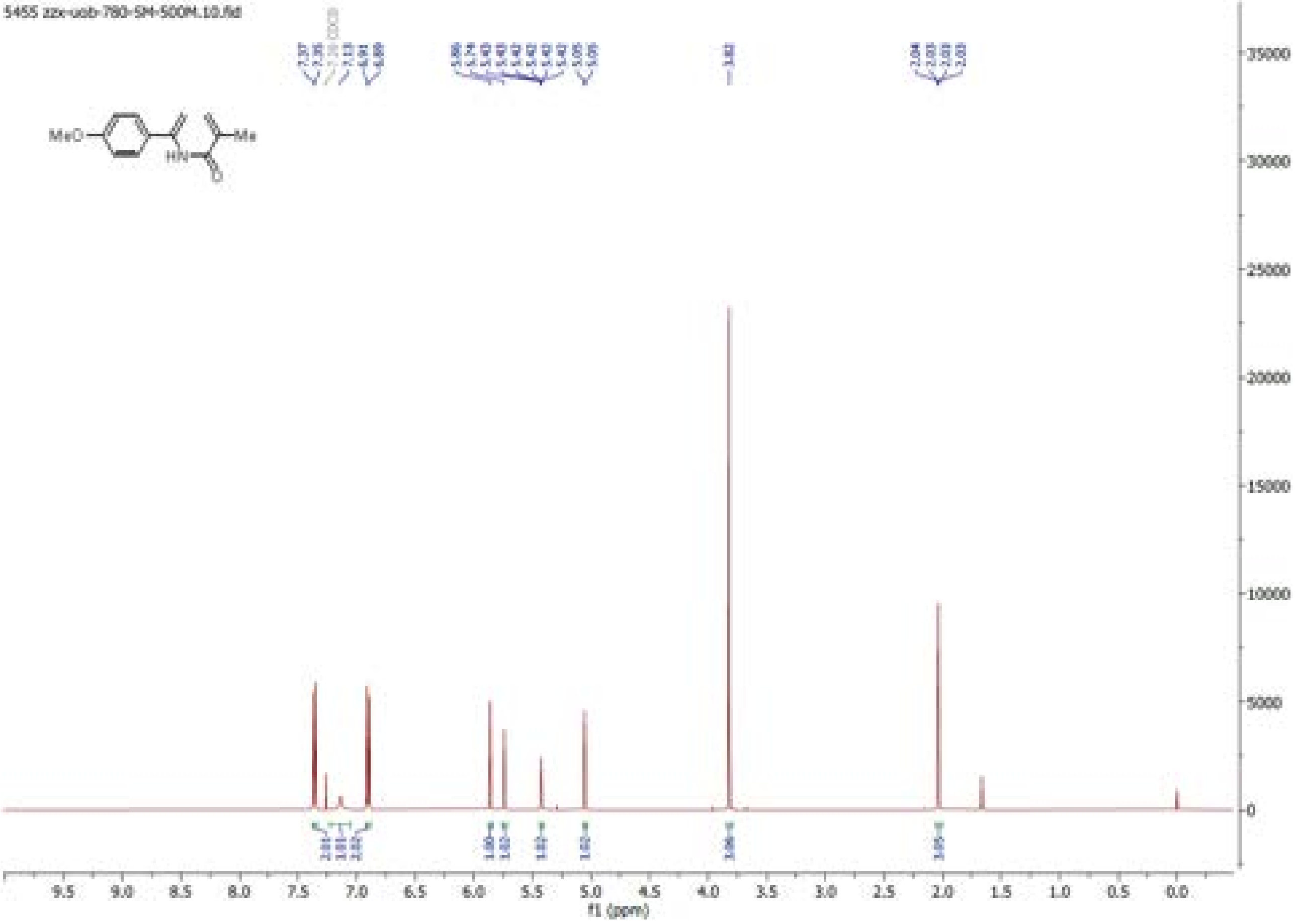

^{13}C NMR (126 MHz, $CDCl_3$) of S**2v**

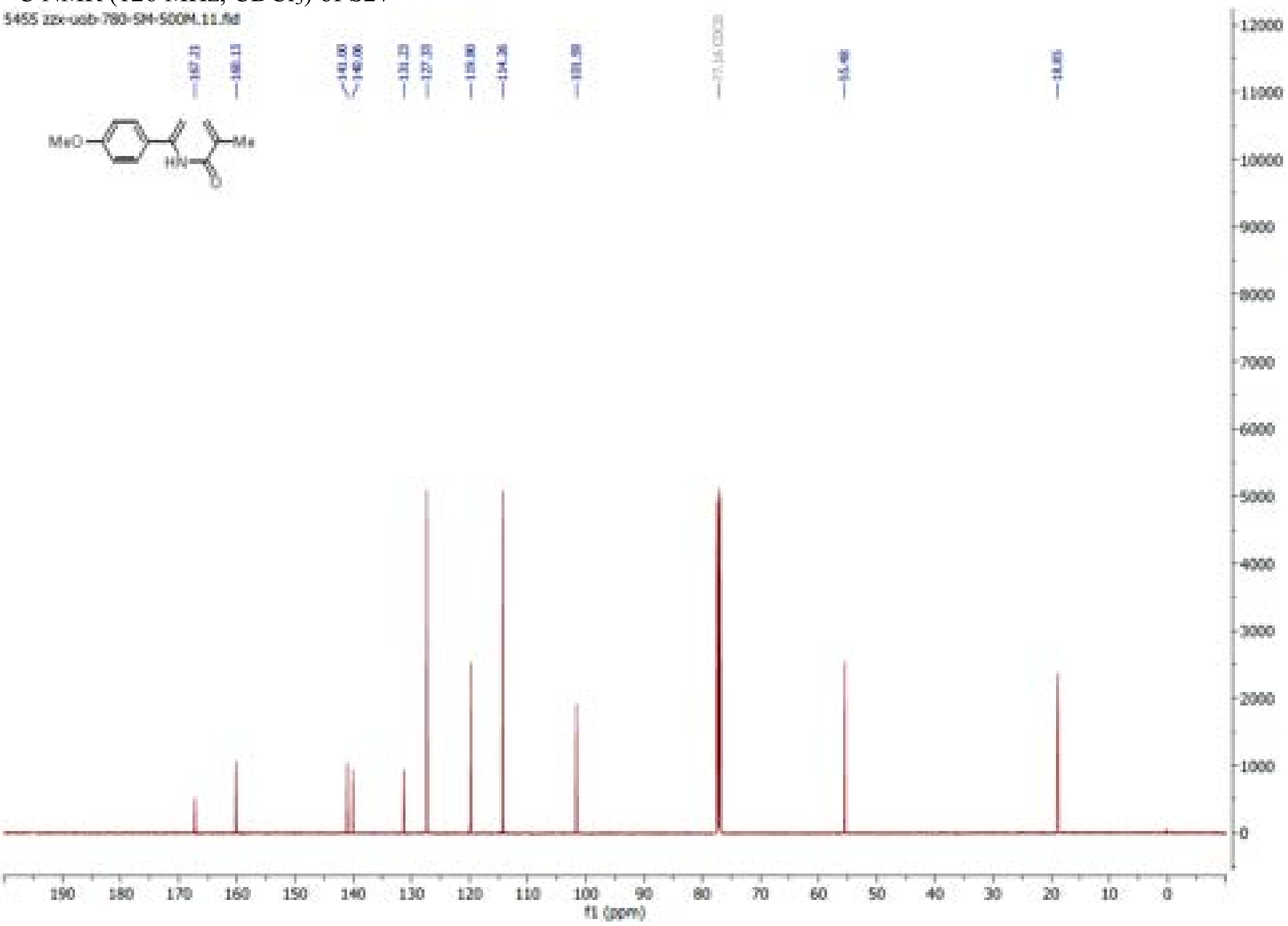

^{1}H NMR (500 MHz, $CDCl_3$) of **2v**

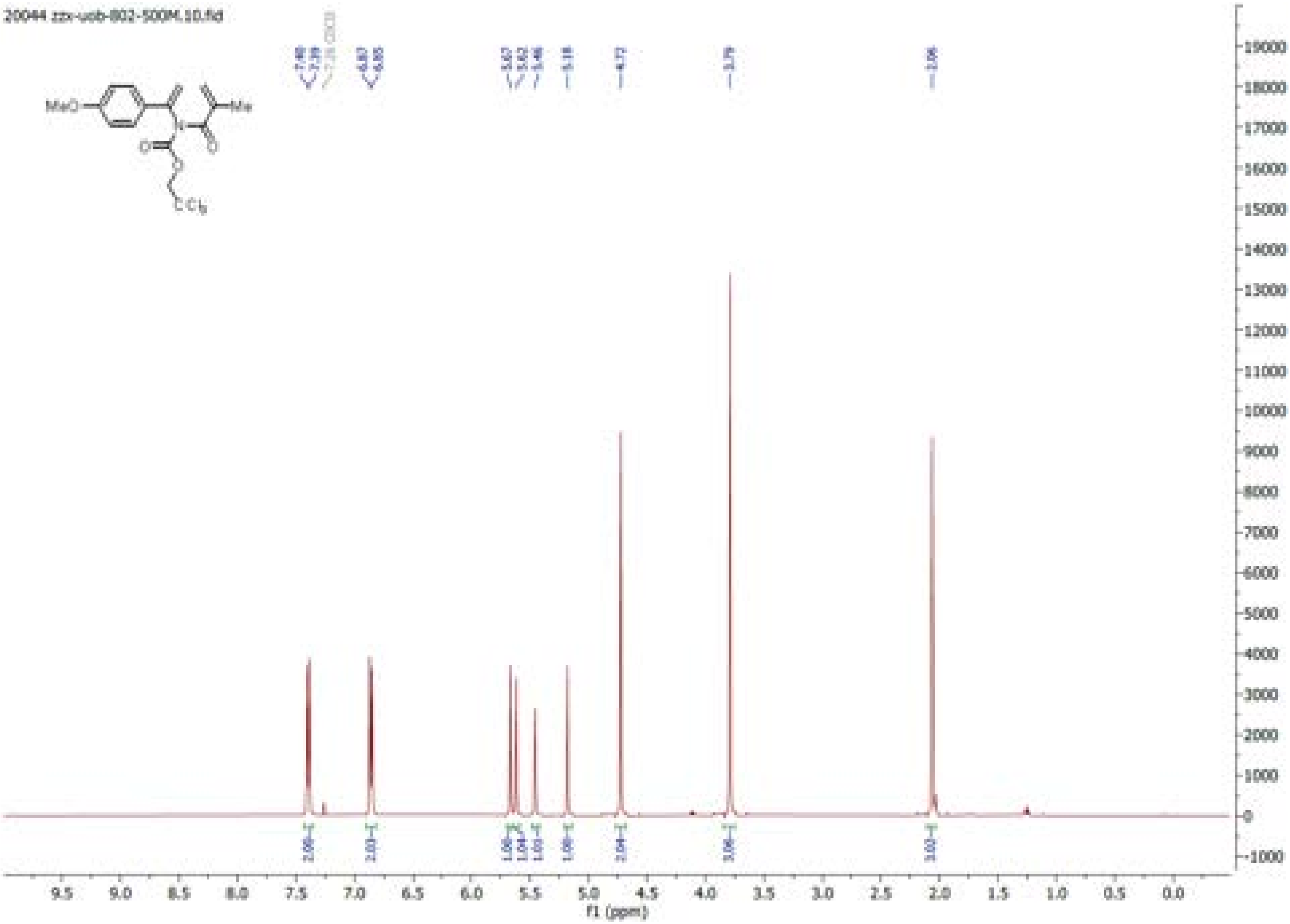

^{13}C NMR (126 MHz, $CDCl_3$) of **2v**

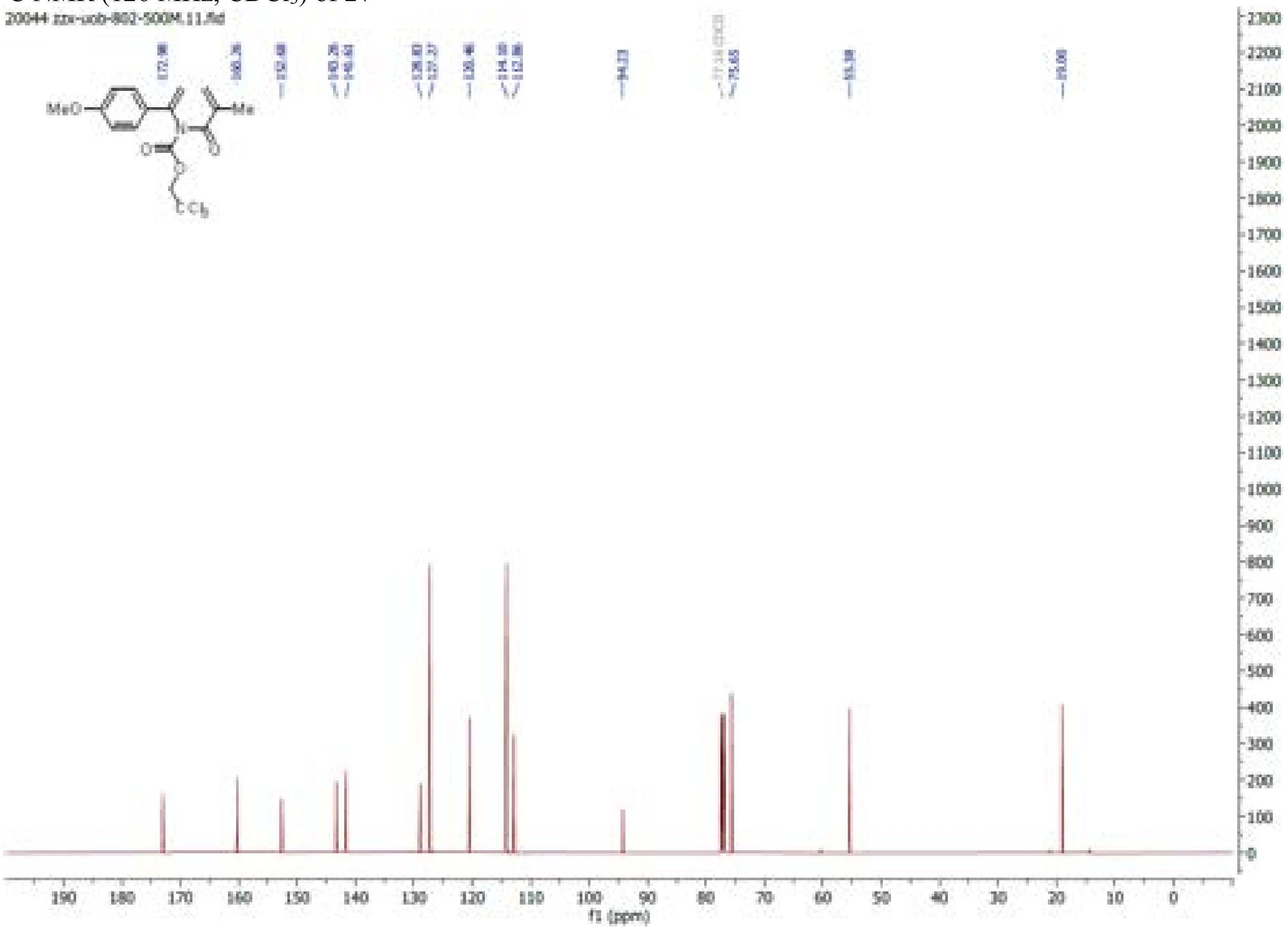

^{1}H NMR (500 MHz, $CDCl_3$) of **2w**

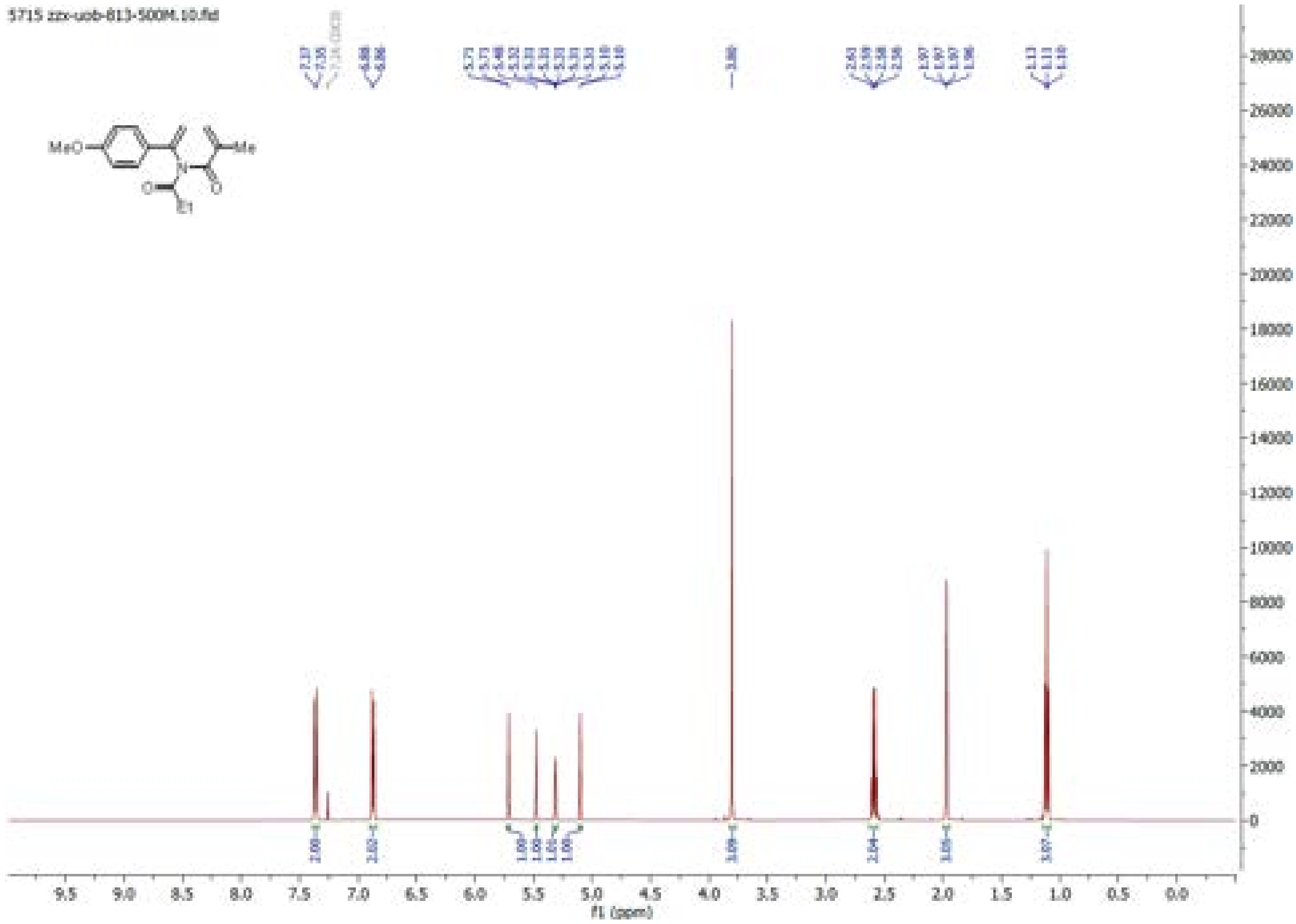

^{13}C NMR (126 MHz, $CDCl_3$) of **2w**

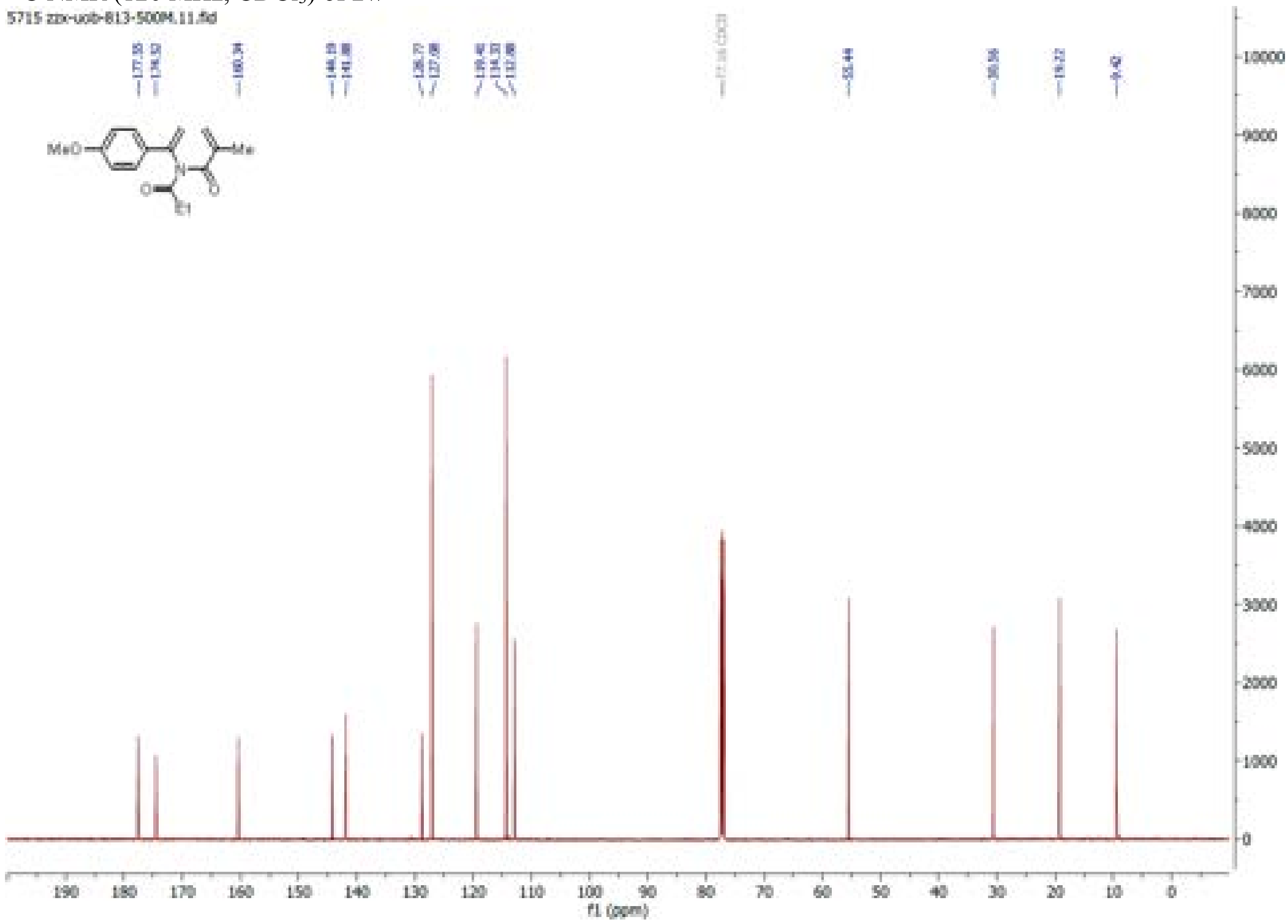

^{1}H NMR (500 MHz, $CDCl_3$) of **2x**

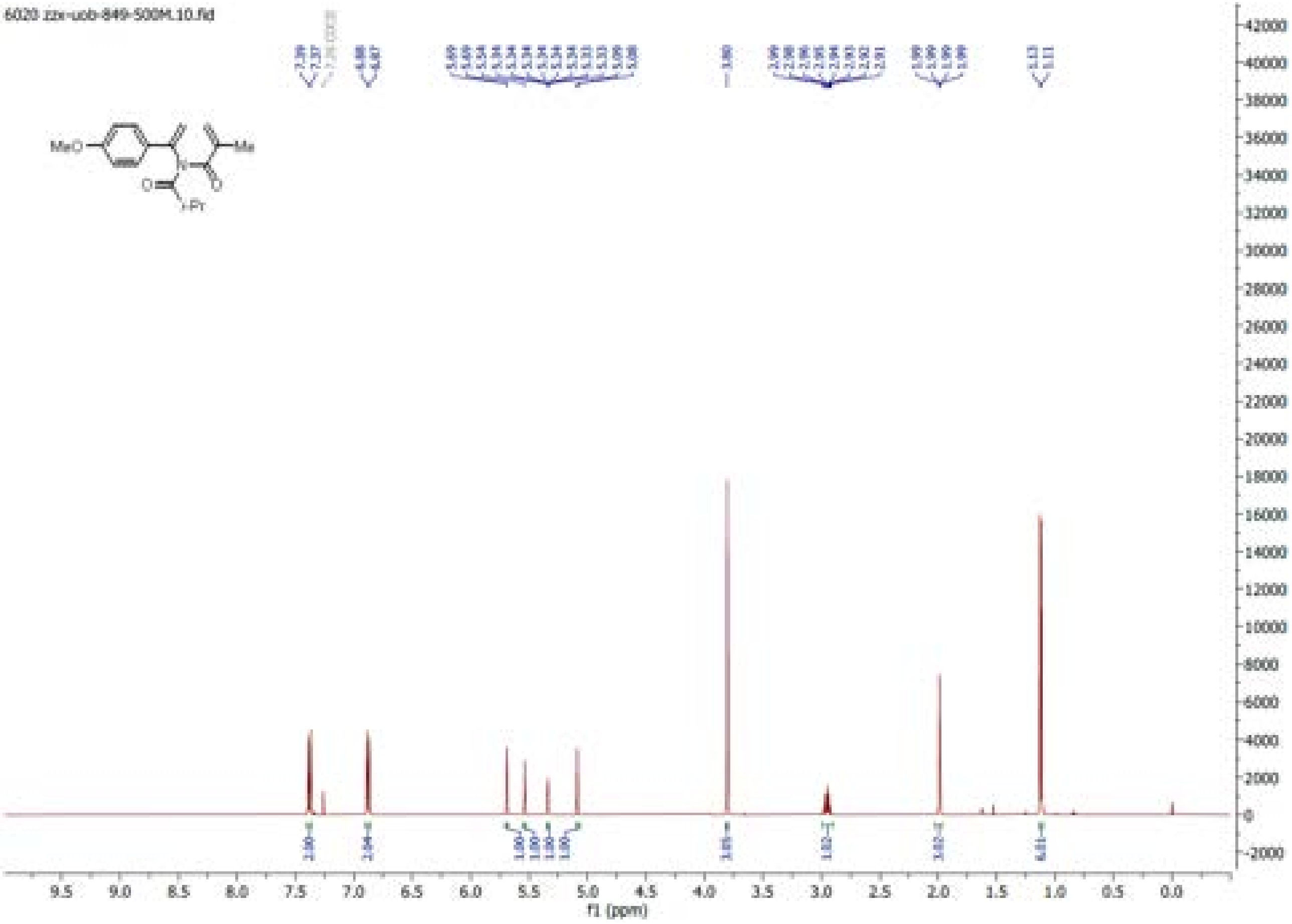

^{13}C NMR (126 MHz, $CDCl_3$) of **2x**

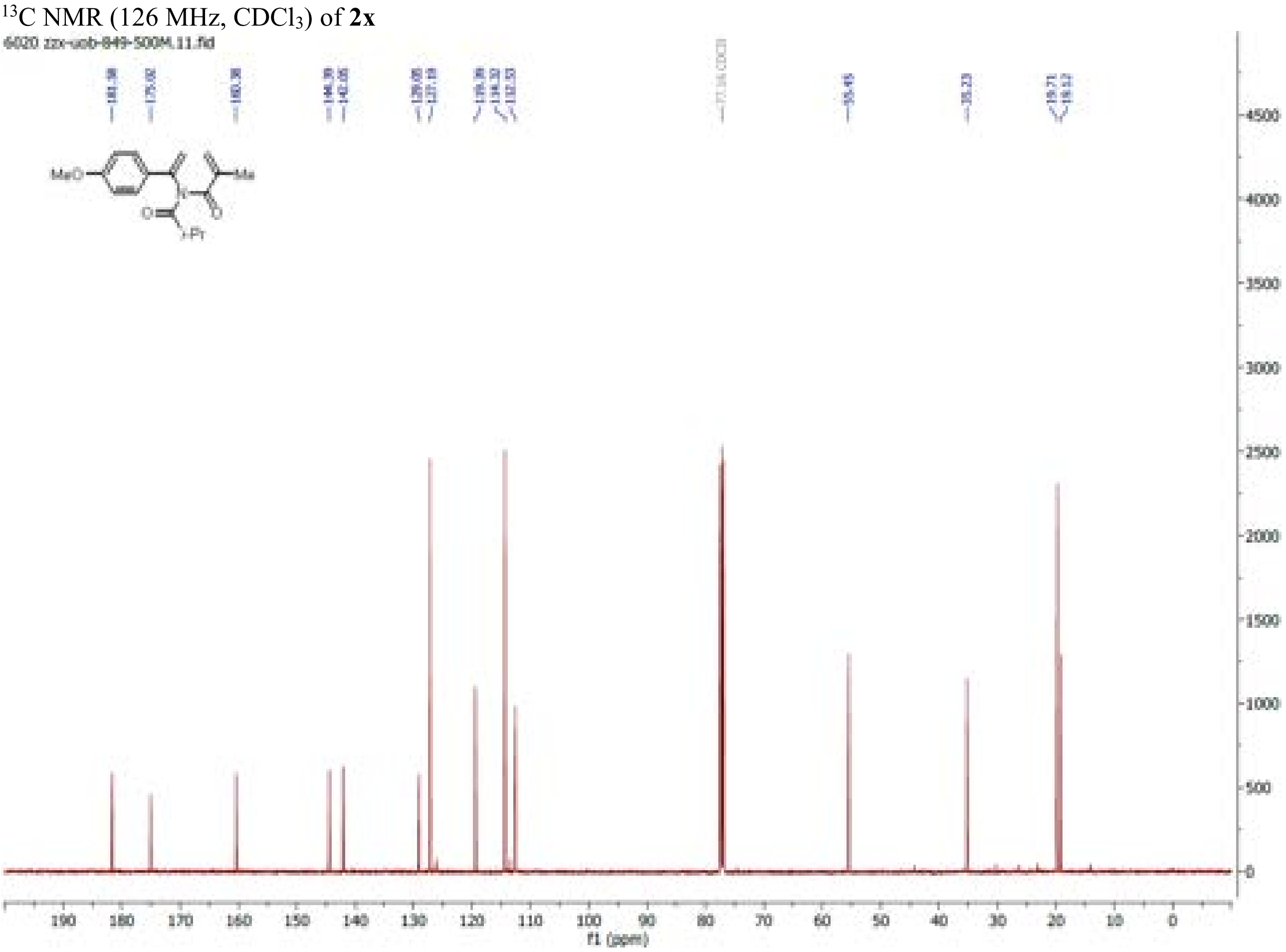

^{1}H NMR (500 MHz, $CDCl_3$) of **2y**

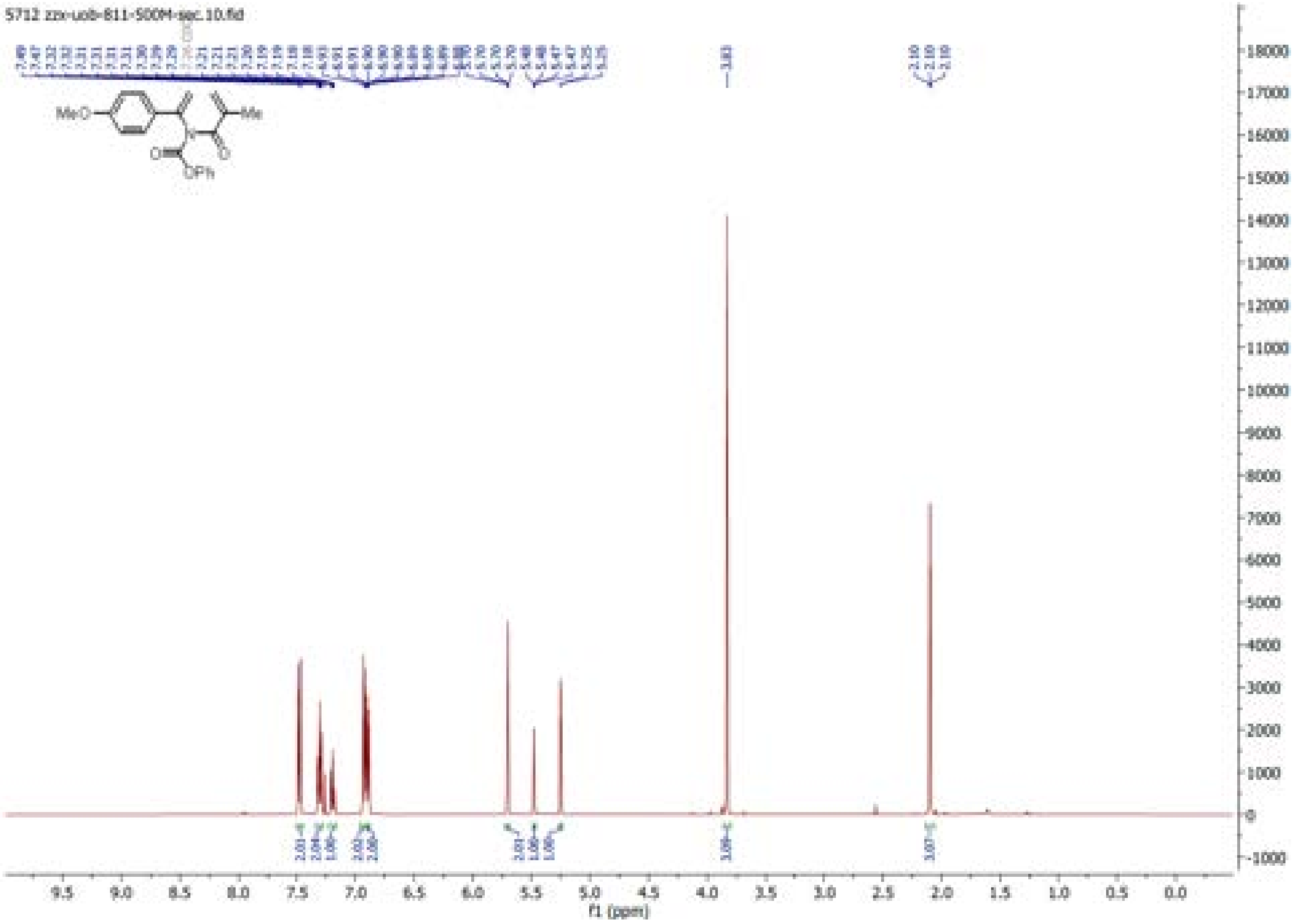

^{13}C NMR (126 MHz, $CDCl_3$) of **2y**

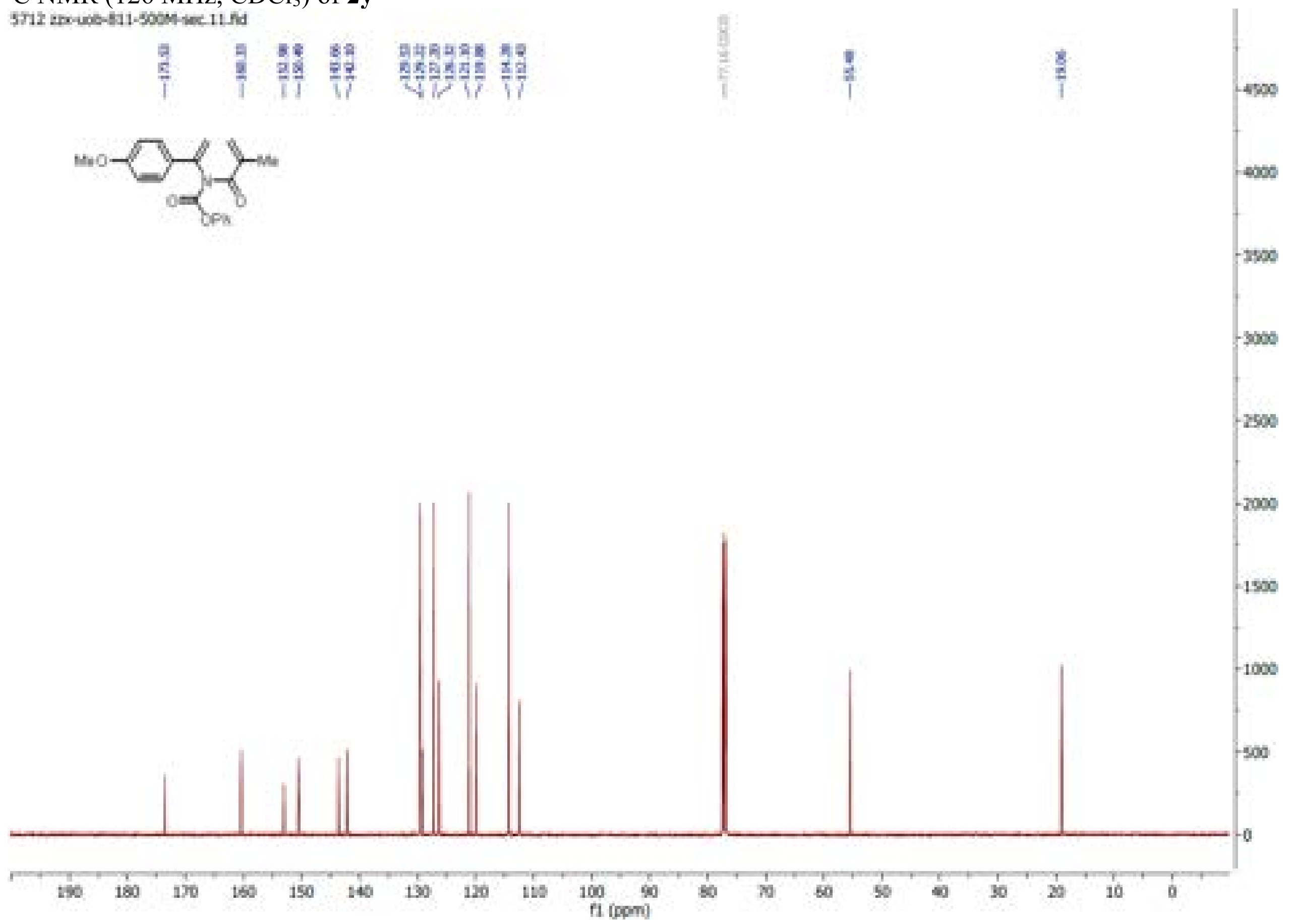

^{1}H NMR (500 MHz, $CDCl_3$) of **2z**

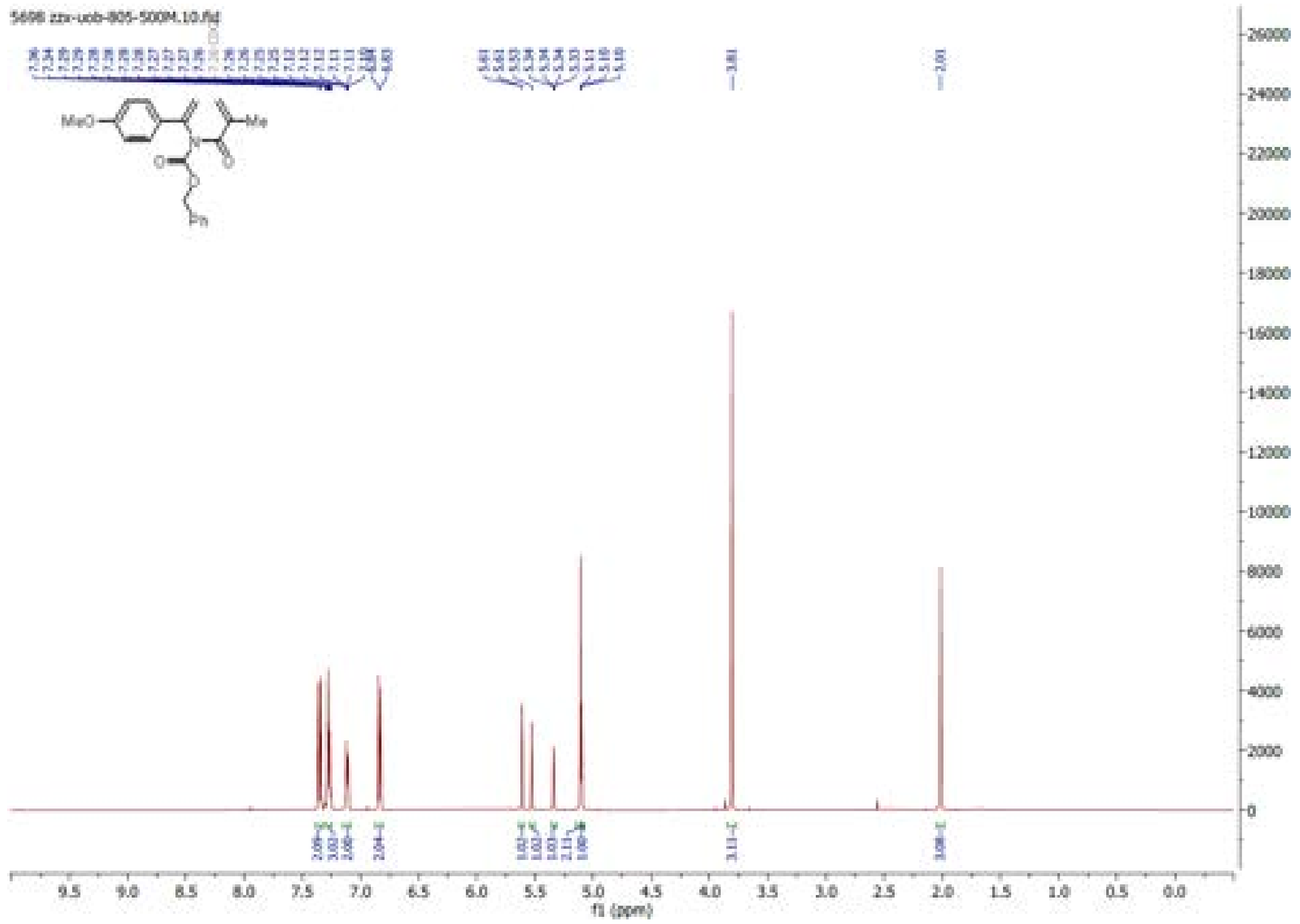

^{13}C NMR (126 MHz, $CDCl_3$) of **2z**

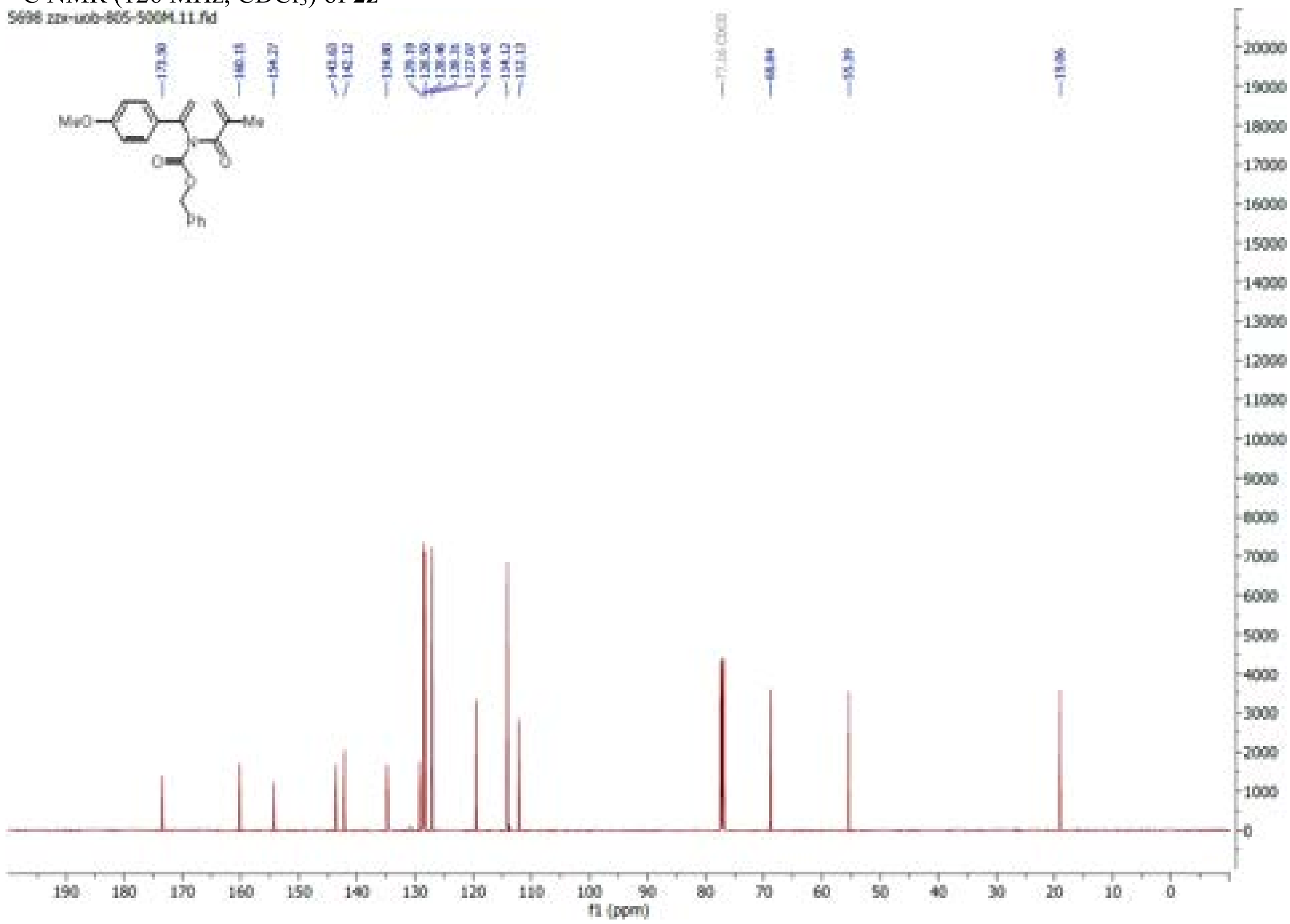

^{1}H NMR (500 MHz, $CDCl_3$) of **2aa**

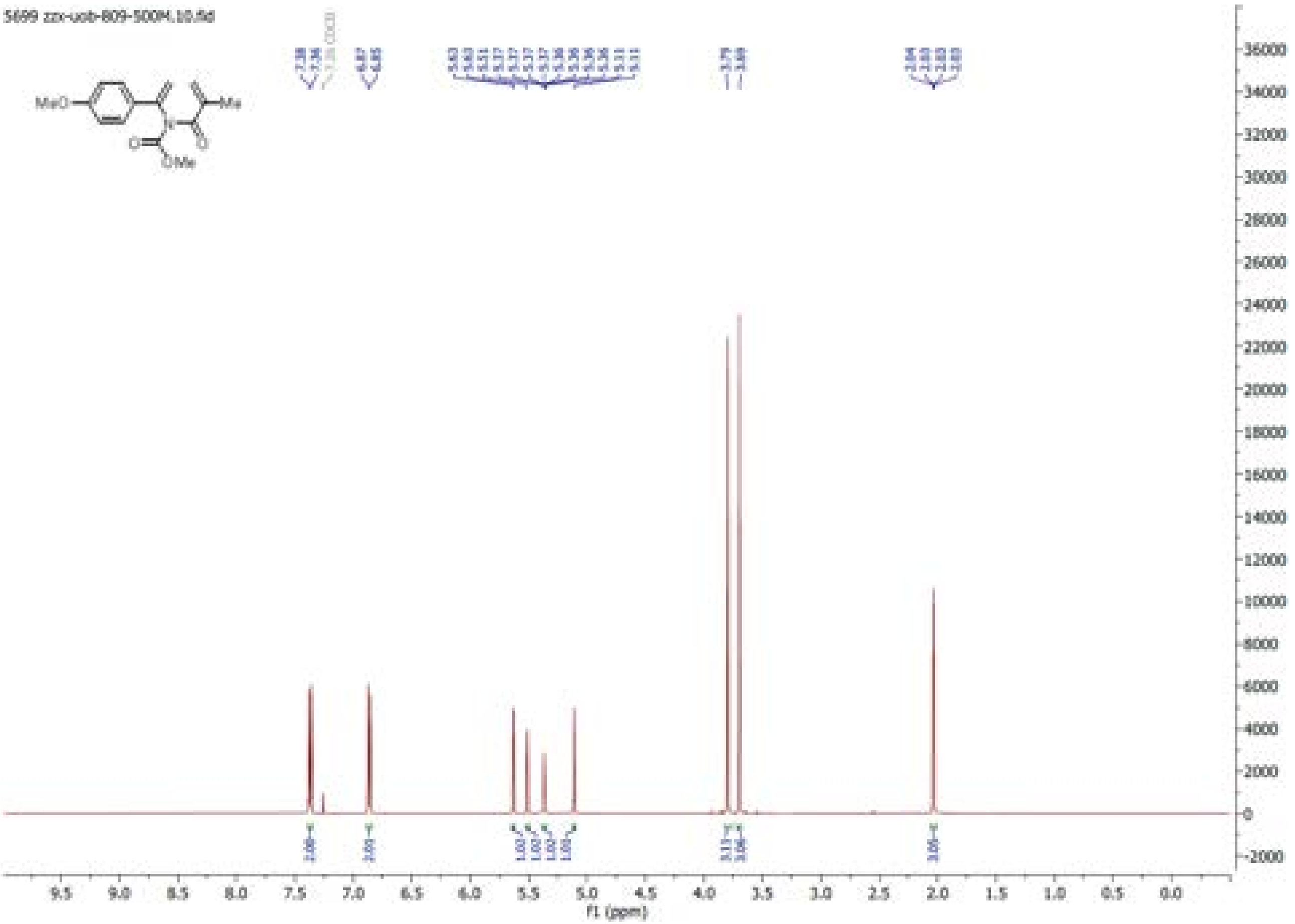

^{13}C NMR (126 MHz, $CDCl_3$) of **2aa**

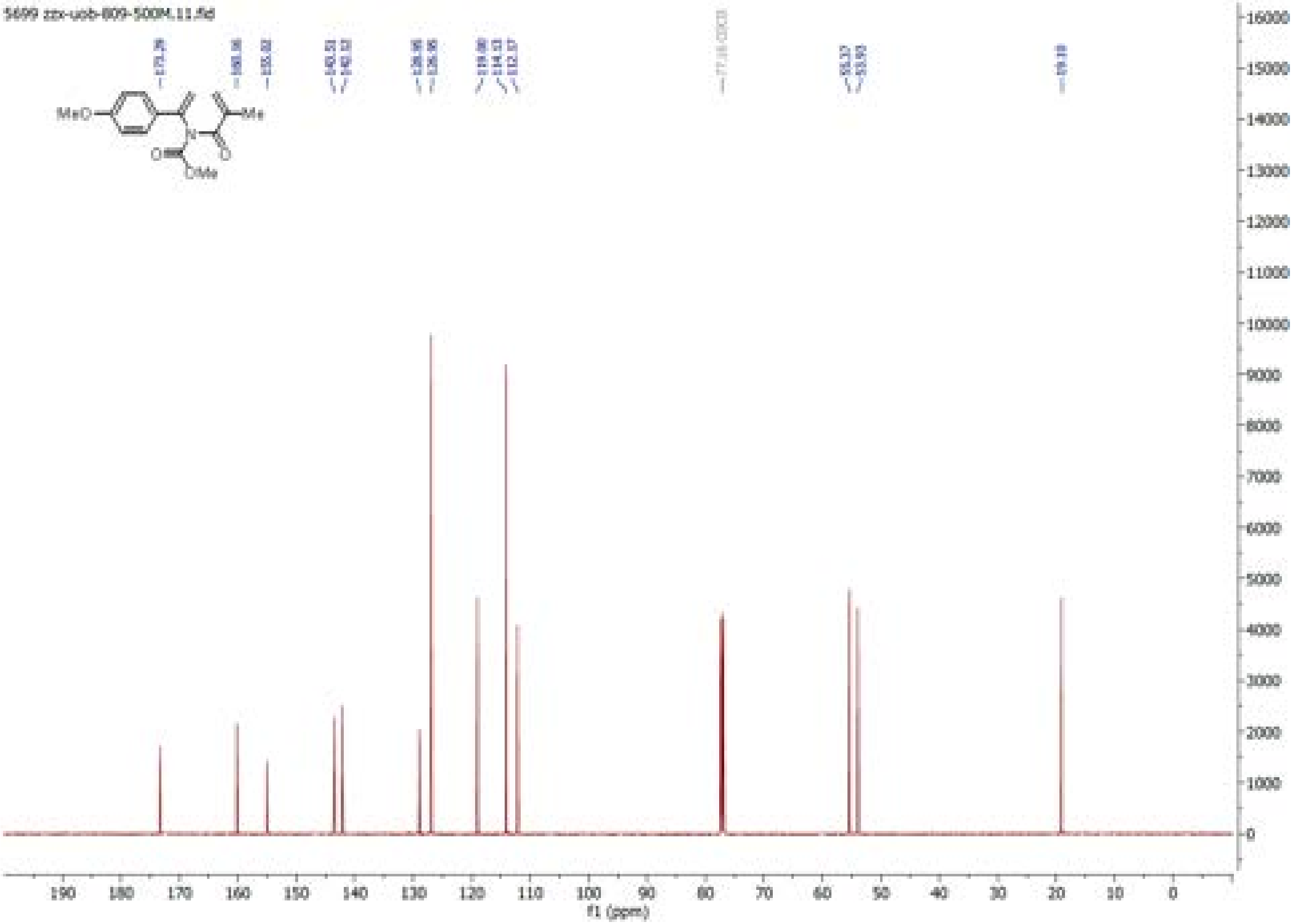

^{1}H NMR (500 MHz, $CDCl_3$) of **2ab**

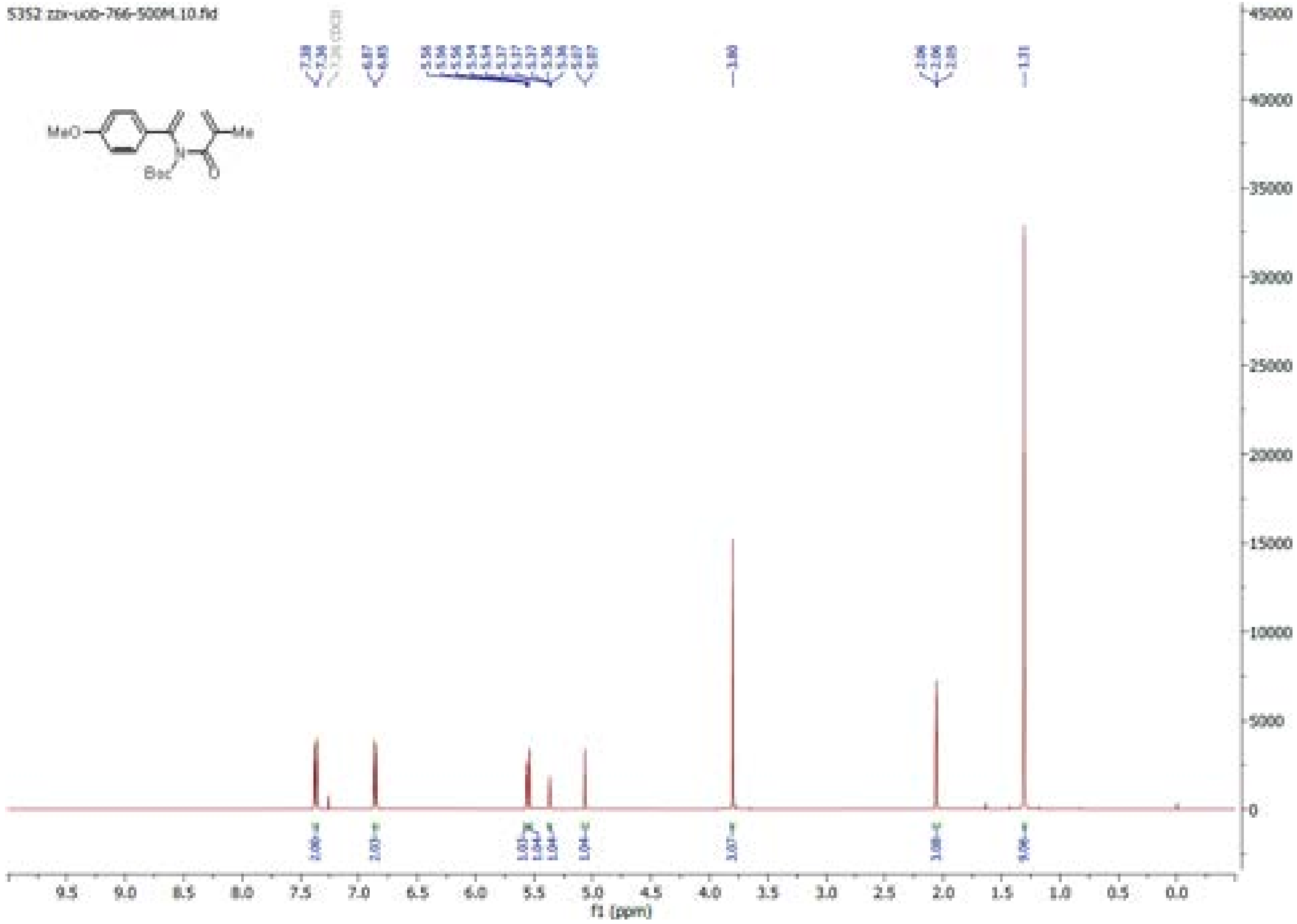

^{13}C NMR (126 MHz, $CDCl_3$) of **2ab**

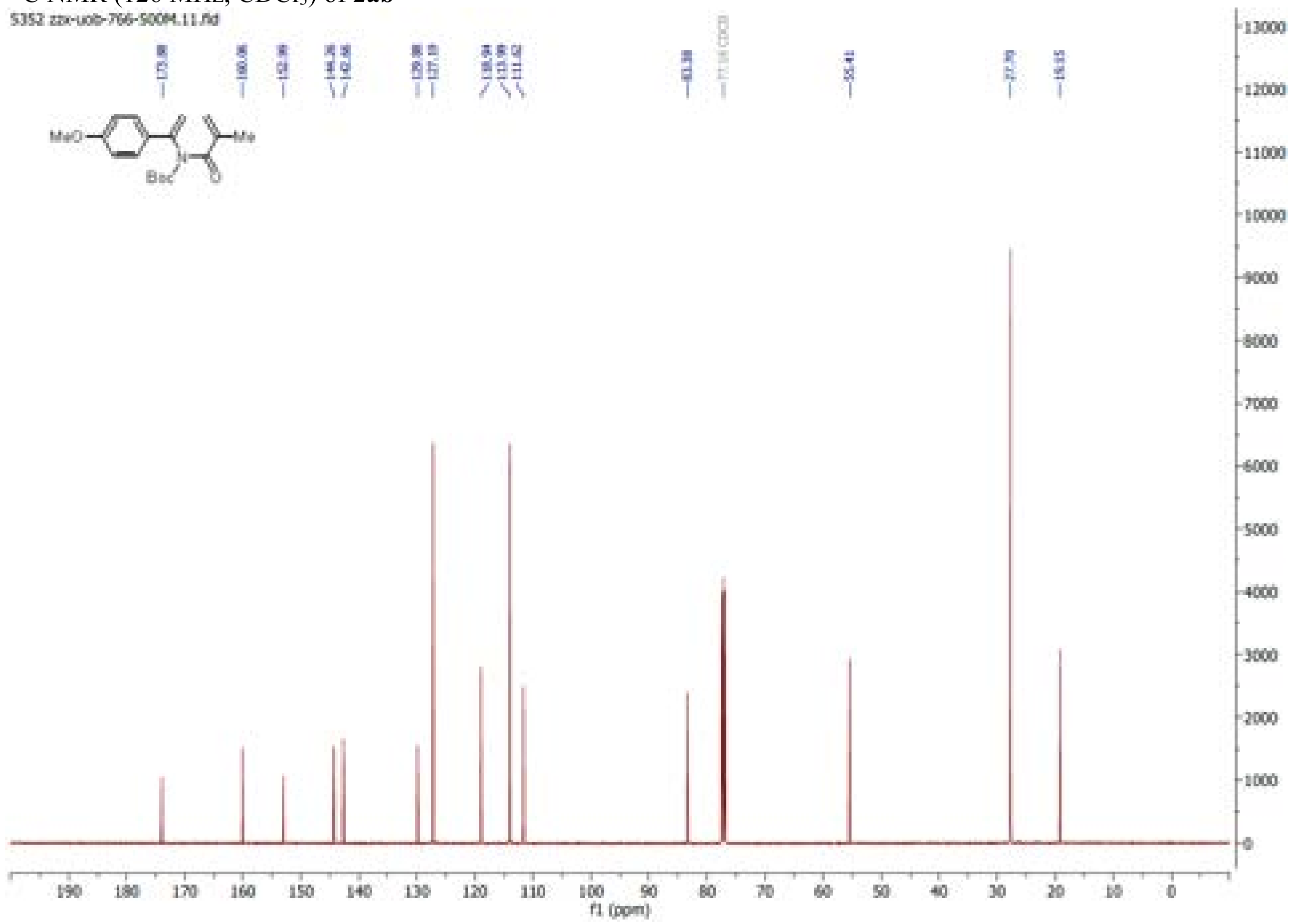

^{1}H NMR (500 MHz, $CDCl_3$) of **2ac**

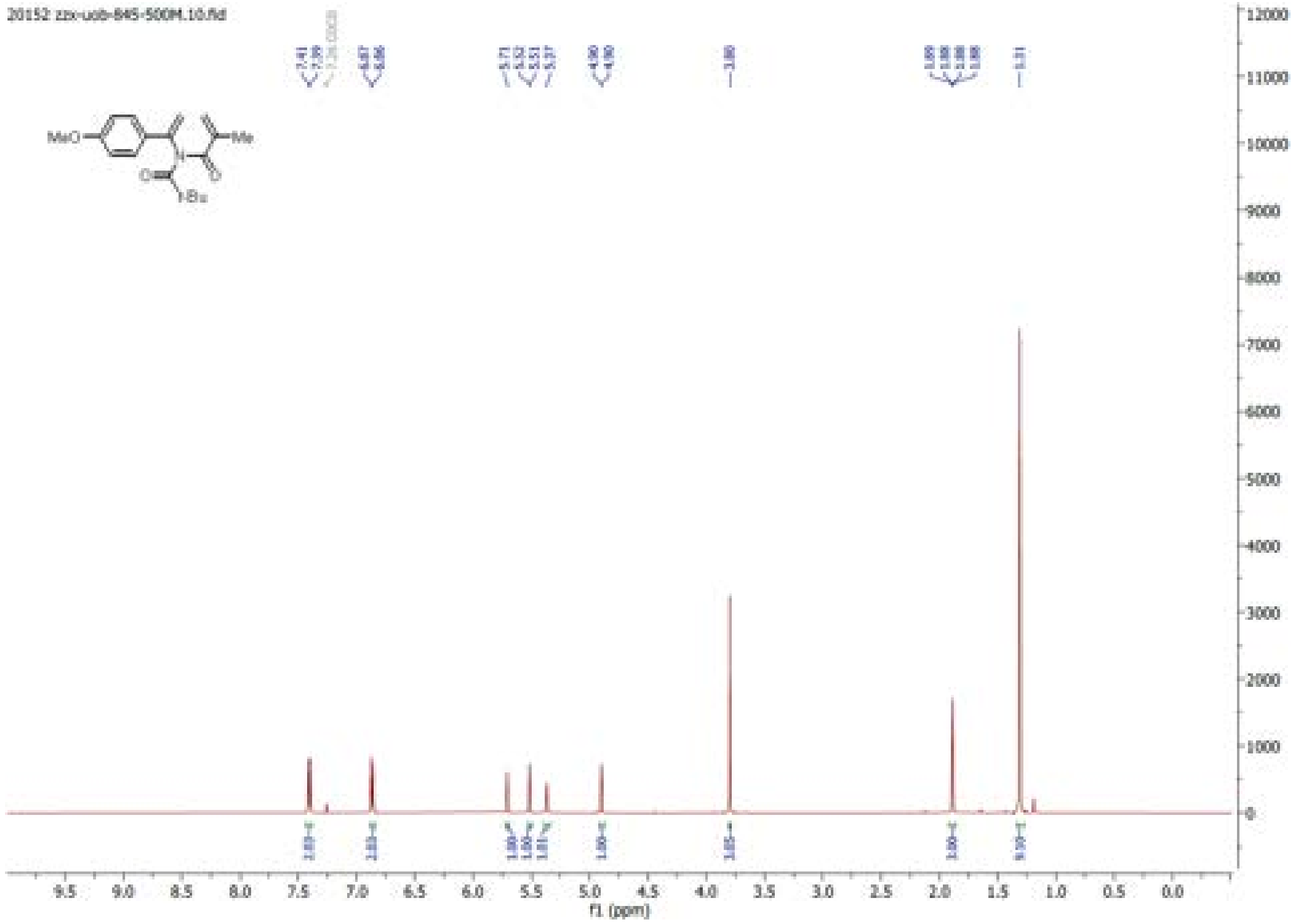

^{13}C NMR (126 MHz, $CDCl_3$) of **2ac**

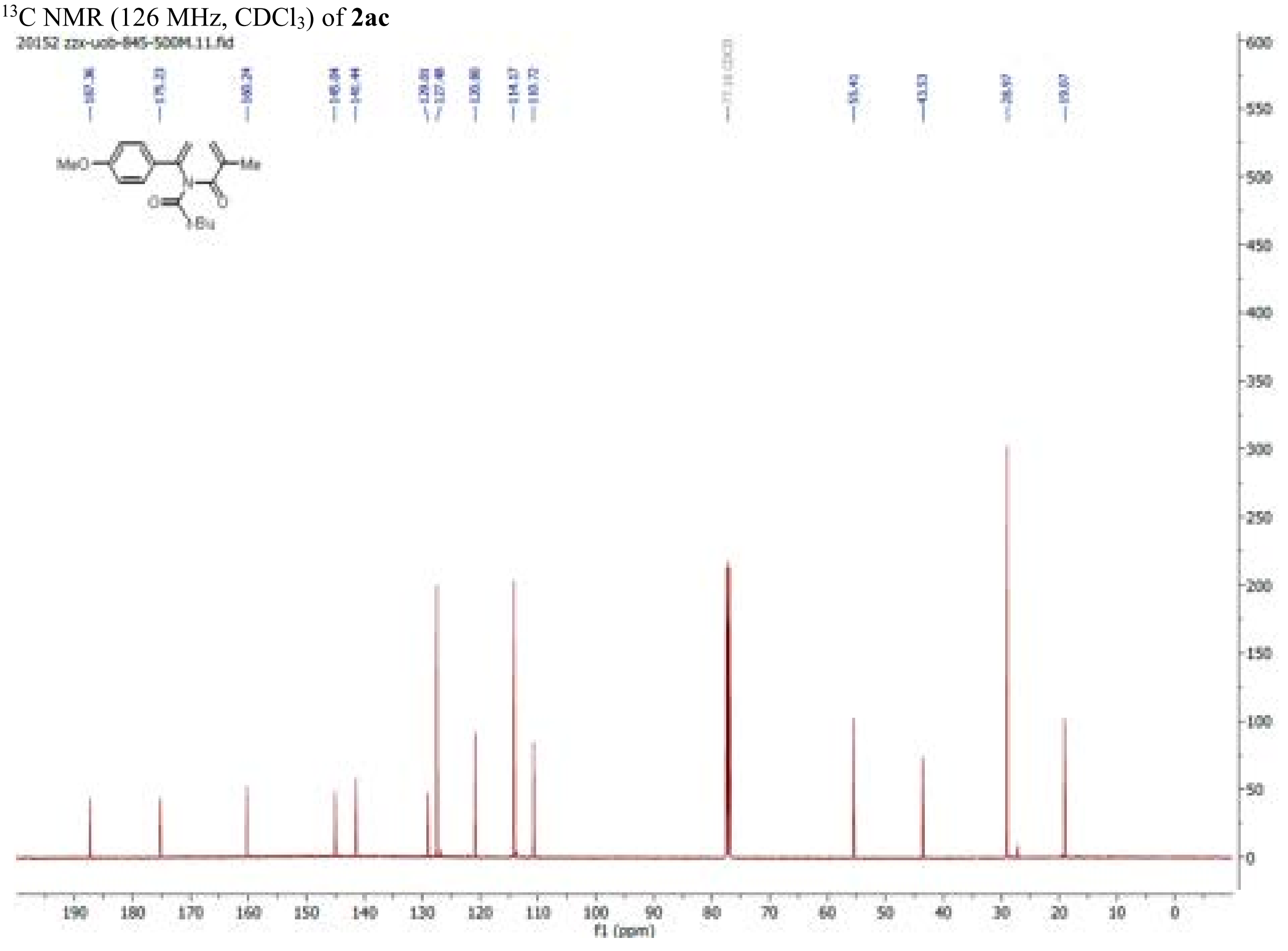

^{1}H NMR (500 MHz, $CDCl_3$) of **2ad**

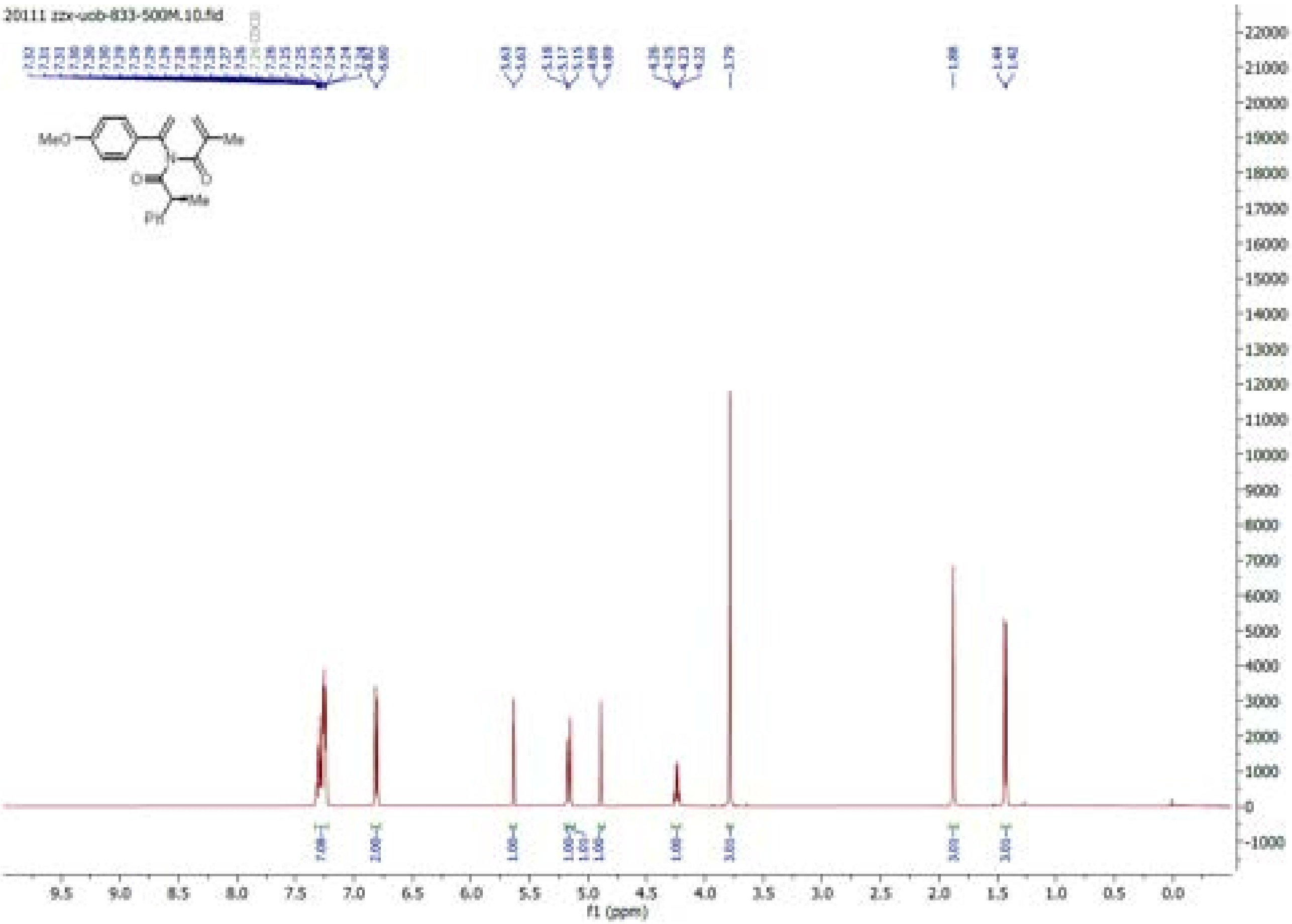

^{13}C NMR (126 MHz, $CDCl_3$) of **2ad**

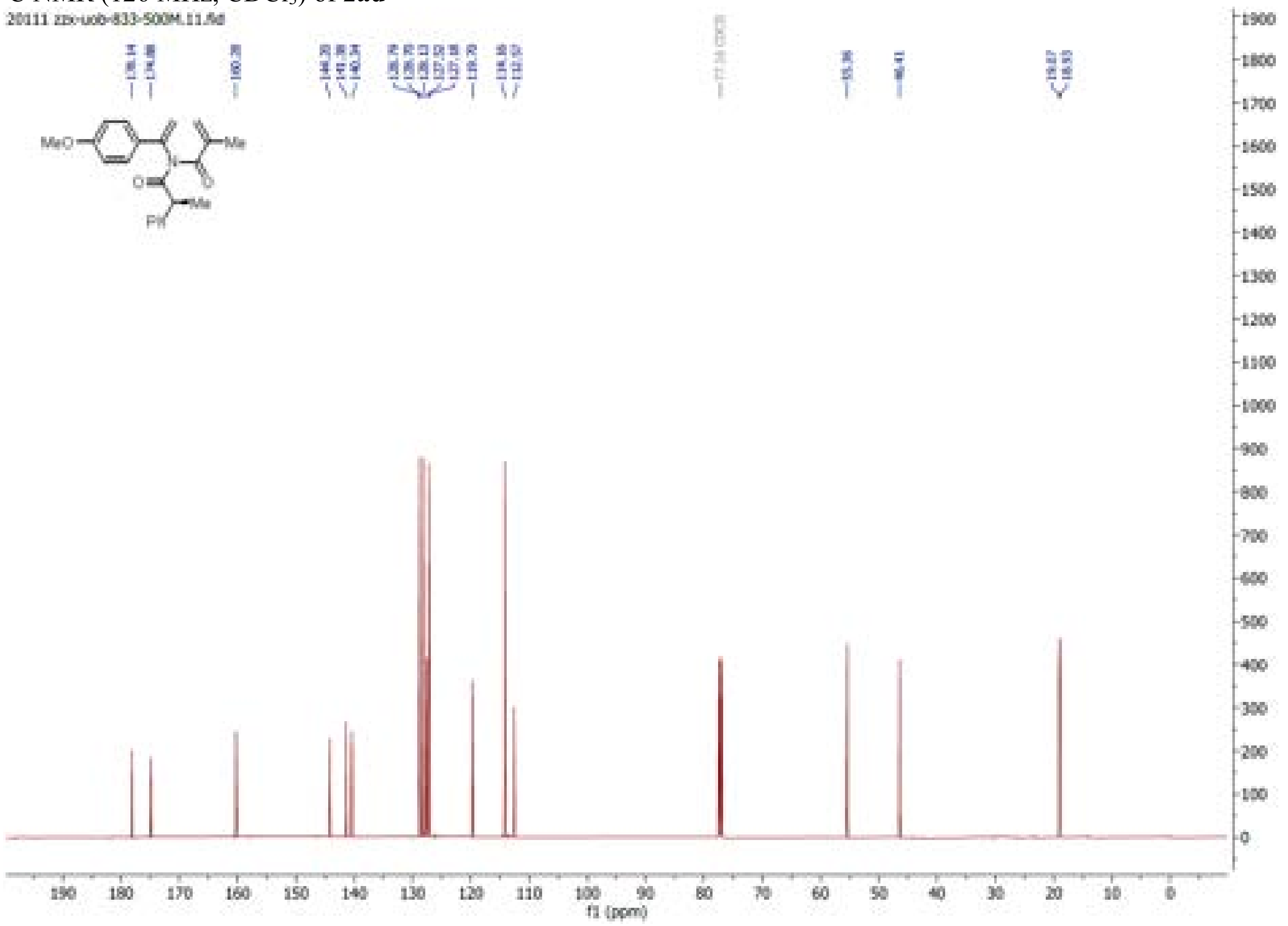

^{1}H NMR (500 MHz, $CDCl_3$) of **2ae**

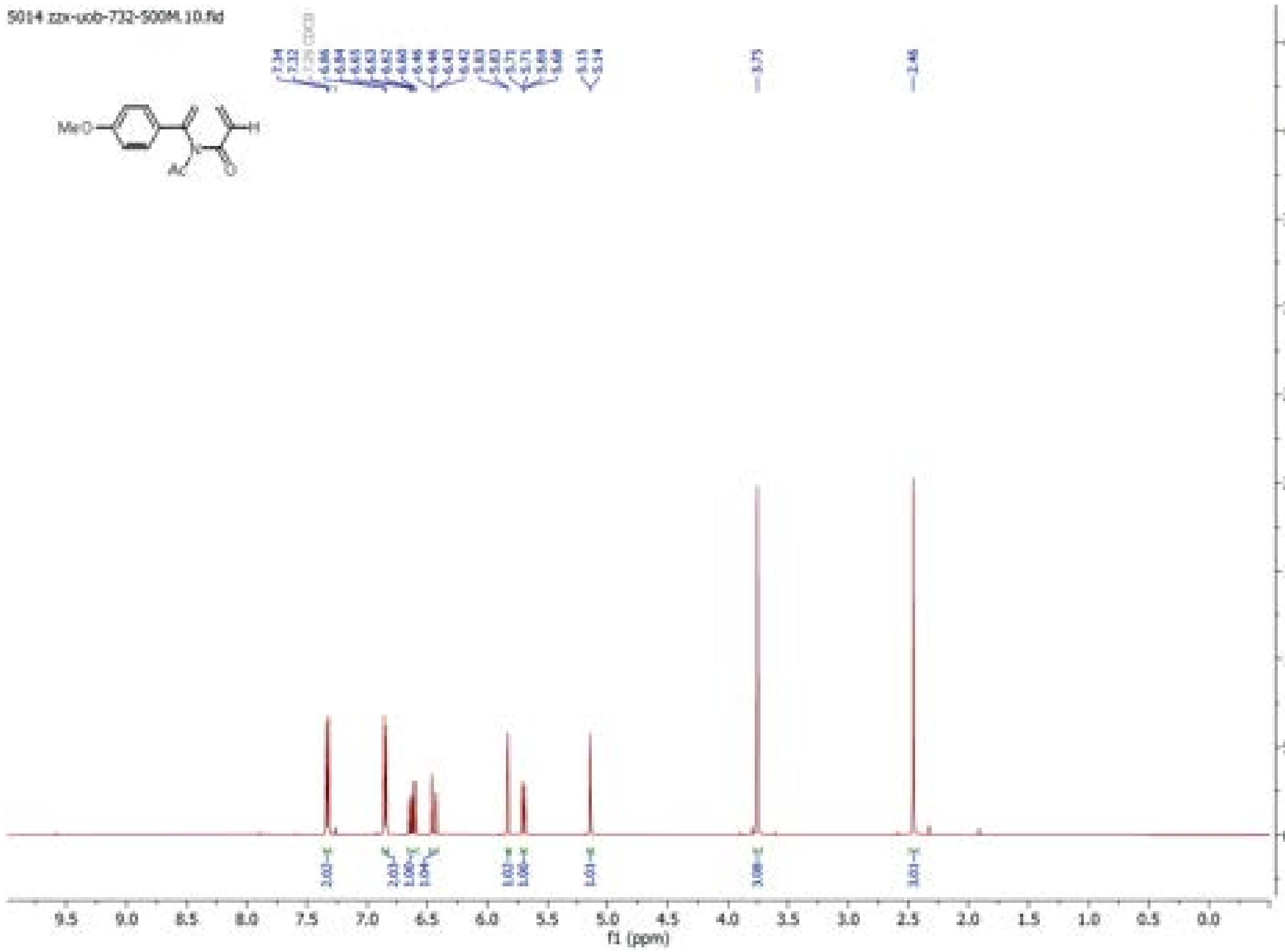

^{13}C NMR (126 MHz, $CDCl_3$) of **2ae**

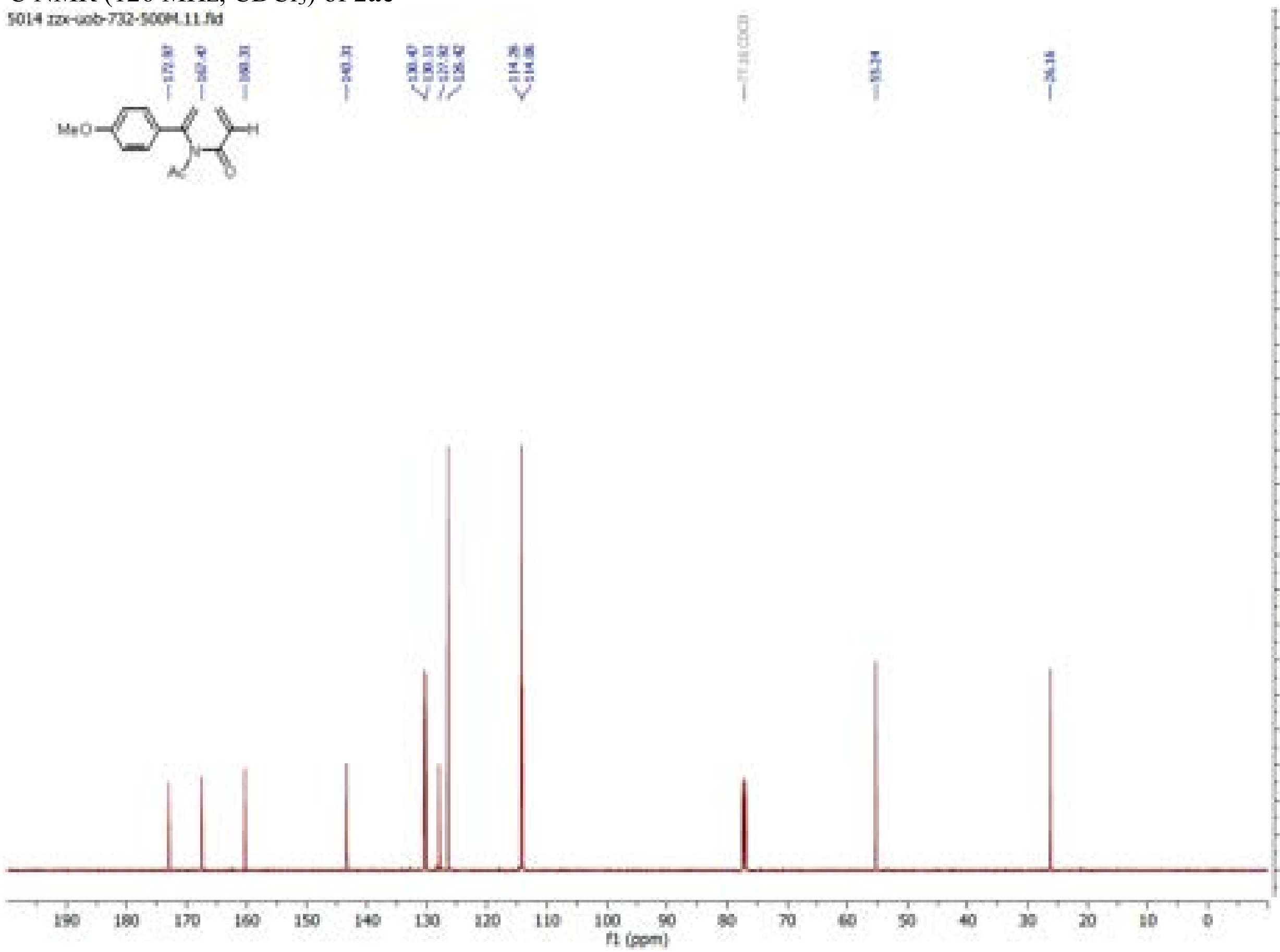

^{1}H NMR (500 MHz, $CDCl_3$) of **2af**

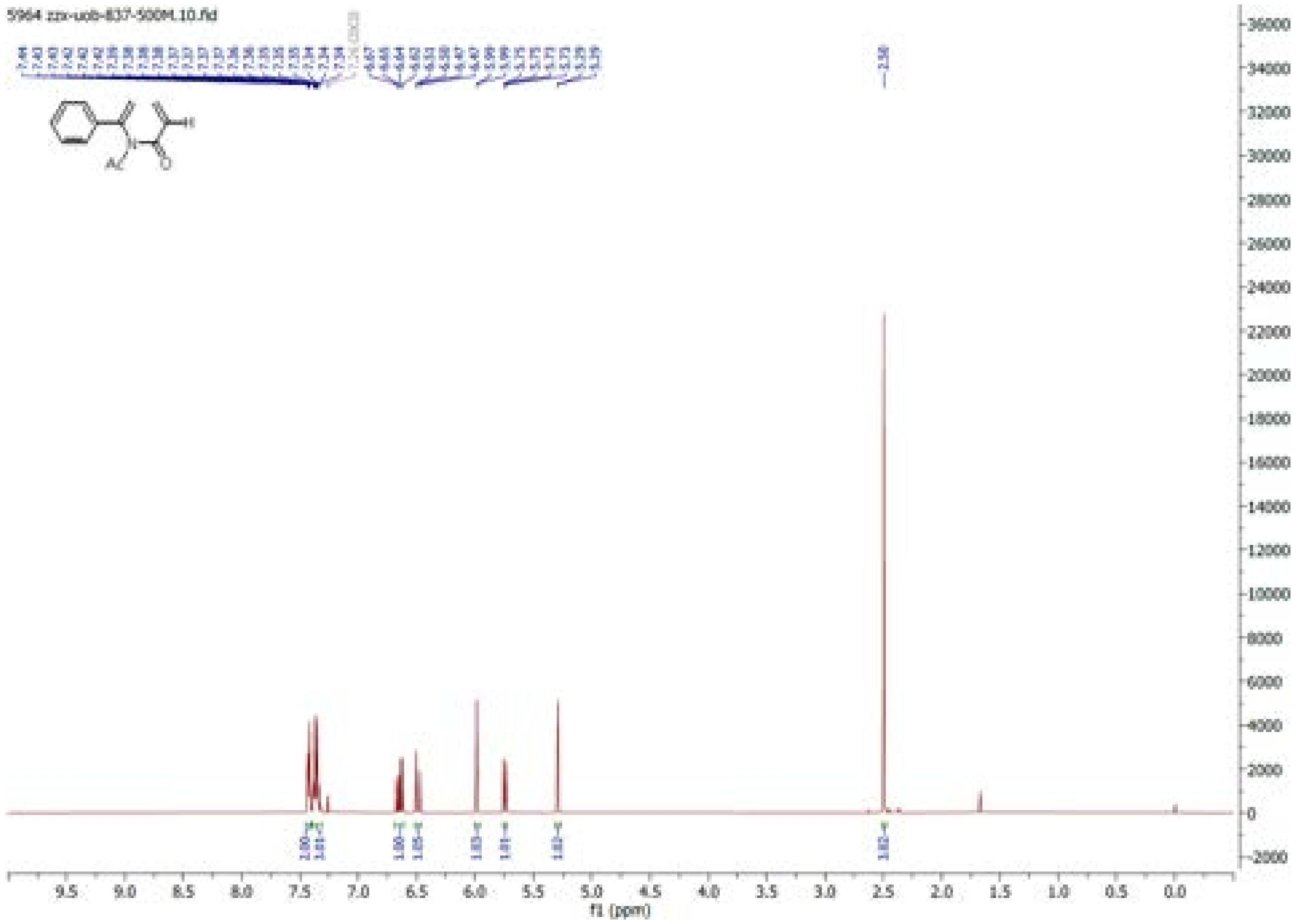

^{13}C NMR (126 MHz, $CDCl_3$) of **2af**

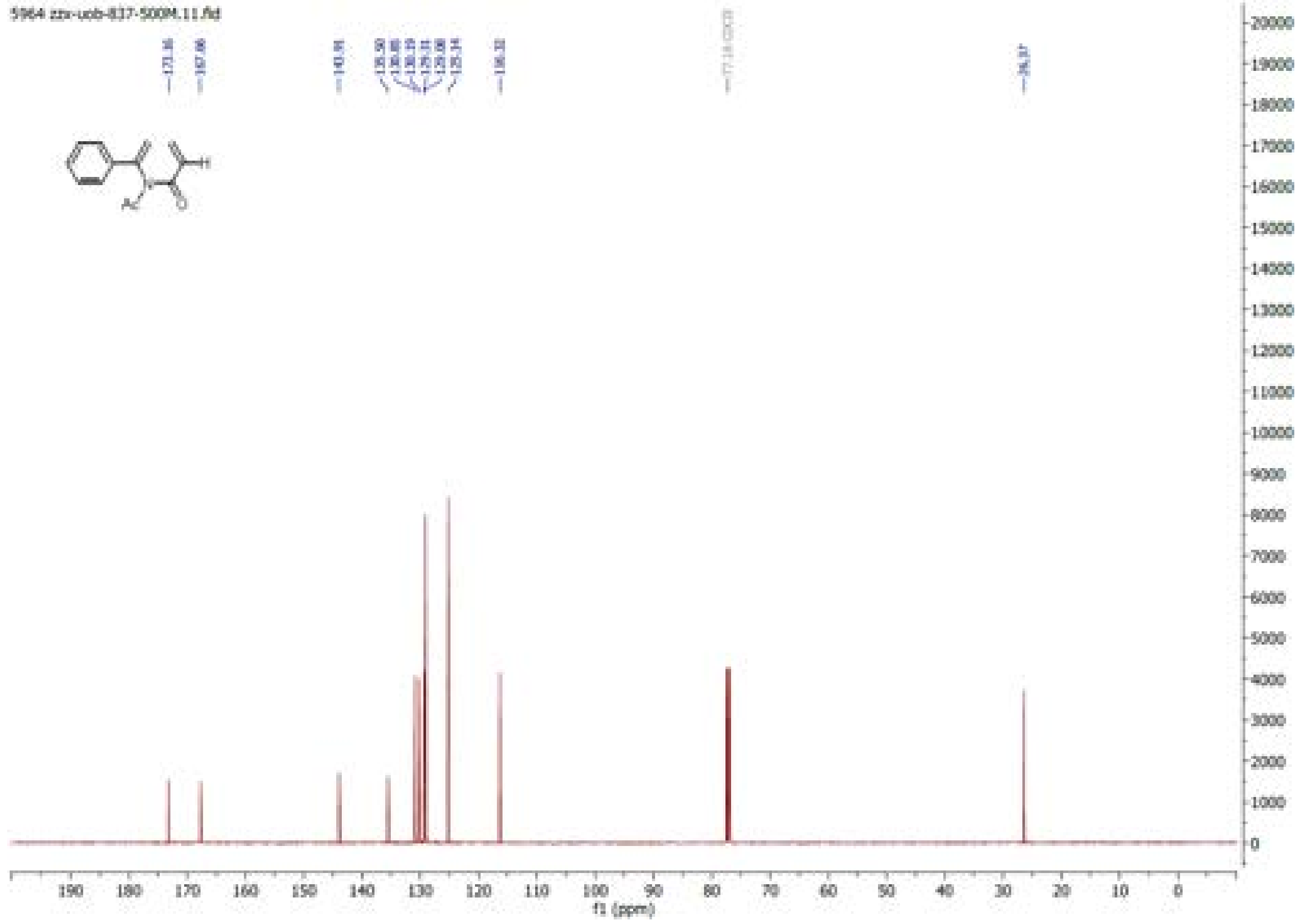

^{1}H NMR (500 MHz, $CDCl_3$) of **2ag**

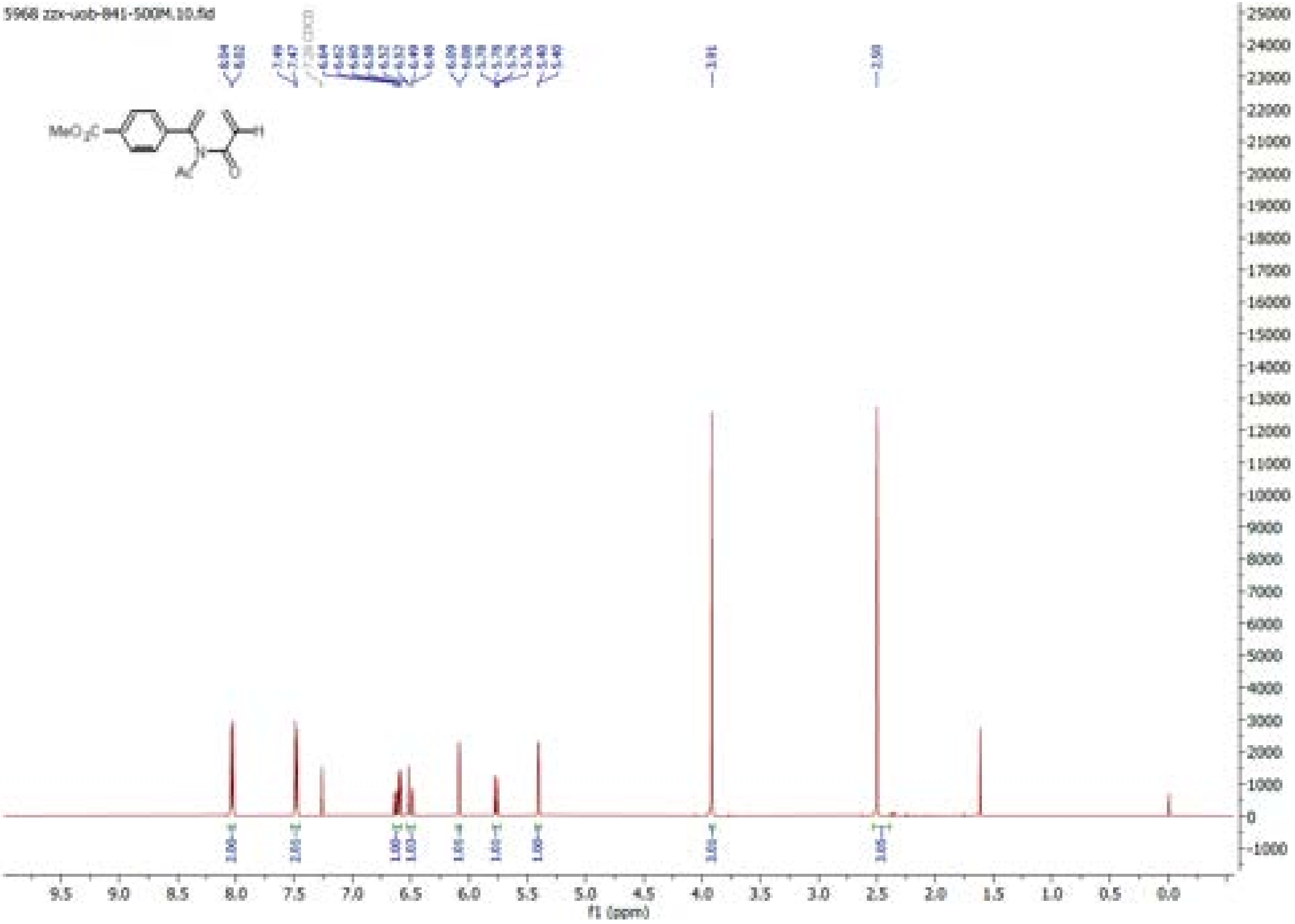

^{13}C NMR (126 MHz, $CDCl_3$) of **2ag**

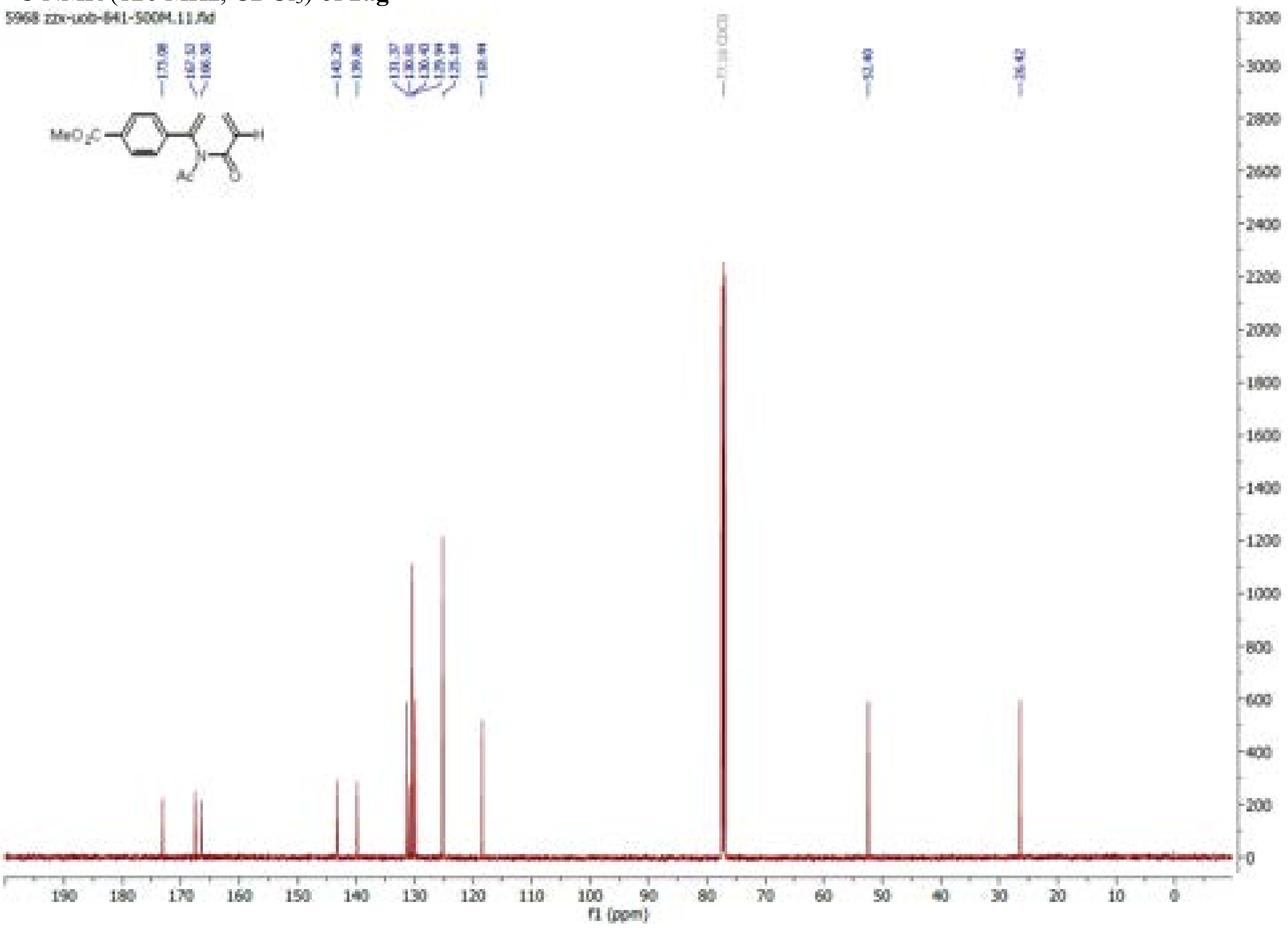

^{1}H NMR (500 MHz, $CDCl_3$) of **2ah**

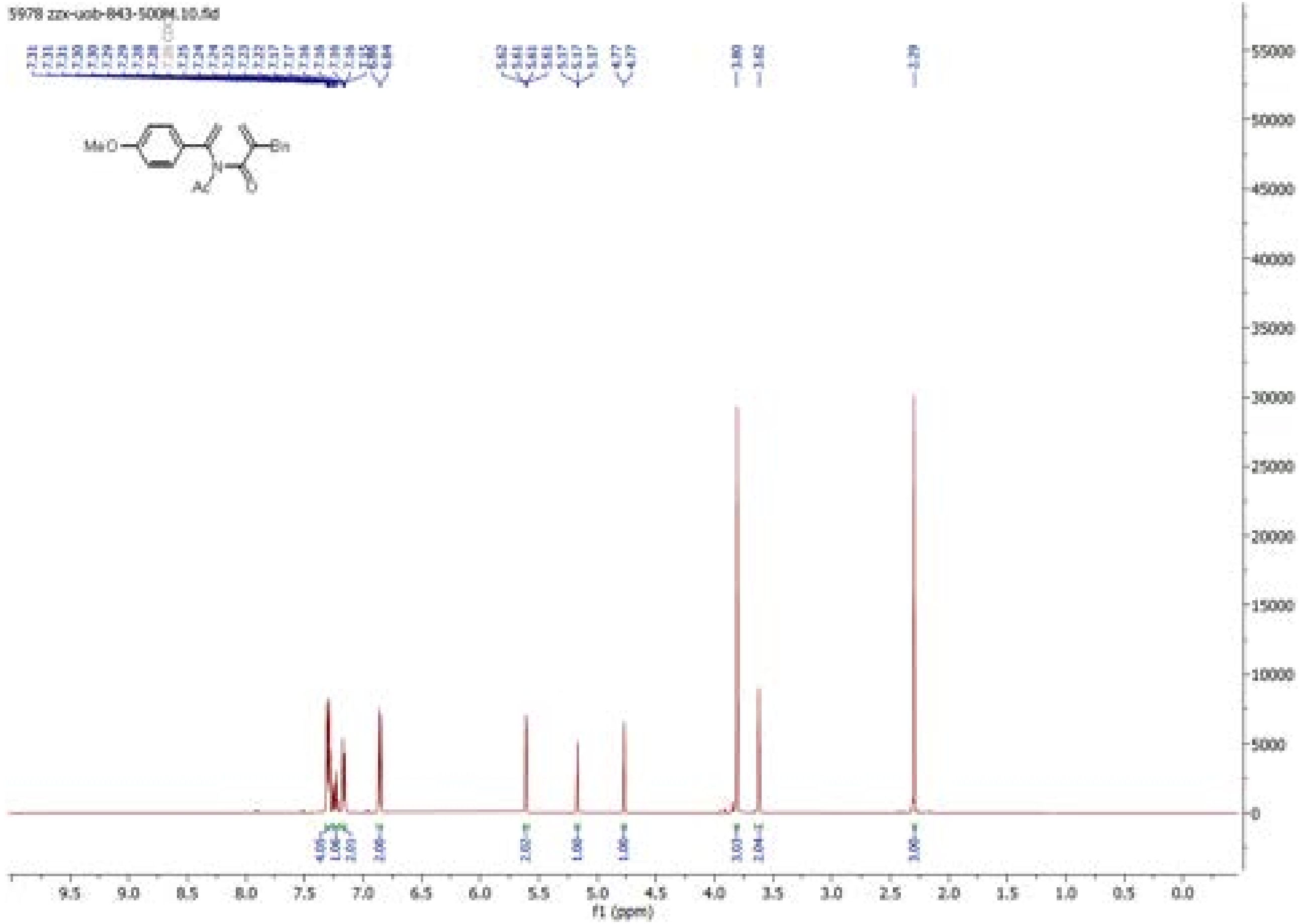

^{13}C NMR (126 MHz, $CDCl_3$) of **2ah**

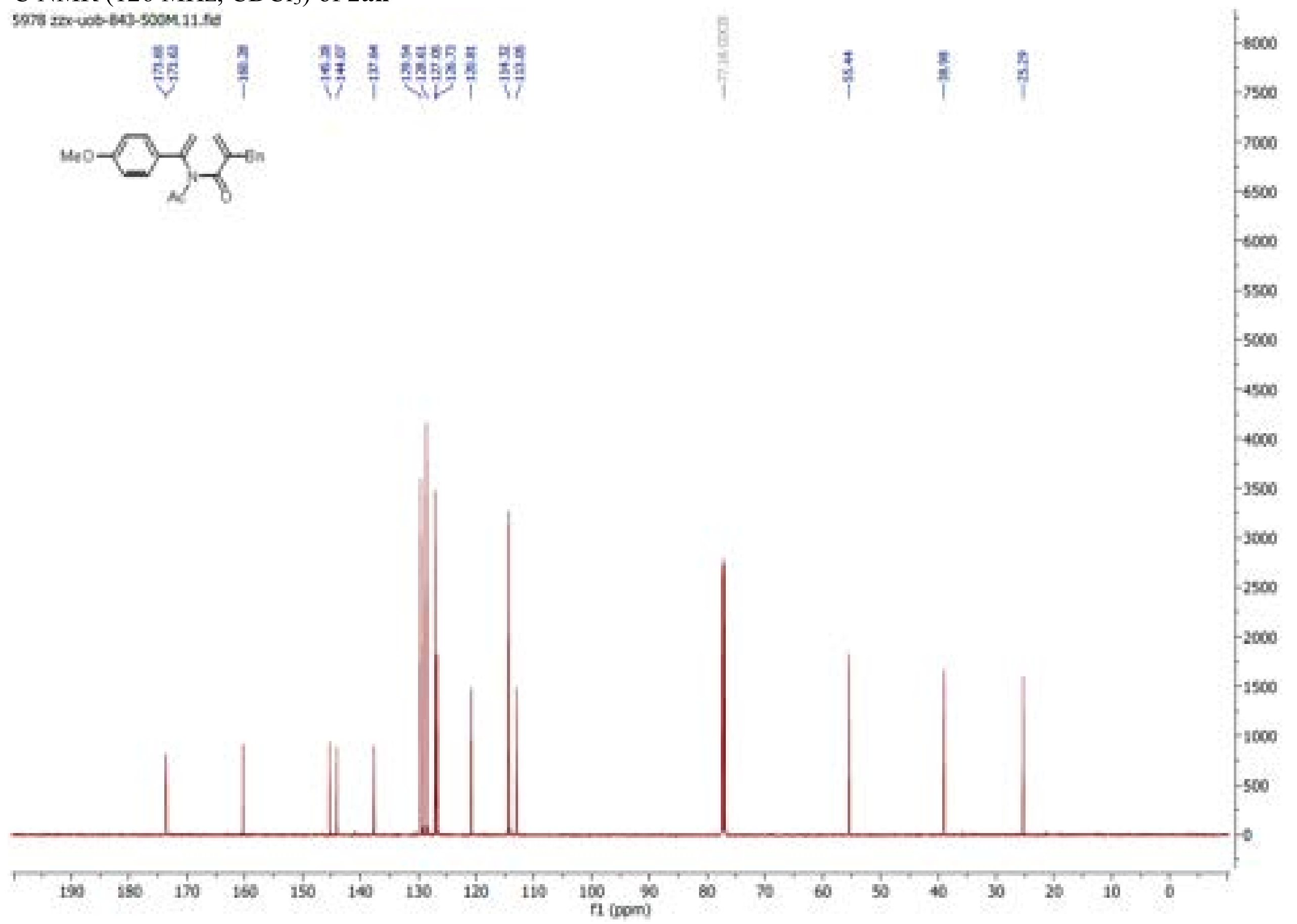

^{1}H NMR (500 MHz, $CDCl_3$) of **2ai**

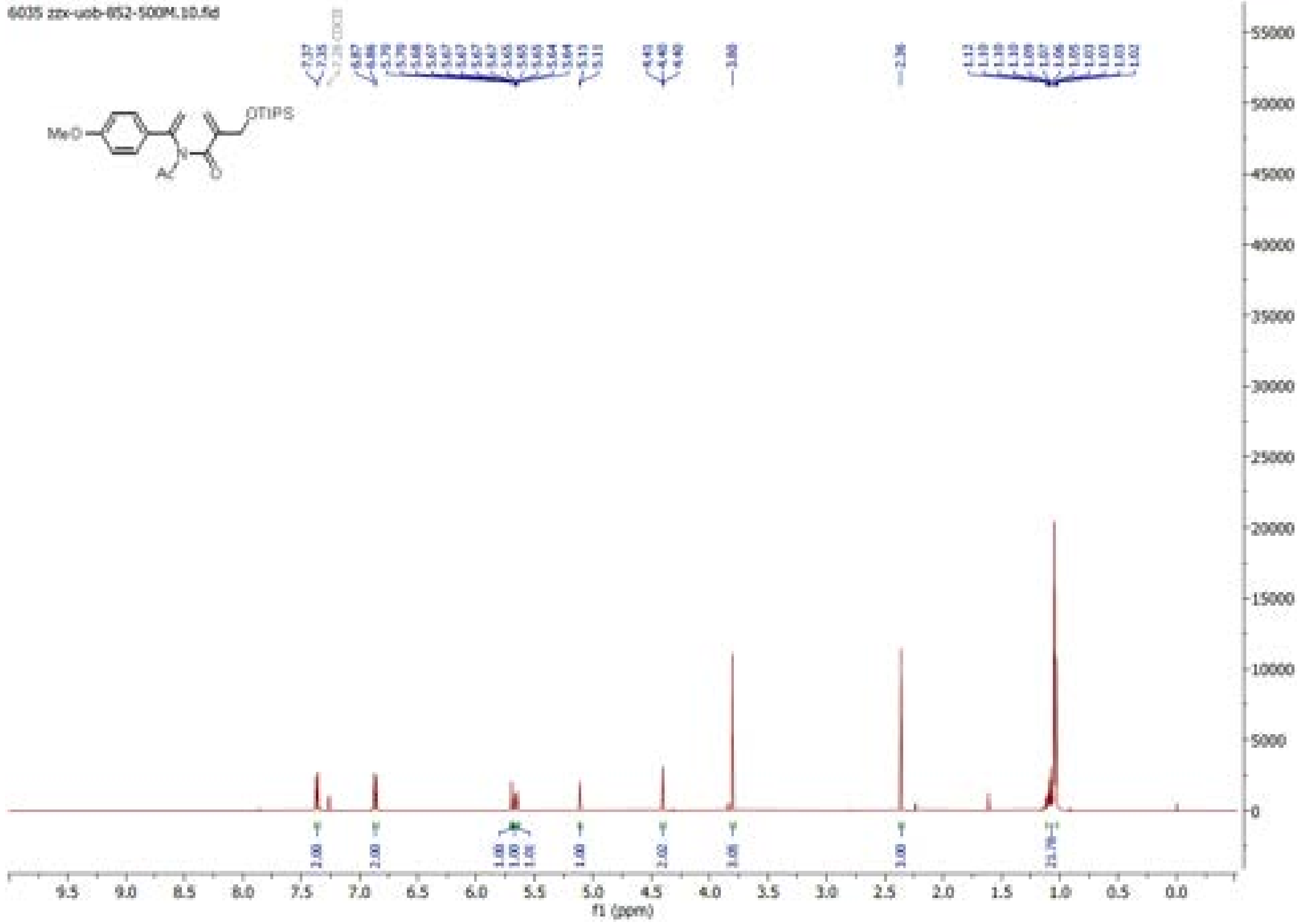

^{13}C NMR (126 MHz, $CDCl_3$) of **2ai**

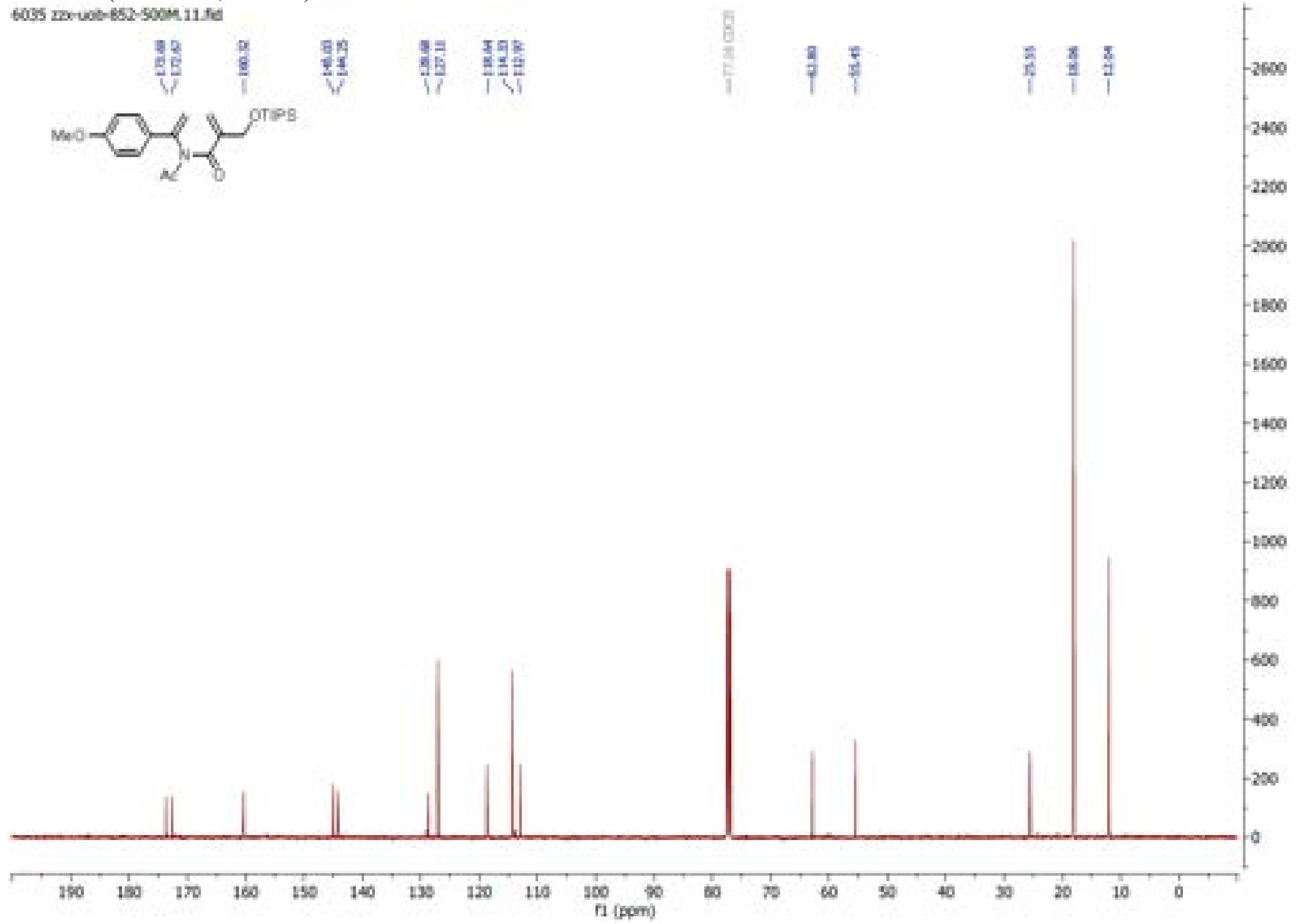

^{1}H NMR (500 MHz, $CDCl_3$) of **2aj**

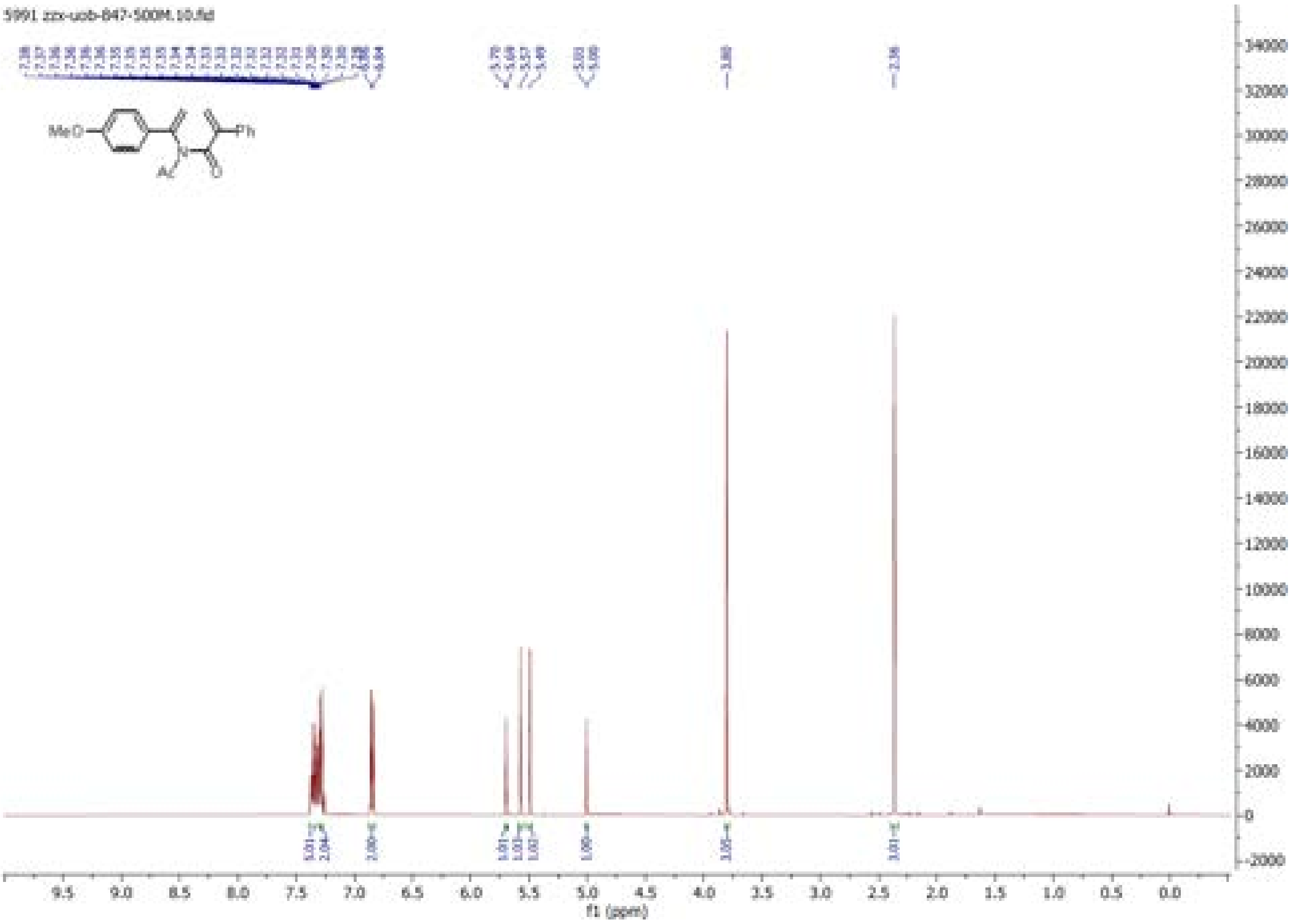

^{13}C NMR (126 MHz, $CDCl_3$) of **2aj**

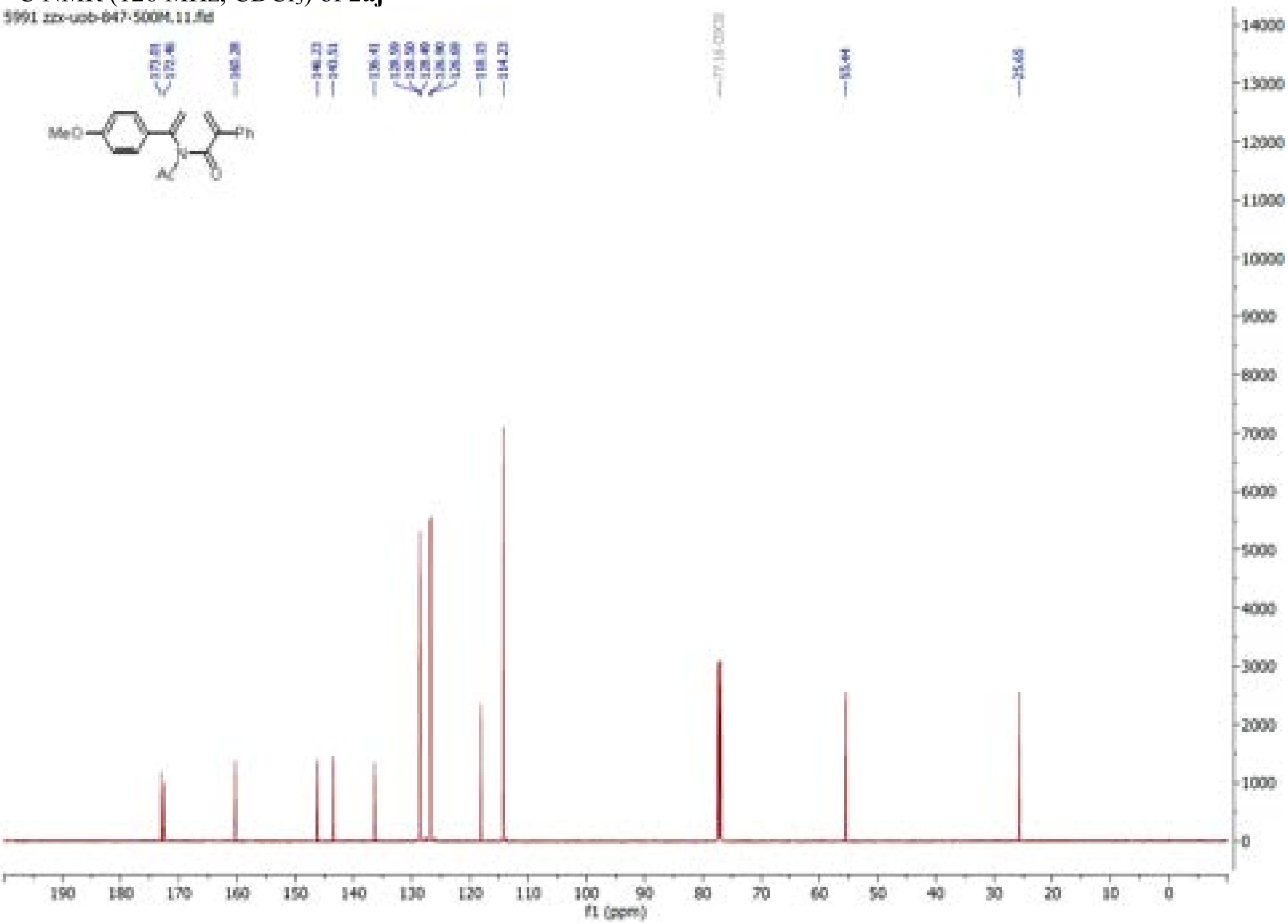

^{1}H NMR (500 MHz, $CDCl_3$) of **2ak**

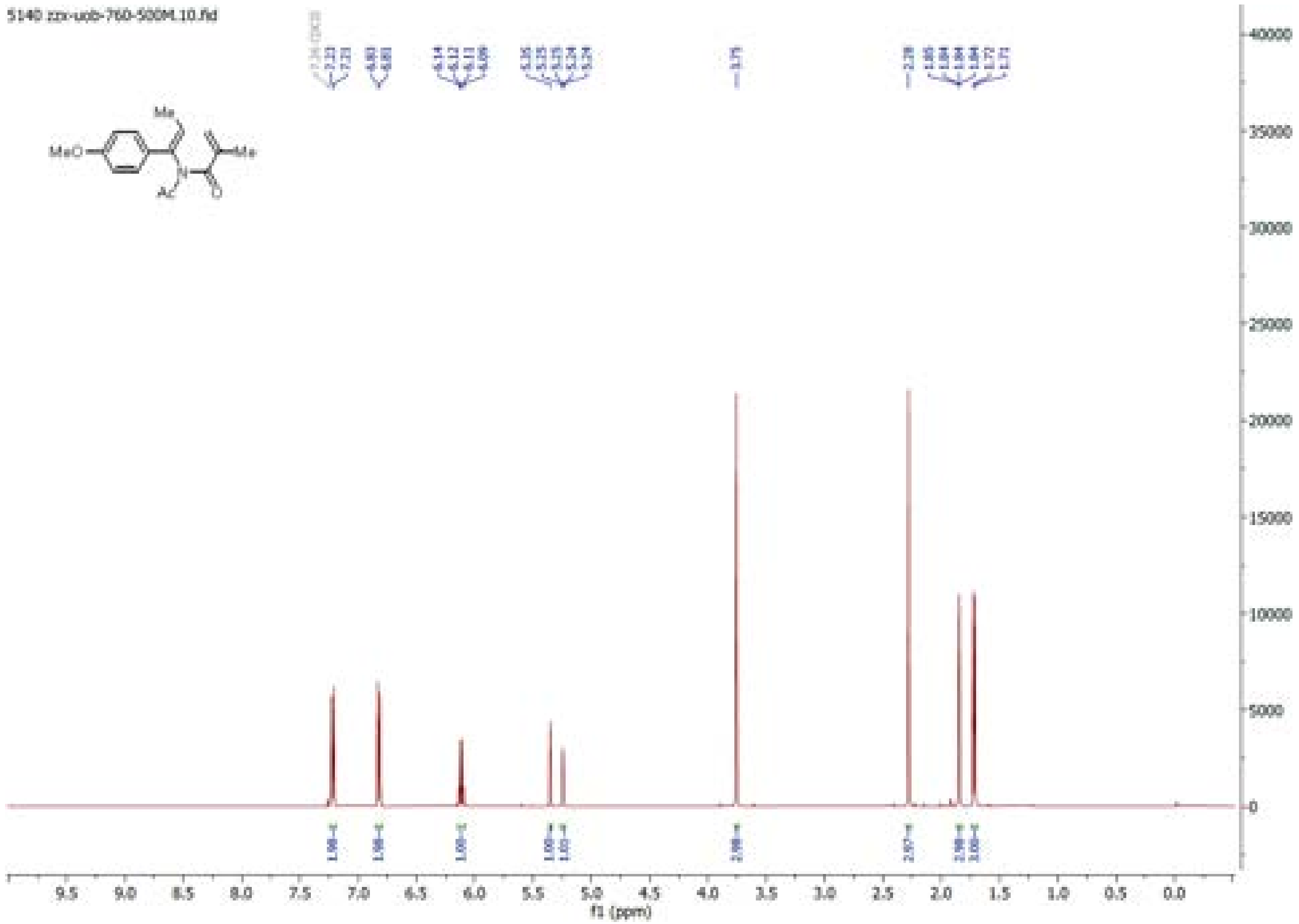

^{13}C NMR (126 MHz, $CDCl_3$) of **2ak**

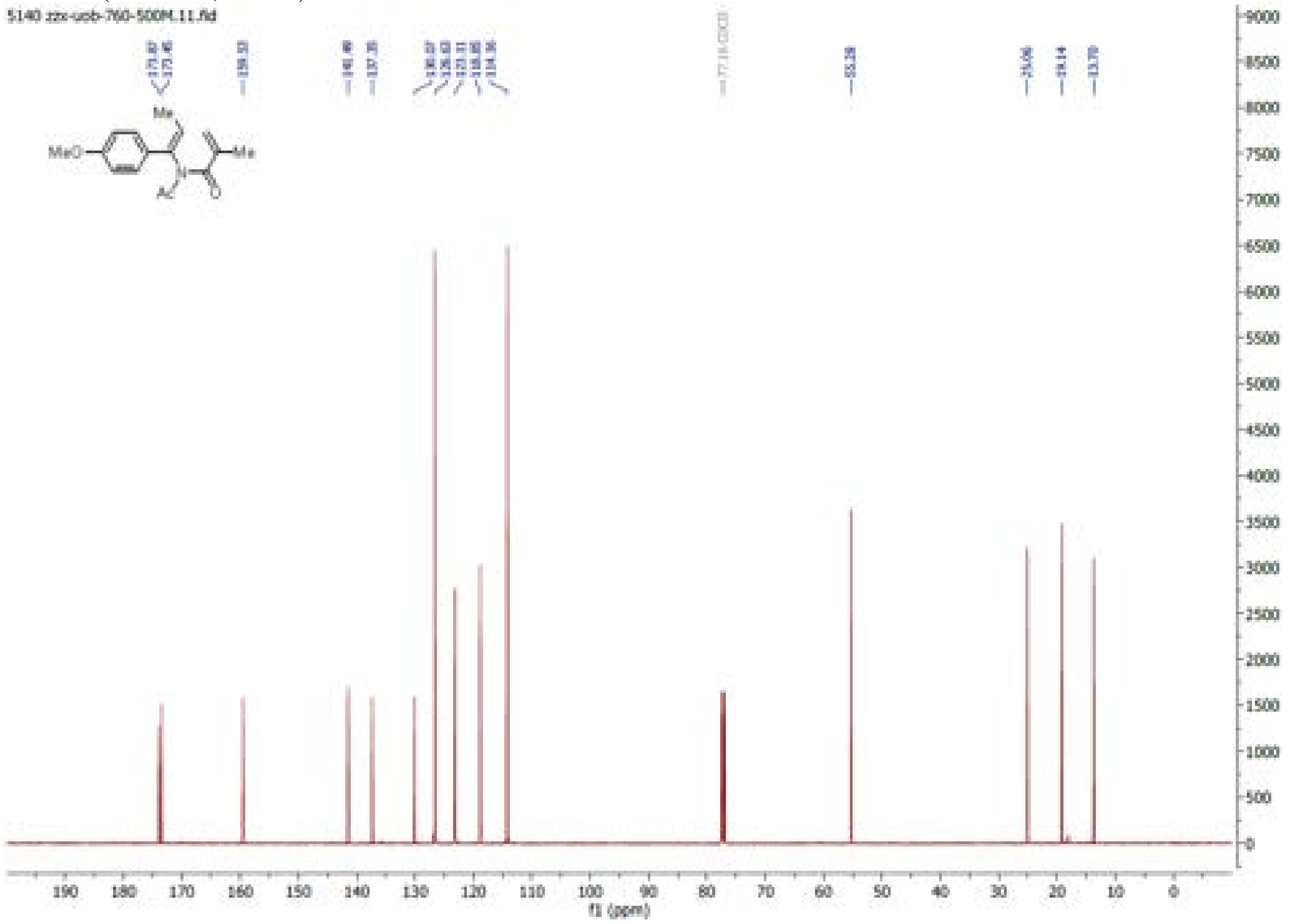

^{1}H NMR (500 MHz, $CDCl_3$) of **2al**

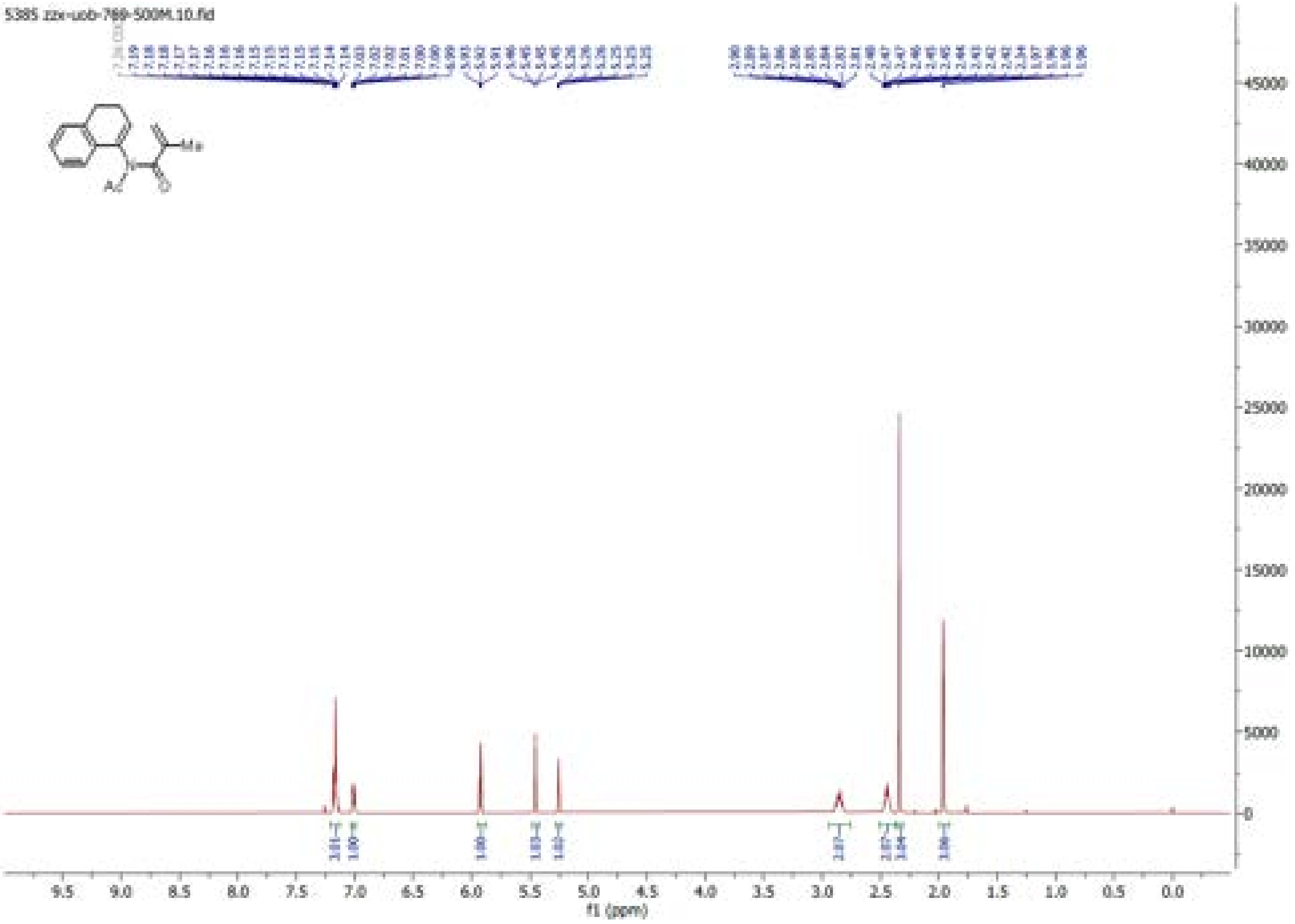

^{13}C NMR (126 MHz, $CDCl_3$) of **2al**

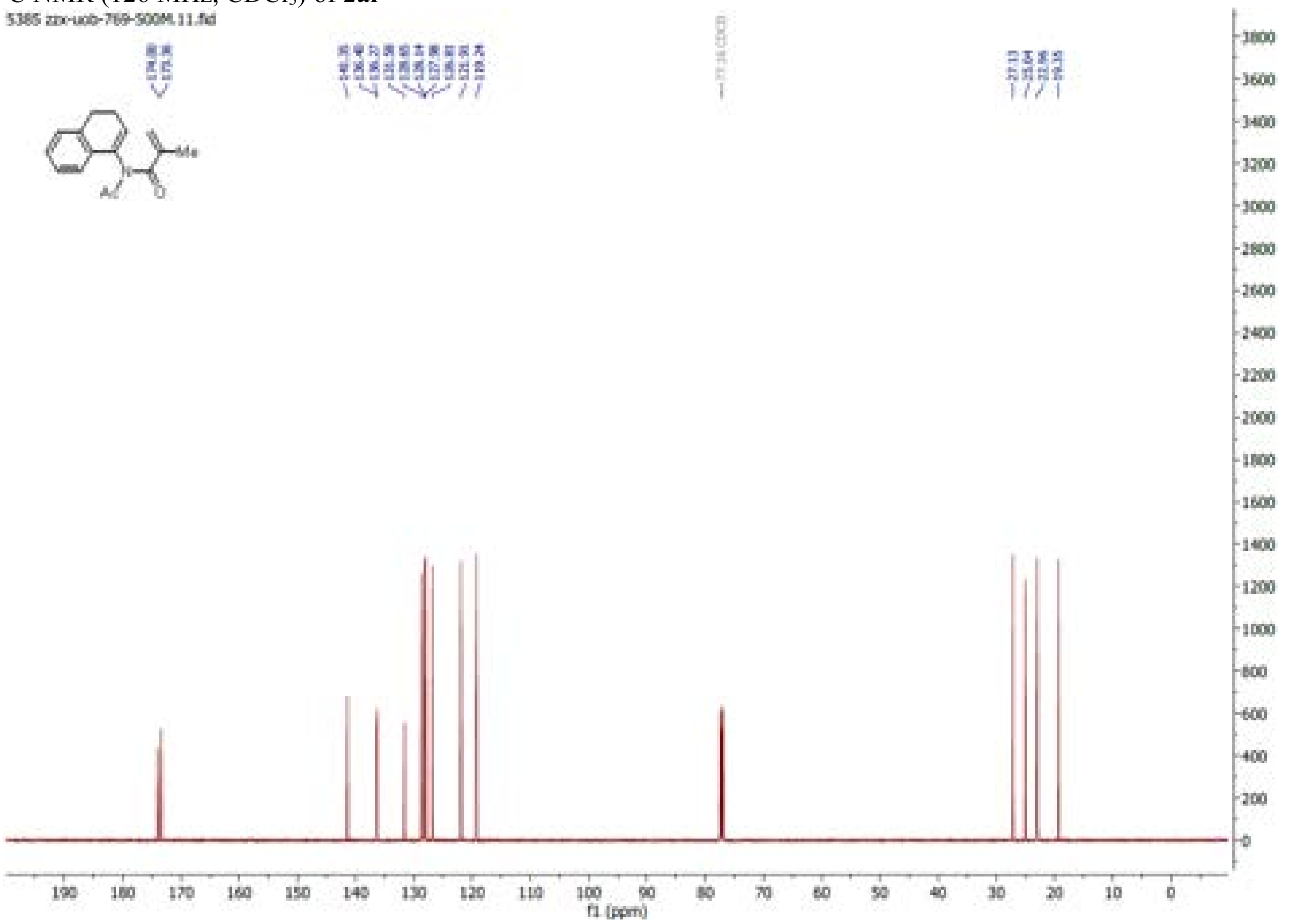

^{1}H NMR (500 MHz, $CDCl_3$) of **2am**

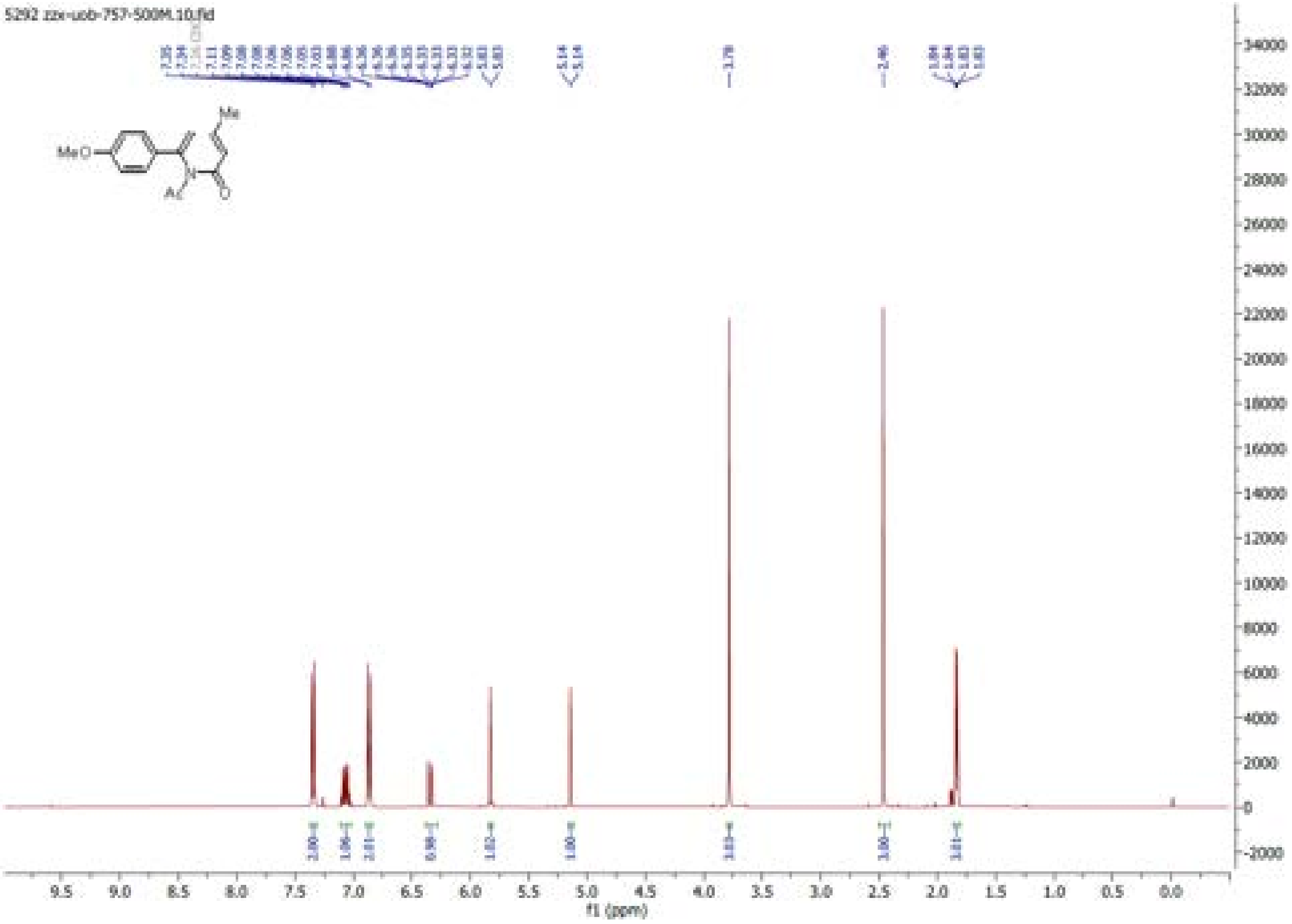

^{13}C NMR (126 MHz, $CDCl_3$) of **2am**

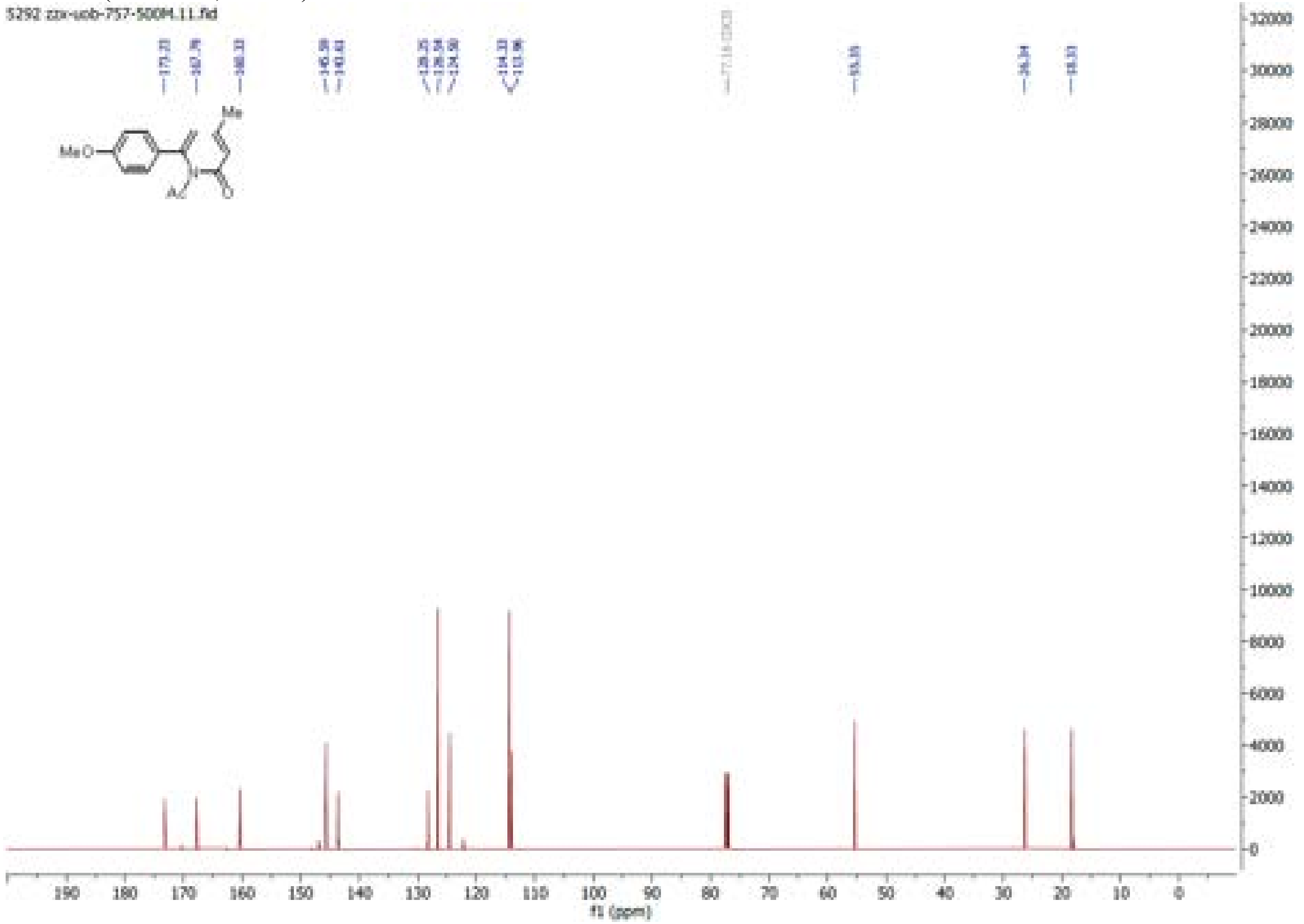

^{1}H NMR (500 MHz, $CDCl_3$) of **S2an'**

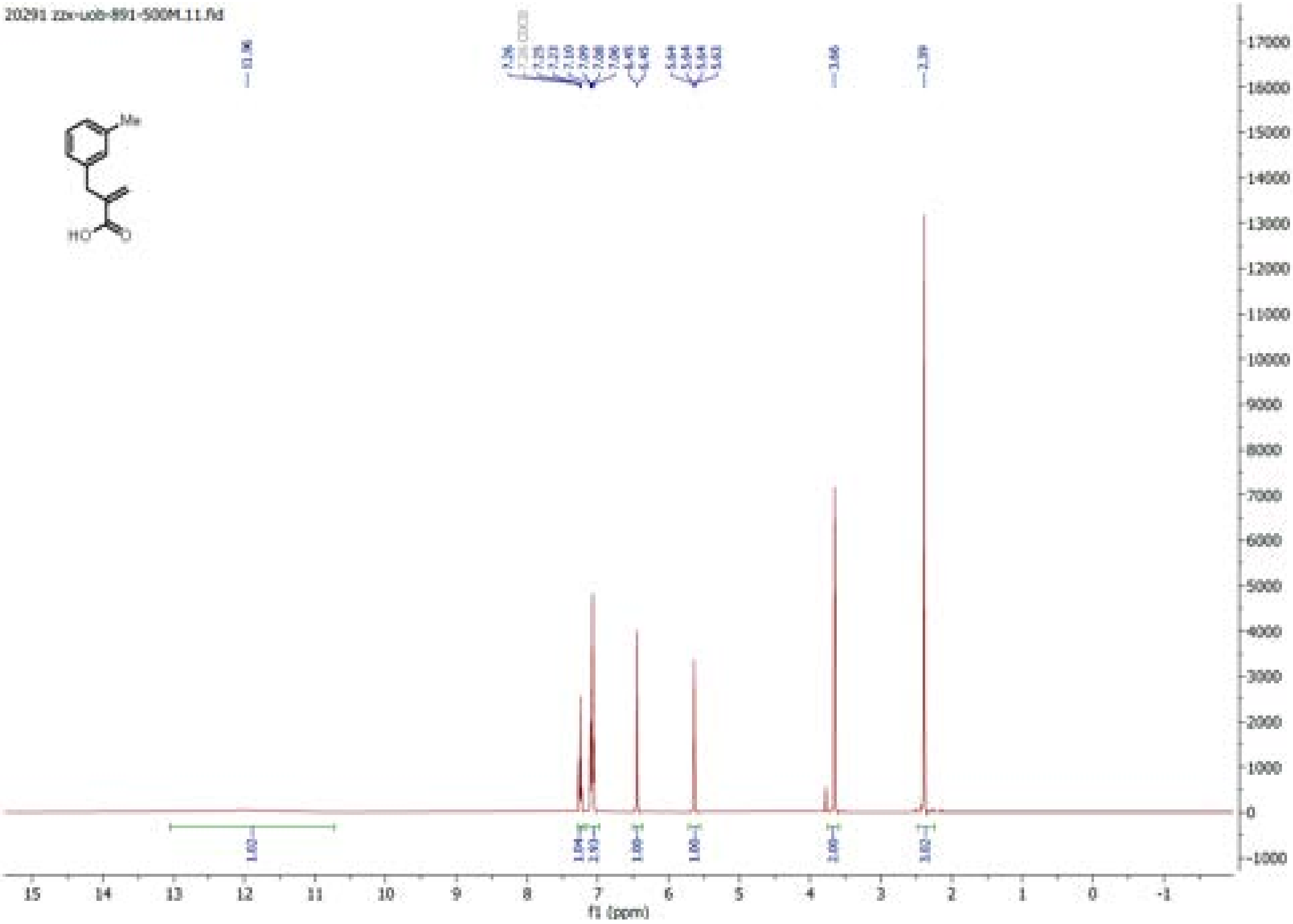

^{13}C NMR (126 MHz, $CDCl_3$) of **S2an'**

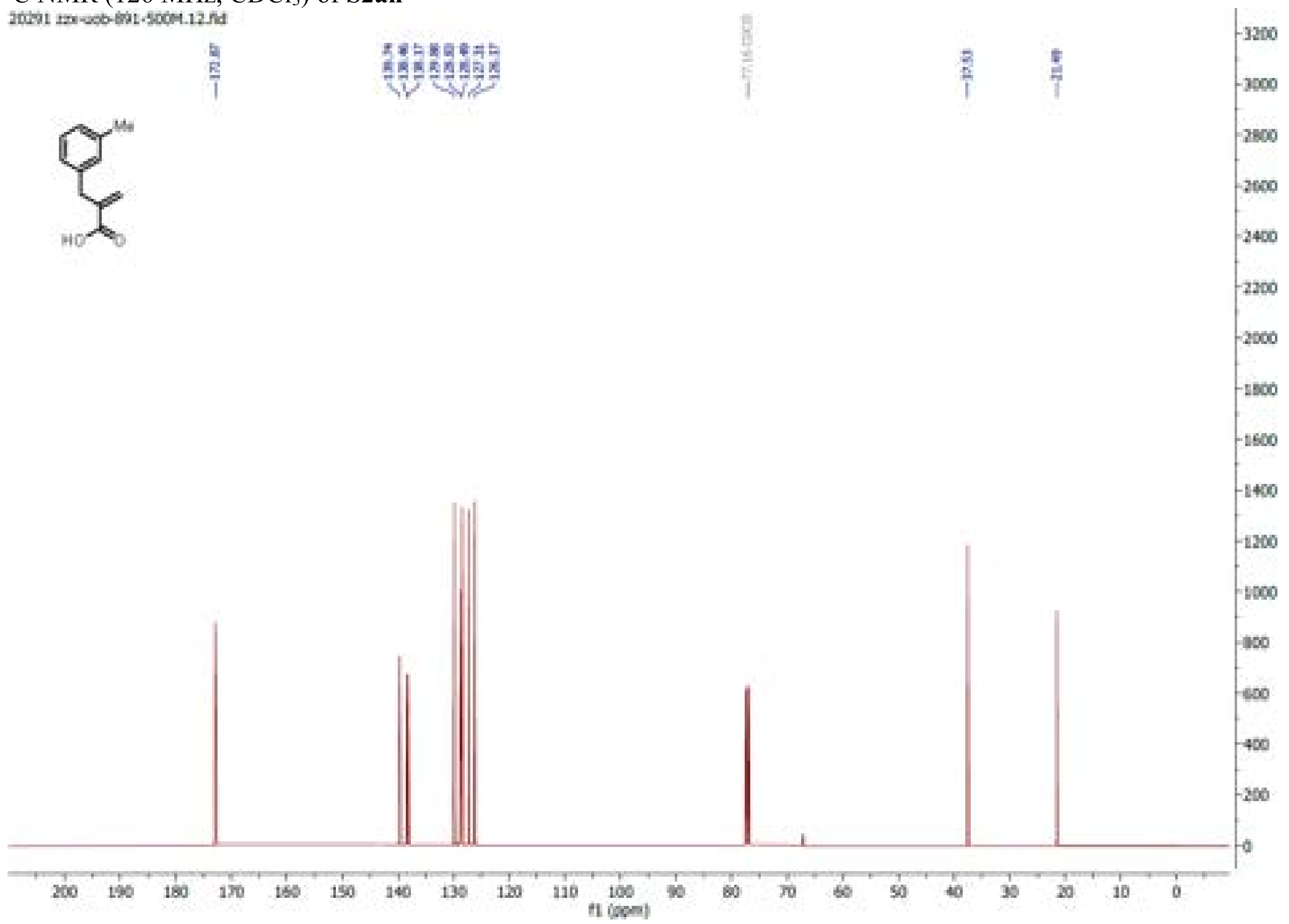

^{1}H NMR (500 MHz, $CDCl_3$) of **S2an**

6186 zzx-uob-892-SM-500M.10.fid

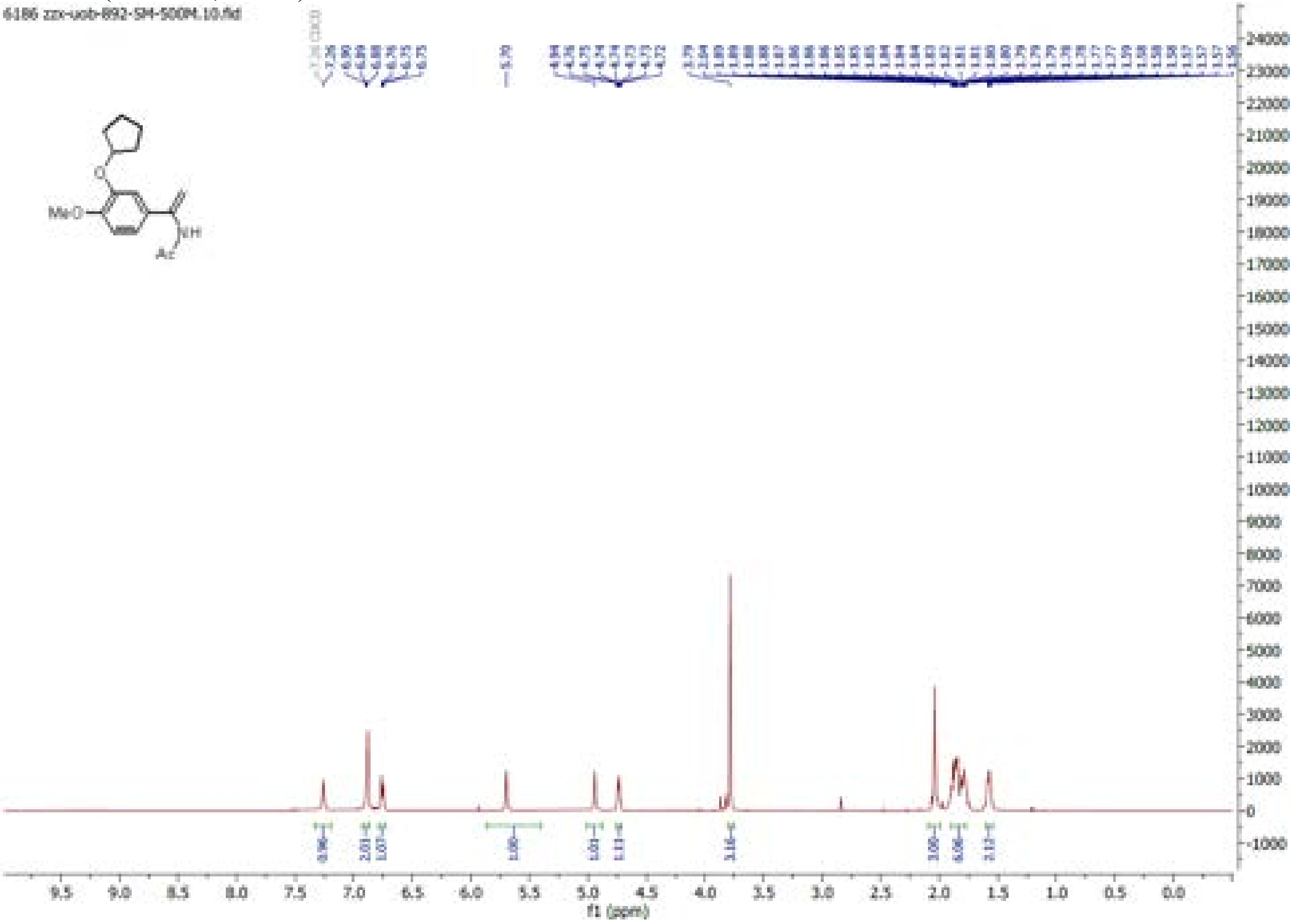

^{13}C NMR (126 MHz, $CDCl_3$) of **S2an**

6186 zzx-uob-892-SM-500M.11.fid

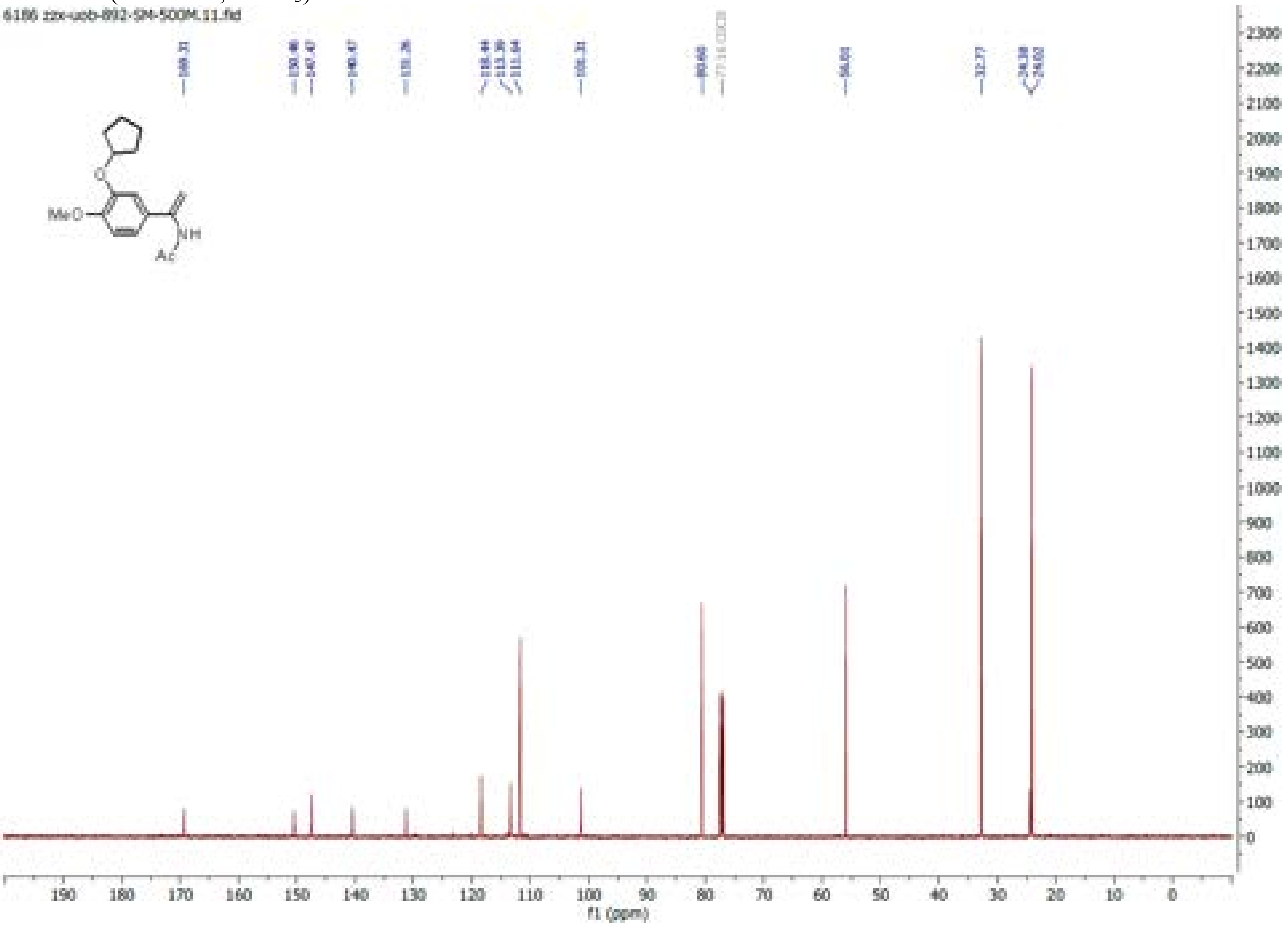

^{1}H NMR (500 MHz, $CDCl_3$) of **2an**

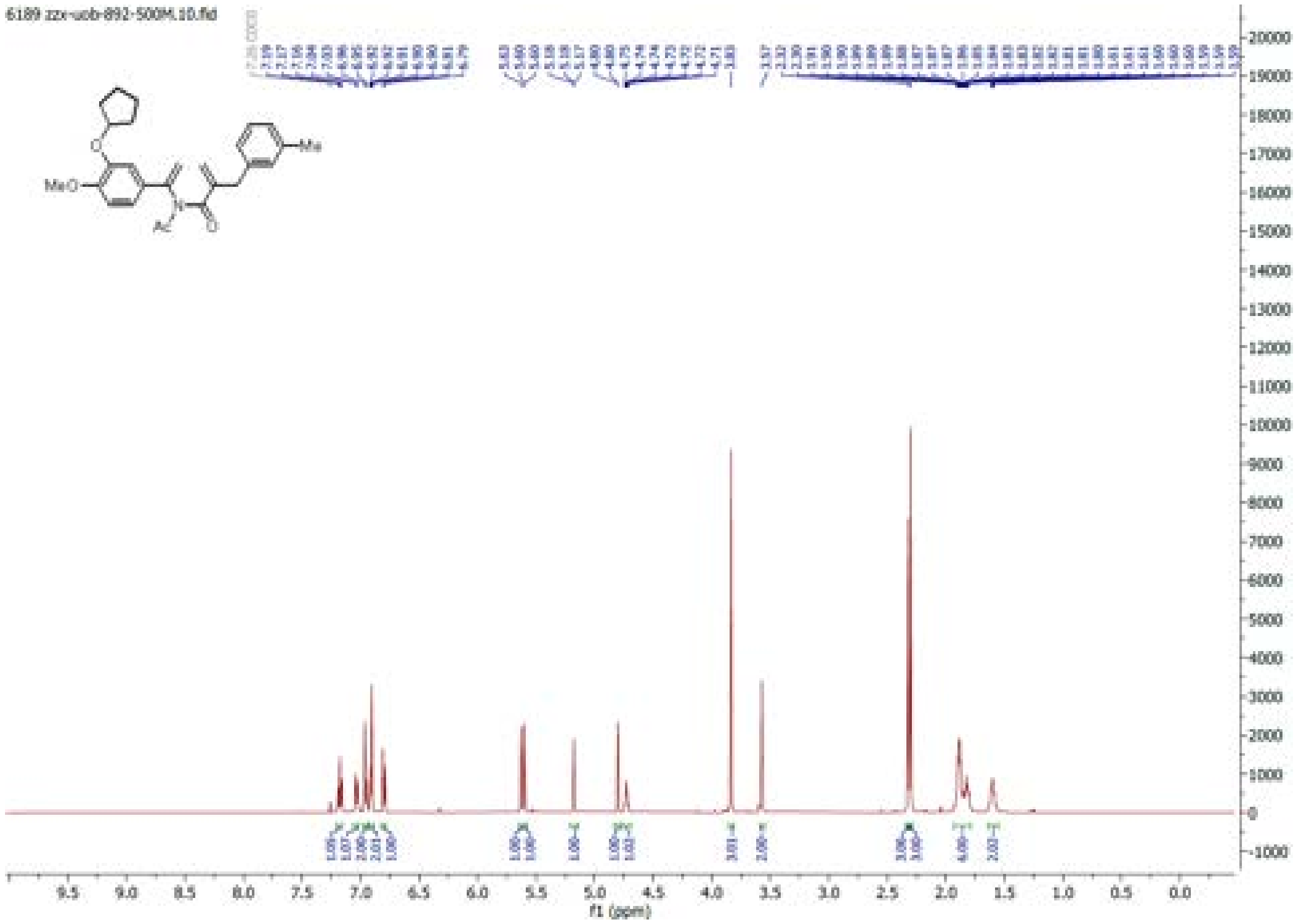

^{13}C NMR (126 MHz, $CDCl_3$) of **2an**

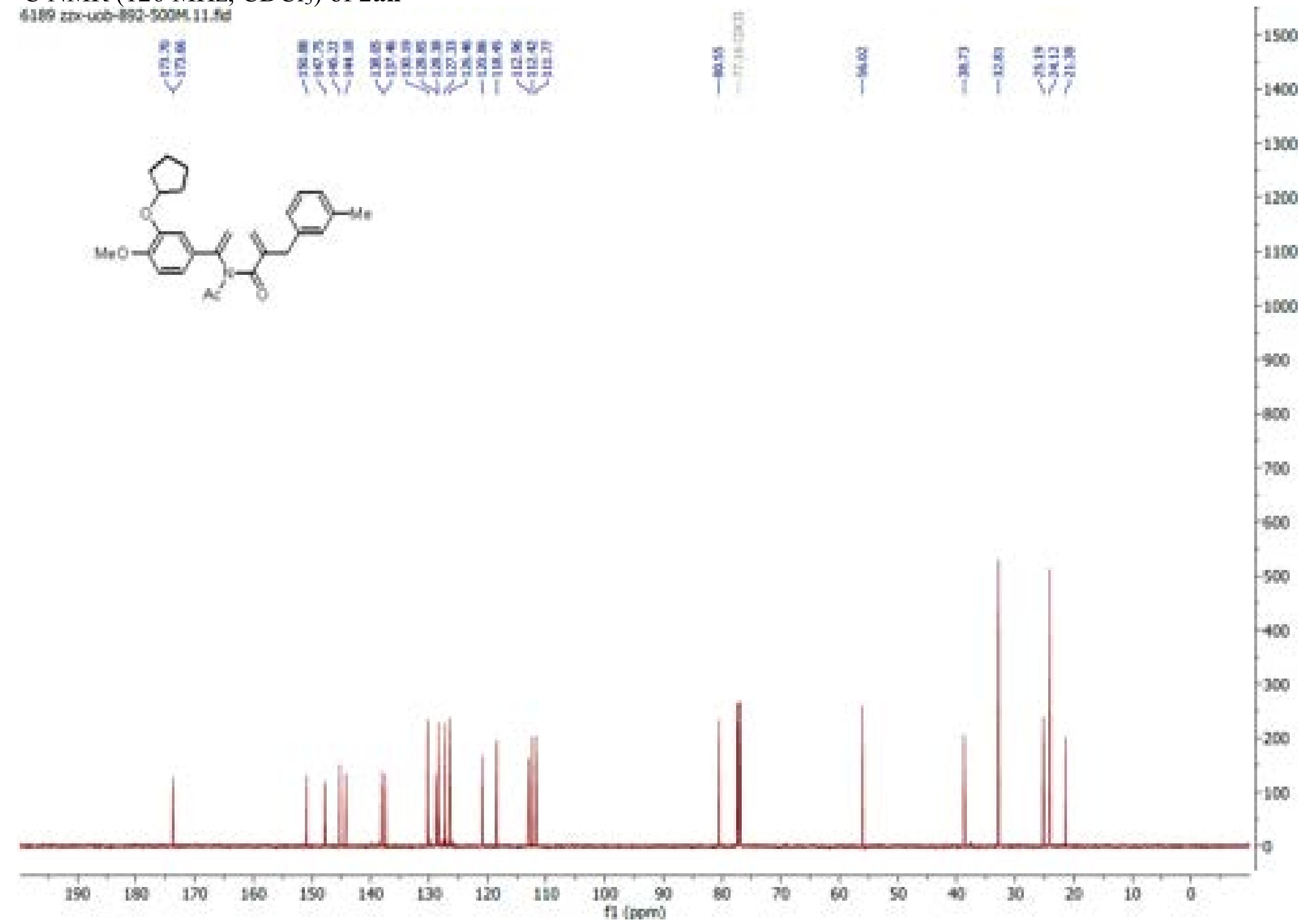

5.4 NMR Spectra of Products (3 and 4)

^{1}H NMR (500 MHz, CDCl$_3$) of **3a**

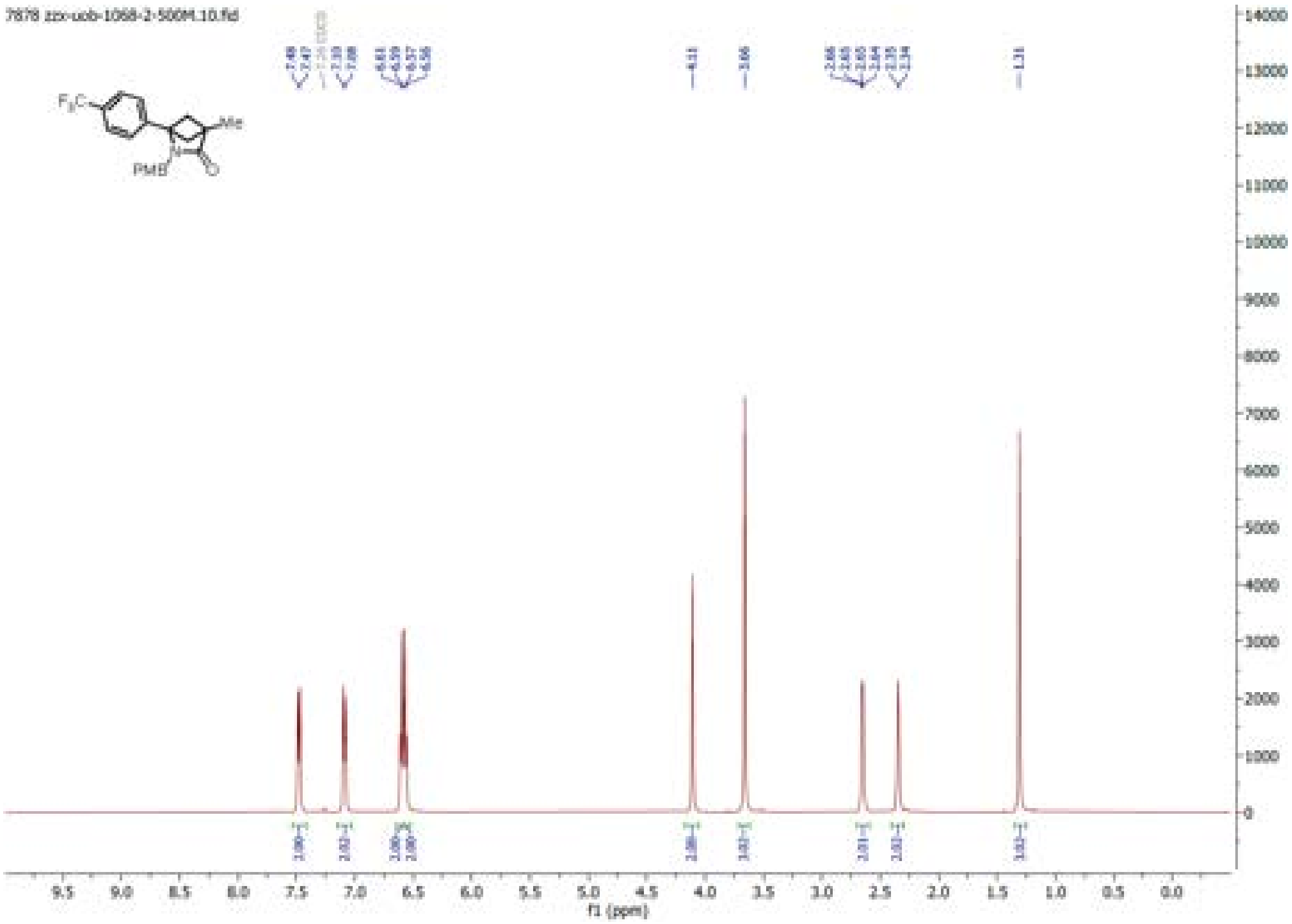

^{13}C NMR (126 MHz, CDCl$_3$) of **3a**

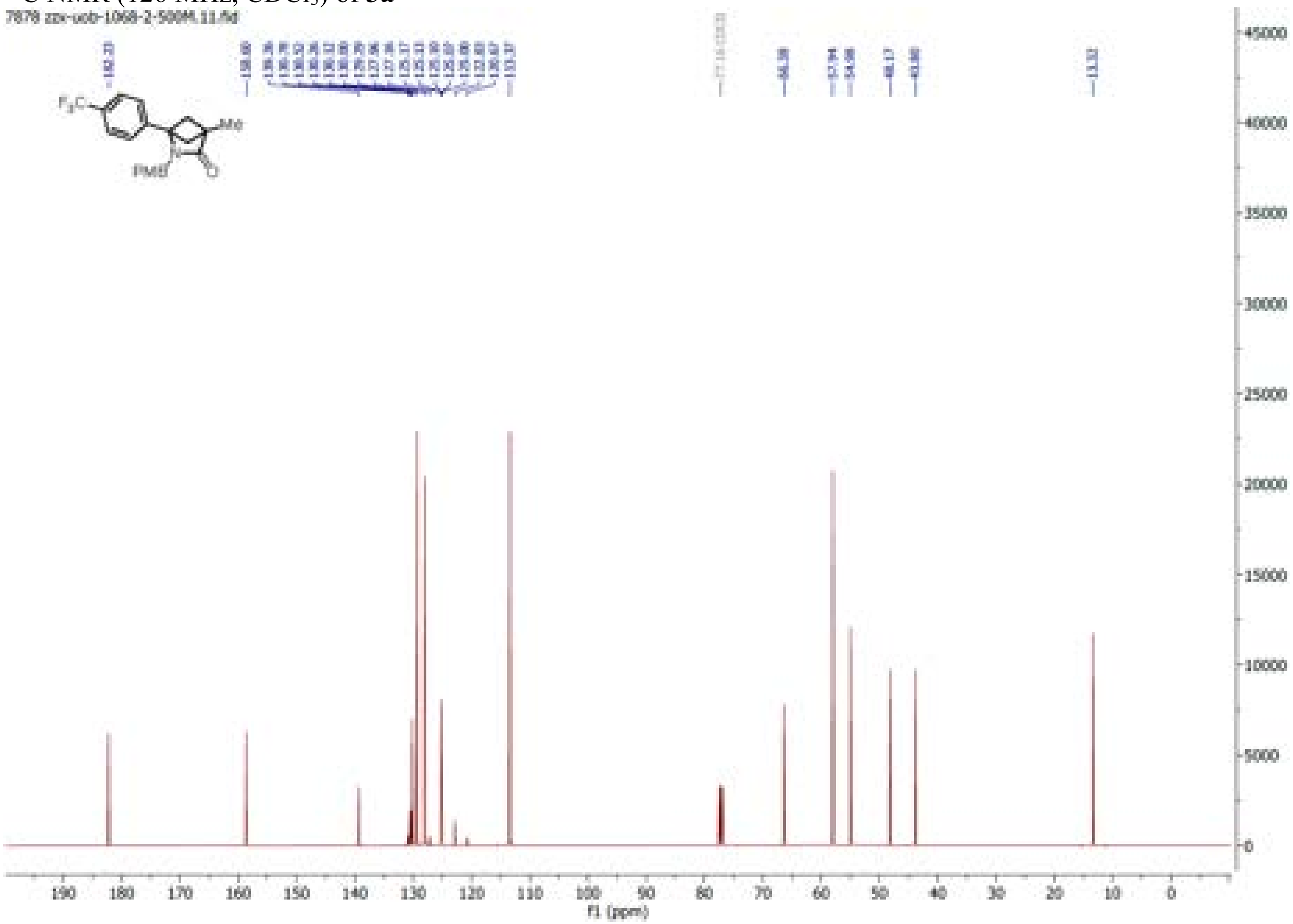

^{19}F NMR (471 MHz, $CDCl_3$) of **3a**

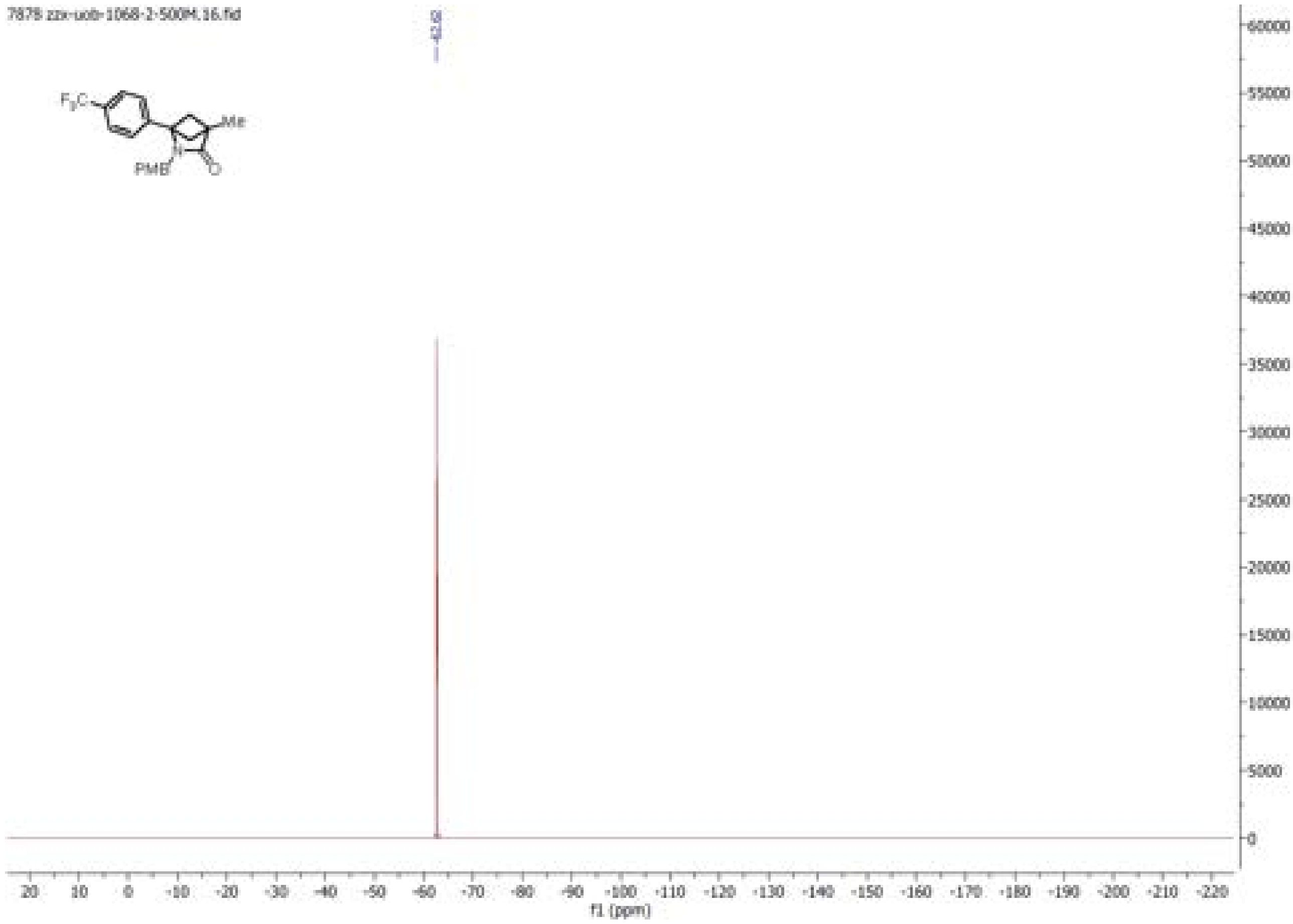

^{1}H NMR (500 MHz, $CDCl_3$) of **4a**

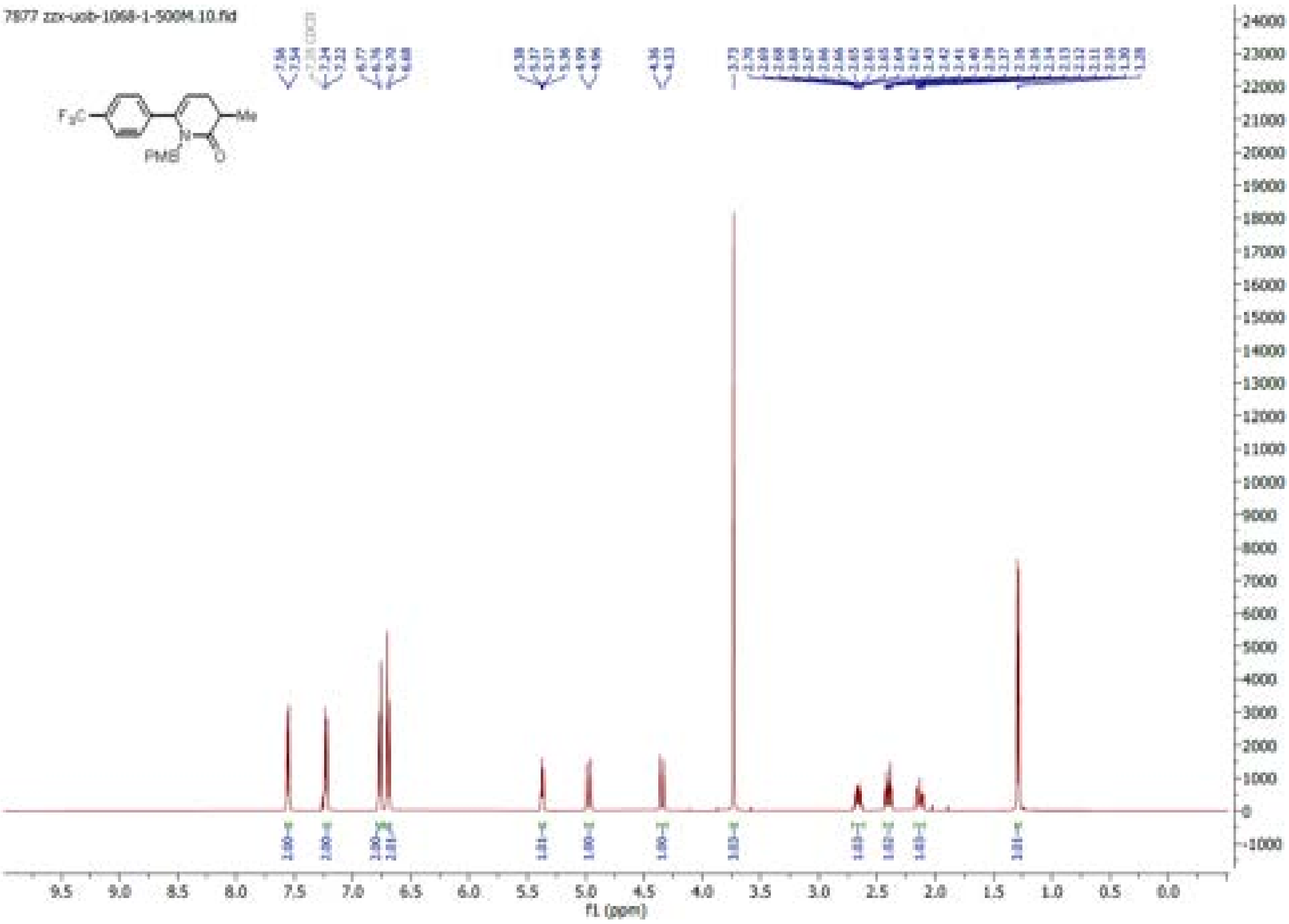

^{13}C NMR (126 MHz, $CDCl_3$) of **4a**

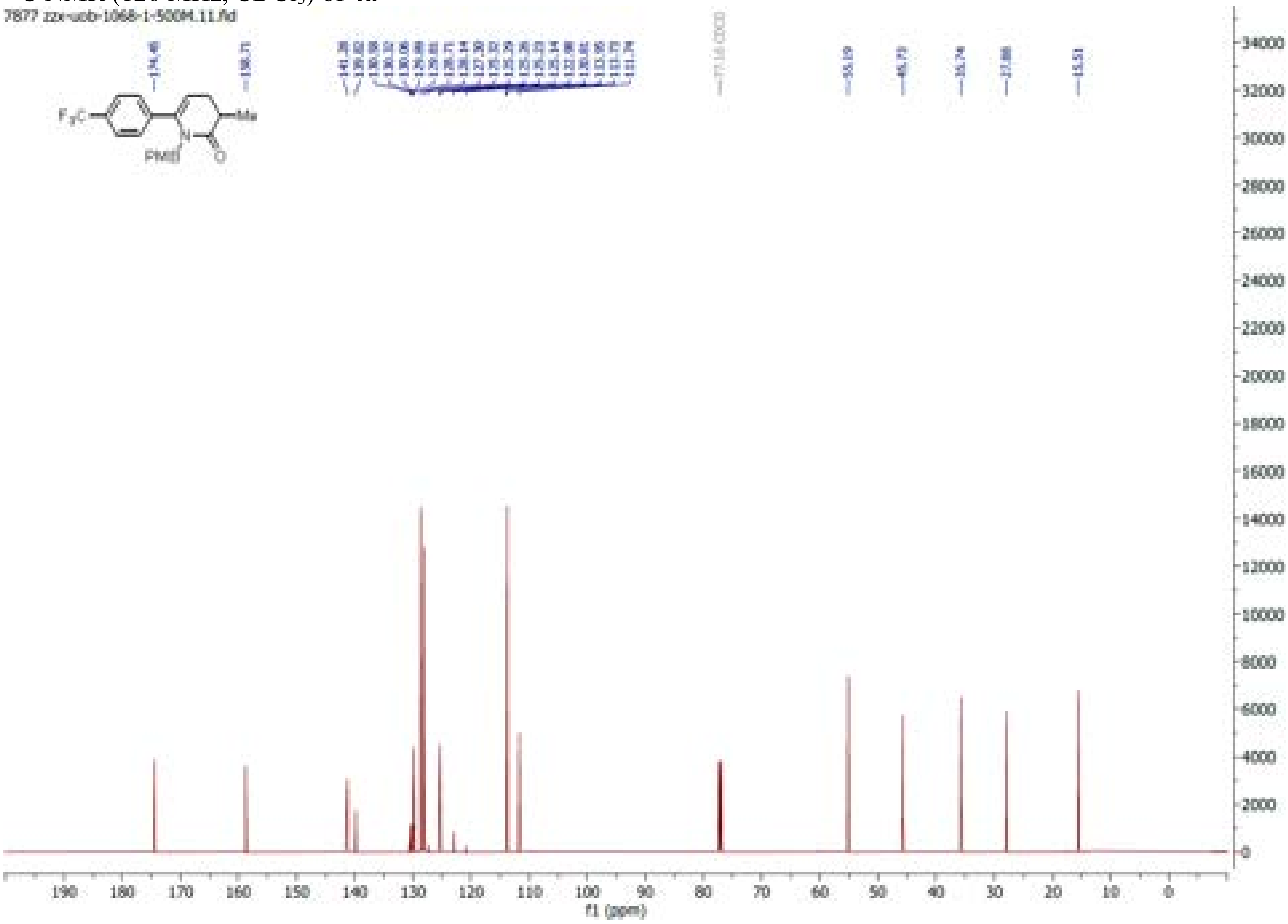

^{19}F NMR (471 MHz, $CDCl_3$) of **4a**

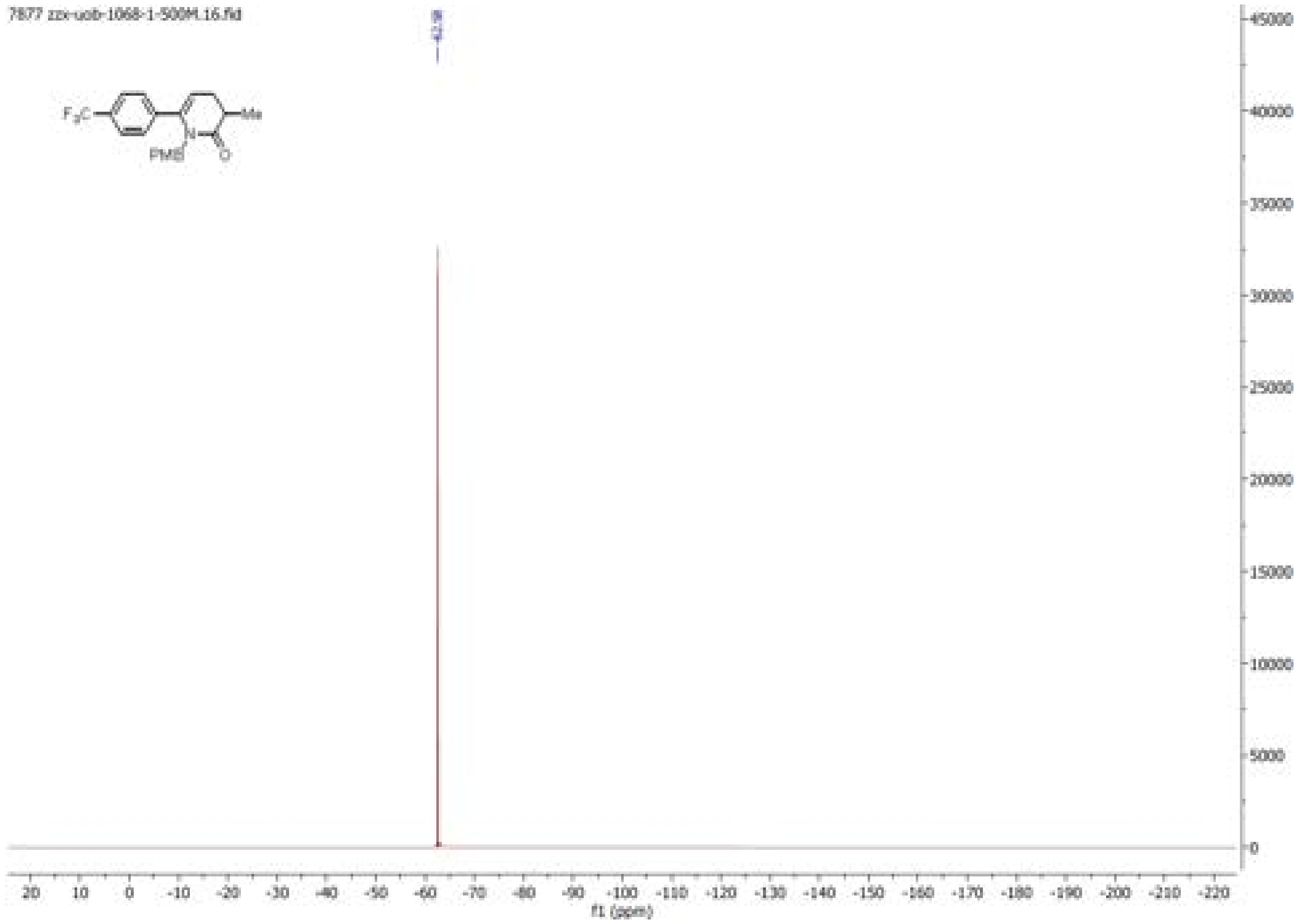

^{1}H NMR (500 MHz, $CDCl_3$) of **3b**

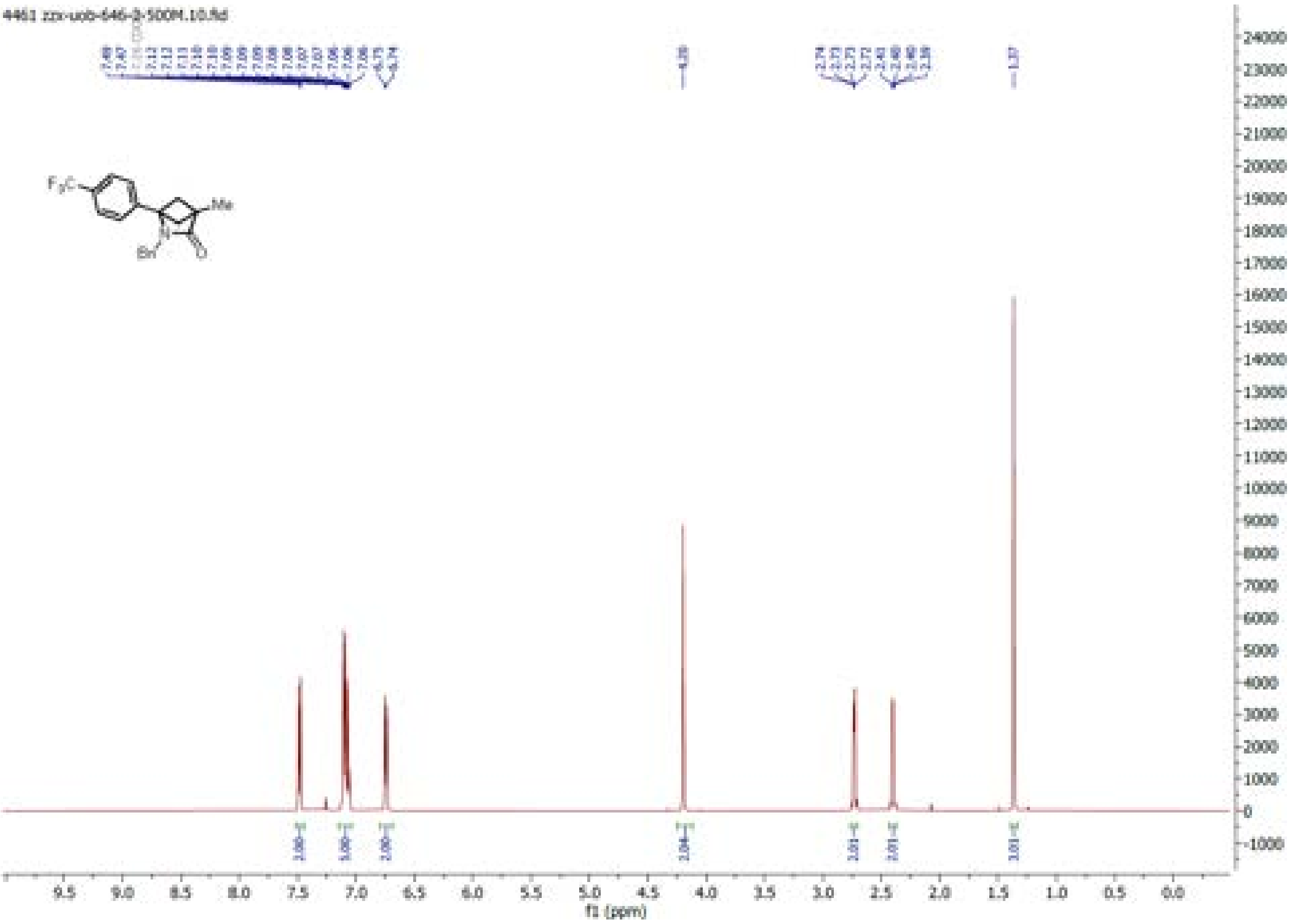

^{13}C NMR (126 MHz, $CDCl_3$) of **3b**

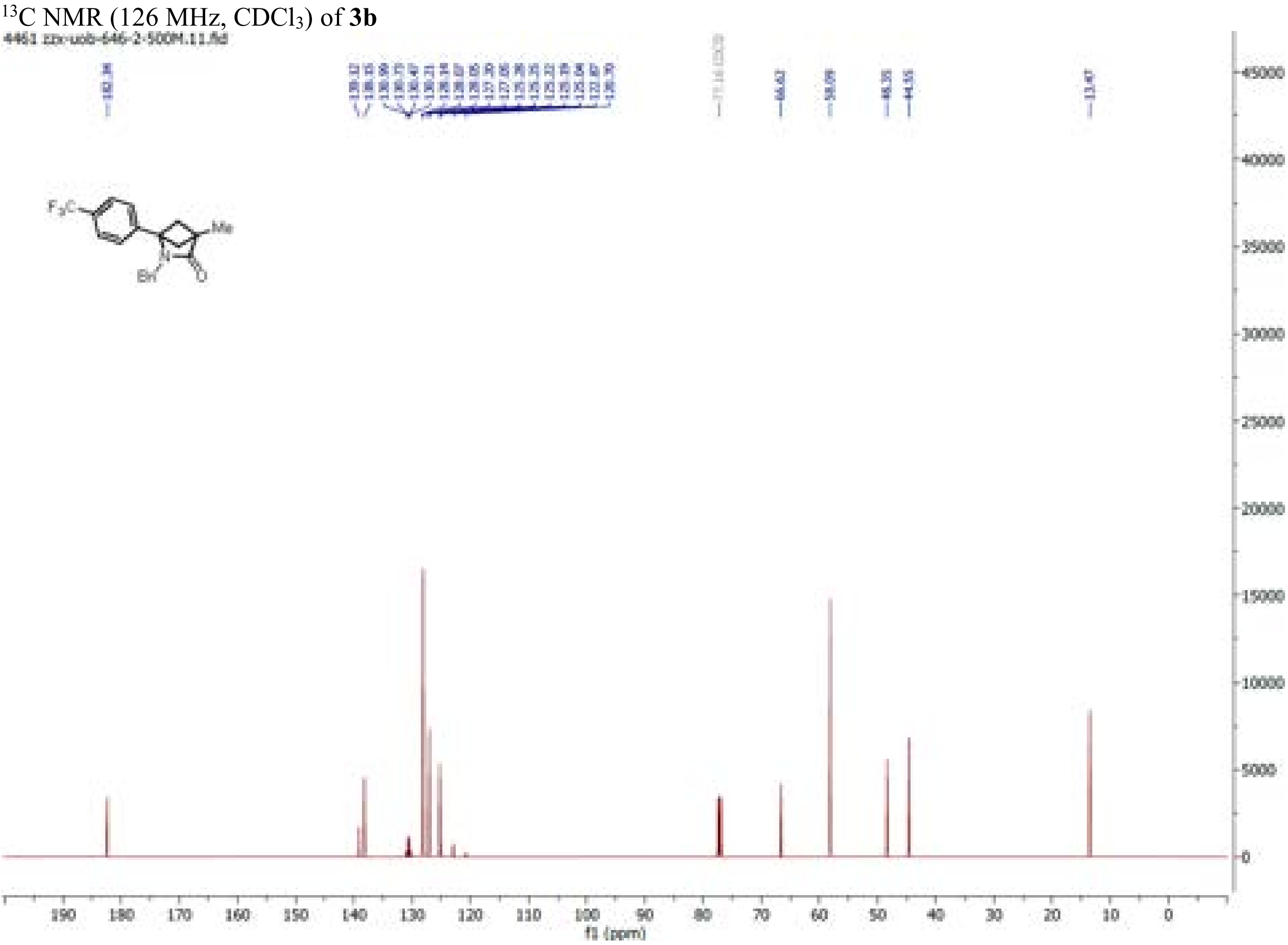

^{19}F NMR (471 MHz, $CDCl_3$) of **3b**

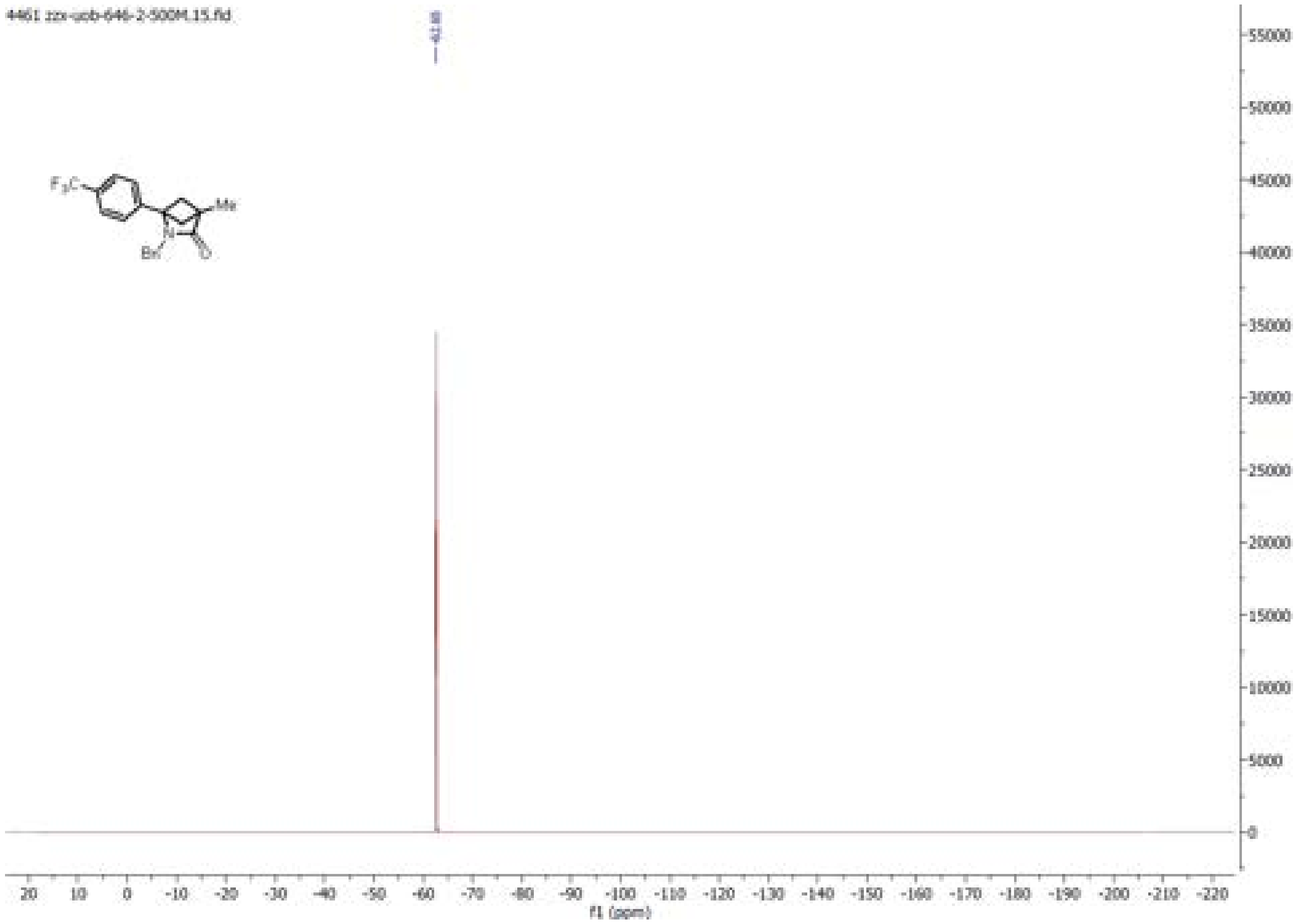

^{1}H NMR (500 MHz, $CDCl_3$) of **4b**

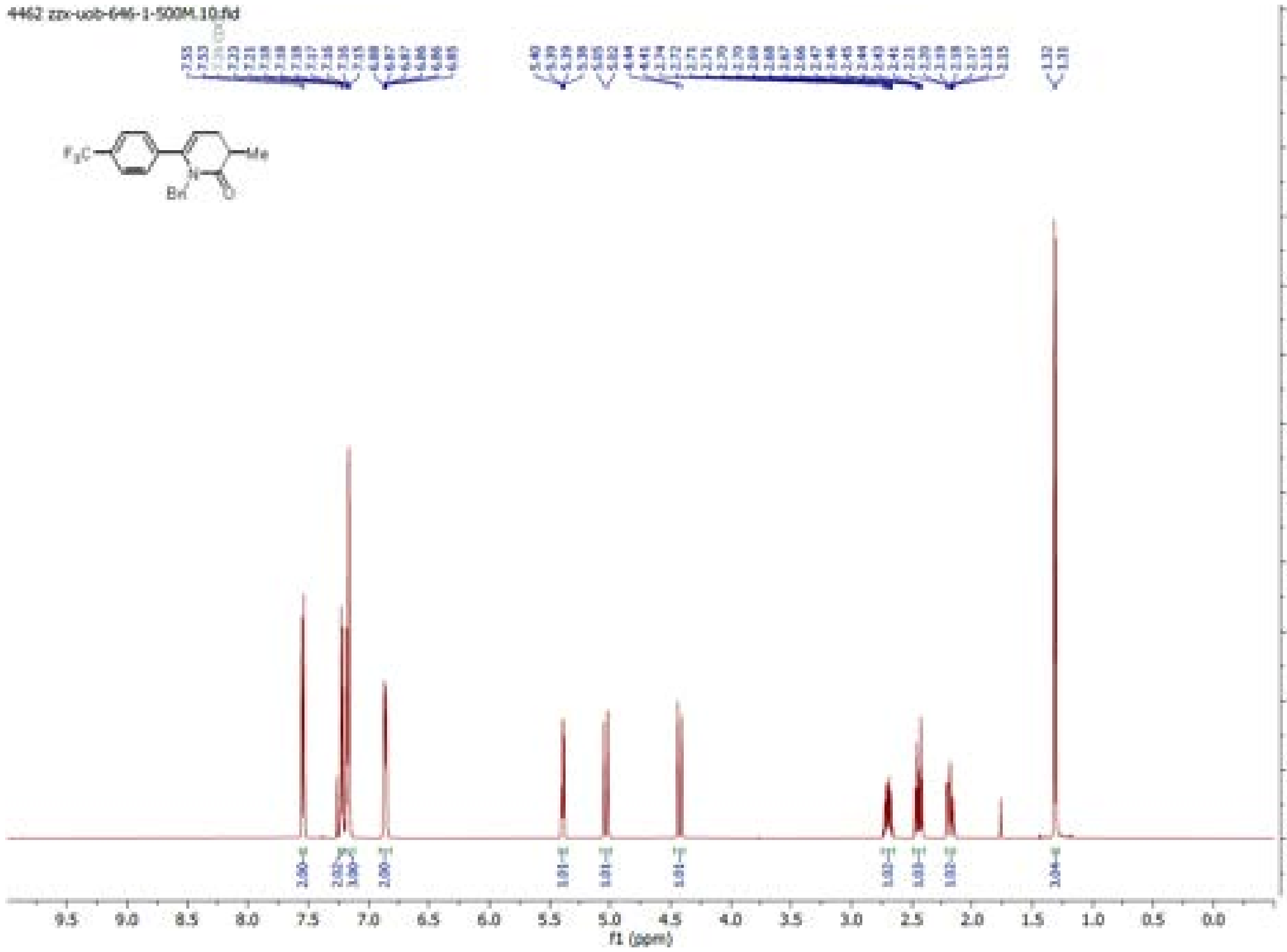

^{13}C NMR (126 MHz, $CDCl_3$) of **4b**

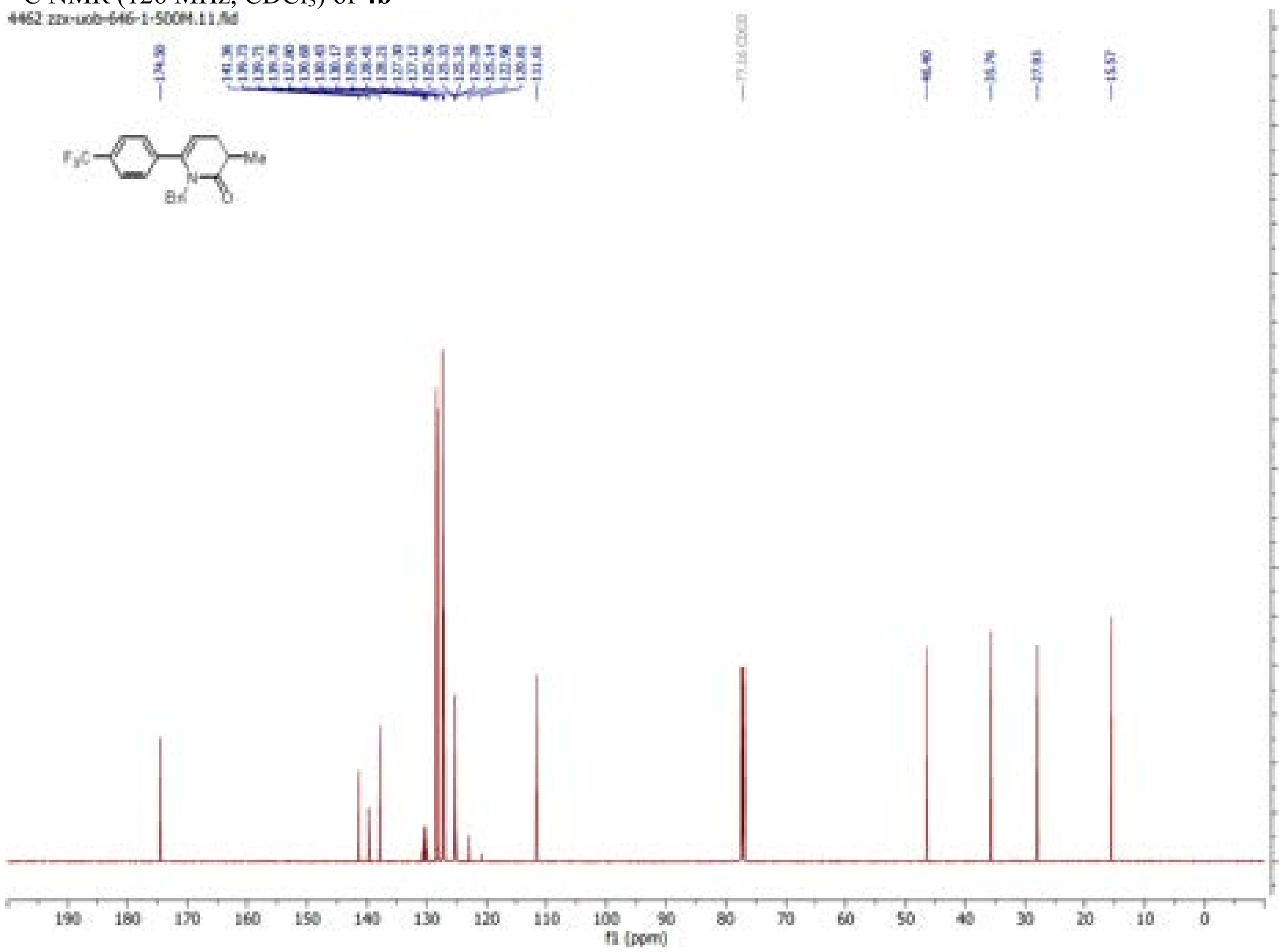

^{19}F NMR (471 MHz, $CDCl_3$) of **4b**

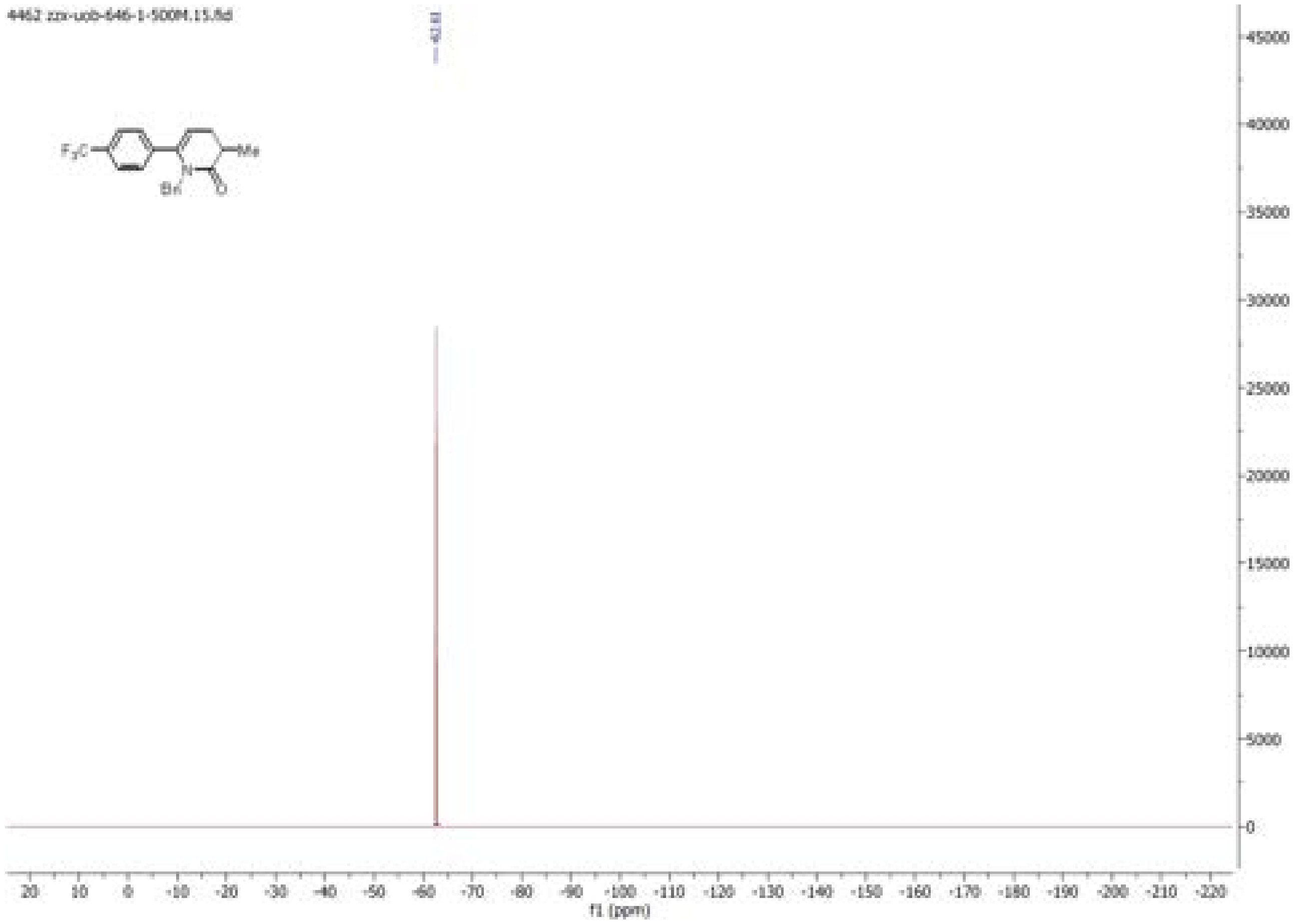

^{1}H NMR (500 MHz, $CDCl_3$) of **3c**

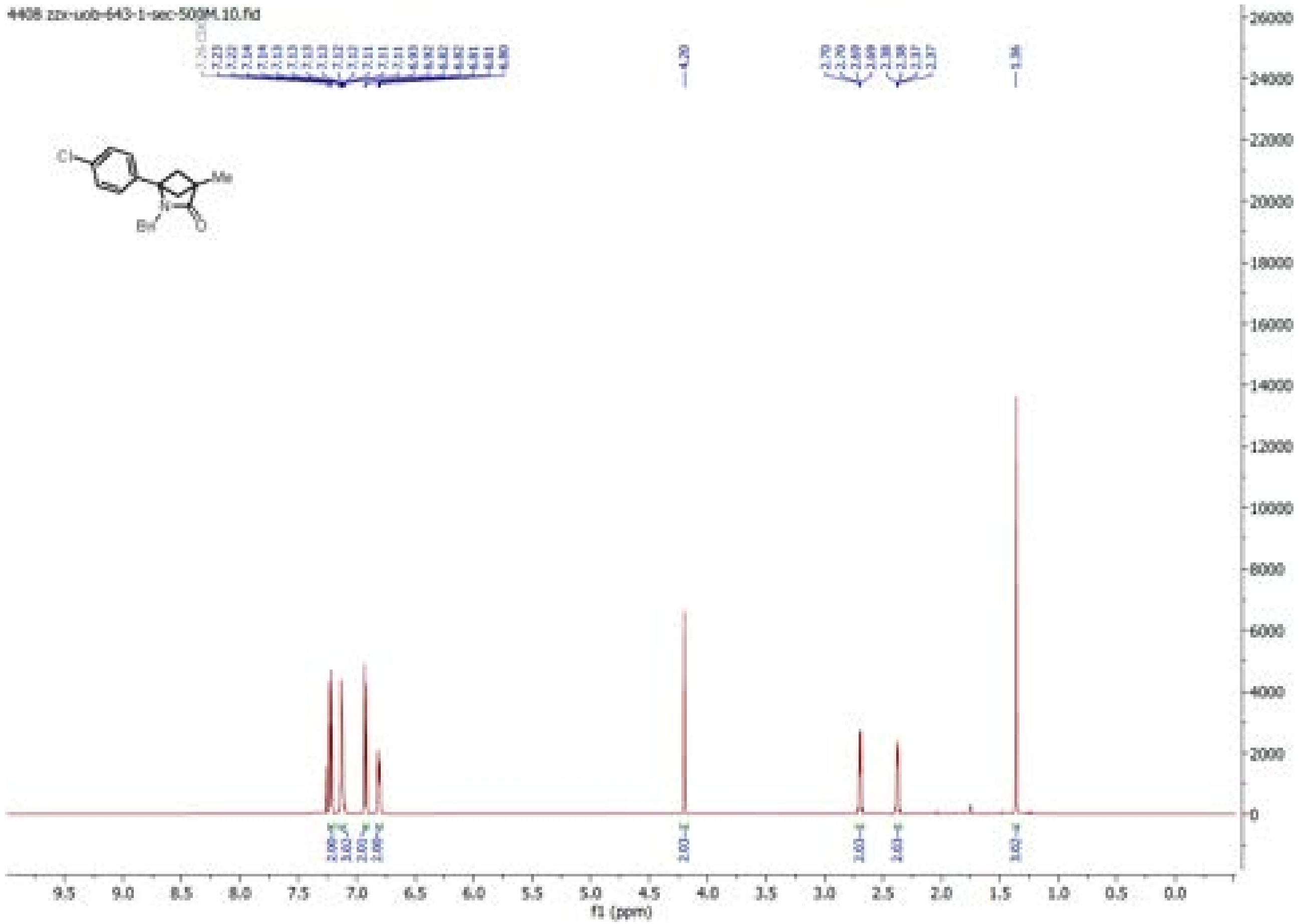

^{13}C NMR (126 MHz, $CDCl_3$) of **3c**

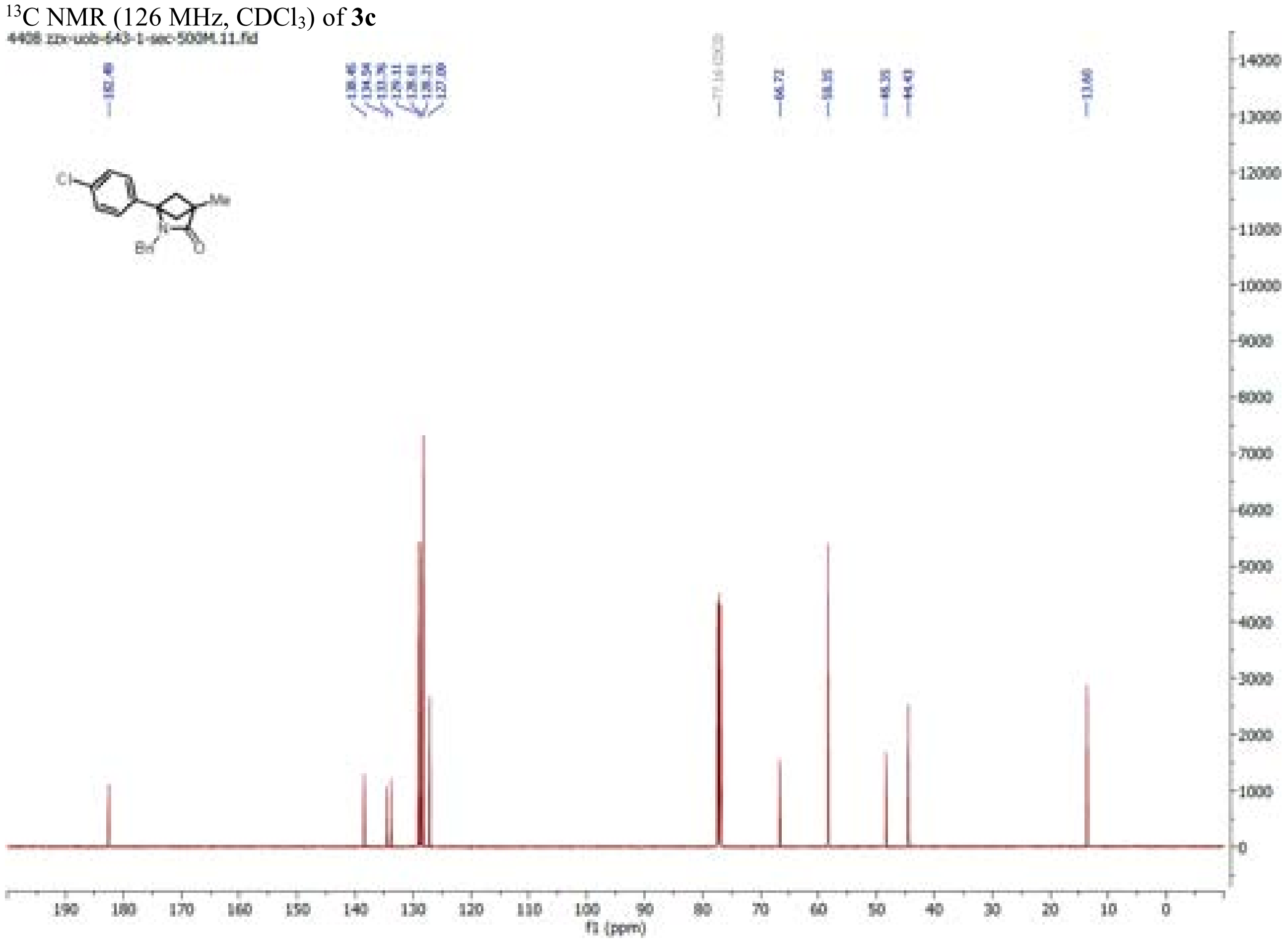

^{1}H NMR (500 MHz, $CDCl_3$) of **4c**

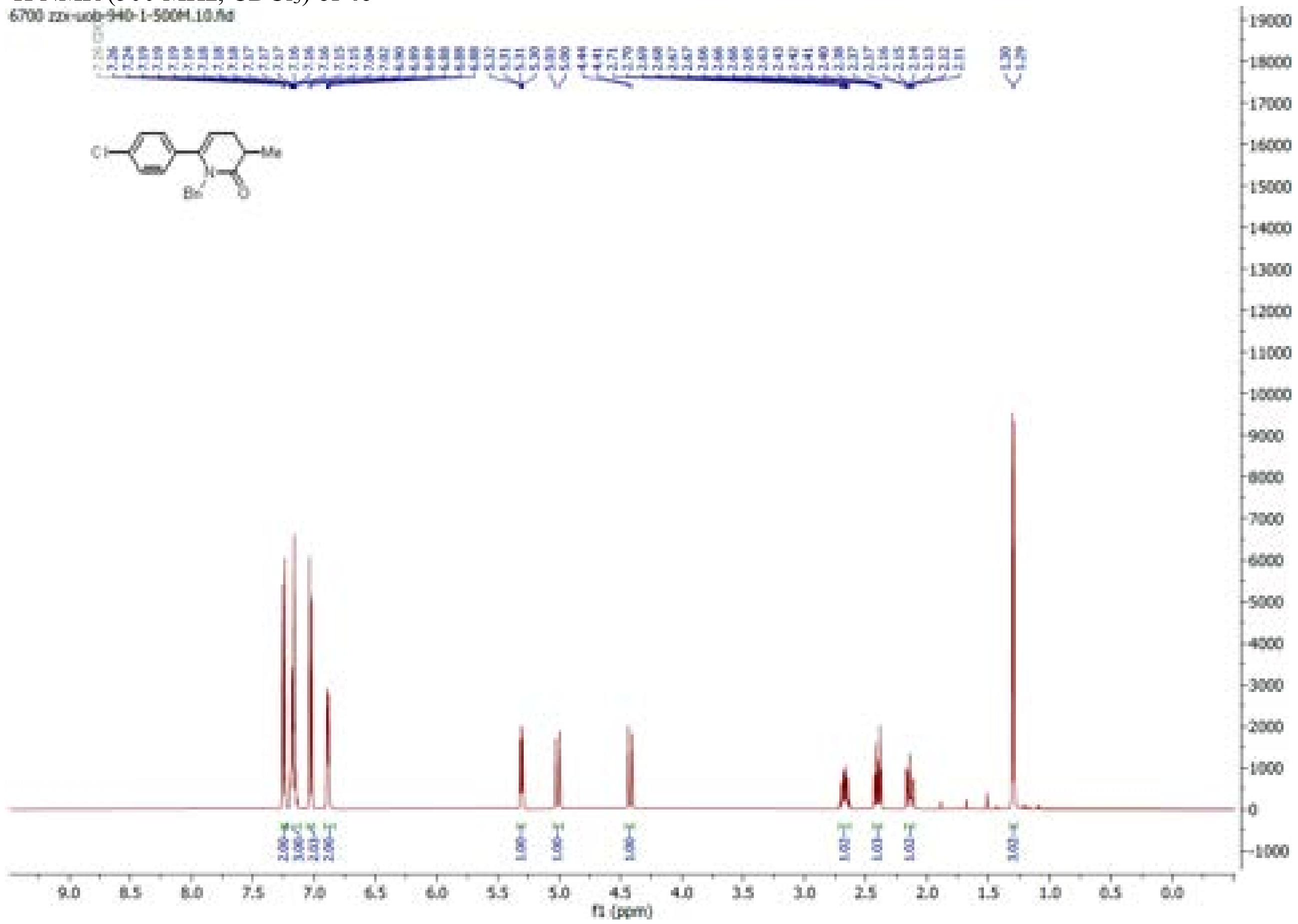

^{13}C NMR (126 MHz, $CDCl_3$) of **4c**

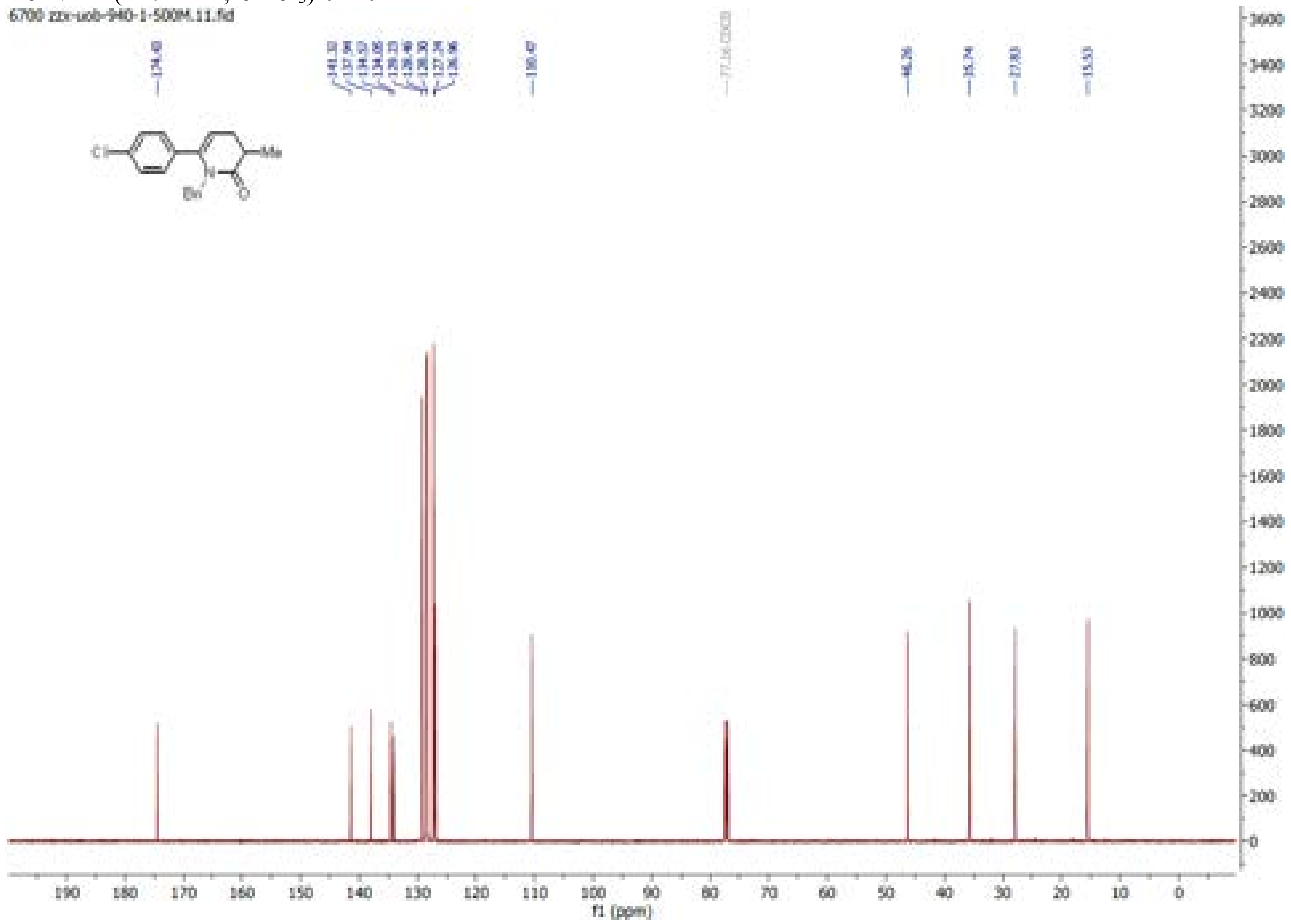

^{1}H NMR (500 MHz, $CDCl_3$) of **3d**

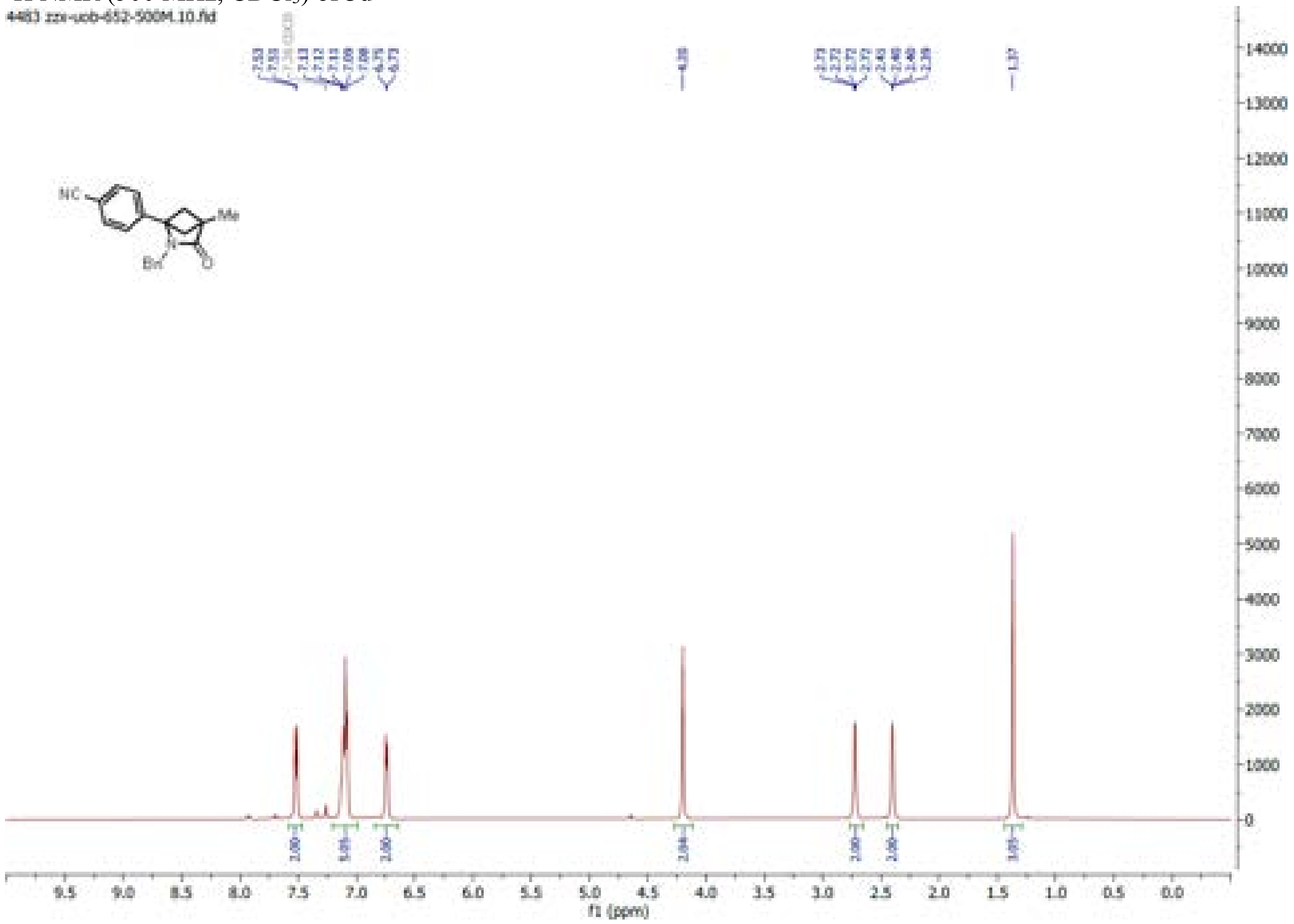

^{13}C NMR (126 MHz, $CDCl_3$) of **3d**

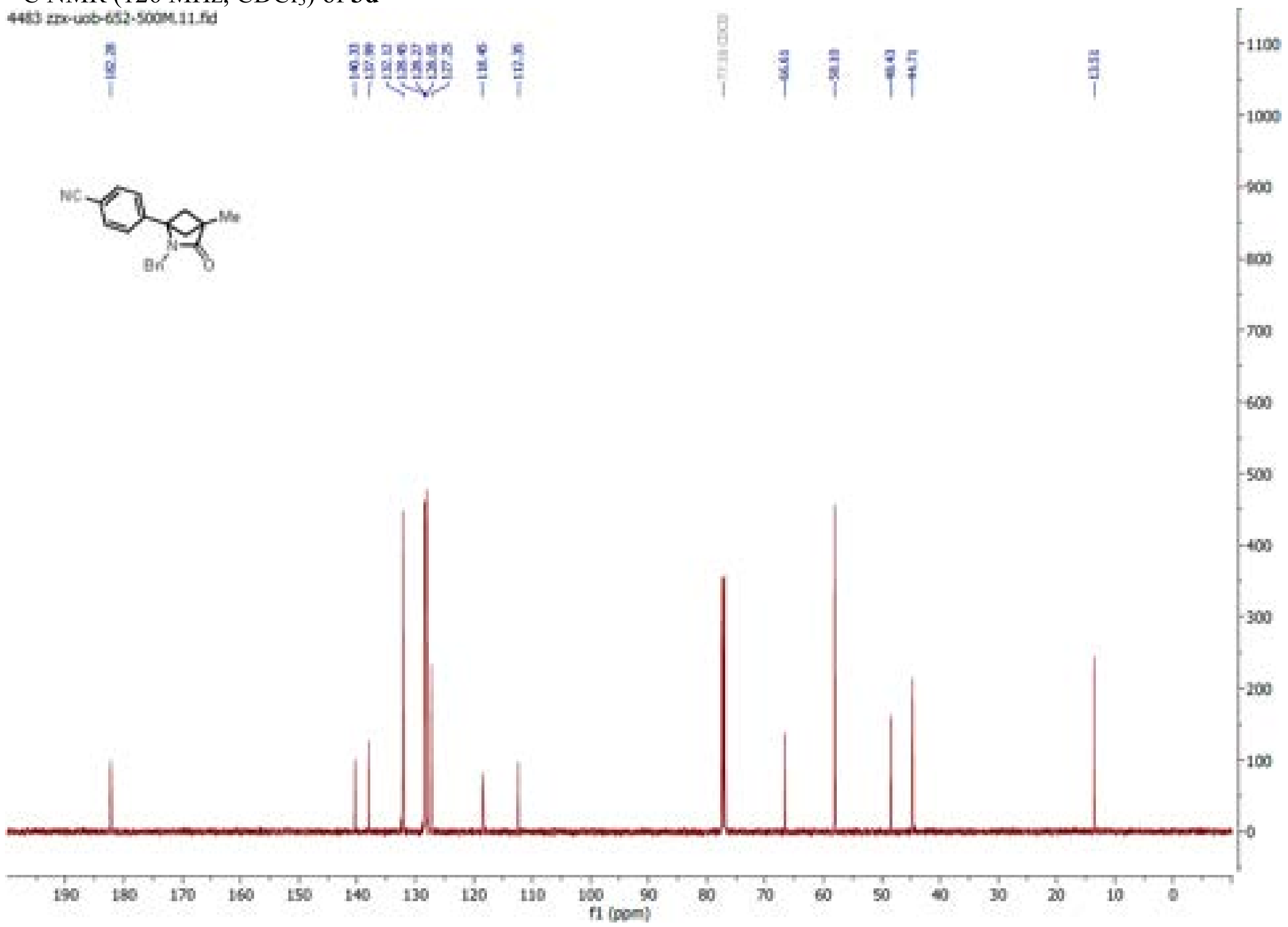

^{1}H NMR (500 MHz, $CDCl_3$) of **3e**

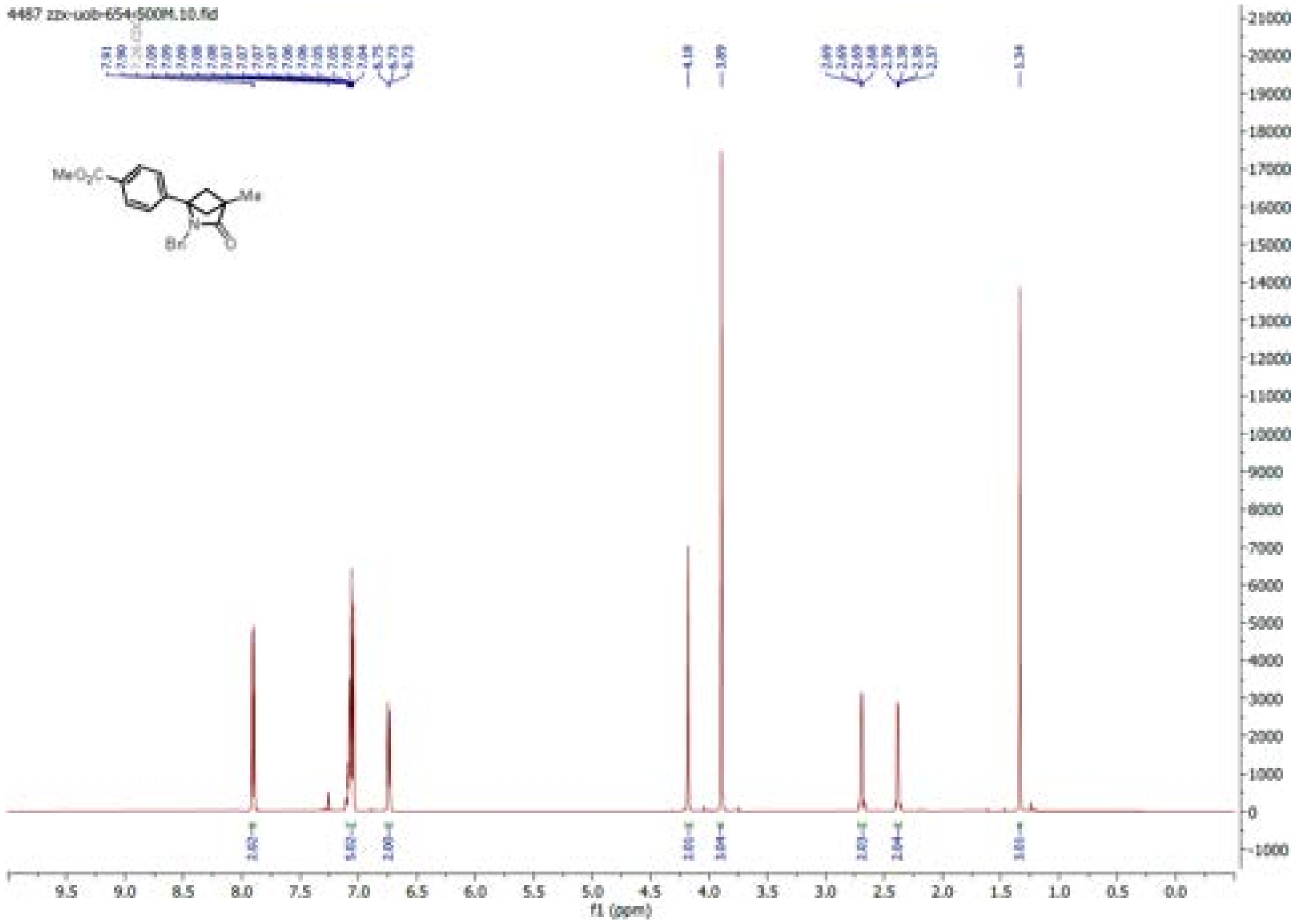

^{13}C NMR (126 MHz, $CDCl_3$) of **3e**

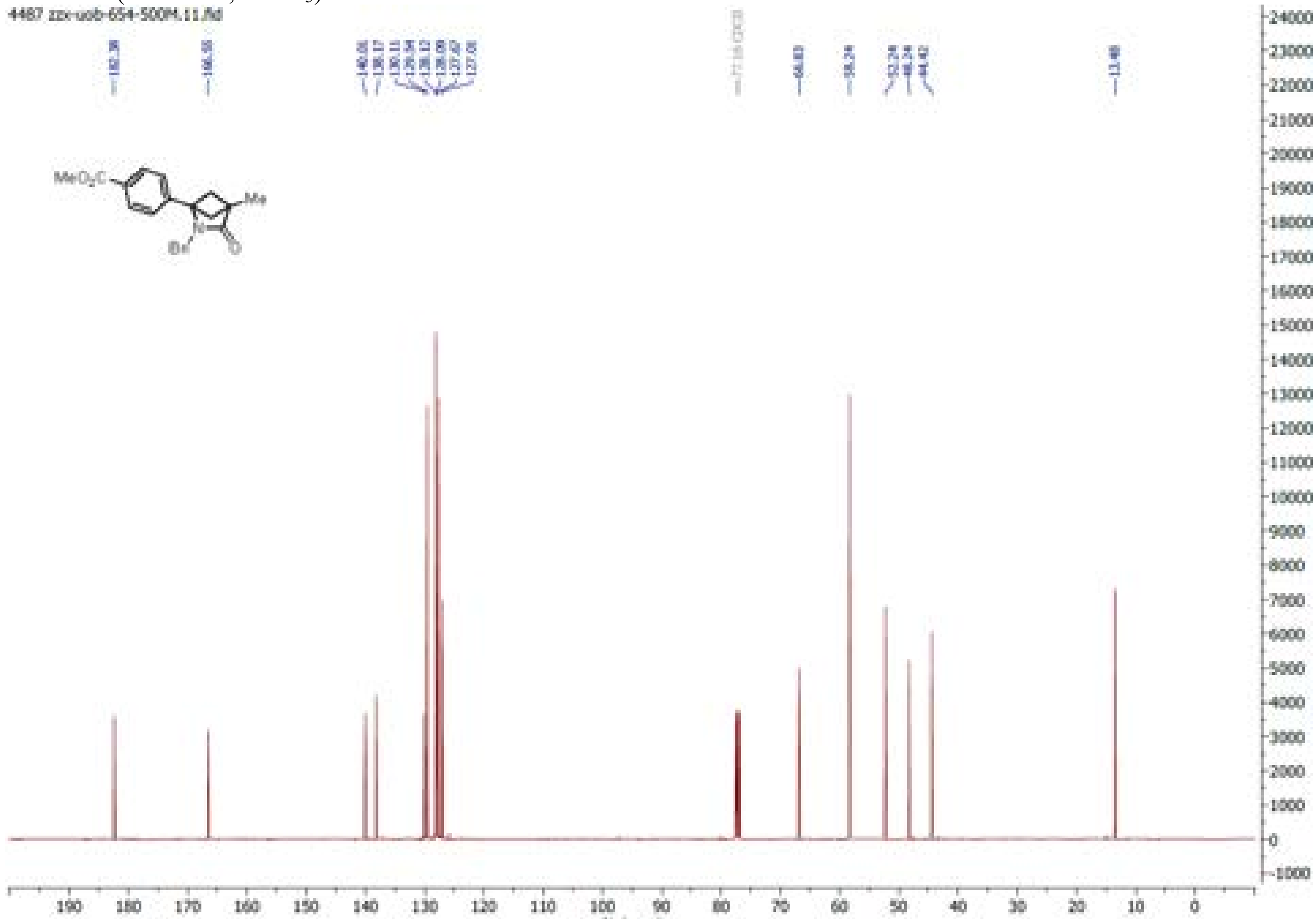

^{1}H NMR (500 MHz, $CDCl_3$) of **3f**

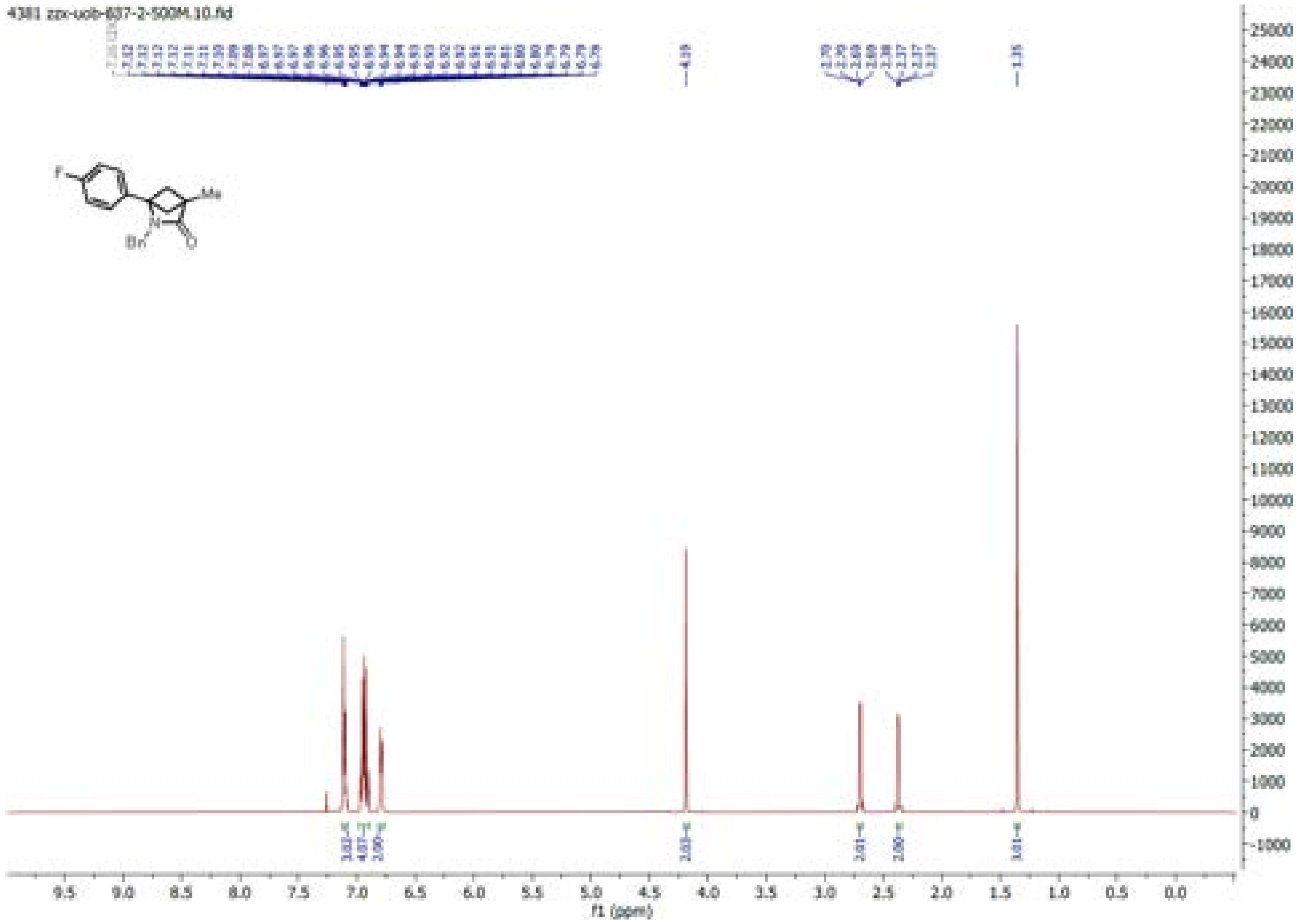

^{13}C NMR (126 MHz, $CDCl_3$) of **3f**

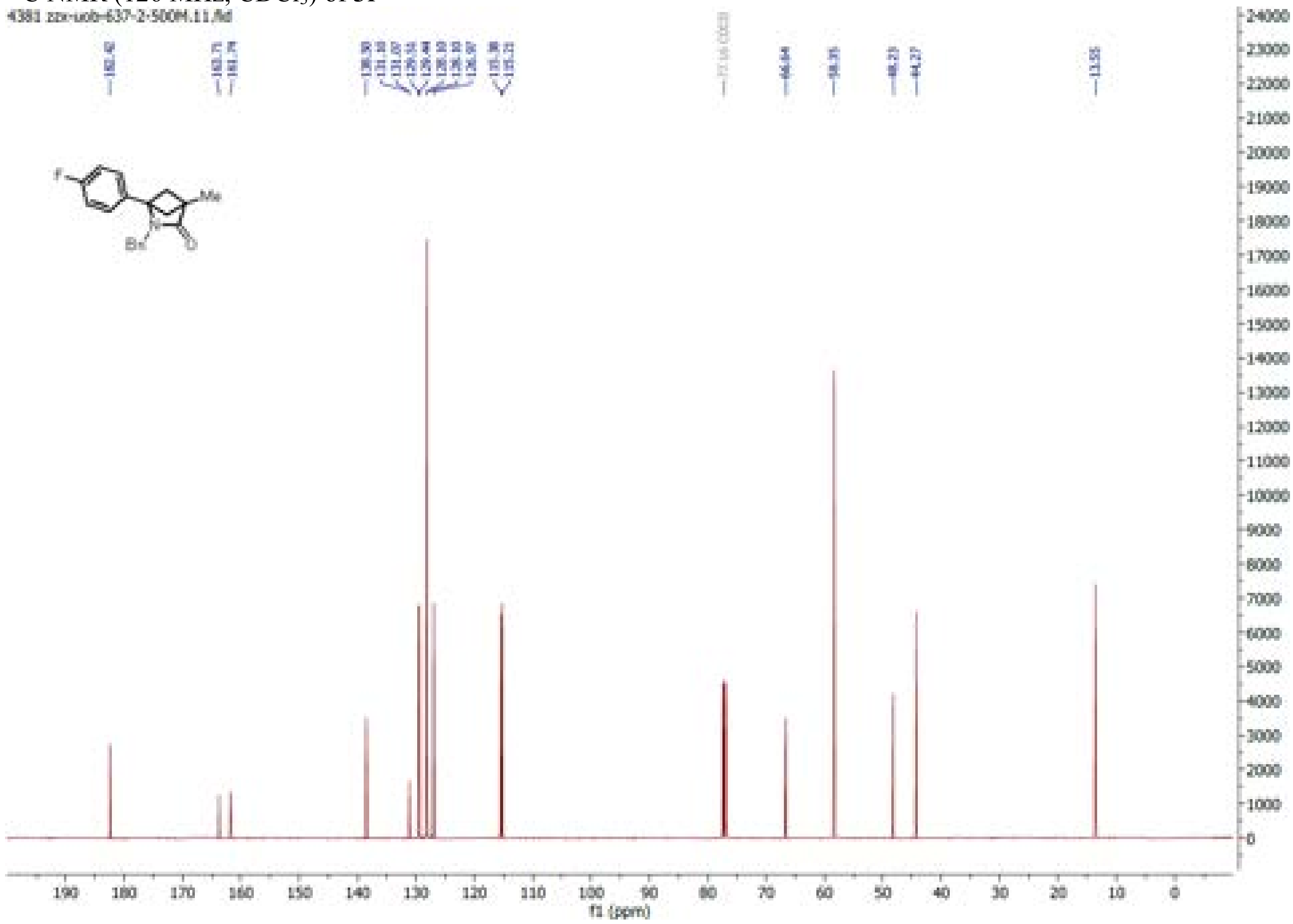

^{19}F NMR (377 MHz, $CDCl_3$) of **3f**

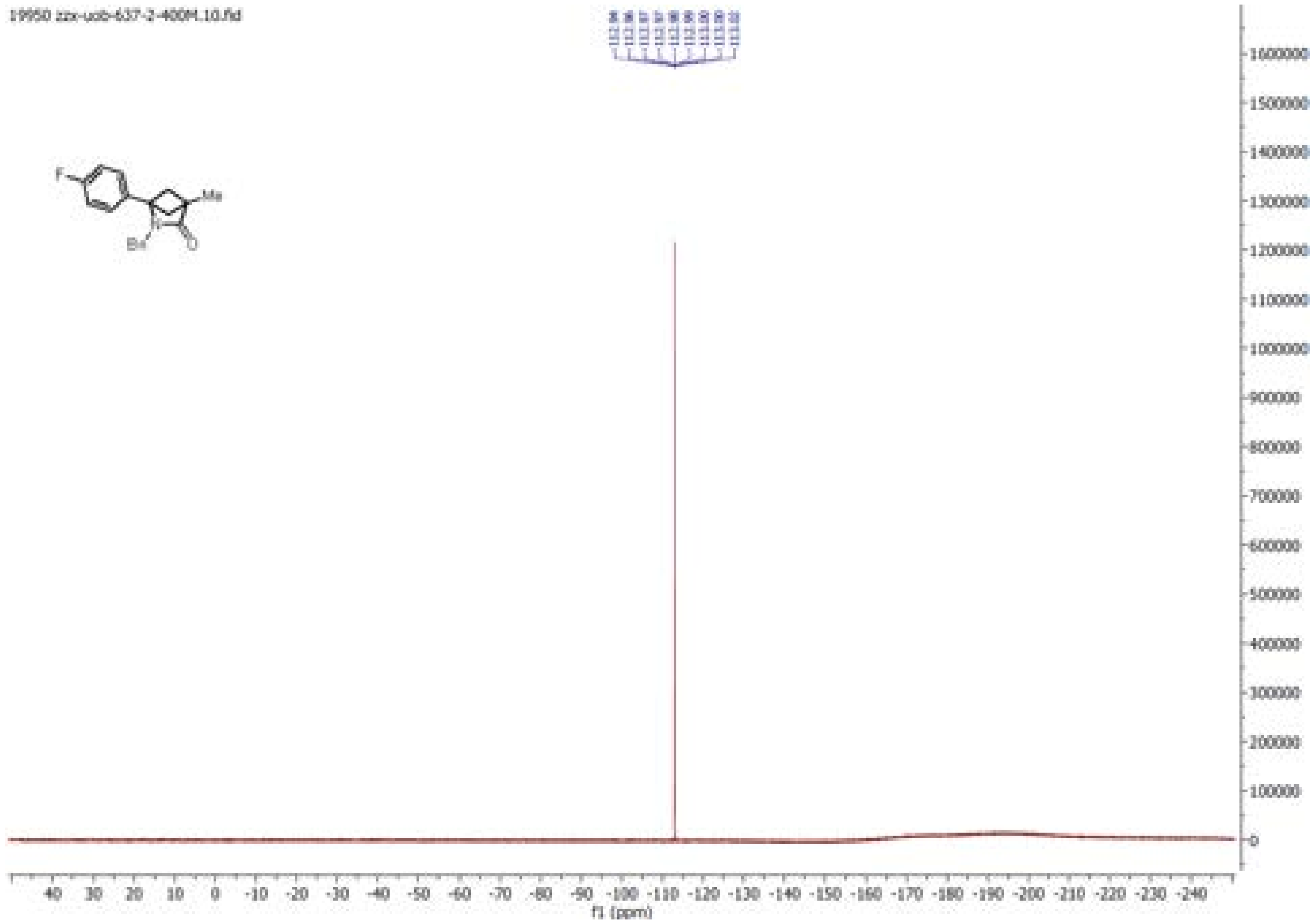

^{1}H NMR (500 MHz, $CDCl_3$) of **3g**

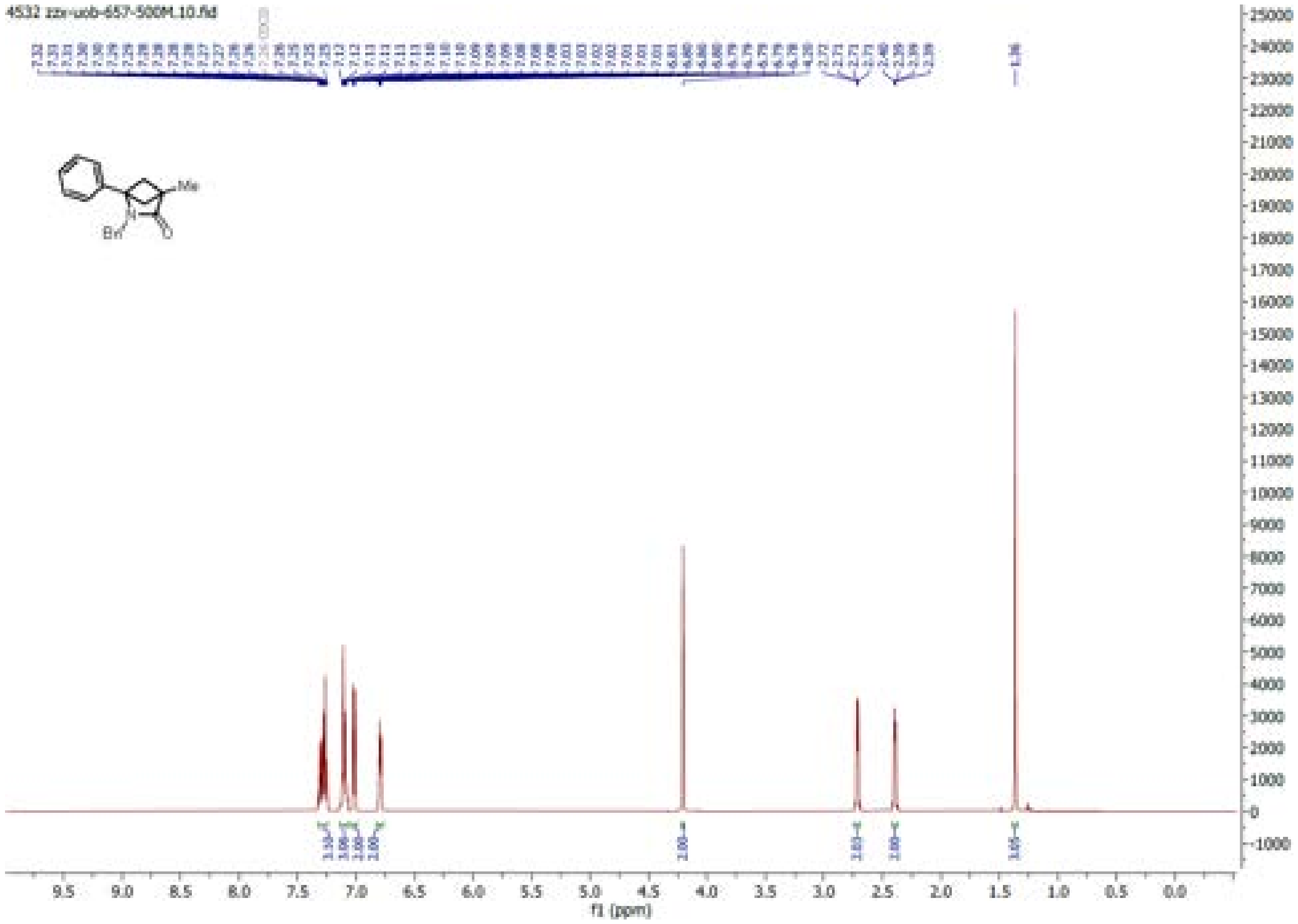

^{13}C NMR (126 MHz, $CDCl_3$) of **3g**

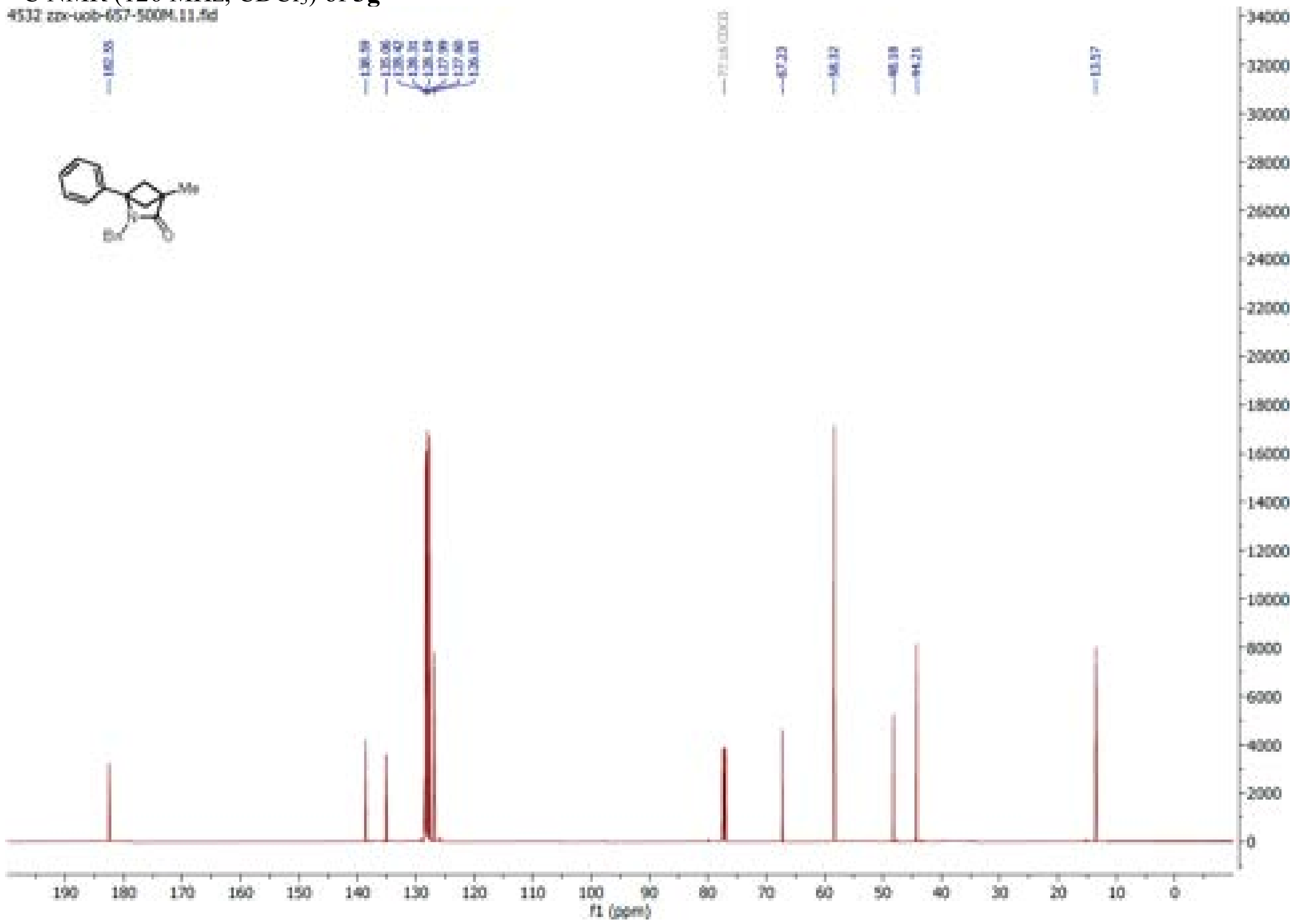

^{1}H NMR (500 MHz, $CDCl_3$) of **3h**

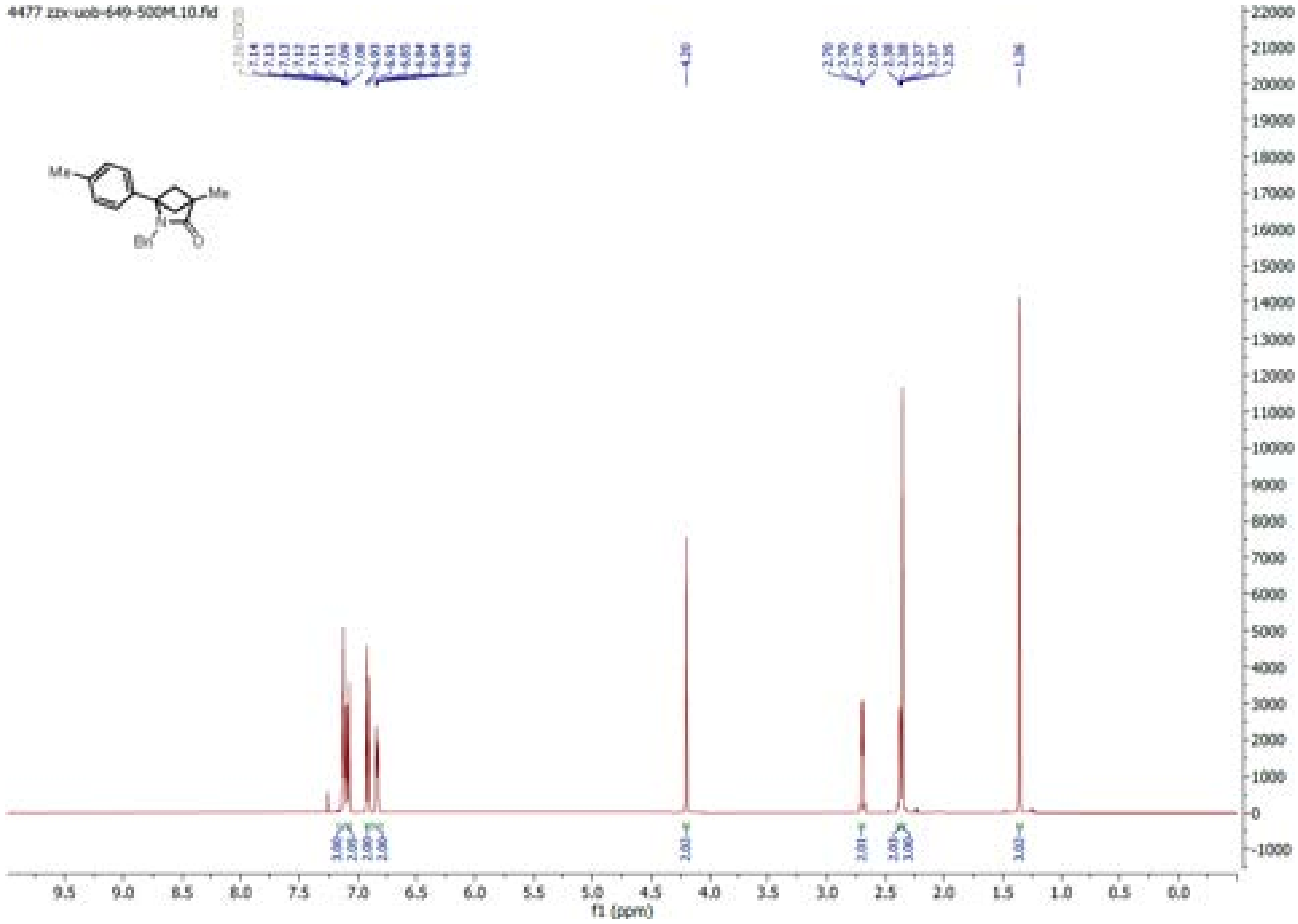

^{13}C NMR (126 MHz, $CDCl_3$) of **3h**

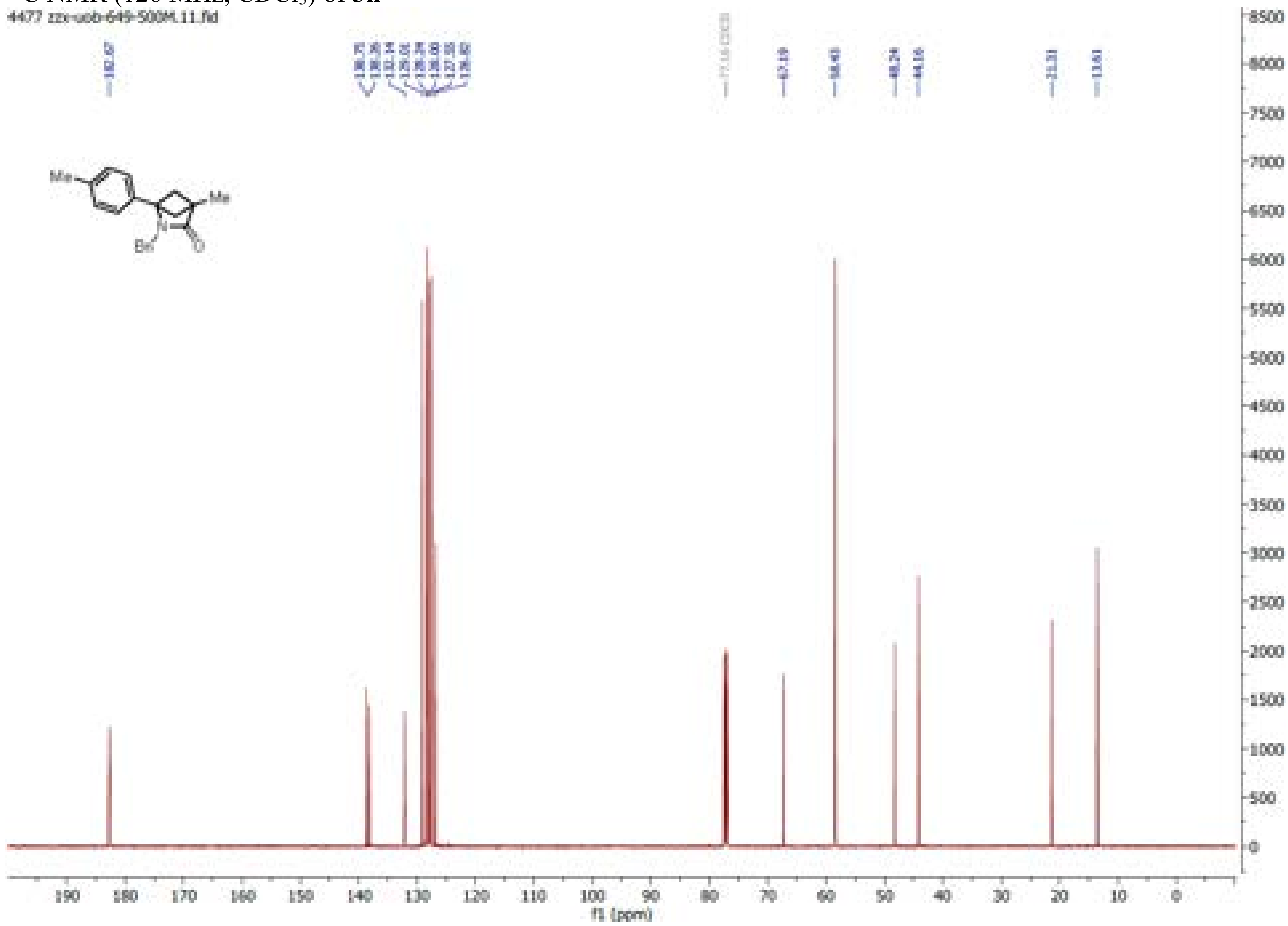

^{1}H NMR (500 MHz, $CDCl_3$) of **3i**

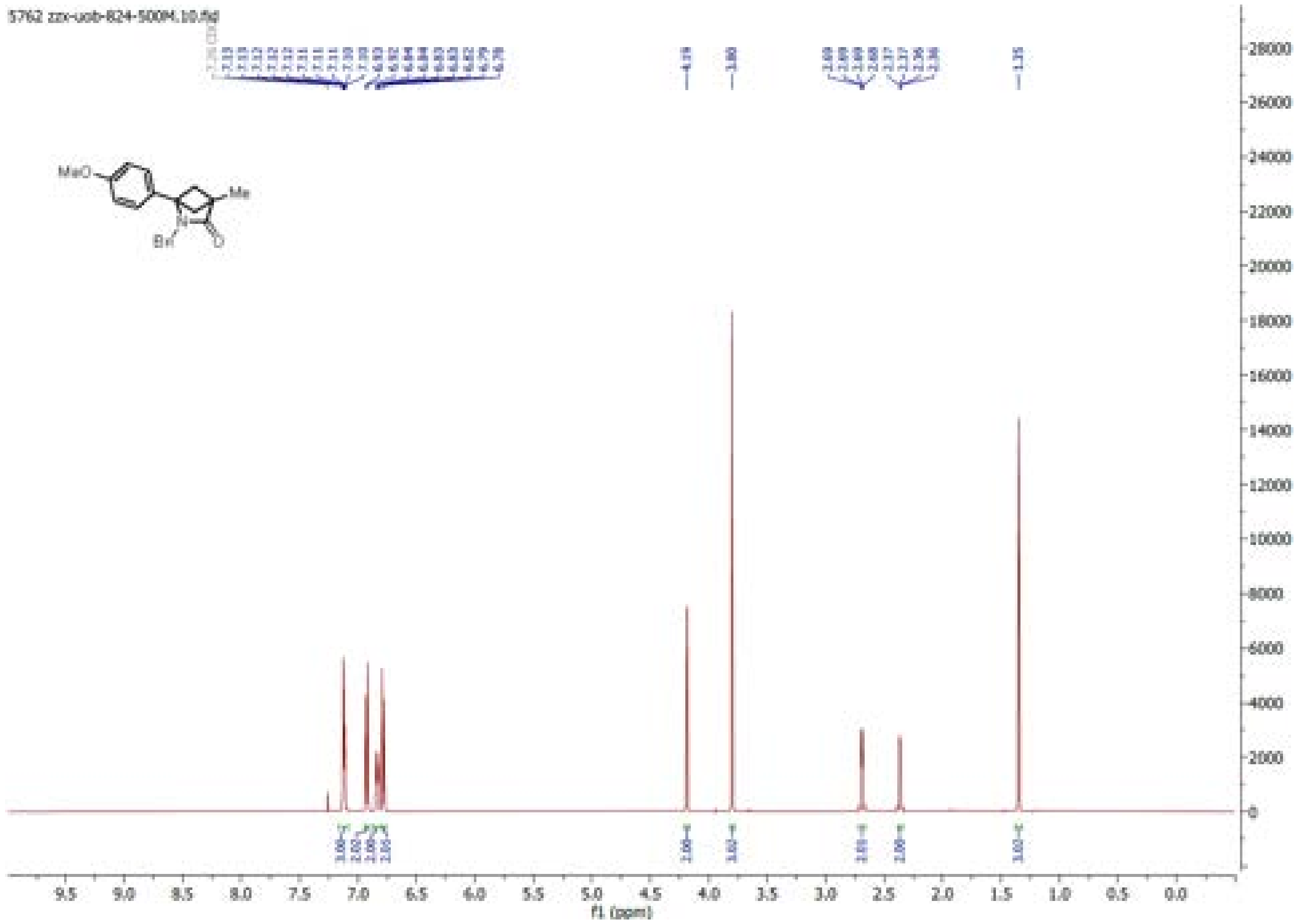

^{13}C NMR (126 MHz, $CDCl_3$) of **3i**

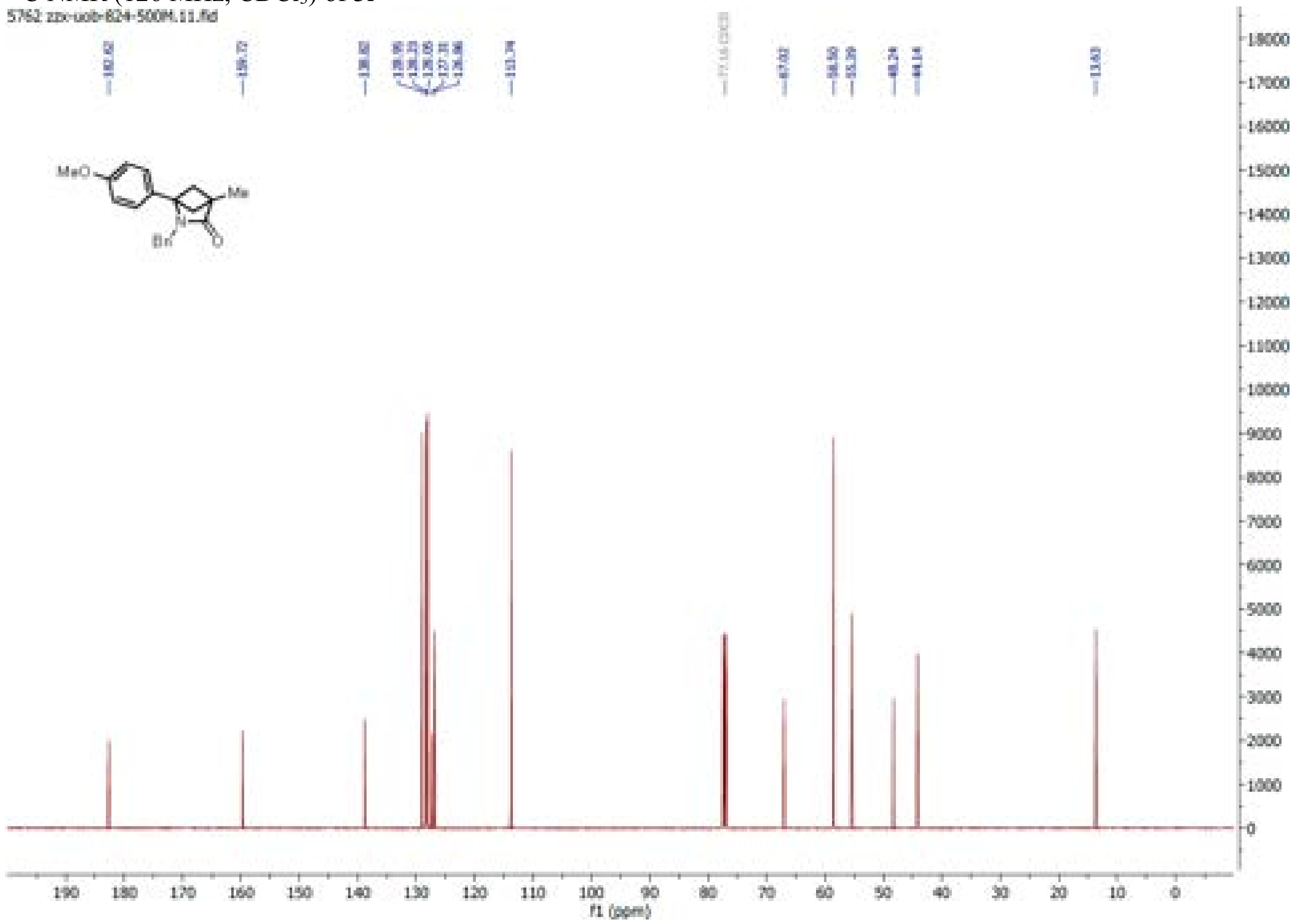

^{1}H NMR (500 MHz, $CDCl_3$) of **3j**

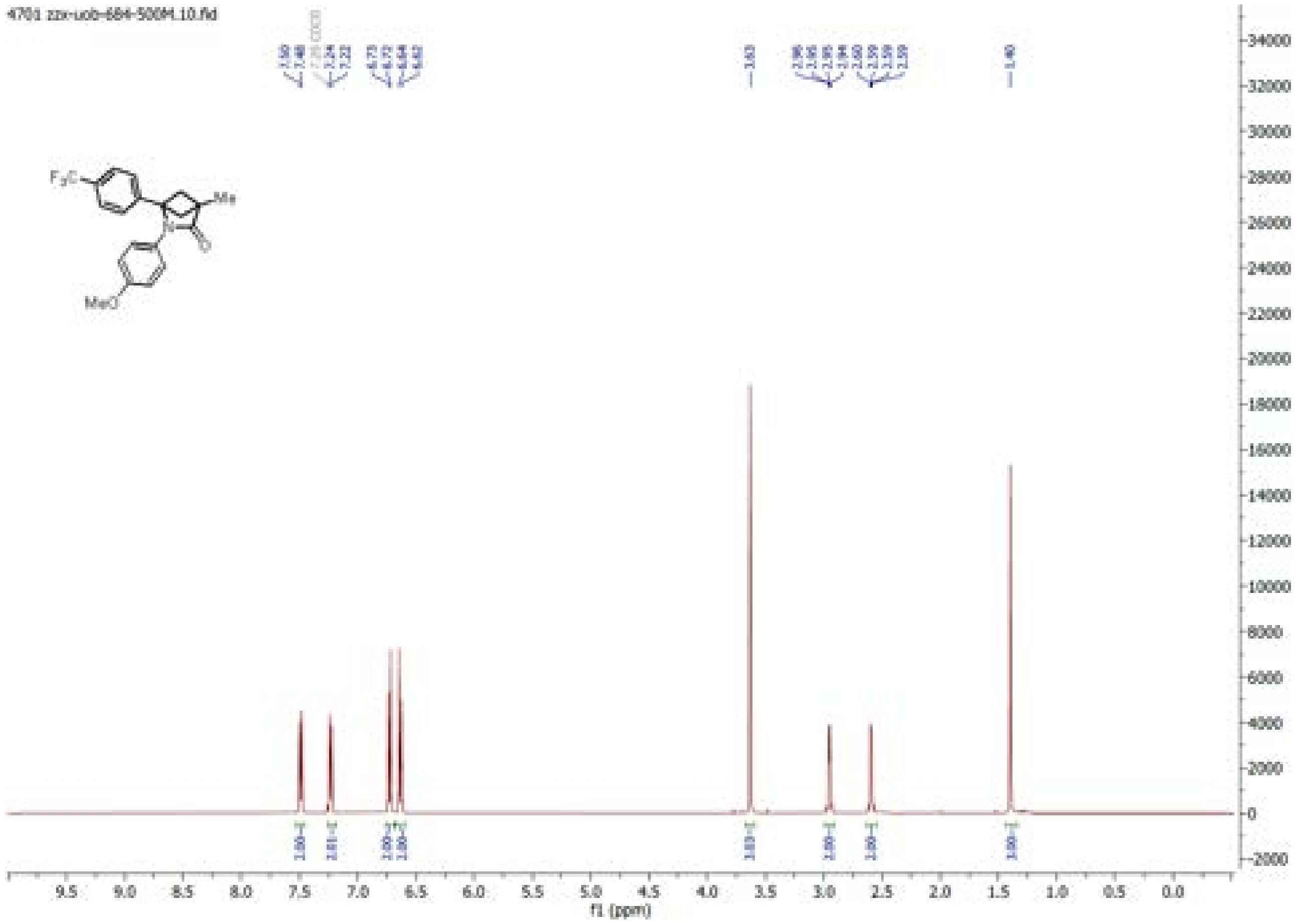

^{13}C NMR (126 MHz, $CDCl_3$) of **3j**

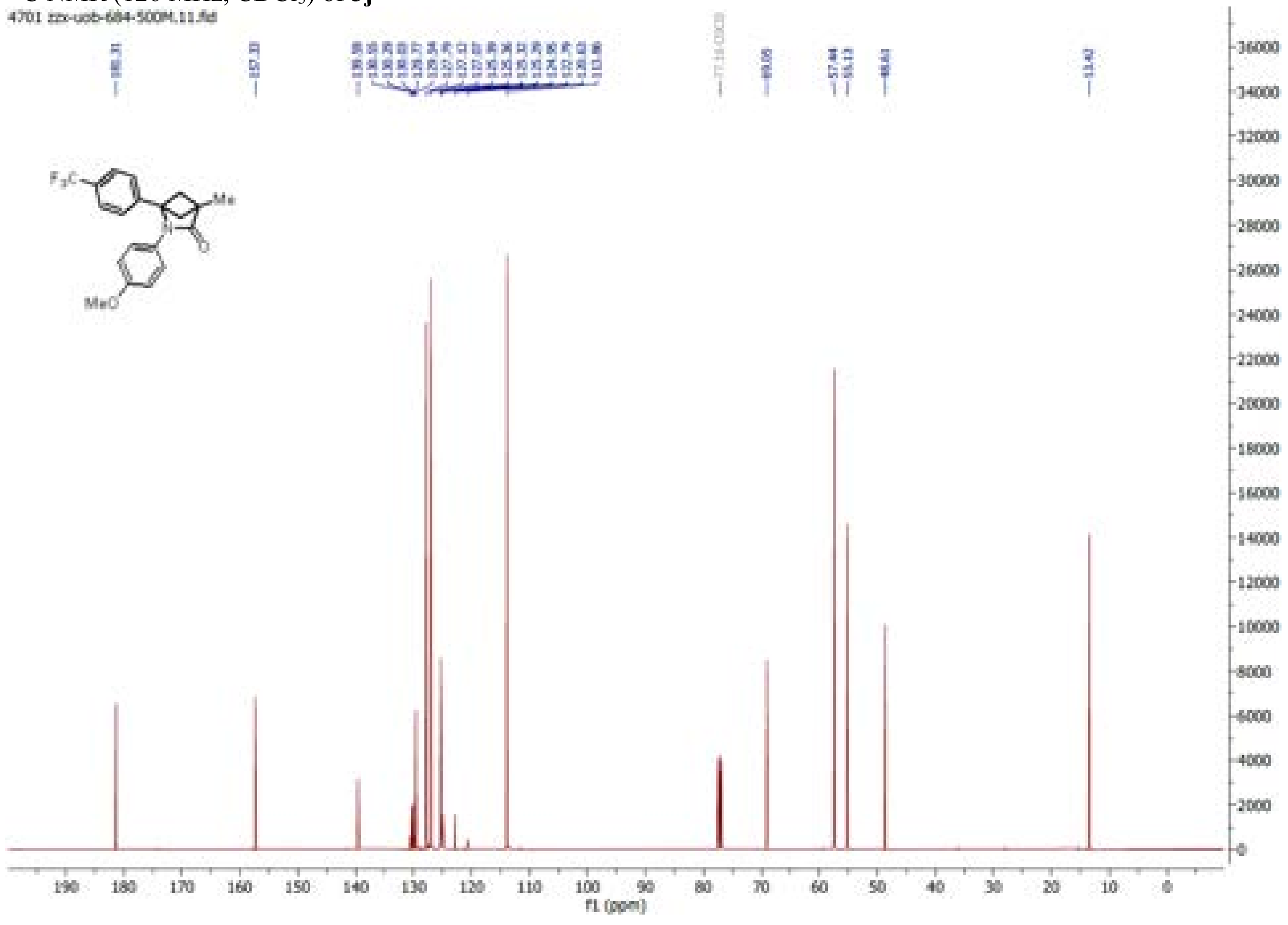

^{19}F NMR (471 MHz, $CDCl_3$) of **3j**

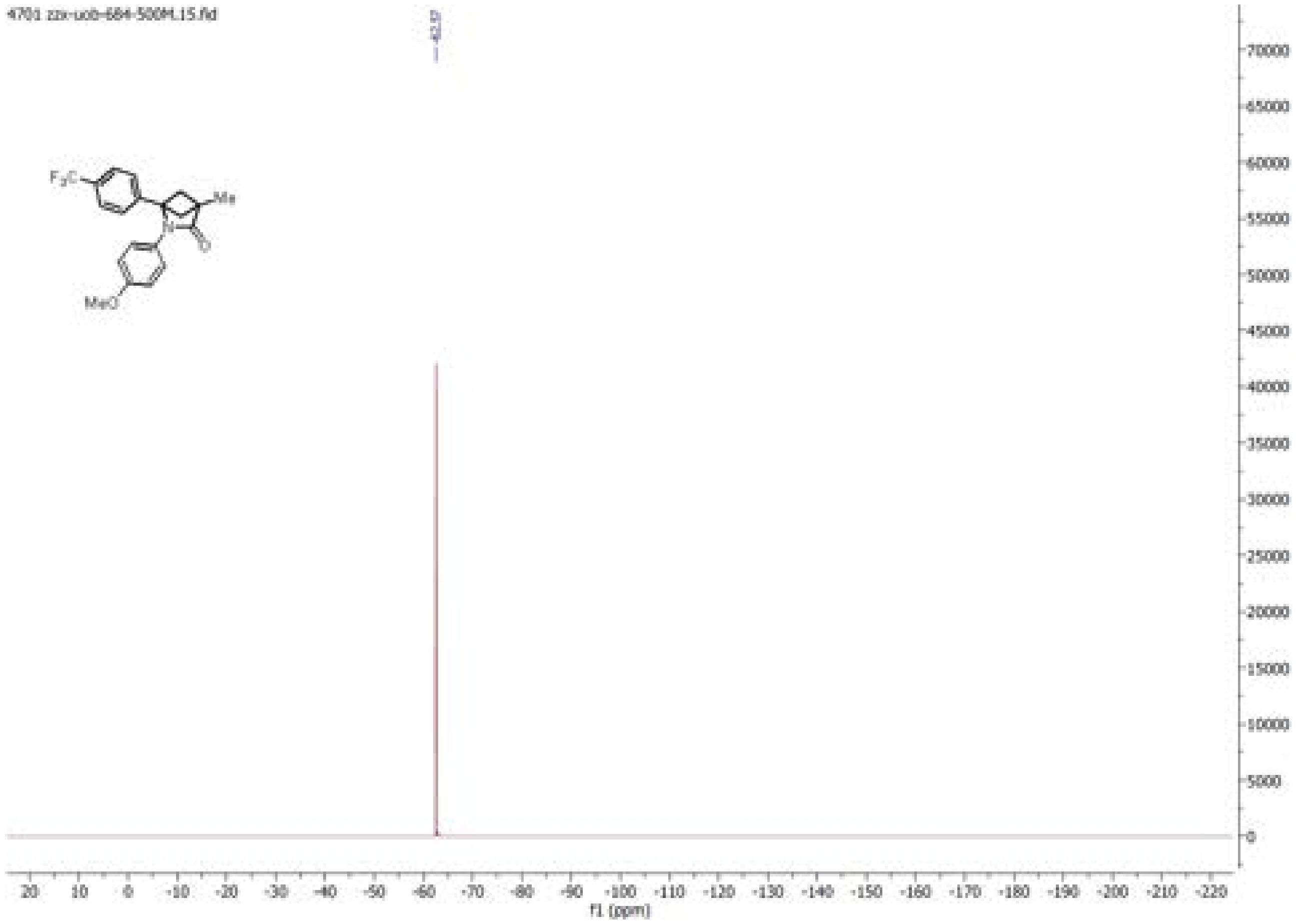

^{1}H NMR (500 MHz, $CDCl_3$) of **3k**

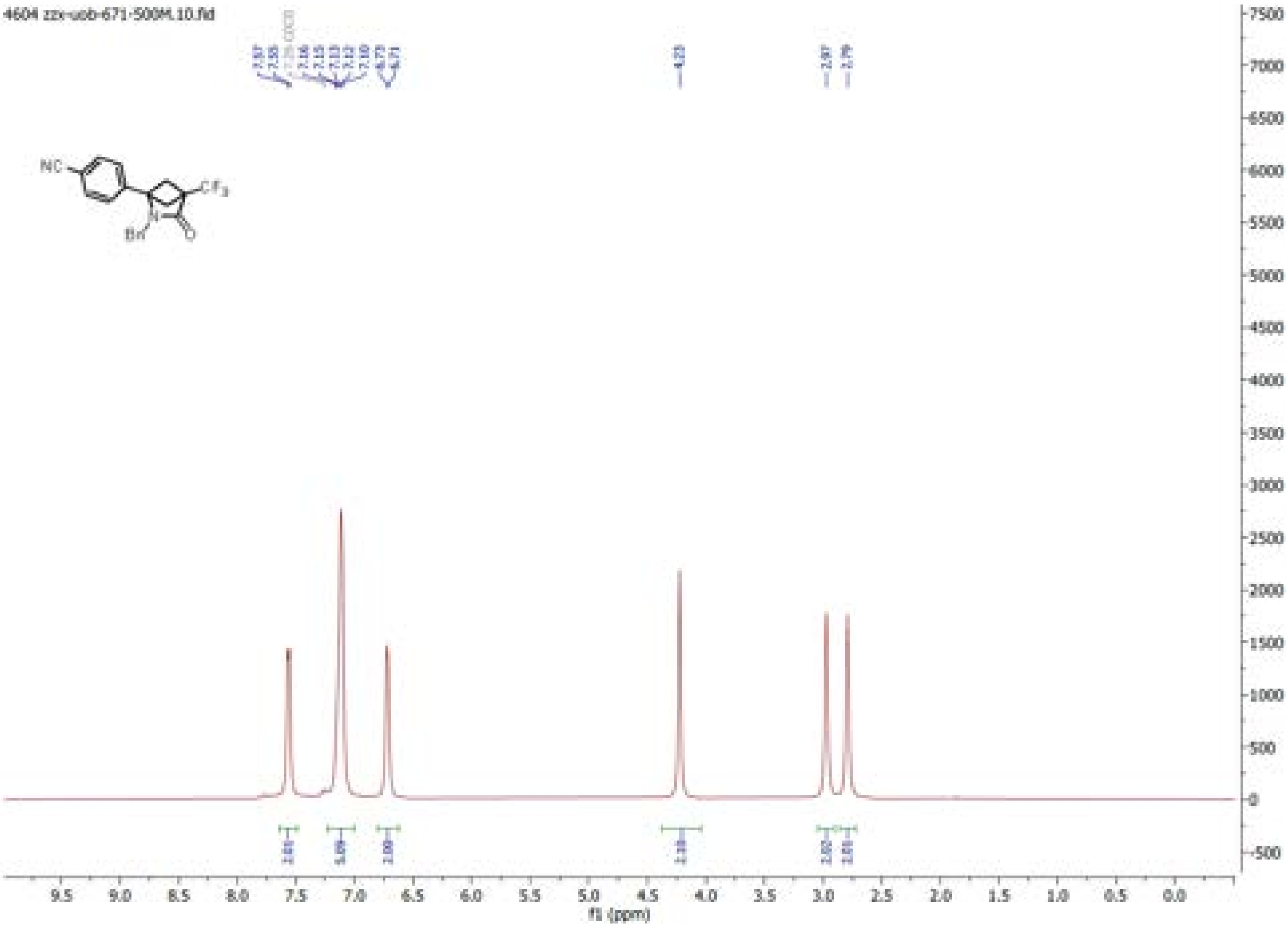

^{13}C NMR (126 MHz, $CDCl_3$) of **3k**

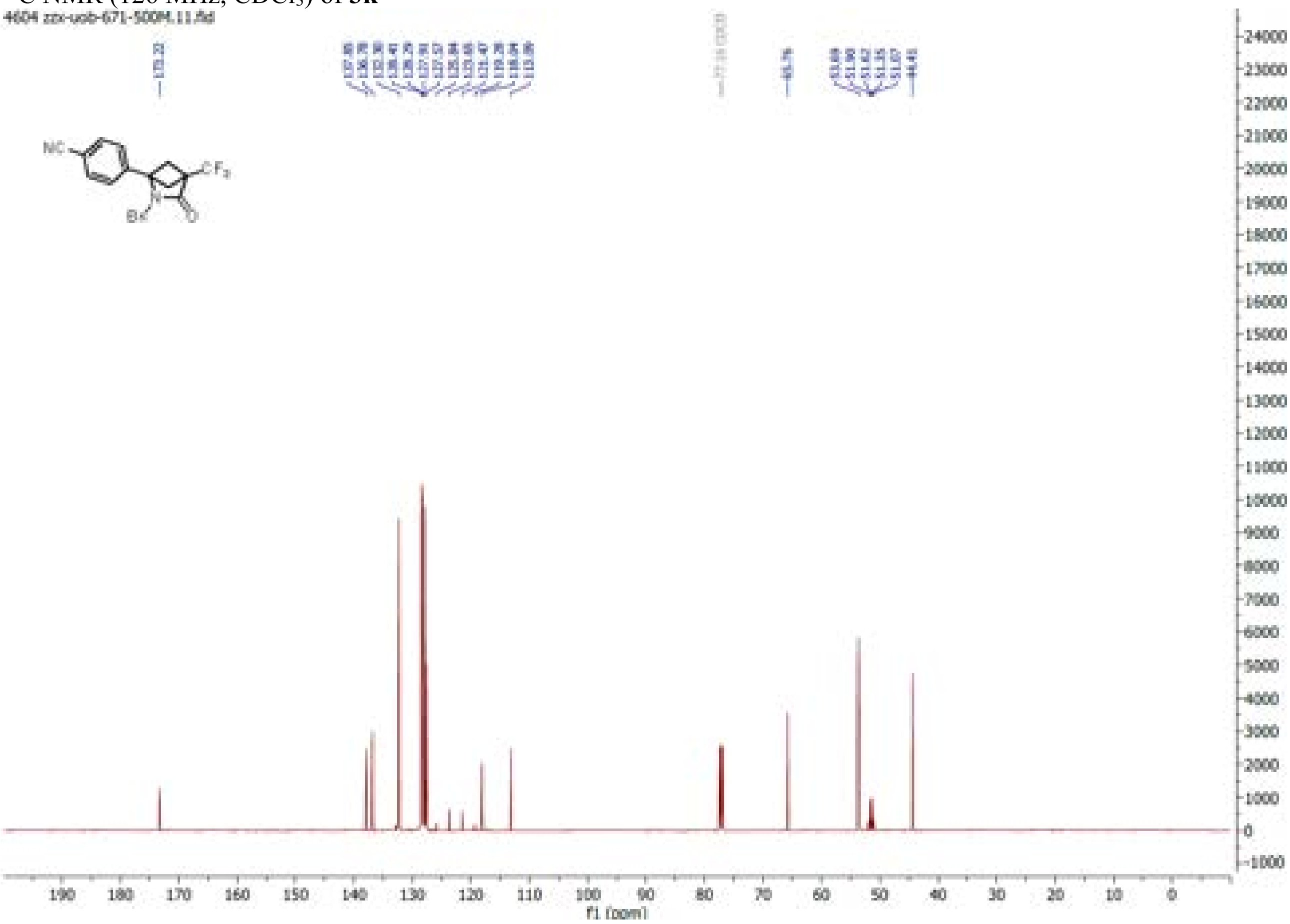

^{19}F NMR (471 MHz, $CDCl_3$) of **3k**

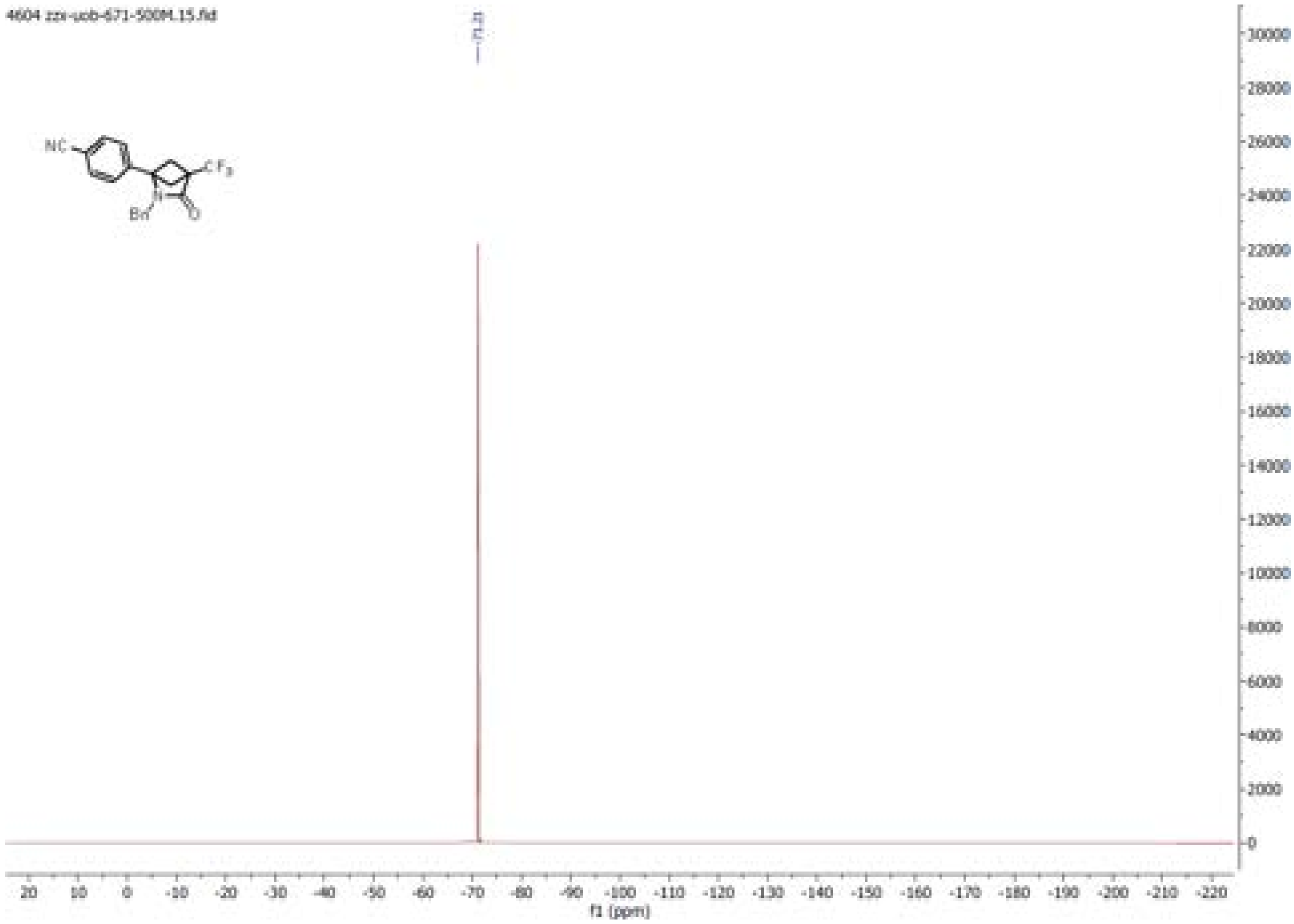

^1H NMR (500 MHz, $CDCl_3$) of **3l**

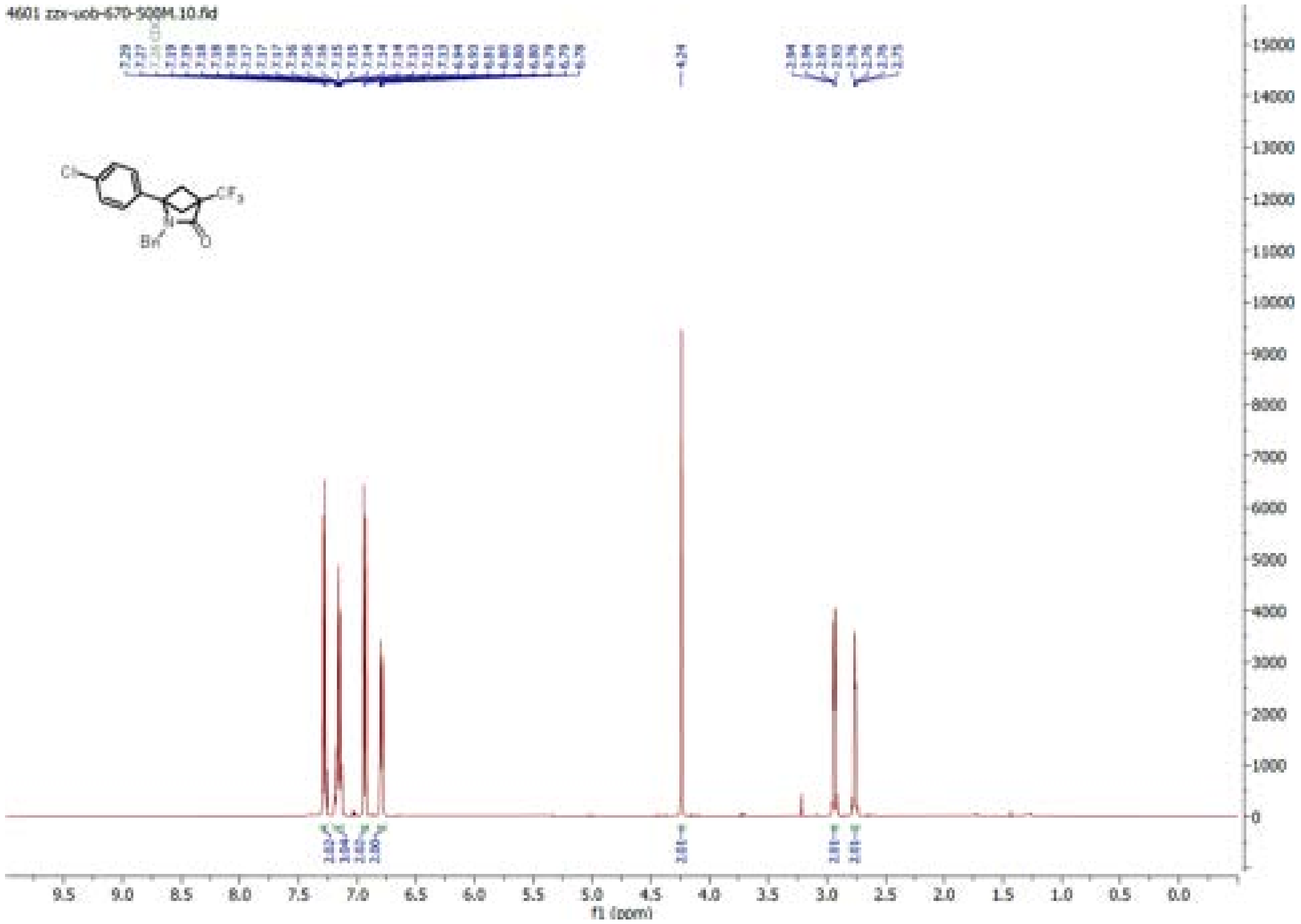

^{13}C NMR (126 MHz, $CDCl_3$) of **3l**

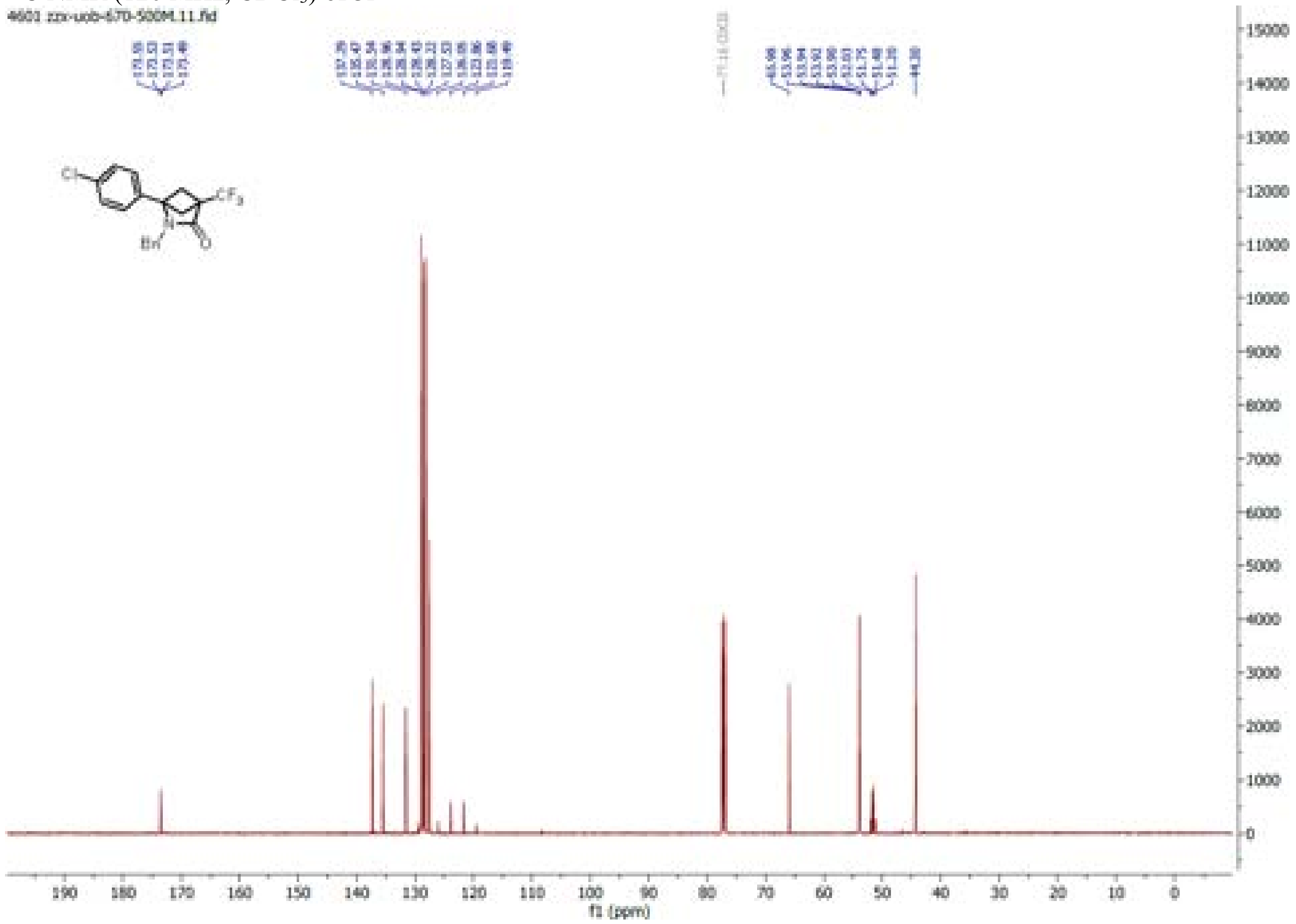

^{19}F NMR (471 MHz, $CDCl_3$) of **3l**

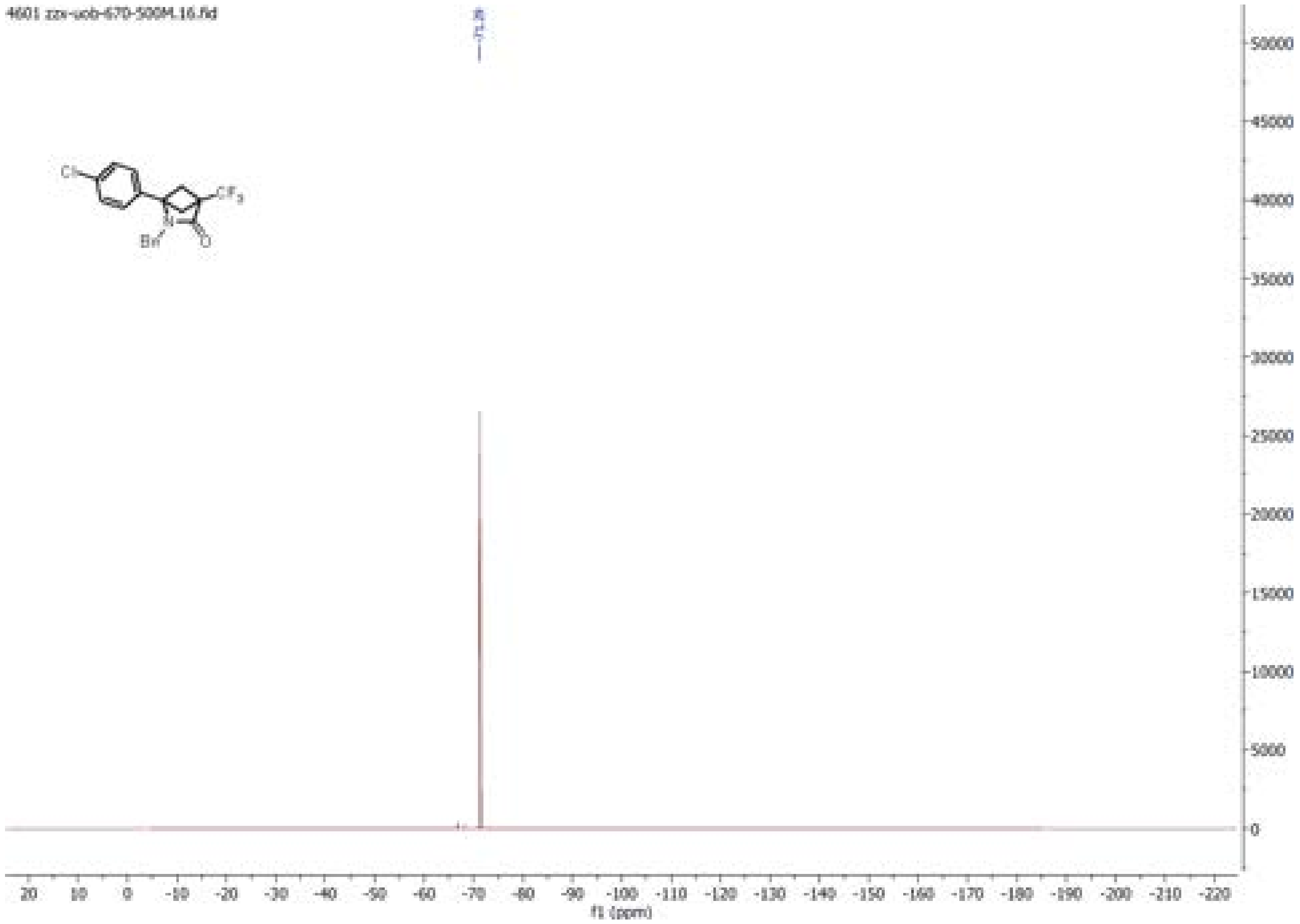

^{1}H NMR (500 MHz, $CDCl_3$) of **3m**

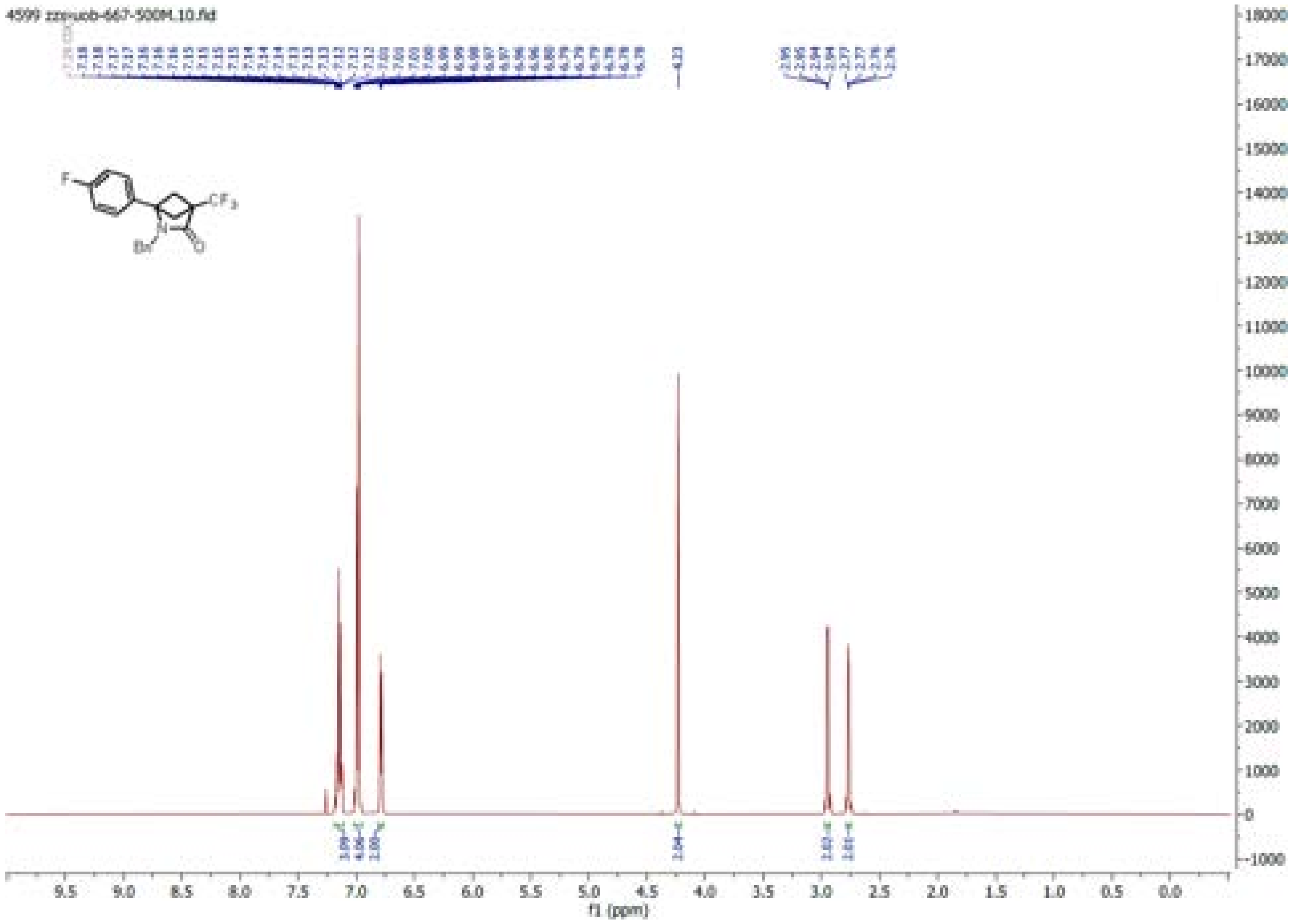

^{13}C NMR (126 MHz, $CDCl_3$) of **3m**

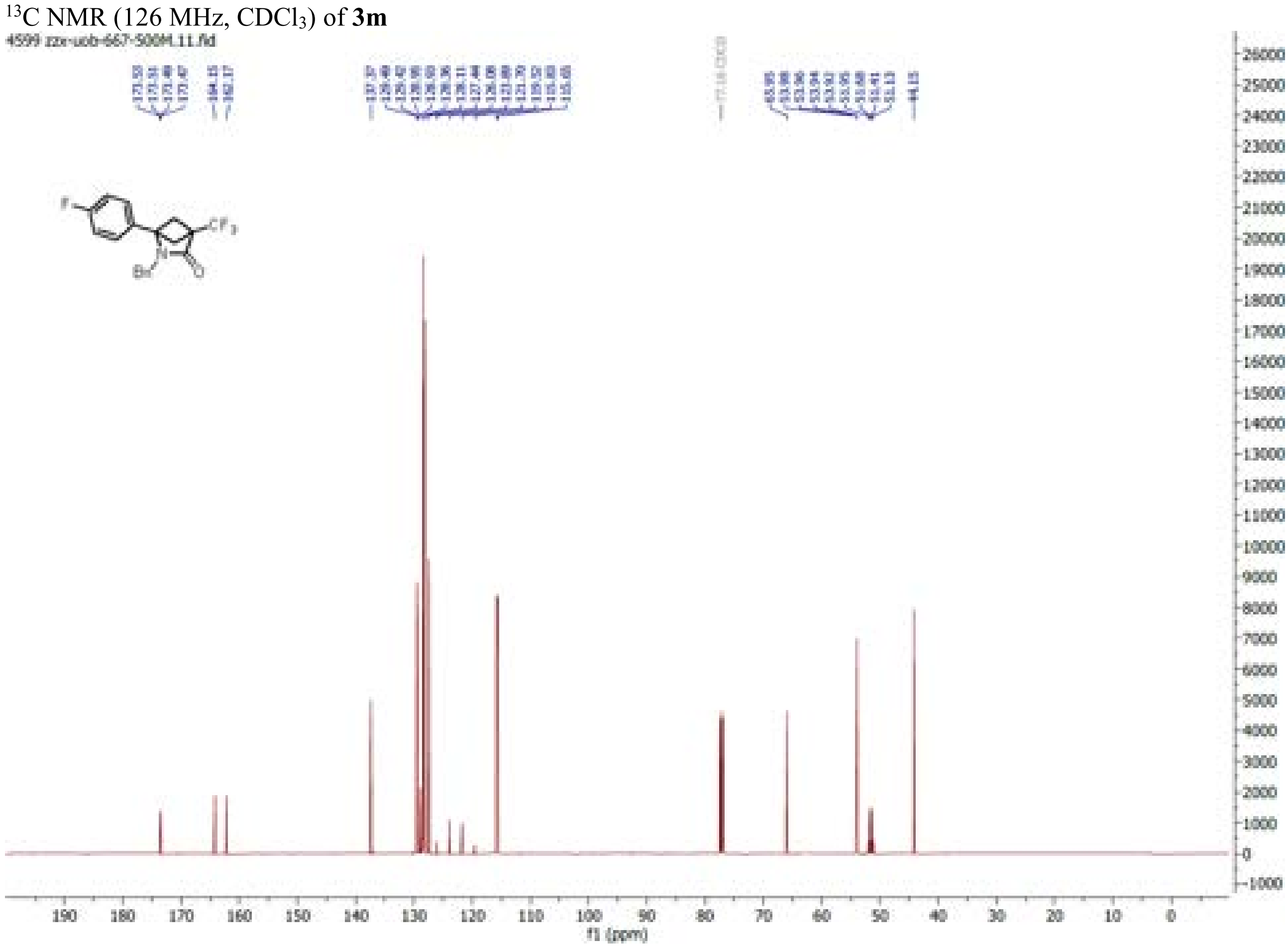

^{19}F NMR (471 MHz, $CDCl_3$) of **3m**

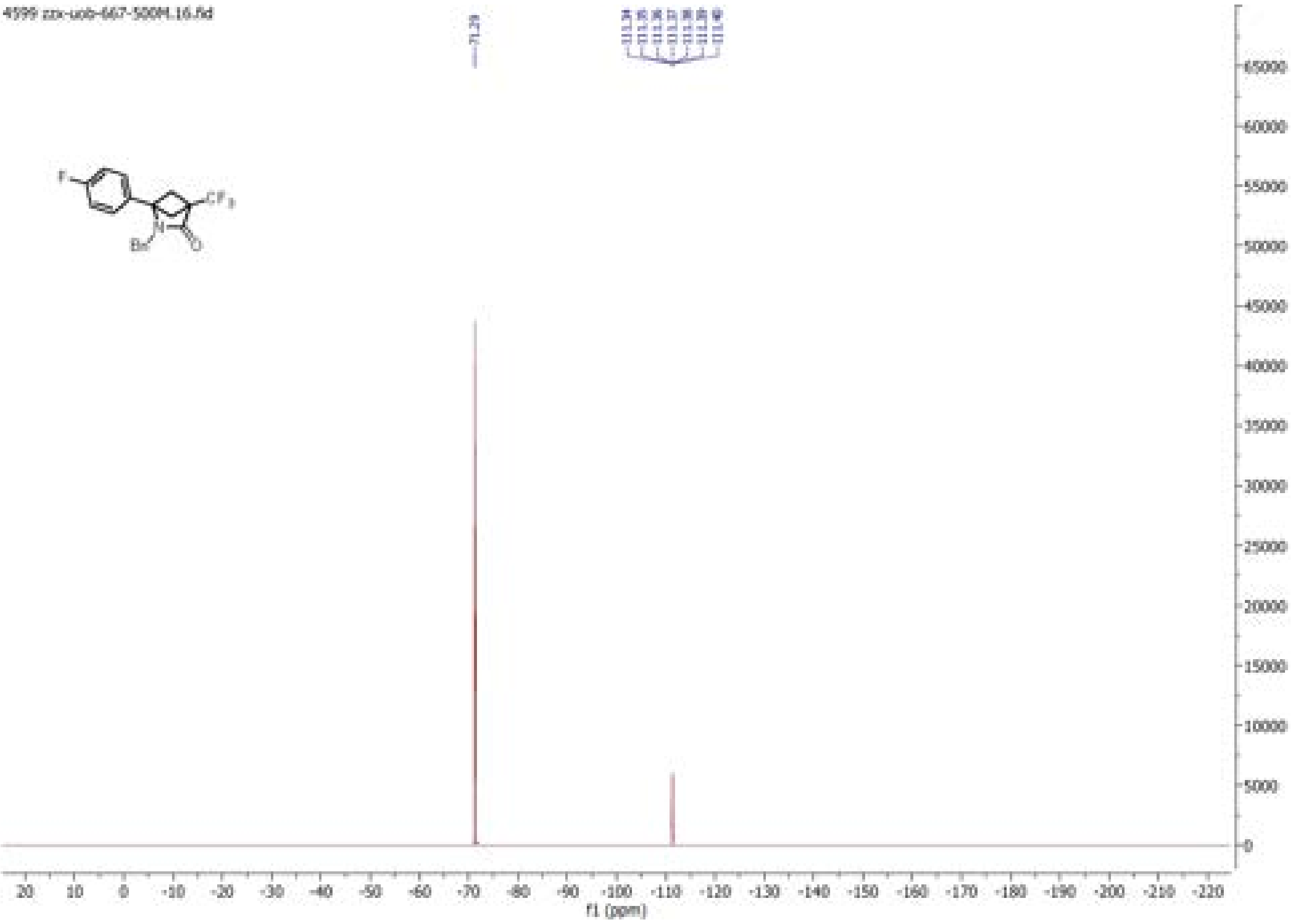

^{1}H NMR (500 MHz, $CDCl_3$) of **3n**

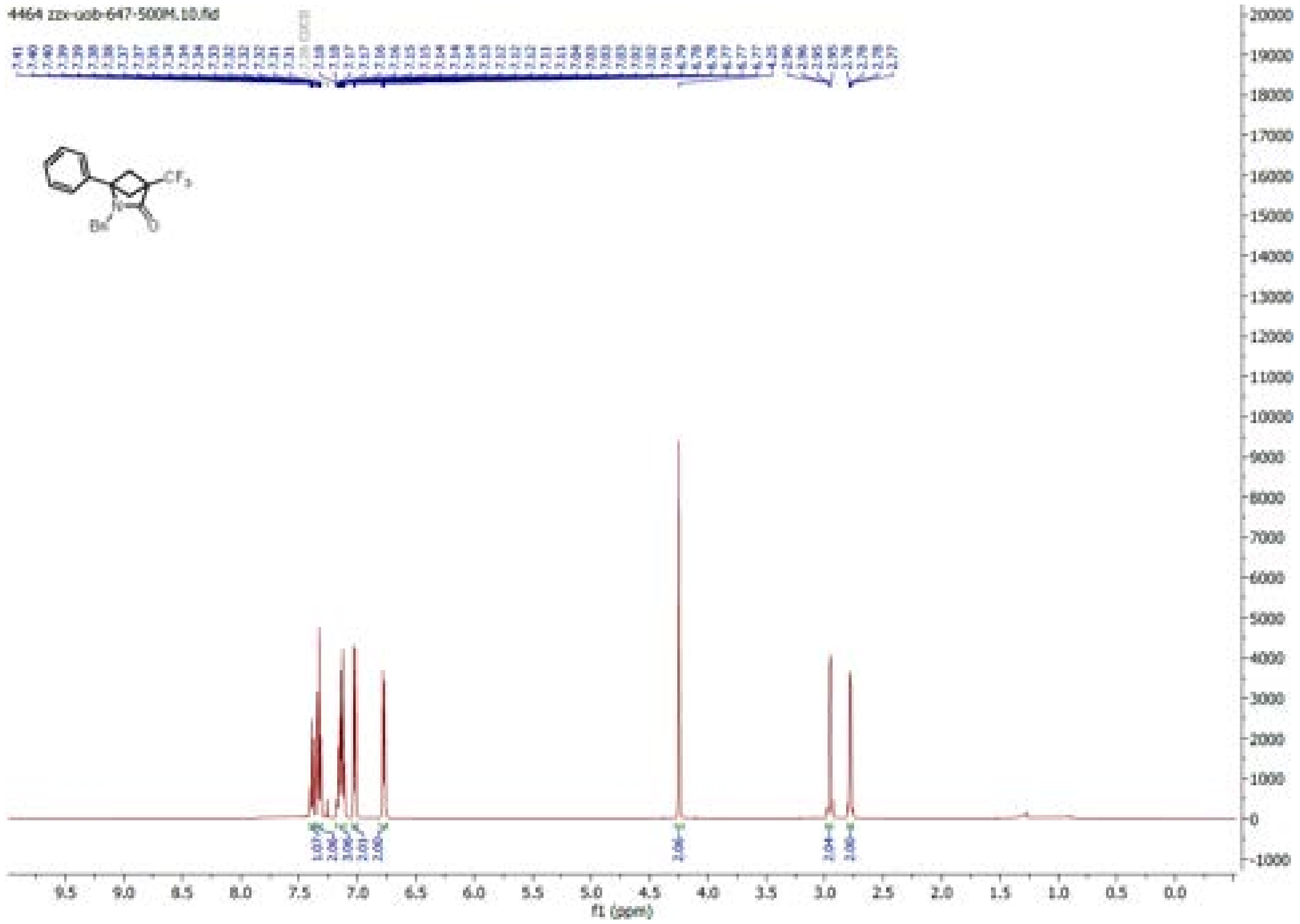

^{13}C NMR (126 MHz, $CDCl_3$) of **3n**

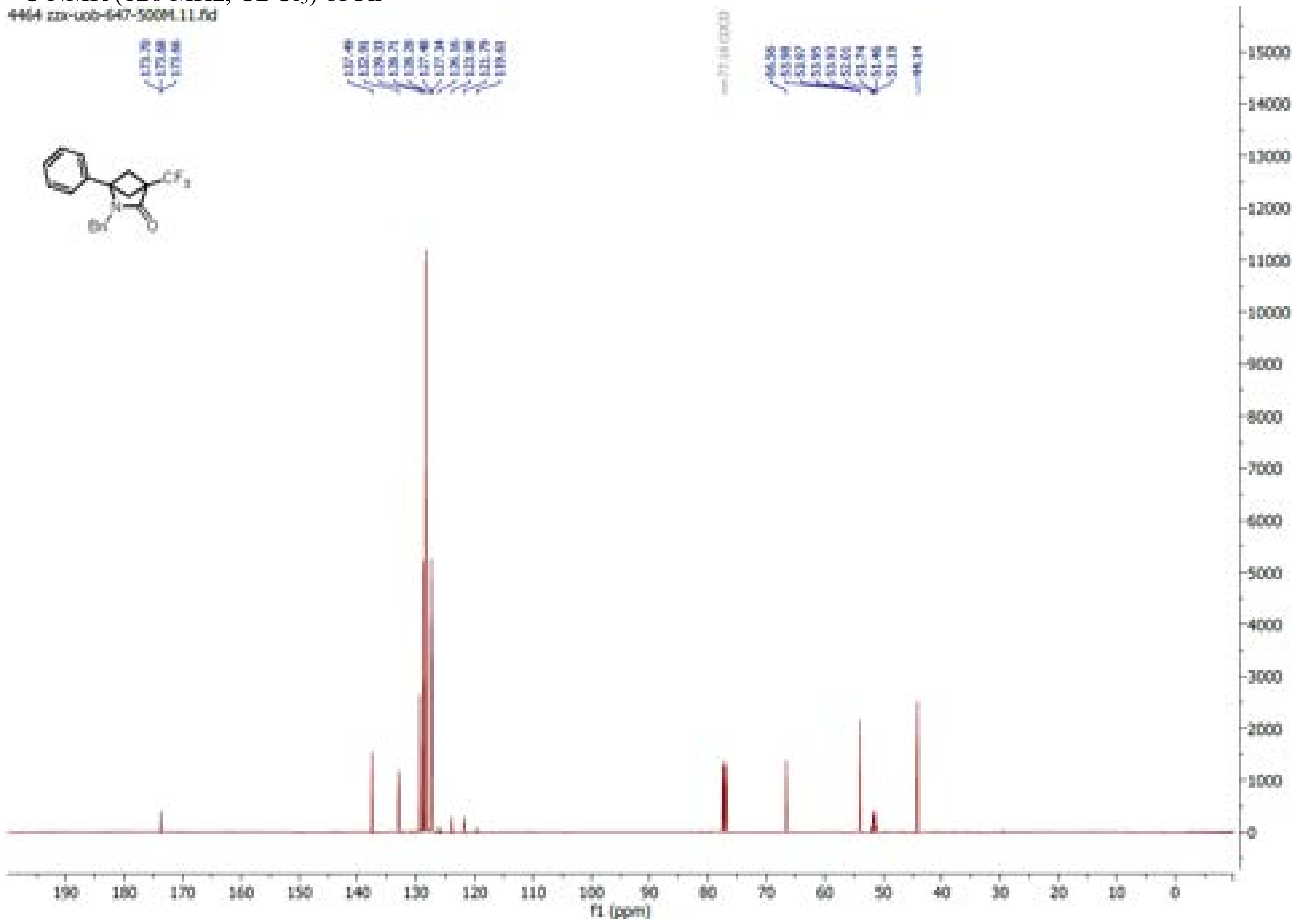

^{19}F NMR (471 MHz, $CDCl_3$) of **3n**

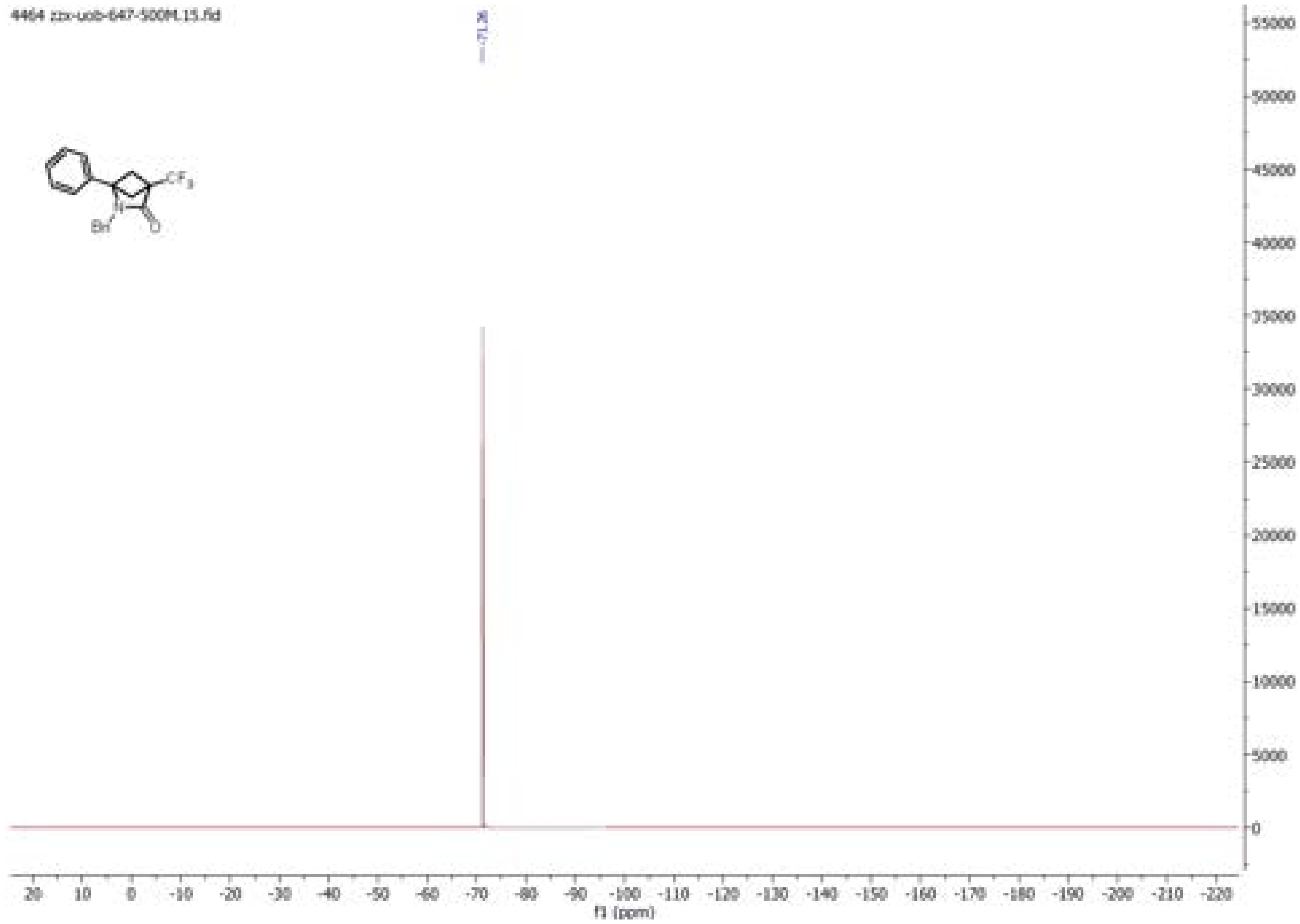

^{1}H NMR (500 MHz, $CDCl_3$) of **3o**

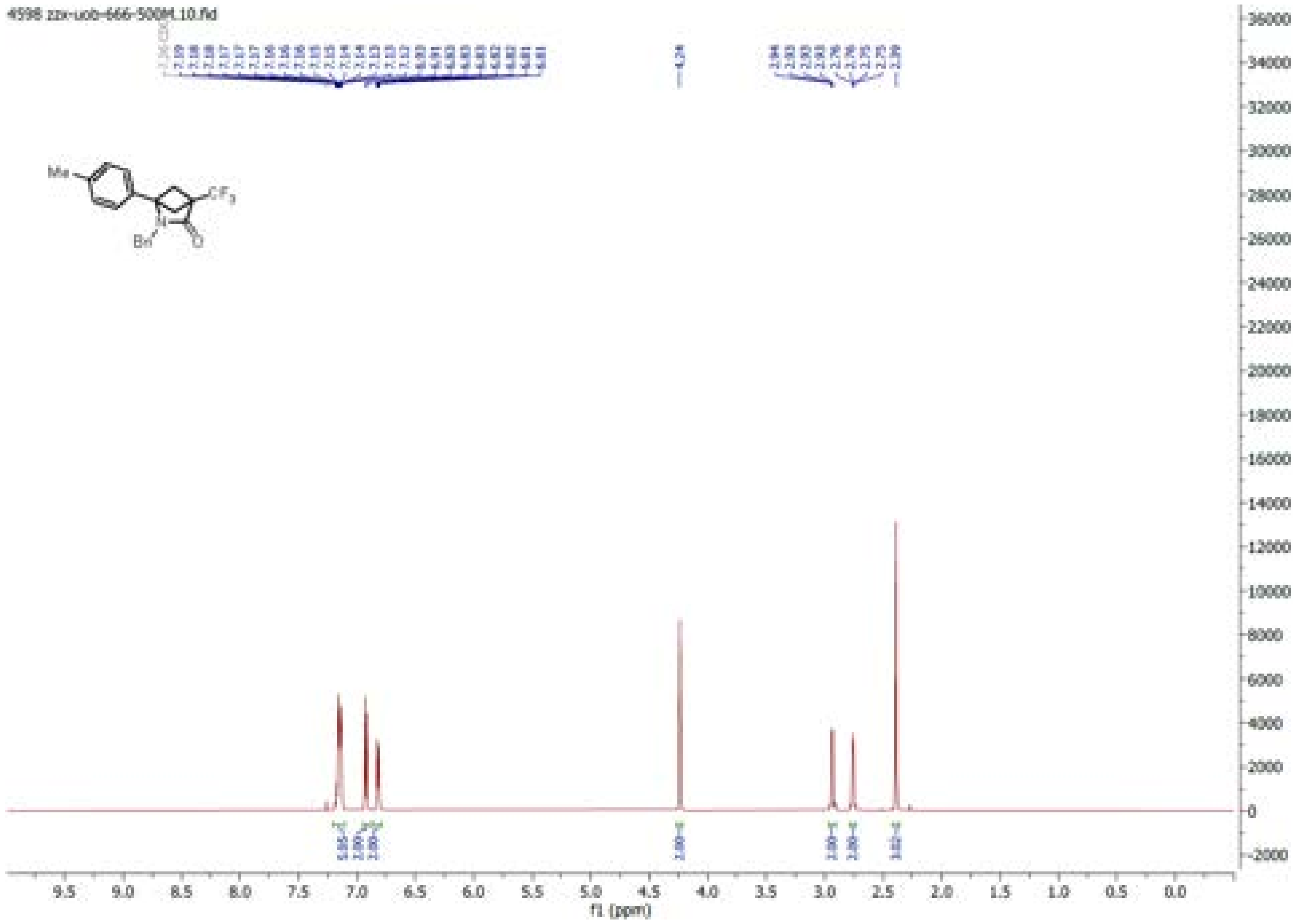

^{13}C NMR (126 MHz, $CDCl_3$) of **3o**

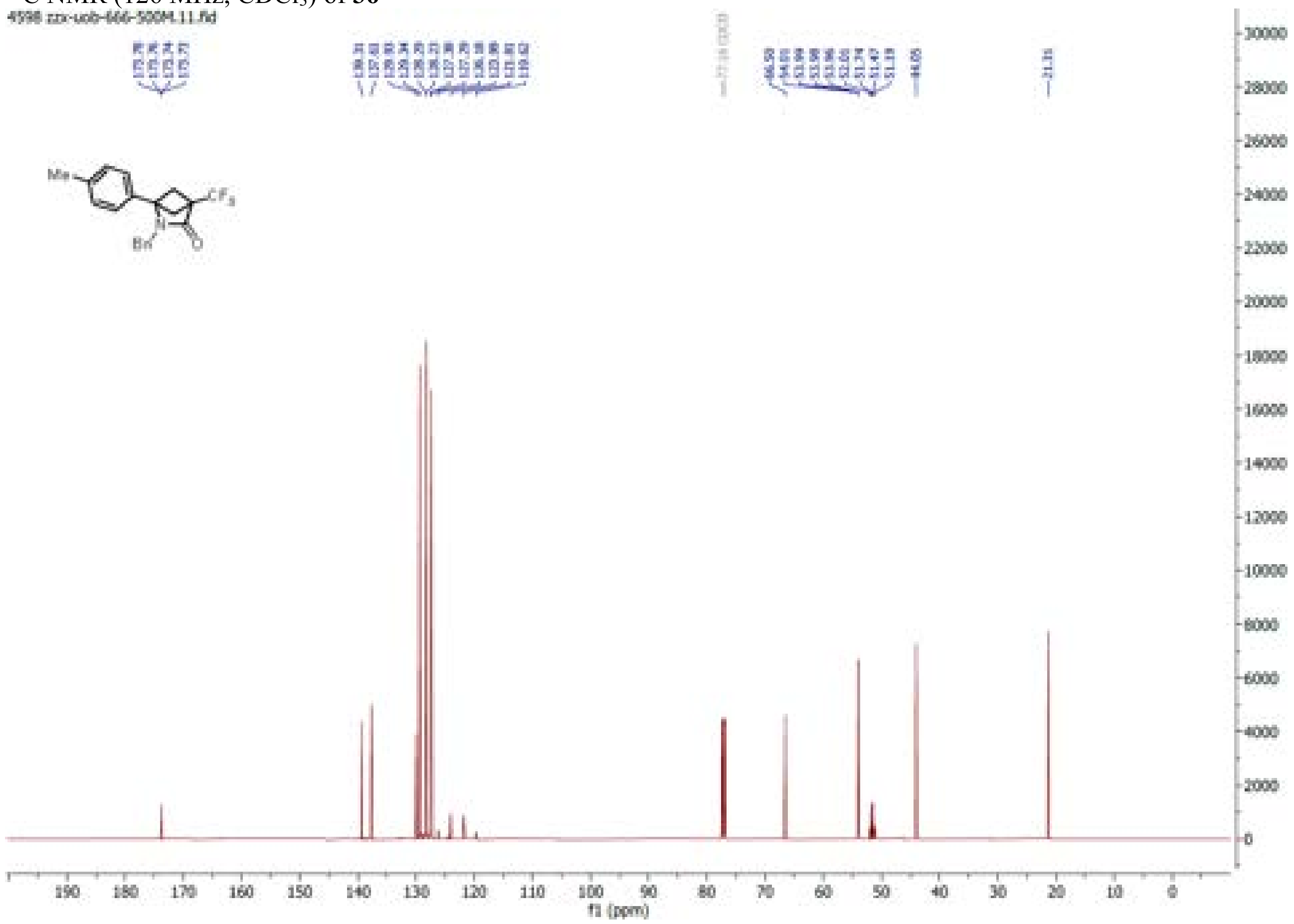

^{19}F NMR (471 MHz, $CDCl_3$) of **3o**

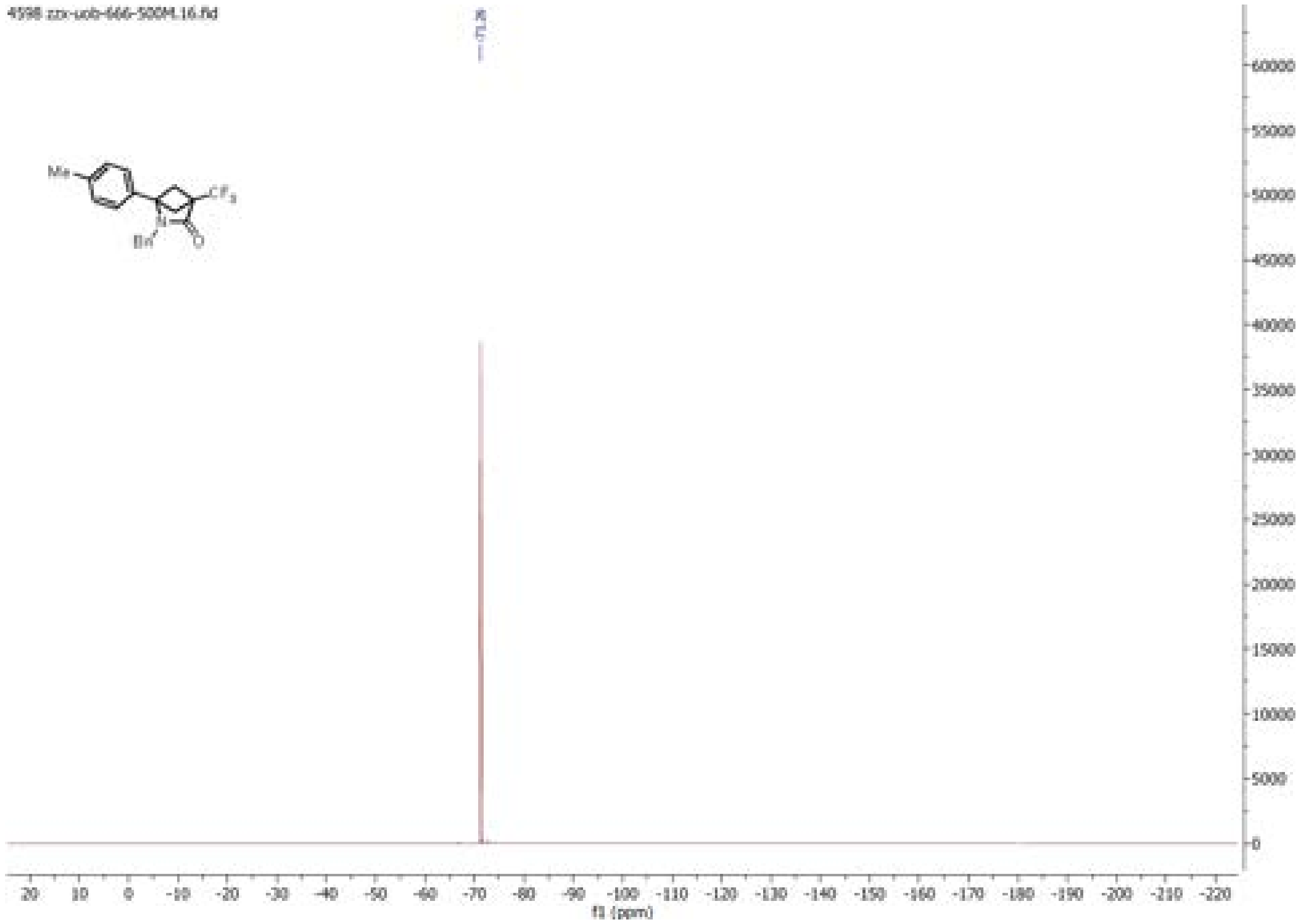

^{1}H NMR (500 MHz, $CDCl_3$) of **3p**

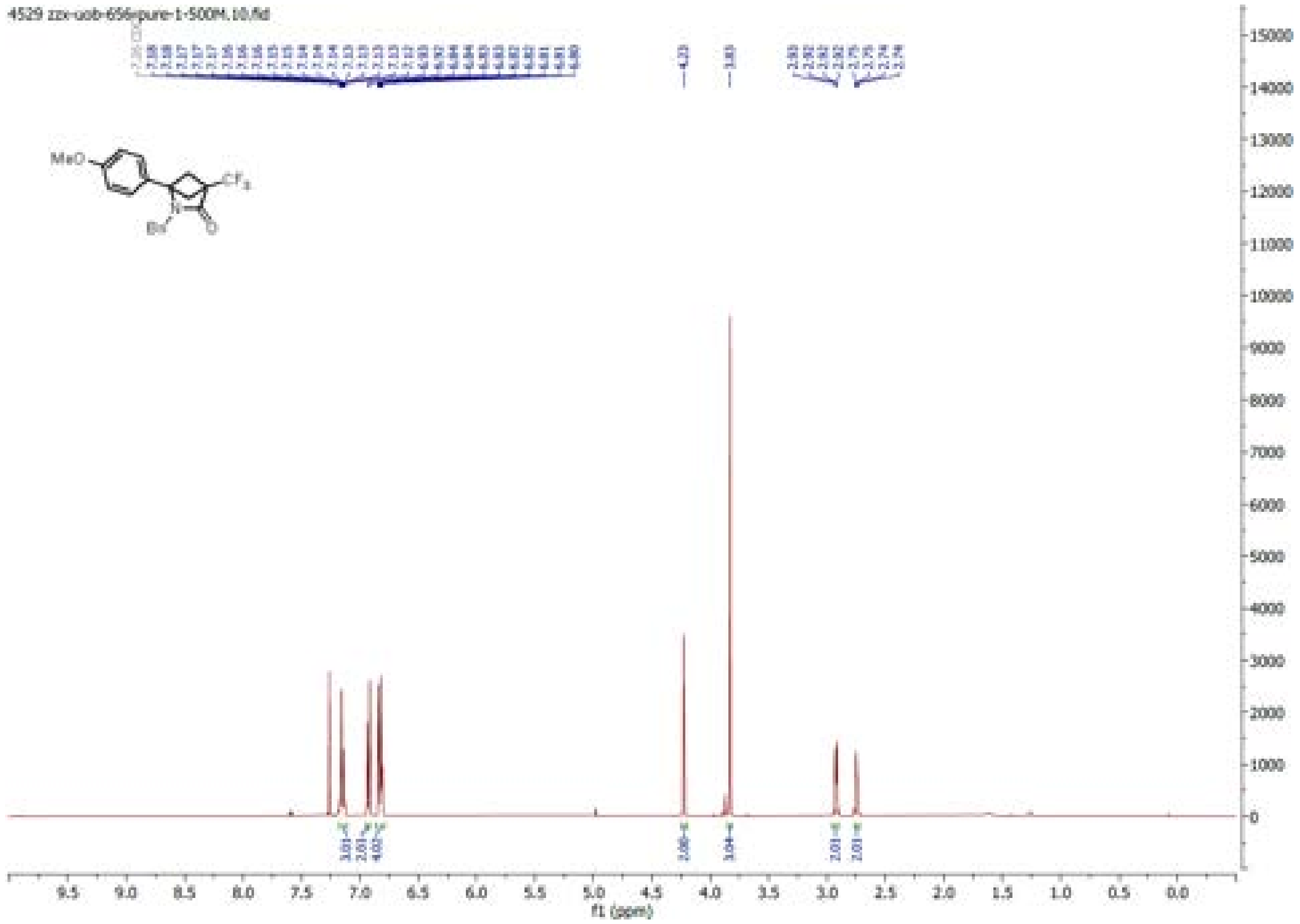

^{13}C NMR (126 MHz, $CDCl_3$) of **3p**

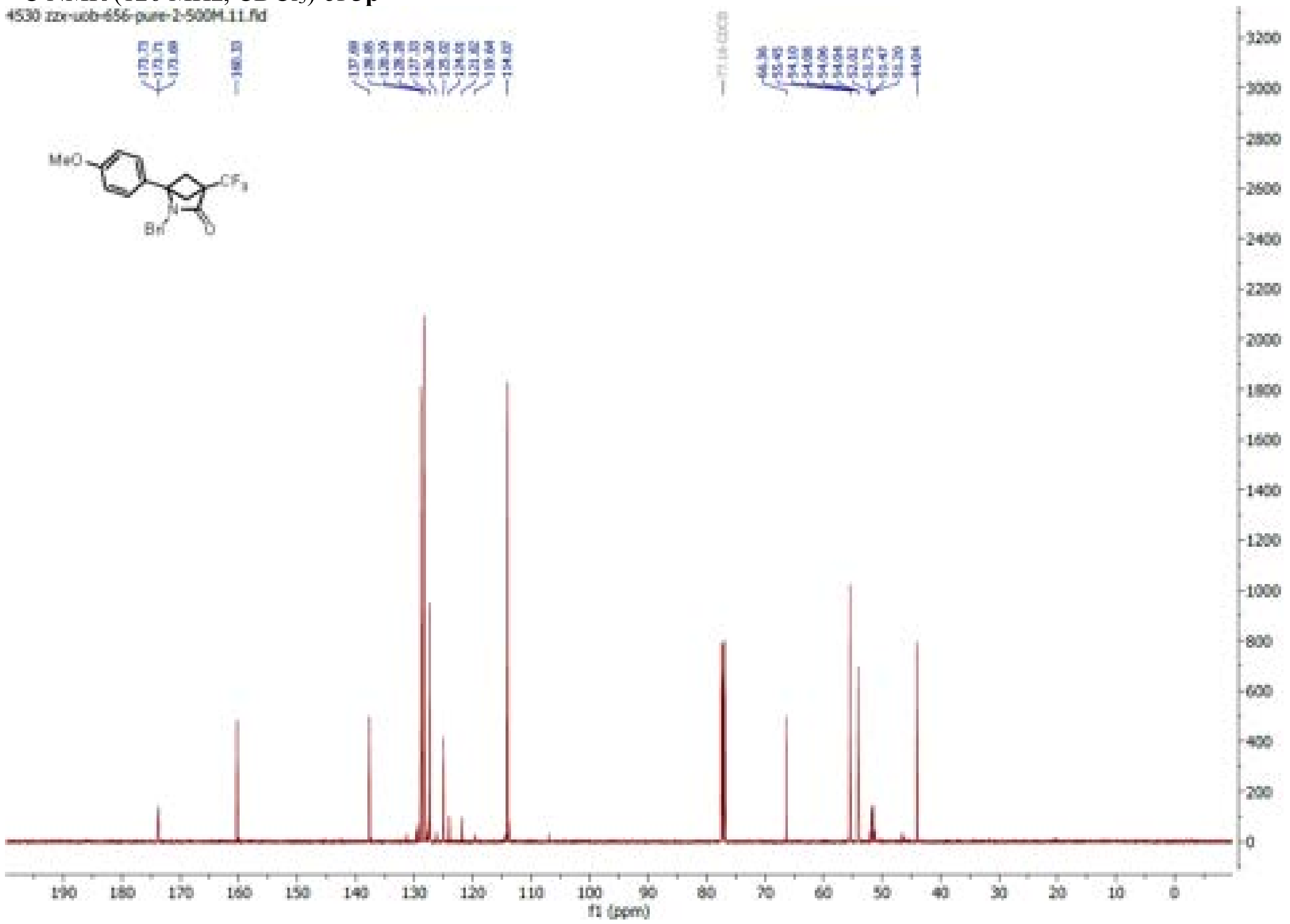

^{19}F NMR (471 MHz, $CDCl_3$) of **3p**

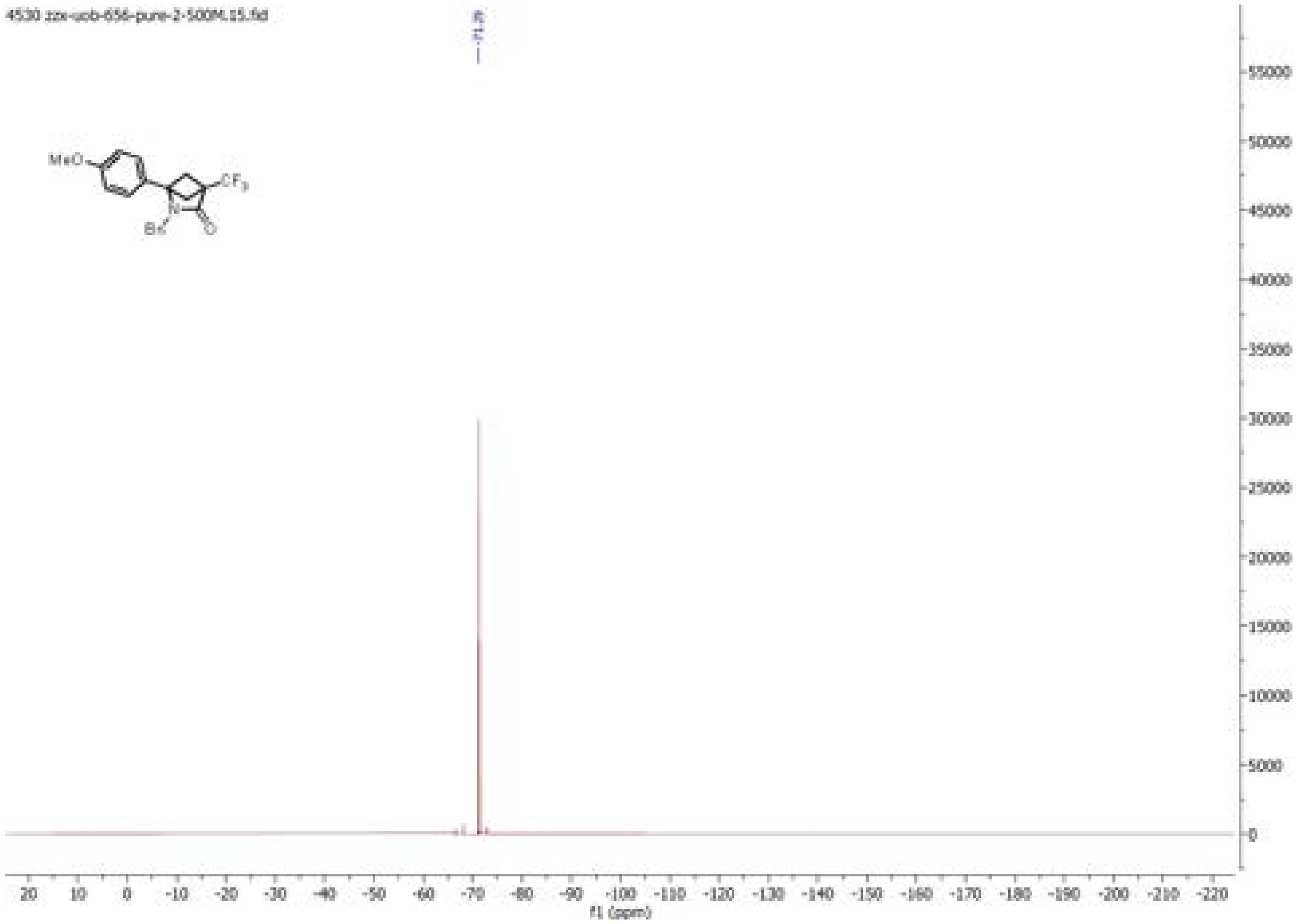

^{1}H NMR (500 MHz, $CDCl_3$) of **3q**

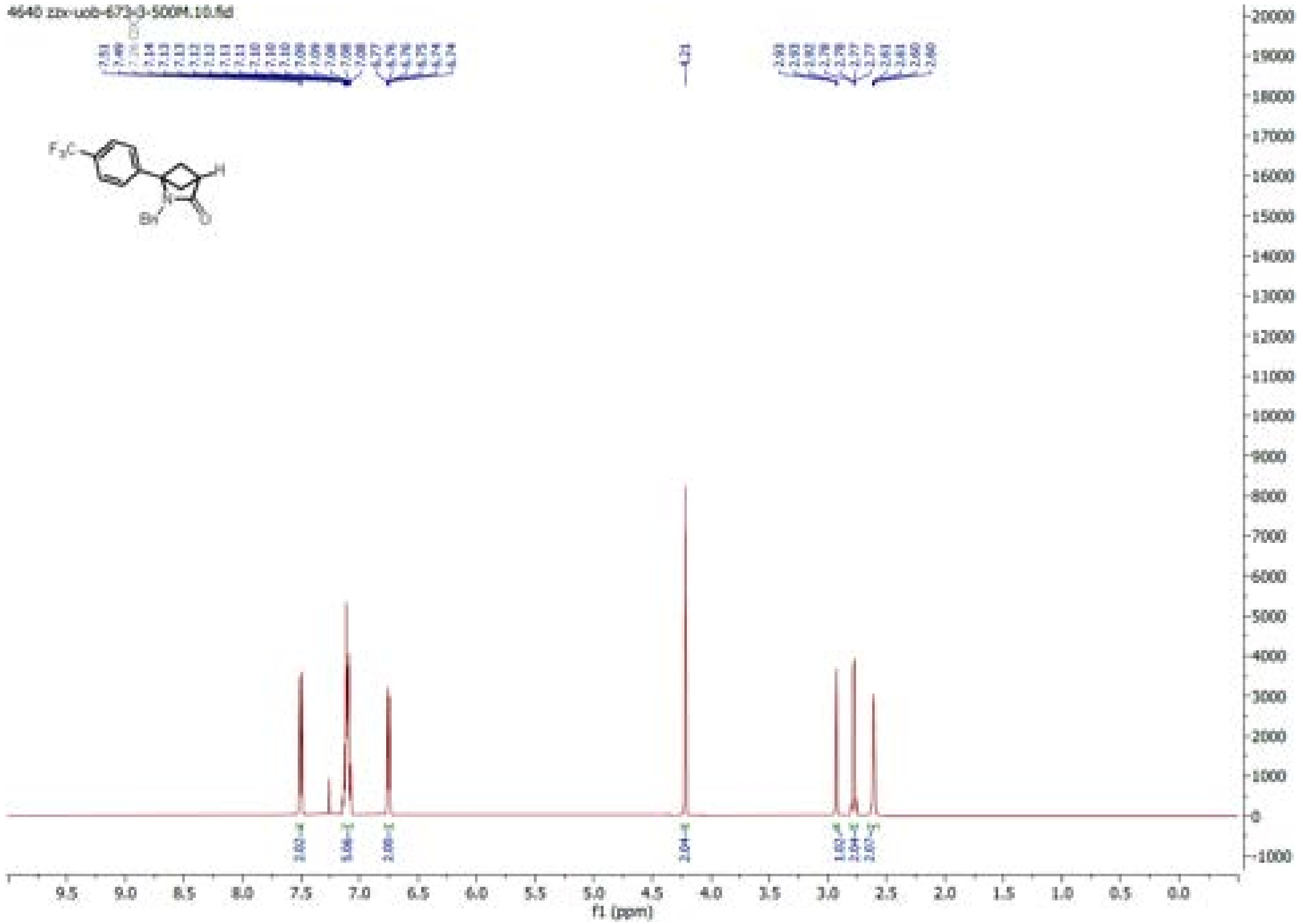

^{13}C NMR (126 MHz, $CDCl_3$) of **3q**

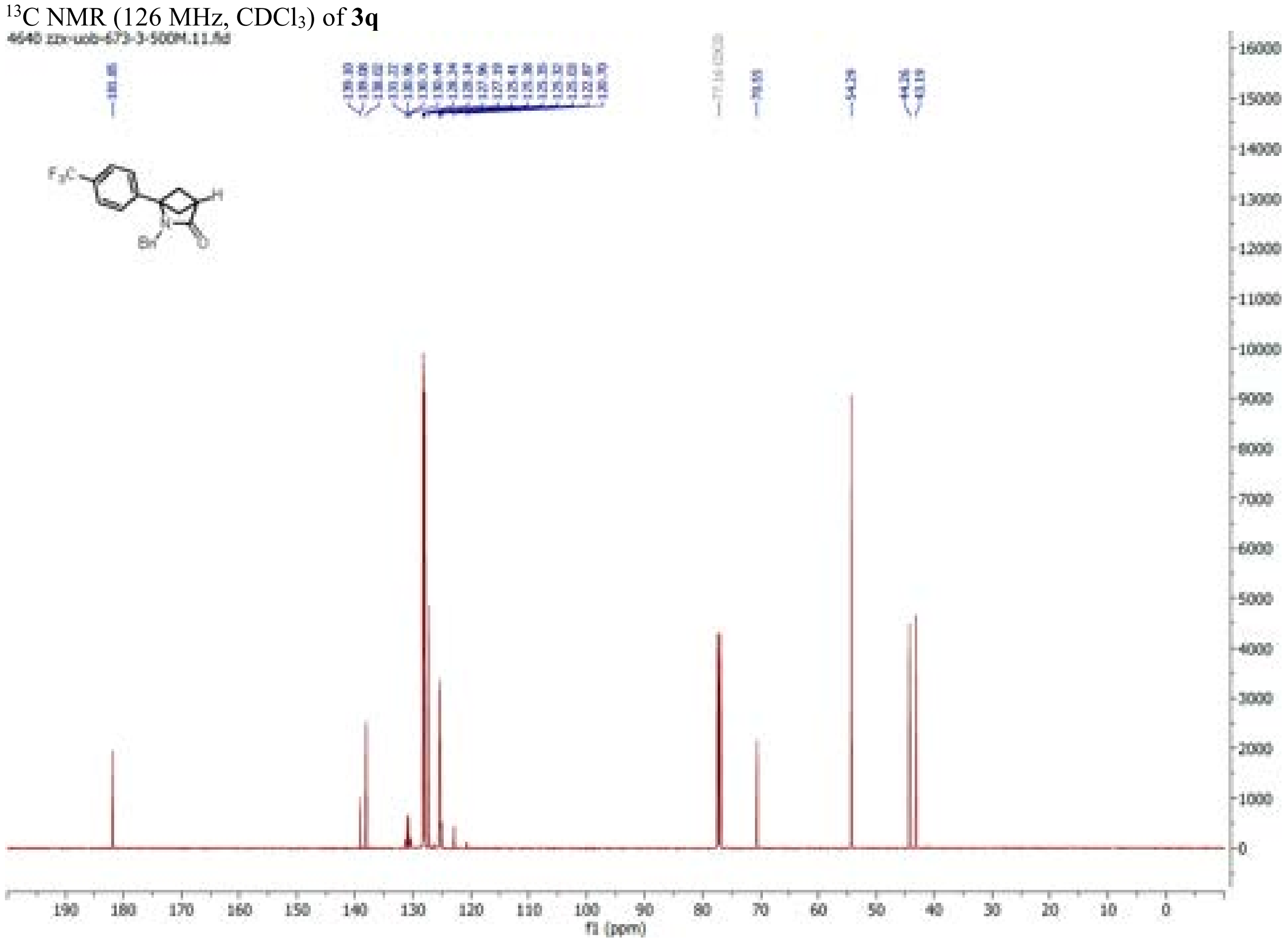

^{19}F NMR (471 MHz, $CDCl_3$) of **3q**

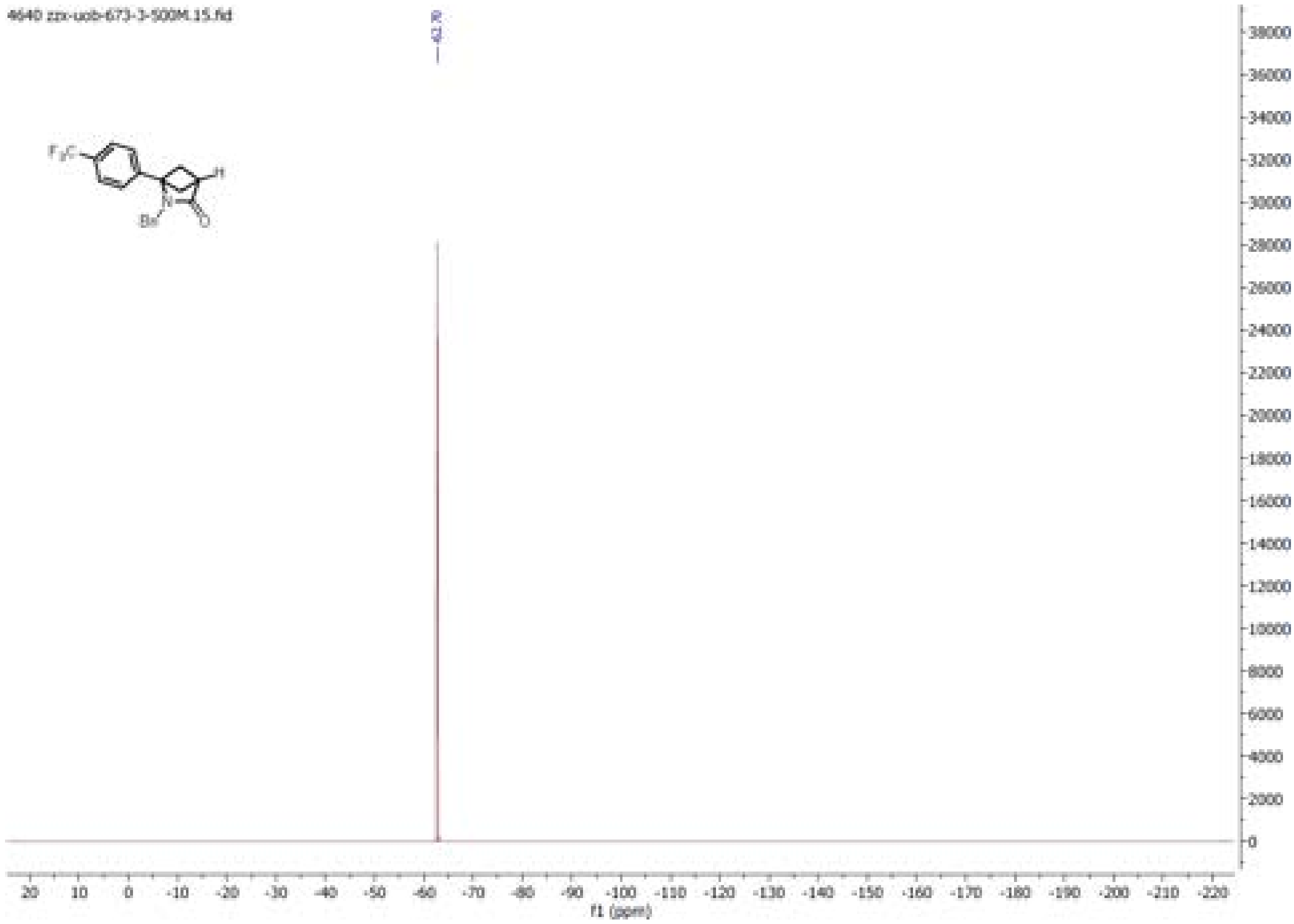

^{1}H NMR (500 MHz, $CDCl_3$) of **3r**

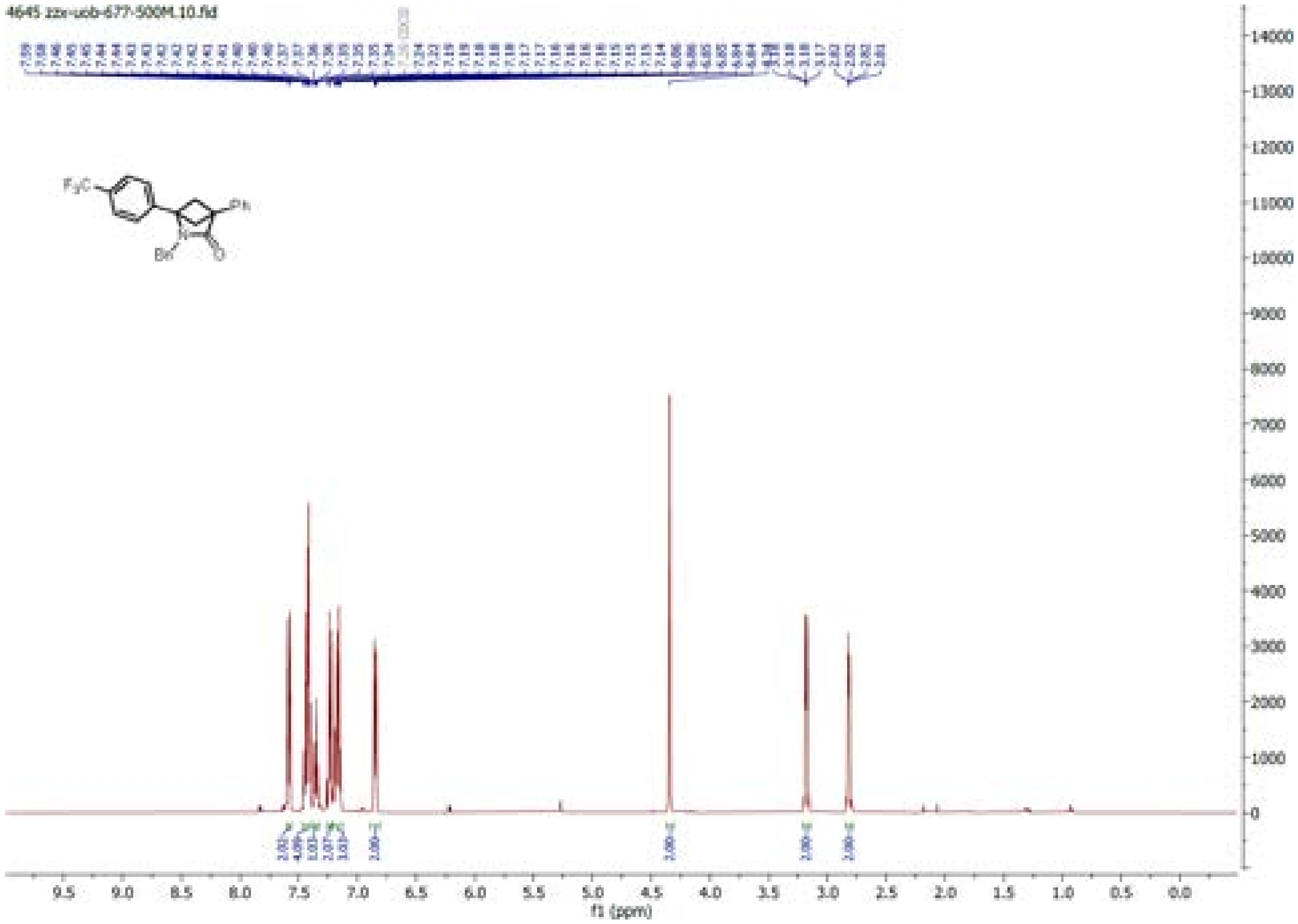

^{13}C NMR (126 MHz, $CDCl_3$) of **3r**

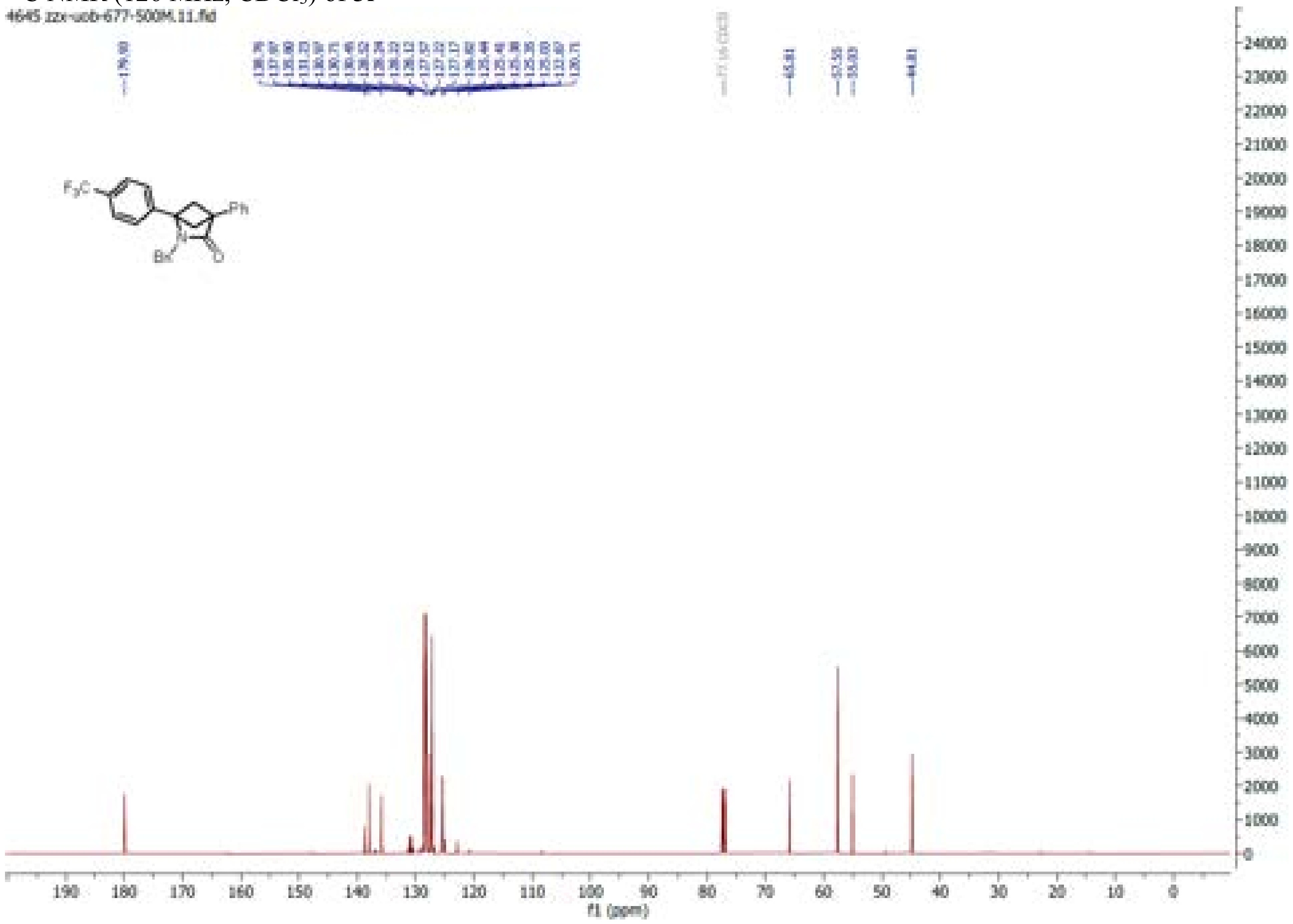

^{19}F NMR (471 MHz, $CDCl_3$) of **3r**

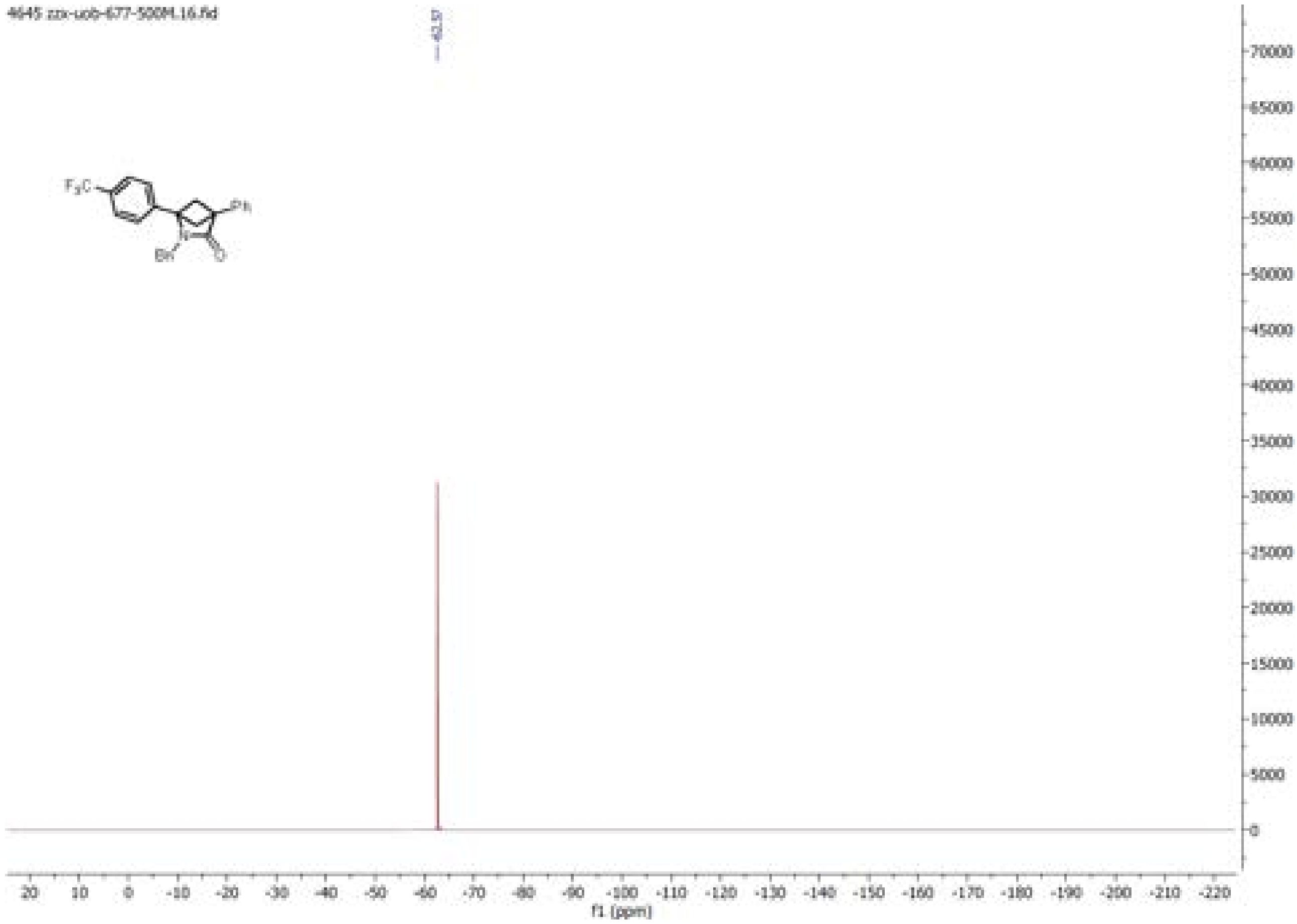

5.5 NMR Spectra of Products (8)

^{1}H NMR (500 MHz, $CDCl_3$) of **8a**

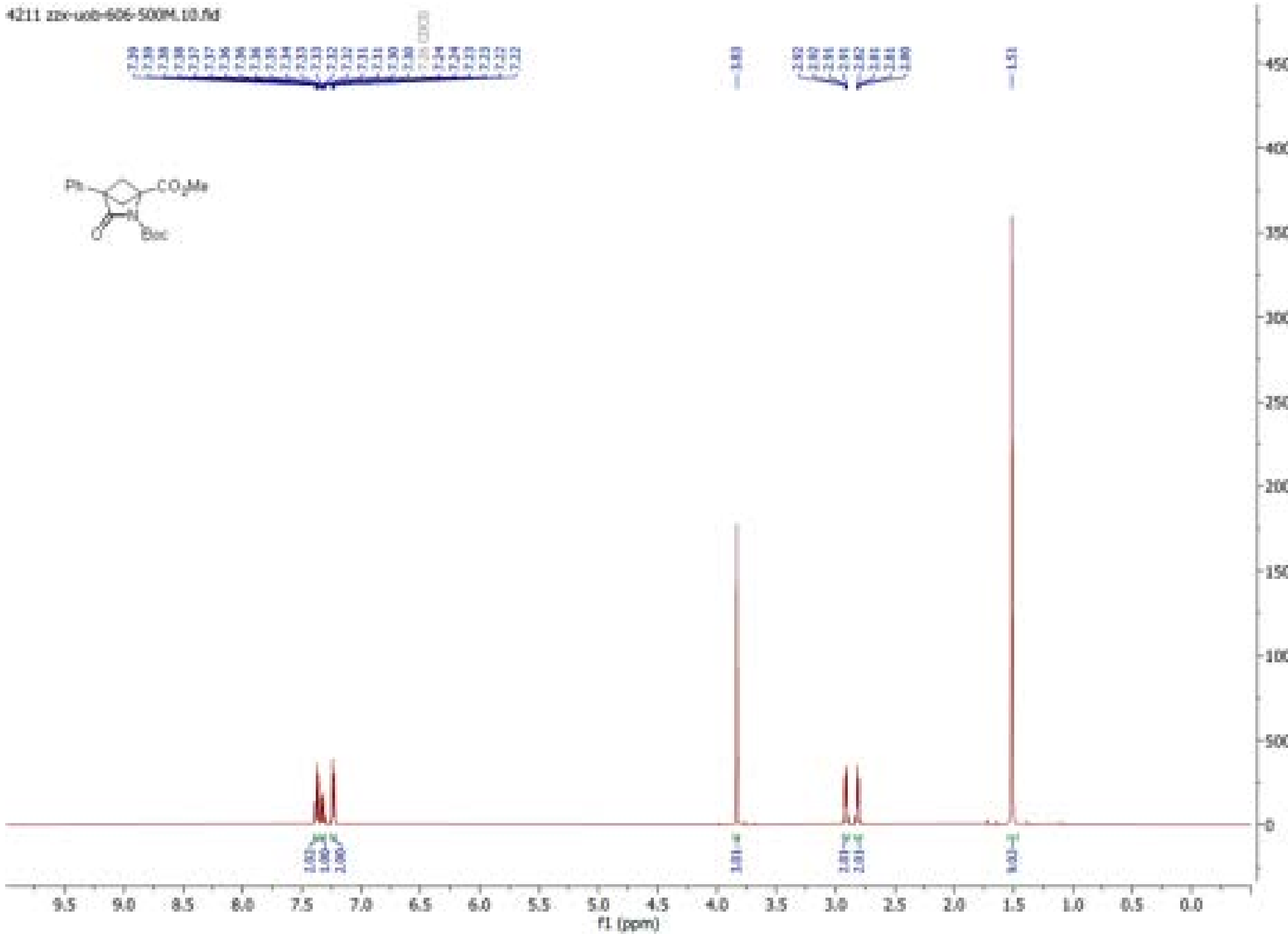

^{13}C NMR (126 MHz, $CDCl_3$) of **8a**

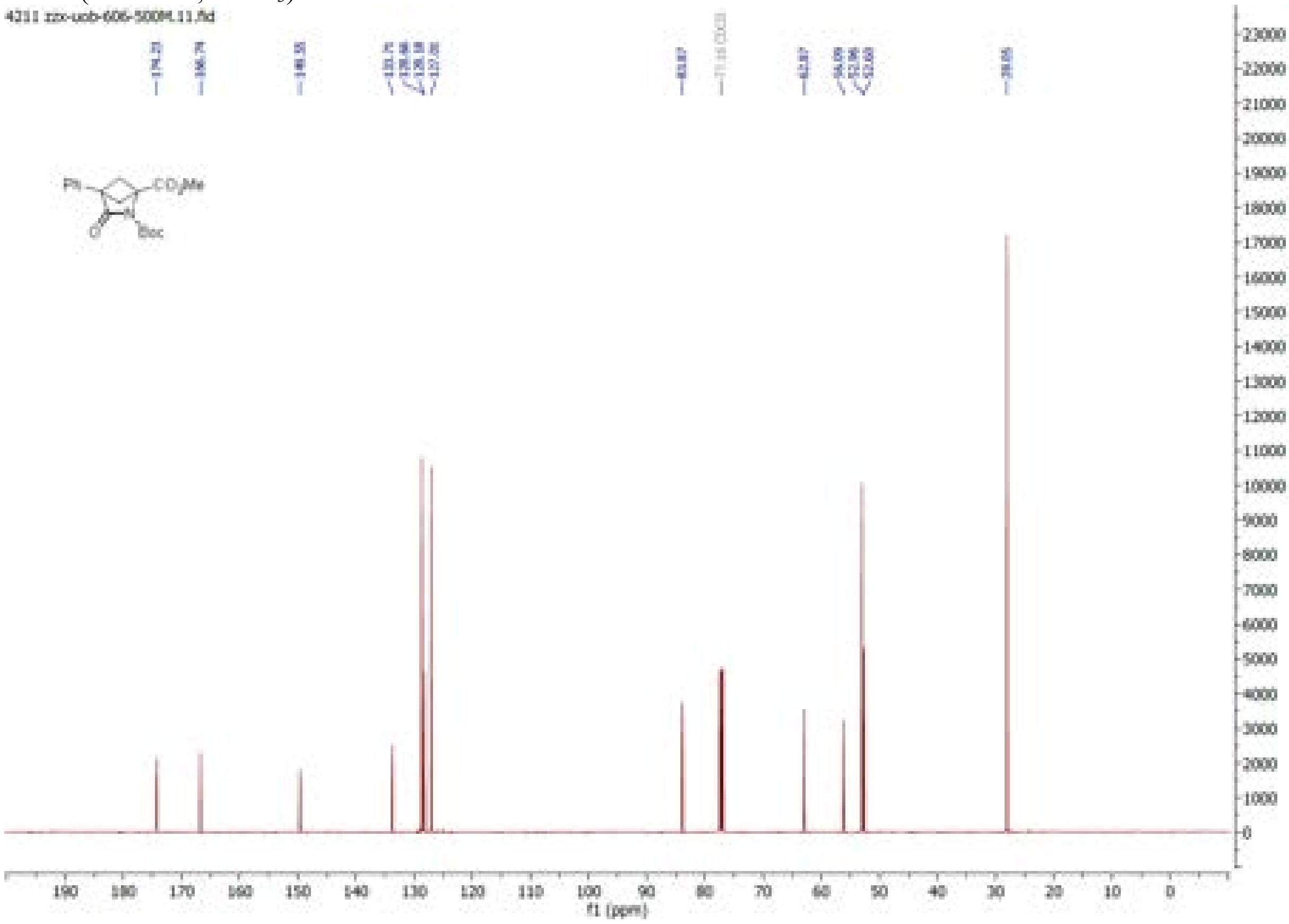

^{1}H NMR (500 MHz, $CDCl_3$) of **8b**

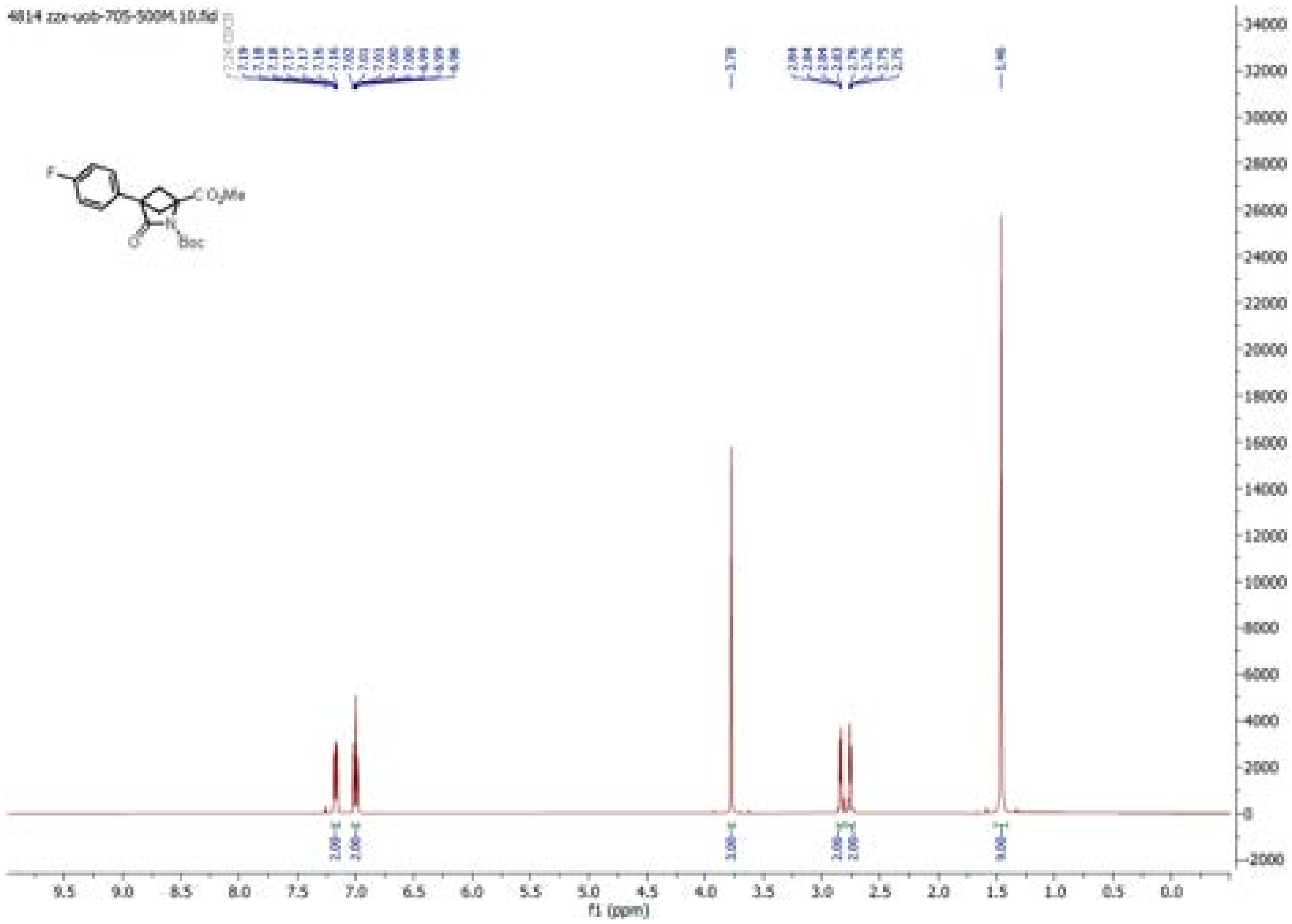

^{13}C NMR (126 MHz, $CDCl_3$) of **8b**

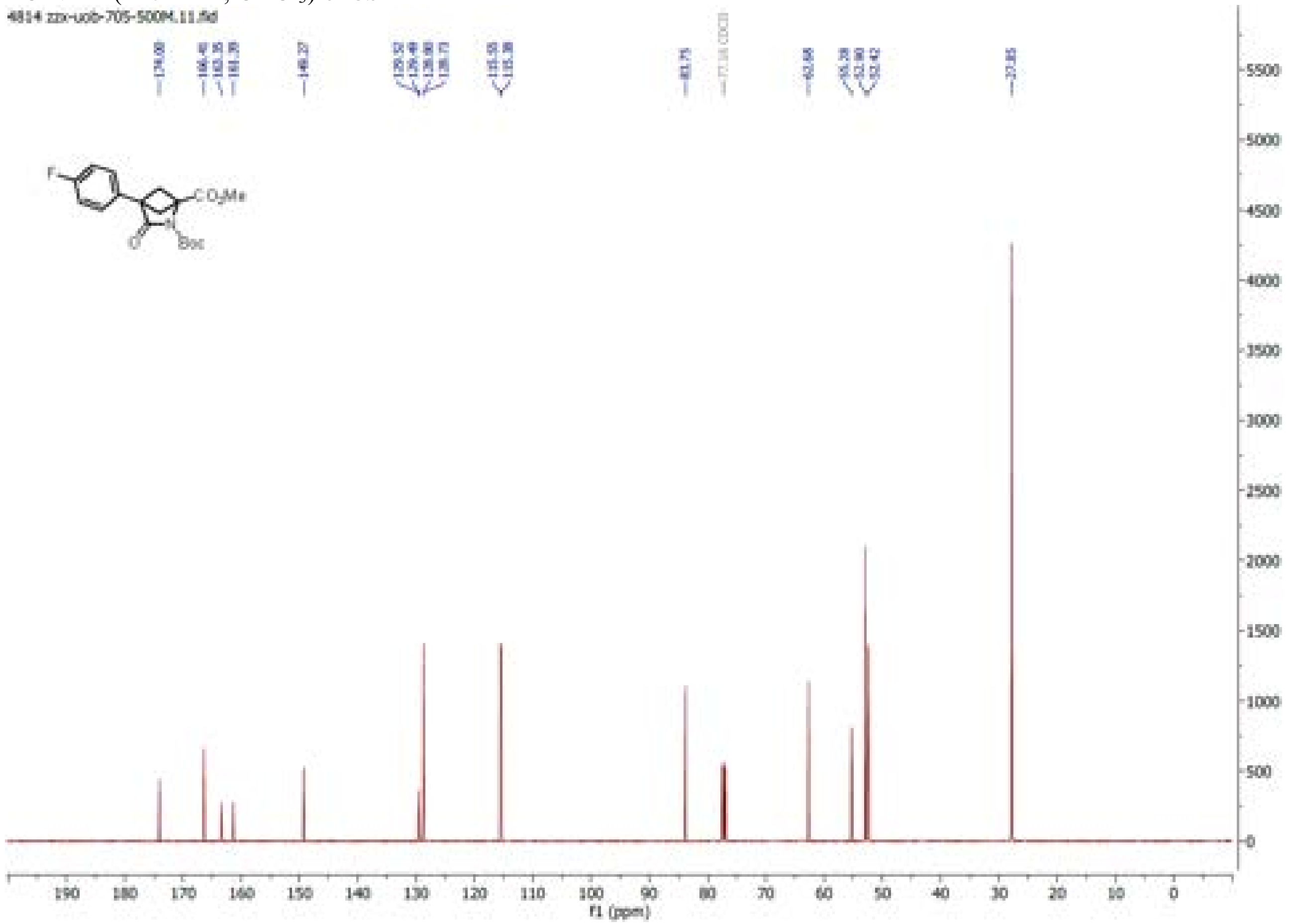

^{19}F NMR (471 MHz, $CDCl_3$) of **8b**

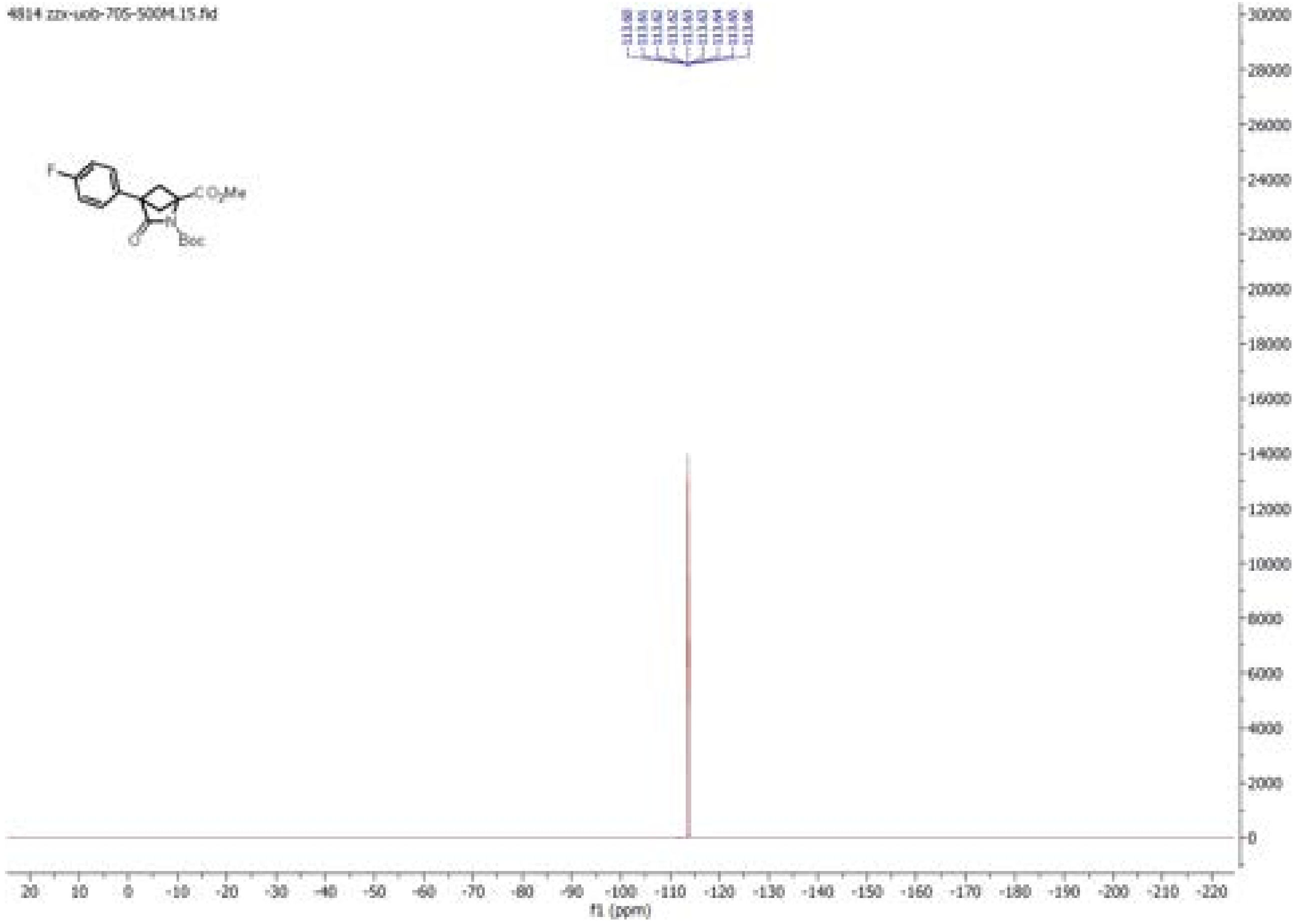

^{1}H NMR (500 MHz, $CDCl_3$) of **8c**

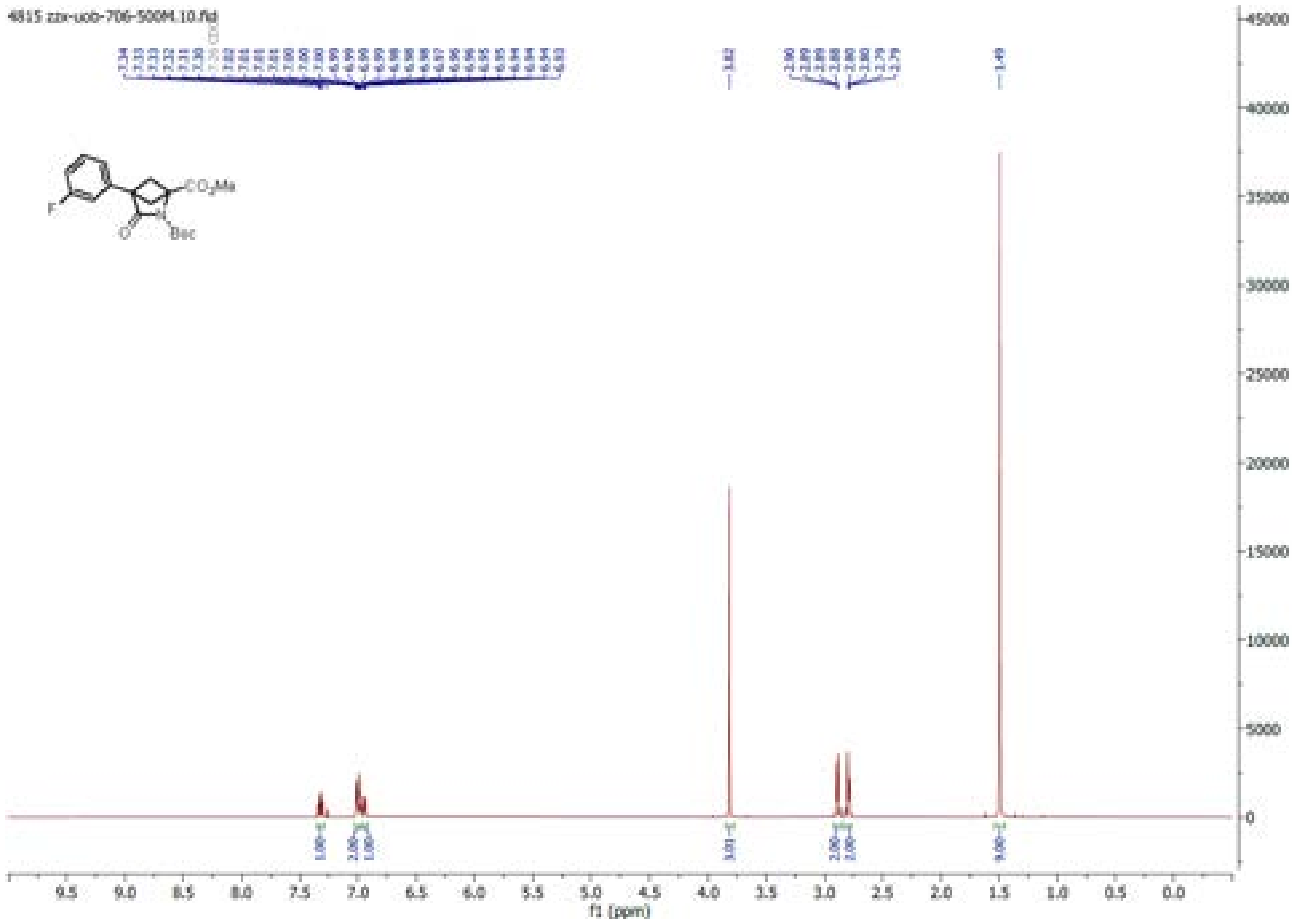

^{13}C NMR (126 MHz, $CDCl_3$) of **8c**

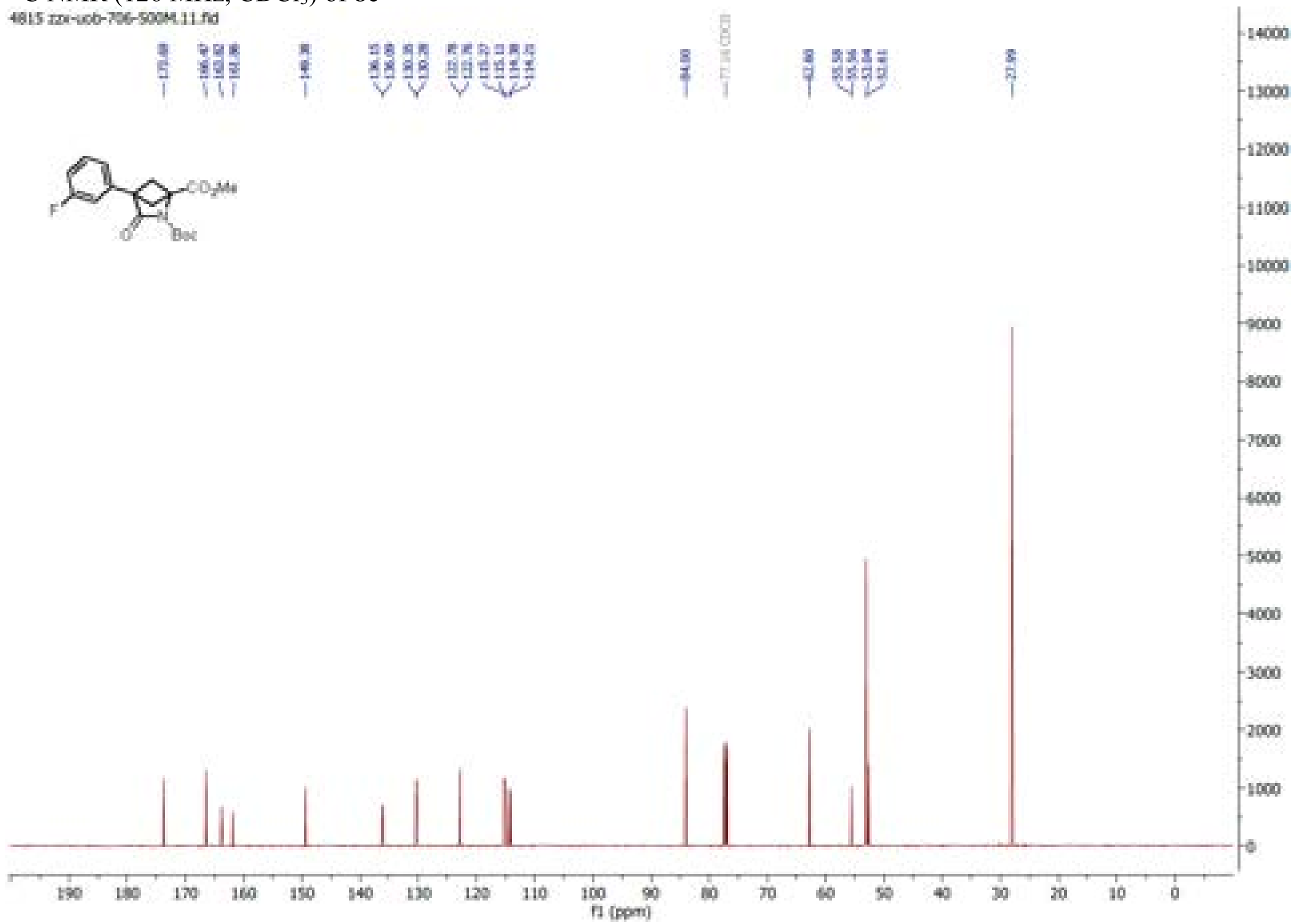

^{19}F NMR (471 MHz, $CDCl_3$) of **8c**

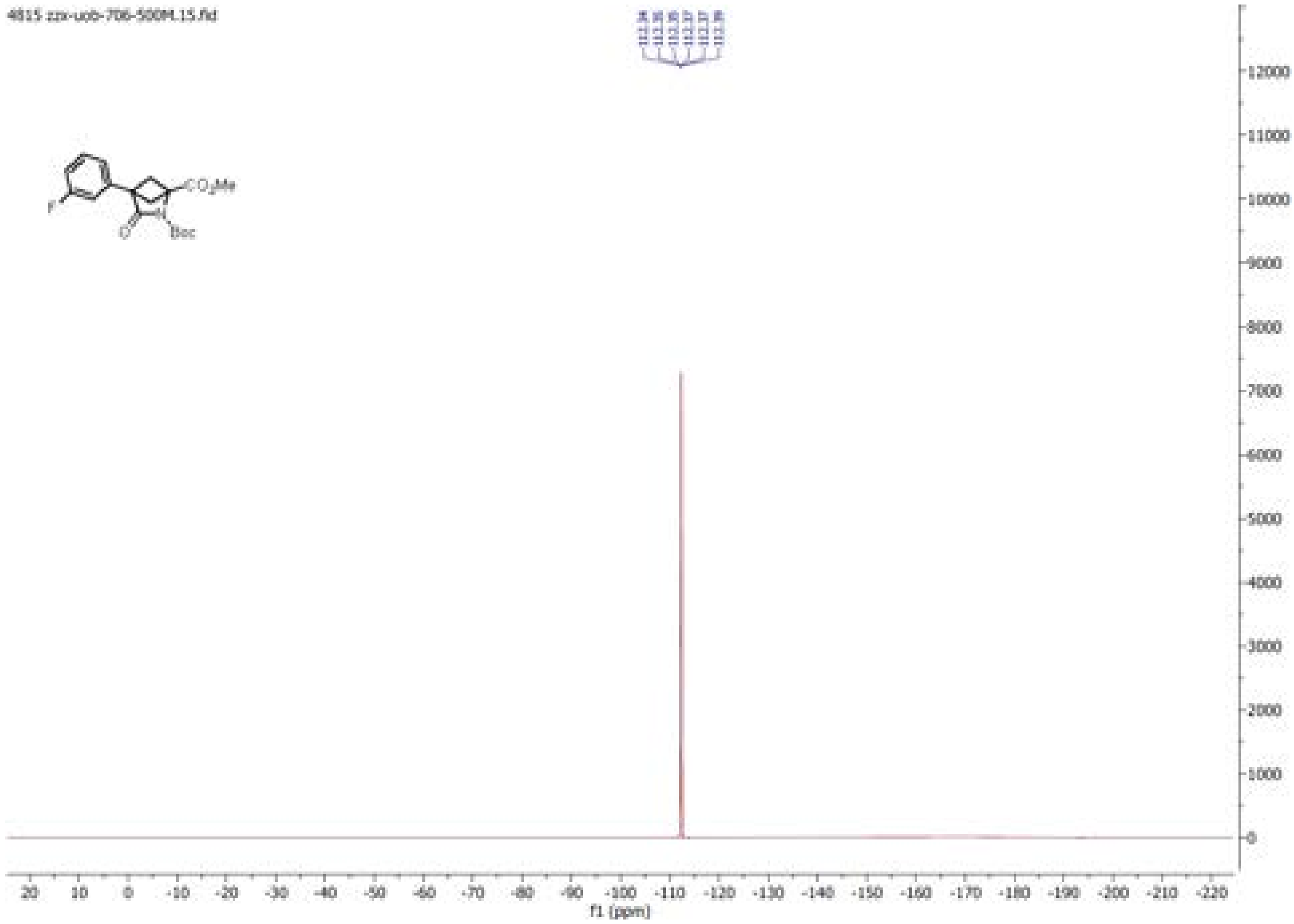

^{1}H NMR (500 MHz, $CDCl_3$) of **8d**

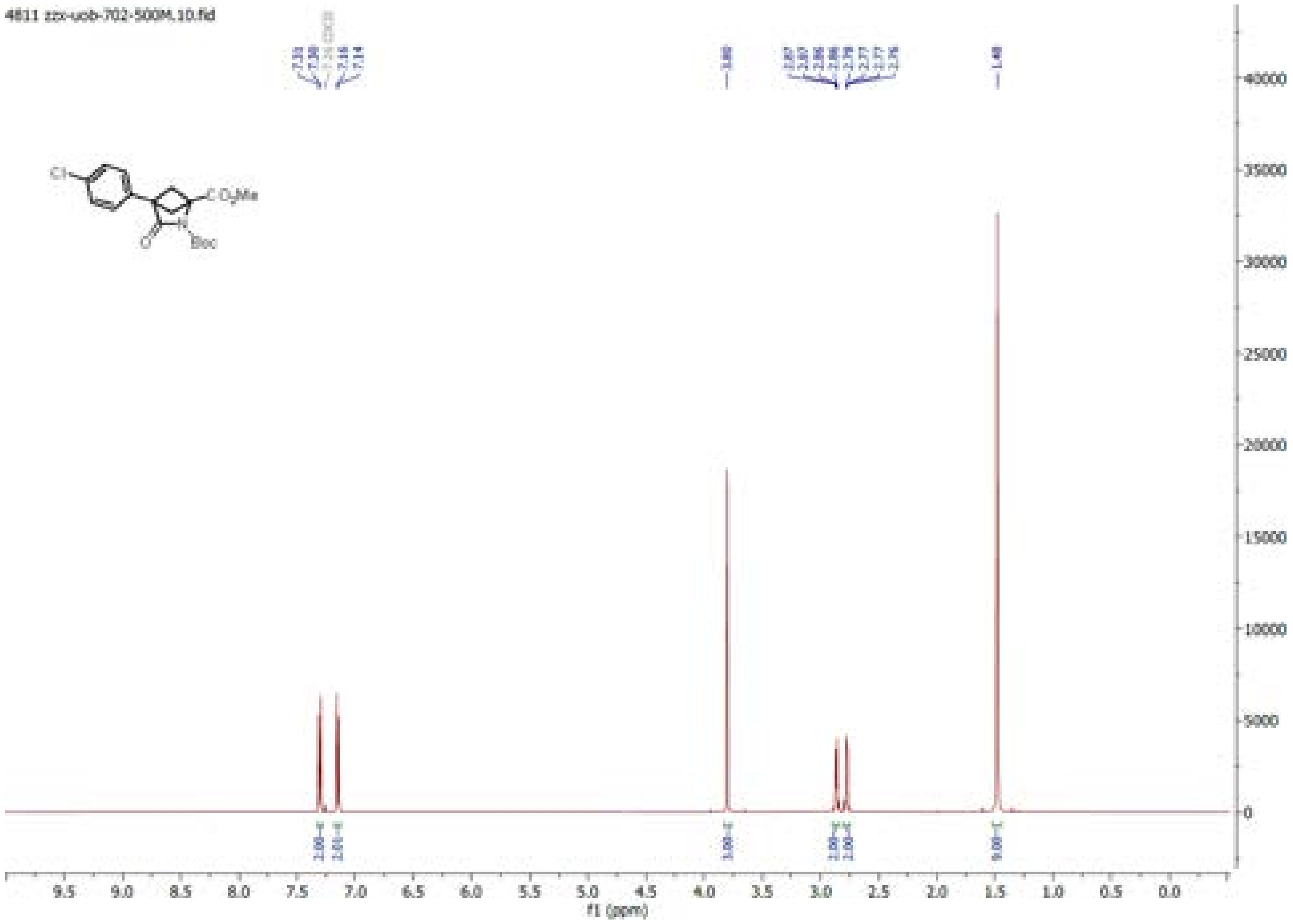

^{13}C NMR (126 MHz, $CDCl_3$) of **8d**

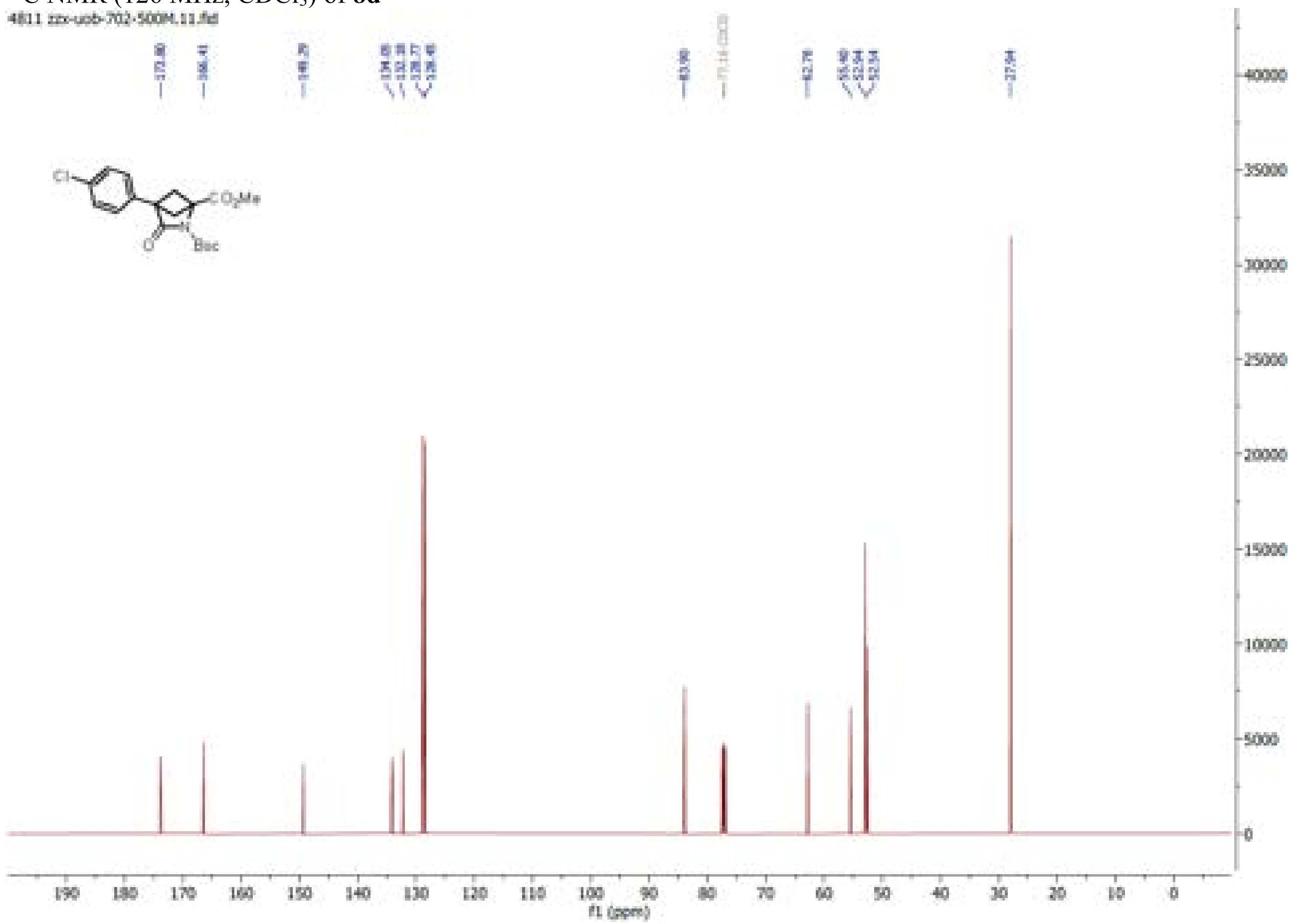

^{1}H NMR (500 MHz, $CDCl_3$) of **8e**

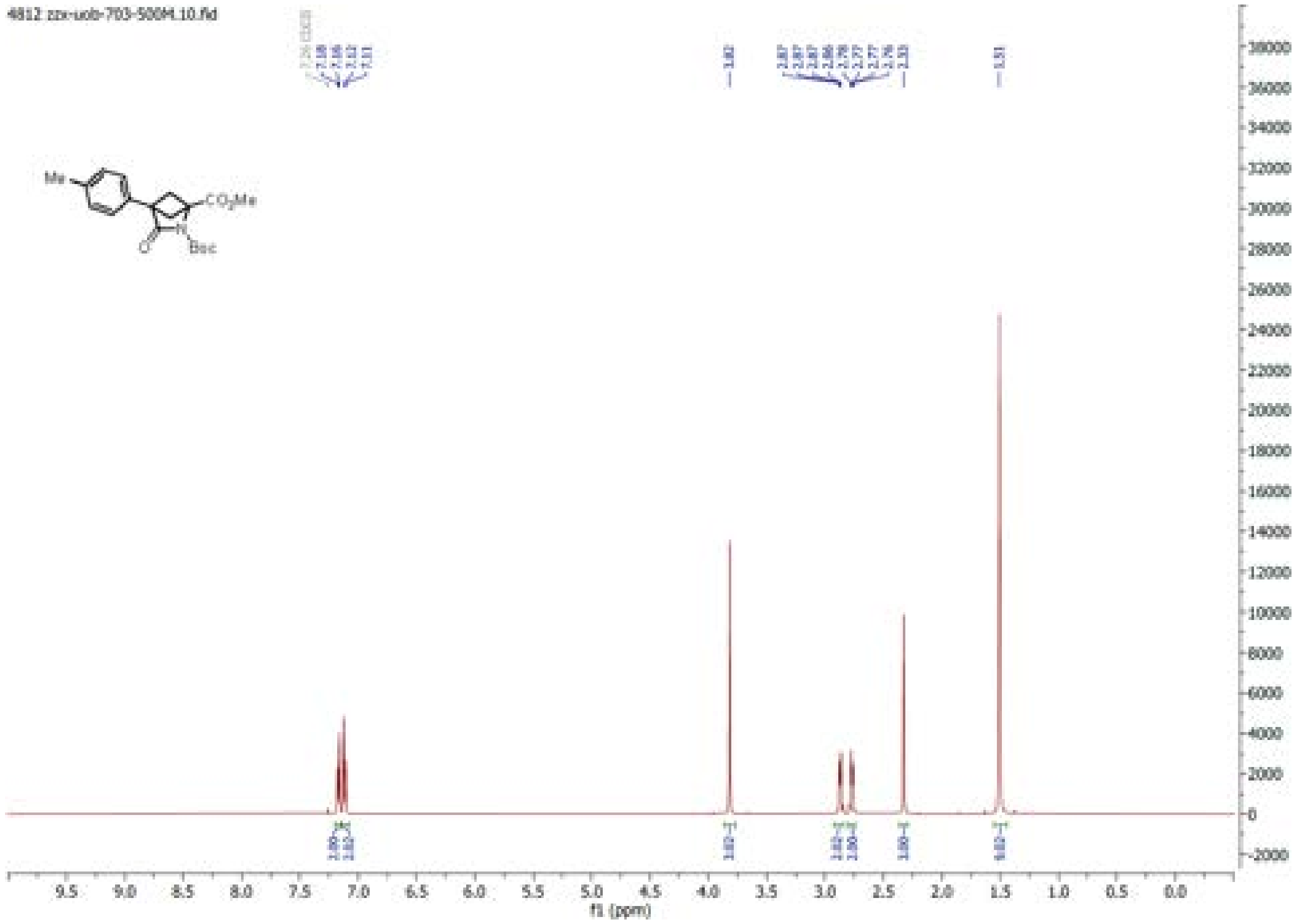

^{13}C NMR (126 MHz, $CDCl_3$) of **8e**

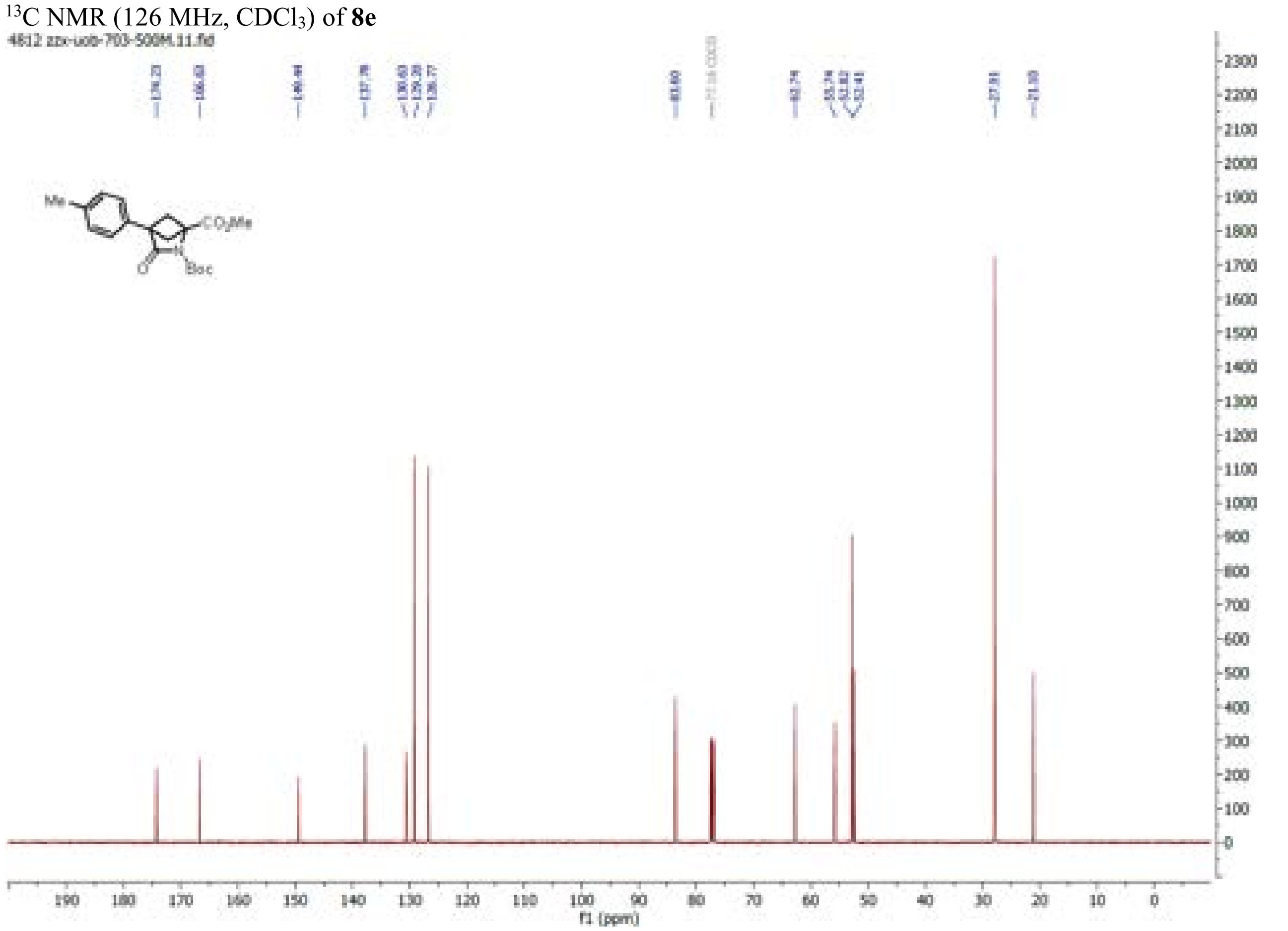

^{1}H NMR (500 MHz, $CDCl_3$) of **8f**

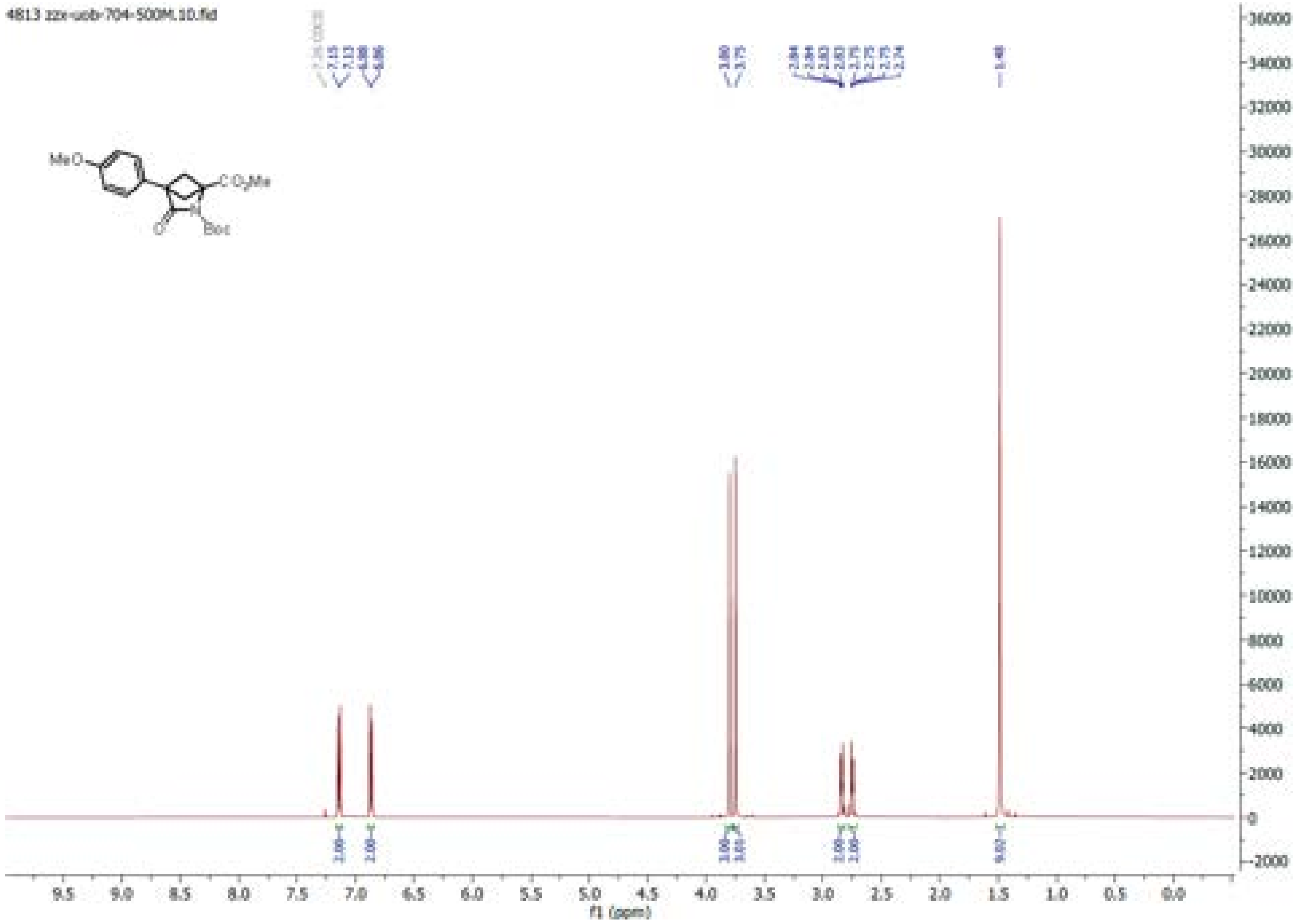

^{13}C NMR (126 MHz, $CDCl_3$) of **8f**

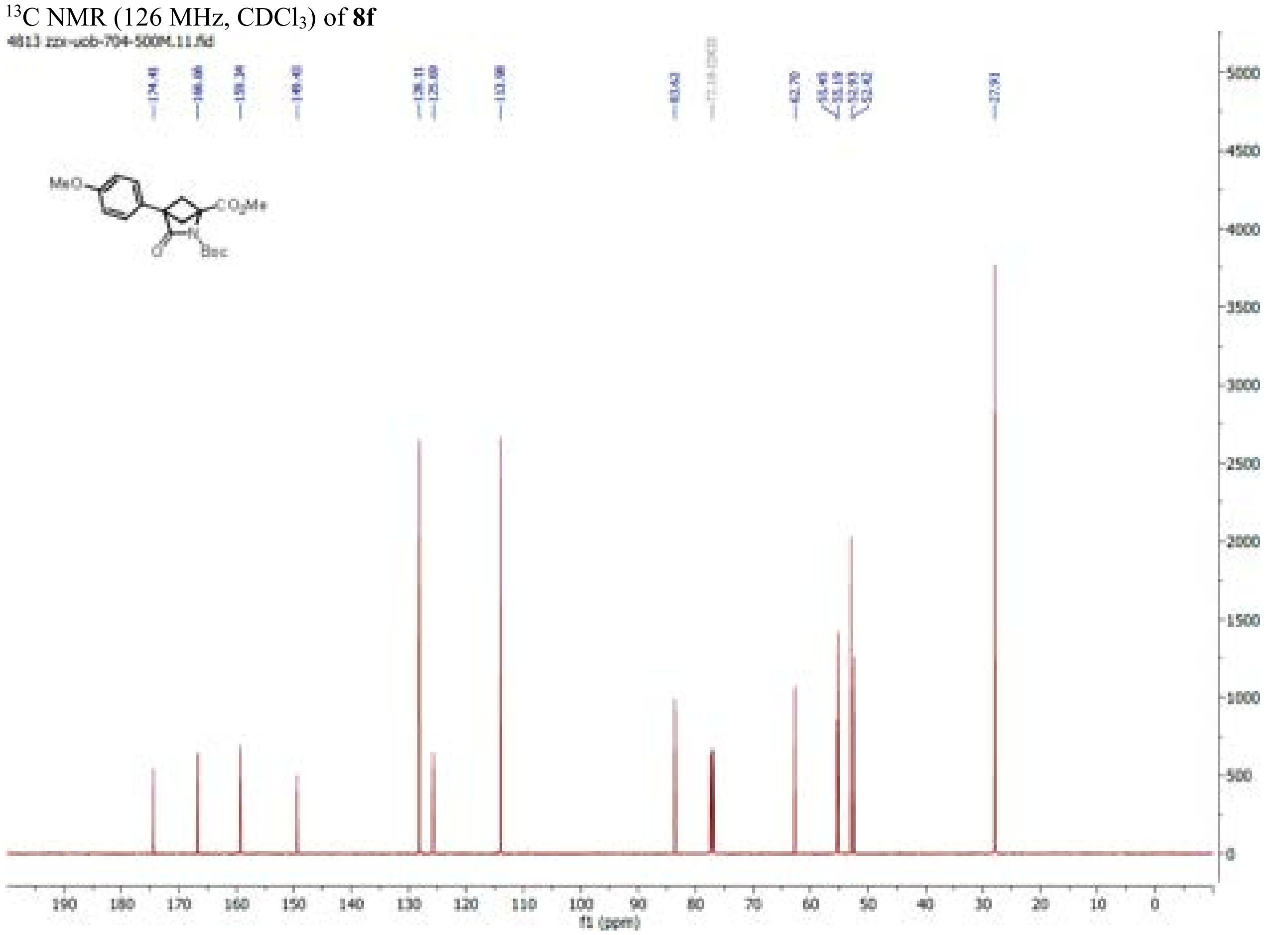

^{1}H NMR (500 MHz, $CDCl_3$) of **8g**

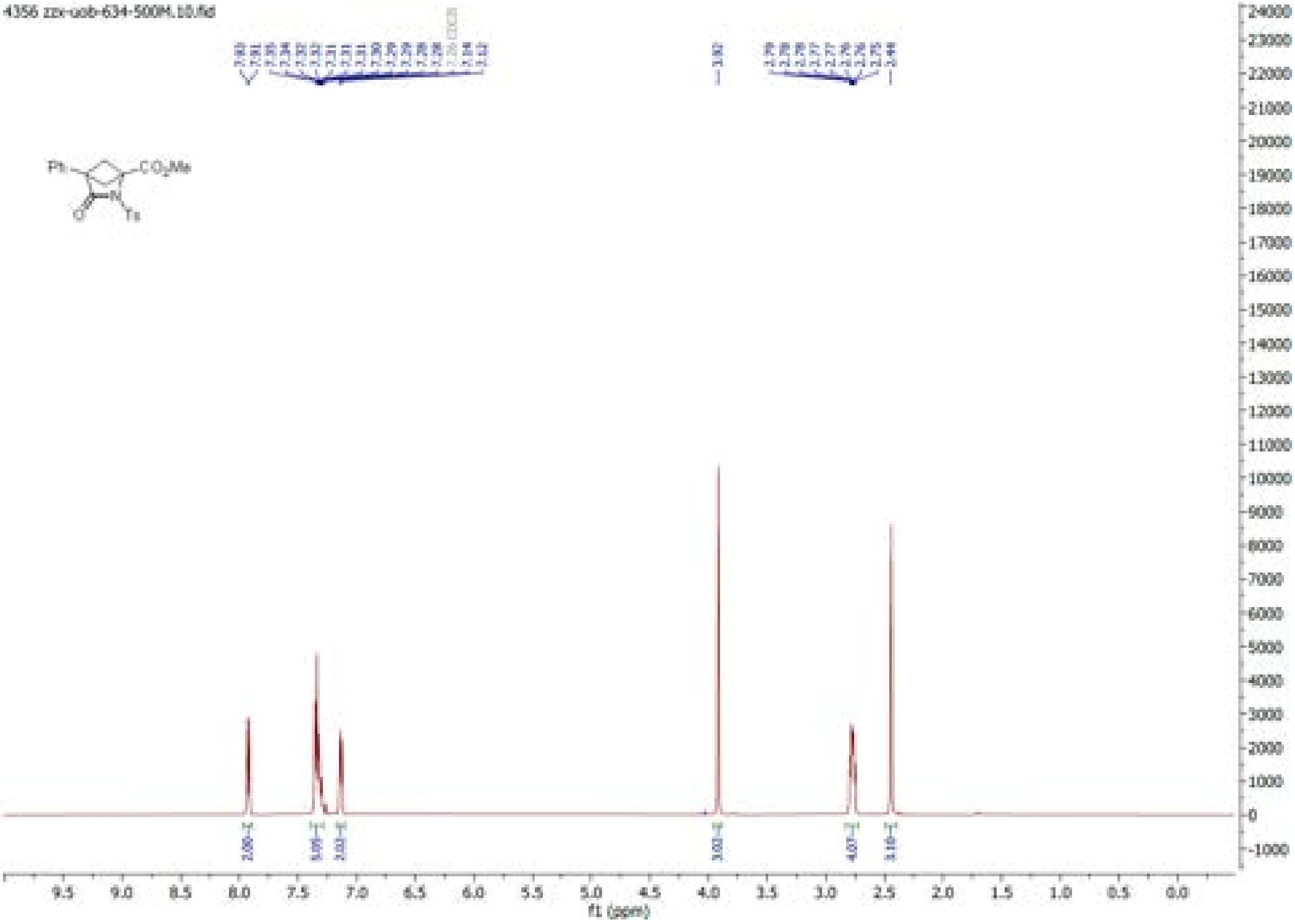

^{13}C NMR (126 MHz, $CDCl_3$) of **8g**

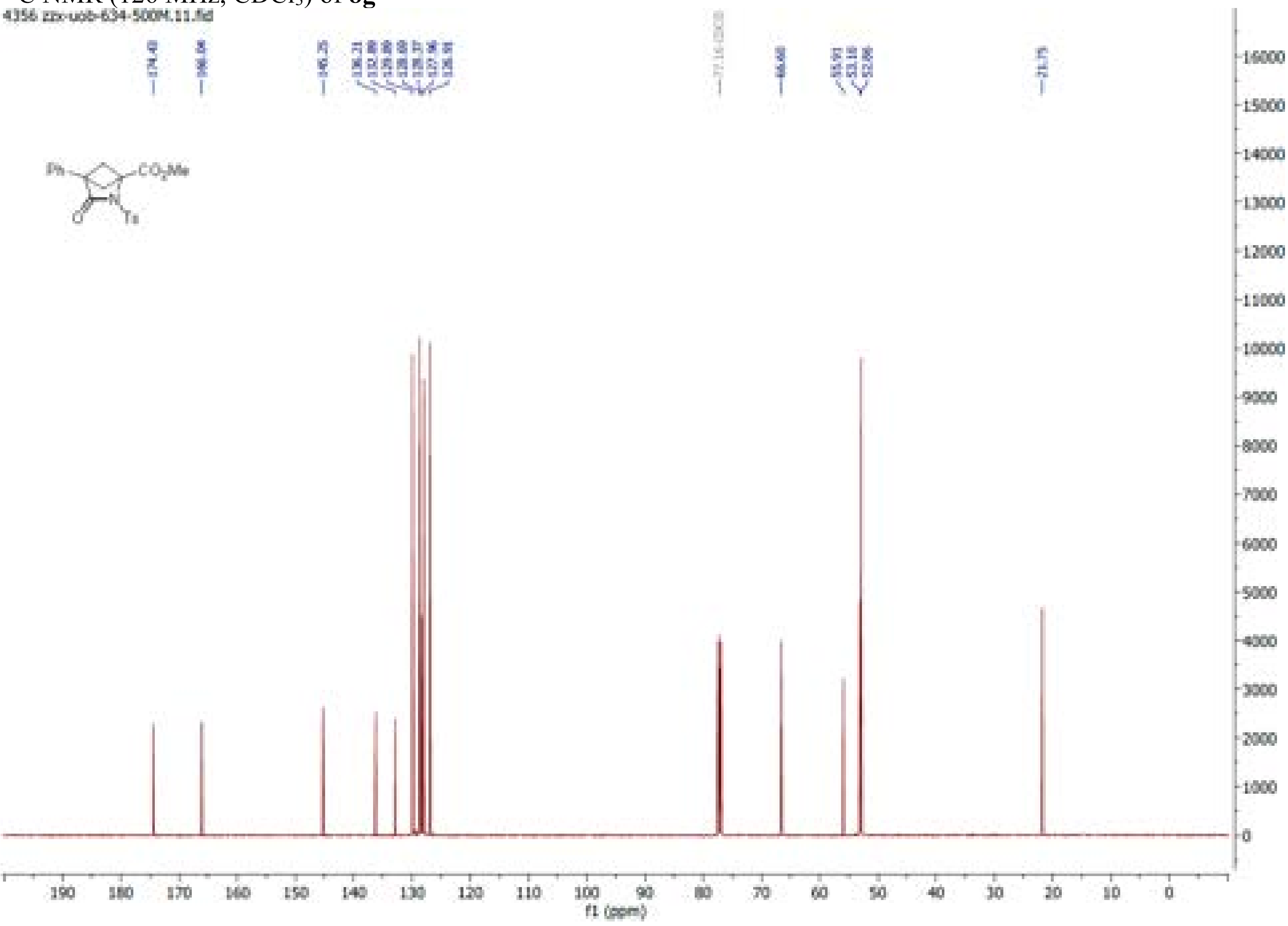

^{1}H NMR (500 MHz, $CDCl_3$) of **8h**

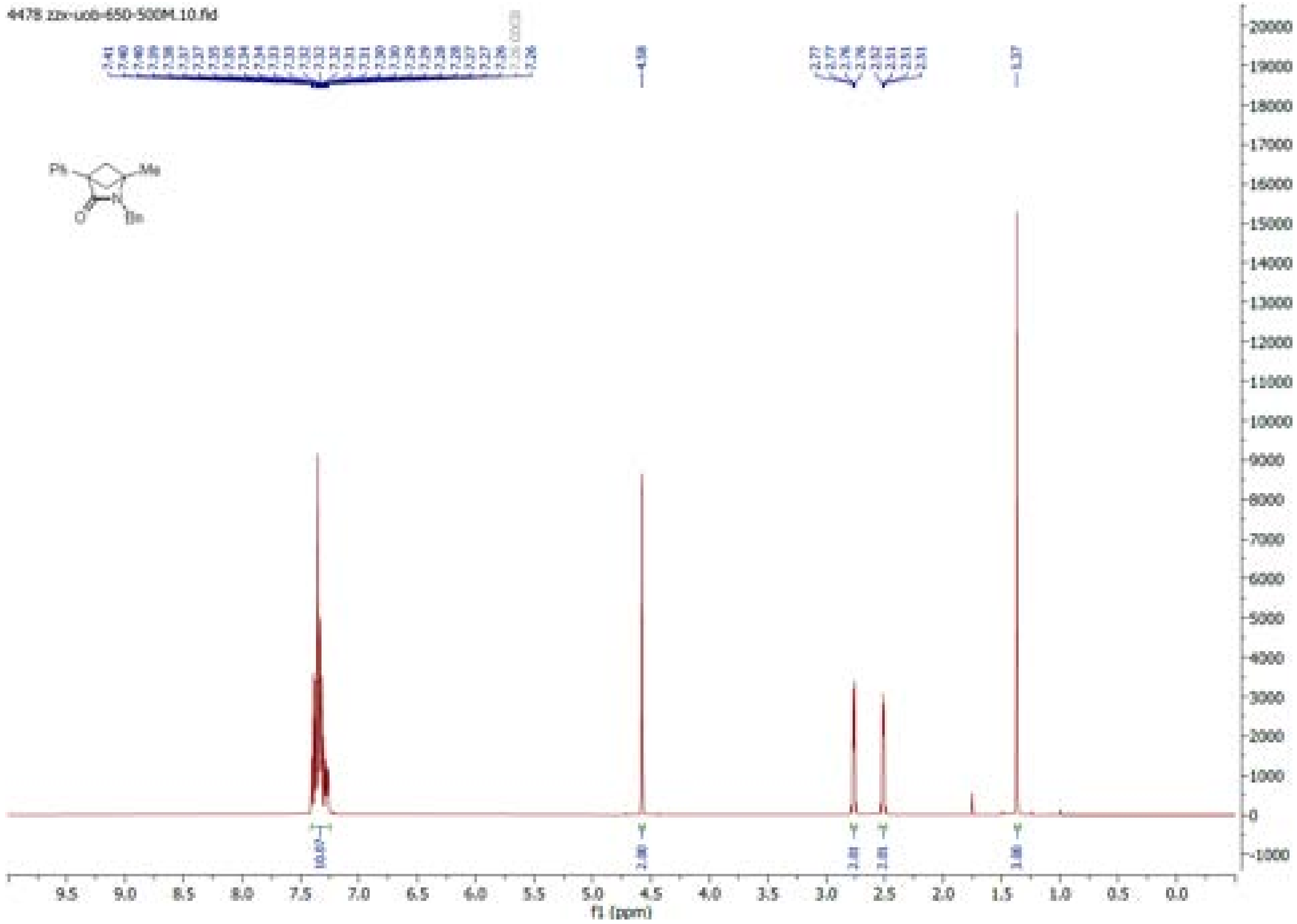

^{13}C NMR (126 MHz, $CDCl_3$) of **8h**

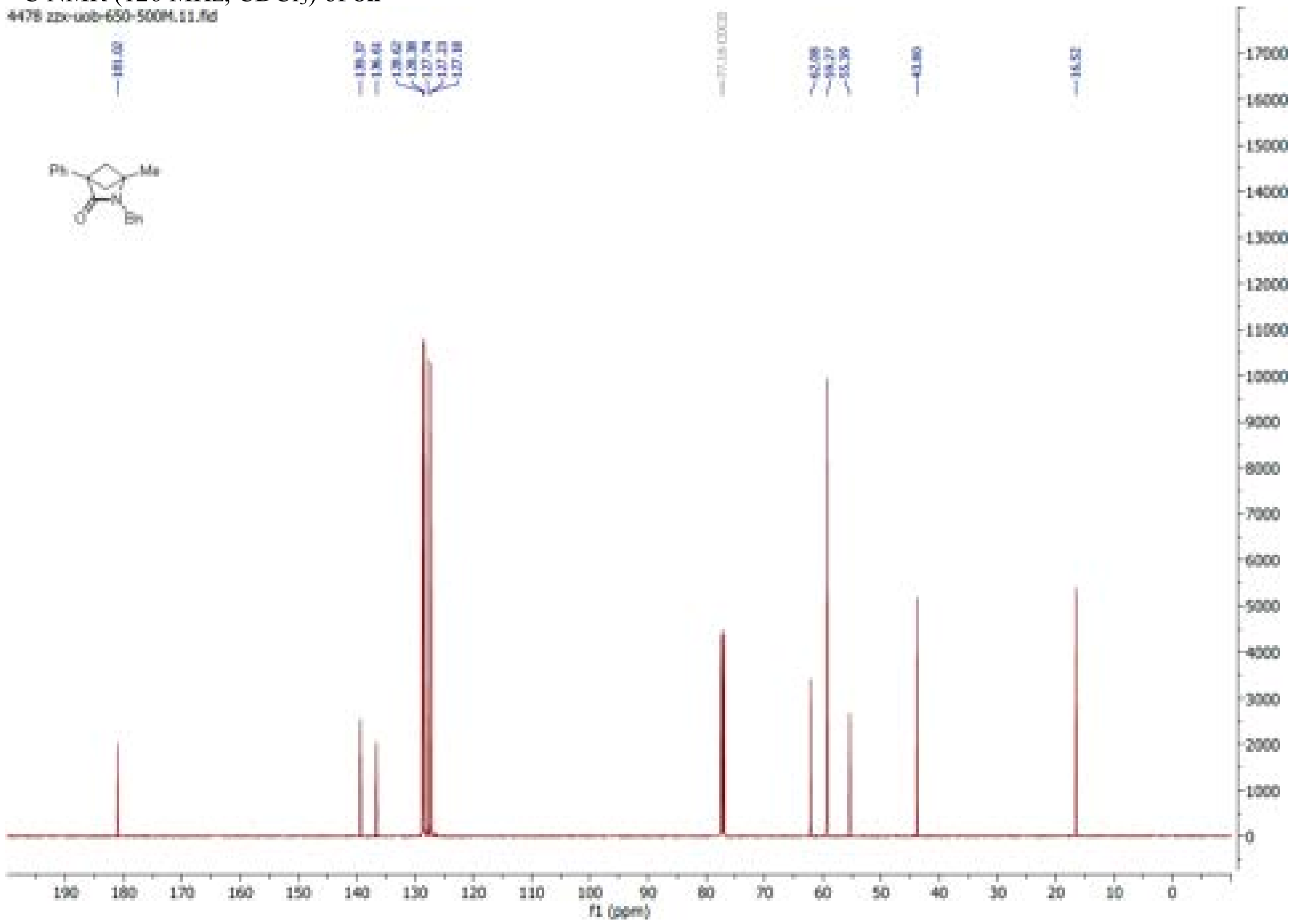

5.6 NMR Spectra of Products (5, 6 and 9)

^{1}H NMR (500 MHz, $CDCl_3$) of **6a**

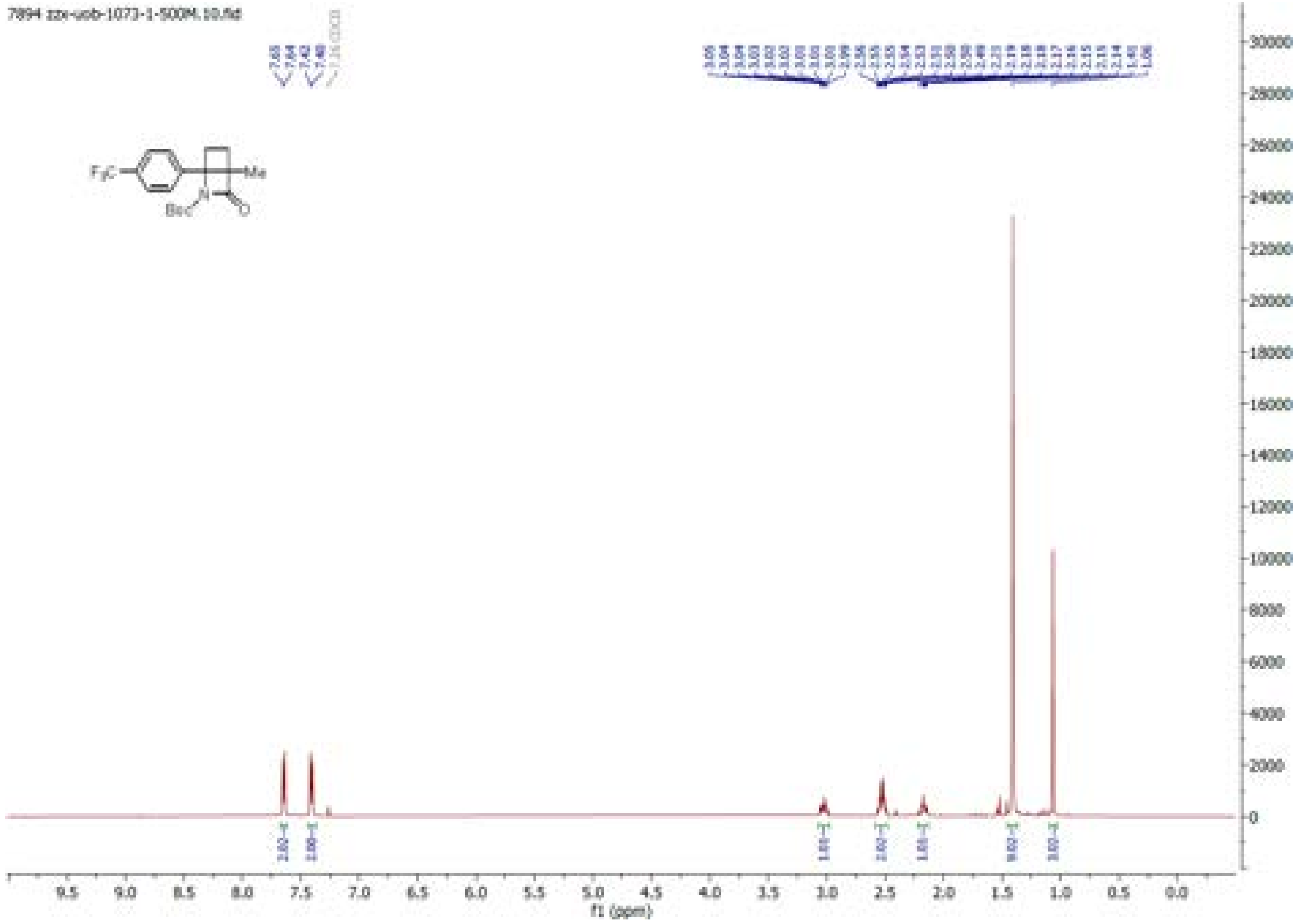

^{13}C NMR (126 MHz, $CDCl_3$) of **6a**

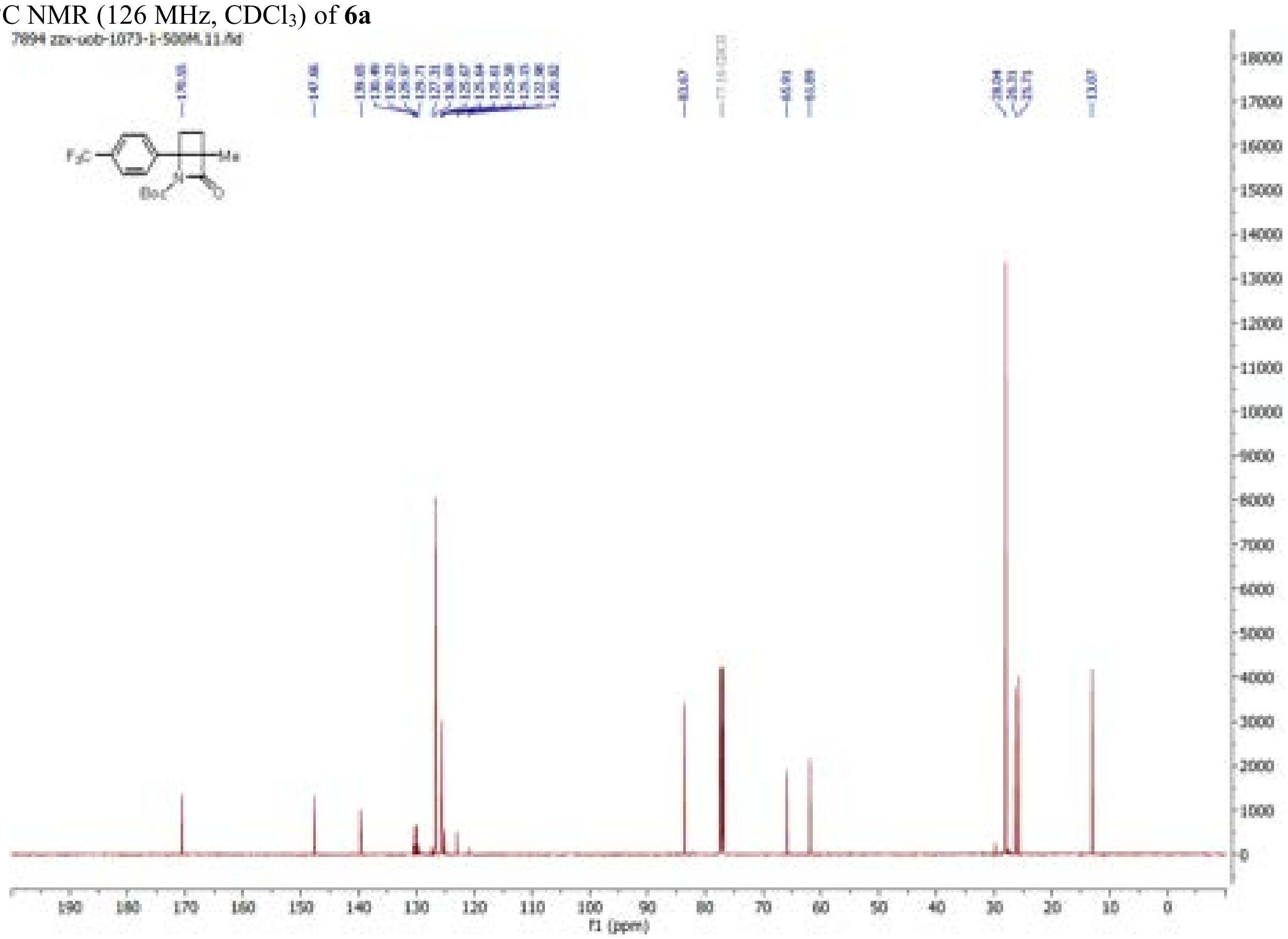

^{19}F NMR (471 MHz, $CDCl_3$) of **6a**

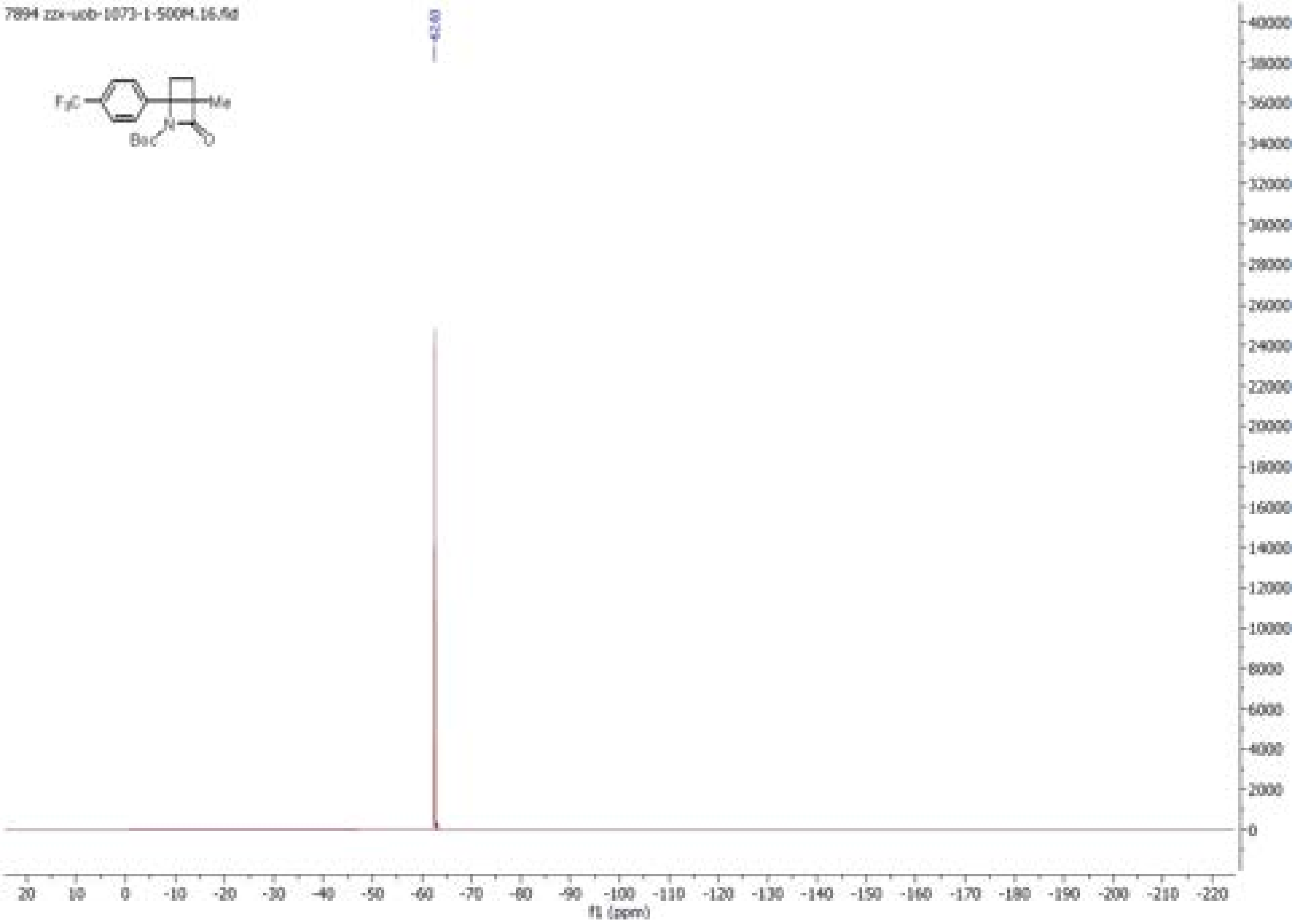

^{1}H NMR (500 MHz, $CDCl_3$) of **5a**

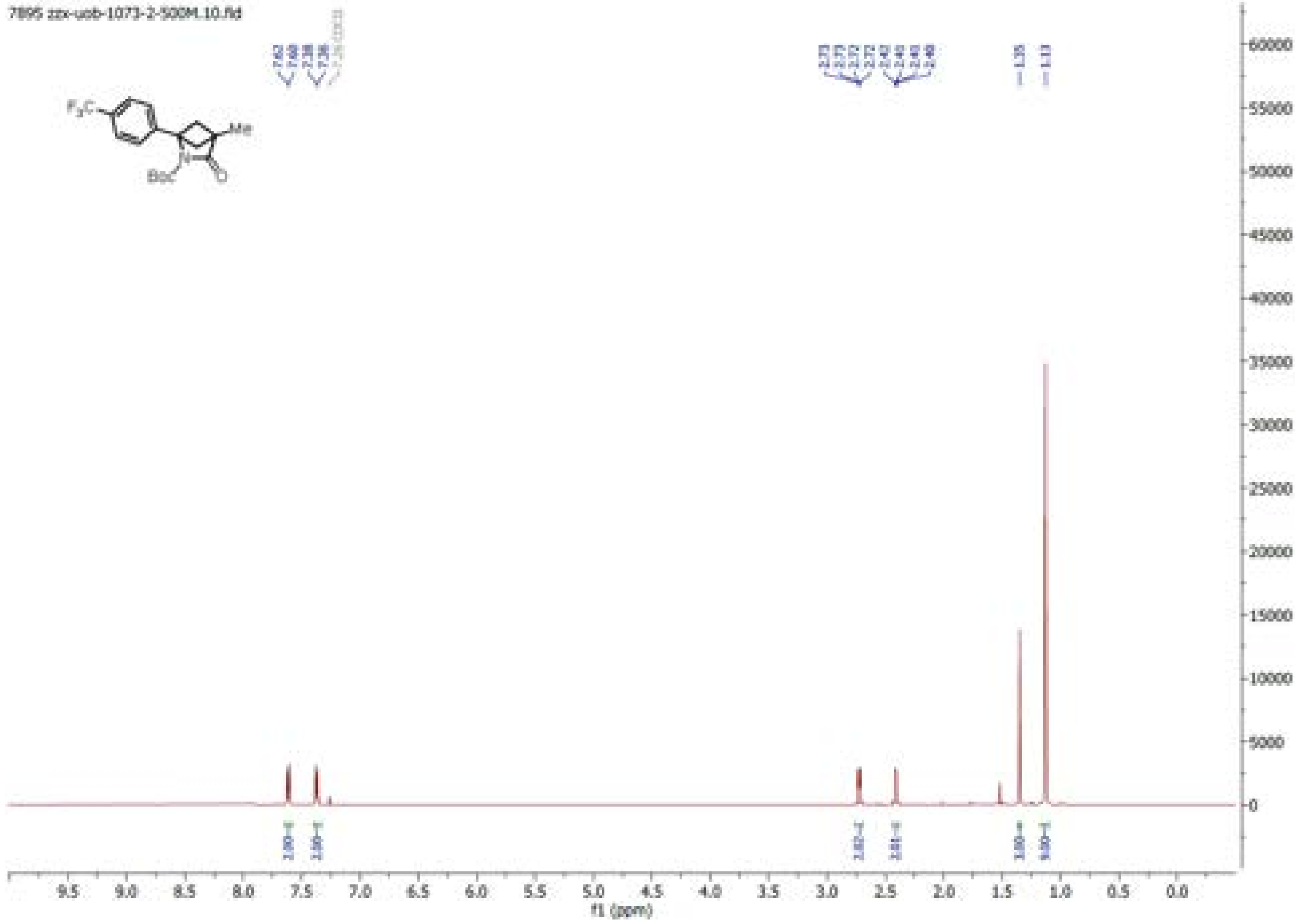

^{13}C NMR (126 MHz, $CDCl_3$) of **5a**

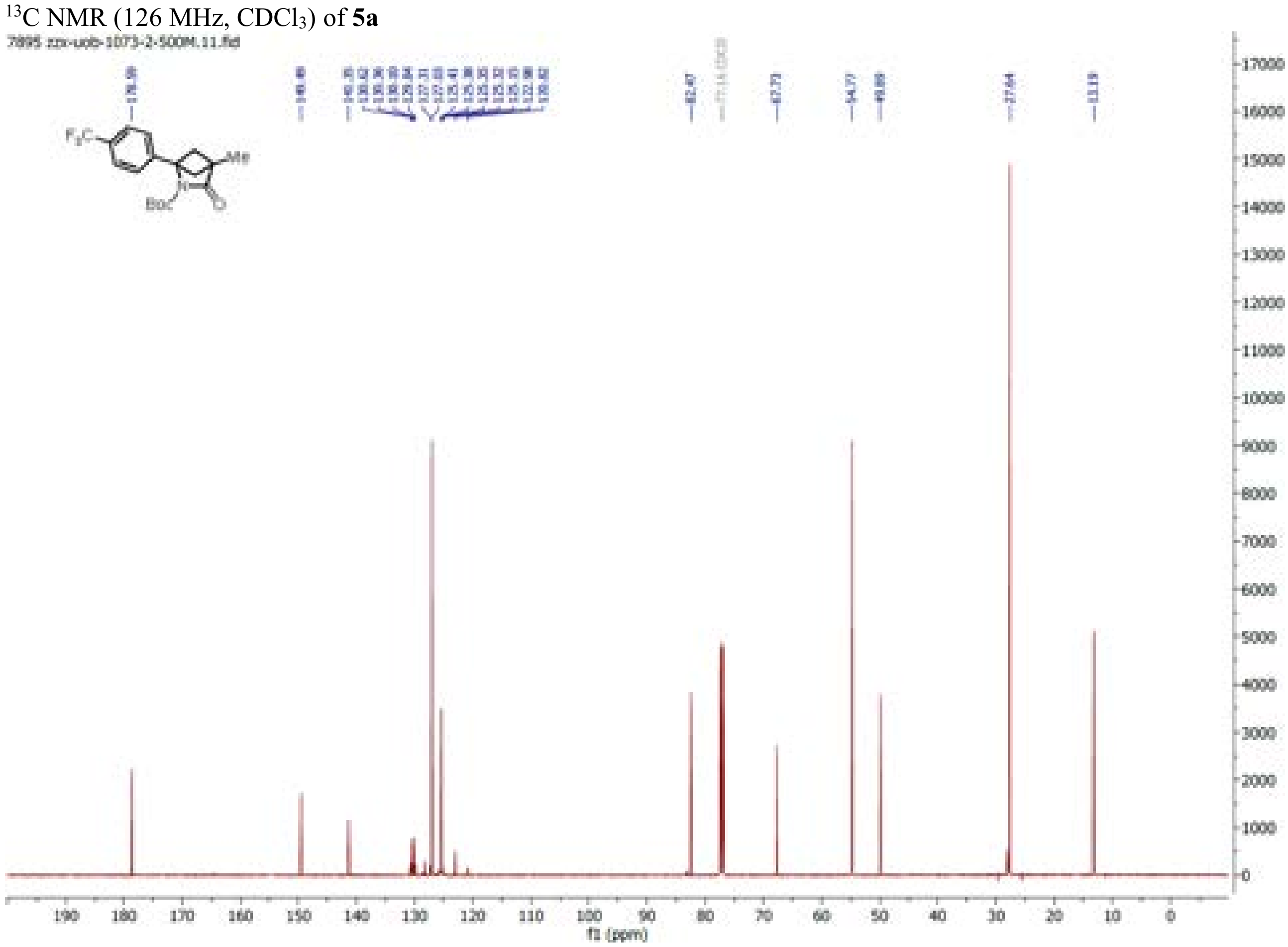

^{19}F NMR (471 MHz, $CDCl_3$) of **5a**

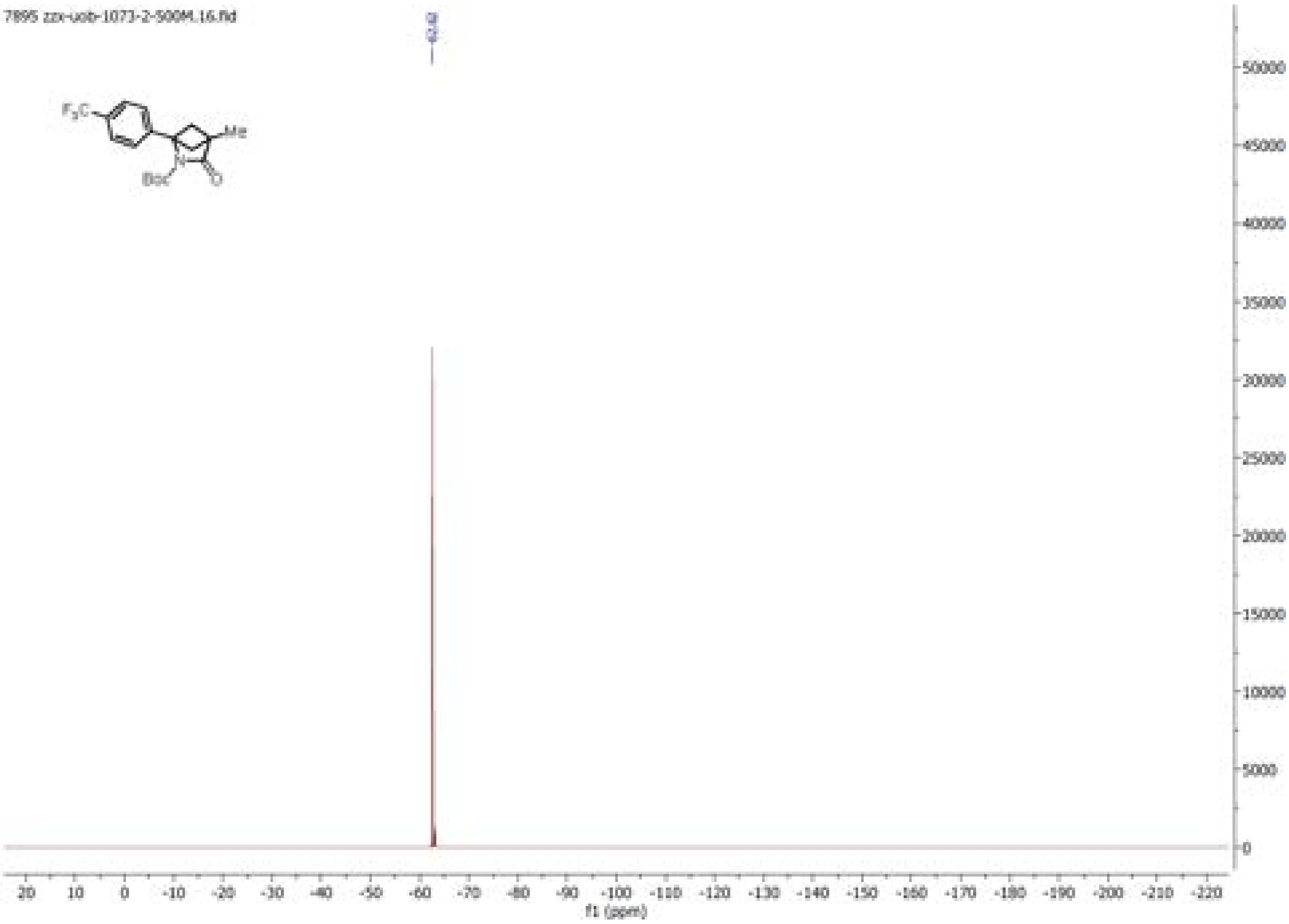

^{1}H NMR (500 MHz, $CDCl_3$) of **6b**

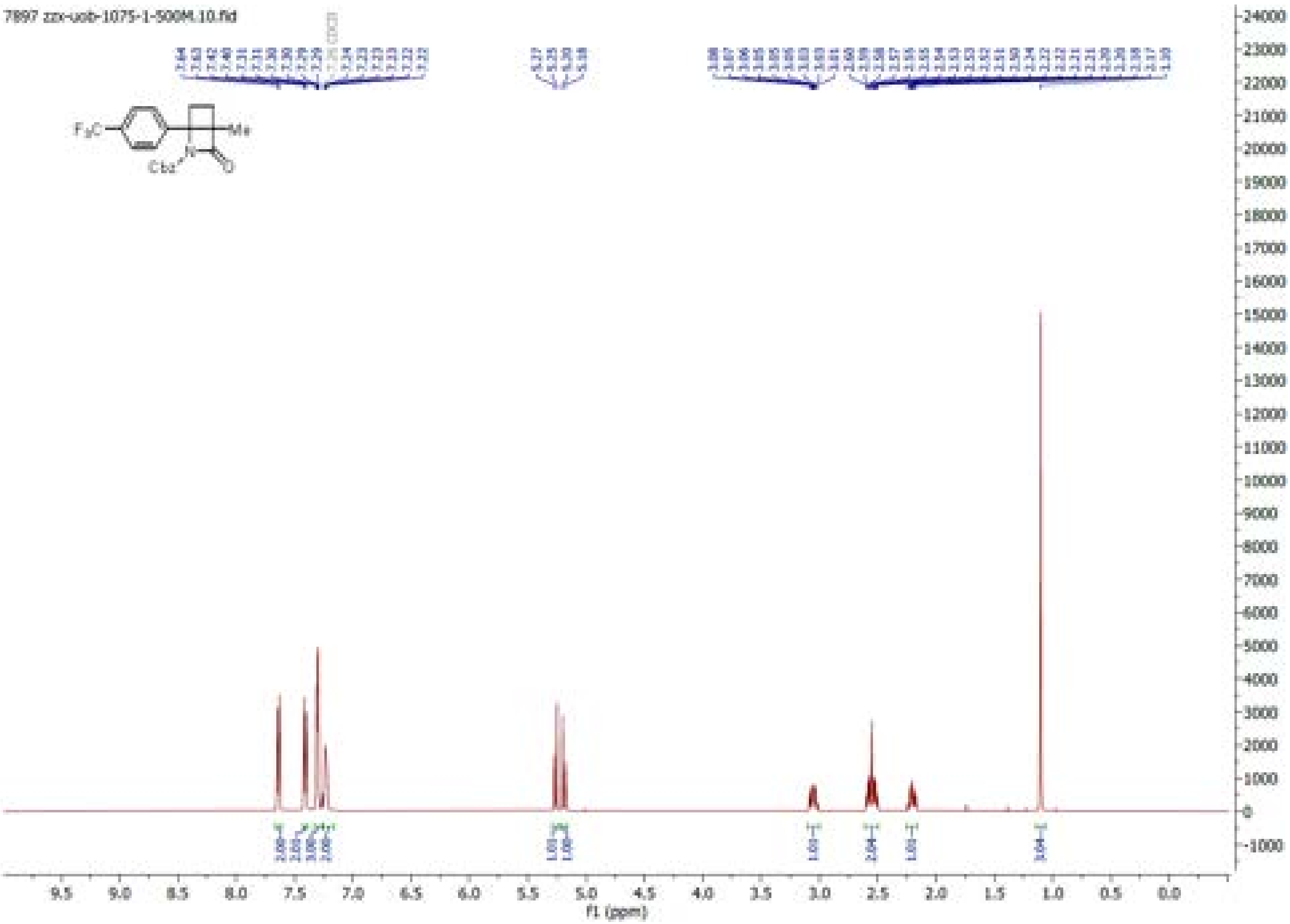

^{13}C NMR (126 MHz, $CDCl_3$) of **6b**

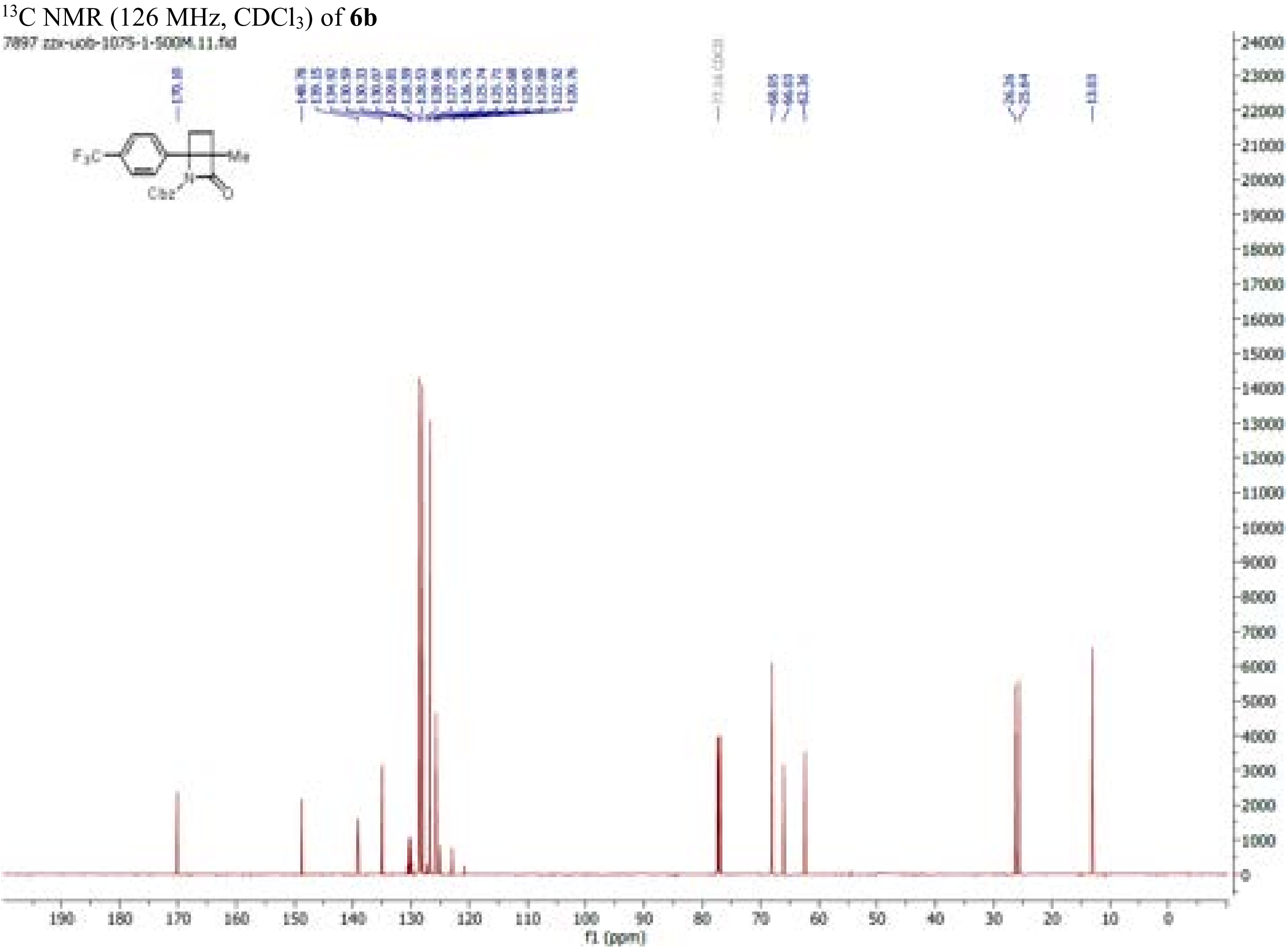

^{19}F NMR (471 MHz, $CDCl_3$) of **6b**

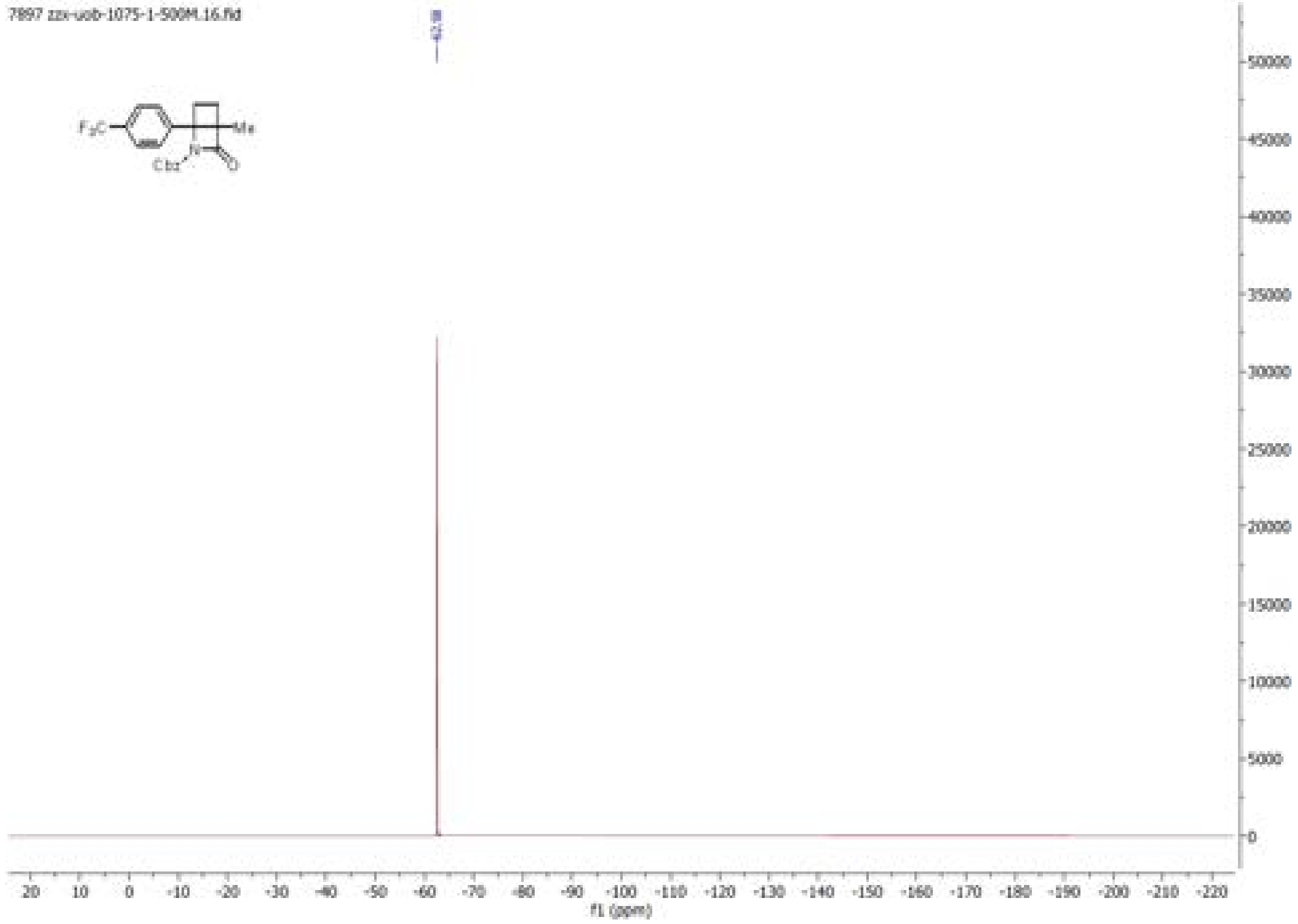

^{1}H NMR (500 MHz, $CDCl_3$) of **5b**

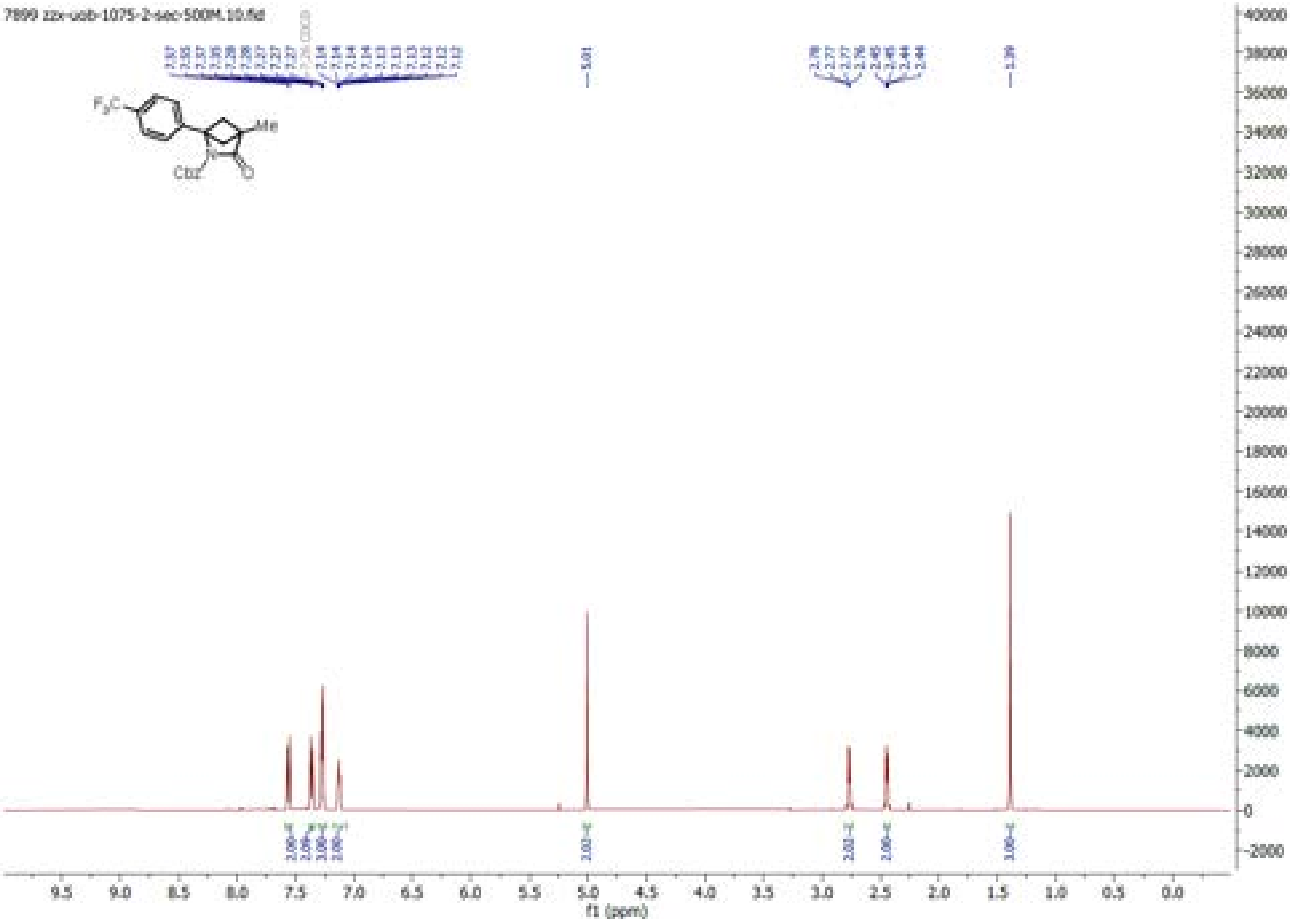

^{13}C NMR (126 MHz, $CDCl_3$) of **5b**

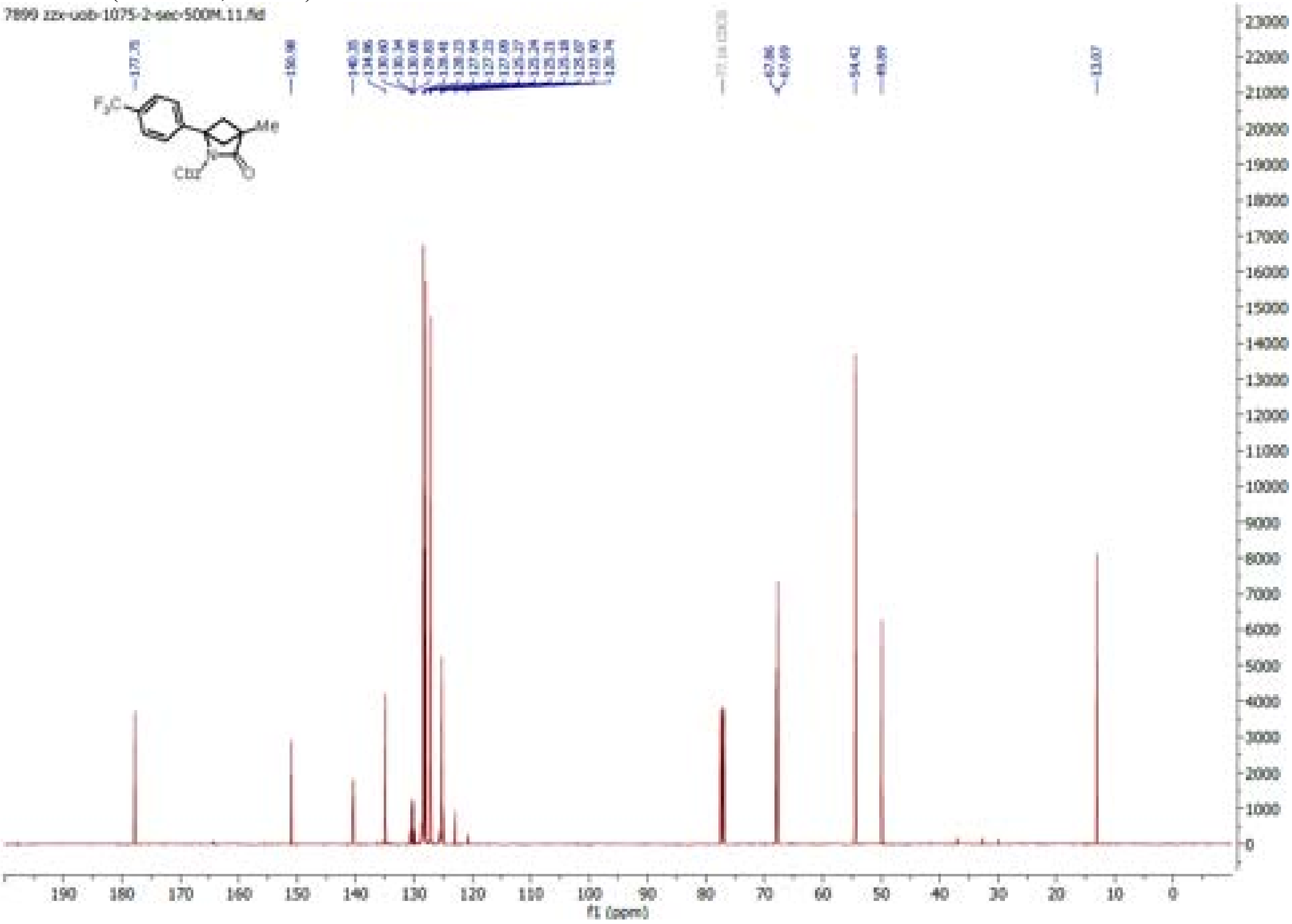

^{19}F NMR (471 MHz, $CDCl_3$) of **5b**

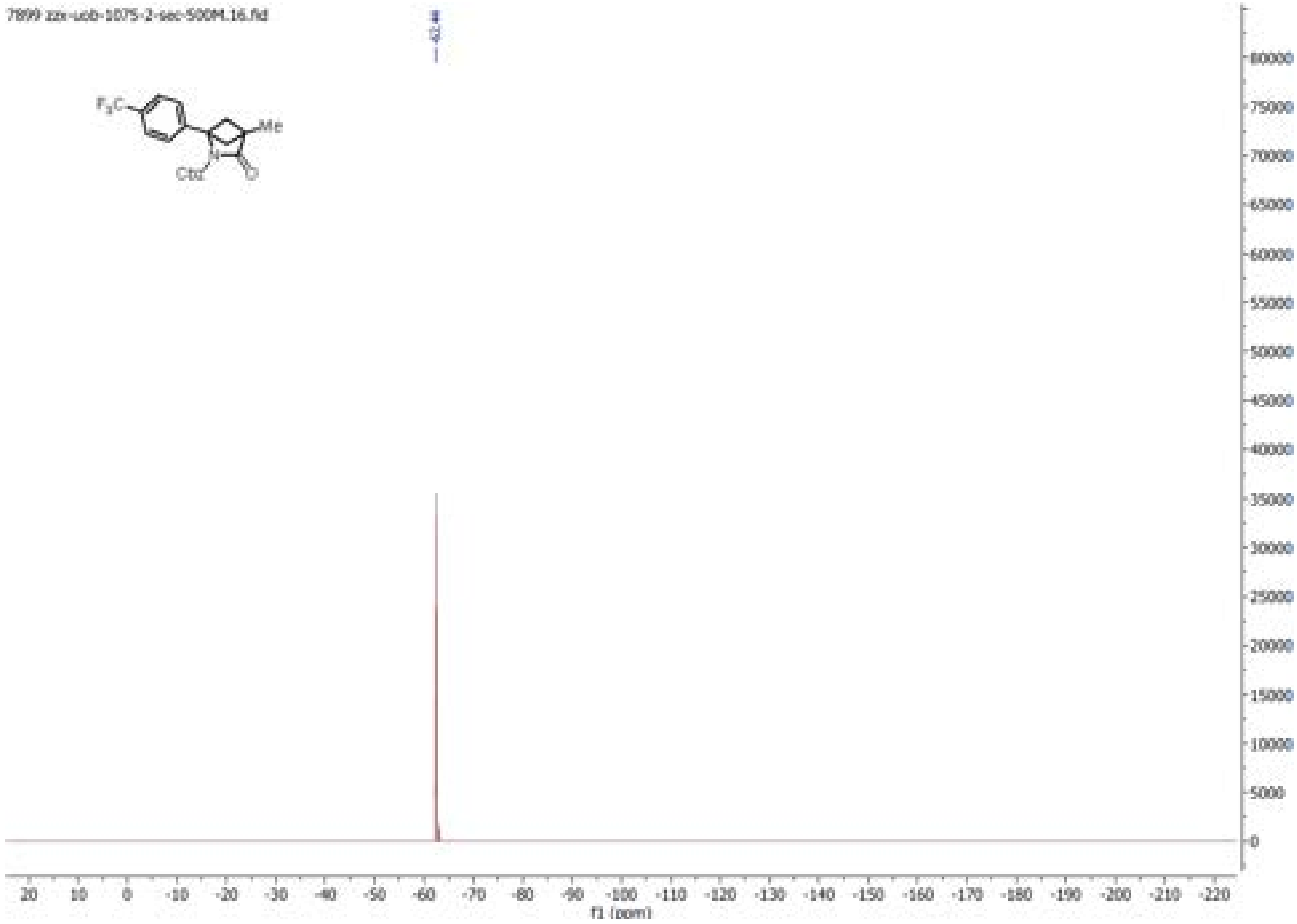

^{1}H NMR (500 MHz, $CDCl_3$) of **6c**

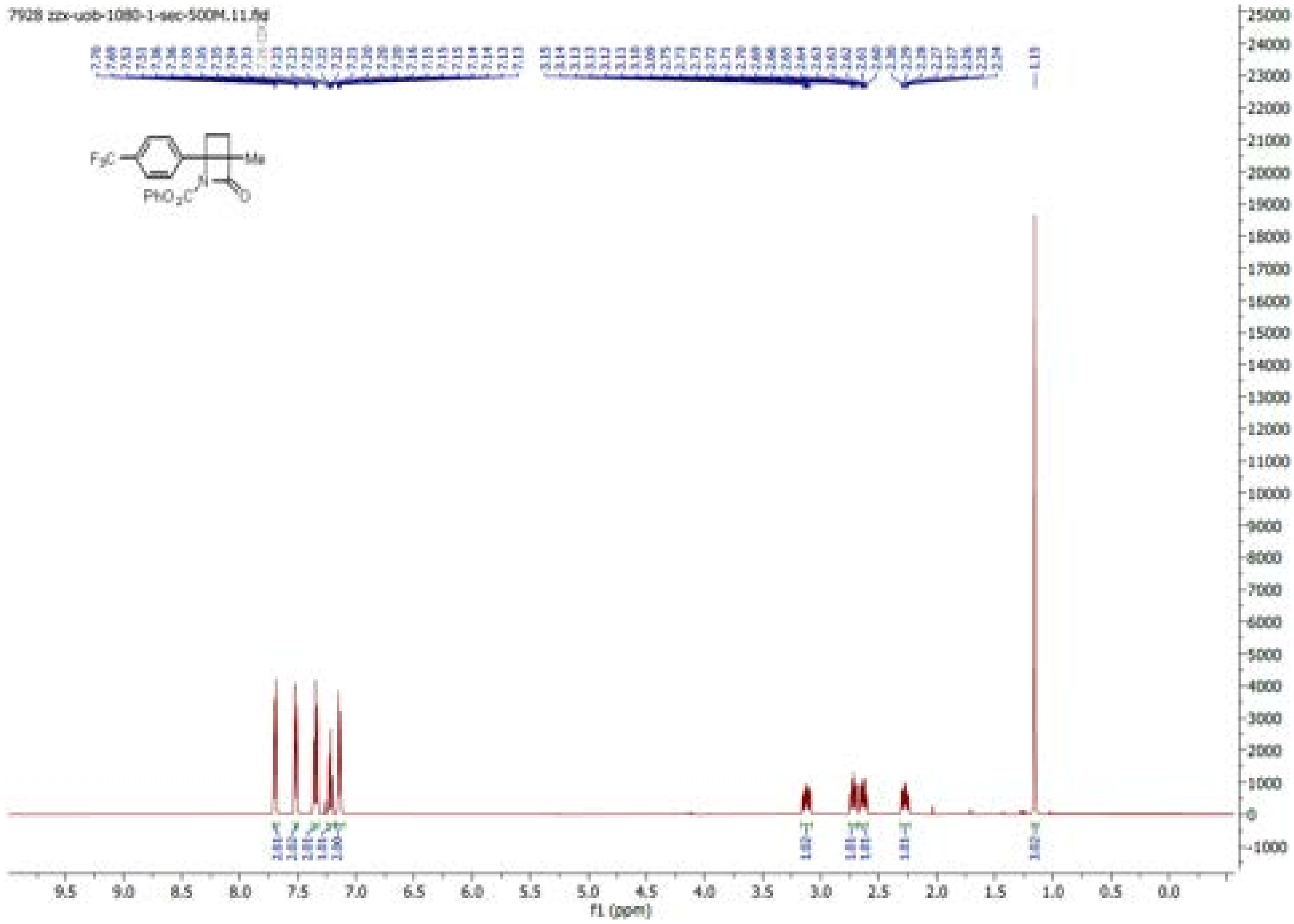

^{13}C NMR (126 MHz, $CDCl_3$) of **6c**

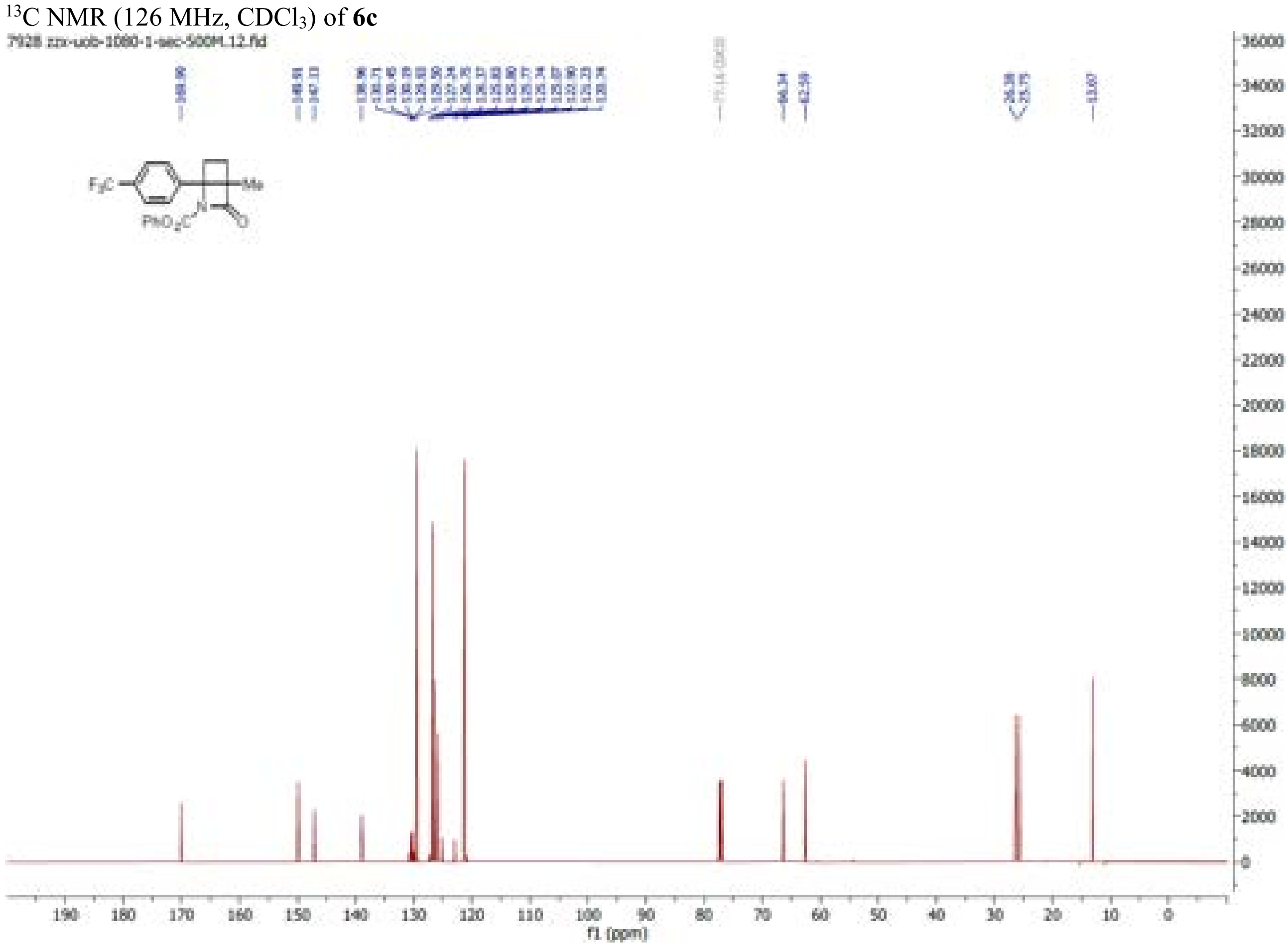

^{19}F NMR (471 MHz, $CDCl_3$) of **6c**

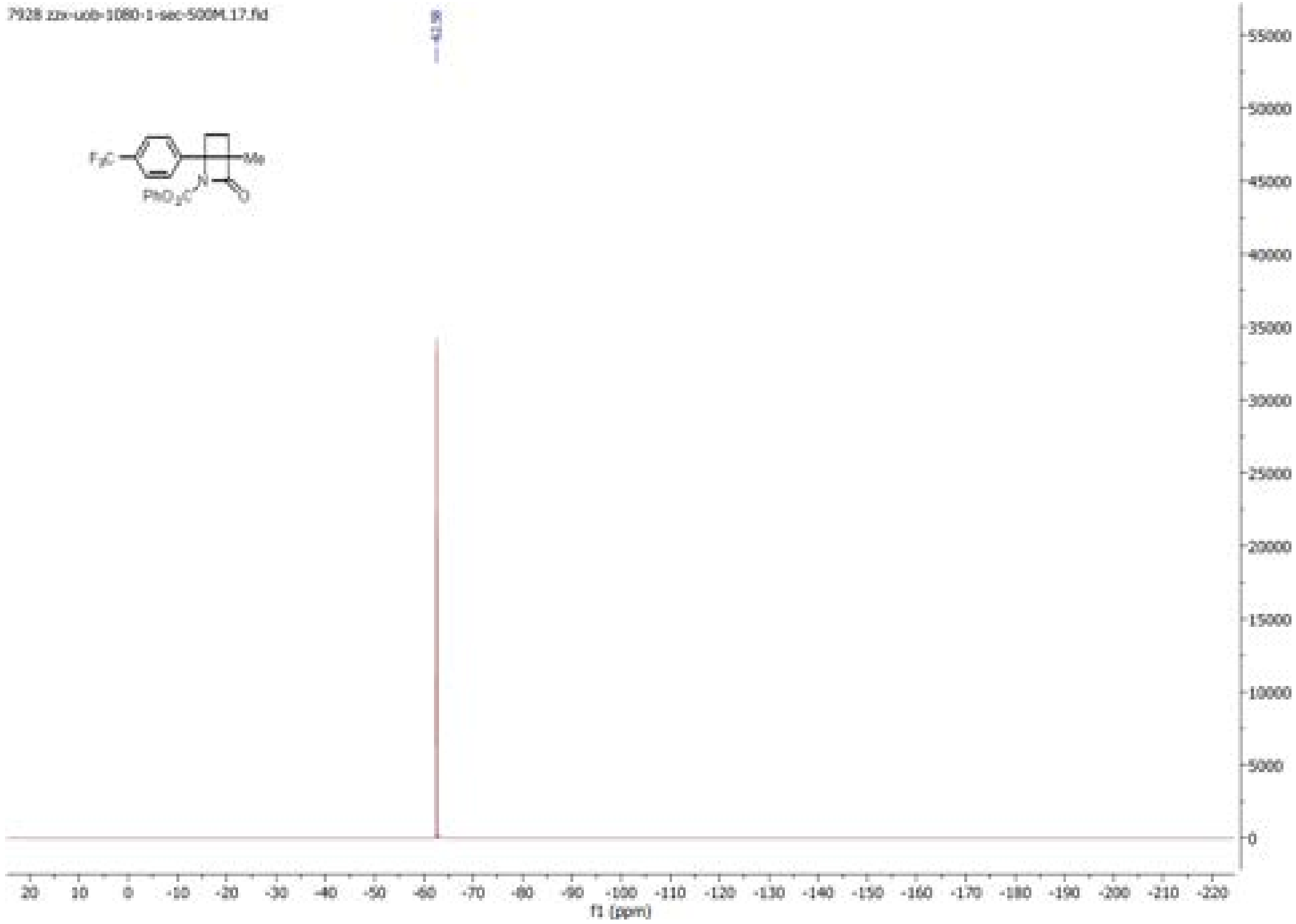

^{1}H NMR (500 MHz, $CDCl_3$) of **5c**

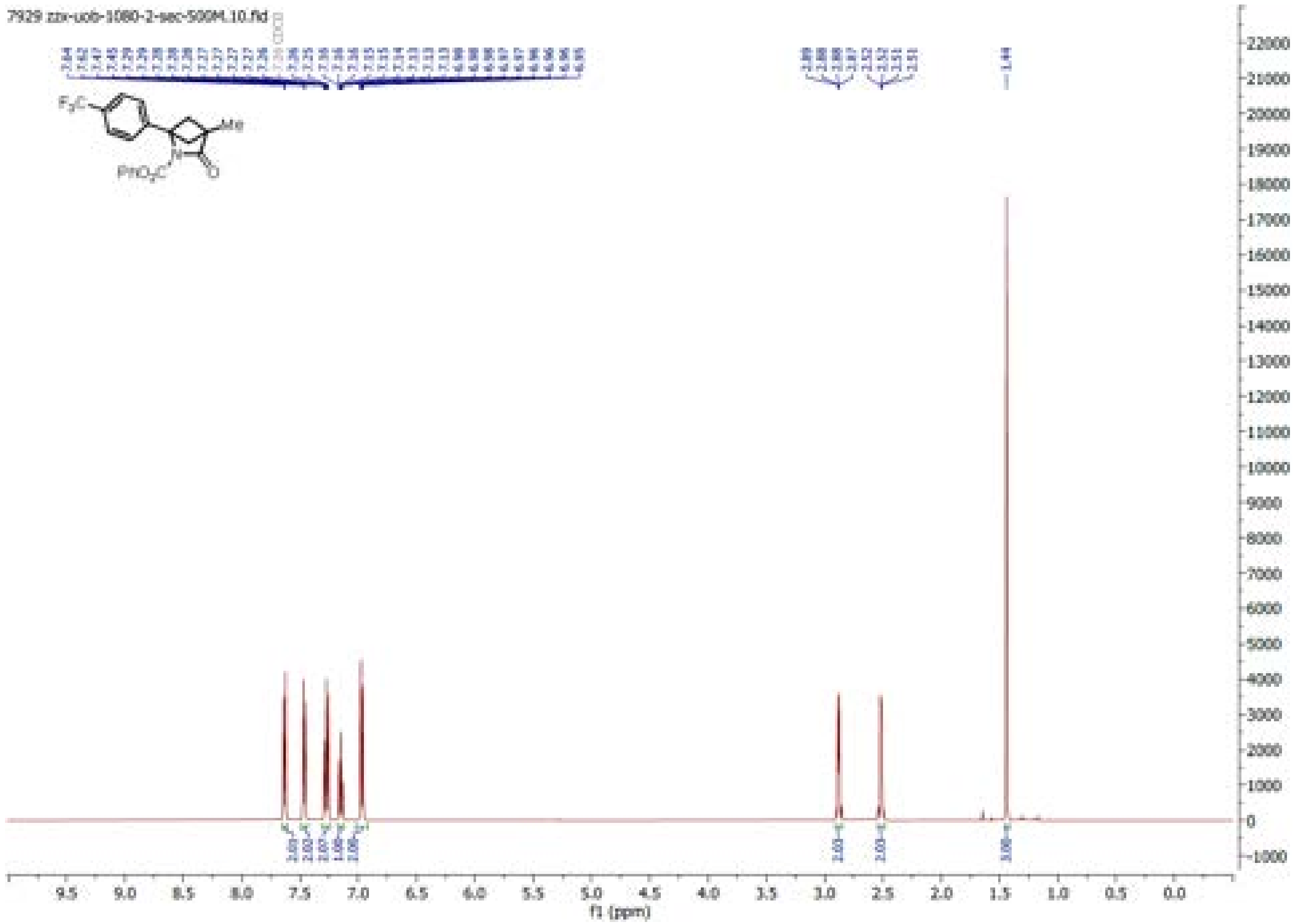

^{13}C NMR (126 MHz, $CDCl_3$) of **5c**

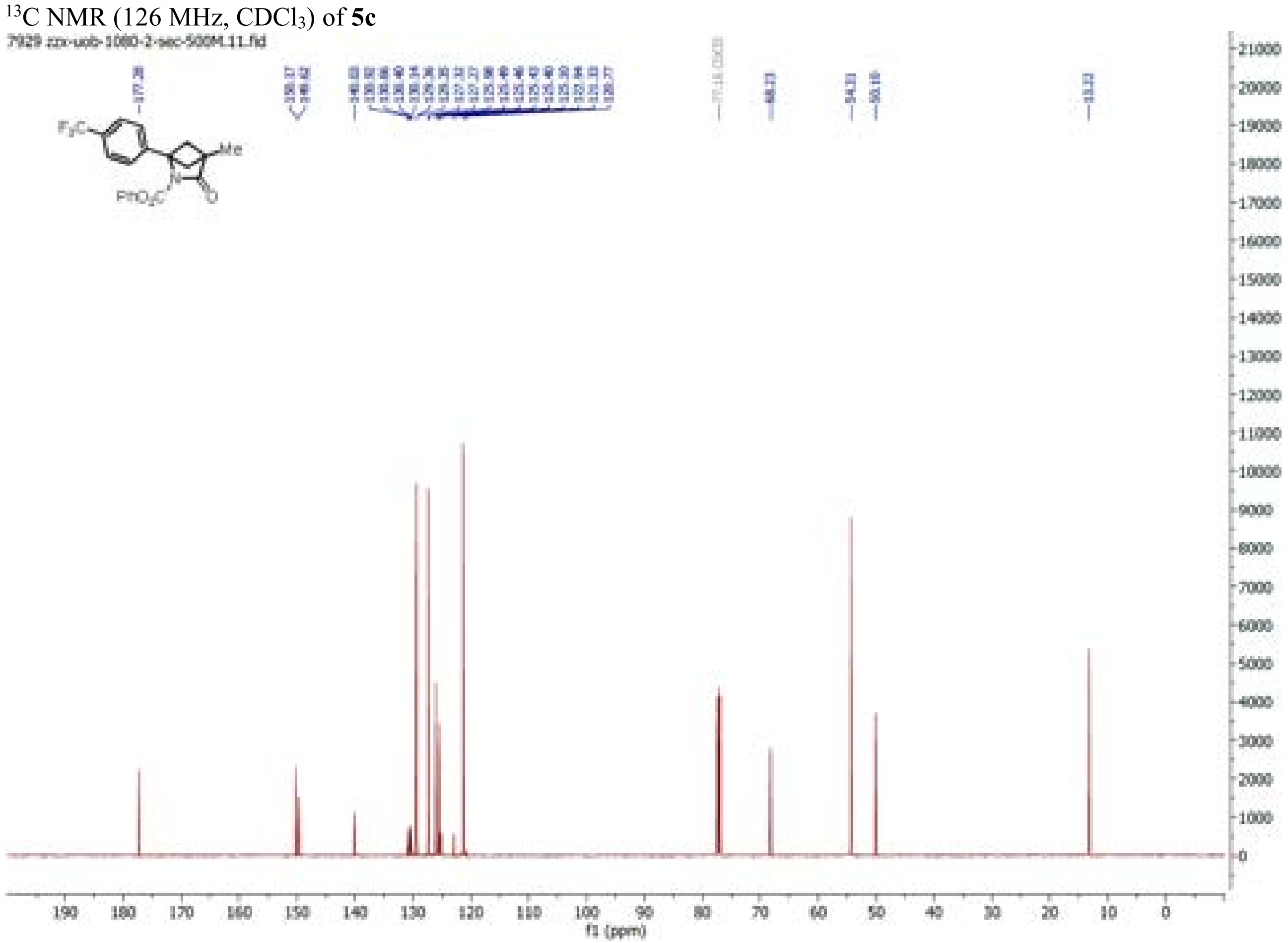

^{19}F NMR (471 MHz, $CDCl_3$) of **5c**

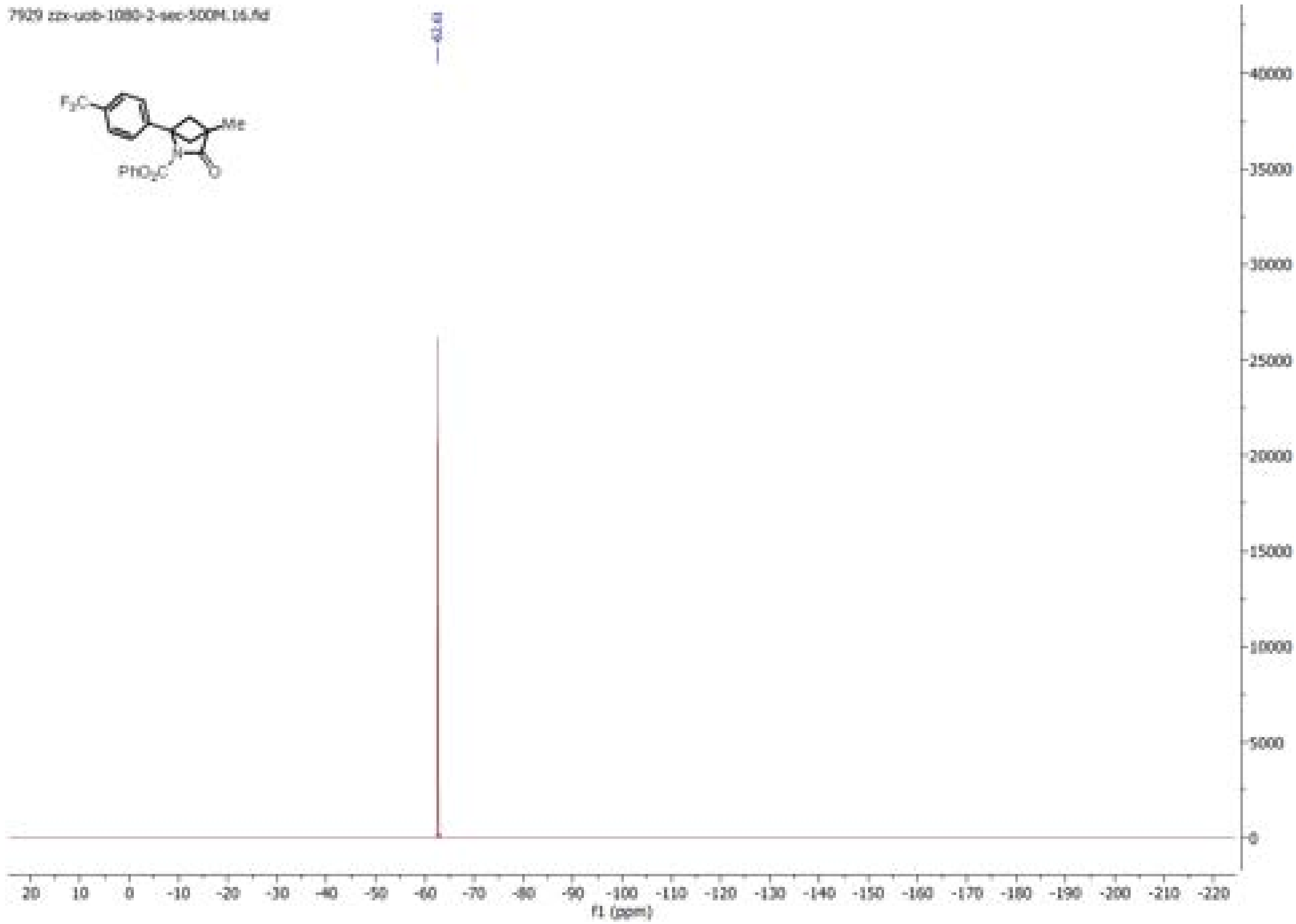

^{1}H NMR (500 MHz, $CDCl_3$) of **6d**

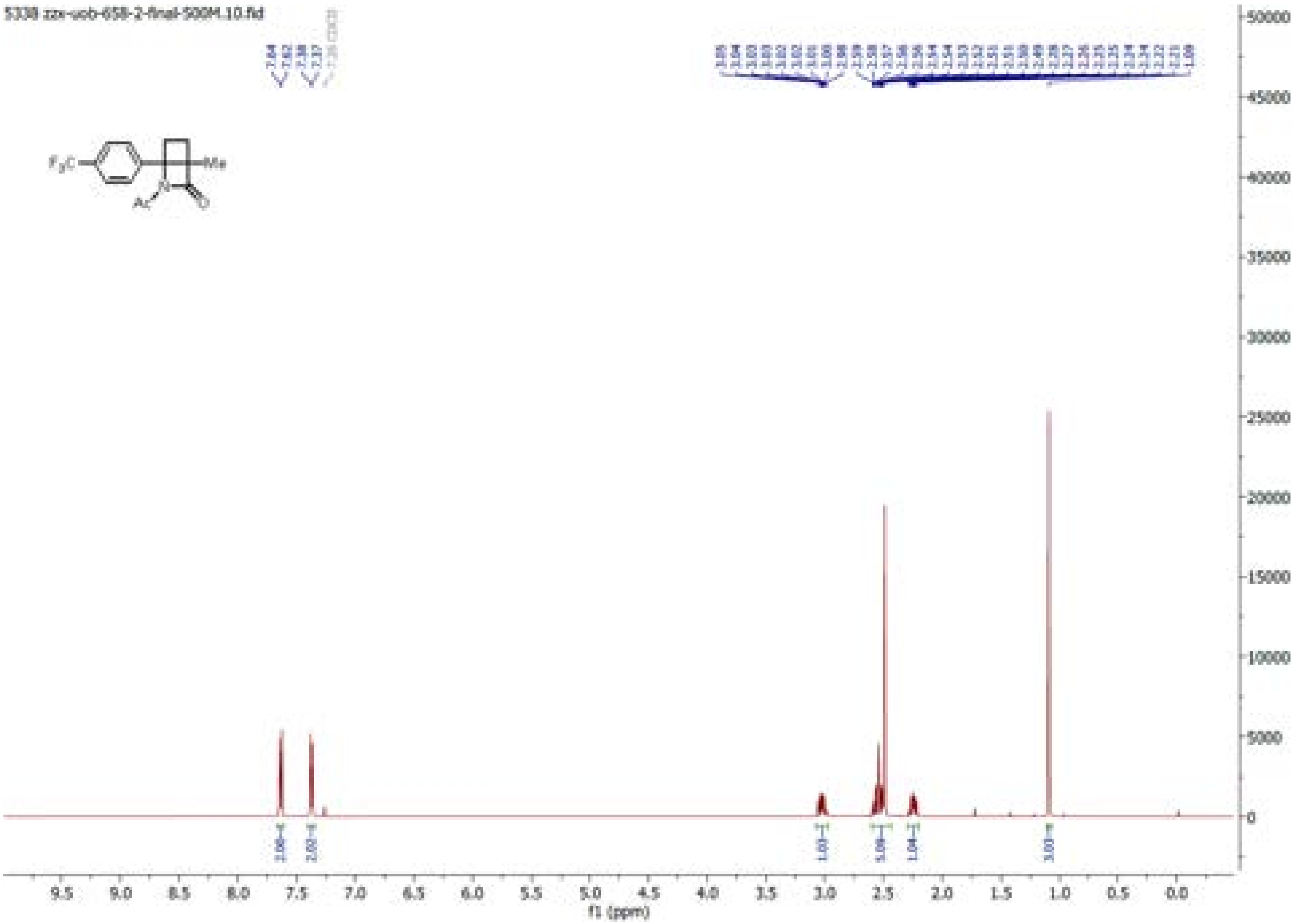

^{13}C NMR (126 MHz, $CDCl_3$) of **6d**

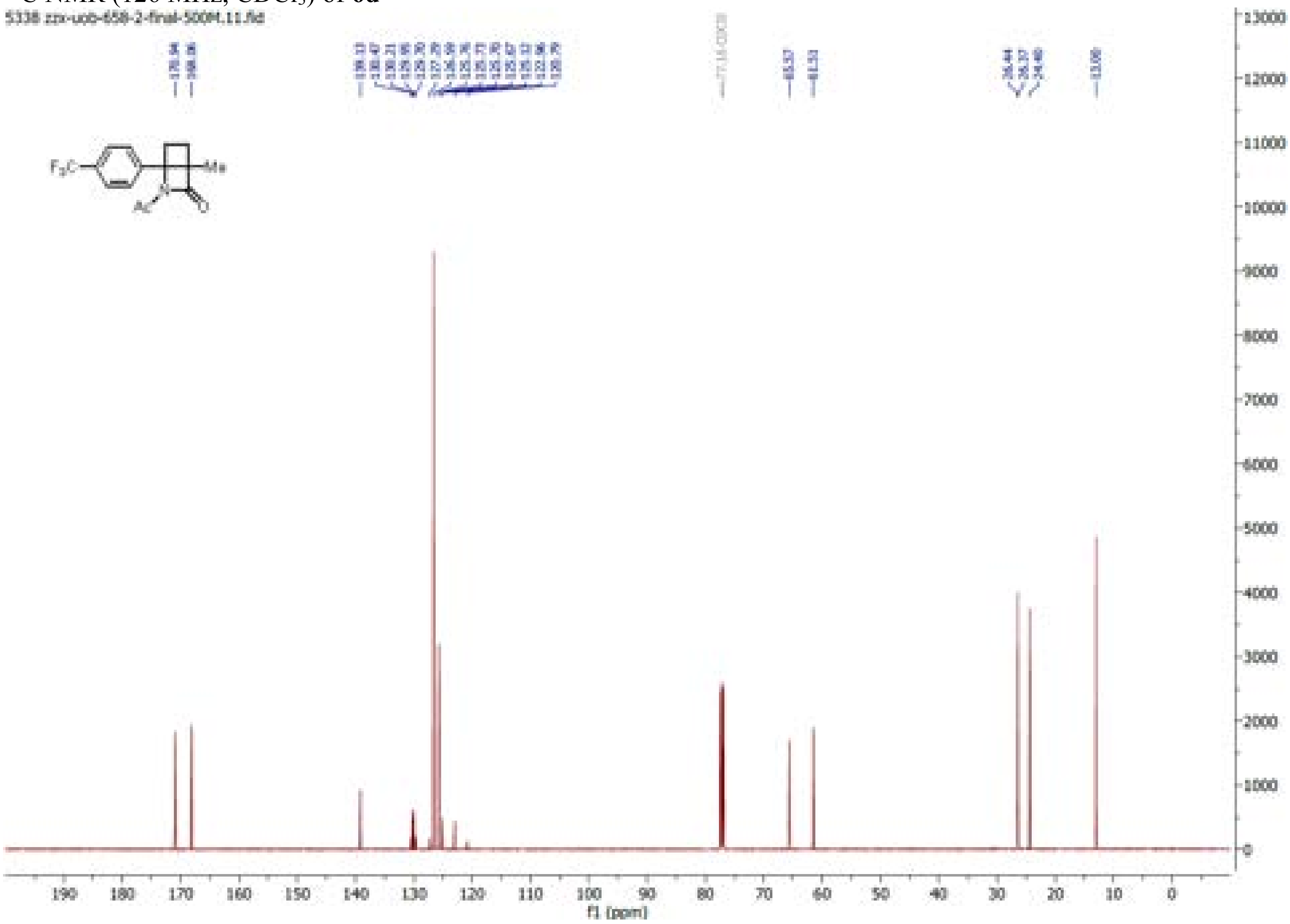

^{19}F NMR (471 MHz, $CDCl_3$) of **6d**

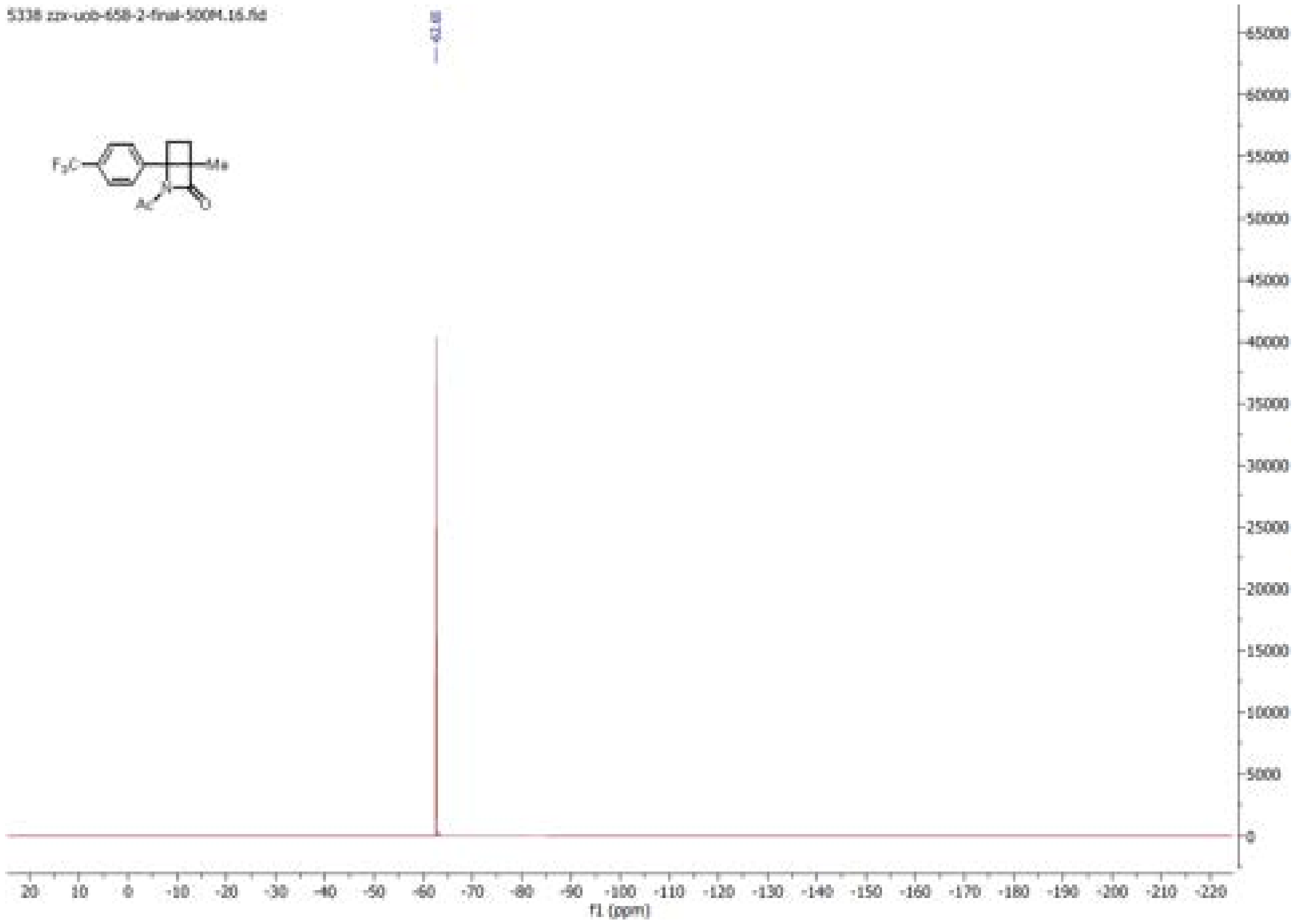

^{1}H NMR (500 MHz, $CDCl_3$) of **5d**

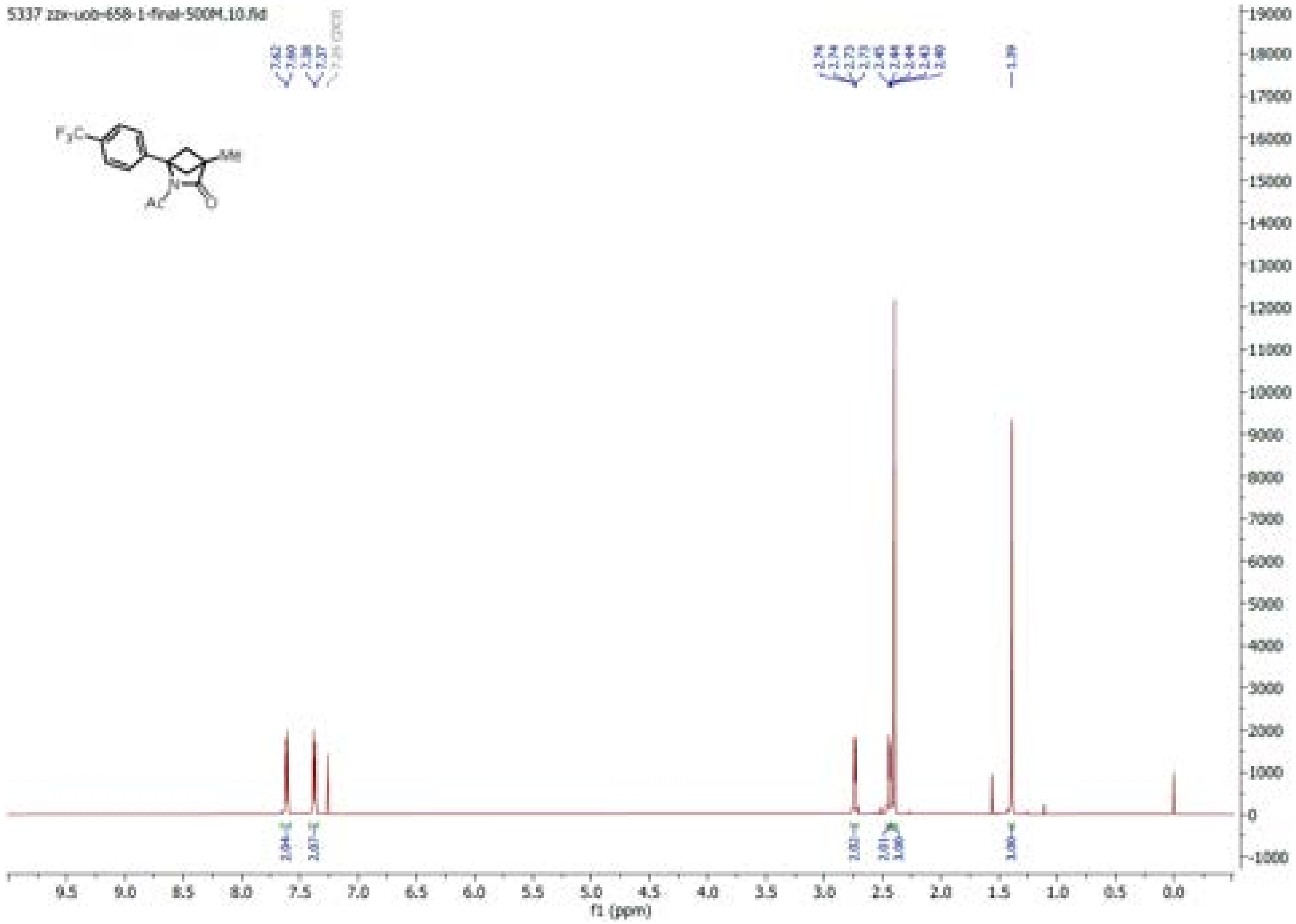

^{13}C NMR (126 MHz, $CDCl_3$) of **5d**

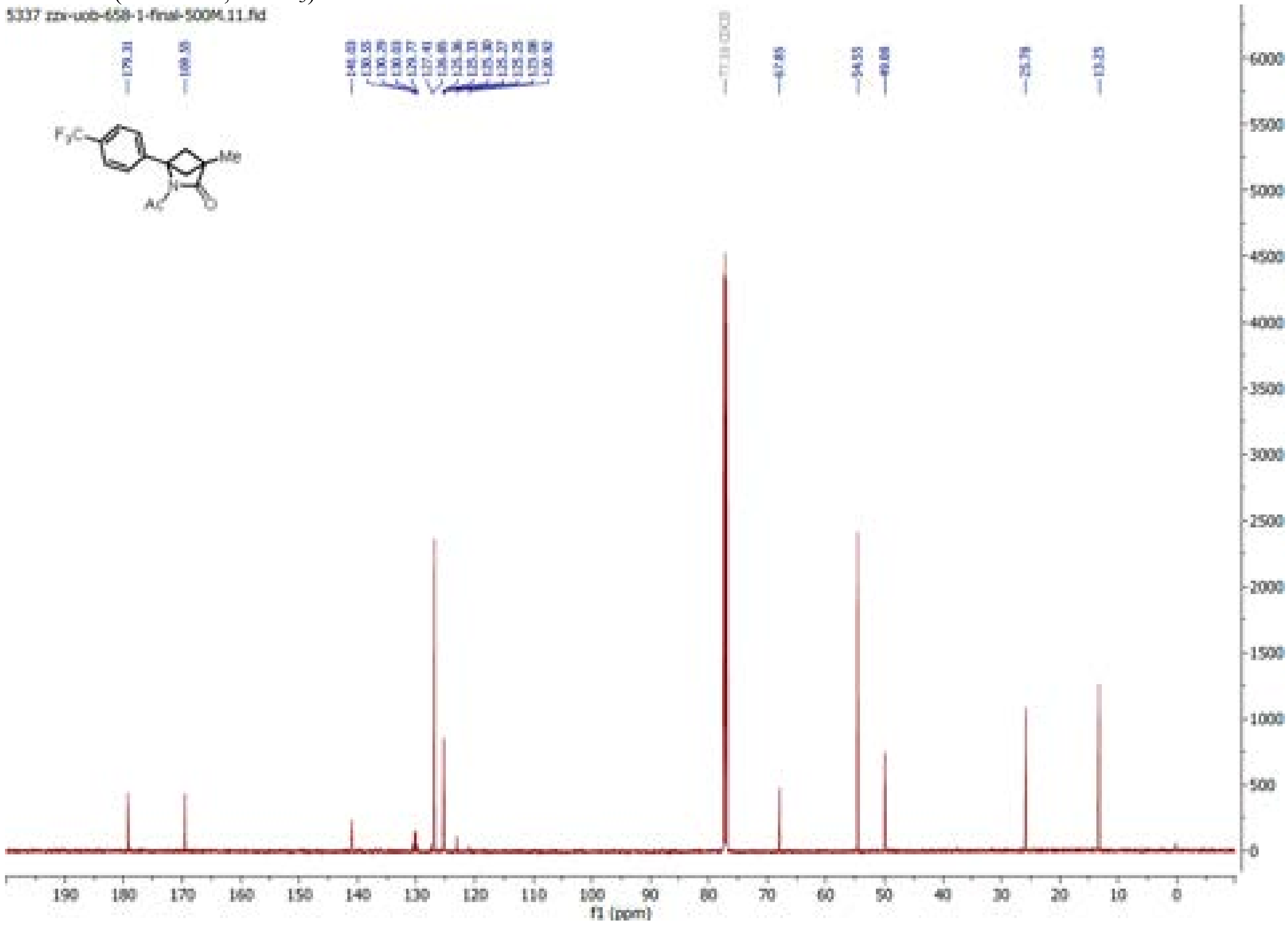

^{19}F NMR (471 MHz, $CDCl_3$) of **5d**

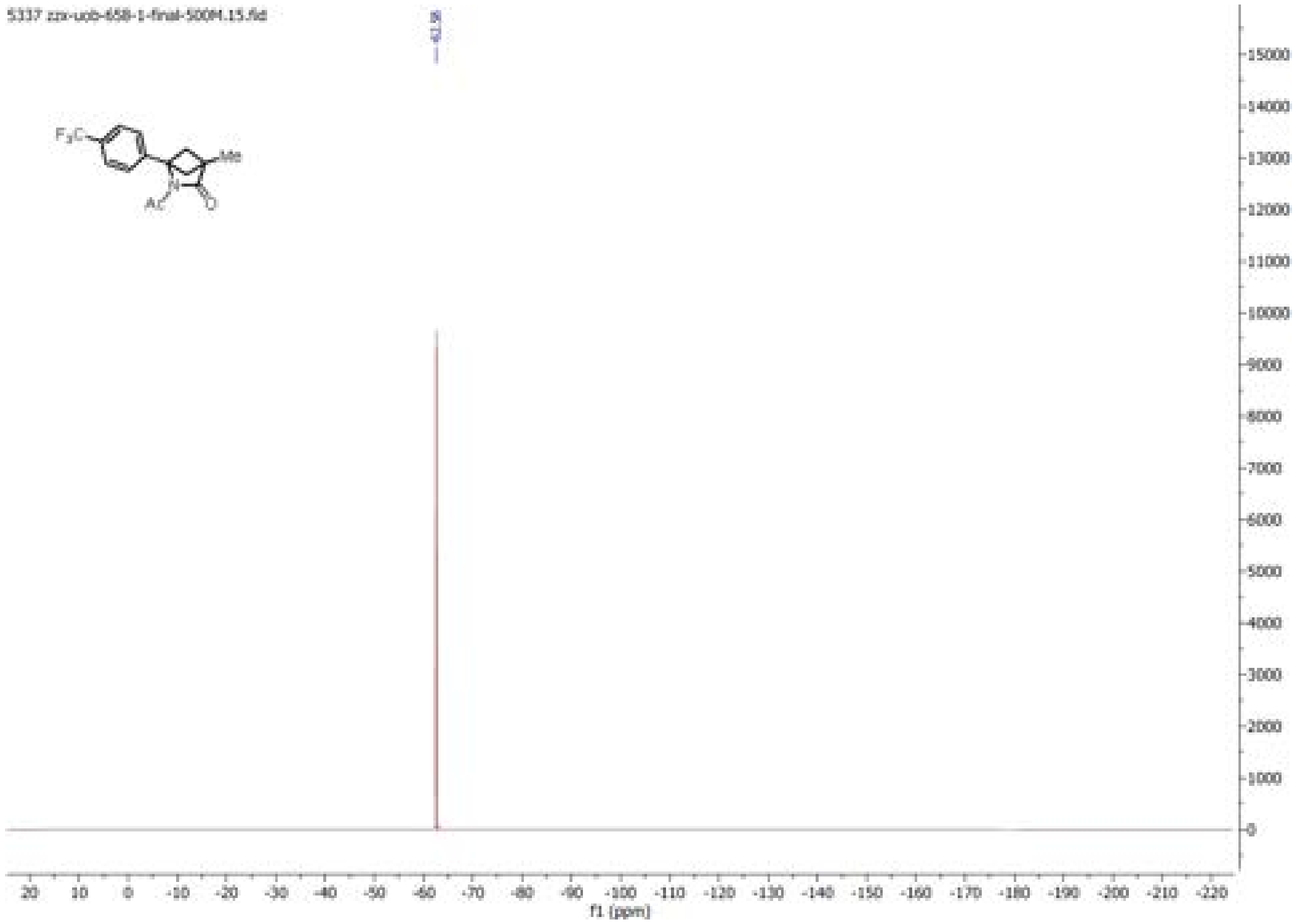

^{1}H NMR (500 MHz, $CDCl_3$) of **6e**

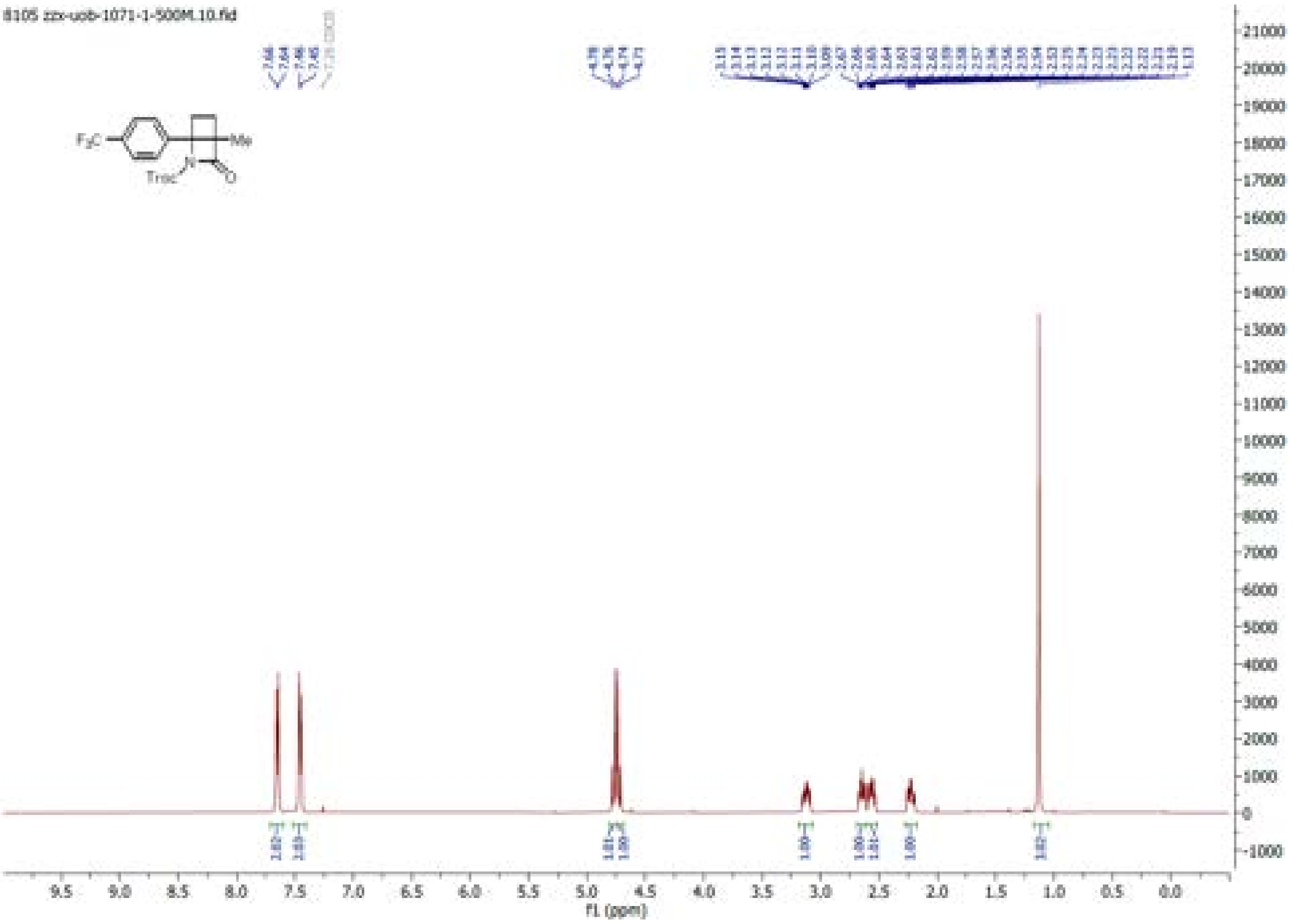

^{13}C NMR (126 MHz, $CDCl_3$) of **6e**

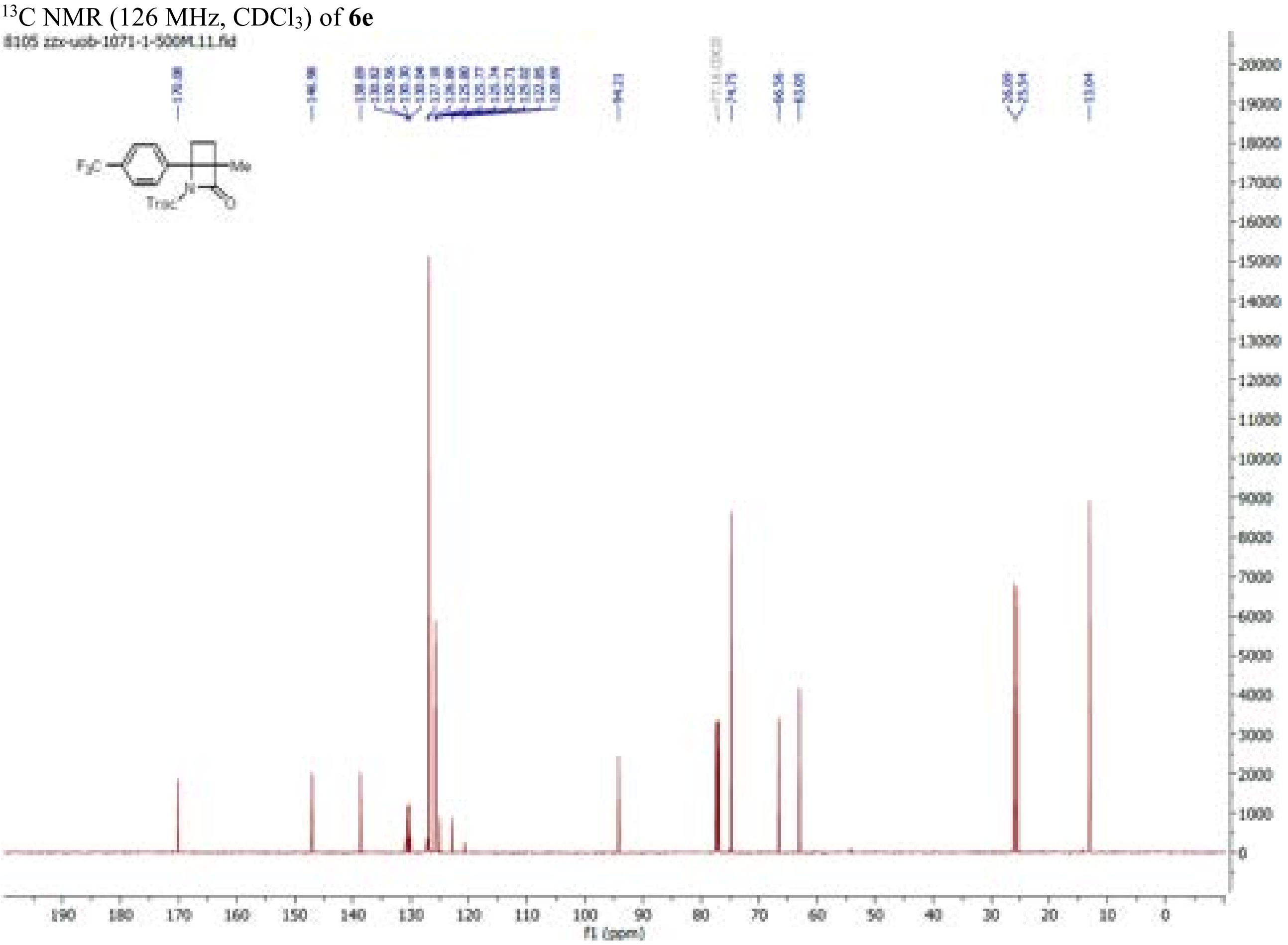

^{19}F NMR (471 MHz, $CDCl_3$) of **6e**

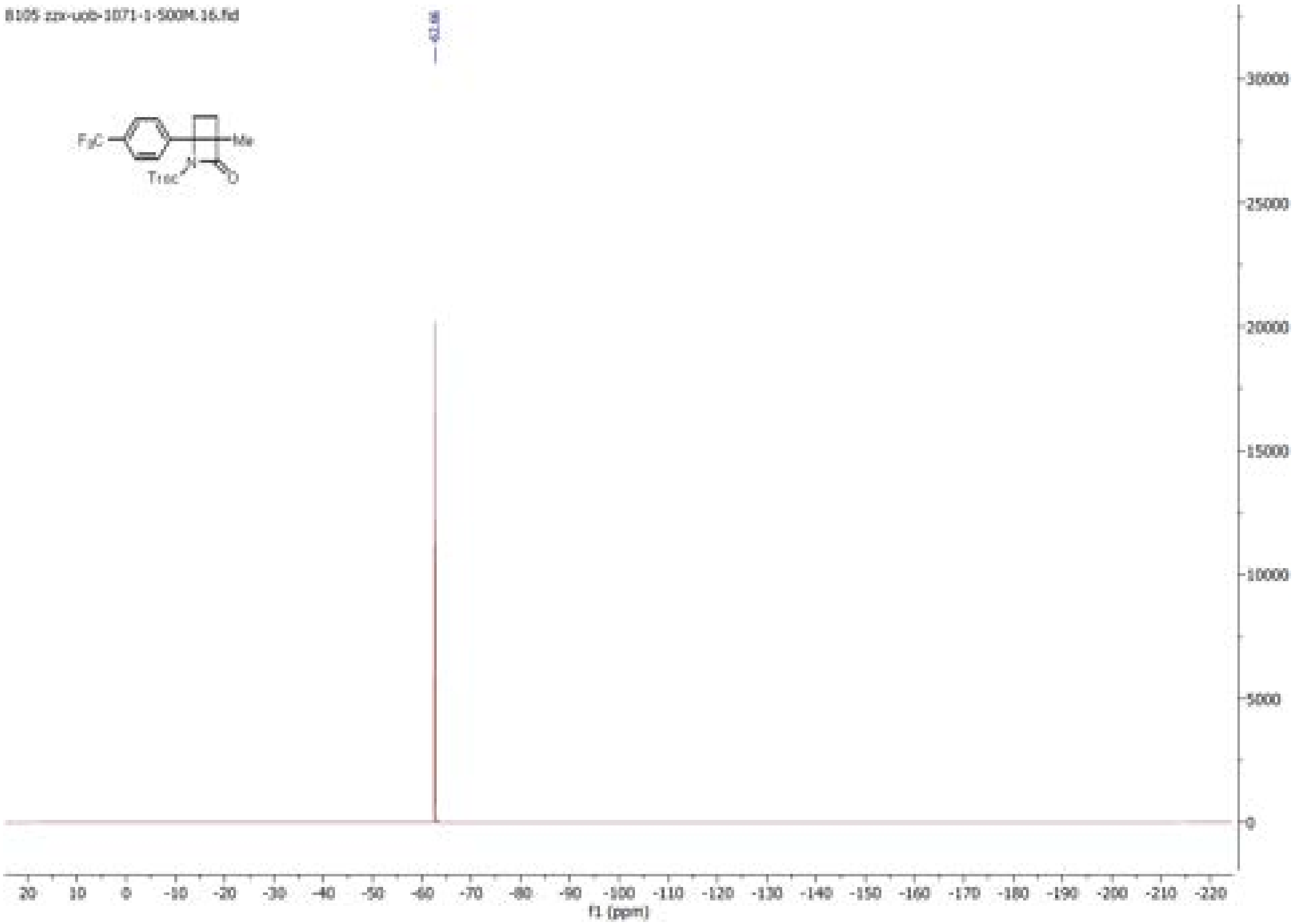

^{1}H NMR (500 MHz, $CDCl_3$) of **6f**

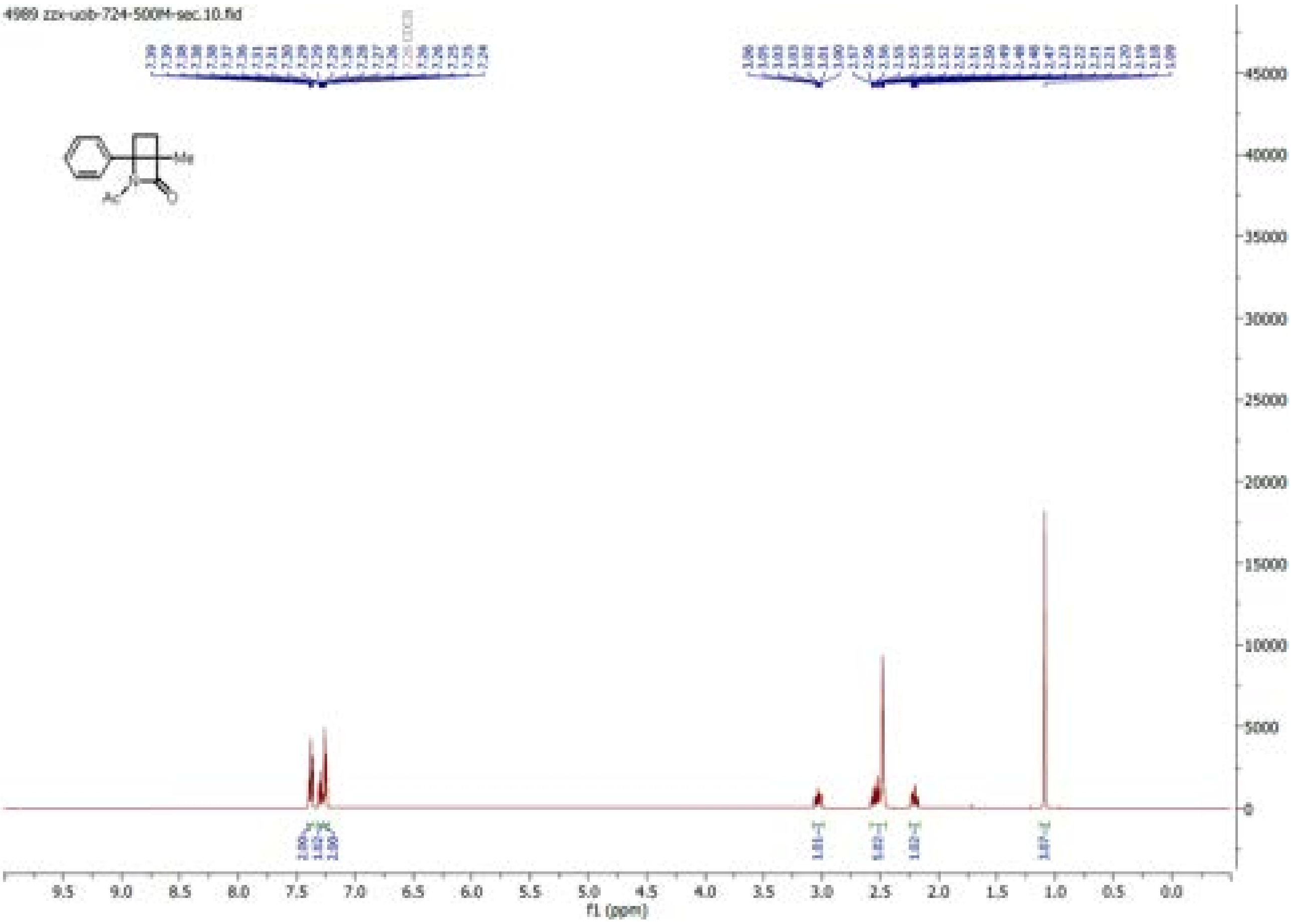

^{13}C NMR (126 MHz, $CDCl_3$) of **6f**

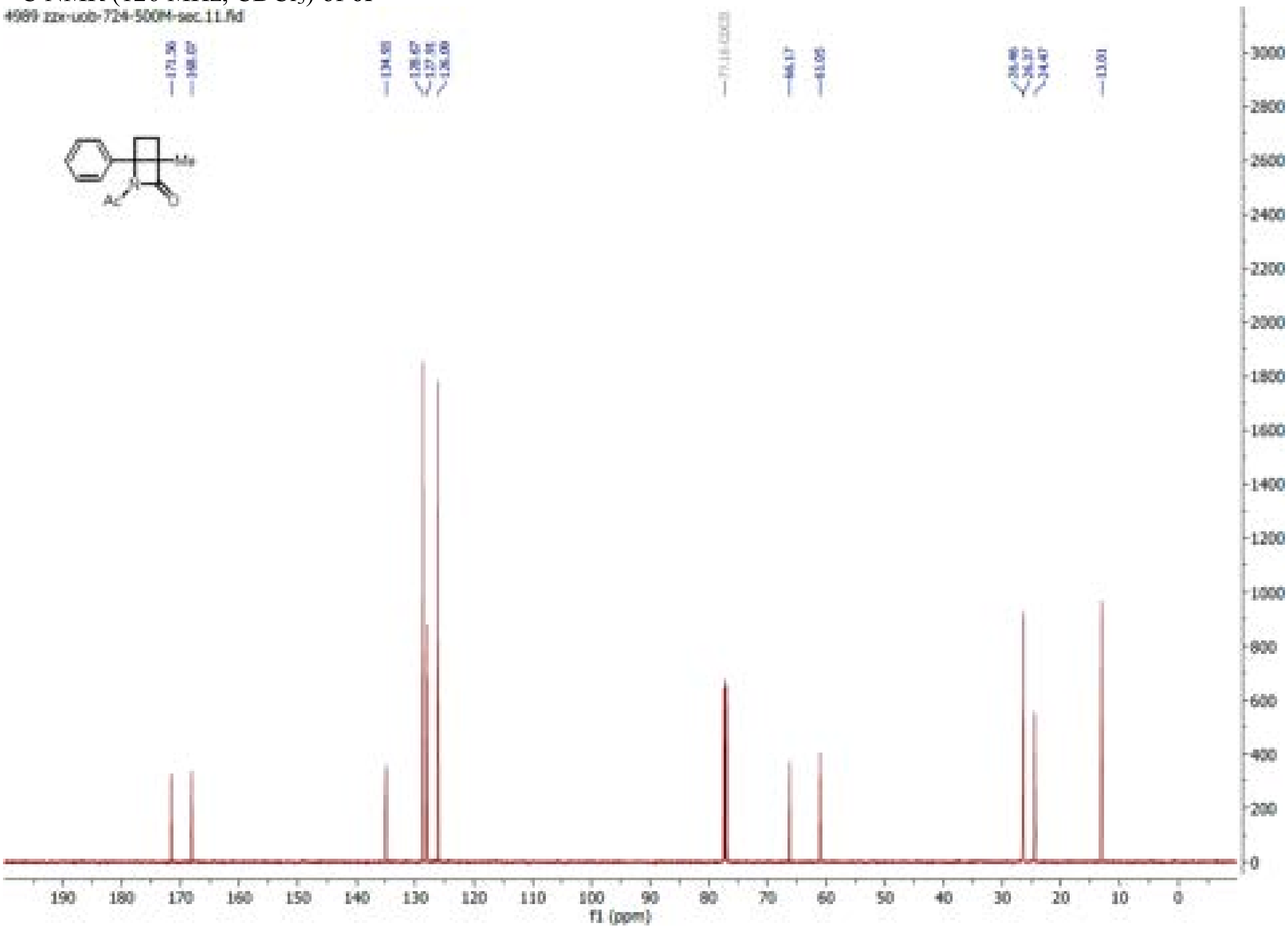

^{1}H NMR (500 MHz, $CDCl_3$) of **5f**

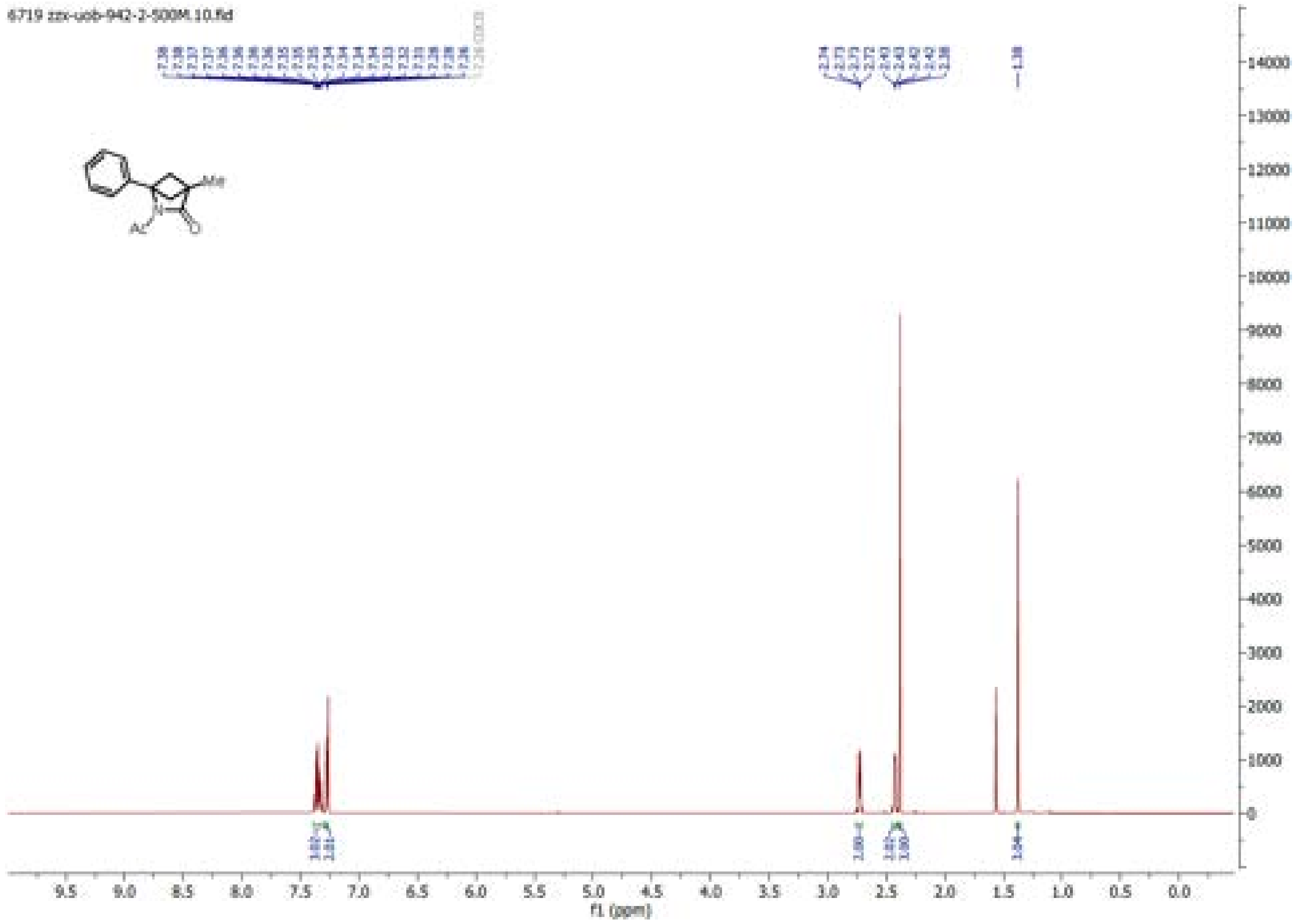

^{13}C NMR (126 MHz, $CDCl_3$) of **5f**

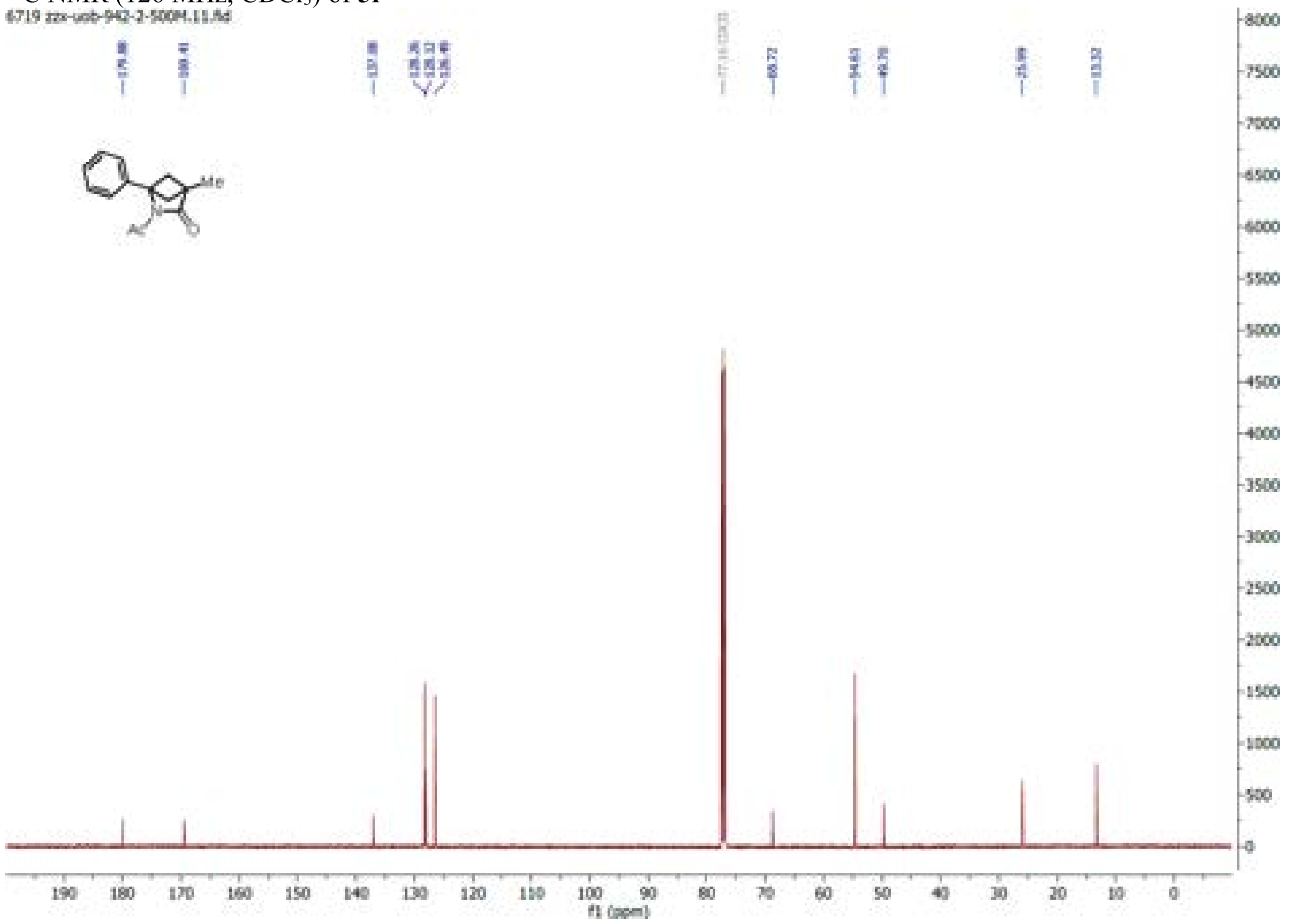

^{1}H NMR (500 MHz, $CDCl_3$) of **6g**

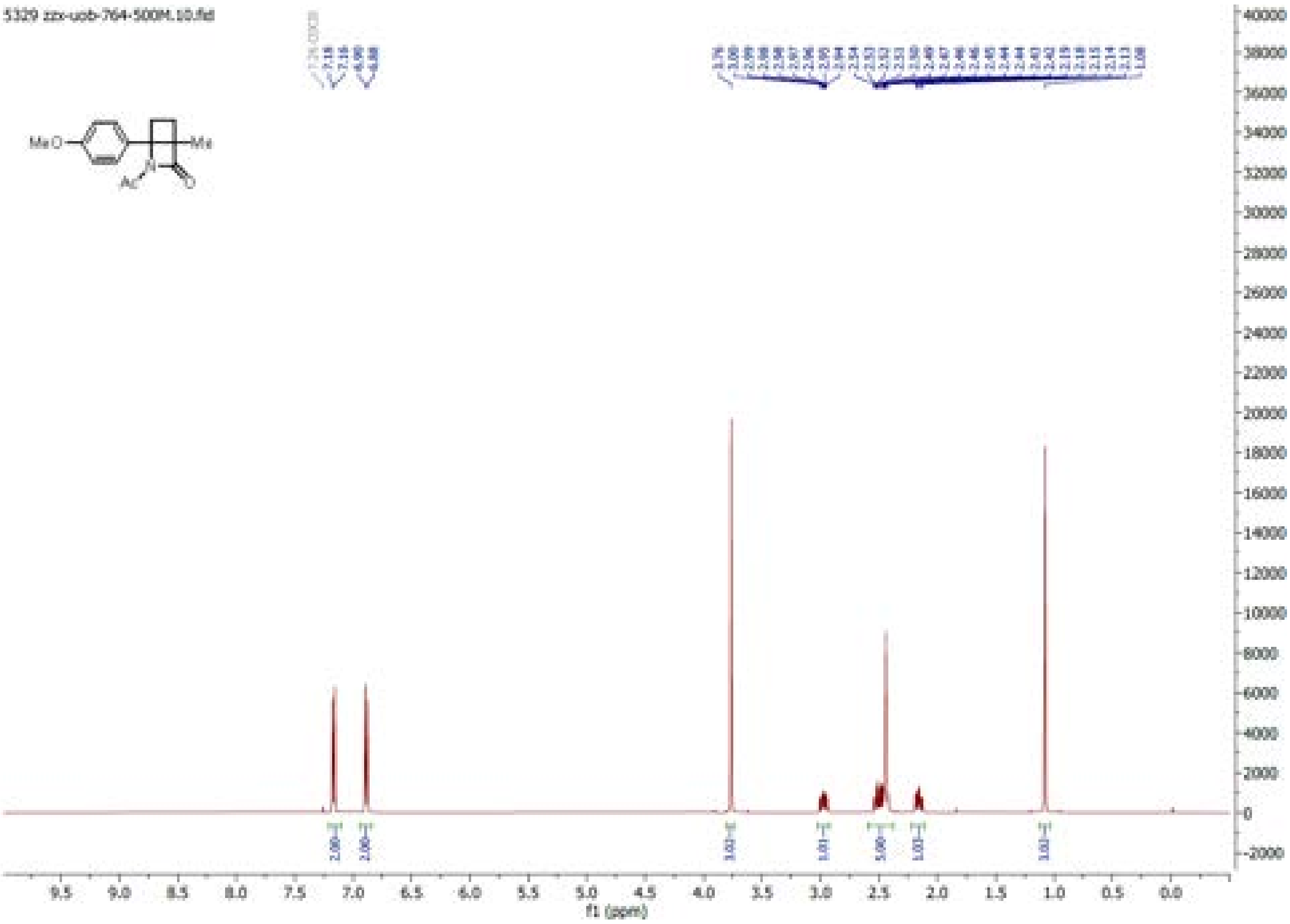

^{13}C NMR (126 MHz, $CDCl_3$) of **6g**

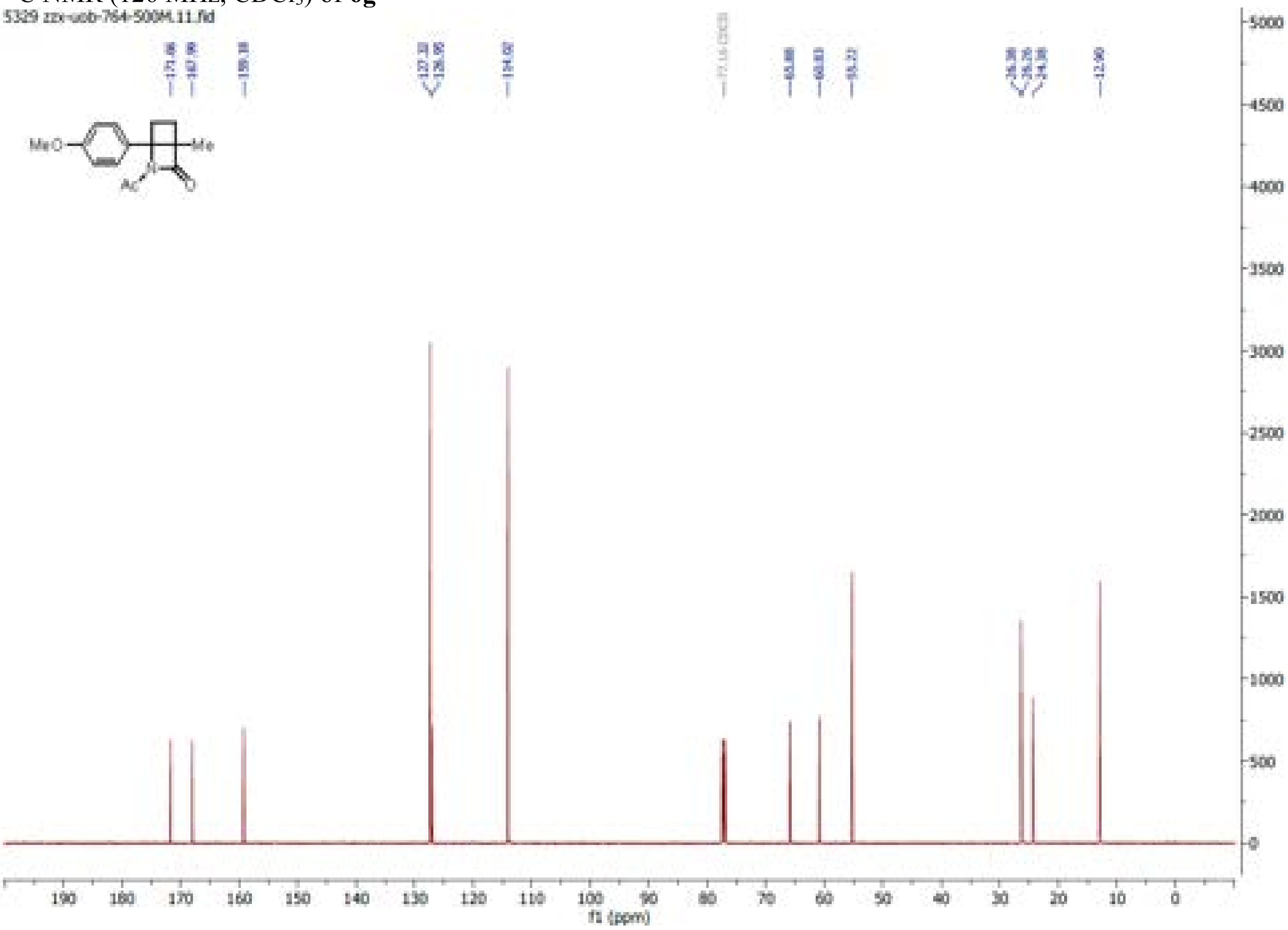

^{1}H NMR (500 MHz, $CDCl_3$) of **6h**

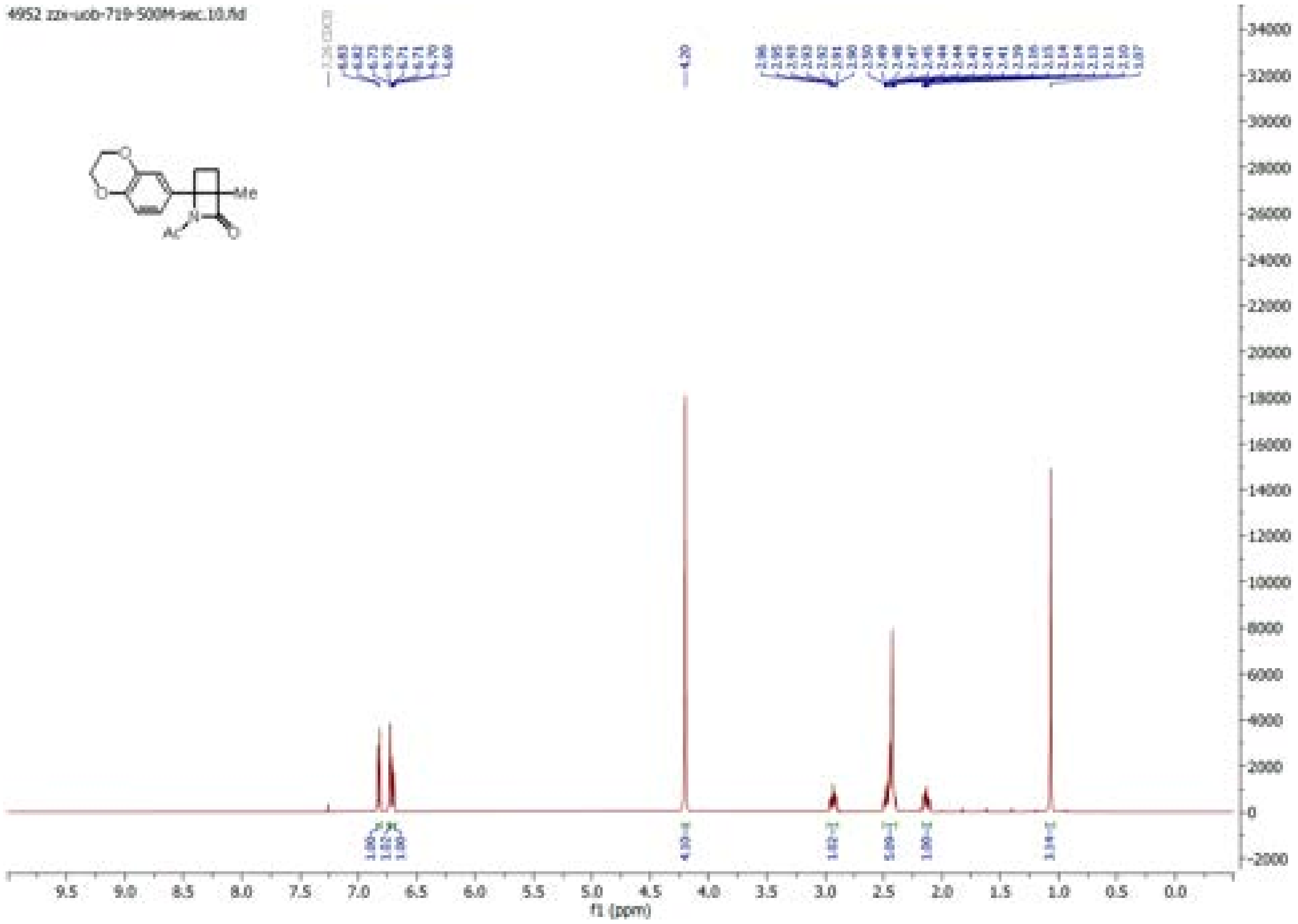

^{13}C NMR (126 MHz, $CDCl_3$) of **6h**

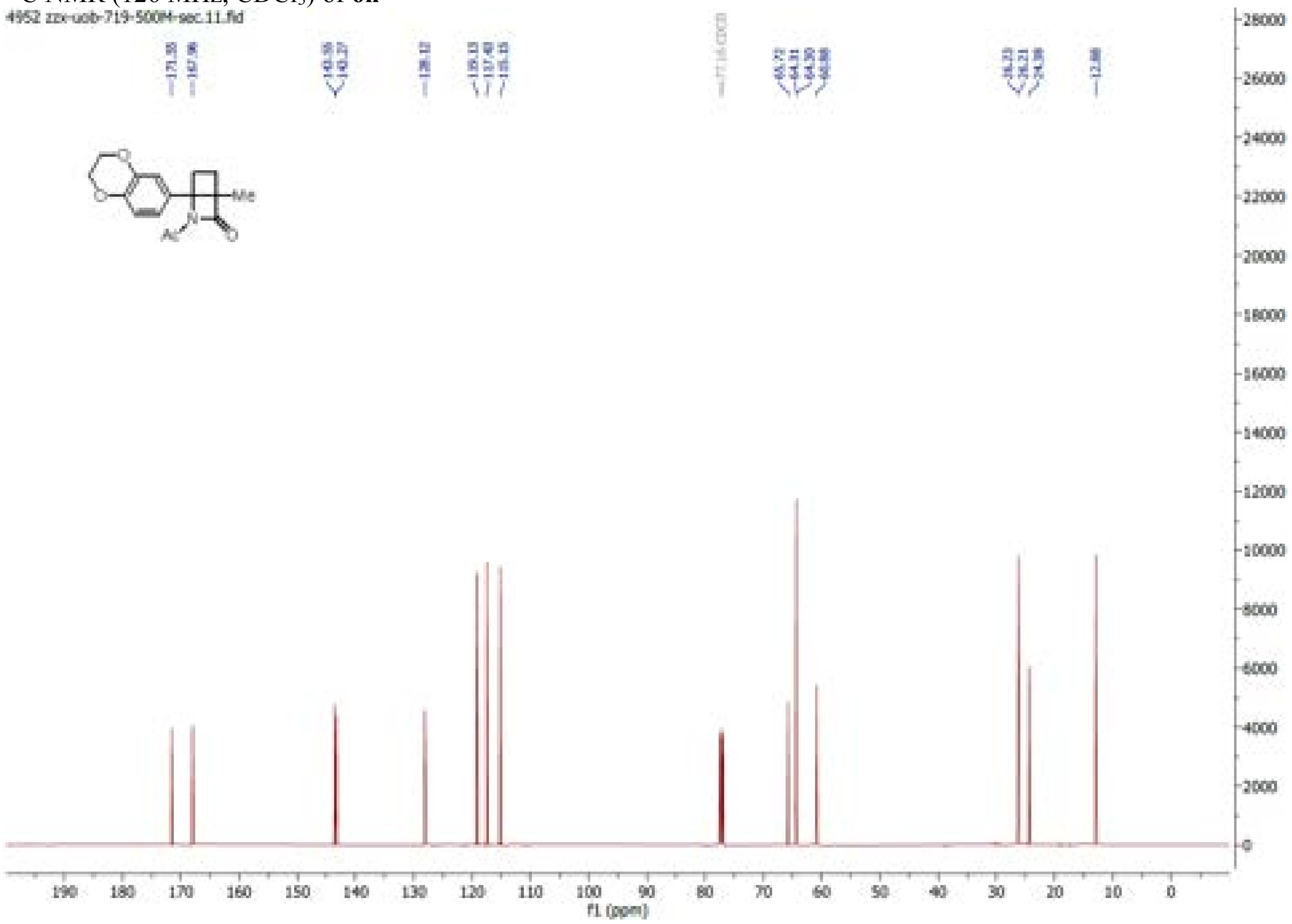

^{1}H NMR (500 MHz, $CDCl_3$) of **6i**

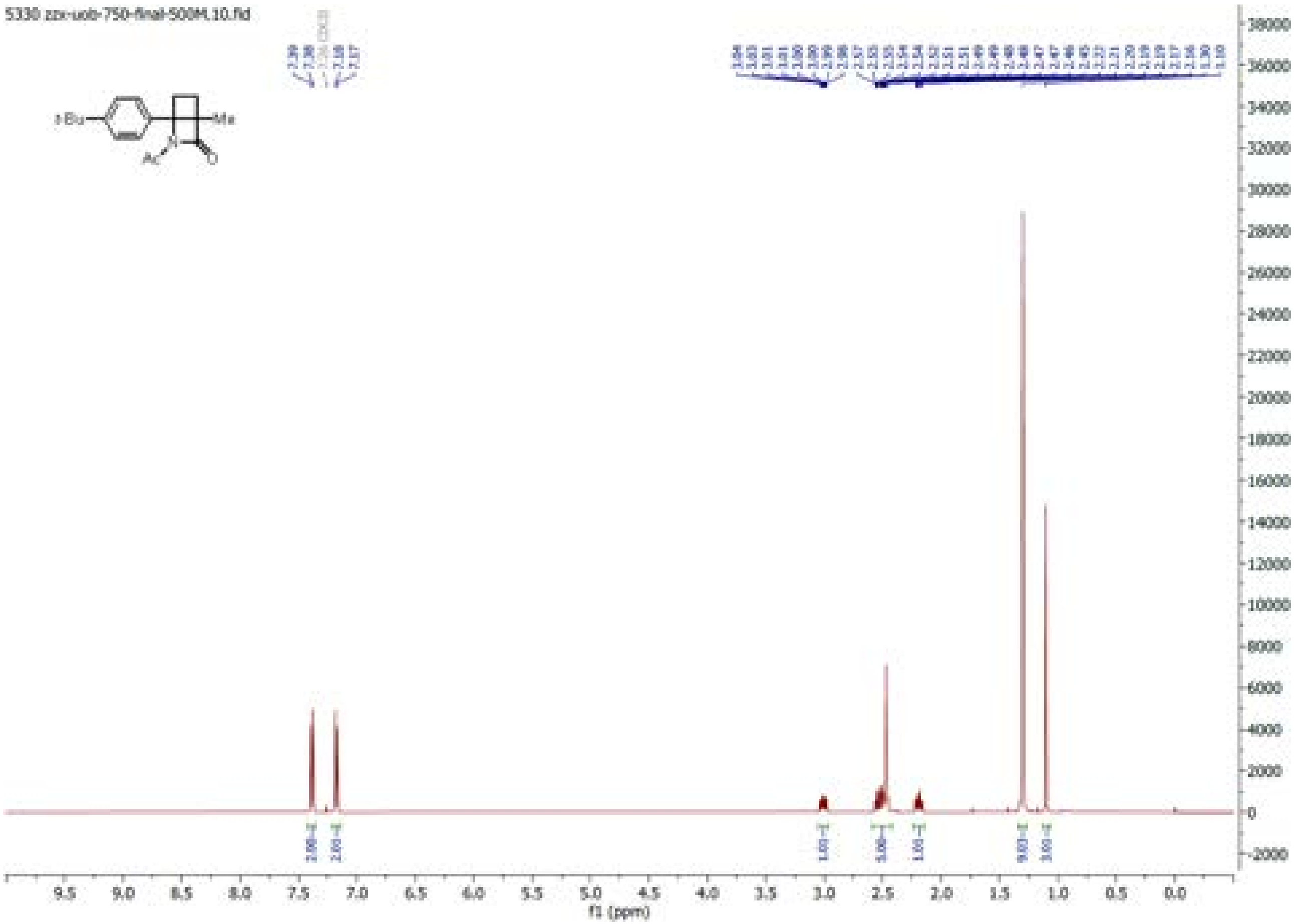

^{13}C NMR (126 MHz, $CDCl_3$) of **6i**

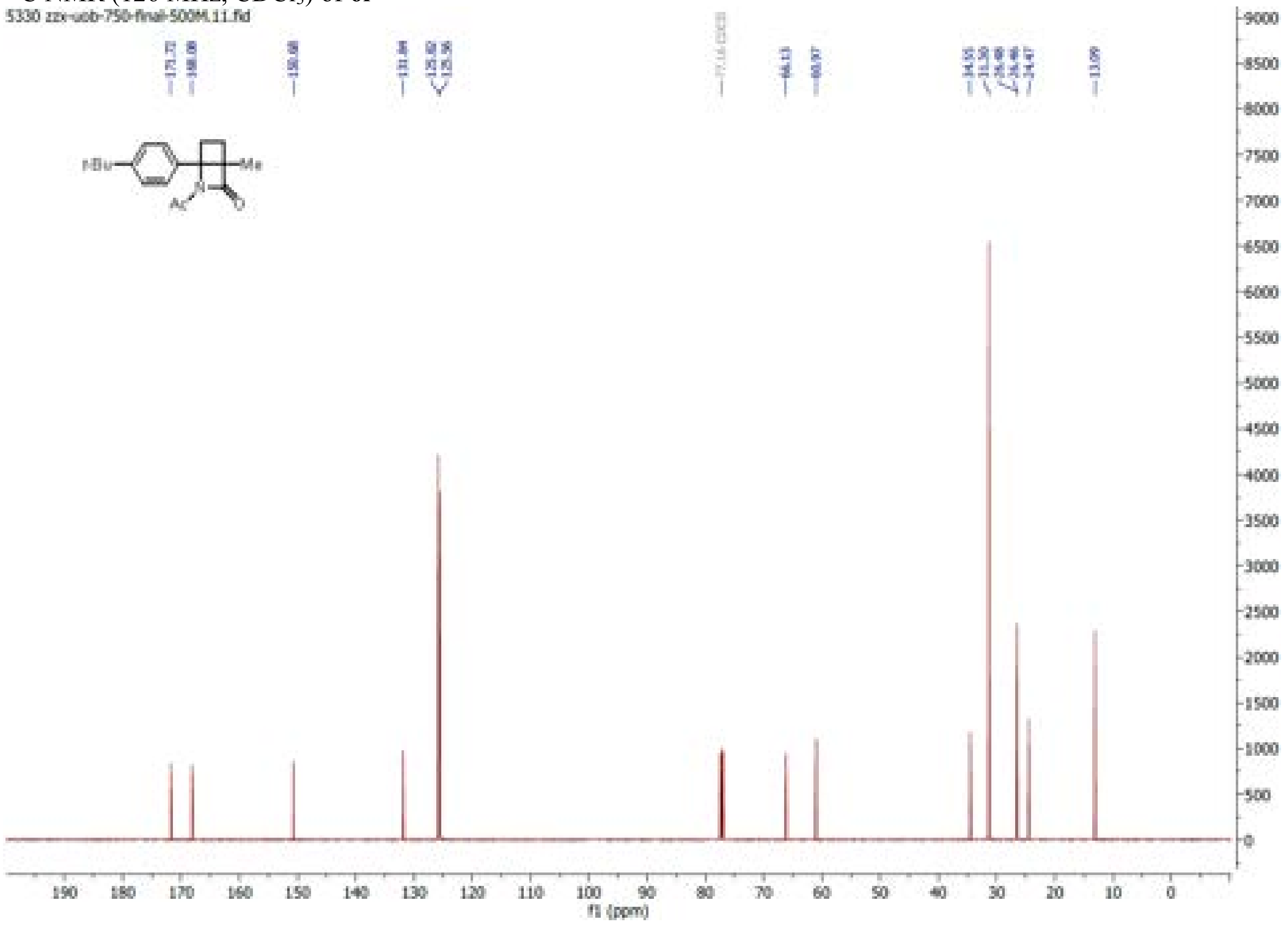

^{1}H NMR (500 MHz, $CDCl_3$) of **6j**

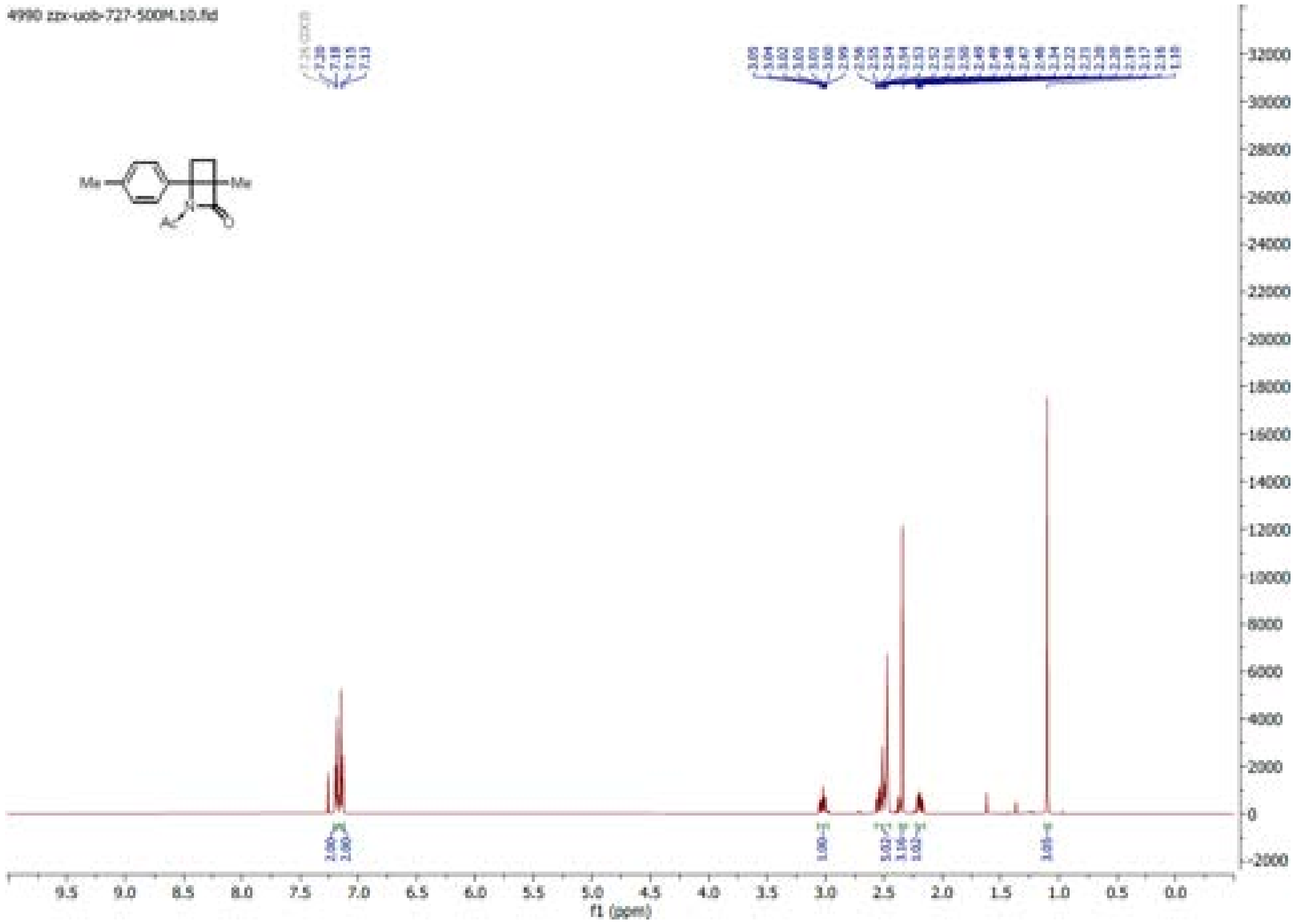

^{13}C NMR (126 MHz, $CDCl_3$) of **6j**

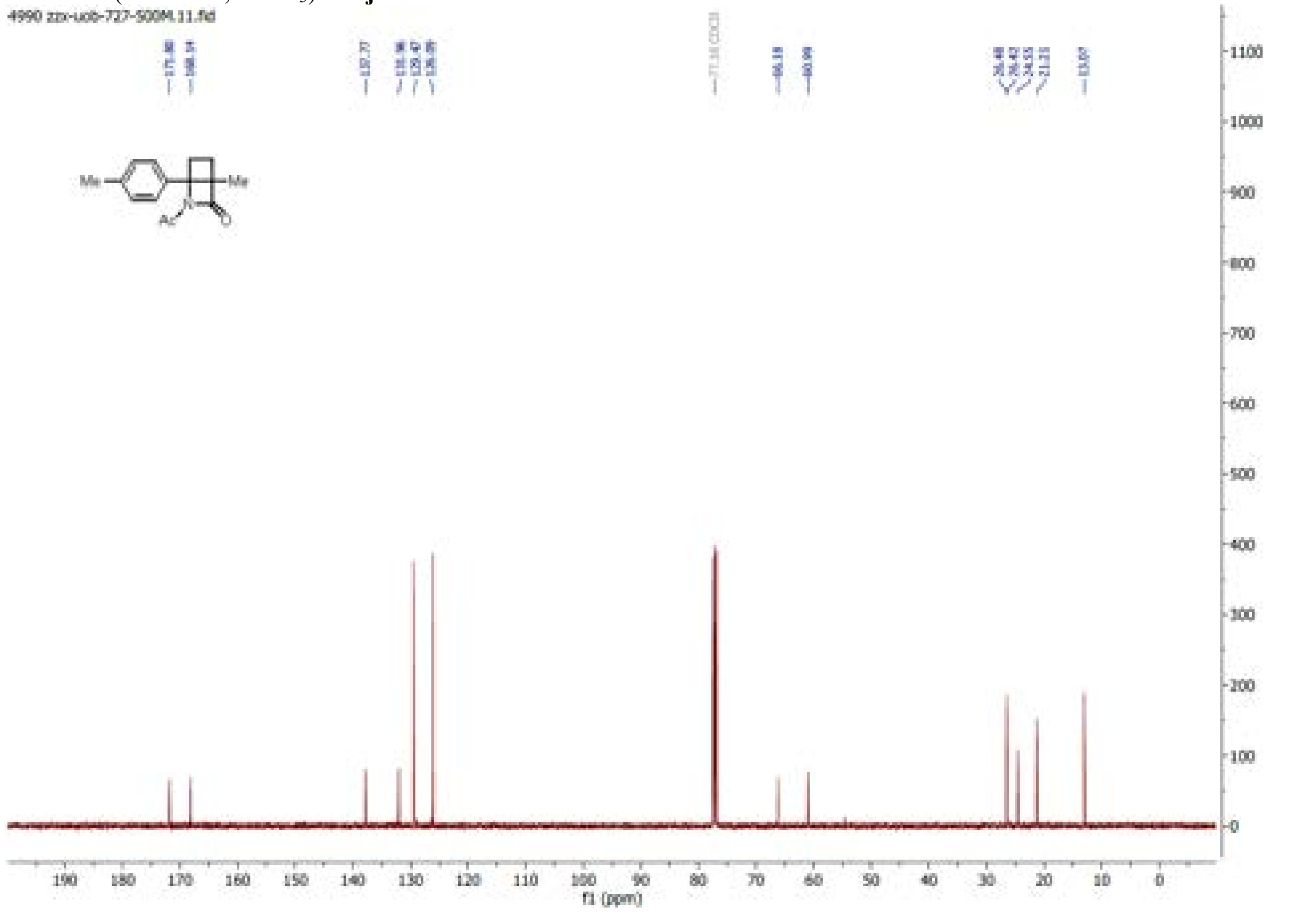

^{1}H NMR (500 MHz, $CDCl_3$) of **6k**

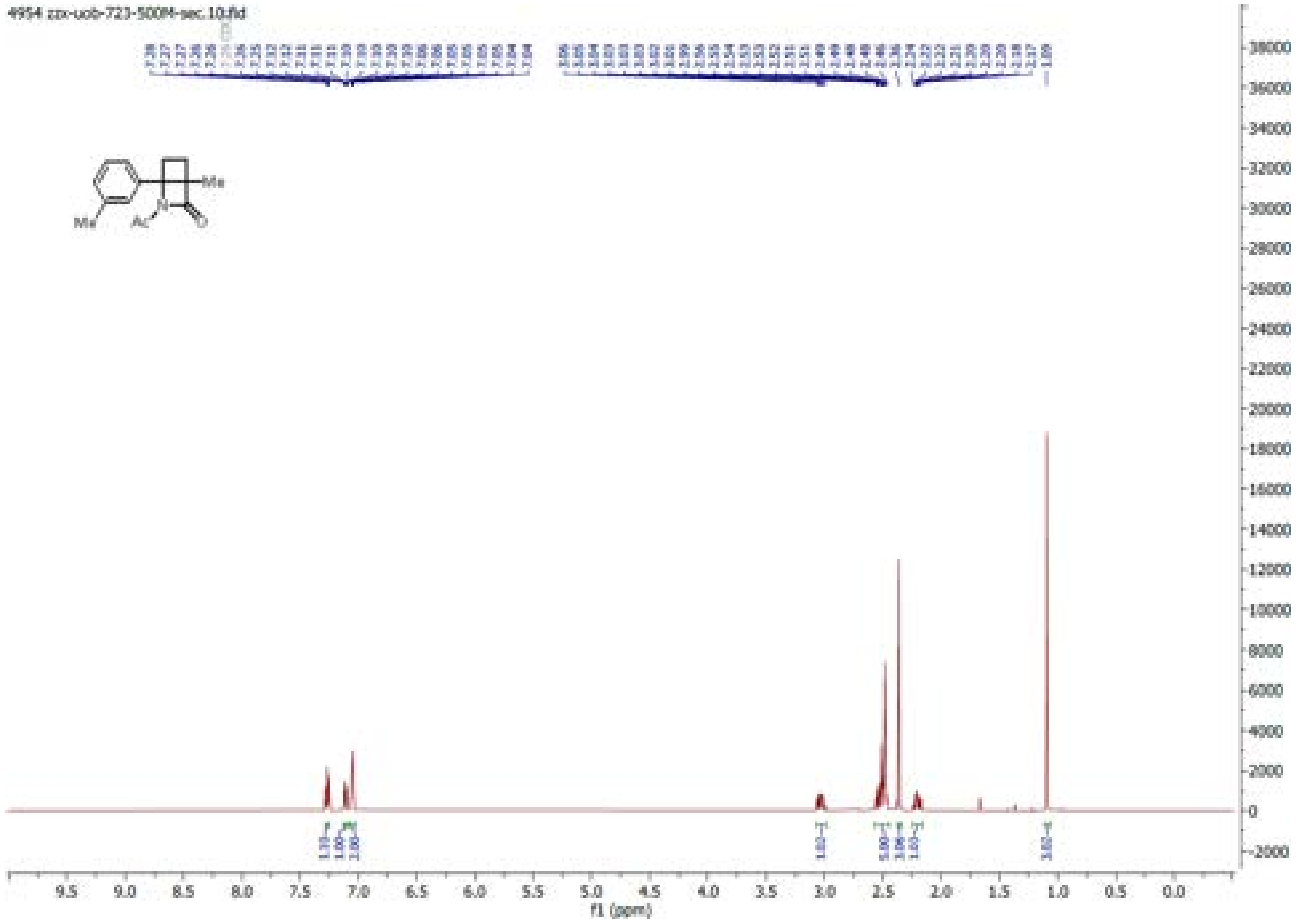

^{13}C NMR (126 MHz, $CDCl_3$) of **6k**

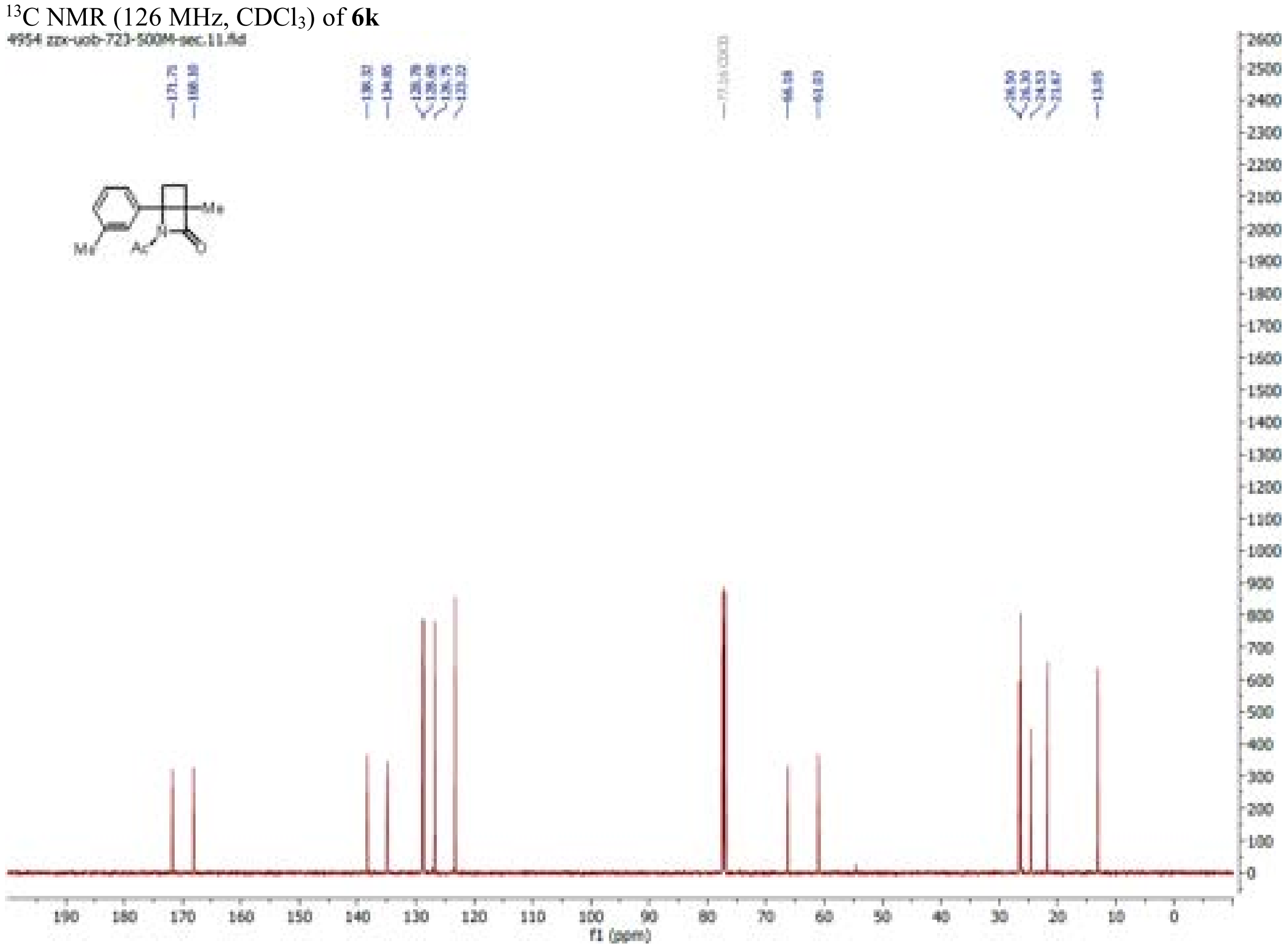

^{1}H NMR (500 MHz, $CDCl_3$) of **6l**

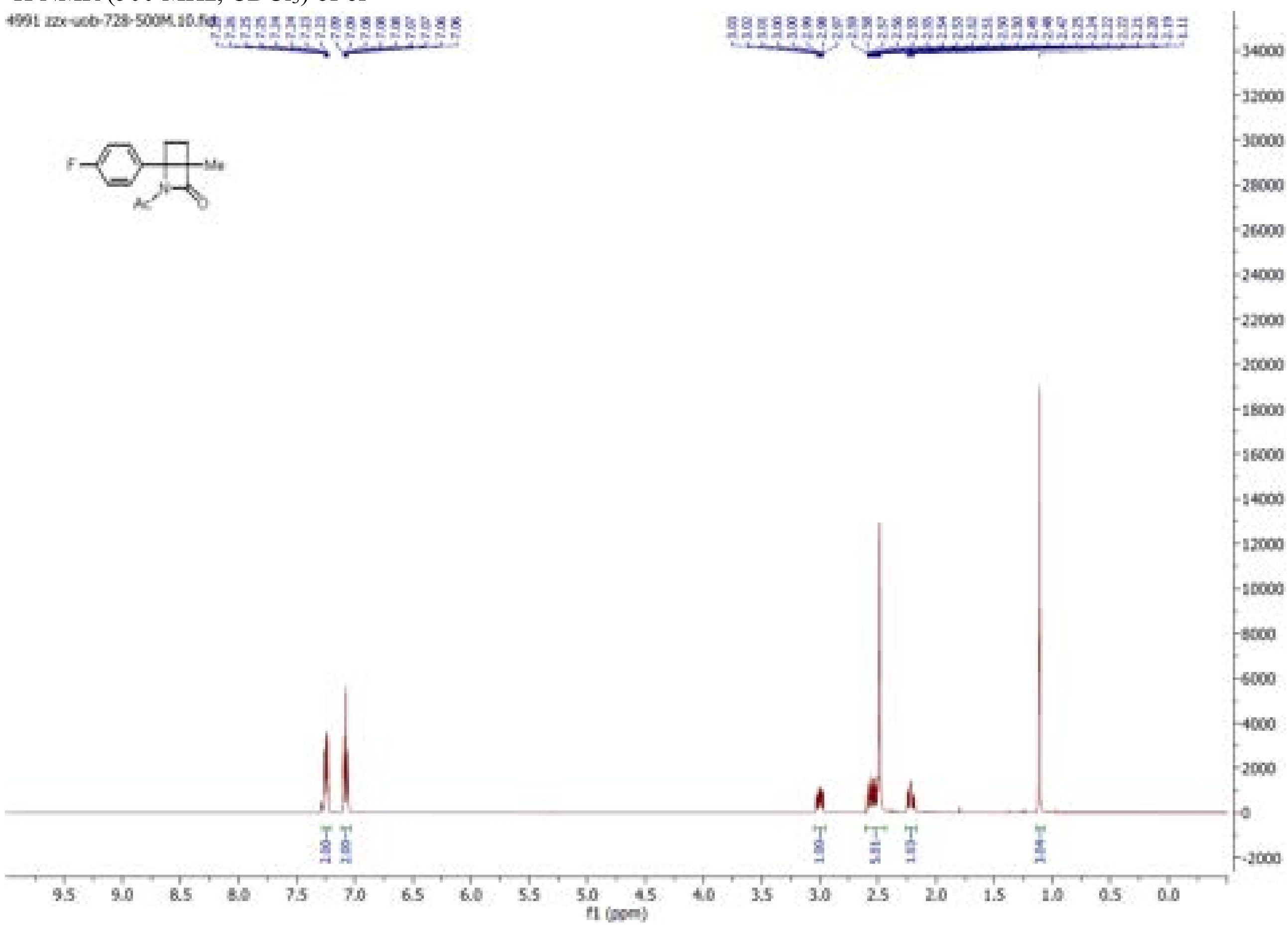

^{13}C NMR (126 MHz, $CDCl_3$) of **6l**

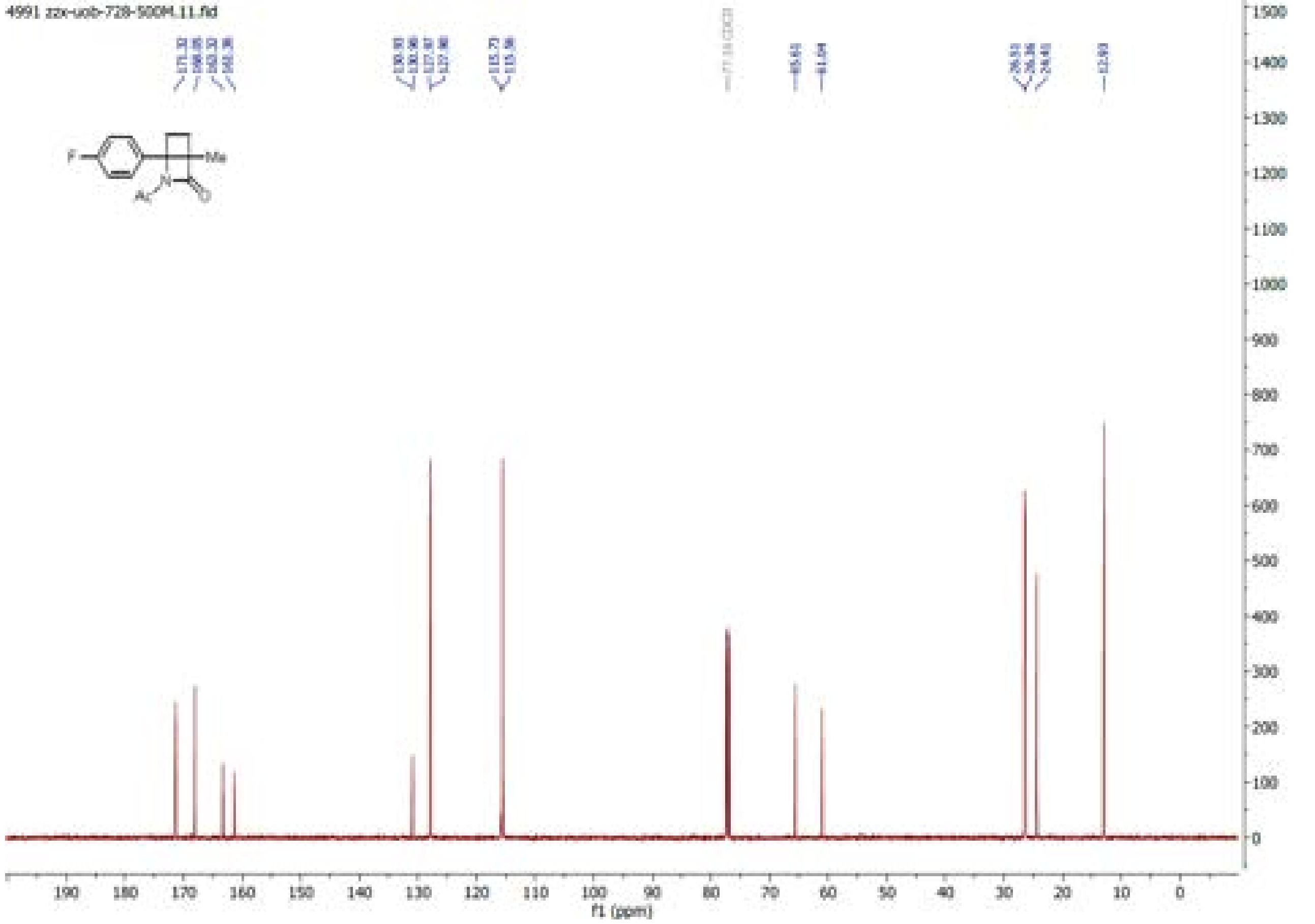

^{19}F NMR (471 MHz, $CDCl_3$) of **6l**

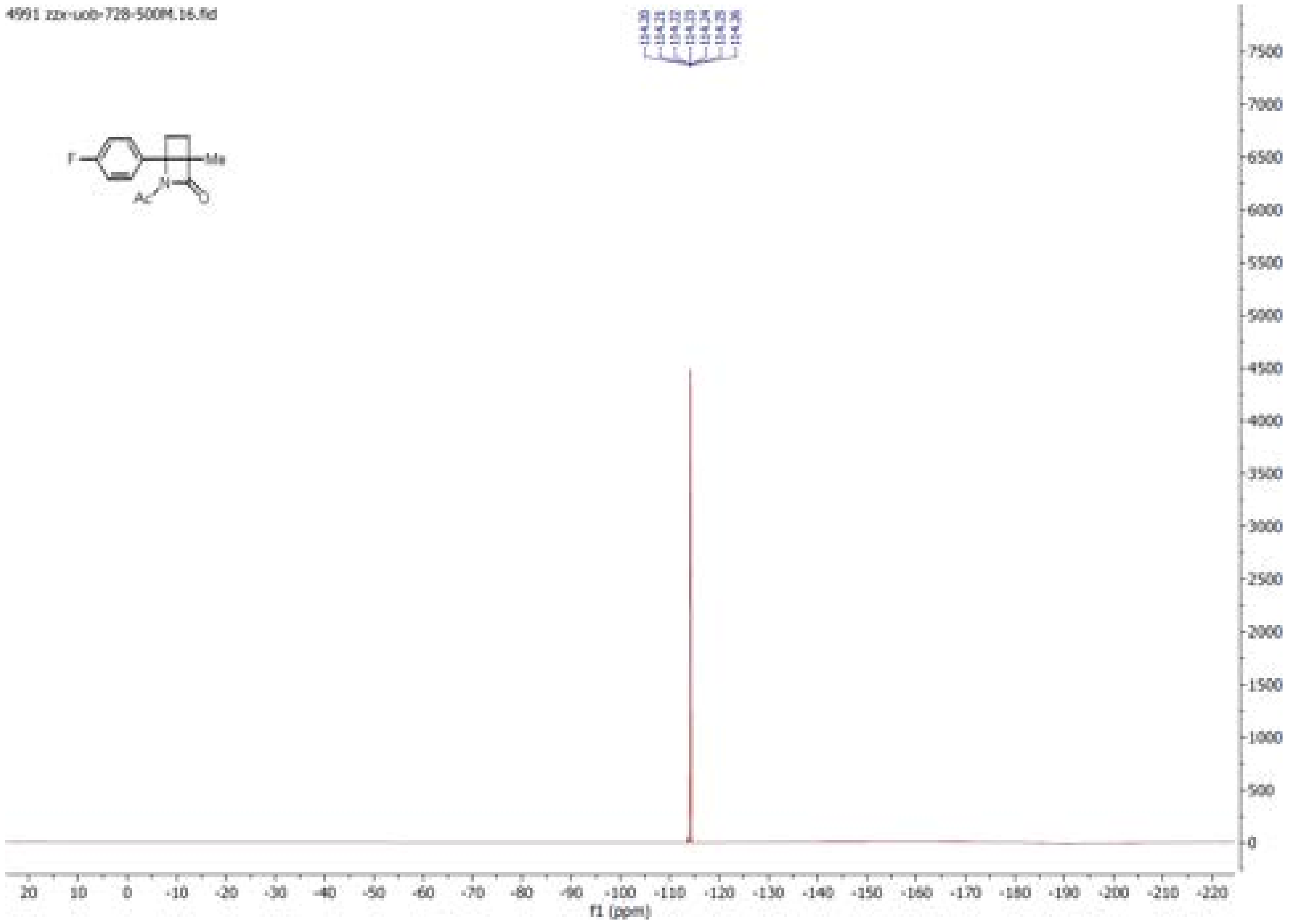

^{1}H NMR (500 MHz, $CDCl_3$) of **6m**

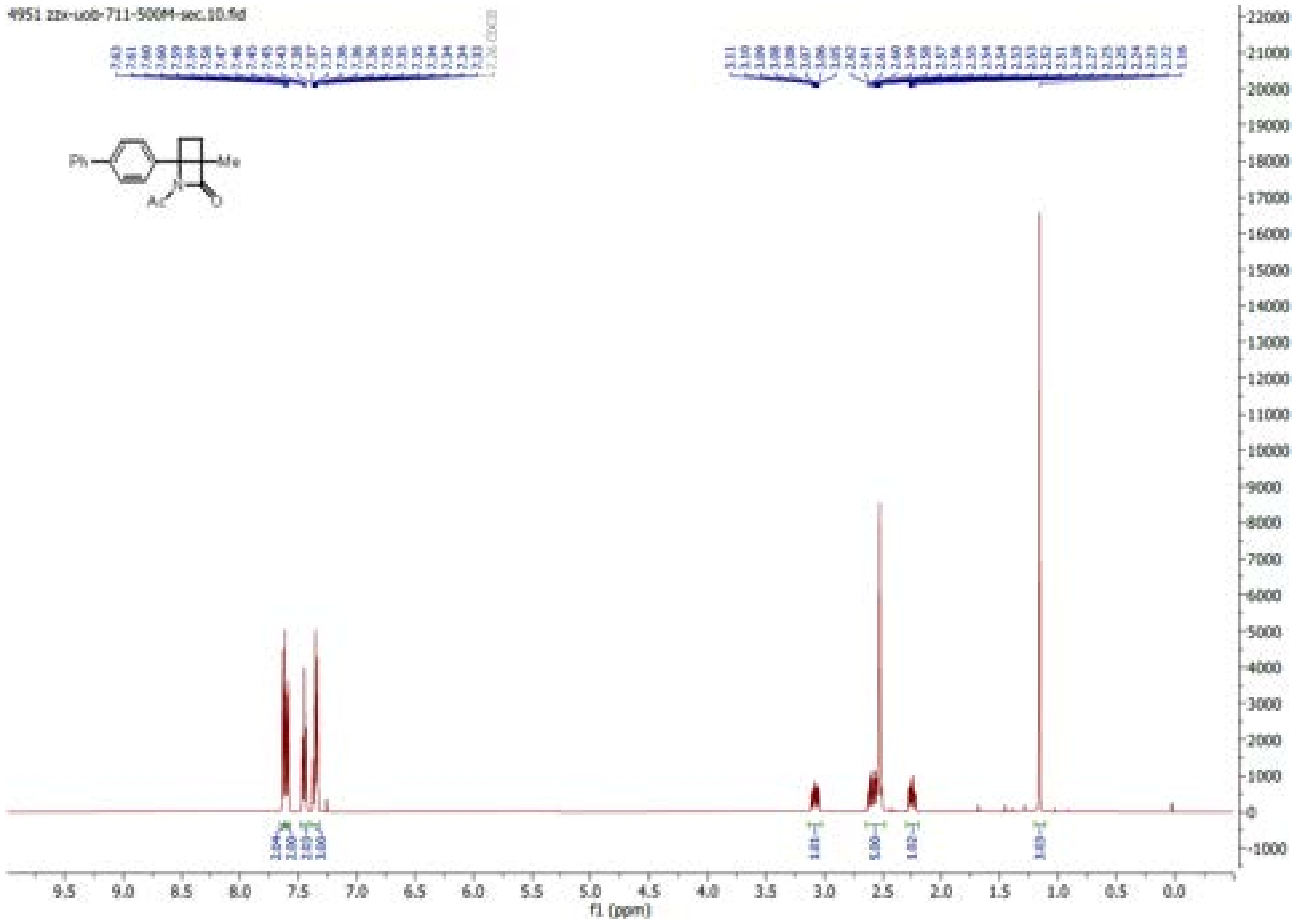

^{13}C NMR (126 MHz, $CDCl_3$) of **6m**

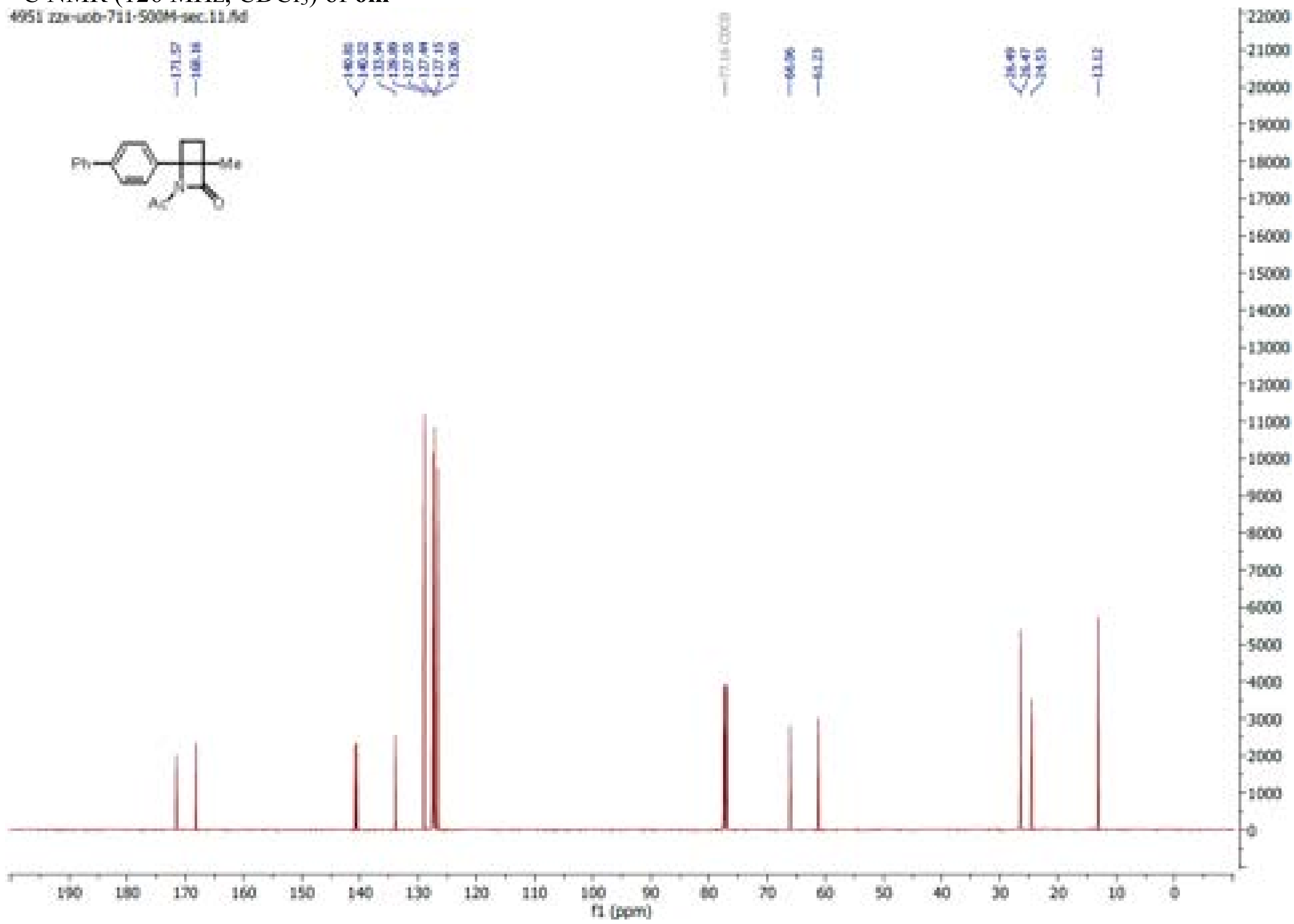

^{1}H NMR (500 MHz, $CDCl_3$) of **6n**

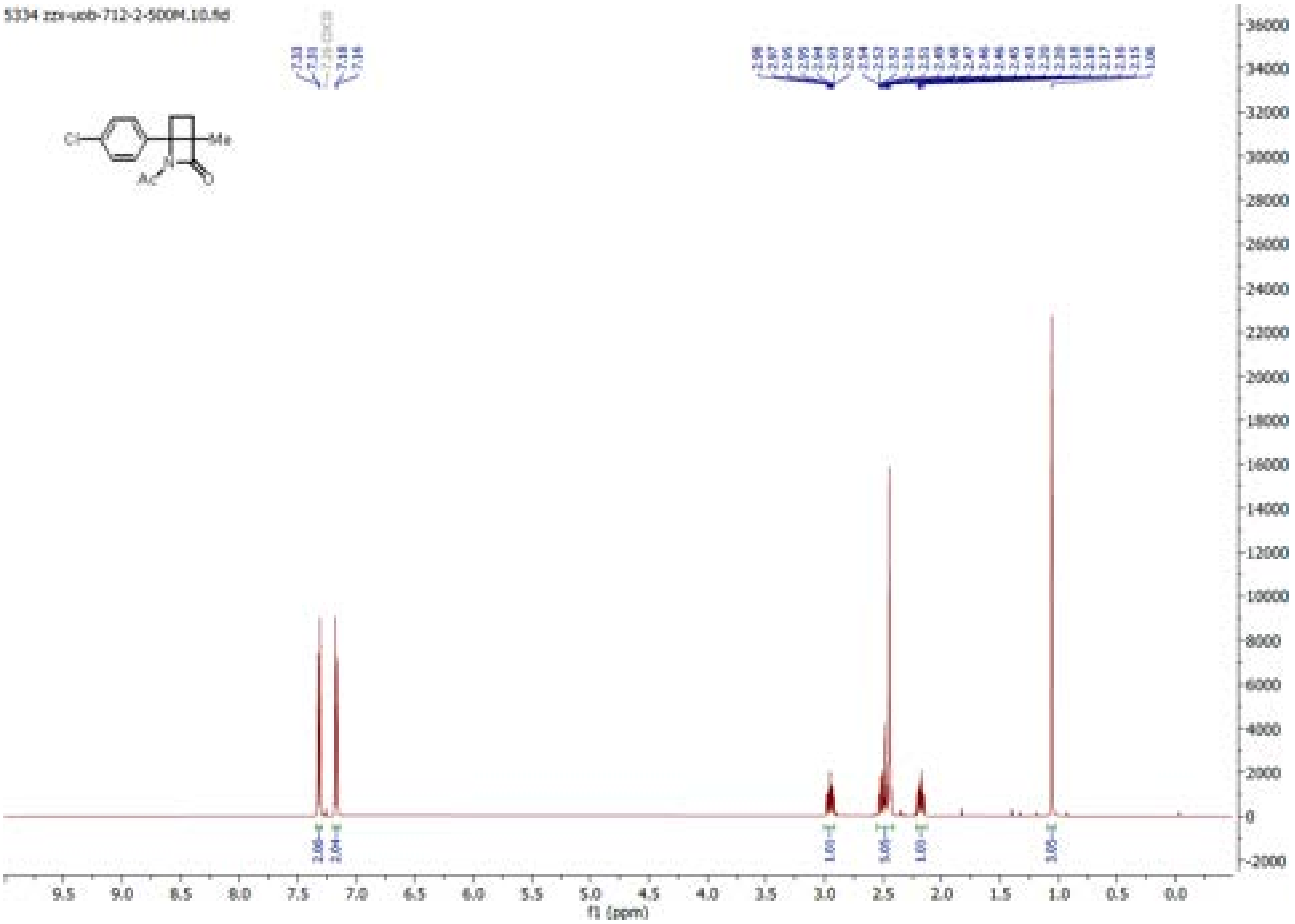

^{13}C NMR (126 MHz, $CDCl_3$) of **6n**

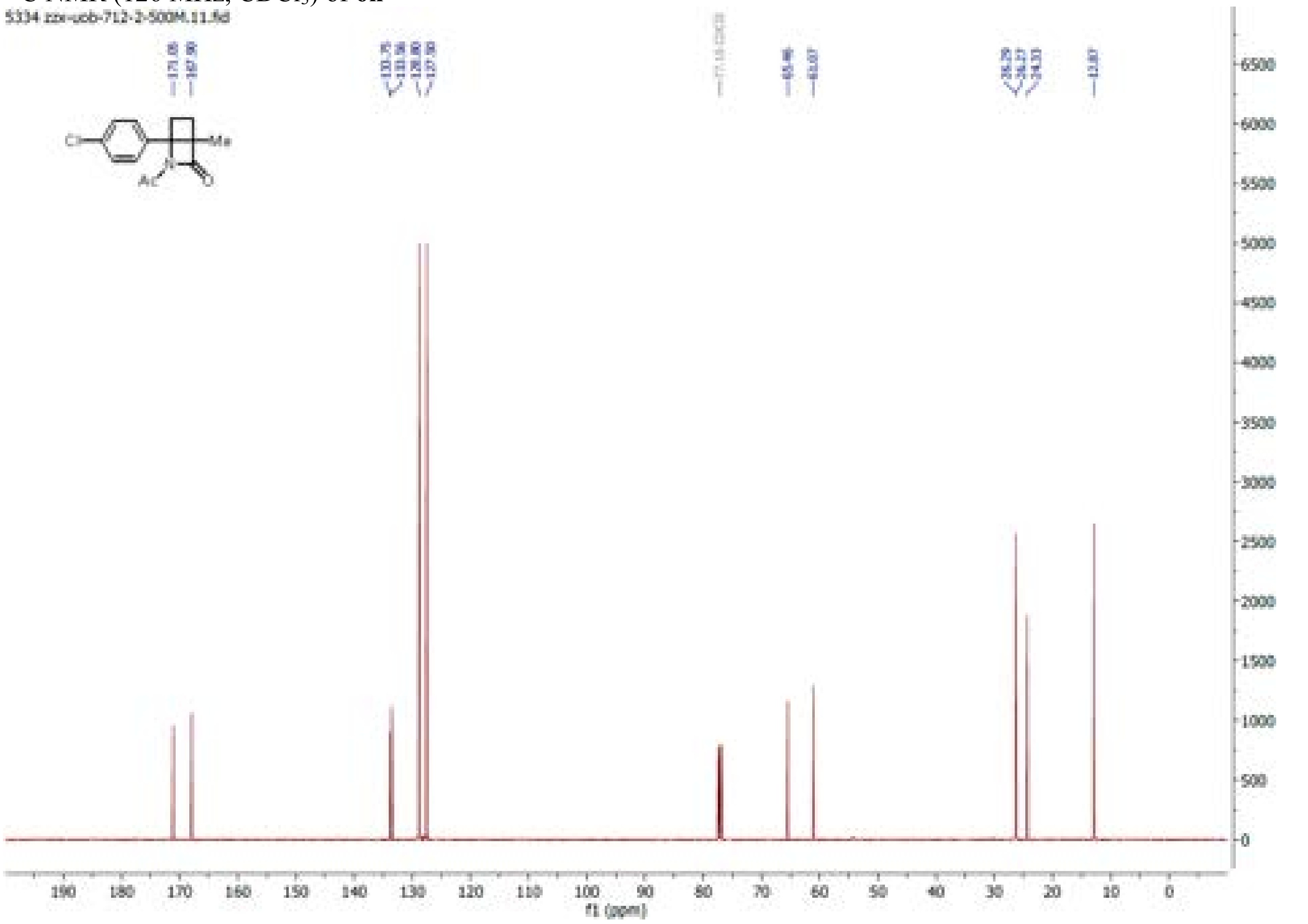

^{1}H NMR (500 MHz, $CDCl_3$) of **6o**

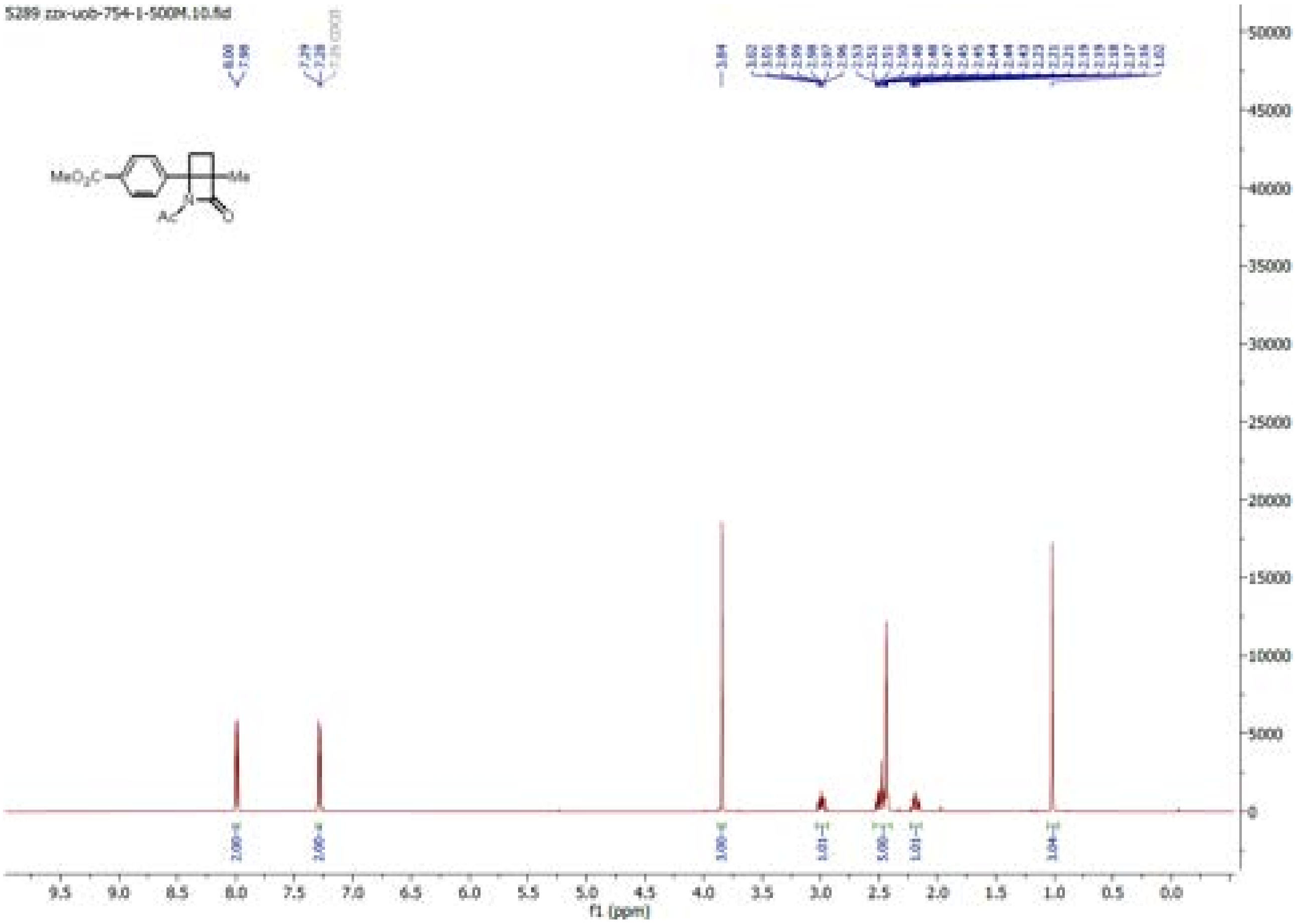

^{13}C NMR (126 MHz, $CDCl_3$) of **6o**

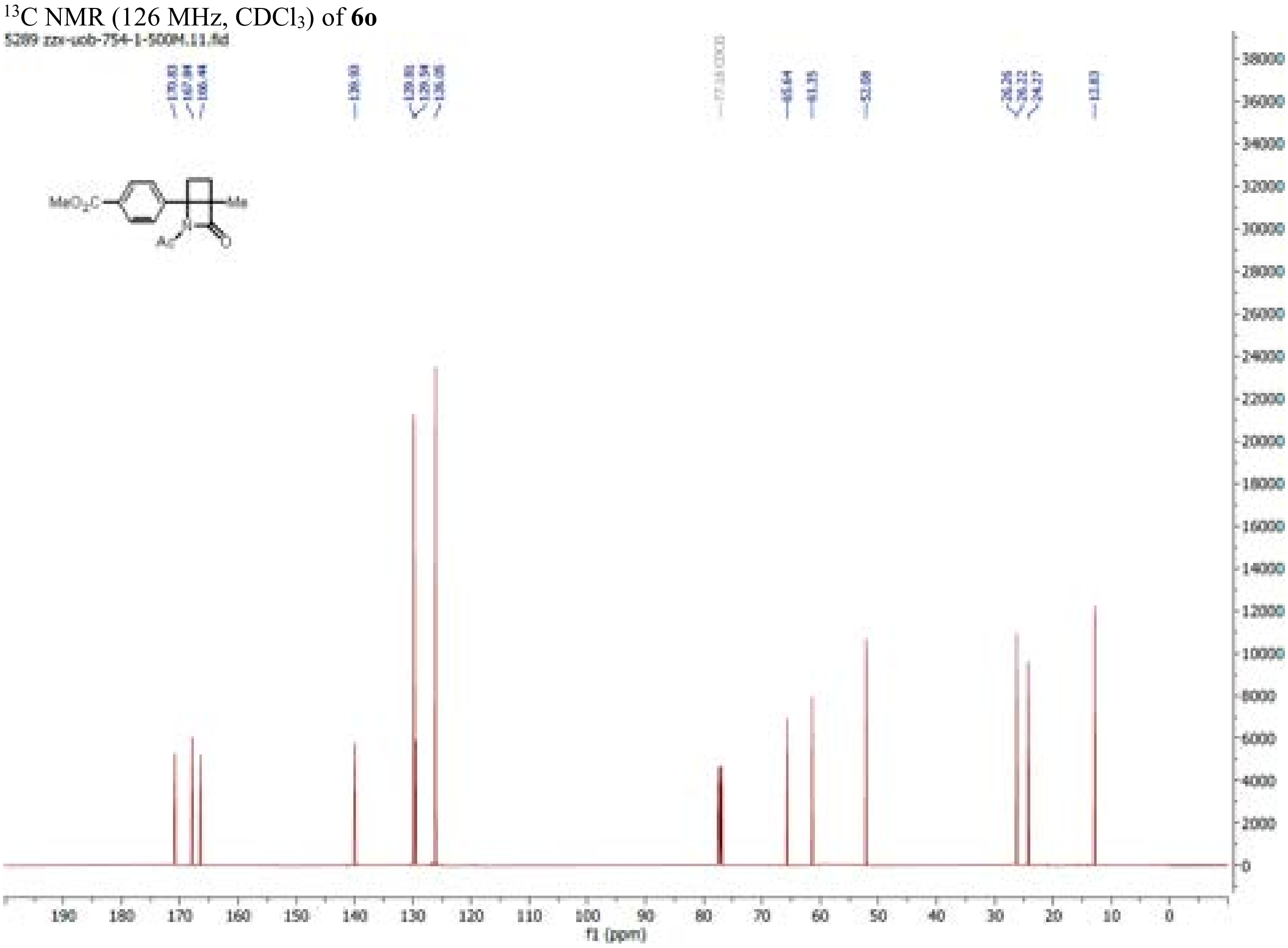

^{1}H NMR (500 MHz, $CDCl_3$) of **5o**

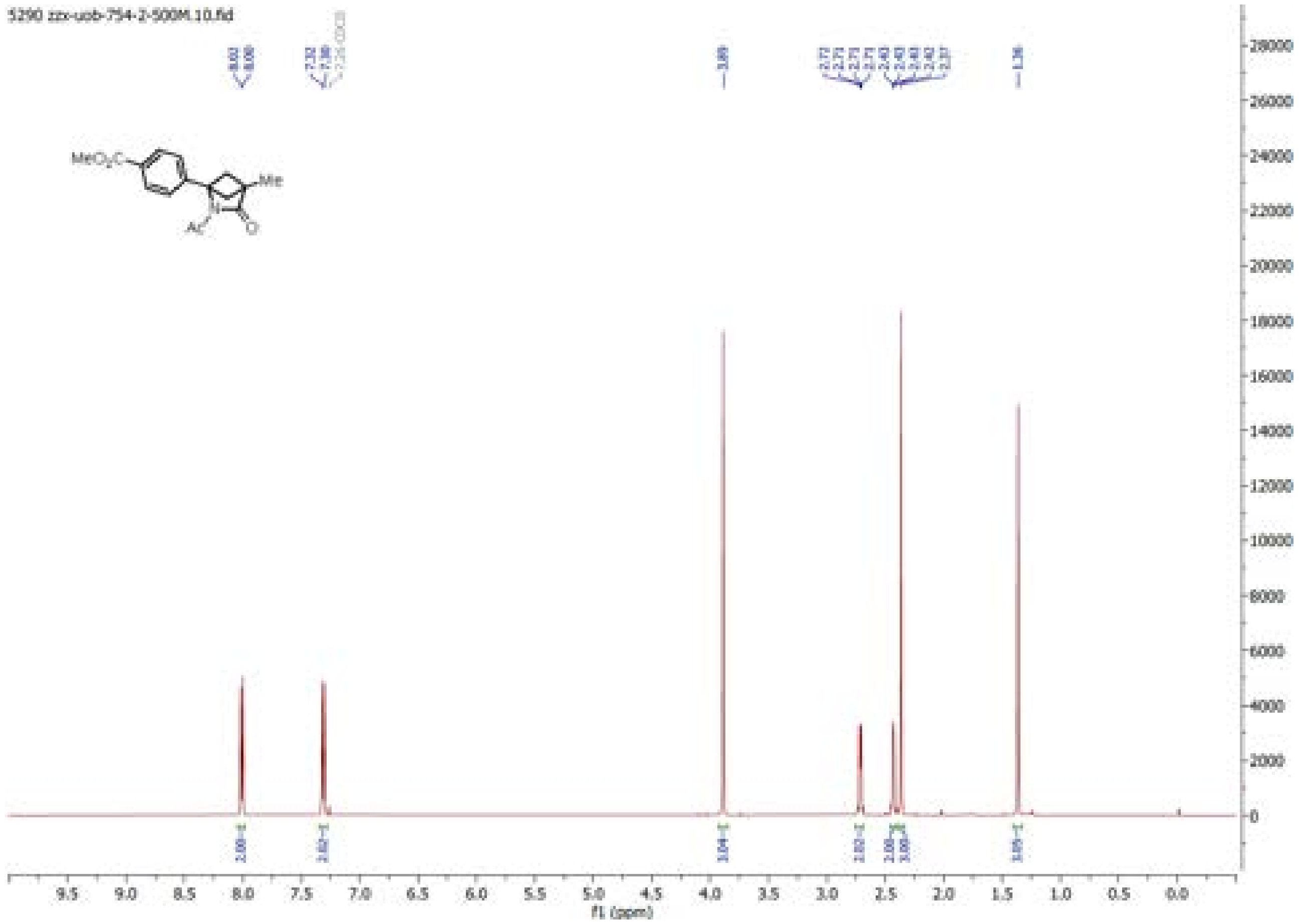

^{13}C NMR (126 MHz, $CDCl_3$) of **5o**

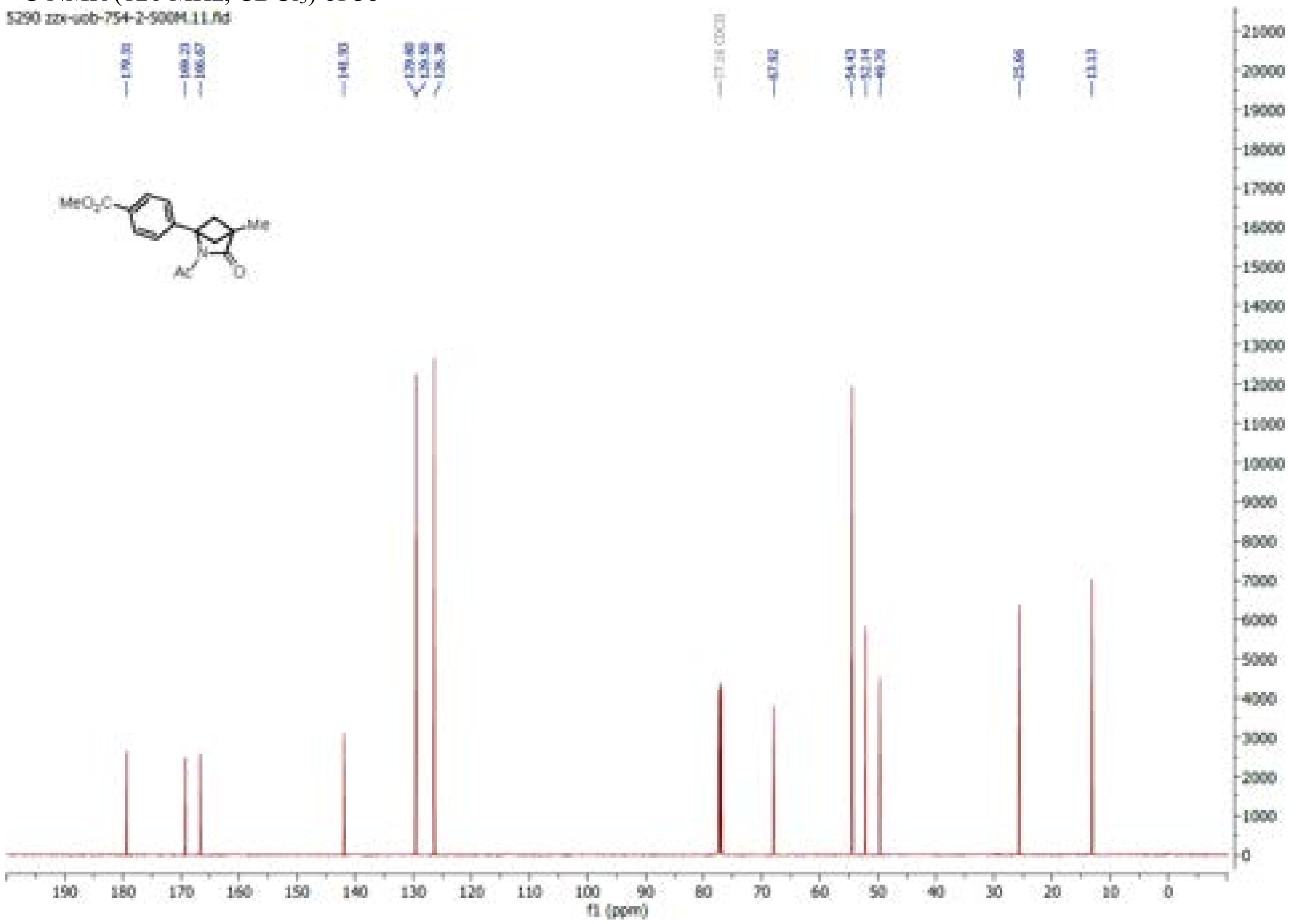

^{1}H NMR (500 MHz, $CDCl_3$) of **6p**

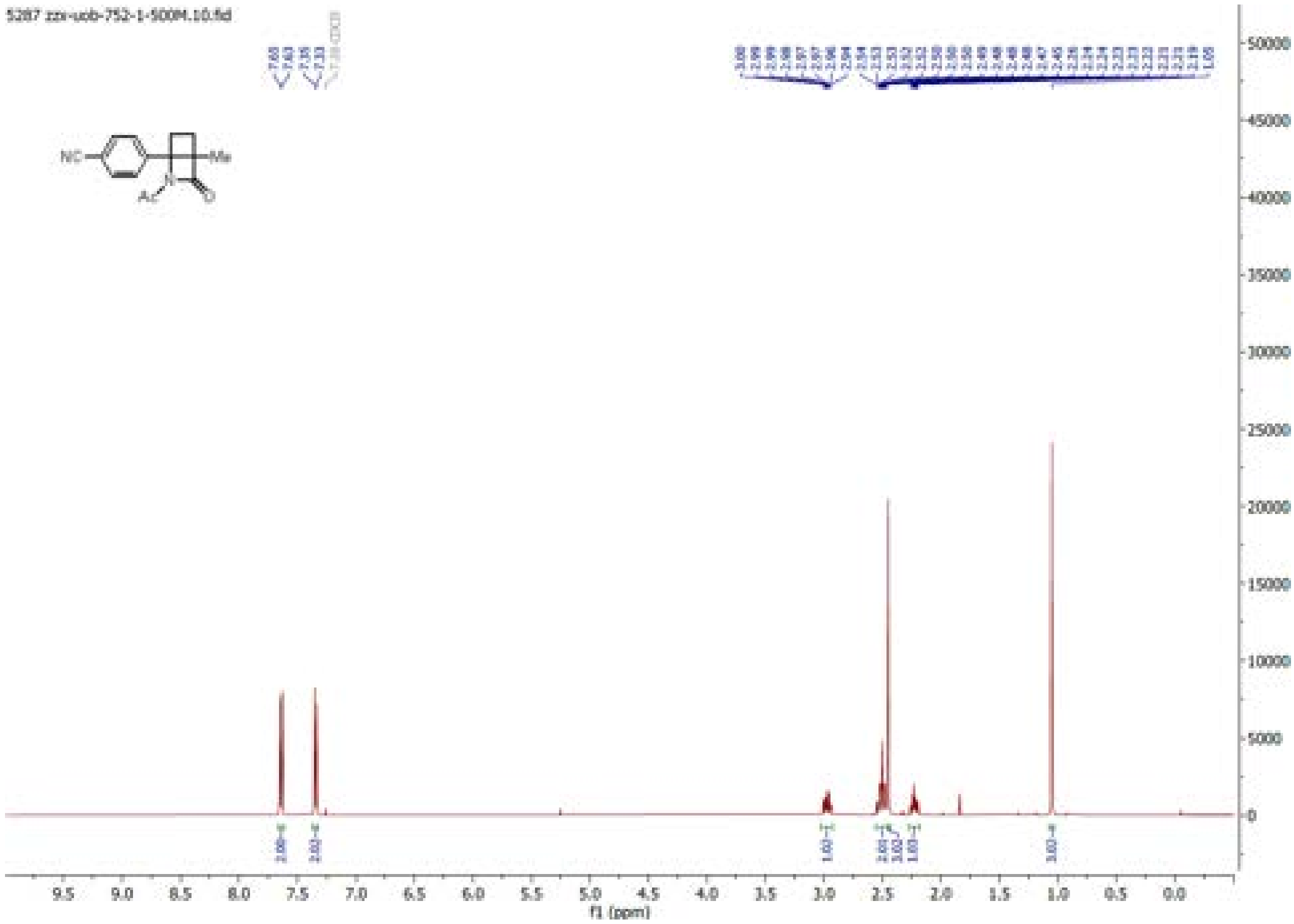

^{13}C NMR (126 MHz, $CDCl_3$) of **6p**

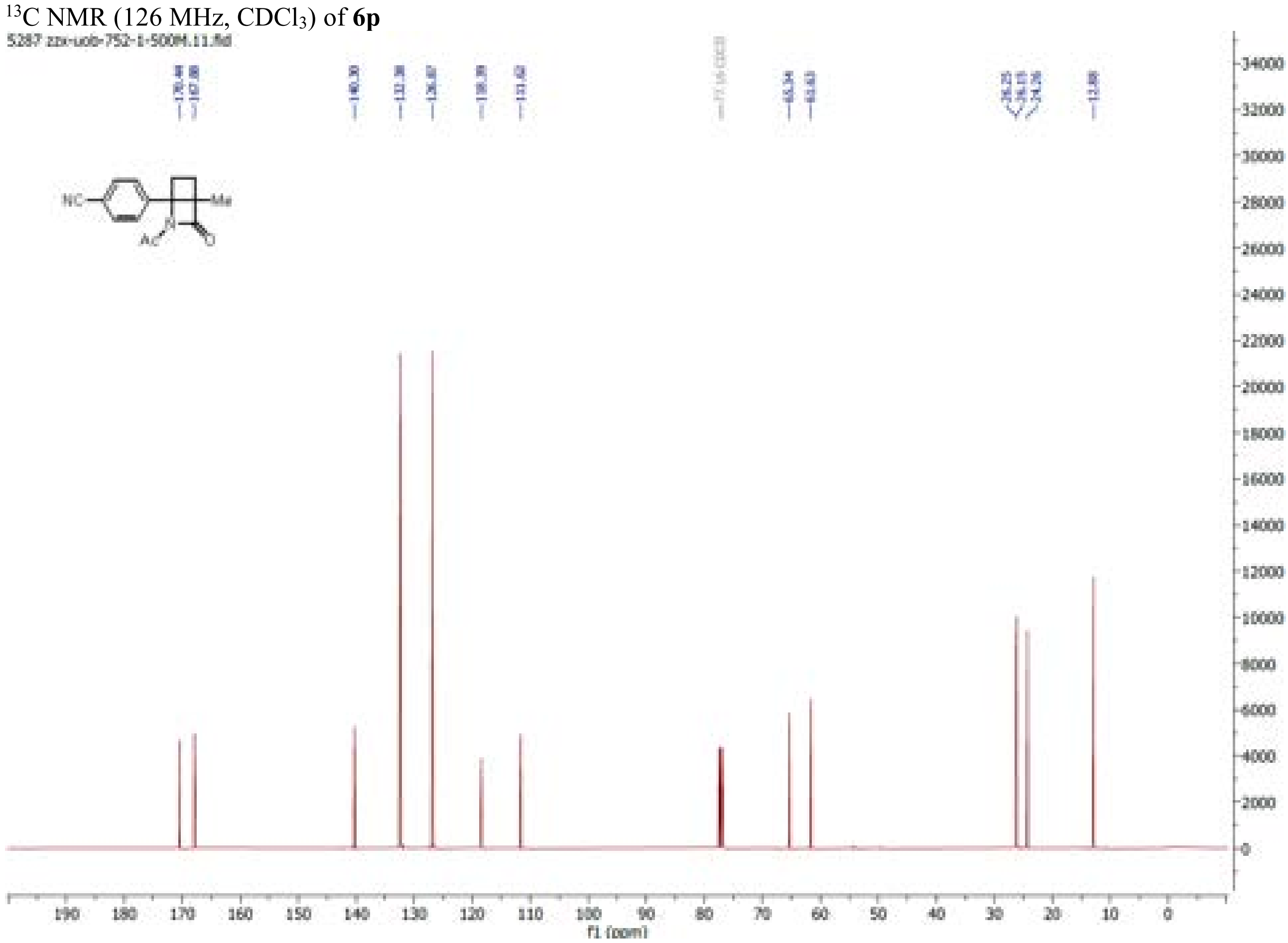

^{1}H NMR (500 MHz, $CDCl_3$) of **5p**

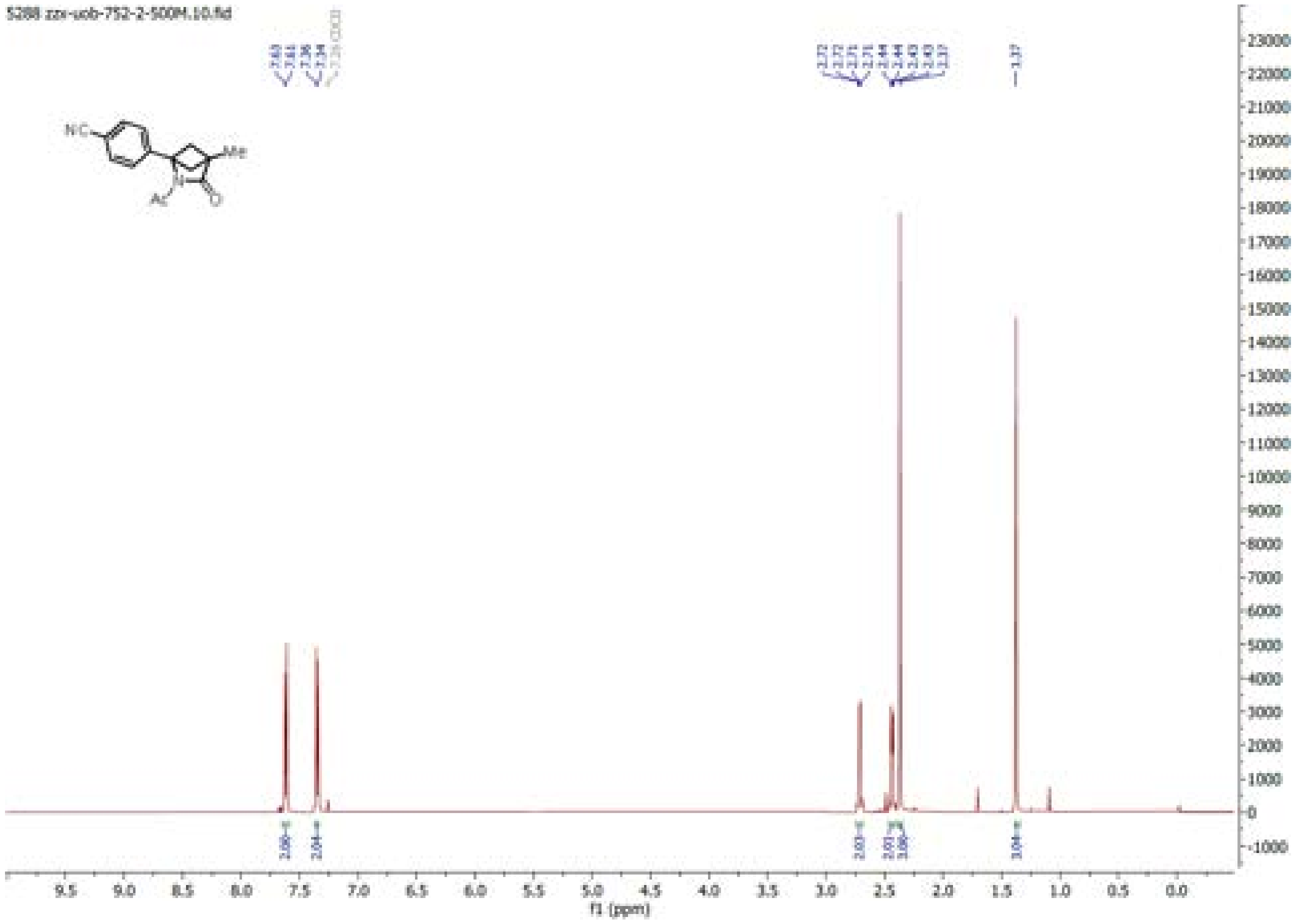

^{13}C NMR (126 MHz, $CDCl_3$) of **5p**

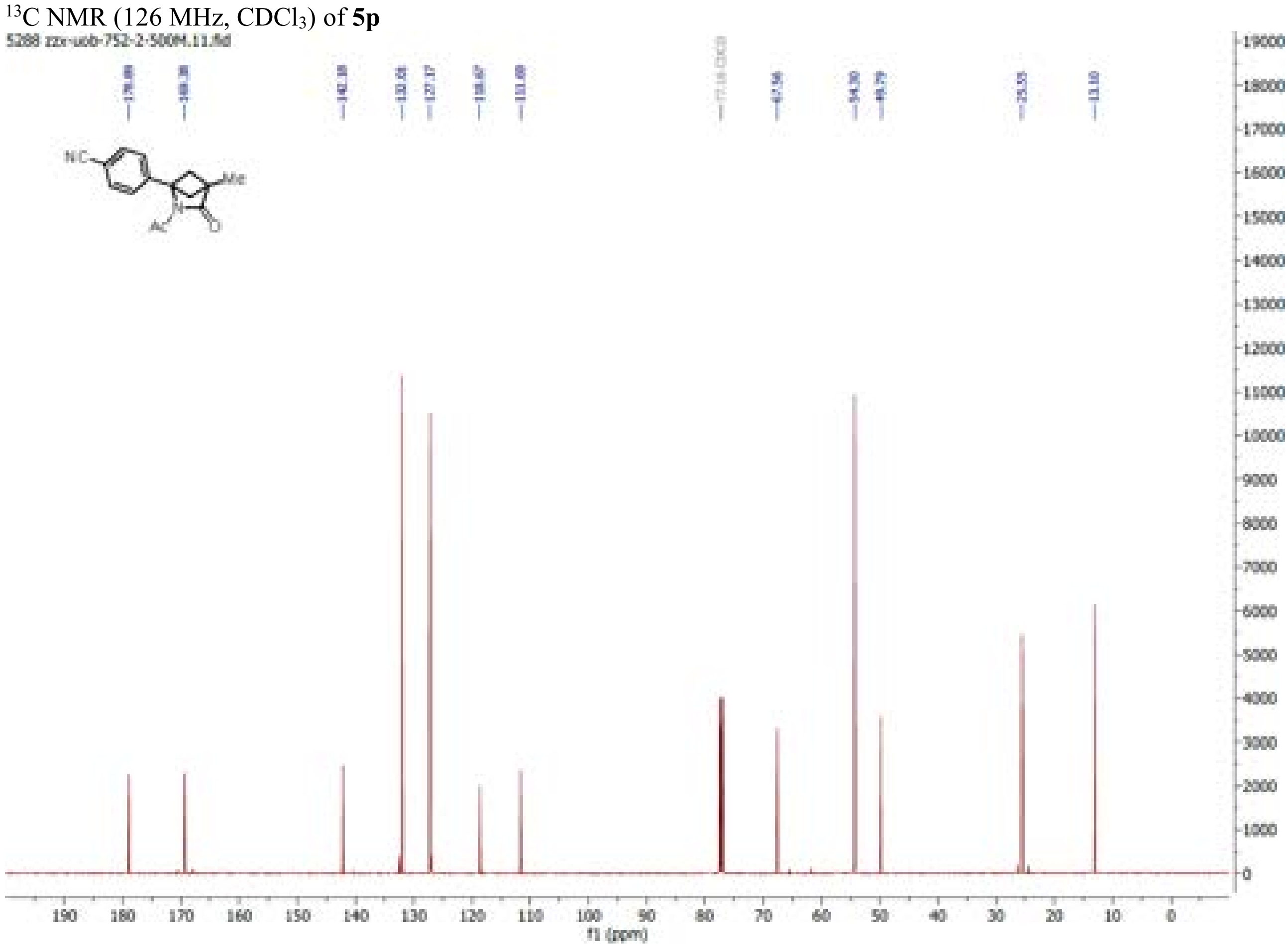

^{1}H NMR (500 MHz, $CDCl_3$) of **6q**

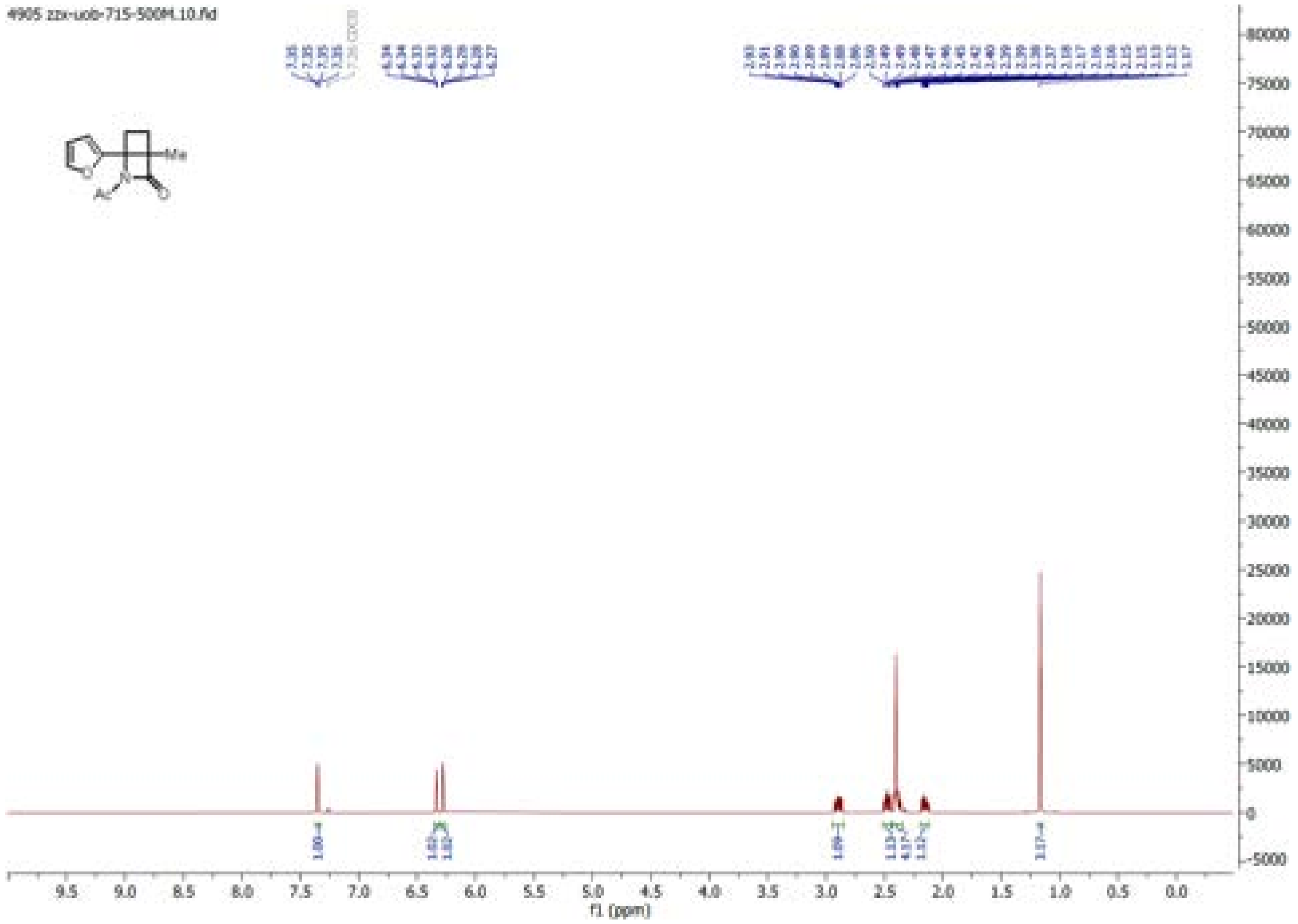

^{13}C NMR (126 MHz, $CDCl_3$) of **6q**

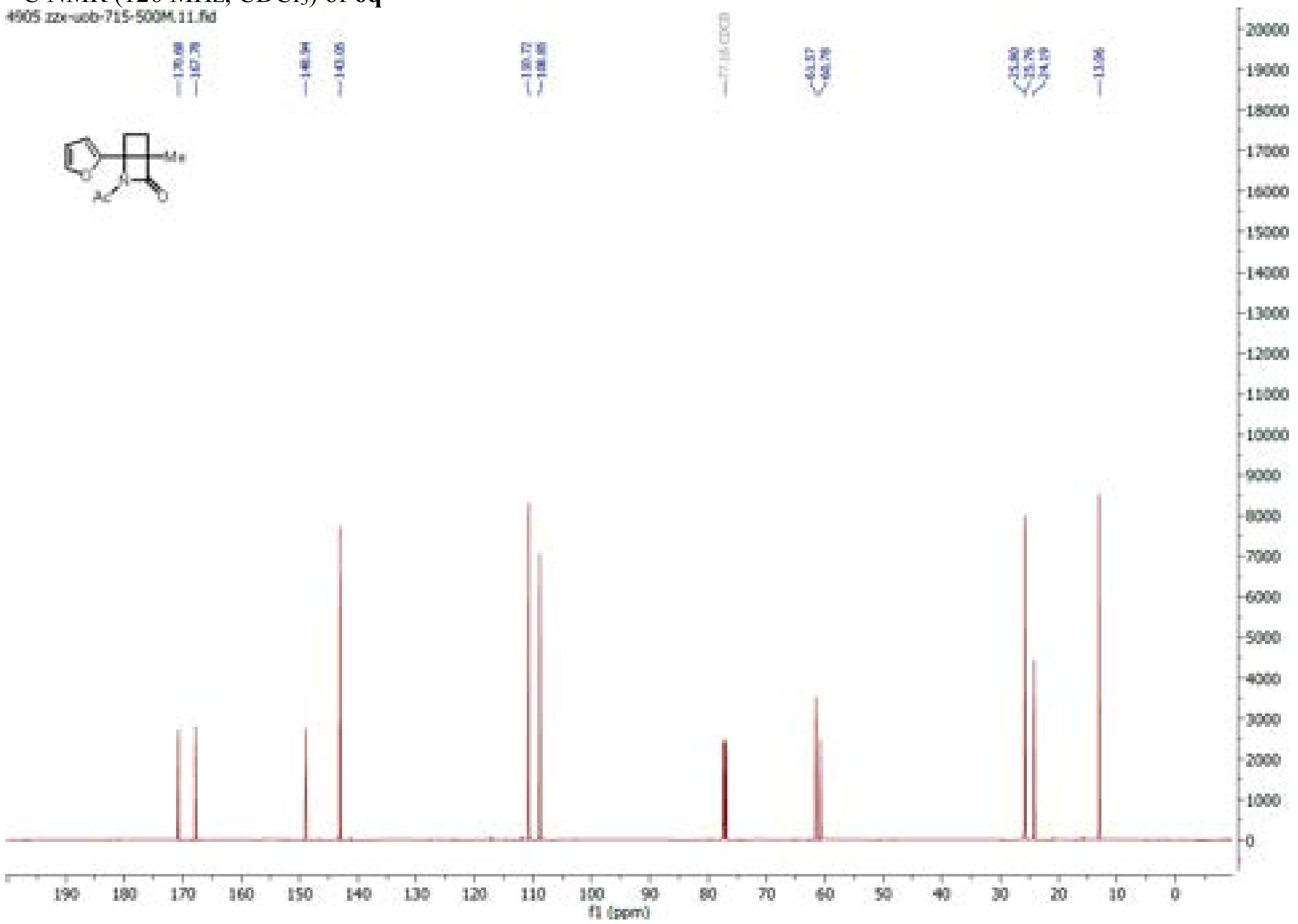

^{1}H NMR (500 MHz, $CDCl_3$) of **6r**

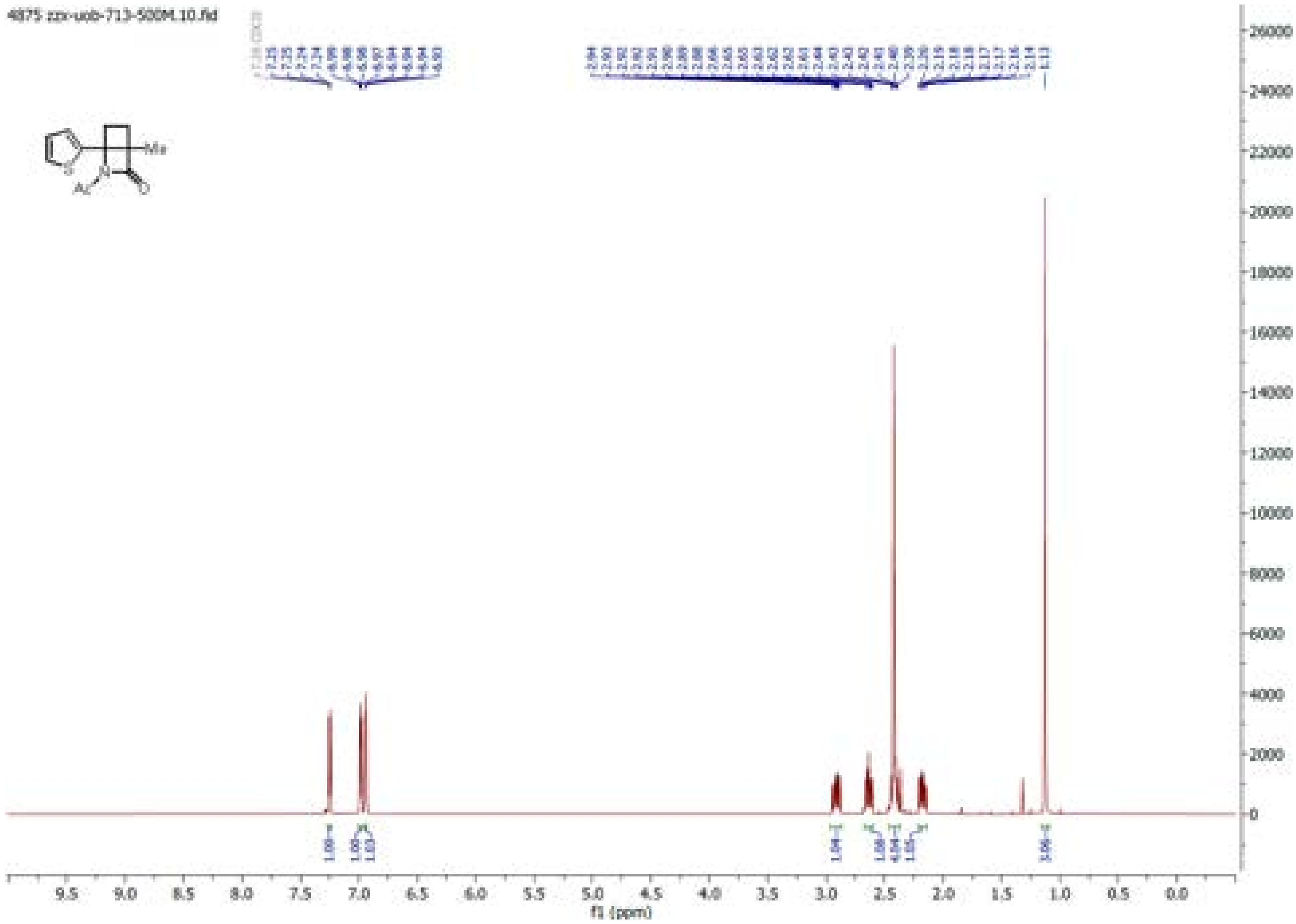

^{13}C NMR (126 MHz, $CDCl_3$) of **6r**

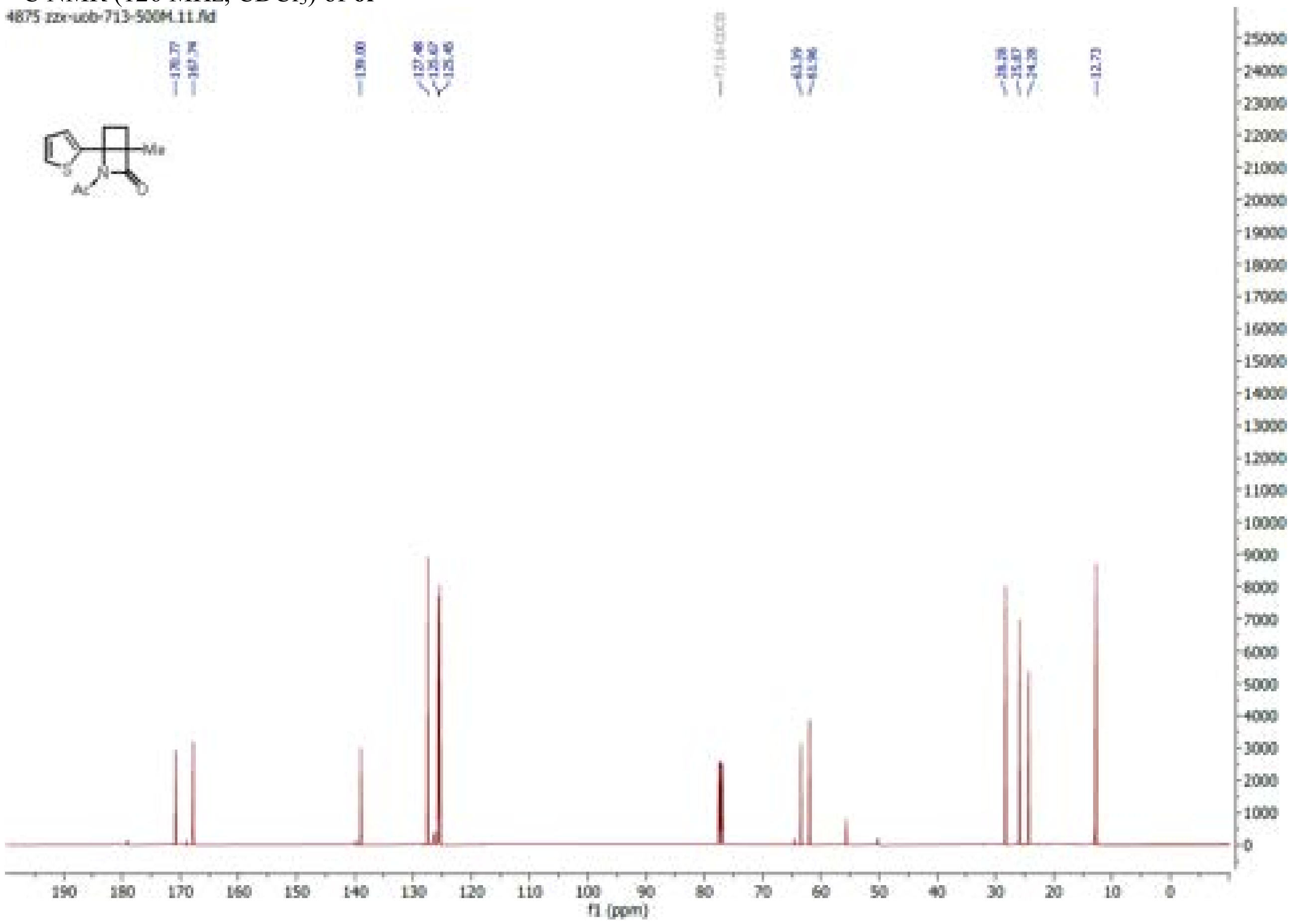

^{1}H NMR (500 MHz, $CDCl_3$) of **6s**

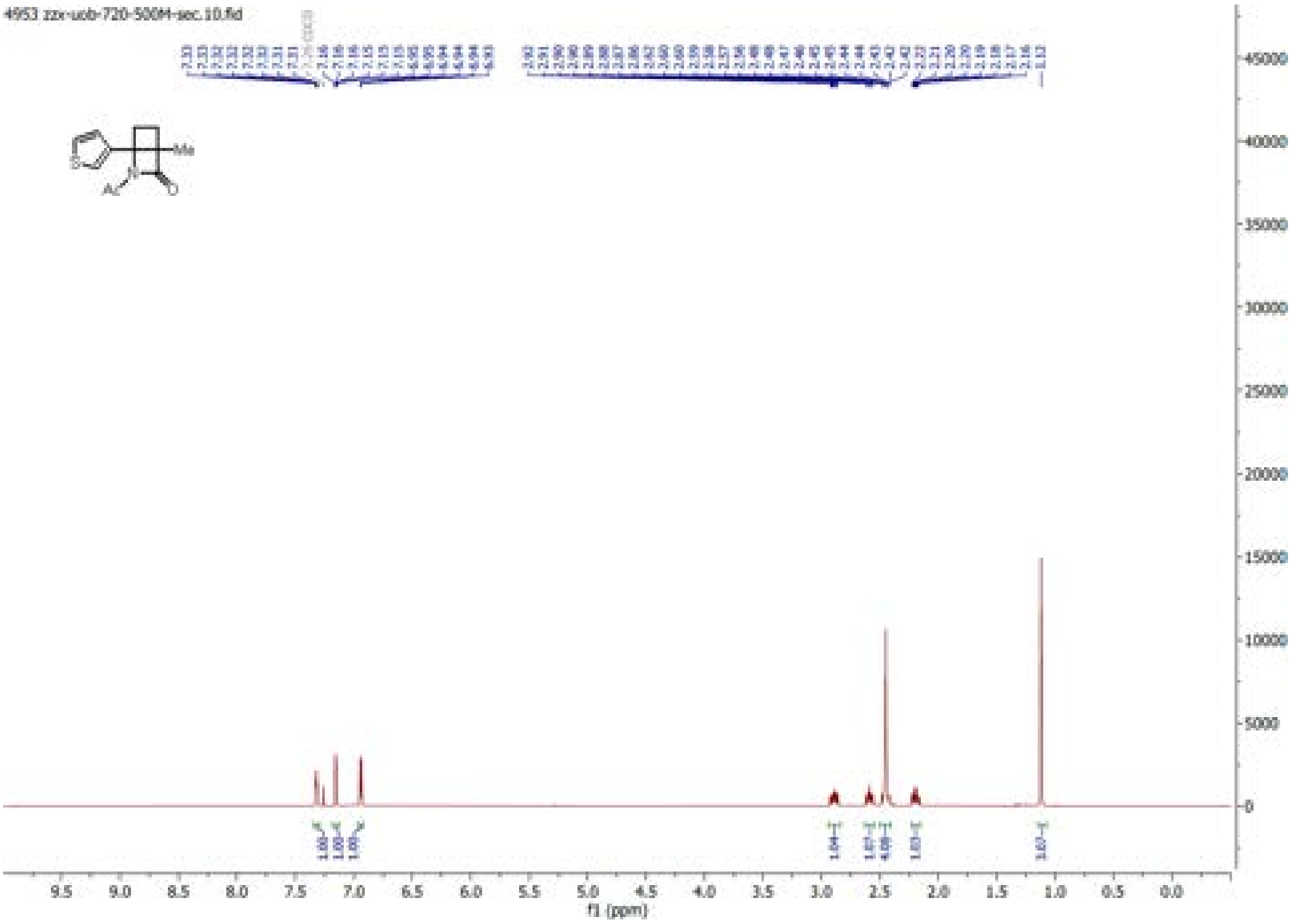

^{13}C NMR (126 MHz, $CDCl_3$) of **6s**

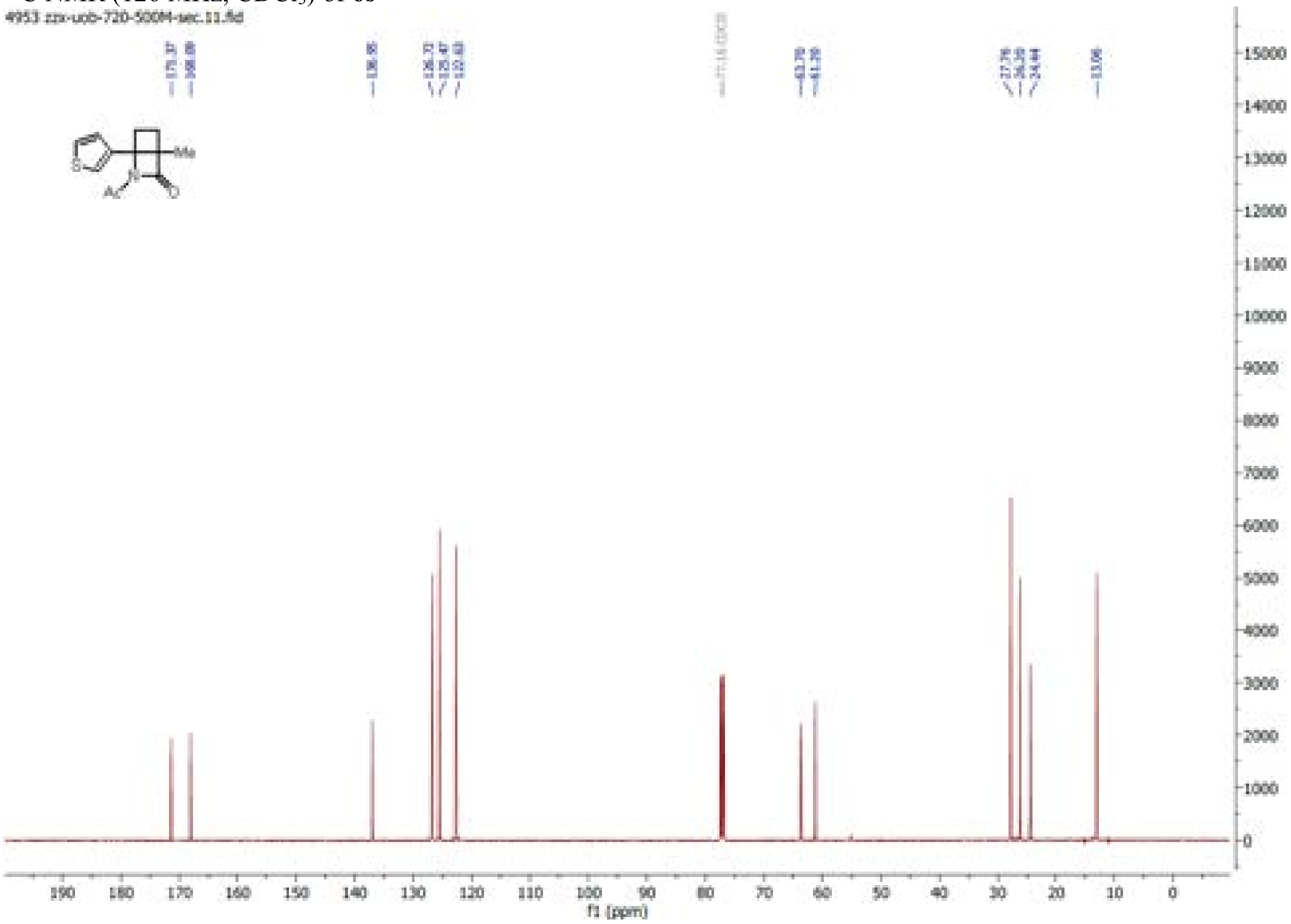

^{1}H NMR (500 MHz, $CDCl_3$) of **6t**

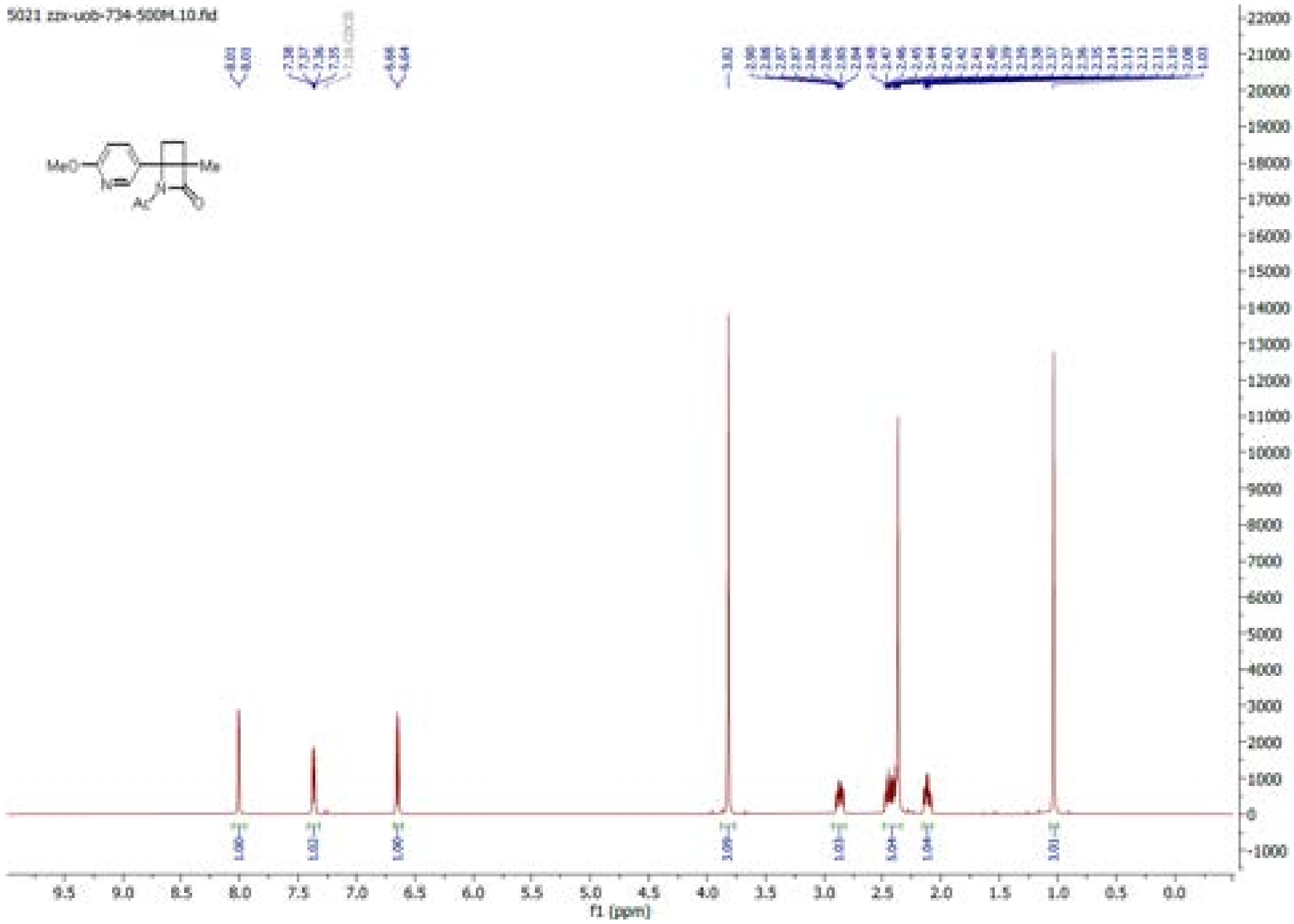

^{13}C NMR (126 MHz, $CDCl_3$) of **6t**

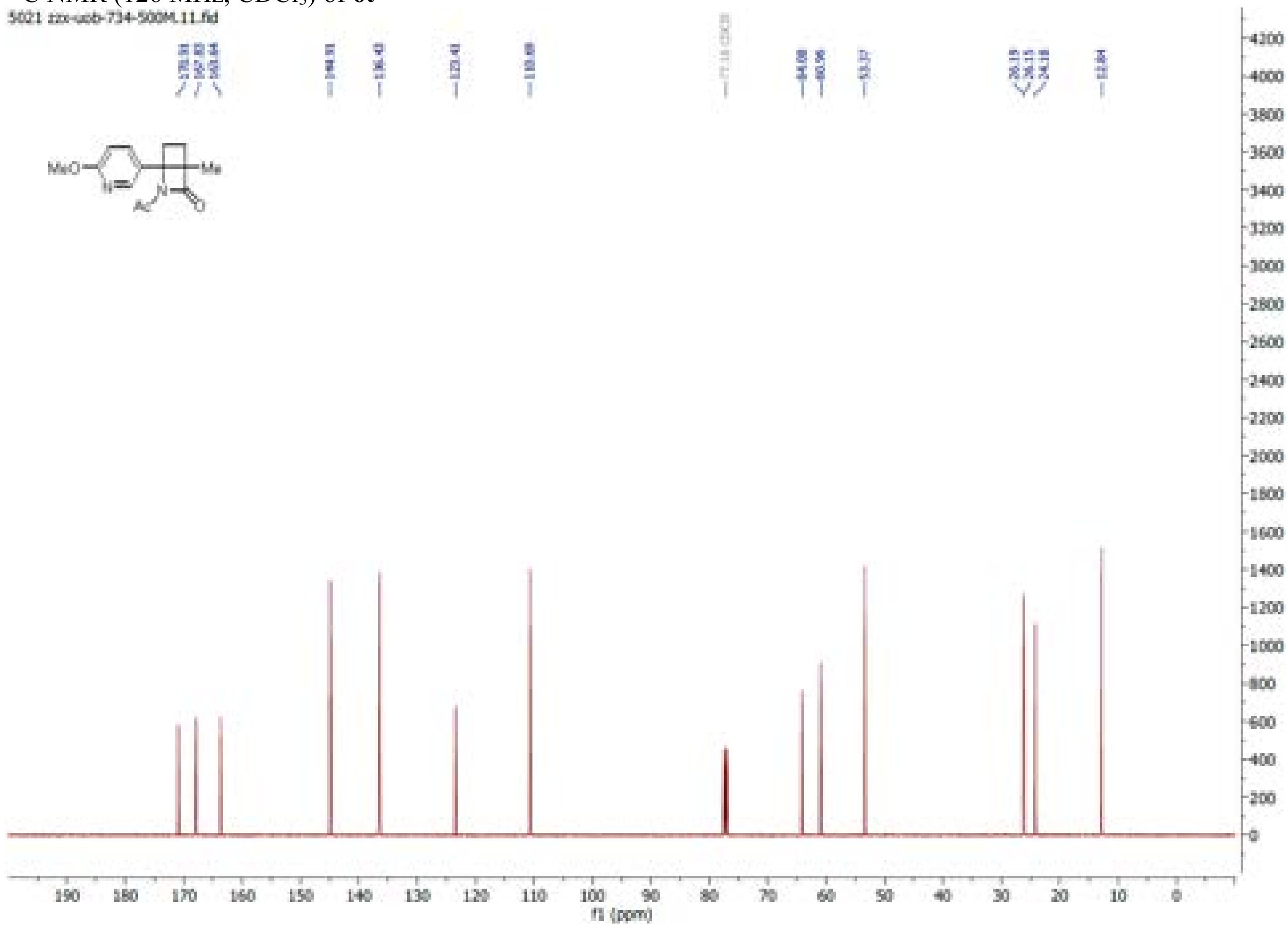

^1H NMR (500 MHz, $CDCl_3$) of **5u**

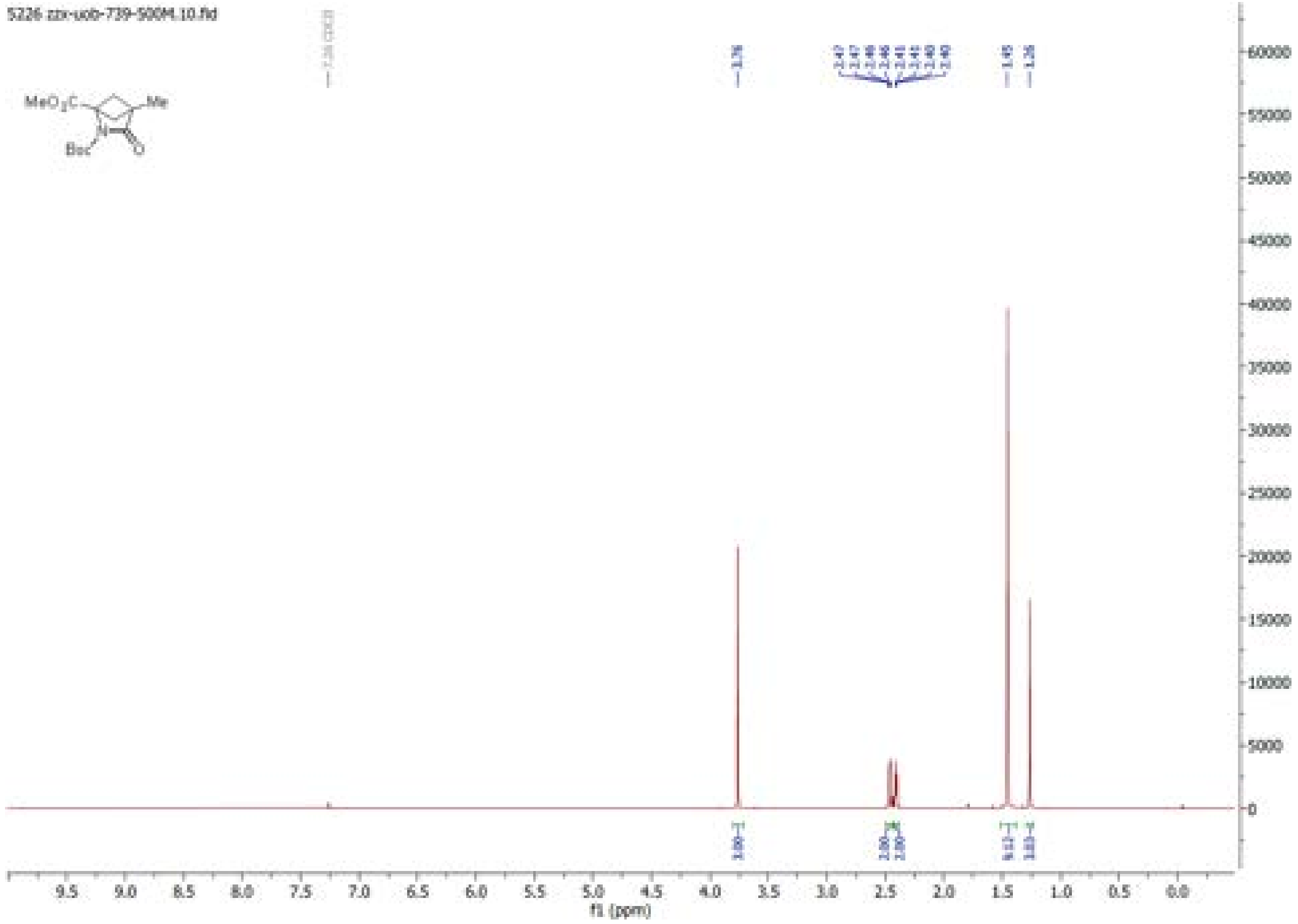

^{13}C NMR (126 MHz, $CDCl_3$) of **5u**

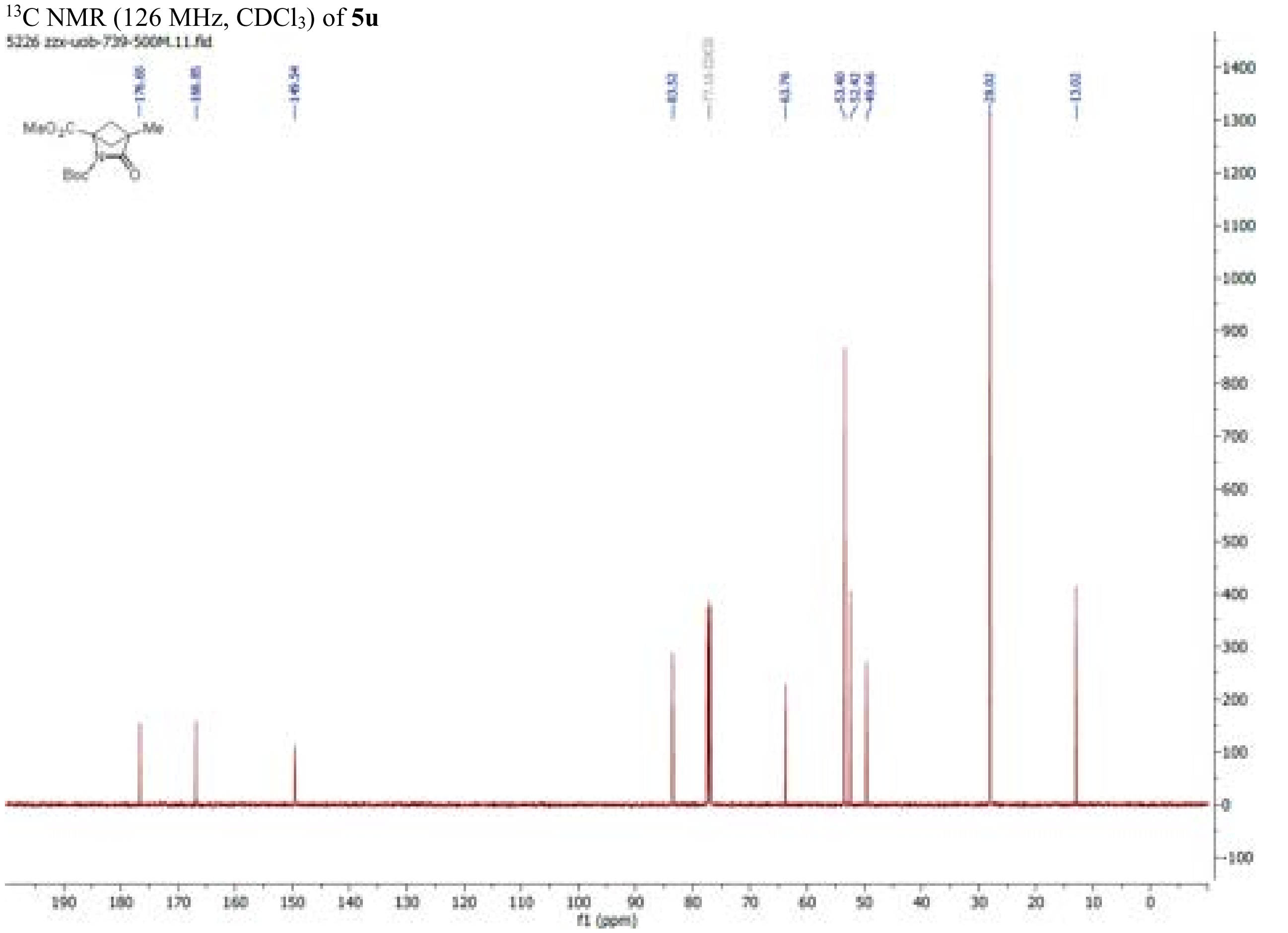

^{1}H NMR (500 MHz, $CDCl_3$) of **6v**

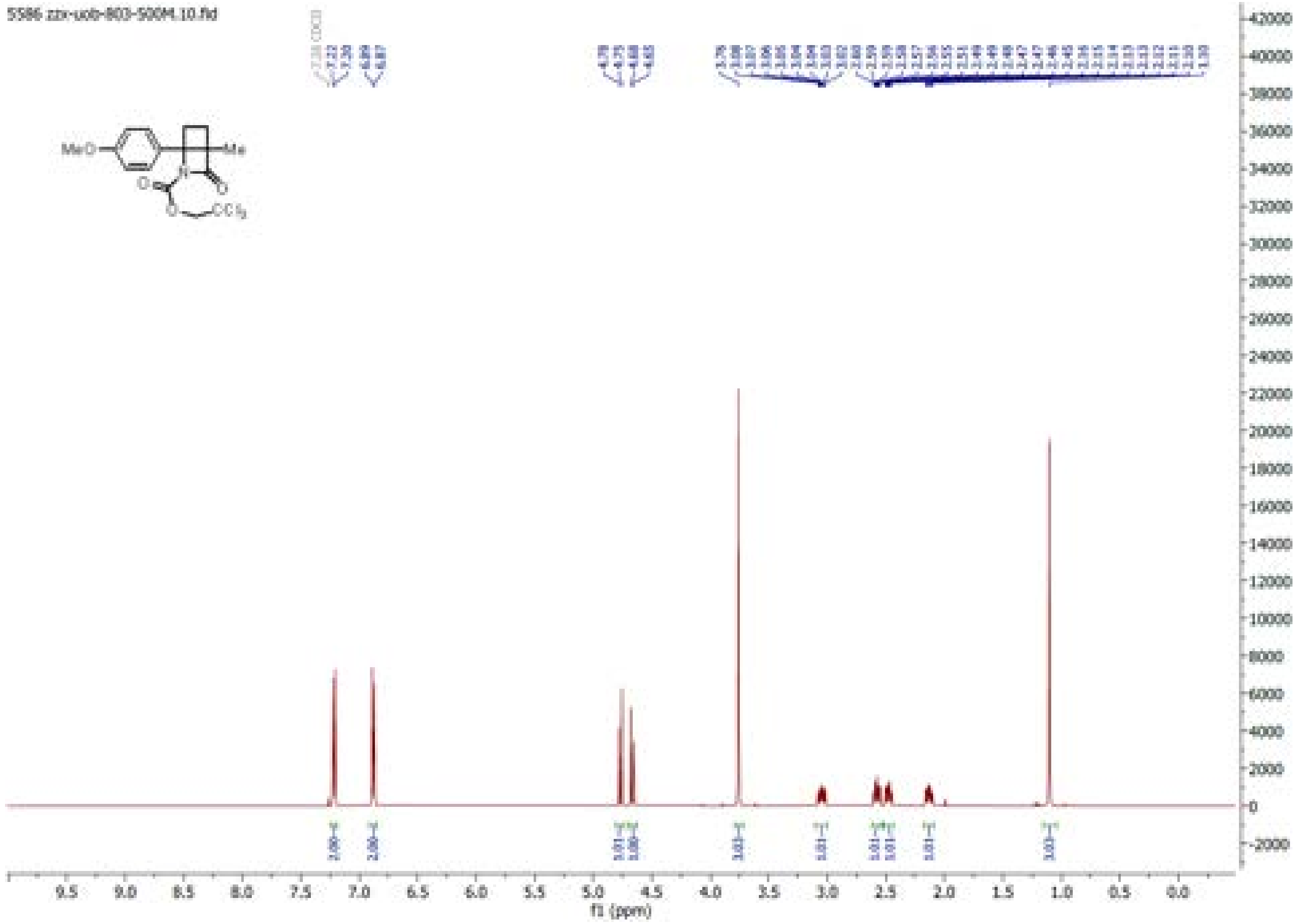

^{13}C NMR (126 MHz, $CDCl_3$) of **6v**

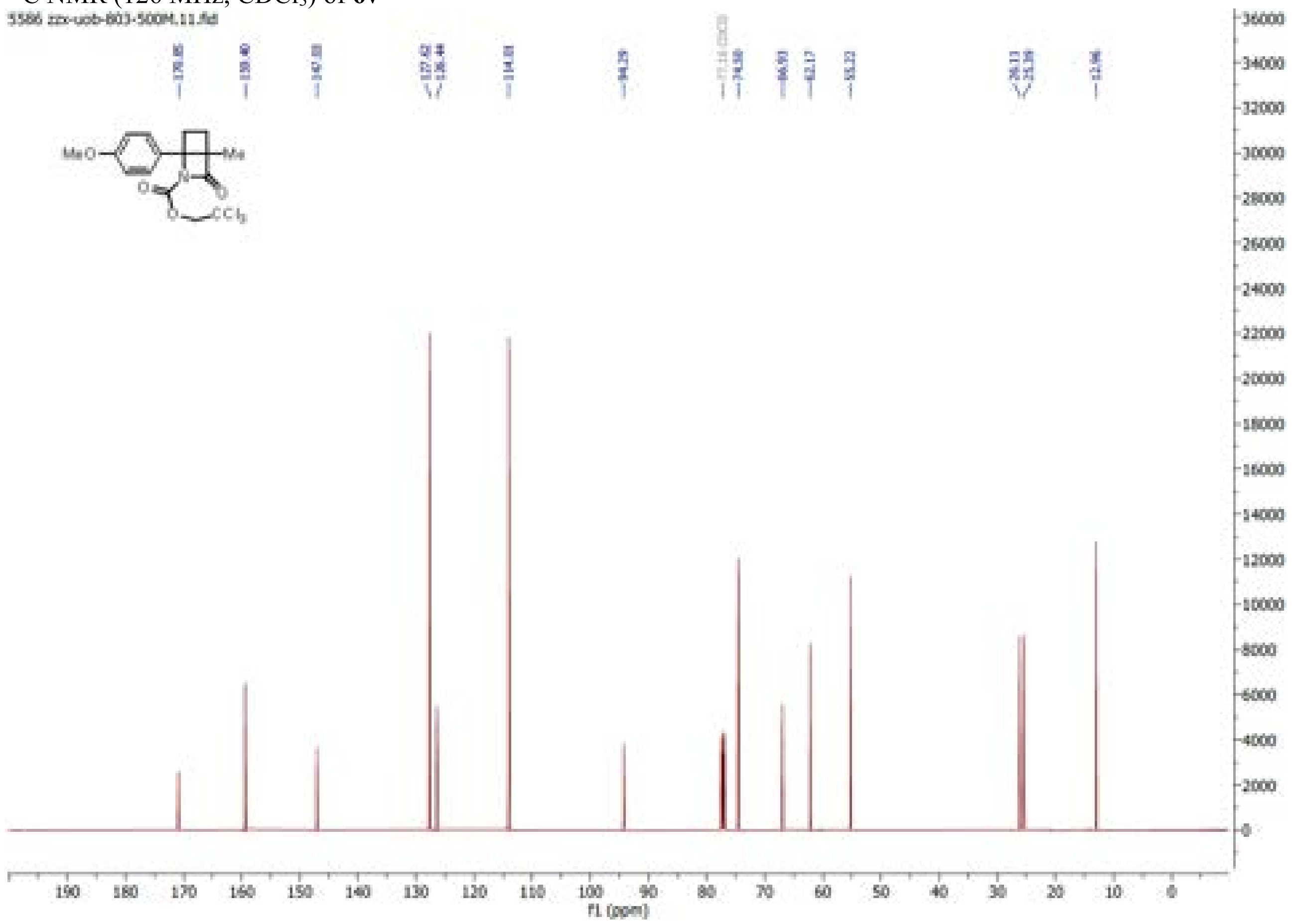

^{1}H NMR (500 MHz, $CDCl_3$) of **6w**

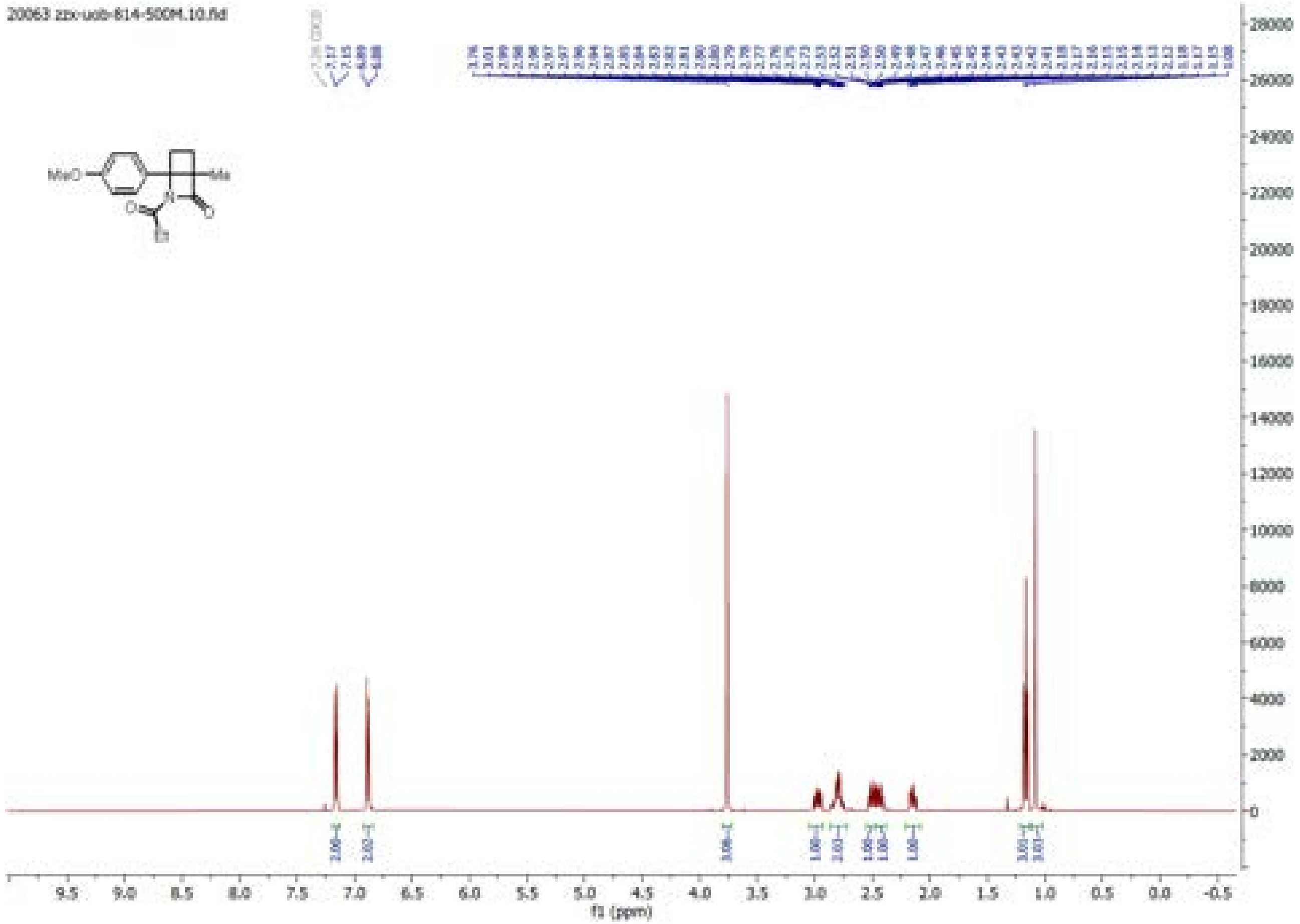

^{13}C NMR (126 MHz, $CDCl_3$) of **6w**

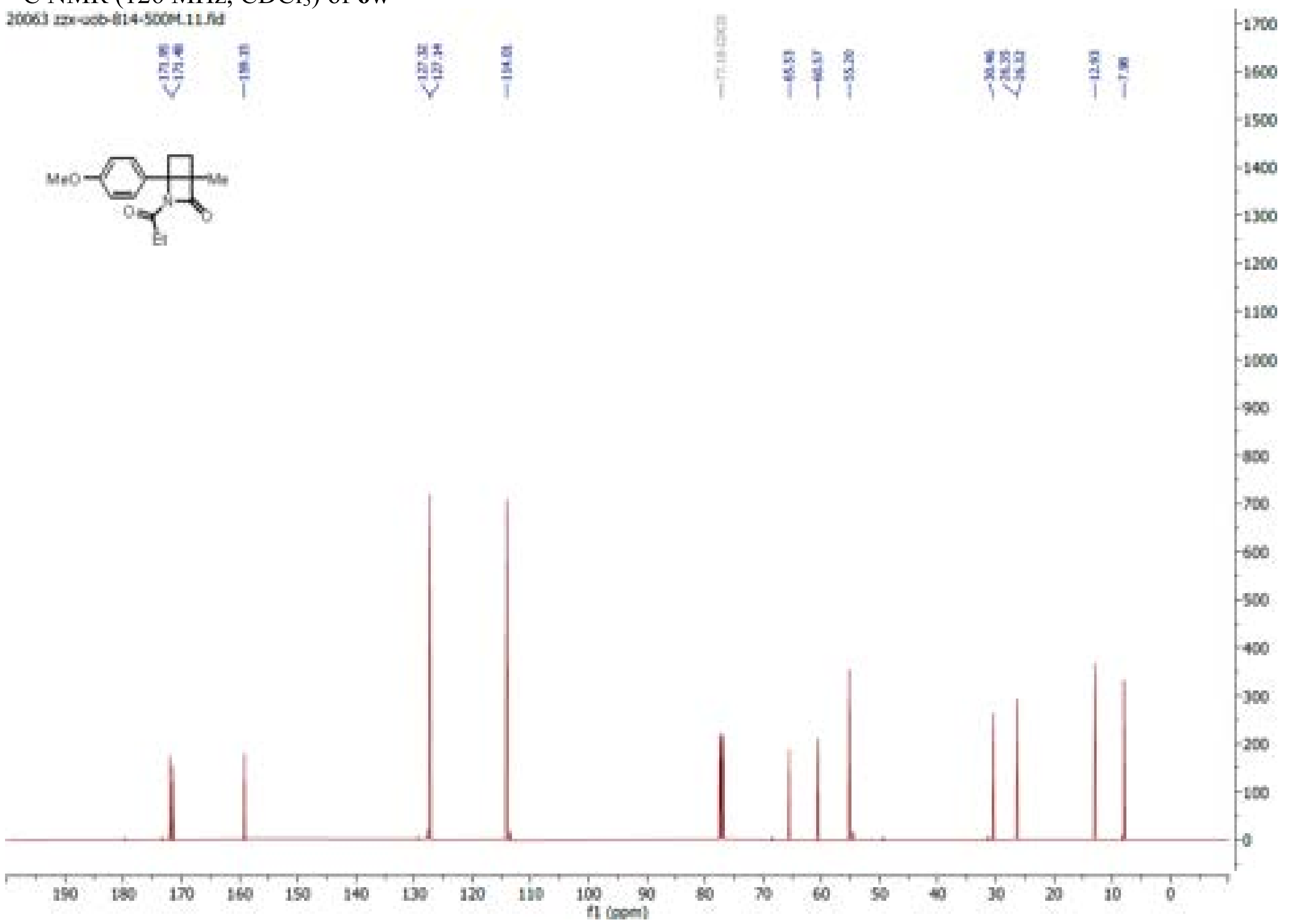

^{1}H NMR (500 MHz, $CDCl_3$) of **6x**

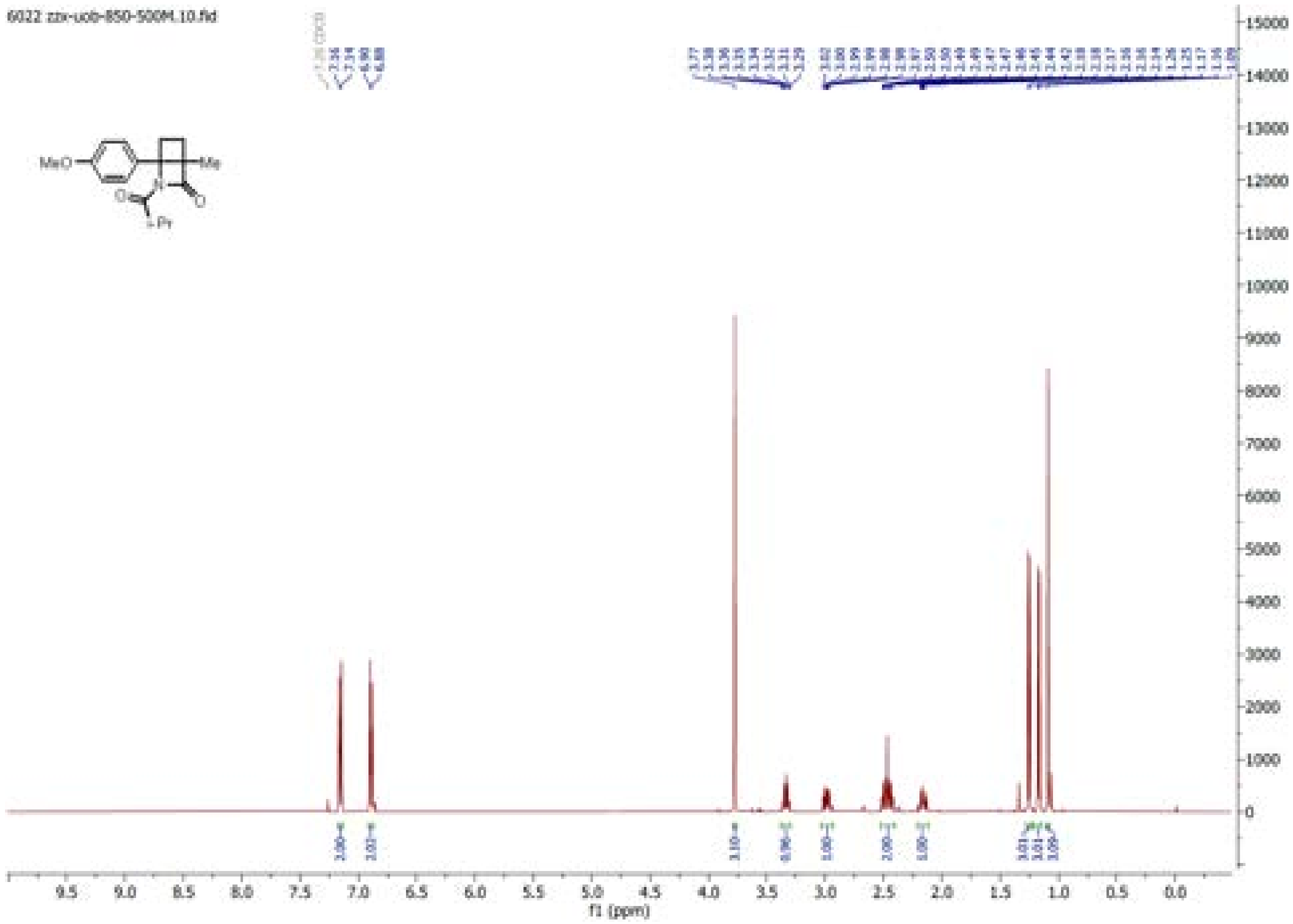

^{13}C NMR (126 MHz, $CDCl_3$) of **6x**

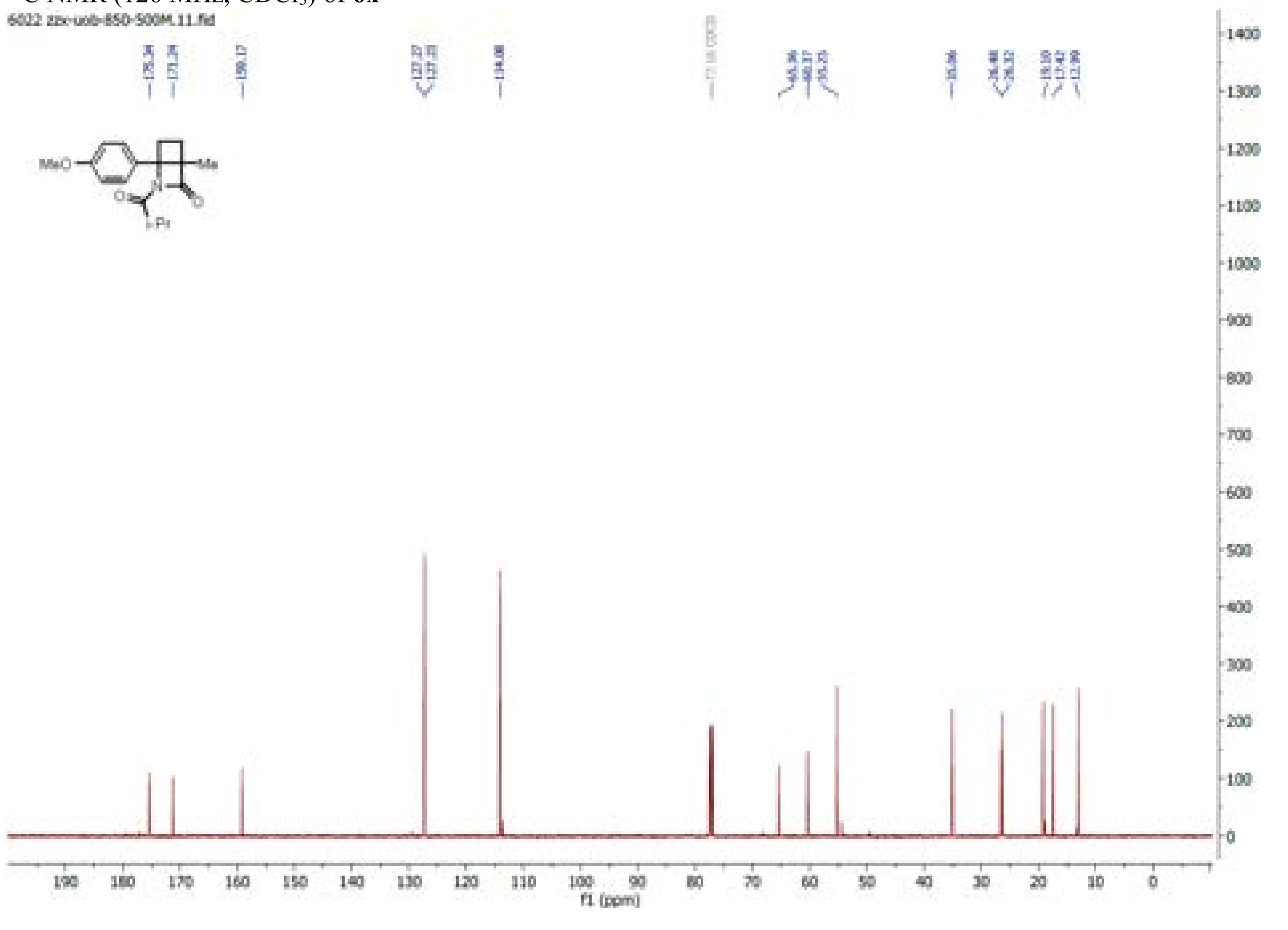

^{1}H NMR (500 MHz, $CDCl_3$) of **6y**

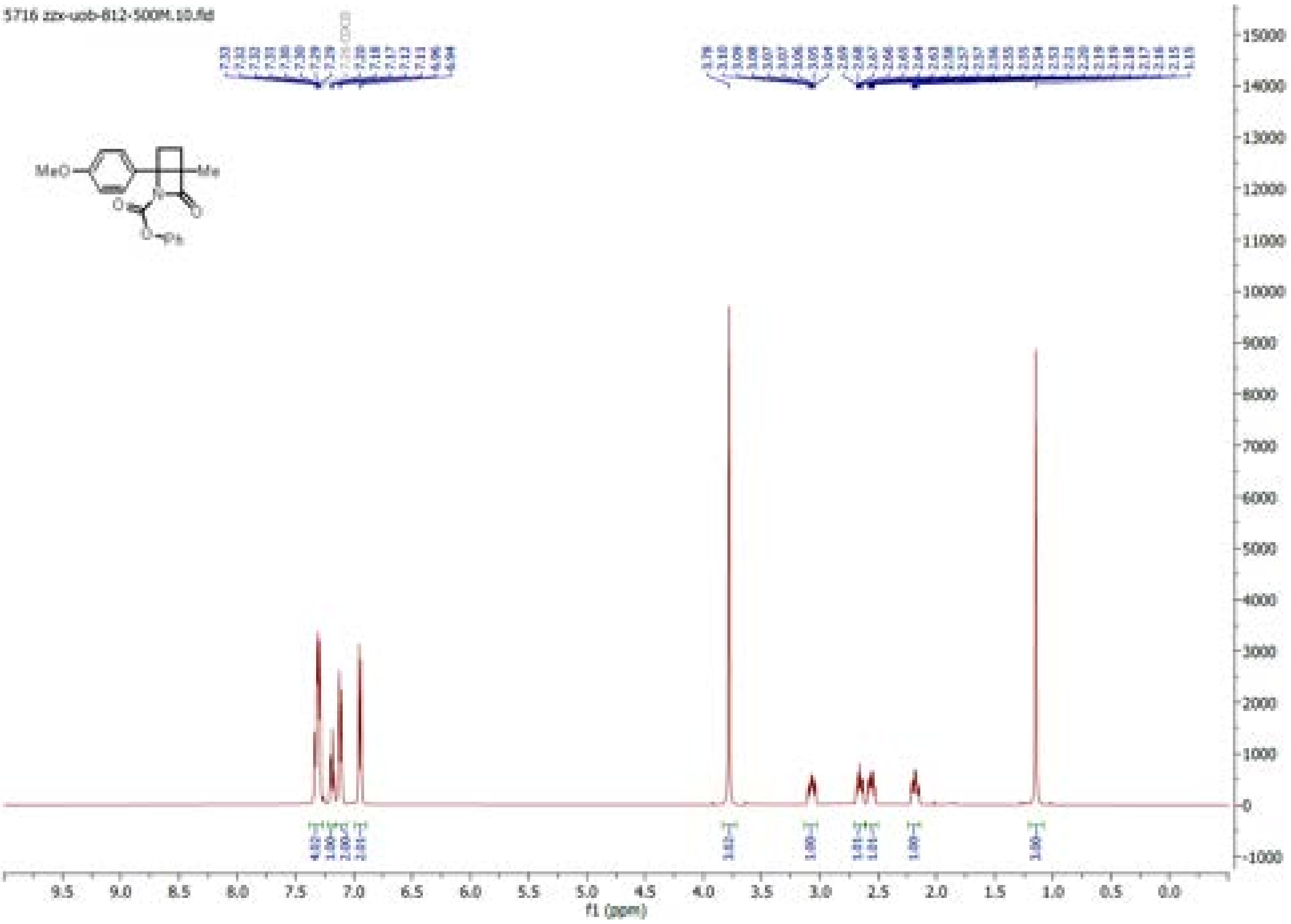

^{13}C NMR (126 MHz, $CDCl_3$) of **6y**

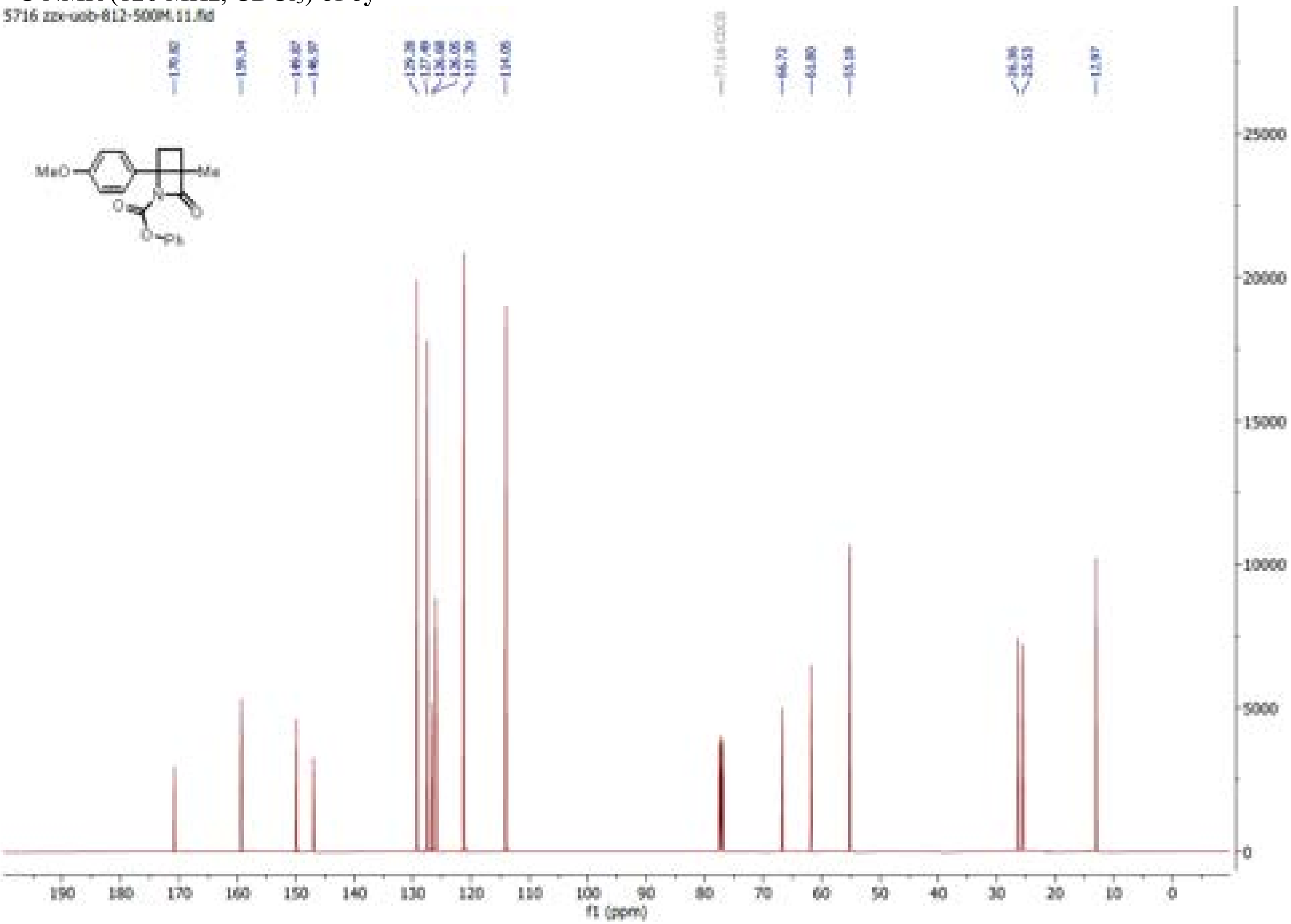

^{1}H NMR (500 MHz, $CDCl_3$) of **6z**

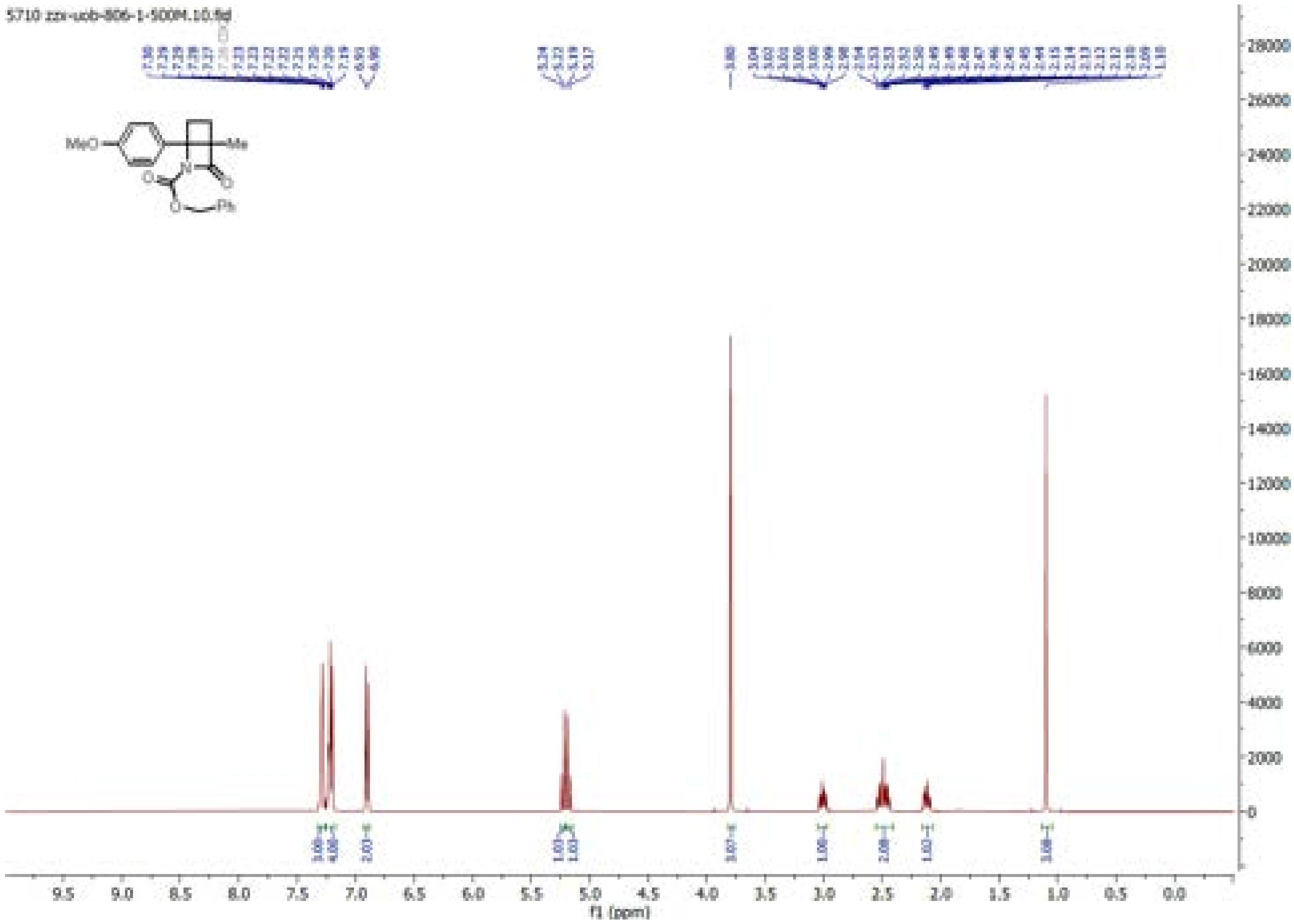

^{13}C NMR (126 MHz, $CDCl_3$) of **6z**

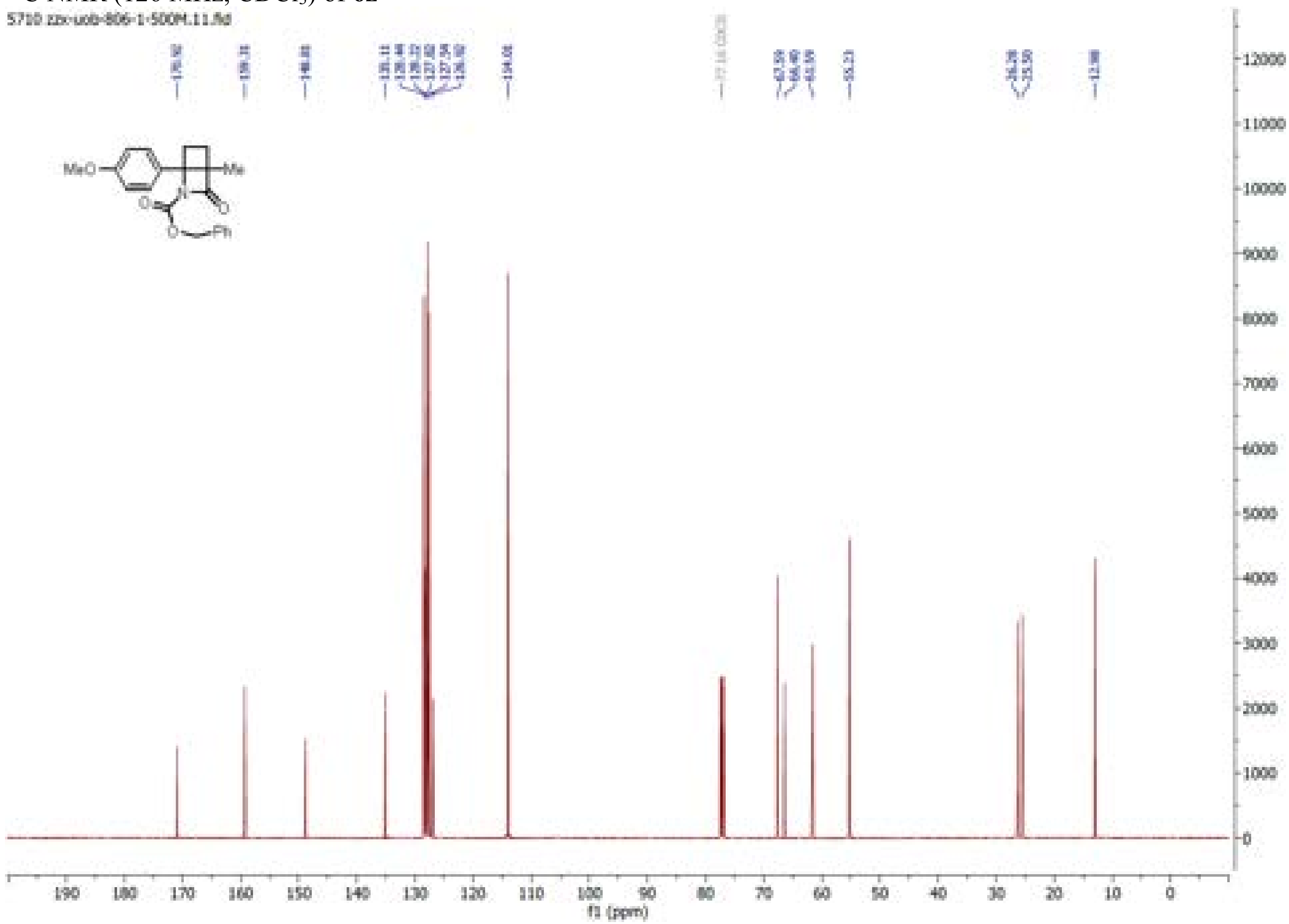

^{1}H NMR (500 MHz, $CDCl_3$) of **6aa**

5708 zzx-uob-810-2-500M.10.fid

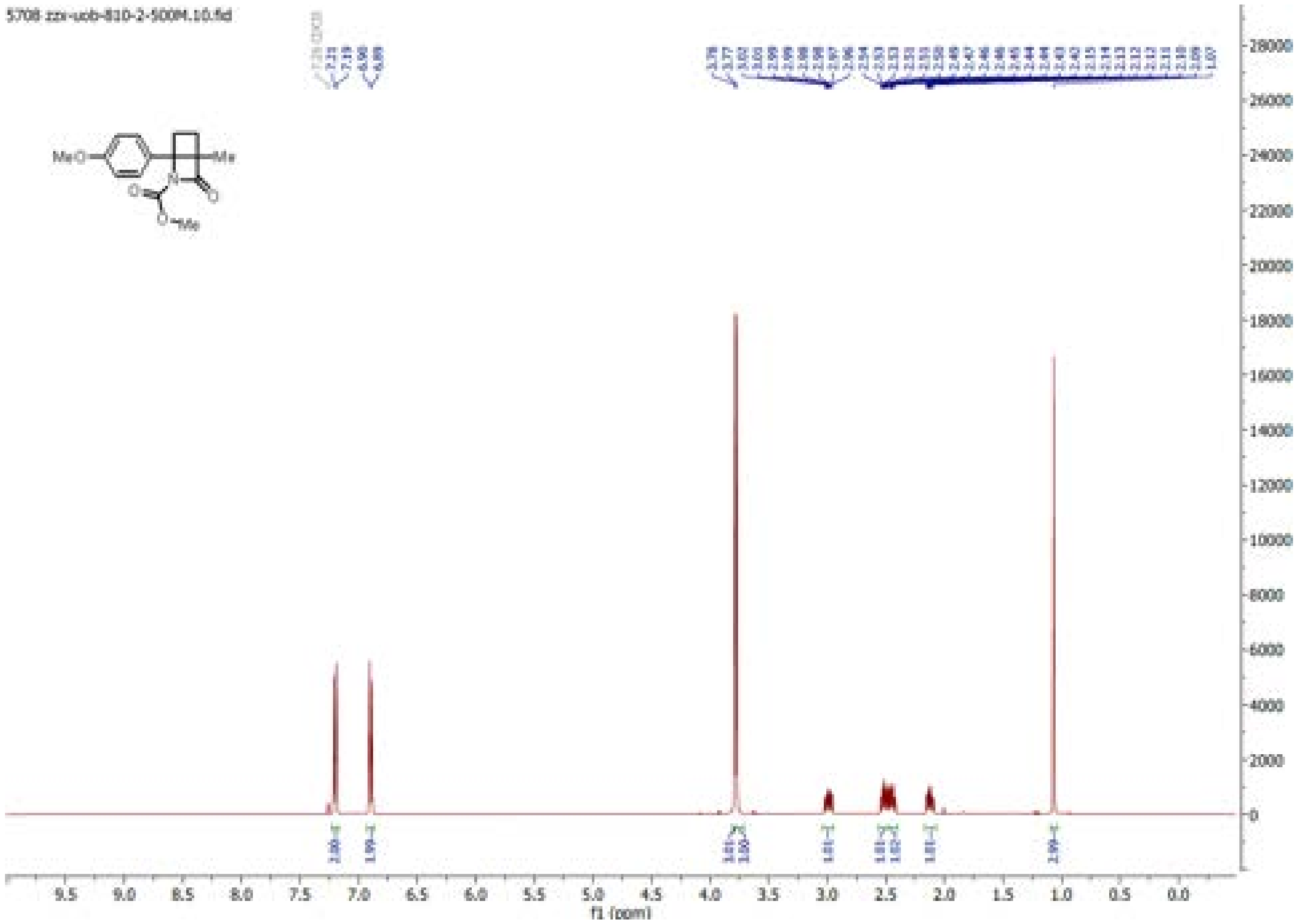

^{13}C NMR (126 MHz, $CDCl_3$) of **6aa**

5708 zzx-uob-810-2-500M.11.fid

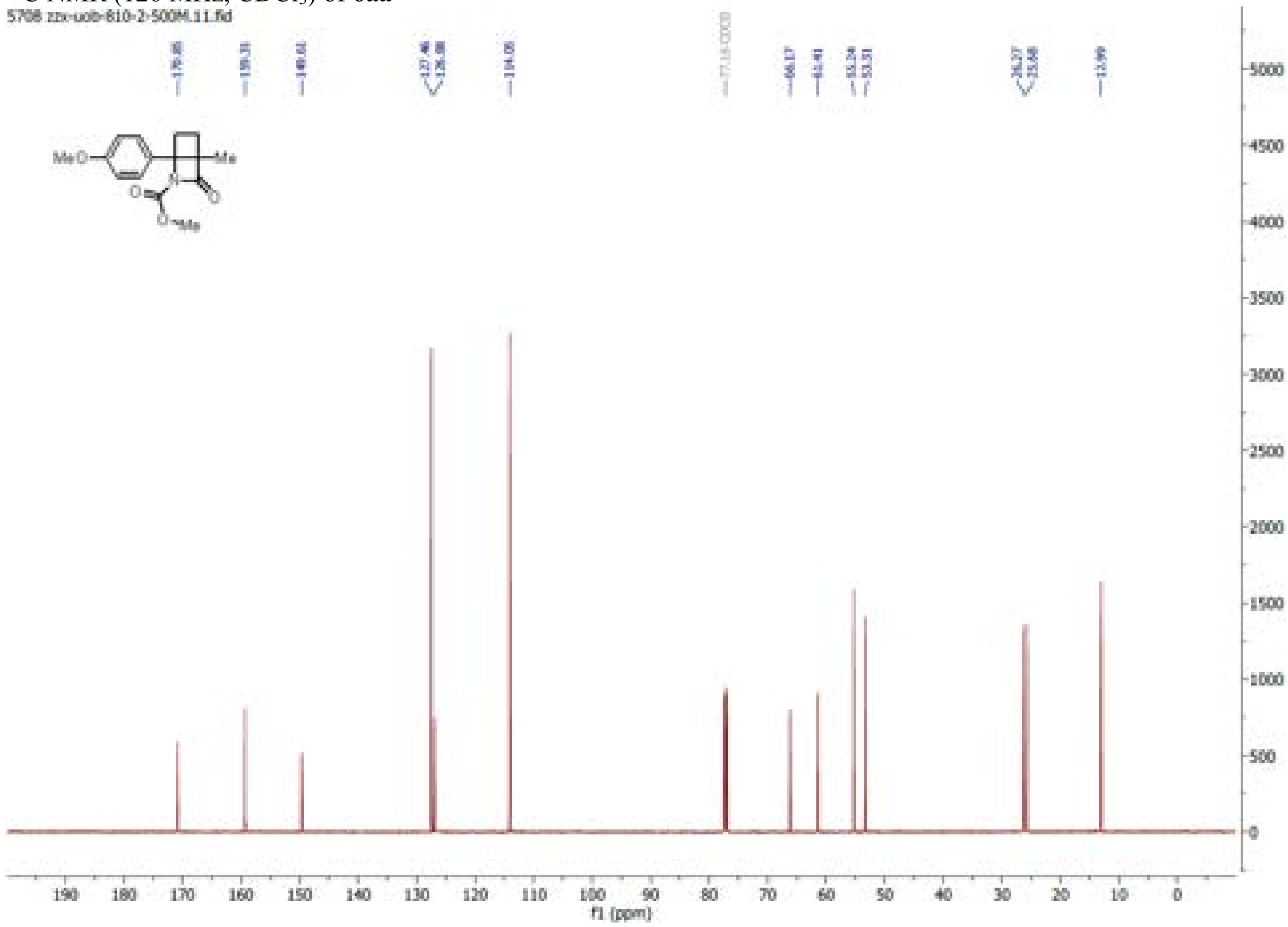

^{1}H NMR (500 MHz, $CDCl_3$) of **6ab**

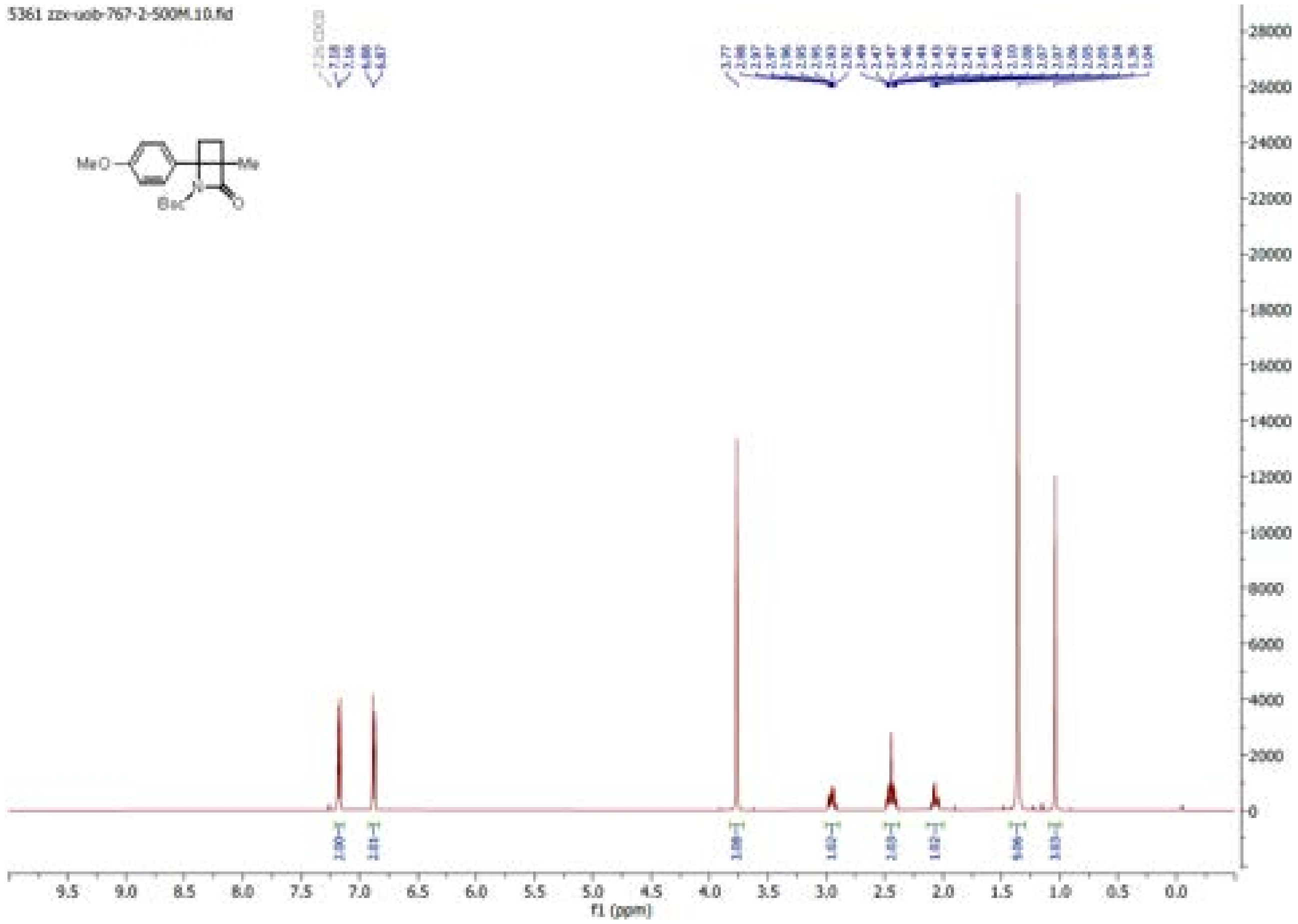

^{13}C NMR (126 MHz, $CDCl_3$) of **6ab**

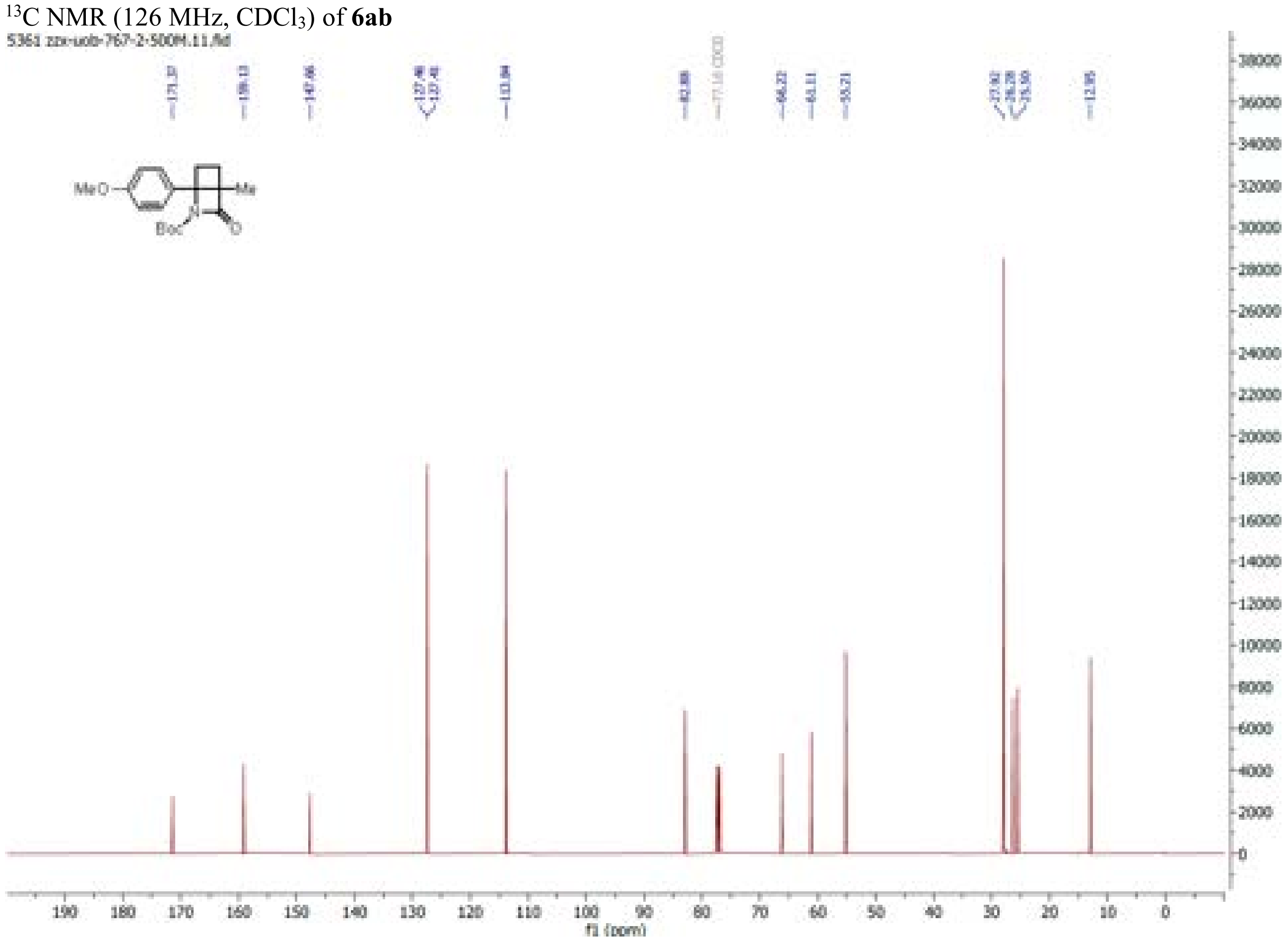

^1H NMR (500 MHz, $CDCl_3$) of **5ab**

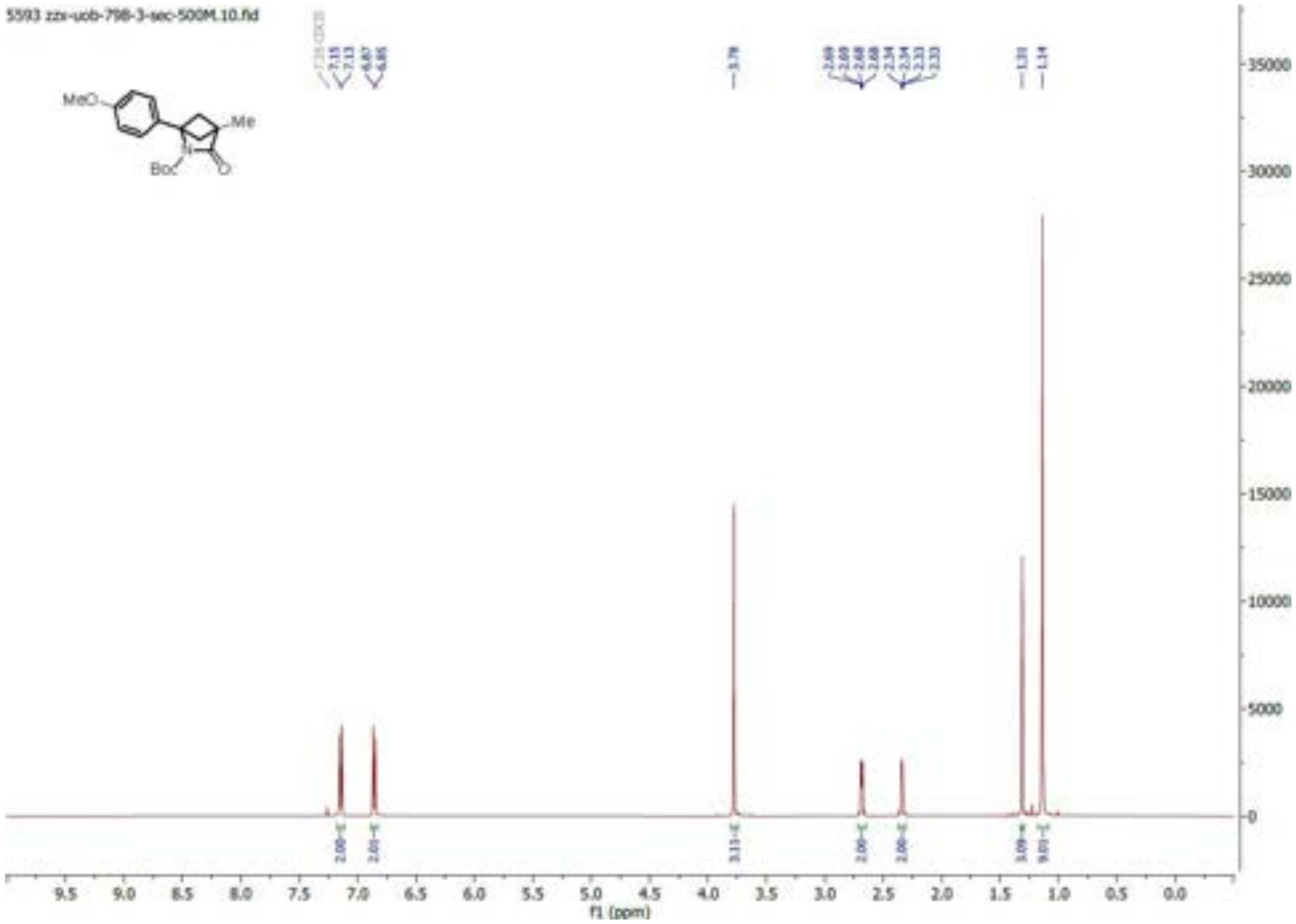

^{13}C NMR (126 MHz, $CDCl_3$) of **5ab**

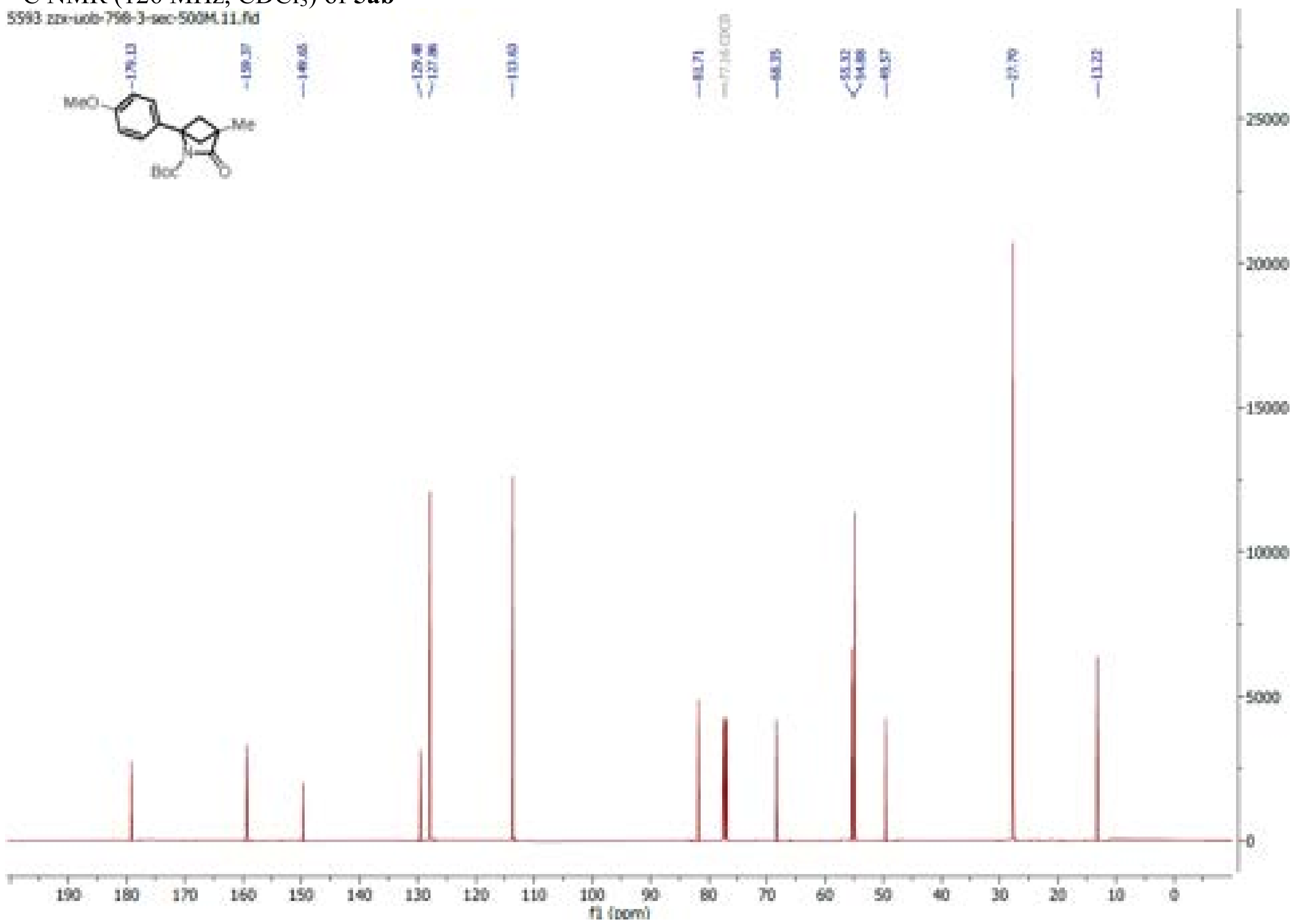

^{1}H NMR (500 MHz, CD_2Cl_2) of **9**

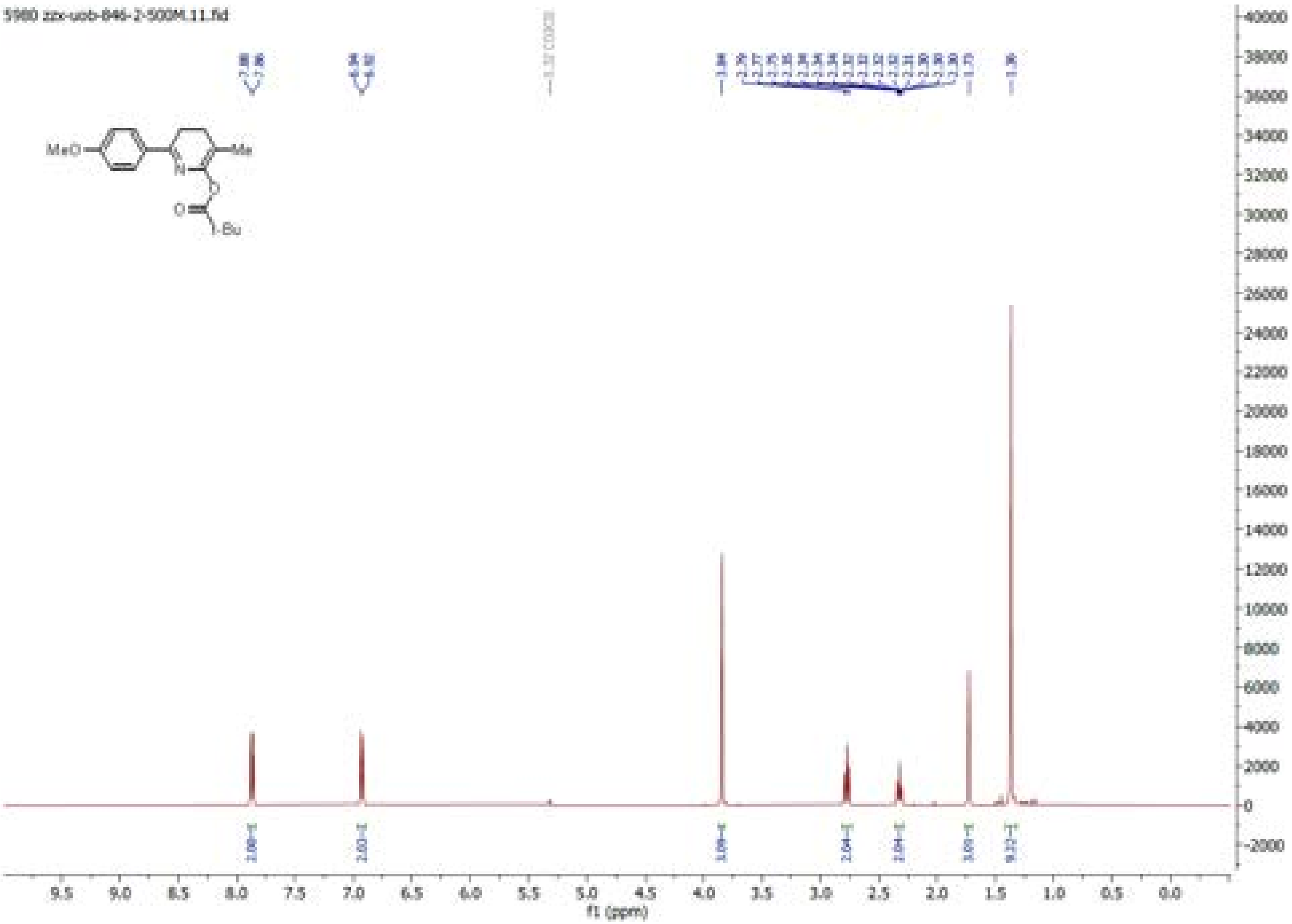

^{13}C NMR (126 MHz, CD_2Cl_2) of **9**

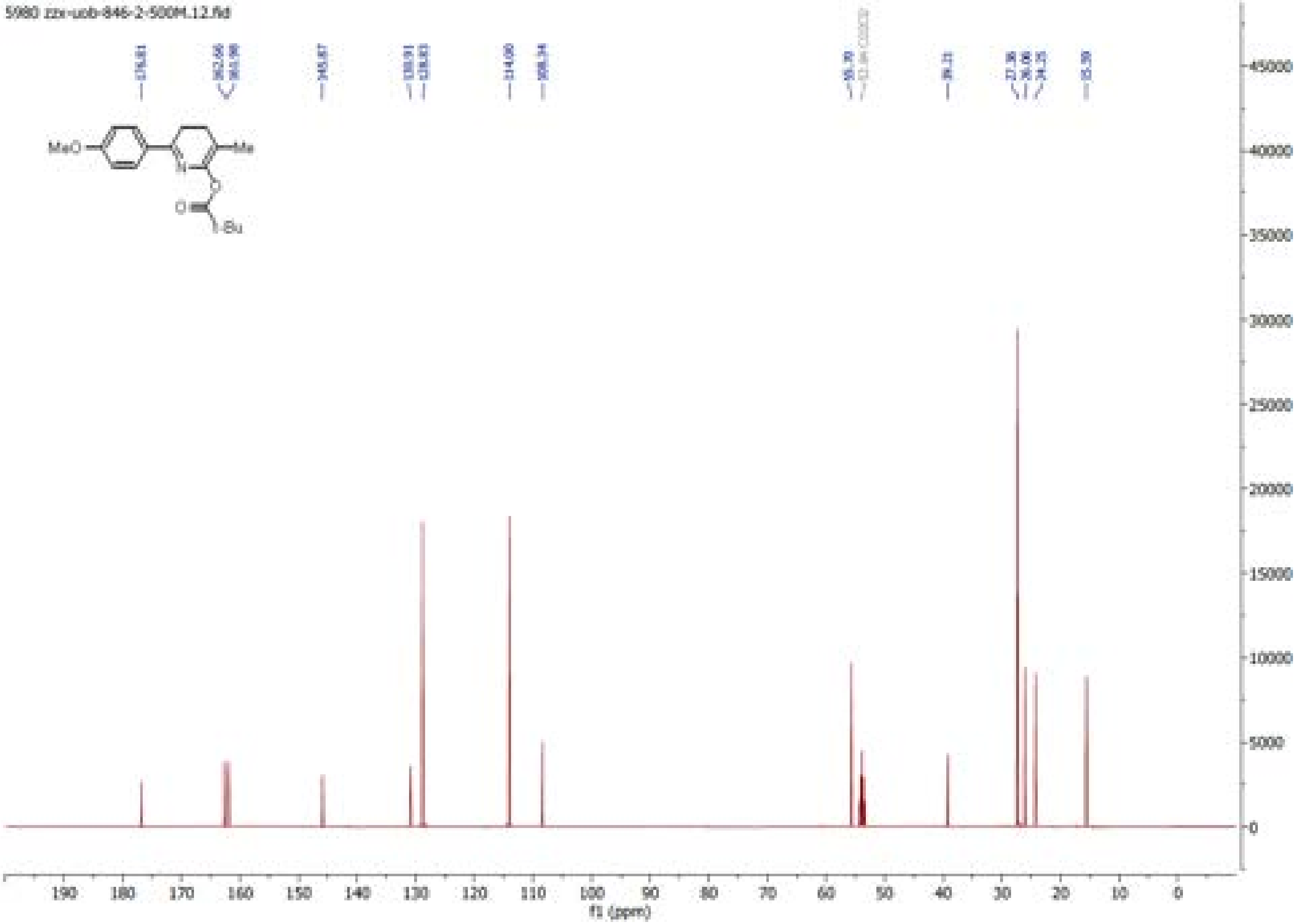

^{1}H NMR (500 MHz, $CDCl_3$) of **6ad and 6ad' (isomer 1)**

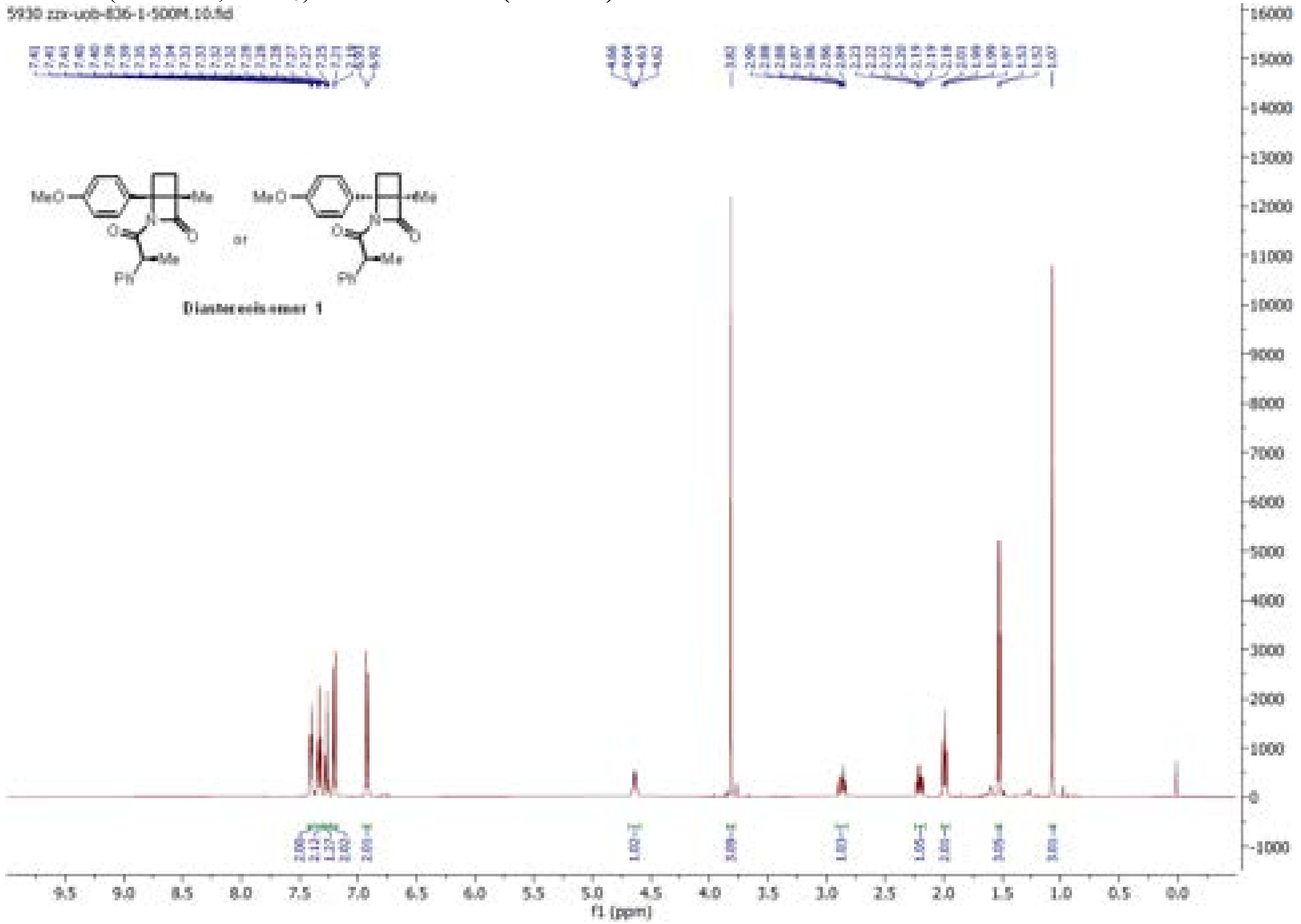

^{13}C NMR (126 MHz, $CDCl_3$) of **6ad and 6ad' (isomer 1)**

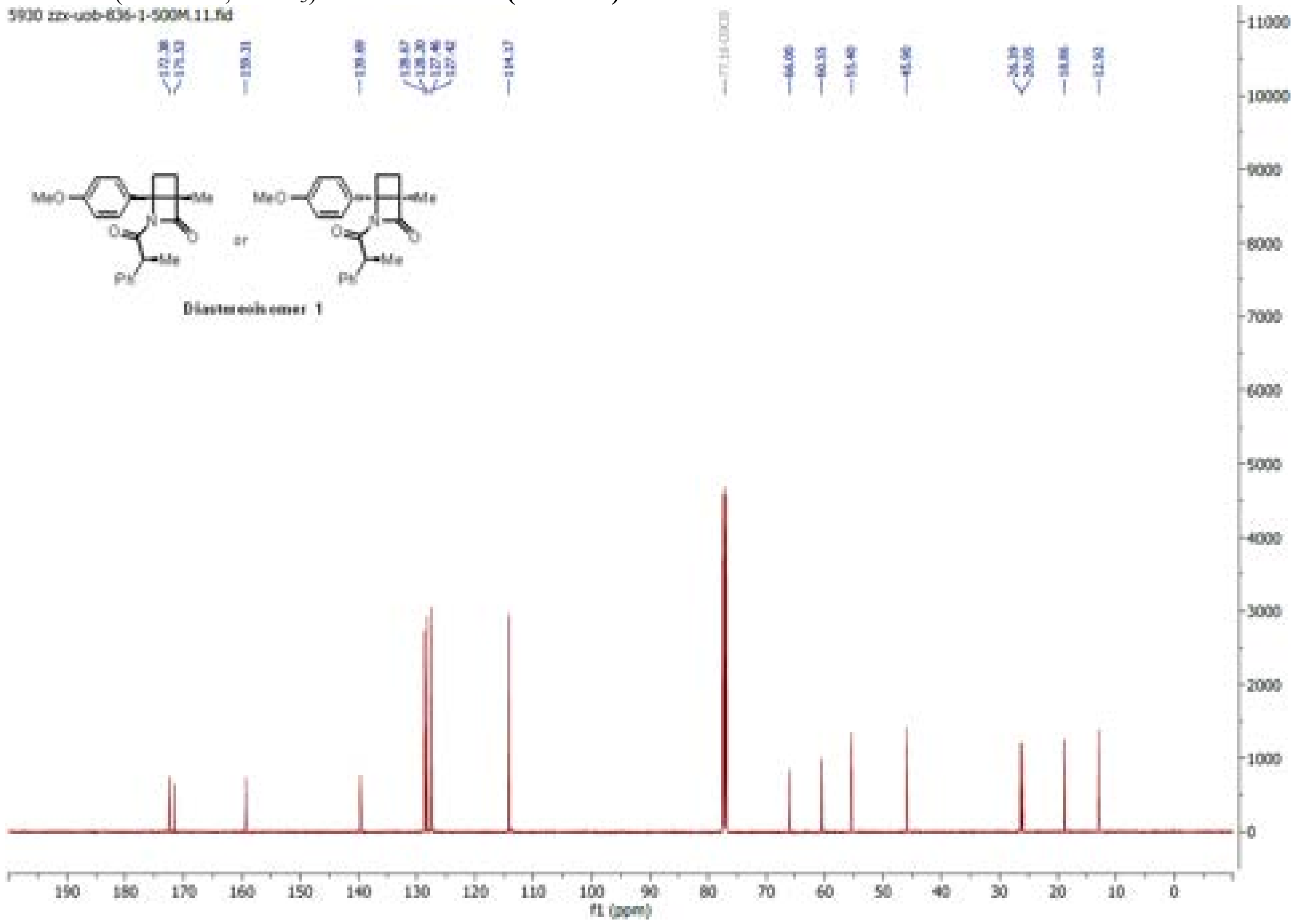

^{1}H NMR (500 MHz, $CDCl_3$) of **6ad and 6ad' (isomer 2)**

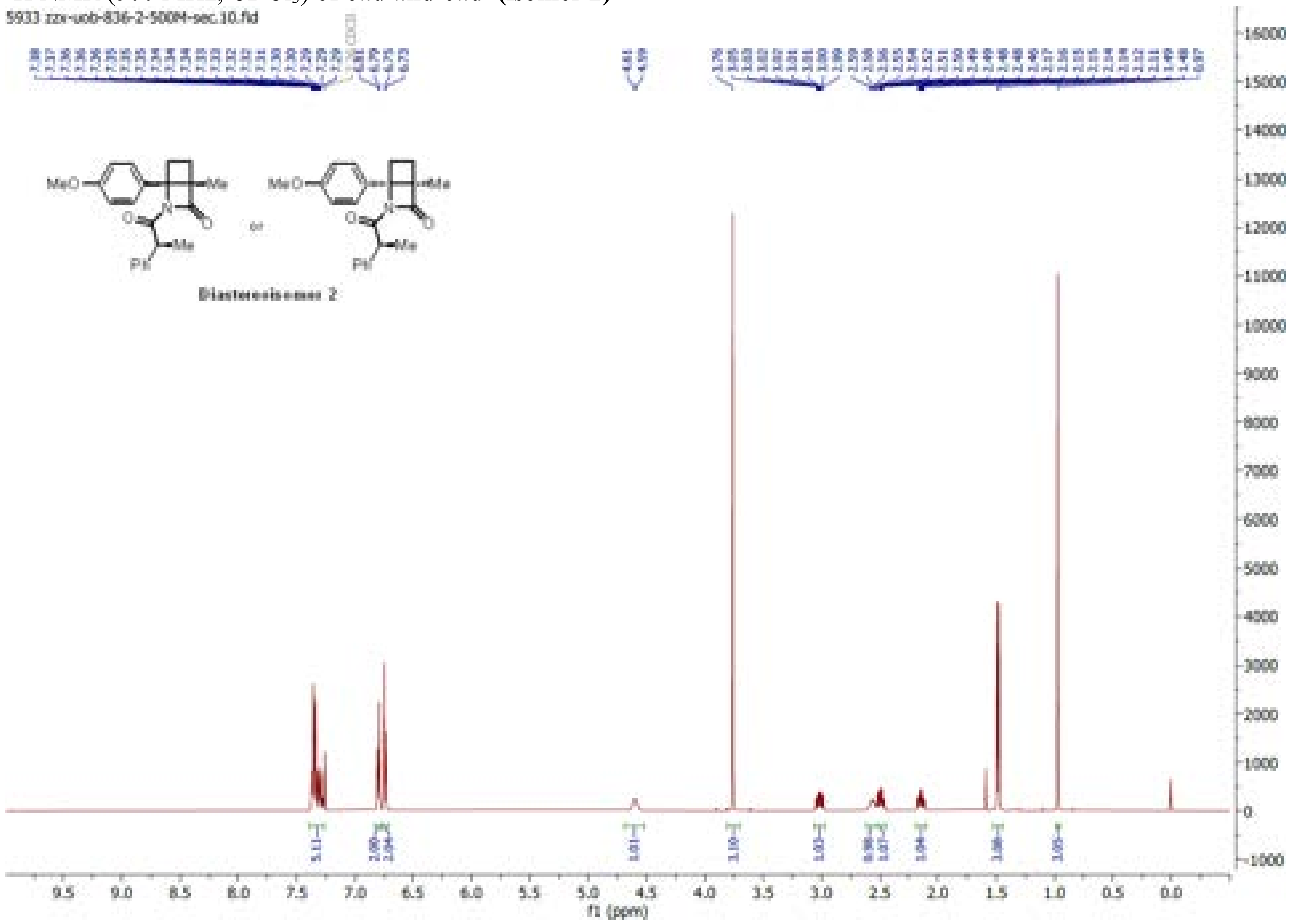

^{13}C NMR (126 MHz, $CDCl_3$) of **6ad and 6ad' (isomer 2)**

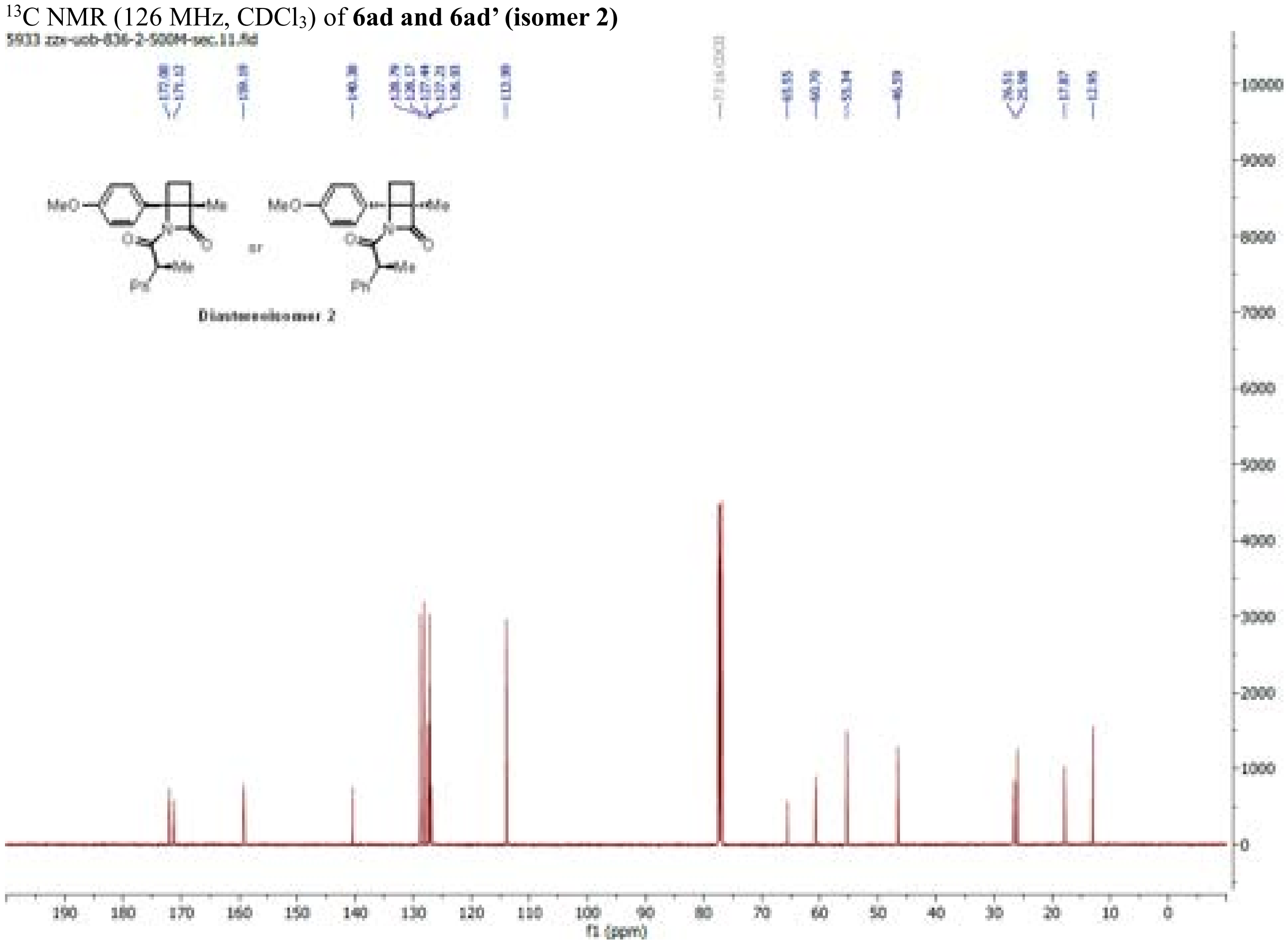

^{1}H NMR (500 MHz, $CDCl_3$) of **6ae**

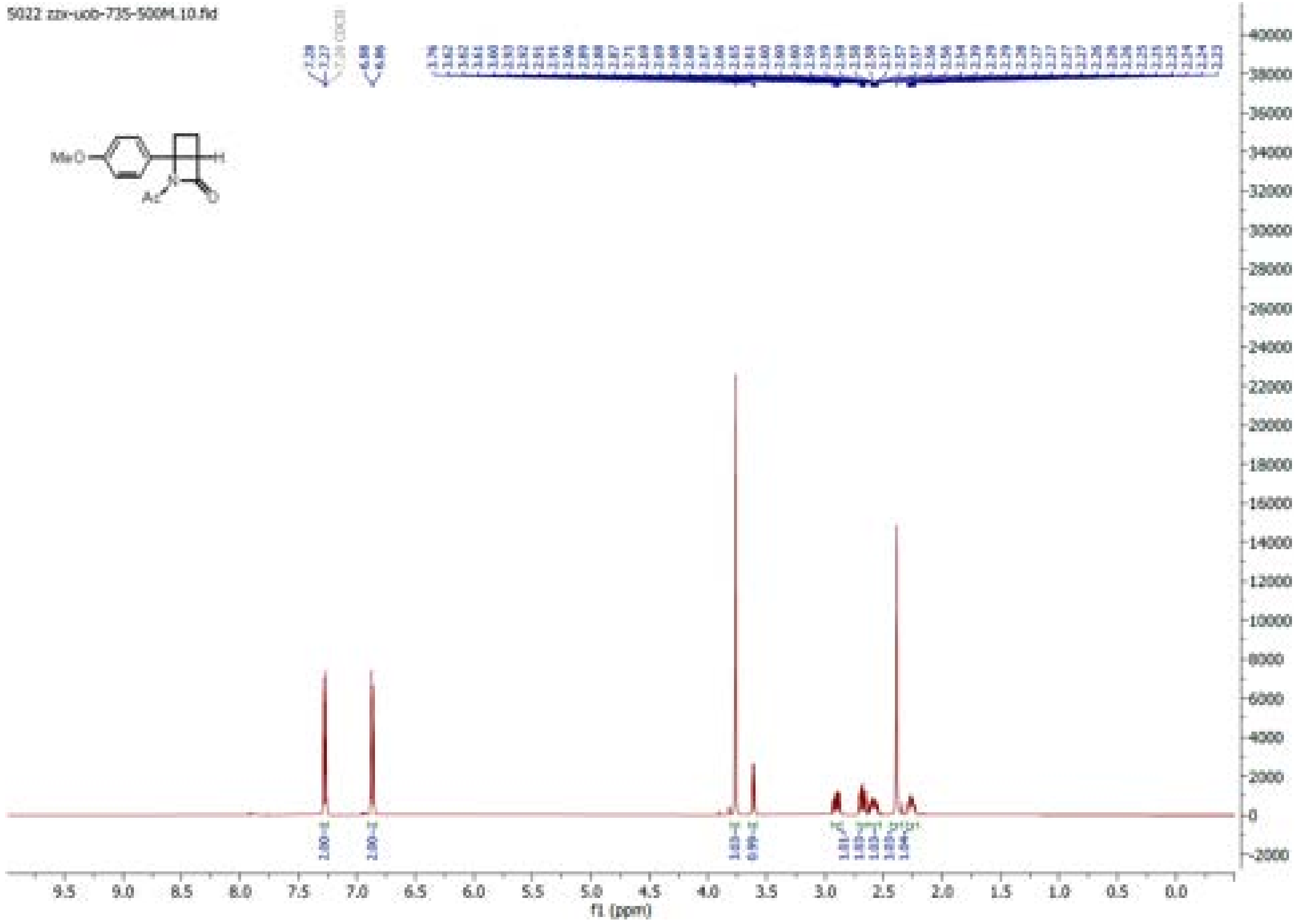

^{13}C NMR (126 MHz, $CDCl_3$) of **6ae**

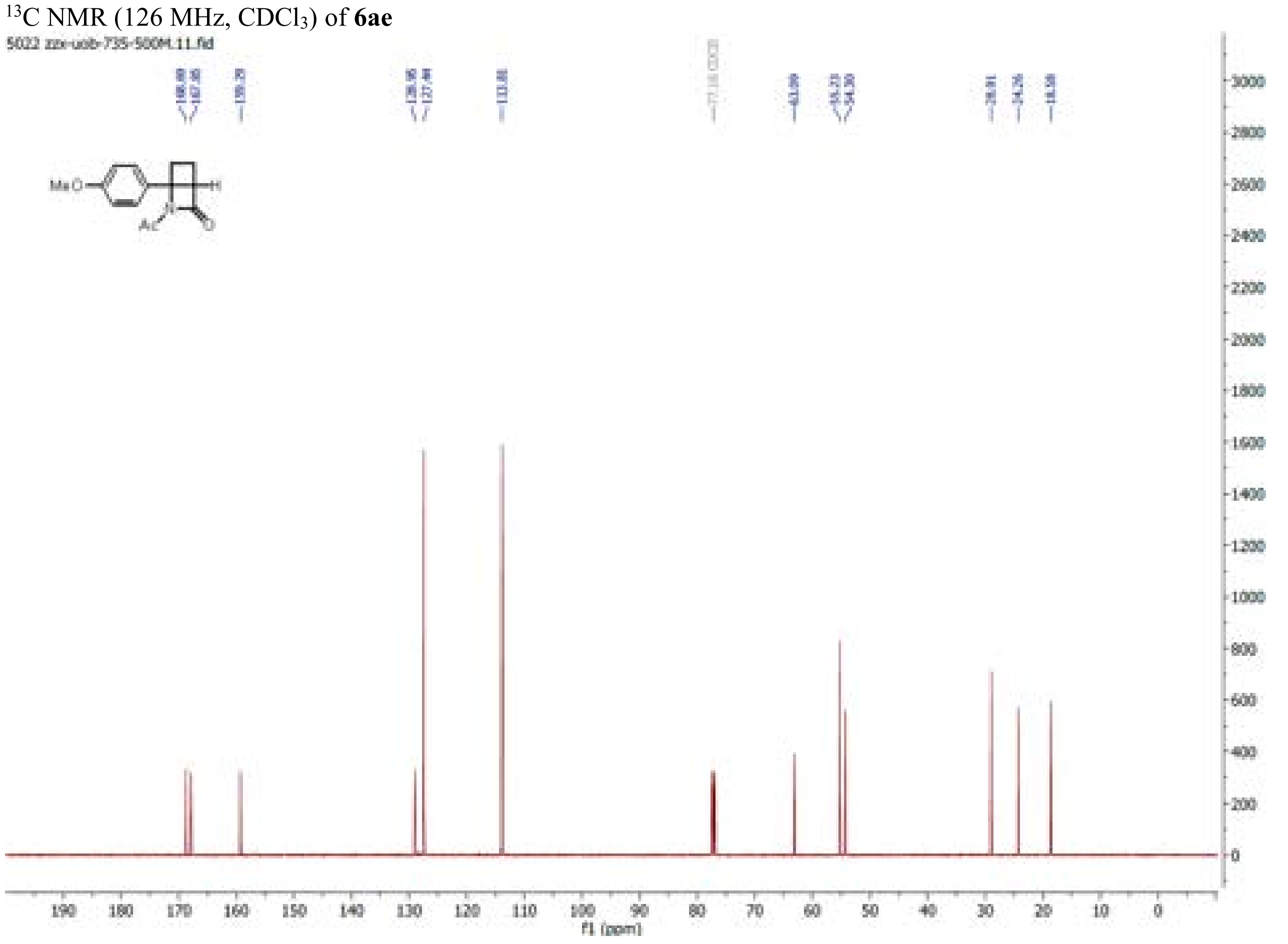

^{1}H NMR (500 MHz, $CDCl_3$) of **5ae**

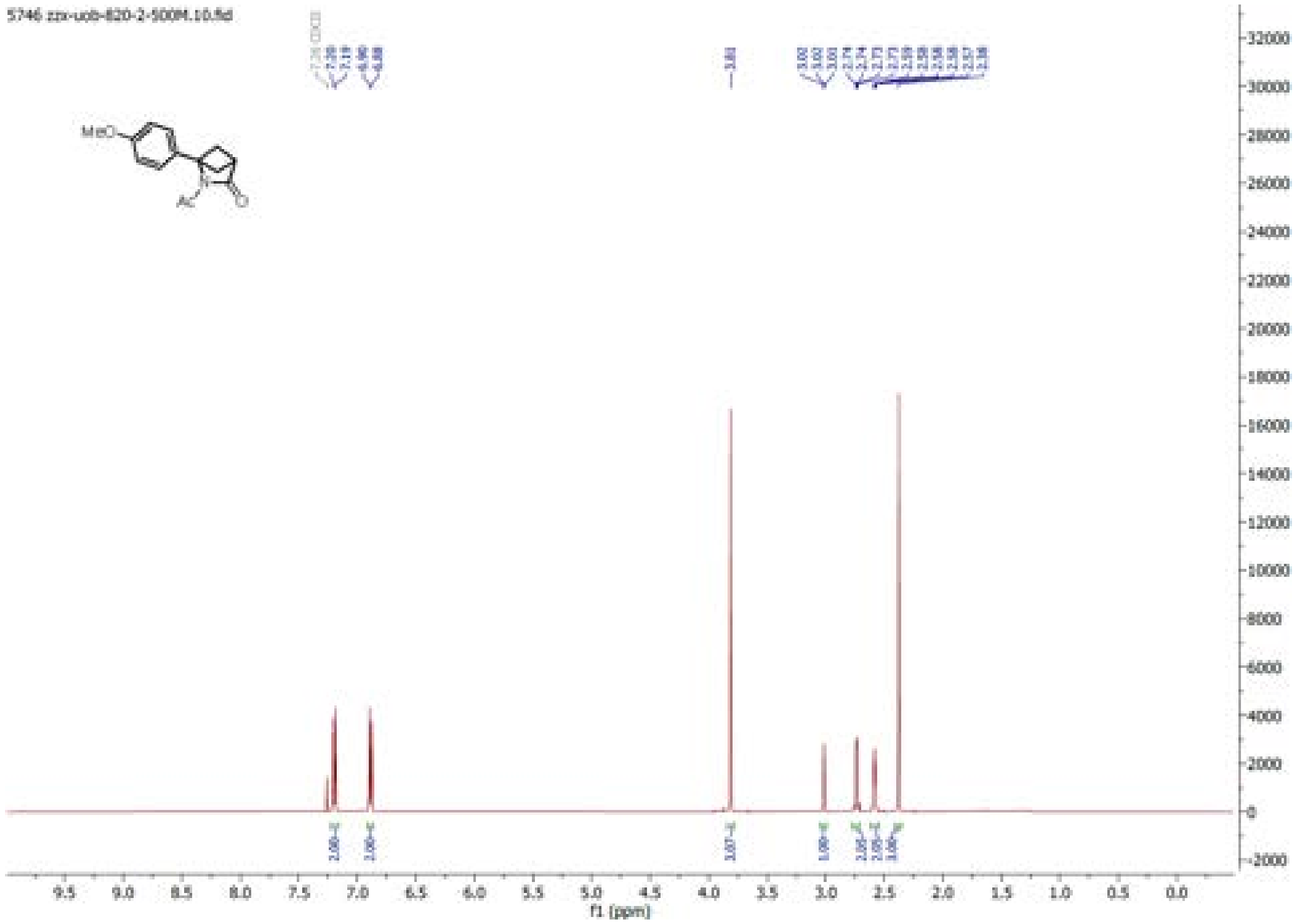

^{13}C NMR (126 MHz, $CDCl_3$) of **5ae**

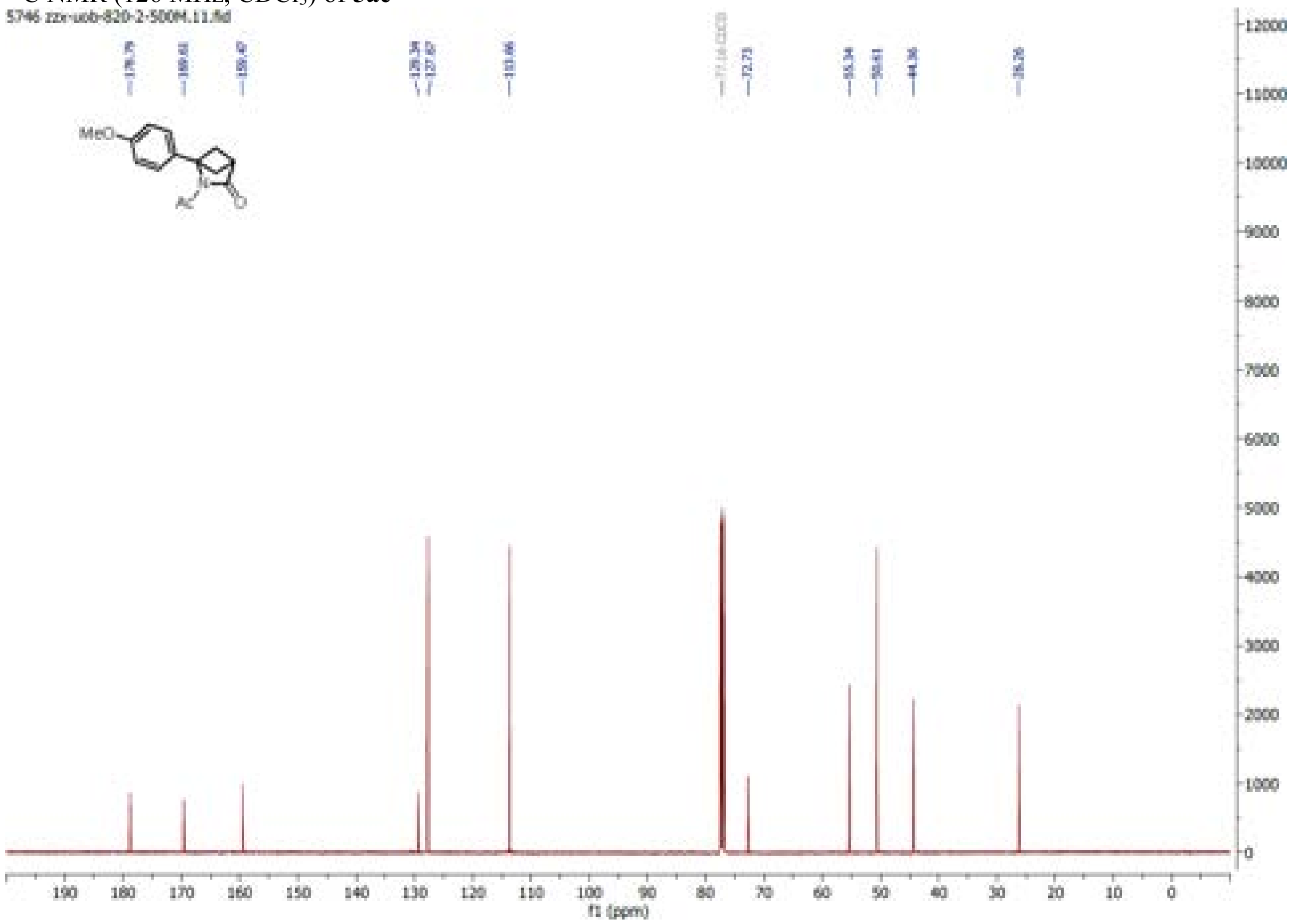

^{1}H NMR (500 MHz, $CDCl_3$) of **6af**

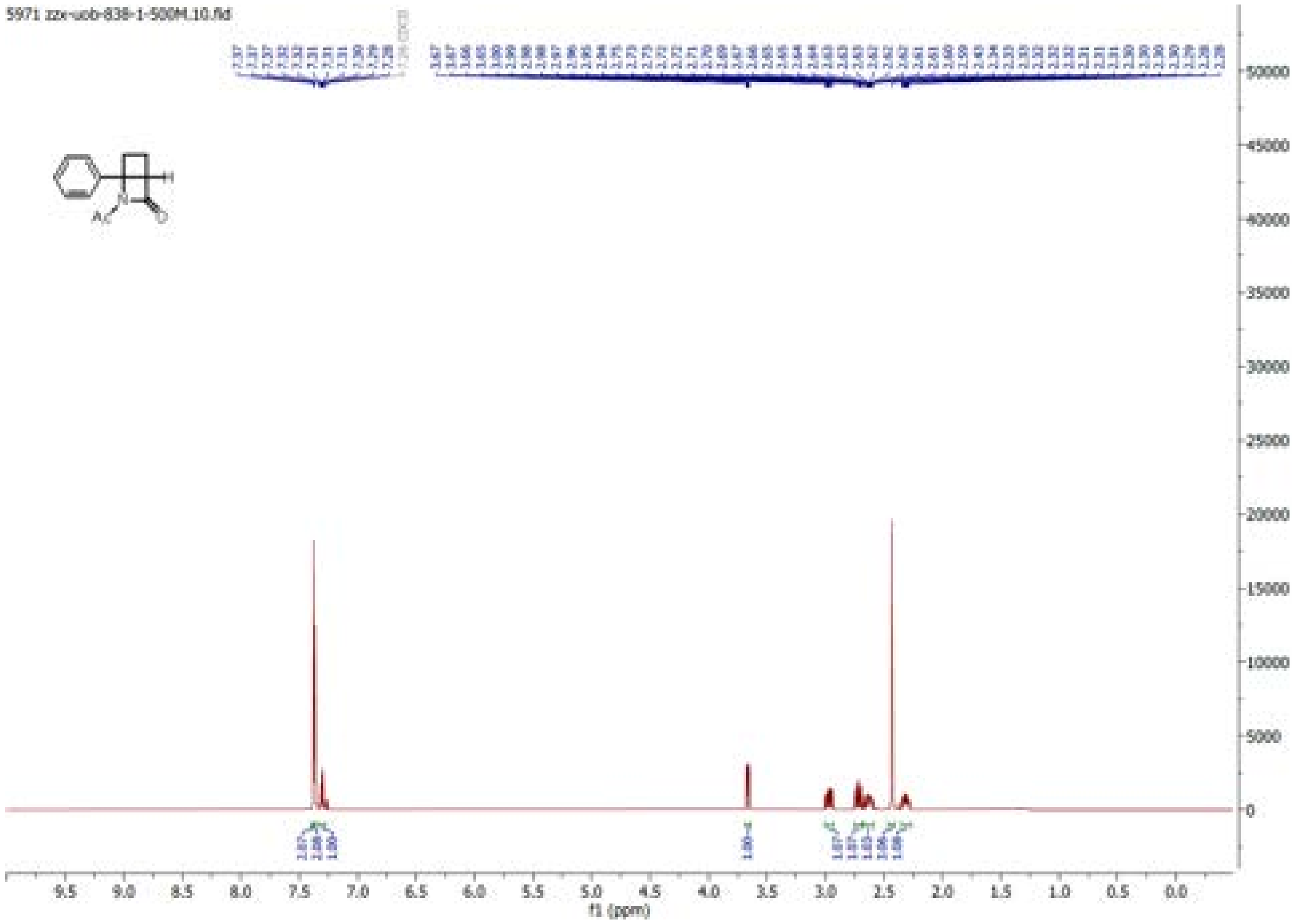

^{13}C NMR (126 MHz, $CDCl_3$) of **6af**

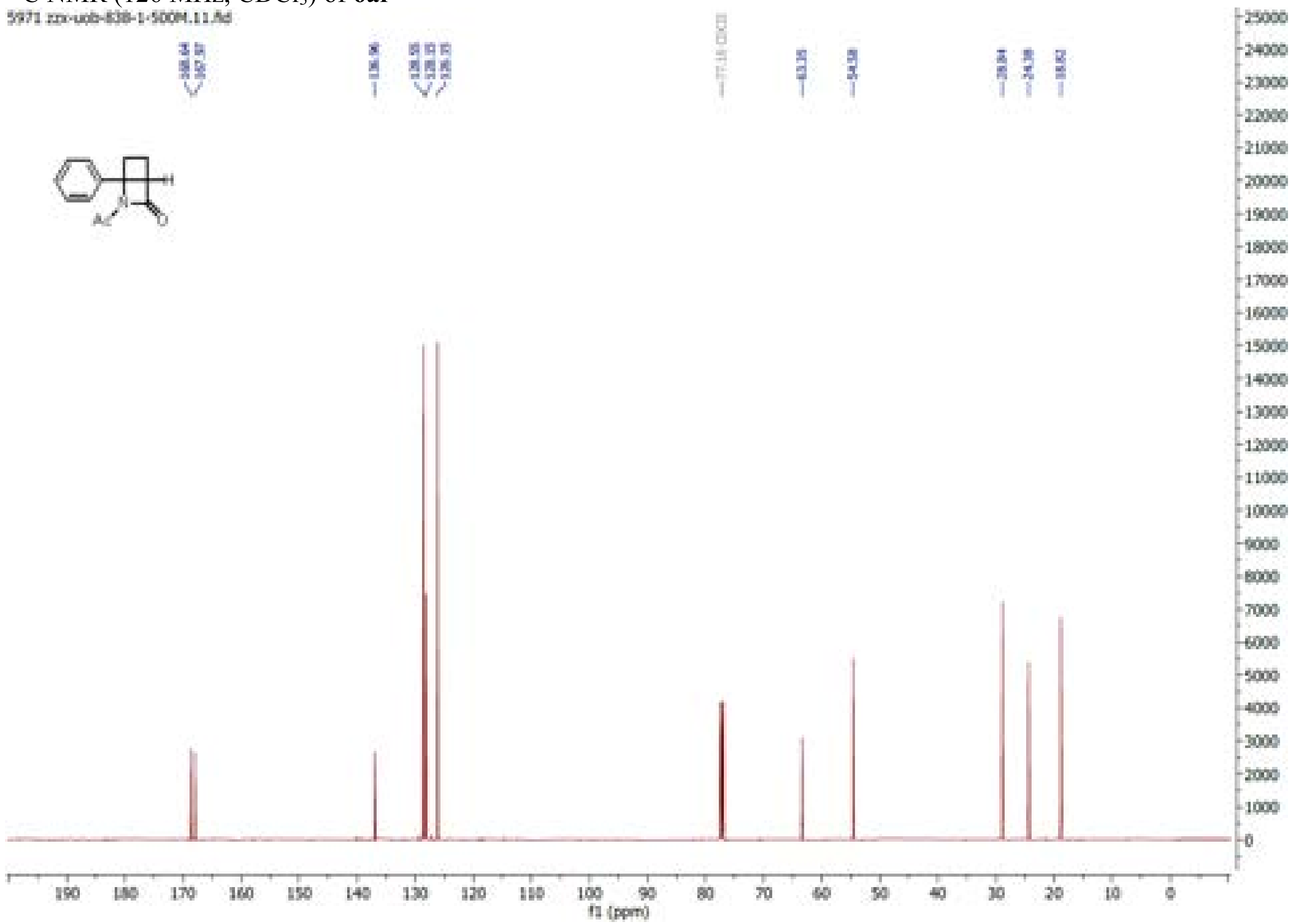

^1H NMR (500 MHz, $CDCl_3$) of **5af**

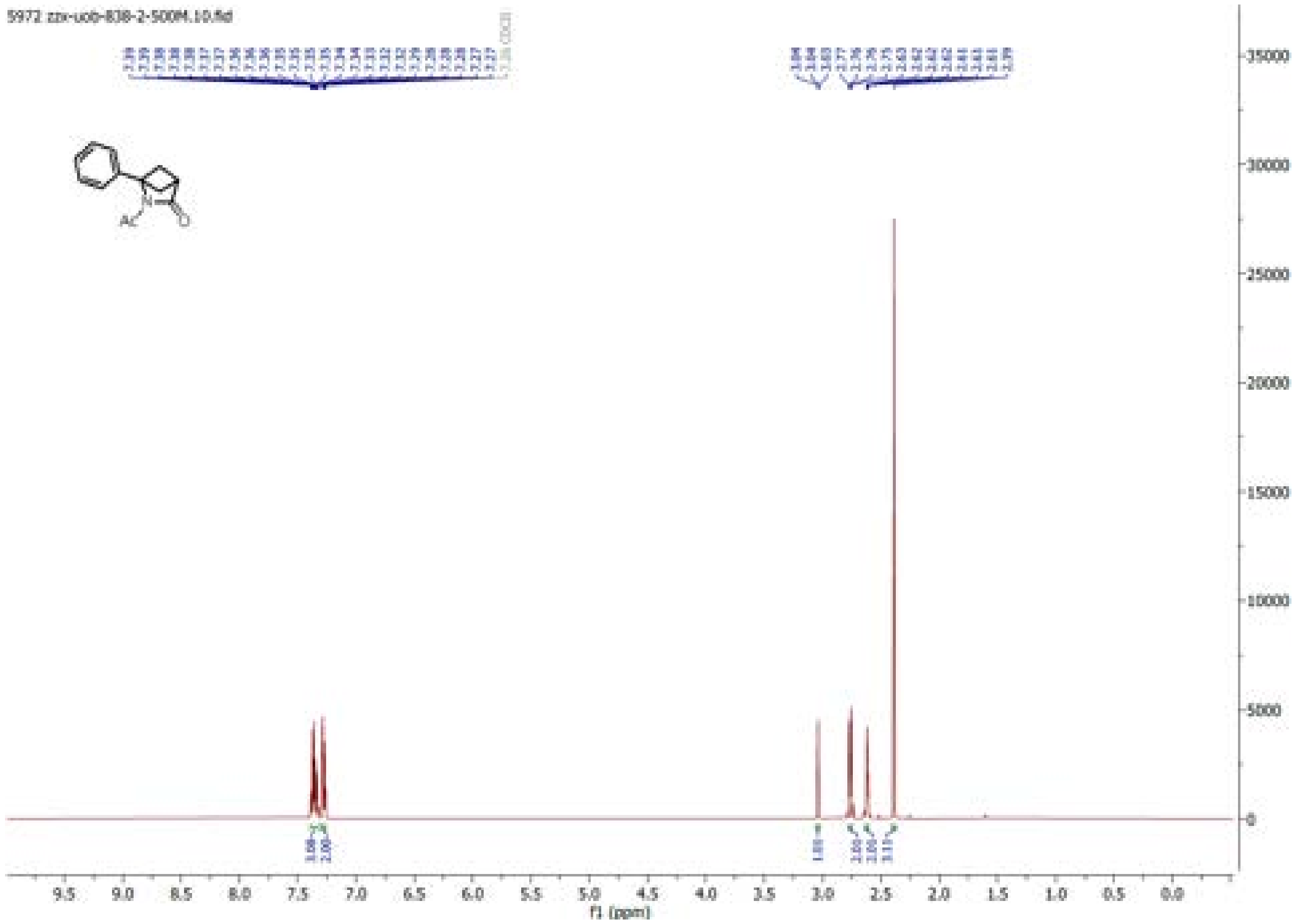

^{13}C NMR (126 MHz, $CDCl_3$) of **5af**

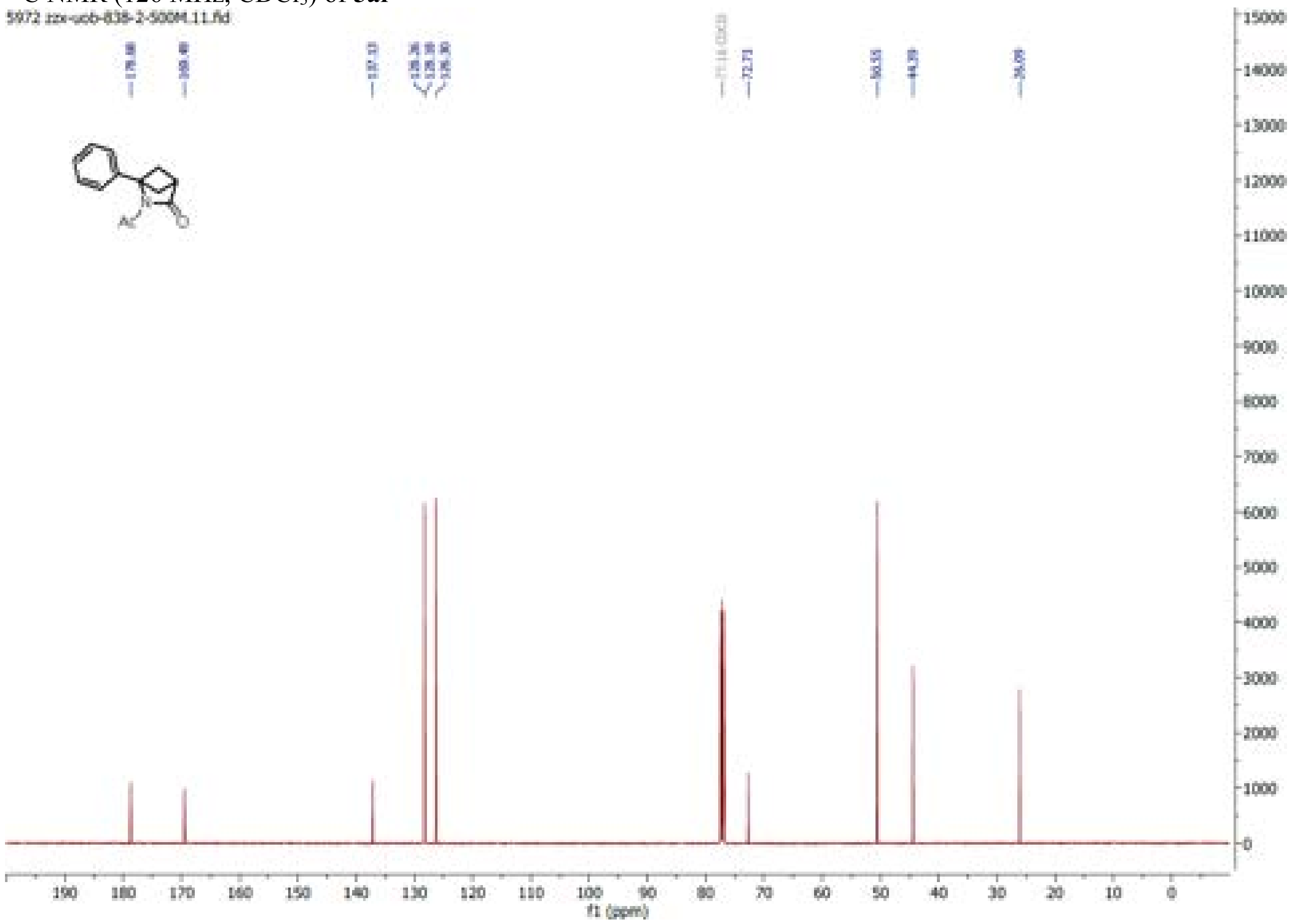

^{1}H NMR (500 MHz, $CDCl_3$) of **6ag**

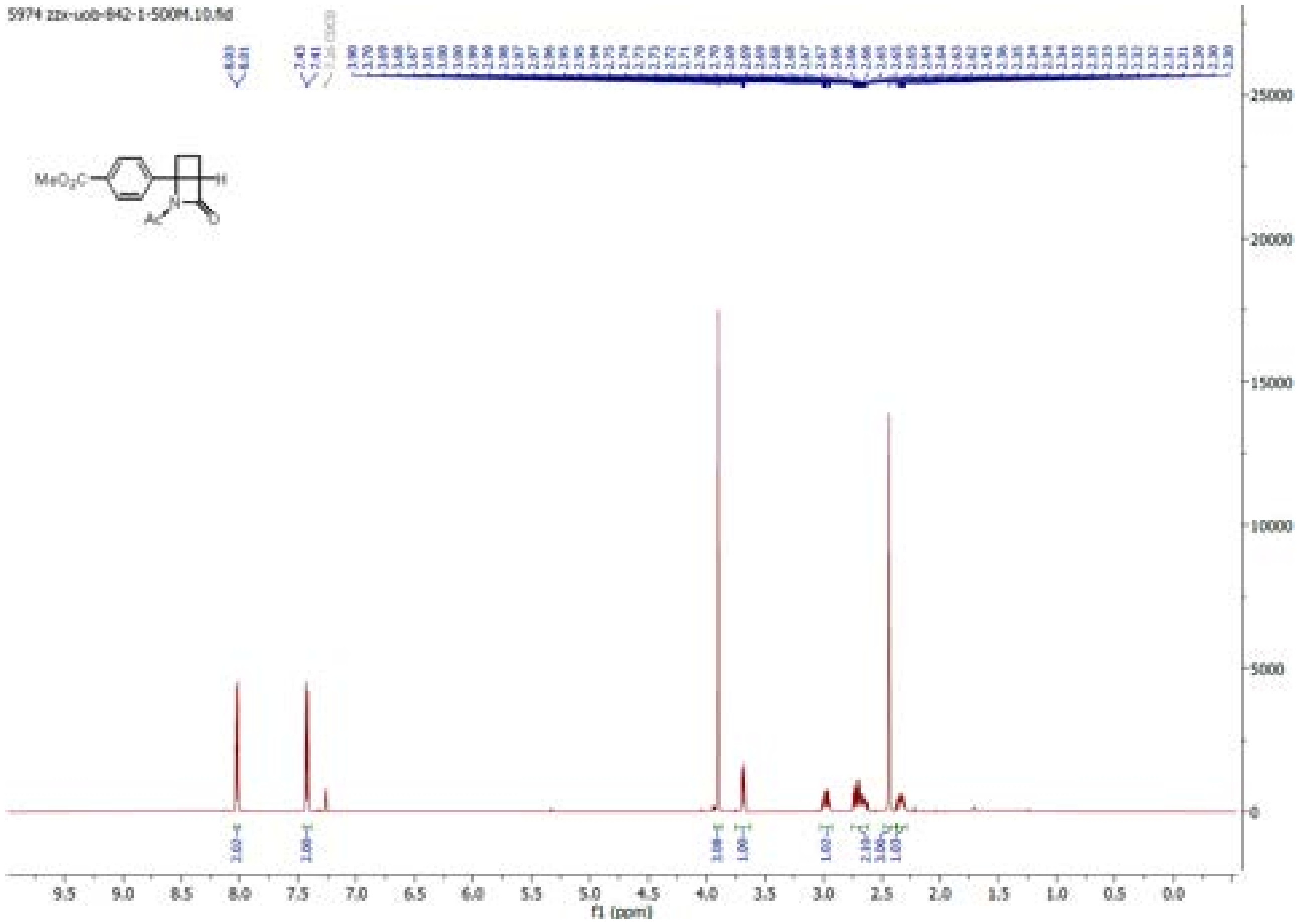

^{13}C NMR (126 MHz, $CDCl_3$) of **6ag**

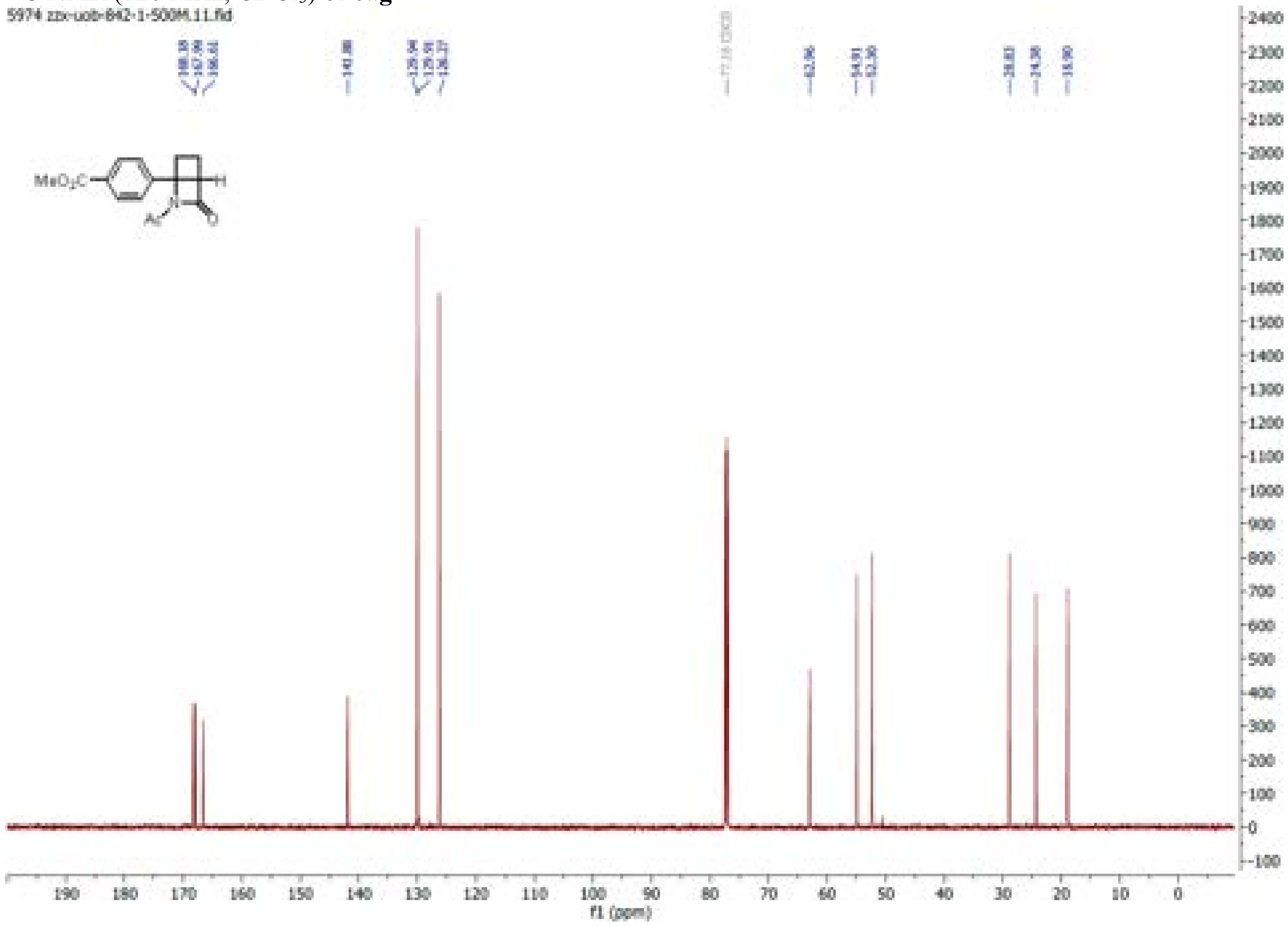

^{1}H NMR (500 MHz, $CDCl_3$) of **5ag**

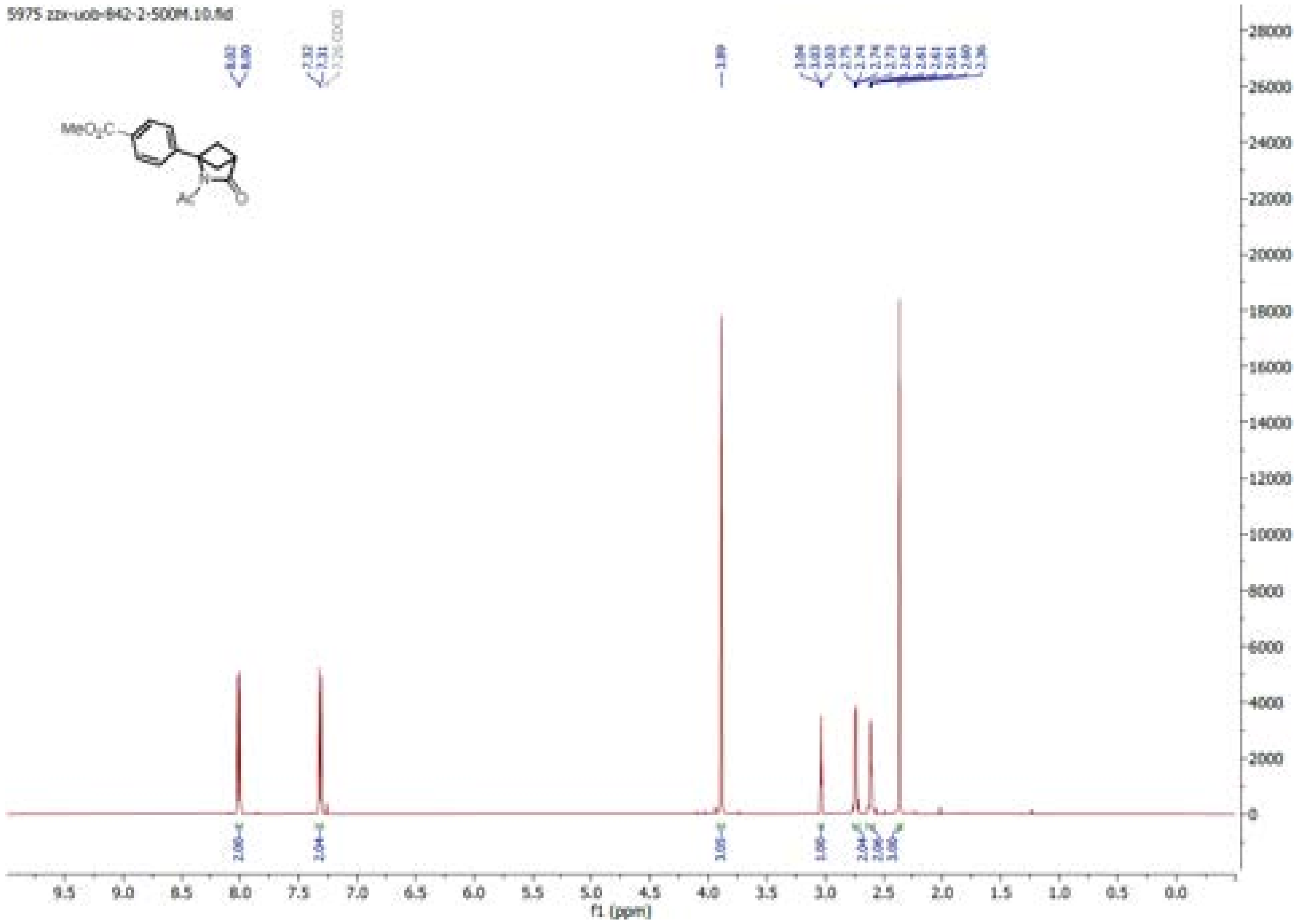

^{13}C NMR (126 MHz, $CDCl_3$) of **5ag**

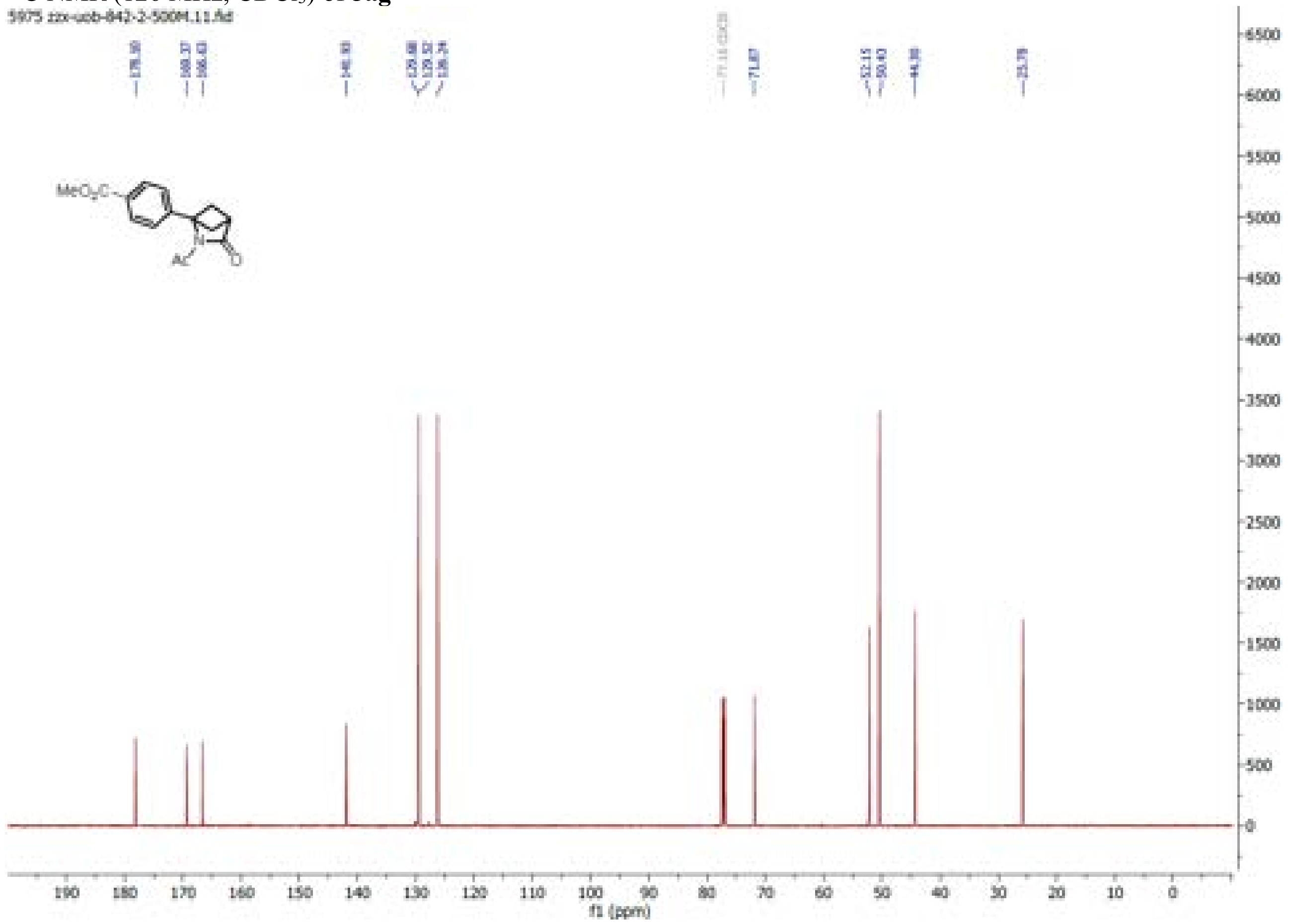

^{1}H NMR (500 MHz, $CDCl_3$) of **6ah**

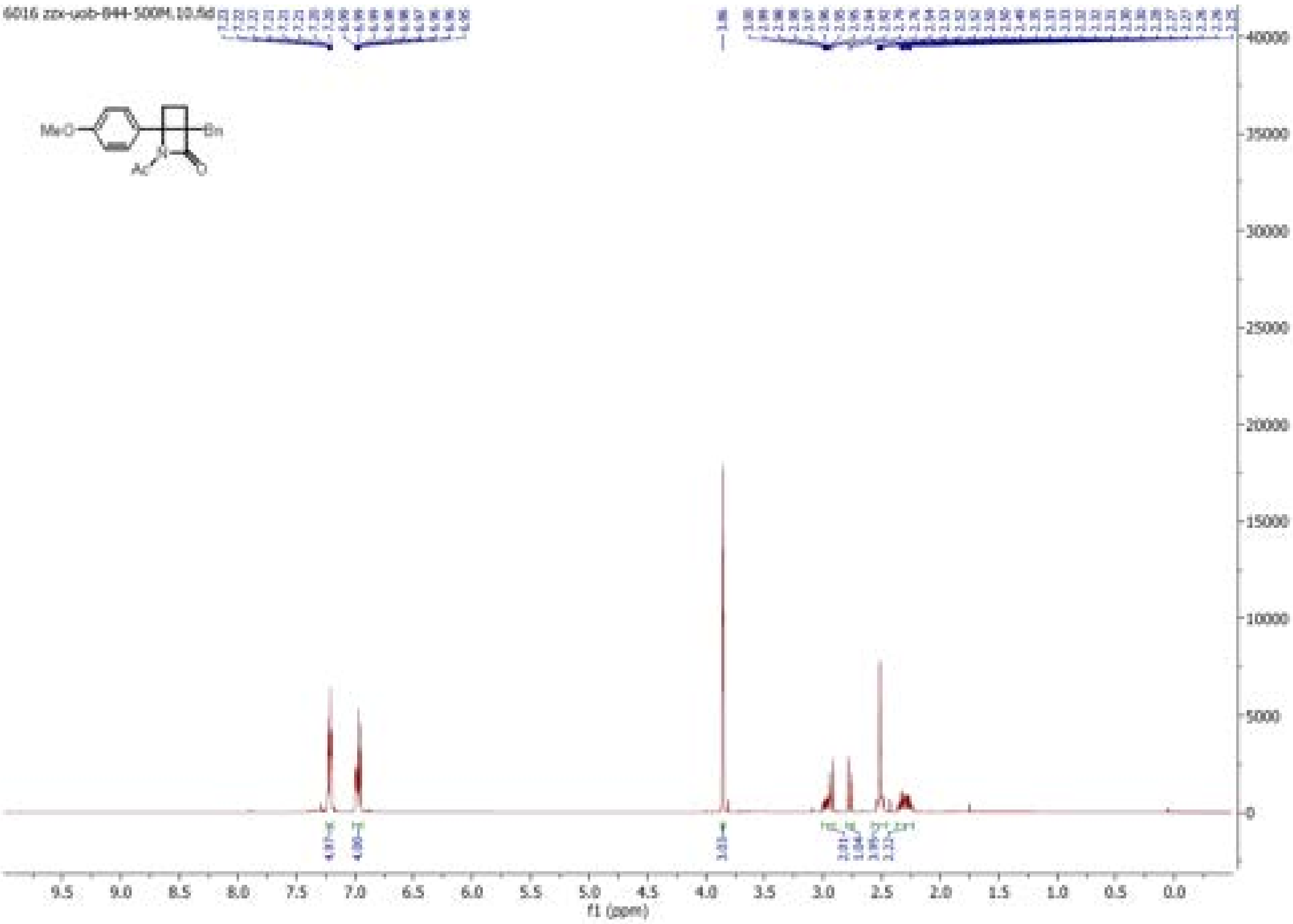

^{13}C NMR (126 MHz, $CDCl_3$) of **6ah**

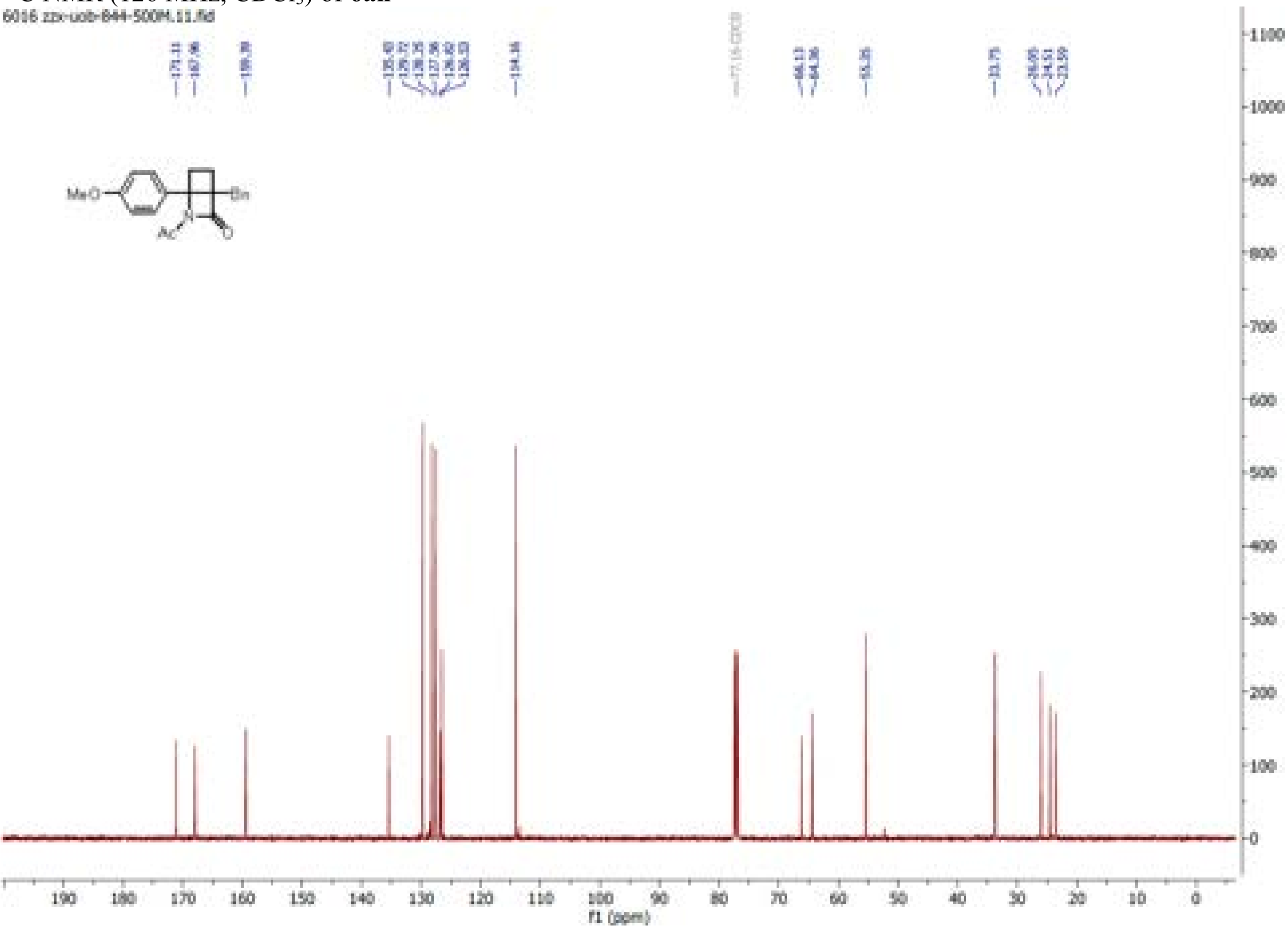

^{1}H NMR (500 MHz, $CDCl_3$) of **6ai**

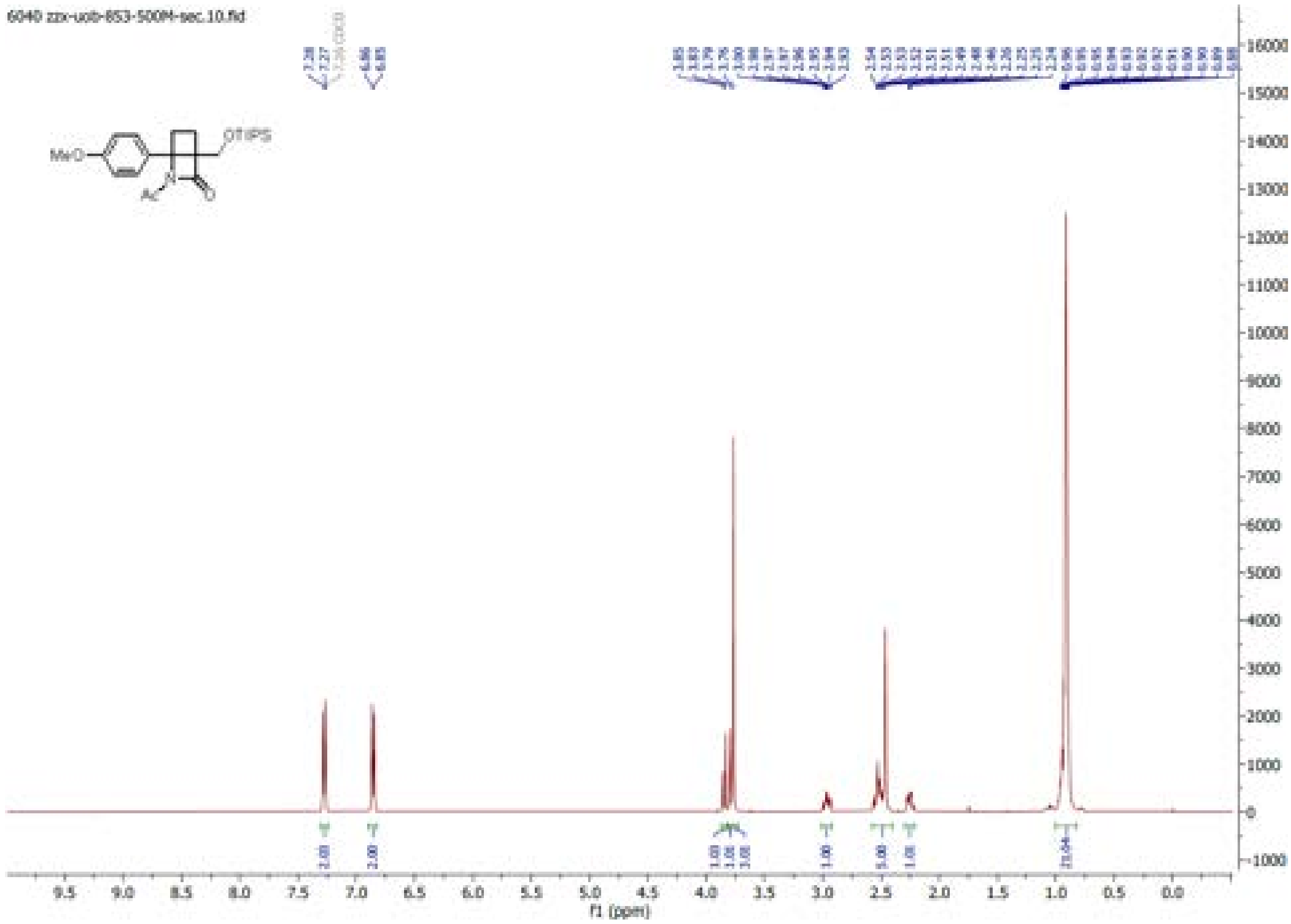

^{13}C NMR (126 MHz, $CDCl_3$) of **6ai**

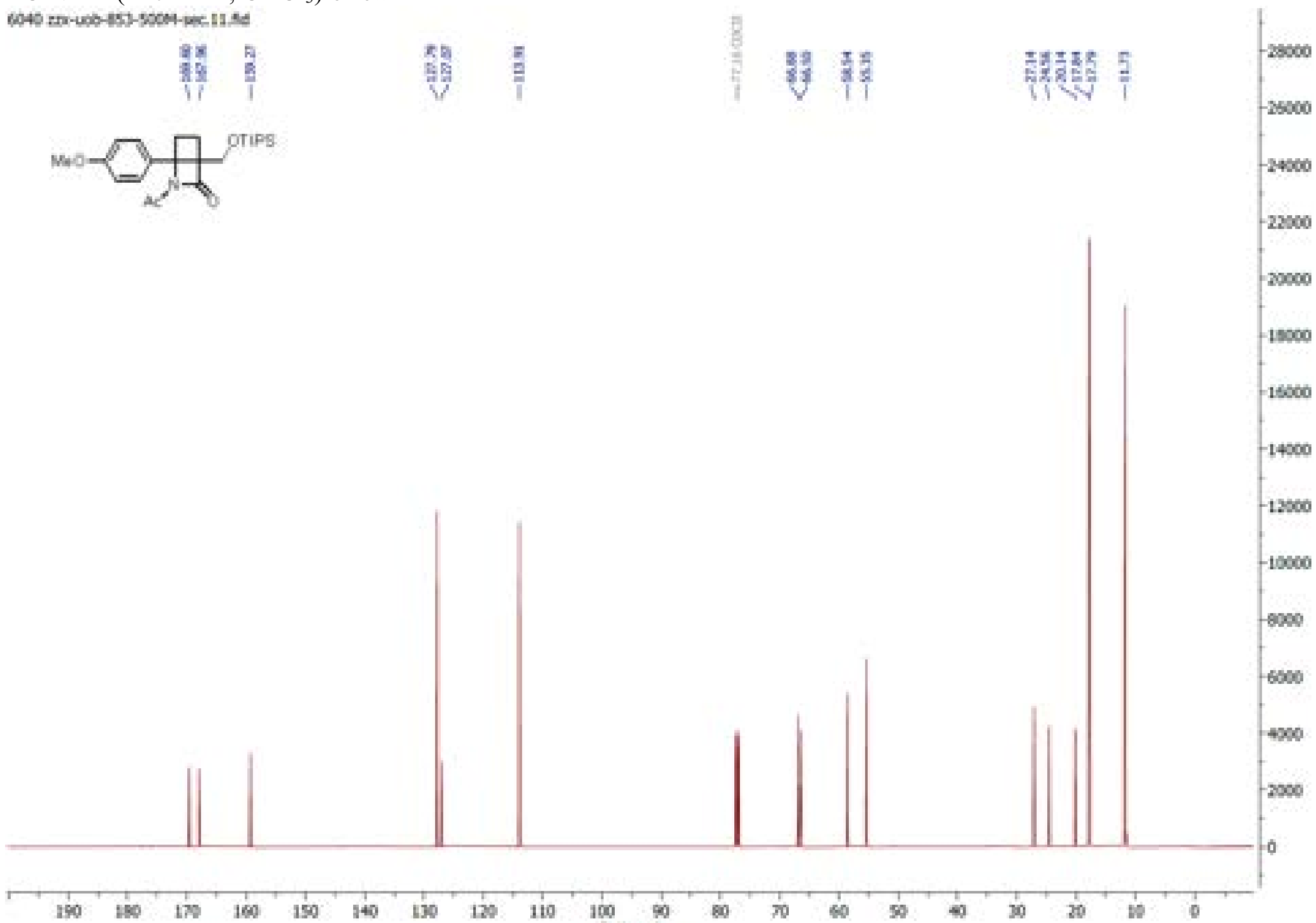

^{1}H NMR (500 MHz, $CDCl_3$) of **6aj**

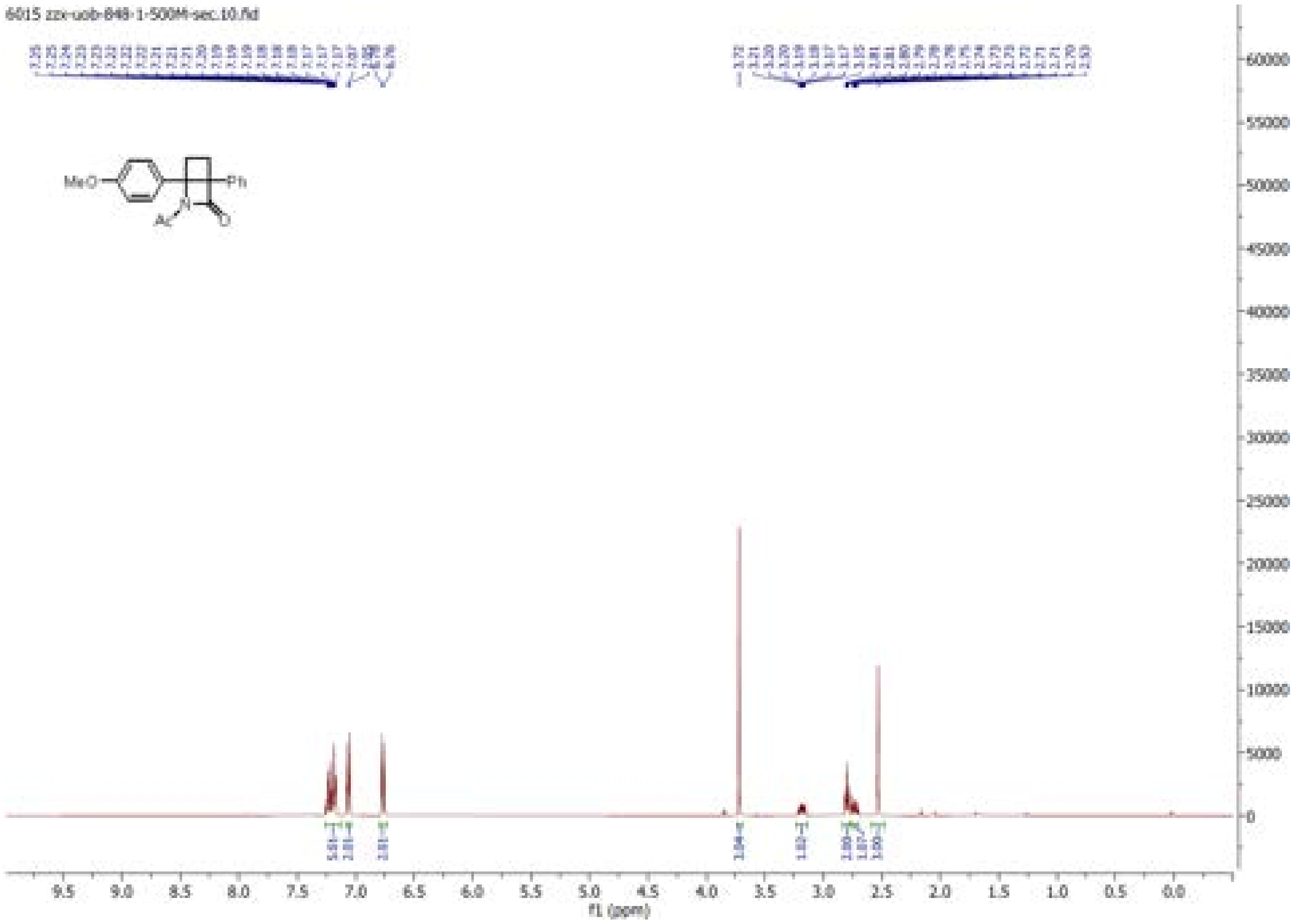

^{13}C NMR (126 MHz, $CDCl_3$) of **6aj**

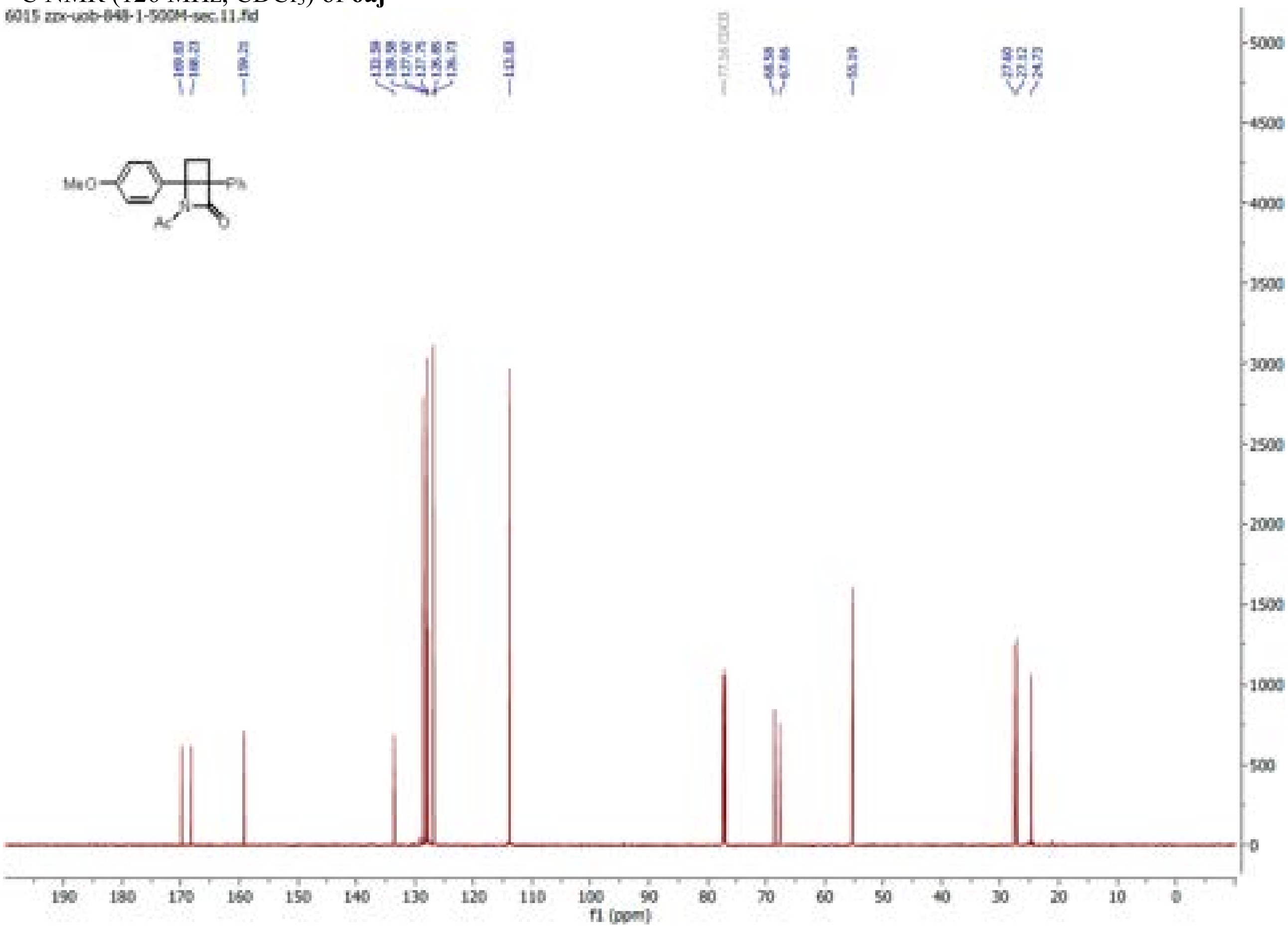

^{1}H NMR (500 MHz, $CDCl_3$) of **5aj**

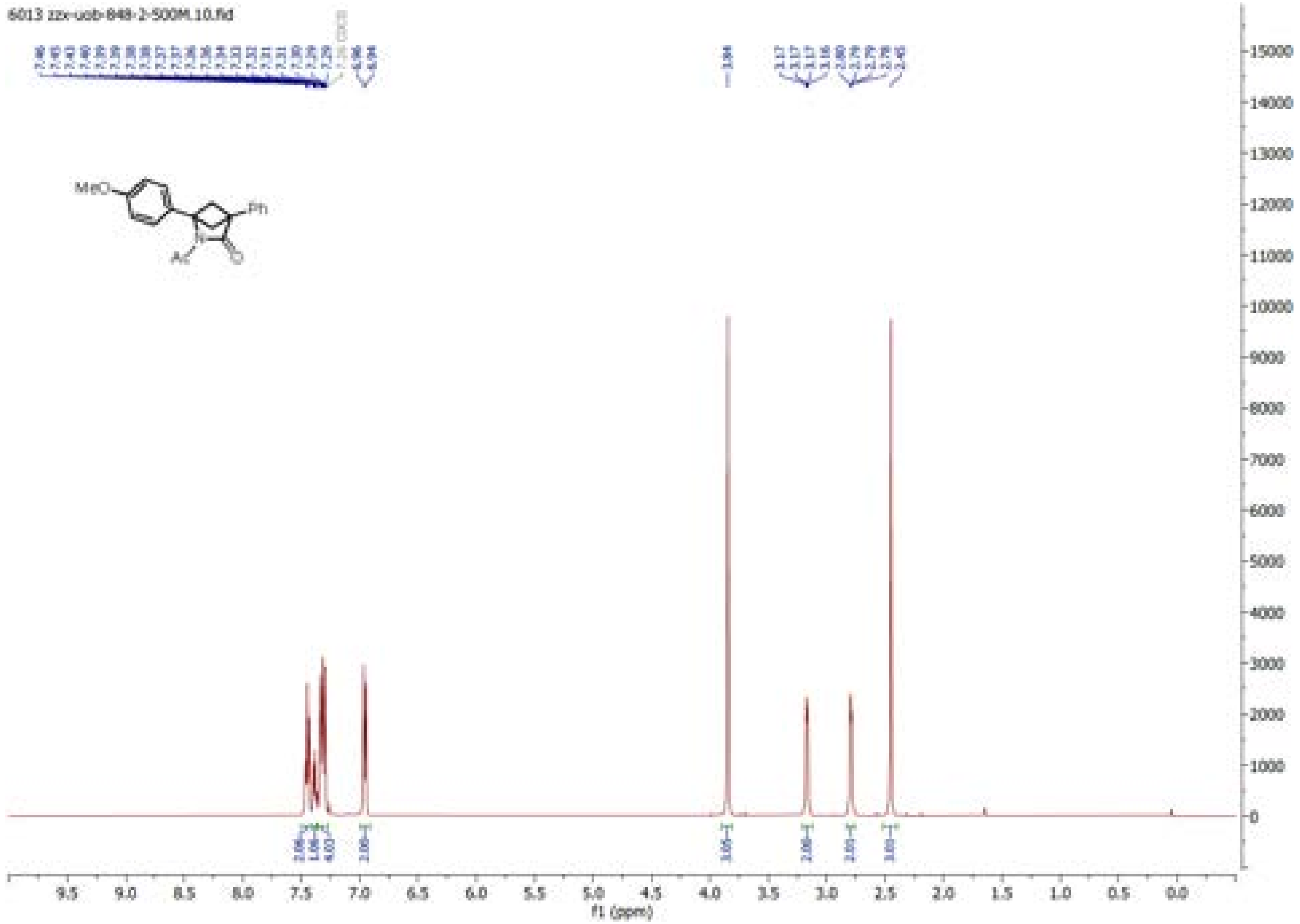

^{13}C NMR (126 MHz, $CDCl_3$) of **5aj**

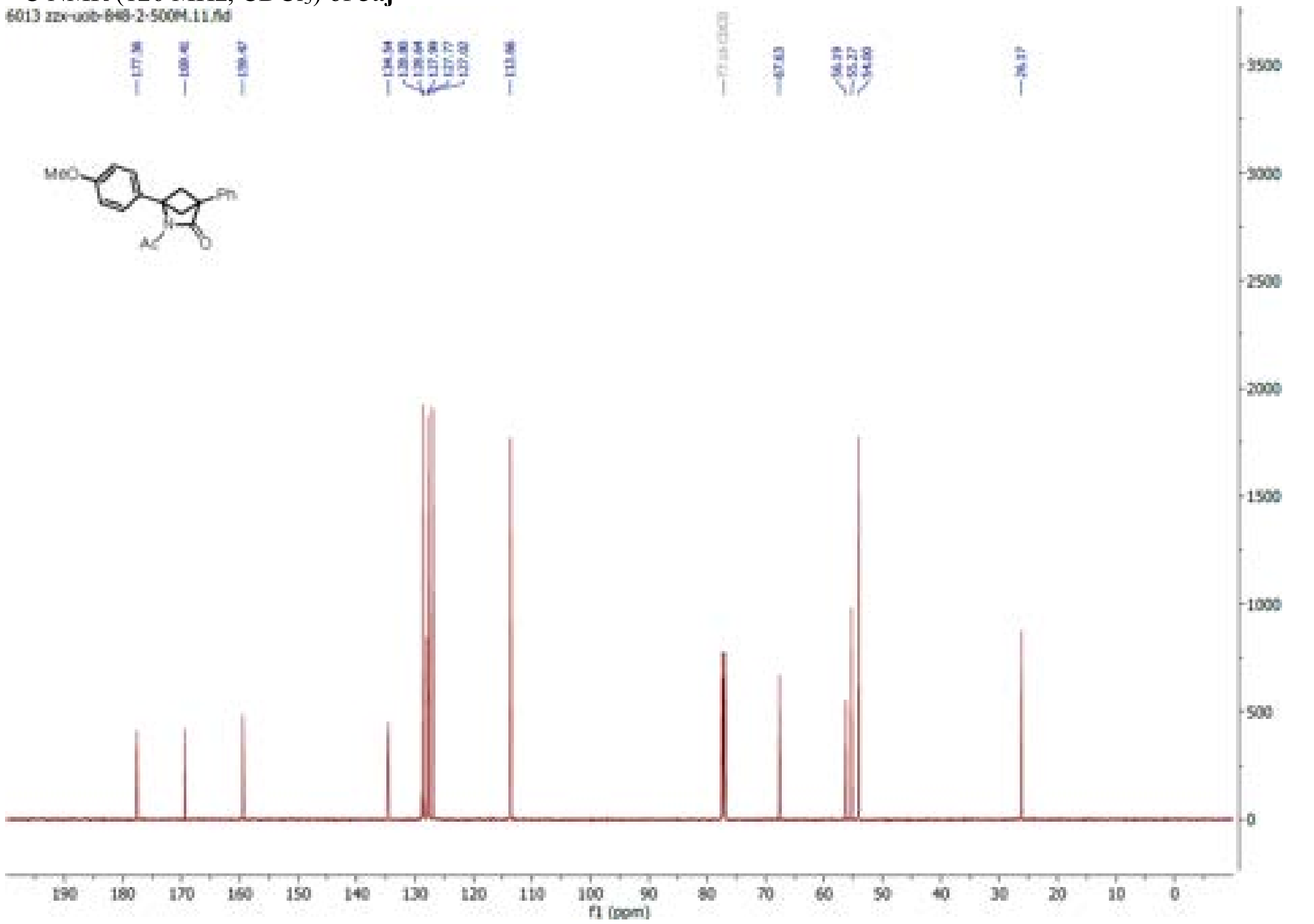

^{1}H NMR (500 MHz, $CDCl_3$) of **6ak**

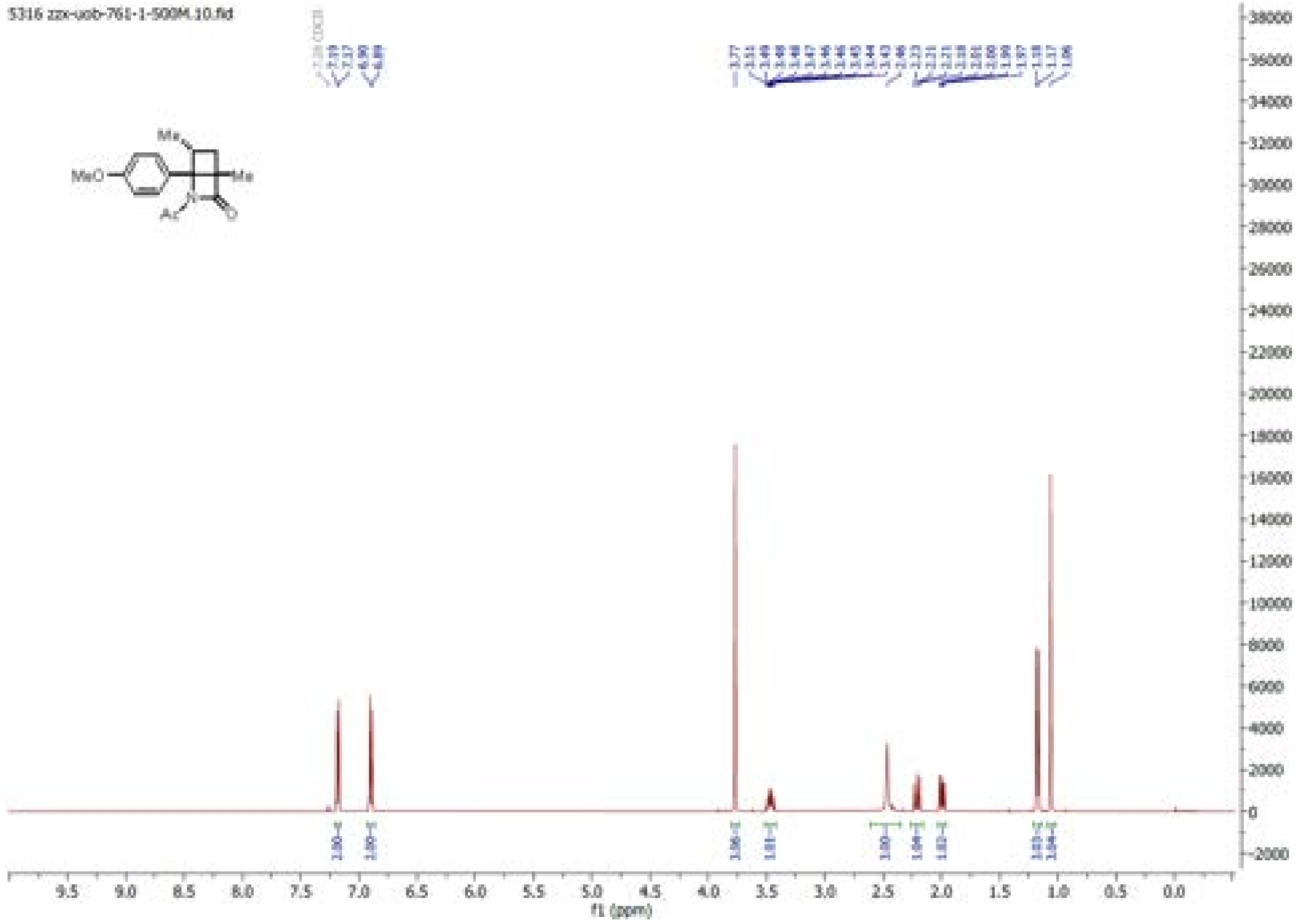

^{13}C NMR (126 MHz, $CDCl_3$) of **6ak**

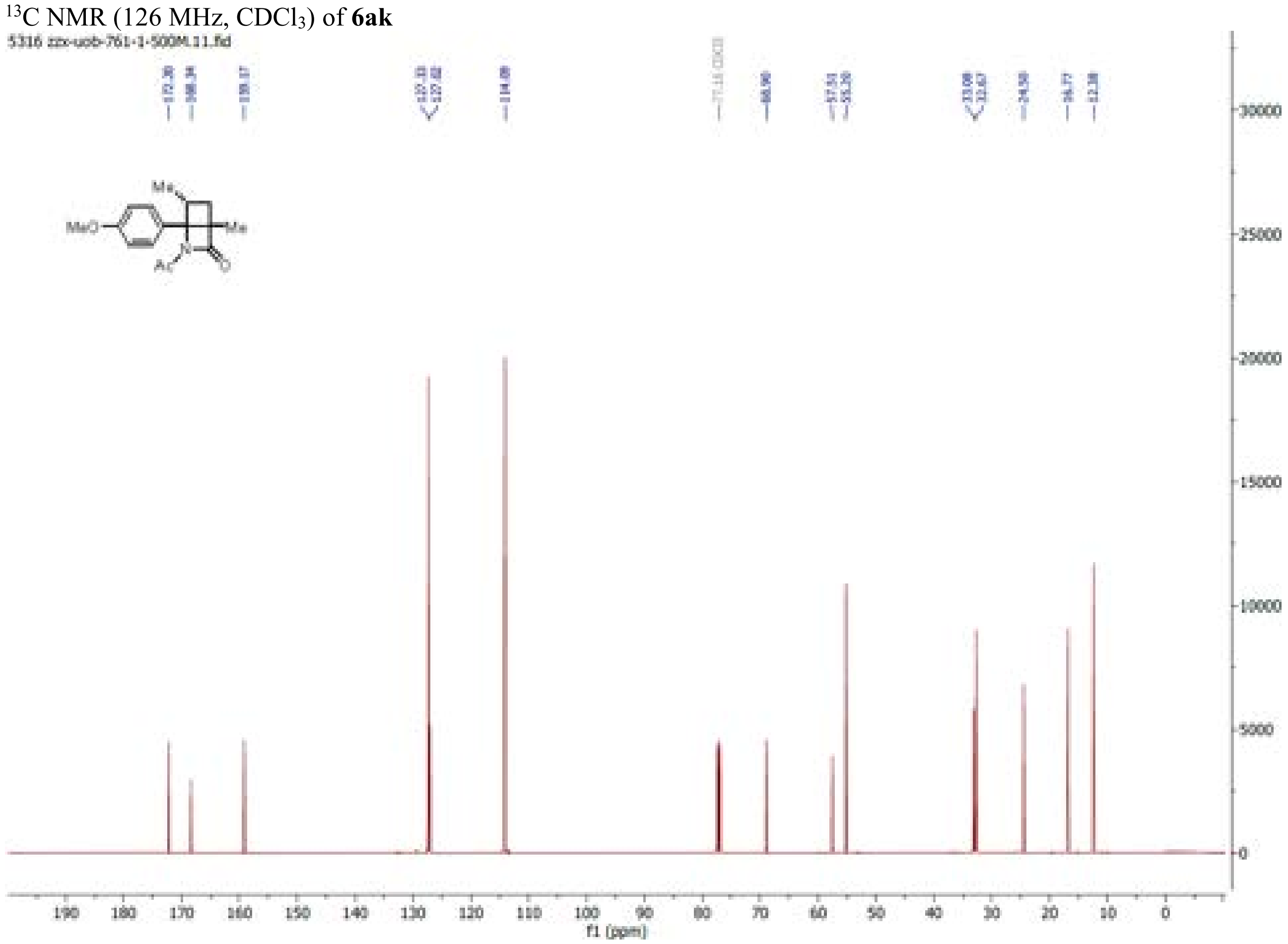

^{1}H NMR (500 MHz, $CDCl_3$) of **6al**

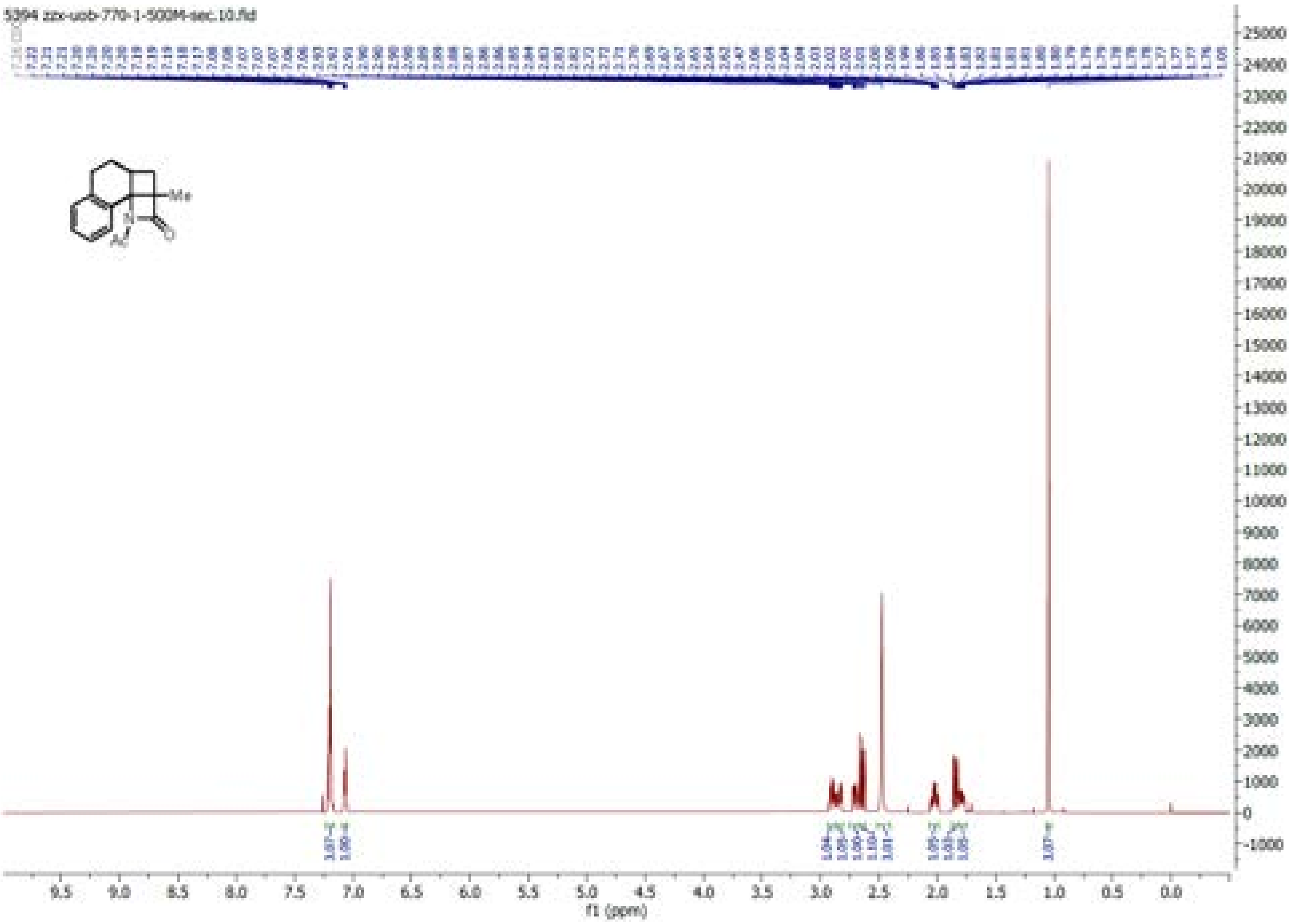

^{13}C NMR (126 MHz, $CDCl_3$) of **6al**

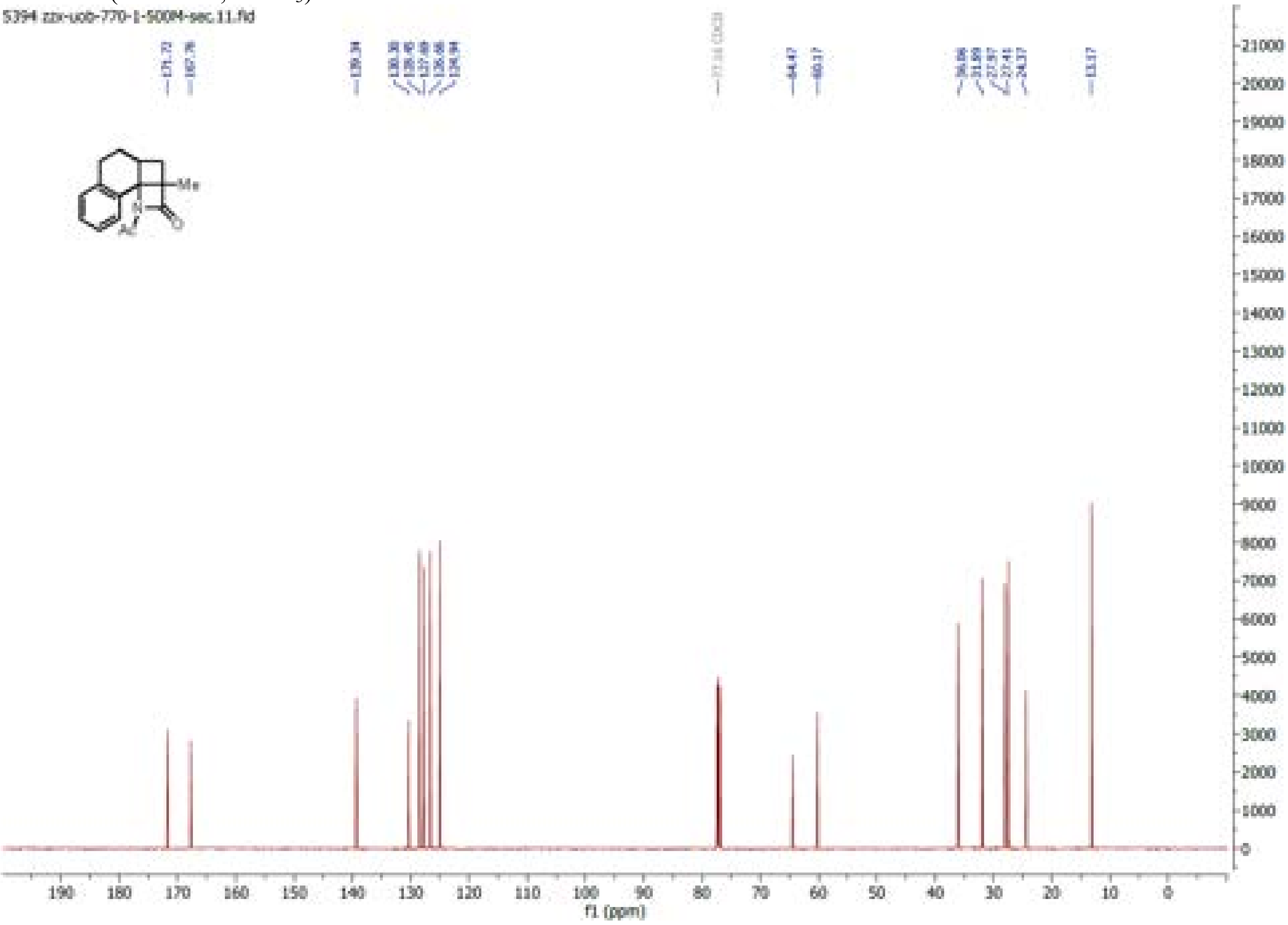

^{1}H NMR (500 MHz, $CDCl_3$) of **5al**

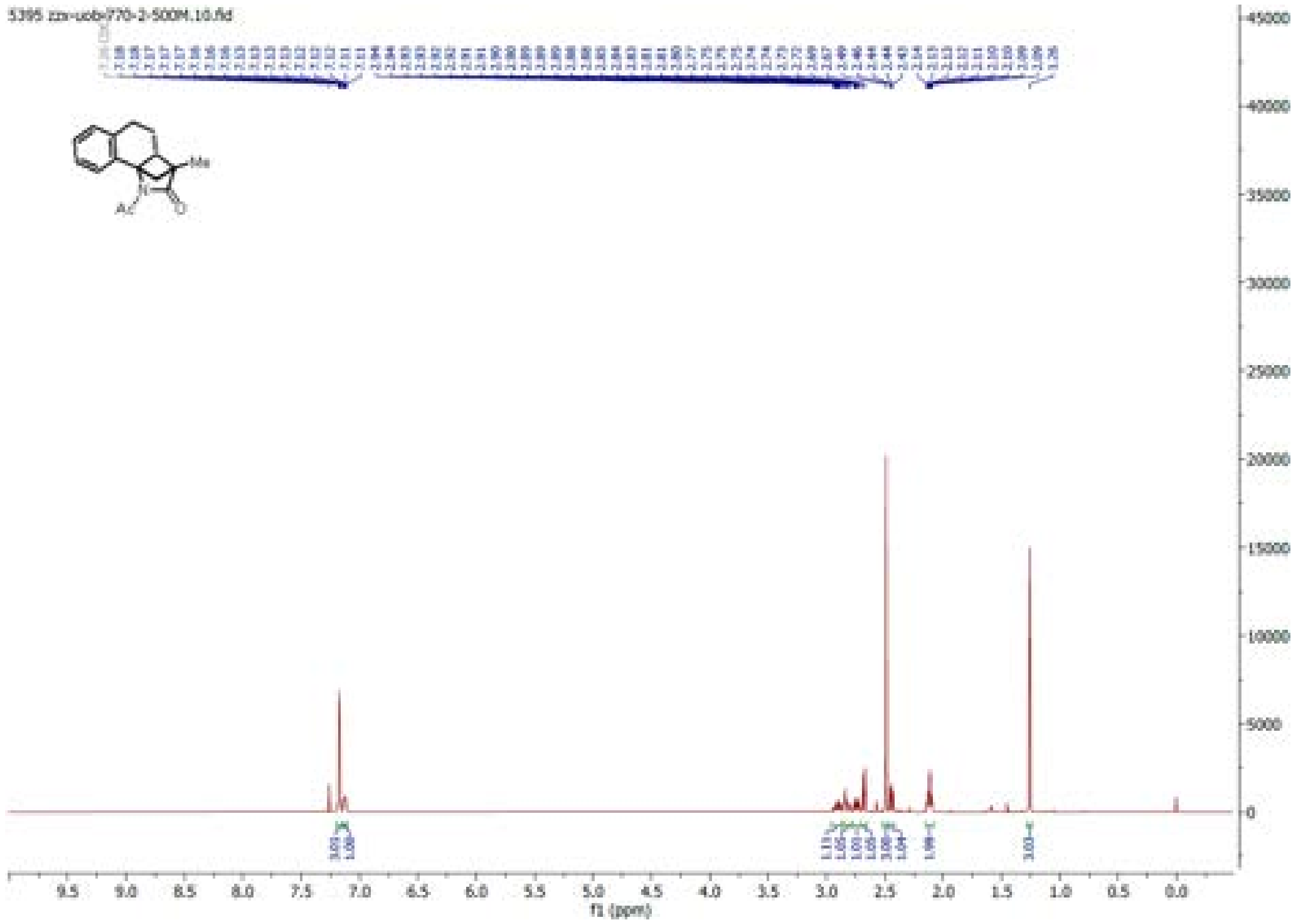

^{13}C NMR (126 MHz, $CDCl_3$) of **5al**

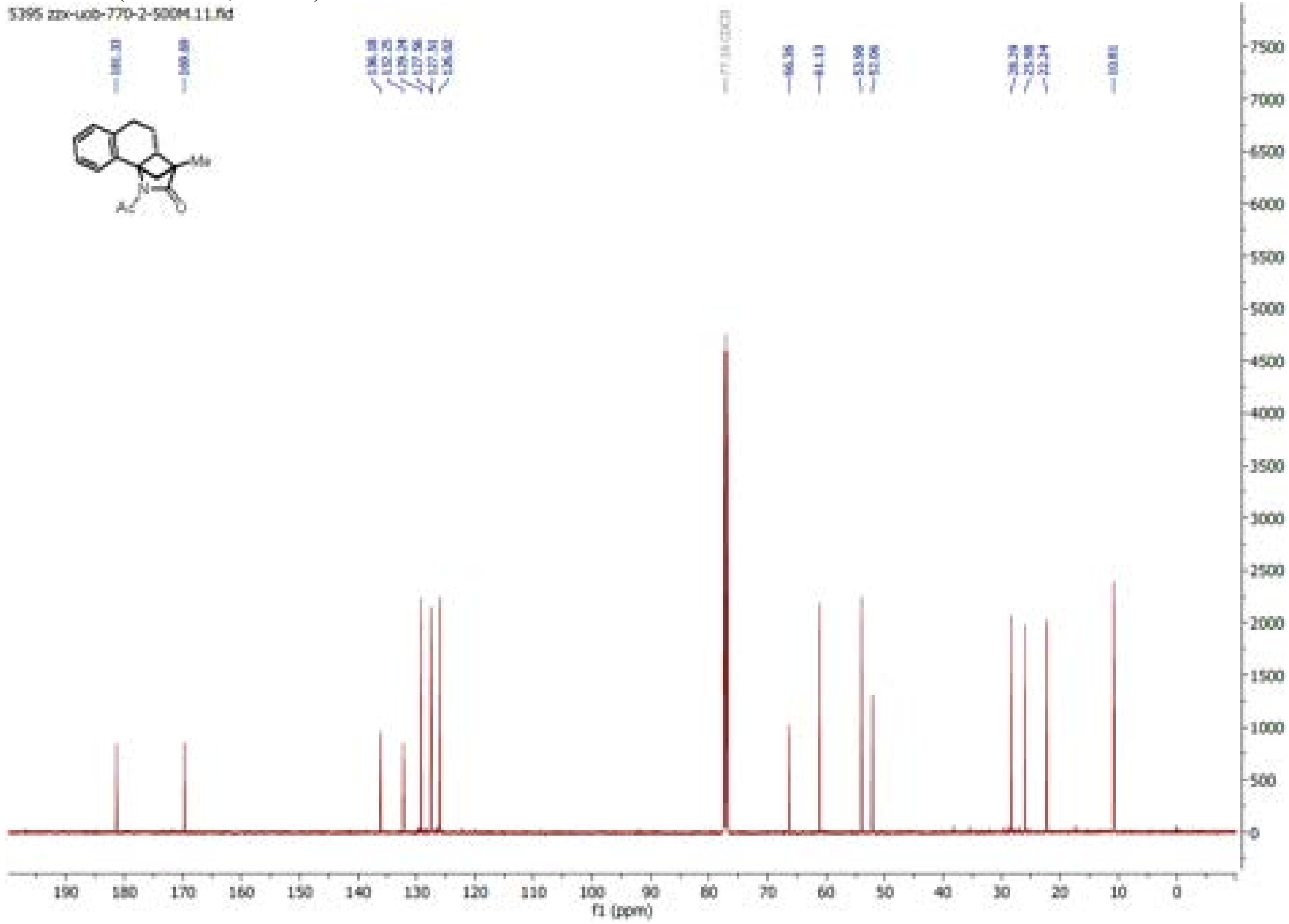

^{1}H NMR (500 MHz, $CDCl_3$) of **6am**

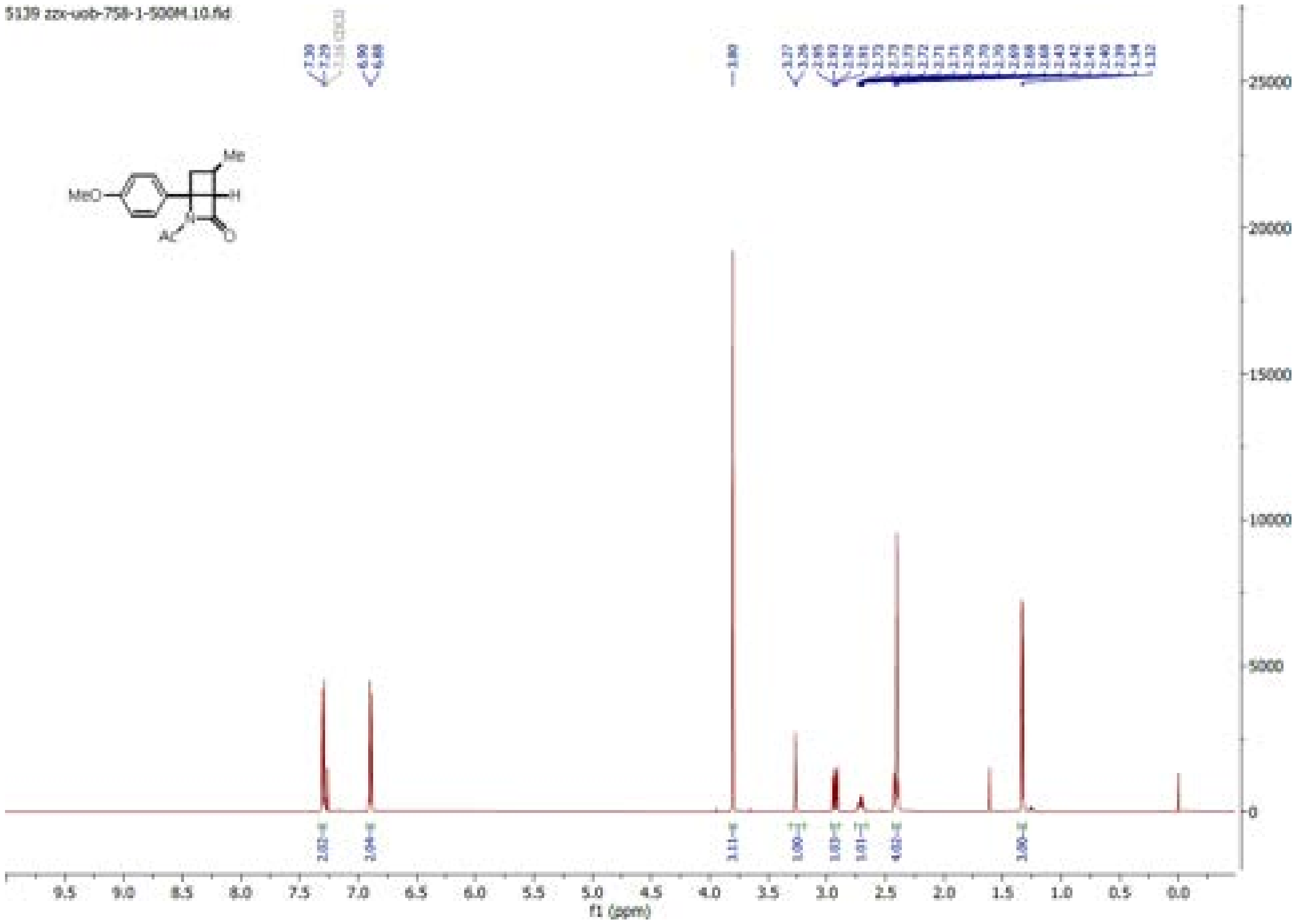

^{13}C NMR (126 MHz, $CDCl_3$) of **6am**

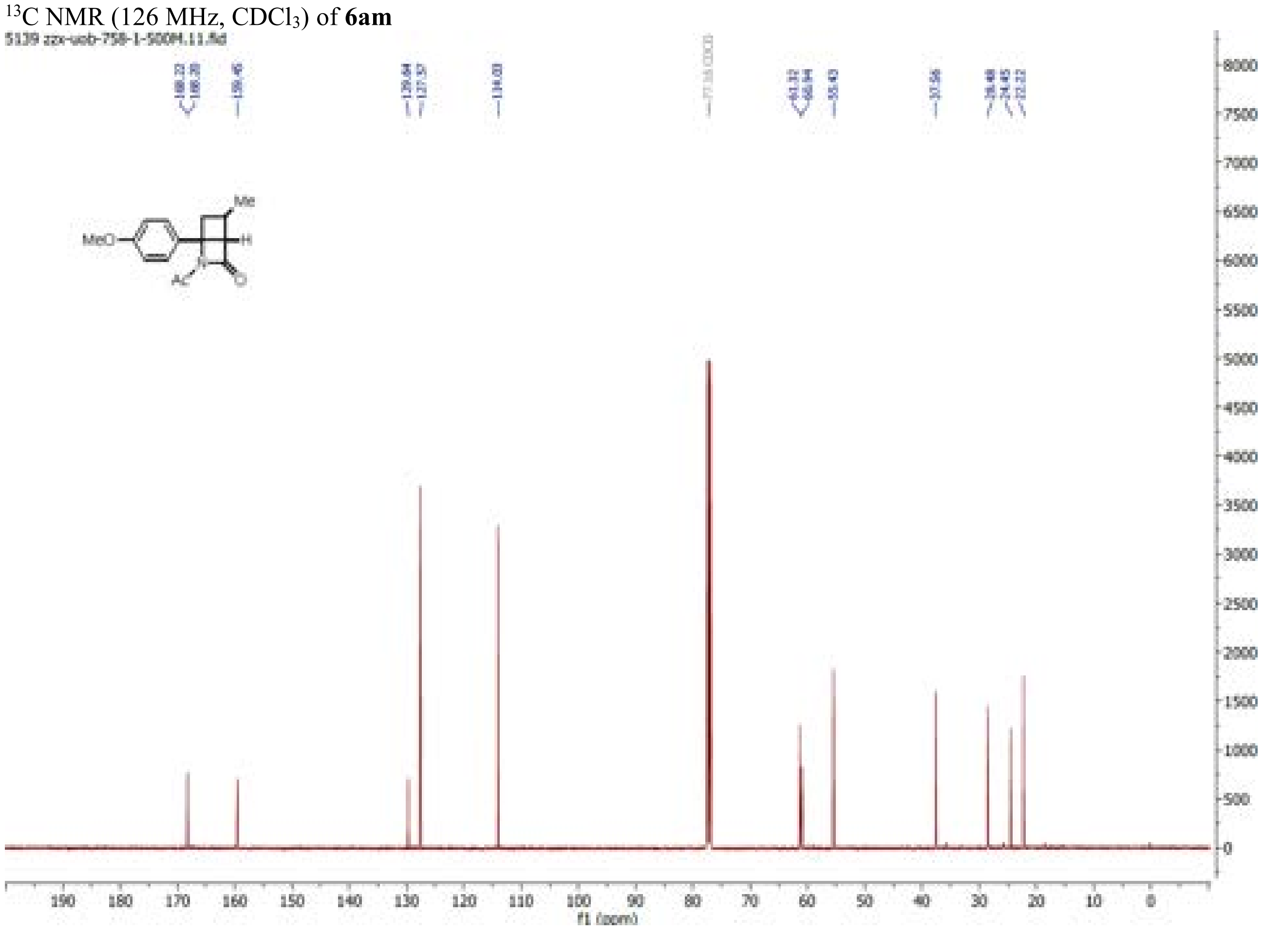

^{1}H NMR (500 MHz, $CDCl_3$) of **5am**

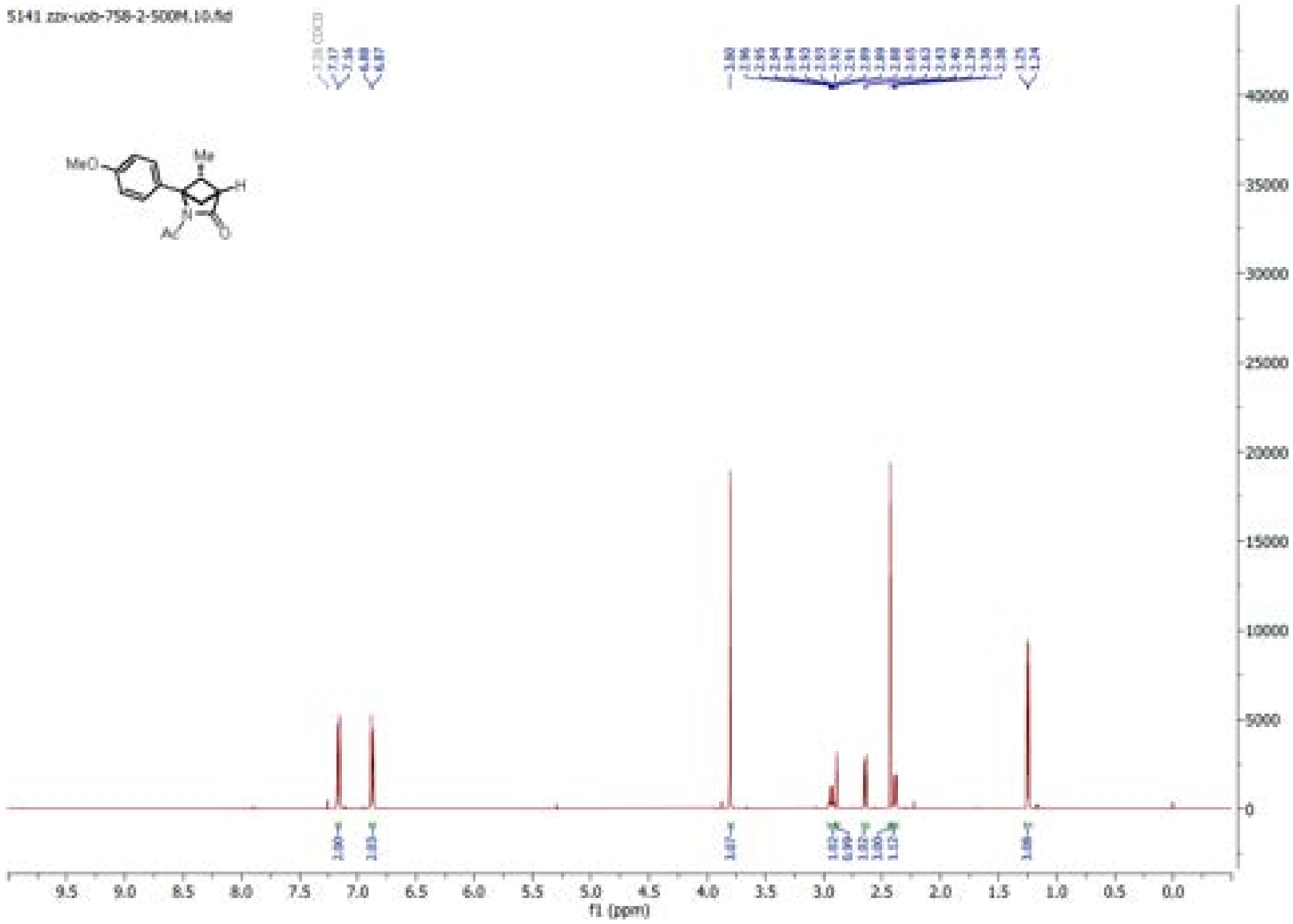

^{13}C NMR (126 MHz, $CDCl_3$) of **5am**

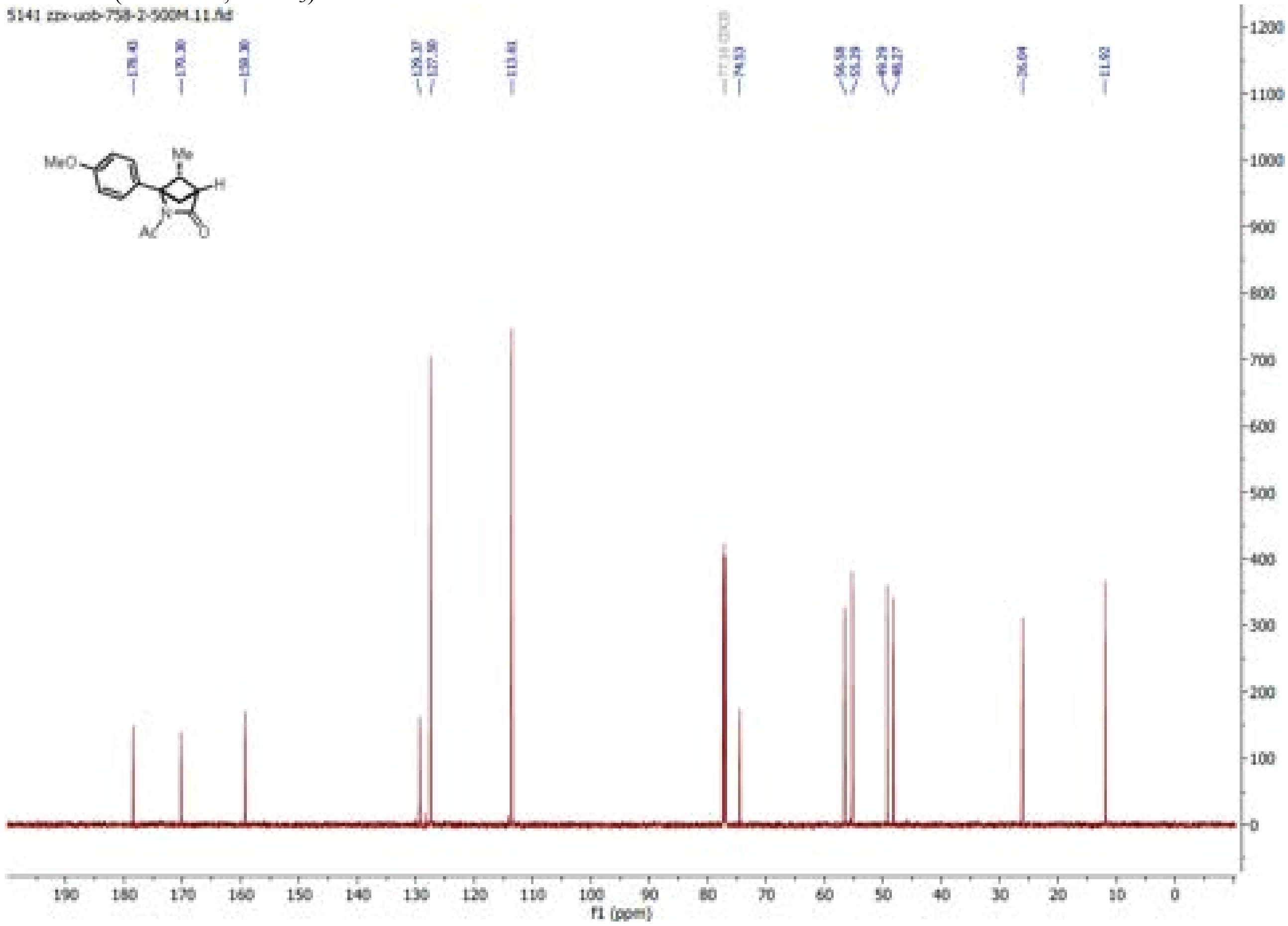

^{1}H NMR (400 MHz, $CDCl_3$) of **6an**

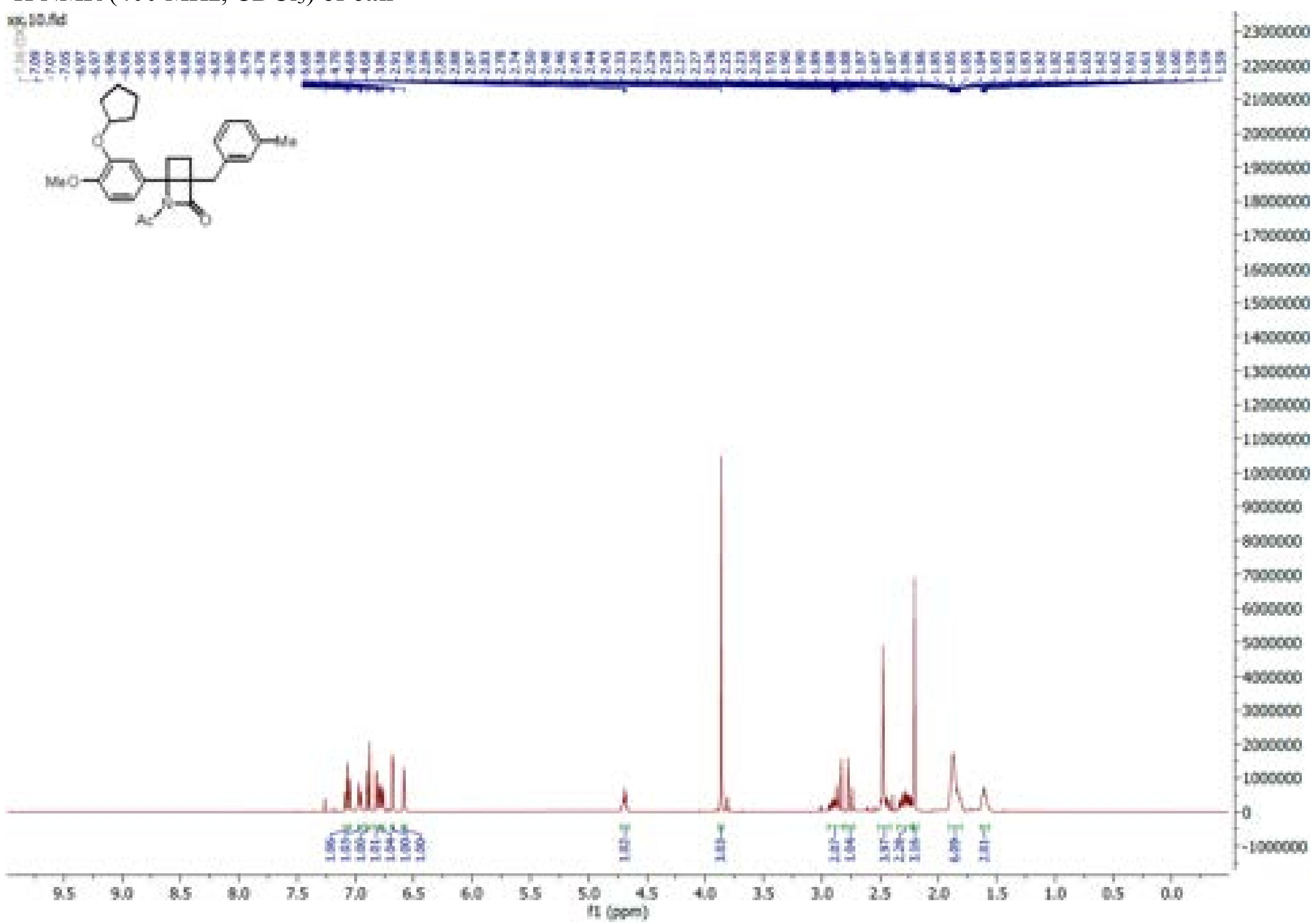

^{13}C NMR (126 MHz, $CDCl_3$) of **6an**

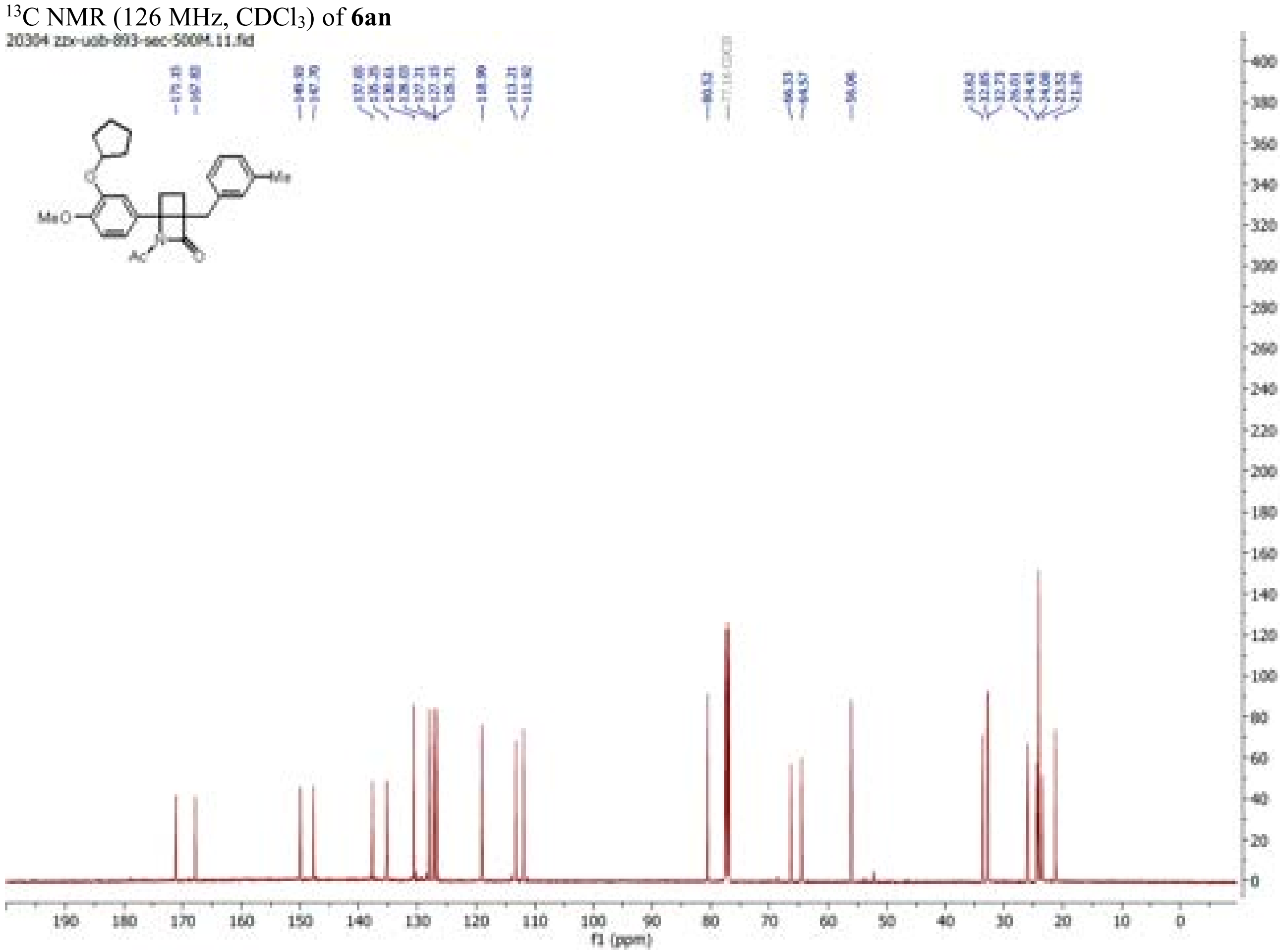

5.7 NMR Spectra of Product Derivatives

^{1}H NMR (500 MHz, $CDCl_3$) of **3c'**

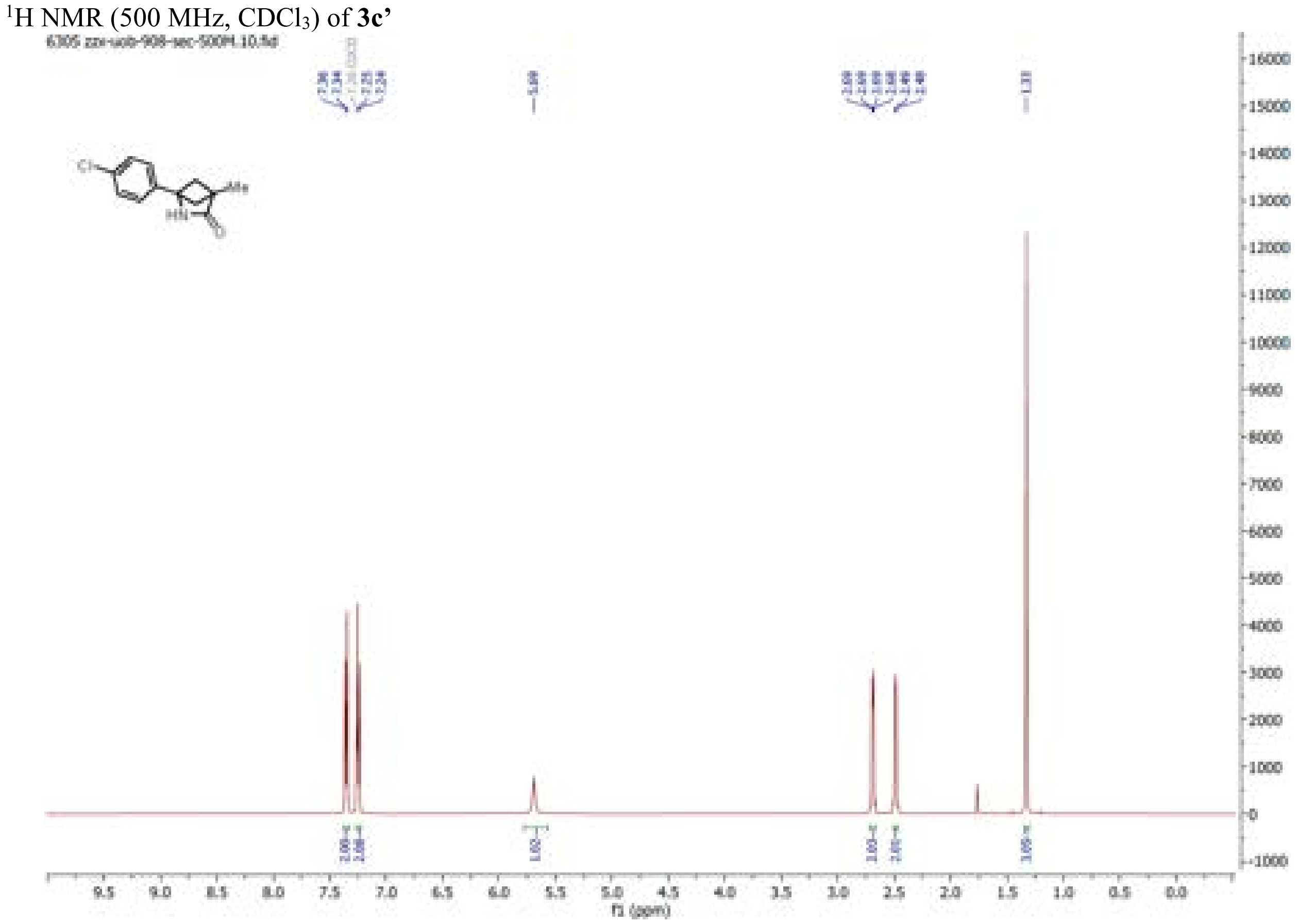

^{13}C NMR (126 MHz, $CDCl_3$) of **3c'**

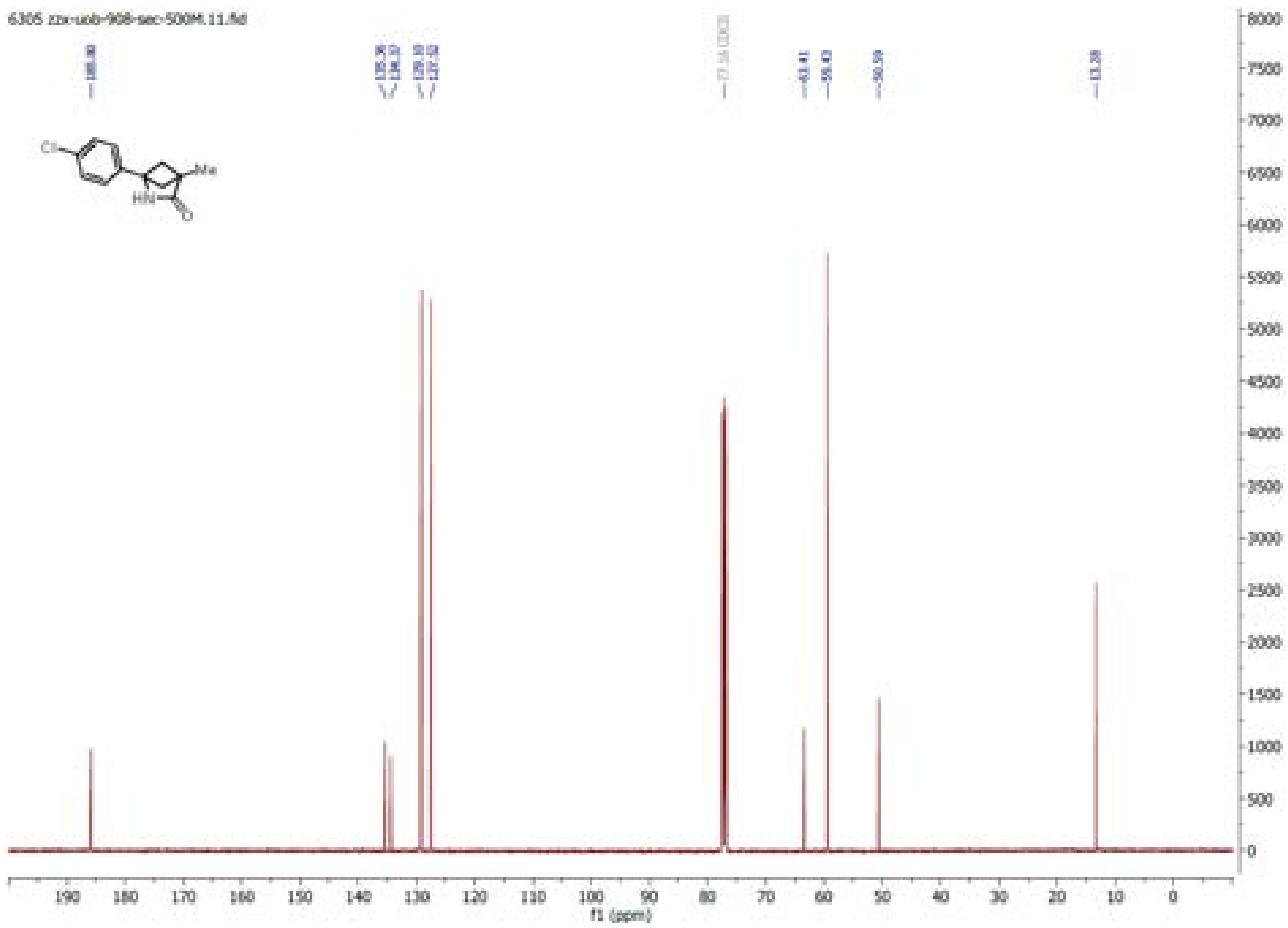

^{1}H NMR (500 MHz, $CDCl_3$) of **8a’**

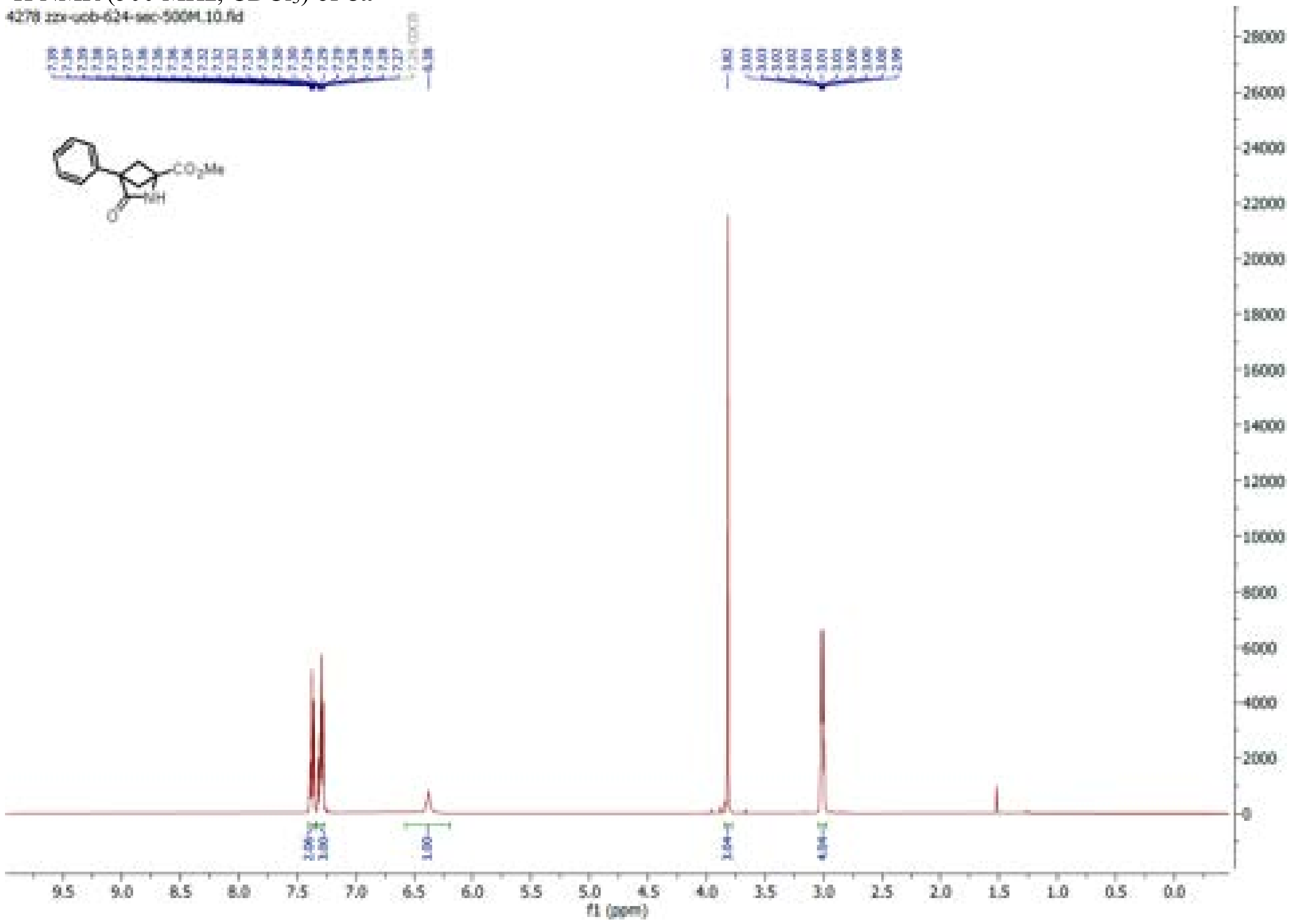

^{13}C NMR (126 MHz, $CDCl_3$) of **8a’**

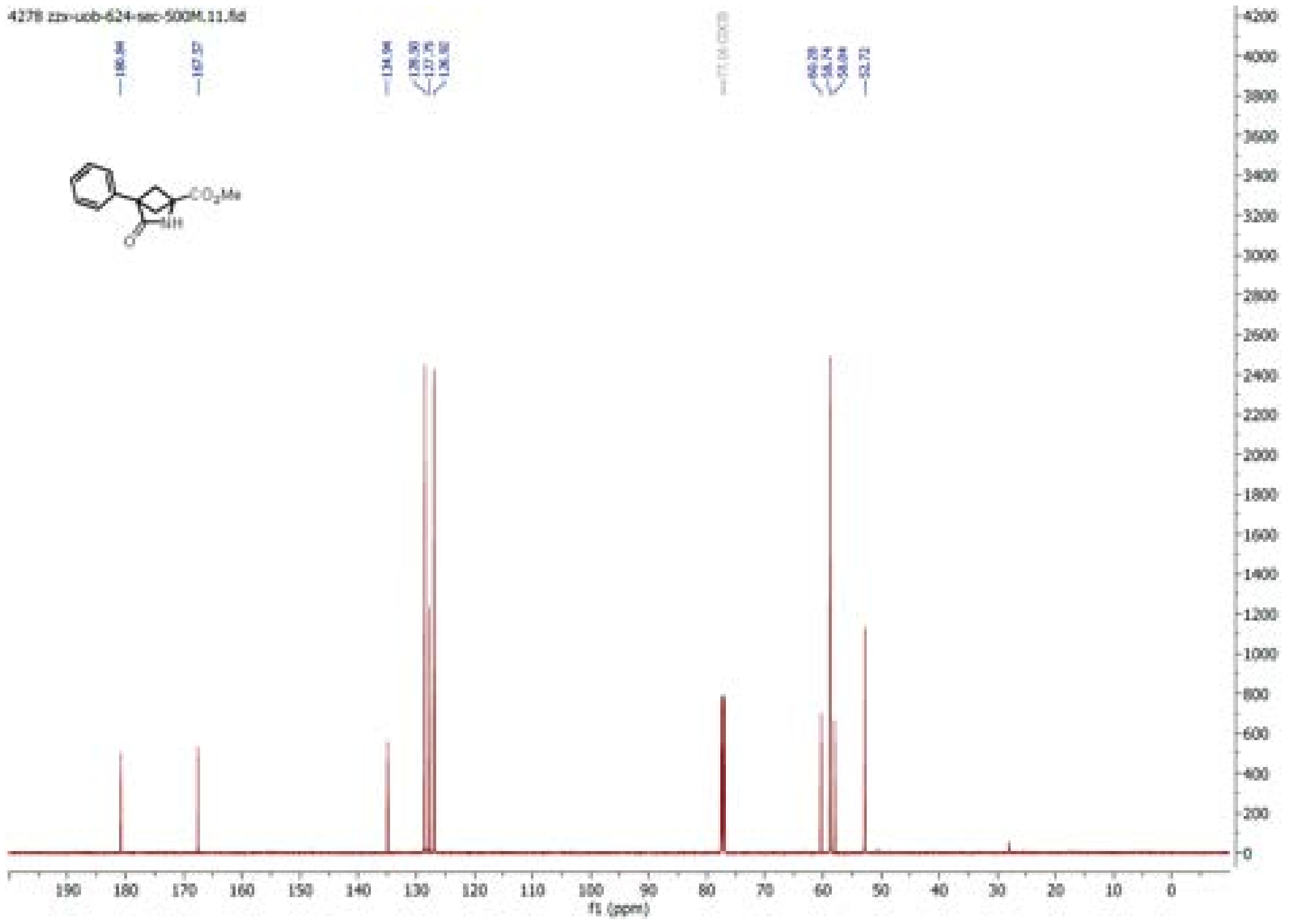

^{1}H NMR (500 MHz, $CDCl_3$) of **8a''**

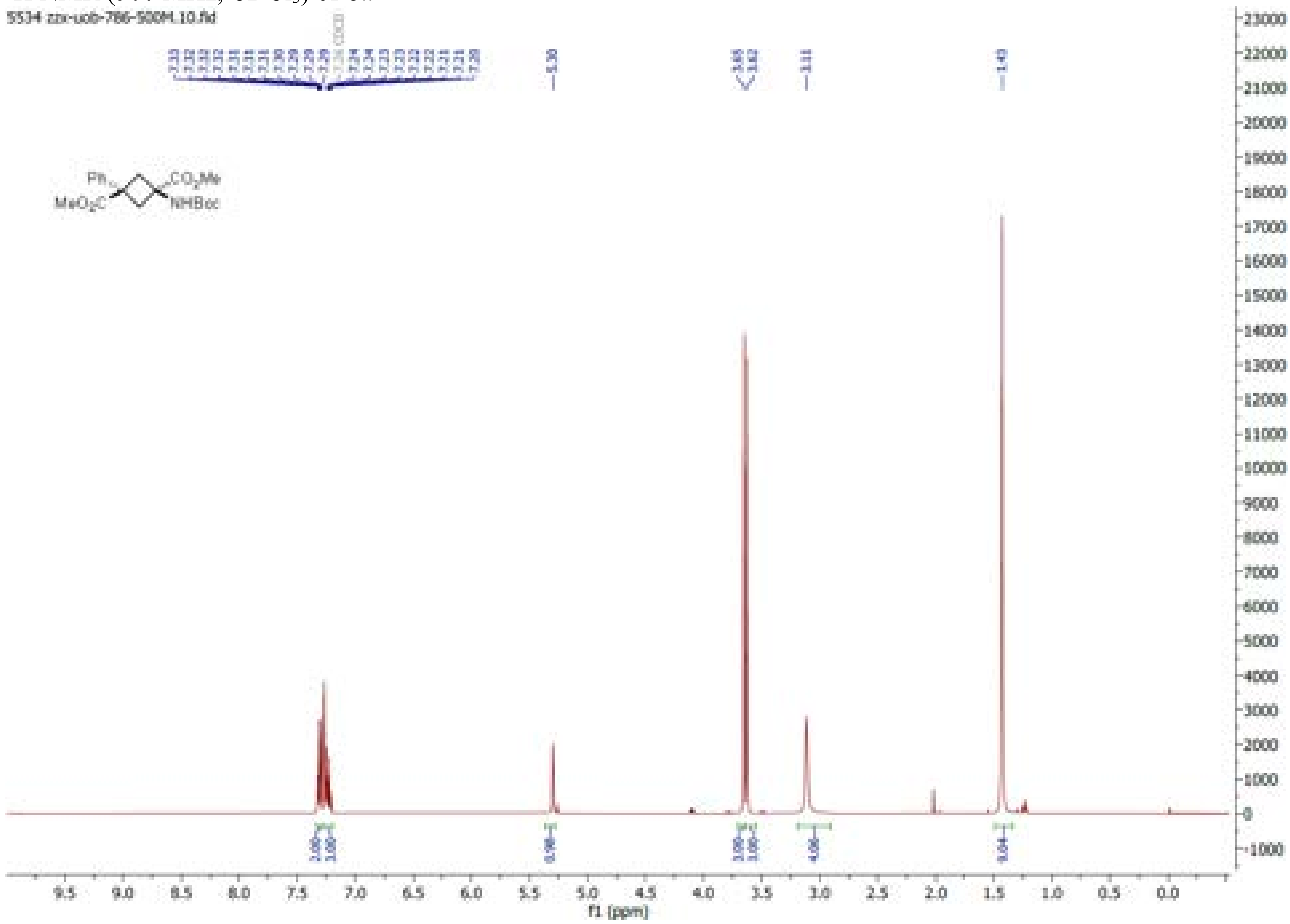

^{13}C NMR (126 MHz, $CDCl_3$) of **8a''**

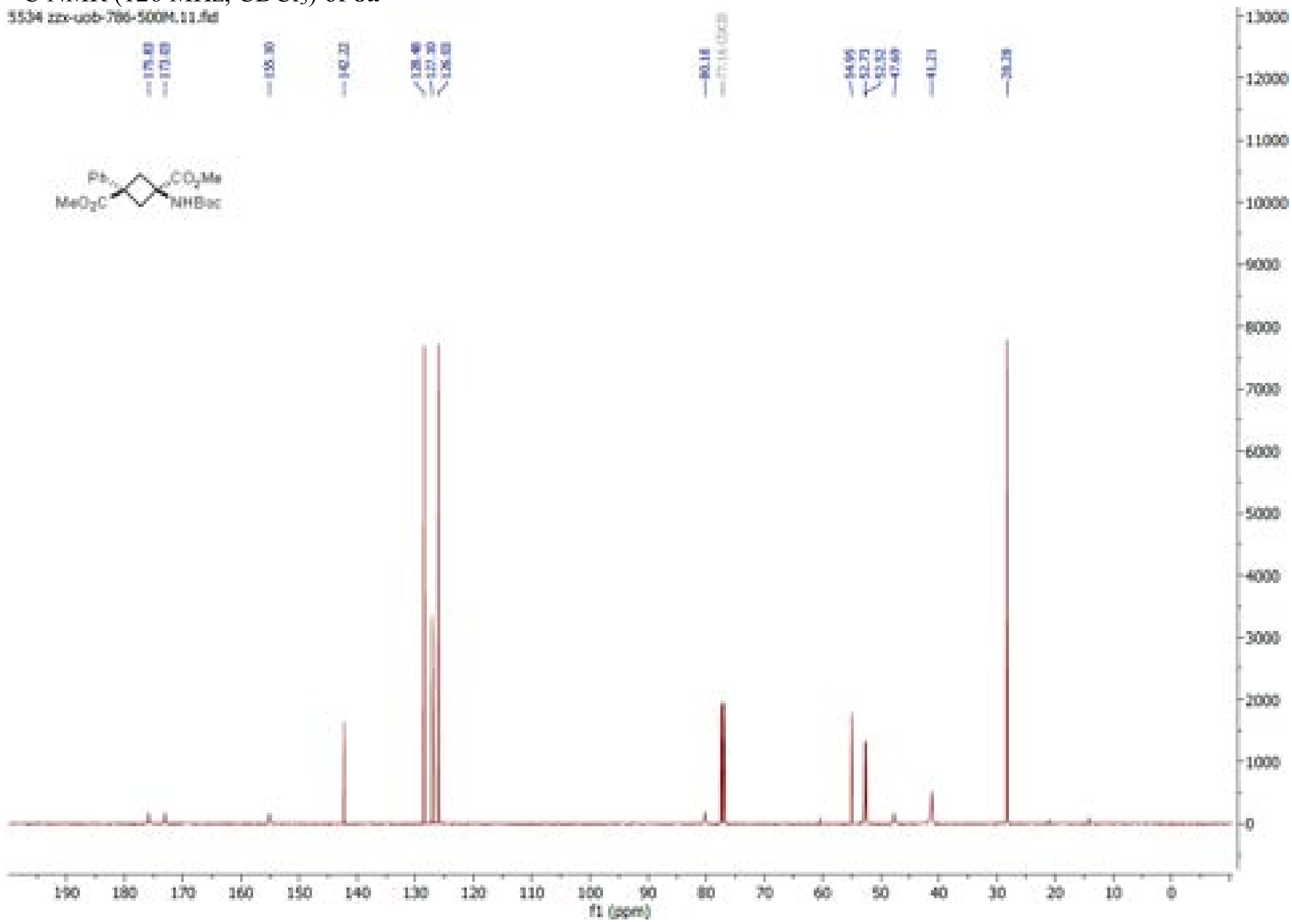

^{1}H NMR (500 MHz, $CDCl_3$) of **10**

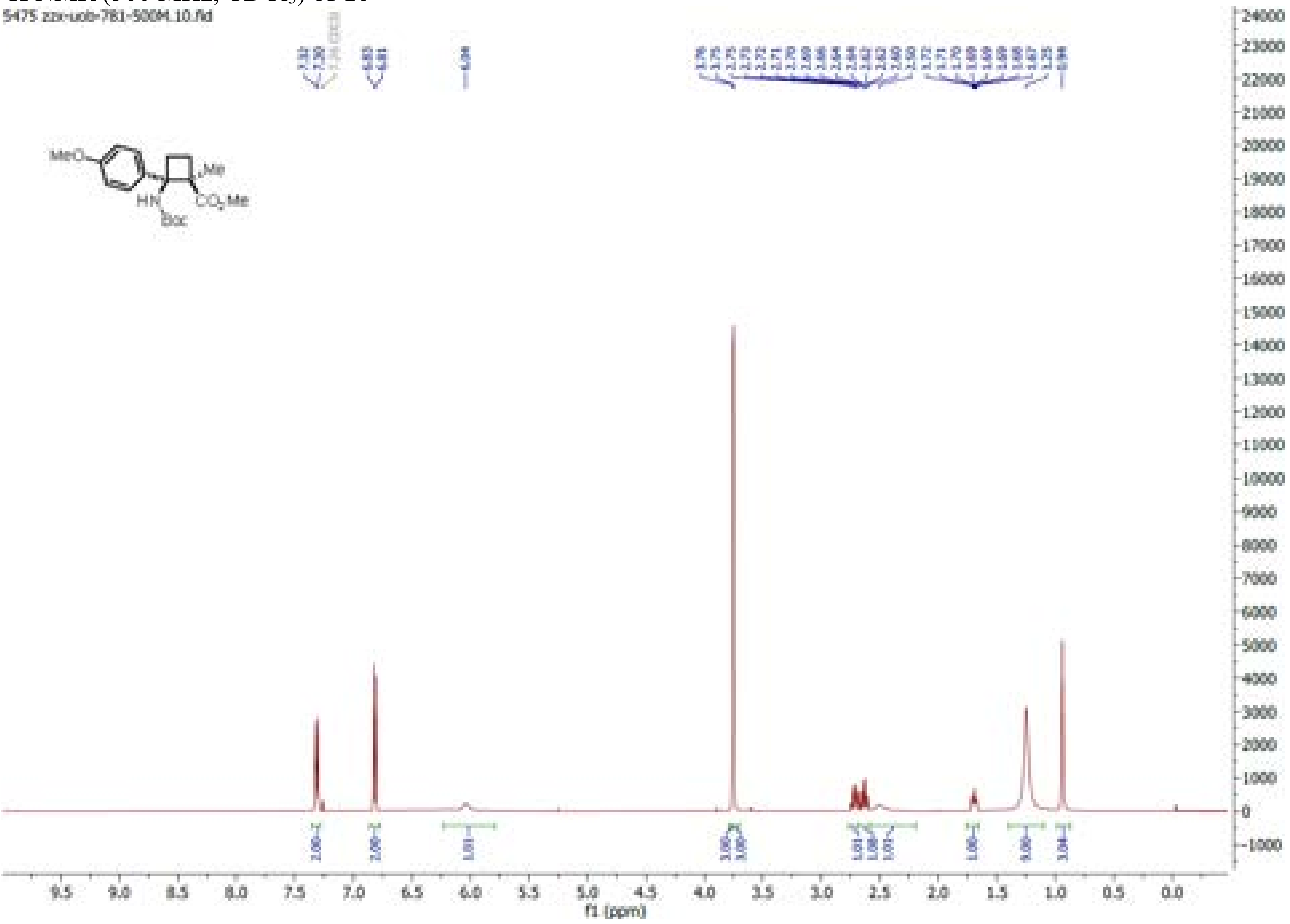

^{13}C NMR (126 MHz, $CDCl_3$) of **10**

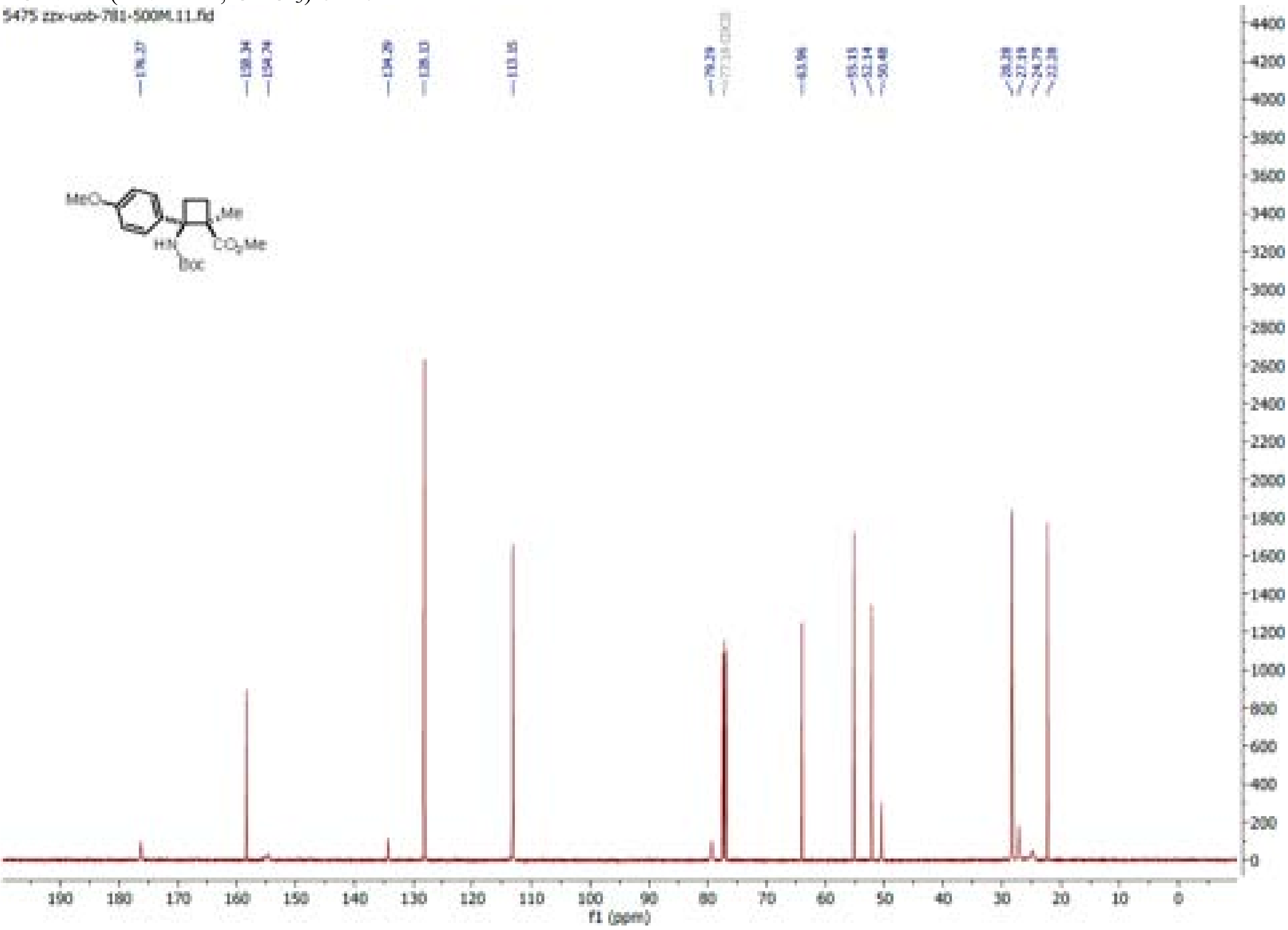

^{1}H NMR (500 MHz, $CDCl_3$) of **11**

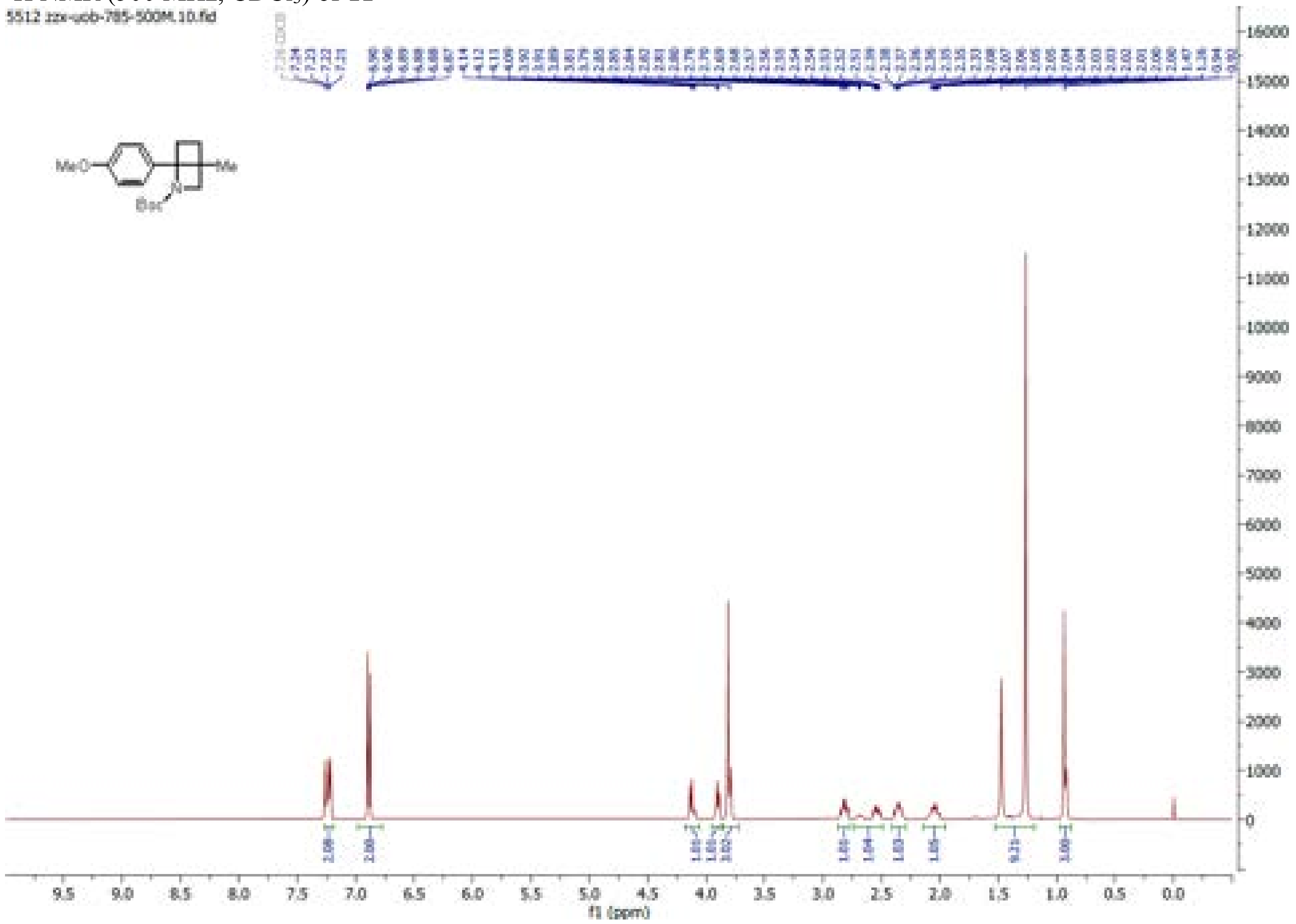

^{13}C NMR (126 MHz, $CDCl_3$) of **11**

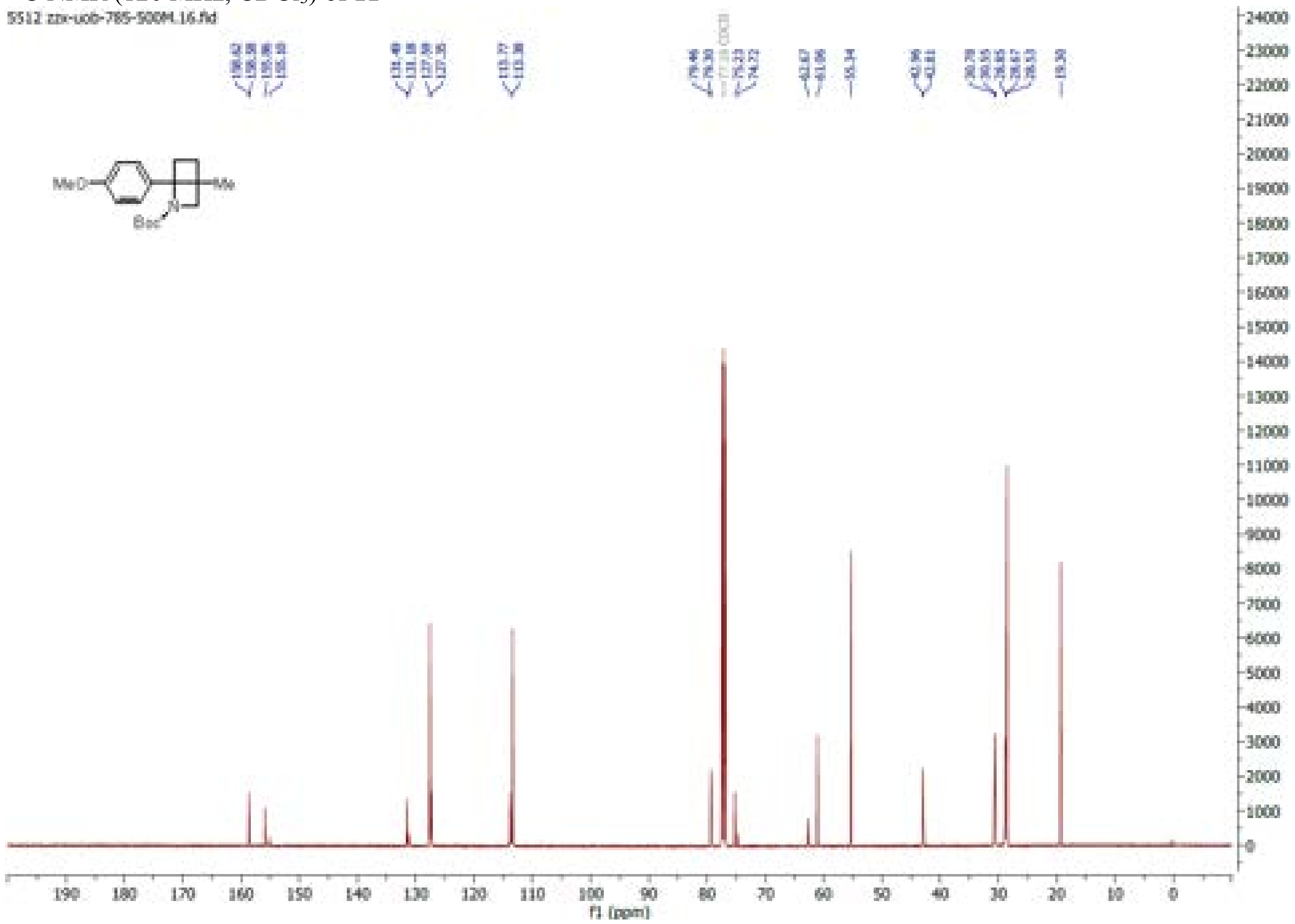

^{1}H NMR (500 MHz, $CDCl_3$) of **12**

5444 zzx-uob-773-1-500M-sec.10.fid

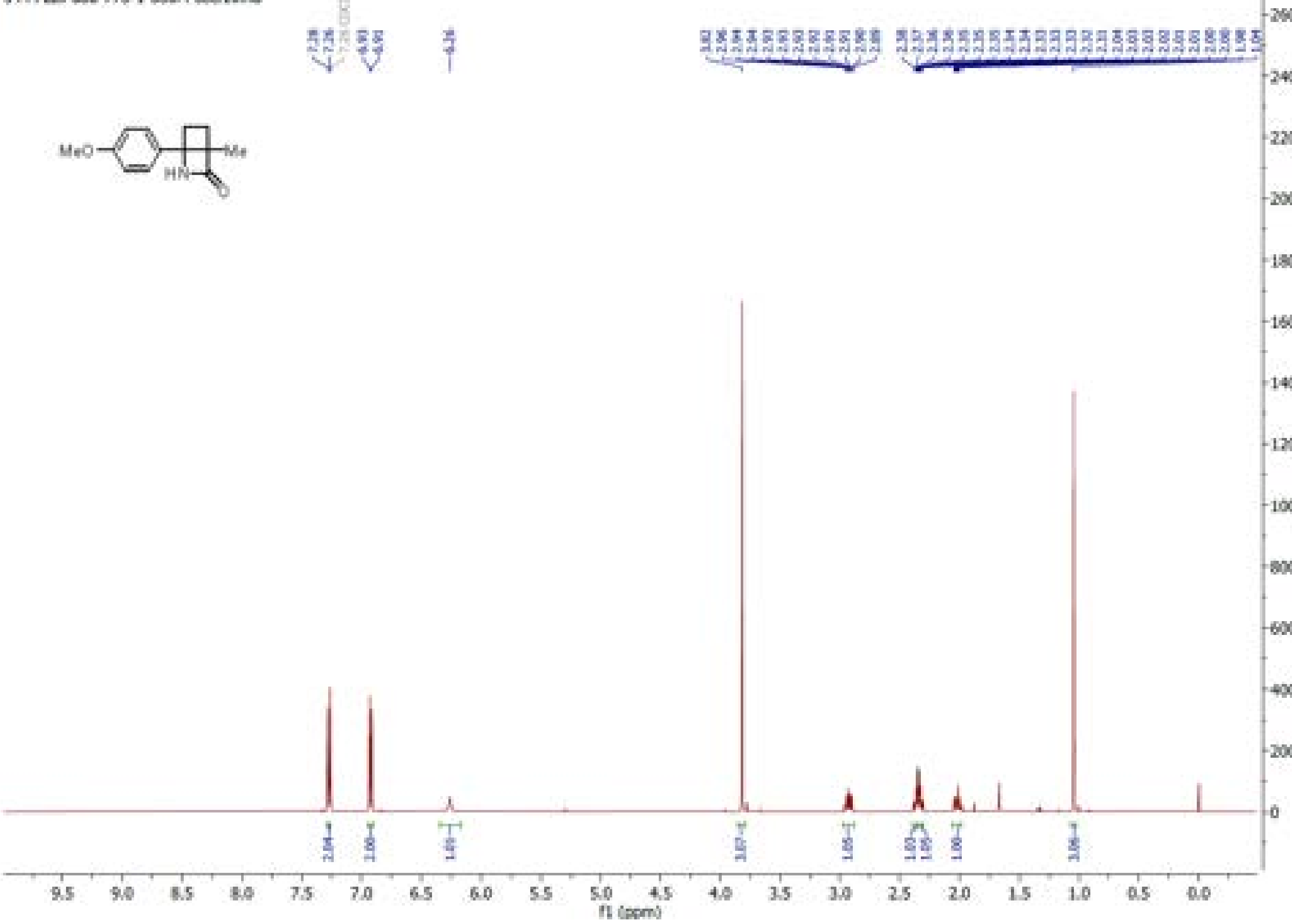

^{13}C NMR (126 MHz, $CDCl_3$) of **12**

5444 zzx-uob-773-1-500M-sec.11.fid

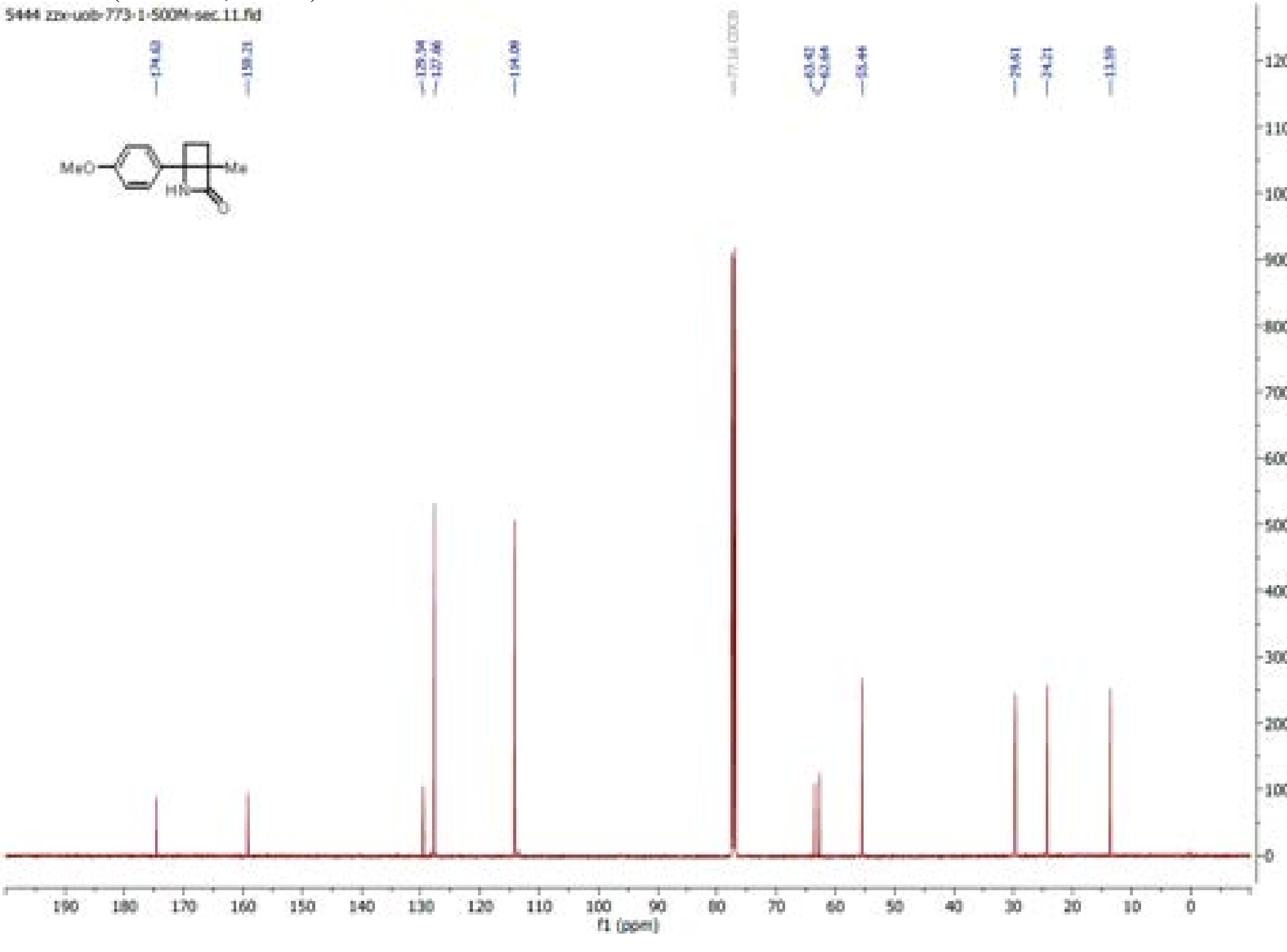

^{1}H NMR (500 MHz, $CDCl_3$) of **12'**

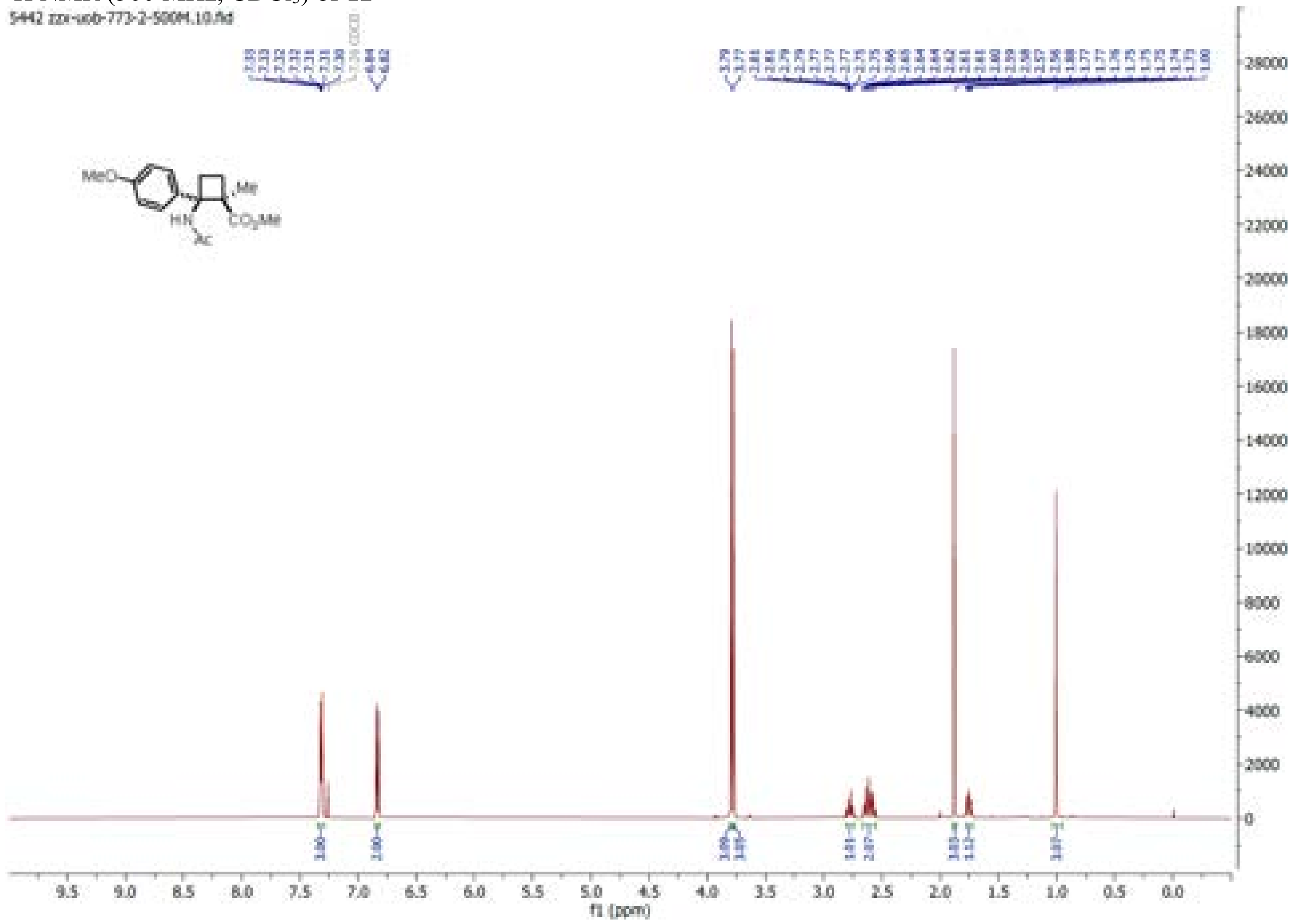

^{13}C NMR (126 MHz, $CDCl_3$) of **12'**

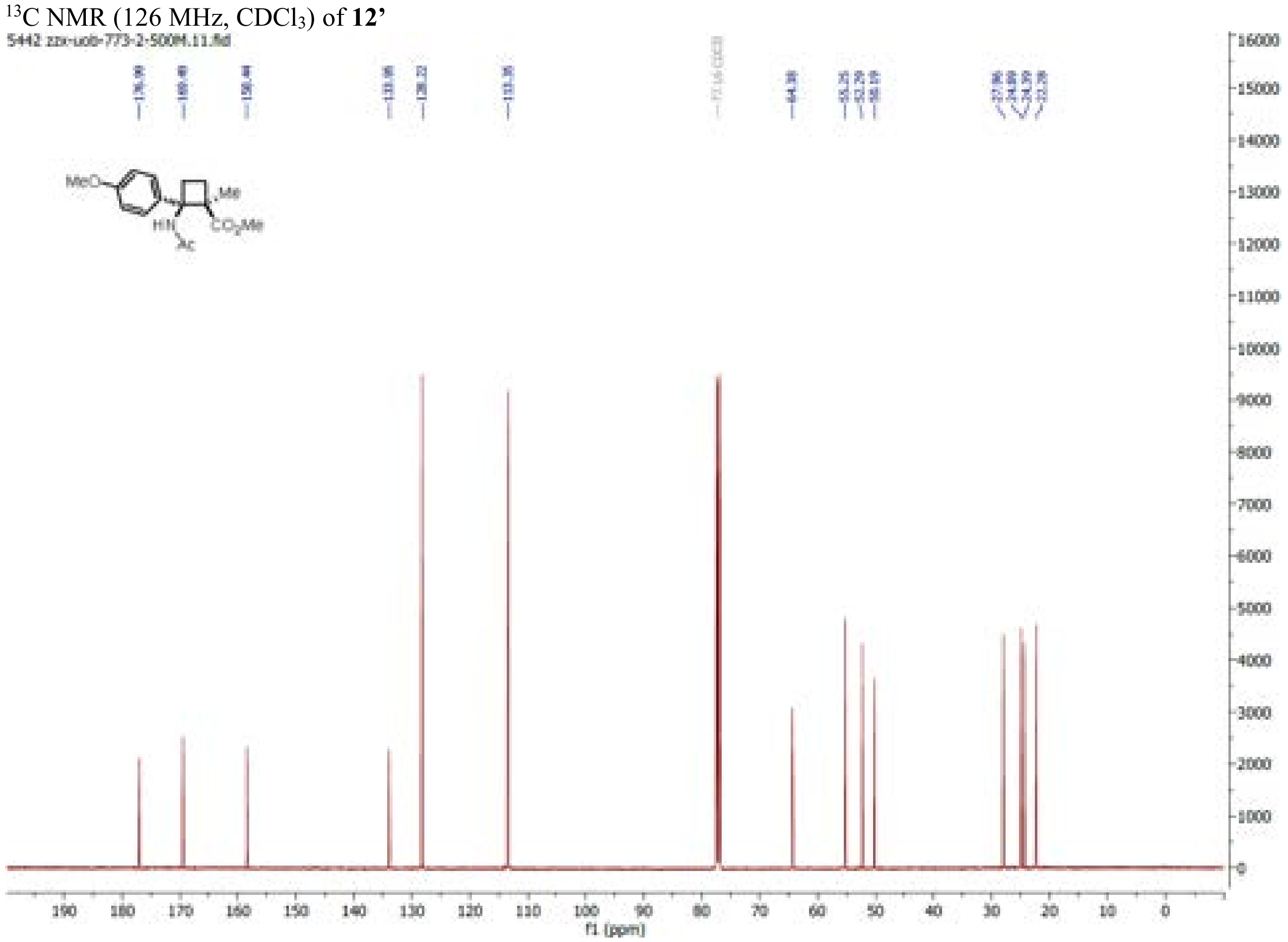

^{1}H NMR (500 MHz, $CDCl_3$) of **13**

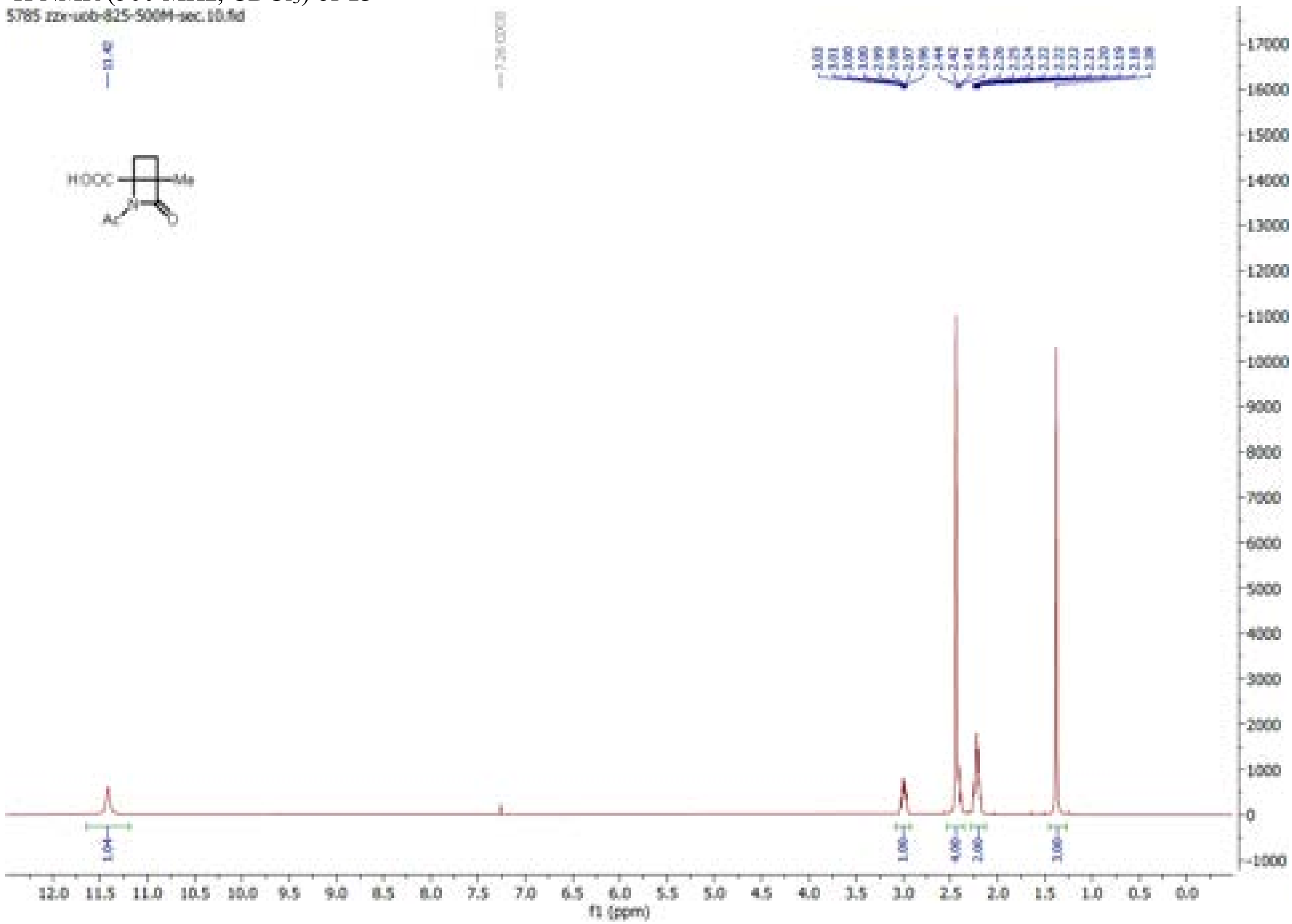

^{13}C NMR (126 MHz, $CDCl_3$) of **13**

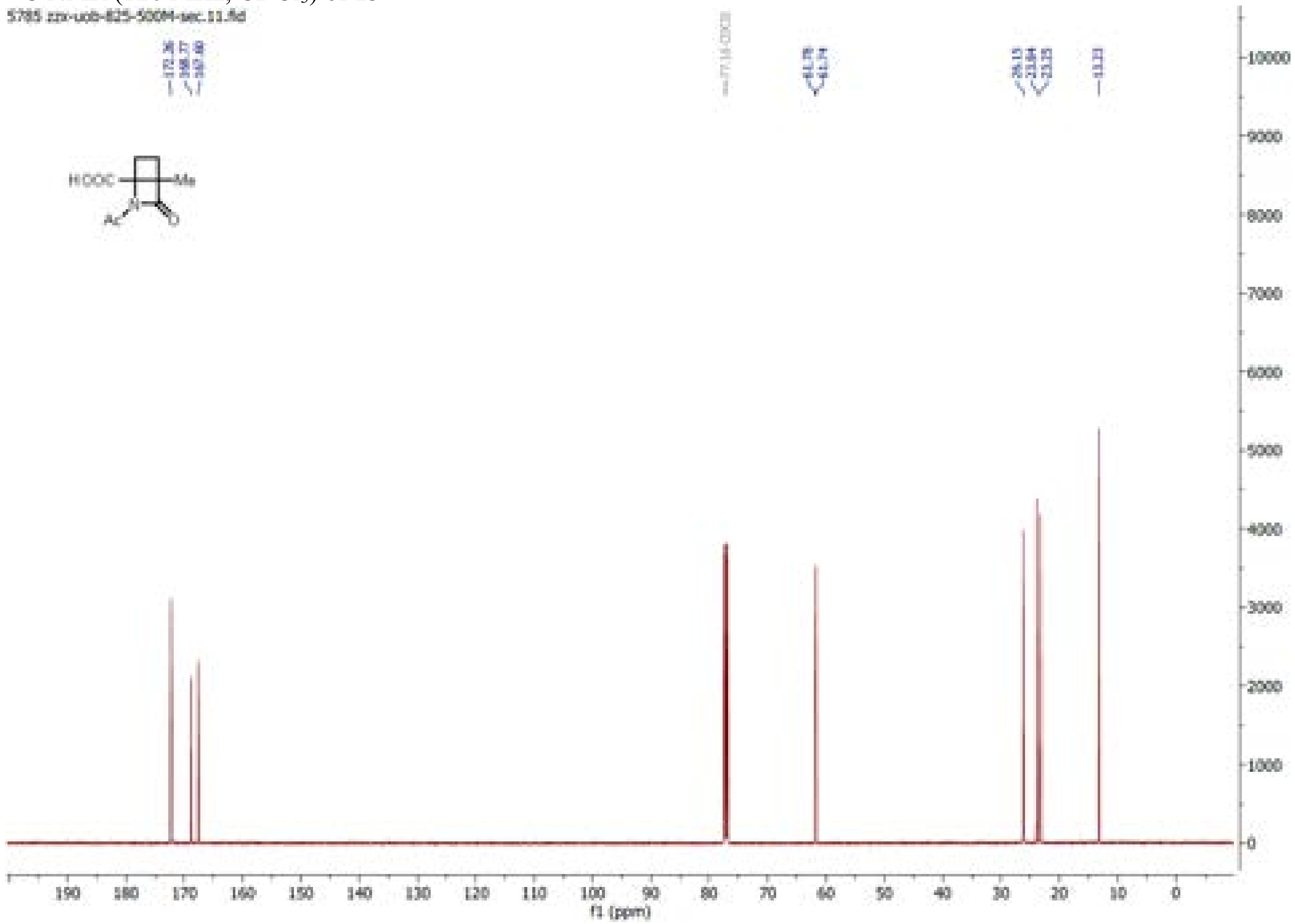

^{1}H NMR (500 MHz, $CDCl_3$) of **14**

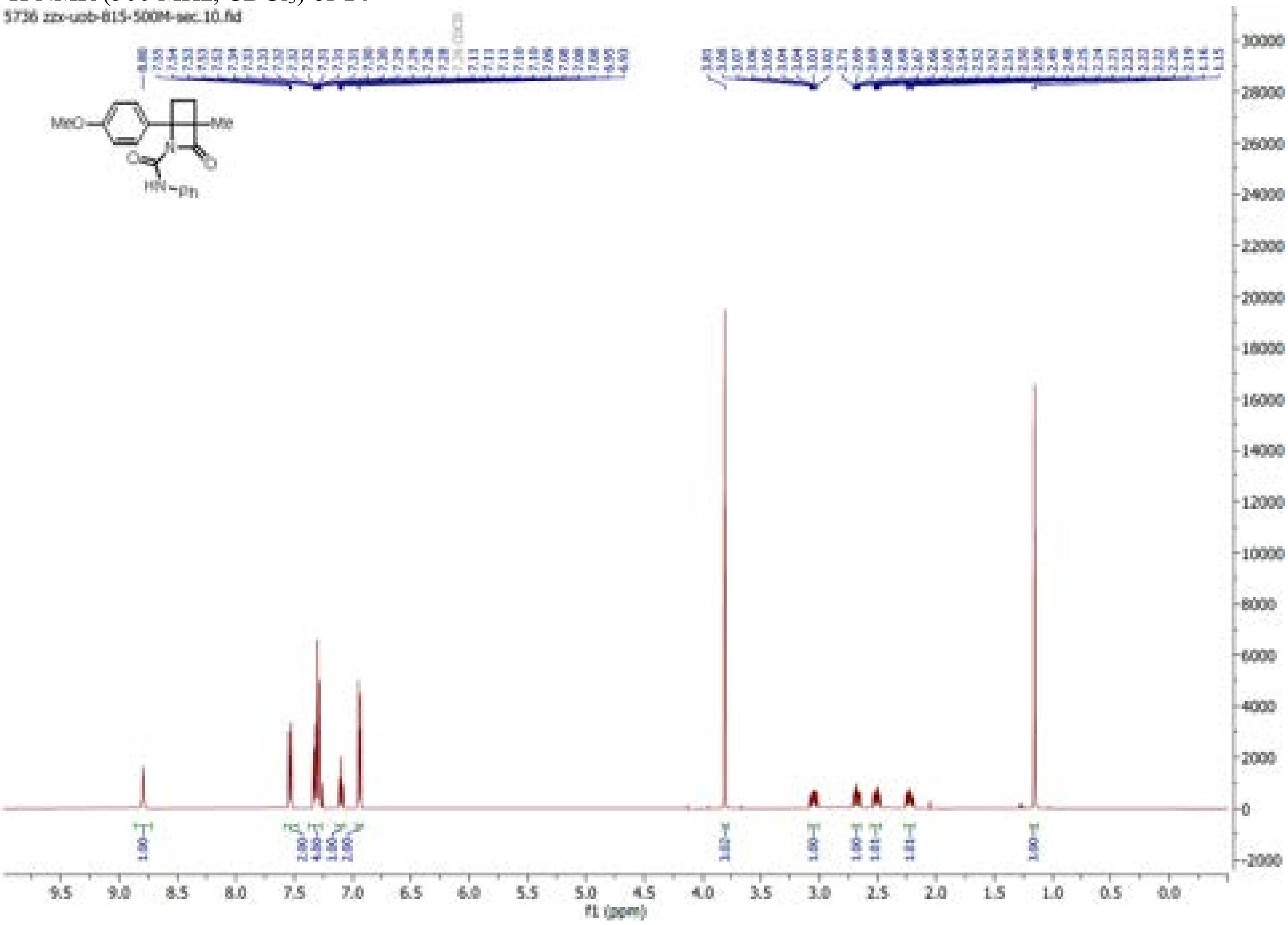

^{13}C NMR (126 MHz, $CDCl_3$) of **14**

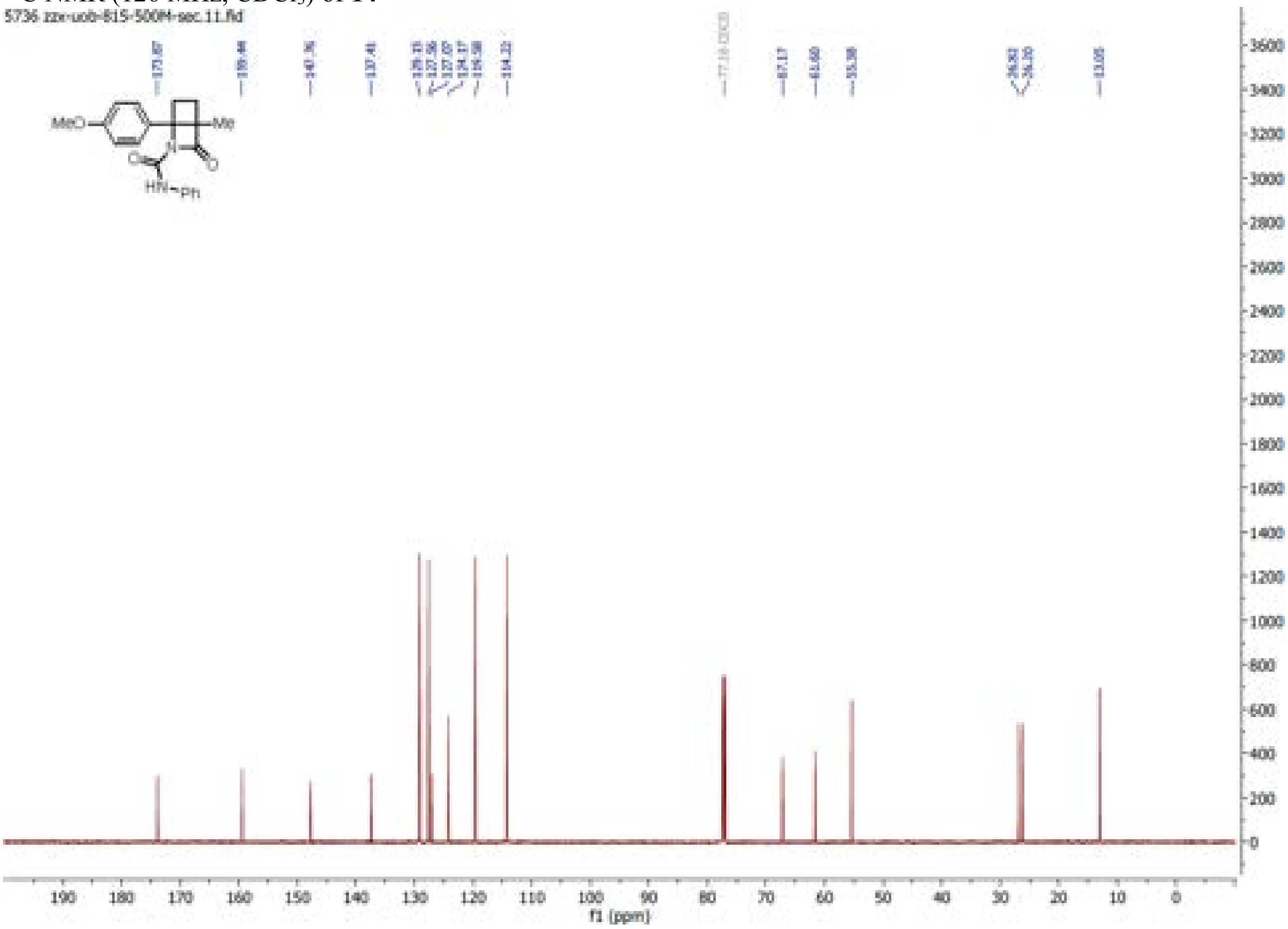

^1H NMR (500 MHz, $CDCl_3$) of **15**

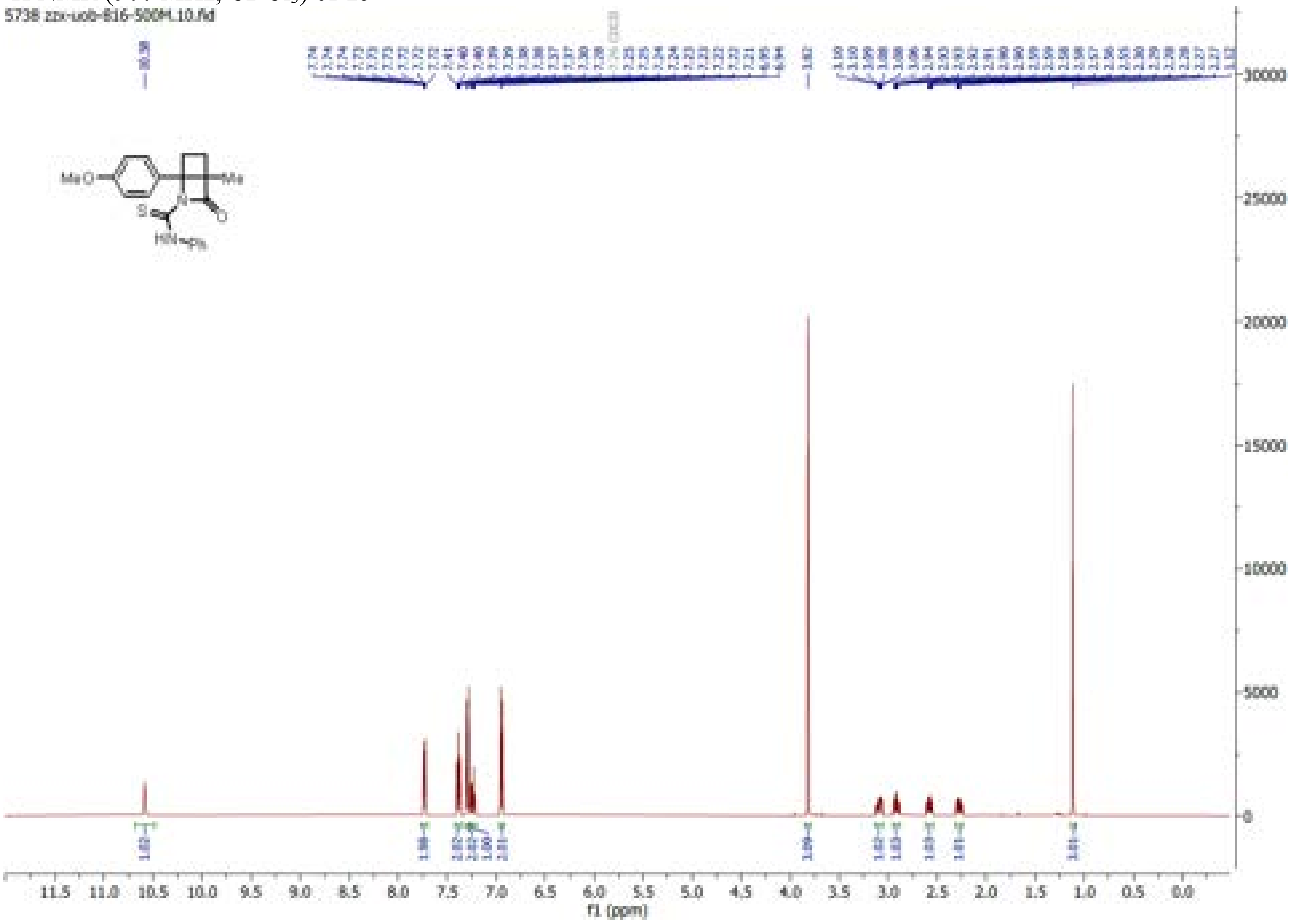

^{13}C NMR (126 MHz, $CDCl_3$) of **15**

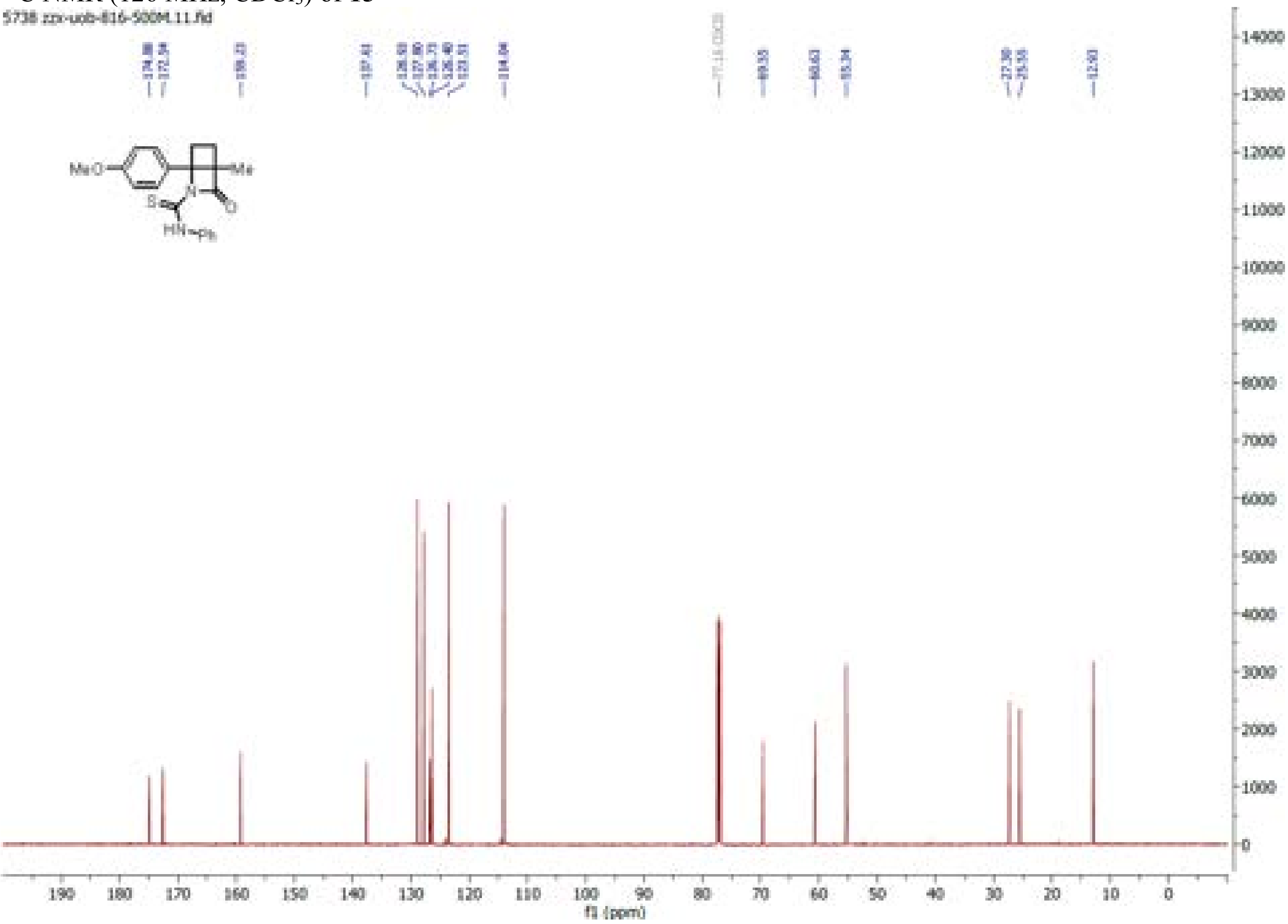

^{1}H NMR (500 MHz, $CDCl_3$) of **16**

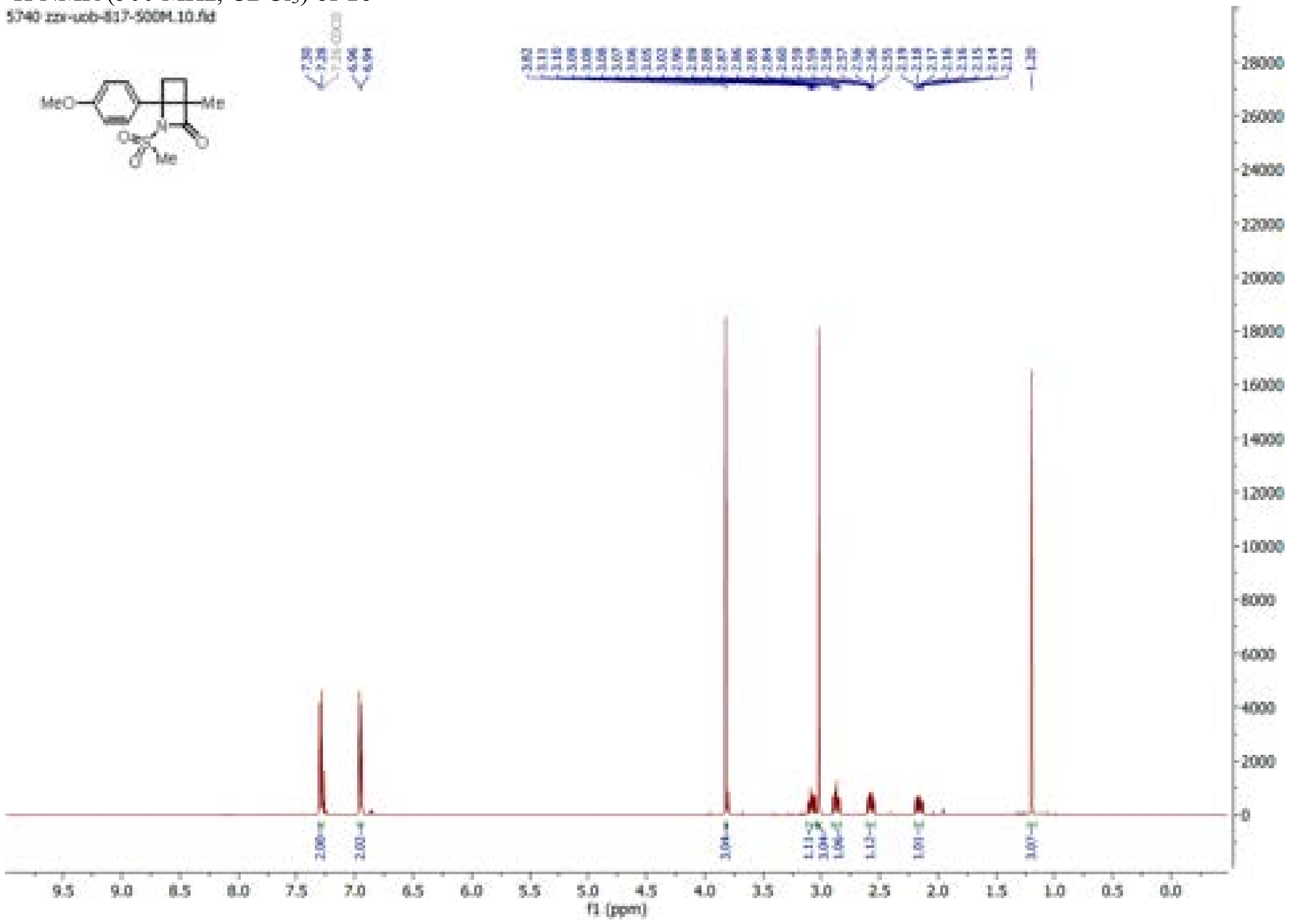

^{13}C NMR (126 MHz, $CDCl_3$) of **16**

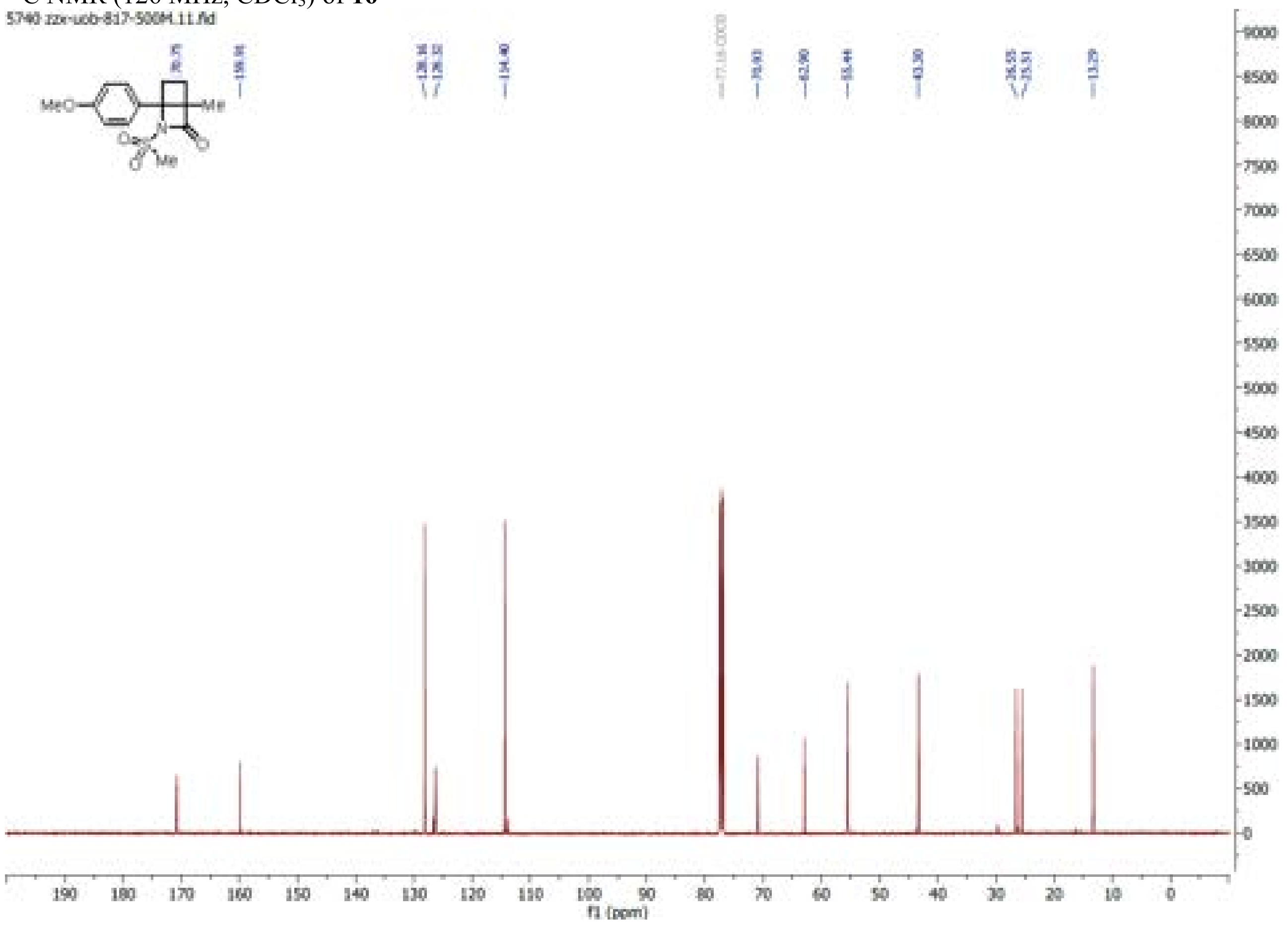

^{1}H NMR (500 MHz, $CDCl_3$) of **17**

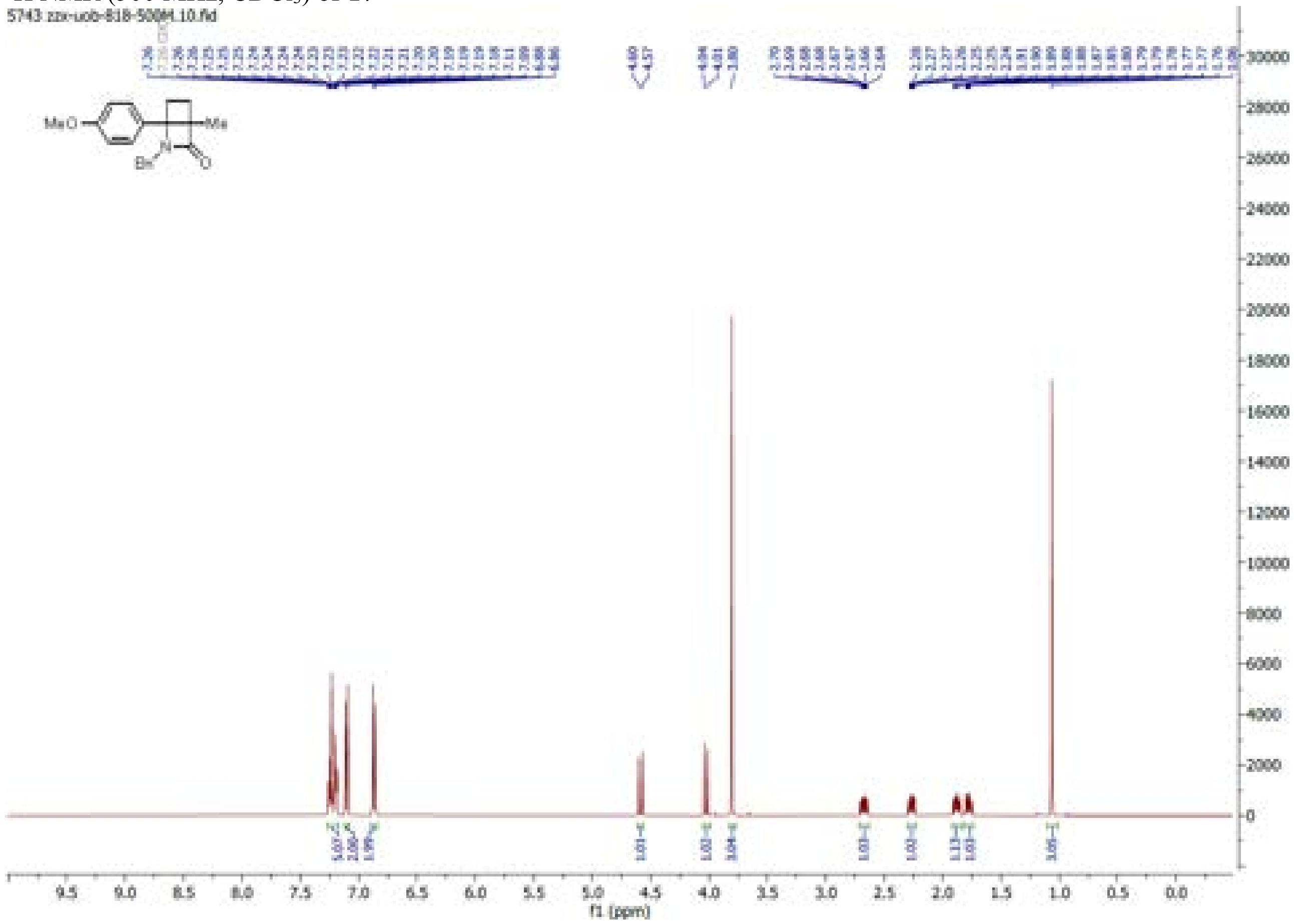

^{13}C NMR (126 MHz, $CDCl_3$) of **17**

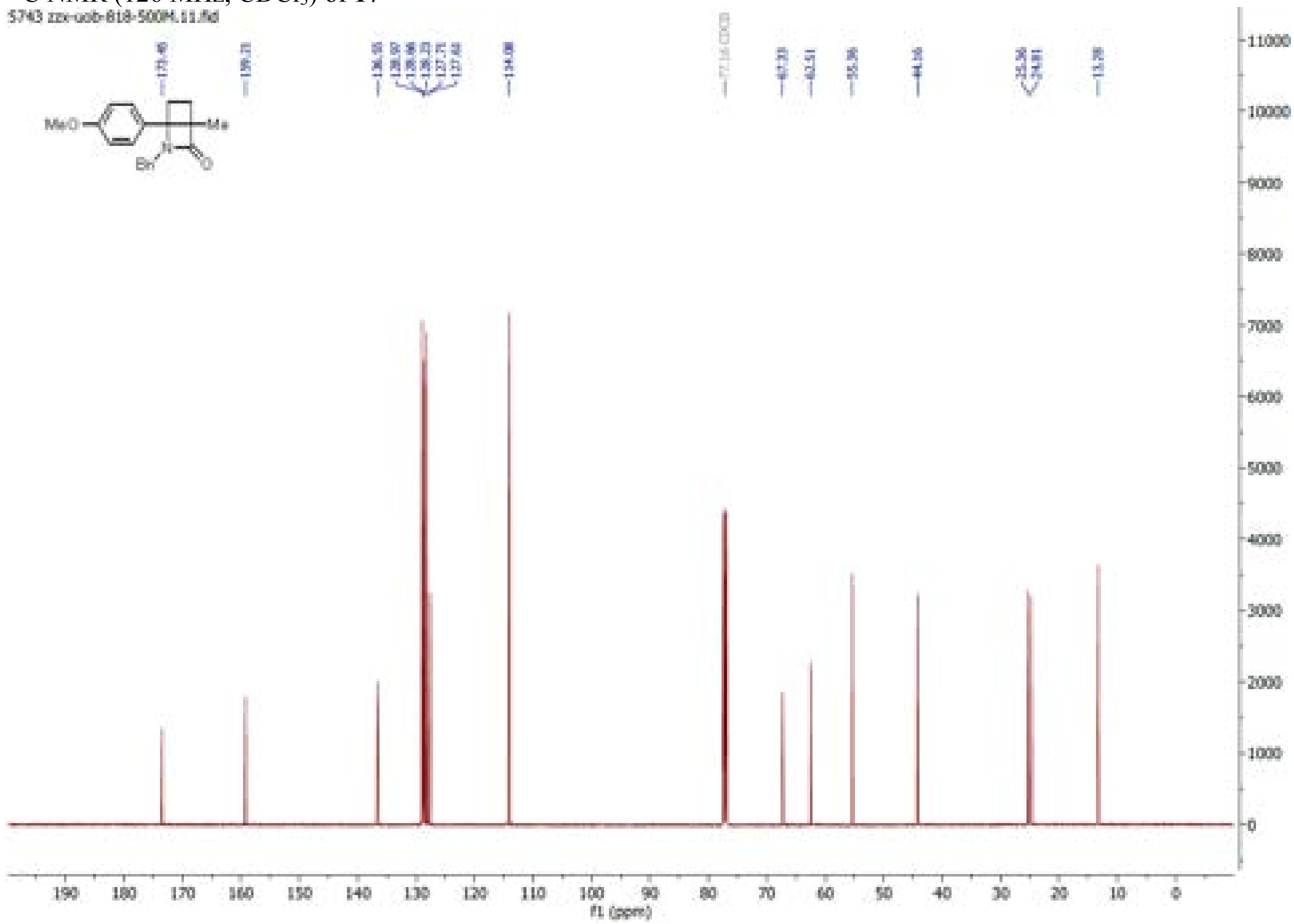

^{1}H NMR (500 MHz, $CDCl_3$) of **18**

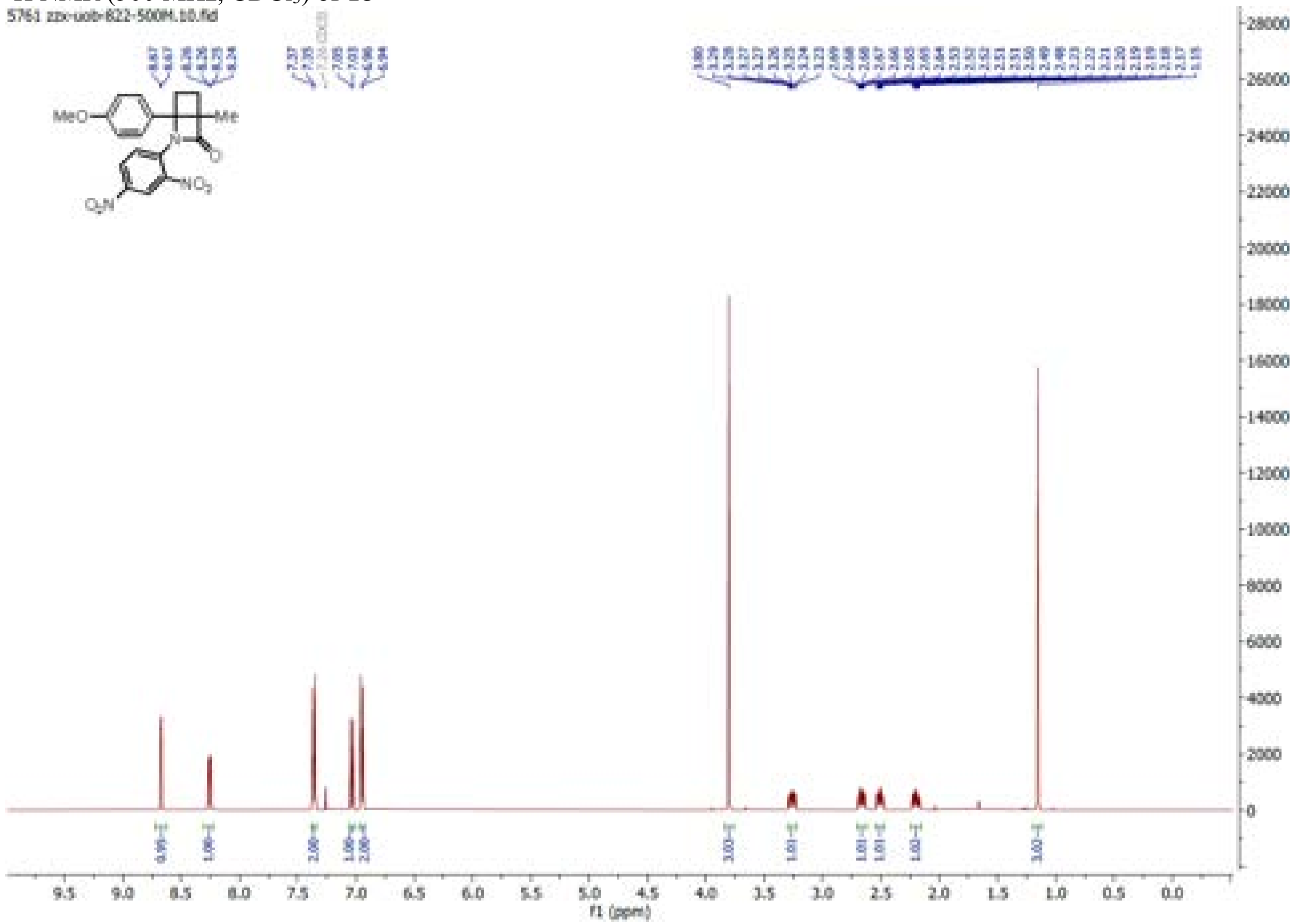

^{13}C NMR (126 MHz, $CDCl_3$) of **18**

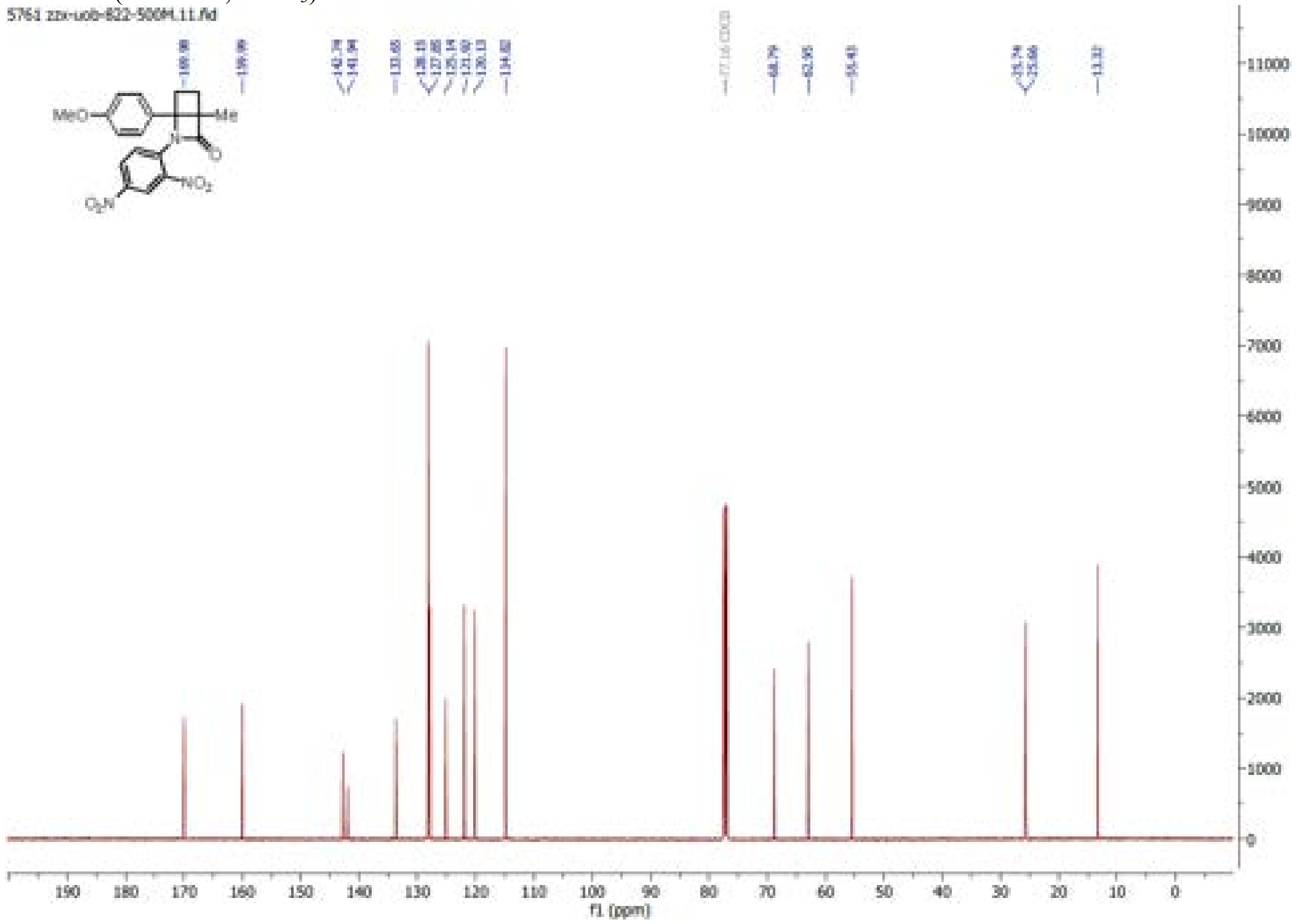

^{1}H NMR (500 MHz, $CDCl_3$) of **19**

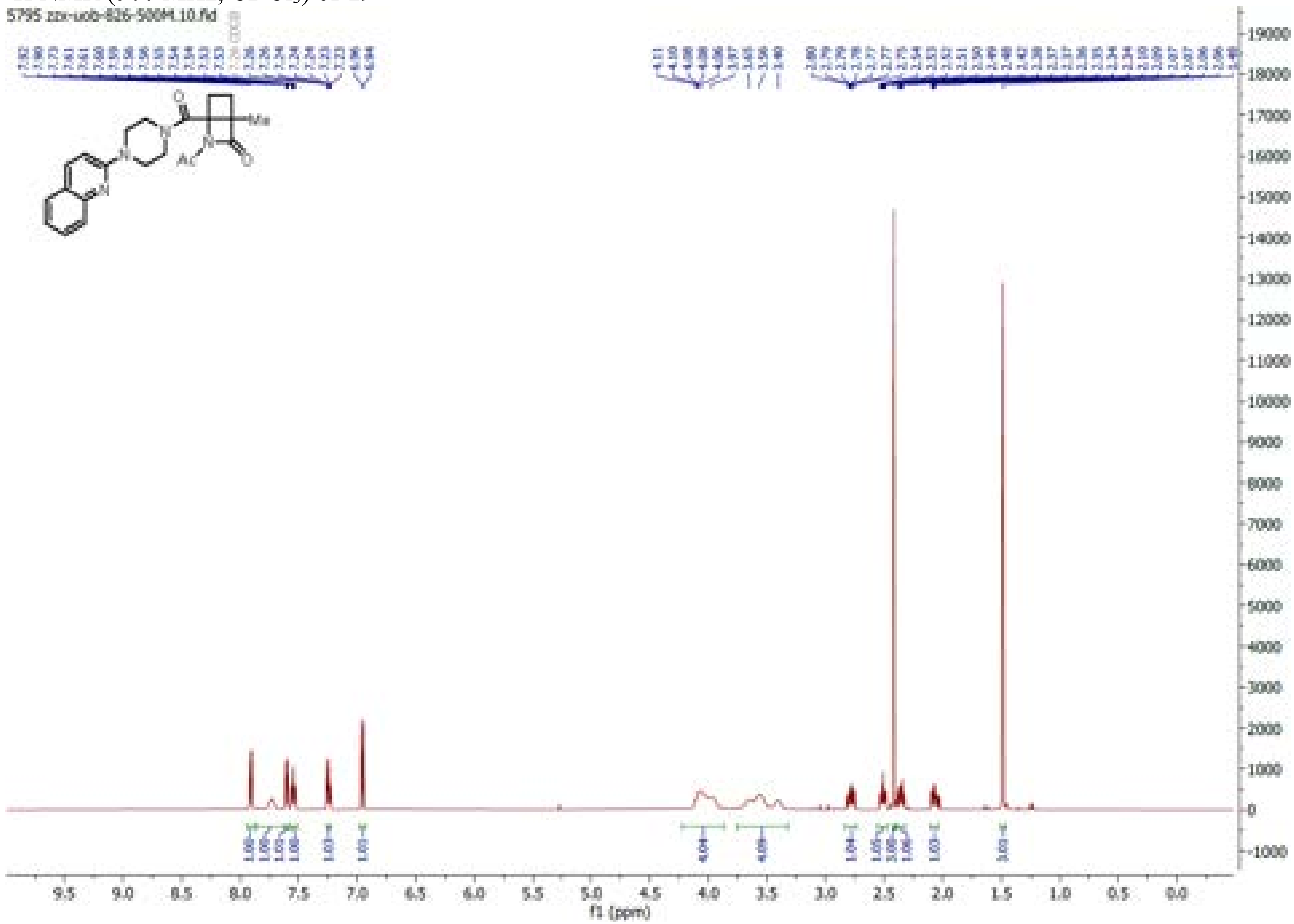

^{13}C NMR (126 MHz, $CDCl_3$) of **19**

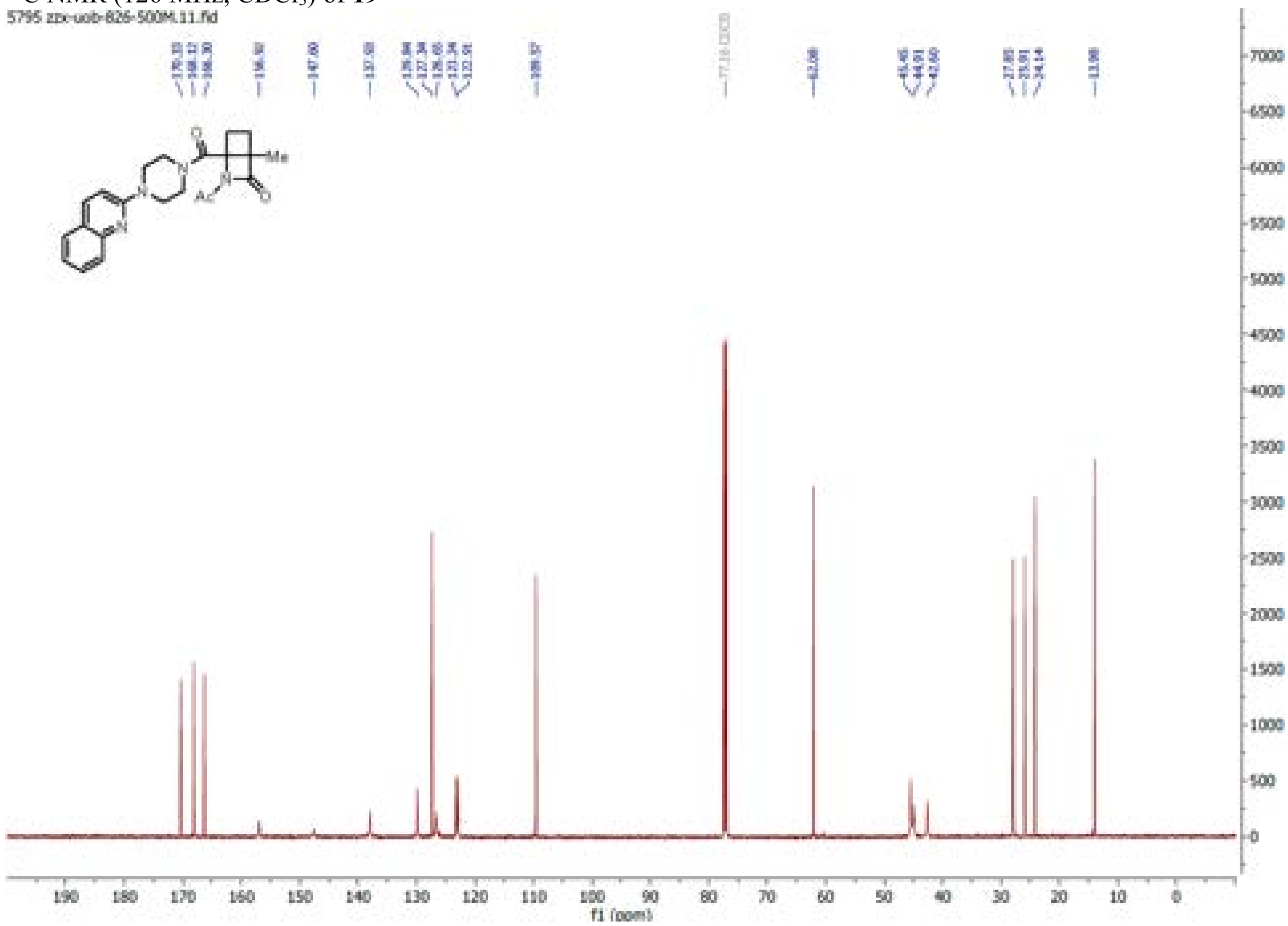

^{1}H NMR (500 MHz, $CDCl_3$) of **20**

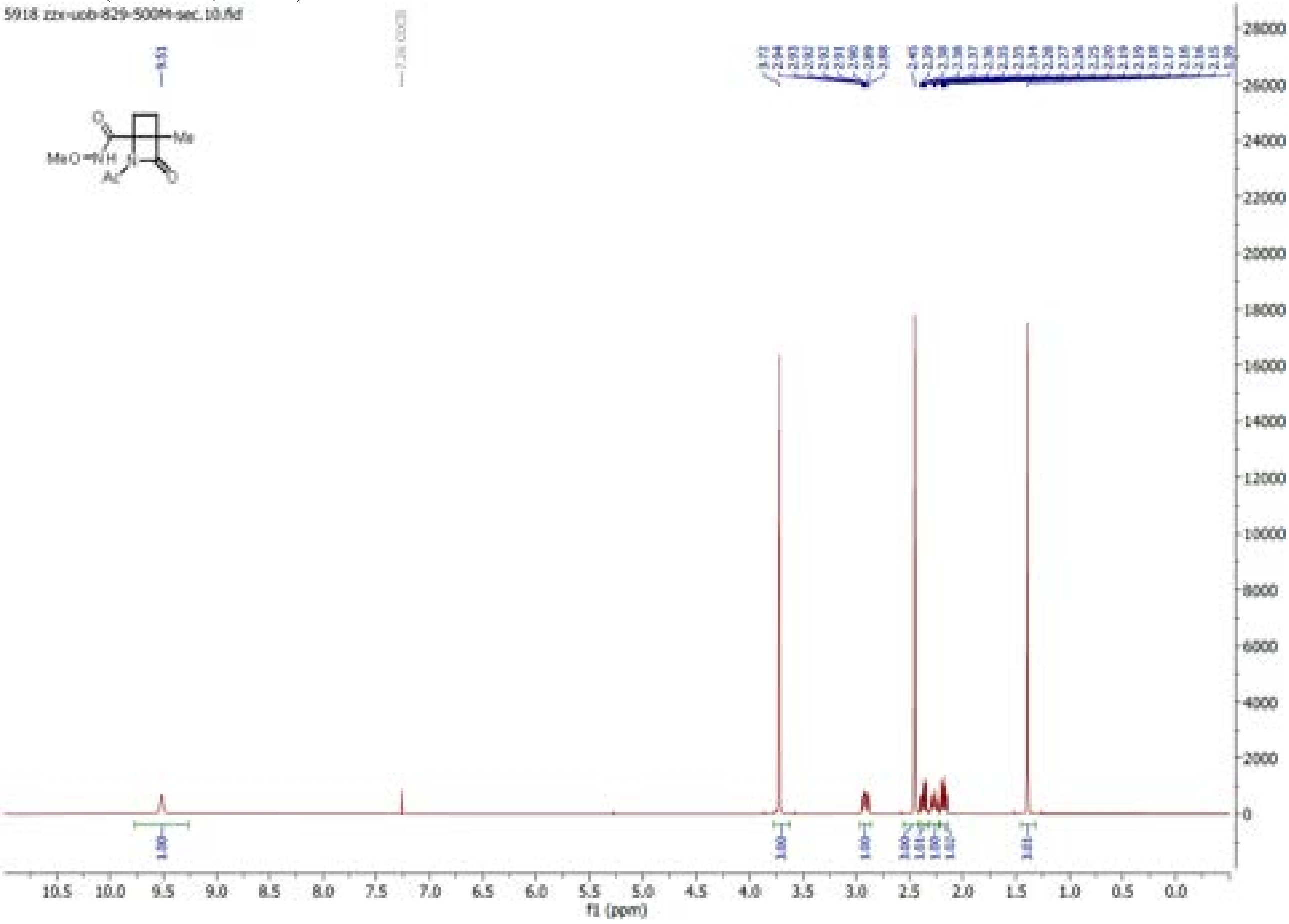

^{13}C NMR (126 MHz, $CDCl_3$) of **20**

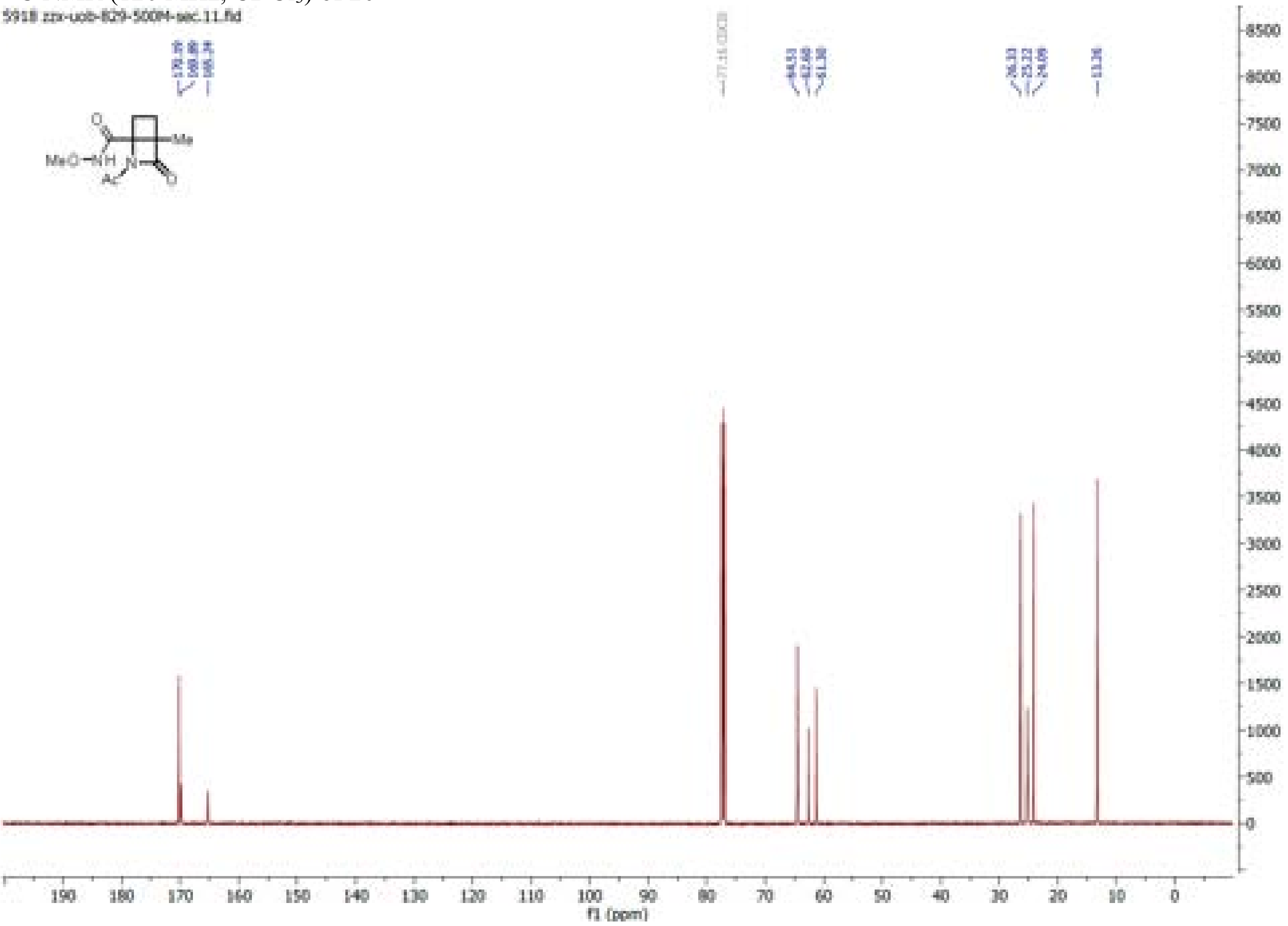

^{1}H NMR (500 MHz, $CDCl_3$) of **21**

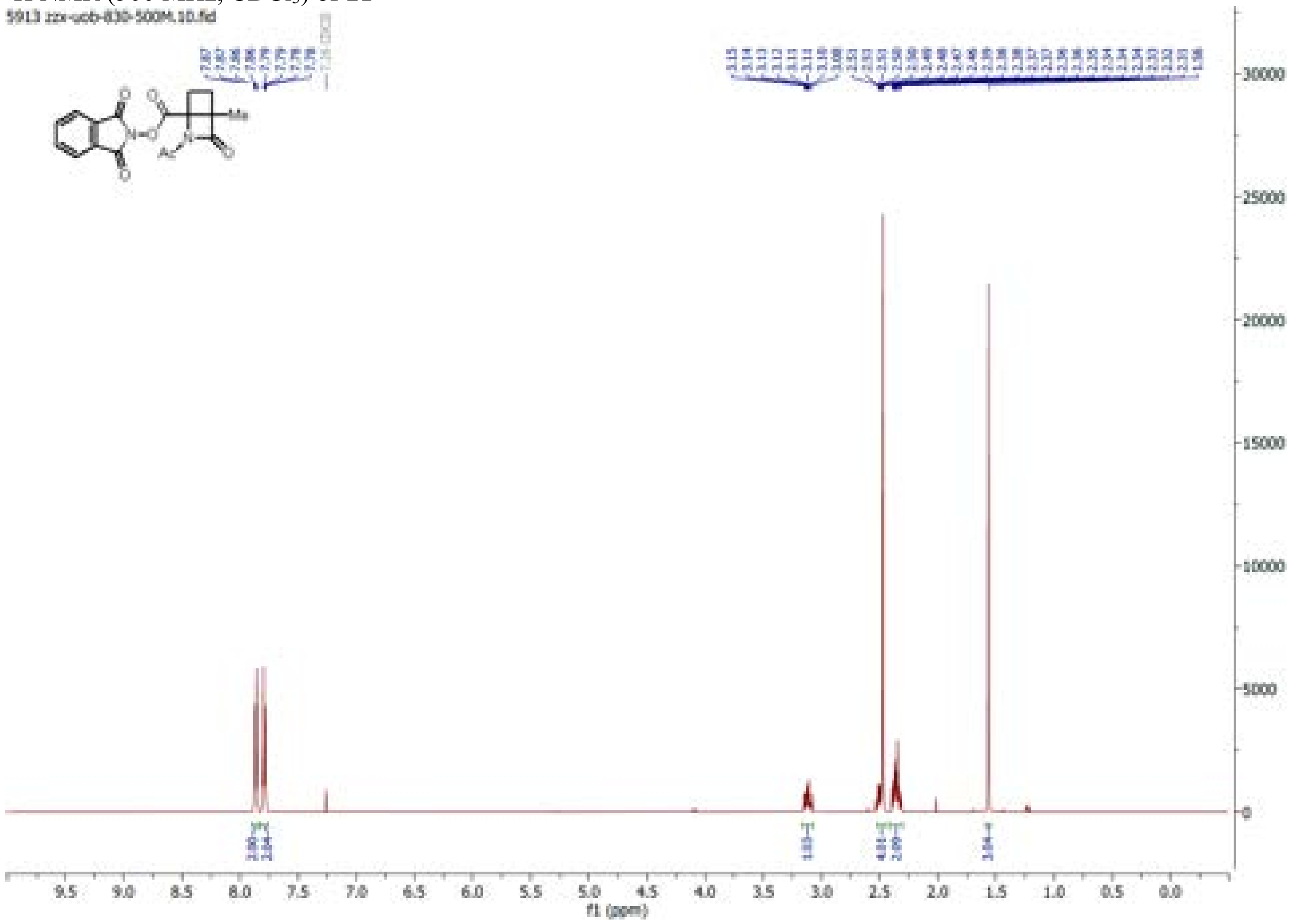

^{13}C NMR (126 MHz, $CDCl_3$) of **21**

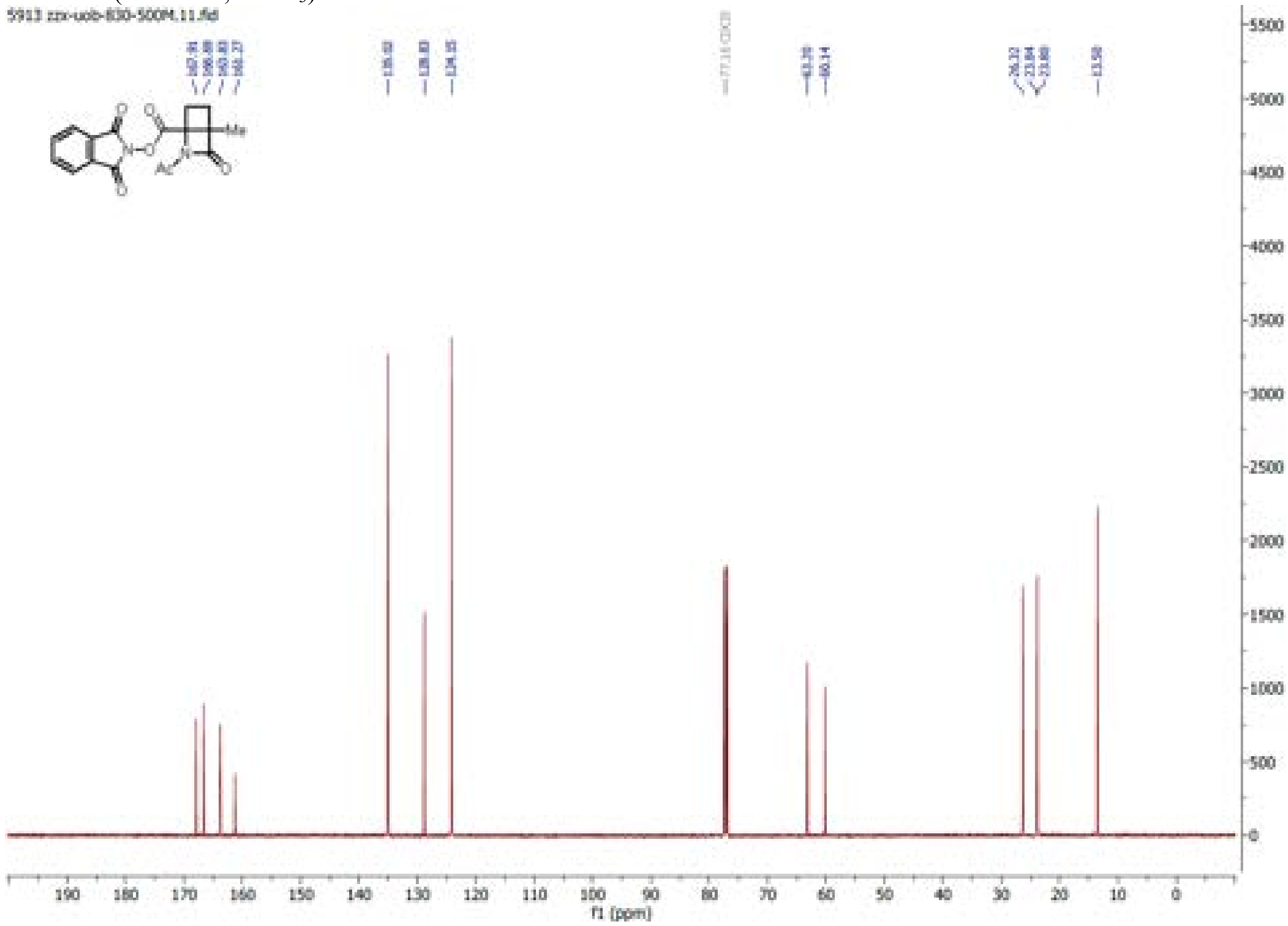

6. Computational Details

6.1. General Information

Density functional theory (DFT) geometry optimizations were carried out using the hybrid meta-GGA M06-2X[26] functional in conjunction with Pople's 6-31+g(d,p) double-z basis set[27-31] and Grimme's zero-damped D3 empirical dispersion[32] correction term. Single-point energy corrections were applied using the double hybrid Pr2SCAN50[33] in conjunction with Aldrich's triple-z def2-TZVPPD basis set[34,35] and D4 dispersion correction[36]. Both optimizations and single-point energy calculations included the integral equation formalism variant of the polarizable continuum model (IEF-PCM), with the SMD solvation model, accounting for an implicit description of solvation by acetonitrile[37]. The choice of level of theory was informed based on recent benchmarking studies performed against DLPNO-CCSD(T)/CBS(3,4) energetics[38]. Similarly, for triplet state calculations, wavefunction stability analyses were performed to ensure that the SCF iterations converged to the lowest energy triplet state[38]. *Gaussian16* revision C.01 was employed for all DFT geometry optimizations[39]. *ORCA* version 6.0.0 was used to compute all single-point energies[40], with the "tightscf" convergence criteria. An "ultrafine" pruned (99,590) grid for numerical integration of the exchange-correlation functional and its derivatives was implemented for all *Gaussian16* calculations, while the *ORCA6* calculations employed the "DEFGRID2" for numerical integration. *ORCA6* additionally implements the resolution of identity (RI) approximation[41]. Open-shell singlet structures located under the Broken-Symmetry formalism have been corrected using the Yamaguchi approximate spin-projection scheme[42], as implemented in *ORCA6*.

Conformational analysis was performed using the Global Optimizer (GOAT) algorithm[43] implemented in *ORCA6* with a GFN2-xTB semiempirical tight-binding Hamiltonian[44], followed by manual conformational augmentation. Vibrational frequency calculations were performed to verify that stationary points were either minima or first-order saddle points on the potential energy surface (PES) and to calculate thermal corrections to Gibbs free energy (G). The identity of the first-order saddle points were further verified using Intrinsic Reaction Coordinate (IRC) calculations[45]. The computed thermochemistry data were corrected following Grimme's quasi-harmonic (QHA)[46] model for entropy with a frequency cut-off value of 100.0 cm^{-1} using the *GoodVibes* program[47] at 298.15 K (25 °C). Additionally, 1 M standard state concentrations were applied to all individual calculations to account for reactions in solution[48]. Unless otherwise stated, the G values of all the energy profiles correspond to the Boltzmann weighted G of all the conformers found in each step[49], calculated by *GoodVibes*. Boltzmann weighted G (G_{av}) were calculated with *GoodVibes* as:

$$G_{av} = \sum_i G_i p_i$$

where G_i is the relative Gibbs free energy of the corresponding conformers of a certain reaction step and p_i is the probability of each conformer calculated as:

$$p_i = \frac{e^{-\frac{G_i}{RT}}}{\sum_i \left(e^{-\frac{G_i}{RT}}\right)}$$

Minimum energy crossing points (MECP) were located using the *MECPro* python package[50] interface with *Gaussian16*, and Gibbs free energy was estimated using the projected frequency keyword ("freq=projected").

Molecular graphics were generated using *PyMol*[51].

Cartesian coordinates have been compiled using *GoodVibes*.

Natural population analysis (NPA) was carried out using NBO7 interfaced with G16[52].

Example G16 optimization input command line:

```
# M06-2X 6-31+G(d,p) emp=gd3 scrf=(smd,solvent=acetonitrile) opt freq
```

NOTE 1: For locating first-order saddle points, the "opt=(TS,calcfc,noeigen)" was used instead.

NOTE 2: For open-shell biradicals the reference wavefunction was obtained in a separate calculation employing the "guess=mix stable=opt" additional keywords.

Example ORCA6 single point energy input command line:

```
! Pr2SCAN50 D4 tightscf def2-tzvppd def2-tzvppd/C SMD(acetonitrile)
```

NOTE: For open-shell biradicals the following keywords were also included.

```
%SCF BrokenSym 1,1

        APMethod 3

END
```

6.2. Reaction Mechanism: Thermochemical Summary

A full summary of the computed reaction pathways for N–Ac substrate **2f** (**¹A_Ac**) is shown in Figure S11 (Figure 4 in the manuscript) and the related pathways for N–Bn substrate **1g** (**¹A_Bn**) is shown in Figure S12. In all cases, the initial C–C bond formation is predicted to occur irreversibly, making the 6-*endo* and 5-*exo* cyclization transition structures regiodetermining. For structure **¹B_Ac** the corresponding open-shell species was also calculated and found to be higher in energy than the closed-shell form by 6.2 kcal/mol (see Table S14; 1B_Ac-CS vs 1B_Ac-OS).

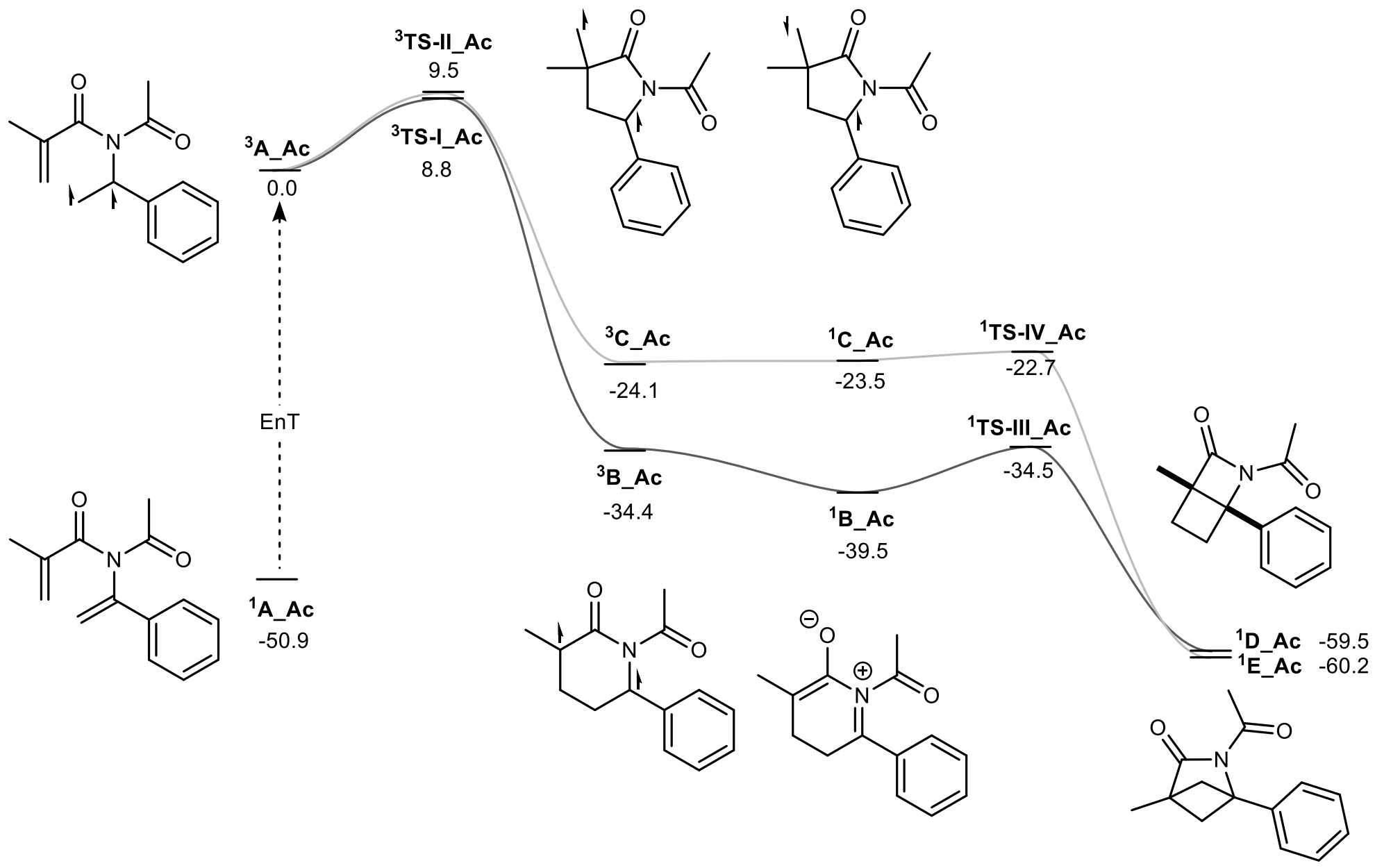

Figure S11: Computed potential energy surface of **¹A_Ac** (**2f**) cycloaddition at the Pr2SCAN50-D4/def2-TZVPPD,(SMD=MeCN)//M06-2X-D3/6-31+G(d,p),(SMD=MeCN) level of theory.

Table S12: Relative thermochemistry computed for **¹A_Ac** (**2f**) cycloaddition at the Pr2SCAN50-D4/def2-TZVPPD,(SMD=MeCN)//M06-2X-D3/6-31+G(d,p),(SMD=MeCN) level of theory.

Species	*ΔE / kcal mol⁻¹*	*ΔZPE / kcal mol⁻¹*	*ΔH / kcal mol⁻¹*	*TΔS / kcal mol⁻¹*	*ΔG(298 K)_SPC / kcal mol⁻¹*
¹A_Ac	−54.8	2.9	−52.4	−1.5	−50.9
³A_Ac	0.0	0.0	0.0	0.0	0.0
³TS-I_Ac	6.8	0.4	6.4	−2.4	8.8
³B_Ac	−38.5	1.9	−37.8	−3.4	−34.4
¹B_Ac	−45.4	3.7	−42.6	−3.1	−39.5
¹TS-III_Ac	−39.7	2.5	−38.2	−3.7	−34.5
¹D_Ac	−67.1	4.5	−63.8	−4.3	−59.5
³TS-II_Ac	7.4	0.1	6.9	−2.7	9.5
³C_Ac	−26.5	0.1	−27.3	−3.2	−24.1
¹C_Ac	−27.6	1.7	−26.6	−3.1	−23.5
¹TS-IV_Ac	−28.1	1.9	−27.4	−4.7	−22.7
¹E_Ac	−68.6	4.9	−65.1	−4.9	−60.2

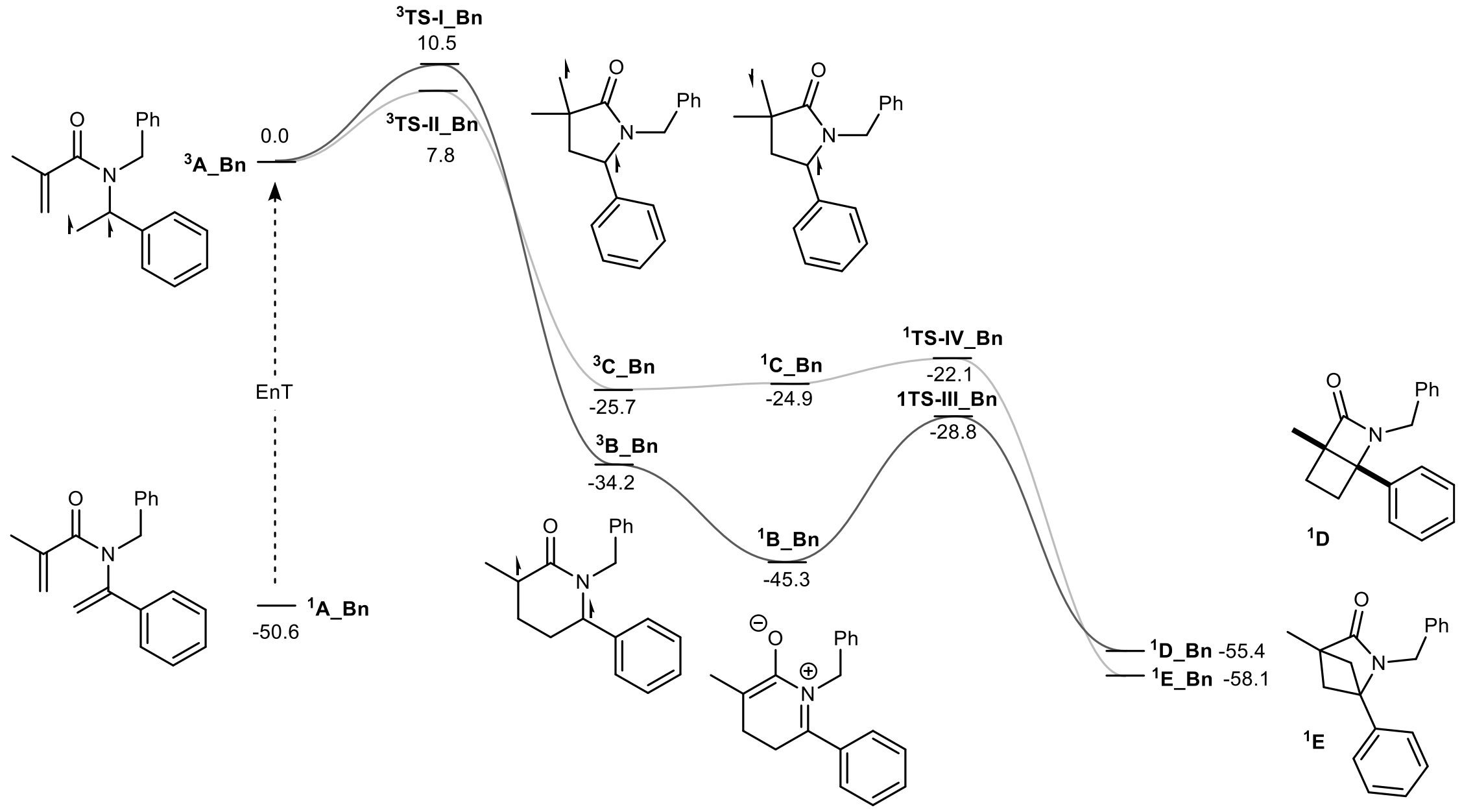

Figure S12: Computed potential energy surface of **^{1}A_Bn** (**1g**) cycloaddition at the Pr2SCAN50-D4/def2-TZVPPD,(SMD=MeCN)//M06-2X-D3/6-31+G(d,p),(SMD=MeCN) level of theory.

Table S13: Relative thermochemistry computed for **^{1}A_Bn** (**1g**) cycloaddition at the Pr2SCAN50-D4/def2-TZVPPD,(SMD=MeCN)//M06-2X-D3/6-31+G(d,p),(SMD=MeCN) level of theory.

Species	*ΔE / kcal mol^{-1}*	*ΔZPE / kcal mol^{-1}*	*ΔH / kcal mol^{-1}*	*TΔS / kcal mol^{-1}*	*ΔG(298 K)_SPC / kcal mol^{-1}*
^{1}A_Bn	−54.3	2.8	−51.9	−1.3	−50.6
^{3}A_Bn	0.0	0.0	0.0	0.0	0.0
^{3}TS-I_Bn	9.1	0.4	8.8	−1.8	10.5
^{3}B_Bn	−37.2	1.8	−36.4	−2.2	−34.2
^{1}B_Bn	−50.7	4.0	−47.7	−2.4	−45.3
^{1}TS-III_Bn	−32.8	2.2	−31.5	−2.8	−28.8
^{1}D_Bn	−62.0	4.3	−58.8	−3.4	−55.4
^{3}TS-II_Bn	6.8	0.1	6.3	−1.6	7.8
^{3}C_Bn	−28.4	1.9	−27.2	−1.5	−25.7
^{1}C_Bn	−28.1	1.8	−26.9	−2.0	−24.9
^{1}TS-IV_Bn	−26.2	1.7	−25.6	−3.5	−22.1
^{1}E_Bn	−65.4	4.6	−62.2	−4.2	−58.1

Minimum Energy Crossing Point (MECP) structures for N–Ac substrates closely resemble the corresponding triplet state minima **^{3}B** and **^{3}C** , such that minimal structural distortion is required to approach these geometries before ISC leads to the following singlet minima **^{1}B** and **^{1}C**. Computed values of ΔΔG‡ obtained from competing 6-*endo* vs. 5-*exo* TSs are highly correlated with experimental selectivities.

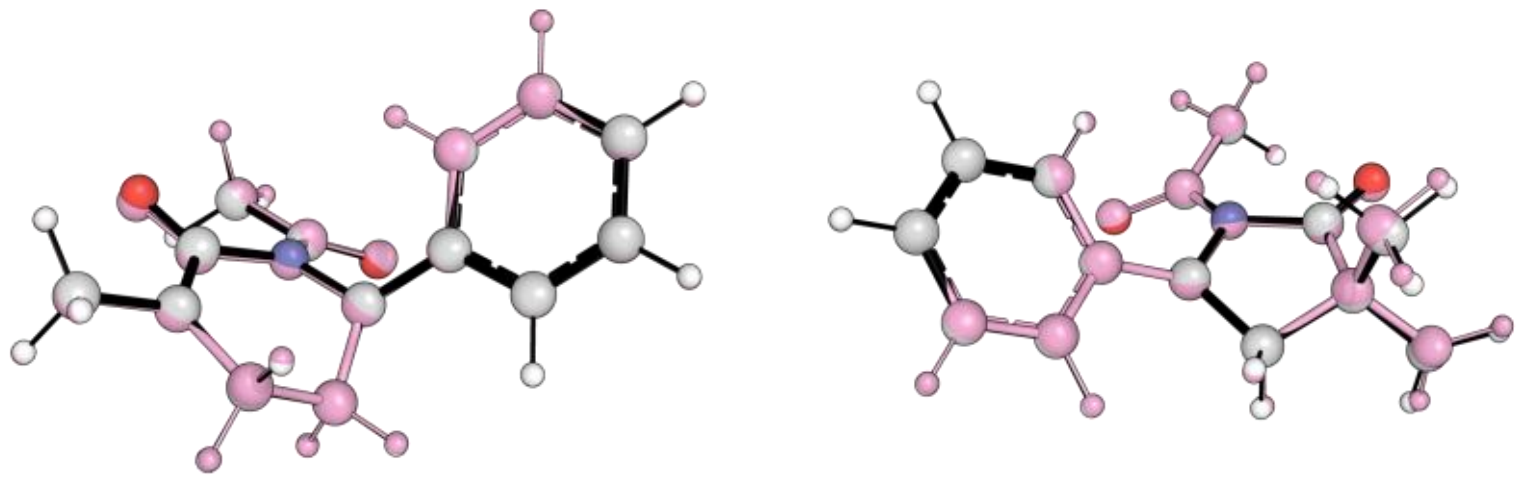

^{3}B_Ac / B_Ac_MECP
E(S_0) = -747.283416 Hartree
E(T_1) = -747.283214 Hartree
ΔE(S_0/T_1) = 0.13 kcal mol^{-1}

^{3}C_Ac / C_Ac_MECP
E(S_0) = -747.264227 Hartree
E(T_1) = -747.264138 Hartree
ΔE(S_0/T_1) = 0.06 kcal mol^{-1}

Figure S13: Overlay of MECP structures (grey) with their corresponding T_1 local minimum (pink), together with the electronic energies of the MECP computed at the Pr2SCAN50-D4/def2-TZVPPD,(SMD=MeCN)//M06-2X-D3/6-31+G(d,p),(SMD=MeCN) level of theory.

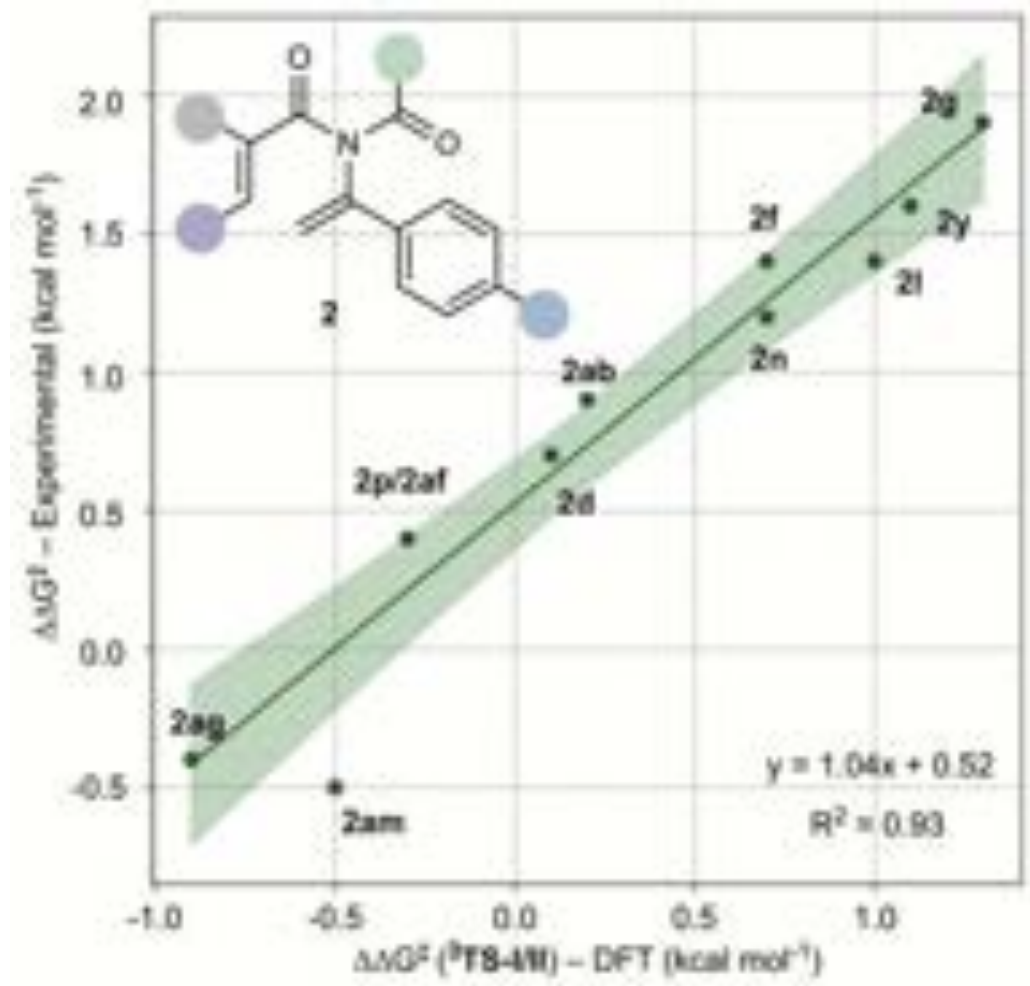

Substrate	*Exp. Ratio (6:5)*	*ΔΔG‡ (exp) / kcal mol^{-1}*	*ΔΔG‡ (DFT)$^{(a)}$ / kcal mol^{-1}*
2g	24:1	1.9	1.3
2y	16:1	1.6	1.1
2l	11:1	1.4	1.0
2f	10:1	1.4	0.7
2n	7:1	1.2	0.7
2ab	4.5:1	0.9	0.2
2d	3.4:1	0.7	0.1
2p	2:1	0.4	-0.3
2af	2:1	0.4	-0.3
2ag	1:2	-0.4	-0.9
2am	1:2.2	-0.5	-0.5

Figure S14: Correlation plot between experimental determined ΔΔG‡ obtained from isolated **6** and **5** product ratios, and DFT computed ΔΔG‡ at the Pr2SCAN50-D4/def2-TZVPPD,(SMD=MeCN)//M06-2X-D3/6-31+G(d,p),(SMD=MeCN) level of theory. [(a)] Only the lowest energy conformer was used. The shaded area represents the 95% confidence interval of the fitted line.

For the *N*-Ac structures, the more favorable 6-*endo* cyclization (**^{3}TS-I-Ac**) benefits from greater stabilisation of the developing radical α-to the carbonyl: this resonance can be seen as a shortening of the adjacent C–C bond to 1.47 Å (**Figure S15**). This stabilization is not available to the 5-*exo* pathway, and is also enhanced relative to the *N*-Bn substrate (**^{3}TS-I-Bn**), where there is stronger N-C=O resonance (N–C = 1.38 Å) making the carbonyl less able to delocalize the developing radical (C–C = 1.49 Å). The N-C=O resonance in **^{3}TS-I-Ac** (N–C = 1.43 Å) is weaker, and so the carbonyl is more able to stabilize the developing radical, stabilizing this TS.

We also note the natural spin densities (obtained from Natural Population Analysis on the M06-2X/6-31+G(d,p) calculations) on the benzylic carbon atom (i.e. the spectator radical not directly involved in the ring-closure). Here a smaller value indicates greater radical delocalization (1 = no

delocalization). The 5-*exo* TS benefits from greater stabilization of the spectator radical. In the case of the *N*-Bn TS, this is maximized (spin density = 0.59), leading to energetic stabilization of this structure. The spin is delocalized into the adjacent phenyl ring as well as the adjacent nitrogen atom.

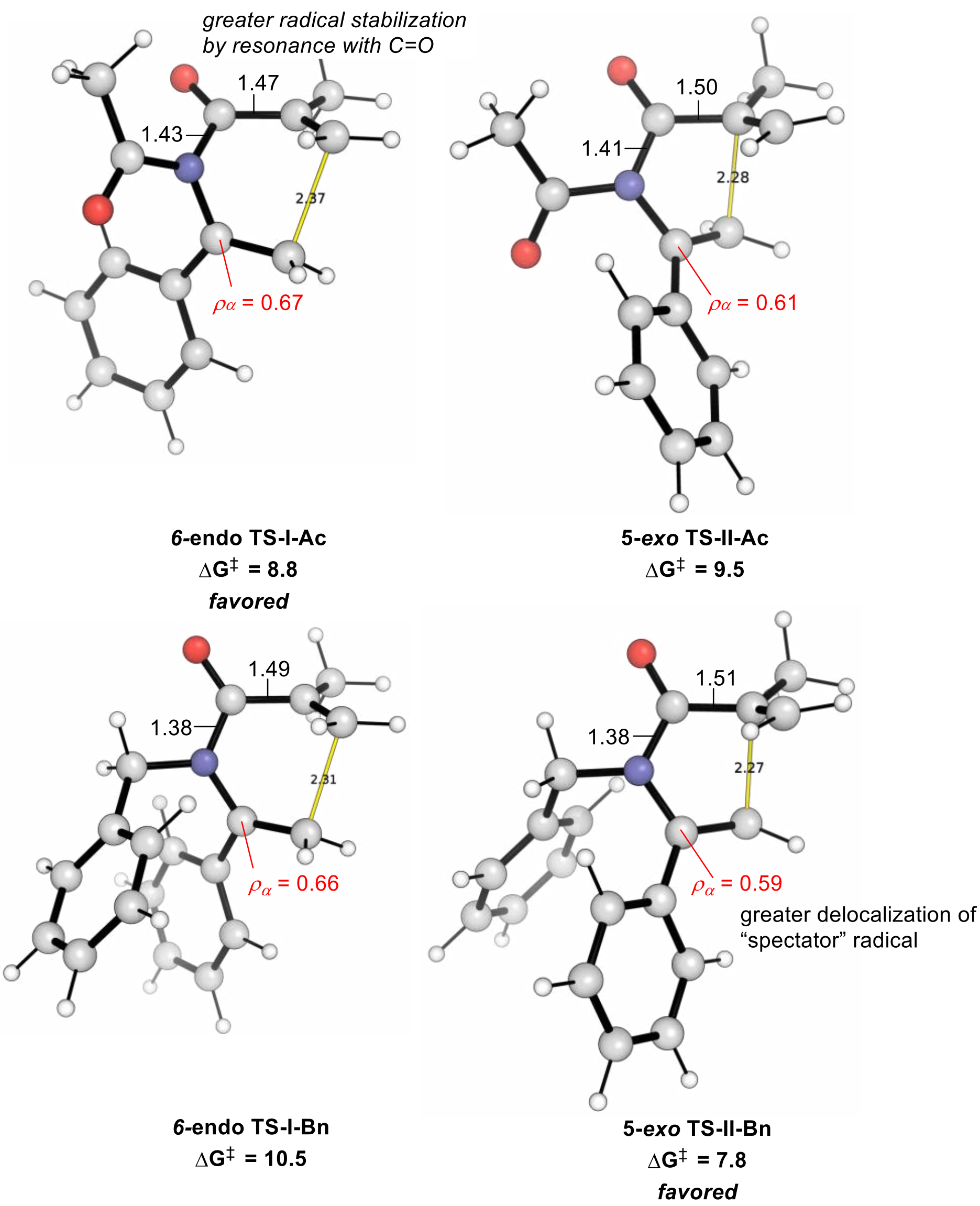

Figure S15: Natural spin densities (ρ_α) of the benzylic carbon atom of the key selectivity-determining TSs, obtained from natural population analysis (NPA) computed at the M06-2X/6-31+G(d,p) level of theory, highlight the greater degree of delocalisation in the lowest energy TSs.

6.3. Dynamic vertical triplet energy analysis

Computation of dynamic vertical triplet energies (DvTE)[53] was undertaken using a total of 50 quasi-classical initiated *ab-initio* molecular dynamics trajectories (298 K, 1 fs timestep, 500 fs simulation length) using the MILO package[54] at the M06-2X/MIDI![55] basis set and employing the SMD solvation model for acetonitrile (**Figure S16**). Vertical S_0-T_1 snapshots were collected at the M06-2X/6-31G(d) (SMD=MeCN) level of theory every 8th frame, leading to a total of 3125 datapoints. The resulting triplet energy of 60.8 kcal/mol was determined using the previously empirically determined 0.8% population of the fitted Gaussian distribution (m = 97.5, s = 7.8). Analysis of the spin density of the out-of-equilibrium structures with the lowest S_0-T_1 gap revealed that the spin density is localised on the styrene chromophore, consistent with Stern-Volmer quenching studies.

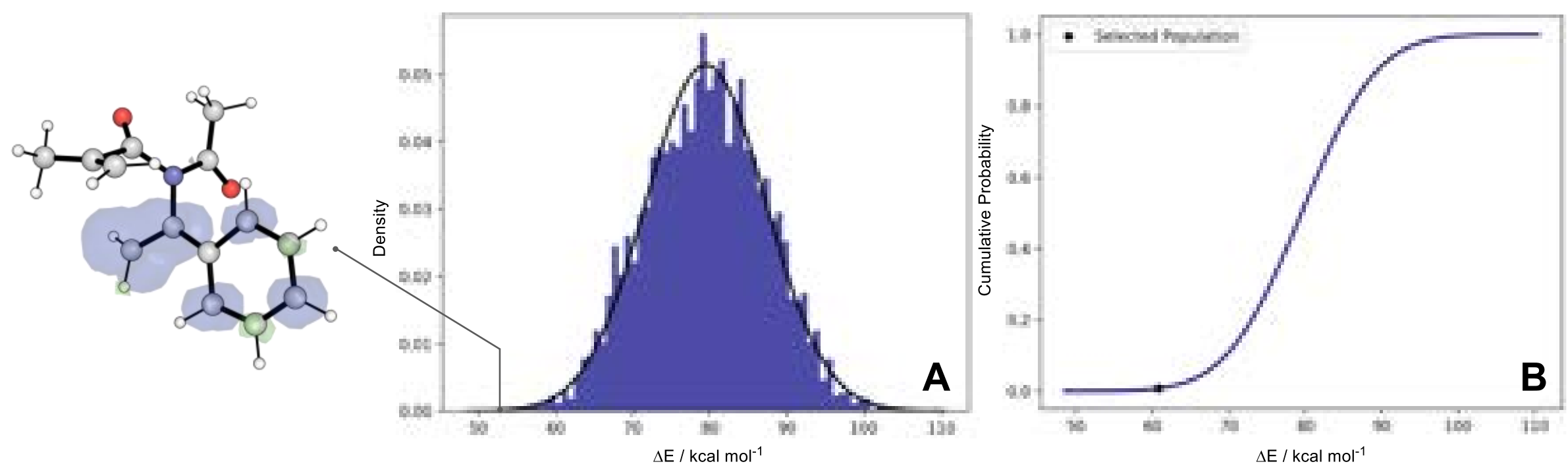

Figure S16: Dynamic vertical triplet energy (DvTE) analysis performed on substrate **2f** (**^{1}A_Ac**) computed at the M06-2X/6-31G(d)//M06-2X/MIDI! level of theory. (**A**) Fitted Gaussian distribution (total sample size of n = 3125) together with the T_1 spin density plot of the frame with the lowest S_0-T_1 gap. (**B**) Cumulative distribution plot of the fitted Gaussian distribution with the highlighted 0.8% population.

6.4. Thermochemistry

Table S14: Compiled thermochemical data in atomic units for structures computed at the Pr2SCAN50-D4/def2-TZVPPD,(SMD=MeCN)//M06-2X-D3/6-31+G(d,p),(SMD=MeCN) level of theory. E_SPC = energy obtained in the single-point energy corrections, E = energy obtained in the geometry optimisations, ZPE = zero-point energy, H_SPC = enthalpy corrected with ESPC, T·S = temperature times entropy, G(T) = Gibbs free energy, n_{imag} = imaginary frequencies

Legend:

Structure	E_SPC	E	ZPE	H	T·S	G(T)_SPC	ν_{imag}
1A_Ac_conf_1	-747.309383	-747.445639	0.251627	-747.039863	0.057210	-747.097073	
1A_Ac_conf_10	-747.308830	-747.445343	0.251608	-747.039182	0.057896	-747.097078	
1A_Ac_conf_11	-747.309110	-747.444933	0.251670	-747.039443	0.057744	-747.097187	
1A_Ac_conf_12	-747.307724	-747.443653	0.252233	-747.037728	0.057154	-747.094881	
1A_Ac_conf_13	-747.309088	-747.444927	0.251689	-747.039418	0.057681	-747.097099	
1A_Ac_conf_2	-747.307713	-747.443711	0.251532	-747.038118	0.057767	-747.095885	
1A_Ac_conf_3	-747.310039	-747.446079	0.251837	-747.040287	0.057473	-747.097760	
1A_Ac_conf_4	-747.306773	-747.443695	0.251749	-747.037150	0.057254	-747.094404	
1A_Ac_conf_5	-747.309471	-747.445509	0.251752	-747.039731	0.057753	-747.097483	
1A_Ac_conf_6	-747.309473	-747.445509	0.251752	-747.039732	0.057751	-747.097483	
1A_Ac_conf_7	-747.308482	-747.444523	0.251235	-747.039039	0.058339	-747.097377	
1A_Ac_conf_8	-747.307684	-747.444184	0.251679	-747.038001	0.057742	-747.095742	
1A_Bn_conf_1	-864.938498	-865.117086	0.322073	-864.595938	0.062828	-864.658766	
1A_Bn_conf_10	-864.935728	-865.114590	0.321996	-864.593251	0.063037	-864.656289	
1A_Bn_conf_11	-864.934476	-865.111966	0.321764	-864.591948	0.063992	-864.655940	
1A_Bn_conf_12	-864.935815	-865.115038	0.322419	-864.593035	0.062464	-864.655500	
1A_Bn_conf_15	-864.934939	-865.113481	0.322504	-864.592168	0.062317	-864.654485	
1A_Bn_conf_16	-864.933743	-865.111282	0.321699	-864.591320	0.063677	-864.654996	
1A_Bn_conf_2	-864.938121	-865.116456	0.322145	-864.595576	0.062732	-864.658308	
1A_Bn_conf_3	-864.937994	-865.116162	0.321998	-864.595443	0.063328	-864.658771	
1A_Bn_conf_4	-864.933043	-865.111694	0.321868	-864.590609	0.063163	-864.653772	
1A_Bn_conf_5	-864.934181	-865.111630	0.322030	-864.591491	0.063340	-864.654832	
1A_Bn_conf_6	-864.936166	-865.114182	0.321881	-864.593730	0.063164	-864.656894	
1A_Bn_conf_7	-864.934461	-865.112754	0.322162	-864.591869	0.062809	-864.654678	
1A_Bn_conf_8	-864.935824	-865.114688	0.321880	-864.593472	0.062900	-864.656372	
1A_Bn_conf_9	-864.937532	-865.116425	0.322212	-864.594870	0.062981	-864.657851	
1B_Ac-CS_conf_1	-747.293936	-747.426128	0.252807	-747.023743	0.056424	-747.080167	
1B_Ac-CS_conf_2	-747.294735	-747.426835	0.253240	-747.024268	0.056057	-747.080325	
1B_Ac-CS_conf_3	-747.293389	-747.425305	0.252869	-747.023163	0.056355	-747.079517	
1B_Ac-OS_conf_1	-747.280488	-747.410811	0.251813	-747.011286	0.056520	-747.067805	
1B_Ac-OS_conf_2	-747.283915	-747.414518	0.252271	-747.014452	0.055985	-747.070437	
1B_Ac-OS_conf_3	-747.281321	-747.412720	0.251834	-747.012143	0.056383	-747.068527	
1B_Ac-OS_conf_4	-747.278773	-747.409964	0.251515	-747.009810	0.056679	-747.066489	
1B_Bn_CS_conf_1	-864.932128	-865.106608	0.323810	-864.588550	0.061742	-864.650291	
1B_Bn_CS_conf_2	-864.930632	-865.105940	0.323954	-864.586994	0.061457	-864.648452	

1B_Bn_CS_conf_3	-864.932683	-865.107515	0.323891	-864.589167	0.061023	-864.650190	
1B_Bn_CS_conf_4	-864.931931	-865.107089	0.323790	-864.588397	0.061376	-864.649774	
1B_Bn_CS_conf_5	-864.931927	-865.107089	0.323788	-864.588394	0.061384	-864.649778	
1B_Bn_CS_conf_6	-864.932280	-865.107337	0.324406	-864.588370	0.060915	-864.649285	
1B_Bn_OSS_conf_3	-864.930781	-865.107515	0.323891	-864.587266	0.061021	-864.648287	
1C_Ac_conf_1	-747.265181	-747.394196	0.249788	-746.997678	0.056695	-747.054373	
1C_Ac_conf_2	-747.261906	-747.390940	0.249580	-746.994458	0.057196	-747.051654	
1C_Ac_conf_3	-747.262895	-747.391620	0.249697	-746.995473	0.056725	-747.052198	
1C_Ac_conf_4	-747.266269	-747.394943	0.249835	-746.998810	0.056382	-747.055192	
1C_Bn_OSS_conf_1	-864.893320	-865.064764	0.320170	-864.552786	0.062599	-864.615384	
1C_Bn_OSS_conf_2	-864.896415	-865.067731	0.320453	-864.555705	0.062162	-864.617867	
1C_Bn_OSS_conf_3	-864.896341	-865.068386	0.320535	-864.555582	0.062015	-864.617597	
1C_Bn_OSS_conf_4	-864.896159	-865.067551	0.320549	-864.555395	0.061970	-864.617365	
1C_Bn_OSS_conf_5	-864.893178	-865.064630	0.320222	-864.552574	0.062677	-864.615251	
1C_Bn_OSS_conf_6	-864.896124	-865.067813	0.320489	-864.555342	0.062317	-864.617658	
1D_Ac_conf_1	-747.328846	-747.460965	0.254298	-747.057712	0.055220	-747.112932	
1D_Ac_conf_2	-747.325617	-747.458018	0.254233	-747.054525	0.055255	-747.109781	
1D_Bn_conf_1	-864.950461	-865.124266	0.324431	-864.606627	0.060647	-864.667274	
1D_Bn_conf_2	-864.948995	-865.123170	0.324407	-864.605130	0.061107	-864.666236	
1E_Ac_conf_1	-747.329926	-747.465815	0.254503	-747.058465	0.055914	-747.114379	
1E_Ac_conf_2	-747.332811	-747.468476	0.254245	-747.061362	0.056594	-747.117956	
1E_Bn_conf_1	-864.955644	-865.129882	0.324801	-864.611904	0.059570	-864.671473	
1E_Bn_conf_2	-864.955282	-865.129765	0.325104	-864.611276	0.059479	-864.670755	
1F_Ac_conf_1	-747.331135	-747.462956	0.254951	-747.059777	0.054354	-747.114131	
1F_Ac_conf_2	-747.326166	-747.458742	0.255186	-747.054822	0.053643	-747.108465	
1F_Bn_conf_1	-864.955644	-865.129882	0.324801	-864.611904	0.059570	-864.671473	
1F_Bn_conf_2	-864.955282	-865.129765	0.325104	-864.611276	0.059479	-864.670755	
1TS-III_Ac_conf_1	-747.287553	-747.416985	0.253079	-747.018073	0.054281	-747.072354	-216.11
1TS-III_Ac_conf_2	-747.281778	-747.401930	0.251193	-747.013523	0.055964	-747.069487	-623.92
1TS-III_Bn_conf_1	-864.884564	-865.056158	0.321788	-864.543505	0.060801	-864.604306	-235.14
1TS-III_Bn_conf_2	-864.903833	-865.064657	0.321136	-864.563002	0.061757	-864.624759	-608.10
1TS-III_Bn_conf_3	-864.903041	-865.063924	0.321150	-864.562252	0.061786	-864.624038	-646.47
1TS-IV_Ac_conf_1	-747.285126	-747.404927	0.251079	-747.016901	0.056198	-747.073099	-629.91
1TS-IV_Ac_conf_2	-747.281778	-747.401930	0.251193	-747.013523	0.055964	-747.069487	-623.92
1TS-IV_Bn_conf_1	-864.884564	-865.056158	0.321788	-864.543505	0.060801	-864.604306	-235.14
1TS-IV_Bn_conf_2	-864.903833	-865.064657	0.321136	-864.563002	0.061757	-864.624759	-608.10
1TS-IV_Bn_conf_3	-864.903041	-865.063924	0.321150	-864.562252	0.061786	-864.624038	-646.47
1TS-V_Ac_conf_1	-747.266568	-747.385165	0.250132	-746.999685	0.054632	-747.054317	-570.65
1TS-V_Bn_conf_1	-864.892512	-865.054165	0.320851	-864.552412	0.060025	-864.612436	-680.81
1TS-V_Bn_conf_2	-864.893432	-865.054362	0.320285	-864.553662	0.060624	-864.614286	-628.57
1TS-V_Bn_conf_3	-864.890238	-865.051554	0.320025	-864.550611	0.061263	-864.611874	-639.16
3A_Ac_conf_10	-747.221247	-747.353832	0.247140	-746.955371	0.059988	-747.015359	
3A_Ac_conf_11	-747.221475	-747.353939	0.247238	-746.955526	0.059993	-747.015519	
3A_Ac_conf_12	-747.221446	-747.354064	0.247575	-746.955316	0.059522	-747.014838	
3A_Ac_conf_13	-747.221474	-747.353939	0.247237	-746.955525	0.059994	-747.015519	

3A_Ac_conf_2	-747.222290	-747.356181	0.247586	-746.956222	0.059150	-747.015372	
3A_Ac_conf_3	-747.223072	-747.356198	0.247370	-746.957106	0.059544	-747.016651	
3A_Ac_conf_5	-747.221195	-747.353947	0.247263	-746.955244	0.059915	-747.015159	
3A_Ac_conf_6	-747.221192	-747.353947	0.247262	-746.955242	0.059913	-747.015155	
3A_Ac_conf_7	-747.221737	-747.354297	0.246782	-746.956030	0.060611	-747.016641	
3A_Ac_conf_8	-747.220888	-747.353776	0.246755	-746.955174	0.060750	-747.015924	
3A_Ac_conf_9	-747.221737	-747.354297	0.246782	-746.956030	0.060611	-747.016641	
3A_Bn_conf_1	-864.852232	-865.027438	0.317519	-864.513456	0.065299	-864.578754	
3A_Bn_conf_10	-864.846912	-865.023439	0.317823	-864.508026	0.064732	-864.572758	
3A_Bn_conf_11	-864.846938	-865.021651	0.317417	-864.508093	0.065840	-864.573933	
3A_Bn_conf_12	-864.850708	-865.026878	0.317630	-864.511879	0.065000	-864.576879	
3A_Bn_conf_13	-864.848924	-865.025528	0.317583	-864.510097	0.066611	-864.576708	
3A_Bn_conf_14	-864.848624	-865.025313	0.317656	-864.509747	0.065436	-864.575183	
3A_Bn_conf_15	-864.847290	-865.023327	0.317788	-864.508418	0.064811	-864.573229	
3A_Bn_conf_16	-864.847048	-865.021653	0.317375	-864.508309	0.065696	-864.574005	
3A_Bn_conf_2	-864.850703	-865.026744	0.317844	-864.511760	0.064989	-864.576748	
3A_Bn_conf_3	-864.850905	-865.026750	0.317581	-864.512056	0.065547	-864.577602	
3A_Bn_conf_4	-864.846483	-865.022072	0.317649	-864.507668	0.064944	-864.572612	
3A_Bn_conf_5	-864.847580	-865.021662	0.317217	-864.508922	0.065947	-864.574869	
3A_Bn_conf_6	-864.849002	-865.024844	0.317587	-864.510263	0.064989	-864.575251	
3A_Bn_conf_7	-864.846418	-865.022301	0.317813	-864.507473	0.064982	-864.572455	
3A_Bn_conf_8	-864.848881	-865.025510	0.317740	-864.509996	0.065121	-864.575117	
3B_Ac_conf_1	-747.279295	-747.410690	0.252022	-747.010031	0.057238	-747.067269	
3B_Ac_conf_2	-747.283289	-747.414516	0.252419	-747.013758	0.056868	-747.070626	
3B_Ac_conf_3	-747.281021	-747.412818	0.252048	-747.011737	0.057130	-747.068867	
3B_Ac_conf_4	-747.278117	-747.409845	0.251583	-747.009115	0.057608	-747.066723	
3B_Ac_MECP	-747.283214	-747.414424	0.249859	-747.016496	0.056385	-747.072881	
3B_Bn_conf_1	-864.905554	-865.078982	0.322246	-864.563283	0.063298	-864.626581	
3B_Bn_conf_2	-864.902908	-865.076227	0.322605	-864.560399	0.062897	-864.623296	
3B_Bn_conf_3	-864.906038	-865.079020	0.322130	-864.563863	0.063188	-864.627050	
3B_Bn_conf_4	-864.910871	-865.084117	0.322450	-864.568537	0.062957	-864.631493	
3B_Bn_conf_5	-864.911328	-865.084326	0.322500	-864.568972	0.062785	-864.631757	
3B_Bn_conf_6	-864.909935	-865.083245	0.322766	-864.567450	0.062488	-864.629938	
3C_Ac_conf_1	-747.264447	-747.393919	0.249736	-746.996948	0.057835	-747.054783	
3C_Ac_conf_2	-747.261162	-747.390634	0.249680	-746.993663	0.058072	-747.051735	
3C_Ac_conf_3	-747.262160	-747.391375	0.249718	-746.994704	0.057812	-747.052516	
3C_Ac_conf_4	-747.265354	-747.394578	0.249861	-746.997865	0.057426	-747.055291	
3C_Ac_MECP	-747.264138	-747.393643	0.247231	-746.999459	0.057170	-747.056629	
3C_Bn_conf_1	-864.893735	-865.065117	0.320257	-864.553153	0.063511	-864.616664	
3C_Bn_conf_2	-864.896958	-865.068139	0.320540	-864.556193	0.063091	-864.619285	
3C_Bn_conf_3	-864.896914	-865.068914	0.320661	-864.556081	0.062943	-864.619025	
3C_Bn_conf_4	-864.896311	-865.067641	0.320588	-864.555509	0.063034	-864.618543	
3C_Bn_conf_5	-864.892988	-865.064621	0.320362	-864.552296	0.063541	-864.615837	
3C_Bn_conf_6	-864.896235	-865.067865	0.320639	-864.555404	0.063034	-864.618439	
3TS-I_2ab_conf_1	-1054.764961	-1054.929854	0.365608	-1054.373782	0.073989	-1054.447772	-458.72

3TS-I_2af_conf_1	-707.913933	-708.040417	0.220609	-707.677338	0.054464	-707.731802	-433.29
3TS-I_2am_conf_1	-861.686759	-861.829384	0.279820	-861.386748	0.066022	-861.452771	-439.44
3TS-I_2ag_conf_1	-935.694972	-935.848858	0.262410	-935.411877	0.064813	-935.476690	-433.13
3TS-I_2d_conf_1	-1084.158613	-1084.299047	0.252115	-1083.885054	0.066498	-1083.951552	-452.65
3TS-I_2g_conf_1	-861.685369	-861.828364	0.279517	-861.385524	0.063511	-861.449035	-450.21
3TS-I_2l_conf_1	-846.420813	-846.553931	0.239476	-846.162746	0.060092	-846.222838	-454.50
3TS-I_2n_conf_1	-1206.684692	-1206.912860	0.238213	-1206.427507	0.061212	-1206.488719	-455.86
3TS-I_2p_conf_1	-839.408805	-839.565257	0.246342	-839.142921	0.062179	-839.205100	-451.56
3TS-I_2y_conf_1	-1128.504015	-1128.705302	0.335546	-1128.143948	0.072888	-1128.216836	-452.32
3TS-I_Ac_conf_1	-747.211367	-747.342546	0.247672	-746.946027	0.057968	-747.003995	-453.19
3TS-I_Ac_conf_2	-747.209178	-747.340486	0.247410	-746.944004	0.058238	-747.002242	-461.74
3TS-I_Bn_conf_1	-864.836325	-865.008841	0.317867	-864.498211	0.063299	-864.561510	-551.91
3TS-I_Bn_conf_2	-864.832403	-865.005149	0.318063	-864.494224	0.063058	-864.557282	-577.29
3TS-I_Bn_conf_3	-864.835542	-865.008071	0.317935	-864.497364	0.063502	-864.560866	-560.84
3TS-I_Bn_conf_4	-864.837873	-865.010101	0.318645	-864.499357	0.062388	-864.561745	-149.60
3TS-II_2ab_conf_1	-1054.764882	-1054.927729	0.365511	-1054.373873	0.073568	-1054.447441	-631.39
3TS-II_2af_conf_1	-707.914493	-708.039911	0.220485	-707.678033	0.054366	-707.732398	-617.82
3TS-II_2am_conf_1	-861.686357	-861.827578	0.279227	-861.386769	0.066442	-861.453211	-578.16
3TS-II_2ag_conf_1	-935.696883	-935.849698	0.262468	-935.413827	0.064322	-935.478150	-601.58
3TS-II_2d_conf_1	-1084.158515	-1084.297699	0.251894	-1083.885257	0.066105	-1083.951362	-612.99
3TS-II_2g_conf_1	-861.683333	-861.825000	0.279090	-861.383973	0.063106	-861.447079	-626.53
3TS-II_2l_conf_1	-846.419443	-846.551326	0.239216	-846.161707	0.059594	-846.221301	-624.29
3TS-II_2n_conf_1	-1206.683862	-1206.910723	0.237990	-1206.426986	0.060658	-1206.487644	-618.70
3TS-II_2p_conf_1	-839.409554	-839.564680	0.246176	-839.143925	0.061610	-839.205535	-601.76
3TS-II_2y_conf_1	-1128.502252	-1128.701799	0.335083	-1128.142616	0.072596	-1128.215212	-631.03
3TS-II_Ac_conf_1	-747.210271	-747.340148	0.247315	-746.945333	0.057590	-747.002923	-623.79
3TS-II_Ac_conf_2	-747.207359	-747.337248	0.247000	-746.942651	0.057808	-747.000459	-620.53
3TS-II_Ac_conf_3	-747.207304	-747.337826	0.247451	-746.942337	0.057303	-746.999641	-646.14
3TS-II_Ac_conf_4	-747.204385	-747.334950	0.247145	-746.939599	0.057654	-746.997253	-638.93
3TS-II_Bn_conf_1	-864.840757	-865.012827	0.317587	-864.502851	0.063453	-864.566303	-595.64
3TS-II_Bn_conf_2	-864.840473	-865.012815	0.317813	-864.502475	0.063104	-864.565579	-593.81
3TS-II_Bn_conf_4	-864.839297	-865.011714	0.318035	-864.501161	0.062836	-864.563997	-598.37
3TS-II_Bn_conf_5	-864.839639	-865.012490	0.318091	-864.501518	0.062710	-864.564227	-587.40
3TS-II_Bn_conf_6	-864.837955	-865.010652	0.317884	-864.499928	0.063089	-864.563016	-616.89

7. References

1 Torres, M. J. *et al.* Controversies in drug allergy: beta-lactam hypersensitivity testing. *J. Allergy Clin. Immunol. Pract.* **7**, 40-45 (2019).

2 Jeimy, S. *et al.* Practical guide for evaluation and management of beta-lactam allergy: position statement from the Canadian Society of Allergy and Clinical Immunology. *Allergy Asthma Clin. Immunol.* **16**, 95 (2020).

3 Mirakian, R. *et al.* Management of allergy to penicillins and other beta-lactams. *Clin. Exp. Allergy* **45**, 300-327 (2015).

4 Suslick, B. A. & Tilley, T. D. Mechanistic interrogation of alkyne hydroarylations catalyzed by highly reduced, single-component cobalt complexes. *J. Am. Chem. Soc.* **142**, 11203-11218 (2020).

5 Berne, D., Poli, R., Caillol, S., Ladmiral, V. & Leclerc, E. Combination of fluorine and tertiary amine activation in catalyst-free thia-Michael covalent adaptable networks. *Macromolecules* **56**, 8260-8274 (2023).

6 Dong, Q.-R. *et al.* Synthesis of unsymmetrical disulfides via photocatalytic hydrodisulfuration. *ACS Catal.* **14**, 18237-18246 (2024).

7 Li, B. *et al.* Nickel-catalyzed asymmetric hydrogenation for the preparation of α-substituted propionic acids. *Nat. Commun.* **15**, 5482 (2024).

8 Yang, Q.-L. *et al.* Electrochemistry-enabled Ir-catalyzed vinylic C–H functionalization. *J. Am. Chem. Soc.* **141**, 18970-18976 (2019).

9 Wang, K. *et al.* Highly efficient and regioselective synthesis of N-acyl protected 2-aminoacrylates through a PPh_3-catalyzed reaction of alkyl propiolates with amides. *ChemistrySelect* **8**, e202300839 (2023).

10 Hu, N. *et al.* Synthesis of Chiral α-Amino Tertiary Boronic Esters by Enantioselective Hydroboration of α-Arylenamides. *J. Am. Chem. Soc.* **137**, 6746-6749 (2015).

11 Kunishige, T. & Sawada, D. Efficient and practical synthesis of N-acetyl enamides from ketoximes by unique iron catalytic system. *Tetrahedron Lett.* **60**, 1562-1565 (2019).

12 Pak, H. K. *et al.* Synthesis of enamides by ruthenium-catalyzed reaction of alkyl azides with acid anhydrides in ionic liquid. *ChemCatChem* **7**, 4030-4034 (2015).

13 Savarin, C. G. *et al.* Direct N-acetyl enamine formation: lithium bromide mediated addition of methyllithium to nitriles. *Org. Lett.* **8**, 3903-3906 (2006).

14 Lyu, X., Zhang, J., Kim, D., Seo, S. & Chang, S. Merging NiH catalysis and inner-sphere metal-nitrenoid transfer for hydroamidation of alkynes. *J. Am. Chem. Soc.* **143**, 5867-5877 (2021).

15 Huang, K. *et al.* Highly enantioselective borane reduction of heteroaryl and heterocyclic ketoxime ethers catalyzed by novel spiroborate ester derived from diphenylvalinol: application to the synthesis of nicotine analogues. *J. Org. Chem.* **73**, 4017-4026 (2008).

16 Jiang, B., Zhao, M., Li, S.-S., Xu, Y.-H. & Loh, T.-P. Macrolide synthesis through intramolecular oxidative cross-coupling of alkenes. *Angew. Chem. Int. Ed.* **57**, 555-559 (2018).

17 Rao, M. L. N. & Giri, S. Pd-catalyzed threefold arylation of Baylis–Hillman bromides and acetates with triarylbismuth reagents. *Eur. J. Org. Chem.* **2012**, 4580-4589 (2012).

18 Brullo, C. *et al.* Design, synthesis, biological evaluation and structural characterization of novel GEBR library PDE4D inhibitors. *Eur. J. Med. Chem.* **223**, 113638 (2021).

19 Pavlishchuk, V. V. & Addison, A. W. Conversion constants for redox potentials measured versus different reference electrodes in acetonitrile solutions at 25°C. *Inorg. Chim. Acta* **298**, 97-102 (2000).

20 Prier, C. K., Rankic, D. A. & MacMillan, D. W. C. Visible light photoredox catalysis with transition metal complexes: applications in organic synthesis. *Chem. Rev.* **113**, 5322-5363 (2013).

21 SAINT integration software version 8.39.0 (Bruker AXS Inc., Madison, WI).

22 SADABS version 2016/2 (Bruker AXS Inc., Madison, WI).

23 Dolomanov, O. V., Bourhis, L. J., Gildea, R. J., Howard, J. A. K. & Puschmann, H. OLEX2: a complete structure solution, refinement and analysis program. *J. Appl. Crystallogr.* **42**, 339-341 (2009).
24 *International Tables for Crystallography*. Vol. C (Wiley, 2016).
25 Parsons, S., Flack, H. D. & Wagner, T. Use of intensity quotients and differences in absolute structure refinement. *Acta Crystallogr. B* **69**, 249-259 (2013).
26 Zhao, Y. & Truhlar, D. G. The M06 suite of density functionals for main group thermochemistry, thermochemical kinetics, noncovalent interactions, excited states, and transition elements: two new functionals and systematic testing of four M06-class functionals and 12 other functionals. *Theor. Chem. Acc.* **120**, 215-241 (2008).
27 Hehre, W. J., Ditchfield, R. & Pople, J. A. Self-consistent molecular orbital methods. XII. Further extensions of Gaussian-type basis sets for use in molecular orbital studies of organic molecules. *J. Chem. Phys.* **56**, 2257-2261 (1972).
28 Hariharan, P. C. & Pople, J. A. The influence of polarization functions on molecular orbital hydrogenation energies. *Theor. Chim. Acta* **28**, 213-222 (1973).
29 McLean, A. D. & Chandler, G. S. Contracted Gaussian basis sets for molecular calculations. I. Second row atoms, Z=11–18. *J. Chem. Phys.* **72**, 5639-5648 (1980).
30 Francl, M. M. *et al.* Self-consistent molecular orbital methods. XXIII. A polarization-type basis set for second-row elements. *J. Chem. Phys.* **77**, 3654-3665 (1982).
31 Rassolov, V. A., Ratner, M. A., Pople, J. A., Redfern, P. C. & Curtiss, L. A. 6-31G* basis set for third-row atoms. *J. Comput. Chem.* **22**, 976-984 (2001).
32 Grimme, S., Antony, J., Ehrlich, S. & Krieg, H. A consistent and accurate ab initio parametrization of density functional dispersion correction (DFT-D) for the 94 elements H-Pu. *J. Chem. Phys.* **132**, 154104 (2010).
33 Wittmann, L., Neugebauer, H., Grimme, S. & Bursch, M. Dispersion-corrected r^2SCAN based double-hybrid functionals. *J. Chem. Phys.* **159**, 224103 (2023).
34 Weigend, F. & Ahlrichs, R. Balanced basis sets of split valence, triple zeta valence and quadruple zeta valence quality for H to Rn: Design and assessment of accuracy. *Phys. Chem. Chem. Phys.* **7**, 3297-3305 (2005).
35 Rappoport, D. & Furche, F. Property-optimized Gaussian basis sets for molecular response calculations. *J. Chem. Phys.* **133**, 134105 (2010).
36 Caldeweyher, E. *et al.* A generally applicable atomic-charge dependent London dispersion correction. *J. Chem. Phys.* **150**, 154122 (2019).
37 Marenich, A. V., Cramer, C. J. & Truhlar, D. G. Universal solvation model based on solute electron density and on a continuum model of the solvent defined by the bulk dielectric constant and atomic surface tensions. *J. Phys. Chem. B* **113**, 6378-6396 (2009).
38 Hughes, W. B., Popescu, M. V. & Paton, R. S. Fundamental study of density functional theory applied to triplet state reactivity: introduction of the TRIP50 data set. *J. Chem. Theory Comput.* **22**, 3530-3542 (2026).
39 Gaussian 16, Revision C.01 (Gaussian, Inc., 2016).
40 Neese, F. The ORCA program system. *WIREs Comput. Mol. Sci.* **2**, 73-78 (2012).
41 Neese, F. An improvement of the resolution of the identity approximation for the formation of the Coulomb matrix. *J. Comput. Chem.* **24**, 1740-1747 (2003).
42 Yamaguchi, K., Kitagawa, Y. & Saito, T. in *Symmetry (Group Theory) and Mathematical Treatment in Chemistry* (ed Takashiro Akitsu) (IntechOpen, 2018).
43 de Souza, B. GOAT: a global optimization algorithm for molecules and atomic clusters. *Angew. Chem. Int. Ed.* **64**, e202500393 (2025).
44 Bannwarth, C., Ehlert, S. & Grimme, S. GFN2-xTB—an accurate and broadly parametrized self-consistent tight-binding quantum chemical method with multipole electrostatics and density-dependent dispersion contributions. *J. Chem. Theory Comput.* **15**, 1652-1671 (2019).
45 Fukui, K. The path of chemical reactions - the IRC approach. *Acc. Chem. Res.* **14**, 363-368 (1981).

46 Luchini, G., Alegre-Requena, J. V., Funes-Ardoiz, I. & Paton, R. S. GoodVibes: automated thermochemistry for heterogeneous computational chemistry data. *F1000Research* **9**, 291 (2020).
47 Grimme, S. Supramolecular binding thermodynamics by dispersion-corrected density functional theory. *Chem. Eur. J.* **18**, 9955-9964 (2012).
48 Bryantsev, V. S., Diallo, M. S. & Goddard III, W. A. Calculation of solvation free energies of charged solutes using mixed cluster/continuum models. *J. Phys. Chem. B* **112**, 9709-9719 (2008).
49 Plata, R. E. & Singleton, D. A. A case study of the mechanism of alcohol-mediated Morita Baylis–Hillman reactions. The importance of experimental observations. *J. Am. Chem. Soc.* **137**, 3811-3826 (2015).
50 MECPro v. 1.0.5: Minimum Energy Crossing Program (Brigham Young University, 2019).
51 The PyMOL Molecular Graphics System v. 2.0.7 (Schrödinger, LLC, 2017).
52 Glendening, E. D., Landis, C. R. & Weinhold, F. NBO 7.0: New vistas in localized and delocalized chemical bonding theory. *J. Comput. Chem.* **40**, 2234-2241 (2019).
53 Popescu, M. V. & Paton, R. S. Dynamic vertical triplet energies: understanding and predicting triplet energy transfer. *Chem.* **10**, 3428-3443 (2024).
54 Milo, revision 1.0.3 (Brigham Young University, Provo, UT, 2021).
55 Easton, R. E., Giesen, D. J., Welch, A., Cramer, C. J. & Truhlar, D. G. The MIDI! basis set for quantum mechanical calculations of molecular geometries and partial charges. *Theor. Chim. Acta* **93**, 281-301 (1996).
